# Enantioconvergent nucleophilic substitution via synergistic phase-transfer catalysis

**DOI:** 10.1038/s41929-024-01288-0

**Published:** 2025-02-13

**Authors:** Claire Dooley, Francesco Ibba, Bence B. Botlik, Chiara Palladino, Christopher A. Goult, Yuan Gao, Andrew Lister, Robert S. Paton, Guy C. Lloyd-Jones, Véronique Gouverneur

**Affiliations:** 1 Chemistry Research Laboratory, University of Oxford, Oxford, UK; 2 School of Chemistry, University of Edinburgh, Edinburgh, UK; 3 Oncology R&D, AstraZeneca, Cambridge, UK; 4 Department of Chemistry, Colorado State University, Fort Collins, CO USA

**Keywords:** Stereochemistry, Organocatalysis

## Abstract

Catalytic enantioconvergent nucleophilic substitution reactions of alkyl halides are highly valuable transformations, but they are notoriously difficult to implement. Specifically, nucleophilic fluorination is a renowned challenge, especially when inexpensive alkali metal fluorides are used as fluorinating reagents due to their low solubility, high hygroscopicity and Brønsted basicity. Here we report a solution by developing the concept of synergistic hydrogen bonding phase-transfer catalysis. Key to our strategy is the combination of a chiral *bis*-urea hydrogen bond donor (HBD) and an onium salt—two phase-transfer catalysts essential for the solubilization of potassium fluoride—as a well-characterized ternary HBD–onium fluoride complex. Mechanistic investigations indicate that this chiral ternary complex is capable of enantiodiscrimination of racemic benzylic bromides and α-bromoketones, and upon fluoride delivery affords fluorinated products in high yields and enantioselectivities. This work provides a foundation for enantioconvergent fluorination chemistry enabled through the combination of a HBD catalyst with a co-catalyst specifically curated to meet the requirement of the electrophile.

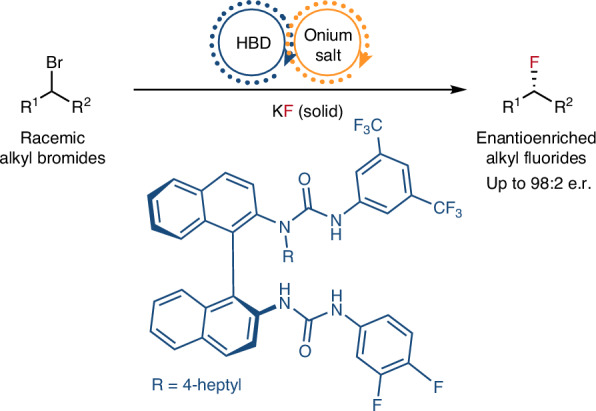

## Abstract (web summary)

The catalytic enantioconvergent nucleophilic fluorination of alkyl halides using inexpensive alkali metal fluorides is a persistent challenge. Now this has been achieved by synergistic hydrogen bonding phase-transfer catalysis combining a chiral bis-urea hydrogen bond donor and an onium salt.

## Main

**Fig. 1:**
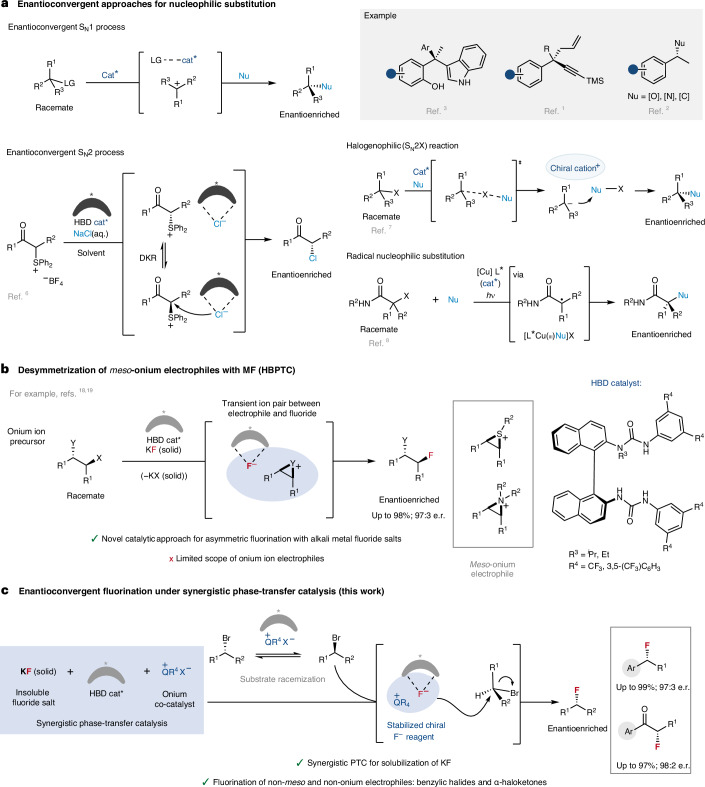
Enantioconvergent approaches for nucleophilic substitution. **a**, Nucleophilic substitution via S_N_1, S_N_2, S_N_2X, transition metal-catalysed and photoredox mechanisms. **b**, HBPTC for the desymmetrization of *meso*-onium-type electrophiles with alkali metal fluoride salts (MF). **c**, S-HBPTC (this work): enantioconvergent nucleophilic substitution of benzylic bromides and α-haloketones with KF and two phase-transfer catalysts (a chiral HBD and an achiral onium salt). *hv*, visible light; cat*, chiral catalyst; DKR, dynamic kinetic resolution; TMS, trimethylsilyl; Nu, nucleophile; LG, leaving group.

Nucleophilic substitutions represent a class of foundational reactions in organic synthesis. Enantioconvergent catalytic variants whereby racemic starting materials can be converted into enantiopure products are synthetically valuable, but difficult to implement. Studies by Jacobsen^1^, List^2^ and Sun^3^ have enabled enantioconvergent substitutions for carbon–carbon, carbon–nitrogen and carbon–oxygen bond formation via the intermediacy of achiral carbocations (unimolecular nucleophilic substitutions (S_N_1)) (Fig. 1a). Enantioconvergent bimolecular nucleophilic substitution (S_N_2) reactions are also known and feature highly reactive electrophiles, such as carbonyls alpha substituted with a leaving group—a class of substrates prone to racemization and therefore allowing for dynamic kinetic resolutions^4,5^. A recent report by Sun^6^ demonstrates this principle with the enantioselective chlorination of α-keto sulfonium salts under liquid–liquid phase transfer with NaCl (aq.) in the presence of a chiral thiourea hydrogen bond donor (HBD) catalyst (Fig. 1a)^6^. Solutions that are based on more unconventional mechanistic scenarios have also been disclosed, including a halogenophilic S_N_2X manifold, as well as elegant transition metal-catalysed and photoredox cross-coupling reactions involving radical intermediates (Fig. 1a)^7–10^.

The development of an enantioconvergent fluorination presents its own challenges, especially when the nucleophile is an alkali metal fluoride including poor solubility in organic solvents and competitive elimination pathways^11,12^. The use of crown ethers or onium salts as phase-transfer catalysts is a well-established way to increase the solubility of fluoride salts (KF and CsF) for halide substitution and is commonly applied in the syntheses of fluorochemicals such as fluoroarenes via nucleophilic aromatic substitution (S_N_Ar) chemistry^13,14^. It is noteworthy that although chiral polyether and ammonium salts have been used as catalysts to solubilize alkali metal fluorides to form chiral F^−^ species, this has been largely employed for desilylative kinetic resolution protocols, which take advantage of the strength of Si–F bonds^15,16^. Similar desilylation strategies were implemented to unmask nucleophilic functionalities allowing for asymmetric C–C bond formation^17^. However, the application of phase-transfer catalysis to asymmetric C–F bond formation remains underdeveloped. Our group disclosed hydrogen bonding phase-transfer catalysis (HBPTC), which exploits the principles of anion-binding catalysis as a solution for asymmetric nucleophilic fluorination with alkali metal fluorides^18^. Chiral *bis*-urea catalysts were developed, which bring these solid fluoride salts into solution, thereby controlling the reactivity of the resulting hydrogen-bonded fluoride ion and allowing for enantioselective fluorination of selected substrate classes. HBPTC is currently limited to asymmetric desymmetrization of onium-type electrophiles, including *meso*-episulfonium and aziridinium salts (generated in situ from the corresponding alkyl halides (Fig. 1b)^18,19^) or preformed achiral azetidinium salts^20^. Mechanistic investigations have highlighted the importance of ion pairing between these onium electrophiles and urea fluoride complexes for successful fluorination under HBPTC with either CsF (lattice energy = 759 kJ mol^−1^; US$72 mol^−1^) or the inexpensive but more energetically demanding salt, KF (lattice energy = 829 kJ mol^−1^; US$8 mol^−1^)^21,22^. The extension of this methodology to alkyl halides other than precursors of *meso*-onium salts represents a notable challenge both in terms of reactivity and enantioselectivity. In this Article, we disclose a solution to this problem with the invention of synergistic HBPTC (S-HBPTC), a catalytic manifold enabling the enantioconvergent substitution of racemic alkyl halides with potassium fluoride.

In this scenario, two organocatalysts comprising a chiral HBD and an achiral onium salt facilitate substrate racemization and contribute to a catalytic cycle leading to a chiral HBD–onium fluoride ion pair for enantioconvergent fluorination (Fig. 1c). This method grants access to enantioenriched benzylic fluorides and α-fluoroketones—a valuable synthetic advance considering the ubiquity of benzylic stereogenic centres in pharmaceutical design and the well-known versatility of ketones in synthesis^23,24^.

## Results

### Reaction development

**Fig. 2:**
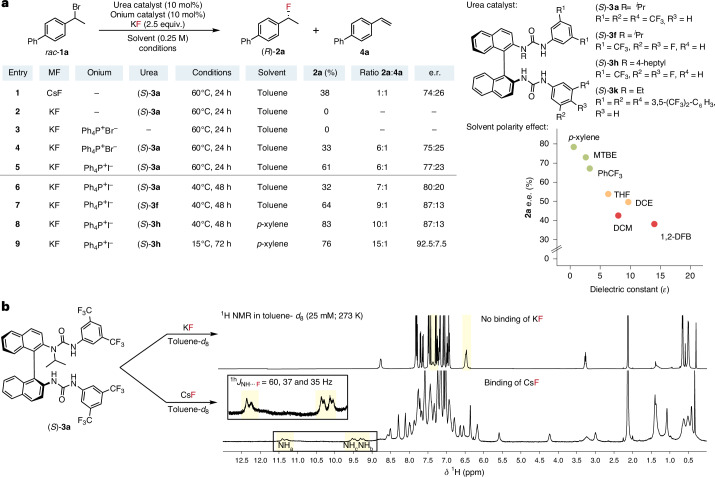
Reaction optimization. **a**, Optimization of enantioselective fluorination under S-HBPTC. Conditions: *rac*-**1a** (0.05 mmol) at 25 mM concentration. ^19^F NMR yields are reported. Enantiomeric ratios were determined using high-performance liquid chromatography with a chiral stationary phase. **b**, Spectroscopic study of the phase transfer of MF salts by *bis*-urea catalyst (*S*)-**3a**. 1,2-DFB, 1,2-difluorobenzene; DCE, dichloroethane; MBTE, methyl *tert*-butyl ester; THF, tetrahydrofuran.

We initially investigated the propensity of the secondary benzylic bromide *rac*-**1a** to undergo reaction with CsF in toluene, under HBPTC by the *bis*-urea (*S*)-**3a**. Although only trace conversion occurred at room temperature, at 60 °C the reaction gave benzylic fluoride (*R*)-**2a** (38%; 74:26 e.r.) and alkene **4a** in an approximately equal ratio (Fig. 2a, entry 1). The soluble fluoride source tetrabutylammonium fluoride generated racemic **2a** and the alkene **4a**, also in a 1:1 ratio, under otherwise similar reaction conditions (Supplementary Table 1). In contrast, no reaction was observed with KF in place of CsF (Fig. 2a, entry 2), thus NMR experiments were conducted to compare the ability of the *bis*-urea catalyst (*S*)-**3a** to bring solid CsF and KF into solution (toluene-*d*_8_). ^1^H NMR spectroscopic analysis of *bis*-urea (*S*)-**3a** after the addition of CsF revealed strong binding of the fluoride ion, as indicated by the diagnostic deshielding (Δ*δ*_NH_ = 2–4 ppm) and ^1^H–^19^F scalar coupling (^1h^*J*_NH–F_ = 60, 37 and 35 Hz) of the urea NH resonances^21^. In contrast, no such changes were observed with KF, with the resultant ^1^H NMR representing unbound (*S*)-**3a** (Fig. 2b). These data led us to reflect on the successful fluorination of β-chloroamines with KF under HBPTC, and to hypothesize that in this instance the reactive *meso-*aziridinium salt aids the solubilization of KF as an onium-type phase-transfer agent in partnership with (*S*)-**3a**^19^. This putative scenario encouraged us to revisit the attempted fluorination of benzylic bromide **1a** with KF (Fig. 2a, entry 2) using a range of onium salt co-catalysts (Supplementary Fig. 1). Although the onium salt Ph_4_P^+^Br^−^ (10 mol%) on its own failed to induce fluorination in the presence of KF (2.5 equiv.) in toluene at 60 °C (Fig. 2a, entry 3), when used in combination with *bis*-urea (*S*)-**3a** (10 mol%) the benzylic fluoride **2a** was generated in moderate yield (33%), chemoselectivity (**2a**:**4a** = 6:1) and enantioselectivity (75:25 e.r.) (Fig. 2a, entry 4). This dual catalytic platform (Ph_4_P^+^Br^−^ and (*S*)-**3a**) not only enabled the use of KF, but also greatly improved the selectivity for fluorination over elimination, a key challenge in nucleophilic fluorination because of the accompanying Brønsted basicity of fluoride^25,26^. These data prompted an in-depth study on this discovered manifold, which we termed synergistic HBPTC (S-HBPTC). The reaction proceeded in the presence of a range of ammonium and sulfonium halide co-catalysts; however, tetraarylphosphonium salts ensured a higher level of enantiocontrol (Supplementary Fig. 1). Employing an enantiopure onium co-catalyst had no beneficial effect on the transformation, and no considerable (mis)match effect was observed using either enantiomer of a chiral ammonium salt (Maruoka phase-transfer catalysts), which gave **2a** with e.r. values of 71:29 and 73:27, respectively (Supplementary Fig. 1). A marked increase in yield (61%; 24 h) and minimal improvement in enantioselectivity were observed with Ph_4_P^+^I^−^ instead of Ph_4_P^+^Br^−^ as the co-catalyst (Fig. 2a, entry 5), whereas an increased loading of Ph_4_P^+^I^−^ was not beneficial for yield or e.r. (Supplementary Table 2). Variation of the urea catalyst was shown to exert the greatest effects on the stereochemical outcome of the reaction. The *bis*-urea catalyst (*S*)-**3f**, having 3,4-difluorophenyl substituents, led to a marked increase in enantioselectivity (87:13 e.r.) (Fig. 2a, entry 7). A profound solvent effect was observed with an inverse correlation between the dielectric constant of the solvent and the enantiomeric ratio observed for **2a**; solvents of lower polarity were optimal, with *p-*xylene selected as the solvent of choice for this transformation (Fig. 2a). Further HBD catalyst optimization investigated the impact of *N*-alkylation, which ultimately led to the development of catalyst (*S*)-**3h**, which gave **2a** in 83% yield while maintaining enantioselectivity (87:13 e.r.) at 40 °C (Fig. 2a, entry 8). The final conditions included the treatment of *rac*-**1a** with KF (2.5 equiv.), catalysts (*S*)-**3h** (10 mol%) and Ph_4_P^+^I^−^ (10 mol%) in *p*-xylene (0.25 M) at 15 °C, which allowed for the formation of fluoride **2a** in 76% yield and 92.5:7.5 e.r.; elimination at this lower temperature was also further minimized (Fig. 2a, entry 9).

### Evaluation of substrate scope

**Fig. 3:**
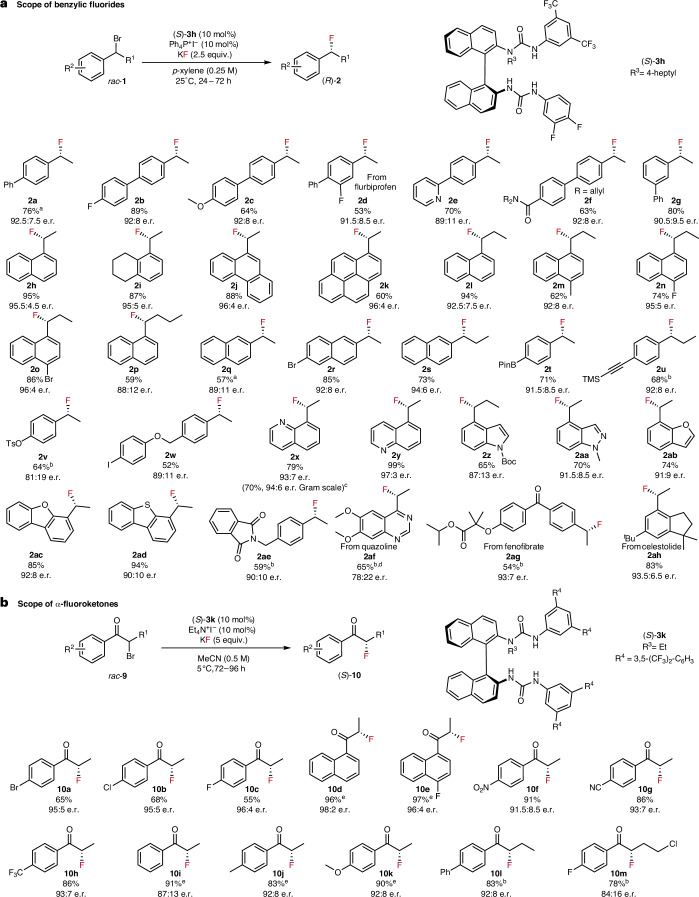
Reaction scope. **a**, Scope of benzylic fluorides. **b**, Scope of α-fluoroketones. The yields of the isolated products are reported. ^a^Performed at 15 °C. ^b^Performed at 40 °C. ^c^98% recovery of (*S*)-**3h**. ^d^In 1,4-difluorobenzene for 96 h. ^e^Perfomed at 25 °C.

With optimized conditions established, a range of substrates were evaluated to probe the reaction scope and gain preliminary insights into the mechanism for this enantioconvergent transformation (Fig. 3a). Variation of the electronic substitution on the biphenyl scaffold had minimal impacts on the enantioselectivity of the product (**2a**–**2c**). Substrates based on a 1-napthalene structure (**2h**–**2p**, **2x** and **2y**) underwent fluorination in the highest enantioselectivities (up to 97:3 e.r.). Common functional groups, including amides (**2f**), ethers (**2c** and **2w**), aryl halides (**2b**, **2r** and **2w**) and carboxylic (**2ag**) and sulfonate esters (**2v**), were well tolerated under the reaction conditions. The mild reaction conditions were highlighted by the tolerance of fluorophilic functionalities, including boron pinacol ester (**2t**) and a trimethylsilyl group (**2u**). Variation of the alkyl chain demonstrated that increasing length from methyl to *n-*propyl had minimal impact on substitution versus elimination, and the fluorinated products were isolated in up to 86% yield and 96:4 e.r. (**2h**, **2l** and **2p**). Departing from phenyl-based substrates, the reaction conditions were further extended to fluorinate a range of *O*- and *N*-heteroaromatic motifs, including quinolines (**2x** and **2y**), indoles (**2z**), indazoles (**2aa**), benzofurans (**2ab** and **2ac**) and benzothiophenes (**2ad**). Finally, analogues of more complex bioactive molecules were subjected to enantioconvergent fluorination, including quazoline (**2af**; 65% yield; 78:22 e.r.)^27^, fenofibrate (**2ag**; 54% yield; 93:7 e.r.) and celestolide derivatives (**2ah**; 83% yield; 93.5:6.5 e.r.). Although reactivity and enantioselectivity were maintained when meta-substituents were present on the aryl ring (**2g**), the reactivity diminished for substrates with large ortho- (**2ai**) or α substituents (**2ak** and **2aj**). The reaction conditions also did not allow for the fluorination of a tertiary benzylic substrate (**2al**), which predominately underwent elimination to form the corresponding alkene side product (see Supplementary Fig. 2).

The successful implementation of S-HBPTC towards the synthesis of enantioenriched benzylic fluorides prompted investigation into the fluorination of other electrophiles. We opted for α-haloketones because all current synthetic approaches towards enantioenriched α-fluoroketones rely on electrophilic fluorinating reagents of poor atom economy (for example, *N*-fluorobenzenesulfonimide)^28^. Gratifyingly, minor modifications to the system were required with urea (*S*)-**3k** and Et_4_N^+^I^−^ as the onium salt, allowing for the catalytic fluorination of α-bromoketone **9a** with KF in 65% yield and 95:5 e.r. (Fig. 3b and Supplementary Table 6). Acetonitrile was selected as the solvent for this transformation due to poor solubility of the substrates in solvents of low polarity. Various α-bromoketones were subjected to the optimized conditions, with good reactivity and enantioselectivity maintained across electron-withdrawing and -donating functionalities (**10c** and **10f**–**10k**). α-Fluoroketones furnishing elongated alkyl chains could also be prepared in good yields and enantioselectivity at higher reaction temperatures (**10l** and **10m**). Of note, ketones featuring 1-naphthalene scaffolds showed the highest enantioselectivities, corroborating the fluorination of electrophiles featuring extended π systems (**10d** and **10e**).

### Spectroscopic investigation

**Fig. 4:**
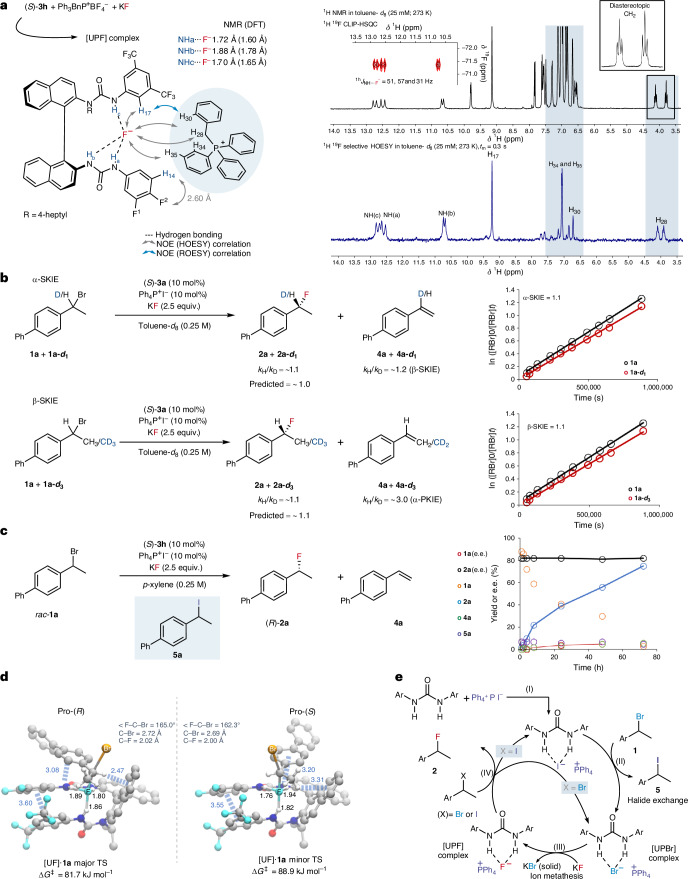
Mechanistic investigations. **a**, Spectroscopic evidence for the formation of [UPF] species. **b**, SKIE studies, predicted values and pseudo first-order kinetic plots. [RBr]_0_, initial concentration of bromide substrate; [RBr]_*t*_, concentration of bromide at time (*t*). **c**, Investigation into the enantiomeric excess of **1a** and **2a** via ex situ time course monitoring. **d**, Diastereomeric transition state structures (computed using: M06-2X-D3ZERO/def2-TZVPP (C, H) ma-def2-TZVPP). **e**, Proposed catalytic cycle. CLIP-HSQC, clean in-phase heteronuclear single quantum coherence; DFT, density functional theory; TS, transition state; *t*_m_, mixing time delay.

Preliminary mechanistic investigation aimed to gain understanding of the synergistic role of the catalysts (Fig. 4a). Interactions of the HBD, the onium salt and KF were investigated through a spectroscopic NMR study of a combination of (*S*)-**3h**, KF and Ph_3_BnP^+^BF_4_^−^ (Supplementary Figs. 13–22). Ph_3_BnP^+^BF_4_^−^ was selected for the non-coordinating nature of the BF_4_^−^ anion and for the presence of methylene protons, which become diastereotopic in a chiral environment. The addition of KF to a solution of (*S*)**-3h** or Ph_3_BnP^+^BF_4_^−^ in toluene-*d*_8_ displayed no spectroscopic changes of proton resonances for (*S*)-**3h** or Ph_3_BnP^+^BF_4_^−^, suggesting that no interaction with KF occurred when only one catalyst was present. In contrast, on the addition of KF to a solution containing both (*S*)-**3h** and Ph_3_BnP^+^BF_4_^−^, the ^1^H NMR spectrum revealed substantial deshielding of several proton resonances, consistent with the formation of a new species. Three diagnostic doublets were detected at 12.7, 12.5 and 10.6 ppm (^1h^*J*_NH···F_ = 51, 57 and 31 Hz), corresponding to the urea NH protons (Δ*δ*_NH_ = ~+6 ppm H_a_ and H_c_ and + 3.5 ppm H_b_)^21^. The ^1h^*J*_NH···F_ scalar coupling, verified by clean in-phase ^1^H–^19^F heteronuclear single quantum coherence measurements (CLIP-HSQC), is indicative of hydrogen bonding of the urea catalyst to fluoride, and its magnitude correlates with the strength of the hydrogen bond interaction^29^. The generation of a [Ph_3_BnP]^+^[UF]^−^ complex (where UF represents urea fluoride) was also confirmed by the ^19^F NMR spectrum, which contained a signal at −75 ppm (Supplementary Fig. 16) characteristic of the hydrogen-bonded fluoride ion and a singlet at 22.3 ppm in the ^31^P{^1^H} NMR spectrum (Supplementary Fig. 17), which was assigned to the phosphonium cation of the complex (Δ*δ*_P_ = ~−0.6 ppm from Ph_3_BnP^+^BF_4_^−^). Signals corresponding to diastereotopic benzylic H(28) protons in the phosphonium ion indicated a close association between Ph_3_BnP^+^ and the chiral [UF]^−^ complex (Fig. 4a). Collectively, the above data indicate that the urea and phosphonium salt can solubilize KF to generate a dynamically stable singular ternary hydrogen-bonded complex. Nuclear Overhauser effect (NOE) cross-peaks in the two-dimensional rotating frame Overhauser effect spectroscopy (ROESY) NMR spectrum of the [Ph_3_BnP]^+^[UF]^−^ complex confirmed the identity of all three urea NH resonances, and cross-peaks were also detected between the urea scaffold and an ortho-proton in the benzyl group of the phosphonium cation (H(17)–H(30)) (Supplementary Fig. 22). The urea–phosphonium fluoride ([UPF]) complex was further characterized by ^1^H–^19^F heteronuclear Overhauser spectroscopy (HOESY). Strong NOE correlations were detected between the bound fluoride ion and protons in both the urea and Ph_3_BnP^+^ (Fig. 4a). ^1^H–^19^F NOE build-up curves were used to investigate the location of the fluoride ion in the ternary [UPF] complex^21^. The HOESY analysis indicated that the fluoride ion is in closest proximity to NH_a_ and NH_c_, with near equidistant binding (1.72 and 1.70 Å, respectively), and more remote (1.88 Å) from NH_b_, which is adjacent to the 1,1′-binaphthyl backbone. This orientation of the bound anion is in agreement with previous data obtained on *bis*-urea–fluoride complexes of (*S*)-**3a**^21^. Notably, protons H(28), H(30) and H(34/35) of the phosphonium cation in the [UPF] complex were also found to be within 3 Å of the urea-bound fluoride, quantitatively showing close spatial proximity between the cation and anion (Supplementary Table 13). Very different behaviour was found for [UPF] complexes generated in the more polar solvent dichloromethane-*d*_2_ (DCM-*d*_2_); ^1^H NMR spectra of the resulting [UPF] complex displayed deshielding of the urea NH signals (Δδ_NH_ = ~3–5 ppm) accompanied by extensive line broadening; no scalar coupling for NH was detected, although a signal corresponding to a fluoride ion (−86 ppm) was detected by ^19^F NMR. This suggests that the dynamic stability of the [UPF] complex is reduced through attenuation of the electrostatic interactions by the DCM medium, which has a higher dielectric constant (Supplementary Figs. 23–25)^30^. The inverse correlation between the dielectric constant of the solvent and the enantiocontrol of (*R*)-**2a** (Fig. 2a; at 40 °C, toluene = 87:13 e.r. and DCM = 71:29 e.r.) may be related to this phenomenon, with apolar solvents favouring the formation of a tighter ternary [UPF] complex. Together, these data validate the synergistic role of the urea HBD catalyst and onium salt in solubilizing KF with the formation of a stable [UPF] ion pair.

Further experiments were performed to answer mechanistic questions on this catalytic manifold, including whether the fluorination occurs via an S_N_1 or S_N_2-like mechanism and the mode of racemization/ionization of the substrate depending on substitution type. We also investigated the basis for the moderate rate acceleration observed when onium iodide salts are employed as co-catalysts instead of onium bromide salts and the nature of the non-covalent interactions between [UPF] species and the electrophile. This study was carried out with benzylic bromides due to their complex mechanistic regimen; for α-haloketones, it is indeed reasonable to exclude an S_N_1 mechanism—and therefore the possibility of ion pairing with a carbocationic intermediate—for this class of substrates^31^.

### Kinetic isotope effect study

The extent of ionization of the benzylic bromides in the nucleophilic substitution was probed by determining ^1^H/^2^H secondary kinetic isotope effects (SKIEs) for reactions of **1a*-d***_**1**_ (α-SKIE) and **1a-*d***_**3**_ (β-SKIE). If the benzylic bromide **1a** undergoes substitution via a transient carbocation and the commitment factor is large, significant normal kinetic isotope effects (KIEs) would be expected (α-SKIE ≥ 1.1; β-SKIE ≥ 1.2) in an apolar medium such as *p*-xylene or toluene^32,33^. Conversely, if C–F bond formation accompanies bromide departure, smaller normal (α and β) or inverse (α) SKIEs would be expected, depending on the degree of synchronization^34^. The SKIEs were estimated via two intermolecular competition experiments between the labelled substrates (**1a**-***d***_**1**_ and **1a-*d***_**3**_) and **1a**, analysed by quantitative ^1^H and ^19^F NMR spectroscopy. The heterogenous reactions require continuous agitation to disperse the solid KF in the medium and this periodic activation was provided by conducting the reactions in sealed NMR tubes that were continuously inverted by mechanical rotation when not in the spectrometer^35^. Analysis of the temporal concentration data showed that the bromide (**1a** and **1a-*d***_**n**_) was consumed with approximately pseudo first-order kinetics to generate the fluoride (**2a** and **2a-*d***_**n**_) and alkene (**4a** and **4a-*d***_**n**_) at a constant ratio. This indicates that both products are generated by irreversible partitioning of the same reaction manifold, allowing estimation of the KIEs arising from fluoride addition (α-SKIE = ~1.1 and β-SKIE = ~1.1) and elimination (β-SKIE = ~1.2 and α-PKIE = ~3.0) (Fig. 4b). The KIE data do not support a substantial charge-separated ionization of the benzylic substrate and are more consistent with an S_N_2-like process for the enantioconvergent fluorination under S-HBPTC in *p*-xylene, which is further supported by predicted KIE values from computed transition state structures (Supplementary Table 19). The values can be compared with a β-SKIE of ~1.5 for fluoride addition and an α-PKIE of ~5.8 for elimination in the uncatalysed homogeneous reaction of **1b** or **1b-*d***_**3**_ with tetrabutylammonium fluoride in DCM, which has kinetics that are more characteristic of an S_N_1/E1 process (Supplementary Fig. 41 and Supplementary Table 18).

### Substrate racemization

An S_N_2-like mechanism for fluorination under S-HBPTC in *p*-xylene requires efficient racemization of the benzylic substrate (**1a**) for high yield and enantioselectivity to be obtained. Reaction monitoring elucidated that **1a** remains racemic over the course of the reaction (Fig. 4c), suggesting that the rate of substrate racemization exceeds the rate of fluorination, with a comparable trend observed for α-bromoketone **9a** (Supplementary Table 22 and Supplementary Fig. 58). No racemization of enantioenriched benzylic bromide **1a** (63:37 e.r.) was detected in the absence of catalysts. However, racemization of **1a** was observed in the presence of (*S*)-**3f** (10 mol%) and to a lesser extent with Ph_4_P^+^I^−^ (10 mol%). When enantioenriched **1a** was subject to both the HBD and onium catalyst, full racemization occurred (Supplementary Fig. 55).

### Iodide effect

The rate of reaction approximately doubles when Ph_4_P^+^I^−^ is employed in the reaction instead of Ph_4_P^+^Br^−^. In situ ^1^H NMR spectroscopic analysis of the reaction mixture revealed that when Ph_4_P^+^I^−^ is used benzylic iodide **5a** is detected, formed through the nucleophilic displacement of **1a**. Further investigations demonstrated that increasing the amount of Ph_4_P^+^I^−^ at identical (*S*)-**3h** loading (10 mol%) did not increase the concentration of **5a**, suggesting the participation of (*S*)**-3h** to form **5a** through initial solubilization of Ph_4_P^+^I^−^ (Supplementary Figs. 44–46). ^1^H NMR spectroscopic analysis of the periodic activation-mediated^33^ fluorination of bromide **1a** (or **1a-*d***_**1**_) in toluene-*d*_8_ showed that the concentration of iodide **5a** rapidly grows to reach a low and approximately constant concentration (~6.5% of [**1a**]_0_) over the majority of the reaction evolution (Fig. 4c). However, the decay of [**1a**] in this case remained pseudo first order, not pseudo zero order, with the observed rate constant doubled compared with the reaction using Ph_4_P^+^Br^−^ (Supplementary Fig. 42). Thus, the generation of **5a**, which may also undergo nucleophilic attack by [UPF], is not essential for turnover or enantioselectivity, but arises as a consequence of this ion metathesis step, resulting in the formation of a urea–phosphonium bromide complex [UPBr].

### Computational investigations

A density functional theory analysis of [UPF] species ((*S*)-**3f**:Ph_4_P^+^:F^−^) obtained following conformational analysis led to the identification of several low-energy conformations with tridentate binding of (*S*)-**3f** to fluoride, with the phosphonium cation displaying π–π stacking and favourable CH–π interactions with the BINAM backbone. The lowest-energy conformers of the [UPF] complex showed close spatial proximity of the phosphonium to fluoride, with one very close intermolecular CH···F contact (1.91 Å in the most stable conformer), comparable with experimental HOESY NMR data (Supplementary Table 23). Alternative conformations (3.5 and 3.7 kJ mol^−1^ less stable in Gibbs energy than the lowest-energy conformer) were also identified with the phosphonium cation engaging in π–π stacking and CH–π interactions between the urea motifs, positioning the cation behind the tridentate-bound fluoride, leaving the surface of [UPF] open for coordination of a substrate molecule (Supplementary Fig. 60).

Having determined some of the key structural features of the [UPF] complexes, we investigated the catalyst–substrate interactions responsible for enantiodiscrimination. Diastereomeric transition state structures for the reaction of *rac*-**1a** with (*S*)-**3f**-derived [UF] predicted the activation free energies (Δ*G*^‡^) leading to the formation of (*R*)-**2a** and (*S*)-**2a** to be 81.7 and 88.9 kJ mol^−1^, respectively (Fig. 4d). The Curtin–Hammett predicted enantioselectivity (Boltzmann-weighted ΔΔ*G*^‡^ = 5.8 kJ mol^−1^) gives an e.r. of 91:9 at 25 °C, in good agreement with the absolute configuration and level of enantioenrichment obtained experimentally (Fig. 2a). Both competing transition states show stabilization of the benzylic substrate through dispersion-dominated interactions of the substrate with the urea motif of the catalyst. However, a key difference between the major and minor transition state structures is the presence of a strong, direct C_Bn_H–π interaction between the benzylic proton of the substrate and the BINAM backbone of (*S*)-**3f** (Fig. 4d). Computed non-covalent interaction isosurface plots qualitatively reinforce this analysis (Supplementary Fig. 70).

Diastereomeric transition state structures were calculated for the formation of **2h** and **2y**, which were shown experimentally to be more selective. The predicted energy barrier, Δ*G*^‡^, of the lowest-energy transition state decreased from 81.7 kJ mol^−1^ for **2a**, to 75.8 and 75.5 kJ mol^−1^ for **2h** and **2y**, respectively (Supplementary Figs. 66–69). Diastereomeric transition state structures for the reactions of *rac*-**1h** and *rac*-**1y** with (*S*)-**3f** predicted Δ*G*^‡^ leading to the formation of (*R*)-**2h** and (*S*)-**2h** to be 75.8 and 83.0 kJ mol^−1^, and Δ*G*^‡^ leading to the formation of (*R*)-**2y** and (*S*)-**2y** to be 75.5 and 83.6 kJ mol^−1^, respectively. The Curtin–Hammett predicted enantioselectivities for *rac*-**1h** and *rac*-**1y** (Boltzmann-weighted ΔΔ*G*^‡^ = 6.6 and 7.2 kJ mol^−1^, respectively) gave respective e.r. values of 93:7 and 95:5 at 25 °C, in good agreement with the level of enantioenrichment determined experimentally (Fig. 3a). As noted with **2a**, a key difference between the major and minor transition state structures is the presence of strong, direct CH–π interaction between the benzylic proton of the substrate and the BINAM backbone of (*S*)-**3f** (Supplementary Figs. 66–69).

### Proposed catalytic cycle

Together, the data suggest a catalytic cycle that involves initial halogen exchange between *rac*-**1a** and Ph_4_P^+^I^−^ facilitated by (*S*)-**3h** to form benzylic iodide **5a** and the [UPBr] complex (Fig. 4e, I–II). Ion metathesis of [UPBr] with KF occurs to form the [UPF] species (III) and KBr. It is notable that this phase-transfer step is observed spectroscopically from [UPBr] and KF, but not from the [UPI] complex, with the rationale that precipitation of KBr is a thermodynamic driving force in the catalytic cycle (KBr lattice energy = 672 kJ mol^−1^; KI lattice energy = 632 kJ mol^−1^) (Supplementary Figs. 47–50)^22^. Fluoride delivery from [UPF] to either **1a** or **5a** yields enantioenriched benzylic fluoride **2a** irreversibly and regenerates [UPBr] or [UPI], respectively (IV).

## Conclusions

This study reports a catalytic strategy enabling enantioconvergent nucleophilic substitution (S_N_2) of racemic alkyl halides (specifically, benzylic bromides and α-bromoketones) with potassium fluoride, an asymmetric fluorination process rendered possible through the introduction of a second phase-transfer catalyst, an onium halide. The data provide compelling evidence that the onium co-catalyst is essential for phase transfer in fulfilling the ion pairing requirement to solubilize potassium fluoride, together with the urea HBD catalyst, as a well-identified [UPF] species. Extensive mechanistic investigations undertaken with benzylic bromides indicated that both catalysts—but more predominantly the HBD—participate in substrate racemization, with the fluorination proceeding via an S_N_2-like mechanism. Favourable dispersion-dominated interactions between substrates and the [UPF] complex allow for enantioconvergent substitution with fluoride. We anticipate that S-HBPTC will offer new opportunities for fluorination chemistry as the co-catalyst does not necessarily need to be an onium salt and can be selected to meet the specific requirements of the electrophile.

## Methods

### General procedure for the fluorination of benzylic bromides

To a 7 ml screw-cap vial equipped with a magnetic stirring bar we sequentially added pre-ground potassium fluoride (2.5 equiv.), the appropriate substrate (0.16–0.38 mmol; 1 equiv.), (*S*)-**3h** (10 mol%), Ph_4_P^+^I^−^ (10 mol%) and *p-*xylene (0.25 M). The vial was sealed and the reaction was stirred at 1,200 r.p.m. at the appropriate temperature for the specified time. The crude reaction mixture was directly purified by flash column chromatography to give the product. The solvent was removed in perfluoroalkoxy (PFA) round-bottom flasks and the products were stored in polypropylene vials at −20 °C.

### General procedure for the fluorination of α-bromoketones

To a 7 ml screw-cap vial equipped with a magnetic stirring bar we sequentially added pre-ground potassium fluoride (2.5 equiv.), the appropriate substrate (0.4 mmol; 1 equiv.), (*S*)-**3k** (10 mol%), Et_4_N^+^I^−^ (10 mol%) and MeCN (0.5 M). The vial was sealed and the reaction was stirred at 1,200 r.p.m. at the appropriate temperature for 96 h. The crude reaction mixture was directly purified by flash column chromatography to give the product.

## Supplementary information

Supplementary Information Supplementary Figs. 1–70, Tables 1–24 and Equations (1)–(10).

Supplementary Data 1 Coordinates for optimized structures.

## Data Availability

Details on the materials and methods, optimization studies, mechanistic studies, ^1^H, ^13^C and ^19^F NMR spectra and high-resolution spectrometry, and infrared and chiral high-performance liquid chromatography data are available in the Supplementary Information. All other data are available from the authors upon reasonable request.
