# Supplementary material for: Enantioconvergent nucleophilic substitution via synergistic phase-transfer catalysis
Source: Nat Catal. 2025 Feb 13;8(2):107–15. doi: 10.1038/s41929-024-01288-0 (PMC11860226; doi:10.1038/s41929-024-01288-0)
Supplement: Supplementary file 1 — Supplementary Figs. 1–70, Tables 1–24 and Equations (1)–(10). [file 41929_2024_1288_MOESM1_ESM.pdf]

nature catalysis

Supplementary information https://doi.org/10.1038/s41929-024-01288-0

# Enantioconvergent nucleophilic substitution via synergistic phase-transfer catalysis

In the format provided by the authors and unedited

## Table of Contents

**Supplementary Methods** .......... **2**

General Information .......... 2

Reaction Optimisation – Benzylic Halides .......... 4

Substrate Synthesis and Characterisation .......... 10

Urea Catalyst Synthesis and Characterisation .......... 34

General Procedure for Enantioselective Benzylic Fluorination .......... 43

Product Characterisation – Benzylic Fluorides .......... 44

Evaluation of Alternative Electrophiles under S-HBPTC .......... 59

Reaction Optimisation – α-Haloketones .......... 60

Substrate Synthesis and Characterisation – α-Bromoketones .......... 62

General Procedure for Enantioselective Fluorination of α-Bromoketones .......... 66

Product Characterisation – α-Fluoroketones .......... 66

**Non-Linear Effect Study** .......... **72**

**NMR Investigations** .......... **73**

**Kinetic Isotope Effect Experiments** .......... **94**

Kinetic Isotope Effect Predictions .......... 113

**Mechanistic NMR Experiments** .......... **115**

Reaction From Stoichiometric UPF complex .......... 120

Control Reactions from α-Iodoketone substrate .......... 120

**Substrate Racemisation Studies** .......... **121**

**Computational Methods** .......... **127**

**Copies of NMR spectra** .......... **141**

**Copies of HPLC Traces** .......... **303**

**Supplementary References** .......... **350**

## Supplementary Methods

### General Information

Unless stated reagents were purchased from commercial suppliers and used without further purification. Unless stated solvents were used without drying or degassing, all reactions that required anhydrous conditions were performed in flamed-dried glassware under an inert atmosphere of nitrogen and solvents from stills were used. CsF (99.9% trace metal basis from Sigma-Aldrich) was ground prior to reactions and used without pre-drying. KF (99.99% trace metal basis from Alfa Aesar) was ground prior to reactions and used without pre-drying. Reactions were performed in glass; and benzylic fluoride products were isolated in PFA round bottom flasks and stored in polypropylene vials at -20 °C where they remained stable for several months. Reactions were monitored by thin layer chromatography (TLC) supplied by Merck (Kieselgel 60 $F_{254}$ plates). Visualisation of reaction on TLC was accomplished by irradiation with UV light at 254 nm and/or cerium ammonium molybdate (CAM) stain and/or permanganate stain. Flash column chromatography (FCC) was performed on Merck silica gel (60, particle size 0.040-0.062 mm). Optical rotations were measured on an Autopol L 2000 (Schmidt-Haensh) at 589 nm, 25 °C. Data are reported as $[\alpha]^{D}_{25\,°C}$ concentration (c in g/100 mL), and solvent. High resolution mass spectra (HRMS, *m/z*) were recorded on a Thermo Exactive mass spectrometer equipped with Waters Acquity liquid chromatography system using the heated electrospray (HESI-II) probe for positive electrospray ionization ($ESI^+$) or atmospheric pressure chemical ionization (APCI) or on an Agilent 7200 Q-TOF spectrometer equipped with a direct insertion probe supplied by Scientific Instrument Manufacturer (SIM) GmbH using electron ionization (EI – 20eV). Some compounds were found to be unstable under a variety of MS ionization methods (CI, EI, ESI, GC-MS) and therefore no HRMS could be obtained for them; this is stated for the relevant compounds. Infrared spectra were recorder as the neat compound or in liquid solution using a Bruker tensor 27 FT-IR spectrometer, absorptions are reported in wavenumber ($cm^{-1}$) Melting points of solids were measured on a Griffin apparatus and are uncorrected. All enantiomeric ratios (e.r.) were determined by HPLC analysis on a Shimadzu *i*-Prominence LC-2030 (PDA detector), employing a chiral stationary phase, post purification and compared to traces of the racemic mixtures, which were independently prepared. All NMR spectra were recorded on Bruker AVIIIHD 400, AVIIIHD 500, AVII 500 or AVIII HD 600. Deuterated solvents were purchased from Sigma-Aldrich and used without purification. Toluene-$d_8$ and DCM-$d_2$ was stored at 4 °C over 3 Å molecular sieves, under nitrogen atmosphere. NMR spectra are recorded at 298 K, unless otherwise specified. NMR spectra are referenced to the residual solvent peak for $^1$H and $^{13}$C spectra, while $^{19}$F NMR spectra are referenced relative to $CFCl_3$ in $CDCl_3$. Coupling constants, *J*, are reported in Hz to the nearest 0.1 Hz. Unless otherwise stated, $^{13}$C spectra are $^1$H decoupled. The following abbreviations are used to describe peak

multiplicities: s = singlet, d = doublet, t = triplet, q= quartet, sext = sextet, sept = septet, m = multiplet). $^{13}C$ NMR for (*S*)-**3e–j** and (*S*)-**8g–j** recorded with simultaneous proton and fluorine decoupling for clarity. Determination of absolute stereochemistry by comparison of the optical rotation value and chromatographic data for compounds **2ae** and **10a** with reported literature values.[1,2]

## Reaction Optimisation – Benzylic Halides

Supplementary Table 1: Preliminary Optimization

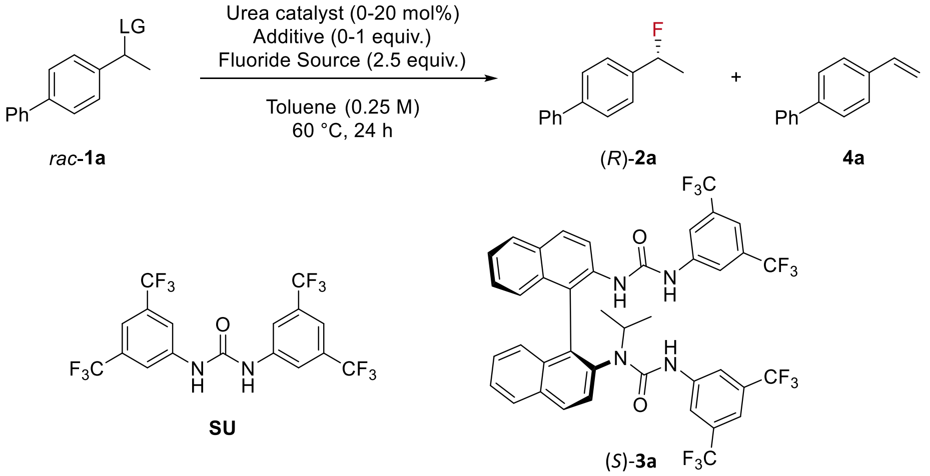


| Entry | Urea Catalyst | LG | Fluoride Source | Additive | **2a** yield (%)[a] | Ratio **2a**: **4a** | **e.r.**[b] |
|---|---|---|---|---|---|---|---|
| **1** | - | Br | KF | - | 0 | - | - |
| **2** | - | Br | CsF | - | 0 | - | - |
| **3** | - | Br | TBAF | - | 48 | 1:1 | - |
| **4** | **SU** (20 mol%) | Br | KF | - | 0 | - | - |
| **5** | **SU** (20 mol%) | Br | CsF | - | 4 | 1:13 | nd |
| **6** | **SU** (20 mol%) | Br | TBAF | - | 52 | 1:1 | 50:50 |
| **7** | (*S*)-**3a** (20 mol%) | Br | KF | - | 0 | - | - |
| **8** | (*S*)-**3a** (20 mol%) | Br | CsF | - | 38 | 1:1 | 74:26 |
| **9** | (*S*)-**3a** (20 mol%) | Br | TBAF | - | 59 | 1:1 | 50:50 |
| **10** | (*S*)-**3a** (20 mol%) | Br | KF | $Bu_4N^+ Br^-$ (20 mol%) | 34 | 7:1 | 73:27 |
| **11** | (*S*)-**3a** (20 mol%) | Br | CsF | $Bu_4N^+ Br^-$ (20 mol%) | 59 | 1:1 | 66:34 |
| **12** | (*S*)-**3a** (20 mol%) | Br | KF | $Ph_4P^+ Br^-$ (20 mol%) | 34 | 9:1 | 75:25 |
| **13** | (*S*)-**3a** (20 mol%) | Br | CsF | $Ph_4P^+ Br^-$ (20 mol%) | 55 | 8:1 | 71:29 |
| **14** | - | Br | KF | $Bu_4N^+ Br^-$ (20 mol%) | 0 | - | - |
| **15** | - | Br | CsF | $Bu_4N^+ Br^-$ (20 mol%) | 0 | - | - |
| **16** | - | Br | KF | $Ph_4P^+ Br^-$ (20 mol%) | 0 | - | - |
| **17** | - | Br | CsF | $Ph_4P^+ Br^-$ (20 mol%) | 0 | - | - |
| **18** | (*S*)-**3a** (10 mol%) | Br | KF | $Ph_4P^+ Br^-$ (10 mol%) | 33 | 6:1 | 75:25 |
| **19** | (*S*)-**3a** (10 mol%) | Br | CsF | $Ph_4P^+ Br^-$ (10 mol%) | 40 | 6:1 | 71:29 |
| **20** | (*S*)-**3a** (10 mol%) | Cl | KF | $Ph_4P^+ Br^-$ (10 mol%) | 16 | 4:1 | 76:24 |
| **21** | (*S*)-**3a** (10 mol%) | Cl | CsF | $Ph_4P^+ Br^-$ (10 mol%) | 4 | 4:1 | nd |

General conditions: Substrate (0.05 mmol), catalyst (10 mol%) and KF (2.5 equiv.) in 200 µL of solvent stirred at 1200 rpm for 24 h at 60 °C [a]Determined by $^{19}F$ NMR using 4-fluoroanisole as internal standard, [b]e.r. was determined by HPLC analysis using a chiral stationary phase, nd = not determined.

Onium Salt Optimisation

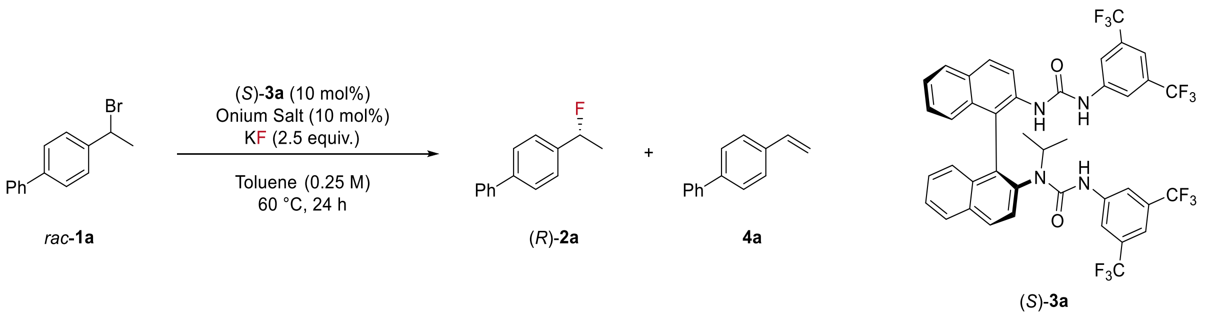


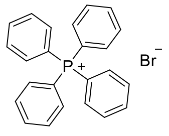


Yield **2a**[a]: **33%**
**4a**: 6%
**76:24 e.r.**

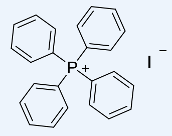


Yield **2a**[a]: **61%**
**4a**: 9%
**77:23 e.r.**

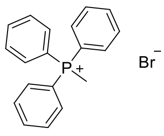


Yield **2a**[a]: **21%**
**4a**: 3%
**74:26 e.r.**

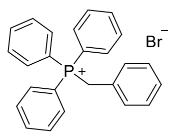


Yield **2a**[a]: **51%**
**4a**: 11%
**76:24 e.r.**

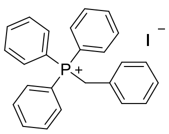


Yield **2a**[a]: **70%**
**4a**: 11%
**76:24 e.r.**

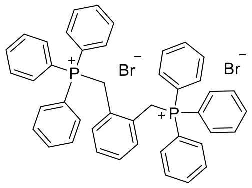


Yield **2a**[a]: **42%**
**4a**: 14%
**77:23 e.r.**

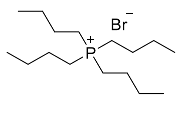


Yield **2a**[a]: **42%**
**4a**: 9%
**73:23 e.r.**

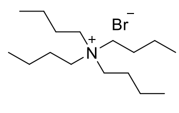


Yield **2a**[a]: **35%**
**4a**: 6%
**70:30 e.r.**

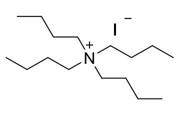


Yield **2a**[a]: **45%**
**4a**: 7%
**71:29 e.r.**

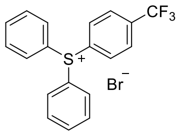


Yield **2a**[a]: **19%**
**4a**: 3%
**70:30 e.r.**

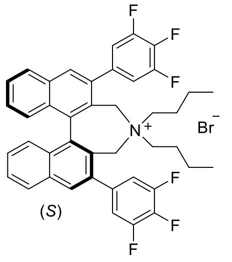


NMR Yield[a]: **54%**
Elimination: 12%
**71:29 e.r.**

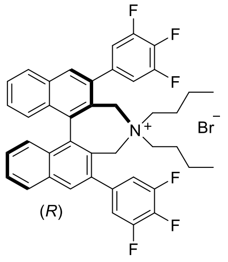


NMR Yield[a]: **61%**
Elimination:11 %
**73:27 e.r.**

**Supplementary Figure 1:** General conditions: Substrate (0.05 mmol), catalysts (10 mol%) and KF (2.25 equiv.) in 200 µL of solvent stirred at 1200 rpm for 24 h at 60 °C [a]Determined by $^{19}$F NMR using 4-fluoroanisole as internal standard, [b]e.r. was determined by HPLC analysis using a chiral stationary phase.

Supplementary Table 2: Phosphonium Iodide Loading

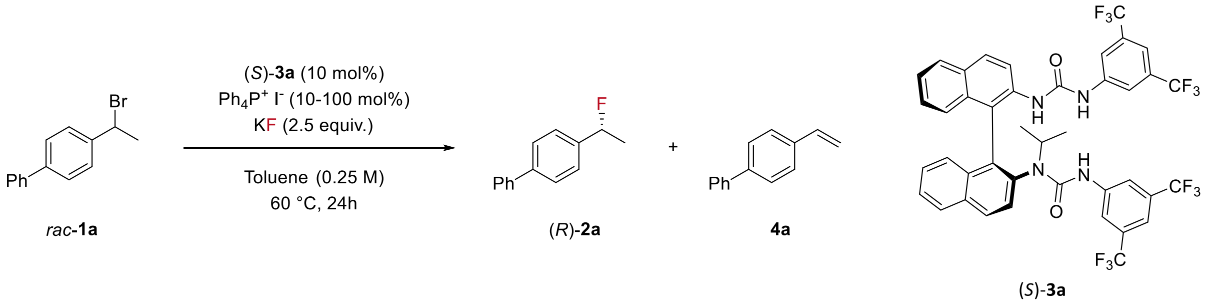


| Entry | $Ph_4P^+ I^-$ (mol%) | **2a** yield (%)[a] | Ratio **2a**: **4a** | **e.r.**[b] |
|---|---|---|---|---|
| **1** | 10 | **61** | **7:1** | **77:23** |
| **2** | 20 | **59** | **7:1** | **77:23** |
| **3** | 50 | **64** | **7:1** | **77:23** |
| **4** | 80 | **62** | **7:1** | **77:23** |
| **5** | 100 | **57** | **7:1** | **77:23** |
| **6** | 200 | **61** | **7:1** | **77:23** |

General conditions: Substrate (0.05 mmol), urea catalyst (10 mol%), $Ph_4P^+ I^-$ (10 – 100 mol%) and KF (2.5 equiv.) in 200 µL of Toluene stirred at 1200 rpm [a]Determined by $^{19}$F NMR using 4-fluoroanisole as internal standard, [b]e.r. was determined by HPLC analysis using a chiral stationary phase. [c]Reactions performed in *p*-xylene as solvent (0.25 M), nd = not determined.

Supplementary Table 3: Urea Catalyst Optimisation

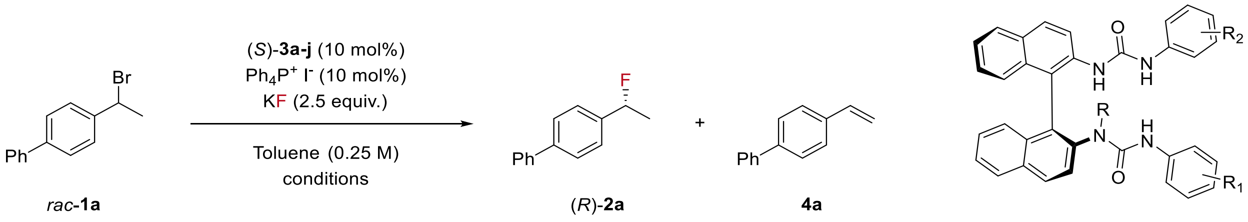


| Entry | Urea Catalyst | Temp/ time | R | R1 | R2 | **2a** yield (%)[a] | Ratio **2a**: **4a** | **e.r.**[b] |
|---|---|---|---|---|---|---|---|---|
| **1** | (*S*)-**3a** | 60 °C/24hr | *i*Pr | 3,5-$CF_3$ | 3,5-$CF_3$ | **61** | 7:1 | **77:23** |
| **2** | (*S*)-**3a** | 40 °C/48hr | *i*Pr | 3,5-$CF_3$ | 3,5-$CF_3$ | **32** | 8:1 | **80:20** |
| **3** | (*S*)-**3b** | 40 °C/48hr | H | 3,5-$CF_3$ | 3,5-$CF_3$ | **0** | - | **nd** |
| **4** | (*S*)-**3c** | 40 °C/48hr | *i*Pr | - | 3,5-$CF_3$ | **6** | 3:1 | **56:44** |
| **5** | (*S*)-**3d** | 40 °C/48hr | *i*Pr | 3,5-$CF_3$ | - | **63** | 6:1 | **83:17** |
| **6** | (*S*)-**3e** | 40 °C/48hr | *i*Pr | 3,5-$CF_3$ | 4-F | **52** | 7:1 | **86:14** |
| **7** | (*S*)-**3f** | 40 °C/48hr | *i*Pr | 3,5-$CF_3$ | 3,4-F | **64** | 9:1 | **87:13** |
| **8**[c] | (*S*)-**3g** | 40 °C/48hr | 3-pent | 3,5-$CF_3$ | 3,4-F | **79** | 10:1 | **87:13** |
| **9**[c] | (*S*)-**3h** | 40 °C/48hr | 4-hept | 3,5-$CF_3$ | 3,4-F | **83** | 10:1 | **87:13** |
| **10**[c] | (*S*)-**3i** | 40 °C/48hr | 5-nona | 3,5-$CF_3$ | 3,4-F | **65** | 8:1 | **87:13** |
| **11**[c] | (*S*)-**3j** | 40 °C/48hr | cyclohex | 3,5-$CF_3$ | 3,4-F | **54** | 9:1 | **87:13** |

General conditions: Substrate (0.05 mmol), urea catalyst (10 mol%), $Ph_4P^+ I^-$ (10 mol%) and KF (2.5 equiv.) in 200 µL of toluene stirred at 1200 rpm [a]Determined by $^{19}F$ NMR using 4-fluoroanisole as internal standard, [b]e.r. was determined by HPLC analysis using a chiral stationary phase. [c]Reactions performed in *p*-xylene as solvent (0.25 M), nd = not determined.

Supplementary Table $: Solvent Optimisation

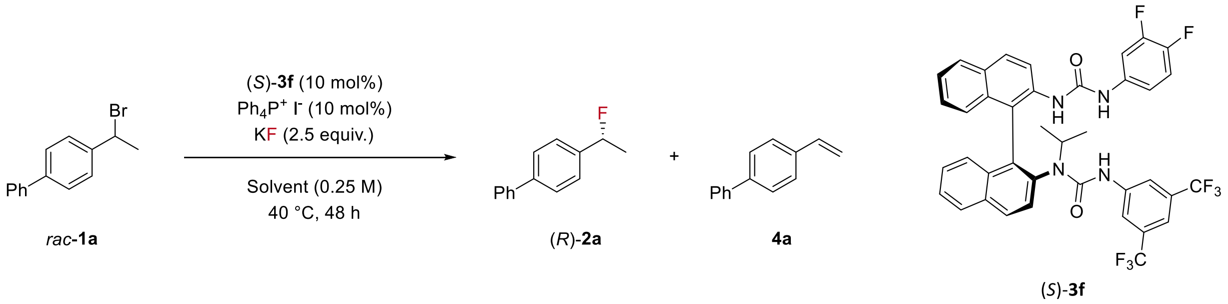


| Entry | Solvent | Dielectric Constant (ε) | **2a** yield (%)[a] | Ratio **2a**: **4a** | **e.r.**[b] |
|---|---|---|---|---|---|
| **1** | Toluene | **2.38** | **64** | 9:1 | **87:13** |
| **2** | 1,2-DFB | **14.26** | **44** | 6:1 | **69:31** |
| **3** | DCE | **10.36** | **24** | 2:1 | **74:26** |
| **4** | $PhCF_3$ | **9.18** | **41** | 7:1 | **81:19** |
| **5** | DCM | **8.93** | **24** | 1:1 | **71:29** |
| **6** | THF | **7.52** | **15** | 4:1 | **78:22** |
| **7** | CPME | **4.76** | **51** | 7:1 | **82:18** |
| **8** | MTBE | **4.50** | **74** | 8:1 | **84:16** |
| **9** | Isopropyl ether | **3.80** | **65** | 7:1 | **83:17** |
| **10** | *o*-xylene | **2.50** | **59** | 7:1 | **86:14** |
| **11** | *m*-xylene | **2.37** | **56** | 8:1 | **87:13** |
| **12** | *p*-xylene | **2.37** | **81** | 10:1 | **87:13** |
| **13** | Benzene | **2.28** | **44** | 8:1 | **84:16** |
| **14** | $CCl_4$ | **1.34** | **66** | 8:1 | **86:14** |

General conditions: Substrate (0.05 mmol), urea catalyst (10 mol%), $Ph_4P^+ I^-$ (10 mol%) and KF (2.5 equiv.) in 200 µL of solvent stirred at 1200 rpm for 48 h at 40 °C [a]Determined by $^{19}F$ NMR using 4-fluoroanisole as internal standard, [b]e.r. was determined by HPLC analysis using a chiral stationary phase.

Supplementary Table 5: Urea Catalyst *N*-alkylation

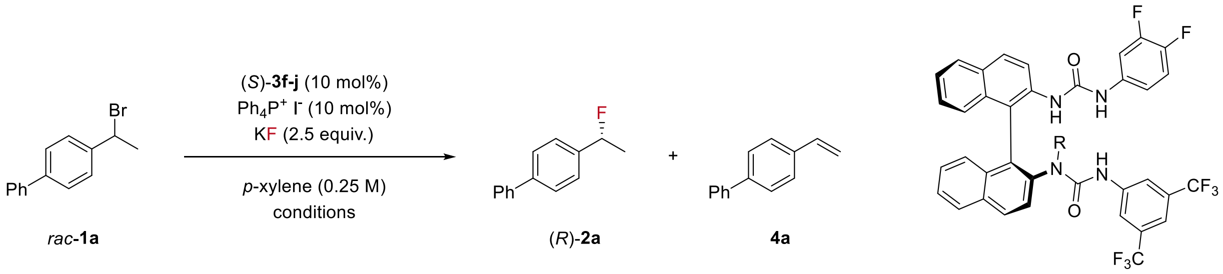


| Entry | Urea Catalyst | R | Temp/ time | **2a** yield (%)[a] | Ratio **2a**: **4a** | **e.r.**[b] |
|---|---|---|---|---|---|---|
| **1** | (*S*)-**3f** | *i*Pr | 40 °C/48hr | **81** | 10:1 | **87:13** |
| **2** | (*S*)-**3f** | *i*Pr | 25 °C/48hr | **35** | 10:1 | **89:11** |
| **3** | (*S*)-**3f** | *i*Pr | 15 °C/72hr | **11** | 6:1 | **92:8** |
| **4** | (*S*)-**3g** | 3-pent | 40 °C/48hr | **79** | 10:1 | **87:13** |
| **5** | (*S*)-**3g** | 3-pent | 25 °C/72hr | **57** | 11:1 | **90:10** |
| **6** | (*S*)-**3g** | 3-pent | 15 °C/72hr | **43** | 14:1 | **92:8** |
| **7** | (*S*)-**3h** | 4-hept | 40 °C/48hr | **83** | 10:1 | **87:13** |
| **8** | (*S*)-**3h** | 4-hept | 25 °C/48hr | **63** | 11:1 | **90:10** |
| **9** | (*S*)-**3h** | 4-hept | 15 °C/72hr | **75** | 15:1 | **92.5:7.5** |
| **10** | (*S*)-**3i** | 5-nona | 40 °C/48hr | **65** | 8:1 | **87:13** |
| **11** | (*S*)-**3i** | 5-nona | 25 °C/48hr | **57** | 11:1 | **90:10** |
| **12** | (*S*)-**3i** | 5-nona | 15 °C/72hr | **36** | 16:1 | **92:8** |
| **13** | (*S*)-**3j** | cyclohex | 40 °C/48hr | **54** | 9:1 | **87:13** |
| **14** | (*S*)-**3j** | cyclohex | 25 °C/48hr | **60** | 12:1 | **90:10** |
| **15** | (*S*)-**3j** | cyclohex | 15 °C/72hr | **47** | 12:1 | **93:7** |

General conditions: Substrate (0.05 mmol), urea catalyst (10 mol%), $Ph_4P^+ I^-$ (10 mol%) and KF (2.5 equiv.) in 200 µL of solvent stirred at 1200 rpm for 48 h or 72 h. [a]Determined by $^{19}F$ NMR using 4-fluoroanisole as internal standard, [b]e.r. was determined by HPLC analysis using a chiral stationary phase.

**Substrate Synthesis and Characterisation**
**Synthesis of Benzylic Bromide Precursors**

General Procedure 1

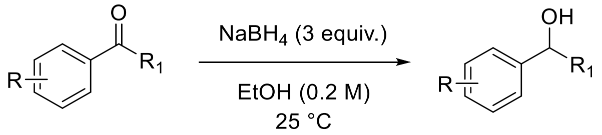


Alcohols were prepared by reduction of corresponding ketone (1 equiv.) with $NaBH_4$ (3 equiv.) in EtOH (0.2 M) at 25 °C. The reactions were monitored by TLC and quenched with $H_2O$ when the ketone was fully consumed. Following work up in EtOAc/$H_2O$ the organic extracts were washed with brine and dried with $MgSO_4$, solvent was removed under reduced pressure to give the corresponding alcohol. When required the mixture was purified by FCC.

General Procedure 2

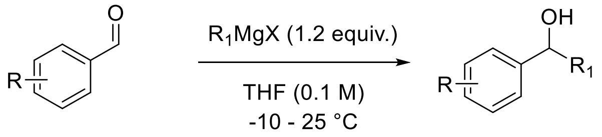


Alcohols were prepared by Grignard addition (1.2 equiv.) to the corresponding aldehyde (1 equiv.) in THF (0.25 M) at -10 °C. The reactions were monitoring by TLC and quenched with saturated $NH_4Cl$ solution when the aldehyde was fully consumed. Following work up in $Et_2O$/$H_2O$ the organic extracts were washed with brine and dried with $MgSO_4$, solvent was removed under reduced pressure to give the corresponding alcohol. When required the mixture was purified by FCC.

Alcohols *rac*-**6a** (CAS: 3562-73-0), *rac*-**6h** (CAS: 1517-72-2), *rac*-**6q** (CAS: 7228-47-9) were commercially available.

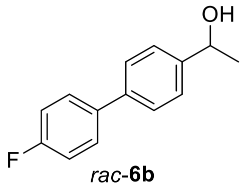


*rac*-**6b**

**1-(4'-fluoro-[1,1'-biphenyl]-4-yl)ethan-1-ol (6b)**

Compound *rac*-**6b** was prepared according to General Procedure 1, the product was isolated as a white solid in in 88 % yield. Spectroscopic data are in accordance with those in literature.[3]

**$^1$H NMR** (400 MHz, $CDCl_3$) δ = 7.59 – 7.49 (m, 4H), 7.49 – 7.41 (m, 2H), 7.18 – 7.07 (m, 2H), 4.96 (q, *J* = 6.4 Hz, 1H), 1.54 (d, *J* = 6.4 Hz, 3H); **$^{19}$F NMR** (377 MHz, $CDCl_3$) δ = -115.8; **$^{13}$C NMR** (126 MHz, $CDCl_3$) δ = 162.6 (d, $J_{CF}$ = 246.3 Hz), 145.0, 139.5, 137.1 (d, $J_{CF}$ = 3.2 Hz), 128.7 (d, $J_{CF}$ = 8.1 Hz), 127.2, 126.0, 115.7 (d, $J_{CF}$ = 21.6 Hz), 70.2, 25.3.

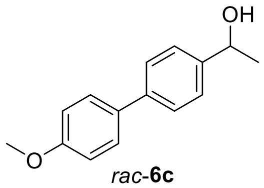
rac-6c

**1-(4’-methoxy-[1,1’-biphenyl]-4-yl)ethan-1-ol (6c)**

Compound *rac*-**6c** was prepared according to General Procedure 1, the product was isolated as a white solid in 75 % yield. Spectroscopic data are in accordance with those in literature.[4]

**$^{1}$H NMR** (400 MHz, $CDCl_3$) δ = 7.58 – 7.48 (m, 4H), 7.46 – 7.39 (m, 2H), 7.04 – 6.94 (m, 2H), 4.94 (q, *J* = 6.5 Hz, 1H), 3.85 (s, 3H), 1.54 (d, *J* = 6.4 Hz, 3H); **$^{13}$C NMR** (101 MHz, $CDCl_3$) δ = 159.3, 144.3, 140.2, 133.5, 128.2, 127.0, 126.0, 114.4, 70.3, 55.5, 25.3.

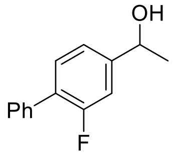
rac-6d

**1-(2-fluoro-[1,1’-biphenyl]-4-yl)ethan-1-ol (6d)**

Compound *rac*-**6d** was prepared according to General Procedure 1, following purification (FCC eluent: 90:10 pentane:EtOAc) the product was isolated as a white solid in 85 % yield. Spectroscopic data are in accordance with those in literature.[5]

**$^{1}$H NMR** (500 MHz, $CDCl_3$) δ = 7.58 – 7.53 (m, 2H), 7.44 (dt, *J* = 14.4, 7.6 Hz, 3H), 7.37 (t, *J* = 7.4 Hz, 1H), 7.24 – 7.17 (m, 2H), 4.94 (q, *J* = 6.5 Hz, 1H), 1.54 (d, *J* = 6.5 Hz, 3H); **$^{19}$F NMR** (377 MHz, $CDCl_3$) δ = -117.7; **$^{13}$C NMR** (126 MHz, $CDCl_3$) δ = 159.9 (d, *J* = 248.4 Hz), 147.6 (d, *J* = 6.9 Hz), 135.7, 130.9 (d, *J* = 3.7 Hz), 129.1 (d, *J* = 2.8 Hz), 128.6, 128.1 (d, $J_{CF}$ = 13.6 Hz), 127.8, 121.4 (d, $J_{CF}$ = 3.3 Hz), 113.2 (d, $J_{CF}$ = 23.6 Hz), 69.7 (d, $J_{CF}$ = 1.5 Hz), 25.3.

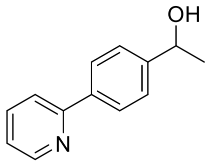
rac-6e

**1-(4-(pyridine-2-yl)phenyl)ethan-1-ol (6e)**

Compound *rac*-**6e** was prepared according to General Procedure 1, following purification (FCC eluent: 95:5 – 80:20 pentane: EtOAc) the product was isolated as a white solid in 72 % yield.

**$^{1}$H NMR** (400 MHz, $CDCl_3$) δ = 8.68 (dt, *J* = 5.0, 1.5 Hz, 1H), 8.00 – 7.93 (m, 1H), 7.81 – 7.68 (m, 2H), 7.60 – 7.54 (m, 1H), 7.46 (dd, *J* = 12.0, 8.3 Hz, 2H), 7.28 – 7.19 (m, 1H), 5.01 – 4.90 (m, 1H), 1.53 (d, *J* = 6.5, 3H); **$^{13}$C NMR**[a] (101 MHz, $CDCl_3$) δ = 157.2, 149.6, 146.9, 145.0, 140.2, 137.1, 127.3 (minor), 127.3 (major), 126.0 (minor), 126.0 (major), 122.3, 120.7, 70.3, 25.4 (major), 25.3 (minor); **HRMS** ($ESI^+$) m/z calculated for $C_{13}H_{13}NO$ $(M+H)^+$ 200.1070, found 200.1068; **IR** (neat) ν = 3271, 2971, 2925, 2867, 1591, 1561, 1473, 1436, 1403, 1363, 1344, 1288, 1207, 1158, 1121, 1091, 1061, 1035, 1013, 994, 907, 850, 519, 785, 752, 736, 721, 626 $cm^{-1}$; **mp** 109-110 ˚C. [a]Mixture of rotamers reported.

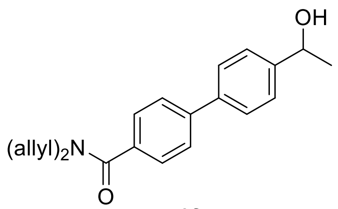
rac-6f

***N,N*-diallyl-4’-(1-hydroxyethyl)-[1,1’biphenyl]-4-carboxamide (6f)**

Compound *rac*-**6f** was prepared according to General Procedure 1, following purification (FCC eluent: 90:10 – 70:30 pentane:EtOAc) the product was isolated as a viscous pale yellow oil in 81 % yield.

**$^{1}$H NMR** (400 MHz, $CDCl_3$) δ = 7.54 (ddd, *J* = 13.2, 8.3, 2.2 Hz, 4H), 7.47 (dd, *J* = 8.3, 2.1 Hz, 2H), 7.41 (dd, *J* = 8.3, 2.3 Hz, 2H), 5.87 (br s, 1H), 5.76 (br s, 1H), 5.28 – 5.16 (m, 4H), 4.90 (qd, *J* = 6.4, 2.9 Hz, 1H), 4.16 – 4.07 (m, 2H), 3.88 (s, 2H), 1.50 (dd, *J* = 6.5, 2.4 Hz, 3H); **$^{13}$C**

NMR (101 MHz, $CDCl_3$) [overlapping signals] δ = 171.7, 145.8, 142.3, 139.1, 134.8, 133.2, 132.8, 127.2, 127.1, 127.0, 126.0, 117.8, 69.8, 50.9, 47.2, 25.3; **HRMS** ($ESI^+$) m/z calculated for $C_{21}H_{23}BrNO$ $(M+H)^+$ 384.0958, found 384.0955; **IR** (neat) ν = 3373, 3082, 3031, 2983, 2926, 2243, 1915, 1620, 1525, 1511, 1414, 1365, 1344, 1261, 1209, 1187, 1114, 1091, 1005, 910, 859, 827, 790, 758, 732, 647, 637, 628, 621 $cm^{-1}$.

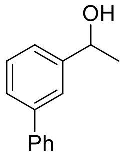

*rac*-**6g**

**1-([1,1'-biphenyl]-3-yl)ethan-1-ol (6g)**

Compound *rac*-**6g** was prepared according to General Procedure 1, the product was isolated as a white solid in 92 % yield. Spectroscopic data are in accordance with those in literature.[6]

**$^1$H NMR** (400 MHz, $CDCl_3$) δ = 7.68 (dd, *J* = 7.9, 1.4 Hz, 1H), 7.47 – 7.27 (m, 7H), 7.22 (dd, *J* = 7.6, 1.4 Hz, 1H), 4.99 (q, *J* = 6.0 Hz, 1H), 1.42 (d, *J* = 6.4 Hz, 3H); **$^{13}$C NMR** (101 MHz, $CDCl_3$) δ = 143.2, 141.1, 140.5, 130.1, 129.4, 128.3, 128.1, 127.3, 127.2, 125.5, 66.6, 25.0.

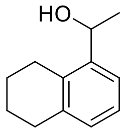

*rac*-**6i**

**1-(5,6,7,8-tetrahydronaphthalen-1-yl)ethan-1-ol (6i)**

Compound *rac*-**6i** was prepared according to General Procedure 2 from 5,6,7,8-tetrahydronaphalene-1-carbaldehyde and MeMgBr, following purification (FCC eluent: 95:5 – 80:20 pentane:$Et_2O$) the product was isolated as an off-white solid in 64 % yield.

**$^1$H NMR** (400 MHz, $CDCl_3$) δ 7.37 (d, *J* = 7.6 Hz, 1H), 7.16 (t, *J* = 7.6 Hz, 1H), 7.01 (d, *J* = 7.5 Hz, 1H), 5.14 (q, *J* = 6.4 Hz, 1H), 2.90 – 2.77 (m, 3H), 2.66 (dt, *J* = 16.7, 6.5 Hz, 1H), 1.89 – 1.72 (m, 4H), 1.46 (d, *J* = 6.4 Hz, 3H); **$^{13}$C NMR** (101 MHz, $CDCl_3$) δ = 144.0, 137.5, 133.5, 128.7, 125.9, 121.9, 66.2, 30.3, 25.7, 24.1, 23.4, 22.8; **IR** (neat) ν = 3347, 2980, 2930, 2589, 1451, 1369, 1266, 1108, 1066, 1014, 903, 831, 779, 737 $cm^{-1}$; No HRMS obtained.

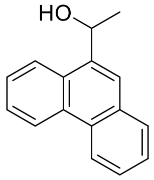

*rac*-**6j**

**1-(phenanthrene-9-yl)ethan-1-ol (6j)**

Compound *rac*-**6j** was prepared according to General Procedure 2 from phenanthrene-9-carbaldehyde and MeMgCl, the product was isolated as a white solid in 95 % yield.

**$^1$H NMR** (400 MHz, $CDCl_3$) δ = 8.46 – 8.36 (m, 1H), 8.36 – 8.28 (m, 1H), 7.86 – 7.75 (m, 1H), 7.59 (s, 1H), 7.58 – 7.50 (m, 1H), 7.39 – 7.21 (m, 4H), 5.33 (qd, *J* = 6.4, 0.9 Hz, 1H), 1.39 (d, *J* = 6.4 Hz, 3H); **$^{13}$C NMR** (101 MHz, $CDCl_3$) δ = 139.6, 131.6, 130.9, 130.1, 129.7, 128.9, 126.9, 126.7, 126.7, 126.4, 124.0, 123.5, 122.8, 122.6, 67.3, 24.2; **HRMS** ($ESI^+$) m/z calculated for $C_{16}H_{15}O$ $(M+H)^+$ 223.1117, found 223.0649; **IR** (neat) ν = 3269, 2981, 2955, 2924, 2853, 1691, 1635, 1608, 1484, 1455, 1392, 1373, 1269, 1250, 1176, 1077, 910, 726 $cm^{-1}$; **mp** 120-123 ˚C.

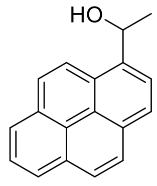

*rac*-**6k**

**1-(pyren-1-yl)ethan-1-ol (6k)**

Compound *rac*-**6j** ws prepared according to General Procedure 1, the product was isolated as an off-white solid in 92 % yield. Spectroscopic data are in accordance with those in literature.[7]

**$^1$H NMR** (500 MHz, $CDCl_3$) δ = 8.36 (d, *J* = 9.3 Hz, 1H), 8.25 (d, *J* = 8.0 Hz, 1H), 8.23 – 8.16 (m, 3H), 8.12 (d, *J* = 9.3 Hz, 1H), 8.05 (s, 2H), 8.01 (t, *J* = 7.6 Hz, 1H), 6.01 (q, *J* = 6.5 Hz, 1H), 1.79 (d, *J* = 6.5 Hz, 3H); **$^{13}$C NMR** (126 MHz, $CDCl_3$) δ = 139.2, 131.5, 130.8, 127.8, 127.6, 127.4, 127.4, 126.1, 125.4, 125.3, 125.2, 125.1, 125.0, 122.7, 122.6, 67.5, 25.2.

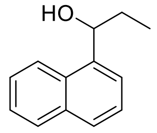

*rac*-**6l**

**1-(napthalen-1-yl)propan-1-ol (6l)**

Compound *rac*-**6k** was prepared according to General Procedure 2 from 1-napthaldehyde and EtMgBr, following purification (FCC eluent: 50:50 – 100:0 DCM:pentane) the product was isolated as viscous colourless oil in 87 % yield. Spectroscopic data are in accordance with those in literature.[8]

**$^1$H NMR** (400 MHz, $CDCl_3$) δ = 8.17 – 8.08 (m, 1H), 7.93 – 7.84 (m, 1H), 7.79 (dt, *J* = 8.2, 1.1 Hz, 1H), 7.64 (dt, *J* = 7.1, 1.0 Hz, 1H), 7.57 – 7.43 (m, 3H), 5.40 (td, *J* = 6.0, 2.1 Hz, 1H), 2.05 – 2.02 (m, 1H), 2.03 – 1.87 (m, 2H), 1.04 (t, *J* = 7.4 Hz, 3H); **$^{13}$C NMR** (101 MHz, $CDCl_3$) δ = 140.4, 134.0, 130.6, 129.0, 128.0, 126.1, 125.6, 125.5, 123.4, 123.0, 72.7, 31.2, 10.7.

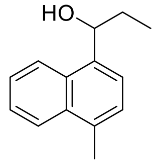

*rac*-**6m**

**1-(4-methylnaphthalen-1-yl)propan-1-ol (6m)**

Compound *rac*-**6l** was prepared according to General Procedure 2 from 4-methyl-1-napthaldehyde and EtMgBr, following purification (FCC eluent: 100% DCM) the product was isolated as a viscous colourless oil in 79 % yield.

**$^1$H NMR** (400 MHz, $CDCl_3$) δ = 8.15 (dt, *J* = 7.8, 2.7 Hz, 1H), 8.09 – 8.00 (m, 1H), 7.60 – 7.48 (m, 3H), 7.33 (dd, *J* = 7.3, 1.1 Hz, 1H), 5.38 (dd, *J* = 7.6, 5.0 Hz, 1H), 2.70 (d, *J* = 0.9 Hz, 3H), 2.10 – 1.85 (m, 2H), 1.04 (t, *J* = 7.4 Hz, 3H); **$^{13}$C NMR** (101 MHz, $CDCl_3$) δ = 138.5, 134.1, 133.0, 130.8, 126.3, 125.7, 125.5, 125.1, 123.9, 122.8, 72.7, 31.2, 19.8, 10.7; **HRMS** ($ESI^+$) m/z calculated for $C_{14}H_{17}O$ $(M+H)^+$ 201.1274, 201.1094; **IR** (neat) ν = 3375, 3074, 2966, 2932, 2875, 1598, 1516, 1457, 1381, 1244, 1216, 1164, 1035, 979, 837, 759 $cm^{-1}$.

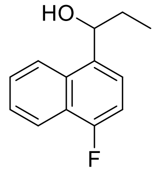

*rac*-**6n**

**1-(4-fluoronaphthalen-1-yl)propan-1-ol (6n)**

Compound *rac*-**6m** was prepared according to General Procedure 2 from 4-fluoro-1-napthaldehyde and EtMgBr, following purification (FCC eluent: 100% DCM) the product was isolated as a viscous colourless oil in 78 % yield.

**$^1$H NMR** (400 MHz, $CDCl_3$) δ = 8.19 – 8.07 (m, 2H), 7.61 – 7.49 (m, 3H), 7.12 (dd, *J* = 10.3, 8.0 Hz, 1H), 5.31 (dd, *J* = 7.6, 5.2 Hz, 1H), 2.06 – 1.82 (m, 3H), 1.01 (t, *J* = 7.4 Hz, 3H); **$^{19}$F NMR** (377 MHz, $CDCl_3$) δ = -124.0; **$^{13}$C NMR** (101 MHz, $CDCl_3$) δ = 158.4 (d, *J* = 251.3 Hz), 136.1 (d, *J* = 4.4 Hz), 131.9 (d, *J* = 4.2

Hz), 127.0, 125.9 (d, *J* = 2.0 Hz), 124.0 (d, *J* = 16.0 Hz), 123.4 (d, *J* = 2.8 Hz), 123.1 (d, *J* = 8.4 Hz), 121.4 (d, *J* = 6.0 Hz), 108.9 (d, *J* = 19.7 Hz), 72.5, 31.2, 10.6; **HRMS** ($ESI^+$) m/z calculated for $C_{12}H_{11}FO$ $(M+H)^+$ 191.0867, found 191.0773; **IR** (neat) ν = 3365, 3072, 2970, 2935, 2876, 1635, 1604, 1585, 1513, 1465, 1396, 1322, 1261, 1143, 1049, 966, 835, 761, 710 $cm^{-1}$.

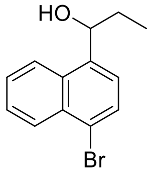
*rac*-**6o**

**1-(4-bromonaphthalen-1-yl)propan-1-ol (6o)**

Compound *rac*-**6n** was prepared according to General Procedure 2 from 4-bromo-1-napthaldehyde and EtMgBr, following purification (FCC eluent: 95:5-80:20 pentane:$Et_2O$) the product was isolated as a white solid in 76 % yield.

**$^1$H NMR** (500 MHz, $CDCl_3$) δ = 8.31 (dd, *J* = 8.5, 1.4 Hz, 1H), 8.10 – 8.06 (m, 1H), 7.77 (d, *J* = 7.8 Hz, 1H), 7.59 (dddd, *J* = 25.7, 8.3, 6.8, 1.3 Hz, 2H), 7.47 (d, *J* = 7.8 Hz, 1H), 5.35 (dd, *J* = 7.6, 4.8 Hz, 1H), 2.02 – 1.83 (m, 2H), 1.02 (t, *J* = 7.4 Hz, 3H); **$^{13}$C NMR** (126 MHz, $CDCl_3$) δ = 140.5, 132.1, 131.8, 129.7, 128.2, 127.0, 126.8, 123.67 123.6, 122.6, 72.4, 31.3, 10.5; **HRMS** ($ESI^+$) m/z calculated for $C_{13}H_{14}BrO$ $(M+H)^+$ 265.0223, found 264.9896; **IR** (liquid film) ν = 3207, 3123, 2931, 1944, 1915, 1868, 1834, 1795, 1594, 1567, 1509, 1455, 1367, 1250, 1202, 1106, 1047, 972, 911, 876, 725, 802, 773, 750, 697, 648 $cm^{-1}$; **mp** 67-68 ˚C.

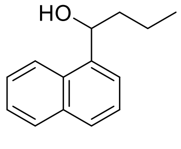
*rac*-**6p**

**1-(naphthalen-1-yl)butan-1-ol (6p)**

Compound *rac*-**6p** was prepared according to General Procedure 2 from 1-napthaldehyde and *n*PropylMgBr, following purification, (FCC eluent: 95:5 – 85:15 pentane:$Et_2O$) the product was isolated as viscous colourless oil in 75 % yield.

**$^1$H NMR** (400 MHz, $CDCl_3$) δ = 8.16 – 8.07 (m, 1H), 7.94 – 7.84 (m, 1H), 7.79 (dt, *J* = 8.2, 1.1 Hz, 1H), 7.63 (dt, *J* = 7.2, 1.0 Hz, 1H), 7.58 – 7.42 (m, 3H), 5.44 (dd, *J* = 7.6, 5.1 Hz, 1H), 2.00 – 1.82 (m, 2H), 1.66 – 1.38 (m, 2H), 0.99 (t, *J* = 7.4 Hz, 3H); **$^{13}$C NMR** (101 MHz, $CDCl_3$) δ = 140.7, 133.9, 130.5, 129.0, 127.9, 126.0, 125.5, 125.5, 123.3, 122.9, 71.0, 40.6, 19.5, 14.1; **HRMS** ($ESI^+$) m/z calculated for $C_{14}H_{17}O$ $(M+H)^+$ 201.1274, found 201.1073; **IR** (liquid film) ν = 3585, 3050, 2957, 2872, 1942, 1834, 1511, 1457, 1395, 1353, 1261, 1229, 1168, 1120, 1081, 1062, 1029, 909, 860, 800, 778, 734, 633 $cm^{-1}$.

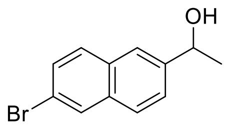
*rac*-**6r**

**1-(6-bromonaphthalen-2-yl)ethan-1-ol (6r)**

Compound *rac*-**6r** was prepared according to General Procedure 1, following purification (FCC eluent: 95:5 – 80:20 pentane:$Et_2O$) the product was isolated as an off-white solid in 97 % yield. Spectroscopic data are in accordance with those in literature.[9]

**$^1$H NMR** (500 MHz, $CDCl_3$) δ = 7.74 (d, *J* = 1.9 Hz, 1H), 7.54 – 7.46 (m, 2H), 7.43 (d, *J* = 8.7 Hz, 1H), 7.33 – 7.24 (m, 2H), 4.80 (q, *J* = 6.5 Hz, 1H), 1.33 (d, *J* = 6.5 Hz, 3H); **$^{13}$C NMR** (126 MHz, $CDCl_3$) δ = 143.8, 134.0, 131.8, 129.8, 129.7, 129.6, 127.5, 125.0, 123.8, 119.8, 70.4, 25.3.

**1-(naphthalen-2-yl)propan-1-ol (6s)**

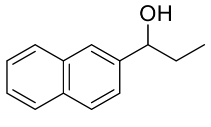

*rac*-**6s**

Compound *rac*-**6k** was prepared according to General Procedure 2 from 2-naphthaldehyde and EtMgBr, following purification (FCC eluent: 90:10 – 80:20 pentane: EtOAc) the product was isolated as a white solid in 62 % yield. Spectroscopic data are in accordance with those in literature.[10]

**$^1$H NMR** (400 MHz, $CDCl_3$) δ = 7.87 – 7.81 (m, 3H), 7.80 – 7.77 (m, 1H), 7.53 – 7.42 (m, 3H), 4.78 (td, *J* = 6.6, 3.1 Hz, 1H), 1.96 (d, *J* = 3.2 Hz, 1H), 1.95 – 1.78 (m, 2H), 0.95 (t, *J* = 7.4 Hz, 3H); **$^{13}$C NMR** (101 MHz, $CDCl_3$) δ = 142.1, 133.4, 133.1, 128.4, 128.1, 127.8, 126.3, 125.9, 124.9, 124.3, 76.3, 31.9, 10.3.

**1-(4-(4,4,5,5-tetramethyl-1,3,2-dioxaborolan-2-yl)phenyl)ethan-1-ol (6t)**

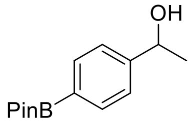

*rac*-**6t**

Compound *rac*-**6t** was prepared according to General Procedure 2, from 4-(4,4,5,5-Tetramethyl-1,3,2-dioxaborolan-2-yl)-benzaldehyde and MeMgBr, following purification (FCC eluent: 100:0 – 90:10 DCM:EtOAc) product was isolated as a white solid in 59 % yield. Spectroscopic data are in accordance with those in literature.[11]

**$^1$H NMR** (400 MHz, $CDCl_3$) δ = 7.83 – 7.76 (m, 2H), 7.41 – 7.33 (m, 2H), 4.91 (q, *J* = 6.4 Hz, 1H), 1.48 (d, *J* = 6.5 Hz, 3H), 1.34 (s, 12H); **$^{13}$C NMR** (101 MHz, $CDCl_3$) δ = 149.1, 135.2, 124.8, 83.9, 70.5, 25.3, 25.0. (*ipso*-carbon bound to boron not observed by $^{13}$C NMR)

**1-(4-((trimethylsilyl)ethynyl)phenyl)propan-1-ol (6u)**

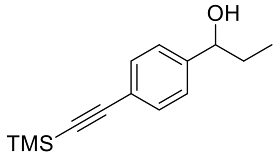

*rac*-**6u**

Compound *rac*-**6u** was prepared according to General Procedure 2 from 4-[(trimethylsilyl)ethynyl]benzaldehyde and EtMgBr Following purification (FCC eluent: 90:10 – 80:20 pentane: $Et_2O$) product was isolated as a colourless oil in 82 % yield.

**$^1$H NMR** (400 MHz, $CDCl_3$) δ = 7.47 – 7.42 (m, 2H), 7.30 – 7.24 (m, 2H), 4.59 (t, *J* = 6.5 Hz, 1H), 1.83 – 1.68 (m, 2H), 0.89 (t, *J* = 7.4 Hz, 3H), 0.25 (s, 9H); **$^{13}$C NMR** (101 MHz, $CDCl_3$) δ = 145.1, 132.2, 125.9, 122.3, 105.1, 94.2, 75.7, 32.0, 10.1, 0.1; **HRMS** ($ESI^+$) m/z calculated for $C_{14}H_{21}OSi$ $(M+H)^+$ 233.1356, found 233.1509; **IR** (neat) ν = 3375, 2963, 2934, 2876, 2158, 1724, 1566, 1503, 1457, 1411, 1379, 1250, 1223, 1200, 1097, 1044, 1016, 976, 864, 842, 760, 702, 650, 639, 625 $cm^{-1}$.

**4-(1-hydroxyethyl)phenyl-4-methylbenzenesulfonate (6v)**

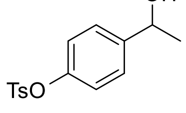

*rac*-**6v**

Compound *rac*-**6v** was prepared according to General Procedure 1, following purification (FCC eluent: 90:10 – 80:20 pentane: $Et_2O$) product was isolated as an off-white solid in 74 % yield. Spectroscopic data are in accordance with those in literature.[12]

**$^1$H NMR** (400 MHz, $CDCl_3$) δ = 7.74 – 7.68 (m, 2H), 7.34 – 7.25 (m, 4H), 6.99 – 6.91 (m, 2H), 4.87 (q, *J* = 6.5 Hz, 1H), 2.45 (s, 3H), 1.83 (s, 1H), 1.45 (dd, *J* = 6.5, 0.7 Hz, 3H); **$^{13}$C NMR** (101 MHz, $CDCl_3$) δ = 148.9, 145.5, 144.8, 132.6, 129.9, 128.6, 126.8, 122.5, 69.8, 25.4, 21.9.

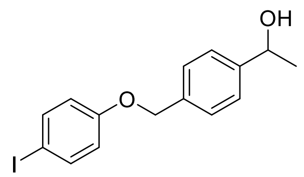

*rac*-**6w**

**1-(4-((4-iodophenoxy)methyl)phenyl)ethan-1-ol (6w)**

Compound *rac*-**6w** was prepared according to General Procedure 1, yielding product as white solid in 93 % yield.

**$^{1}$H NMR** (500 MHz, $CDCl_3$) δ = 7.59 – 7.52 (m, 2H), 7.40 (s, 4H), 6.78 – 6.71 (m, 2H), 5.02 (s, 2H), 4.92 (q, *J* = 6.5 Hz, 1H), 1.59 (br s, 1H), 1.50 (d, *J* = 6.5 Hz, 3H); **$^{13}$C NMR** (151 MHz, $CDCl_3$) δ = 158.7, 145.9, 138.4, 135.9, 127.8, 125.9, 117.4, 83.2, 70.3, 70.0, 25.4; **HRMS** ($ESI^+$) m/z calculated for $C_{15}H_{16}IO_2$ $(M+H)^+$ 355.0190, found 355.0681; **IR** (neat) ν = 2962, 2927, 2854, 1776, 1702, 1587, 1486, 1464, 1400, 1383, 1339, 1282, 1246, 1177, 1072, 1014, 997, 872, 832, 809, 760 cm$^{-1}$; **mp** 110-112 ˚C.

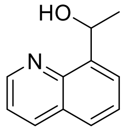

*rac*-**6x**

**1-(quinoline-8-yl)ethan-1-ol (6x)**

Compound *rac*-**6x** was prepared according to General Procedure 2 from quinoline-8-carbaldehyde and MeMgBr, following purification (FCC eluent: 90:10 – 80:20 pentane: EtOAc) affording product as a yellow viscous oil in 80 % yield.

**$^{1}$H NMR** (400 MHz, $CDCl_3$) δ = 8.85 (dd, *J* = 4.3, 1.8 Hz, 1H), 8.19 (dd, *J* = 8.3, 1.8 Hz, 1H), 7.73 (dd, *J* = 8.1, 1.5 Hz, 1H), 7.59 (ddd, *J* = 7.1, 1.6, 0.7 Hz, 1H), 7.50 (dd, *J* = 8.1, 7.1 Hz, 1H), 7.43 (dd, *J* = 8.3, 4.2 Hz, 1H), 5.47 (q, *J* = 6.6 Hz, 1H), 1.74 (d, *J* = 6.6 Hz, 3H); **$^{13}$C NMR** (101 MHz, $CDCl_3$) δ = 148.5, 146.6, 142.0, 137.2, 128.8, 127.2, 126.6, 126.5, 121.0, 70.3, 24.3; **HRMS** ($ESI^+$) m/z calculated for $C_{11}H_{12}NO$ $(M+H)^+$ 174.0913, found 174.0912; **IR** (liquid film) ν = 3373, 3054, 2969, 2922m 1616, 1596, 1580, 1500, 1418, 1367, 1326, 1283, 1242, 1168, 1114, 1069, 1055, 1020, 1001, 988, 901, 831, 794, 763, 667, 636, 621 cm$^{-1}$.

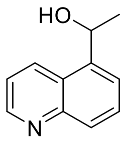

*rac*-**6y**

**1-(quinoline-5-yl)ethan-1-ol (6y)**

Compound *rac*-**6y** was prepared according to General Procedure 2 from quinoline-5-carbaldehyde and MeMgBr, following purification (FCC eluent: 100:0 -90:10 DCM:EtOAc) the product was isolated as a white solid in 87 % yield.

**$^{1}$H NMR** (400 MHz, $CDCl_3$) δ = 8.79 (dd, *J* = 4.2, 1.7 Hz, 1H), 8.51 (ddd, *J* = 8.7, 1.7, 0.9 Hz, 1H), 7.96 (dt, *J* = 8.0, 1.2 Hz, 1H), 7.67 – 7.62 (m, 2H), 7.35 (dd, *J* = 8.6, 4.2 Hz, 1H), 5.57 (q, *J* = 6.3 Hz, 1H), 1.65 (d, *J* = 6.5 Hz, 3H); **$^{13}$C NMR** (101 MHz, $CDCl_3$) δ = 149.9, 148.5, 142.0, 132.3, 129.2, 129.1, 125.7, 123.0, 120.8, 67.2, 24.7; **HRMS** ($ESI^+$) m/z calculated for $C_{11}H_{11}NO$ $(M+H)^+$ 174.0913, found 174.0913; **IR** (liquid film) ν = 3170, 2987, 2973, 2873, 1742, 1505, 1448, 1365, 1313, 1284, 1245, 1170, 1119, 1072, 1011, 905, 833, 805, 716 cm$^{-1}$; **mp** 95-96 ˚C.

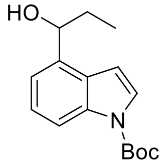

*rac*-**6z**

***tert*-butyl-4-(1-hydroxypropyl)-1*H*-indole-1-carboxylate (6z)**

Compound *rac*-**6z** was prepared according to General Procedure 2 from *tert*-butyl-4-formyl-1*H*-indole-1-carboxylate and EtMgBr. Following purification (FCC eluent: 95:5-70:30 pentane:$Et_2O$) the product was isolated as a viscous colourless oil in 62 % yield.

**$^{1}$H NMR** (400 MHz, $CDCl_3$) δ = 8.11 (d, *J* = 8.2 Hz, 1H), 7.62 (d, *J* = 3.8 Hz, 1H), 7.31 (t, *J* = 7.9 Hz, 1H), 7.25 – 7.23 (m, 1H), 6.75 (dd, *J* = 3.8, 0.8 Hz, 1H), 4.95 (t, *J* = 6.6 Hz, 1H), 1.96 (ddt, *J* = 15.1, 13.6, 7.3 Hz, 2H), 1.70 (s, 9H), 0.94 (t, *J* = 7.4 Hz, 3H); **$^{13}$C NMR** (101 MHz, $CDCl_3$) δ = 149.8, 136.9, 135.5, 128.1, 125.7, 124.2, 119.6, 114.4, 105.8, 83.8, 74.4, 31.2, 28.2, 10.4; **HRMS** ($ESI^+$) m/z calculated for $C_{16}H_{22}NO_3$ $(M+H)^+$ 276.1594, found 276.1591; **IR** (liquid film) ν = 2981, 2964, 2933, 2873, 1734, 1602, 1538, 1481, 1457, 1430, 1386, 1347, 1283, 1157, 1134, 1105, 1045, 910, 853, 761, 733 $cm^{-1}$.

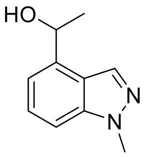
*rac*-**6aa**

**1-(1-methyl-1*H*-indazol-4-yl)ethan-1-ol (6aa)**

Compound *rac*-**6aa** was prepared according to General Procedure 2 from 1-methyl-1*H*-indazole-4-carbaldehyde and MeMgBr, following purification (FCC eluent: 90:10-60:40 DCM:EtOAc) the product was isolated as a colourless oil in 69 % yield.

**$^{1}$H NMR** (400 MHz, $CDCl_3$) δ = 8.11 (d, *J* = 1.0 Hz, 1H), 7.41 – 7.27 (m, 2H), 7.13 (dt, *J* = 6.9, 0.9 Hz, 1H), 5.29 (q, *J* = 6.5, 1H), 4.07 (s, 3H), 1.63 (d, *J* = 6.5 Hz, 3H); **$^{13}$C NMR** (101 MHz, $CDCl_3$) δ = 140.5, 139.5, 131.8, 126.5, 121.4, 116.4, 108.4, 69.6, 35.7, 24.9; **HRMS** ($ESI^+$) m/z calculated for $C_{10}H_{13}N_2O$ $(M+H)^+$ 177.1022, found 177.1023; **IR** (liquid film) ν = 3367, 2947, 2877, 2829, 1701, 1613, 1510, 1449, 1411, 1271, 1203, 1165, 1110, 1080, 1013, 964, 899, 850, 789, 745, 631 $cm^{-1}$.

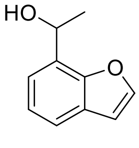
*rac*-**6z**

**1-(benzofuran-7-yl)ethan-1-ol (6ab)**

Compound *rac*-**6ab** was prepared according to General Procedure 2 from benzofuran-7-carbaldehyde and MeMgBr, following purification (FCC eluent: 100% DCM) the product was isolated as a colourless oil in 71 % yield.

**$^{1}$H NMR** (500 MHz, $CDCl_3$) δ = 7.63 (d, *J* = 2.2 Hz, 1H), 7.52 (dd, *J* = 7.7, 1.3 Hz, 1H), 7.33 (dt, *J* = 7.3, 1.0 Hz, 1H), 7.23 (t, *J* = 7.6 Hz, 1H), 6.78 (d, *J* = 2.2 Hz, 1H), 5.37 (q, *J* = 6.5 Hz, 1H), 2.51 (s, 1H), 1.65 (d, *J* = 6.5 Hz, 3H); **$^{13}$C NMR** (126 MHz, $CDCl_3$) δ = 151.9, 144.8, 129.6, 127.6, 123.1, 120.7, 120.4, 106.8, 66.4, 23.7; **HRMS** ($ESI^+$) m/z calculated for $C_{10}H_{11}O_2$ $(M+H)^+$ 163.0754, found 163.0389; **IR** (liquid film) ν = 3400, 2975, 2930, 1482, 1447, 1427, 1369, 1324, 1259, 1163, 1118, 1031, 892, 868, 854, 831, 799, 742 $cm^{-1}$.

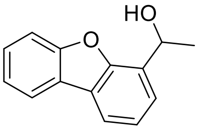
*rac*-**6ac**

**1-(dibenzo[*b*,*d*]furan-4-yl)ethan-1-ol (6ac)**

Compound *rac*-**6ac** was prepared according to General Procedure 2 from dibenzofuran-4-carbaldehyde and MeMgBr, following purification (FCC eluent: 100% DCM) the product was isolated as a colourless oil in 95 % yield.

**$^{1}$H NMR** (500 MHz, $CDCl_3$) δ = 7.96 (dt, *J* = 7.6, 0.9 Hz, 1H), 7.87 (dd, *J* = 7.6, 1.3 Hz, 1H), 7.60 (d, *J* = 8.2 Hz, 1H), 7.52 (dt, *J* = 7.5, 0.9 Hz, 1H), 7.47 (ddd, *J* = 8.4, 7.3, 1.3 Hz, 1H), 7.39 – 7.32 (m, 2H), 5.49 (qd, *J* = 6.5, 4.6 Hz, 1H), 1.71 (d, *J* = 6.5 Hz, 3H); **$^{13}$C NMR (**126 MHz, $CDCl_3$) δ = 156.2, 153.1, 129.9, 127.3, 124.5, 124.3, 123.7, 123.1, 123.0, 120.8, 119.8, 111.9, 66.5, 23.9; **HRMS** ($ESI^+$) m/z calculated for

$C_{14}H_{13}O_2$ (M+H)$^+$ 213.0910, found 213.0553; **IR** (liquid film) ν = 3393, 3066, 3048, 2956, 1722, 1588, 1495, 1475, 1452, 1422, 1267, 1188, 1125, 1058, 1010, 916, 867, 755, 643, 626 cm$^{-1}$.

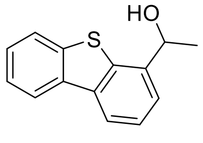
*rac*-**6ad**

**1-(dibenzo[*b,d*]thiophen-4-yl)ethan-1-ol (2ad)**

Compound *rac*-**2ad** was prepared according to General Procedure 2 from dibenzothiophene-4-carbaldehyde and MeMgBr, following purification (FCC eluent: 100% DCM) the product was isolated as a colourless oil in 94% yield.

**$^1$H NMR** (400 MHz, $CDCl_3$) δ = 8.20 – 8.11 (m, 1H), 8.07 (dd, *J* = 7.7, 1.4 Hz, 1H), 7.91 – 7.82 (m, 1H), 7.56 – 7.41 (m, 4H), 5.25 (q, *J* = 6.5 Hz, 1H), 1.66 (d, *J* = 6.5 Hz, 3H); **$^{13}$C NMR** (126 MHz, $CDCl_3$) δ = 140.2, 139.6, 136.5, 136.4, 135.6, 127.0, 125.0, 124.5, 123.0, 122.8, 121.7, 120.8, 70.1, 23.4; **HRMS** (ESI$^+$) m/z calculated for $C_{14}H_{13}SO$ (M+H)$^+$ 229.0682, found 229.0495; **IR** (neat) ν = 3381, 3063, 2975, 2927, 2871, 1584, 1561, 1444, 1404, 1368, 1304, 1252, 1171, 1121, 1069, 911, 752 cm$^{-1}$.

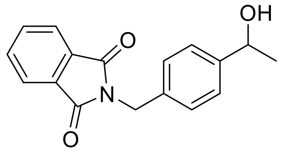
*rac*-**6ae**

**2-(4-(1-hydroxyethyl)benzyl)isoindoline-1,3-dione (6ae)**

Compound *rac*-**6ae** was prepared according to General Procedure 2, from 4-((1,3-dioxoisoindolin-2-yl)methyl)benzaldehyde and MeMgBr, following purification (FCC eluent: 100:0 - 90:10 DCM:EtOAc) the product was isolated as a white solid in 90 % yield.

**$^1$H NMR** (400 MHz, $CDCl_3$) δ = 7.84 (dd, *J* = 5. 4, 3.1 Hz, 2H), 7.70 (dd, *J* = 5.5, 3.0 Hz, 2H), 7.46 – 7.38 (m, 2H), 7.36 – 7.28 (m, 2H), 4.91 – 4.84 (m, 1H), 4.83 (s, 2H), 1.45 (d, *J* = 6.4 Hz, 3H); **$^{13}$C NMR** (101 MHz, $CDCl_3$) δ = 168.2, 145.6, 135.7, 134.1, 132.3, 129.0, 125.9, 123.5, 70.2, 41.5, 25.3; **HRMS** (ESI$^+$) m/z calculated for $C_{17}H_{16}NO_3$ (M+H)$^+$ 282.1125, found 282.1121; **IR** (neat) ν = 3496, 2925, 2856, 2361, 1768, 1708, 1612, 1499, 1468, 1428, 1394, 1364, 1298, 1261, 1202, 1170, 1111, 1089, 1017, 959, 938, 904, 854, 813, 739, 616 cm$^{-1}$; **mp** 102-103 ˚C.

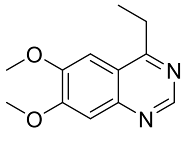
**6af**

**4-ethyl-6,7-dimethoxyquinazoline (6af)**

Compound **6af** was prepared following a modified literature procedure[13], 6,7-dimethoxy-4-chloroquinazoline (3.0 mmol) and $Fe(acac)_2$ (5 mol%) were added into a flamed-dried Schlenk flask, THF (20 mL) and NMP (2 mL) were added followed by dropwise addition of EtMgBr (3.0 M solution in $Et_2O$) at 0 °C. The reaction was left to stir at room temperature for 16 h after which it was quenched with $H_2O$ and extracted with EtOAc. Organic extracts were dried with $MgSO_4$, and solvent removed under reduced pressure. The crude mixture was purified (FCC eluent 80:20 – 60:40 DCM:EtOAc) to afford product as a white solid in 66 % yield.

**$^1$H NMR** (400 MHz, $CDCl_3$) δ = 9.01 (s, 1H), 7.27 (s, 1H), 7.20 (s, 1H), 4.01 (s, 3H), 4.01 (s, 3H), 3.18 (q, *J* = 7.5 Hz, 2H), 2.24 (t, *J* = 7.6 Hz, 3H); **$^{13}$C NMR** (101 MHz, $CDCl_3$) δ = 168.9, 155.6, 153.6, 150.2, 147.9, 119.3, 107.3, 101.8, 56.4, 56.2, 27.9, 12.6; **HRMS** (ESI$^+$) m/z calculated for $C_{12}H_{14}N_2O_2$ (M+H)$^+$ 219.1128, found 219.1127; **IR** (neat) ν = 3009, 2974, 2935, 1668, 1619, 1581, 1556, 1507, 1482, 1464, 1429, 1414,

1373, 1341, 1305, 1282, 1234, 1211, 1176, 1131, 1079, 1027, 1000, 968, 913, 887, 859, 847, 744 $cm^{-1}$; **mp** 152-153 ˚C.

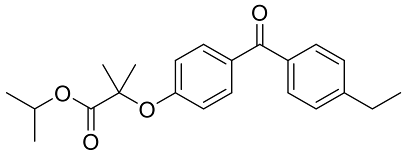

**6ag**

**Isopropyl-2-(4-(4-ethylbenzoyl)phenoxy-3-methylpropanoate (6ag)**

Compound **6ag** was prepared following a modified literature procedure[14], fenofibrate (2.0 mmol), $Cs_2CO_3$ (6.0 mmol) and $Pd(dppf)Cl_2$ (2 mol%) were added to a flamed-dried Schlenk tube under $N_2$ atmosphere and dry THF was added (4 mL). To the stirred suspension, triethylborane (3 mmol, 1.0 M solution in hexanes) was added dropwise at 0 °C and mixture was refluxed for 16 h. The reaction was cooled to 0 °C and quenched with $H_2O$ and extracted with EtOAc, organic extracts were combined, washed with brine and dried with $MgSO_4$. Solvent was removed under reduced pressure and the crude reaction mixture was purified (FCC eluent 90:10 – 80:20 pentane: $Et_2O$) to afford product as white solid in 40 % yield.

**$^1$H NMR** (400 MHz, $CDCl_3$) δ = 7.80 – 7.72 (m, 2H), 7.72 – 7.64 (m, 2H), 7.35 – 7.27 (m, 2H), 6.90 – 6.80 (m, 2H), 5.09 (hept, *J* = 6.3 Hz, 1H), 2.73 (q, *J* = 7.6 Hz, 2H), 1.66 (d, *J* = 1.3 Hz, 6H), 1.28 (t, *J* = 7.6 Hz, 3H), 1.20 (d, *J* = 6.3 Hz, 6H); **$^{13}$C NMR** (101 MHz, $CDCl_3$) δ = 195.5, 173.4, 159.5, 149.0, 135.7, 132.1, 131.1, 130.2, 127.8, 117.3, 79.5, 69.4, 29.1, 25.5, 21.7, 15.4; **HRMS** ($ESI^+$) m/z calculated for $C_{22}H_{17}O_4$ $(M+H)^+$ 355.1904, found 355.1898; **IR** (liquid film) ν = 2984, 2938, 2875, 1732, 1655, 1601, 1574, 1530, 1502, 1467, 1416, 1385, 1252, 1178, 1149, 1104, 1062, 1013, 974, 930, 899, 855, 765 $cm^{-1}$; **mp** 82-83 ˚C.

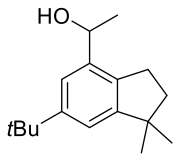

*rac*-**6ah**

**1-(6-(*tert*-butyl)-1,1-dimethyl-2,3-dihydro-1*H*-inden-4-yl)ethan-1-ol (6ah)**

Compound *rac*-**6ah** was prepared according to General Procedure 1, product isolated as a white solid in 95 % yield. Spectroscopic data are in accordance with those in literature.[15]

**$^1$H NMR** (600 MHz, $CDCl_3$) δ = 7.35 – 7.32 (m, 1H), 7.10 (d, *J* = 1.8 Hz, 1H), 5.00 (q, *J* = 6.5 Hz, 1H), 2.86 (qt, *J* = 15.7, 7.2 Hz, 2H), 1.94 (t, *J* = 7.2 Hz, 2H), 1.73 (s, 1H), 1.49 (d, *J* = 6.5 Hz, 3H), 1.34 (s, 9H), 1.26 (d, *J* = 1.6 Hz, 6H); **$^{13}$C NMR** (151 MHz, $CDCl_3$) δ = 152.9, 150.5, 140.8, 136.7, 119.5, 118.1, 68.8, 44.0, 41.6, 35.0, 31.8, 28.9, 28.9, 27.9, 23.9.

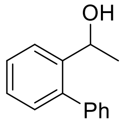

*rac*-**6ai**

**1-([1,1'-biphenyl]-2-yl)ethan-1-ol (6ai)**

Compound *rac*-**6ai** was prepared according to General Procedure 1, affording product as an off-white solid in 89 % yield. Spectroscopic data are in accordance with those in literature.[16]

**$^1$H NMR** (400 MHz, $CDCl_3$) δ = 7.68 (dd, *J* = 7.8, 1.4 Hz, 1H), 7.47 – 7.27 (m, 7H), 7.21 (dd, *J* = 7.7, 1.5 Hz, 1H), 4.99 (qd, *J* = 6.4, 3.2 Hz, 1H), 1.42 (d, *J* = 6.4 Hz, 3H); **$^{13}$C NMR** (101 MHz, $CDCl_3$) δ = 143.2, 141.0, 140.5, 130.1, 128.3, 128.1, 127.2, 125.5, 66.6, 25.0.

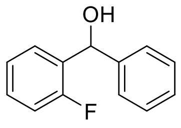

*rac*-**6aj**

**(2-fluorophenyl)(phenyl)methanol (6aj)**

Compound *rac*-**6aj** was prepared according to General Procedure 1, following purification (FCC eluent: 100% DCM), the product was isolated as a colourless oil in 55 % yield. Spectroscopic data are in accordance with those in literature.[17]

**$^1$H NMR** (500 MHz, $CDCl_3$) δ = 7.53 (td, *J* = 7.6, 1.8 Hz, 1H), 7.43 (d, *J* = 7.2 Hz, 2H), 7.36 (dd, *J* = 8.5, 6.8 Hz, 2H), 7.32 – 7.23 (m, 2H), 7.17 (td, *J* = 7.6, 1.2 Hz, 1H), 7.04 (ddd, *J* = 10.5, 8.2, 1.2 Hz, 1H), 6.16 (s, 1H); **$^{13}$C NMR** (126 MHz, $CDCl_3$) δ = 160.0 (d, *J* = 246.2 Hz), 142.9, 131.1 (d, *J* = 13.1 Hz), 129.2 (d, *J* = 8.3 Hz), 128.6, 127.8, 127.8, 126.5, 124.4 (d, *J* = 3.4 Hz), 115.5 (d, *J* = 21.6 Hz), 70.1 (d, *J* = 3.3 Hz).

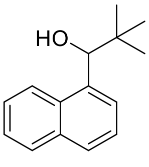

*rac*-**6ak**

**2,2-dimethyl-1-(naphthalen-1-yl)propan-1-ol (6ak)**

Compound *rac*-**6ak** was prepared according to General Procedure 2 from 1-naphthaldehyde and *t*BuMgCl, following purification (FCC eluent: 100% DCM) the product was isolated as a white solid in 51 % yield.

**$^1$H NMR** (400 MHz, $CDCl_3$) δ = 8.16 (d, *J* = 8.2 Hz, 1H), 7.94 – 7.83 (m, 1H), 7.80 – 7.76 (m, 1H), 7.70 – 7.68 (m, 1H), 7.54 – 7.40 (m, 3H), 5.43 (s, 1H), 1.00 (s, 9H); **$^{13}$C NMR** (151 MHz, $CDCl_3$) δ = 138.9, 133.6, 132.0, 129.0, 128.0, 125.7, 125.6 (br), 125.3, 125.1, 124.1 (br), 76.3 (br), 37.1, 26.6; **HRMS** (EI) m/z calculated for $C_{15}H_{18}O$ $(M)^{+\cdot}$ 214.13522, found 214.13577; **IR** (liquid film) ν = 3368, 3349, 3320, 1835, 1780, 1631, 1597, 1550, 1513, 1481, 1393, 1375, 1300, 1281, 1258, 1165, 1102, 1063, 998, 898, 843, 752, 627 $cm^{-1}$; **mp** 70-73 ˚C.

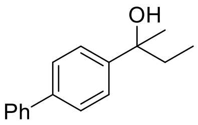

*rac*-**6al**

**2-([1,1'-biphenyl]-4-yl)butan-2-ol (6al)**

Compound *rac*-**6al** was prepared according to General Procedure 2 from 4-acetylbiphenyl and EtMgBr, following purification (FCC eluent: 90:10 – 100:0 DCM:Pentane), affording product as white solid, 34 % yield.

**$^1$H NMR** (500 MHz, $CDCl_3$) δ = 7.66 – 7.54 (m, 4H), 7.54 – 7.51 (m, 2H), 7.50 – 7.40 (m, 2H), 7.37 – 7.30 (m, 1H), 1.96 – 1.81 (m, 2H), 1.59 (s, 3H), 0.85 (t, *J* = 7.4 Hz, 3H); **$^{13}$C NMR** (126 MHz, $CDCl_3$) δ = 147.0, 141.0, 139.5, 128.9, 127.3, 127.2, 127.0, 125.5, 75.0, 36.8, 29.8, 8.5; **HRMS** ($ESI^+$) m/z calculated for $C_{16}H_{18}O$ $(M+H)^+$ 227.1641, found 227.1642; **IR** (neat) ν = 3568, 3030, 2247, 1670, 1602, 1488, 1401, 1353, 1267, 1039, 1007, 909, 840, 766, 732, 678 $cm^{-1}$; **mp** 33-34 ˚C.

**Synthesis of Bromide Substrates**

General Procedure 3

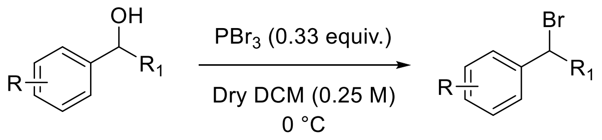


Bromide substrates were prepared from corresponding alcohols via bromination with $PBr_3$ (0.33 equiv.) in dry DCM (0.5 M) at 0 °C following a literature procedure.[18] After completion the reaction was quenched with ice-cold $H_2O$. The aqueous layer was extracted with $Et_2O$ (3 x 10mL) and combined organic extracts were washed with brine, dried with $MgSO_4$ and concentrated in vacuo to give pure products or subsequently purified by FCC.

General Procedure 4

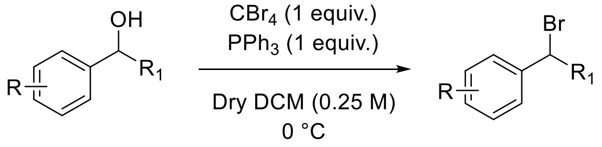


Bromide substrates were prepared from corresponding alcohols via bromination with $CBr_4$ (1 equiv.) and $PPh_3$ (1 equiv.) in dry DCM (0.25 M). After completion, monitored by TLC, the reactions were filtered over celite and washed with $Et_2O$ (3 x 10mL). The combined organic extracts were washed with brine, dried with $MgSO_4$ and concentrated in vacuo. Triphenylphosphine oxide ($PPh_3O$) was removed using $CaBr_2$ (3 equiv.) in $Et_2O$ following literature procedure[19] and following filtration and concentration under reduced pressure, pure products were obtained or subsequently purified by FCC.

General Procedure 5

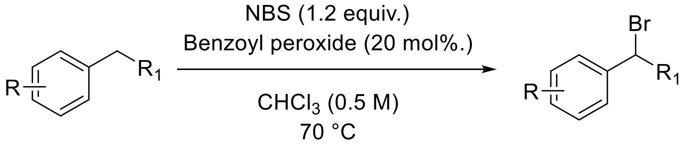


Bromide substrates were prepared from corresponding alkyl precursors through NBS bromination. Alkyl substrates (1 equiv.) were dissolved in $CHCl_3$ (0.5 M), NBS (1.2 equiv.) and benzoyl peroxide (20 mol%) were added, and the reaction mixture was brought to reflux for 16 h. Following which the reaction was partitioned between $H_2O$ and $Et_2O$ and combined organic extracts were washed with brine and dried with $MgSO_4$ and concentrated under reduced pressure. Reaction mixtures were purified by FCC to give product.

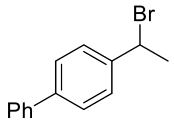

*rac*-**1a**

**4-(1-bromoethyl)-1,1'-biphenyl (1a)**

Compound *rac*-**1a** was prepared according to General Procedure 3 from *rac*-**6a** (CAS: 3562-73-0) in 98 % yield. Spectroscopic data are in accordance with those in literature.[18]

**$^1$H NMR** (500 MHz, $CDCl_3$) δ = 7.62 – 7.55 (m, 4H), 7.55 – 7.49 (m, 2H), 7.45 (dd, *J* = 8.3, 6.9 Hz, 2H), 7.40 – 7.33 (m, 1H), 5.28 (q, *J* = 6.9 Hz, 1H), 2.10 (d, *J* = 6.9 Hz, 3H); **$^{13}$C NMR** (126 MHz, $CDCl_3$) δ = 142.3, 141.5, 140.6, 129.0, 127.7, 127.6, 127.4, 127.3, 49.5, 26.9.

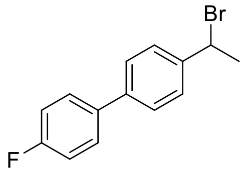

*rac*-**1b**

**4-(1-bromoethyl)-4'-fluoro-1,1'biphenyl (1b)**

Compound *rac*-**1b** was prepared according to General Procedure 3 from *rac*-**6b.** The product was isolated as a white solid in 84 % yield.

**$^1$H NMR** (500 MHz, $CDCl_3$) δ = 7.56 – 7.51 (m, 6H), 7.18 – 7.10 (m, 2H), 5.27 (q, *J* = 6.9 Hz, 1H), 2.09 (d, *J* = 6.9 Hz, 3H); **$^{19}$F NMR** (471 MHz, $CDCl_3$) δ = -115.3; **$^{13}$C NMR** (126 MHz, $CDCl_3$) δ = 162.7 (d, *J* = 246.8 Hz), 142.4, 140.4, 136.7, 136.7, 128.8 (d, *J* = 8.1 Hz), 127.4 (d, *J* = 7.2 Hz), 115.8 (d, *J* = 21.5 Hz), 49.4, 26.8; **IR** (neat) ν = 2990, 2979, 1914, 1834, 1601, 1528, 1496, 1443, 1398, 1251, 1237, 1199, 1067, 1046, 1006, 963, 910, 821, 738, 663 $cm^{-1}$; **mp** 61-62 ˚C; No HRMS obtained.

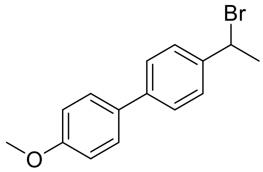

*rac*-**1c**

**4-(1-bromoethyl)-4'-methoxy-1,1'-biphenyl (1c)**

Compound *rac*-**1c** was prepared according to General Procedure 3 from *rac*-**6c**. The product was isolated as a white solid in 97 % yield.

**$^1$H NMR** (500 MHz, $CDCl_3$) δ = 7.57 – 7.46 (m, 6H), 7.01 – 6.95 (m, 2H), 5.28 (q, *J* = 7.0 Hz, 1H), 3.85 (s, 3H), 2.09 (dd, *J* = 6.9, 1.1 Hz, 3H); **$^{13}$C NMR** (126 MHz, $CDCl_3$) δ = 159.5, 141.7, 141.1, 133.2, 128.3, 127.4, 127.1, 114.4, 55.5, 49.7, 26.9; **IR** (neat) ν = 2955, 2925, 2853, 1713, 1607, 1496, 1465, 1433, 1400, 1367, 1289, 1276, 1221, 1178, 1110, 1050, 1021, 1005, 971, 958, 833, 794, 773, 733, 700, 647 $cm^{-1}$; **mp** 97-99 ˚C; No HRMS obtained.

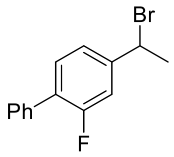

*rac*-**1d**

**4-(1-bromoethyl)-2-fluoro-1,1'-biphenyl (1d)**

Compound *rac*-**1d** was prepared according to General Procedure 3 from *rac*-**5d**. Following purification (FCC eluent 80:20 pentane: $Et_2O$) the product was isolated as a white solid in 61 % yield.

**$^1$H NMR** (600 MHz, $CDCl_3$) δ = 7.58 (dt, *J* = 8.1, 1.5 Hz, 2H), 7.52 – 7.46 (m, 2H), 7.44 – 7.42 (m, 1H), 7.42 – 7.39 (m, 1H) 7.34 – 7.26 (m, 2H), 5.25 (q, *J* = 6.9 Hz, 1H), 2.11 (d, *J* = 6.9 Hz, 3H); **$^{19}$F NMR** (565 MHz, $CDCl_3$) δ = -117.1; **$^{13}$C NMR** (151 MHz, $CDCl_3$) δ = 159.7 (d, *J* = 249.0 Hz), 144.6 (d, *J* = 7.6 Hz), 135.4, 131.1 (d, *J* = 3.9 Hz), 129.2 (d, *J* = 13.7 Hz), 129.1 (d, *J* = 3.0 Hz), 128.6, 128.02, 122.9 (d, *J* = 3.3 Hz), 114.8 (d, *J* = 24.1 Hz), 48.0 (d, *J* = 1.7 Hz), 26.7; **HRMS** (EI) m/z calculated for $C_{14}H_{12}BrF$ $(M)^{+\cdot}$

278.01009, found 278.01064; **IR** (neat) ν = 3051, 2962, 2924, 2852, 1969, 1910, 1779, 1664, 1622, 1581, 1562, 1513, 1485, 1453, 1417, 1377, 1330, 1267, 1221, 1184, 1130, 10772, 1044, 1011, 956, 911, 873, 738, 651 cm$^{-1}$; **mp** 56-57 ˚C.

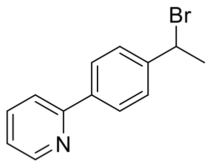


*rac*-**1e**

**2-(4-(1-bromoethyl)phenyl)pyridine (1e)**

Compound *rac*-**1e** was prepared according to General Procedure 3 from *rac*-**5e**. The product was isolated as a viscous colourless oil in 89 % yield.

**$^{1}$H NMR** (400 MHz, $CDCl_3$) δ = 8.71 (ddd, *J* = 5.0, 1.8, 0.9 Hz, 1H), 8.00 – 7.95 (m, 1H), 7.85 – 7.72 (m, 2H), 7.61 – 7.40 (m, 3H), 7.26 (ddd, *J* = 7.4, 5.0, 1.2 Hz, 1H), 5.27 (q, *J* = 6.9, 2.4 Hz, 1H), 2.09 (d, *J* = 7.0, 1.4 Hz, 3H); **$^{13}$C NMR**[a] (101 MHz, $CDCl_3$)[a] δ = 156.8, 149.7, 144.1, 142.6, 140.7, 139.3, 137.1, 127.5 (minor), 127.4 (major), 127.4 (major), 122.5, 120.8, 49.4 (minor), 49.2 (major), 30.5 (minor), 29.9 (major); **HRMS** (ESI$^+$) m/z calculated for $C_{13}H_{12}BrN$ $(M+H)^+$ 262.0226, found 262.0222; **IR** (neat) ν = 2973, 2925, 2855, 1624, 1540, 1467, 1411, 1380, 1341, 1380, 1341, 1305, 1162, 1130, 1109, 1010, 951, 896, 851, 816, 781, 720, 647, 622 cm$^{-1}$. [a]Mixture of rotamers reported.

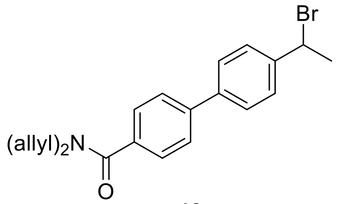


*rac*-**1f**

***N,N*-diallyl-4'-(1-bromoethyl)-[1,1'-biphenyl]-4-carboxamide (1f)**

Compound *rac*-**1f** was prepared according to General Procedure 3 from *rac*-**6f**. The product was isolated as a viscous pale-yellow oil in 63 % yield.

**$^{1}$H NMR** (400 MHz, $CDCl_3$) δ = 7.65 – 7.55 (m, 4H), 7.54 – 7.47 (m, 4H), 5.89 (br s, 1H), 5.78 (br s, 1H), 5.33 – 5.17 (m, 5H), 4.15 (br s, 2H), 3.90 (br s, 2H), 2.08 (d, *J* = 6.9 Hz, 3H); **$^{13}$C NMR** (101 MHz, $CDCl_3$) [overlapping signals] δ = 171.6, 142.8, 141.9, 140.4, 135.4, 133.3, 132.9, 127.5, 127.5, 127.4, 127.1, 117.8, 50.9 (br), 49.2, 47.2 (br), 26.8; **HRMS** (ESI$^+$) m/z calculated for $C_{21}H_{22}BrNO$ $(M+H)^+$ 384.0958, found 384.0950; **IR** (neat) ν = 2979, 2924, 1715, 1647, 1630, 1608, 1527, 1458, 1412, 1340, 1261, 1180, 1114, 1099, 1046, 1005, 986, 926, 862, 828, 772, 738 cm$^{-1}$.

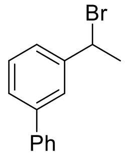


*rac*-**1g**

**3-(1-bromoethyl)-1,1'-biphenyl (1g)**

Compound *rac*-**1g** was prepared according to General Procedure 3 from *rac*-**6g**. The product was isolated as a viscous yellow oil in 88 % yield.

**$^{1}$H NMR** (600 MHz, $CDCl_3$) δ = 7.68 (q, *J* = 1.4 Hz, 1H), 7.65 – 7.58 (m, 2H), 7.58 – 7.49 (m, 1H), 7.51 – 7.43 (m, 4H), 7.43 – 7.35 (m, 1H), 5.28 (q, *J* = 6.9 Hz, 1H), 2.10 (d, *J* = 6.9 Hz, 3H); **$^{13}$C NMR** (151 MHz, $CDCl_3$) [overlapping signal] δ = 143.9, 141.9, 140.9, 129.3, 129.0, 127.7, 127.4, 125.9, 125.8, 49.6, 27.1; **HRMS** (EI) m/z calculated for $C_{14}H_{13}Br$ $(M)^{+\cdot}$ 260.01951, found 260.02006; **IR** (neat) ν = 3060,

3033, 2969, 2921, 2866, 1599, 1480, 1454, 1422, 1376, 1198, 1101, 1075, 1042, 968, 893, 801, 758, 700, 639 $cm^{-1}$.

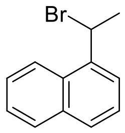

*rac*-**1h**

**1-(1-bromoethyl)naphthalene (1h)**

Compound *rac*-**1h** was prepared according to General Procedure 3 from *rac*-**6h** (CAS: 57605-95-5). The product was isolated as an off-white solid in 90 % yield. Spectroscopic data are in accordance with those in literature.[20]

**$^1$H NMR** (500 MHz, $CDCl_3$) δ = 8.28 (d, *J* = 8.4 Hz, 1H), 7.93 (d, *J* = 8.1 Hz, 1H), 7.87 (d, *J* = 8.2 Hz, 1H), 7.79 (d, *J* = 7.2 Hz, 1H), 7.66 (ddq, *J* = 8.4, 6.8, 1.4 Hz, 1H), 7.60 – 7.48 (m, 2H), 6.05 (q, *J* = 6.8 Hz, 1H), 2.31 (dd, *J* = 6.9, 1.5 Hz, 3H); **$^{13}$C NMR** (126 MHz, $CDCl_3$) δ = 138.1, 134.0, 130.4, 129.4, 129.0, 126.5, 126.0, 125.5, 123.6, 123.3, 45.1, 25.5.

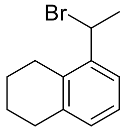

*rac*-**1i**

**5-(1-bromoethyl)-1,2,3,4-tetrahydronaphthalene (1i)**

Compound *rac*-**1i** was prepared according to General Procedure 3 from *rac*-**6i**. The product was isolated as a pale-yellow viscous oil in 64 % yield.

**$^1$H NMR** (400 MHz, $CDCl_3$) δ = 7.41 (dd, *J* = 7.8, 1.3 Hz, 1H), 7.15 (t, *J* = 7.7 Hz, 1H), 7.04 (dd, *J* = 7.6, 1.3 Hz, 1H), 5.45 (q, *J* = 6.9 Hz, 1H), 2.95 (dt, *J* = 16.9, 6.1 Hz, 1H), 2.90 – 2.58 (m, 2H), 2.08 (d, *J* = 6.9 Hz, 3H), 1.96 – 1.67 (m, 4H); **$^{13}$C NMR** (101 MHz, $CDCl_3$) δ = 140.8, 138.0, 134.5, 129.8, 125.9, 123.5, 45.9, 30.3, 25.9, 25.4, 23.3, 22.7; **IR** (neat) ν = 2981, 2931, 2860, 2663, 1459, 1436, 1376, 1243, 1164, 1039, 972, 779, 717 $cm^{-1}$; No HRMS obtained.

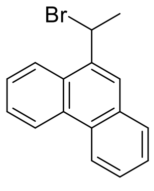

*rac*-**1j**

**9-(1-bromoethyl)phenanthrene (1j)**

Compound *rac*-**1j** was prepared according to General Procedure 3 from *rac*-**6j**. The product was isolated as a pale-yellow viscous oil in 64 % yield.

**$^1$H NMR** (400 MHz, $CDCl_3$) δ = 8.79 – 8.71 (m, 1H), 8.66 (ddt, *J* = 8.8, 1.3, 0.7 Hz, 1H), 8.36 – 8.26 (m, 1H), 8.00 (s, 1H), 7.91 (dd, *J* = 7.8, 1.5 Hz, 1H), 7.77 – 7.57 (m, 4H), 5.99 (q, *J* = 6.9 Hz, 1H), 2.37 (d, *J* = 6.9 Hz, 3H); **$^{13}$C NMR** (101 MHz, $CDCl_3$) δ = 136.3, 131.3, 131.0, 130.7, 129.5, 129.2, 127.5, 127.1, 126.9, 126.8, 124.8, 124.2, 123.4, 122.7, 45.4, 25.3; **IR** (neat) ν = 2981, 2925, 1735, 1448, 1379, 1252, 1157, 1065, 951, 903, 767, 748, 723 $cm^{-1}$; No HRMS obtained.

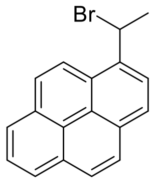

*rac*-**1k**

**1-(1-bromoethyl)pyrene (6k)**

Compound *rac*-**1k** was prepared according to General Procedure 3 from *rac*-**6k**. The product was isolated as a off white solid in 78 % yield.

**$^{1}$H NMR** (400 MHz, $CDCl_3$) δ = 8.45 (d, *J* = 9.3 Hz, 1H), 8.30 – 8.18 (m, 5H), 8.12 – 7.97 (m, 3H), 6.37 (q, *J* = 6.9 Hz, 1H), 2.41 (d, *J* = 6.9 Hz, 3H); **$^{13}$C NMR** (101 MHz, $CDCl_3$) δ = 135.6, 131.6, 131.5, 130.8, 128.2, 128.1, 127.9, 127.5, 126.4, 125.8, 125.6, 125.3, 125.1, 124.9, 123.6, 122.4, 45.8, 26.3; **IR** (neat) ν = 2984, 2882, 2880, 1607, 1517, 1400, 1381, 1189, 1073, 1035, 839, 800, 755, 613 $cm^{-1}$; **mp** 82-85˚C, No HRMS obtained.

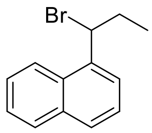

*rac*-**1l**

**1-(1-bromopropyl)naphthalene (1l)**

Compound *rac*-**1l** was prepared according to General Procedure 3 from *rac*-**6l**. The product was isolated as a colourless oil in 90 % yield.

**$^{1}$H NMR** (500 MHz, $CDCl_3$) δ = 8.24 (d, *J* = 7.7 Hz, 1H), 7.95 – 7.88 (m, 1H), 7.85 (dd, *J* = 8.6, 3.4 Hz, 1H), 7.75 (t, *J* = 5.8 Hz, 1H), 7.62 (ddq, *J* = 8.7, 5.3, 1.7 Hz, 1H), 7.57 – 7.46 (m, 2H), 5.75 – 5.72 (m, 1H), 2.66 – 2.52 (m, 1H), 2.51 – 2.38 (m, 1H), 1.21 – 1.14 (m, 3H); **$^{13}$C NMR** (126 MHz, $CDCl_3$) δ = 137.3, 134.1, 130.7, 129.2, 129.1, 126.5, 126.0, 125.6, 124.6, 123.1, 53.2, 32.1, 13.4; **IR** (neat) ν = 3052, 2958, 2872, 1699, 1598, 1511, 1464, 1208, 1180, 1105, 1020, 798, 775, 750, 733, 656, 630, 616 $cm^{-1}$; No HRMS obtained.

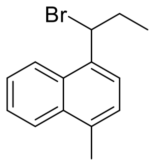

*rac*-**1m**

**1-(1-bromopropyl)-4-methylnaphthalene (1m)**

Compound *rac*-**1l** was prepared according to General Procedure 3 from *rac*-**6l**. The product was isolated as a colourless oil in 90 % yield

**$^{1}$H NMR** (400 MHz, $CDCl_3$) δ = 8.22 (d, *J* = 8.4 Hz, 1H), 8.09 – 8.02 (m, 1H), 7.65 – 7.48 (m, 3H), 7.32 (d, *J* = 7.4 Hz, 1H), 5.71 (t, *J* = 7.3 Hz, 1H), 2.69 (s, 3H), 2.64 – 2.34 (m, 2H), 1.14 (t, *J* = 7.2 Hz, 3H); **$^{13}$C NMR** (101 MHz, $CDCl_3$) δ = 135.6, 135.5, 133.2, 130.7, 126.5, 126.2, 125.9, 125.1, 124.3, 123.7, 53.6, 32.0, 19.9, 13.5; **IR** (neat) ν = 2980, 2884, 1597, 1517, 1457, 1381, 1179, 1073, 1035, 962, 833, 800, 755 $cm^{-1}$; No HRMS obtained.

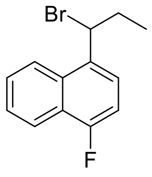

*rac*-**1n**

**1-(1-bromopropyl)-4-fluoronaphthalene (1n)**

Compound *rac*-**1m** was prepared according to General Procedure 3 from *rac*-**6m**. The product was isolated as a white solid in 85 % yield

**$^{1}$H NMR** (400 MHz, $CDCl_3$) δ = 8.23 – 8.14 (m, 2H), 7.70 – 7.53 (m, 3H), 7.13 (dd, *J* = 10.0, 8.1 Hz, 1H), 5.64 (dd, *J* = 8.4, 6.2 Hz, 1H), 2.67 – 2.26 (m, 2H), 1.14 (t, *J* = 7.2 Hz, 3H); **$^{19}$F NMR** (376 MHz, $CDCl_3$) δ = -121.4; **$^{13}$C NMR** (101 MHz, $CDCl_3$) δ = 158.9 (d, *J* = 254.2 Hz), 133.4 (d, *J* = 4.8 Hz), 132.1 (d, *J* = 4.7 Hz), 127.5, 126.4 (d, *J* = 2.1 Hz), 124.8 (d, *J* = 8.7 Hz), 124.2 (d, *J* = 16.0 Hz), 123.3, 121.5 (d, *J* = 6.1 Hz), 109.2 (d, *J* = 20.5 Hz), 52.7, 32.1, 13.4; **IR** (neat) ν = 2981, 2888, 1607, 1512, 1464, 1393, 1256, 1158, 961, 807, 770 $cm^{-1}$; **mp** 43-44 ˚C. No HRMS obtained.

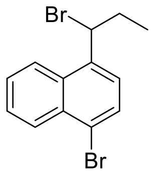

*rac*-**1o**

**1-bromo-4-(1-bromopropyl)naphthalene (1o)**

Compound *rac*-**1n** was prepared according to General Procedure 3 from *rac*-**6n**. The product was isolated as a white solid in 89 % yield.

**$^1$H NMR** (500 MHz, $CDCl_3$) δ = 8.37 – 8.30 (m, 1H), 8.20 (d, *J* = 8.0 Hz, 1H), 7.79 (d, *J* = 7.8 Hz, 1H), 7.69 – 7.60 (m, 2H), 7.56 (d, *J* = 7.9 Hz, 1H), 5.65 (t, *J* = 7.3 Hz, 1H), 2.53 (ddt, *J* = 14.5, 8.5, 7.1 Hz, 1H), 2.39 (dqd, *J* = 14.4, 7.2, 5.9 Hz, 1H), 1.14 (t, *J* = 7.2 Hz, 3H); **$^{13}$C NMR** (126 MHz, $CDCl_3$) δ = 137.5, 132.4, 131.9, 129.8, 128.3, 127.5, 127.3, 125.0, 124.0, 123.6, 52.2 (br), 32.0, 13.4; **IR** (neat) ν = 3081, 2909, 2841, 1806, 1565, 1508, 1450, 1385, 1353, 1326, 1203, 1176, 1153, 1120, 1034, 934, 884, 834, 799, 777, 756, 744, 638 cm$^{-1}$; **mp** 81-83 ˚C; No HRMS obtained.

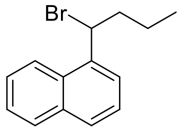

*rac*-**1p**

**1-(1-bromobutyl)naphthalene (1p)**

Compound *rac*-**1** was prepared according to General Procedure 3 from *rac*-**6p**. The product was isolated as a colourless oil in 95 % yield.

**$^1$H NMR** (500 MHz, $CDCl_3$) δ = 8.20 (d, *J* = 8.5 Hz, 1H), 7.92 – 7.86 (m, 1H), 7.86 – 7.78 (m, 1H), 7.73 (d, *J* = 7.1 Hz, 1H), 7.60 (ddd, *J* = 8.5, 6.8, 1.4 Hz, 1H), 7.57 – 7.40 (m, 2H), 5.88 – 5.68 (m, 1H), 2.59 – 2.48 (m, 1H), 2.40 – 2.31 (m, 1H), 1.74 – 1.60 (m, 1H), 1.54 – 1.45 (m, 1H), 1.01 (t, *J* = 7.4 Hz, 3H); **$^{13}$C NMR** (126 MHz, $CDCl_3$) δ = 137.5, 134.1, 130.6, 129.2, 129.1, 126.5, 126.0, 125.6, 124.7, 123.1, 50.9, 40.9, 21.8, 13.6; **IR** (neat) ν = 3050, 2961, 2872, 1701, 1597, 1511, 1463, 1180, 1151, 1110, 1034, 936, 863, 799, 776, 733 cm$^{-1}$; No HRMS obtained.

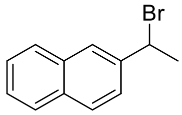

*rac*-**1q**

**2-(1-bromoethyl)naphthalene (1q)**

Compound *rac*-**1q** was prepared according to General Procedure 3 from *rac*-**6q** (CAS: 7228-47-9). The product was isolated as an off-white solid in 95% yield. Spectroscopic data are in accordance with those in literature.[21]

**$^1$H NMR** (500 MHz, $CDCl_3$) δ = 7.88 – 7.80 (m, 4H), 7.61 (dd, *J* = 8.4, 1.9 Hz, 1H), 7.55 – 7.45 (m, 2H), 5.41 (q, *J* = 6.9 Hz, 1H), 2.16 (d, *J* = 6.9 Hz, 3H); **$^{13}$C NMR** (126 MHz, $CDCl_3$) δ = 140.6, 133.3, 133.2, 128.8, 128.2, 127.8, 126.6, 126.6, 125.3, 125.2, 50.1, 26.9.

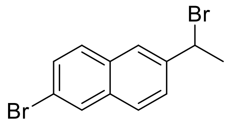

*rac*-**1r**

**2-bromo-6-(1-bromoethyl)naphthalene (1r)**

Compound *rac*-**1r** was prepared according to General Procedure 3 from *rac*-**6r**. The product was isolated as an off-white solid in 97 % yield.

**$^1$H NMR** (400 MHz, $CDCl_3$) δ = 7.99 (d, *J* = 1.9 Hz, 1H), 7.79 (d, *J* = 1.8 Hz, 1H), 7.75 (d, *J* = 8.6 Hz, 1H), 7.73 – 7.65 (m, 1H), 7.61 (dd, *J* = 8.6, 1.9 Hz, 1H), 7.56 (dd, *J* = 8.7, 2.0 Hz, 1H), 5.36 (q, *J* = 6.9 Hz, 1H), 2.13 (d, *J* = 6.9 Hz, 3H); **$^{13}$C NMR** (126 MHz, $CDCl_3$) δ = 141.1, 134.3, 131.6, 130.0, 129.9, 129.8, 127.9, 126.4, 125.2, 120.7, 49.6, 26.7; **IR** (neat) ν = 2980, 2918, 2850, 128, 1588, 1497, 1462, 1438, 1375,

1338, 1302, 1253, 1195, 1162, 1132, 1061, 1038, 968, 948, 896, 884, 821, 809, 791, 763, 725, 665, 642 $cm^{-1}$; **mp** 85-86 ˚C; No HRMS obtained.

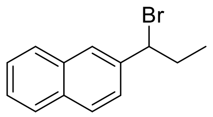

*rac*-**1s**

**2-(1-bromopropyl)naphthalene (1s)**

Compound *rac*-**1s** was prepared according to General Procedure 3 from *rac*-**6s**. The product was isolated as an off-white solid in 89 % yield.

**$^{1}$H NMR** (400 MHz, $CDCl_3$) δ = 7.89 – 7.77 (m, 4H), 7.57 (dt, *J* = 8.6, 1.9 Hz, 1H), 7.54 – 7.45 (m, 2H), 5.08 (t, *J* = 7.5 Hz, 1H), 2.50 – 2.21 (m, 2H), 1.04 (t, *J* = 7.2 Hz, 3H); **$^{13}$C NMR** (101 MHz, $CDCl_3$) [overlapping signal] δ = 139.5, 133.3, 133.2, 128.8, 128.2, 127.8, 126.6, 126.2, 125.3, 58.1, 33.3, 13.2; **HRMS** (EI) m/z calculated for $C_{13}H_{13}Br$ $(M)^{+\cdot}$ 248.01951, found 248.02006; **IR** (neat) ν = 3057, 2969, 2933, 2874, 1739, 1722, 1599, 1509, 1453, 1381, 1367, 1278, 1231, 1216, 1187, 1126, 1096, 1067, 1018, 904, 861, 825, 797, 750, 658 $cm^{-1}$; **mp** 52-53 ˚C.

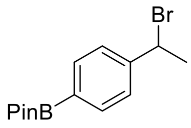

*rac*-**1t**

**2-(4-(1-bromoethyl)phenyl)-4,4,5,5-tetramethyl-1,3,2-dioxaborolane (1t)**

Compound *rac*-**1t** was prepared according to General Procedure 3 from *rac*-**6t**. The product was isolated as a colourless oil in 80 % yield.

**$^{1}$H NMR** (500 MHz, $CDCl_3$) δ = 7.82 – 7.76 (m, 2H), 7.46 – 7.41 (m, 2H), 5.20 (q, *J* = 6.9 Hz, 1H), 2.04 (d, *J* = 6.9 Hz, 3H), 1.34 (s, 12H); **$^{13}$C NMR** (126 MHz, $CDCl_3$) δ = 146.2, 135.3, 126.3, 84.0, 49.4, 26.8, 25.0; **IR** (liquid film) ν = 2981, 2929, 2248, 1612, 1517, 1443, 1400, 1361, 1324, 1297, 1271, 1144, 1092, 1065, 1042, 1020, 963, 910, 859, 837, 768, 735, 658 $cm^{-1}$; No HRMS obtained.
(*ipso*-carbon bound to boron not observed by $^{13}$C NMR).

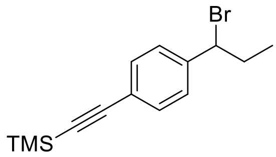

*rac*-**1u**

**((4-(1-bromopropyl)phenyl)ethynyl)trimethylsilane (1u)**

Compound *rac*-**1u** was prepared according to General Procedure 3 from *rac*-**6u**. Following purification (FCC eluent: 100% DCM) the product was isolated as a colourless oil in 64 % yield.

**$^{1}$H NMR** (500 MHz, $CDCl_3$) δ = 7.46 – 7.35 (m, 2H), 7.34 – 7.27 (m, 2H), 4.84 (dd, *J* = 7.9, 6.9 Hz, 1H), 2.33 – 2.21 (m, 1H), 2.14 (dp, *J* = 14.3, 7.2 Hz, 1H), 0.98 (t, *J* = 7.3 Hz, 3H), 0.25 (s, 9H); **$^{13}$C NMR** (126 MHz, $CDCl_3$) δ = 142.5, 132.4, 127.4, 123.2, 104.7, 95.1, 56.8, 33.2, 13.0, 0.1; **HRMS** (EI) m/z calculated for $C_{14}H_{19}BrSi$ $(M)^{+}$ 294.04339, found 294.04394; **IR** (neat) ν = 2966, 2917, 2849, 2159, 1508, 1463, 1409, 1393, 1352, 1250, 1221, 1116, 1088, 1018, 866, 844, 760, 672, 640 $cm^{-1}$.

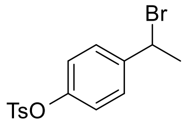

*rac*-**1v**

**4-(1-bromoethyl)phenyl-4-methylbenzenesulfonate (1v)**

Compound *rac*-**1v** was prepared according to General Procedure 3 from *rac*-**6v**. The product was isolated as a colourless oil in 87 % yield.

**$^1$H NMR** (500 MHz, $CDCl_3$) δ = 7.72 (dt, *J* = 8.9, 1.6 Hz, 2H), 7.39 – 7.30 (m, 4H), 6.99 – 6.92 (m, 2H), 5.14 (q, *J* = 6.9 Hz, 1H), 2.46 (s, 3H), 2.00 (dd, *J* = 6.9, 1.1 Hz, 3H); **$^{13}$C NMR** (126 MHz, $CDCl_3$) δ = 149.3, 145.6, 142.3, 132.5, 130.0, 128.6, 128.3, 122.7, 48.1, 26.9, 21.9; **IR** (neat) ν = 2985, 2958, 2859, 1599, 1503, 1443, 1375, 1216, 1199, 1177, 1154, 1119, 1093, 1044, 1018, 956, 943, 867, 846, 813, 785, 755, 707, 682, 659 $cm^{-1}$; No HRMS obtained.

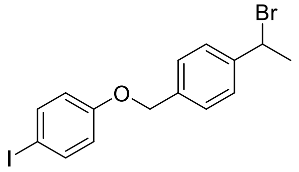

*rac*-**1w**

**1-(1-bromoethyl)-4-((4-iodophenoxy)methyl)benzene (1w)**

Compound *rac*-**1w** was prepared according to General Procedure 3 from *rac*-**6w**. The product was isolated as a white solid in 78 % yield.

**$^1$H NMR** (500 MHz, $CDCl_3$) δ = 7.59 – 7.52 (m, 2H), 7.49 – 7.43 (m, 2H), 7.41 – 7.32 (m, 2H), 6.79 – 6.71 (m, 2H), 5.22 (q, *J* = 6.9 Hz, 1H), 5.02 (s, 2H), 2.05 (d, *J* = 7.0 Hz, 3H); **$^{13}$C NMR** (126 MHz, $CDCl_3$) δ = 158.6, 143.3, 138.4, 136.8, 127.8, 127.3, 117.4, 83.3, 69.7, 49.1, 26.9; **IR** (neat) ν = 2919, 2865, 1585, 1572, 1515, 1486, 1459, 1422, 1381, 1280, 1242, 1173, 115, 1042, 1015, 1000, 967, 874, 823, 751, 719, 634 $cm^{-1}$; **mp** 92-93 ˚C; No HRMS obtained.

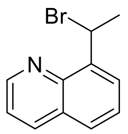

*rac*-**1x**

**8-(1-bromoethyl)quinoline (1x)**

Compound *rac*-**1x** was prepared according to General Procedure 3 from *rac*-**6x**. The product was isolated as a yellow viscous oil in 40 % yield.

**$^1$H NMR** (400 MHz, $CDCl_3$) δ = 8.99 (dd, *J* = 4.2, 1.8 Hz, 1H), 8.17 (dd, *J* = 8.3, 1.8 Hz, 1H), 8.03 (dd, *J* = 7.3, 1.4 Hz, 1H), 7.78 (dd, *J* = 8.2, 1.4 Hz, 1H), 7.58 (dd, *J* = 8.2, 7.3 Hz, 1H), 7.44 (dd, *J* = 8.3, 4.2 Hz, 1H), 6.80 (q, *J* = 7.0 Hz, 1H), 2.20 (d, *J* = 7.0 Hz, 3H); **$^{13}$C NMR** (101 MHz, $CDCl_3$) δ = 149.9, 144.6, 141.3, 136.6, 128.4, 128.3, 127.6, 126.7, 121.5, 43.5, 26.4; **HRMS** ($ESI^+$) m/z calculated for $C_{11}H_{10}BrN$ $(M+H)^+$ calculated 236.0069, found 236.0069; **IR** (neat) ν = 3039, 2921, 2861, 1947, 1820, 1596, 1576, 1497, 1441, 1375, 1322, 1253, 1193, 1165, 1134, 1089, 1041, 1008, 963, 910, 856, 831, 794, 760, 733 $cm^{-1}$.

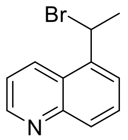

*rac*-**1y**

**5-(1-bromoethyl)quinoline (1y)**

Compound *rac*-**1y** was prepared according to General Procedure 3 from *rac*-**6y**. The product was isolated as a pale-yellow solid in 70 % yield.

**$^1$H NMR** (400 MHz, $CDCl_3$) δ = 9.36 (d, *J* = 8.5 Hz, 1H), 9.18 (m, 1H), 8.94 (dd, *J* = 6.8, 2.8 Hz, 1H), 8.14 – 8.12 (m, 1H), 8.08 – 8.05 (m, 2H), 5.87 (q, *J* = 6.8 Hz, 1H), 2.31 (d, *J* = 6.8 Hz, 3H); **$^{13}$C NMR** (101 MHz, $CDCl_3$) δ = 142.8, 142.5, 140.2, 138.7, 134.9, 127.4, 127.0, 122.3, 121.3, 41.1, 24.8; **HRMS** ($ESI^+$) m/z calculated for $C_{11}H_{10}BrN$ $(M+H)^+$ calculated 236.0069, found 236.0069; **IR** (neat) ν = 2627, 2852, 2620, 1630, 1597, 1554, 1410, 1372, 1293, 1225, 1194, 1027, 1007, 991, 919, 825, 803, 704, 660 $cm^{-1}$; **mp** 183-184 ˚C.

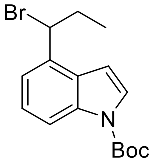
rac-**1z**

**_tert_-butyl-4-(1-bromopropyl)-1_H_-indole-1-carboxylate (1z)**

Compound *rac*-**1z** was prepared according to General Procedure 4 from *rac*-**6z**. The product was isolated as a yellow oil in 45 % yield.

**$^1$H NMR** (500 MHz, $CDCl_3$) δ = 8.12 (d, *J* = 7.3 Hz, 1H), 7.67 (d, *J* = 3.8 Hz, 1H), 7.32 – 7.27 (m, 2H), 6.78 (dd, *J* = 3.8, 0.9 Hz, 1H), 5.26 (dd, *J* = 8.2, 6.8 Hz, 1H), 2.52 – 2.28 (m, 2H), 1.67 (s, 9H), 1.04 (t, *J* = 7.3 Hz, 3H); **$^{13}$C NMR** (126 MHz, $CDCl_3$) δ = 149.8 (br), 134.0, 128.8, 126.1, 125.2, 124.5, 120.9, 115.5, 105.5, 84.1, 55.2, 32.5, 28.3, 13.3; **HRMS** ($ESI^+$) m/z calculated for $C_{16}H_{20}BrNO_2$ $(M+H)^+$ 338.0750, found 338.1078; **IR** (liquid film) ν = 3008, 2926, 2854, 2360, 2340, 1740, 1537, 1426, 1382, 1371, 1349, 1284, 1258, 1158, 1135, 1048, 913, 758, 734, 646 $cm^{-1}$.

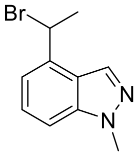
rac-**1aa**

**4-(1-bromoethyl)-1-methyl-1_H_-indazole (1aa)**

Compound *rac*-**1aa** was prepared according to General Procedure 3 from *rac*-**1aa**. The product was isolated as a colourless oil in 88 % yield.

**$^1$H NMR** (400 MHz, $CDCl_3$) δ = 8.22 (d, *J* = 0.9 Hz, 1H), 7.41 – 7.28 (m, 2H), 7.26 – 7.17 (m, 1H), 5.57 (q, *J* = 7.0 Hz, 1H), 4.09 (s, 3H), 2.21 (d, *J* = 7.0 Hz, 3H); **$^{13}$C NMR** (101 MHz, $CDCl_3$) δ = 140.5, 136.3, 131.6, 126.3, 121.9, 117.8, 109.6, 47.0, 35.9, 26.2; **HRMS** ($ESI^+$) m/z calculated for $C_{10}H_{12}BrN_2$ $(M+H)^+$ 239.0178, found 239.0179; **IR** (neat) ν = 2917, 2851, 1611, 1509, 1452, 1409, 1376, 1276, 1182, 1074, 1030, 987, 942, 912, 836, 789, 735, 660, 641, 629 $cm^{-1}$.

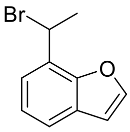
rac-**1ab**

**7-(1-bromoethyl)benzofuran (1ab)**

Compound *rac*-**1ab** was prepared according to General Procedure 3 from *rac*-**1ab**. The product was isolated as a colourless oil in 66 % yield.

**$^1$H NMR** (400 MHz, $CDCl_3$) δ =7.69 (d, *J* = 2.2 Hz, 1H), 7.56 (dd, *J* = 7.8, 1.2 Hz, 1H), 7.41 (dd, *J* = 7.5, 1.2 Hz, 1H), 7.24 (t, *J* = 7.7 Hz, 1H), 6.80 (d, *J* = 2.2 Hz, 1H), 5.73 (q, *J* = 7.0 Hz, 1H), 2.20 (d, *J* = 7.0 Hz, 3H); **$^{13}$C NMR** (101 MHz, $CDCl_3$) δ = 151.8, 145.2, 128.1, 127.2, 123.2, 122.1, 121.6, 106.9, 43.3, 25.7; **IR** (neat) ν =3063, 2818, 1542, 1430, 1377, 1314, 1267, 1198, 1169, 1125, 1091, 1030, 949, 872, 838, 799, 738, 680, 660, 635 $cm^{-1}$; No HRMS obtained.

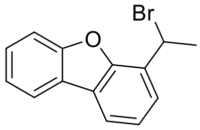
rac-**1ac**

**4-(1-bromoethyl)dibenzo[_b_,_d_]furan (1ac)**

Compound *rac*-**1ac** was prepared according to General Procedure 3 from *rac*-**6ac**. The product was isolated as a colourless oil in 46 % yield.

**$^1$H NMR** (400 MHz, $CDCl_3$) δ = 7.96 (d, *J* = 0.6 Hz, 1H), 7.90 (dd, *J* = 7.6, 1.2 Hz, 1H), 7.65 (dt, *J* = 8.3, 0.9 Hz, 1H), 7.61 (dd, *J* = 7.7, 1.2 Hz, 1H), 7.50 (ddd, *J* = 8.3, 7.2, 1.4 Hz, 1H), 7.42 – 7.31 (m, 2H), 5.87 (q, *J* = 7.0 Hz, 1H), 2.26 (d, *J* = 7.0 Hz, 3H); **$^{13}$C NMR** (101 MHz, $CDCl_3$) δ = 156.2, 152.8, 127.5, 127.4, 124.9,

124.8, 124.1, 123.2, 123.0, 120.8, 120.8, 112.0, 42.8, 25.9; **IR** (neat) ν = 3063, 2818, 1542, 1430, 1377, 1314, 1267, 1125, 1091, 1030, 949, 872, 838, 799, 738, 680, 660, 635 $cm^{-1}$; No HRMS obtained.

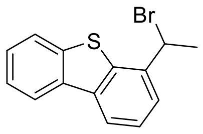

*rac*-**1ad**

**4-(1-bromoethyl)dibenzo[*b*,*d*]thiophene (1ad)**

Compound *rac*-**1ad** was prepared according to General Procedure 3 from *rac*-**6ad**. Following purification (FCC eluent: 50:50 pentane:DCM) the product was isolated as a yellow oil in 44% yield.

$^{1}$**H NMR** (400 MHz, $CDCl_3$) δ = 8.21 – 8.14 (m, 1H), 8.11 (dd, *J* = 7.9, 1.1 Hz, 1H), 7.94 – 7.84 (m, 1H), 7.63 (dd, *J* = 7.7, 1.1 Hz, 1H), 7.54 – 7.45 (m, 3H), 5.53 (q, *J* = 6.9 Hz, 1H), 2.26 (d, *J* = 6.9 Hz, 3H); $^{13}$**C NMR** (101 MHz, $CDCl_3$) δ = 139.2, 138.3, 137.2, 136.6, 135.7, 127.2, 125.2, 124.7, 124.2, 122.9, 121.9, 121.7, 47.4, 25.4; **IR** (liquid film) ν = 2973, 2931, 1657, 1468, 1408, 1380, 1301, 1163, 1130, 1036, 953, 818, 753, 682, 627 $cm^{-1}$; No HRMS obtained.

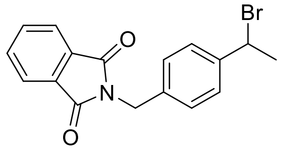

*rac*-**1ae**

**2-(4-(1-bromoethyl)benzyl)isoindoline-1,3-dione (1ae)**

Compound *rac*-**1ae** was prepared according to General Procedure 3 from *rac*-**6ae**. The product was isolated as a white solid in 92 % yield.

$^{1}$**H NMR** (500 MHz, $CDCl_3$) δ = 7.86 (dd, *J* = 5.4, 3.0 Hz, 2H), 7.71 (dd, *J* = 5.5, 3.0 Hz, 2H), 7.44 – 7.35 (m, 4H), 5.17 (q, *J* = 6.9 Hz, 1H), 4.83 (s, 2H), 2.00 (d, *J* = 6.9 Hz, 3H); $^{13}$**C NMR** (126 MHz, $CDCl_3$) δ = 168.1, 143.0, 136.6, 134.2, 132.2, 129.2, 127.3, 123.5, 49.1, 41.3, 26.9; **HRMS** ($ESI^+$) m/z calculated for $C_{17}H_{15}BrNO_2$ $(M+H)^+$ 344.0281, found 344.0276; **IR** (neat) ν = 2923, 2854, 1765, 1718, 1613, 1511, 1466, 1426, 1392, 1342, 1328, 1298, 1260, 1211, 1186, 1096, 1067, 1047, 1018, 976, 940, 752, 727, 812, 765, 715, 692, 627 $cm^{-1}$; **mp** 79-81 ˚C.

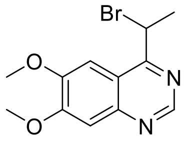

*rac*-**1af**

**4-(1-bromoethyl)-6,7-dimethoxyquinazoline (1af)**

Compound *rac*-**1af** was prepared according to General Procedure 5 from *rac*-**6af**. Following purification (FCC eluent: 90:10 – 85:15 DCM:EtOAc) the product was isolated as an off-white solid in 51 % yield.

$^{1}$**H NMR** (400 MHz, $CDCl_3$) δ = 9.14 (s, 1H), 7.38 (s, 1H), 7.36 (s, 1H), 5.78 (q, *J* = 6.7 Hz, 1H), 4.08 (s, 3H), 4.07 (s, 3H), 2.24 (d, *J* = 6.7 Hz, 3H); $^{13}$**C NMR** (101 MHz, $CDCl_3$) δ = 164.3, 156.3, 153.3, 150.6, 149.0, 118.0, 107.2, 101.4, 56.7, 56.5, 42.8, 22.7; **HRMS** ($ESI^+$) m/z calculated for $C_{12}H_{13}BrN_2O_2$ $(M+H)^+$ 297.0233, found 297.0233; **IR** (neat) ν = 2941, 2834, 2363, 1713, 1620, 1553, 1507, 1476, 1434, 1372, 1372, 1343, 1291, 1238, 1214, 1166, 1133, 1021, 991, 889, 868, 842, 824, 779, 735 $cm^{-1}$; **mp** 149-150 ˚C.

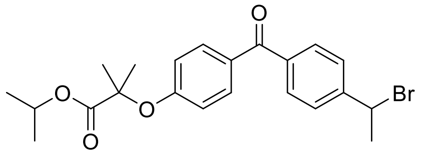


*rac*-**1ag**

**isopropyl-2-(4-(4-(1-bromoethyl)benzoyl)phenoxy)-2-methylpropanoate (1ag)**

Compound *rac*-**1ag** was prepared according to General Procedure 5 from *rac*-**6ag**. Following purification (FCC eluent: 100% DCM) the product was isolated as a viscous colourless oil in 77 % yield.

**$^1$H NMR** (400 MHz, $CDCl_3$) δ = 7.80 – 7.65 (m, 4H), 7.58 – 7.49 (m, 2H), 6.95 – 6.77 (m, 2H), 5.23 (q, *J* = 6.9 Hz, 1H), 5.08 (hept, *J* = 6.3 Hz, 1H), 2.06 (d, *J* = 6.9 Hz, 3H), 1.65 (s, 6H), 1.20 (d, *J* = 6.3 Hz, 6H); **$^{13}$C NMR** (101 MHz, $CDCl_3$) δ = 194.9, 173.2, 159.8, 147.1, 138.1, 132.1, 130.5, 130.3, 126.8, 117.3, 79.5, 69.4, 48.3, 26.7, 25.5, 21.6; **HRMS** ($ESI^+$) m/z calculated for $C_{22}H_{26}BrO_4$ $(M+H)^+$ 433.1009, found 433.1008; **IR** (liquid film) ν = 3042, 2945, 2908, 2361, 1731, 1655, 1600, 1505, 1444, 1287, 1253, 1179, 1102, 1044, 930, 857, 771, 690, 617 $cm^{-1}$.

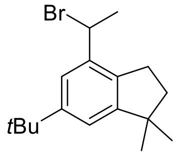


*rac*-**1ah**

**4-(1-bromoethyl)-6-(*tert*-butyl)-1,1-dimethyl-2,3-dihydro-1*H*-indene (1ah)**

Compound *rac*-**1ah** was prepared according to General Procedure 3 from *rac*-**6ah**. The product was isolated as an off-white solid in 82 % yield.

**$^1$H NMR** (400 MHz, $CDCl_3$) δ = 7.35 (d, *J* = 1.8 Hz, 1H), 7.12 (d, *J* = 1.8 Hz, 1H), 5.33 (q, *J* = 6.9 Hz, 1H), 3.06 – 2.80 (m, 2H), 2.09 (d, *J* = 7.0 Hz, 3H), 2.02 – 1.94 (m, 2H), 1.35 (s, 9H), 1.27 (s, 3H), 1.26 (s, 3H); **$^{13}$C NMR** (101 MHz, $CDCl_3$) δ = 153.1, 150.5, 137.9, 137.7, 120.8, 119.3, 48.3, 44.2, 41.4, 35.0, 31.7, 28.9, 28.8, 27.9, 25.9; **IR** (neat) ν = 3000, 2945, 1769, 1609, 1479, 1462, 1394, 1362, 1318, 1254, 1224, 1189, 1144, 1055, 1014, 933, 907, 878, 773, 722, 655, 641, 630 $cm^{-1}$; **mp** 61-63 ˚C; No HRMS obtained.

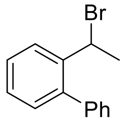


*rac*-**1ai**

**2-(1-bromoethyl)-1,1'-biphenyl (1ai)**

Compound *rac*-**1ai** was prepared according to General Procedure 3 from *rac*-**6ai**. The product was isolated as an off-white solid in 98 % yield.

**$^1$H NMR** (400 MHz, $CDCl_3$) δ = 7.78 (dd, *J* = 7.9, 1.3 Hz, 1H), 7.51 – 7.37 (m, 6H), 7.33 (td, *J* = 7.5, 1.3 Hz, 1H), 7.20 (dd, *J* = 7.6, 1.5 Hz, 1H), 5.28 (q, *J* = 6.9 Hz, 1H), 1.98 (d, *J* = 6.9 Hz, 3H); **$^{13}$C NMR** (101 MHz, $CDCl_3$) δ = 140.9, 140.5, 140.3, 130.2, 129.2, 128.5, 128.3, 128.1, 127.6, 127.5, 47.0, 27.6; **IR** (neat) ν = 3022, 2947, 2924, 2850, 1708, 1595, 1479, 1438, 1338, 1266, 1198, 1172, 1122, 1070, 1037, 1010, 970, 913, 791, 765, 753, 721, 702 $cm^{-1}$; **mp** 35-36 ˚C; No HRMS obtained.

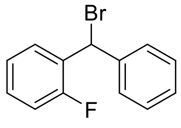


*rac*-**1aj**

**1-(bromo(phenyl)methyl)-2-fluorobenzene (1aj)**

Compound *rac*-**1i** was prepared according to General Procedure **3** from *rac*-**6i**. The product was isolated as a colourless oil in 61 % yield.

$^1$**H NMR** (500 MHz, $CDCl_3$) δ = 7.57 (td, *J* = 7.8, 1.8 Hz, 1H), 7.49 (d, *J* = 7.6 Hz, 2H), 7.41 (t, *J* = 7.5 Hz, 2H), 7.27 (qd, *J* = 7.3, 3.3 Hz, 2H), 7.14 (t, *J* = 7.6 Hz, 1H), 7.03 (dd, *J* = 10.3, 8.2 Hz, 1H), 6.57 (s, 1H); $^{19}$**F NMR** (471 MHz, $CDCl_3$) δ = -118.4; $^{13}$**C NMR** (126 MHz, $CDCl_3$) δ = 159.2 (d, *J* = 248.9 Hz), 140.0, 130.8 (d, *J* = 2.6 Hz), 130.1 (d, *J* = 8.3 Hz), 128.7, 128.7, 128.4, 128.3, 124.5 (d, *J* = 3.7 Hz), 115.7 (d, *J* = 21.7 Hz), 47.1 (d, *J* = 4.4 Hz); **IR** (neat) ν = 3034, 2249, 1615, 1542, 1493, 1456, 1330, 1268, 1224, 1191, 1151, 1078, 1023, 909, 865, 806, 758, 732, 698, 650 $cm^{-1}$; No HRMS obtained.

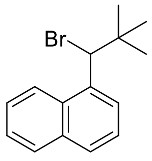


*rac*-**1ak**

**1-(1-bromo-2,2-dimethylpropyl)naphthalene (1ak)**

Compound *rac*-**1p** was prepared according to General Procedure 3 from *rac*-**6p**. The product was isolated as a colourless oil in 42 % yield.

$^1$**H NMR** (500 MHz, $CDCl_3$) δ = 8.10 (d, *J* = 8.6 Hz, 1H), 7.94 (d, *J* = 7.4 Hz, 1H), 7.87 (d, *J* = 8.1 Hz, 1H), 7.79 (d, *J* = 8.1 Hz, 1H), 7.57 – 7.44 (m, 3H), 5.99 (s, 1H), 1.15 (s, 9H); $^{13}$**C NMR** (126 MHz, $CDCl_3$) δ = 136.7, 133.5, 131.1, 129.7, 129.2, 128.5, 126.4, 125.5, 125.1, 123.1, 62.1, 38.4, 28.0; **HRMS** (EI) m/z calculated for $C_{15}H_{17}Br$ $(M)^{+\cdot}$ 276.05081, found 276.05136; **IR** (neat) ν = 3049, 2961, 2874, 1701, 1596, 1511, 1462, 1181, 1152, 1111, 1034, 962, 776, 736 $cm^{-1}$.

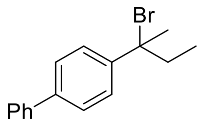


*rac*-**1al**

**4-(2-bromobutan-2-yl)-1,1'-biphenyl (1al)**

Compound *rac*-**1r** was prepared according to General Procedure 4 from *rac*-**6r**. The product was isolated as a colourless oil in 57 % yield.

$^1$**H NMR** (400 MHz, $CDCl_3$) δ = 7.67 – 7.55 (m, 4H), 7.54 – 7.49 (m, 2H), 7.49 – 7.38 (m, 2H), 7.38 – 7.29 (m, 1H), 1.88 (p, *J* = 7.3 Hz, 2H), 1.59 (s, 3H), 0.84 (t, *J* = 7.4 Hz, 3H); $^{13}$**C NMR** (101 MHz, $CDCl_3$) δ = 147.0, 141.0, 139.5, 128.9, 127.3, 127.2, 127.0, 125.5, 75.0, 36.8, 29.8, 8.5; **IR** (liquid film) ν = 3059, 3030, 2926, 2851, 1682, 1604, 1487, 1450, 1402, 1273, 1172, 1094, 1077, 1008, 916, 842, 767, 734, 699 $cm^{-1}$; No HRMS obtained.

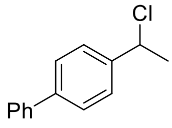


*rac*-**1a-Cl**

**4-(1-chloroethyl)-1,1'-biphenyl (1a-Cl)**

Compound *rac*-**1a-Cl** was prepared through the dropwise addition of thionyl chloride (1.5 equiv.) to a solution of 1-([1,1'-biphenyl)-4-yl)ethan-1-ol (1 equiv.) in $Et_2O$ (0.1 M) at 0 °C. The reaction mixture was stirred at 25 °C for 16 h. Upon completion, monitored by TLC, the reaction mixture was quenched with ice-cold water and washed with $NaHCO_3$ (sat). The aqueous phase was extracted with $Et_2O$, dried with $MgSO_4$ and concentrated under reduced pressure. Following purification (95:5 -80:20 pentane:$Et_2O$) the product was isolated as a white solid in 89 % yield. Spectroscopic data are in accordance with those in literature.[22]

**$^{1}$H NMR** (400 MHz, $CDCl_3$) δ = 7.69 – 7.58 (m, 4H), 7.57 – 7.45 (m, 4H), 7.45 – 7.35 (m, 1H), 5.19 (q, *J* = 6.8 Hz, 1H), 1.94 (d, *J* = 6.8 Hz, 3H); **$^{13}$C NMR** (101 MHz, $CDCl_3$) δ = 141.9, 141.4, 140.7, 128.9, 127.6, 127.5, 127.2, 127.1, 58.7, 26.6.

**Urea Catalyst Synthesis and Characterisation**
**Binaphthalene Synthesis**

Alkylated *bis*-anilines were prepared according to general procedure described. Aniline **7a** and **7b** spectroscopic data are in accordance with those in literature.[23,24,25]

General Procedure 6: In two neck round-bottom flask equipped with a stirrer bar, the appropriate ketone (1.4 equiv.) was dissolved in THF (0.25 M) and 20% aqueous $H_2SO_4$ solution (2 mL/mmol$_{substrate}$). (*S*)-(-)-1,1'-binapthalene-2,2'-diamine (1 equiv.) was added and after 5 minutes, the solution was cooled to 0 °C and $NaBH_4$ (10 equiv.) was added portion-wise. The mixture was brought up to room temperature and stirred for 1 hour, then quenched with 1M KOH solution to a pH of 8 and diluted with EtOAc. The mixture was extracted with EtOAc and combined organic phases were washed with brine prior to drying with $MgSO_4$. The filter organic phase was concentrated under reduced pressure and the crude product purified by FCC.

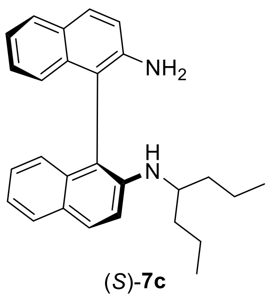


(*S*)-**7c**

**(*S*)-N$^2$ -heptan-5-yl-[1,1'-binaphthalene]-2,2'-diamine (7c)**

Prepared according to General Procedure 6 from (*S*)-(-)-1,1'-binapthalene-2,2'-diamine and 4-heptanone. Following purification (FCC eluent 95:5 – 90:10 pentane:$Et_2O$), the product was isolated as a as an off-white solid in 38 % yield.

**$^1$H NMR** (500 MHz, $CDCl_3$, 298 K) δ = 7.98 (d, *J* = 9.0 Hz, 1H), 7.95 – 7.84 (m, 3H), 7.37 (d, *J* = 5.2 Hz, 1H), 7.37 – 7.23 (m, 5H), 7.20 (d, *J* = 8.4, 1H), 7.16 – 7.04 (m, 1H), 3.80 (br s, 2H), 3.73 – 3.60 (m, 1H), 3.56 (br s, 1H), 1.58 – 1.40 (m, 5H), 1.40 – 1.28 (m, 2H), 1.28 – 1.17 (m, 1H), 0.98 (t, *J* = 7.0 Hz, 3H), 0.85 (t, *J* = 7.3 Hz, 3H); **$^{13}$C NMR** (126 MHz, $CDCl_3$, 298 K) δ = 144.5, 143.2, 134.1, 134.0, 129.6, 129.5, 128.6, 128.2, 128.1, 127.4, 126.8, 126.7, 124.4, 123.7, 122.5, 121.6, 118.3, 114.5, 112.6, 111.7, 52.4, 37.6, 37.5, 19.1, 18.7, 14.3, 14.16; **HRMS** (ESI$^+$) m/z calculated for $C_{27}H_{31}N_2$ $(M+H)^+$ 383.2482, found 383.2480; **IR** (neat) ν = 3057, 2981, 2971, 1620, 1598, 1512, 1463, 1380, 1350, 1249, 1152, 956, 813, 660 cm$^{-1}$; **mp** 153-154 ˚C; **[α]$^D$$_{25\,°C}$** = -121.0 ° (c = 1.0, $CHCl_3$).

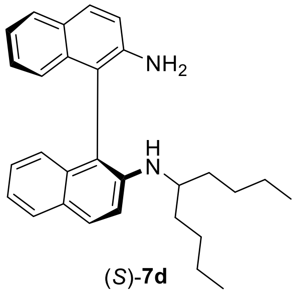


(*S*)-**7d**

**(*S*)-N$^2$ -nonan-7-yl-[1,1'-binaphthalene]-2,2'-diamine (7d)**

Prepared according to General Procedure 6 from (*S*)-(-)-1,1'-binapthalene-2,2'-diamine and 5-nonanone. Following purification (FCC eluent 95:5 – 90:10 pentane:$Et_2O$), the product was isolated as a viscous yellow oil in 21 % yield.

**$^1$H NMR** (500 MHz, $CDCl_3$, 298 K) δ = 7.88 (d, *J* = 9.0 Hz, 1H), 7.83 (d, *J* = 8.7 Hz, 1H), 7.79 – 7.76 (m, 2H), 7.28 – 7.11 (m, 6H), 7.10 (dd, *J* = 8.4, 1.2 Hz, 1H), 7.03– 6.99 (m, 1H), 3.69 (br s, 2H), 3.53 – 3.51

(m, 1H), 1.52 – 1.23 (m, 8H), 1.23 – 1.00 (m, 4H), 0.87 – 0.80 (t, *J* = 6.9 Hz, 3H), 0.70 (t, *J* = 7.1 Hz, 3H). **$^{13}$C NMR** (126 MHz, $CDCl_3$, 298 K) δ = 144.4, 143.2, 134.1, 134.0, 129.6, 129.5, 128.6, 128.2, 128.1, 127.3, 126.8, 126.8, 124.4, 123.8, 122.5, 121.6, 118.3, 114.5, 112.6, 111.8, 53.0, 35.0, 34.8, 28.1, 27.7, 22.9, 22.8, 14.2, 14.1 ; **HRMS** ($ESI^+$) m/z calculated for $C_{29}H_{35}N_2$ $(M+H)^+$ 411.2975, found 411.2789; **IR** (neat) ν = 3371, 3056, 2956, 2930, 2859, 1619, 1597, 1511, 1467, 1426, 1379, 1350, 1302, 1248, 1212, 1146, 1119, 1024, 930, 860, 811, 746, 666, 639 $cm^{-1}$; **$[\alpha]^D_{25\,°C}$** = -87.7 ° (c = 1.0, $CHCl_3$).

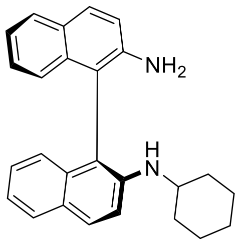

(*S*)-**7e**

**(*S*)-N$^2$ -cyclohexyl-[1,1'-binaphthalene]-2,2'-diamine (7e)**

Prepared according to General Procedure 6 from (*S*)-(-)-1,1'-binapthalene-2,2'-diamine and cyclohexanone. Following purification (FCC eluent 90:10 pentane:EtOAc), the product was isolated as an off-white solid in 30 % yield.

**$^1$H NMR** (500 MHz, $CDCl_3$) δ = 7.86 (d, *J* = 9.0 Hz, 1H), 7.84 – 7.78 (m, 2H), 7.77 (d, *J* = 7.6 Hz, 1H), 7.28 (d, *J* = 9.1 Hz, 1H), 7.23 (ddd, *J* = 8.0, 6.7, 1.3 Hz, 1H), 7.20 – 7.12 (m, 4H), 7.05 (dd, *J* = 8.4, 1.2 Hz, 1H), 6.97 (dd, *J* = 7.8, 1.7 Hz, 1H), 3.66 (br s, 2H), 3.53 – 3.50 (m, 1H), 3.41 (br s, 1H), 1.99 – 1.91 (m, 1H), 1.91 – 1.83 (m, 1H), 1.66 – 1.60 (m, 1H), 1.58 – 1.50 (m, 2H), 1.36 – 1.22 (m, 2H), 1.09 – 0.97 (m, 1H), 0.98 – 0.88 (m, 1H), 0.81 (tdd, *J* = 12.8, 10.2, 3.5 Hz, 1H). **$^{13}$C NMR** (126 MHz, $CDCl_3$, 298 K) δ = 144.0, 143.0, 134.1, 133.9, 129.6, 129.5, 128.6, 128.2, 128.1, 127.7, 126.8, 126.7, 124.3, 123.9, 122.5, 121.9, 118.4, 115.4, 112.7, 112.5, 52.3, 33.9, 33.8, 25.8, 25.2, 25.1; **HRMS** ($ESI^+$) m/z calculated for $C_{29}H_{26}N_2$ $(M+H)^+$ 367.2169, found 367.2166; **IR** (neat) ν = 3460, 3367, 3051, 2931, 2846, 1947, 1618, 1596, 1513, 1488, 1449, 1425, 1382, 1348, 1291, 1250, 1216, 1147, 1110, 1052, 1022, 962, 920, 813, 774, 754, 687, 671, 634, 623 $cm^{-1}$; **mp** 138-140 ˚C; **$[\alpha]^D_{25\,°C}$** = -143.5 ° (c = 1.0, $CHCl_3$).

**Catalyst Synthesis**

Catalysts (*S*)-**3a-d** and **k** were synthesised according to literature procedure and spectroscopic data matches literature.[21,22]

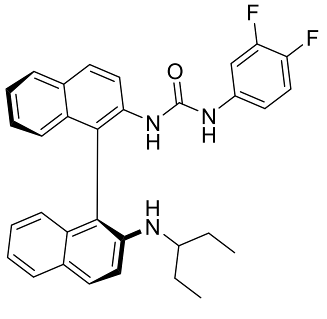

(*S*)-**8g**

**(*S*)-1-(3,4-difluoromethylphenyl)-3-(2'-(pentylamino)-[1,1'-binaphthalen]-2-yl)urea (8g)**

In a flame-dried Schlenk under inert atmosphere, aniline **7b** (300 mg, 0.92 mmol, 1 equiv.) was dissolved in dry DCM (0.4 M) and 3,4-difluorophenyl isocyanate (1 equiv.) was added dropwise. The mixture was stirred for 30 minutes until full consumption of starting material was seen by TLC. The reaction was quenched with MeOH and solvent was

removed under reduced pressure. The crude mixture was purified (FCC eluent: DCM:pentane 90:10 – 100:0) to afford desired product as a white solid in 92 % yield.

**$^{1}$H NMR** (500 MHz, $CDCl_3$) δ = 8.50 (d, *J* = 9.1 Hz, 1H), 7.98 (d, *J* = 9.1 Hz, 1H), 7.89 (d, *J* = 8.1 Hz, 1H), 7.82 (d, *J* = 9.0 Hz, 1H), 7.73 (d, *J* = 7.9 Hz, 1H), 7.41 – 7.36 (m, 1H), 7.25 – 7.19 (m, 1H), 7.19 – 7.07 (m, 4H), 6.86 (br t, *J* = 7.9 Hz, 1H), 6.75 (d, *J* = 8.2 Hz, 1H), 6.71 (q, *J* = 9.6 Hz, 1H), 6.55 (s, 1H), 6.47 – 6.37 (m, 1H), 6.33 (br s, 1H), 3.47 – 3.30 (m, 1H), 3.27 (br s, 1H), 1.45 – 1.27 (m, 2H), 1.27 – 1.10 (m, 2H), 0.73 (t, *J* = 7.4 Hz, 3H), 0.57 (t, *J* = 7.4 Hz, 3H); **$^{19}$F NMR** (471 MHz, $CDCl_3$, 298 K ) δ = -134.5 (br), - 141.4 (br); **$^{13}$C NMR{$^{1}$H, $^{19}$F}** (126 MHz, $CDCl_3$) [overlapping signals] δ = 153.2, 150.1, 147.7, 144.2 (br), 135.6, 133.8, 133.5, 132.8, 131.0, 130.3, 129.5, 128.3, 127.3, 127.2, 126.9, 125.4, 125.0, 123.2, 122.1, 120.3, 119.7, 118.9, 117.4, 114.4, 112.5, 109.5 (br), 55.5 (br), 27.2, 27.1, 10.1, 9.9; **IR** (liquid film) ν = 3649, 3353, 3021, 2943, 2761, 1645, 1609, 1541, 1510, 1447, 1409, 1215, 996, 752; **HRMS** (ESI$^{+}$) m/z calculated for $C_{32}H_{30}F_2N_3O$ $(M+H)^+$ 510.2351, found 510.2346; **mp** 171-175 ˚C; **[α]$^{D}$$_{25\,°C}$** = -104.1 ° (c = 0.5, $CHCl_3$).

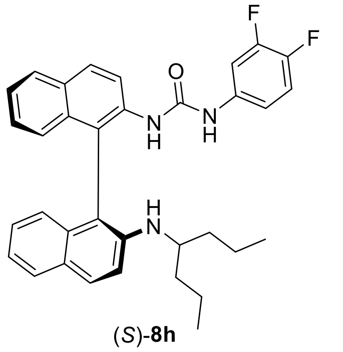
(*S*)-**8h**

**(*S*)-1-(3,4-difluoromethylphenyl)-3-(2'-(heptylamino)-[1,1'-binaphthalen]-2-yl)urea (8h)**

In a flame-dried Schlenk under inert atmosphere, aniline **7c** (300 mg, 0.92 mmol, 1 equiv.) was dissolved in dry DCM (0.4 M) and 3,4-difluorophenyl isocyanate (1 equiv.) was added dropwise. The mixture was stirred for 30 minutes at room temperature until full conversion of starting material was seen by TLC. The reaction was quenched with MeOH and solvent was removed under reduced pressure. The crude mixture was purified (FCC eluent: pentane: $Et_2O$ 90:10) to afford the desired product as a white solid in 95 % yield.

**$^{1}$H NMR** (500 MHz, $CDCl_3$, 298 K) δ = 8.50 (d, *J* = 9.0 Hz, 1H), 7.98 (d, *J* = 9.1 Hz, 1H), 7.89 (d, *J* = 8.2 Hz, 1H), 7.82 (d, *J* = 9.0 Hz, 1H), 7.73 (d, *J* = 7.8 Hz, 1H), 7.39 (t, *J* = 7.5 Hz, 1H), 7.22 (t, *J* = 7.5 Hz, 1H), 7.18 – 7.07 (m, 4H), 6.89 (ddd, *J* = 10.6, 7.0, 2.6 Hz, 1H), 6.75 (dd, *J* = 16.6, 8.7 Hz, 1H), 6.70 (q, *J* = 8.8 Hz, 1H), 6.56 (s, 1H), 6.42 (dd, *J* = 8.7, 4.0 Hz, 1H), 6.28 (s, 1H), 3.50 – 3.42 (m, 1H), 3.20 (s, 1H), 1.35 – 0.95 (m, 8H), 0.77 (t, *J* = 6.7 Hz, 3H), 0.68 (t, *J* = 6.7 Hz, 3H); **$^{19}$F NMR** (471 MHz, $CDCl_3$, 298 K ) δ = - 134.5 (br), -141.5 (br); **$^{13}$C NMR{$^{1}$H, $^{19}$F}** (125 MHz, $CDCl_3$, 298 K) δ = 153.2, 150.1, 147.7, 146.7, 144.5, 139.6, 135.6, 133.9, 133.6, 132.8, 131.0, 130.3, 129.5, 128.3, 127.3, 126.9, 125.4, 125.0, 123.1, 122.0, 120.3, 120.0, 118.5, 118.3, 117.4, 116.8, 114.2, 52.3, 37.5, 37.4, 19.2, 18.7, 14.2, 14.1; **HRMS** (ESI$^{+}$) m/z calculated for $C_{34}H_{34}F_2N_3O$ $(M+H)^+$ 538.2664, found 538.2657; **IR** (liquid film) ν = 3350, 3104, 2873, 1744, 1716, 1684, 1621, 1599, 1503, 1451, 1231, 1023, 939, 747 $cm^{-1}$; **mp** 108-110 ˚C; **[α]$^{D}$$_{25\,°C}$** = -64.4 ° (c = 1.0, $CHCl_3$).

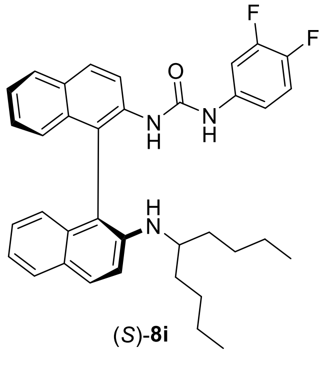

$(S)$-**8i**

**(*S*)-1-(3,4-difluorophenyl)-3-(2'-(nonan-5-ylamino)-[1,1'-binaphthalen]-2- yl)urea (8i)**

In a flame-dried Schlenk under inert atmosphere, aniline **7d** (200 mg, 0.92 mmol, 1 equiv.) was dissolved in dry DCM (0.4 M) and 3,4-difluorophenyl isocyanate (1 equiv.) was added dropwise. The mixture was stirred for 30 minutes at room temperature until full conversion of starting material was seen by TLC. The reaction was quenched with MeOH and solvent was removed under reduced pressure. The crude mixture was purified (FCC eluent: pentane: $Et_2O$ 90:10) to afford the desired product as a white solid in 68 % yield.

**$^1$H NMR** (500 MHz, $CDCl_3$) δ = 8.56 (dd, *J* = 9.1, 3.2 Hz, 1H), 7.99 (d, *J* = 9.1 Hz, 1H), 7.89 (d, *J* = 8.1 Hz, 1H), 7.85 (d, *J* = 9.0 Hz, 1H), 7.75 (d, *J* = 8.0 Hz, 1H), 7.38 (ddd, *J* = 8.0, 6.6, 1.2 Hz, 1H), 7.25 – 7.07 (m, 5H), 6.95 (s, 1H), 6.80 – 6.70 (m, 2H), 6.54 (s, 1H), 6.49 (s, 1H), 6.35 (s, 1H), 3.43 (br s, 1H), 3.23 (br s, 1H), 1.42 – 1.31 (m, 2H), 1.31 – 1.24 (m, 4H), 1.22 – 1.17 (m, 2H), 1.17 – 1.07 (m, 2H), 1.06 – 0.91 (m, 1H), 0.90 – 0.81 (m, 1H), 0.78 (t, *J* = 6.7 Hz, 3H), 0.65 (t, *J* = 7.2 Hz, 3H); **$^{19}$F NMR** (471 MHz, $CDCl_3$) δ = -134.6, -141.6; **$^{13}$C NMR{$^1$H, $^{19}$F}** (125 MHz, $CDCl_3$, 298 K) δ = [overlapping signals] 153.1, 150.1, 147.4, 143.3 (br), 135.9, 133.9, 133.8, 132.9, 130.9, 130.5, 129.7, 128.3, 127.7 (br), 127.5, 126.9, 125.3, 125.0, 123.5, 122.6 (br), 120.3, 119.2, 117.8, 117.3, 114.9, 111.7, 53.8 (br), 34.5 (br), 34.3 (br), 28.2, 27.4, 22.8, 22.6, 14.1, 14.0; **HRMS** ($ESI^+$) m/z calculated for $C_{36}H_{38}F_2N_3O$ $(M+H)^+$ 566.2977, found 566.2964; **IR** (neat) ν = 2958, 2928, 2857, 1740, 1720, 1658, 1621, 1599, 1563, 1516, 1498, 1465, 1429, 1379, 1330, 1281, 1262, 1227, 1206, 1153, 1115, 1024, 908, 864, 811, 807, 745, 654, 623 $cm^{-1}$; **mp** =112-114 ˚C; **$[\alpha]^D_{25\,°C}$** = -64.18° (c = 0.4, $CHCl_3$).

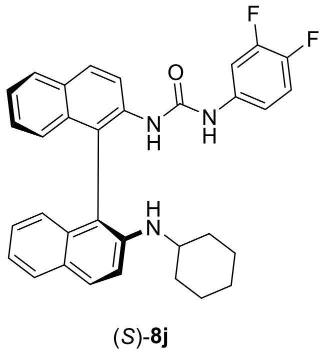

$(S)$-**8j**

**(*S*)-1-(2'-(cyclohexylamino)-[1,1'-binaphthalen]-2- yl)-3-(3,4-difluoropheyl)urea (8j)**

In a flame-dried Schlenk under inert atmosphere, aniline **7d** (300 mg, 0.92 mmol, 1 equiv.) was dissolved in dry DCM (0.4 M) and 3,4-difluorophenyl isocyanate (1 equiv.) was added dropwise. The mixture was stirred for 30 minutes at room temperature until full conversion of starting material was seen by TLC. The reaction was quenched with MeOH and solvent was removed under reduced pressure. The crude mixture was purified (FCC eluent: pentane: $Et_2O$ 90:10) to afford the desired product as a white solid in 95 % yield.

**$^1$H NMR** (500 MHz, $CDCl_3$) δ = 8.50 (d, *J* = 9.1, 1H), 7.98 (d, *J* = 9.1 Hz, 1H), 7.89 (d, *J* = 8.1 Hz, 1H), 7.86 (d, *J* = 9.1 Hz, 1H), 7.77 (d, *J* = 7.9, 1H), 7.39 (t, *J* = 7.6 Hz, 1H), 7.25 – 7.18 (m, 3H), 7.12 (t, *J* = 7.5 Hz,

1H), 7.08 (d, *J* = 8.4 Hz, 1H), 6.96 – 6.92 (m, 1H), 6.78 (d, *J* = 8.2 Hz, 1H), 6.77 – 6.71 (m, 1H), 6.55 – 6.43 (m, 3H), 3.57 (br s, 1H), 3.39 – 3.31 (m, 1H), 1.84 (d, *J* = 11 Hz, 1H), 1.78 (d, *J* = 11 Hz, 1H) 1.59 – 1.48 (m, 3H), 1.24 – 1.15 (m, 2H), 1.05 – 0.95 (m, 1H), 0.91 – 0.81 (m, 2H), 0.80– 0.70 (m, 1H); **$^{19}$F NMR** (471 MHz, $CDCl_3$) δ = -134.8, -141.9; **$^{13}$C NMR{$^{1}$H, $^{19}$F}** (125 MHz, $CDCl_3$, 298 K) δ = 153.0, 150.1, 147.6, 142.9, 135.7, 133.7, 133.7, 132.8, 130.9, 130.4, 129.7, 128.3, 128.3, 127.9, 127.4, 127.0, 125.3, 125.0, 123.6, 122.7, 120.2, 119.2, 118.2, 117.4, 115.3, 112.0, 111.3, 52.6, 33.5, 33.2, 25.5, 24.9, 24.9; **HRMS** ($ESI^+$) m/z calculated for $C_{33}H_{30}F_2N_3O$ $(M+H)^+$ 522.2351, found 522.2342; **IR** (neat) ν = 2969, 2932, 2581, 2849, 1739, 1719, 1657, 1620, 1599, 1567, 1518, 1502, 1452, 1428, 1378, 1367, 1347, 1287, 1262, 1230, 1214, 1207, 1150, 1113, 957, 867, 810, 772, 747, 665, 646, 623 $cm^{-1}$; **mp** 110-113 ˚C; **$[\alpha]^{D}_{25\,°C}$** = -81.6° (c = 0.4, $CHCl_3$).

**Urea catalysts:**

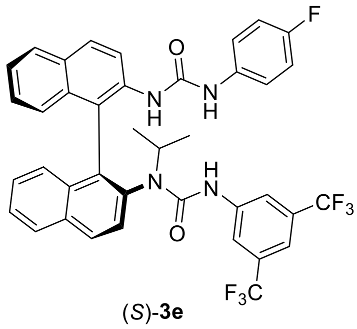


(*S*)-**3e**

**(*S*)-3-(3,5-bis(trifluoromethyl)phenyl)-1-isopropyl-1-(2'-(3-(4-fluorophenyl)ureido)-[1,1'-binaphthalen]-2- yl)urea (3e)**

In a flame-dried Schlenk under inert atmosphere, aniline **7a** (300 mg, 0.92 mmol, 1 equiv.) was dissolved in dry DCM (0.4 M) and 4-fluorophenyl isocyanate (1 equiv.) was added dropwise. The mixture was stirred for 10 minutes at room temperature until full conversion of starting material was seen by TLC. 3,5-bistrifluoromethylphenyl isocyanate (1 equiv.) was added dropwise and mixture was stirred at reflux for 24 h. The reaction was quenched with MeOH and solvent was removed under reduced pressure. The crude mixture was purified (FCC eluent: 1$^{st}$ column pentane: EtOAc 80:20, 2$^{nd}$ column 100% DCM) and recrystallised in MeCN to afford the desired product as a white solid in 63 % yield.

**$^{1}$H NMR** (500 MHz, $CDCl_3$, 298 K) δ = 8.49 (d, *J* = 8.9 Hz, 1H), 8.10 (d, *J* = 8.4 Hz, 1H), 7.98 (d, *J* = 9.0 Hz, 2H), 7.89 (d, *J* = 8.1 Hz, 1H), 7.78 (br s, 2H), 7.55 (t, *J* = 7.3 Hz, 1H), 7.51 (d, *J* = 8.8 Hz, 1H), 7.46 (s, 1H), 7.35 (t, *J* = 7.5 Hz, 1H), 7.31 (t, *J* = 7.4 Hz, 1H), 7.24–7.05 (m, 3H), 6.93 – 6.80 (m, 3H), 6.79 – 6.53 (m, 4H), 3.53 (br s, 1H), 1.02 (br s, 3H), 0.67 (br s, 3H); **$^{19}$F NMR** (471 MHz, $CDCl_3$, 298 K ) δ = -62.9 (s), -120.4 (br s); **$^{13}$C NMR{$^{1}$H, $^{19}$F}** (125 MHz, $CDCl_3$, 298 K) δ = 158.8 (br), 155.3 (br), 152.8, 140.1, 139.8, 136.1 (br), 133.8, 133.2, 132.3, 132.1, 130.8, 130.3, 129.9, 128.7, 128.4, 128.2, 127.9, 127.8, 127.5, 127.0, 126.9 (br), 125.0 (br), 124.6, 123.2, 120.9 (br), 120.2, 120.0, 119.8, 116.9 (br), 115.5 (br), 53.7 (br), 20.8 (br), 20.6; **HRMS** ($ESI^+$) m/z calculated for $C_{39}H_{30}N_4O_2F_7$ $(M+H)^+$ 719.2252, found 719.2252; **IR** (solid) ν = 3658, 2981, 2888, 1665, 1545, 1504, 1473, 1439, 1385, 1278, 1216, 1178, 1138, 1073, 966, 940, 884, 825, 754, 701, 682 $cm^{-1}$; **mp** 151–154 ˚C; **$[\alpha]^{D}_{25\,°C}$** = -49.8 ° (c = 1.0, $CHCl_3$).

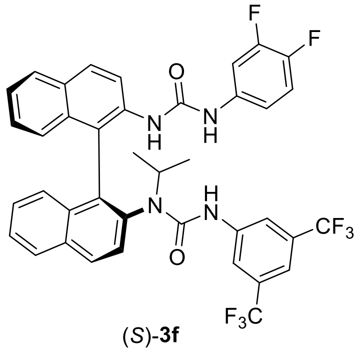

(*S*)-**3f**

**(*S*)-3-(3,5-bis(trifluoromethyl)phenyl)-1-isopropyl-1-(2'-(3-(3,4-difluorophenyl)ureido)-[1,1'-binaphthalen]-2- yl)urea (3f)**

In a flame-dried Schlenk under inert atmosphere, aniline 7**a** (300 mg, 0.92 mmol, 1 equiv.) was dissolved in dry DCM (0.4 M) and 3,4-difluorophenyl isocyanate (1 equiv.) was added dropwise. The mixture was stirred for 30 minutes at room temperature until full conversion of starting material was seen by TLC. 3,5-bistrifluoromethylphenyl isocyanate (1 equiv.) was added dropwise and mixture was stirred at reflux for 24 hours. The reaction was quenched with MeOH and solvent was removed under reduced pressure. The crude mixture was purified (FCC eluent DCM:pentane 80:20) and recrystallised in MeCN to afford the desired product as a white solid in 64 % yield.

**$^{1}$H NMR** (500 MHz, $CDCl_3$, 298 K) δ = 8.50 (d, *J* = 8.9 Hz, 1H), 8.11 (d, *J* = 8.7 Hz, 1H), 8.00 (d, *J* = 9.1 Hz, 1H), 7.99 (d, *J* = 8.1 Hz, 1H), 7.90 (d, *J* = 8.1 Hz, 1H), 7.80 (br s, 2H), 7.57 – 7.45 (m, 2H), 7.47 (s, 1H), 7.37 (t, *J* = 7.2 Hz, 1H), 7.29 (t, *J* = 7.7 Hz, 1H), 7.21 (t, *J* = 7.4 Hz, 1H), 7.18 (d, *J* =8.6 Hz, 1H), 7.09 (br s, 1H), 6.92 (br s, 1H), 6.86 (d, *J* = 8.5 Hz, 2H), 6.85 – 6.73 (m, 2H), 6.54–6.45 (m, 1H), 3.58 (br s, 1H), 1.03 (d, *J* = 6.2 Hz, 3H), 0.66 (br s, 3H), **$^{19}$F NMR** (471 MHz, $CDCl_3$, 298 K ) δ = -63.0 (s), - 136.6 (br s), - 145.4 (br s); **$^{13}$C NMR{$^{1}$H, $^{19}$F}** (125 MHz, $CDCl_3$, 298 K) [overlapping signals] δ = 155.6 (br), 152.3, 150.0, 146.2 (br), 140.0, 139.6, 136.0, 134.8 (br), 133.9, 133.3, 133.2, 132.4, 132.0, 130.7, 130.3, 129.9, 128.7, 128.3, 128.1, 127.7, 127.4, 127.1, 127.0, 125.2, 124.6, 123.2, 120.1, 120.0, 117.1, 116.9, 114.3 (br), 108.5 (br), 53.5 (br), 20.9 (br), 20.7; **HRMS** ($ESI^+$) m/z calculated for $C_{39}H_{29}N_4O_2F_8$ $(M+H)^+$ 759.1977, found 759.1976; **IR** (liquid film) ν = 3337, 2981, 2882, 2351, 1722, 1681, 1462, 1382, 1285, 1250, 1151, 1072, 955, 863, 802, 774, 712, 690, 577, 668, 653, 643, 622, 607 $cm^{-1}$; **mp** 149–150 ˚C; **$[\alpha]^{D}_{25 °C}$** = -67.7 ° (c = 1.0, $CHCl_3$).

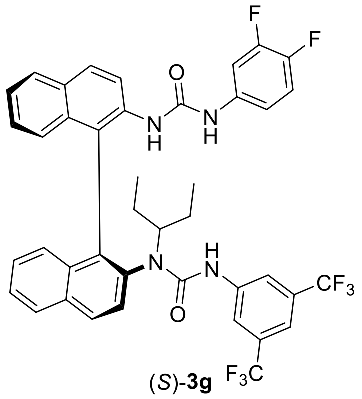

(*S*)-**3g**

**(*S*)-3-(3,5-bis(trifluoromethyl)phenyl)-1-pentyl-1-(2'-(3-(3,4-difluorophenyl)ureido)-[1,1'-binaphthalen]-2- yl)urea (3g)**

In a flame-dried Schlenk under inert atmosphere, intermediate **8g** was dissolved in dry DCM (0.4 M) and 3,5-*bis*(trifluoromethyl)phenyl isocyanate (1 equiv.) was added dropwise. The mixture was refluxed for 72 hours. The reaction was quenched with MeOH and solvent was removed under reduced pressure. The crude reaction was (FCC eluent: 1st column 95:5 pentane:$Et_2O$ to 80:20, 2nd column 100% $CHCl_3$) to afford the desired product as a white solid in 48 % yield.

**$^{1}$H NMR** (500 MHz, $CDCl_3$, 298 K) δ = 8.57 (d, *J* = 9.1 Hz, 1H), 8.10 (d, *J* = 8.8 Hz, 1H), 8.01 (d, *J* = 9.2 Hz, 1H), 7.97 (d, *J* = 8.1 Hz, 1H), 7.92 (d, *J* = 8.1 Hz, 1H), 7.82 (s, 2H), 7.62 (d, *J* = 8.8 Hz, 1H), 7.57 – 7.50 (m, 2H), 7.38 (ddd, *J* = 8.0, 6.7, 1.1 Hz, 1H), 7.32 – 7.26 (m, 2H), 7.25 – 7.15 (m, 2H), 7.13 (d, *J* = 8.5 Hz, 1H), 6.94 (d, *J* = 8.4 Hz, 1H), 6.91 (br s, 1H), 6.88 – 6.75 (m, 2H), 6.57 (d, *J* = 8.9 Hz, 1H), 2.97 (s, 1H), 1.74 (s, 1H), 1.64 (m, 1H), 0.98 (s, 3H), 0.87 (s, 1H), 0.53 (s, 1H), -0.21 (s, 3H); **$^{19}$F NMR** (471 MHz, $CDCl_3$, 298 K ) δ = -63.0 (s), -136.6 (br s), -145.4 (br s); **$^{13}$C NMR{$^{1}$H, $^{19}$F}** (126 MHz, $CDCl_3$) [overlapping signals] δ = 156.0 (br), 152.1, 150.1, 146.2, 142.1, 139.5, 136.2, 134.9, 133.8, 133.2, 133.1, 132.5, 130.8, 130.4, 130.3, 130.0, 128.8, 128.3, 127.5, 127.3, 127.3, 127.0, 125.2, 124.7, 123.1, 119.9, 119.8, 117.3, 116.9, 114.2 (br), 108.3 (br), 66.7, 28.9 (br), 26.4, 12.9, 11.1; **HRMS** (ESI$^+$) m/z calculated for $C_{41}H_{33}F_8N_4O_2$ $(M+H)^+$ 765.2470, found 765.2462; **IR** (liquid film) ν = 3649, 2067, 2994, 2954, 1685, 1602, 1541, 1520, 1475, 1279, 1252, 1118, 970, 886 cm$^{-1}$; **mp** 119-122 ˚C; **[α]$^{D}$$_{25\,°C}$** = -88.6 ° (c = 0.5, $CHCl_3$).

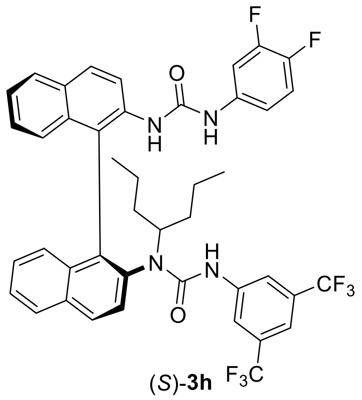
(*S*)-**3h**

**(*S*)-3-(3,5-bis(trifluoromethyl)phenyl)-1-heptyl-1-(2'-(3-(3,4-difluorophenyl)ureido)-[1,1'-binaphthalen]-2- yl)urea (3h)**

In a flame-dried Schlenk under inert atmosphere, intermediate **8h** was dissolved in dry DCM (0.4 M) and 3,5-*bis*(trifluoromethyl)phenyl isocyanate (1 equiv.) was added dropwise. The mixture was refluxed for 72 hours. The reaction was quenched with MeOH and solvent was removed under reduced pressure. The crude reaction was purified (FCC eluent: 1$^{st}$ column 95:5 pentane:$Et_2O$ to 80:20, 2$^{nd}$ column 100% $CHCl_3$) to afford the desired product as a white solid in 40 % yield.

**$^{1}$H NMR** (600 MHz, $CDCl_3$, 298 K) δ = 8.60 (d, *J* = 9.1 Hz, 1H), 8.10 (d, *J* = 8.8 Hz, 1H), 8.02 (d, *J* = 9.1 Hz, 1H), 7.98 (d, *J* = 8.2 Hz, 1H), 7.91 (d, *J* = 8.2 Hz, 1H), 7.84 (s, 2H), 7.60 (d, J = 8.8 Hz, 1H), 7.57 – 7.51 (m, 2H), 7.38 (t, *J* = 7.3 Hz, 1H), 7.34 – 7.26 (m, 2H), 7.26 – 7.16 (m, 2H), 7.14 (d, *J* =8.5 Hz, 1H), 7.00 – 6.89 (m, 2H), 6.88 – 6.70 (m, 2H), 6.58 (d, *J* = 8.9 Hz, 1H), 3.15 – 3.00 (m, 1H), 1.78 (s, 1H), 1.52 – 1.45 (m, 2H), 1.30 (s, 2H), 0.78 (t, *J* = 6.9 Hz, 3H), 0.57 (s, 1H), 0.34 (m, 4H), -0.69 (s, 1H); **$^{19}$F NMR** (565 MHz, $CDCl_3$, 298 K) δ = -62.9 (s), -136.6 (br s), -145.5 (br s); **$^{13}$C NMR{$^{1}$H, $^{19}$F}** (125 MHz, $CDCl_3$, 298 K) [overlapping signals] δ = 155.9 (br), 152.2, 150.1, 146.2, 142.1, 139.6, 136.2, 135.0, 133.8, 133.2, 133.1, 132.5, 130.8, 130.4, 130.3, 129.9, 128.7, 128.3, 128.2, 127.5, 127.3, 127.3, 127.1, 125.7, 125.2, 124.7, 124.3, 123.2, 122.0, 119.9 (br), 117.3, 116.9, 114.2, 108.3 (br), 63.8, 38.7 (br), 36.3, 22.8, 20.5, 14.3, 14.0; **IR** (neat) ν = 3367, 2926, 2872, 2855, 1717, 1686, 1620, 1519, 1505, 1472, 1432, 1381, 1278, 1209, 1180, 1133, 1037, 1016, 936, 882, 817, 752, 700, 683, 610 cm$^{-1}$; **mp** 142–144 ˚C; **HRMS** (ESI$^+$) m/z calculated for $C_{43}H_{37}F_8N_4O_2$ $(M+H)^+$ 793.2783, found 793.2770; **[α]$^{D}$$_{25\,°C}$** = -83.2° (c = 1.0, $CHCl_3$).

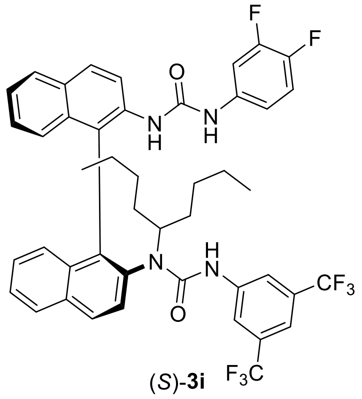


(*S*)-**3i**

**(*S*)-3-(3,5-bis(trifluoromethyl)phenyl)-1-nonan-5-yl-1-(2'-(3-(3,4-difluorophenyl)ureido)-[1,1'-binaphthalen]-2- yl)urea (3i)**

In a flame-dried Schlenk under inert atmosphere, intermediate **8i** was dissolved in dry DCM (0.4 M) and 3,5-*bis*(trifluoromethyl)phenyl isocyanate (1 equiv.) was added dropwise. The mixture was refluxed for 72 hours. The reaction was quenched with MeOH and solvent was removed under reduced pressure. The crude reaction was (FCC eluent: 1st column 95:5 pentane:$Et_2O$ to 80:20, 2nd column 100% $CHCl_3$) to afford the desired product as a white solid 26 % yield.

**$^1$H NMR** (500 MHz, $CDCl_3$) δ = 8.57 (d, *J* = 9.1 Hz, 1H), 8.10 (d, *J* = 8.8 Hz, 1H), 8.01 (d, *J* = 9.2 Hz, 1H), 7.97 (d, *J* = 8.1 Hz, 1H), 7.90 (d, *J* = 8.1 Hz, 1H), 7.84 (s, 2H), 7.60 (d, *J* = 8.8 Hz, 1H), 7.57 – 7.49 (m, 2H), 7.44 – 7.32 (m, 2H), 7.31 – 7.26 (m, 2H), 7.24 – 7.17 (m, 1H), 7.12 (d, *J* = 8.5 Hz, 1H), 7.03 -6.91 (m, 2H), 6.90 – 6.77 (m, 2H), 6.57 (d, *J* = 8.9 Hz, 1H), 3.08 (s, 1H), 1.78 (br s, 1H), 1.60 (br s, 1H), 1.58 -1.50 (m, 1H), 1.43 (br s, 1H), 1.21 – 1.05 (m, 2H), 0.96 – 0.85 (m, 1H), 0.82 (t, *J* = 7.2 Hz, 3H), 0.80 – 0.67 (m, 1H) 0.66 – 0.52 (m, 4H), 0.50 – 0.27 (m, 2H), -0.71 (br s, 1H); **$^{19}$F NMR** (471 MHz, $CDCl_3$) δ = -63.0 (s), -136.5 (br s), -145.3 (br s); **$^{13}$C NMR{$^1$H, $^{19}$F}** (125 MHz, $CDCl_3$, 298 K) δ = 156.0, 152.2, 150.1, 146.2, 142.2, 140.3, 139.5, 136.1, 134.9, 133.8, 133.2, 133.1, 132.6, 132.2, 130.8, 130.4, 130.0, 128.8, 128.3, 128.2, 127.5, 127.3, 127.1, 125.3, 124.7, 123.1, 119.8, 118.8, 117.3, 116.9, 116.2, 114.2, 108.4, 63.9, 36.7, 33.4, 30.2, 29.9, 22.8, 22.5, 14.0, 13.9; **HRMS** (ESI$^+$) m/z calculated for $C_{45}H_{41}F_8N_4O_2$ 821.3096, 821.3083; **IR** (neat) ν = 3814, 3692, 3335, 1711, 1688, 1659, 1623, 1505, 1474, 1437, 1383, 1280, 1211, 1182, 1137, 882, 757, 683, 671, 657, 646, 620 cm$^{-1}$; **mp** 108-109 ˚C; **[α]$^D$$_{25\,°C}$** = -53.7° (c = 0.2, $CHCl_3$).

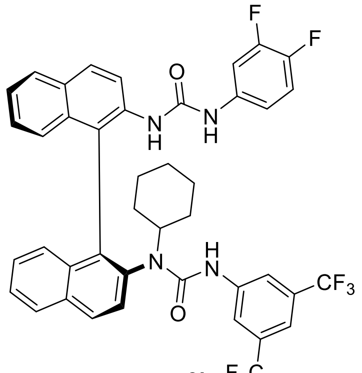


(*S*)-**3j**

**(*S*)-3-(3,5-bis(trifluoromethyl)phenyl)-1-cyclohexyl-1-(2'-(3-(3,4-difluorophenyl)ureido)-[1,1'-binaphthalen]-2- yl)urea (3j)**

In a flame-dried Schlenk under inert atmosphere, intermediate **8h** was dissolved in dry DCM (0.4 M) and 3,5-*bis*(trifluoromethyl)phenyl isocyanate (1 equiv.) was added dropwise. The mixture was refluxed for 72 hours. The reaction was quenched with MeOH and solvent was removed under reduced pressure. The crude reaction was (FCC eluent: 1st column 90:10 pentane:$Et_2O$ to 80:20, 2nd column 100% $CHCl_3$) to afford the desired product as a white solid in 50 % yield.

**$^1$H NMR** (500 MHz, $CDCl_3$) δ = 8.42 (d, *J* = 9.1 Hz, 1H), 8.09 (d, *J* = 8.7 Hz, 1H), 7.98 (d, *J* = 9.2 Hz, 1H), 7.96 (d, *J* = 8.0 Hz, 1H), 7.92 (d, *J* = 8.2 Hz, 1H), 7.81 (s, 2H), 7.57 – 7.52 (m, 2H), 7.51 (br s, 1H), 7.42 (t,

*J* = 7.5 Hz, 1H), 7.38 – 7.26 (m, 3H), 7.25 (d, *J* = 8.3 Hz, 1H), 7.10 (br s, 1H), 7.02 – 6.90 (m, 3H), 6.82 (q, *J* = 9.2 Hz, 1H), 6.49 (d, *J* = 8.6 Hz, 1H), 3.16 (br s, 1H), 1.80 (s, 1H), 1.54 (d, *J* = 12.5 Hz, 1H), 1.40 (s, 1H), 1.33 – 1.18 (m, 3H), 0.99 – 0.82 (m, 1H), 0.80 – 0.51 (m, 2H), 0.06 (br s, 1H); **$^{19}$F NMR** (471 MHz, $CDCl_3$) δ = -62.9 (s), -136.6 (br s), -145.3 (br s); **$^{13}$C NMR{$^{1}$H, $^{19}$F}** (125 MHz, $CDCl_3$, 298 K) δ = 155.7, 152.4, 149.9, 146.2, 140.2, 139.7, 135.9, 134.7, 133.9, 133.1, 133.1, 132.3, 131.7, 130.6, 130.4, 129.8, 128.7, 128.3, 128.2, 127.6, 127.3, 127.0, 126.9, 125.5, 124.7, 123.2, 120.4, 120.2, 120.1, 117.0, 116.8, 114.4, 108.4, 62.5, 31.7, 30.8, 26.1, 25.7, 25.0; **HRMS** ($ESI^+$) m/z calculated for $C_{42}H_{33}F_8N_4O_2$ $(M+H)^+$ 777.2470, found 777.2449; **IR** (neat) ν = 3359, 2933, 2860, 2360, 1683, 1621, 1539, 1505, 1473, 1432, 1383, 1339, 1278, 1209, 1180, 1134, 1040, 1003, 909, 882, 844, 804, 750, 735, 701, 682, 650, 627 $cm^{-1}$; **mp** 154-155 ˚C; **$[\alpha]^{D}_{25\,°C}$** = -80.2° (c = 0.5, $CHCl_3$).

**General Procedure for Enantioselective Benzylic Fluorination**

For reaction optimisation: In a 1.75 mL screw-cap vial equipped with a magnetic stirring bar were sequentially added, pre-ground potassium fluoride (2.5-5 equiv.), the appropriate substrate (0.05 mmol, 1 equiv.), urea catalyst (10–20 mol%), onium co-catalyst (0–100 mol%) and solvent (0.25-0.5M). The vial was sealed, and reaction was stirred at 1200 rpm at the appropriate temperature for 24-72h. The crude reaction was filtered over celite, solvent removed under reduced pressure in a polypropylene vial. Samples were dissolved in deuterated solvent and analysed by $^{1}H$ and $^{19}F$ NMR (4-fluoroanisole used as an internal standard).

For substrate scope (General Procedure 7): In a 7 mL screw-cap vial equipped with a magnetic stirring bar were sequentially added, pre-ground potassium fluoride (2.5 equiv.), the appropriate substrate (0.16 – 0.38 mmol, 1 equiv.), (*S*)-**3h** (10 mol%), $Ph_4P^+ I^-$ (10 mol%) and *p*-xylene (0.25 M). The vial was sealed, and reaction was stirred at 1200 rpm at the appropriate temperature for specified time. The crude reaction mixture was directly purified by FCC to give product. Solvent was removed in PFA round bottom flask and products were stored in polypropylene vials at -20 °C.

For gram scale reaction: In a 50 mL round bottom flask equipped with a magnetic stirring bar were sequentially added, pre-ground potassium fluoride (2.5 equiv.), 8-(1-bromoethyl)quinoline (1.1g, 4.7 mmol), (*S*)-**3h** (10 mol%), $Ph_4P^+ I^-$ (10 mol%) and *p*-xylene (19 mL, 0.25 M). The flask was sealed, and the reaction was stirred at 1200 rpm at 25 °C for 72 h. The crude reaction mixture was directly purified by FCC to give product. Solvent was removed in PFA round bottom flask and products were stored in polypropylene vials at -20 °C.

**General Procedure for Synthesis of Racemic Standards**

Racemic standards for all products were prepared by either (i) deoxyfluorination from corresponding alcohol or (ii) TBAF fluorination of corresponding bromide to produce racemic fluoride products – these were purified by preparative TLC for HPLC.

**Product Characterisation – Benzylic Fluorides**

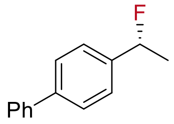


(*R*)-**2a**

**(*R*)-4-(1-fluoroethyl)-1,1'-biphenyl (2a)**

(*R*)-**2a** was prepared according to General Procedure 7 employing 0.38 mmol of *rac*-**1a** (100 mg), the reaction was stirred at 15 °C at 1200 rpm for 72 h. Following purification (FCC eluent: 100:0 – 98:2 pentane:DCM) the product was isolated as a white solid, 54 mg, 76 % yield, 92.5:7.5 e.r.

**$^1$H NMR** (400 MHz, $CDCl_3$) δ = 7.63 – 7.60 (m, 4H), 7.50 – 7.40 (m, 4H), 7.40 – 7.31 (m, 1H), 5.70 (dq, *J* = 47.7, 6.4 Hz, 1H), 1.71 (dd, *J* = 23.8, 6.4 Hz, 3H). **$^{19}$F NMR** (376 MHz, $CDCl_3$) δ = -166.6; **$^{13}$C NMR** (126 MHz, $CDCl_3$) δ = 141.4 (d, $J_{C-F}$ = 2.2 Hz), 140.8, 140.5 (d, $J_{C-F}$ = 20.1 Hz), 129.0, 127.6, 127.4, 127.3, 125.9 (d, $J_{C-F}$ = 6.6 Hz), 90.93 (d, $J_{C-F}$ = 167.3 Hz), 23.0 (d, $J_{C-F}$ = 25.2 Hz); **HRMS** (EI) m/z calculated for $C_{14}H_{12}F$ $(M)^{+\cdot}$ 200.09958**,** found 200.10013; **IR** (liquid film) ν = 2955, 2928, 1487, 1376, 1260, 1219, 1076, 1009, 841, 763, 698 $cm^{-1}$; **mp** 72-73 ˚C; **$[\alpha]^D$ $_{25\ °C}$** = +17.7 ° (c = 0.5, $CHCl_3$); **HPLC separation:** DAICEL CHIRALPAK® OJ-3, Heptane: iPrOH = 99:1, 1 mL/min; $t_1$ = 12.3 (minor), $t_2$ = 13.7 min (major).

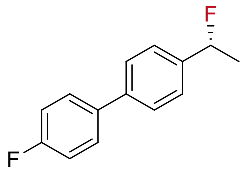


(*R*)-**2b**

**(*R*)-4-fluoro-4'-(1-fluoroethyl)-1,1'-biphenyl (2b)**

(*R*)-**2b** was prepared according to General Procedure 7 employing 0.38 mmol of *rac*-**1b** (106 mg), the reaction was stirred at 25 °C at 1200 rpm for 72 h. Following purification (FCC eluent: 100% pentane) the product was isolated as an off-white solid, 75 mg, 89 % yield, 92:8 e.r.

**$^1$H NMR** (400 MHz, $CDCl_3$) δ = 7.58 – 7.50 (m, 4H), 7.46 – 7.39 (m, 2H), 7.18 – 7.08 (m, 2H), 5.68 (dq, *J* = 47.6, 6.4 Hz, 1H), 1.68 (dd, *J* = 23.8, 6.4 Hz, 3H); **$^{19}$F NMR** (377 MHz, $CDCl_3$) δ = -115.5, -166.8; **$^{13}$C NMR** (126 MHz, $CDCl_3$) δ = 162.7 (d, $J_{C-F}$ = 246.6 Hz), 140.6 (d, $J_{C-F}$ = 19.7 Hz), 140.3 (d, $J_{C-F}$ = 2.2 Hz), 136.9 (d, $J_{C-F}$ = 3.3 Hz), 128.8 (d, $J_{C-F}$ = 7.9 Hz), 127.2, 125.9 (d, $J_{C-F}$ = 6.7 Hz), 115.8 (d, $J_{C-F}$ = 21.5 Hz), 90.9 (d, $J_{C-F}$ = 167.5 Hz), 23.0 (d, $J_{C-F}$ = 25.3 Hz); **HRMS** (EI) m/z calculated for $C_{14}H_{12}F_2$ $(M)^{+\cdot}$ 218.09016, found 218.09071; **IR** (liquid film) ν = 3021, 2981, 2892, 1616, 1605, 1586, 1216, 1069, 825, 754, 669 $cm^{-1}$; **mp** 56-58 ˚C; **$[\alpha]^D$ $_{25\ °C}$** = +14.1 ° (c = 1.0, $CHCl_3$); **HPLC separation:** DAICEL CHIRALPAK® OJ-3, Heptane: iPrOH = 99.5:0.5, 1 mL/min; $t_1$ = 25.9 (major), $t_2$ = 30.4 min (minor).

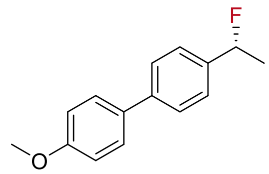


(*R*)-**2c**

**(*R*)-4-(1-fluoroethyl)-4'-methoxy-1,1'-biphenyl (2c)**

(*R*)-**2c** was prepared according to General Procedure 7 employing 0.3 mmol of *rac*-**1c** (87 mg), the reaction was stirred at 25 °C at 1200 rpm for 72h. Following purification (FCC eluent: 100:0 – 95:5 pentane:EtOAc) the product was isolated as a white solid, 44 mg, 64 % yield, 92:8 e.r.

**$^{1}$H NMR** (600 MHz, $CDCl_3$) δ = 8.14 – 8.09 (m, 2H), 7.69 – 7.61 (m, 4H), 7.48 – 7.43 (m, 2H), 5.69 (dq, *J* = 47.6, 6.5 Hz, 1H), 3.94 (s, 3H), 1.69 (dd, *J* = 23.9, 6.5 Hz, 3H); **$^{19}$F NMR** (565 MHz, $CDCl_3$) δ = -167.7; **$^{13}$C NMR** (151 MHz, $CDCl_3$) δ = 145.2, 141.6 (d, $J_{C-F}$ = 19.8 Hz), 140.1 (d, $J_{C-F}$ = 2.2 Hz), 130.3, 129.2, 127.5, 127.2, 125.9 (d, $J_{C-F}$ = 6.9 Hz), 90.8 (d, $J_{C-F}$ = 167.8 Hz), 52.3, 23.1 (d, $J_{C-F}$ = 25.2 Hz); **IR** (liquid film) ν = 3018, 2931, 1721, 1609, 1437, 1399, 1283, 1218, 1184, 1114, 1070, 1007, 867, 833, 700 $cm^{-1}$; **mp** 95-96 ˚C; **$[\alpha]^{D}_{25\,°C}$** = +21.7 ° (c = 0.4, $CHCl_3$); **HPLC separation:** DAICEL CHIRALPAK® OJ-3 Heptane: iPOH = 97:3, 1 mL/min; $t_1$ = 21.5 (minor), $t_2$ = 26.1 min (major). No HRMS obtained.

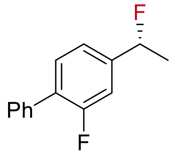
(*R*)-**2d**

**(*R*)-2-fluoro-4-(1-fluoroethyl)-1,1'-biphenyl (2d)**

(*R*)-**2d** was prepared according to General Procedure 7 employing 0.38 mmol of *rac*-**1d** (106 mg), the reaction was stirred at 25 °C at 1200 rpm for 72 hours. Following purification (FCC eluent: 100% pentane) the product was isolated as a colourless oil, 44 mg, 53 % yield, 92:8 e.r.

**$^{1}$H NMR** (600 MHz, $CDCl_3$) δ = 7.55 (dt, *J* = 7.9, 1.5 Hz, 2H), 7.45 (td, *J* = 7.8, 1.7 Hz, 3H), 7.41 – 7.35 (m, 1H), 7.18 (t, *J* = 10.5 Hz, 2H), 5.66 (dq, *J* = 47.5, 6.4 Hz, 1H), 1.68 (dd, *J* = 23.9, 6.5 Hz, 3H); **$^{19}$F NMR** (565 MHz, $CDCl_3$, 298 K) δ = -177.5, -168.3; **$^{13}$C NMR** (151 MHz, $CDCl_3$) δ = 159.8 (d, $J_{C-F}$ = 248.4 Hz), 143.1 (dd, $J_{C-F}$ = 20.2, 7.4 Hz), 135.5, 131.0 (d, $J_{C-F}$ = 3.5 Hz), 129.1 (d, $J_{C-F}$ = 2.9 Hz), 128.6, 127.9, 121.2 (dd, $J_{C-F}$ = 6.7, 3.4 Hz), 113.2 (d, $J_{C-F}$ = 7.4 Hz), 113.1 (d, $J_{C-F}$ = 7.4 Hz), 90.1 (d, $J_{C-F}$ = 169.2 Hz), 23.0 (d, $J_{C-F}$ = 24.9 Hz); **HRMS** (EI) m/z calculated for $C_{14}H_{12}F_2$ $(M)^{+\cdot}$ 218.09016, found 218.09071; **IR** (liquid film) ν = 3813, 3421, 2921, 1651, 1486, 1419, 1273, 1217, 1072, 1011, 837, 833, 759, 644 $cm^{-1}$; **$[\alpha]^{D}_{25\,°C}$** = +6.1° (c = 1.2, $CHCl_3$); **HPLC separation:** DAICEL CHIRALPAK® IB-3, Heptane 100%, 0.5 mL/min; $t_1$ = 14.3 (major), $t_2$ = 17.5 min (minor).

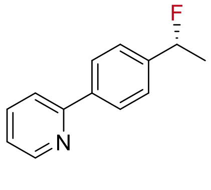
(*R*)-**2e**

**(*R*)-2-(4-(1-fluoroethyl)phenylpyridine (2e)**

(*R*)-**2e** was prepared according to General Procedure 7 employing 0.29 mmol of *rac*-**1e** (75 mg), the reaction was stirred at 25 °C at 1200 rpm for 72 h. Following purification (FCC eluent: 1st column: 90:10 pentane:EtOAc, 2nd column: 100% DCM) the product was isolated as a colourless oil, 40 mg, 70 % yield, 89:11 e.r.

**$^{1}$H NMR** (500 MHz, $CDCl_3$) δ = 8.73 (ddd, *J* = 4.8, 1.8, 1.0 Hz, 1H), 8.04 – 8.01 (m, 2H), 7.81 – 7.73 (m, 2H), 7.51 – 7.46 (m, 2H) 7.26 – 7.22 (m, 1H), 5.72 (dq, *J* = 47.7, 6.5 Hz, 1H), 1.70 (dd, *J* = 23.9, 6.5 Hz, 3H); **$^{19}$F NMR** (377 MHz, $CDCl_3$, 298 K) δ = -168.1; **$^{13}$C NMR** (126 MHz, $CDCl_3$) δ = 157.1, 149.8, 142.4 (d, $J_{C-F}$ = 19.5 Hz), 139.3, 137.1, 127.2, 125.7 (d, $J_{C-F}$ = 6.8 Hz), 122.4, 120.7, 90.9 (d, $J_{C-F}$ = 168.0 Hz), 23.1 (d, $J_{C-F}$ = 25.3 Hz); **HRMS** ($ESI^+$) m/z calculated for $C_{13}H_{14}FN$ $(M+H)^+$ 201.1027, found 202.1025; **IR** (liquid film) ν = 3769, 3743, 2980, 2929, 1590, 1469, 1437, 1375, 1260, 1155, 1018, 951, 785 $cm^{-1}$; **$[\alpha]^{D}_{25\,°C}$** =

+16.7 ° (c = 0.15, $CHCl_3$); **HPLC separation:** DAICEL CHIRALPAK® OJ-3, Heptane: iPrOH = 90:10, 1 mL/min; $t_1$ = 12.2 (minor), $t_2$ = 19.4 min (major).

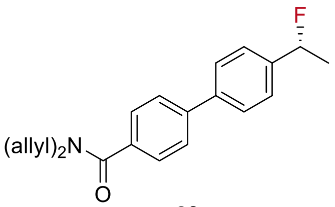

(*R*)-**2f**

**(*R*)-*N,N*-diallyl-4'-(1-fluoroethyl)-[1,1'-biphenyl]-4-carboxamide (2f)**

(*R*)-**2f** was prepared according to General Procedure 7 employing 0.25 mmol of *rac*-**1f** (96 mg), the reaction was stirred at 25 °C at 1200 rpm for 72 h. Following purification (FCC eluent: 100:0 – 95:5 DCM:EtOAc) the product was isolated as a yellow oil, 61 mg, 63 % yield, 92:8 e.r.

**$^1$H NMR** (400 MHz, $CDCl_3$) δ = 7.66 – 7.57 (m, 4H), 7.55 – 7.49 (m, 2H), 7.44 (dd, *J* = 8.1, 1.3 Hz, 2H), 5.89 (br s, 1H), 5.78 (br s, 1H), 5.68 (dq, *J* = 47.8, 6.4 Hz, 1H), 5.30 – 5.18 (m, 4H), 4.16 (s, 2H), 3.90 (s, 2H), 1.68 (dd, *J* = 23.8, 6.4 Hz, 3H); **$^{19}$F NMR** (377 MHz, $CDCl_3$) δ = -168.1; **$^{13}$C NMR** (101 MHz, $CDCl_3$) δ = 171.7, 142.1, 141.1 (d, $J_{C-F}$ = 19.6 Hz), 140.4 (d, $J_{C-F}$ = 2.1 Hz), 135.3, 133.4 (br), 133.0 (br), 127.4, 127.2, 125.9 (d, $J_{C-F}$ = 6.6 Hz), 117.8, 90.8 (d, $J_{C-F}$ = 167.8 Hz), 50.9 (br), 47.2 (br), 23.1 (d, $J_{C-F}$ = 25.2 Hz); **HRMS** ($ESI^+$) m/z calculated for $C_{21}H_{23}FNO$ $(M+H)^+$ 324.1758, found 324.1748; **IR** (liquid film) ν = 3078, 2925, 2857, 1636, 1457, 1414, 1288, 1261, 1217, 1112, 1069, 1007, 927, 885, 830, 769, 647 $cm^{-1}$; **$[\alpha]^D_{25\,°C}$** = +13.3 ° (c = 0.4, $CHCl_3$); **HPLC separation:** DAICEL CHIRALPAK® IC-3, Heptane: iPOH = 90:10, 1mL/min; $t_1$ = 38.5 (minor), $t_2$ = 42.8 min (major).

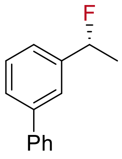

(*R*)-**2g**

**(*R*)-3-(1-fluoroethyl)-1,1'-biphenyl (2g)**

(*R*)-**2g** was prepared according to General Procedure 7 employing 0.38 mmol of *rac*-**1g** (50 mg), the reaction was stirred at 25 °C at 1200 rpm for 72 h. Following purification (FCC eluent: 100% pentane) the product was isolated as a colourless oil, 61 mg, 80 % yield, 90.5:9.5 e.r.

**$^1$H NMR** (400 MHz, $CDCl_3$) δ = 7.67 – 7.51 (m, 4H), 7.51 – 7.42 (m, 3H), 7.42 – 7.31 (m, 2H), 5.71(dq, *J* = 47.7, 6.4 Hz, 1H), 1.70 (dd, *J* = 23.9, 6.4 Hz, 3H); **$^{19}$F NMR** (377 MHz, $CDCl_3$, 298 K) δ = -167.3; **$^{13}$C NMR** (101 MHz, $CDCl_3$) δ = 142.2 (d, $J_{C-F}$ = 19.6 Hz), 141.7, 141.1, 129.1, 128.9, 127.6, 127.4, 127.2 (d, $J_{C-F}$ = 1.9 Hz), 124.2 (d, $J_{C-F}$ = 6.1 Hz), 124.2 (d, $J_{C-F}$ = 6.9 Hz), 91.1 (d, $J_{C-F}$ = 168.0 Hz), 23.2 (d, $J_{C-F}$ = 25.3 Hz); **HRMS** (EI) m/z calculated for $C_{14}H_{13}F$ $(M)^{+\cdot}$ 200.09958, found 200.10013; **IR** (liquid film) ν = 3020, 2982, 1600, 1482, 1376, 1216, 1068, 896, 703, 668 $cm^{-1}$; **$[\alpha]^D_{25\,°C}$** = +14.6 ° (c = 0.25, $CHCl_3$); **HPLC separation:** DAICEL CHIRALPAK® OJ-3, Heptane: iPrOH = 99.5:0.5, 1 mL/min; $t_1$ = 12.2 (minor), 13.2 min (major).

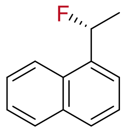

(*R*)-**2h**

**(*R*)-1-(1-fluoroethyl)naphthalene (2h)**

(*R*)-**2h** was prepared according to General Procedure 7 employing 0.38 mmol of *rac*-**1h** (89 mg), the reaction was stirred at 25 °C at 1200 rpm for 24 h. Following purification (FCC eluent: 100% pentane) the product was isolated as a colourless oil, 63 mg, 95 % yield, 95.5:4.5 e.r.

**$^1$H NMR** (600 MHz, $CDCl_3$) δ = 8.04 – 7.99 (m, 1H), 7.91 – 7.85 (m, 1H), 7.83 (dd, *J* = 8.2, 1.2 Hz, 1H), 7.61 (dd, *J* = 7.1, 1.0 Hz, 1H), 7.57 – 7.47 (m, 3H), 6.36 (dq, *J* = 46.7, 6.4 Hz, 1H), 1.84 (dd, *J* = 23.8, 6.5 Hz, 3H); **$^{19}$F NMR** (565 MHz, $CDCl_3$, 298 K ) δ = -169.8; **$^{13}$C NMR** (151 MHz, $CDCl_3$) δ = 137.1 (d, $J_{C\text{-}F}$ = 18.1 Hz), 133.9, 129.8 (d, $J_{C\text{-}F}$ = 160.7 Hz), 128.9, 128.9, 126.5, 125.9, 125.5, 123.3, 122.7 (d, $J_{C\text{-}F}$ = 10.2 Hz), 89.0 (d, $J_{C\text{-}F}$ = 167.4 Hz), 22.6 (d, $J_{C\text{-}F}$ = 25.2 Hz); **HRMS** (EI) m/z calculated for $C_{12}H_{11}F$ $(M)^{+\cdot}$ 174.08393, found 174.08448; **IR** (liquid film) ν = 2924, 2850, 1599, 1510, 1146, 1054, 851, 801, 645, 638 $cm^{-1}$; **$[\alpha]^D$ $_{25\,°C}$** = +17.6 ° (c = 0.3, $CHCl_3$); **HPLC separation:** DAICEL CHIRALPAK® OJ-3, Heptane: iPrOH = 99:1, 1 mL/min; $t_1$ = 6.2 (major), $t_2$ = 7.0 min (minor).

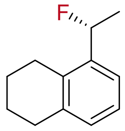

(*R*)-**2i**

**5-(1-fluoroethyl)-1,2,3,4-tetrahydronaphthalene (2i)**

(*R*)-**2i** was prepared according to General Procedure 7 employing 0.25 mmol of *rac*-**1i** (60 mg), the reaction was stirred at 25 °C at 1200 rpm for 72 h. Following purification (FCC eluent: 100:0 – 95:5 pentane:DCM) the product was isolated as a white solid, 38 mg, 87% yield, 95:5 e.r.

**$^1$H NMR** (400 MHz, $CDCl_3$) δ = 7.39 – 7.27 (m, 1H), 7.17 (t, *J* = 7.6 Hz, 1H), 7.10 – 7.03 (m, 1H), 5.85 (dq, *J* = 46.9, 6.4 Hz, 1H), 2.82 (dq, *J* = 11.8, 5.4 Hz, 3H), 2.62 (dt, *J* = 16.8, 6.4 Hz, 1H), 1.90 – 1.71 (m, 4H), 1.62 (dd, *J* = 23.9, 6.4 Hz, 3H); **$^{19}$F NMR** (376 MHz, $CDCl_3$) δ = -169.3; **$^{13}$C NMR** (101 MHz, $CDCl_3$) δ = 139.6 (d, $J_{C\text{-}F}$ = 18.2 Hz), 137.6, 133.5 (d, $J_{C\text{-}F}$ = 4.8 Hz), 129.4 (d, $J_{C\text{-}F}$ = 2.2 Hz), 125.7, 122.2 (d, $J_{C\text{-}F}$ = 9.1 Hz), 87.9 (d, $J_{C\text{-}F}$ = 165.7 Hz), 30.2, 25.7, 23.3, 22.8, 22.1 (d, $J_{C\text{-}F}$ = 26.0 Hz); **HRMS** (EI) m/z calculated for $C_{12}H_{15}F$ $(M)^{+\cdot}$ 178.11523 found 178.11578; **IR** (liquid film) ν = 2972, 2930, 2887, 1469, 1380, 1310, 1252, 1162, 953, 818 $cm^{-1}$; **mp** 61-62 ˚C; **$[\alpha]^D$ $_{25\,°C}$** = +16.3 ° (c = 0.3, $CHCl_3$); **HPLC separation:** DAICEL CHIRALPAK® IB-3, Heptane 100%, 1 mL/min; $t_1$ = 4.5 (minor), $t_2$ = 4.8 min (major).

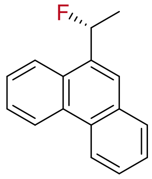

(*R*)-**2j**

**(*R*)-9-(1-fluoroethyl)phenanthrene (2j)**

(*R*)-**2j** was prepared according to General Procedure 7 employing 0.25 mmol of *rac*-**1j** (71 mg), the reaction was stirred at 25 °C at 1200 rpm for 72 h. Following purification (FCC eluent: 100:0 – 95:5 pentane:DCM) the product was isolated as a white solid, 49 mg, 88 % yield, 96:4 e.r.

$^{1}$**H NMR** (400 MHz, $CDCl_3$) δ = 8.82 – 8.72 (m, 1H), 8.71 – 8.62 (m, 1H), 8.11 – 8.02 (m, 1H), 7.95 – 7.91 (m, 1H), 7.89 (s, 1H), 7.75 – 7.58 (m, 4H), 6.39 (dq, *J* = 46.6, 0.9 Hz, 1H), 1.92 (dd, *J* = 23.8, 6.4 Hz, 3H); $^{19}$**F NMR** (376 MHz, $CDCl_3$) δ = -170.5; $^{13}$**C NMR** (101 MHz, $CDCl_3$) δ = 135.3 (d, $J_{C\text{-}F}$ = 17.7 Hz), 131.3, 130.8, 130.5 (d, $J_{C\text{-}F}$ = 1.3 Hz), 129.2 (d, $J_{C\text{-}F}$ = 3.0 Hz), 129.1, 127.2, 127.0, 126.9, 126.6, 124.0 (d, $J_{C\text{-}F}$ = 1.7 Hz), 123.7 (d, $J_{C\text{-}F}$ = 10.9 Hz), 123.5, 122.6, 89.1 (d, $J_{C\text{-}F}$ = 168.1 Hz), 22.3 (d, $J_{C\text{-}F}$ = 25.0 Hz); **IR** (liquid film) ν = 3021, 2981, 2892, 1616, 1609, 1584, 1500, 1216, 1070, 826, 854 $cm^{-1}$; **mp** 63-64 ˚C; **$[\alpha]^{D}_{25\,°C}$** = +21.2 ° (c = 0.5, $CHCl_3$); **HPLC separation:** DAICEL CHIRALPAK® IF-3, Heptane: iPrOH = 99.5:0.5, 1 mL/min; $t_1$ = 5.8 (minor), $t_2$ = 6.6 min (major). No HRMS obtained.

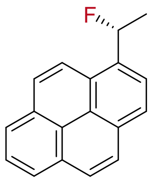

(*R*)-**2k**

**1-(1-fluoroethyl)pyrene (2k)**

(*R*)-**2k** was prepared according to General Procedure 7 employing 0.25 mmol of *rac*-**1k** (77 mg), the reaction was stirred at 25 °C at 1200 rpm for 72 h. Following purification – plugged over short silica column (FCC eluent: 100:0 – 90:10 pentane:DCM) the product was isolated as a viscous pale-yellow oil, 37 mg, 60 % yield, 96:4 e.r.

$^{1}$**H NMR** (500 MHz, $CDCl_3$) δ = 8.26 (d, *J* = 9.3 Hz, 1H), 8.22 (dd, *J* = 7.8, 5.0 Hz, 2H), 8.16 (s, 1H), 8.08 (m, 2H), 8.06 – 8.00 (m, 2H), 6.68 (dq, *J* = 46.8, 6.6 Hz, 1H), 1.95 (dd, *J* = 23.7, 6.5 Hz, 3H); $^{19}$**F NMR** (470 MHz, $CDCl_3$) δ = -168.4; $^{13}$**C NMR** (126 MHz, $CDCl_3$) δ = 139.1, 134.8, 134.7, 131.5, 130.7, 128.1, 127.7 (d, $J_{C\text{-}F}$ = 23.4 Hz), 127.6 (d, $J_{C\text{-}F}$ = 11.2 Hz), 127.1 (d, $J_{C\text{-}F}$ = 8.4 Hz), 126.2, 126.0, 125.6 (d, $J_{C\text{-}F}$ = 26.1 Hz), 125.1, 125.0, 122.7 (d, $J_{C\text{-}F}$ = 9.5 Hz), 122.4, 89.2 (d, $J_{C\text{-}F}$ = 167.6 Hz), 23.3 (d, $J_{C\text{-}F}$ = 25.5 Hz); **IR** (liquid film) ν = 2941, 2934, 2262, 1558, 1476, 1372, 1243, 1291, 1166, 1021, 991, 889, 867, 620 $cm^{-1}$; **$[\alpha]^{D}_{25\,°C}$** = +22.6 ° (c = 0.3, $CHCl_3$); **HPLC separation:** DAICEL CHIRALPAK® IB-3, Heptane 100%, 0.5 mL/min; $t_1$ = 14.2 (minor), $t_2$ = 16.0 min (major). No HRMS obtained.

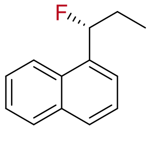

(*R*)-**2l**

**(*R*)-1-(1-fluoropropyl)naphthalene (2l)**

(*R*)-**2l** was prepared according to General Procedure 7 employing 0.3 mmol of *rac*-**1l** (75 mg), the reaction was stirred at 25 °C for at 1200 rpm 48 hours. Following purification (FCC eluent: 100% Pentane) the product was isolated as a colourless oil, 53 mg, 94 % yield, 93:7 e.r.

$^{1}$**H NMR** (400 MHz, $CDCl_3$) δ = 8.04 – 7.95 (m, 1H), 7.94 – 7.84 (m, 1H), 7.65 – 7.57 (m, 1H), 7.57 – 7.42 (m, 3H), 6.10 (ddd, 46.8, 7.2, 5.3 Hz 1H), 2.25 – 2.04 (m, 2H), 1.10 (t, *J* = 7.4 Hz, 3H); $^{19}$**F NMR** (377 MHz, $CDCl_3$, 298 K) δ = -179.4; $^{13}$**C NMR** (101 MHz, $CDCl_3$) δ = 136.1 (d, $J_{C\text{-}F}$ = 18.4 Hz), 133.9, 130.2 (d, $J_{C\text{-}F}$ = 3.7 Hz), 129.1, 128.8 (d, $J_{C\text{-}F}$ = 1.9 Hz), 126.3, 125.8, 125.4, 123.3 (d, $J_{C\text{-}F}$ = 10.5 Hz), 123.3 (d, $J_{C\text{-}F}$ = 1.3 Hz), 93.9 (d, $J_{C\text{-}F}$ = 171.1 Hz), 29.8 (d, $J_{C\text{-}F}$ = 23.9 Hz), 10.0 (d, $J_{C\text{-}F}$ = 4.7 Hz); **HRMS** (EI) m/z calculated for $C_{13}H_{13}F$ $(M)^{+\cdot}$ 188.09958, found 188.10013; **IR** (liquid film) ν = 3019, 2976, 1599, 1513, 1463, 1217,

1095, 1044, 958, 912, 762 $cm^{-1}$; **$[\alpha]^{D}_{25\,°C}$** = +10.8 ° (c = 1.0, $CHCl_3$); **HPLC separation:** DAICEL CHIRALPAK® OJ-3, Heptane: iPrOH = 99:1, 0.5 mL/min; $t_1$ = 9.1 (major), 9.9 min (minor).

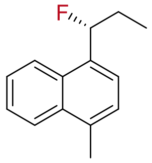

(*R*)-**2m**

**(*R*)-1-(1-fluoropropyl)-4-methylnaphthalene (2m)**

(*R*)-**2m** was prepared according to General Procedure 7 employing 0.25 mmol of *rac*-**1m** (66 mg), the reaction was stirred at 25 °C for at 1200 rpm 48 hours. Following purification (FCC eluent: 100% Hexane) the product was isolated as a colourless oil, 31 mg, 62 % yield, 92:8 e.r.

**$^1$H NMR** (400 MHz, $CDCl_3$) δ = 8.10 – 7.97 (m, 2H), 7.59 – 7.50 (m, 2H), 7.47 (d, *J* = 7.3 Hz, 1H), 7.34 (d, *J* = 7.3 Hz, 1H), 6.16 – 5.96 (m, 1H), 2.71 (t, *J* = 1.1 Hz, 3H), 2.22 – 2.02 (m, 2H), 1.09 (t, *J* = 7.4 Hz, 3H); **$^{19}$F NMR** (376 MHz, $CDCl_3$) δ = -178.1; **$^{13}$C NMR** (101 MHz, $CDCl_3$) δ = 135.0 (d, $J_{C-F}$ = 1.9 Hz), 134.2 (d, $J_{C-F}$ = 18.6 Hz), 133.0, 130.4 (d, $J_{C-F}$ = 3.6 Hz), 126.2, 126.0, 125.6, 125.1, 123.8, 123.2 (d, $J_{C-F}$ = 10.2 Hz), 94.0 (d, $J_{C-F}$ = 170.4 Hz), 29.8 (d, $J_{C-F}$ = 23.9 Hz), 19.8, 10.1 (d, $J_{C-F}$ = 4.7 Hz); **IR** (liquid film) ν = 3077, 2931, 2874, 1634, 1603, 1583, 1513, 1464, 1398, 1320, 1254, 1223, 1143, 1108, 1050, 916, 832, 763, 710, 644, 618 $cm^{-1}$; **$[\alpha]^{D}_{25\,°C}$** = +10.1 ° (c = 0.2, $CHCl_3$); **HPLC separation:** DAICEL CHIRALPAK® IF-3, Heptane 100%, 0.8 mL/min; $t_1$ = 9.5 (minor), 10.7 min (major). No HRMS obtained.

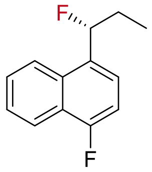

(*R*)-**2n**

**(*R*)-1-fluoro-4-(1-fluoropropyl)naphthalene (2n)**

(*R*)-**2n** was prepared according to General Procedure 7 employing 0.25 mmol of *rac*-**1n** (67 mg), the reaction was stirred at 25 °C for at 1200 rpm 48 hours. Following purification (FCC eluent: 100% Hexane) the product was isolated as a colourless oil, 38 mg, 74 % yield, 95:5 e.r.

**$^1$H NMR** (400 MHz, $CDCl_3$) δ = 8.22 – 8.10 (m, 1H), 8.06 – 7.97 (m, 1H), 7.64 – 7.54 (m, 2H), 7.53 – 7.47 (m, 1H), 7.15 (dd, *J* = 10.2, 8.0 Hz, 1H), 6.03 (ddd, *J* = 46.8, 7.4, 5.1 Hz, 1H), 2.31 – 1.95 (m, 2H), 1.08 (t, *J* = 7.4 Hz, 3H); **$^{19}$F NMR** (376 MHz, $CDCl_3$) δ = -122.7, -177.6; **$^{13}$C NMR** (101 MHz, $CDCl_3$) δ = 158.9 (d, $J_{C-F}$ = 254.6 Hz), 132.2 – 131.5 (m), 127.3, 126.2 (d, $J_{C-F}$ = 2.1 Hz), 124.0 (d, $J_{C-F}$ = 16.3 Hz), 123.6 (d, $J_{C-F}$ = 8.9 Hz), 123.5 (d, $J_{C-F}$ = 8.7 Hz), 123.4 – 123.3 (m), 121.5 (d, $J_{C-F}$ = 6.0 Hz), 108.8 (d, $J_{C-F}$ = 20.2 Hz), 93.7 (d, $J_{C-F}$ = 171.1 Hz), 29.7 (d, $J_{C-F}$ = 24.0 Hz), 10.0 (d, $J_{C-F}$ = 4.9 Hz); **IR** (liquid film) ν = 3073, 2965, 2929, 2874, 1722, 1634, 1603, 1583, 1513, 1464, 1427, 1395, 1321, 1253, 1223, 1145, 1108, 1050, 1008, 832, 764, 711, 637 $cm^{-1}$; **$[\alpha]^{D}_{25\,°C}$** = +15.4 ° (c = 0.3, $CHCl_3$); **HPLC separation:** DAICEL CHIRALPAK® IB-3, Heptane 100%, 0.8 mL/min; $t_1$ = 5.5 (minor), 9.4 min (major). No HRMS obtained.

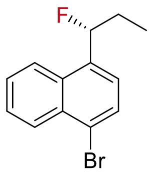

(*R*)-**2o**

**(*R*)- 1-bromo-4-(1-fluoropropyl)naphthalene (2o)**

(*R*)-**2o** was prepared according to General Procedure 7 employing 0.3 mmol of *rac*-**1o** (98 mg), the reaction was stirred at 25 °C at 1200 rpm for 72 h. Following purification (FCC eluent: 100% pentane) the product was isolated as a pale-yellow oil, 69 mg, 86 % yield, 96:4 e.r.

**$^1$H NMR** (400 MHz, $CDCl_3$) δ = 8.4 – 8.3 (m, 1H), 8.0 – 7.9 (m, 1H), 7.8 (d, *J* = 7.8 Hz, 1H), 7.7 – 7.5 (m, 2H), 7.4 (d, *J* = 7.8 Hz, 1H), 6.3 – 5.8 (m, 1H), 2.2 – 2.0 (m, 2H), 1.1 (t, *J* = 7.4 Hz, 3H); **$^{19}$F NMR** (377 MHz, $CDCl_3$, 298 K) δ = -180.2; **$^{13}$C NMR** (101 MHz, $CDCl_3$) δ = 136.2 (d, $J_{C-F}$ = 18.7 Hz), 132.1, 131.3 (d, $J_{C-F}$ = 3.9 Hz), 129.6, 128.3, 127.2, 127.2, 123.8 (d, $J_{C-F}$ = 11.1 Hz), 123.6 (d, $J_{C-F}$ = 1.3 Hz), 123.5 (d, $J_{C-F}$ = 2.3 Hz), 93.4 (d, $J_{C-F}$ = 172.1 Hz), 29.8 (d, $J_{C-F}$ = 23.8 Hz), 9.9 (d, $J_{C-F}$ = 4.6 Hz); **HRMS** (EI) m/z calculated for $C_{13}H_{12}BrF$ $(M)^{+\cdot}$ 266.01009, found 266.01064; **IR** (liquid film) ν = 3552, 3092, 3049, 2931, 1727, 1568, 1510, 1458, 1385, 1200, 1125, 961, 922, 835, 761, 641 $cm^{-1}$; **$[\alpha]^{D}_{25\,°C}$** = +27.2 ° (c = 0.35, $CHCl_3$); **HPLC separation:** DAICEL CHIRALPAK® OJ-3 Heptane 100%, 0.5 mL/min; $t_1$ = 10.8 (minor) $t_2$ = 11.7 min (major).

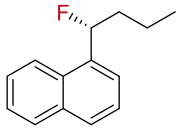

(*R*)-**2p**

**(*R*)-1-(1-fluorobutyl)naphthalene (2p)**

(*R*)-**2p** was prepared according to General Procedure 7 employing 0.3 mmol of *rac*-**1p** (79 mg), the reaction was stirred at 25 °C at 1200 rpm for 48 h. Following purification (FCC eluent: 100% pentane) the product was isolated as a yellow oil, 36 mg, 59 % yield, 88:12 e.r.

**$^1$H NMR** (400 MHz, $CDCl_3$) δ = 7.99 (d, *J* = 8.0 Hz, 1H), 7.92 – 7.86 (m, 1H), 7.85 – 7.80 (m, 1H), 7.58 (dd, *J* = 7.2, 1.3 Hz, 1H), 7.57 – 7.36 (m, 3H), 6.18 (ddd, *J* = 47.2, 8.3, 4.2 Hz, 1H), 2.22 – 1.92 (m, 2H), 1.72 – 1.46 (m, 2H), 1.07 – 0.94 (m, 3H); **$^{19}$F NMR** (377 MHz, $CDCl_3$) δ = -178.5; **$^{13}$C NMR** (101 MHz, $CDCl_3$) δ = 136.4 (d, *J* = 18.4 Hz), 133.9, 130.1 (d, *J* = 3.7 Hz), 129.1, 128.7 (d, *J* = 1.8 Hz), 126.4, 125.8, 125.4, 123.3 (d, *J* = 3.1 Hz), 123.2 (d, *J* = 6.6 Hz), 92.6 (d, *J* = 170.7 Hz), 39.1 (d, *J* = 24.2 Hz), 19.0 (d, *J* = 3.7 Hz), 14.0; **IR** (liquid film) ν = 2959, 2926, 2873, 2853, 2614, 2002, 1832, 1649, 1597, 1514, 1432, 1335, 1274, 1192, 1062, 1025, 1003, 944, 916, 864, 799, 756, 670, 620 $cm^{-1}$; **HRMS** (EI) m/z calculated for $C_{14}H_{15}F$ $(M)^{+\cdot}$ 202.11523, found 202.11578; **$[\alpha]^{D}_{25\,°C}$** = +9.9 ° (c = 0.9, $CHCl_3$); **HPLC separation:** DAICEL CHIRALPAK® IB-3 Heptane: iPOH 99:1, 1 mL/min; $t_1$ = 3.8 (minor), $t_2$ = 4.7 min (major).

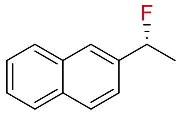

(*R*)-**2q**

**(*R*)- 2-(1-fluoroethyl)naphthalene (2q)**

(*R*)-**2q** was prepared according to General Procedure 7 employing 0.38 mmol of *rac*-**1q** (89 mg), the reaction was stirred at 15 °C at 1200 rpm for 72 h. Following

purification (FCC eluent: 100:0 – 97:3 pentane: DCM) the product was isolated as a white solid, 38 mg, 57 % yield, 89:11 e.r.

**$^1$H NMR** (400 MHz, $CDCl_3$) δ = 8.00 – 7.72 (m, 4H), 7.64 – 7.41 (m, 3H), 5.81 (dq, *J* = 47.6, 6.4 Hz, 1H), 1.74 (dd, *J* = 23.8, 6.4 Hz, 3H); **$^{19}$F NMR** (377 MHz, $CDCl_3$, 298 K) δ = -167.0; **$^{13}$C NMR** (126 MHz, $CDCl_3$) δ = 139.0 (d, *J* = 19.5 Hz), 133.3 (d, *J* = 1.6 Hz), 133.2, 128.5, 128.2, 127.9, 126.5, 126.3, 124.3 (d, *J* = 8.1 Hz), 123.3 (d, *J* = 5.6 Hz), 91.3 (d, *J* = 167.7 Hz), 23. 1 (d, *J* = 25.2 Hz); **HRMS** (EI) m/z calculated for $C_{12}H_{11}F$ $(M)^{+\cdot}$ 174.08393, found 174.08448; **IR** (neat) ν = 3036, 2961, 2952, 2918, 1573, 1543, 1522, 1451, 1420, 1377, 1100, 1027, 973, 943, 899, 692 $cm^{-1}$; **mp** 58-60 ˚C; **[α]$^D$$_{25\,°C}$** = +13.3 ° (c = 0.5, $CHCl_3$); **HPLC separation:** DAICEL CHIRALPAK® OJ-3, Heptane: iPrOH = 99:1, 1 mL/min; $t_1$ = 9.2 (minor), $t_2$ = 10.6 min (major).

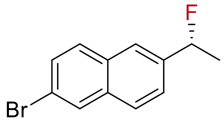

(*R*)-**2r**

**(*R*)-2-bromo-6-(1-fluoroethyl)naphthalene (2r)**

(*R*)-**2r** was prepared according to General Procedure 7 employing 0.38 mmol of *rac*-**1r** (119 mg), the reaction was stirred at 25 °C at 1200 rpm for 72 h. Following purification (FCC eluent: 100% pentane) the product was isolated as a white solid, 82 mg, 85 % yield, 92:8 e.r.

**$^1$H NMR** (400 MHz, $CDCl_3$) δ = 8.01 (d, *J* = 2.0 Hz, 1H), 7.84 – 7.74 (m, 2H), 7.72 (d, *J* = 8.7 Hz, 1H), 7.57 (dd, *J* = 8.7, 2.0 Hz, 1H), 7.49 (dd, *J* = 8.6, 1.6 Hz, 1H), 5.78 (dq, *J* = 47.5, 6.4 Hz, 1H), 1.73 (dd, *J* = 23.8, 6.4 Hz, 3H); **$^{19}$F NMR** (377 MHz, $CDCl_3$, 298 K) δ = -167.8; **$^{13}$C NMR** (101 MHz, $CDCl_3$) δ = 139.5 (d, $J_{C\text{-}F}$ = 19.5 Hz), 134.3, 134.3, 131.7, 129.9, 129.9, 127.6, 124.3 (d, $J_{C\text{-}F}$ = 5.8 Hz), 124.2 (d, $J_{C\text{-}F}$ = 8.0 Hz), 120.3, 91.0 (d, $J_{C\text{-}F}$ = 168.4 Hz), 23.0 (d, $J_{C\text{-}F}$ = 25.1 Hz); **IR** (neat) ν = 2998, 2982, 2955, 2918, 2849, 1631, 1589, 1499, 1463, 1391, 1366, 1315, 1257, 1220, 1169, 1135, 1061, 1021, 956, 898, 808, 773, 728, 615 $cm^{-1}$; **mp** 69-71 ˚C; **[α]$^D$$_{25\,°C}$** = +21.6 ° (c = 0.3, $CHCl_3$); **HPLC separation:** DAICEL CHIRALPAK® OJ-3, Heptane: iPrOH = 99:1, 1 mL/min; $t_1$ = 9.0 (minor), $t_2$ = 10.9 min (major). No HRMS obtained.

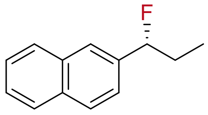

(*R*)-**2s**

**(*R*)-1-(2-fluoropropyl)naphthalene (2s)**

(*R*)-**2s** was prepared according to General Procedure 7 employing 0.38 mmol of *rac*-**1s** (94 mg), the reaction was stirred at 25 °C at 1200 rpm for 72 h. Following purification (FCC eluent: 100% pentane) the product was isolated as a colourless oil, 52 mg, 73 % yield, 94:6 e.r.

**$^1$H NMR** (400 MHz, $CDCl_3$) δ = 7.90 – 7.80 (m, 3H), 7.78 (br s, 1H), 7.53 – 7.48 (m, 2H), 7.45 (dd, *J* = 8.5, 1.8 Hz, 1H), 5.54 (ddd, *J* = 47.6, 7.5, 5.4 Hz, 1H), 2.18 – 1.87 (m, 2H), 1.02 (t, *J* = 7.4 Hz, 3H); **$^{19}$F NMR** (377 MHz, $CDCl_3$, 298 K ) δ = -175.3; **$^{13}$C NMR** (126 MHz, $CDCl_3$) δ 137.7 (d, $J_{C\text{-}F}$ = 19.8 Hz), 133.2, 133.1, 128.3, 128.1, 127.7, 126.3, 126.2, 124.8 (d, $J_{C\text{-}F}$ = 8.2 Hz), 123.5 (d, $J_{C\text{-}F}$ = 5.8 Hz), 96.0 (d, $J_{C\text{-}F}$ = 170.8 Hz),

30.1 (d, $J_{C\text{-}F}$ = 24.1 Hz), 9.4 (d, $J_{C\text{-}F}$ = 5.8 Hz); **HRMS** (EI) m/z calculated for $C_{13}H_{13}F$ $(M)^{+\cdot}$ 188.09958, found 188.10013; **IR** (liquid film) ν = 3061, 2971, 2922, 1464, 1380, 1177, 966, 918, 819, 632 $cm^{-1}$; **$[\alpha]^{D}_{25\,°C}$** = +15.7 ° (c = 1.0, $CHCl_3$); **HPLC separation:** DAICEL CHIRALPAK® OJ-3, Heptane: iPrOH = 99.5:0.5, 1 mL/min; $t_1$ = 10.6 (minor), $t_2$ = 12.2 min (major).

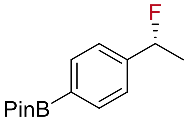

(*R*)-**2t**

**(*R*)-2-(4-(1-fluoroethyl)phenyl)-4,4,5,5-tetramethyl-1,3-dioxolane (2t)**

(*R*)-**2t** was prepared according to General Procedure 7 employing 0.24 mmol of *rac*-**1t** (75 mg), the reaction was stirred at 25 °C at 1200 rpm for 72 h. Following purification (FCC eluent: 100:0 – 97:3 pentane:EtOAc) the product was isolated as a white solid, 43 mg, 71 % yield, 91.5:8.5 e.r.

**$^{1}$H NMR** (500 MHz, $CDCl_3$) δ = 7.83 (d, *J* = 7.6 Hz, 2H), 7.35 (d, *J* = 7.6 Hz, 2H), 5.64 (dq, *J* = 47.7, 6.4 Hz, 1H), 1.63 (dd, *J* = 24.0, 6.4 Hz, 3H), 1.35 (s, 12H); **$^{19}$F NMR** (471 MHz, $CDCl_3$) δ = -169.6; **$^{13}$C NMR** (126 MHz, $CDCl_3$) δ = 144.7 (d, *J* = 19.5 Hz), 135.1, 124.5, 124.4, 91.0 (d, *J* = 168.2 Hz), 84.0, 25.0, 23.2 (d, *J* = 25.0 Hz); **HRMS** (EI) m/z calculated for $C_{14}H_{20}BFO_2$ $(M)^{+\cdot}$ 249.15712, found 249.15767; **IR** (liquid film) ν = 2983, 1733, 1616, 1519, 1401, 1364, 1324 1273, 1215, 1146, 1092, 1071, 1022, 963, 887, 860 $cm^{-1}$; **mp** 52-54 °C; **$[\alpha]^{D}_{25\,°C}$** = +14.4 ° (c = 0.15, $CHCl_3$); **HPLC separation:** DAICEL CHIRALPAK® IB-3, Heptane: iPOH 99.8:0.2, 0.8 mL/min; $t_1$ = 3.7 (major), $t_2$ = 4.1 min (minor).

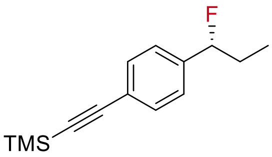

(*R*)-**2u**

**(*R*)-((4-(1-fluoropropyl)phenyl)ethynyl)trimethylsilane (2u)**

(*R*)-**2u** was prepared according to General Procedure 7 employing 0.3 mmol of *rac*-**1u** (89 mg), the reaction was stirred at 40 °C at 1200 rpm for 72 h. Following purification (FCC eluent: 100% pentane) the product was isolated as a colourless oil, 48 mg, 68 % yield, 92:8 e.r.

**$^{1}$H NMR** (500 MHz, $CDCl_3$) δ = 7.50 – 7.43 (m, 2H), 7.26 – 7.22 (m, 2H), 5.35 (ddd, *J* = 47.6, 7.5, 5.2 Hz, 1H), 2.02 – 1.78 (m, 2H), 0.96 (t, *J* = 7.4 Hz, 3H), 0.25 (s, 9H); **$^{19}$F NMR** (377 MHz, $CDCl_3$) δ = -177.4; **$^{13}$C NMR** (126 MHz, $CDCl_3$) δ = 140.7 (d, *J* = 19.2 Hz), 132.1, 125.5 (d, *J* = 7.0 Hz), 123.0, 104.8, 95.4 (d, *J* = 171.6 Hz), 94.7, 30.2 (d, *J* = 24.0 Hz), 9.3, 0.1; **IR** (liquid film) ν = 3735, 2959, 2929, 2856, 2161, 1731, 1511, 1502, 1464, 1412, 1379, 1362, 1306, 1251, 1222, 1208, 1088, 1078, 1046, 1019, 1002, 963, 867, 845, 762, 701, 646, 629 $cm^{-1}$; **$[\alpha]^{D}_{25\,°C}$** = +10.1 ° (c = 0.2, $CHCl_3$); **HPLC separation:** DAICEL CHIRALPAK® OJ-3 Heptane: iPOH 99:1, 0.5 mL/min; $t_1$ = 5.2 (minor), $t_2$ = 5.6 min (minor). No HRMS obtained.

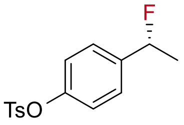

(*R*)-**2v**

**(*R*)-4-(1-fluoroethyl)phenyl-4-methylbenzenesulfonate (2v)**

(*R*)-**2v** was prepared according to General Procedure 7 employing 0.3 mmol of *rac*-**1v** (89 mg) and the reaction was stirred at 40 °C at 1200 rpm for 72 h. Following

purification (FCC eluent: 100:0 – 95-5 pentane:EtOAc) the product was isolated as a colourless oil, 47 mg, 64 % yield, 81:19 e.r.

**1H NMR** (500 MHz, $CDCl_3$) δ = 7.75 – 7.67 (m, 2H), 7.32 (d, *J* = 8.0 Hz, 2H), 7.29 – 7.25 (m, 2H), 7.02 – 6.95 (m, 2H), 5.59 (dq, *J* = 47.4, 6.4 Hz, 1H), 2.45 (s, 3H), 1.60 (dd, *J* = 23.9, 6.4 Hz, 3H); **$^{19}$F NMR** (377 MHz, $CDCl_3$, 298 K) δ = -167.5; **$^{13}$C NMR** (101 MHz, $CDCl_3$) δ = 149.4 (d, $J_{C\text{-}F}$ = 2.2 Hz), 145.6, 140.5 (d, $J_{C\text{-}F}$ = 20.0 Hz), 132.5, 129.9, 128.6 (d, $J_{C\text{-}F}$ = 1.9 Hz), 126.6 (d, $J_{C\text{-}F}$ = 6.8 Hz), 122.6, 90.3 (d, $J_{C\text{-}F}$ = 168.6 Hz), 23.0 (d, $J_{C\text{-}F}$ = 25.0 Hz), 21.8; **HRMS** (EI) m/z calculated for $C_{15}H_{15}FO_3S$ $(M)^{+\cdot}$ 294.07204, found 294.07259; **IR** (liquid film) ν = 2928, 1599, 1505, 1377, 1295, 1200, 1180, 1158, 1095, 1070, 1019, 870, 817, 779, 709, 659 $cm^{-1}$; **$[\alpha]^D$ $_{25\,°C}$** = +15.0 ° (c = 0.4, $CHCl_3$); **HPLC separation:** DAICEL CHIRALPAK® AS-3, Heptane: iPOH 98:2, 1 mL/min; $t_1$ = 22.0 (major), $t_2$ = 23.9 min (minor).

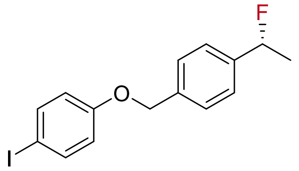

(*R*)-**2w**

**(*R*)-1-(1-fluoroethyl)-4-((4-iodophenoxy)methyl)benzene (2w)**

(*R*)-**2w** was prepared according to General Procedure 7 employing 0.3 mmol *rac*-**1w** (125 mg), the reaction was stirred at 25 °C at 1200 rpm for 72 h. Following purification (FCC eluent: 100:0 – 90:10 pentane: DCM) the product was isolated as a white solid, 55 mg, 52 % yield, 89:11 e.r.

**$^1$H NMR** (400 MHz, $CDCl_3$) δ = 7.60 – 7.51 (m, 2H), 7.45 – 7.39 (m, 2H), 7.39 -7.34 (d, 2H), 6.79 – 6.70 (m, 2H), 5.64 (dd, *J* = 47.6, 6.4 Hz, 1H), 5.04 (s, 2H), 1.64 (dd, *J* = 23.9, 6.5 Hz, 3H); **$^{19}$F NMR** (377 MHz, $CDCl_3$) δ = -167.5; **$^{13}$C NMR** (101 MHz, $CDCl_3$) δ = 158.7, 141.6 (d, $J_{C\text{-}F}$ = 19.7 Hz), 138.4, 136.7 (d, $J_{C\text{-}F}$ = 2.0 Hz), 127.7, 125.7 (d, $J_{C\text{-}F}$ = 6.7 Hz), 117.4, 90.8 (d, $J_{C\text{-}F}$ = 167.8 Hz), 83.3, 69.9, 23.1 (d, $J_{C\text{-}F}$ = 25.2 Hz); **IR** (neat) ν = 2959, 2925, 2853, 1584, 1467, 1380, 1280, 1235, 1177, 1060, 1009, 861, 809, 741, 633 $cm^{-1}$; **mp** 45-47 ˚C; **$[\alpha]^D$ $_{25\,°C}$** = +7.3 ° (c = 0.4, $CHCl_3$); **HPLC separation:** DAICEL CHIRALPAK® OJ-3, Heptane:iPOH 98.0:2.0, 1 mL/min; $t_1$ = 24.1 (major), $t_2$ = 29.0 min (minor). No HRMS obtained.

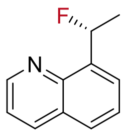

(*R*)-**2x**

**(*R*)-8-(1-fluoroethyl)quinoline (2x)**

(*R*)-**2x** was prepared according to General Procedure 7 employing 0.3 mmol of *rac*-**1x** (71 mg), the reaction was stirred at 25 °C at 1200 rpm for 72 h. Following purification (FCC eluent: 80:20 – 50:50 pentane:DCM) the product was isolated as a pale yellow oil, 42 mg, 79 % yield, 93:7 e.r.

**Scale up:** A 100 mL round bottom flask was charged with 4.7 mmol of *rac*-**1u** (1.1 g), catalyst (*S*)-**3h** (10 mol%), $Ph_4P^+$ $I^-$ (10 mol%) and KF (2.5 equiv.). *p*-xylene was added (20 mL) and the reaction was stirred at 25 °C at 1200 rpm for 72 h. The crude was filtered and washed with DCM was removed under reduced pressure. Following purification (FCC eluent: 80:20 – 50:50 pentane:DCM), the product was

isolated as a pale yellow oil (576 mg, 70% yield, 6:94 e.r.) and (S)-**3h** was recovered eluting off the column in 50:50 DCM:pentane (98% recovered).

**$^1$H NMR** (400 MHz, $CDCl_3$) δ = 8.91 (dd, *J* = 4.2, 1.8 Hz, 1H), 8.17 (dd, *J* = 8.3, 1.8 Hz, 1H), 7.87 (dt, *J* = 7.2, 1.2 Hz, 1H), 7.78 (dd, *J* = 8.2, 1.5 Hz, 1H), 7.59 (dd, *J* = 8.2, 7.2 Hz, 1H), 7.47 – 7.38 (m, 1H), 6.82 (dq, *J* = 47.4, 6.4 Hz, 1H), 1.81 (dd, *J* = 24.5, 6.4 Hz, 3H); **$^{19}$F NMR** (377 MHz, $CDCl_3$, 298 K) δ = -177.5; **$^{13}$C NMR** (151 MHz, $CDCl_3$) δ = 149.7, 144.8 (d, $J_{C\text{-}F}$ = 5.4 Hz), 140.4 (d, $J_{C\text{-}F}$ = 19.3 Hz), 136.4, 128.1, 127.7, 126.6, 125.0 (d, $J_{C\text{-}F}$ = 10.8 Hz), 121.26, 88.2 (d, $J_{C\text{-}F}$ = 166.2 Hz), 23.3 (d, $J_{C\text{-}F}$ = 24.8 Hz); **HRMS** ($ESI^+$) m/z calculated for $C_{11}H_{11}FN$ $(M+H)^+$ 176.0870, found 176.0869; **IR** (liquid film) ν = 3650, 2981, 2890, 1732, 1714, 1466, 1378, 1291, 1259, 1194, 1092, 1028, 891, 842, 793, 667 $cm^{-1}$; **$[\alpha]^D{}_{25\,°C}$** = +11.6 ° (c = 0.2, $CHCl_3$); **HPLC separation:** DAICEL CHIRALPAK® IB-3, Heptane: iPrOH = 99.5:0.5, 1 mL/min; $t_1$ = 4.0 (minor), $t_2$ = 4.3 min (major).

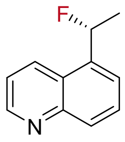


(*R*)-**2y**

**(*R*)-5-(1-fluoroethyl)quinoline (2y)**

(*R*)-**2y** was prepared according to General Procedure 7 employing 0.35 mmol of *rac*-**1y** (82 mg), the reaction was stirred at 25 °C at 1200 rpm for 48 h. Following purification (FCC eluent: 100:0 – 90:10 DCM: EtOAc) the product was isolated as a yellow oil, 60 mg, 99 % yield, e.r. 97:3.

**$^1$H NMR** (400 MHz, $CDCl_3$) δ = 8.95 (dd, *J* = 4.2, 1.7 Hz, 1H), 8.44 (ddt, *J* = 8.7, 1.8, 1.0 Hz, 1H), 8.11 (dq, *J* = 8.5, 1.1 Hz, 1H), 7.72 (ddd, *J* = 8.3, 7.2, 0.8 Hz, 1H), 7.67 – 7.59 (m, 1H), 7.45 (dd, *J* = 8.6, 4.2 Hz, 1H), 6.28 (dq, *J* = 46.8, 6.5 Hz, 1H), 1.84 (dd, *J* = 23.6, 6.5 Hz, 3H); **$^{19}$F NMR** (377 MHz, $CDCl_3$, 298 K) δ = -167.6; **$^{13}$C NMR** (126 MHz, $CDCl_3$) δ = 150.4, 148.7, 137.3 (d, $J_{C\text{-}F}$ = 18.4 Hz), 132.1, 130.5, 129.0, 125.6 (d, $J_{C\text{-}F}$ = 2.7 Hz),123.6 (d, $J_{C\text{-}F}$ = 8.8 Hz), 121.3, 88.8 (d, $J_{C\text{-}F}$ = 168.1 Hz), 22.4 (d, $J_{C\text{-}F}$ = 24.9 Hz); **HRMS** ($ESI^+$) m/z calculated for $C_{11}H_{11}FN$ $(M+H)^+$ 176.0870, found 176.0868; **IR** (liquid film) ν = 3659, 2981, 2889, 1599, 1505, 1462, 1378, 1316, 1252, 1165, 1075, 1054, 952, 829 $cm^{-1}$; **$[\alpha]^D{}_{25\,°C}$** = +17.9 ° (c = 0.5, $CHCl_3$); **HPLC separation:** DAICEL CHIRALPAK® OJ-3, Heptane: iPrOH = 90:10, 1 mL/min; $t_1$ = 6.2 (major), 6.8 min (minor).

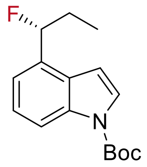


(*R*)-**2z**

**(*R*)-*tert*-butyl-4-(1-fluoropropyl)-1*H*-indole-1-carboxylate (2z)**

(*R*)-**2z** was prepared according to General Procedure 7 employing 0.2 mmol of *rac*-**1z** (66 mg) and the reaction was stirred at 25 °C at 1200 rpm for 72 h. Following purification (FCC eluent: 100:0 – 80:20 pentane:DCM) the product was isolated as a colourless oil, 35 mg, 65 % yield, e.r. 87:13.

**$^1$H NMR** (400 MHz, $CDCl_3$) δ = 8.14 (d, *J* = 8.3 Hz, 1H), 7.62 (d, *J* = 3.8 Hz, 1H), 7.31 (ddd, *J* = 8.3, 7.3, 1.0 Hz, 1H), 7.19 (dq, *J* = 7.6, 1.1 Hz, 1H), 6.68 (ddd, *J* = 3.8, 1.3, 0.8 Hz, 1H), 5.69 (ddd, *J* = 47.2, 7.6, 5.4 Hz,

1H), 2.21 – 1.88 (m, 2H), 1.67 (s, 9H), 1.00 (t, *J* = 7.4 Hz, 3H); **$^{19}$F NMR** (377 MHz, $CDCl_3$, 298 K) δ = -176.7; **$^{13}$C NMR** (126 MHz, $CDCl_3$) δ = 149.8, 135.6, 132.5 (d, $J_{C\text{-}F}$ = 20.1 Hz), 127.8, 126.2, 124.2, 119.8 (d, $J_{C\text{-}F}$ = 7.7 Hz), 115.2, 105.6 (d, $J_{C\text{-}F}$ = 1.9 Hz), 95.1 (d, $J_{C\text{-}F}$ = 170.6 Hz), 84.0, 29.9 (d, $J_{C\text{-}F}$ = 24.4 Hz), 28.3, 9.8 (d, $J_{C\text{-}F}$ = 5.8 Hz); **HRMS** ($ESI^+$) m/z calculated for $C_{16}H_{21}FNO_2$ $(M+H)^+$ calculated 278.1551, found 278.1812; **IR** (liquid film) ν = 3021, 2936, 2843, 1737, 1536, 1432, 1389, 1371, 1349, 1324, 1285, 1261, 1217, 1139, 1031, 810, 764 $cm^{-1}$; **$[\alpha]^D_{25\,°C}$** = +20.6 ° (c = 0.2, $CHCl_3$); **HPLC separation:** DAICEL CHIRALPAK® IC-3 Heptane:iPOH = 99.8:0.2, 1mL/min; $t_1$ = 10.7 (minor), $t_2$ = 11.3 min (major).

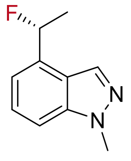
(*R*)-**2aa**

**(*R*)-4-(1-fluoroethyl)-1-methyl-1*H*-indazole (2aa)**

(*R*)-**2aa** was prepared according to General Procedure 7 employing 0.3 mmol of *rac*-**1aa** (71 mg), the reaction was stirred at 25 °C at 1200 rpm for 72 h. Following purification (FCC eluent: 100% DCM) the product was isolated as a pale-yellow oil, 37 mg, 70 % yield, 91.5:8.5 e.r.

**$^1$H NMR** (400 MHz, $CDCl_3$) δ = 8.12 – 8.07 (m, 1H), 7.55 – 7.24 (m, 2H), 7.13 – 7.05 (m, 1H), 5.99 (dqd, *J* = 47.1, 6.5, 0.8 Hz, 1H), 4.09 (s, 3H), 1.78 (dd, *J* = 23.9, 6.5 Hz, 3H); **$^{19}$F NMR** (377 MHz, $CDCl_3$, 298 K) δ = -169.7; **$^{13}$C NMR** (101 MHz, $CDCl_3$) δ = 140.4, 135.0 (d, $J_{C\text{-}F}$ = 20.6 Hz), 131.7 (d, $J_{C\text{-}F}$ = 2.9 Hz), 126.2, 121.0 (d, $J_{C\text{-}F}$ = 3.8 Hz), 116.6 (d, $J_{C\text{-}F}$ = 8.3 Hz), 109.1 (d, $J_{C\text{-}F}$ = 1.5 Hz), 90.4 (d, $J_{C\text{-}F}$ = 167.8 Hz), 35.7, 22.8 (d, $J_{C\text{-}F}$ = 25.0 Hz); **HRMS** (EI) m/z calculated for $C_{10}H_{11}FN_2$ $(M)^{+\cdot}$ 178.09008, found 178.09063; **IR** (liquid film) ν = 2934, 1726, 1676, 1613, 1513, 1452, 1411, 1375, 1345, 1275, 1204, 1169, 1111, 1080, 1038, 996, 847, 789, 652 $cm^{-1}$; **$[\alpha]^D_{25\,°C}$** = +17.4 ° (c = 0.4, $CHCl_3$); **HPLC separation:** DAICEL CHIRALPAK® IB-3, Heptane: iPrOH = 99:1, 1 mL/min; $t_1$ = 7.4 (minor), $t_2$ = 7.9 min (major).

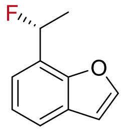
(*R*)-**2ab**

**(*R*)-7-(1-fluoroethyl)benzofuran (2ab)**

(*R*)-**2ab** was prepared according to General Procedure 7 employing 0.3 mmol of *rac*-**1ab** (67 mg), the reaction was stirred at 25 °C at 1200 rpm for 72 h. Following purification (FCC eluent: 100% pentane) the product was isolated as a colourless oil, 36 mg, 74 % yield, 91:9 e.r.

**$^1$H NMR** (400 MHz, $CDCl_3$) δ = 7.65 (d, *J* = 2.2 Hz, 1H), 7.59 (dt, *J* = 7.7, 1.1 Hz, 1H), 7.36 (dq, *J* = 7.5, 1.1 Hz, 1H), 7.30 – 7.22 (m, 1H), 6.80 (d, *J* = 2.2 Hz, 1H), 6.13 (dq, *J* = 47.0, 6.5 Hz, 1H), 1.81 (dd, *J* = 24.0, 6.5 Hz, 3H); **$^{19}$F NMR** (377 MHz, $CDCl_3$, 298 K) δ = -171.3; **$^{13}$C NMR** (151 MHz, $CDCl_3$) δ = 151.6 (d, $J_{C\text{-}F}$ = 5.0 Hz), 145.1, 127.9, 125.4 (d, $J_{C\text{-}F}$ = 20.9 Hz), 123.0, 121.4 (d, *J* = 2.3 Hz), 120.9 (d, $J_{C\text{-}F}$ = 7.6 Hz), 106.8, 87.3 (d, $J_{C\text{-}F}$ = 167.7 Hz), 21.9 (d, $J_{C\text{-}F}$ = 25.2 Hz); **IR** (liquid film) ν = 2929, 1721, 1428, 1325, 1262, 1217, 1032, 867, 832, 764, 668 $cm^{-1}$; **$[\alpha]^D_{25\,°C}$** = +19.4 ° (c = 0.4, $CHCl_3$); **HPLC separation:** DAICEL CHIRALPAK® ID-3, Heptane 100%, 1 mL/min, $t_1$ = 6.2 (minor), $t_2$ = 9.1 min (major). No HRMS obtained.

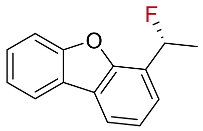

(*R*)-**2ac**

**(*R*)-4-(1-fluoroethyl)dibenzo[*b,d*]furan (2ac)**

(*R*)-**2ac** was prepared according to General Procedure 7 employing 0.16 mmol of *rac*-**1ac** (45 mg) and the reaction was stirred at 25 °C at 1200 rpm for 72 h. Following purification (FCC eluent: 100% pentane) the product was isolated as a colourless oil, 30 mg, 85 % yield, 92:8 e.r.

**$^{1}$H NMR** (400 MHz, $CDCl_3$) δ = 8.00 – 7.89 (m, 2H), 7.60 (dt, *J* = 8.3, 0.9 Hz, 1H), 7.57 – 7.51 (m, 1H), 7.48 (ddd, *J* = 8.3, 7.3, 1.4 Hz, 1H), 7.45 – 7.31 (m, 2H), 6.24 (dq, *J* = 47.0, 6.5 Hz, 1H), 1.89 (dd, *J* = 24.0 Hz, 6.5 Hz, 3H); **$^{19}$F NMR** (377 MHz, $CDCl_3$, 298 K) δ = -171.9; **$^{13}$C NMR** (126 MHz, $CDCl_3$) δ = 156.3, 152.7 (d, $J_{C-F}$ = 5.1 Hz), 127.5, 125.9, 125.7, 124.6, 124.1, 123.6 (d, $J_{C-F}$ = 7.7 Hz), 123.0 (d, $J_{C-F}$ = 2.2 Hz), 120.9, 120.6 (d, $J_{C-F}$ = 1.9 Hz), 111.9, 87.0 (d, $J_{C-F}$ = 168.1 Hz), 22.1 (d, $J_{C-F}$ = 25.0 Hz); **HRMS** (EI) m/z calculated for $C_{14}H_{11}FO$ $(M)^{+\cdot}$ 214.07884, found 214.07939; **IR** (liquid film) ν = 3500, 3444, 1453, 1425, 1343, 1219, 1191, 1127, 1056, 1009, 910, 861, 757 $cm^{-1}$; **$[\alpha]^{D}$$_{25\,°C}$** = +7.6 ° (c = 0.8, $CHCl_3$); **HPLC separation:** DAICEL CHIRALPAK® OJ-3 Heptane: iPOH = 99:1, 1mL/min; $t_1$ = 5.9 (major), $t_2$ = 6.7 min (minor).

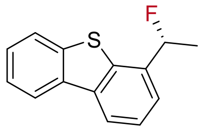

(*R*)-**2ad**

**(*R*)-4-(1-fluoroethyl)dibenzo[*b,d*]thiophene (2ad)**

(*R*)-**2ad** was prepared according to General Procedure 7 employing 0.25 mmol of *rac*-**1ad** (73 mg) and the reaction was stirred at 25 °C at 1200 rpm for 72 h. Following purification (FCC eluent: 100% pentane) the product was isolated as a pale-yellow oil, 54 mg, 94 % yield, 92:8 e.r.

**$^{1}$H NMR** (500 MHz, $CDCl_3$) δ = 8.21 – 8.09 (m, 2H), 7.92 – 7.84 (m, 1H), 7.55 – 7.44 (m, 4H), 5.97 (dq, *J* = 46.7, 6.5 Hz, 1H), 1.82 (dd, *J* = 24.0, 6.5 Hz, 3H); **$^{19}$F NMR** (470 MHz, $CDCl_3$) δ = -170.6; **$^{13}$C NMR** (126 MHz, $CDCl_3$) δ = 139.4 (d, $J_{C-F}$ = 1.5 Hz), 136.5, 136.2 (d, $J_{C-F}$ = 3.8 Hz), 136.1 (d, $J_{C-F}$ = 20.5 Hz), 135.5, 127.1, 124.9, 124.7, 123.0 (d, $J_{C-F}$ = 8.0 Hz), 122.8, 121.8, 121.5 (d, $J_{C-F}$ = 1.6 Hz), 90.3 (d, $J_{C-F}$ = 170.1 Hz), 21.5 (d, $J_{C-F}$ = 25.0 Hz); **IR** (liquid film) ν = 3066, 2985, 2926, 1707, 1561, 1444, 1407, 1375, 1348, 1304, 1256, 1176, 1122, 1074, 1041, 1022, 1005, 904, 831, 780, 752, 723, 706, 625 $cm^{-1}$; **$[\alpha]^{D}$$_{25\,°C}$** = +9.0 ° (c = 0.3, $CHCl_3$); **HPLC separation:** DAICEL CHIRALPAK® OJ-3, Heptane:iPOH 99:1, 1mL/min; $t_1$ = 12.7 (major), $t_2$ = 18.8 min (minor). No HRMS obtained.

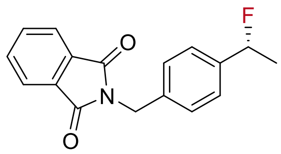

(*R*)-**2ae**

**(*R*)-2-(4-(1-fluoroethyl)benzyl)isoindoline-1,3-dione (2ae)**

(*R*)-**2ae** was prepared according to General Procedure 7 employing 0.25 mmol of *rac*-**1ae** (93 mg) and the reaction was stirred at 40 °C at 1200 rpm for 72 h. Following purification (FCC eluent: 90:10 pentane: EtOAc), 42 mg, 59 % yield, 90:10 e.r.

**$^{1}$H NMR** (500 MHz, $CDCl_3$) δ = 7.85 (dd, *J* = 5.4, 3.1 Hz, 2H), 7.75 – 7.67 (m, 2H), 7.45 (d, *J* = 7.9 Hz, 2H), 7.30 (d, *J* = 7.7 Hz, 2H), 5.59 (dq, *J* = 47.6, 6.5 Hz, 1H), 4.85 (s, 2H), 1.60 (dd, *J* = 23.9, 6.4 Hz, 3H); **$^{19}$F NMR** (471 MHz, $CDCl_3$) δ = -167.5; **$^{13}$C NMR** (151 MHz, $CDCl_3$) δ = 168.2, 141.3 (d, *J* = 19.6 Hz), 136.5 (d, *J* = 2.1 Hz), 134.2, 132.3, 128.9, 125.7 (d, *J* = 6.6 Hz), 123.5, 90.8 (d, *J* = 167.8 Hz), 41.4, 23.0 (d, *J* = 25.0 Hz); **HRMS** ($ESI^+$) m/z calculated for $C_{17}H_{14}FNO_2$ $(M+H)^+$ 284.1081, found 284.0734; **IR** (liquid film) ν = 2991, 2953, 2926, 2854, 1770, 1719, 1612, 1462, 1428, 1395, 1354, 1262, 1150, 1087, 1024, 939, 895, 808, 738, 677, 653, 638, 624, 613 $cm^{-1}$; **$[\alpha]^{D}_{25\,°C}$** = +17.4° (c = 0.2, $CHCl_3$); **mp** 88-90 ˚C; **HPLC separation:** DAICEL CHIRALPAK® OJ-3, Heptane:iPOH 90:10, 1 mL/min; $t_1$ = 15.8 (major), $t_2$ = 17.5 min (minor).

*Absolute configuration* was determined by comparing optical rotation and chromatograph of **2ae** to that of literature values[1].

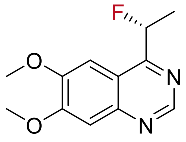

(*R*)-**2af**

**(*R*)-4-(1-fluoroethyl)-6,7-dimethoxyquinazoline (2af)**

(*R*)-**2af** was prepared according to modified General Procedure 7 using 1,4-difluorobenzene (0.25 M) as solvent, employing 0.24 mmol of *rac*-**1af** (71 mg), the reaction was stirred at 40 °C at 1200 rpm for 96 h. Following purification (FCC eluent: 80:20 – 50:50 pentane:EtOAc) the product was isolated as an off-white solid, 65 % yield, 78:22 e.r.

**$^{1}$H NMR** (500 MHz, $CDCl_3$) δ 9.11 (d, *J* = 0.9 Hz, 1H), 7.55 (s, 1H), 7.36 (s, 1H), 6.14 (dq, *J* = 48.0, 6.6 Hz, 1H), 4.07 (s, 3H), 4.05 (s, 3H), 1.89 (dd, *J* = 24.3, 6.6 Hz, 3H); **$^{19}$F NMR** (471 MHz, $CDCl_3$) δ = -174.1; **$^{13}$C NMR** (151 MHz, $CDCl_3$) δ = 163.5 (d, $J_{C\text{-}F}$ = 20.7 Hz), 156.1, 153.1, 150.5, 149.5, 118.1, 107.3, 102.2 (d, $J_{C\text{-}F}$ = 9.2 Hz), 91.7 (d, $J_{C\text{-}F}$ = 171.1 Hz), 56.5, 56.4, 20.5 (d, $J_{C\text{-}F}$ = 23.3 Hz); **IR** (neat) ν = 3011, 2989, 2940, 2838, 2363, 1711, 1619, 1503, 1476, 1430, 1352, 1303, 1286, 1237, 1209, 1182, 1135, 1086, 1032, 1014, 979, 890, 872, 817, 784, 761, 633 $cm^{-1}$; **HRMS** ($ESI^+$) m/z calculated for $C_{12}H_{14}FN_2O_2$ $(M+H)^+$ 237.1034, found 237.1030; **mp** 105-106 ˚C; $[\alpha]^{D}_{25\,°C}$ = +14.6 ° (c = 0.4, $CHCl_3$); **HPLC separation:** DAICEL CHIRALPAK® OJ-3 Heptane: iPOH 92:8, 1 mL/min; $t_1$ = 9.9 (major), $t_2$ = 10.8 min (minor).

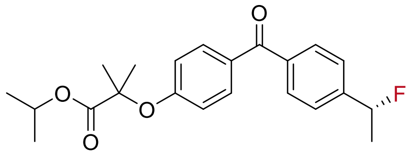

(*R*)-**2ag**

**isopropyl (*R*)-2-(4-(4-(1-fluoroethyl)benzoyl)phenoxy)-2-methylpropanoate (2ag)**

(*R*)-**2ag** was prepared according to General Procedure 7 employing 0.3 mmol of *rac*-**1ag** (130 mg), the reaction was stirred at 40 °C at 1200 rpm for 72 h. Following purification (FCC eluent: 1st column: 100% DCM, 2nd column: 100:0 – 90:10 pentane:EtOAc) the product was isolated as an colourless oil, 60 mg, 54 % yield, 93:7 e.r.

[1]**H NMR** (600 MHz, $CDCl_3$) δ = 7.76 (dd, *J* = 8.6, 1.9 Hz, 4H), 7.47 – 7.40 (m, 2H), 6.91 – 6.83 (m, 2H), 5.70 (dq, *J* = 47.6, 6.5 Hz, 1H), 5.09 (hept, *J* = 6.3 Hz, 1H), 1.72 – 1.62 (m, 9H), 1.21 (s, 3H), 1.20 (s, 3H); [19]**F NMR** (565 MHz, $CDCl_3$) δ = -170.4; [13]**C NMR** (151 MHz, $CDCl_3$) δ = 195.2, 173.3, 159.8, 145.6 (d, $J_{C\text{-}F}$ = 19.4 Hz), 138.1, 132.2, 130.7, 130.2, 125.0 (d, $J_{C\text{-}F}$ = 7.1 Hz), 117.3, 90.6 (d, $J_{C\text{-}F}$ = 169.4 Hz), 79.5, 69.5, 25.5, 23.2 (d, $J_{C\text{-}F}$ = 24.9 Hz), 22.5; **IR** (liquid film) ν = 3130, 2973, 2884, 2825, 2356, 2338, 1746, 1625, 1575, 1503, 1476, 1380, 1338, 1225, 1162, 1050, 952, 817, 784, 643 $cm^{-1}$; **HRMS** ($ESI^+$) m/z calculated for $C_{22}H_{16}FO_4$ $(M+H)^+$ 373.1810, found 373.1806; **$[\alpha]^D_{25\,°C}$** = +9.2 ° (c = 0.2, $CHCl_3$); **HPLC separation:** DAICEL CHIRALPAK® OJ-3, Heptane:iPOH 97:3, 0.8 mL/min; $t_1$ = 13.1 (minor), $t_2$ = 14.0 min (major).

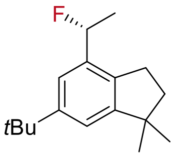

(*R*)-**2ah**

**(*R*)-6-(*tert*-butyl)-4-(1-fluoroethyl)-1,1-dimethyl-2,3-dihydro-1*H*-indene (2ah)**

(*R*)-**2ah** was prepared according to General Procedure 7 employing 0.3 mmol of *rac*-**1ah** (93 mg), the reaction was stirred at 25 °C at 1200 rpm for 48 h. Following purification (FCC eluent: 100% pentane) the product was isolated as a colourless oil, 62 mg, 83 % yield, 93.5:6.5 e.r.

[1]**H NMR** (400 MHz, $CDCl_3$) δ = 7.25 – 7.24 (m, 1H), 7.16 (br s, 1H), 5.71 (dq, *J* = 47.3, 6.5 Hz, 1H), 2.97 – 2.77 (m, 2H), 1.95 (t, *J* = 7.2 Hz, 2H), 1.65 (dd, *J* = 23.5, 6.5 Hz, 3H), 1.34 (s, 9H), 1.27 (s, 3H), 1.26 (s, 3H); [19]**F NMR** (377 MHz, $CDCl_3$, 298 K) δ = -168.7; [13]**C NMR** (101 MHz, $CDCl_3$) δ = 153.1, 150.4, 136.7 (d, $J_{C\text{-}F}$ = 4.7 Hz), 136.4 (d, $J_{C\text{-}F}$ = 18.9 Hz), 119.8 (d, $J_{C\text{-}F}$ = 7.4 Hz), 118.9 (d, $J_{C\text{-}F}$ = 2.0 Hz), 90.0 (d, $J_{C\text{-}F}$ = 166.5 Hz), 43.9, 41.6, 35.0, 31.8, 28.9, 27.9, 22.1 (d, $J_{C\text{-}F}$ = 25.8 Hz); **IR** (liquid film) ν = 2864, 1629, 1480, 1395, 1375, 1216, 1073, 755, 670, 643 $cm^{-1}$; **$[\alpha]^D_{25\,°C}$** = +18.4° (c = 0.2, $CHCl_3$); **HPLC separation:** DAICEL CHIRALPAK® DAICEL CHIRALPAK® IB-3, Heptane:iPOH 99.8:0.2, 0.4 mL/min; $t_1$ = 5.3 (major), $t_2$ = 5.8 min (minor). No HRMS obtained.

## Challenging Substrates

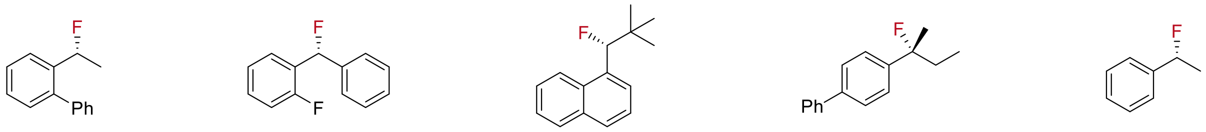

**2ai** 6%[a] | **2ag** 0%[b] | **2ak** 0%[b] | **2al** 0%[b] | **2am** 21%[a] 83:17 e.r.

**Supplementary Figure 2**: Challenging substrates under S-HBPTC. General conditions: Substrate (0.05 mmol), (*S*)-**3h** (10 mol%), $Ph_4P^+ I^-$ (10 mol%) and KF (2.5 equiv.) in 200 µL of *p*-xylene stirred at 1200 rpm at 25 °C for 72 h [a]Determined by $^{19}$F NMR using 4-fluoroanisole as internal standard [b]Reactions performed at 40 °C

## Evaluation of Alternative Electrophiles under S-HBPTC

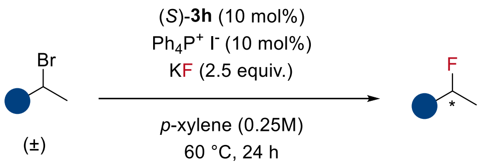


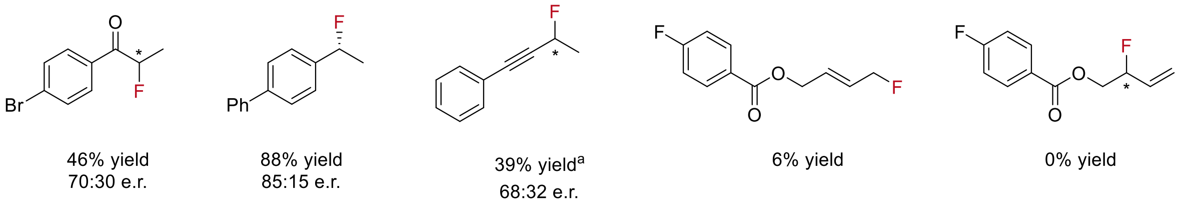


**Supplementary Figure 3**: Reactions performed under S-HBPTC conditions evaluating different electrophiles

General conditions: Substrate (0.05 mmol), urea catalyst (10 mol%), $Ph_4P^+$ $I^-$ and KF (2.5 equiv.) in 200 µL of *p*-xylene stirred at 1200 rpm. Yields determined by $^{19}F$ NMR using 4-fluoroanisole as internal standard, e.r. was determined by HPLC analysis using a chiral stationary phase. nd = not determined. [a]Reaction performed for 72 h.

## Reaction Optimisation – α-Haloketones

Supplementary Table 6: Optimisation of for the fluorination of α-bromoketones

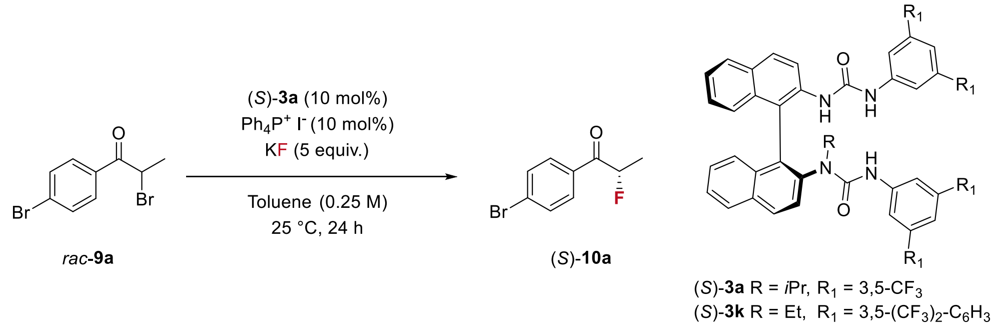


| Entry | Urea Catalyst | Onium catalyst | Solvent | Temperature/ Time | **10a** yield (%)[a] | **9a** (%) (R.S.M) | **e.r.**[b] |
|---|---|---|---|---|---|---|---|
| **1** | (*S*)-**3a** | $Ph_4P^+ I^-$ | Toluene (0.25 M) | 25 °C / 24 h | 21 | 79 | 63:37 |
| **2** | (*S*)-**3a** | $Ph_4P^+ I^-$ | MeCN (0.25 M) | 25 °C / 24 h | 28 | 72 | 71:29 |
| **3** | (*S*)-**3k** | $Ph_4P^+ I^-$ | MeCN (0.25 M) | 25 °C / 24 h | 44 | 47 | 92:8 |
| **4** | (*S*)-**3k** | $Et_4N^+ I^-$ | MeCN (0.25 M) | 25 °C / 24 h | 60 | 39 | 92:8 |
| **5** | (*S*)-**3k** | $Et_4N^+ I^-$ | MeCN (0.25 M) | 5 °C, 96 h | 43 | 55 | 95:5 |
| **6** | (*S*)-**3k** | $Et_4N^+ I^-$ | MeCN (0.5 M) | 5 °C, 96 h | 73 | 26 | 95:5 |

General conditions: Substrate (0.05 mmol), urea catalyst (10 mol%), onium salt (10 mol%) and KF (5 equiv.) in 200 μL of solvent stirred at 1200 rpm for specified time. [a]Determined by $^{19}F$ NMR using 4-fluoroanisole as internal standard, [b]e.r. was determined by HPLC analysis using a chiral stationary phase. R.S.M = remaining starting material.

Supplementary Table 7: Control investigations for the fluorination of α-ketosulfonium salt

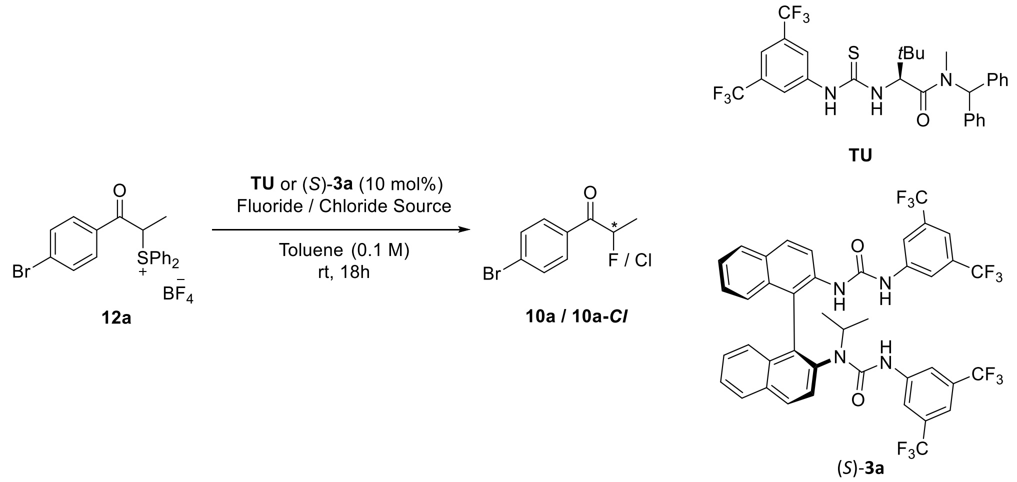


| Entry | Catalyst | Fluoride / Chloride source | Yield **10**[a] | **e.r.** |
|---|---|---|---|---|
| **1** | **TU** | NaCl (sat aq) | 57 | 88:12 |
| **2** | **TU** | NaCl (s) | 61 | 67:33 |
| **3** | **TU** | KF (s) | 0 | - |
| **4** | **TU** | CsF (s) | 1 | nd |
| **5** | **TU** | KF (sat aq) | 0 | - |
| **6** | (*S*)-**3a** | KF (s) | 0 | - |
| **7** | (*S*)-**3a** | CsF (s) | 0 | - |
| **8** | (*S*)-**3a** | KF (sat aq) | 0 | - |

General conditions: Substrate (0.05 mmol), HBD catalyst (10 mol%), KF, CsF or NaCl (2.5 equiv.) or KF or NaCl (sat aq) (0.5 mL) in toluene (0.1 M). [a]Determined by quantitative $^{19}F$ using 4-fluoroanisole as internal standard. nd = not determined. Sulfonium **12a** prepared according to literature procedure[26]. **TU** catalyst commercially available (CAS: 1186602-28-7)

**Substrate Synthesis and Characterisation – α-Bromoketones**

General procedure 8

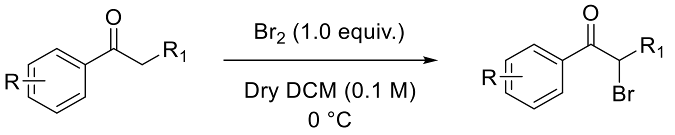


Bromide substrates were prepared from corresponding ketones via bromination with $Br_2$ (2.5 mmol, 1 equiv.) in dry DCM (0.1 M) at 0°C following a literature procedure.[26] After the completion of the reaction (18 h) the mixture was quenched slowly with $Na_2S_2O_3$ (sat aq.) and the aqueous layer was extracted with DCM (3 x 20 mL). The combined organic extracts were dried with $Na_2SO_4$ and concentrated under reduced pressure. Products were subsequently purified by FCC.

General procedure 9

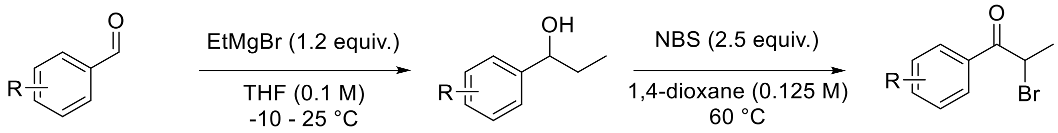


Bromide substrates were prepared via a two-step protocol following a literature procedure.[27] Alcohol intermediates were prepared through Grignard addition to the corresponding aldehydes in THF at -10 °C. The reactions were monitoring by TLC and quenched with saturated $NH_4Cl$ solution when the aldehyde was fully consumed. Following work up in $Et_2O/H_2O$ the organic extracts were washed with brine and dried with $MgSO_4$, solvent was removed under reduced pressure to give the corresponding alcohol.

The alcohol was dissolved in 1,4-dioxane (0.125 M), NBS (2.5 equiv.) was added and the reaction was stirred at 60 °C. The reaction was monitored by TLC and upon completion quenched with $Na_2S_2O_3$ (sat aq.), aqueous layer the extracted with $Et_2O$ (15 mL x 3). The combined organic extracts were washed with brine and dried with $MgSO_4$. After concentration under reduced pressure the products were purified by FCC.

Bromides *rac*-**9a** (CAS: 38786-67-3), *rac*-**9b** (CAS: 877-37-2), *rac*-**9c** (CAS: 345-94-8), *rac*-**9i** (CAS: 2114-00-3), *rac*-**9j** (CAS: 1451-82-7), *rac*-**9k** (CAS 21086-33-9) were commercially available.

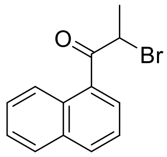

*rac*-**9d**

**2-bromo-1-(naphthalen-1-yl)propan-1-one (9d)**

Compound *rac*-**9d** was prepared according to General Procedure 9. Following purification (FCC eluent: pentane:DCM 80:20) the product was isolated as a white solid in 84 % yield.

**$^{1}$H NMR** (400 MHz, $CDCl_3$) δ = 8.44 (d, *J* = 8.6, 1H), 8.02 (dt, *J* = 8.2, 1.1 Hz, 1H), 7.93 – 7.83 (m, 2H), 7.67 – 7.46 (m, 3H), 5.37 (q, *J* = 6.7 Hz, 1H), 1.98 (d, *J* = 6.7 Hz, 3H); **$^{13}$C NMR** (101 MHz, $CDCl_3$) δ = 196.7, 134.0, 133.8, 133.2, 130.9, 128.5, 128.2, 126.7, 126.7, 125.6, 124.2, 45.6, 20.5; **HRMS** ($ESI^+$) m/z calculated for $C_{11}H_{12}BrO$ $(M+H)^+$ 263.0066, found 263.0065; **IR** (neat) ν = 3059, 2975, 2879, 2360, 1954, 1681, 1604, 1560, 1529, 1478, 1450, 1407, 1368, 1324, 1300, 1273, 1227, 1205, 1165, 1132, 1114, 1068, 1004, 951, 931, 901, 854, 806, 738, 696, 643 $cm^{-1}$; **mp** 81-83 ˚C.

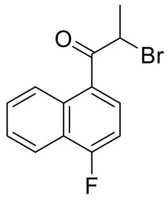

*rac*-**9e**

**2-bromo-1-(4-fluoronaphthalen-1-yl)propan-1-one (9e)**

Compound *rac*-**9e** was prepared according to General Procedure 9. Following purification (FCC eluent: pentane:DCM 70:30) the product was isolated as a white solid in 86 % yield.

**$^{1}$H NMR** (600 MHz, $CDCl_3$) δ = 8.58 (m, 1H), 8.21 – 8.15 (m, 1H), 7.91 (dd, *J* = 8.1, 5.2 Hz, 1H), 7.69 (ddd, *J* = 8.5, 6.8, 1.4 Hz, 1H), 7.63 (ddd, *J* = 8.1, 6.8, 1.1 Hz, 1H), 7.17 (dd, *J* = 9.7, 8.1 Hz, 1H), 5.36 (q, *J* = 6.6 Hz, 1H), 1.97 (d, *J* = 6.6 Hz, 3H); **$^{19}$F NMR** (565 MHz, $CDCl_3$) δ = - 131.5; **$^{13}$C NMR** (151 MHz, $CDCl_3$) δ = 195.3, 161. (d, $J_{C\text{-}F}$ = 260.7 Hz), 133.1 (d, $J_{C\text{-}F}$ = 5.5 Hz), 129.6 (d, $J_{C\text{-}F}$ = 4.5 Hz), 129.3, 128.1 (d, $J_{C\text{-}F}$ = 10.0 Hz), 127.1 (d, $J_{C\text{-}F}$ = 2.2 Hz), 125.9 (d, $J_{C\text{-}F}$ = 2.7 Hz), 124.3 (d, $J_{C\text{-}F}$ = 15.9 Hz), 120.9 (d, $J_{C\text{-}F}$ = 6.3 Hz), 108.1 (d, $J_{C\text{-}F}$ = 21.3 Hz), 45.0, 20.5; **HRMS** ($ESI^+$) m/z $C_{13}H_{11}BrFO$ $(M+H)^+$ 280.9972, found 280.9969; **IR** (neat) ν = 3346, 3075, 2966, 2936, 2869, 1855, 1683, 1630, 1602, 1578, 1509, 1466, 1424, 1394, 1379, 1338, 1230, 1186, 1168, 1143, 1109, 1061, 1044, 1019, 979, 898, 832, 706, 636 $cm^{-1}$; **mp** 80-81 ˚C.

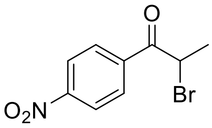

*rac*-**9f**

**2-bromo-1-(4-nitrophenyl)propan-1-one 9 (9f)**

Compound *rac*-**9f** was prepared according to General Procedure 8. Following purification (FCC eluent: pentane:DCM 60:40) the product was isolated as a yellow solid in 86 % yield.

**$^{1}$H NMR** (400 MHz, $CDCl_3$) δ = 8.37 – 8.29 (m, 2H), 8.22 – 8.14 (m, 2H), 5.26 (q, J = 6.6 Hz, 1H), 1.94 (d, J = 6.6 Hz, 3H).8.35-8.31 (m, 2H); **$^{13}$C NMR** (101 MHz, $CDCl_3$) δ = 191.7, 150.5, 138.8, 130.0, 123.9, 41.4, 19.8; **HRMS** ($ESI^+$) m/z calculated for $C_9H_8BrNO_3$ $(M+H)^+$ 257.9760, found 257.9948; **IR** (neat) ν = 2954, 2928, 2855, 1691, 1606, 1531, 1448, 1340, 1319, 1236, 1165, 1112, 997, 951, 854, 755, 714 $cm^{-1}$; **mp** 45-46 ˚C.

**4-(2-bromopropanoyl)benzonitrile (9g)**

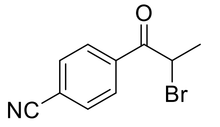

*rac*-**9g**

Compound *rac*-**9g** was prepared according to General Procedure 8. Following purification (FCC eluent: pentane:DCM 60:40) as a white solid in 85 % yield.

**$^1$H NMR** (400 MHz, $CDCl_3$) δ = 8.15 – 8.08 (m, 2H), 7.81 – 7.78 (m, 2H), 5.23 (q, J = 6.6 Hz, 1H), 1.92 (dd, J = 6.5, 1.7 Hz, 3H); **$^{13}$C NMR** (101 MHz, $CDCl_3$) δ= 191.9, 137.3, 132.5, 129.4, 117.8, 116.9, 41.2, 19.8; **HRMS** ($ESI^+$) m/z calculated for $C_{10}H_9BrNO$ $(M+H)^+$ 237.9862, found 237.9998; **IR** (neat) ν = 2988, 2356, 2228, 1934, 1692, 1566, 1439, 1407, 1376, 1339, 1294, 1241, 1152, 1119, 1056, 997, 951, 851, 756, 711, 688, 651 $cm^{-1}$; **mp** 75-76 ˚C.

**2-bromo-1-(4-(trifluoromethyl)phenyl)propan-1-one (9h)**

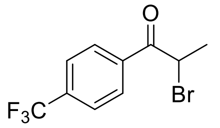

*rac*-**9h**

Compound *rac*-**9h** was prepared according to General Procedure 8. Following purification (FCC eluent: pentane:DCM, 70:30) the product was isolated as a white solid in 91 % yield.

**$^1$H NMR** (400 MHz, $CDCl_3$) δ = 8.17-8.09 (m, 2H), 7.79 – 7.72 (m, 2H), 5.26 (q, J = 6.6 Hz, 1H), 1.93 (d, J = 6.6 Hz, 3H); **$^{19}$F NMR** (376 MHz, $CDCl_3$) δ= -63.2; **$^{13}$C NMR** (101 MHz, $CDCl_3$) δ = 192.3, 136.9, 134.9 (q, $J_{C\text{-}F}$ =32.8 Hz) 129.3, 125.80 (q, $J_{C\text{-}F}$ = 3.7 Hz), 123.5, (q, $J_{C\text{-}F}$ = 272.8 Hz), 41.3, 19.9; **HRMS** ($ESI^+$) m/z calculated for $C_{10}H_8BrF_3O$ $(M+H)^+$ 280.9783, found 280.9403; **IR** (neat) ν = 2982, 2933, 2871, 1687, 1529, 1446, 1411, 1382, 1324, 1242, 1165, 1126, 1069, 1016, 998, 953, 861, 807, 768, 737, 701, 668 $cm^{-1}$; **mp** 40-42 ˚C.

**1-([1,1'-biphenyl]-4-yl)-2-bromobutan-1-one (9l)**

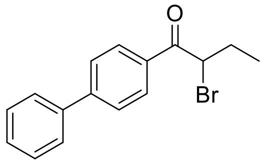

*rac*-**9l**

Compound *rac*-**9l** was prepared according to General Procedure 8. Following purification (FCC eluent: pentane:DCM 80:20) the product was isolated as a pale-yellow solid in 87 % yield.

**$^1$H NMR** (400 MHz, $CDCl_3$) δ = 8.12 – 8.09 (m, 2H), 7.73 – 7.70 (m, 2H), 7.65 – 7.62 (m, 2H), 7.51 – 7.39 (m, 2H), 7.48 – 7.37 (m, 1H), 5.11 (dd, *J* = 7.7, 6.4 Hz, 1H), 2.34 – 2.12 (m, 2H), 1.11 (t, *J* = 7.3 Hz, 3H); **$^{13}$C NMR** (101 MHz, $CDCl_3$) δ = 192.8, 146.4, 139.7, 133.2, 129.5, 129.0, 128.4, 127.4, 127.3, 49.2, 27.0, 12.2; **HRMS** ($ESI^+$) m/z calculated for $C_{16}H_{16}BrO$ $(M+H)^+$ 303.0379, found 303.0377; **IR** (neat) ν = 3340, 2975, 2939, 2879, 1680, 1604, 1560, 1529, 1487, 1450, 1407, 1324, 1300, 1273, 1226, 1206, 1164, 1133, 1068, 1004, 951, 901, 855, 801, 772, 738, 716, 696, 642 $cm^{-1}$; **mp** 66-68 ˚C.

**2-bromo-4-chloro-1-(4-fluorophenyl)butan-1-one (9m)**

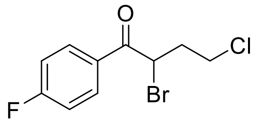

*rac*-**9m**

Compound *rac*-**9m** was prepared according to General Procedure 8. Following purification (FCC eluent: pentane:DCM 90:10) the product was isolated as a white solid in 73 % yield.

**$^1$H NMR** (400 MHz, $CDCl_3$) 8.12 – 8.03 (m, 2H), 7.23 – 7.13 (m, 2H), 5.43 (dd, *J* = 8.1, 5.8 Hz, 1H), 3.88 – 3.71 (m, 2H), 2.65 – 2.48 (m, 2H); **$^{19}$F NMR** (376 MHz, $CDCl_3$) δ= -103.2; **$^{13}$C NMR** (101 MHz, $CDCl_3$) δ

= 191.0, 166.2 (d, $J_{C-F}$ = 256.8 Hz), 131.8 (d, $J_{C-F}$ = 9.5 Hz), 130.4 (d, $J_{C-F}$ = 3.0 Hz), 116.1 (d, $J_{C-F}$ = 22.0 Hz), 43.5, 42.4, 35.7; **HRMS** (ESI$^+$) m/z calculated for $C_{10}H_{10}BrClFO$ $(M+H)^+$ 278.9582 found 279.0056; **IR** (neat) ν = 3355, 2973, 2880, 1683, 1602, 1560, 1529, 1507, 1487, 1448, 1408, 1372, 1322, 1300, 1260, 1228, 1205, 1158, 1129, 1023, 1004, 945, 901, 862, 844, 818, 772, 752, 739, 697, 652 cm$^{-1}$; **mp** 42-43 ˚C.

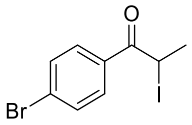

*rac*-**11a**

**1-(4-bromophenyl)-2-iodopropan-1-one (11a)**

Compound *rac*-**9a-*I*** was prepared through the addition of KI (3 equiv.) to **9a-*I*** in MeCN (0.25 M). Following filtration over celite the product was isolated as an off-white solid in 85 % yield. Spectroscopic data are in accordance with those in literature.[28]

**$^1$H NMR** (400 MHz, $CDCl_3$) δ 7.92 – 7.82 (m, 2H), 7.68 – 7.56 (m, 2H), 5.42 (q, *J* = 6.7 Hz, 1H), 2.07 (d, *J* = 6.7 Hz, 3H); **$^{13}$C NMR** (101 MHz, $CDCl_3$) δ 193.9, 132.5, 132.2, 130.3, 128.8, 22.0, 17.9;

**General Procedure for Enantioselective Fluorination of α-Bromoketones**

General Procedure 10: In a 7 mL screw-cap vial equipped with a magnetic stirring bar were sequentially added, pre-ground potassium fluoride (2.5 equiv.), the appropriate substrate (0.4 mmol, 1 equiv.), (*S*)-**3k** (10 mol%), $Et_4N^+$ $I^-$ (10 mol%) and MeCN (0.5 M). The vial was sealed, and reaction was stirred at 1200 rpm at the appropriate temperature for 96 h. The crude reaction mixture was directly purified by FCC to give product.

**Product Characterisation – α-Fluoroketones**

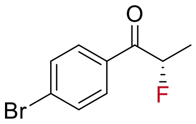
(*S*)-**10a**

**(*S*)-1-(4-bromophenyl)-2-fluoropropan-1-one (10a)**

(S)-**10a** was prepared according to General Procedure 10 employing 0.4 mmol of *rac*-**9a** (117 mg), the reaction was stirred at 5 °C at 1200 rpm for 96 h. Following purification (FCC eluent: pentane:DCM 70:30) the product was isolated as an off-white solid in 65 % yield, 60 mg, 95:5 e.r.

**$^1$H NMR** (600 MHz, $CDCl_3$) δ = 7.90 – 7.82 (m, 2H), 7.67 – 7.60 (m, 2H), 5.62 (dq, *J* = 48.5, 6.8 Hz, 1H), 1.66 (dd, *J* = 24.1, 6.8 Hz, 3H); **$^{19}$F NMR** (565 MHz, $CDCl_3$) δ = -180.5; **$^{13}$C NMR** (151 MHz, $CDCl_3$) δ = 196.3 (d, $J_{C-F}$ = 20.4 Hz), 132.9 (d, $J_{C-F}$ = 2.2 Hz), 132.2, 130.7 (d, $J_{C-F}$ = 5.0 Hz), 129.3, 90.8 (d, $J_{C-F}$ = 180.5 Hz), 18.3 (d, $J_{C-F}$ = 22.6 Hz); **HRMS** (ESI$^+$) m/z calculated for $C_9H_9BrFO$ $(M+H)^+$ 230.9815, found 230.9815; **IR** (neat) ν = 2991, 2901, 1701, 1586, 1567, 1486, 1445, 1399, 1378, 1280, 1239, 1132, 1072, 1037, 1012, 974, 887, 840, 774, 738, 702, 643 $cm^{-1}$; **mp** 39-42 ˚C; **$[\alpha]^D{}_{25\,°C}$** = **-**5.0 (c = 0.5, $CHCl_3$); **HPLC separation:** DAICEL CHIRALPAK® ID-3, Heptane: iPOH = 99.2:0.8, 1 mL/min; $t_1$ = 5.0 (major), $t_2$= 5.7 min (minor).

*Absolute configuration* was determined by comparing optical rotation and chromatograph of **10a** to that of literature values[2].

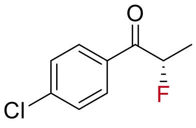
(*S*)-**10b**

**(*S*)-1-(4-chlorophenyl)-2-fluoropropan-1-one (10b)**

(*S*)-**10b** was prepared according to General Procedure 10 employing 0.4 mmol of *rac*-**9b** (99 mg), the reaction was stirred at 5 °C at 1200 rpm for 96 h. Following purification (FCC eluent: pentane:DCM 70:30) the product was isolated as a white solid in 68 % yield, 48 mg, 95:5 e.r.

**$^1$H NMR** (600 MHz, $CDCl_3$) δ = 7.97 – 7.91 (m, 2H), 7.49 – 7.43 (m, 2H), 5.63 (dq, *J* = 48.6, 6.8 Hz, 1H), 1.66 (dd, *J* = 24.1, 6.8 Hz, 3H); **$^{19}$F NMR** (565 MHz, $CDCl_3$) δ = -180.46 (m); **$^{13}$C NMR** (151 MHz, $CDCl_3$) δ = 195.9 (d, $J_{C-F}$ = 20.3 Hz), 140.3, 132.3 (d, $J_{C-F}$ = 2.4 Hz), 130.5 (d, $J_{C-F}$ = 4.4 Hz), 129.1, 90.6 (d, $J_{C-F}$ = 180.5 Hz), 18.2 (d, $J_{C-F}$ = 22.6 Hz); **HRMS** (ESI$^+$) m/z calculated for $C_9H_9ClFO$ $(M+H)^+$ 187.0321, found 187.0554; **IR** (neat) ν = 3379, 2996, 1929, 1978, 1698, 1591, 1571, 1489, 1447, 1403, 1379, 1283, 1230,

1132, 1092, 1038, 1014, 974, 889, 843, 778, 742, 672, 651 $cm^{-1}$; **mp** 33-34 ˚C; **$[\alpha]^{D}_{25\,°C}$** = -5.4 (c = 0.5, $CHCl_3$); **HPLC separation:** DAICEL CHIRALPAK® ID-3, Heptane: iPOH = 99.2:0.8 mL/min; $t_1$ = 4.4 (major), $t_2$= 5.0 min (minor).

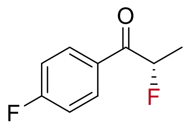

(*S*)-**10c**

**(*S*)-2-fluoro-1-(4-fluorophenyl)propan-1-one (10c)**

(*S*)-**10c** was prepared according to General Procedure 10 employing 0.4 mmol of *rac*-**9c** (92 mg), the reaction was stirred at 5 °C at 1200 rpm for 96 h. Following purification (FCC eluent: pentane:DCM 70:30) the product was isolated as a colourless oil in 55 % yield, 37 mg, 96:4 e.r. (the product is highly volatile).

**$^1$H NMR** (600 MHz, $CDCl_3$) δ = 8.08 – 8.01 (m, 2H), 7.20 – 7.12 (m, 2H), 5.63 (dq, *J* = 48.6, 6.8 Hz, 1H), 1.66 (dd, *J* = 24.1, 6.8 Hz, 3H); **$^{19}$F NMR** (565 MHz, $CDCl_3$) δ = -103.6, -180.2; **$^{13}$C NMR** (151 MHz, $CDCl_3$) δ = 194.4 (d, $J_{C\text{-}F}$ = 20.0 Hz), 165.0 (d, $J_{C\text{-}F}$ = 256.1 Hz), 130.8 (dd, $J_{C\text{-}F}$ = 9.4, 4.6 Hz), 129.4 (dd, $J_{C\text{-}F}$ = 2.7, 1.5 Hz), 114.9 (d, $J_{C\text{-}F}$ = 22.0 Hz), 89.6 (d, $J_{C\text{-}F}$ = 180.3 Hz), 17.2 (d, $J_{C\text{-}F}$ = 22.6 Hz); **HRMS** ($ESI^+$) m/z calculated for $C_9H_9F_2O$ $(M+H)^+$ 171.0616, found 171.0617; **IR** (neat) ν = 3380, 2981, 2902, 1699, 1600, 1508, 1450, 1413, 1381, 1381, 1302, 1231, 1161, 1130, 1082, 1037, 974, 889, 847, 818, 767, 671 $cm^{-1}$; **$[\alpha]^{D}_{25\,°C}$** = -3.2 (c = 0.2, $CHCl_3$); **HPLC separation:** DAICEL CHIRALPAK® ID-3, Heptane: iPOH = 99.2:0.8, 1 mL/min; $t_1$ = 4.1 (major), $t_2$= 4.6 min (minor).

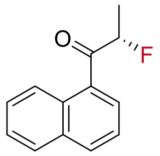

(*S*)-**10d**

**(*S*)-2-fluoro-1-(naphthalen-1-yl)propan-1-one (10d)**

(*S*)-**10d** was prepared according to General Procedure 10 employing 0.4 mmol of *rac*-**9d** (105 mg), the reaction was stirred at 25 °C at 1200 rpm for 96 h Following purification (FCC eluent: pentane:DCM 80:20) the product was isolated as a colourless oil in 96 % yield, 77 mg, 98:2 e.r.

**$^1$H NMR** (600 MHz, $CDCl_3$) δ = 8.48 – 8.43 (m, 1H), 8.03 (dd, *J* = 8.2, 1.2 Hz, 1H), 7.90 (ddd, *J* = 8.1, 1.4, 0.7 Hz, 1H), 7.84 (dt, *J* = 7.2, 1.3 Hz, 1H), 7.61 (ddd, *J* = 8.5, 6.8, 1.5 Hz, 1H), 7.56 (ddd, *J* = 8.1, 6.8, 1.2 Hz, 1H), 7.52 (dd, *J* = 8.2, 7.2 Hz, 1H), 5.77 (dq, *J* = 48.9, 6.9 Hz, 1H), 1.67 (dd, *J* = 23.5, 6.9 Hz, 3H); **$^{19}$F NMR** (565 MHz, $CDCl_3$) δ = - 179.8; **$^{13}$C NMR** (151 MHz, $CDCl_3$) δ = 200.9 (d, $J_{C\text{-}F}$ = 20.0 Hz), 134.0, 133.2, 132.5, 130.5, 128.6, 128.2, 127.9 (d, $J_{C\text{-}F}$ = 4.4 Hz), 126.7, 125.3, 124.2, 90.7 (d, $J_{C\text{-}F}$ = 182.7 Hz), 18.2 (d, $J_{C\text{-}F}$ = 22.6 Hz); **HRMS** ($ESI^+$) m/z calculated for $C_{13}H_{12}FO$ $(M+H)^+$ 203.0867, found 203.0868; **IR** (neat) ν = 2981, 2899, 1699, 1594, 1574, 1509, 1440, 1375, 1263, 1241, 1184, 1140, 1085, 1067, 1031, 942, 889, 805, 780, 760, 647 $cm^{-1}$; **$[\alpha]^{D}_{25\,°C}$** = +43.2 (c = 0.5, $CHCl_3$); **HPLC separation:** DAICEL CHIRALPAK® IC-3, Heptane: iPOH = 99.5:0.5, 1 mL/min; $t_1$ = 15.4 (major), $t_2$= 15.8 min (minor).

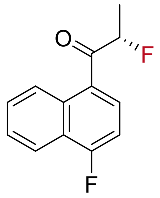

(*S*)-**10e**

**(*R*)-2-fluoro-1-(4-fluoronaphthalen-1-yl)propan-1-one (10e)**

(*S*)-**10e** was prepared according to General Procedure 10 employing 0.4 mmol of *rac*-**9e** (112 mg), the reaction was stirred at 25 °C at 1200 rpm for 96 h. Following

purification (FCC eluent: pentane:DCM 80:20) the product was isolated as a colourless oil in 97 % yield, 85 mg, 96:4 e.r.

**$^1$H NMR** (600 MHz, $CDCl_3$) δ = 8.59 (ddt, *J* = 8.7, 1.9, 0.9 Hz, 1H), 8.20 – 8.15 (m, 1H), 7.90 (ddd, *J* = 8.1, 5.4, 1.4 Hz, 1H), 7.68 (ddd, *J* = 8.5, 6.9, 1.4 Hz, 1H), 7.63 (ddd, *J* = 8.1, 6.8, 1.1 Hz, 1H), 7.18 (dd, *J* = 9.7, 8.1 Hz, 1H), 5.74 (dq, *J* = 48.8, 6.9 Hz, 1H), 1.68 (dd, *J* = 23.6, 6.8 Hz, 3H); **$^{19}$F NMR** (565 MHz, $CDCl_3$) δ = -178.7, -113.3; **$^{13}$C NMR** (151 MHz, $CDCl_3$) δ = 199.4 (d, $J_{C-F}$ = 20.3 Hz), 161.5 (d, $J_{C-F}$ = 260.8 Hz), 132.8 (d, $J_{C-F}$ = 5.5 Hz), 129.5 (dd, $J_{C-F}$ = 10.1, 5.7 Hz), 129.3, 128.4 (d, $J_{C-F}$ = 4.4 Hz), 127.1 (d, $J_{C-F}$ = 2.1 Hz), 125.6 (d, $J_{C-F}$ = 2.6 Hz), 124.3 (d, $J_{C-F}$ = 15.6 Hz), 120.9 (d, $J_{C-F}$ = 6.4 Hz), 108.2 (d, $J_{C-F}$ = 21.0 Hz), 90.7 (d, $J_{C-F}$ = 182.7 Hz), 18.3 (d, $J_{C-F}$ = 22.6 Hz); **HRMS** ($ESI^+$) m/z calculated for $C_{13}H_{11}F_2O$ $(M+H)^+$ 221.0773, found 221.0772; **IR** (neat) ν = 2981, 1692, 1630, 1602, 1576, 1511, 1488, 1465, 1427, 1376, 1263, 1230, 1178, 1133, 1073, 1053, 1032, 969, 917, 864, 832, 793, 768, 747, 967, 644, 616 $cm^{-1}$; **$[\alpha]^D_{25\,°C}$** = +27.8 (c = 0.5, $CHCl_3$); **HPLC separation:** DAICEL CHIRALPAK® IC-3, Heptane: iPOH = 99.5:0.5, 1 mL/min; $t_1$ =8.8 (major), $t_2$= 9.4 min (minor).

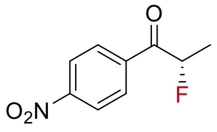

(*S*)-**10f**

**(*S*)-2-fluoro-1-(4-nitrophenyl)propan-1-one (10f)**

(*S*)-**10f** was prepared according to General Procedure 10 employing 0.4 mmol of *rac*-**9f** (103 mg), the reaction was stirred at 5 °C at 1200 rpm for 96 h. Following purification (FCC eluent: pentane:DCM 70:30) the product was isolated as a yellow solid in 91 % yield, 72 mg, 91.5:8.5 e.r.

**$^1$H NMR** (600 MHz, $CDCl_3$) δ = 8.36 – 8.31 (m, 2H), 8.19 – 8.14 (m, 2H), 5.64 (dq, *J* = 48.3, 6.8 Hz, 1H), 1.70 (dd, *J* = 24.1, 6.8 Hz, 3H); **$^{19}$F NMR** (565 MHz, $CDCl_3$) δ = - 181.7; **$^{13}$C NMR** (151 MHz, $CDCl_3$) δ = 196.0 (d, $J_{C-F}$ = 21.6 Hz), 150.6, 138.7 (d, $J_{C-F}$ = 2.2 Hz), 130.3 (d, $J_{C-F}$ = 5.0 Hz), 123.8, 91.0 (d, $J_{C-F}$ = 180.9 Hz), 17.9 (d, $J_{C-F}$ = 22.5 Hz); **HRMS** ($ESI^+$) m/z calculated for $C_9H_8FNO_3$ $(M+H)^+$ 198.0561, found 198.0141; **IR** (neat) ν = 2980, 2887, 1703, 1601, 1526, 1509, 1450, 1413, 1344, 1301, 1263, 1240, 1160, 1128, 1087, 1041, 974, 938, 889, 871, 851, 810, 742, 712, 669 $cm^{-1}$; **mp** 44-45 ˚C; **$[\alpha]^D_{25\,°C}$** = -15.2 (c = 0.5, $CHCl_3$); **HPLC separation:** DAICEL CHIRALPAK® ID-3, Heptane: iPOH = 99.2:0.8, 1 mL/min; $t_1$ = 13.5 (major), $t_2$= 20.0 min (minor).

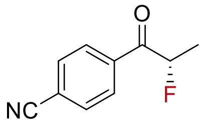

(*S*)-**10g**

**(*S*)-4-(2-fluoropropanoyl)benzonitrile (10g)**

(*S*)-**10g** was prepared according to General Procedure 10 employing 0.4 mmol of *rac*-**9g** (95 mg), the reaction was stirred at 5 °C at 1200 rpm for 96 h. Following purification (FCC eluent: pentane:DCM 70:30) the product was isolated as a white solid in 86 % yield, 61 mg, 93:7 e.r.

**$^1$H NMR** (600 MHz, $CDCl_3$) δ = 8.11 – 8.05 (m, 2H), 7.81 – 7.75 (m, 2H), 5.63 (dq, *J* = 48.3, 6.8 Hz, 1H), 1.67 (dd, *J* = 24.1, 6.8 Hz, 3H); **$^{19}$F NMR** (565 MHz, $CDCl_3$) δ = -180.5; **$^{13}$C NMR** (151 MHz, $CDCl_3$) δ =

196.1 (d, $J_{C-F}$ = 21.4 Hz), 137.2 (d, $J_{C-F}$ = 2.2 Hz), 132.5, 129.6 (d, $J_{C-F}$ = 4.6 Hz), 117.3 (d, $J_{C-F}$ = 123.3 Hz), 90.9 (d, $J_{C-F}$ = 180.9 Hz), 17.9 (d, $J_{C-F}$ = 22.3 Hz); **HRMS** ($ESI^+$) m/z calculated for $C_{10}H_9FNO$ $(M+H)^+$ 178.0663, found 178.1073; **IR** (neat) ν = 2980 1685, 1599, 1508, 1447, 1411, 1372, 1321, 1292, 1258, 1236, 1192, 1179, 1157, 1132, 1097, 1022, 973, 943, 862, 844, 819, 771, 752, 689, 657 $cm^{-1}$; **mp** 55-56 ˚C; **$[\alpha]^D_{25\,°C}$** = -2.0 (c = 0.2, $CHCl_3$); **HPLC separation:** DAICEL CHIRALPAK® ID-3, Heptane: iPOH = 99.2:0.8, 1 mL/min; $t_1$ = 16.2 (major), $t_2$= 19.5 min (minor).

**(*S*)-2-fluoro-1-(4-(trifluoromethyl)phenyl)propan-1-one (10h)**

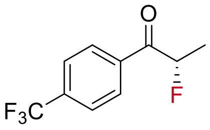

(*S*)-**10h**

(*S*)-**10h** was prepared according to General Procedure 10 employing 0.4 mmol of *rac*-**9h** (112 mg), the reaction was stirred at 5 °C at 1200 rpm for 96 h. Following purification (FCC eluent: pentane:DCM 70:30) the product was isolated as a white solid in 78 % yield, 69 mg, 92:8 e.r.

**$^1$H NMR** (600 MHz, $CDCl_3$) δ = 8.10 (m, 2H), 7.79 – 7.72 (m, 2H), 5.66 (dq, *J* = 48.4, 6.8 Hz, 1H), 1.68 (dd, *J* = 24.1, 6.8 Hz, 3H); **$^{19}$F NMR** (565 MHz, $CDCl_3$) δ = -63.3, -180.7; **$^{13}$C NMR** (151 MHz, $CDCl_3$) δ = 196.3 (d, $J_{C-F}$ = 20.9 Hz), 136.8, 134.9 (q, $J_{C-F}$ = 32.8 Hz), 129.5 (d, $J_{C-F}$ = 4.4 Hz), 125.7 (q, $J_{C-F}$ = 3.9 Hz), 123.5 (q, $J_{C-F}$ = 272.7 Hz), 90.8 (d, $J_{C-F}$ = 180.6 Hz), 18.0 (d, $J_{C-F}$ = 22.4 Hz); **HRMS** ($ESI^+$) m/z calculated for $C_{10}H_9F_4O$ $(M+H)^+$ 221.0584, found 221.0836; **IR** (neat) ν = 2981, 2918, 1702, 1601, 1508, 1449, 1413, 1381, 1327, 1263, 1233, 1162, 1132, 1068, 1038, 1018, 975, 890, 853, 818, 766, 694, 672 $cm^{-1}$; **mp** 41-42 ˚C; **$[\alpha]^D_{25\,°C}$** = -4.6 (c = 0.5, $CHCl_3$); **HPLC separation:** DAICEL CHIRALPAK® ID-3, Heptane: iPOH = 99.5:0.5, 1 mL/min; $t_1$ = 3.7 (major), $t_2$ = 4.1 min (minor).

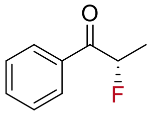

(*S*)-**10i**

**(*S*)-2-fluoro-1-phenylpropan-1-one (10i)**

(*S*)-**10i** was prepared according to General Procedure 10 employing 0.4 mmol of *rac*-**9i** (85 mg), the reaction was stirred at 25 °C at 1200 rpm for 96 h. Following purification (FCC eluent: pentane:DCM 80:20) the product was isolated as a colourless oil in 91 % yield, 55 mg, 87:13 e.r. (the product is highly volatile).

**$^1$H NMR** (600 MHz, $CDCl_3$) δ = 8.00 – 7.95 (m, 2H), 7.62 – 7.58 (m, 1H), 7.52 – 7.46 (m, 2H), 5.71 (dq, *J* = 48.6, 6.8 Hz, 1H), 1.67 (dd, *J* = 24.0, 6.8 Hz, 3H); **$^{19}$F NMR** (565 MHz, $CDCl_3$) δ = -181.4; **$^{13}$C NMR** (151 MHz, $CDCl_3$) δ =196.9 (d, $J_{C-F}$ = 19.4 Hz), 137.8, 129.0 (d, $J_{C-F}$ = 3.8 Hz), 128.7, 90.3 (d, $J_{C-F}$ = 180.2 Hz), 18.4 (d, $J_{C-F}$ =22.6 Hz); **HRMS** ($ESI^+$) m/z calculated for $C_9H_9FO$ $(M+H)^+$ 153.0710, found 153.0711; **IR** (neat) ν = 2981, 2889, 1702, 1598, 1579, 1451, 1380, 1318, 1281, 1234, 1132, 1085, 1036, 1002, 951, 887, 793, 742, 699, 659 $cm^{-1}$; **$[\alpha]^D_{25\,°C}$** = +3.2 (c = 0.5, $CHCl_3$); **HPLC separation:** DAICEL CHIRALPAK® ID-3, Heptane: iPOH = 99.2:0.8, 1 mL/min; $t_1$ = 4.7 (major), $t_2$= 5.4 min (minor).

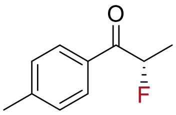

(*S*)-**10j**

**(*S*)-2-fluoro-1-(p-tolyl)propan-1-one (10j)**

(*S*)-**10j** was prepared according to General Procedure 10 employing 0.4 mmol of *rac*-**9j** (91 mg), the reaction was stirred at 25 °C at 1200 rpm for 96 h. Following purification (FCC eluent: pentane:DCM 80:20) the product was isolated as a colourless oil in 83 % yield, 55 mg, 92:8 e.r.

**$^1$H NMR** (600 MHz, $CDCl_3$) δ = 7.88 (d, *J* = 8.1 Hz, 2H), 7.31 – 7.26 (m, 2H), 5.69 (dq, *J* = 48.7, 6.8 Hz, 1H), 2.43 (s, 3H), 1.66 (dd, *J* = 24.0, 6.8 Hz, 3H); **$^{19}$F NMR** (565 MHz, $CDCl_3$) δ = -181.3; **$^{13}$C NMR** (151 MHz, $CDCl_3$) δ = 196.6 (d, $J_{C-F}$ = 19.3 Hz), 144.9, 131.6, 129.6, 129.2 (d, $J_{C-F}$ = 3.9 Hz), 90.4 (d, $J_{C-F}$ = 179.9 Hz), 21.9, 18.6 (d, $J_{C-F}$ = 23.1 Hz); **HRMS** (ESI$^+$) m/z calculated for $C_{10}H_{12}FO$ $(M+H)^+$ 167.0867, found 167.0867; **IR** (neat) v = 2981, 1698, 1608, 1571, 1447, 1411, 1379, 1282, 1262, 1239, 1212, 1887, 1132, 1085, 1037, 971, 887, 826, 760, 645 cm$^{-1}$; **$[\alpha]^D_{25\,°C}$** = +2.1 (c = 0.5, $CHCl_3$); **HPLC separation:** DAICEL CHIRALPAK® ID-3, Heptane: iPOH = 99.2:0.8, 1 mL/min; $t_1$ = 5.1 (major), $t_2$= 5.8 min (minor).

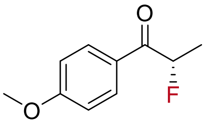

(*S*)-**10k**

**(*S*)-2-fluoro-1-(4-methoxyphenyl)propan-1-one (10k)**

(*S*)-**10k** was prepared according to General Procedure 10 employing 0.4 mmol of *rac*-**9k** (97 mg), the reaction was stirred at 25 °C at 1200 rpm for 96 h. Following purification (FCC eluent: pentane:DCM 70:30) the product was isolated as a colourless oil in 90 % yield, 65 mg, 92:8 e.r.

**$^1$H NMR** (600 MHz, $CDCl_3$) δ = 8.03 – 7.95 (m, 2H), 7.01 – 6.90 (m, 2H), 5.65 (dq, *J* = 48.8, 6.8 Hz, 1H), 3.88 (s, 3H), 1.65 (dd, *J* = 24.2, 6.8 Hz, 3H). **$^{19}$F NMR** (565 MHz, $CDCl_3$) δ = -180.4; **$^{13}$C NMR** (151 MHz, $CDCl_3$) δ = 195.3 (d, $J_{C-F}$ = 19.6 Hz), 164.0, 131.4 (d, $J_{C-F}$ = 4.0 Hz), 126.9, 114.0, 90.4 (d, $J_{C-F}$ = 179.6 Hz), 55.5, 18.5 (d, $J_{C-F}$ = 22.7 Hz); **HRMS** (ESI$^+$) m/z calculated for $C_{10}H_{12}FO_2$ $(M+H)^+$ 183.0816, found 183.0816; **IR** (neat) v = 2981, 2907, 1689, 1601, 1574, 1512, 1460, 1422, 1379, 1313, 1264, 1242, 1177, 1130, 1083, 1030, 972, 887, 844, 805, 775, 762 cm$^{-1}$; **$[\alpha]^D_{25\,°C}$** = +1.4 (c = 0.5, $CHCl_3$); **HPLC separation:** DAICEL CHIRALPAK® ID-3, Heptane: iPOH = 99.5:0.5, 0.8 mL/min; $t_1$ = 21.2 (major), $t_2$= 23.7 (minor).

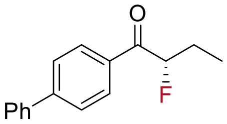

(*S*)-**10l**

**(*S*)-1-([1,1'-biphenyl]-4-yl)-2-fluorobutan-1-one (10l)**

(*S*)-**10l** was prepared according to General Procedure 10 employing 0.4 mmol of *rac*-**9l** (121 mg), the reaction was stirred at 40 °C at 1200 rpm for 96 h. Following purification (FCC eluent: pentane:DCM 80:20) the product was isolated as a white solid in 83 % yield, 80 mg, 92:8 e.r.

**$^1$H NMR** (600 MHz, $CDCl_3$) δ = 8.08 – 8.04 (m, 2H), 7.73 – 7.68 (m, 2H), 7.65 – 7.62 (m, 2H), 7.51 – 7.45 (m, 2H), 7.45 – 7.39 (m, 1H), 5.54 (ddd, *J* = 49.3, 7.8, 4.5 Hz, 1H), 2.16 – 1.96 (m, 2H), 1.11 (t, *J* = 7.4 Hz, 3H); **$^{19}$F NMR** (565 MHz, $CDCl_3$) δ = -190.5; **$^{13}$C NMR** (151 MHz, $CDCl_3$) δ = 196.4 (d, $J_{C-F}$ = 19.7 Hz), 146.4, 139.7, 133.1 (d, $J_{C-F}$ = 1.4 Hz), 129.5 (d, $J_{C-F}$ = 3.9 Hz), 129.0, 128.4, 127.3 (d, $J_{C-F}$ = 6.6 Hz), 95.0 (d, $J_{C-F}$ = 183.6 Hz), 26.2 (d, $J_{C-F}$ = 21.5 Hz), 9.1 (d, $J_{C-F}$ = 4.5 Hz); **HRMS** (ESI$^+$) m/z calculated for $C_{16}H_{16}FO$

$(M+H)^+$ 243.1180, found 243.1180; **IR** (neat) ν = 2980, 2885, 1954, 1690, 1604, 1561, 1488, 1459, 1407, 1383, 1264, 1229, 1208, 1131, 1095, 1052, 1026, 1006, 968, 896, 842, 808, 768, 745, 692, 643 $cm^{-1}$; **mp** 51-52 ˚C; **[α]$^D$ $_{25\ °C}$** = +17.0 (c = 0.5, $CHCl_3$); **HPLC separation:** DAICEL CHIRALPAK® ID-3, Heptane: iPOH = 99.2:0.8, 1 mL/min; $t_1$ = 7.5 (major), $t_2$= 8.5 min (minor).

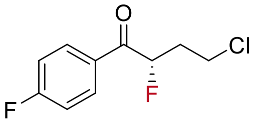

(*S*)-**10m**

**(*S*)-4-chloro-2-fluoro-1-(4-fluorophenyl)butan-1-one (10m)**

(*S*)-**10m** was prepared according to General Procedure 10 employing 0.4 mmol of *rac*-**9m** (112 mg), the reaction was stirred at 40 °C at 1200 rpm for 96 h. Following purification (FCC eluent: pentane:DCM 80:20) the product was isolated as a white solid in 78 % yield, 68 mg, 84:16 e.r.

**$^1$H NMR** (600 MHz, $CDCl_3$) δ = 8.04 (m, 2H), 7.20 – 7.15 (m, 2H), 5.90 – 5.78 (m, 1H), 3.84 – 3.74 (m, 2H), 2.46 – 2.34 (m, 2H); **$^{19}$F NMR** (565 MHz, $CDCl_3$) δ = -182.8, -191.9; **$^{13}$C NMR** (151 MHz, $CDCl_3$) δ = 193.9 (d, $J_{C\text{-}F}$ = 18.8 Hz), 166.2 (d, $J_{C\text{-}F}$ = 256.9 Hz), 131.8 (dd, $J_{C\text{-}F}$ = 9.5, 4.1 Hz), 130.4, 116.1 (d, $J_{C\text{-}F}$ = 22.0 Hz), 89.9 (d, $J_{C\text{-}F}$ = 183.3 Hz), 40.0 (d, $J_{C\text{-}F}$ = 3.8 Hz), 35.1 (d, $J_{C\text{-}F}$ = 21.4 Hz); **HRMS** ($ESI^+$) m/z calculated for $C_{10}H_{10}ClF_2O$ $(M+H)^+$ 219.0383, found 219.0382; **IR** (neat) ν = 2980, 2890, 1688, 1601, 1509, 1459, 1415, 1382, 1312, 1264, 1243, 1177, 1159, 1131, 1086, 1032, 972, 845, 809, 653 $cm^{-1}$; **mp** 40-42 ˚C; **[α]$^D$ $_{25\ °C}$** = +5.8 (c = 0.5, $CHCl_3$); **HPLC separation:** DAICEL CHIRALPAK® ID-3, Heptane: iPOH = 99.2:0.8, 1 mL/min; $t_1$ = 5.5 (major), $t_2$= 7.6 min (minor).

## Challenging Substrates

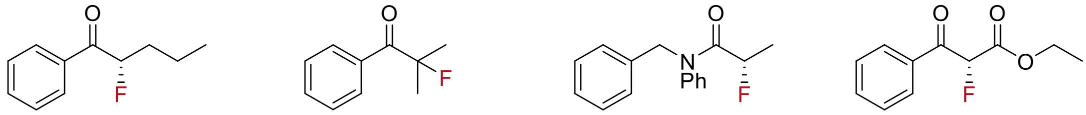

| 10n | 10o | 10p | 10q |
|---|---|---|---|
| 11%[a] | 0%[b] | 0%[b] | 0%[b] |
| 84:16 e.r. | | | |

**Supplementary Figure 4**: Challenging substates under S-HBPTC. General conditions: Substrate (0.05 mmol), (*S*)-**3k** (10 mol%), $Et_4N^+ I^-$ (10 mol%) and KF (5 equiv.) in 200 µL of MeCN stirred at 1200 rpm at 25 °C for 72 h. [a]Determined by $^{19}$F NMR using 4-fluoroanisole as internal standard [b]Reactions performed at 60 °C. Bromide substrates were commercially available **9n** (CAS: 49851-31-2), **9o** (CAS: 10409-54-8), **9p** (851073-30-8)**, 9q** (CAS: 55919-47-6).

## Non-Linear Effect Study

The non-linear effect study was performed on model substrate (*rac*-**1a**) with scalemic mixtures of catalyst (*S*)-**3h** (10 mol%), $Ph_4P^+ I^-$ (10 mol%), KF (2.5 equiv.) in *p*-xylene at 25 °C. Under the standard conditions (*S*)-**3h** affords (*R*)-**2a** in 91:9 e.r. No background reaction is observed under these conditions and the results shown are mean values of two sets of experiments.

Supplementary Table 8: Non-linear effect study

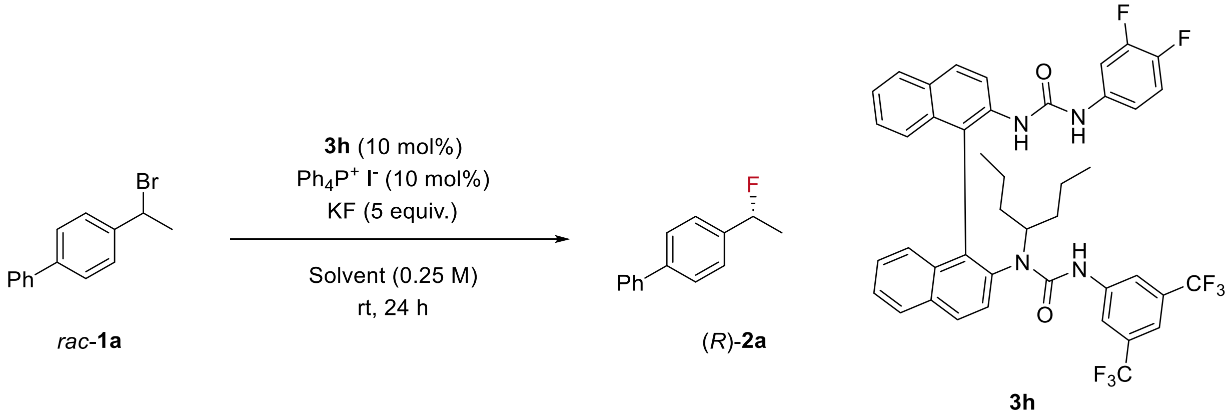


| Entry | %ee catalyst **3h**[a] | %ee **2a** (product) |
|---|---|---|
| **1** | 0 | **0** |
| **2** | 21.5 | **18.2** |
| **3** | 39.7 | **38.3** |
| **4** | 60.1 | **58.7** |
| **5** | 79.6 | **70.5** |
| **6** | 100 | **82.0** |

[a]Scalemic mixtures of catalyst **3h** were prepared by mixing (*S*)-**3h** with (*rac*)-**3h**, dissolving in $CH_2Cl_2$ and evaporating to dryness. The enantiopurity of each catalyst batch was determined by chiral HPLC. **HPLC separation:** DAICEL CHIRALPAK® IA-3, Heptane: EtOH = 97.8:2.2, 0.6 mL/min; $t_1$ = 6.2, $t_2$ = 7.5 min

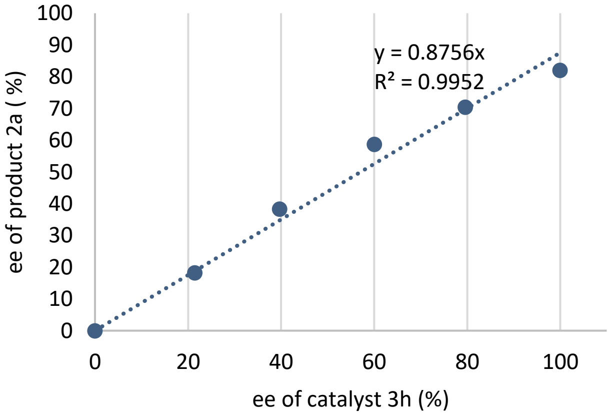


**Supplementary Figure 5**: Non-linear effect study

## NMR Investigations

(*S*)-**3h** Assignment and copies of NMR spectra

*Sample preparation*: (*S*)-**3h** (0.125 mmol) was weighted into an NMR tube and dissolved in toluene-*d*8.

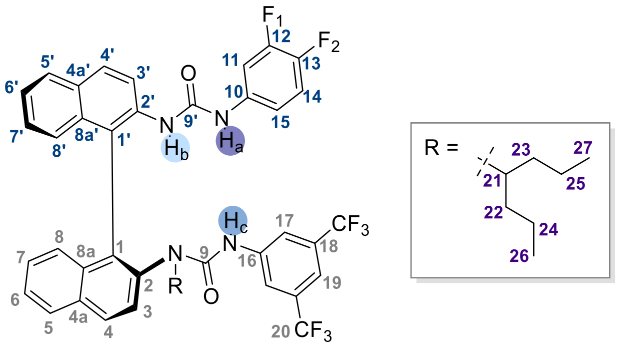

Supplementary Table 9: Assignment of (*S*)-**3h**

| **$^{1}$H** | **δ [ppm]** | **Multiplicity $^{n}J$ [Hz]** | **$^{13}$C** | **δ [ppm]** | **Multiplicity $^{n}J_{CF}$ [Hz]** |
|---|---|---|---|---|---|
| NH(b) | 7.04 | very br s | 9 | 155.5 | br s |
| NH(c) | 6.71 | br s | 9′ | 151.9 | s |
| NH(a) | 6.41 | br s | 13 | 150.2 | dd $^{2}J_{CF}$ = 245.5; $^{3}J_{CF}$ =13.2 |
| 3′ | 8.68 | br d, $^{3}J$ = 8.5 | 12 | 146.4 | dd $^{2}J_{CF}$ = 242.6; $^{3}J_{CF}$ =12.9 |
| 17 | 7.87 | s | 2 | 142.6 | s |
| 4′ | 7.70 | d, $^{3}J$ = 9.1 | 16 | 140.6 | s |
| 4 | 7.67 | d, $^{3}J$ = 8.9 | 2′ | 136.8 | s |
| 5′ | 7.60 | d, $^{3}J$ = 8.2 | 10 | 135.4 | d, $^{3}J_{CF}$ = 7.8 |
| 5 | 7.54 | d, $^{3}J$ = 8.2 | 8a | 134.3 | s |
| 3 | 7.43 | d, $^{3}J$ = 8.9 | 8a′ | 133.7 | s |
| 19 | 7.41 | s | 4a or 1 | 133.2 | s |
| 8 | 7.14 | d, overlapping | 18 | 132.6 | q, $^{3}J_{CF}$ = 33.4 |
| 6′ or 6 | 7.09 or 7.08 | t, overlapping | 4a or 1 | 131.1 | s |
| 11 | 7.03 | overlapping | 1′ or 4a′ | 130.9 | s |
| 8′ | 6.92 | d, overlapping | 4 | 130.2 | s |
| 7′ or 7 | 6.88 or 6.83 | t, $^{3}J$ = 8.0 | 4′ | 130.0 | s |
| 14 | 6.52 | q, $^{2}J_{HF}$ = 9.1 | 3 | 128.9 | overlapping |
| 15 | 6.42 | overlapping | 5′ | 128.8 | overlapping |
| 21 | 2.94 | m | 3 | 128.4 | overlapping |
| 22, 23 | 1.60 and 1.21 | br s and m | 7′ or 7 | 127.6 | s |
| 24 or 25 | 1.30 and 1.11 | m | 8′ or 8 | 127.3 | s |
| 26 or 27 | 0.64 or 0.37 | t, $^{3}J$ = 7.1 or br s | 6′ or 6 | 127.2 | s |
| **$^{19}$F** | **δ [ppm]** | **Multiplicity $^{n}J$ [Hz]** | 8′ or 8 | 127.1 | s |
| $CF_3$ | -62.8 | s | 20 | 123.7 | q, $^{1}J_{CF}$ = 273.1 |
| $F_1$ | -138.8 | br s | 3′ | 121.6 | s |
| $F_2$ | -145.6 | br s | 17 | 119.7 | s |
| | | | 14 | 116.7 | d, $^{3}J_{CF}$ = 17.8 overlapping |
| | | | 19 | 116.7 | s overlapping |
| | | | 15 | 114.2 | br s |
| | | | 11 | 108.2 | d, $^{3}J_{CF}$ = 17.9 |
| | | | 21 | 64.1 | s |
| | | | 22 or 23 | 36.3 | s |
| | | | 24 or 25 | 21.6 | s |
| | | | 26 or 27 | 14.0 or 14.1 | s |

*overlapping signals masked by solvent peaks

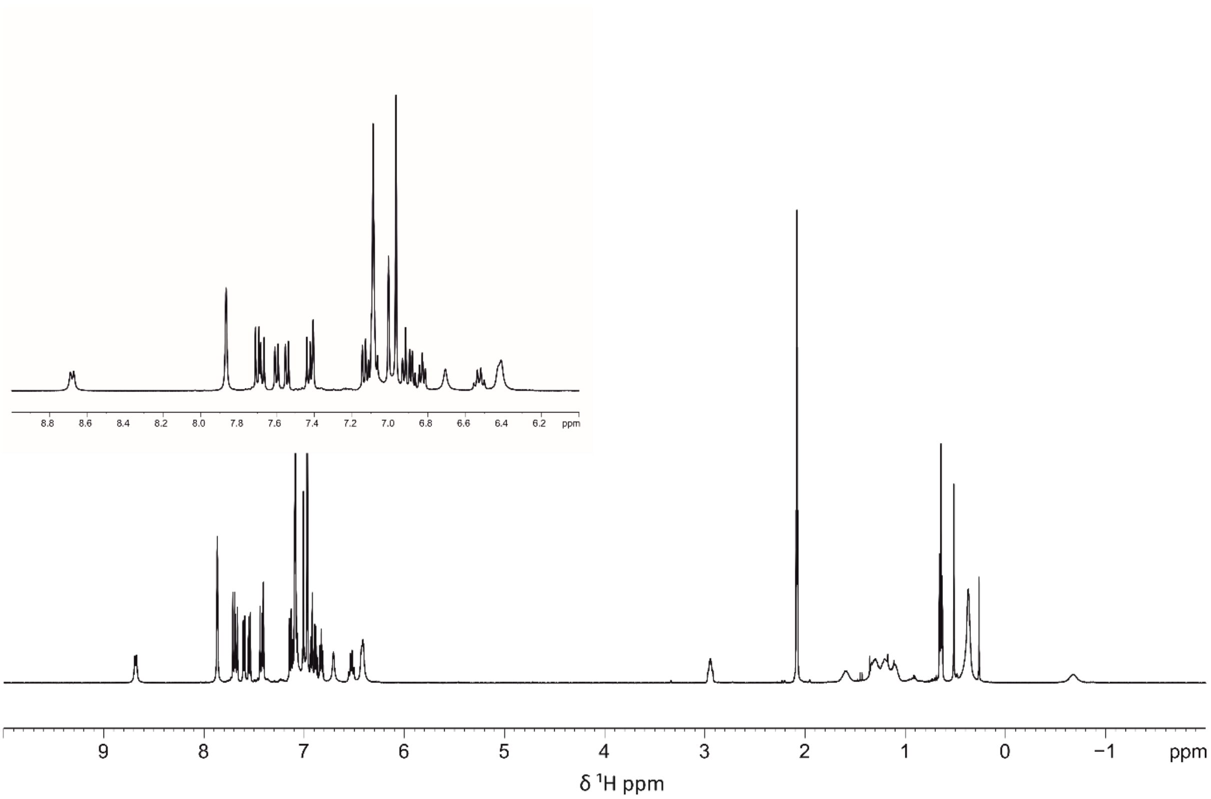


**Supplementary Figure 6**: $^{1}$H NMR of (*S*)-**3h** (500 MHz, Toluene-$d_8$, 25 mM, 298 K).

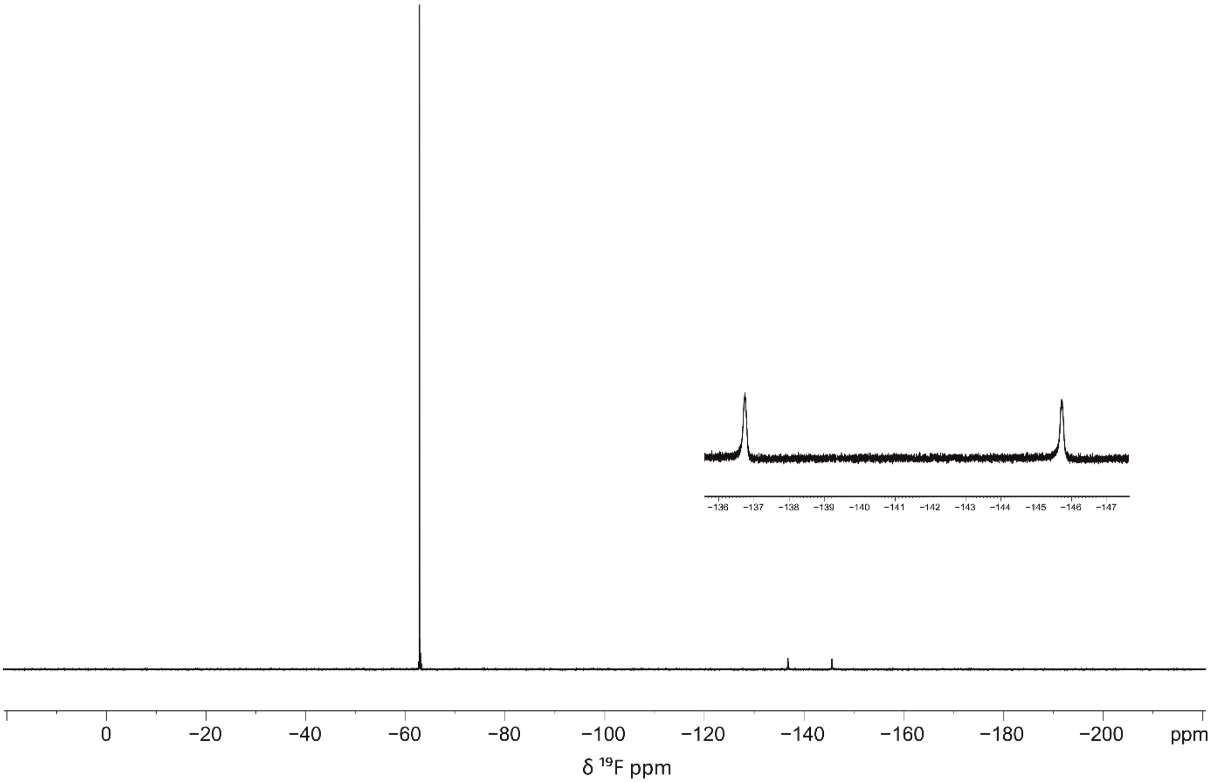


**Supplementary Figure 7**: $^{19}$F NMR of (*S*)-**3h** (471 MHz, Toluene-$d_8$, 25 mM, 298 K).

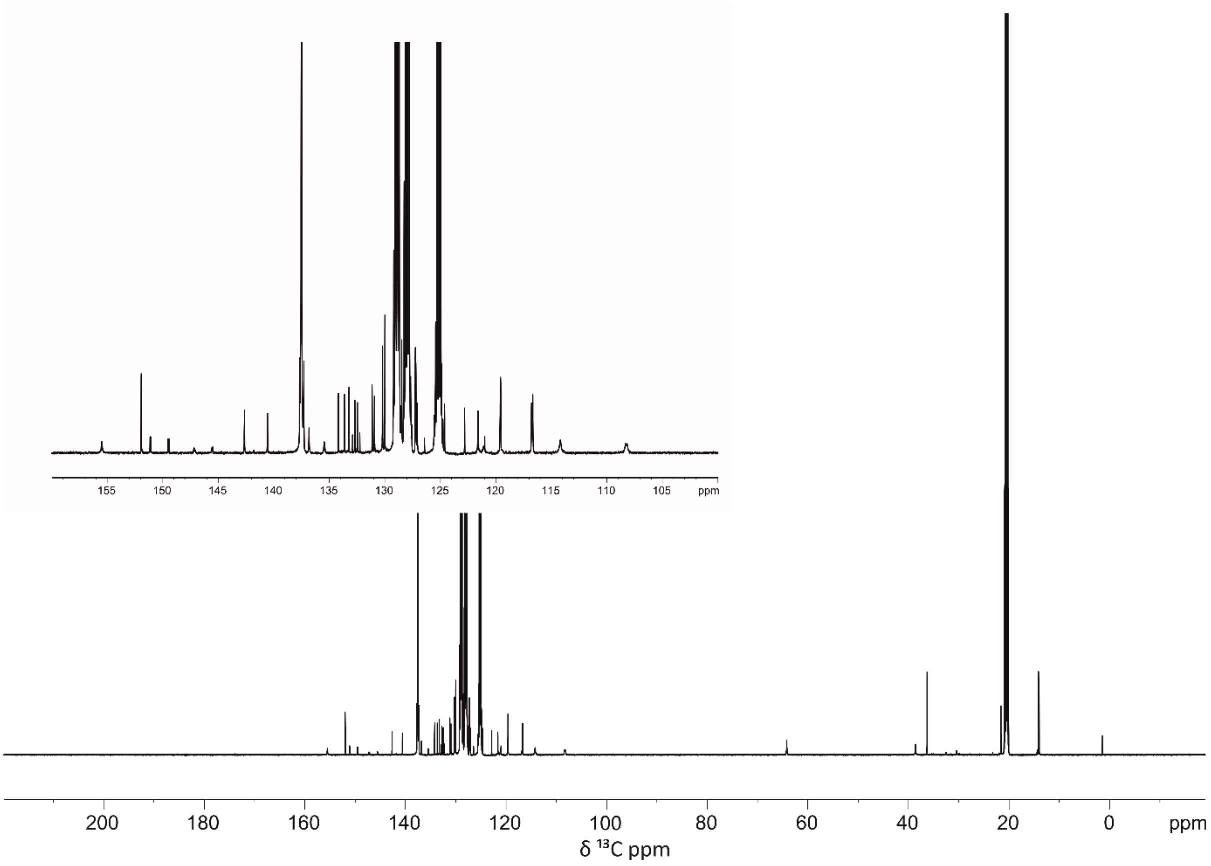


**Supplementary Figure 8**: $^{13}C$ NMR of (*S*)-**3h** (151 MHz, Toluene-$d_8$, 25 mM, 298 K).

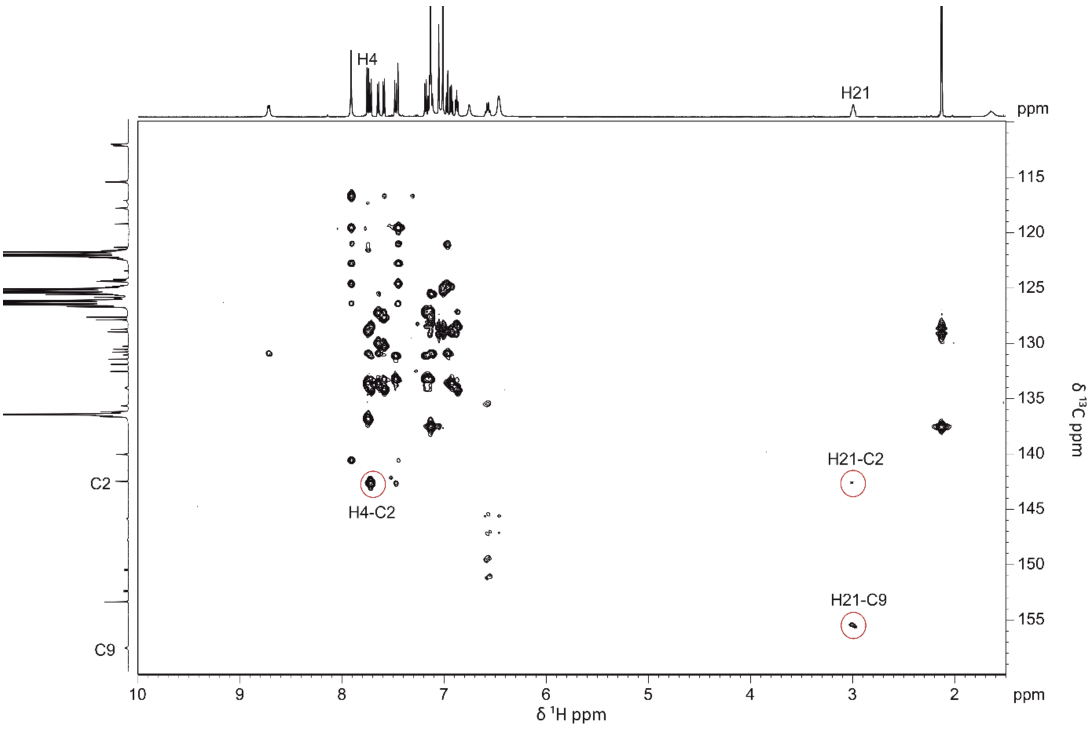


**Supplementary Figure 9**: $^{1}H$-$^{13}C$ HMBC of (*S*)-**3h** (600 MHz, Toluene-$d_8$, 25 mM, 298 K).

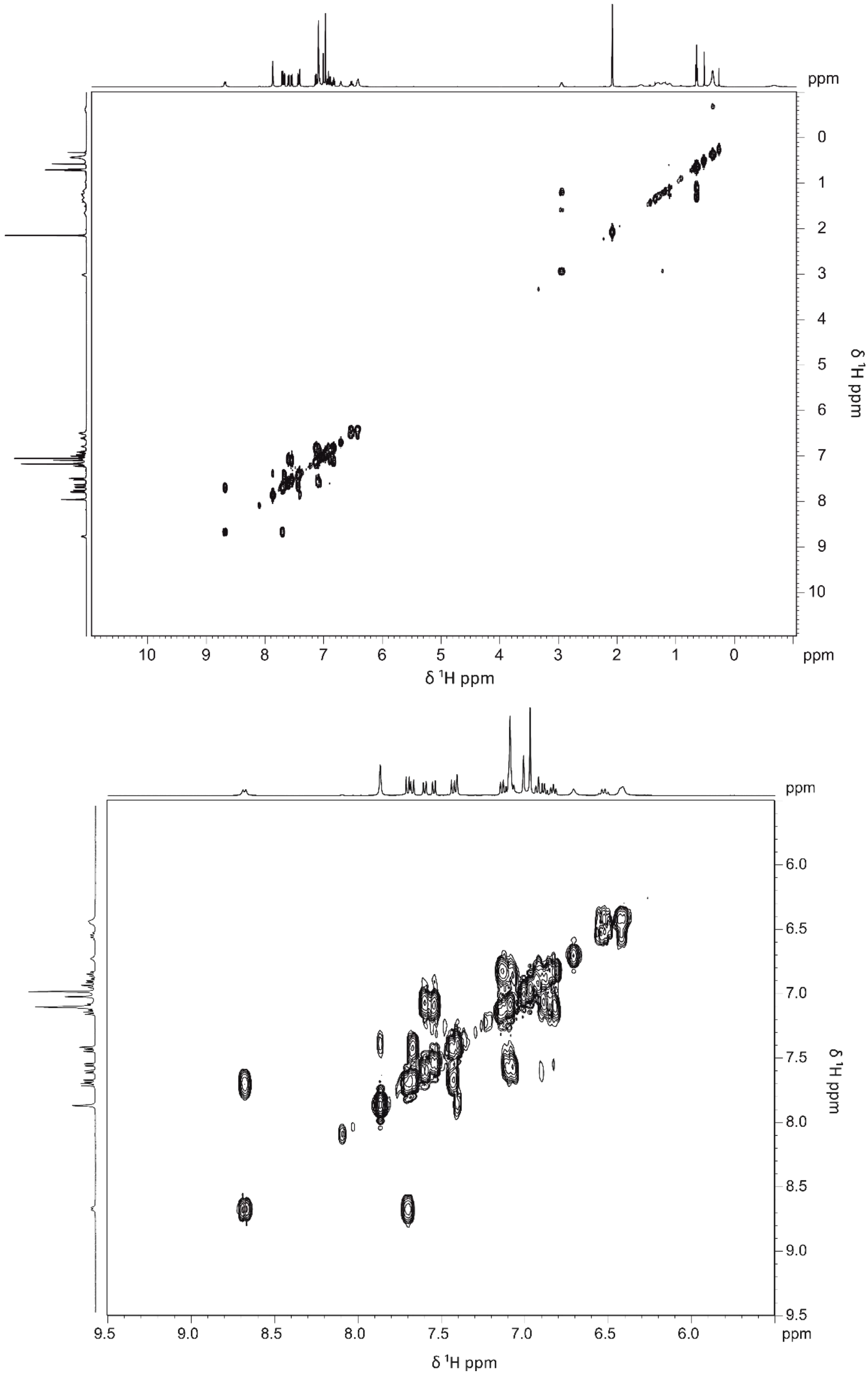


Supplementary Figure 10: ¹H COSY of (*S*)-**3h** (500 MHz, Toluene-$d_8$, 25 mM, 298 K).

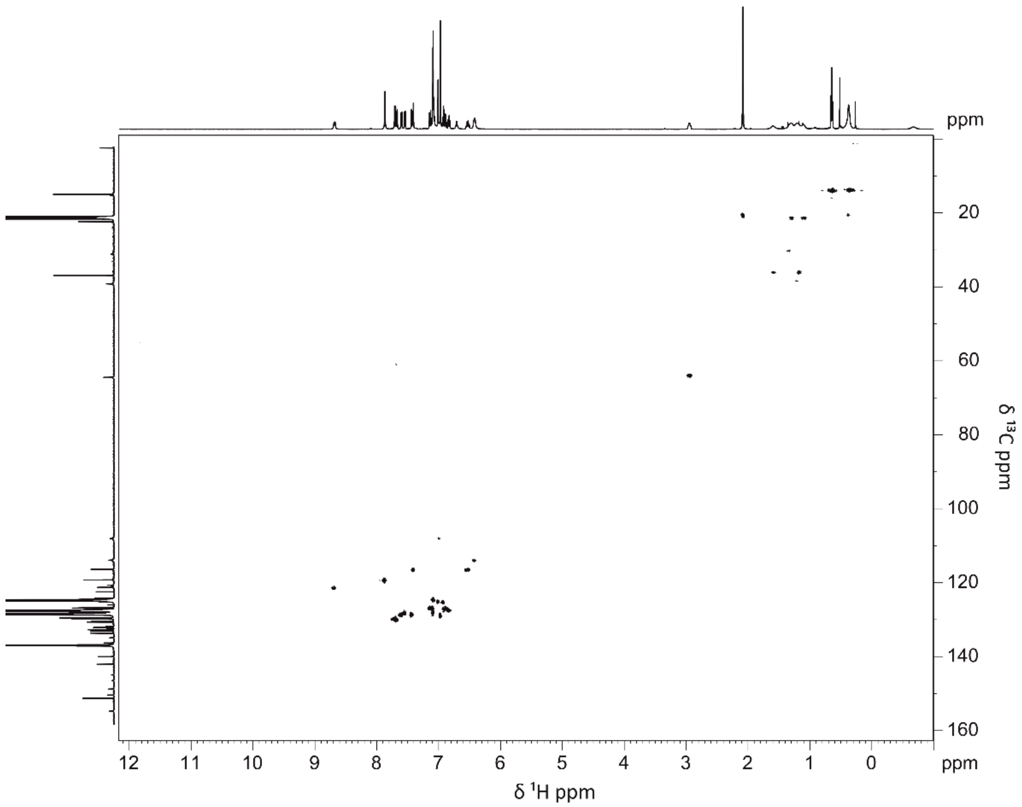


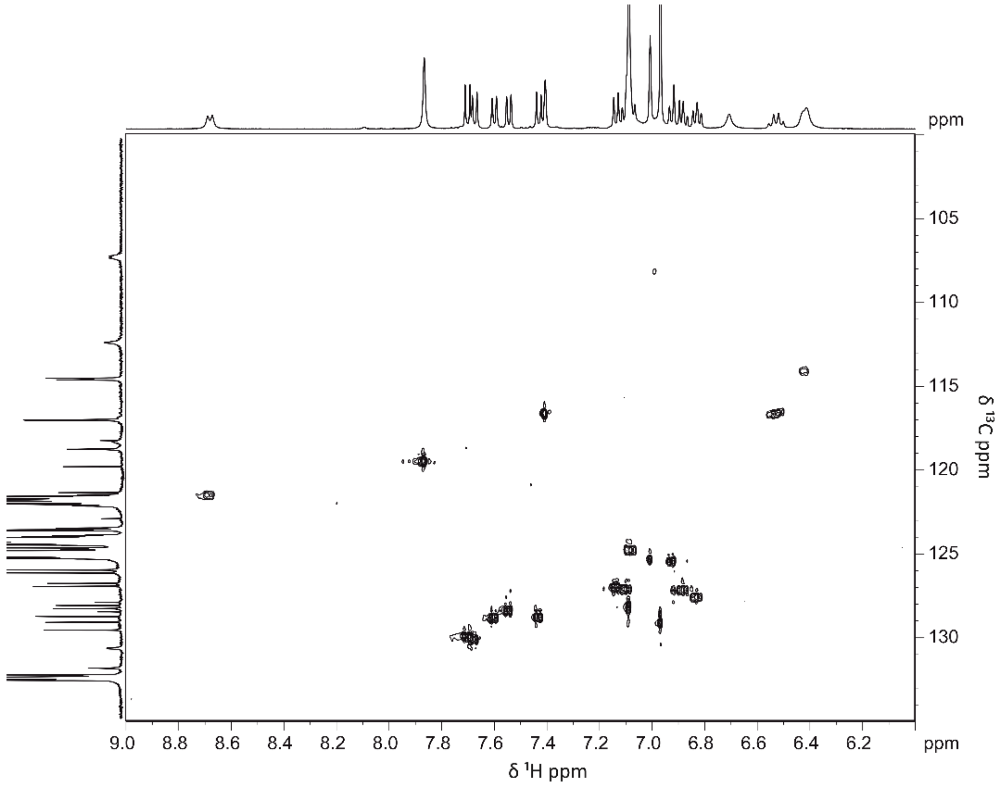


**Supplementary Figure 11**: $^{1}$H-13C HSQC of (*S*)-**3h** (500 MHz, Toluene-$d_8$, 25 mM, 298 K).

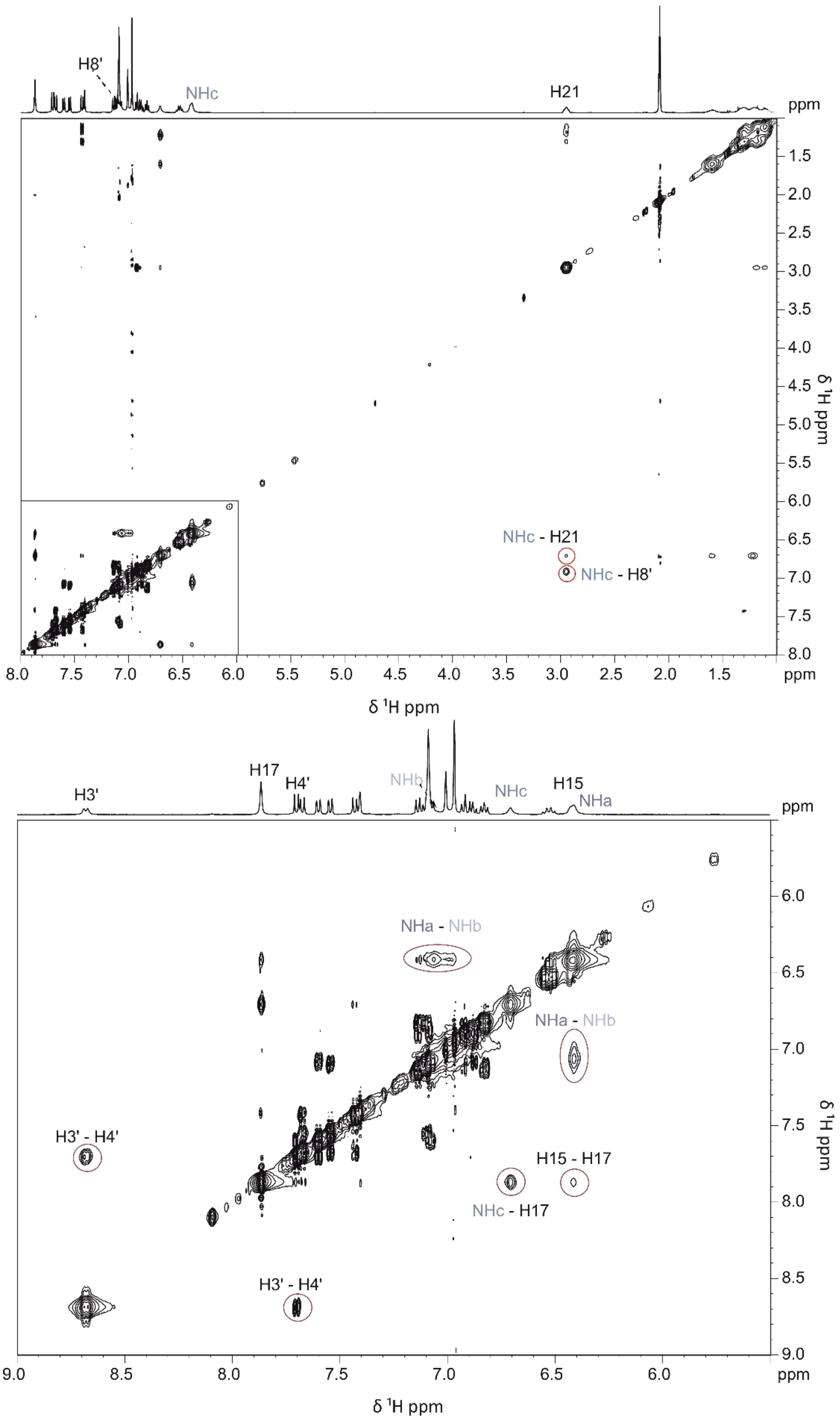


**Supplementary Figure 12**: ¹H NOESY of (*S*)-**3h** (600 MHz, Toluene-$d_8$, 25 mM, 298 K).

(*S*)-**3h**: $BnPh_3P^+$: $F^-$ [UPF] Complex Assignment

*Sample Preparation:* (*S*)-**3h** (0.125 mmol), $BnPh_3P^+$ $BF_4^-$ and KF (2.5 equiv.) was weighted into a J Young Norell 5mm NMR tube and solvent (0.5 mL) was added. The tube was sealed and sonicated for 45 minutes to form the complex. The sample remained stable for several months stored at 253 K.

NMR spectra of (*S*)-**3h**: $BnPh_3P^+$: $F^-$ complexes were recorded on an AVIII HD 500 or AVIII HD 600 spectrometer.

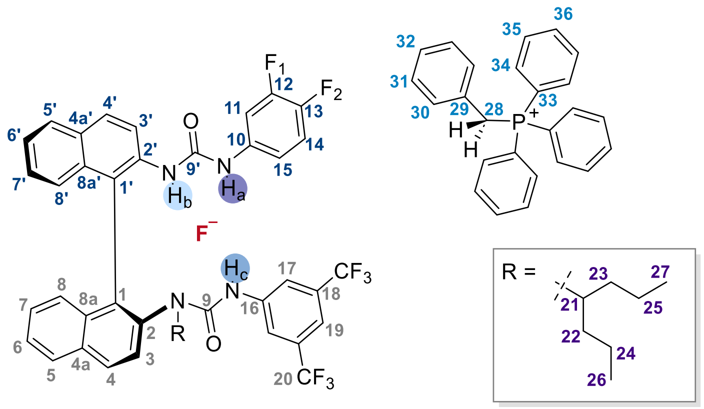

Supplementary Table 10: Assignment of (*S*)-**3h**: $BnPh_3P^+$: $F^-$ [UPF]

| $^{1}$H | δ [ppm] | Multiplicity $^{n}J$ [Hz] | $^{13}$C | δ [ppm] | Multiplicity $^{n}J_{CF}$ [Hz] |
|---|---|---|---|---|---|
| NH(c) | 12.7 | d, $^{1h}J_{FH}$ = 51 | 9 or 9’ | 155.6 or 154.7 | s |
| NH(a) | 12.4 | d, $^{1h}J_{FH}$ = 57 | 12 or 13 | 150.2 | dd, $^{1}J_{CF}$ =243.3 $^{3}J_{CF}$ = 12.4 |
| NH(b) | 10.6 | d, $^{1h}J_{FH}$ = 31 | 16 | 145.5 | s |
| 3’ | 9.72 | d, $^{3}J_{HH}$ = 9.2 | 8a | 145.0 | s |
| 17 | 9.09 | s | 12 or 13 | 144.9 | dd, $^{1}J_{CF}$ =238.5 $^{3}J_{CF}$ = 13.5 |
| 4’ | 7.84 | d, $^{3}J_{HH}$ 9.2 | 2’ | 139.4 | s |
| 5’ | 7.61 | d, $^{3}J_{HH}$ | 10 | 138.6 | d, $^{3}J_{CF}$= 8.9 |
| 3 or 4 | 7.59 or 7.34 | d, $^{3}J_{HH}$ | 8 | 135.6 | s |
| 11 | 7.51 | m | P36 | 134.9 | d, $J_{CP}$ = 2.4 |
| 19 | 7.46 | s | 8a’ | 134.3 | s |
| 5 | 7.32 | d, $^{3}J_{HH}$ | P34 | 133.9 | d, $J_{CP}$ = 9.8 |
| 6’ | 7.03 | t, overlapping | 1 | 132.7 | s |
| 8, 8’ and 6 | 7.01-6.95 | overlapping | P31 | 132.3 | d, $J_{CP}$ = 8.0 |
| P31 | 6.86 | overlapping | 4a | 131.8 | s |
| 15 | 6.84 | overlapping | P32 | 131.6 | d, $^{4}J_{CP}$ = 2.4 |
| 30 | 6.68 | br d | P30 | 131.4 | d, $^{3}J_{CP}$ = 4.7 |
| 7 or 7’ | 6.63 | t, J = 8.0 | 3 or 4 | 130.5 | s |
| 14 | 6.54 | q $^{2}J_{HF}$ = 9.3 | P35 | 130.0 | d, $^{3}J_{CP}$ = 10.4 |
| 28 | 4.25 and 3.82 | dd, $^{2}J_{HH}$ = 15.1, $^{2}J_{HP}$ = 14.8 | 4’ | 129.5 | s |
| 30 | 6.68 | br d | 3 or 4 | 129.4 | s |
| 21 | 3.03 | br s | 5’ | 128.5 | overlapping |
| 22 or 23 | 2.59 | br m | 5 | 128.4 | overlapping |
| 22 or 23 | 2.10 | overlapping | P29 | 127.9 | overlapping |
| 22 or 23 | 1.59 | br m | 1’ | 126.5 | s |
| 24 or 25 | 1.47 | br m | 20 | 124.7 | q, $^{1}J_{CF}$ = 273.1 |
| 24 or 25 | 1.22 | br m | 3’ | 120.4 | s |
| 22 or 23 | 0.81 | br m | 17 | 119.4 | s |
| 26 or 27 | 0.79 | t $^{3}J_{HH}$ = 6.9 | P33 | 118.1 | d, $^{1}J_{CP}$ = 85.5 |
| 24 or 25 | 0.52 | br m | 14 | 116.4 | d, $^{2}J_{CF}$ = 17.5 |
| 26 or 27 | 0.49 | t $^{3}J_{HH}$ = 6.3 | 15 | 113.6 | s |
| 24 or 25 | -0.40 | br s | 19 | 113.3 | s |
| **$^{19}$F** | **δ [ppm]** | **Multiplicity $^{n}J$ [Hz]** | 11 | 107.3 | d |
| $CF_3$ | -62.1 | s | 21 | 65.7 | s |
| $F^-$ | -74.2 | br s | 22 or 23 | 36.6 | s |
| F1 | -137.4 | m | 22 or 23 | 35.5 | s |
| F2 | -148.5 | m | P28 | 28.5 | d, $^{2}J_{CP}$ = 46.8 |
| | | | 24 or 25 | 22.1 | s |
| **$^{31}$P** | **δ [ppm]** | **Multiplicity $^{n}J$ [Hz]** | 24 or 25 | 20.4 | overlapping |
| $P^+$ | 22.3 | br s | 26 or 27 | 14.7 | s |
| | | | 26 or 27 | 14.4 | s |

*overlapping signals masked by solvent peaks, phosphonium protons 32, 34, 35, 36 overlapping with solvent peaks, urea carbons 2, 6, 7, 6’, 7’, 8’ indistinguishable

(*S*)-**3h**: $BnPh_3P^+$: $F^-$ complex Spectra in Toluene-$d_8$

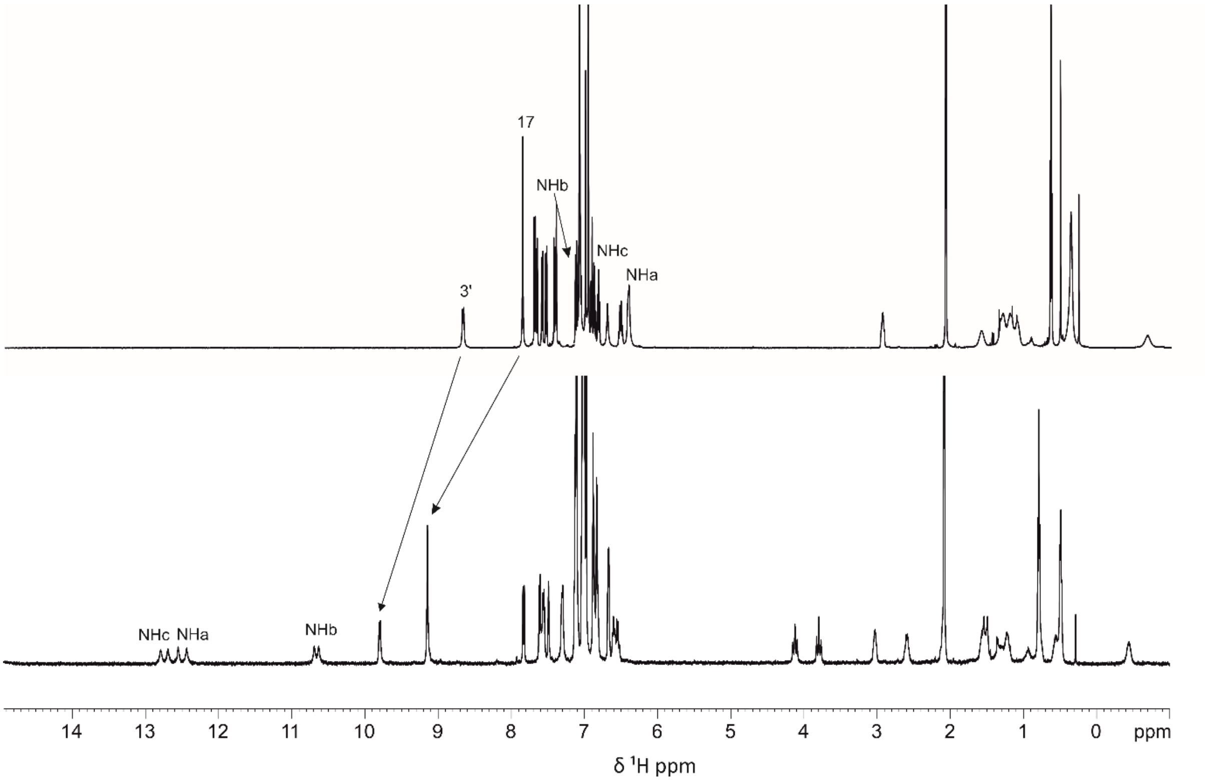


**Supplementary Figure 13**: Overlapping 1H of (*S*)-**3h** unbound and (*S*)-**3h**: $BnPh_3P^+$: $F^-$ complex (500 MHz, Toluene-$d_8$, 25

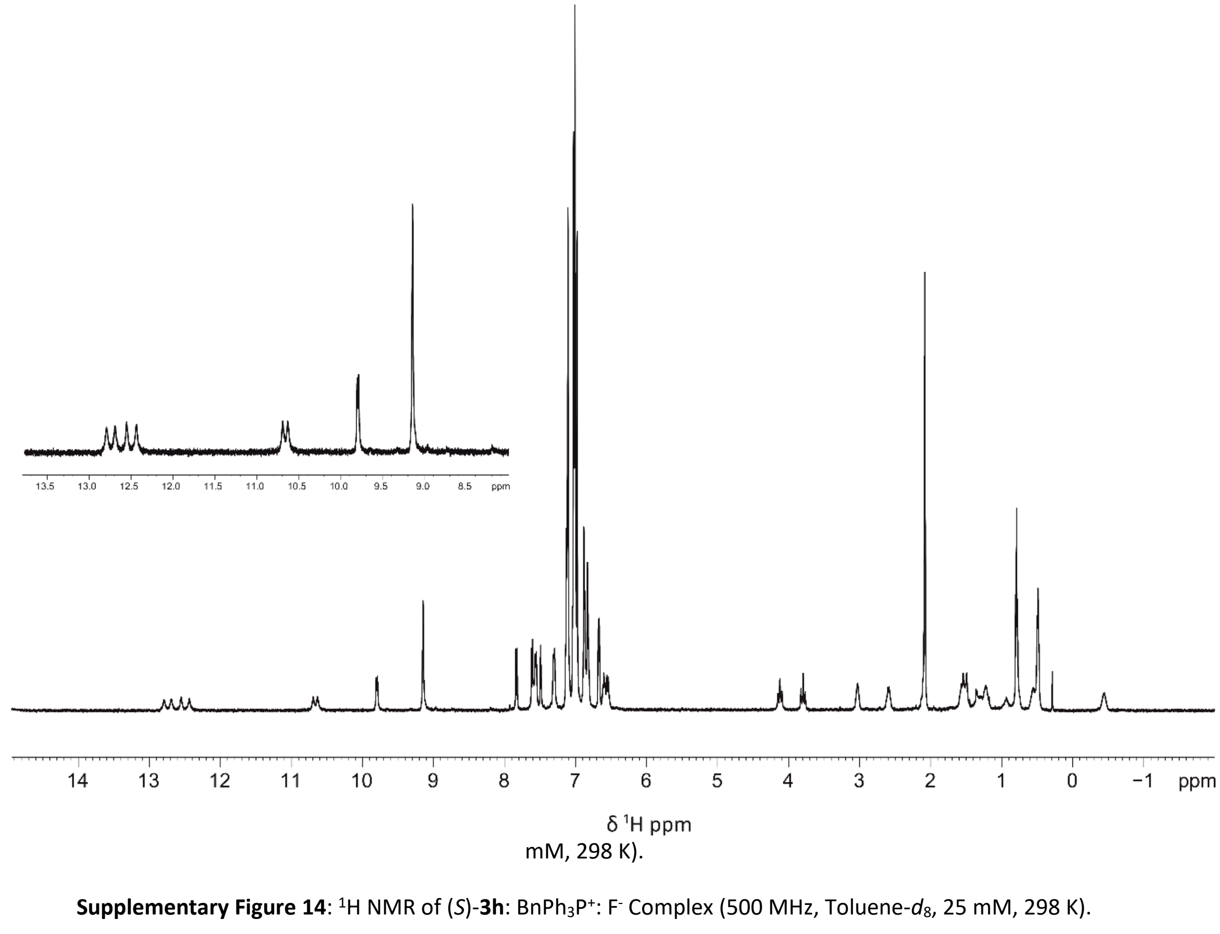


mM, 298 K).

**Supplementary Figure 14**: $^1H$ NMR of (*S*)-**3h**: $BnPh_3P^+$: $F^-$ Complex (500 MHz, Toluene-$d_8$, 25 mM, 298 K).

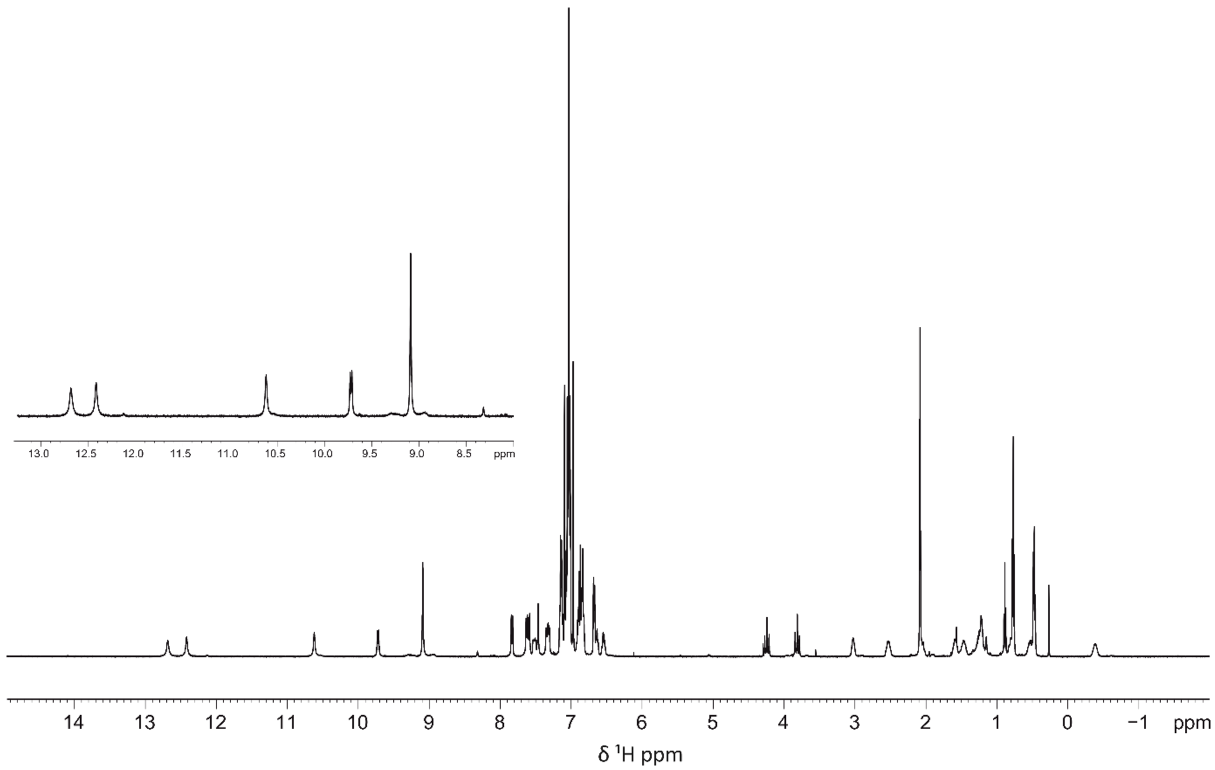


**Supplementary Figure 15**: $^{1}H\{^{19}F\}$ NMR of (*S*)-**3h**: $BnPh_3P^+$: $F^-$ Complex (500 MHz, Toluene-$d_8$, 25 mM, 298 K).

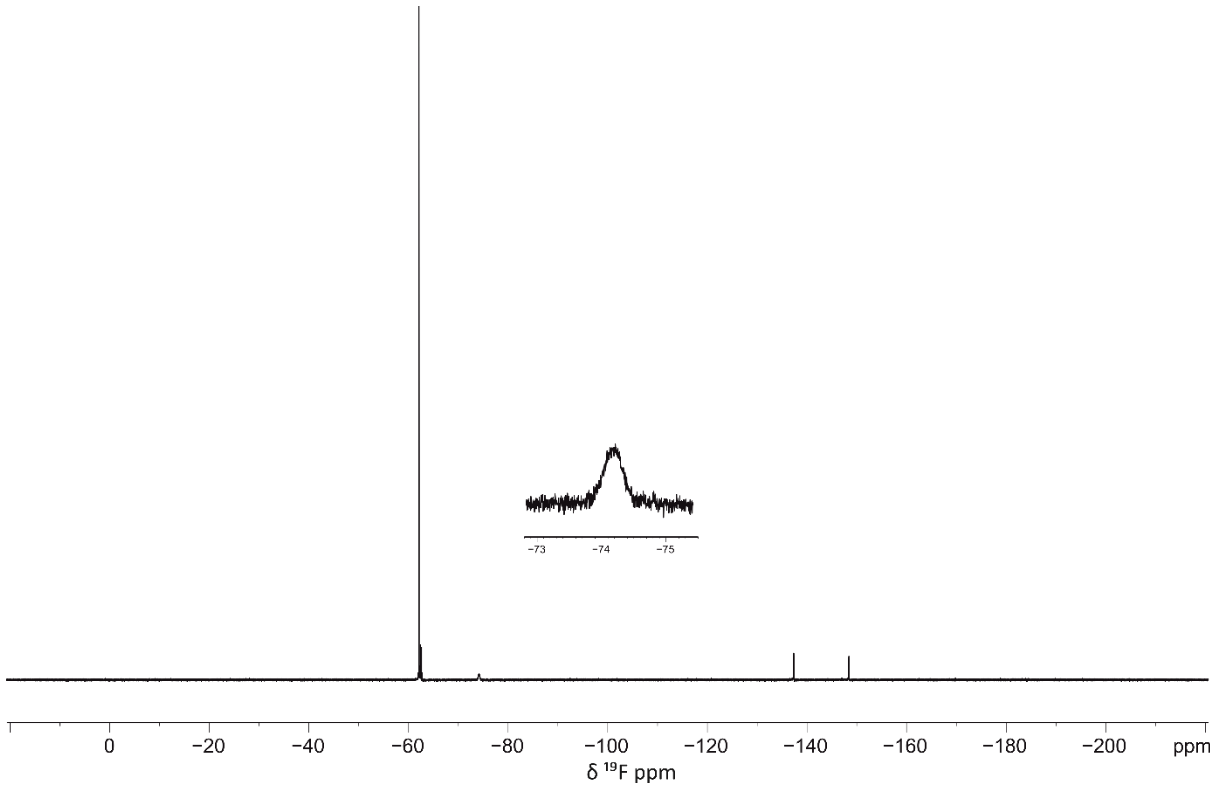


**Supplementary Figure 16**: $^{19}F$ NMR of (*S*)-**3h**: $BnPh_3P^+$: $F^-$ Complex (471 MHz, Toluene-$d_8$, 25 mM, 298 K).

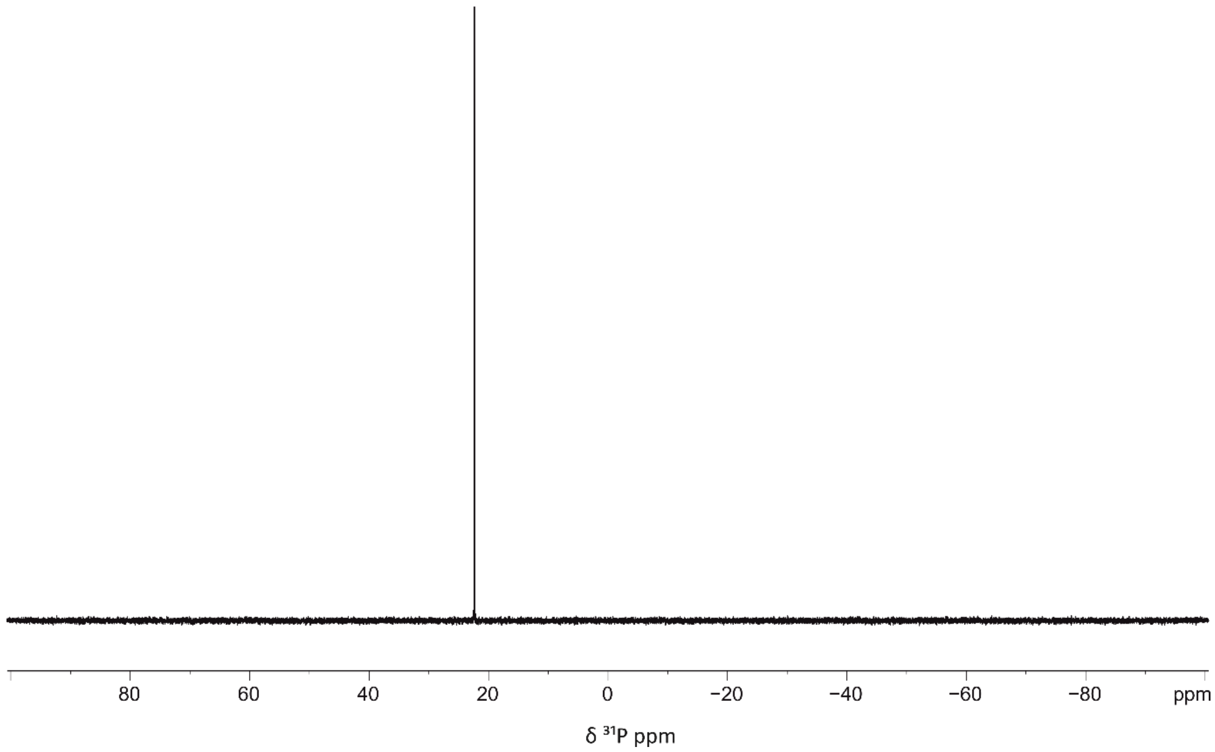


**Supplementary Figure 17**: $^{31}P$ NMR of (*S*)-**3h**: $BnPh_3P^+$: $F^-$ Complex (203 MHz, Toluene-$d_8$, 25 mM, 298 K).

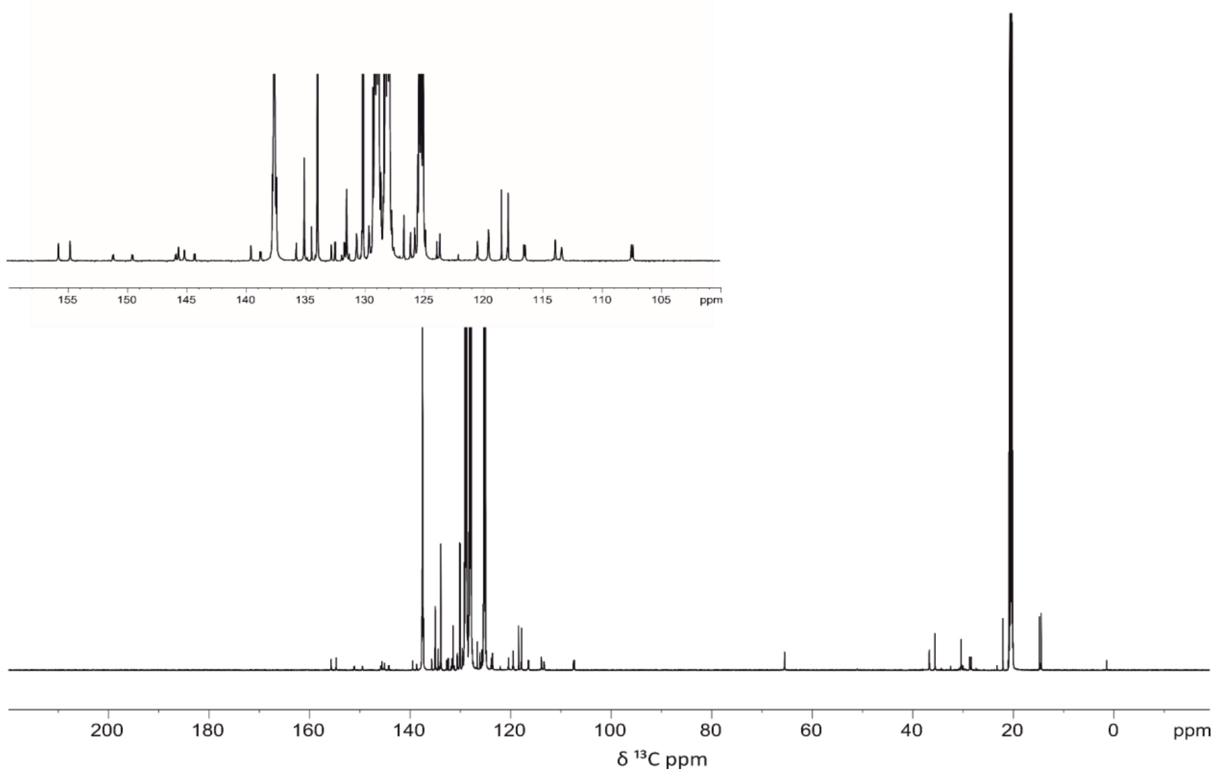


**Supplementary Figure 18**: $^{13}C$ NMR of (*S*)-**3h**: $BnPh_3P^+$: $F^-$ Complex (151 MHz, Toluene-$d_8$, 25 mM, 298 K).

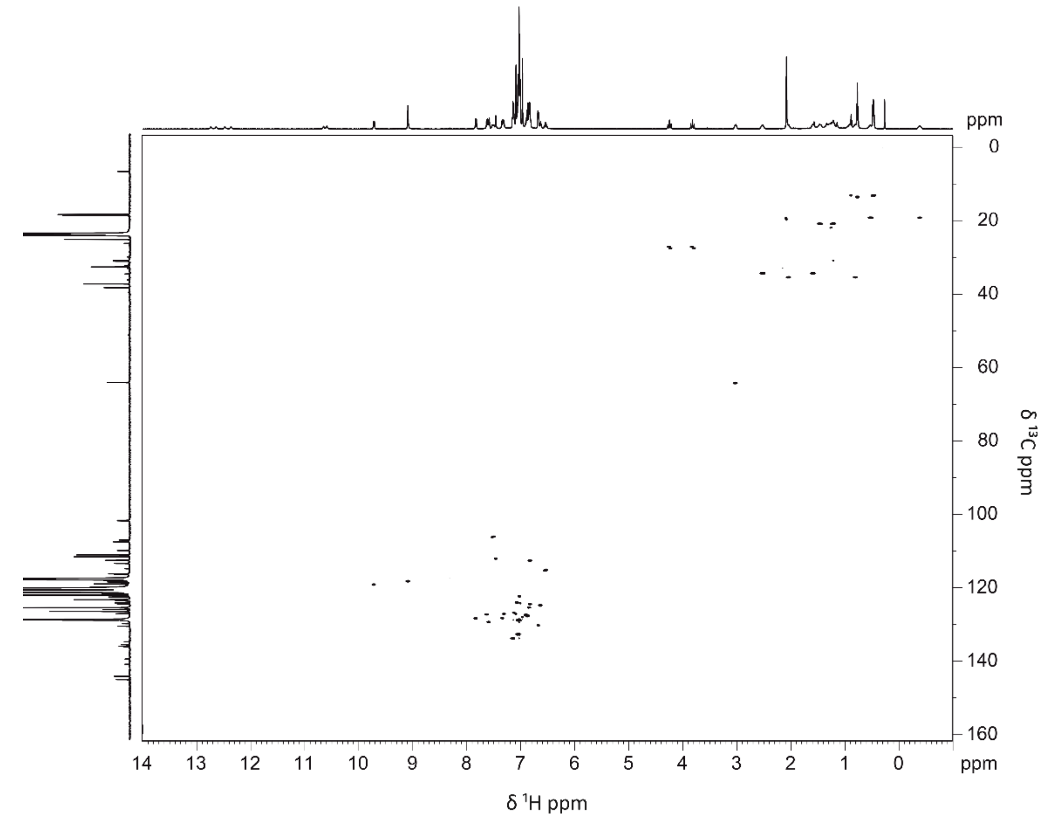


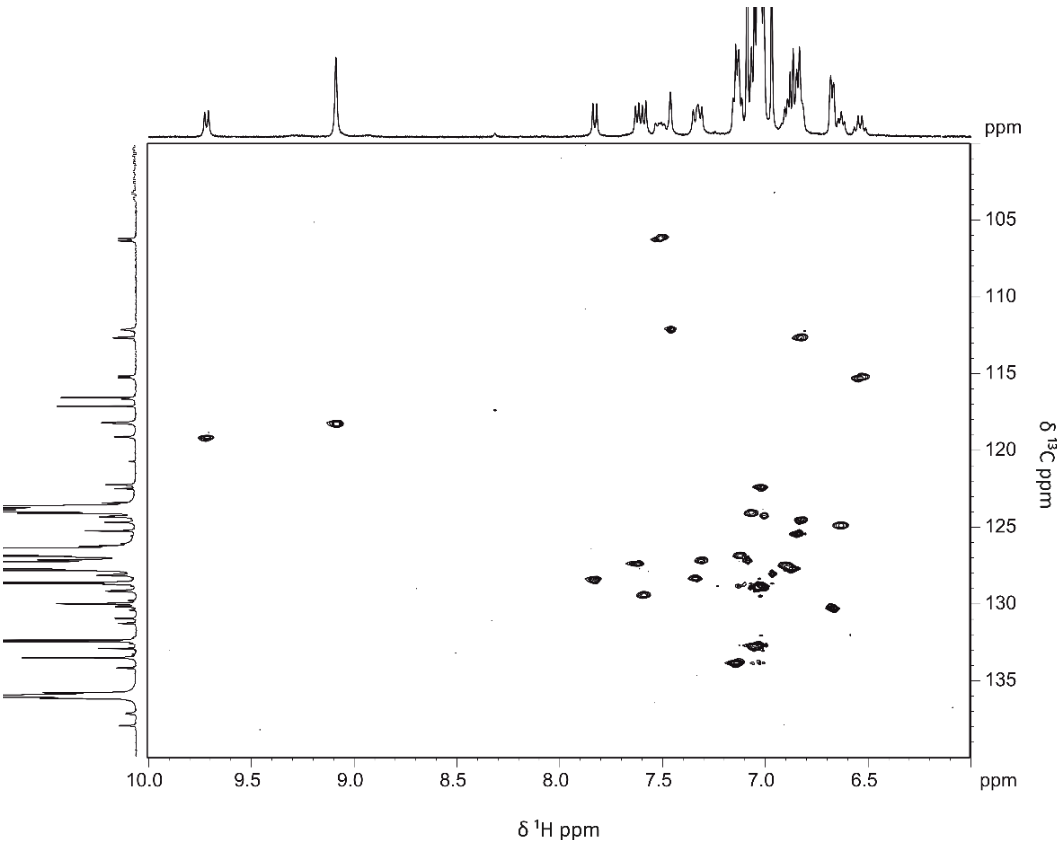


**Supplementary Figure 19**: $^{1}H$-$^{13}C$ HSQC of (*S*)-**3h**: $BnPh_3P^+$: $F^-$ Complex (500 MHz, Toluene-$d_8$, 25 mM, 298 K).

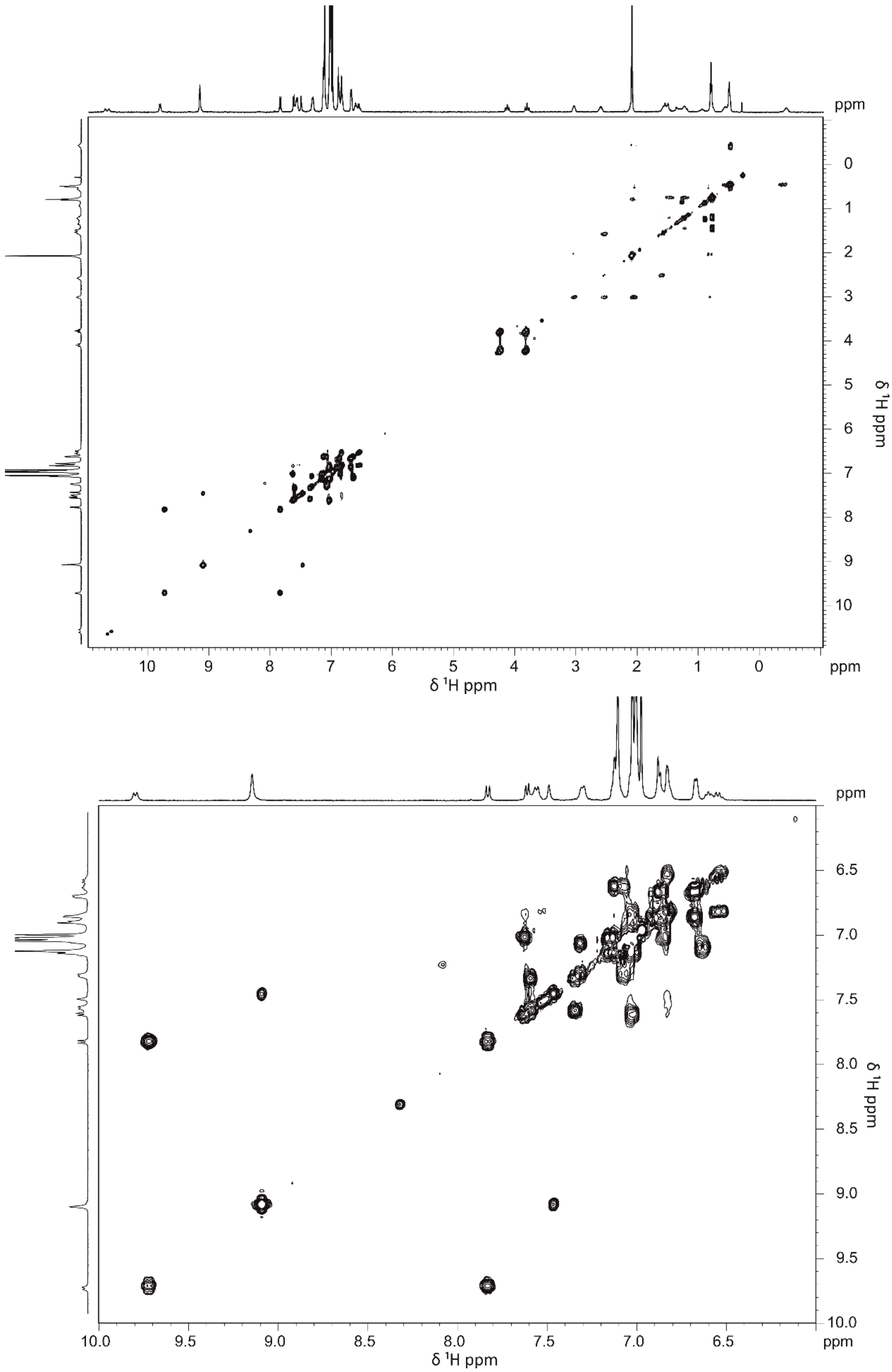


**Supplementary Figure 20**: $^{1}$H COSY of (*S*)-**3h**: $BnPh_3P^+$: $F^-$ Complex (500 MHz, Toluene-$d_8$, 25 mM, 298 K).

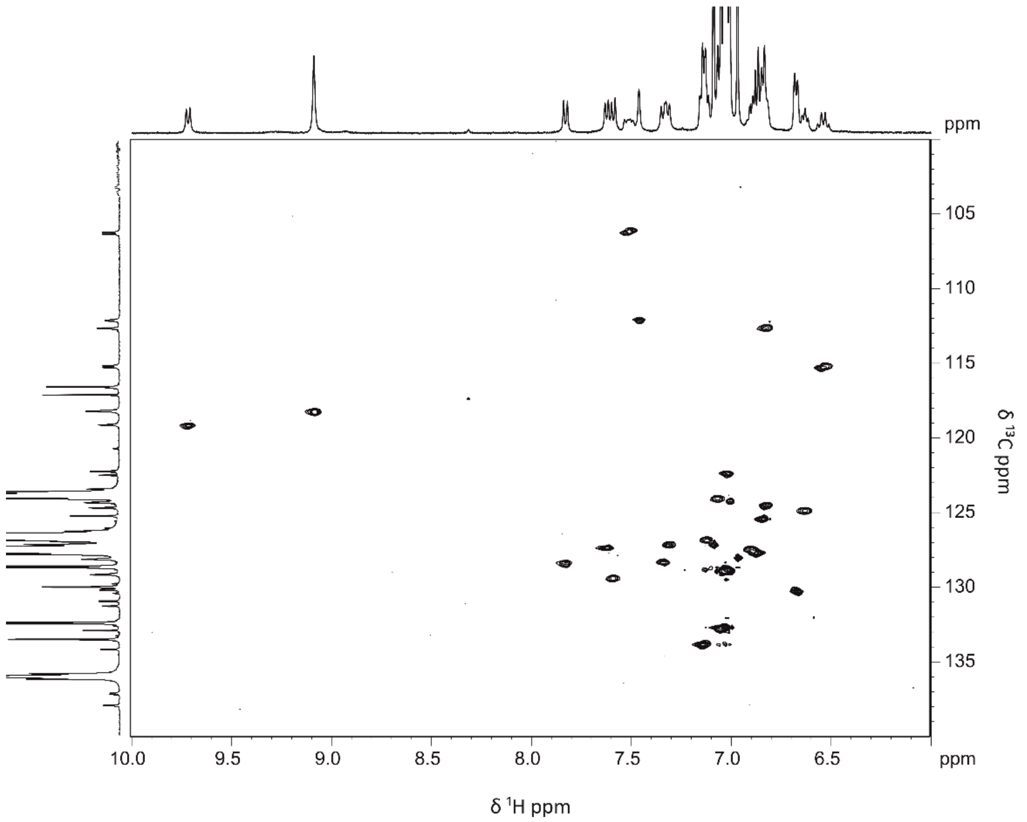


**Supplementary Figure 21**: ¹H- 13C HMBC of (*S*)-**3h**: $BnPh_3P^+$: $F^-$ Complex (500 MHz, Toluene-$d_8$, 25 mM, 298 K).

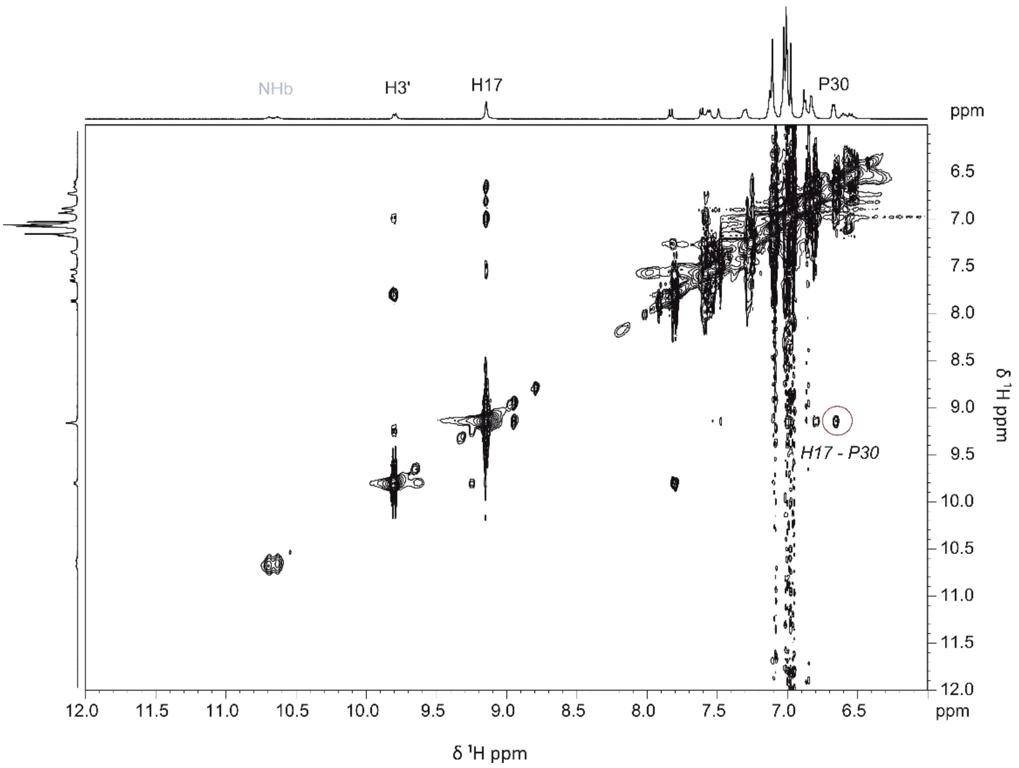


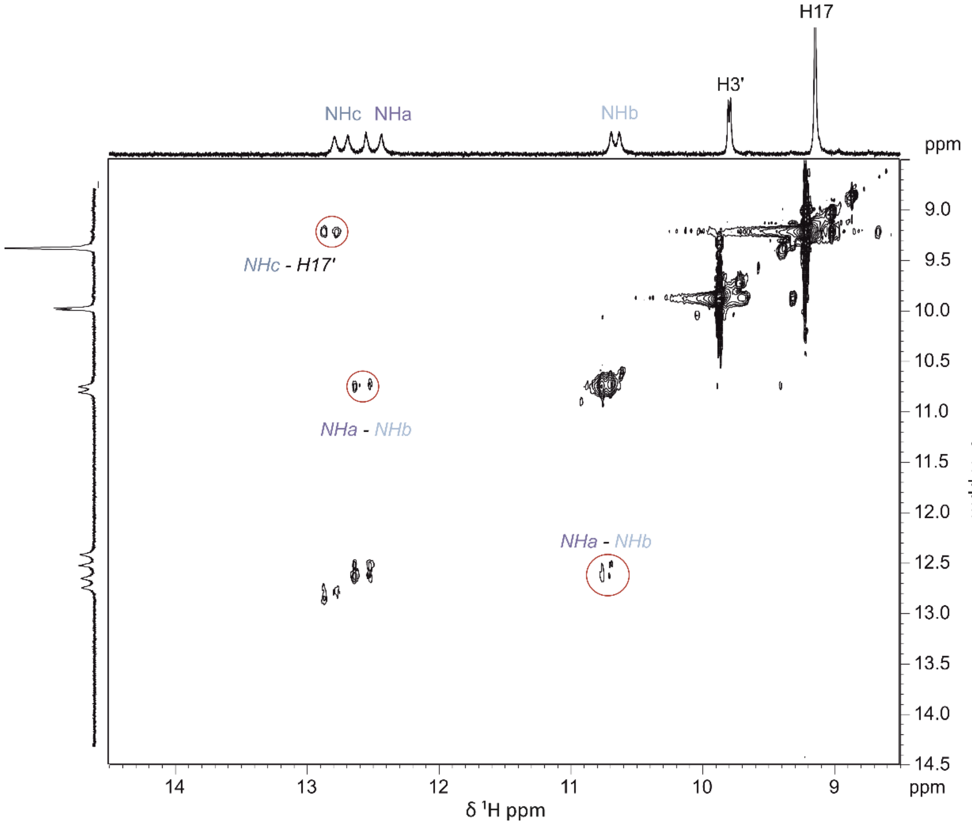


**Supplementary Figure 22**: $^{1}$H ROESY of (*S*)-**3h**: $BnPh_3P^+$: $F^-$ Complex spin lock pulse (P15) = 100 ms (500 MHz, Toluene-$d_8$, 25 mM, 298 K).

Investigation into solvent effect: (*S*)-**3h**: $BnPh_3P^+$: $F^-$ [UPF] Complex Spectra in DCM-$d_2$

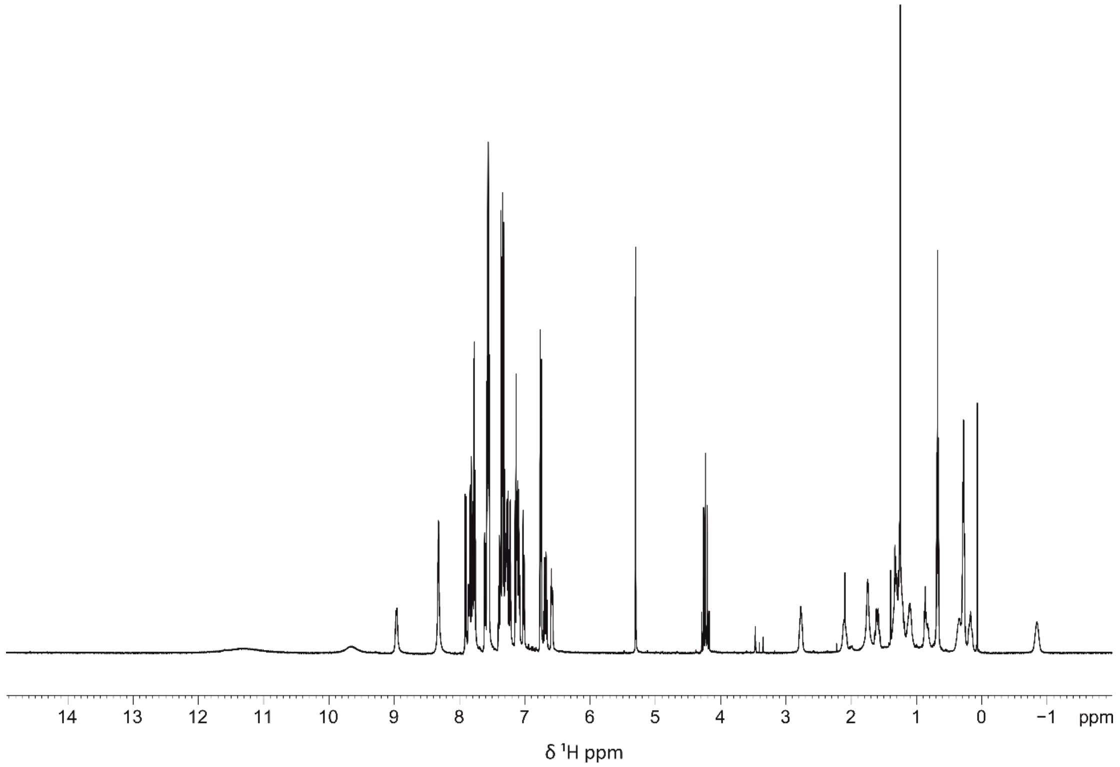


**Supplementary Figure 23**: $^1H$ NMR of (*S*)-**3h**: $BnPh_3P^+$: $F^-$ Complex (500 MHz, DCM-$d_2$, 25 mM, 298 K).

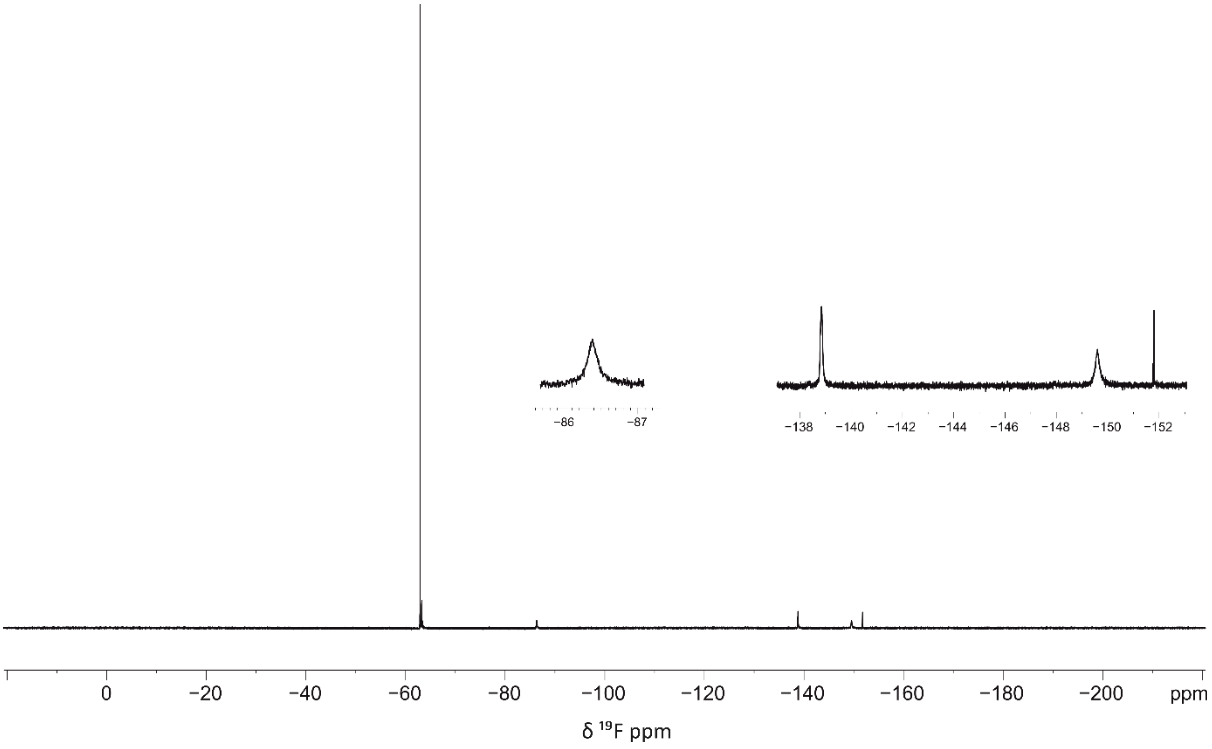


**Supplementary Figure 24**: $^{19}F$ NMR of (*S*)-**3h***:* $BnPh_3P^+$*:* $F^-$ Complex (471 MHz, DCM-$d_2$, 25 mM, 298 K).

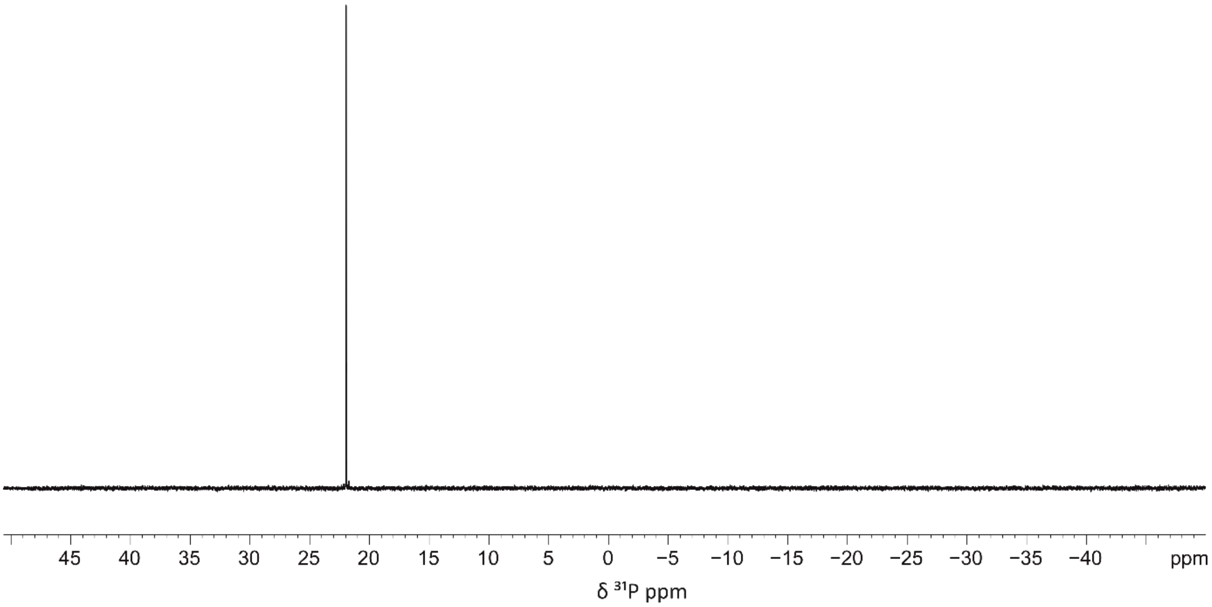


**Supplementary Figure 25**: $^{31}P$ NMR of (*S*)-**3h**: $BnPh_3P^+$: $F^-$ Complex (203 MHz, DCM-$d_2$, 25 mM, 298 K).

**HOESY**

Relative and absolute distance determination – A 25 mM sample of (*S*)-**3h:** $Ph_3BnP^+$: $F^-$ was prepared in toluene-$d_8$.

1D heteronuclear $^1H$—$^{19}F$ nOe experiments (HOESY) utilising $^{19}F$ inversion and $^1H$ detection, were acquired on an AVIII HD 500 using a previously developed pulse sequence (hoesyfhgpsp1d_v2cpd.wvm)[29] and the parameters below.

Supplementary Table 11: Parameters for $^1H$—$^{19}F$ nOe experiments (HOESY):

| Parameter | Value |
|---|---|
| **TD** | 16384 |
| **SW** | 20 ppm |
| **o1p/ o2p** | 4.7/ -74 |
| **NS (DS)** | 256 |
| **relaxation delay d1** | 3 s |
| **mixing time d8** | 10-600 ms |

nOe build-up curves (Supplementary Figure 26, Supplementary Table 12) for the nOe developed between fluoride and NH or CH were used to calculate the relative distances according to supplementary equation 1.

**Supplementary Equation 1:**

$$\eta_{\mathrm{H}}\{F^-\} \propto r_{H-F}^{-6}$$

$\eta_{\mathrm{NH}}\{F^-\}$ = H—F nOe build up rate

$r_{NH-F}$ = H—F distance

Supplementary Table 12: Determination of relative distances:

| nOe correlation | nOe build up rate | $\sqrt[6]{\frac{1}{\eta}}$ | Distance relative to H(a)-F = 1.0 |
|---|---|---|---|
| **NH(a)-F** | 63.704 | 0.500 | 1.00 |
| **NH(b)-F** | 40.905 | 0.538 | 1.08 |
| **NH(c)-F** | 74.290 | 0.488 | 0.98 |
| **CH(17)-F** | 15.075 | 0.636 | 1.27 |
| **P-CH(34/35)-F** | 16.200 | 0.629 | 1.26 |
| **P-CH(30)-F** | 3.137 | 0.827 | 1.65 |
| **P-CH(28)-F** | 20.985 | 0.602 | 1.20 |

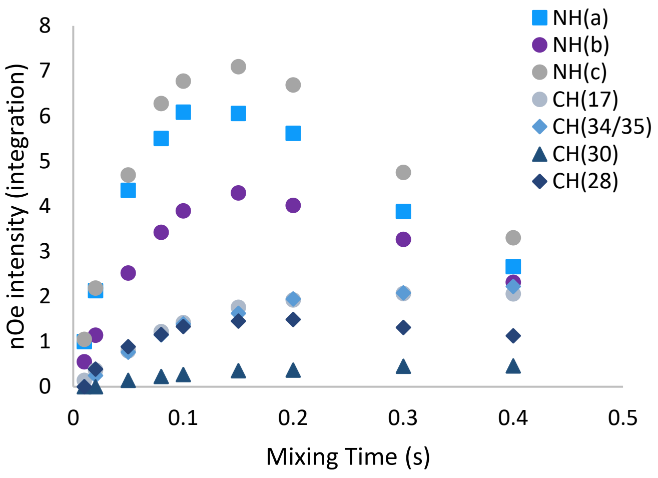


**Supplementary Figure 26**: $^{1}H$–$^{9}F$ nOe build-up curves of (*S*)-**3h:** $Ph_3BnP^+$: $F^-$.

*Absolute Distance Determination:*

1D heteronuclear $^{1}H$–$^{19}F$ nOe experiments (HOESY) utilising $^{19}F$ inversion and $^{1}H$ detection, were acquired on an AVIII HD 500 using a previously developed pulse sequence (hoesyfhgpsp1d_v2cpd.wvm) under equivalent experiments.

An additional set of experiments irradiating F reference (-148 ppm) was acquired H–F distance for reference – 2.60 Å.[24] A mixing time which lay within the linear region of growth of nOe for F- (-74 ppm) and C-F (-148 ppm), (50 ms) was selected to determine the absolute distances according to Supplementary Equation 2.

**Supplementary Equation 2:**

$$r_{NH-F} = \sqrt[6]{\frac{I_{H-F\,ref.}}{I_{H-F}}}\, r_{H-F\,ref.}$$

$r_{NH-F}$ = NH-F distance

$r_{NH-F\,ref}$ = H-F distance reference = 2.60 Å

$I_{NH-F}$ = nOe intensity between H-F

Supplementary Table 13: Determination of absolute distances for (*S*)-**3h**: $Ph_3BnP^+$: $F^-$

| nOe correlation | $I_{NH-F}$ | Absolute H-F distance (Å) |
|---|---|---|
| **NH(a)–F** | 1.00 | 1.72 |
| **NH(b)–F** | 0.59 | 1.88 |
| **NH(c)–F** | 1.08 | 1.70 |
| **CH(17)–F** | 0.18 | 2.28 |
| **P-CH(30)–F** | 0.03 | 3.05 |
| **P-CH(28)–F** | 0.20 | 2.24 |
| **CH(14)–$F_2$ *(Reference)*** | 0.08 | 2.60 |

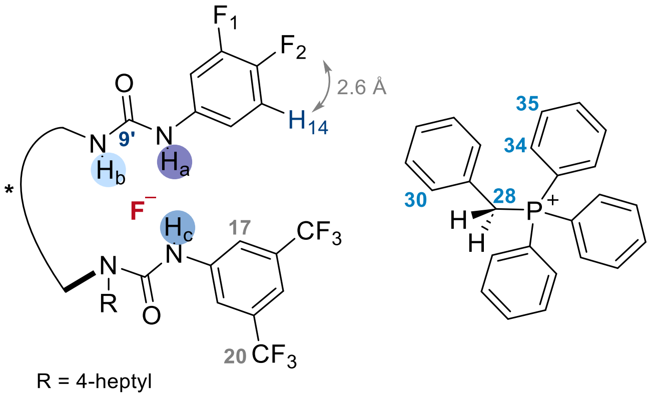


**Supplementary Figure 27**: $^1H–^{19}F$ HOESY correlations of (*S*)-**3h:** $Ph_3BnP^+$: $F^-$.

## $^1H–^{19}F$ CLIP-HSQC

$^1H–^{19}F$ CLIP-HSQC experiments were recorded on an AVIII HD 500 using a 25 mM sample of (*S*)-**3h:** $Ph_3BnP^+$: $F^-$ in toluene-$d_8$ at 298 K. The experiment was acquired using pulse sequence (*hsqcetgpclip)*[30] under the following parameters:

Supplementary Table 14: Parameters for $^1H–^{19}F$ CLIP-HSQC experiments:

| **Parameter** | **Value** |
|---|---|
| **TD** | 2048 (f2) x 128 (f1) |
| **SW** | 8 (f2) x 25 (f1) |
| **o1p/ o2p** | 12/ -75.5 ppm |
| **NS (DS)** | 4 (4) |
| **relaxation delay d1** | 2 s |
| **delay for evolution of coupling CNST[2]** | 50 |

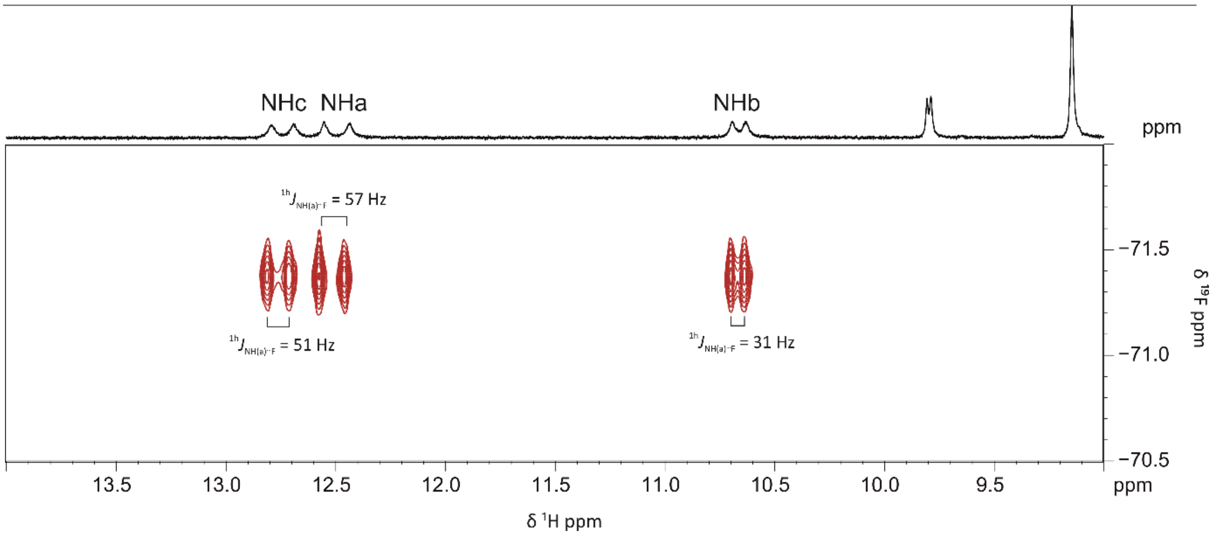


**Supplementary Figure 28**: $^1H–^{19}F$ CLIP-HSQC of (*S*)-**3h**: $Ph_3BnP^+$: $F^-$

**Kinetic Isotope Effect Experiments**

General Procedures and Reaction Assembly

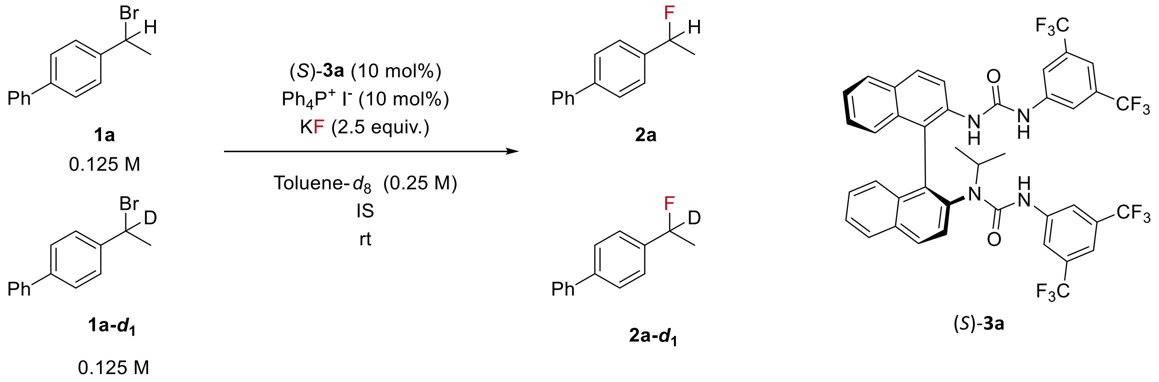


Benzylic bromide **1a** (0.0625 mmol), deuterated benzylic bromide **1a-*d*₁** (or **1a-*d*₃**) (0.0625 mmol) and anhydrous toluene-$d_8$ (0.5 mL) were loaded into a J. Young Norell 5mm NMR tube. 4-fluoroanisole (0.044 mmol) or 1,2,3-trifluoro-5-methoxybenzene (0.0396 mmol) was added and used as internal standard. The NMR tube was then sealed and inserted into the NMR spectrometer. A reference spectrum was taken using $^1$H NMR with a 90° excitation pulse (zg) and a relaxation delay of $t_{D1}$ = 30.0 s, NS(DS) = 8(0). After that, (*S*)-**3a** (0.0125 mmol), $Ph_4P^+$ $I^-$ (0.0125 mmol) and KF (0.3125 mmol) were added to the NMR tube to initiate the reaction, and the time was recorded. The initial timepoint was analysed immediately with quantitative $^1$H NMR (zg) and $^{19}$F{$^1$H} NMR (90° excitation pulse (zgig), a relaxation delay of $t_{D1}$ = 30.0 s, NS(DS)= 12(4), o1p = -146 ppm, SW = 100 ppm).

Following initial quantification NMR tubes were secured on an external motor (Supplementary Figure 29) and rotated at 5 rpm at room temperature. The reaction was rotated for specified time before being swiftly transferred back to spectrometer where quantitative $^1$H and $^{19}$F{$^1$H} spectra (under same parameters) were taken. This was repeated over the course of reaction monitoring to build kinetic curves.

All NMR spectra were recorded on AVIIIHD 500 NMR spectrometer at 298 K. All spectra acquired were processed using Mestrenova version 14.0, with 128 K points zero filling, manual phase correction and baseline correction. In some cases, regional baseline correction and line fitting were performed to get a more accurate estimation of the integral of the peak. Examples and more details of spectral processing are included in later section. Each peak was integrated, and concentrations of substrates and products were calculated by comparison of the corresponding integral to that of internal standard.

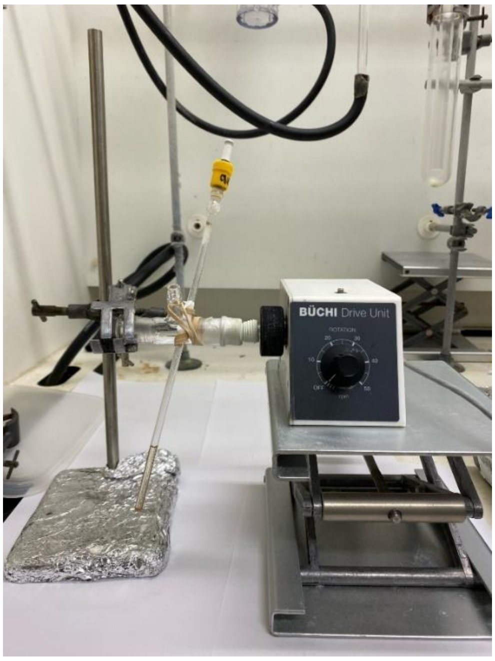


**Supplementary Figure 29**: Motor set up for periodic rotation of NMR tube

### ***$T_1$* Measurements**

The longitudinal relaxation time constants $T_1$ of the benzylic fluoride product **2a**, benzylic bromide substrate **1a** and internal standards, 4-fluoroanisole and 1,2,3-trifluoroanisole, were measured *via* $^{1}H$ and $^{19}F$ inversion recovery experiments (t1ir) on an AVIII 500. $T_1$ values were calculated by non-linear fitting using Dynamic centre on Topspin version 4.1.1. The results are summarised in Supplementary Table 17.

*Sample preparation*: A 0.25 M sample of bromide **1a** (0.0625 mmol), fluoride **2a** (0.0625 mmol), and $Ph_4P^+$ $I^-$ (0.0125 mmol) was prepared in toluene-$d_8$ with 5 µL of internal standard (4-fluoroanisole or 1,2,3-trifluoroanisole). The experiment was acquired with the following parameters.

Supplementary Table 15: Parameters for $^{1}H$ $T_1$ measurements –bromide **1a**, fluoride **2a** and internal standards (IS):

| Parameter | Value |
|---|---|
| TD | 65536 |
| SW | 18 |
| O1P | 5.0 |
| NS (DS) | 4 (0) |
| Relaxation delay d1 | 60 s |
| Variable delay d1 | 0.001-60 s (15) |

Supplementary Table 16: Parameters for $^{19}F$ $T_1$ measurement –fluoride **2a** and internal standards (IS):

| Parameter | Value |
|---|---|
| TD | 65536 |
| SW | 10 |
| O1P | -124.2 (IS)/ -167.5 (2a) |
| NS (DS) | 2 (2) |
| Relaxation delay d1 | 45 s |
| Variable delay d1 | 0.001-60 s (15) |

Supplementary Table 17: Summary of $T_1$ measurements

| Compound | $^1H$ $T_1$ (s) | $^{19}F$ $T_1$ (s) |
|---|---|---|
| **1a** | 3.564 | - |
| **2a** | 3.944 | 3.820 |
| 4-fluoroanisole (OMe) | 3.914 | 4.828 |
| 1,2,3-trifluoro-5-methoxybenzene (OMe) | 2.975 | 2.798 (-134 ppm) |

$t_{D1}$ value of 30.0 s was selected for quantitative $^1H$ and $^{19}F$ NMR.

## Synthesis of Deuterated Compounds

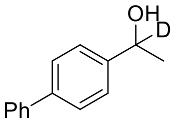


6a-$d_1$

### 1-([1,1'-biphenyl)-4-yl)ethan-1-*d*-1-ol (6a-$d_1$)

Compound **6a-*d*₁** was prepared according to a literature procedure[31] – 4-acetylbiphenyl (1 equiv.) was added to a round bottom flask and dissolved in EtOH (0.1 M), $NaBD_4$ (2 equiv.) was added and the reaction was stirred for 2 hours at 25 °C. Upon completion, monitored by TLC, the reaction was quenched with $H_2O$ and diluted with EtOAc. The aqueous phase was washed with EtOAc (3 x 10 mL) and organic extracts were washed with brine and dried with $MgSO_4$. Solvent was removed under reduced pressure and following purification (FCC eluent: 100% DCM), the product was isolated as a white solid in 75 % yield. Spectroscopic data are in accordance with those in the literature.[31]

**$^1$H NMR** (400 MHz, $CDCl_3$) δ = 7.65 – 7.56 (m, 4H), 7.46 (ddd, *J* = 7.9, 4.4, 2.1 Hz, 4H), 7.41 – 7.32 (m, 1H), 1.55 (s, 3H); **$^{13}$C NMR** (126 MHz, $CDCl_3$) δ = 142.3, 141.4, 140.6, 129.0, 127.7, 127.6, 127.4, 127.2, 49.2 (t, $J_{C\text{-}D}$ = 22.1 Hz), 26.8.

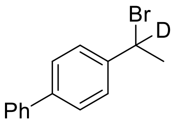

1a-$d_1$

**4-(1-bromoethyl-1-*d*)-1,1'-biphenyl (1a-*d*$_1$)**

Compound **1a-*d*$_1$ was** prepared according to General procedure 3 from *rac*-**6a-*d*$_1$**. The product was isolated as a white solid in 84 % yield.

**$^1$H NMR** (500 MHz, $CDCl_3$) δ = 7.62 – 7.56 (m, 4H), 7.53 (d, *J* = 8.4 Hz, 2H), 7.46 (t, *J* = 7.7 Hz, 2H), 7.41 – 7.34 (m, 1H), 2.12 (s, 3H); **$^{13}$C NMR** (126 MHz, $CDCl_3$) δ = 142.3, 141.4, 140.6, 129.0, 127.7, 127.6, 127.4, 127.2, 49.2 (t, $J_{C-D}$ = 23.2 Hz), 26.8; **IR** (neat) ν = 3030, 2927, 2854, 1739, 1564, 1486, 1404, 1376, 1258, 1231, 1205, 1072, 1044, 1006, 884, 938, 813, 765, 726, 692, 640 $cm^{-1}$; **mp** 68-69 ˚C; No HRMS obtained.

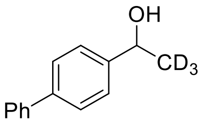

6a-$d_3$

**1-([1,1'-biphenyl]-4-yl)ethan-2,2,2-*d*$_3$-1-ol (6a-*d*$_3$)**

Compound **6a-*d*$_3$** was prepared through dropwise addition of $CD_3MgI$ (1.2 equiv.) to a solution of 4-phenylbenzaldehyde (1 equiv.) in dry THF (0.5 M) at -10 °C. The reaction was then stirred at 25 °C for 2 hours. Upon completion of the reaction, monitored by TLC, the reaction mixture was quenched with $NH_4Cl$ (sat) and diluted with $Et_2O$. The organic extracts were washed with brine and dried with $MgSO_4$. Solvent was removed under reduced pressure and the following purification (FCC eluent: 100% DCM) the product was isolated as a white solid in 98 % yield.

**$^1$H NMR** (600 MHz, $CDCl_3$) δ = 7.63 – 7.56 (m, 4H), 7.48 – 7.42 (m, 4H), 7.40 – 7.32 (m, 1H), 4.95 (s, 1H); **$^{13}$C NMR** (151 MHz, $CDCl_3$) δ = 144.9, 141.0, 140.6, 128.9, 127.4, 127.2, 126.0, 70.2, 24.4 (sept, $J_{C-D}$ = 19.3 Hz); **IR** (neat) ν = 3386, 3059, 3034, 2958, 2926, 1599, 1566, 1524, 1487, 1404, 1334, 1304, 1274, 1241, 1168, 1039, 1005, 965, 902, 833, 788, 623 $cm^{-1}$; **mp** 92-93 ˚C; No HRMS obtained.

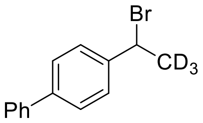

1a-$d_3$

**4-(1-bromoethyl-2,2,2-*d*$_3$)-1,1'-biphenyl (1a-*d*$_3$)**

Compound **1a-*d*$_3$** was prepared according to General procedure 3 from *rac*-**6a-*d*$_3$**. The product was isolated as a white solid in 84 % yield.

**$^1$H NMR** (600 MHz, $CDCl_3$) δ = 7.66 – 7.56 (m, 4H), 7.56 – 7.47 (m, 2H), 7.46 (tt, *J* = 6.6, 1.0 Hz, 2H), 7.42 – 7.30 (m, 1H), 5.28 (s, 1H); **$^{13}$C NMR** (151 MHz, $CDCl_3$) δ = 142.3, 141.4, 140.6, 129.0, 127.7, 127.6, 127.4, 127.2, 49.3, 26.0 (sept, $J_{C-D}$ = 19.3 Hz); **IR** (neat) ν = 3030, 2970, 2924, 1739, 1722, 1600, 1486, 1452, 1410, 1377, 1367, 1350, 1231, 1216, 1203, 1160, 1041, 1006, 901, 920, 765, 725, 692 $cm^{-1}$; **mp** 68-69 ˚C; No HRMS obtained.

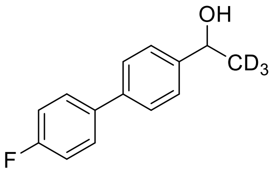

6b-$d_3$

**1-(4'-fluoro-[1,1'-biphenyl]-4-yl)ethan-2,2-2,2-*d*$_3$-1-ol (6b-*d*$_3$)**

Compound **6b-*d*$_3$** was prepared through dropwise addition of $CD_3MgI$ (1.2 equiv.) to a solution of 4-(4-fluorophenyl)benzaldehyde (1 equiv.) in dry THF (0.5 M) at -10 °C. The reaction was then stirred at 25 °C for 2 hours. Upon

completion of the reaction, monitored by TLC, the reaction mixture was quenched with $NH_4Cl$ (sat) and extracted with $Et_2O$. The organic extracts were washed with brine, dried with $MgSO_4$. Solvent was removed under reduced pressure and the following purification (FCC eluent: 100% DCM) the product was isolated as a white solid in 63 % yield.

**$^1$H NMR** (400 MHz, $CDCl_3$) δ = 7.60 – 7.49 (m, 4H), 7.49 – 7.41 (m, 2H), 7.18 – 7.07 (m, 2H), 4.95 (s, 1H); **$^{19}$F NMR** (376 MHz, $CDCl_3$) δ = -115.8; **$^{13}$C NMR** (101 MHz, $CDCl_3$) δ = 162.6 (d, *J* = 246.3 Hz), 145.0, 139.6, 137.1 (d, *J* = 3.3 Hz), 128.8 (d, *J* = 8.0 Hz), 127.3, 126.1, 115.8 (d, *J* = 21.4 Hz), 70.1, 24.2 (sept, $J_{C\text{-}D}$ = 19.3 Hz); **IR** (neat) ν = 3314, 2891, 2225, 1894, 1603, 1528, 1497, 1395, 1344, 1252, 1190, 1163, 1120, 1100, 1061, 1038, 1025, 1006, 964, 911, 821, 741, 649 $cm^{-1}$; **mp** 94-96 ˚C; No HRMS obtained.

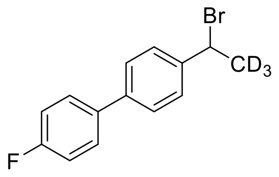


**1b-*d*$_3$**

**4-(1-bromoethyl-2,2,2-*d*$_3$)-4'-fluoro-1,1'-biphenyl (1b-*d*$_3$)**

Compound **1b-*d*$_3$** was prepared according to General procedure 3 from *rac*-**6a-*d*$_3$**. The product was isolated as a white solid in 71 % yield.

**$^1$H NMR** (600 MHz, $CDCl_3$) δ = 7.60 – 7.43 (m, 6H), 7.18 – 7.08 (m, 2H), 5.26 (s, 1H); **$^{19}$F NMR** (565 MHz, $CDCl_3$) δ = -115.3; **$^{13}$C NMR** (151 MHz, $CDCl_3$) δ = 162.7 (d, *J* = 246.8 Hz), 142.4, 140.4, 136.7 (d, *J* = 3.3 Hz), 128.8 (d, *J* = 8.0 Hz), 127.5, 127.4, 115.9 (d, *J* = 21.5 Hz), 49.1, 26.0 (sept, $J_{C\text{-}D}$ = 19.6 Hz); **IR** (neat) ν = 3002, 2952, 1600, 1498, 1240, 1196, 1163, 1041, 1006, 935, 813, 656, 637 $cm^{-1}$; **mp** 58-59 ˚C; No HRMS obtained.

## Representative Spectra and Concentration-Time Profiles

Competition experiment between **1a** and **1a-*d*$_1$**

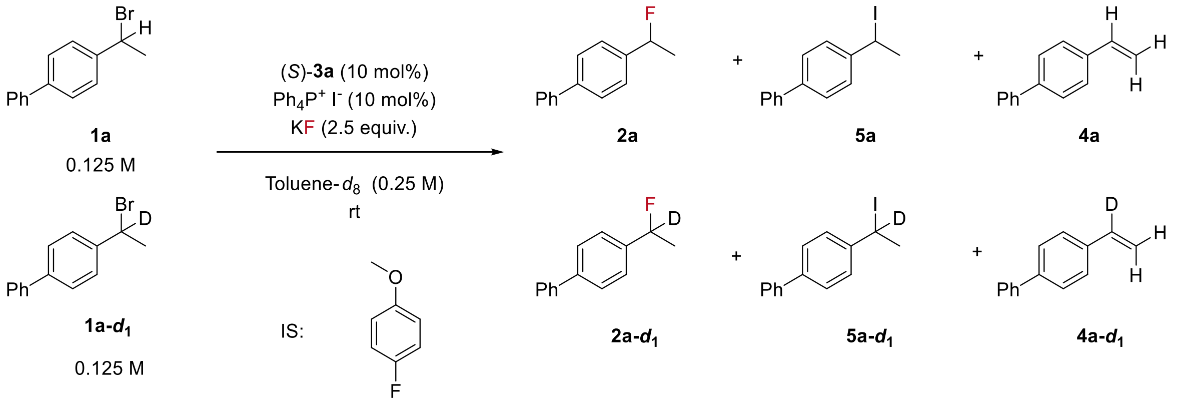


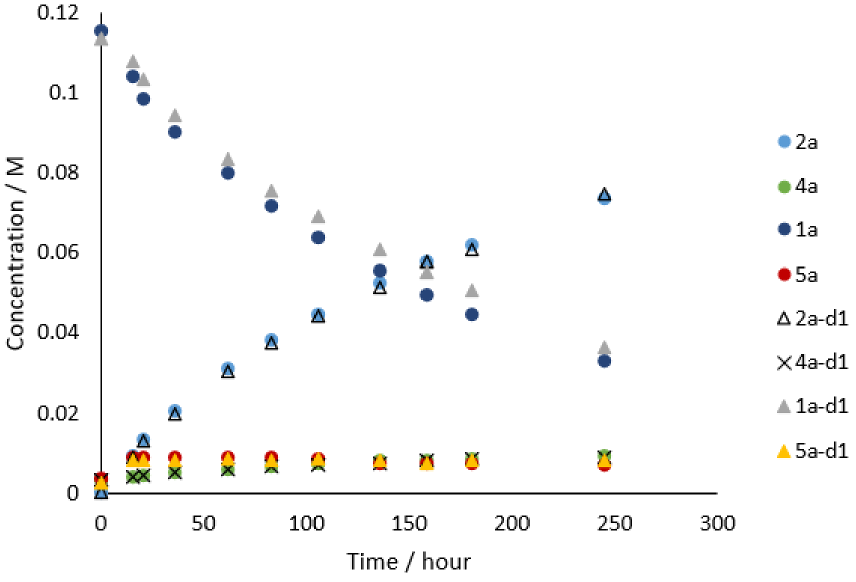


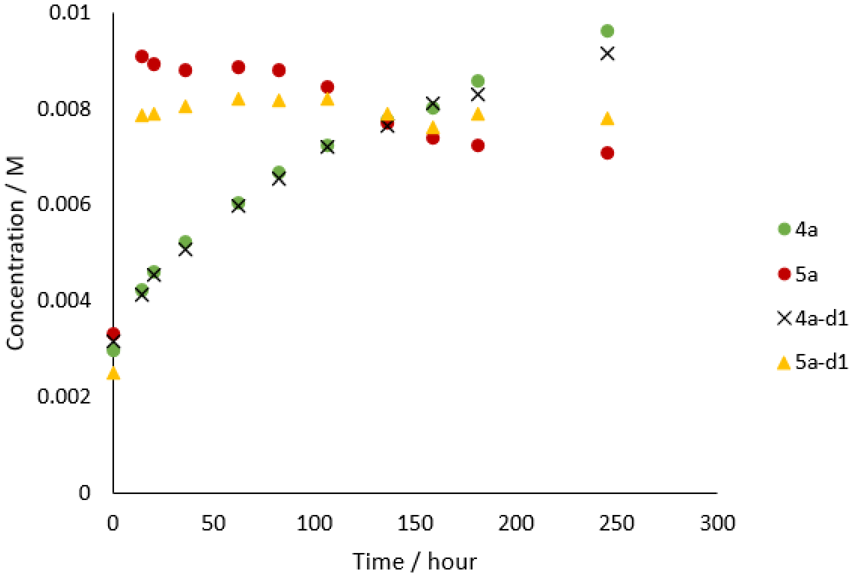


**Supplementary Figure 30**: Kinetic profile of the competition experiment between **1a** and **1a-*d*$_1$**

All spectra acquired within the same experiment were processed via the same method to minimise the error introduced. Typical $^{1}H$ NMR spectra after manual phase correction and baseline correction were shown in Supplementary Figure 31. By referring to the integral of the internal standard, the concentrations of benzylic bromides, iodides and eliminated side products were calculated. The concentrations of fluorides were calculated based on $^{19}F\{^{1}H\}$ NMR because of peak overlap in the $^{1}H$ NMR spectra. Typical $^{19}F\{^{1}H\}$ NMR spectra after processing are shown in Supplementary Figure 33.

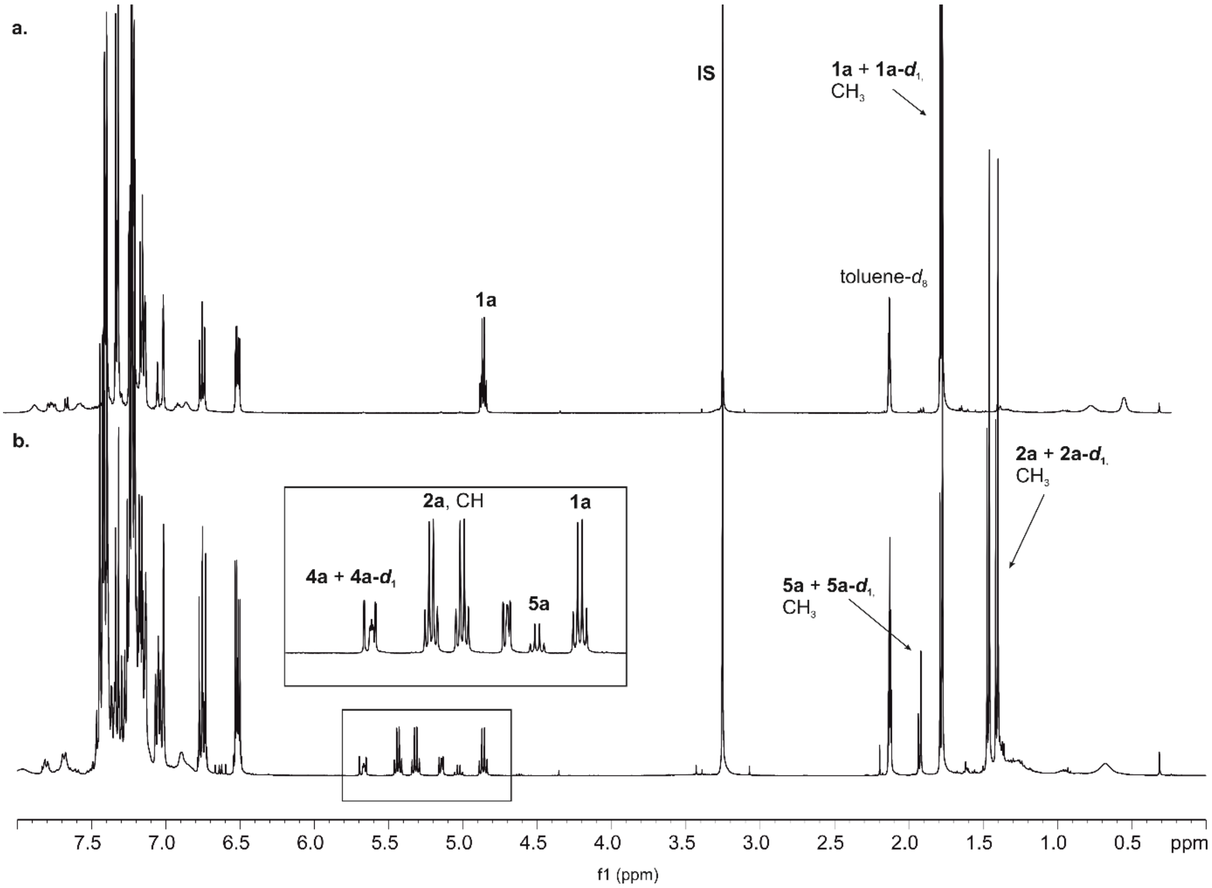


**Supplementary Figure 31**: Typical $^{1}H$ NMR spectra of the reaction mixture a) before starting of the reaction, b) at 70% conversion.

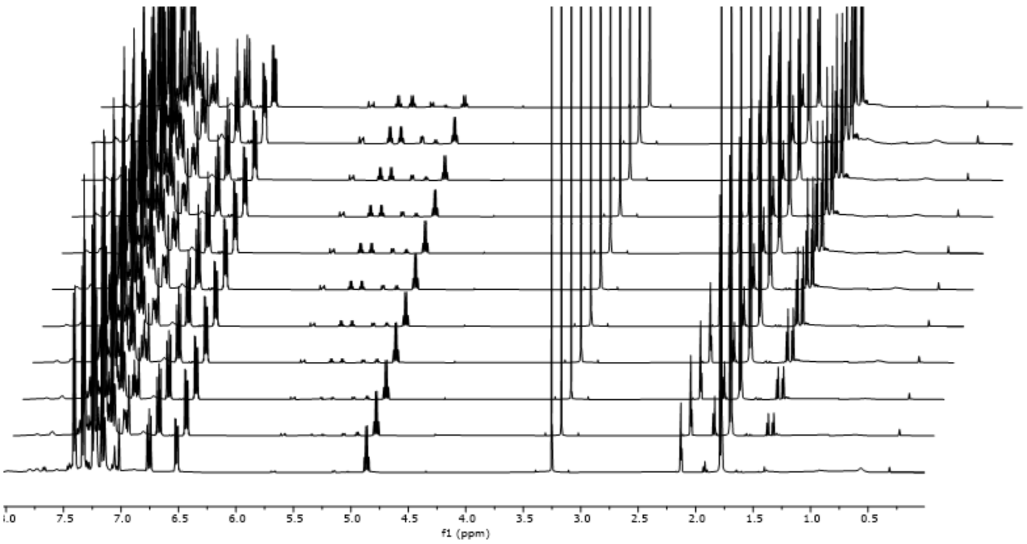


**Supplementary Figure 32**: The stacked $^{1}H$ NMR spectra for competition experiment between **1a** and **1a-*d*$_1$**

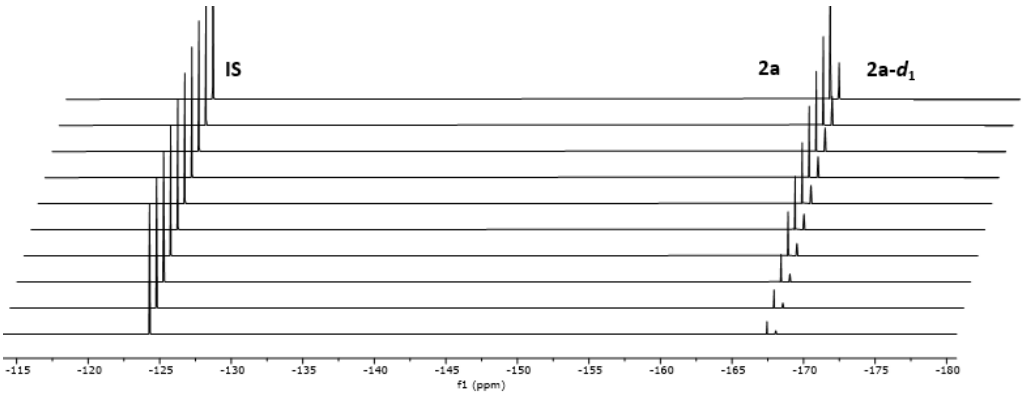


**Supplementary Figure 33**: The stacked $^{19}F\{^{1}H\}$ NMR spectra.

Competition experiment between **1a** and **1a-d₃**

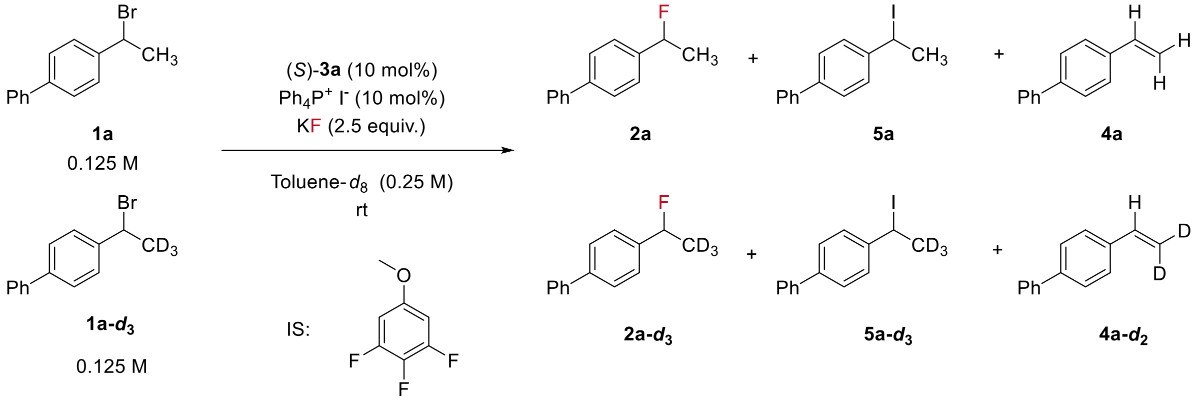


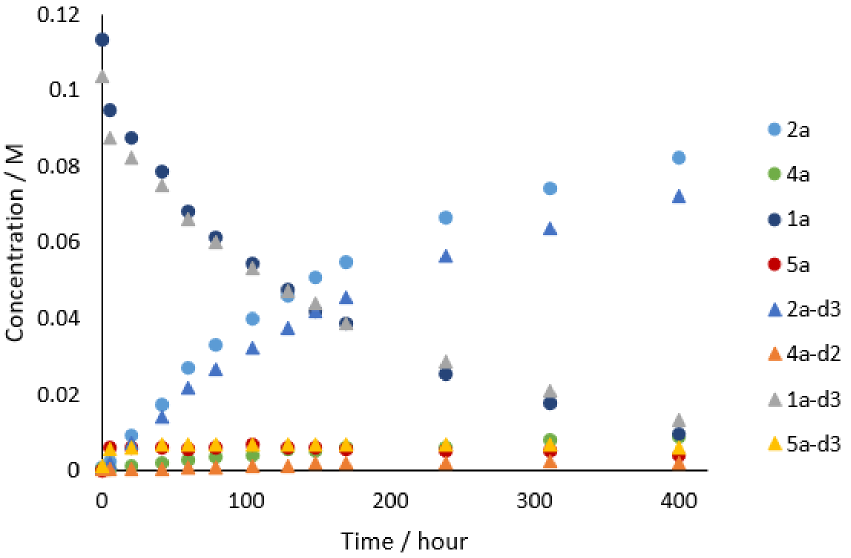


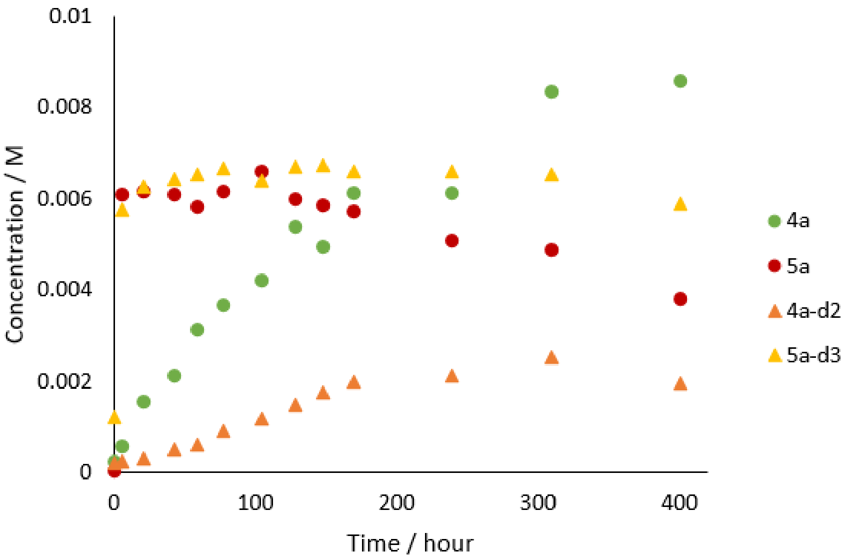


**Supplementary Figure 34**: Kinetic profile of the competition experiment between **1a** and **1a-*d*₃**.

All spectra acquired within the same experiment were processed via the same method to minimise the error introduced. Typical $^{1}H$ NMR spectra after manual phase correction and baseline correction were shown in Supplementary Figure 35. By referring to the integral of the internal standard, the concentrations of benzylic bromides and iodides were calculated. The concentrations of fluorides were calculated based on $^{19}F\{^{1}H\}$ NMR because of peak overlap in the $^{1}H$ NMR spectra. Typical $^{19}F\{^{1}H\}$ NMR spectra after processing are shown in Supplementary Figure 37. Regional baseline correction (Whittaker smoother, filter 3.81 Hz, from 4 to 10 ppm) was then performed to minimise the effect of neighbouring peaks on the integration of elimination side products. After performing line fitting on the region of 6.35 to 6.45 ppm, the peaks of the vinylic protons in **4a** (doublet of doublets) and **4a-*d*$_2$** (singlet) can be deconvoluted (Supplementary Figure 38) and their concentrations were calculated.

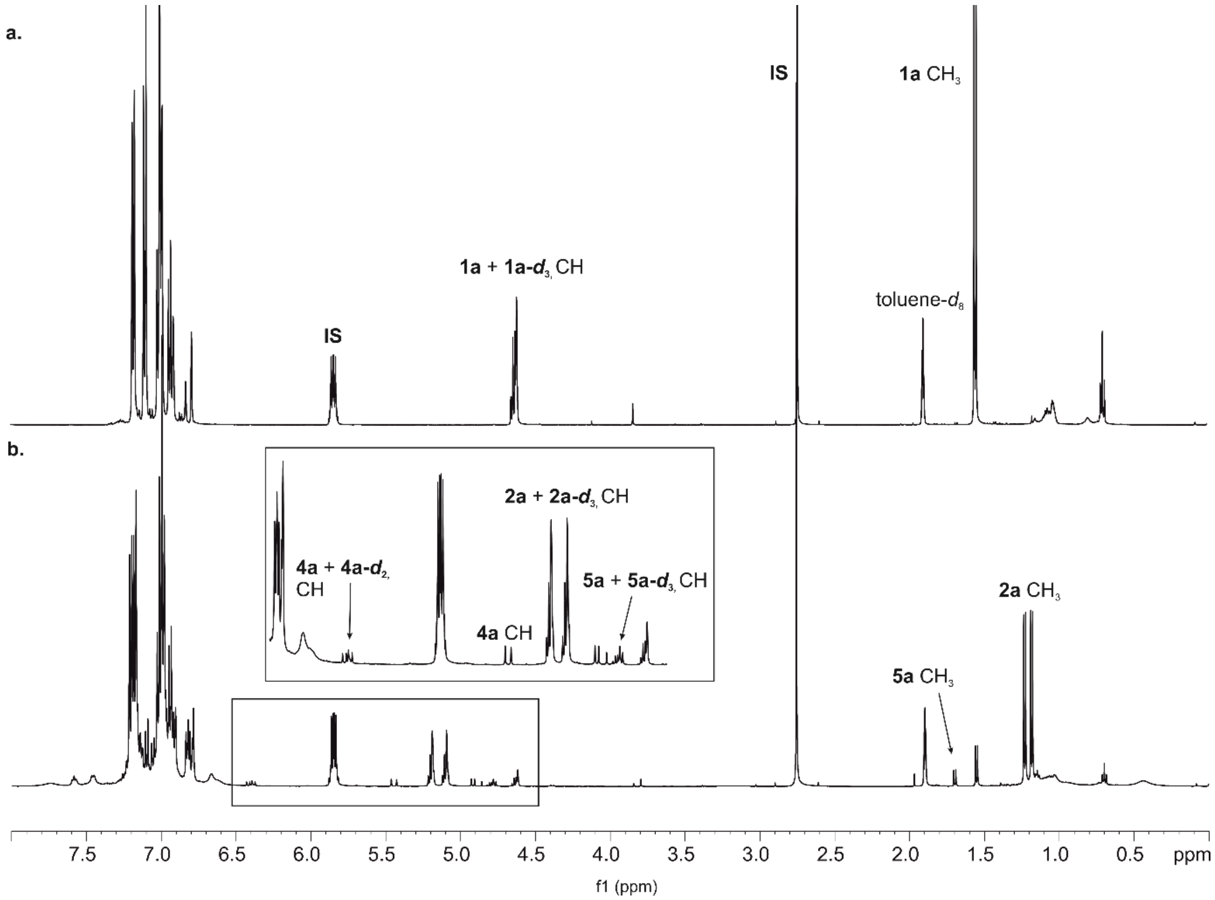


**Supplementary Figure 35**: Typical $^{1}H$ NMR spectra of the reaction mixture a) before the reaction start, b) when the reaction reached 90% conversion.

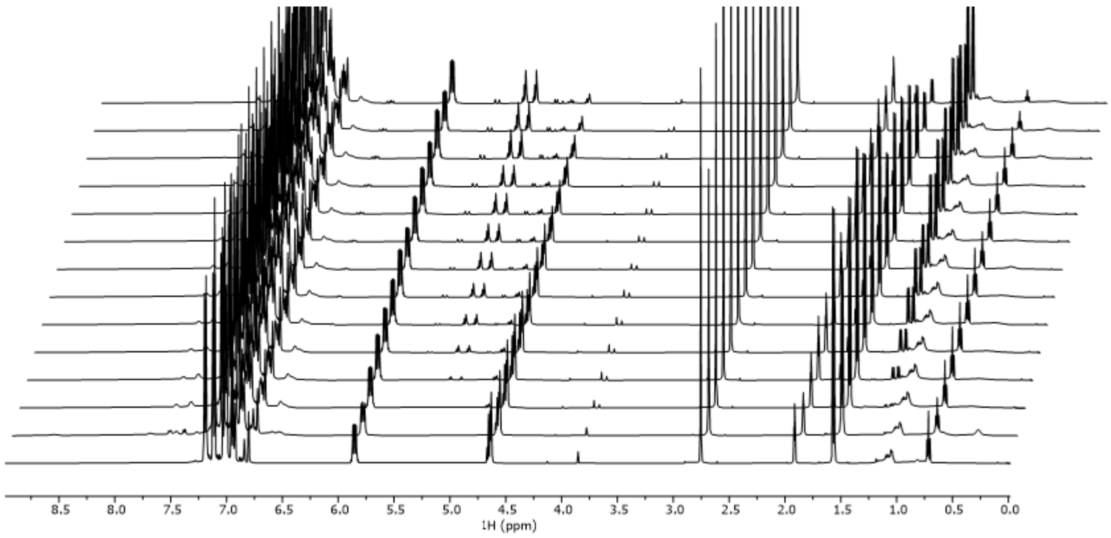


**Supplementary Figure 36**: The stacked $^{1}$H NMR spectra for competition experiment between **1a** and **1a-*d*$_3$**.

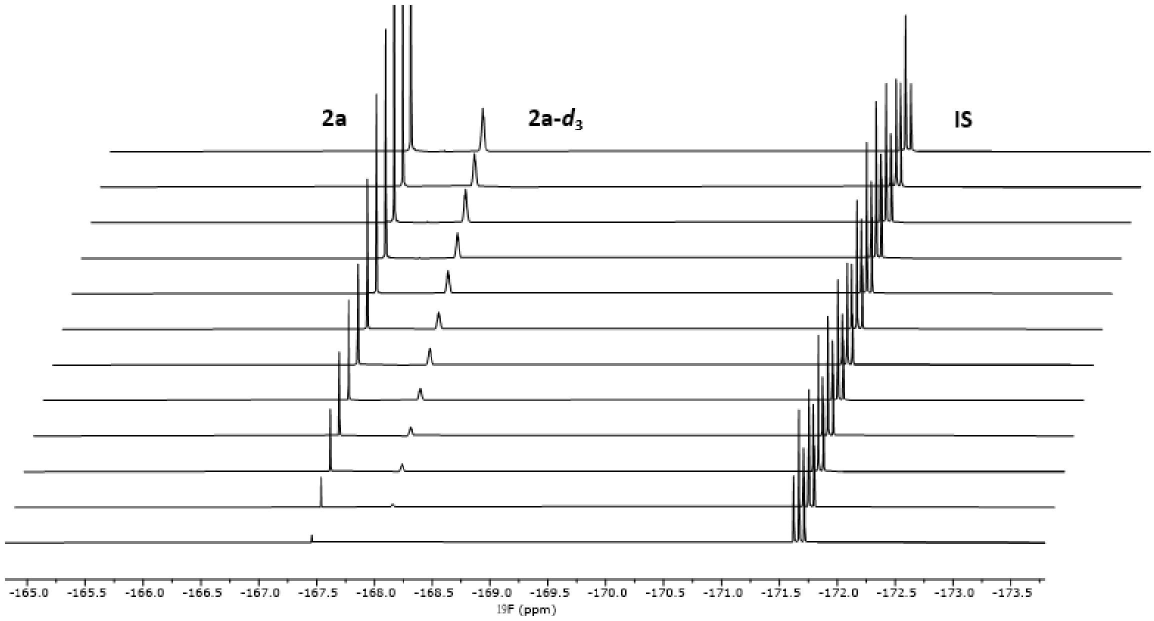


**Supplementary Figure 37**: The stacked $^{19}$F{$^{1}$H} NMR spectra.

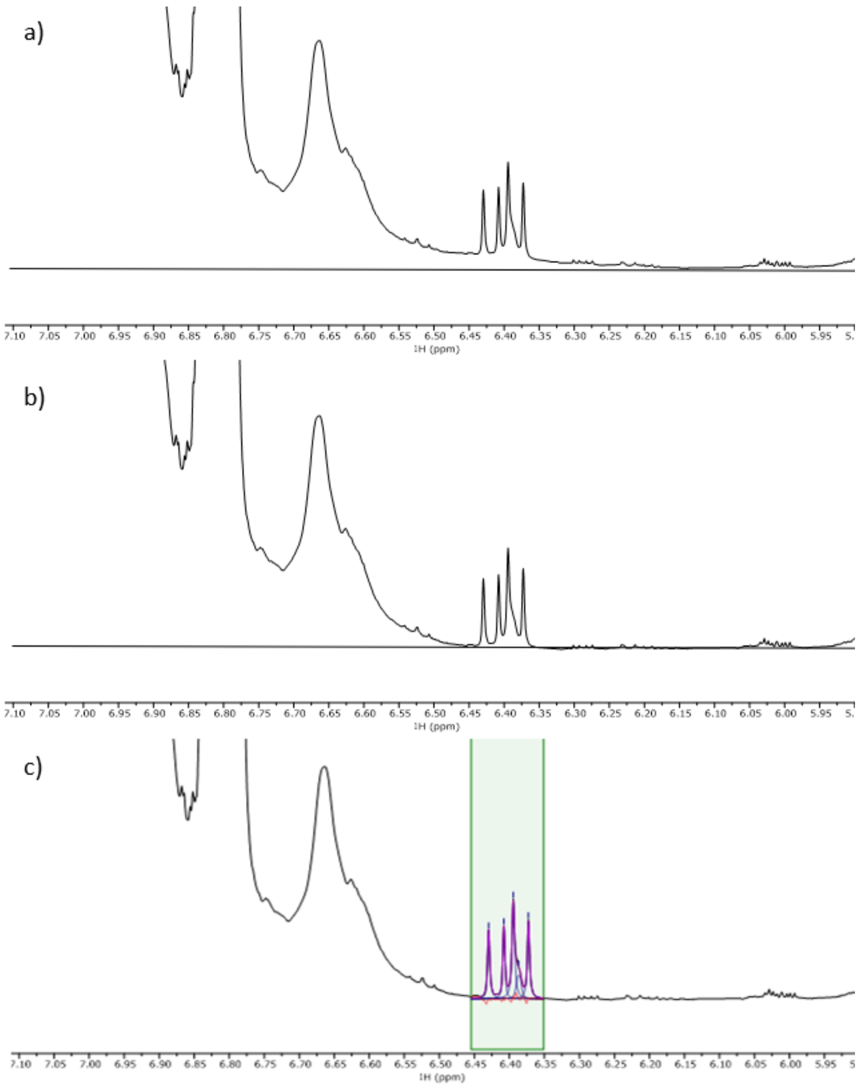


**Supplementary Figure 38**: An example of $^{1}$H NMR spectra for overlapping vinylic **4a** (dd) and **4a-*d*$_2$** (s) a) before regional baseline correction, b) after regional baseline correction, c) after line fitting.

Competition α-SKIE

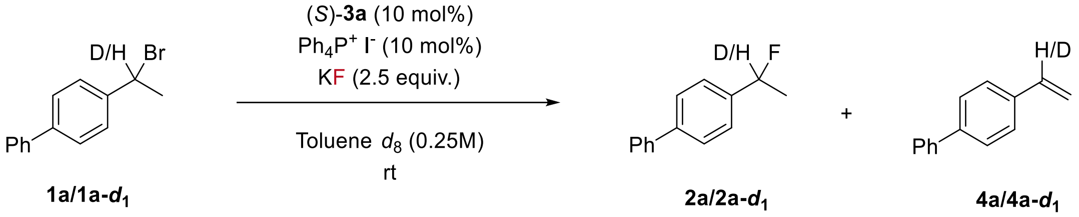


The pseudo first-order rate constants, $k_{obs(H)}$ and $k_{obs(D)}$ for the non-labelled and labelled substrates respectively, were estimated semi-logarithmically, ln([$A_0$]/[$A_t$]) = $k_{obs}$ $t$; where $A_0$ is the initial concentration of benzylic bromide, and $A_t$ is the concentration of benzylic bromide at time $t$. The same calculation was performed by considering both benzylic bromide and benzylic iodide as reactants, i.e., $A_0$ is the initial concentration of benzylic bromide, and $A_t$ is the total concentration of benzylic bromide and benzylic iodide at time $t$. These plots are shown in Supplementary Figure 39 a-d.

Elimination side products were formed during the reaction and the partition between addition and elimination was found to be approximately constant (supplementary figure 39 e-f). By using the partition ratios, $r_{A(H)}$ and $r_{A(D)}$, and $KIE_{net} = k_{obs(H)} / k_{obs(D)}$ (Supplementary Equations 3-5), the KIEs for the fluorination and the elimination can be estimated as shown below, with the results summarised in Supplementary Table 18.

**Supplementary Equation 3:**

$$KIE_{\mathrm{net}} = \frac{k_{\mathrm{F(H)}} + k_{\mathrm{Elim(H)}}}{k_{\mathrm{F(D)}} + k_{\mathrm{Elim(D)}}}\ ;\ r_{\mathrm{AH}} = \frac{k_{\mathrm{F(H)}}}{k_{\mathrm{Elim(H)}}}\ and\ r_{\mathrm{AD}} = \frac{k_{\mathrm{F(D)}}}{k_{\mathrm{Elim(D)}}}$$

**Supplementary Equation 4:**

$$\mathrm{KIE\ (F\text{–}\,addition)} \approx KIE_{\mathrm{net}}\left(\frac{1+\frac{1}{r_{\mathrm{AD}}}}{1+\frac{1}{r_{\mathrm{AH}}}}\right) = \left(\frac{1.38}{1.27}\right)\left(\frac{1+\frac{1}{13}}{1+\frac{1}{12}}\right) = 1.1$$

**Supplementary Equation 5:**

$$\mathrm{KIE\ (elimination)} \approx KIE_{\mathrm{net}}\left(\frac{1+\ r_{\mathrm{AD}}}{1+\ r_{\mathrm{AH}}}\right) = \left(\frac{1.38}{1.27}\right)\left(\frac{1+13}{1+12}\right) = 1.2$$

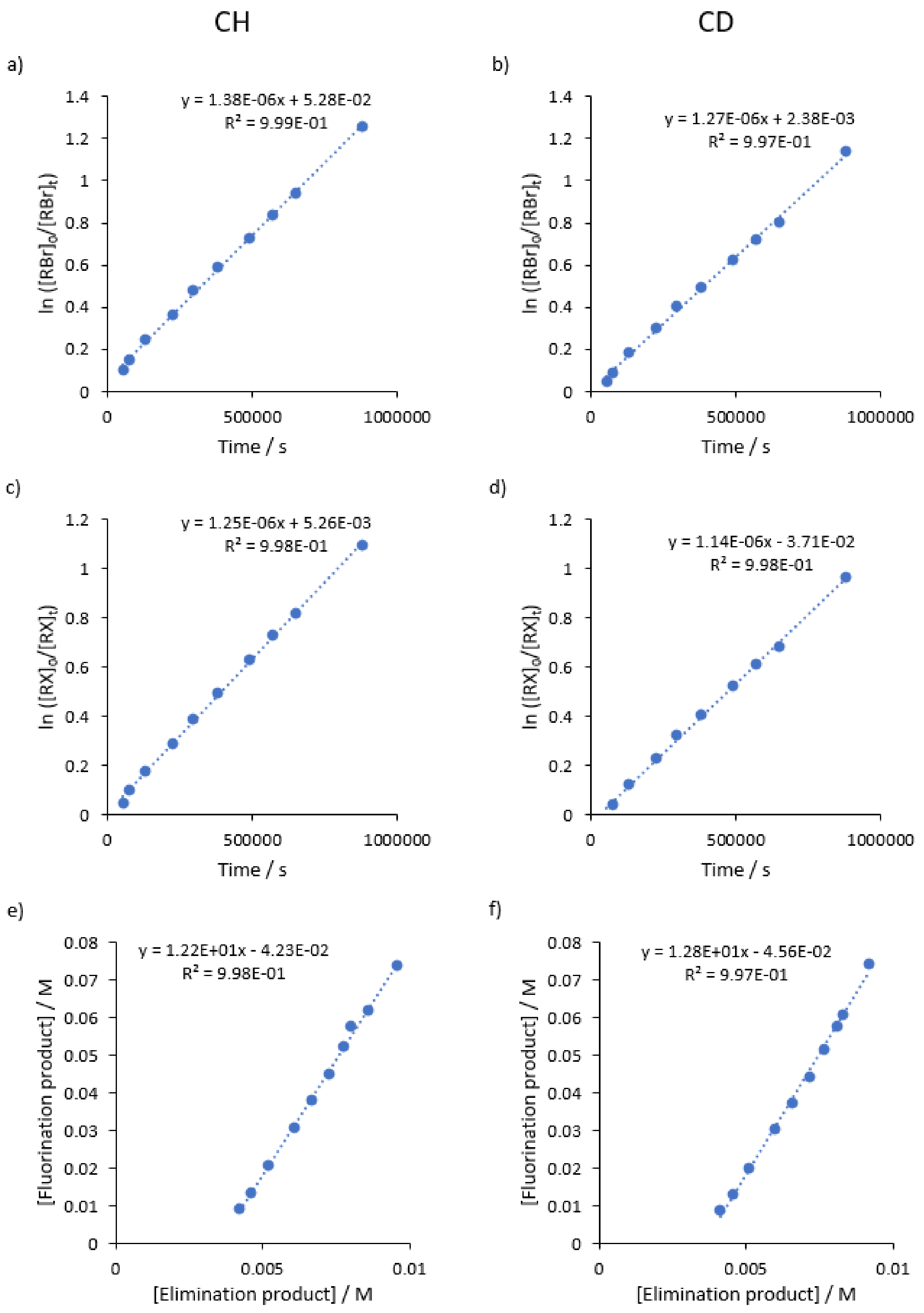


**Supplementary Figure 39**: Plots of ln([$A_0$]/[$A_t$]) against time for non-deuterated (a,c) and deuterated substrate (b,d); [RX] = [RBr]+[RI]. Partitions $r_{A(H)}$ and $r_{A(D)}$ for addition versus elimination for non-deuterated (e) and deuterated substrate (f).

Competition β-SKIE

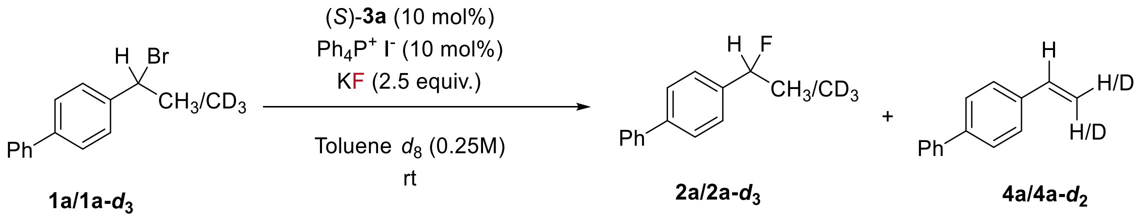


The same method was used for calculation of β-SKIE, with the key plots shown in Supplementary Figure 40 and the results summarised in Supplementary Table 18.

Independent experiments performing the reaction from the non-labelled (**1a**) and the labelled substrate (**1a-*d*$_3$**) were also performed, and the partition ratios, $r_{A(H)}$ and $r_{A(D)}$, for fluorination over elimination were found to be 10 and 27 respectively, i.e. the same as that observed in the competition β-SKIE experiment.

**Supplementary Equation 3:**

$$KIE_{\text{net}} = \frac{k_{\text{F(H)}} + k_{\text{Elim(H)}}}{k_{\text{F(D)}} + k_{\text{Elim(D)}}} \; ; \; r_{\text{AH}} = \frac{k_{\text{F(H)}}}{k_{\text{Elim(H)}}} \; and \; r_{\text{AD}} = \frac{k_{\text{F(D)}}}{k_{\text{Elim(D)}}}$$

**Supplementary Equation 4:**

$$\text{KIE (F– addition)} \approx KIE_{\text{net}} \left( \frac{1 + \frac{1}{r_{\text{AD}}}}{1 + \frac{1}{r_{\text{AH}}}} \right) = \left( \frac{1.56}{1.31} \right) \left( \frac{1 + \frac{1}{27}}{1 + \frac{1}{10}} \right) = 1.1$$

**Supplementary Equation 5:**

$$\text{KIE (elimination)} \approx KIE_{\text{net}} \left( \frac{1 + r_{\text{AD}}}{1 + r_{\text{AH}}} \right) = \left( \frac{1.56}{1.31} \right) \left( \frac{1 + 27}{1 + 10} \right) = 3.0$$

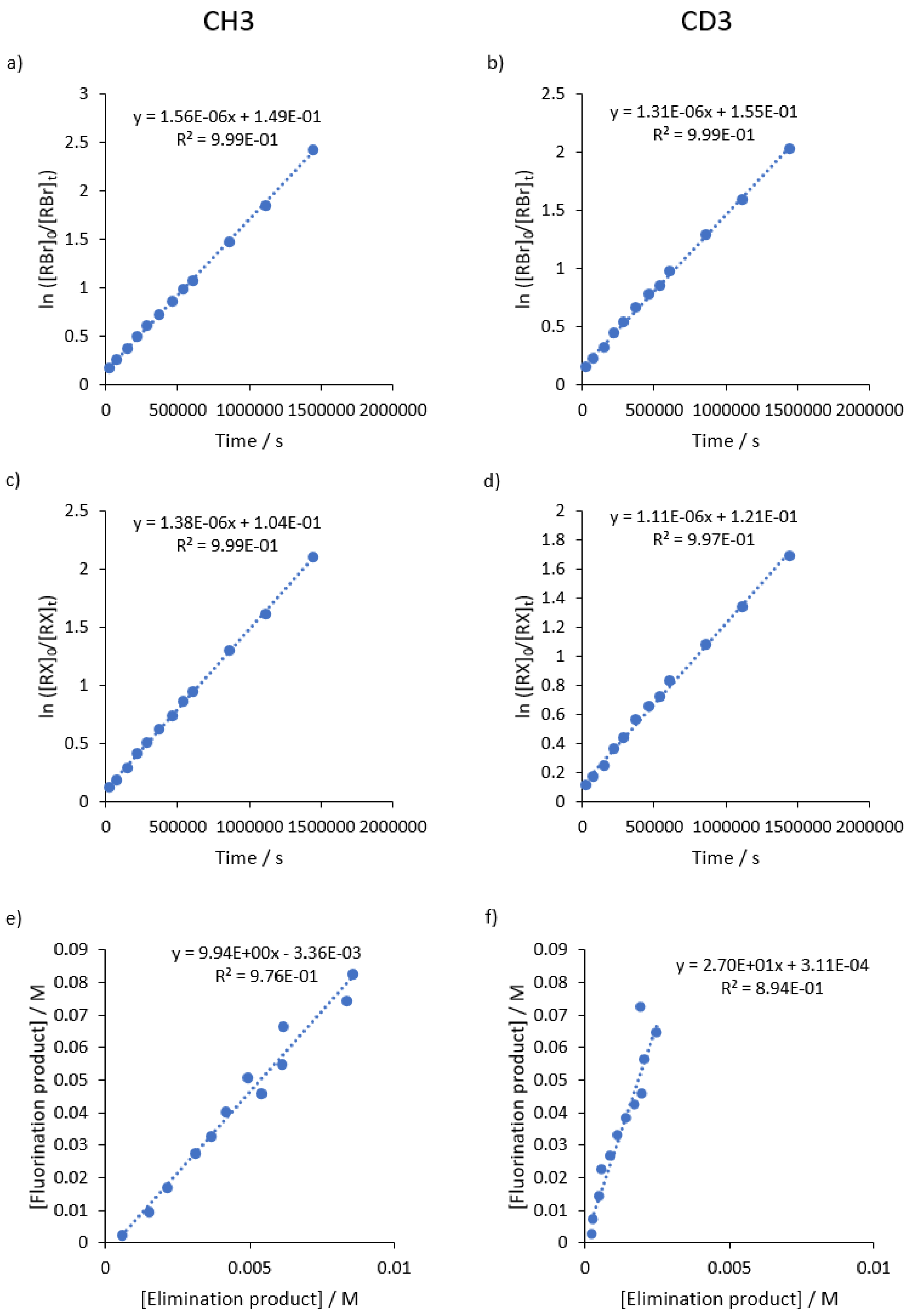


**Supplementary Figure 40**: Plots of ln([$A_0$]/[$A_t$]) against time for non-deuterated (a,c) and deuterated substrate (b,d); [RX] = [RBr]+[RI]. Partitions $r_{A(H)}$ and $r_{A(D)}$ for addition versus elimination for non-deuterated (e) and deuterated substrate (f).

Competition β-SKIE (with TBAF)

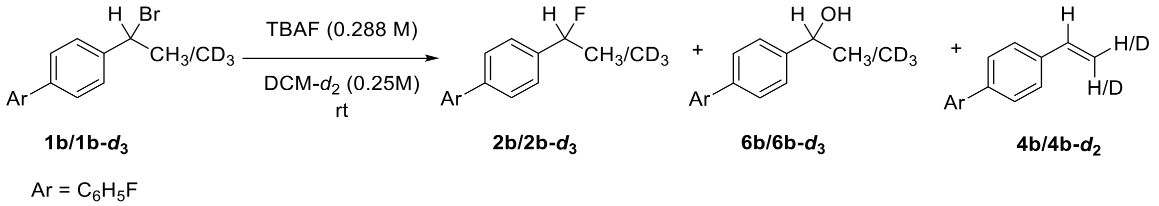


In this experiment, tetra-n-butylammonium fluoride (TBAF) was used in place of KF and catalysts for comparison. The reaction was performed in DCM-$d_2$ due to the poor solubility of TBAF in toluene. The homogeneous reaction was set up in a sealed NMR tube and left in the spectrometer taking quantitative $^1$H and $^{19}$F hourly over the course of the reaction. During the reaction, formation of secondary alcohols **6b** and **6b-*d*₃** were observed, and their identities were confirmed through spiking. The data obtained for this reaction did not correspond to simple pseudo first order decay, and the net KIE was calculated using the Bigeleisen-Wolfsberg equation (Supplementary Equation 6),[32,33] in which F is the total fractional conversion of the substrates (**1b** + **1b-$d_3$**) to products (**2b**, **2b-$d_3$**, **4b**, **4b-$d_2$**, **6b**, **6b-$d_2$**), $R_0$ is the initial ratio of labelled to unlabelled substrate ([**1a-$d_3$**]$_0$/[**1a**]$_0$) and R the same ratio measured after a given fractional conversion, F.

**Supplementary Equation 6:**

$$\frac{k_{\mathrm{H}}}{k_{\mathrm{D}}} = \frac{\ln\left((1-\mathrm{F})\left(\frac{1+\mathrm{R}_0}{1+\mathrm{R}}\right)\right)}{\ln\left((1-\mathrm{F})\left(\frac{\mathrm{R}}{\mathrm{R}_0}\right)\left(\frac{1+\mathrm{R}_0}{1+\mathrm{R}}\right)\right)}$$

The Bigeleisen-Wolfsberg equation (supplementary equation 6) can be rearranged to an equation that defines F, and the KIE then calculated by non-linear regression of R/$R_0$ versus F using the KIE as the fitting parameter. The fitting for R/$R_0$ against F is shown in Supplementary Figure 41. The overall KIE was estimated to be 1.93.

$$\mathrm{F} = 1 - \left(\frac{1+\mathrm{R}}{1+\mathrm{R}_0}\right)\left(\frac{\mathrm{R}}{\mathrm{R}_0}\right)^{\frac{\frac{k_{\mathrm{H}}}{k_{\mathrm{D}}}}{1-\frac{k_{\mathrm{H}}}{k_{\mathrm{D}}}}}$$

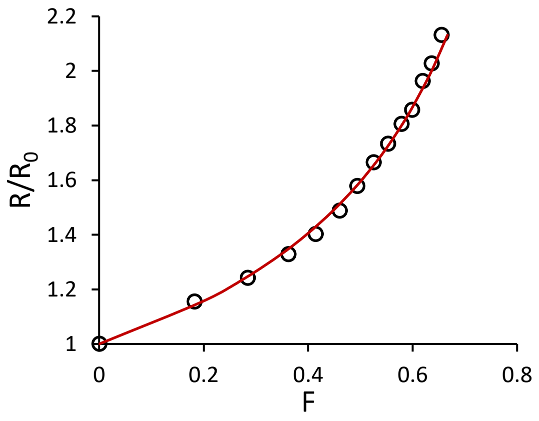


**Supplementary Figure 41**: R/$R_0$ versus fractional conversion, F, for the intermolecular competition of [**1a-$d_3$**] against [**1a**] for reaction with TBAF-hydrate in DCM. Experimental data (black open circles) and fitting of the Bigeleisen-Wolfsberg equation, when $KIE_{net}$ = 1.93 (red line).

The addition / elimination partition ratios, $r_H$ and $r_D$, were estimated by plotting the temporal concentrations of the addition products (**2b**, **2b-*d*$_3$**, **6b**, **6b-*d*$_2$**) against those of the elimination products (**4b**, **4b-*d*$_2$**), and fluoride addition / water addition partition ratios, $r_{FH}$ and $r_{FD}$, estimated by plotting the temporal concentrations of (**2b**, **2b-*d*$_3$**) against those of (**6b**, **6b-*d*$_2$**) (Supplementary Figure 42).

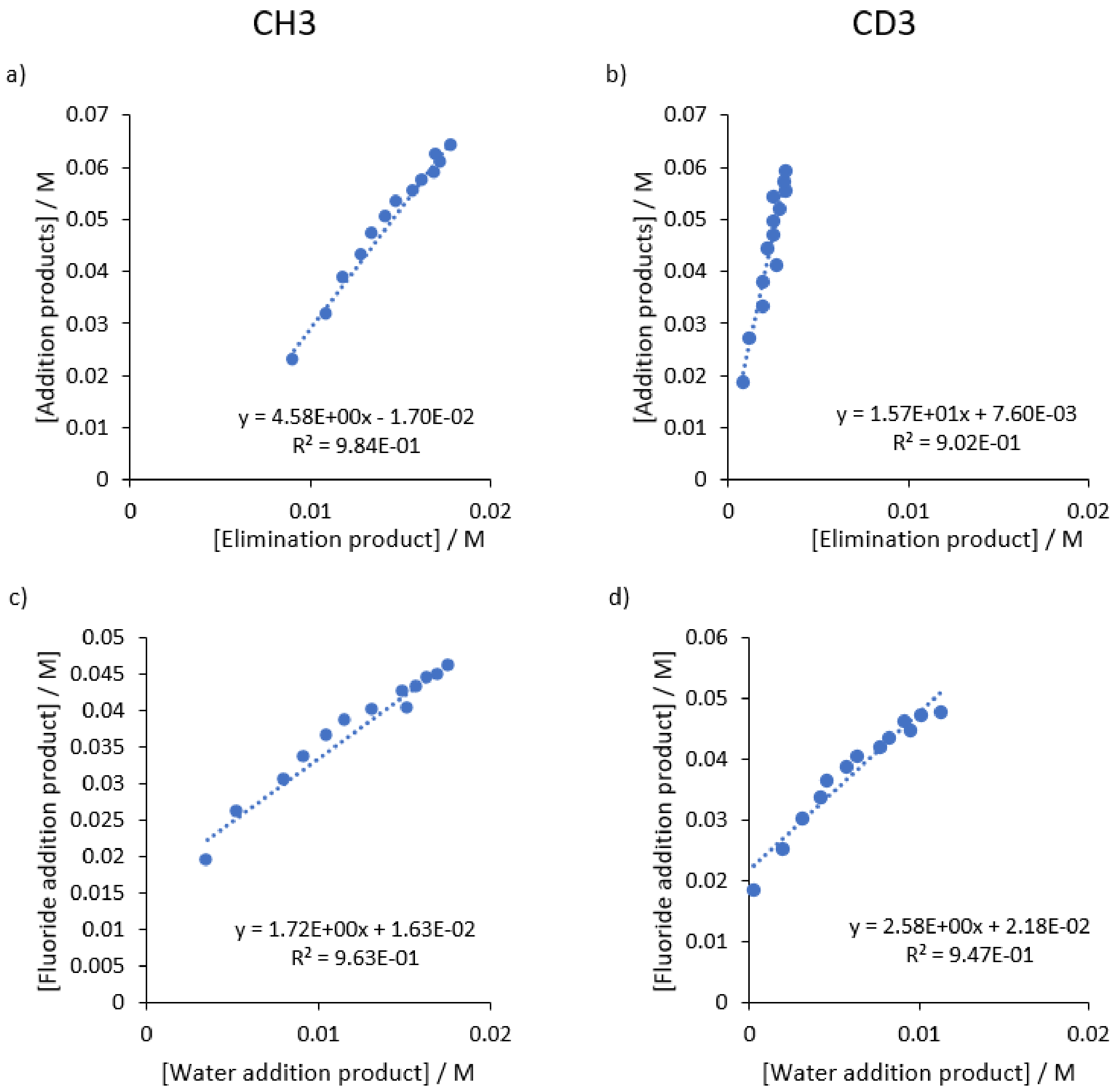


**Supplementary Figure 42**: Partitions $r_{A(H)}$ and $r_{A(D)}$ for addition versus elimination for non-deuterated (a) and deuterated substrate (b). Partitions $r_{F(H)}$ and $r_{F(D)}$ for fluoride addition versus water addition for non-deuterated (c) and deuterated substrate (d).

The value of $KIE_{net}$ from the Bigeleisen-Wolfsberg analysis was then used to estimate the KIEs for fluoride addition and elimination, based on the relationships below (Supplementary Equations 7 – 10).

**Supplementary Equation 7:**

$$r_{\mathrm{FH}} = \frac{k_{\mathrm{F(H)}}}{k_{\mathrm{H2O(H)}}}; r_{\mathrm{AH}} = \frac{k_{\mathrm{F(H)}} + k_{\mathrm{H2O(H)}}}{k_{\mathrm{Elim(H)}}}; r_{\mathrm{FD}} = \frac{k_{\mathrm{F(D)}}}{k_{\mathrm{H2O(D)}}}; r_{\mathrm{AD}} = \frac{k_{\mathrm{F(D)}} + k_{\mathrm{H2O(D)}}}{k_{\mathrm{Elim(D)}}}$$

**Supplementary Equation 8:**

$$KIE_{\mathrm{net}} = \frac{k_{\mathrm{F(H)}} + k_{\mathrm{H2O(H)}} + k_{\mathrm{Elim(H)}}}{k_{\mathrm{F(D)}} + k_{\mathrm{H2O(D)}} + k_{\mathrm{Elim(D)}}}$$

**Supplementary Equation 9:**

$$\mathrm{KIE\ (F\text{–}\,addition)} \approx KIE_{\mathrm{net}} \frac{1 + \left(\frac{1 + \frac{1}{r_{\mathrm{FD}}}}{r_{\mathrm{AD}}}\right) + \left(\frac{1}{r_{\mathrm{FD}}}\right)}{+\left(\frac{1 + \frac{1}{r_{\mathrm{FH}}}}{r_{\mathrm{AH}}}\right) + \left(\frac{1}{r_{\mathrm{FH}}}\right)} = 1.93 \frac{1 + \left(\frac{1 + \frac{1}{2.6}}{15.7}\right) + \left(\frac{1}{2.6}\right)}{1 + \left(\frac{1 + \frac{1}{1.7}}{4.6}\right) + \left(\frac{1}{1.7}\right)} = 1.5$$

**Supplementary Equation 10:**

$$\mathrm{KIE\ (elimination)} \approx KIE_{\mathrm{net}} \left(\frac{1 + r_{\mathrm{AD}}}{1 + r_{\mathrm{AH}}}\right) = 1.93 \left(\frac{1 + 15.7}{1 + 4.6}\right) = 5.8$$

It should be noted that the analysis assumes first-order competition between the labelled and unlabelled substrate, that the competing reactions are irreversible, and the net KIE is constant with conversion. The stoichiometry and kinetics of the competing hydrolysis and the potential impact of an accumulating bromide common ion-effect on fluoride addition and elimination $S_N1$/E1-like pathways, may therefore affect the validity of these assumptions.

**Calculation of KIEs**

Supplementary Table 18: A summary of KIEs:

| | | KIE |
|---|---|---|
| **Competition α-SKIE** | Fluorination | 1.1 |
| | Elimination | 1.2 |
| **Competition β-SKIE** | Fluorination | 1.1 |
| | Elimination | 3.0 |
| **Competition β-SKIE (with TBAF)** | Fluorination | 1.5 |
| | Elimination | 5.8 |

## Kinetic Isotope Effect Predictions

KIE values were predicted from DFT computed stationary points using the open-source program KINISOT.[34] Stationary points and harmonic frequencies were computed at the M06-2X/def2-SV(P)+def2-TZVPPD level of theory with CPCM *p*-xylene. For this computational analysis, we used the $S_N2$ transition structures formed with benzyl bromide and urea-fluoride $[UF]^-$ complex and the preceding complex formed between these species. We repeated this analysis for both diastereomeric pathways, and with benzyl iodide as electrophile. Secondary - and β- $^{1}H/^{2}H$ KIE values were obtained using the Bigeleisen-Mayer equation, using $PhC(D)XCH_3$ and $PhCHXCD_3$ isotopologues (X = Br or I), respectively, as were studied experimentally. A one-dimensional tunneling correction, the Bell infinite-parabola model, was also included. No vibrational scaling factors were applied. At this level of theory, transition structures corresponding to an $S_N1$ pathway cannot be located.

Supplementary Table 19: Predicted KIEs from computed transition structures for the fluorination of benzylic bromide and iodide electrophiles. Corrected KIE values includes a 1D-tunneling correction:

| | KIE | Corrected α-SKIE | KIE | Corrected β-SKIE |
|---|---|---|---|---|
| **3f·F + (*S*)-bromide** | 1.02 | 1.02 | 1.03 | 1.03 |
| **3f·F + (*R*)-bromide** | 1.03 | 1.03 | 1.04 | 1.05 |
| **3f·F + (*S*)-iodide** | 1.02 | 1.02 | 1.04 | 1.05 |
| **3f·F + (*R*)-iodide** | 1.03 | 1.03 | 1.05 | 1.06 |

Effect of using $Ph_4P^+ Br^-$

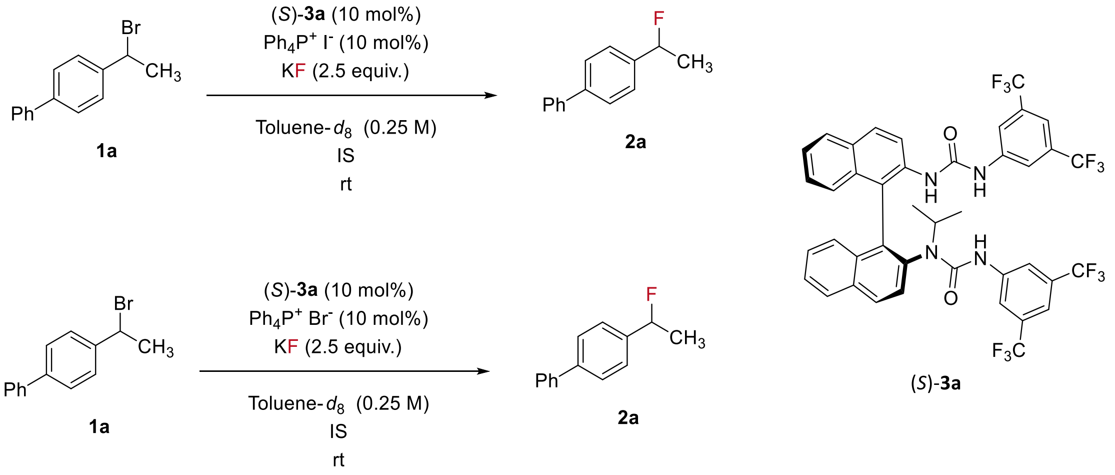


The effect of using $Ph_4P^+ Br^-$ instead of $Ph_4P^+ I^-$ was investigated by running two individual experiments following the general procedure. To minimise the effect of changing mixing efficient and temperature on the reaction rate, these two NMR tubes was secured on an external motor together and the two reactions were performed and monitored at the same time. The overall rate of reaction was estimated by plotting ln([$RBr_0$]/[$RBr_t$]) against time, and the plots are shown in Supplementary Figure 43.

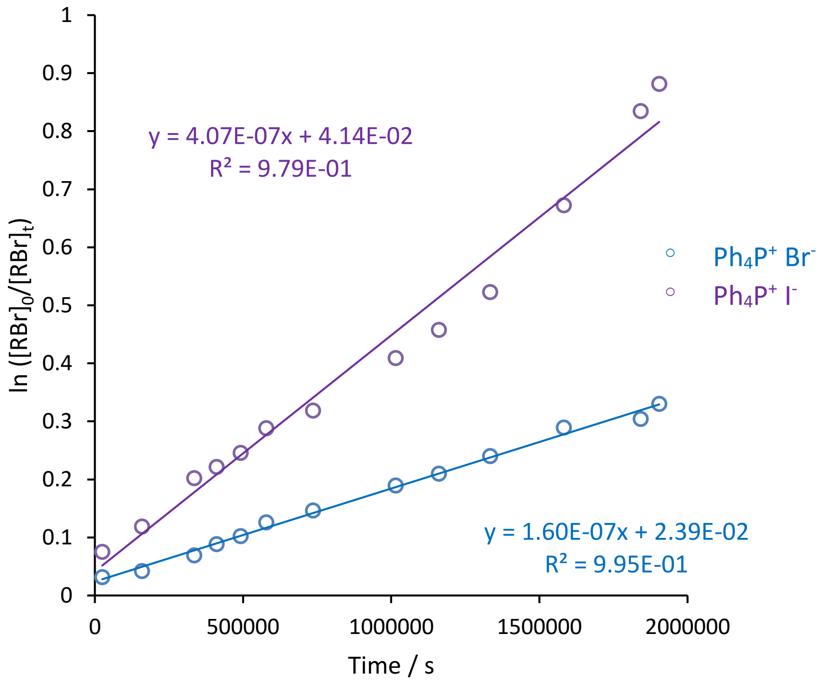


**Supplementary Figure 43**: Plots of ln([$RBr_0$]/[$RBr_t$]) against time for reactions with $Ph_4P^+ Br^-$ (blue) and $Ph_4P^+ I^-$ (purple).

## Mechanistic NMR Experiments

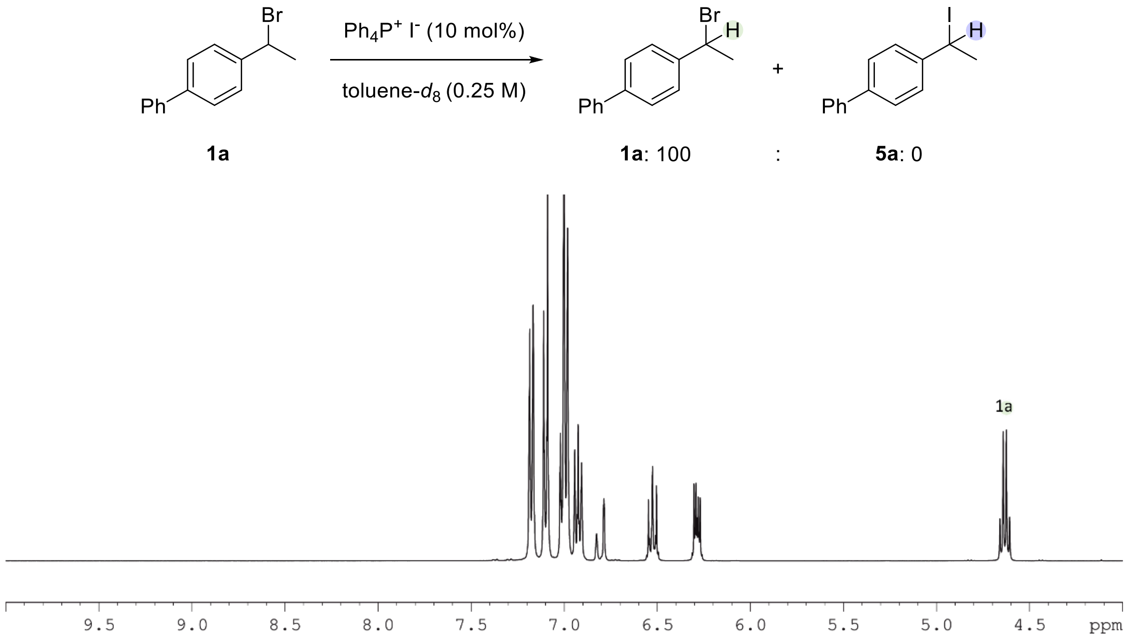


**Supplementary Figure 44**: Investigating iodide (**5a**) formation from bromide (**1a**): $^{1}$H spectrum of *rac*-**1a** (0.125 mmol) + $Ph_4P^+$ $I^-$ (10 mol%) in toluene-$d_8$ (25 mM, 298 K) sonicated for 30 minutes showing no formation of **5a**

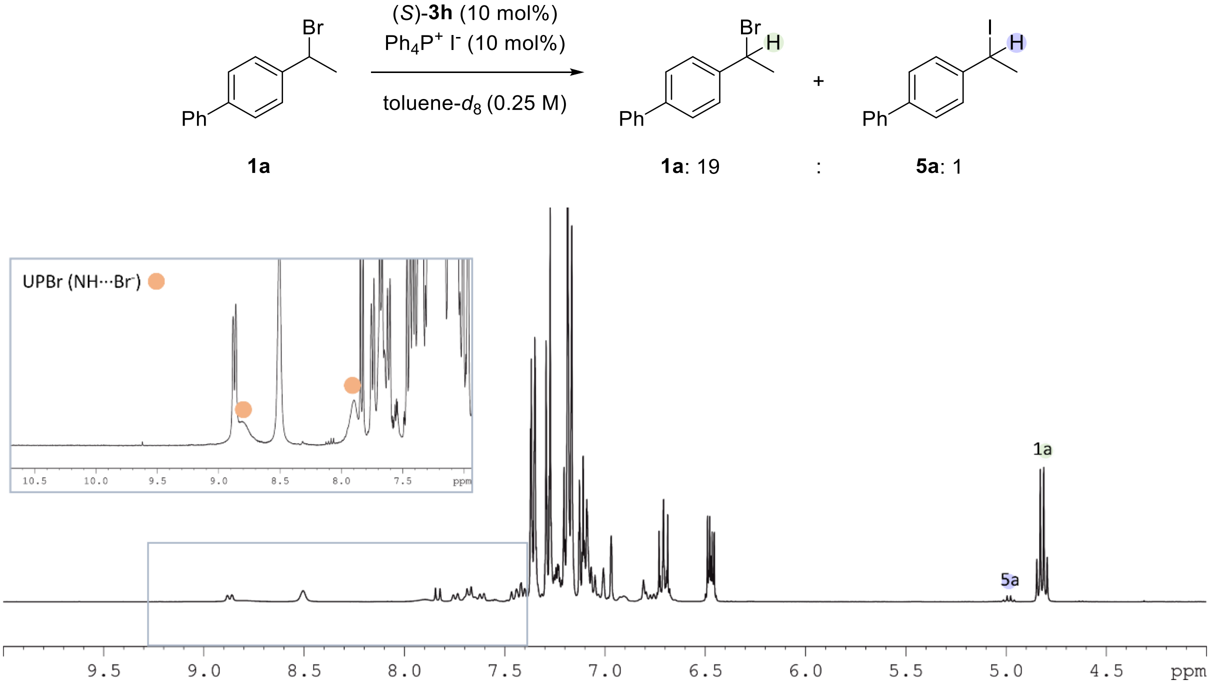


**Supplementary Figure 45**: Investigating iodide (**5a**) formation from bromide (**1a**): $^{1}$H spectrum of *rac*-**1a** (0.125 mmol) + (*S*)-**3h** (10 mol%) + $Ph_4P^+$ $I^-$ (10 mol%) in toluene-$d_8$ (25 mM, 298 K) sonicated for 30 minutes; showing the formation of iodide **5a** (inset – evidence of urea NH resonances deshielding through binding to resultant phosphonium bromide: UPBr complex)

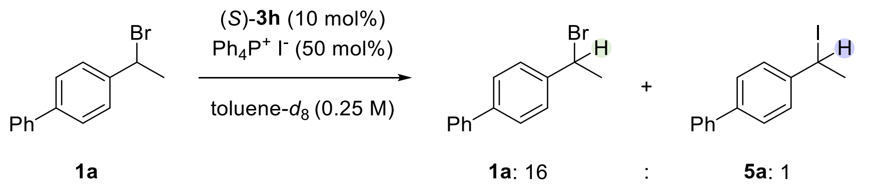


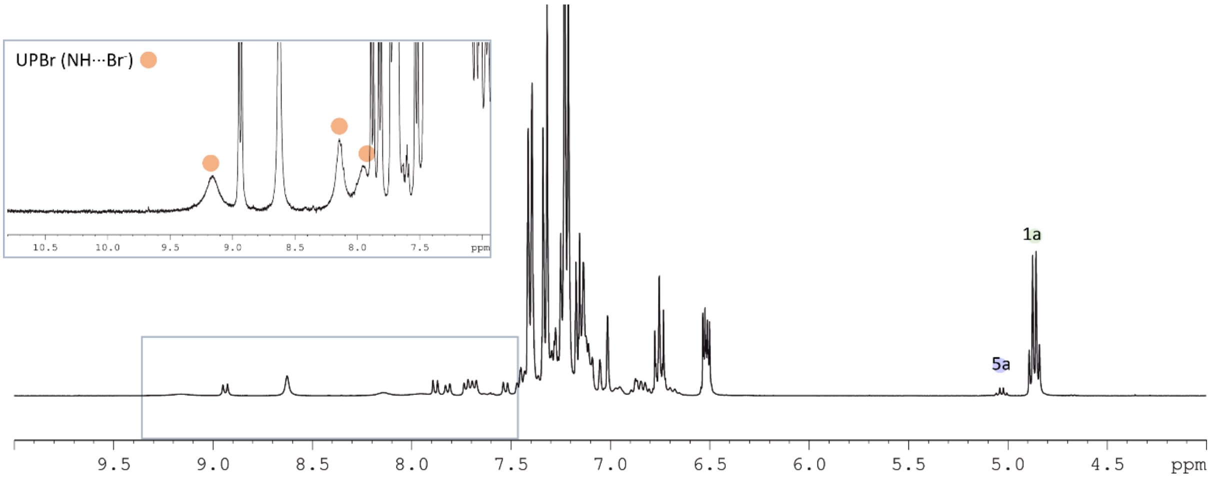


**Supplementary Figure 46**: Investigating iodide (**5a**) formation from bromide (**1a**): $^{1}H$ spectrum of *rac*-**1a** (0.125 mmol) + (*S*)-**3h** (10 mol%) + $Ph_4P^+ I^-$ (50 mol%) in toluene-$d_8$ (25 mM, 298 K) sonicated for 30 minutes; showing increased concentration of iodide **5a** (inset – evidence of urea NH resonances deshielding through binding to resultant phosphonium bromide: UPBr complex)

**Phase Transfer of KF by Urea-Phosphonium-Halide Complexes**

(*S*)-**3h** + $Ph_4P^+$ $Br^-$ + KF → [UPBr] + [UPF]

*Sample preparation*: Urea catalyst (*S*)-**3h** (0.0125 mmol), $Ph_4P^+$ $Br^-$ (0.0125 mmol) and KF (0.313 mmol) added to NMR tube, toluene-$d_8$ was added (25 mM concentration) and tube was sonicated for 30 minutes.

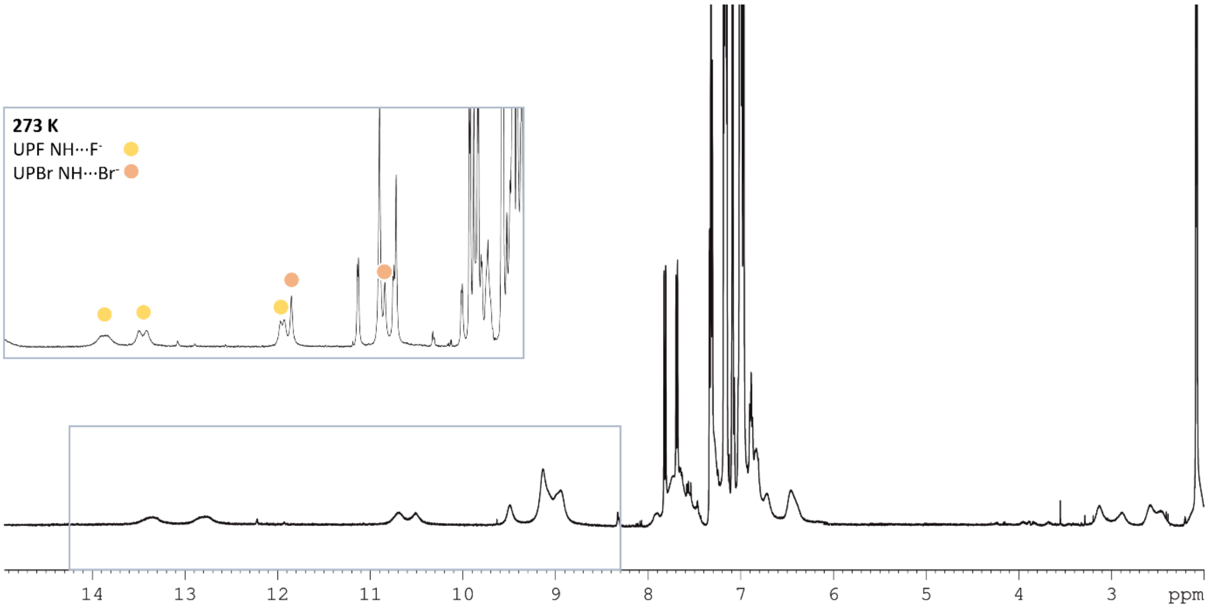


**Supplementary Figure 47**: $^1H$ NMR of (*S*)-**3h** + $Ph_4P^+$ $Br^-$ + KF. (inset - showing deshielding of the urea NH resonances through the formation of both a UPBr complex as well exchange to a UPF complex characterised through scalar coupling to fluoride (500 MHz, Toluene-$d_8$, 25 mM, 298 K).

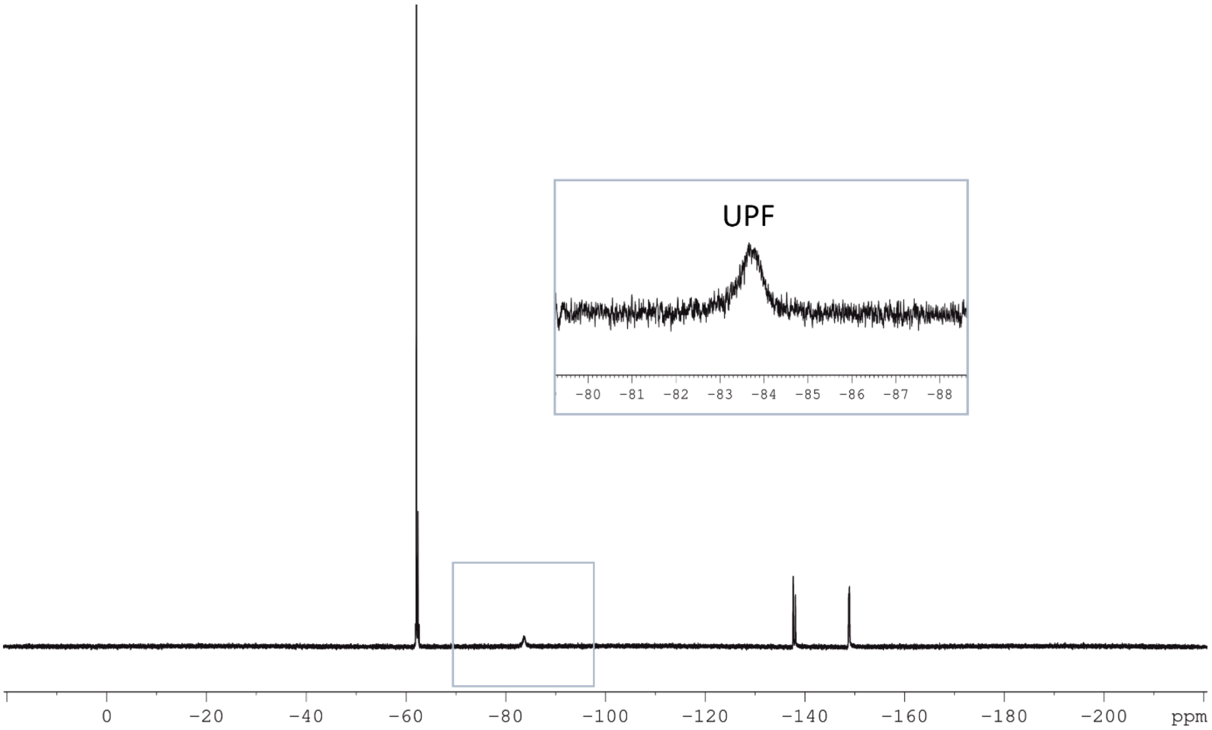


**Supplementary Figure 48**: $^{19}F$ NMR of (*S*)-**3h**: $Ph_4P^+$: $Br^-$ + KF showing a strong fluoride resonance at -84 ppm indicative of the formation of a UPF complex (471 MHz, Toluene-$d_8$, 25 mM, 298 K).

($S$)-**3h** + $Ph_4P^+$ $I^-$ + KF → [UPI] (no [UPF] formation)

*Sample preparation*: Urea catalyst ($S$)-**3h** (0.0125 mmol), $Ph_4P^+$ $I^-$ (0.0125 mmol) and KF (0.313 mmol) added to NMR tube, toluene-$d_8$ was added (25 mM concentration) and tube was sonicated for 30 minutes.

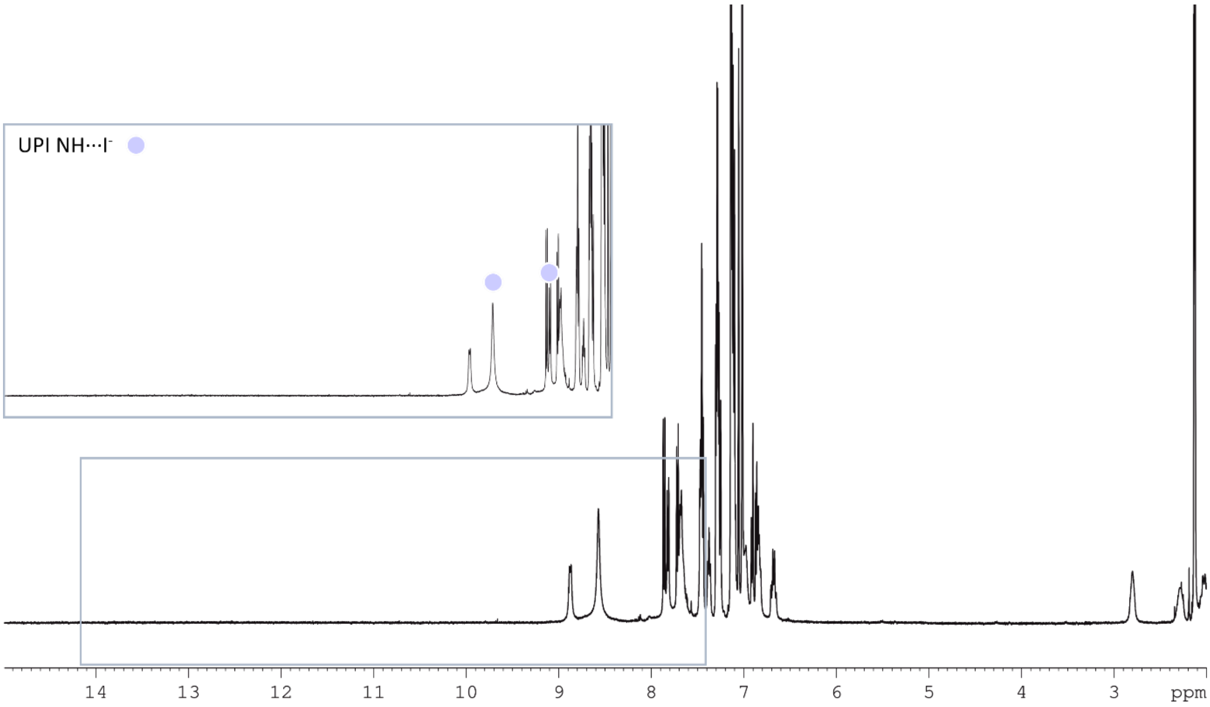


**Supplementary Figure 49**: $^1$H NMR of ($S$)-**3h** + $Ph_4P^+$ $I^-$ + KF. (inset - showing deshielding of the urea NH resonances through the formation of both a UPI complex, no exchange forming UPF complex detected (500 MHz, Toluene-$d_8$, 25 mM, 298 K).

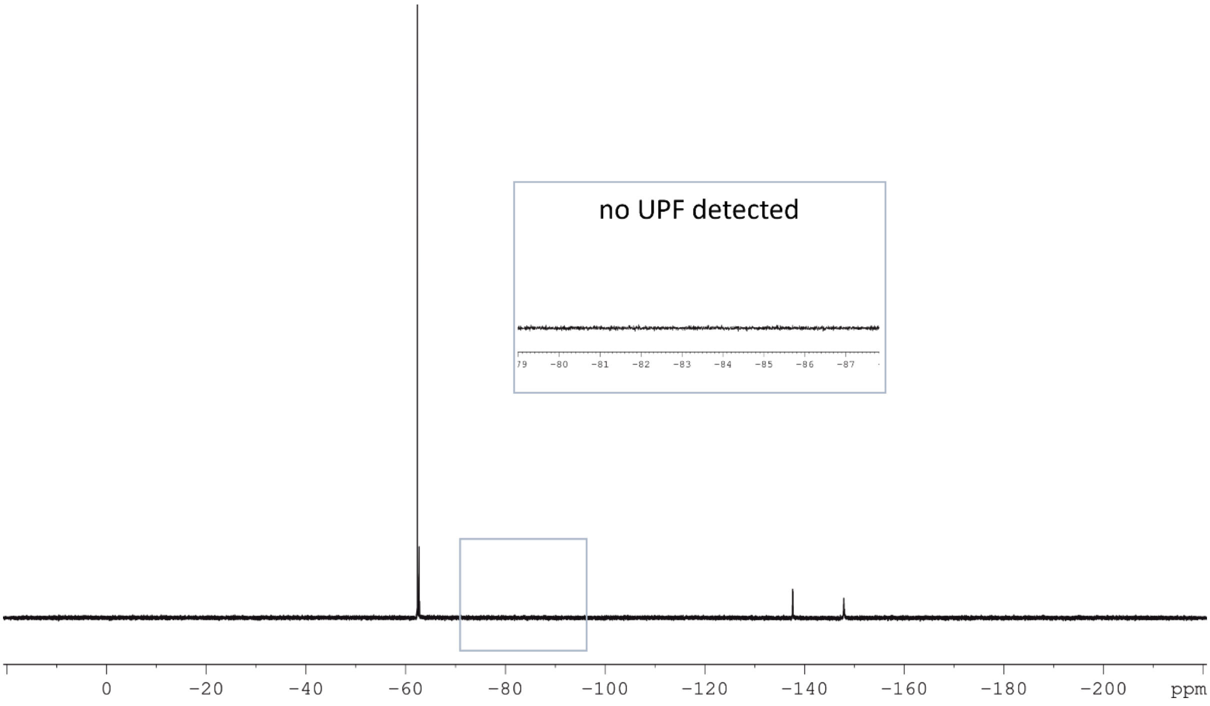


**Supplementary Figure 50**: $^{19}$F NMR of ($S$)-**3h**: $Ph_4P^+$: $I^-$ + KF (inset – no fluoride resonance at ~84 ppm suggesting no formation of a UPF complex (471 MHz, Toluene-$d_8$, 25 mM, 298 K).

($S$)-**3h**: $Ph_4P^+$: $F^-$ [UPF] complex + *rac*-1a → **2a** + **4a**

*Sample preparation*: Urea catalyst ($S$)-**3h** (0.0125 mmol), $Ph_4P^+$ $BF_4^-$ (0.0125 mmol) and KF (0.313 mmol) were added to NMR tube, toluene-$d_8$ added and tube was sonicated for 30 minutes to form [UPF] species. (*rac*)-**1a** (0.125 mmol) was then added to the NMR tube and sonicated for 30 additional minutes showing the formation of fluoride product **2a** (Fig. S50 and S51).

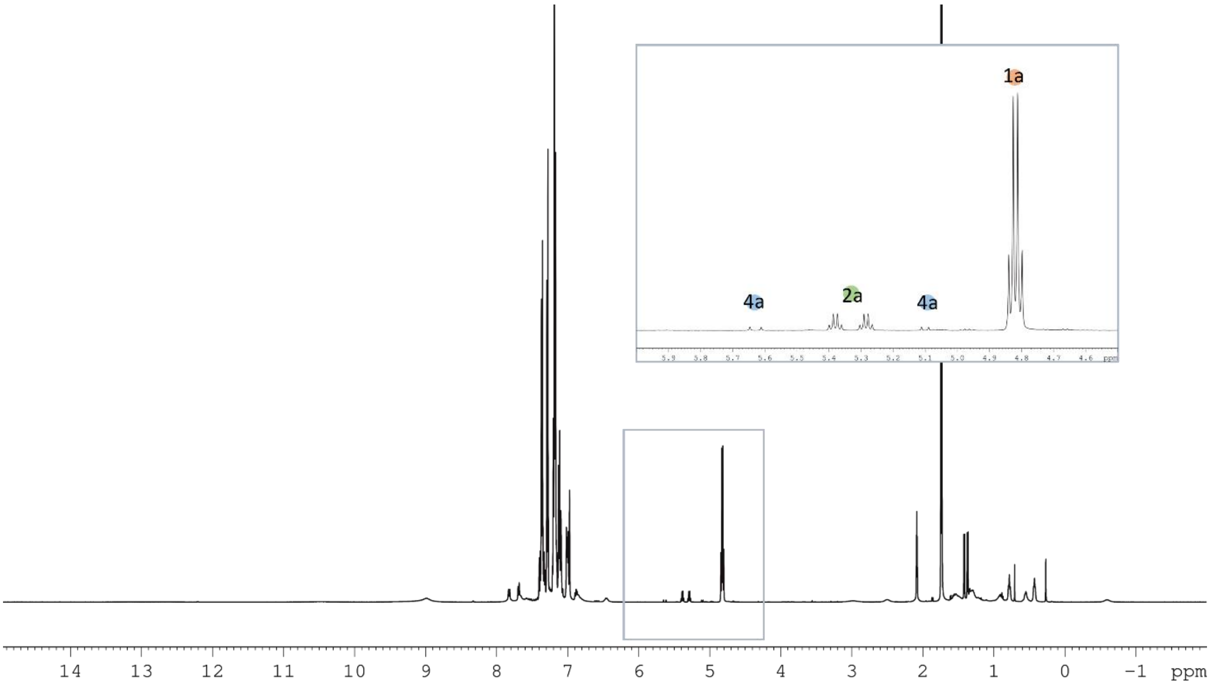


**Supplementary Figure 51**: $^1$H NMR ($S$)-**3h**: $Ph_4P^+$: $F^-$ (UPF) + *rac*-**1a** after 30 minutes sonication (inset- showing the formation of fluoride **2a,** alkene **4a**, as well as remaining bromide **1a**) (500 MHz, Toluene-$d_8$, 25 mM, 298 K).

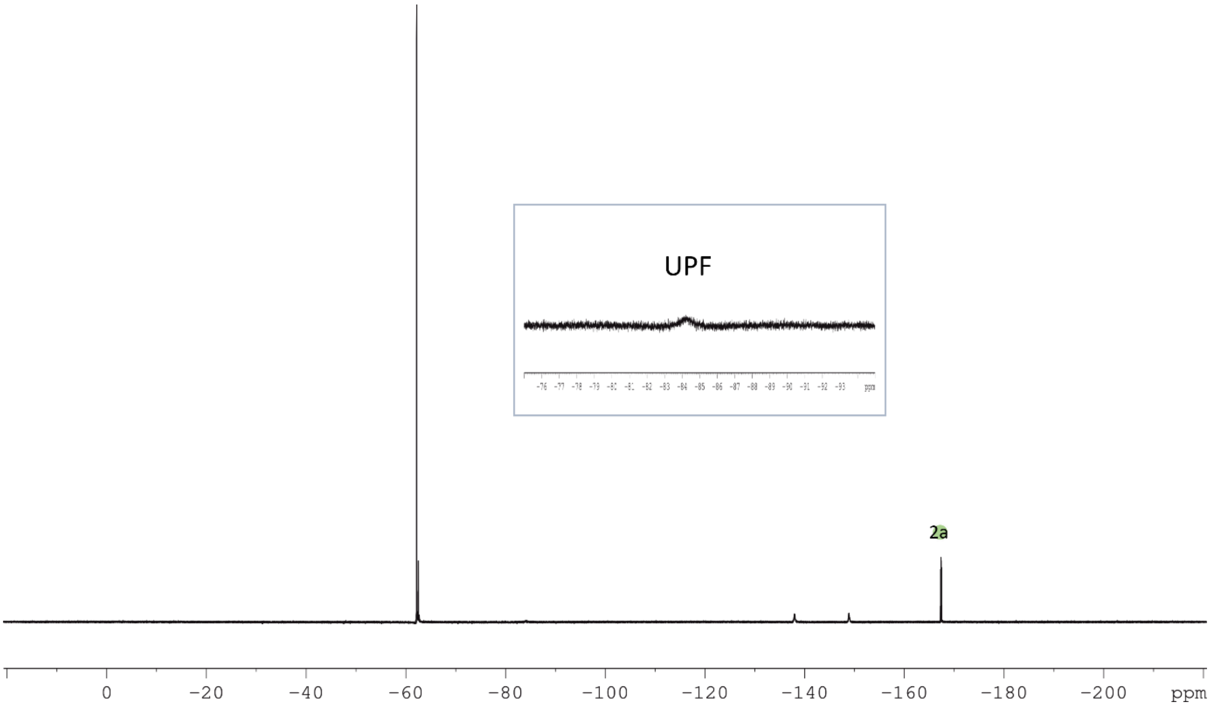


**Supplementary Figure 52**: $^{19}$F NMR ($S$)-**3h**: $Ph_4P^+$: $F^-$ + *rac*-**1a** after 30 minutes sonication showing the formation of fluoride **2a** (inset – showing weak fluoride resonance at -84 ppm indicating presence of UPF complex) (500 MHz, Toluene-$d_8$, 25 mM, 298 K).

**Reaction From Stoichiometric UPF complex**

*Sample preparation:* Urea catalyst (*S*)-**3h** (0.05 mmol), $Ph_4P^+$ $BF_4^-$ (0.05 mmol) and KF (0.125 mmol) were added to a vial, toluene (0.5 mL) was added and stirred at 60 °C, 1200 rpm for 18 hours. The reaction was filtered over celite, washed with DCM and solvent was removed under reduced pressure to form UPF complex as a white crystalline solid, confirmed by NMR.

The UPF complex was added as a stoichiometric reagent (28.5 mg, 0.025 mmol) into a vial with bromide **1a** (0.025 mmol), *p*-xylene (0.1 mL) was added and the reaction was left to stir at 40 °C, 48 h.

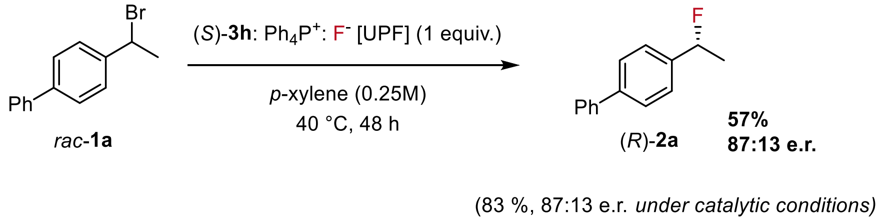


**Supplementary Figure 53**: Reaction performed from UPF complex (1 equiv.)

**Control Reactions from α-Iodoketone substrate**

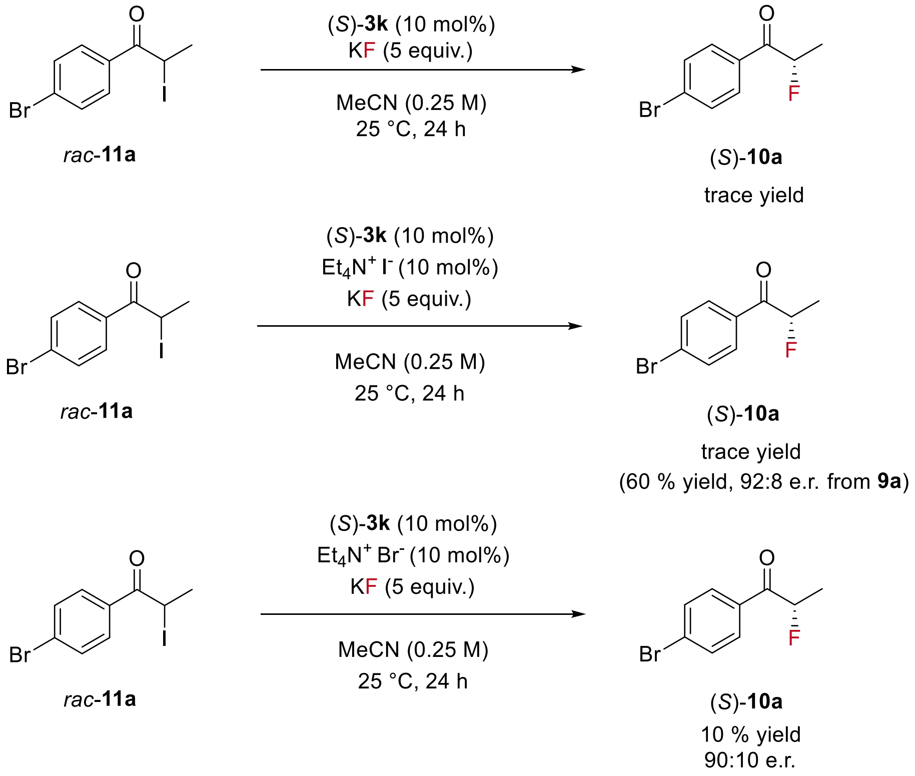


**Supplementary Figure 54**: Reactions performed from α-iodoketone substrate **11a**

## Substrate Racemisation Studies

Enantioenriched bromide **1a** was prepared according to literature procedure[35] in 63:37 e.r., and subjected to the following conditions.

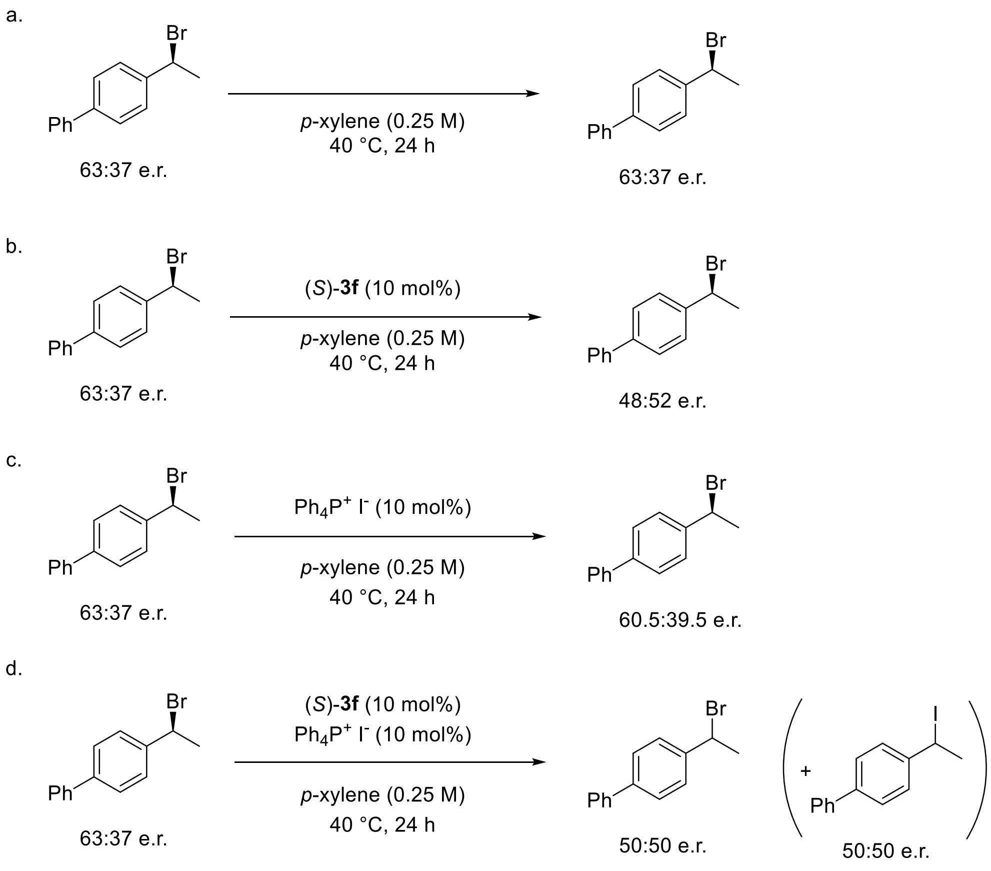


**Supplementary Figure 55**: Racemisation studies performed on enantioenriched substrate

**a.**

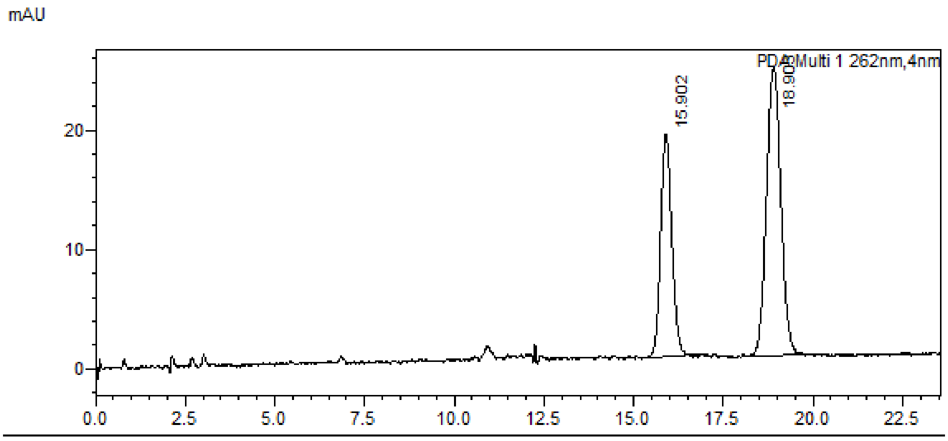


PDA Ch1 262nm

| Peak# | Ret. Time | Area% |
|---|---|---|
| 1 | 15.902 | 37.087 |
| 2 | 18.903 | 62.913 |

**b.**

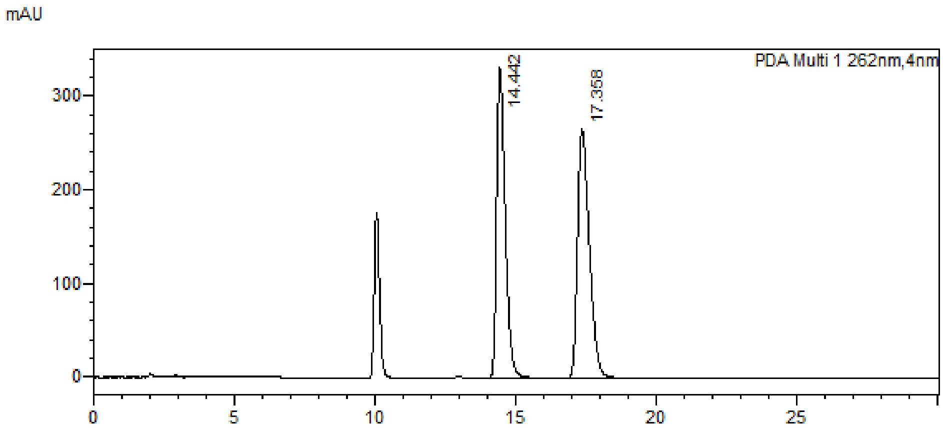


PDA Ch1 262nm

| Peak# | Ret. Time | Area% |
|---|---|---|
| 1 | 14.442 | 47.721 |
| 2 | 17.358 | 52.279 |

**c.**

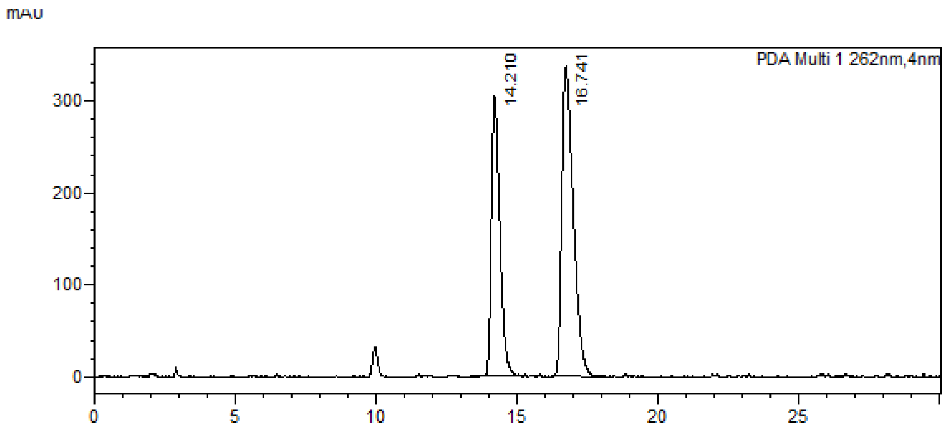


PDA Ch1 262nm

| Peak# | Ret. Time | Area% |
|---|---|---|
| 1 | 14.210 | 39.504 |
| 2 | 16.741 | 60.496 |

**d.**

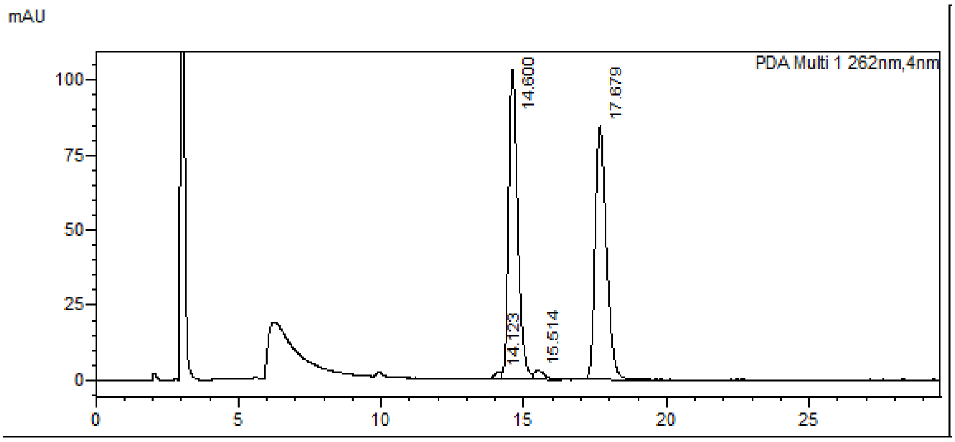


PDA Ch1 262nm

| Peak# | Ret. Time | Area% |
|---|---|---|
| 1 | 14.123 | 0.789 |
| 2 | 14.600 | 48.852 |
| 3 | 15.514 | 1.553 |
| 4 | 17.679 | 48.806 |

## Monitoring of Enantiomeric Ratios Over Time

Supplementary Table 20: *Ex situ* monitoring of the enantiomeric ratio of **1a** and **2a** over the time-course of reaction.

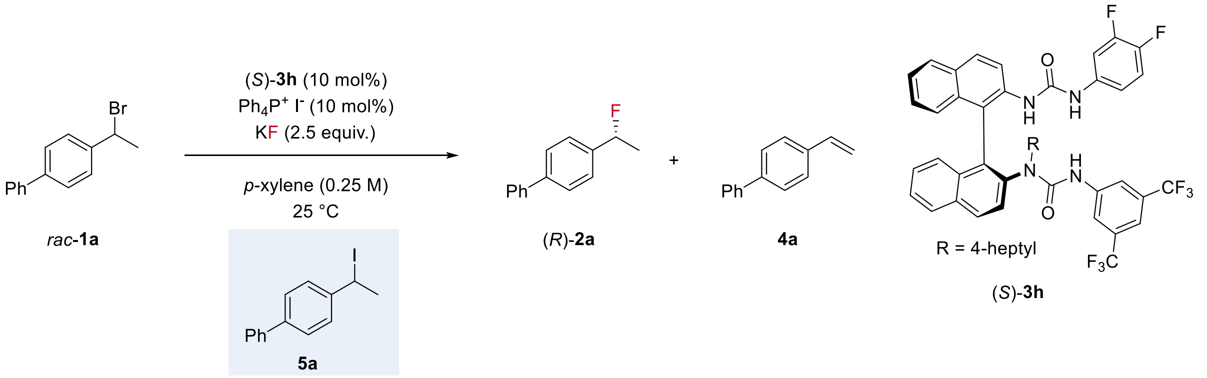


| Entry | Time (h) | **2a** (%)[a] | **1a** (%)[a] | **5a** (%)[a] | **2a** e.r.[b] | **1a** e.r.[b] |
|---|---|---|---|---|---|---|
| **1** | 1 | 2 | 88 | 7 | 91:9 | 51:49 |
| **2** | 2 | 4 | 86 | 6 | 91:9 | 51:49 |
| **3** | 4 | 10 | 72 | 7 | 91:9 | 50:50 |
| **4** | 8 | 22 | 59 | 7 | 91:9 | 51:49 |
| **5** | 24 | 39 | 41 | 6 | 91:9 | 52:48 |
| **6** | 48 | 56 | 30 | 7 | 90.5:9.5 | 53:47 |
| **7** | 72 | 75 | 3 | 5 | 91:9 | nd |

General conditions: Substrate (0.05 mmol), urea catalyst (10 mol%), $Ph_4P^+ I^-$ (10 mol%), and KF (2.5 equiv.) in 200 µL of *p*-xylene stirred at 1200 rpm [a]Determined by $^{1}$H and/or $^{19}$F NMR using 4-fluoroanisole as internal standard, [b]e.r. was determined by HPLC analysis using a chiral stationary phase. nd = not determined

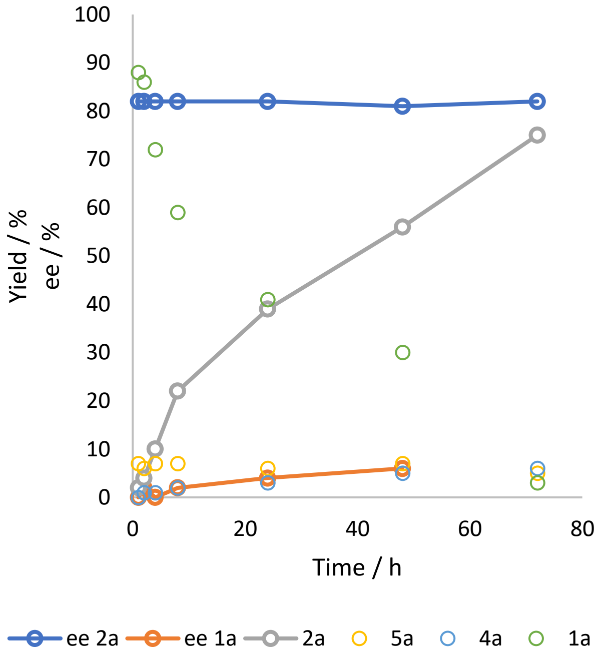


**Supplementary Figure 56**: Plot of enantiomeric ratios and yields of components over time course

Supplementary Table 21: *Ex situ* monitoring of the enantiomeric ratio of **1q** and **2q** over the time-course of reaction.

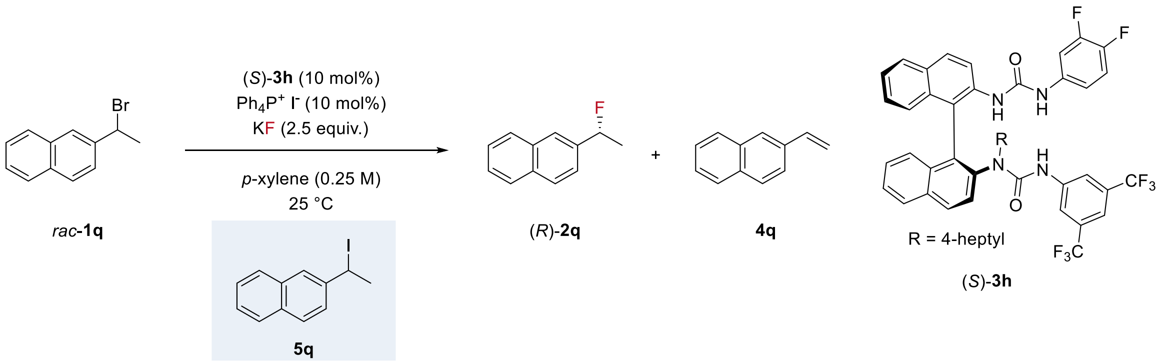


| Entry | Time (h) | **2q** (%)[a] | **1q** (%)[a] | **5q** (%)[a] | **2q** e.r.[b] | **1q** e.r.[b] |
|---|---|---|---|---|---|---|
| **1** | 1 | 1 | 92 | 6 | 87:13 | 50:50 |
| **2** | 4 | 4 | 89 | 8 | 87:13 | 51:49 |
| **3** | 8 | 10 | 84 | 8 | 87:13 | 52:48 |
| **4** | 24 | 24 | 66 | 8 | 87:13 | 52:48 |
| **5** | 48 | 42 | 50 | 8 | 87:13 | 53:47 |
| **6** | 72 | 59 | 35 | 8 | 87:13 | 53:47 |
| **7** | 120 | 73 | 14 | 8 | 87:13 | 54:46 |

General conditions: Substrate (0.05 mmol), urea catalyst (10 mol%), $Ph_4P^+ I^-$ (10 mol%), and KF (2.5 equiv.) in 200 µL of *p*-xylene stirred at 1200 rpm [a]Determined by $^1H$ and/or $^{19}F$ NMR using 4-fluoroanisole as internal standard, [b]e.r. was determined by HPLC analysis using a chiral stationary phase. nd = not determined

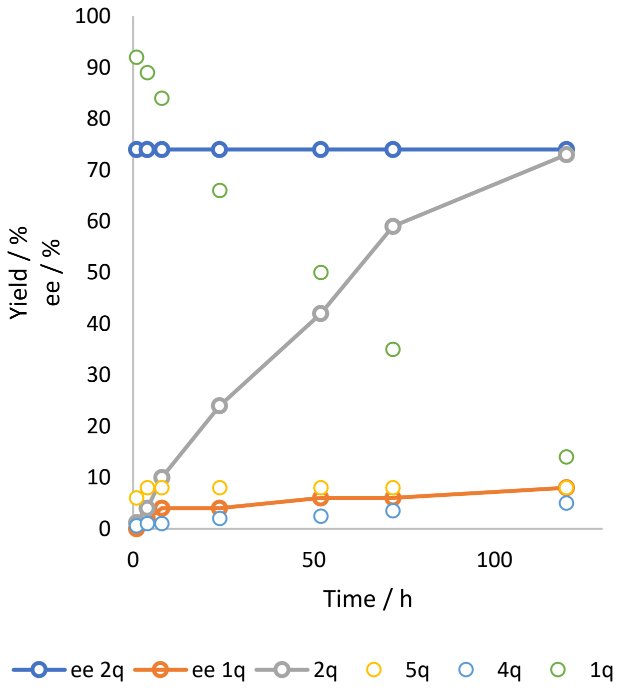


**Supplementary Figure 57**: Plot of enantiomeric ratios and yields of components over time course

Supplementary Table 22: *Ex situ* monitoring of the enantiomeric ratio of **9a** and **10a** over the time-course of reaction.

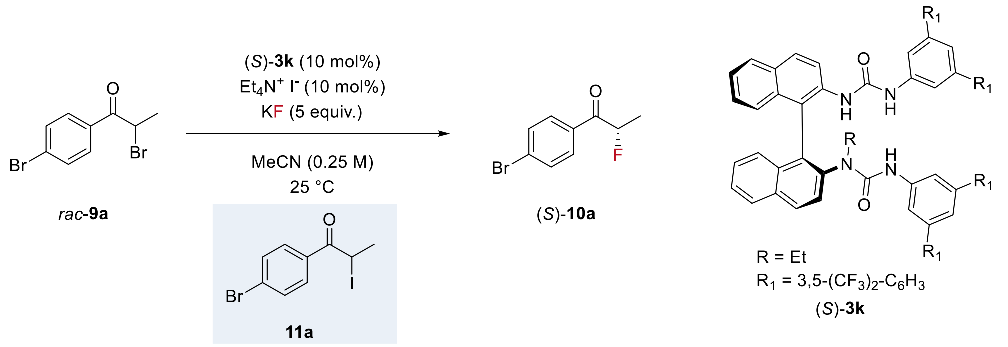


| Entry | Time (h) | **10q** (%)[a] | **9a** (%)[a] | **11a** (%)[a] | **10a** e.r.[b] | **9a** e.r.[b] |
|---|---|---|---|---|---|---|
| **1** | 1 | 6 | 81 | 10 | 92:8 | 50:50 |
| **2** | 2 | 10 | 76 | 10 | 93:7 | 51:49 |
| **3** | 4 | 15 | 71 | 10 | 92:8 | 52:48 |
| **4** | 6 | 19 | 70 | 10 | 92:8 | 52:48 |
| **5** | 8 | 26 | 60 | 10 | 92:8 | 53:47 |
| **6** | 24 | 65 | 20 | 10 | 92:8 | 54:46 |

General conditions: Substrate (0.05 mmol), urea catalyst (10 mol%), $Et_4N^+ I^-$ (10 mol%), and KF (5 equiv.) in 200 µL of MeCN stirred at 1200 rpm [a]Determined by $^1H$ and/or $^{19}F$ NMR using 4-fluoroanisole as internal standard, [b]e.r. was determined by HPLC analysis using a chiral stationary phase. nd = not determined

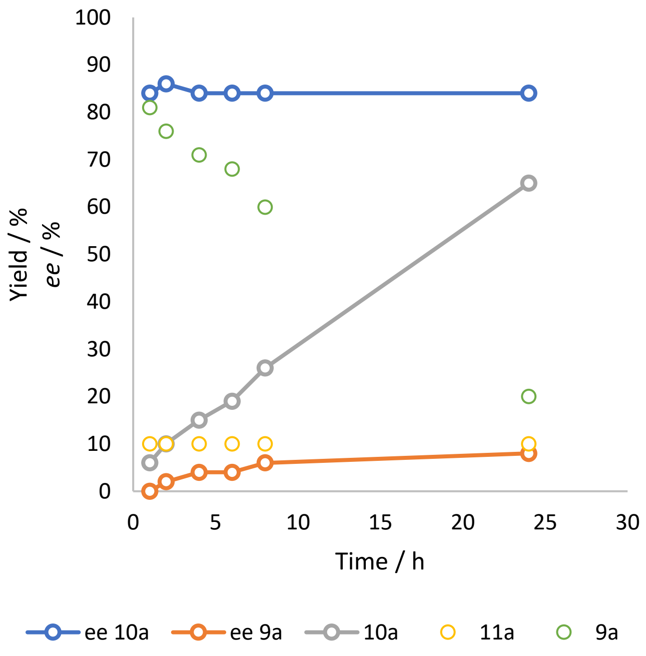


**Supplementary Figure 58**: Plot of enantiomeric ratios and yields of components over time course

## Stability of fluoride products under reactions conditions

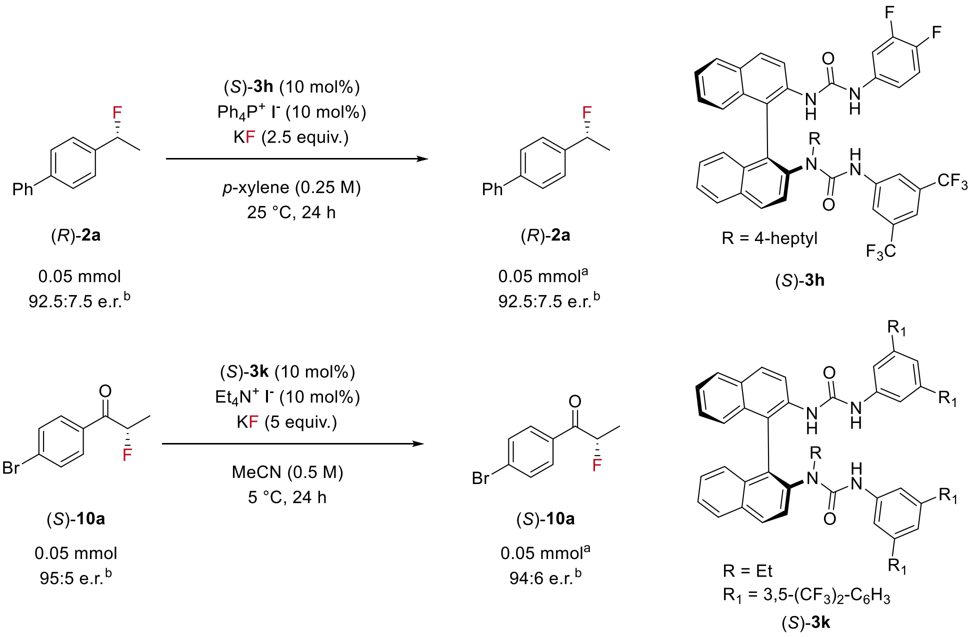


**Supplementary Figure 59**: General conditions: Substrate (0.05 mmol), urea catalyst (10 mol%), onium salt (10 mol%), and KF (2.5/ 5 equiv.) in 200 µL of solvent stirred at 1200 rpm [a]Determined by $^1H$ and/or $^{19}F$ NMR using 4-fluoroanisole as internal standard, [b]e.r. was determined by HPLC analysis using a chiral stationary phase. No side products were observed – fluoride products fully recovered.

## Computational Methods
### General Computational Details

Geometry optimisation and vibrational frequency calculations were performed using Gaussian 16, revision C.01.[36] The geometry optimisation and frequency calculations were performed at the M06-2X/def2-SV(P) (C, H, O) def2-TZVPPD (Other Heteroatoms) level of theory. Ground state geometries were identified by the absence of imaginary frequency vibrations. Single point energy calculations were performed using ORCA 5.0.3-gompi-2021b, at the M06-2X-D3ZERO/def2-TZVPP (C, H) ma-def2-TVPP (Other Heteroatoms) level of theory. These basis functions have previously been used to describe fluoride binding urea motifs and showed good agreement with experimental data.[23] Transition states were identified by the presence of a single imaginary frequency vibrational mode. In calculations including iodine, the 28 core electrons of I were replaced with a 28 electron quasi-relativistic ECP. Solvent corrections were applied to the calculations using the CPCM polarisable conductor calculation model for *p*-xylene.[37,38] Transition state structures were initially calculated using a model substrate **1am** ($C_8H_9Br$). Quasi-harmonic corrections were applied to generate thermochemical data using GoodVibes, with a frequency cutoff value of 100 $cm^{-1}$.[39]

### Conformational Sampling of HBD·F·$PPh_4$

Conformational sampling for the ternary mixture of the hydrogen bond donor catalyst, fluoride, and the tetraphenylphosphonium cation was performed using the Conformer-Rotamer Ensemble Sampling Tool, CREST, software.[40-45] A 25 kJ $mol^{-1}$ energy window was applied during sampling. Following initial geometry sampling, clustering analysis was used to reduce the number of conformers to 30 which were optimised at DFT level as described above. The 10 lowest energy adduct geometries are shown in Supplementary Figure 61.

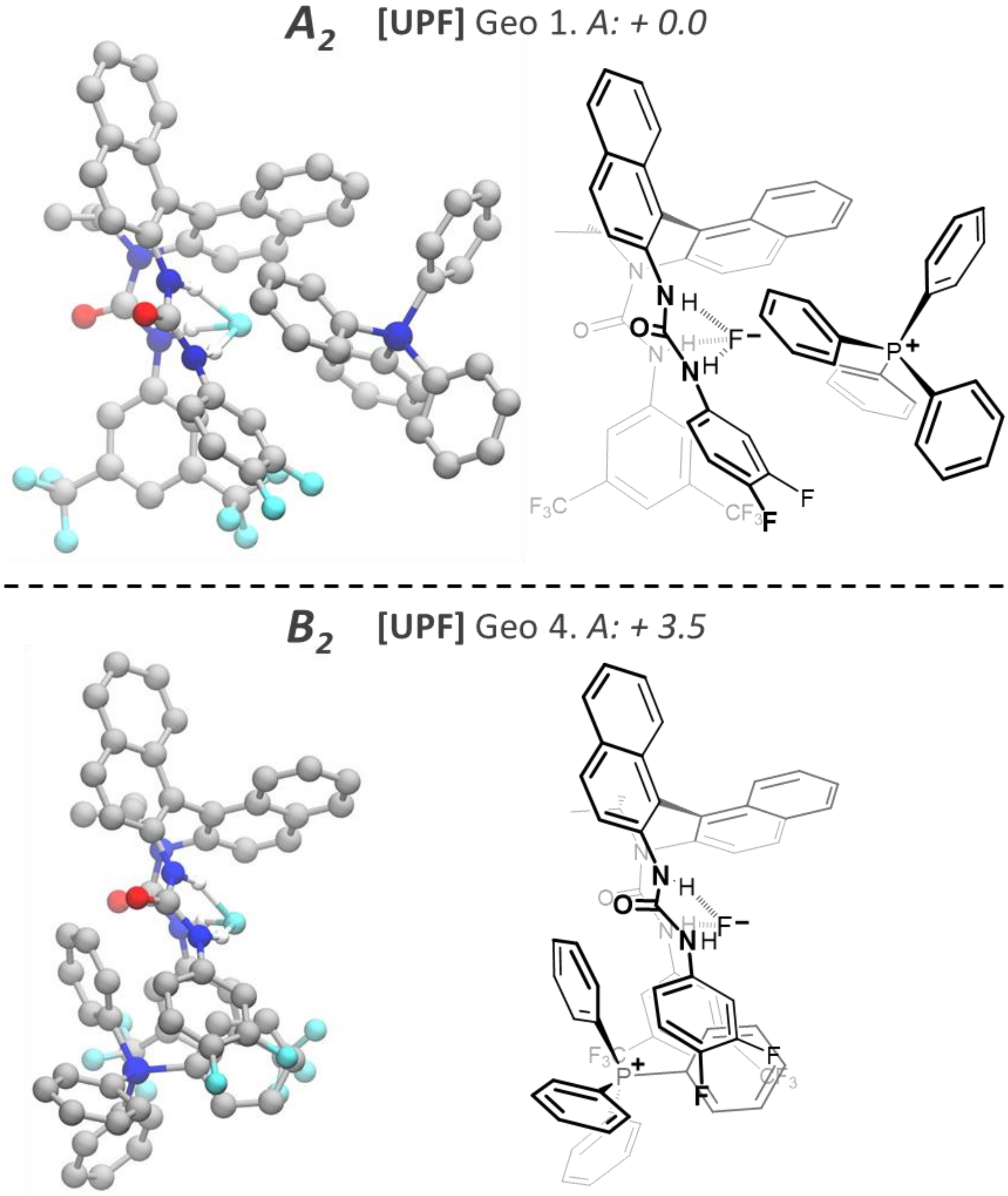


**Supplementary Figure 60**: Lowest energy calculated structures of [UPF] adducts representing the two key binding modes, A and B. The A binding mode is shown on the left. The B binding mode is shown on the right. Hydrogen atoms have been removed for clarity. Relative Gibbs energies of the adducts are given in kJ $mol^{-1}$. Summaries of the key bond distances are shown in Table S23.

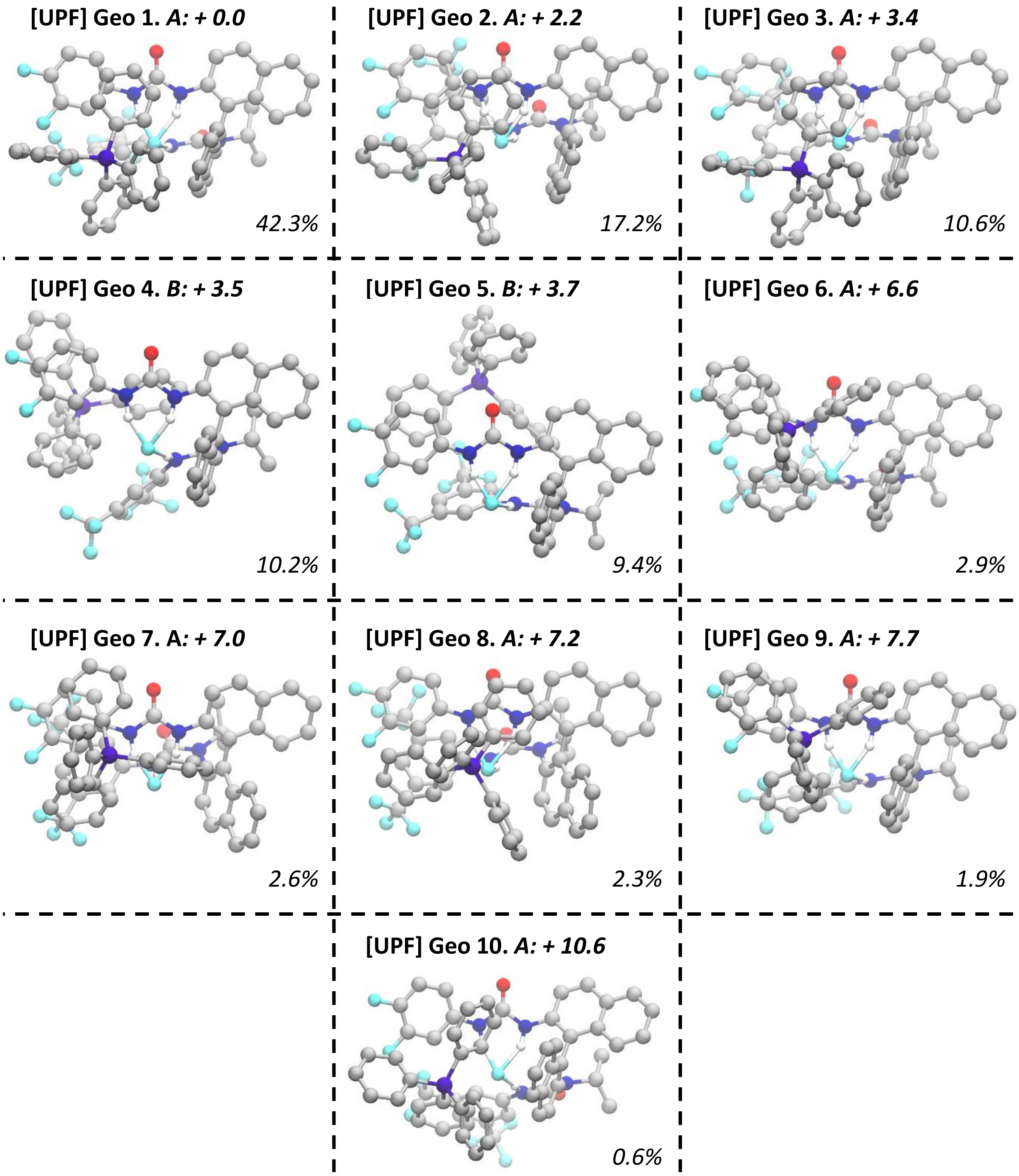


**Supplementary Figure 61**: 10 Lowest energy structures of HBD catalyst (*S*)-**3f** bound to fluoride and the tetraphenylphosphonium cation. A-type structures contain the phosphonium on the top surface of the HBD catalyst, occupying the reactive site. B-type structures contain the phosphonium in the back of the HBD catalyst leaving the fluoride exposed. Hydrogen atoms have been removed for clarity. Relative Gibbs free energies are shown above each structure, given in kJ $mol^{-1}$ relative to the lowest energy structure. Boltzmann percentage populations are shown below each structure. Summaries of the key bond distances are shown in Table S23.

Supplementary Table 23: Calculated distances from fluoride to close contacts identified by $^{1}H$-$^{19}F$ nOe experiments (Supplementary Table 12). B- type geometries are denoted by an asterisk, *.

| **Geometry** | **Bond Length / Å** | | | | |
|---|---|---|---|---|---|
| | **$N-H_a\cdots F$** | **$N-H_b\cdots F$** | **$N-H_c\cdots F$** | **$C-H_{17}\cdots F$** | **$C-H_{PPh4}\cdots F$** |
| **Experimental** | 1.72 | 1.88 | 1.70 | 2.28 | - |
| **Boltzmann Averaged** | 1.63 | 1.77 | 1.66 | 2.34 | 2.24 |
| **3f·F·$PPh_4$ 1** | 1.60 | 1.78 | 1.65 | 2.17 | 1.91 |
| **3f·F·$PPh_4$ 2** | 1.62 | 1.80 | 1.70 | 2.54 | 1.91 |
| **3f·F·$PPh_4$ 3** | 1.68 | 1.72 | 1.67 | 2.49 | 1.84 |
| **3f·F·$PPh_4$ 4*** | 1.77 | 1.75 | 1.64 | 2.48 | 2.29 |
| **3f·F·$PPh_4$ 5*** | 1.58 | 1.77 | 1.66 | 2.23 | 4.71 |
| **3f·F·$PPh_4$ 6** | 1.59 | 1.73 | 1.64 | 2.16 | 2.38 |
| **3f·F·$PPh_4$ 7** | 1.67 | 1.74 | 1.75 | 2.74 | 2.30 |
| **3f·F·$PPh_4$ 8** | 1.66 | 1.84 | 1.77 | 2.85 | 1.87 |
| **3f·F·$PPh_4$ 9** | 1.62 | 1.71 | 1.62 | 2.15 | 2.31 |
| **3f·F·$PPh_4$ 10** | 1.60 | 1.77 | 1.61 | 2.29 | 2.10 |

$C-H_{PPh_4}\cdots F$ refers to the closest contact between the fluoride and a hydrogen atom of the phosphonium cation

**TS of 1am – 2am (A – major, B – minor) and 5am – 2am (C – major, D – minor)**

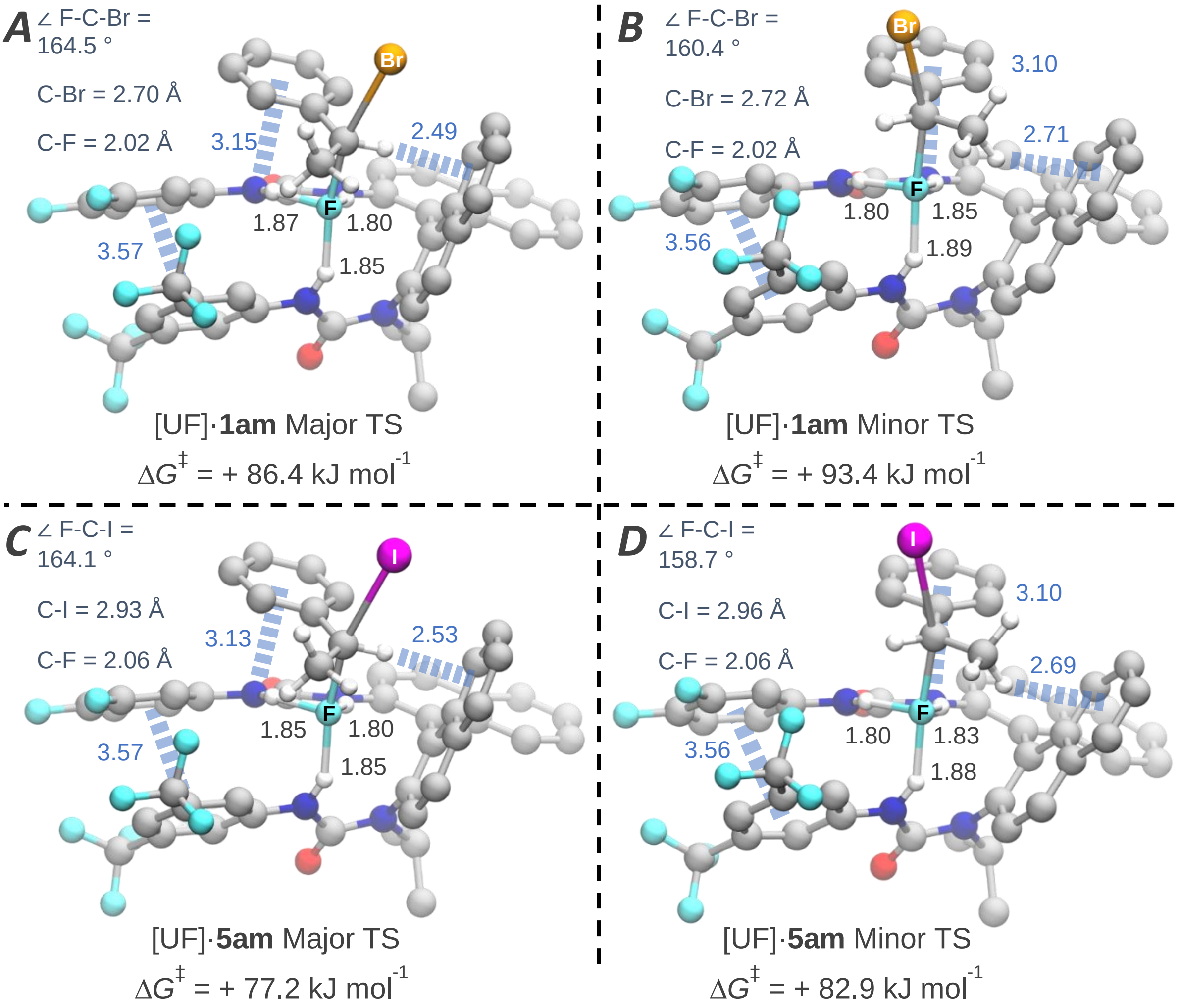


**Supplementary Figure 62**: Calculated major (left) and minor (right) $S_N2$ transition structures for [UF]·BnX. **A**: X = Br – major pathway, **B**: X = Br – minor pathway, **C**: X = I – major pathway, **D**: X = I – minor pathway. Key bond lengths and angles are also shown in Table 24. Hydrogen atoms have been removed for clarity.

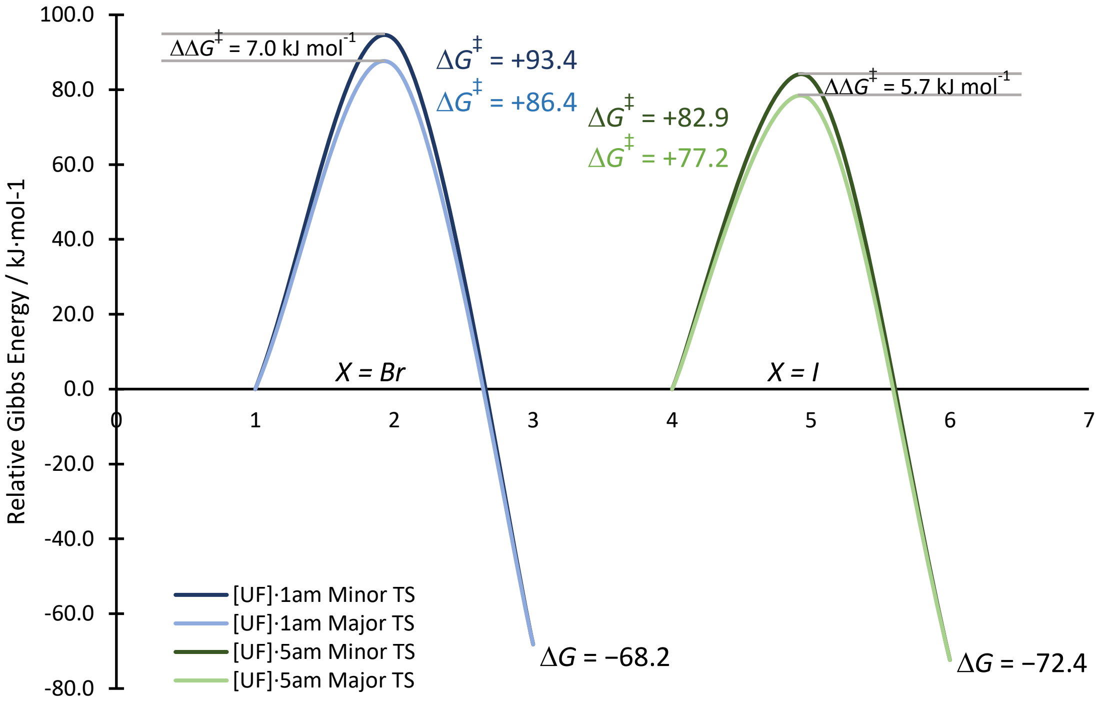


**Supplementary Figure 63**: Conversion of benzyl halide (*left: bromide* **1am***, right: iodide* **5am**) into **2am**. Gibbs energies relative to separated nucleophile and electrophile are shown in kJ mol$^{-1}$.

The structures of the reactants, products, and $S_N2$ TSs were optimised as described above. In all cases, the phosphonium cation was not considered in the TS to reduce conformational and computational complexity. Activation barriers for these model systems are consistent with reactivity at 15 – 60 °C, and the sense of enantioselectivity is also consistent with experiment. With either a bromide or iodide leaving group, the lowest energy pathway leads to (*R*)-fluoride formation, with the iodide electrophile having predicted overall lower barriers by 10 kJ mol$^{-1}$. The computed level of selectivity between major and minor pathways, $\Delta\Delta G^{\ddagger}$, was shown to be similar for both leaving groups (7.0 and 5.8 kJ mol$^{-1}$ when X = Br and I, respectively). By considering the Boltzmann distribution of the major and minor pathways (two geometries calculated for each pathway), the predicted enantioselectivities at 25 °C are 93:7 or 89:11 e.r., respectively. The major TS geometry is distinct from the minor by the deviation from linearity, with the F–C–Br angle being 164.5 ° in the major TS and 160.4 ° in the minor TS. This trend is mirrored in the F–C–I angle, being 164.5 ° in the major TS and 158.7 ° in the minor TS.

**TS of 1a – 2a (A – major, B – minor) and 5a – 2a (C – major, D – minor)**

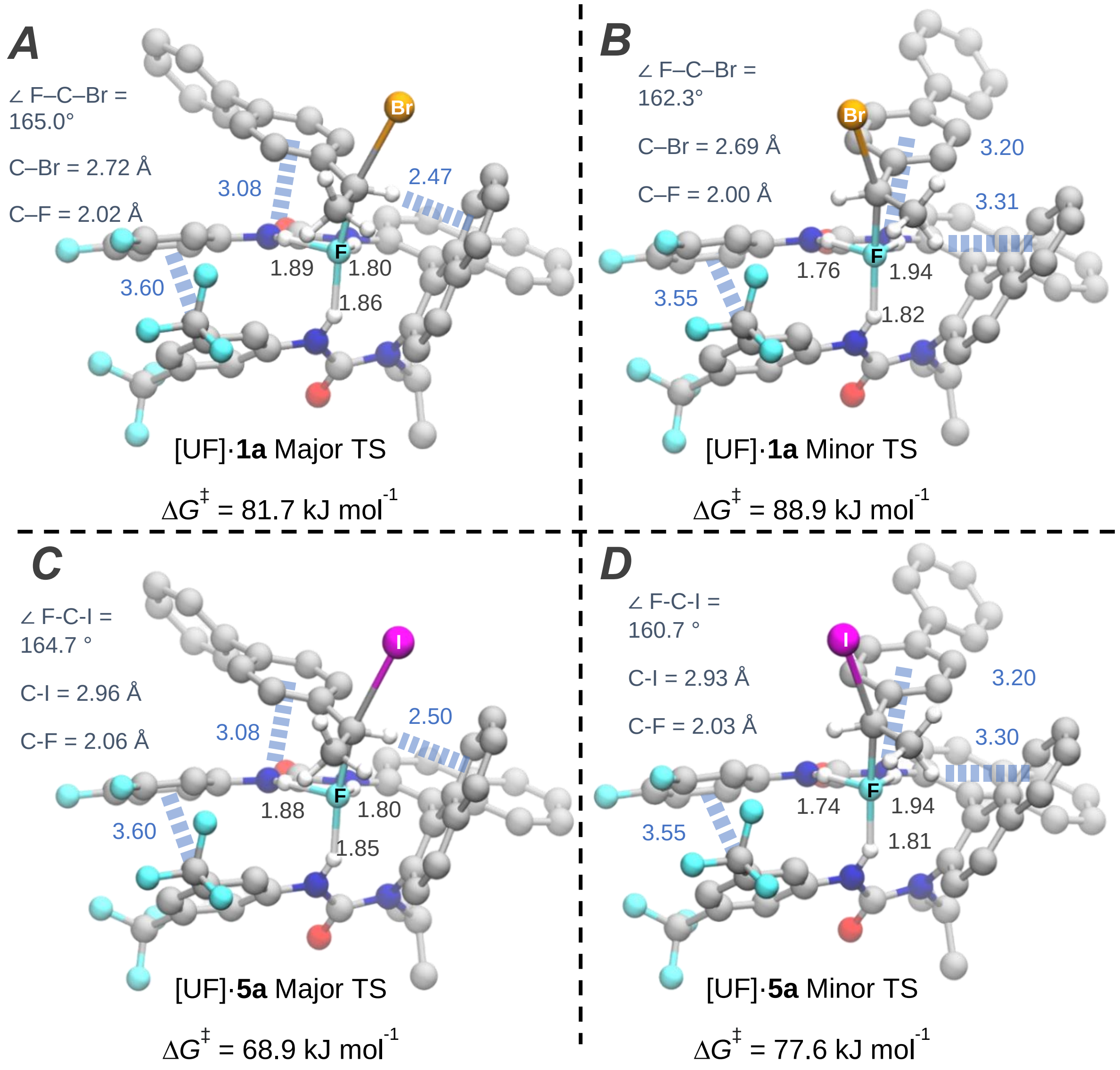


**Supplementary Figure 64**: Calculated major (left) and minor (right) $S_N2$ transition structures for [UF]·**1a** and [UF]·**5a**. **A**: X = Br – major pathway, **B**: X = Br – minor pathway, **C**: X = I – major pathway, **D**: X = I – minor pathway. Key bond lengths and angles are also shown in Table 24, bond lengths are shown in Angstroms. Hydrogen atoms have been removed for clarity.

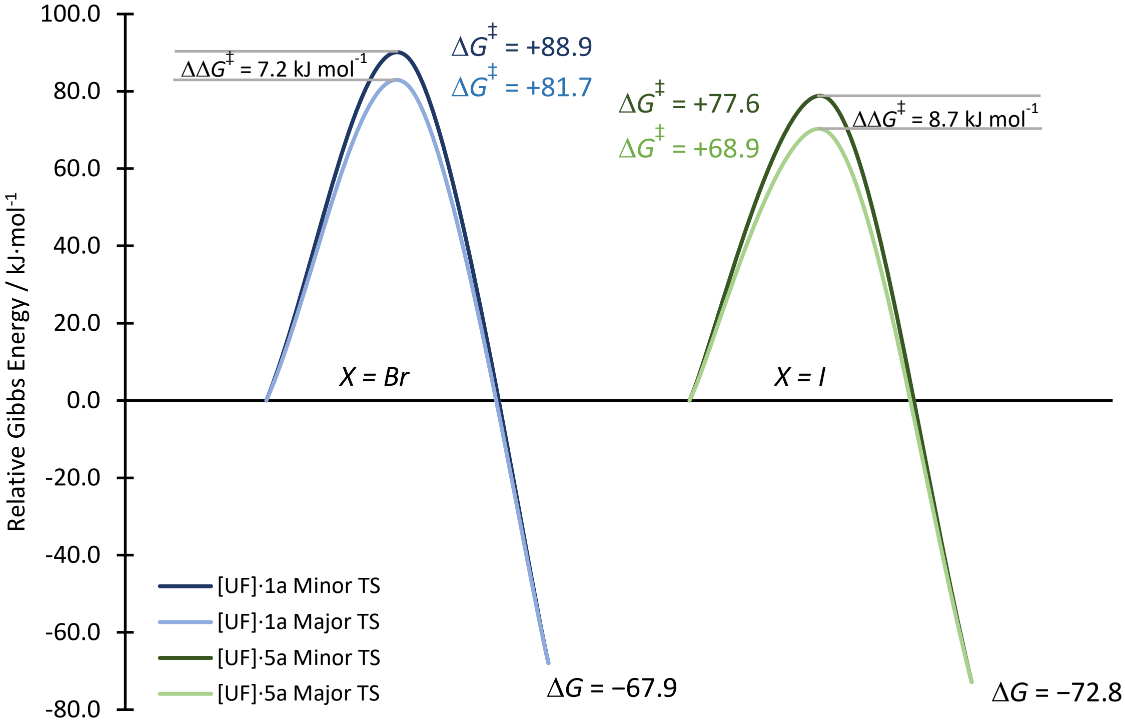


**Supplementary Figure 65**: Conversion of *(left)* **1a** *and (right)* **5a** into **2a**. Gibbs energies relative to separated nucleophile and electrophile are shown in kJ mol$^{-1}$.

As with the benzyl electrophile **1am**, TSs obtained for fluorination of bromide **1a** and iodide **5a** gave selectivity's consistent with experiment, again with the iodide showing greater reactivity towards nucleophilic substitution. Quantitatively, the predictive levels of enantioselectivities obtained were similar with this electrophile, the major TS being stabilized more than the minor TS in relation to the barrier heights obtained for the smaller electrophile. The energy difference between the major and minor pathways, $\Delta\Delta G^{\ddagger}$, is again similar for both halide leaving groups (7.2 and 8.7 kJ mol$^{-,1}$ when X = Br and I, respectively). By considering the Boltzmann distribution of the major and minor pathways (two geometries calculated for each pathway), the predicted enantioselectivities at 25 °C are 91:9 or 95:5 e.r., respectively. As observed in the case of **1am** and **5 am**, the F–C–Br angle is 165.0 ° in the major TS and 162.3 ° in the minor TS. This trend is mirrored in the F–C–I angle, being 164.7 ° in the major TS and 160.7 ° in the minor TS.

**TS of 1h – 2h (A – major, B – minor) and 5h – 2h (C – major, D – minor)**

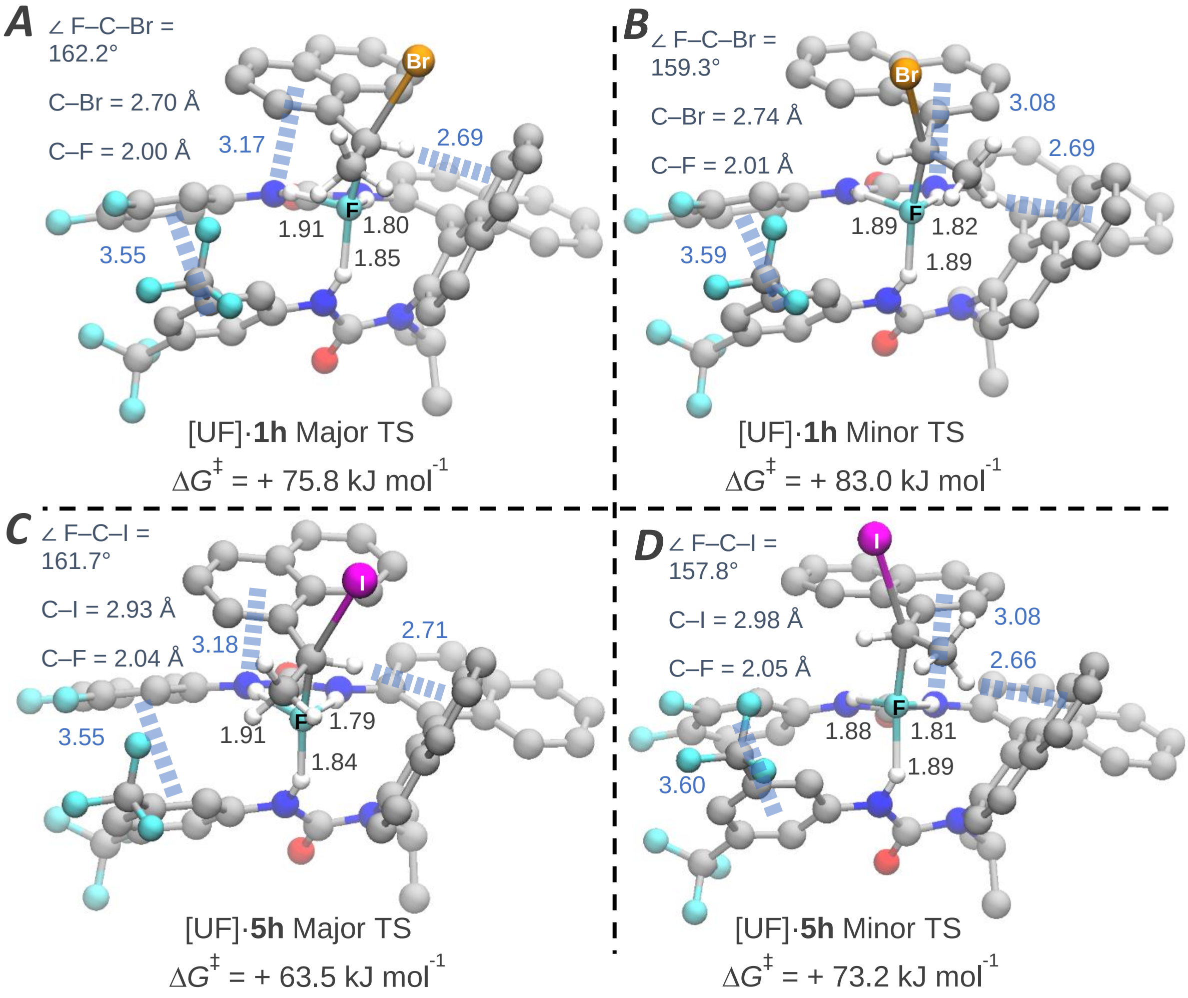


**Supplementary Figure 66**: Calculated major (left) and minor (right) $S_N2$ transition structures for [UF]·**1h** and [UF]·**5h**. **A**: X = Br – major pathway, **B**: X = Br – minor pathway, **C**: X = I – major pathway, **D**: X = I – minor pathway. Key bond lengths and angles are also shown in Table S24, bond lengths are shown in Angstroms. Hydrogen atoms have been removed for clarity.

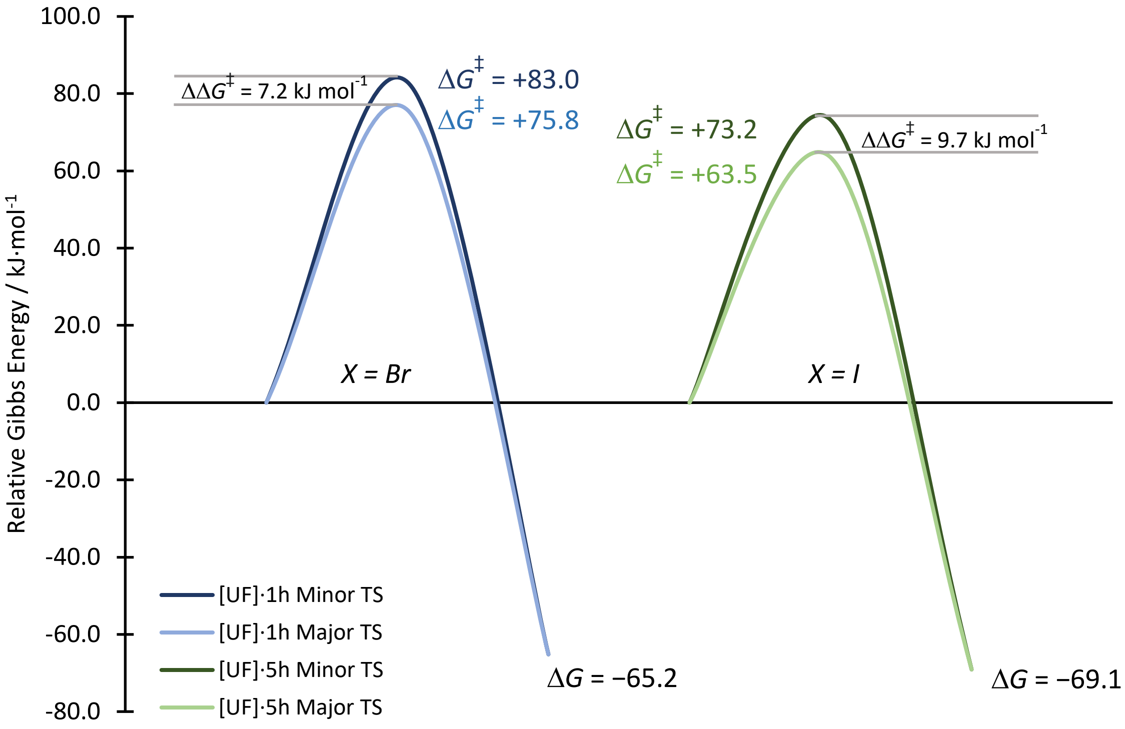


**Supplementary Figure 67**: Conversion of (*left)* **1h** *and (right)* **5h** into **2h**. Gibbs energies relative to separated nucleophile and electrophile are shown in kJ mol$^{-1}$.

As with the benzyl electrophile **1am**, TSs obtained for fluorination of bromide **1h** and iodide **5h** gave selectivity consistent with experiment, again with the iodide showing greater reactivity towards nucleophilic substitution. Quantitatively, the predictive levels of enantioselectivities obtained were similar with this electrophile, the major TS being stabilized more than the minor TS in relation to the barrier heights obtained for the smaller electrophile. The energy difference between the major and minor pathways, $\Delta\Delta G^{‡}$, is again similar for both halide leaving groups (7.2 and 9.7 kJ mol$^{-,1}$ when X = Br and I, respectively). By considering the Boltzmann distribution of the major and minor pathways (two geometries for the major pathway, three geometries for the minor pathway), the predicted enantioselectivities at 25 °C are 93:7 or 97:3 e.r., respectively.

The increase in selectivity is in line with experimental observation. The TS barrier is significantly lower for the naphthyl containing substrate. With the relative energy barrier of the lowest energy geometry of the major pathway decreasing **1am**>**1a**>**1h** (+86.4 kJ mol$^{-1}$ *vs.* +81.7 kJ mol$^{-1}$ *vs.* +75.8 kJ mol$^{-1}$). The naphthyl subunit is positioned above the urea motif of the HBD catalyst, with a ring centroid located above each nitrogen atom of the urea moiety. As has been noted in the transition states of **1am**, **5am**, **1a**, and **5a**, the major transition state has a more linear geometry along the $S_N2$ axis (F–C–Br = 162.2 ° major, 159.3 ° minor, F–C–I = 161.7 ° major, 157.8 ° minor).

**TS of 1y – 2y (A – major, B – minor) and 5y – 2y (C – major, D – minor)**

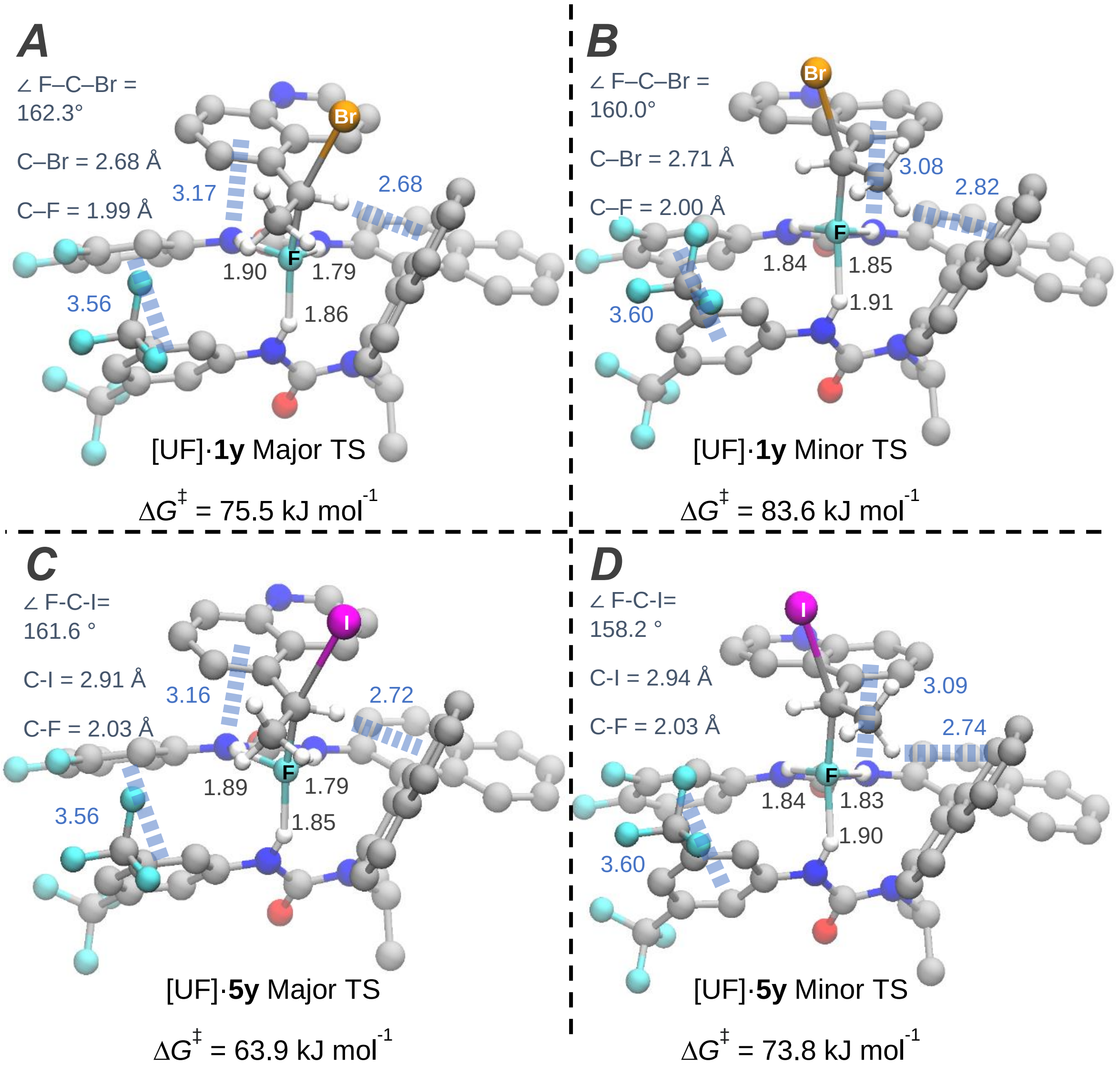


**Supplementary Figure 68**: Calculated Major (left) and Minor (right) $S_N2$ transition state structures for [UF]·QuinolineX. **A**: X = Br – major pathway, **B**: X = Br – minor pathway, **C**: X = I – major pathway, **D**: X = I – minor pathway. Key bond lengths and angles are also shown in Table S24. Hydrogen atoms have been removed for clarity. The relative transition state energy is shown in kJ·mol$^{-1}$.

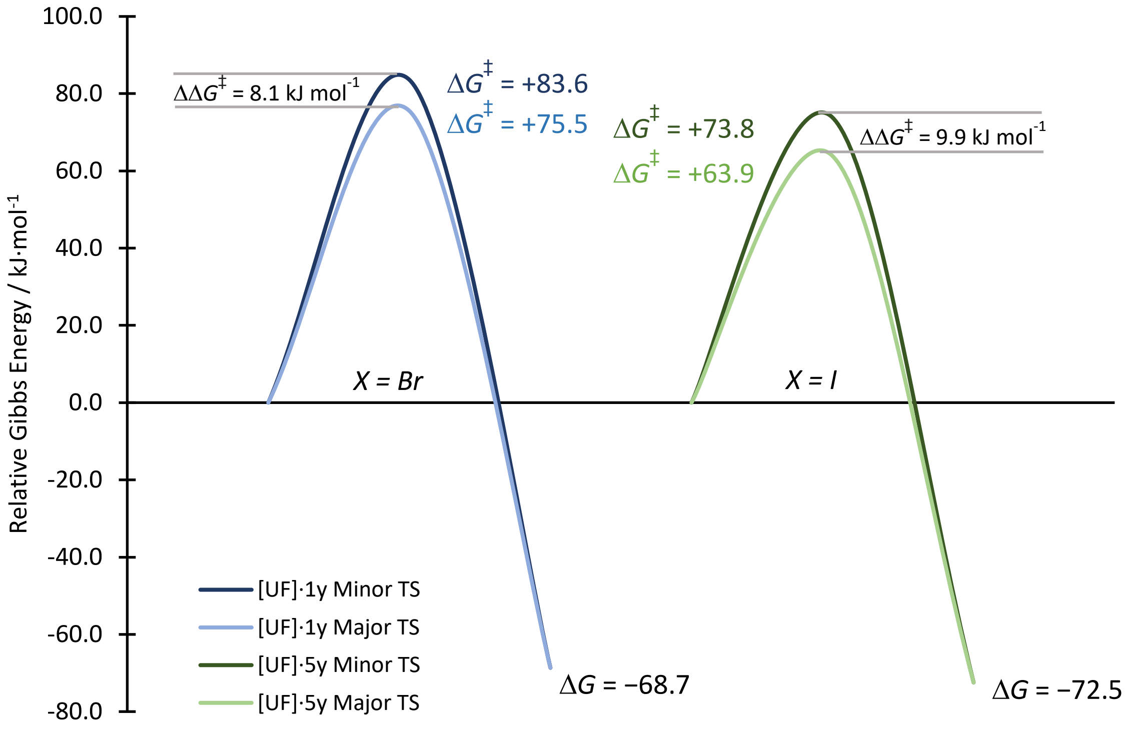


**Supplementary Figure 69**: Conversion of (*left*) **1y** *and (right)* **5y** into **2y**. Gibbs energies relative to separated nucleophile and electrophile are shown in kJ mol$^{-1}$.

TSs obtained for fluorination of bromide **1y** and iodide **5y** gave selectivity consistent with experiment, again with the iodide showing greater a lower $S_N2$ TS barrier. The major TS being stabilized more than the minor TS. The energy difference between the major and minor pathways, $\Delta\Delta G^{\ddagger}$, is again similar for both halide leaving groups (8.1 and 10.0 kJ mol$^{-,1}$ when X = Br and I, respectively). By considering the Boltzmann distribution of the major and minor pathways (two geometries calculated for the major pathway, three geometries calculated for the minor pathway), the predicted enantioselectivities at 25 °C are 95:5 or 98:2 e.r., respectively.

The increase in selectivity is in line with experimental observation. The TS geometry and energy barrier the quinoline bearing substrates is the very similar to naphthyl bearing substrates, with the fused rings positioned over both of the urea nitrogen atoms. With the relative energy barrier of the lowest energy geometry of the major pathway decreasing **1am**>**1a**>**1h**>**1y** (+86.4 kJ mol$^{-1}$ *vs.* +81.7 kJ mol$^{-1}$ *vs.* +75.8 kJ mol$^{-1}$ *vs*. 75.5 kJ mol$^{-1}$). The naphthyl subunit is positioned above the urea motif of the HBD catalyst, with a ring centroid located above each nitrogen atom of the urea moiety. As has been noted in the transition states of **1am**, **5am**, **1a**, and **5a**, the major transition state has a more linear geometry along the $S_N2$ axis (F–C–Br = 162.3 ° major, 160.0 ° minor, F–C–I = 161.6 ° major, 158.2 ° minor).

Supplementary Table 24: Key bond distances and angles of the $S_N2$ TS for the fluorination of benzyl- (**1am** and **5am**), biphenyl- (**1a** and **5a**), naphthyl- (**1h** and **5h**), and quinoline- (**1y** and **5y**) bromide and iodide electrophiles. The major pathway forms the (*R*)-fluorinated product from the preceding (*S*)-halide. The CH–π distances are defined as the distance from the substrate (closest –CH– or –$CH_3$) to the BINAM π-system. The π–π distances refer to intramolecular π–stacking distance between the two centroids of the aromatic groups of the catalyst. The N–π bond length is defined as the intermolecular distance between the closest urea N atom to the closest centroid of the aromatic group of the substrate.

| | | | | **Bond Distance / Å** | | | | |
|---|---|---|---|---|---|---|---|---|
| Substrate | **1am/5am** | | | | **1a/5a** | | | |
| Leaving group | **Br** | | **I** | | **Br** | | **I** | |
| Pathway | **Major** | **Minor** | **Major** | **Minor** | **Major** | **Minor** | **Major** | **Minor** |
| **C–F** | 2.02 | 2.02 | 2.06 | 2.06 | 2.02 | 2.00 | 2.06 | 2.03 |
| **C–X** | 2.70 | 2.72 | 2.93 | 2.96 | 2.72 | 2.69 | 2.96 | 2.93 |
| **$NH_a$–F** | 1.87 | 1.80 | 1.85 | 1.80 | 1.89 | 1.76 | 1.88 | 1.74 |
| **$NH_b$–F** | 1.80 | 1.85 | 1.80 | 1.83 | 1.80 | 1.94 | 1.80 | 1.94 |
| **$NH_c$–F** | 1.85 | 1.89 | 1.85 | 1.88 | 1.86 | 1.82 | 1.85 | 1.81 |
| **CH–π** | 2.49 | 2.71 | 2.53 | 2.69 | 2.47 | 3.31 | 2.50 | 3.30 |
| **π–π** | 3.57 | 3.56 | 3.57 | 3.56 | 3.60 | 3.55 | 3.60 | 3.55 |
| **N–π** | 3.15 | 3.10 | 3.13 | 3.10 | 3.08 | 3.20 | 3.08 | 3.20 |
| | | | | **Bond Angle / °** | | | | |
| **∠ F–C–X** | 164.5 | 160.4 | 164.1 | 158.7 | 165.0 | 162.3 | 164.7 | 160.7 |
| Substrate | **1h/5h** | | | | **1y/5y** | | | |
| Leaving Group | **Br** | | **I** | | **Br** | | **I** | |
| Pathway | **Major** | **Minor** | **Major** | **Minor** | **Major** | **Minor** | **Major** | **Minor** |
| **C–F** | 2.00 | 2.01 | 2.04 | 2.05 | 1.99 | 2.00 | 2.03 | 2.03 |
| **C–X** | 2.70 | 2.74 | 2.93 | 2.98 | 2.68 | 2.71 | 2.91 | 2.94 |
| **$NH_a$–F** | 1.91 | 1.89 | 1.91 | 1.88 | 1.90 | 1.84 | 1.89 | 1.84 |
| **$NH_b$–F** | 1.80 | 1.82 | 1.79 | 1.81 | 1.79 | 1.85 | 1.79 | 1.83 |
| **$NH_c$–F** | 1.85 | 1.89 | 1.84 | 1.89 | 1.86 | 1.91 | 1.85 | 1.90 |
| **CH–π** | 2.69 | 2.69 | 2.71 | 2.66 | 2.68 | 2.82 | 2.72 | 2.74 |
| **π–π** | 3.55 | 3.59 | 3.55 | 3.60 | 3.56 | 3.60 | 3.56 | 3.60 |
| **N–π** | 3.17 | 3.08 | 3.18 | 3.08 | 3.17 | 3.08 | 3.16 | 3.09 |
| | | | | **Bond Angle / °** | | | | |
| **∠ F–C–X** | 162.2 | 159.3 | 161.7 | 157.8 | 162.3 | 160.0 | 161.6 | 158.2 |

## Non-Covalent Interaction (NCI) Plots

For a qualitative comparison of the non-covalent interactions taking place between substrate and catalyst in the enantiodetermining TSs, we generated NCI isosurfaces with NCIPlot.[46] In these visualizations, blue isosurface values, corresponding to large negative values of sign($\lambda_2$)$\rho$, represent strong attractive interactions such as hydrogen bonds. Green isosurface values, corresponding to values of sign($\lambda_2$)$\rho$ close to 0, represent attractive, dispersion-dominated interactions. Red isosurface values, corresponding to large positive values of sign($\lambda_2$)$\rho$, represent repulsive steric interactions. The NCI isosurface plots reveal the strong N–H···F hydrogen bonds present in each TS, in addition to multiple weaker non-covalent interactions between the substrate and BINAM catalyst framework. Comparing major and minor pathways, in the major TS the benzylic C-H is oriented towards the BINAM naphthyl backbone, resulting in a favourable CH–$\pi$ interaction. Of note, the leaving halide, a polarizable halide anion also experiences favourable dispersive interactions with the naphthyl backbone. In the minor pathway, a methyl C–H is instead oriented towards the catalyst backbone, with a longer CH-$\pi$ distance, and less favourable intermolecular interaction. Any interaction between the leaving halide and the BINAM catalyst is no longer present in the minor TS. In both pathways, the aromatic ring of the electrophile experiences favourable dispersion-dominated interactions with the catalyst, which contributes to barrier lowering. This is consistent with the electrophiles that have extended $\pi$-systems, such as biphenyl or naphthyl groups, having overall lower activation barriers compared to benzyl electrophiles.

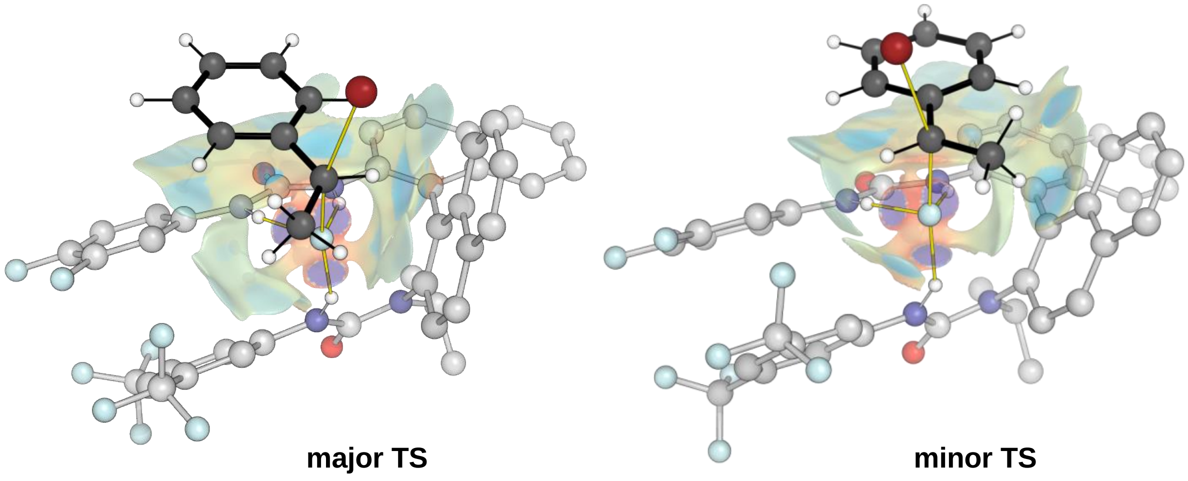


**Supplementary Figure 70**: NCI isosurfaces for competing major and minor diastereomeric $S_N2$ TSs.

## Cartesian coordinates of the computed structures

## Copies of NMR spectra

## Fluoride products

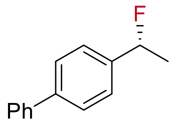


(*R*)-**2a**

**(*R*)-4-(1-fluoroethyl)-1,1'-biphenyl (2a)**

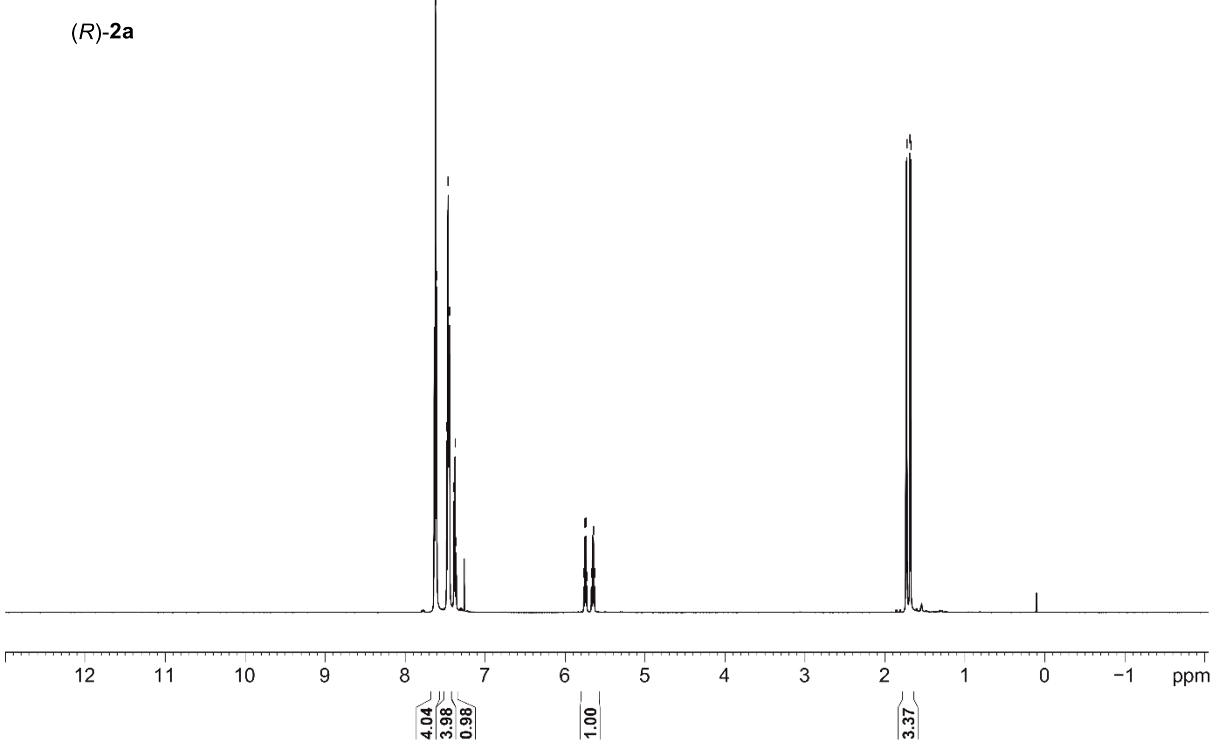


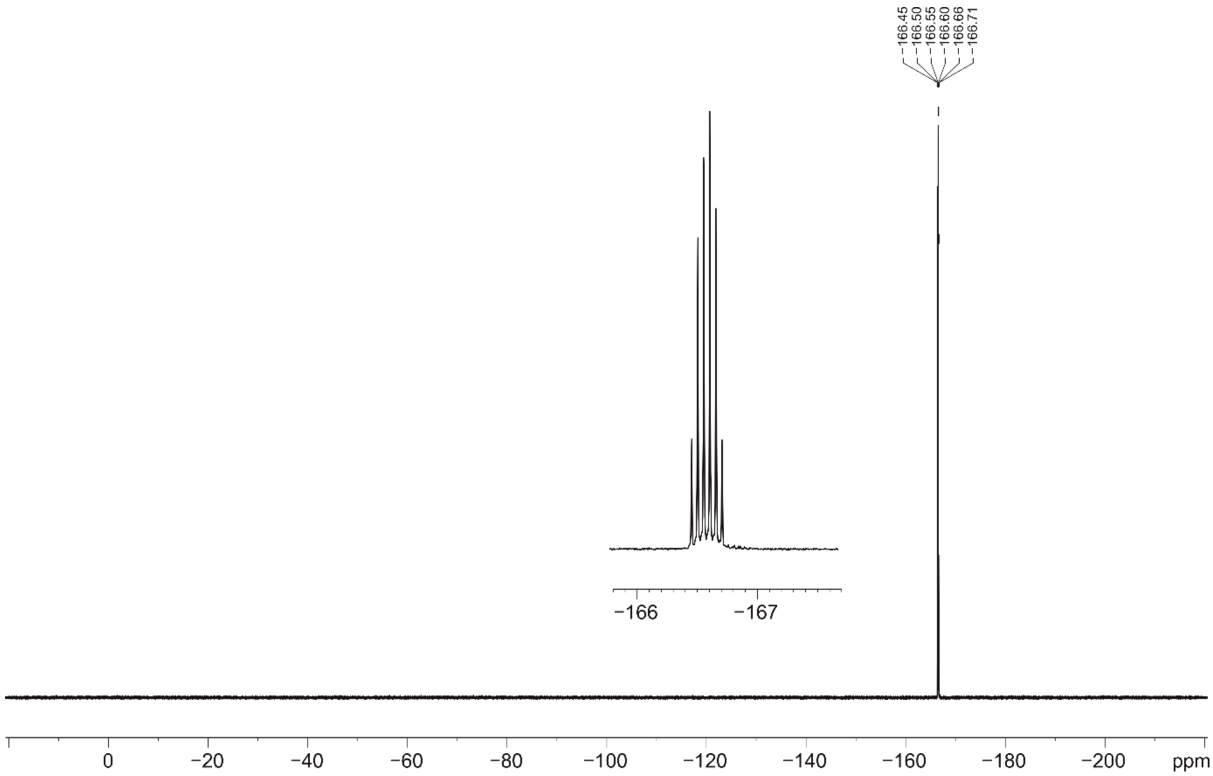

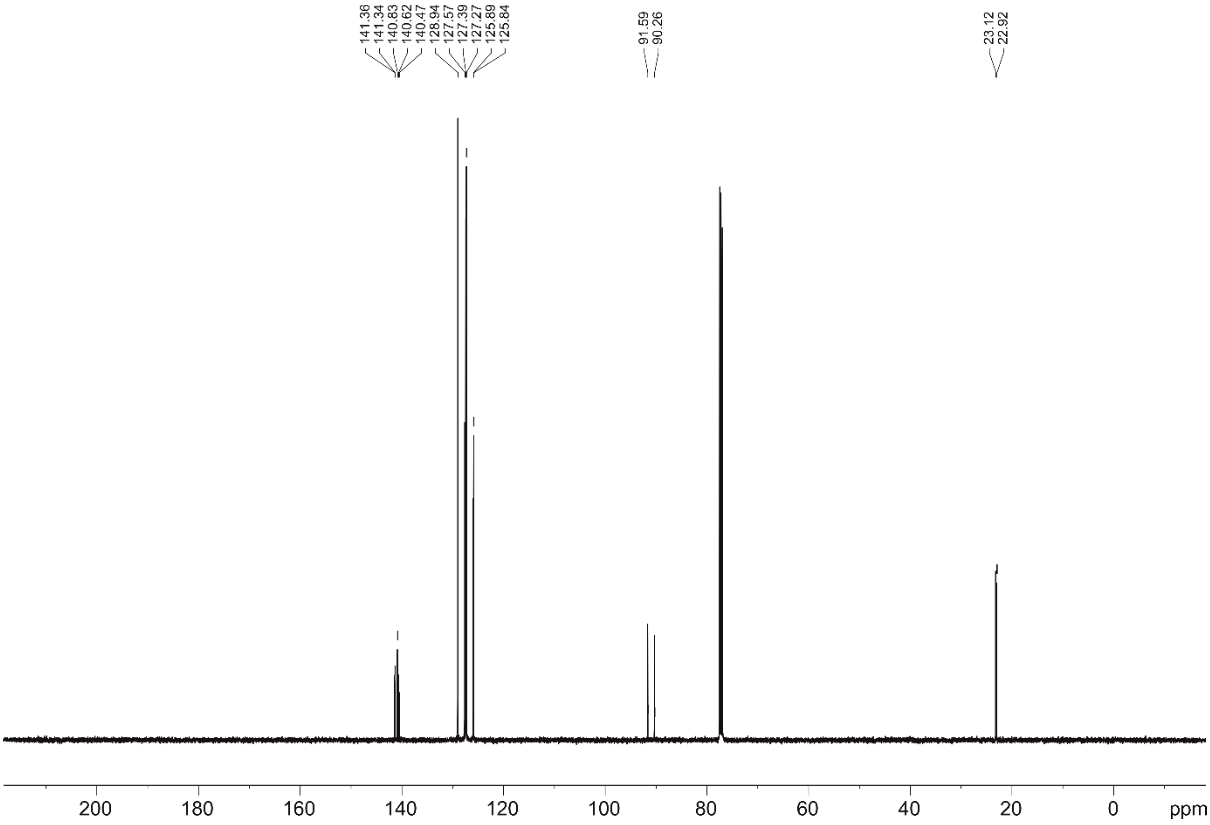


**(*R*)-4-fluoro-4'-(1-fluoroethyl)-1,1'-biphenyl (2b)**

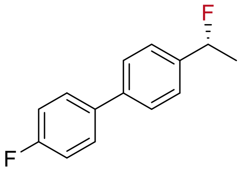


(*R*)-**2b**

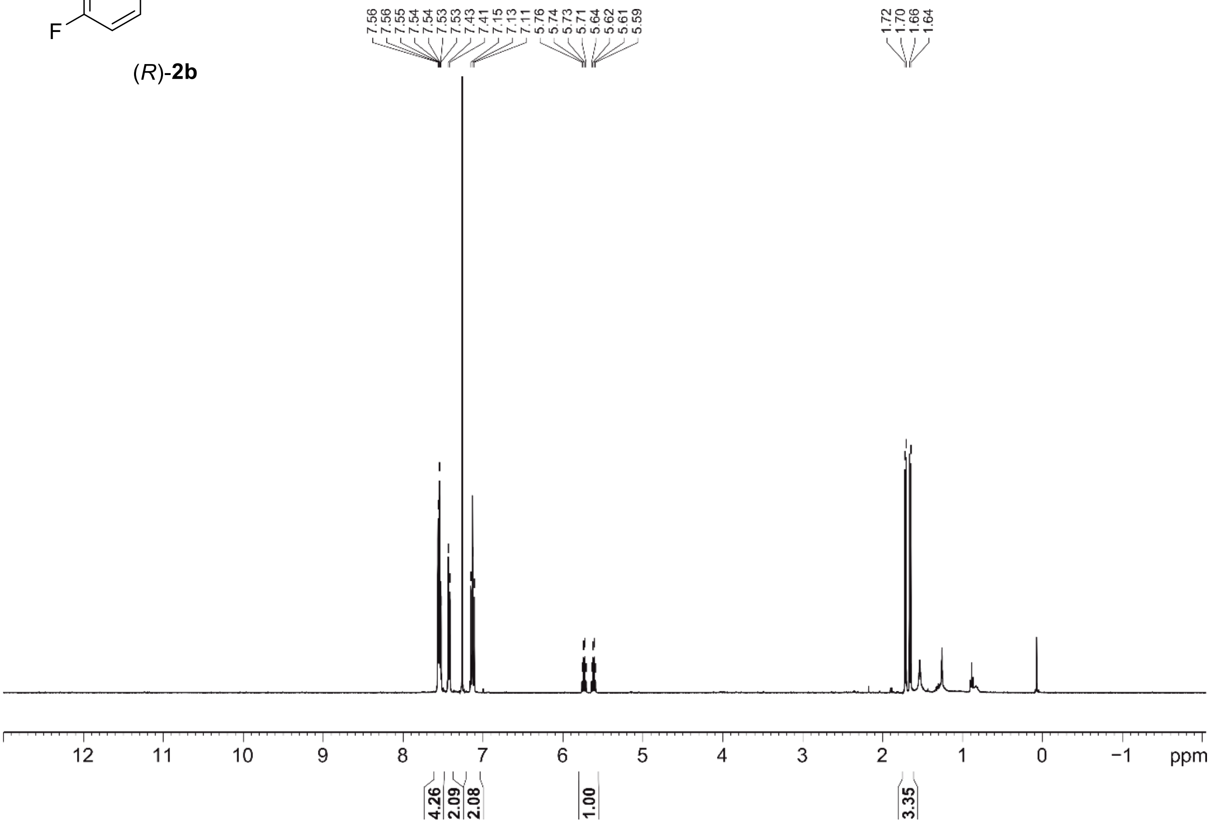

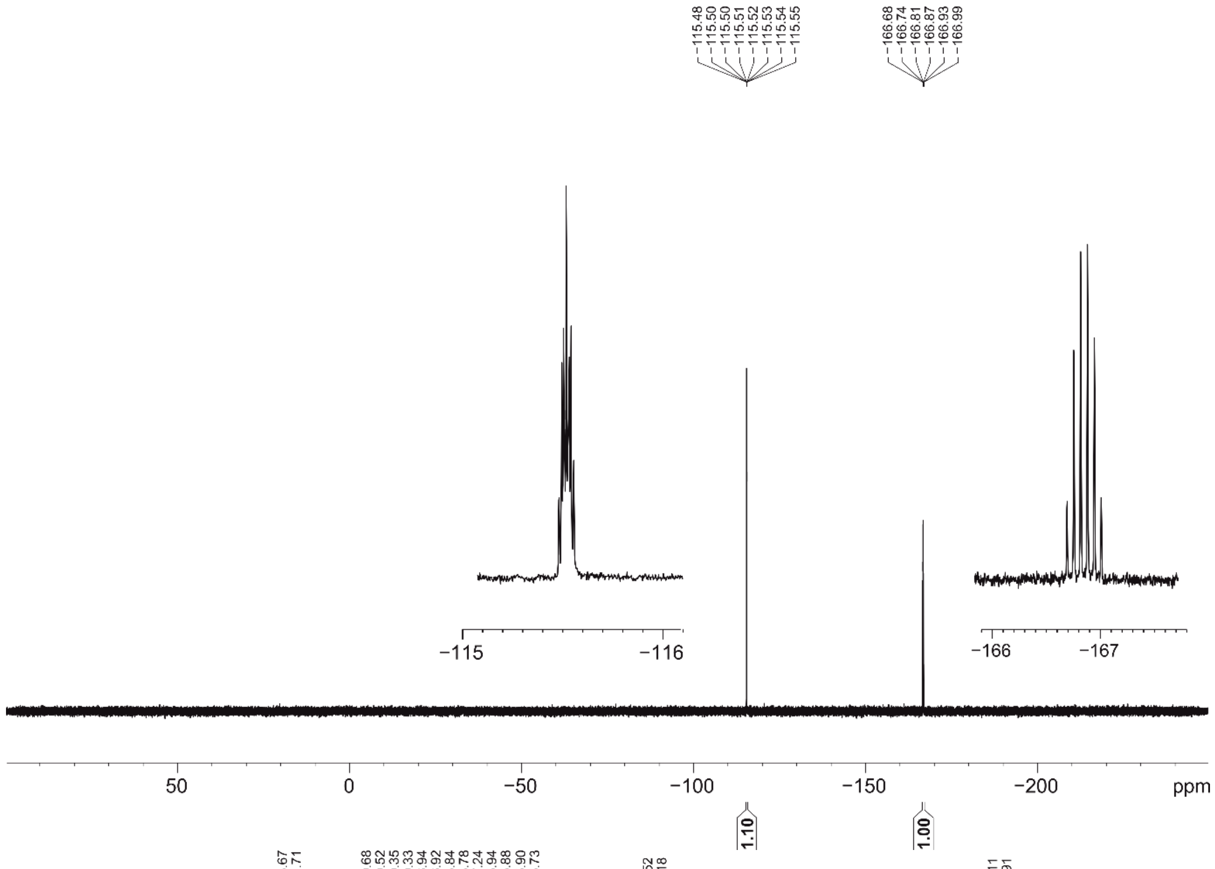

115.48
115.50
115.50
115.51
115.52
115.53
115.54
115.55
166.68
166.74
166.81
166.87
166.93
166.99
−115
−116
−166
−167
50
0
−50
−100
−150
−200
ppm
1.10
1.00


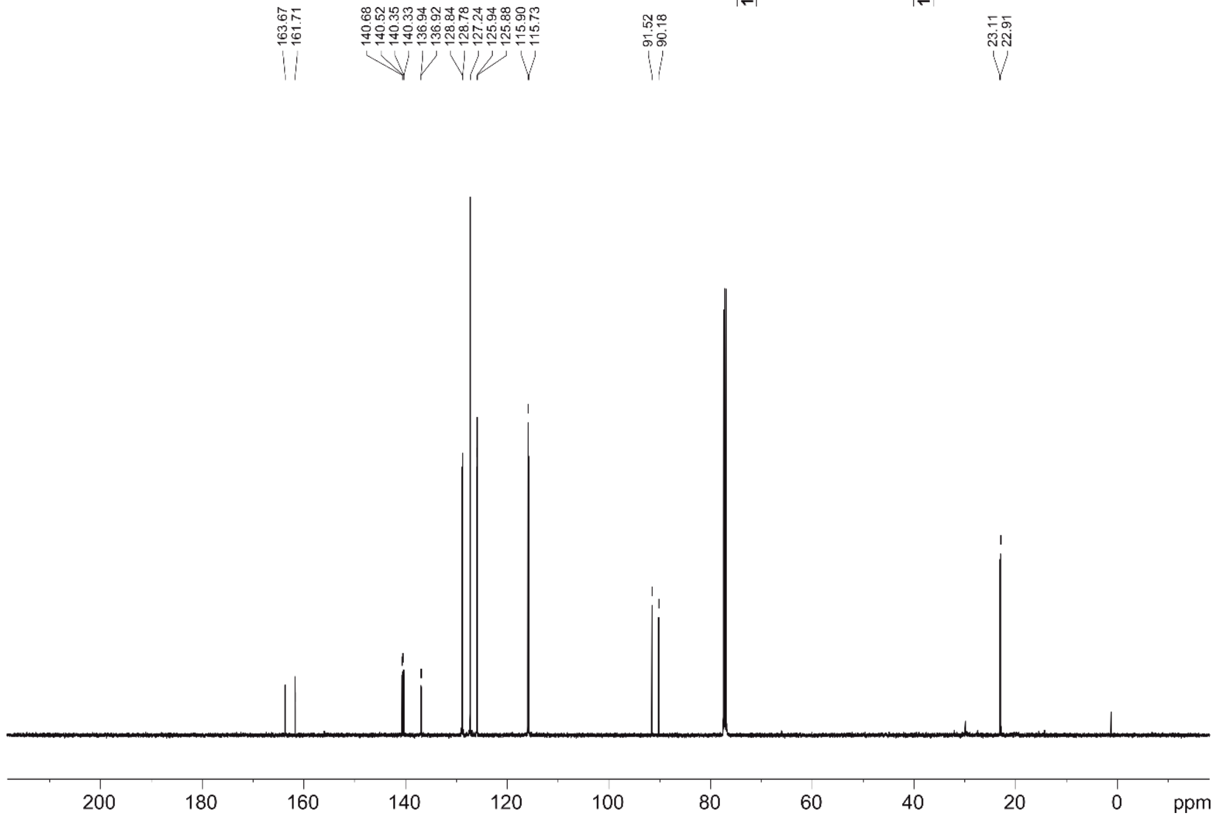

163.67
161.71
140.68
140.52
140.35
140.33
136.94
136.92
128.84
128.78
127.24
125.94
125.88
115.90
115.73
91.52
90.18
23.11
22.91
200
180
160
140
120
100
80
60
40
20
0
ppm

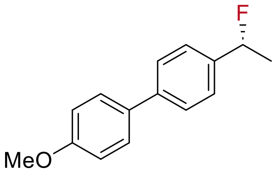


(*R*)-**2c**

## (*R*)-4-(1-fluoroethyl)-4'-methoxy-1,1'-biphenyl (2c)

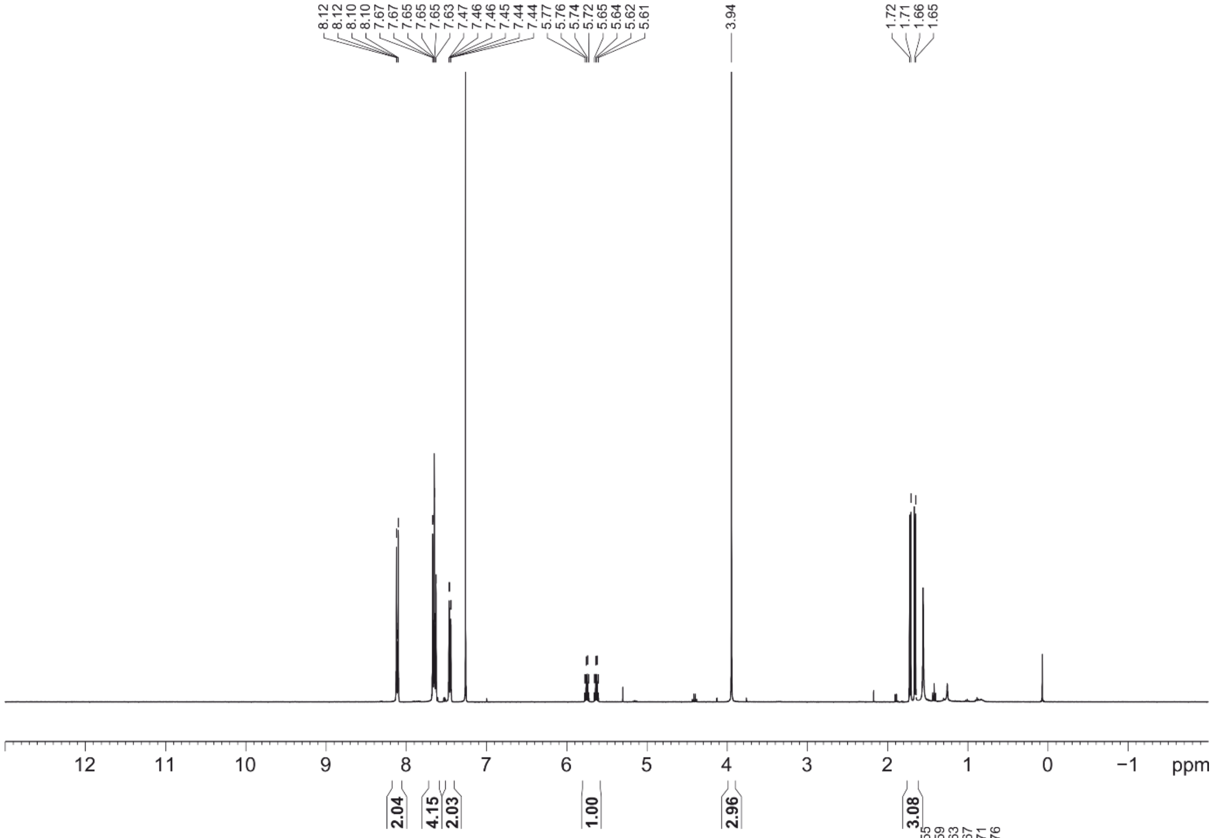


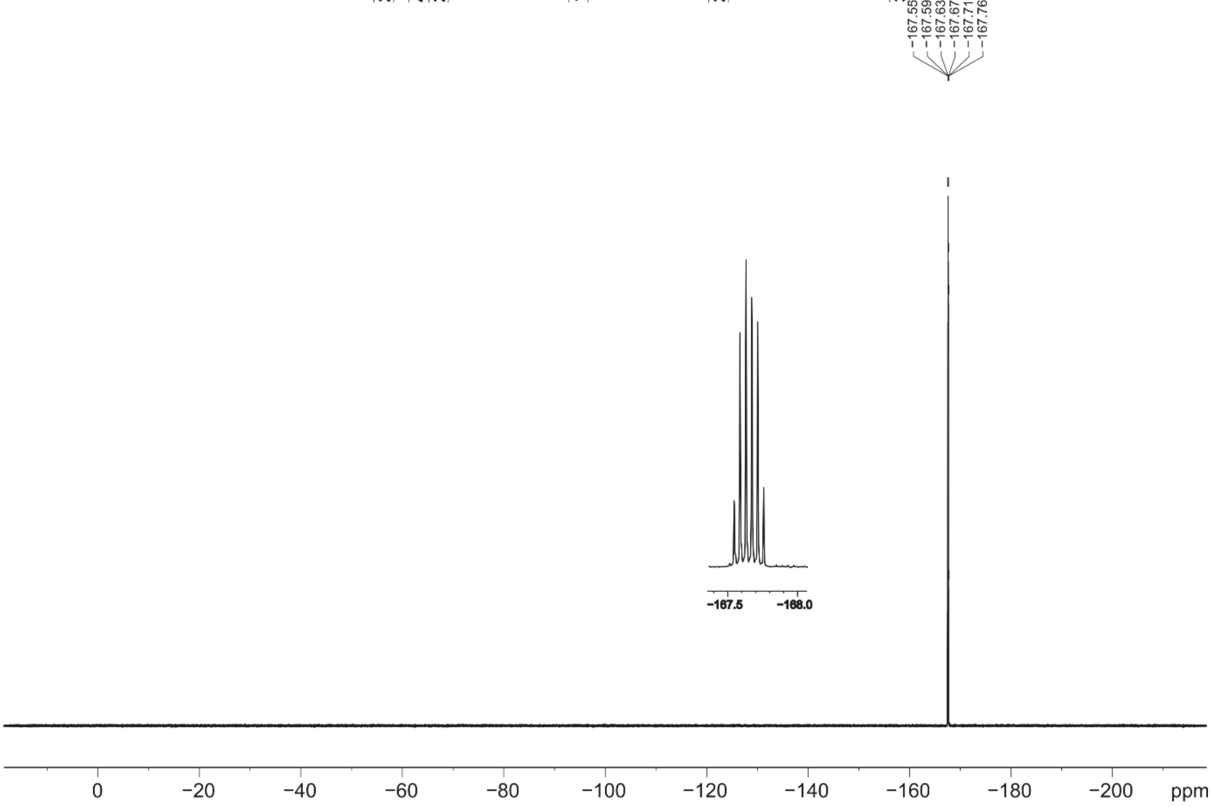

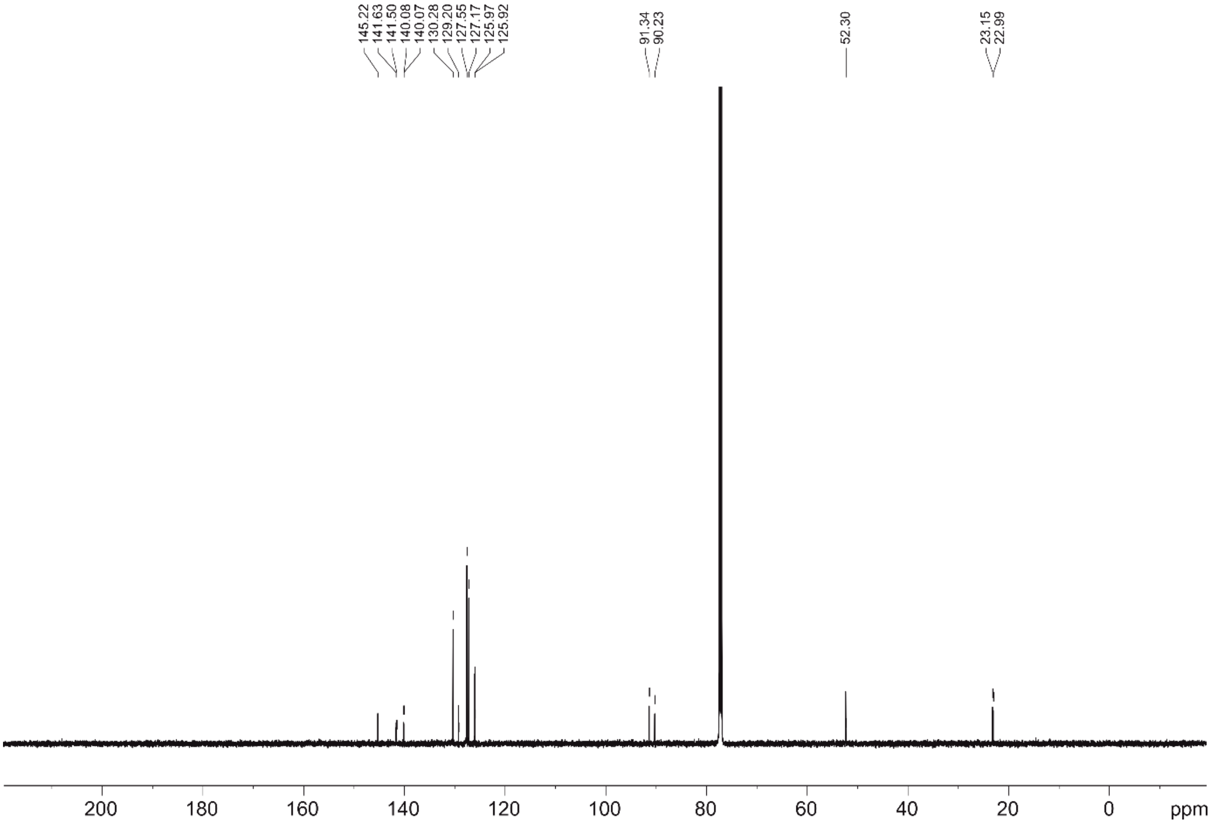


**(*R*)-2-fluoro-4-(1-fluoroethyl)-1,1’-biphenyl (2d)**

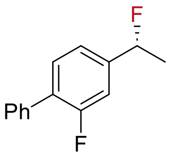


(*R*)-**2d**

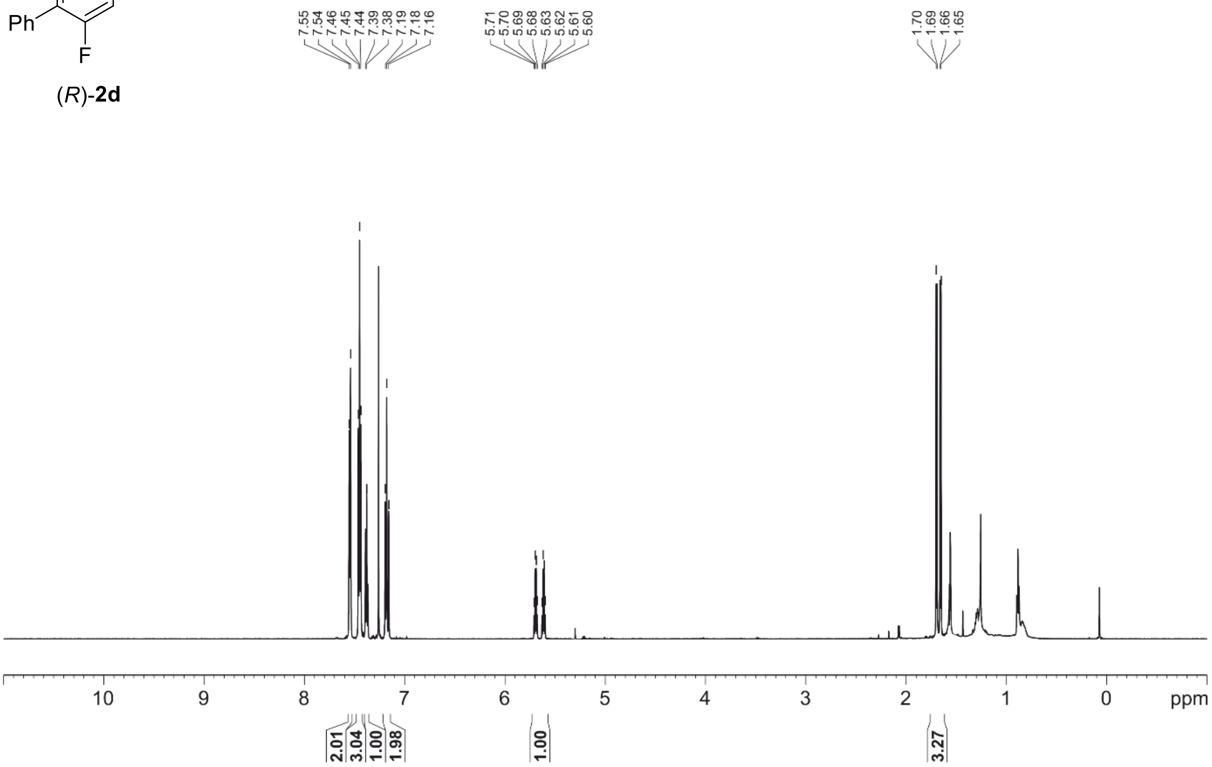

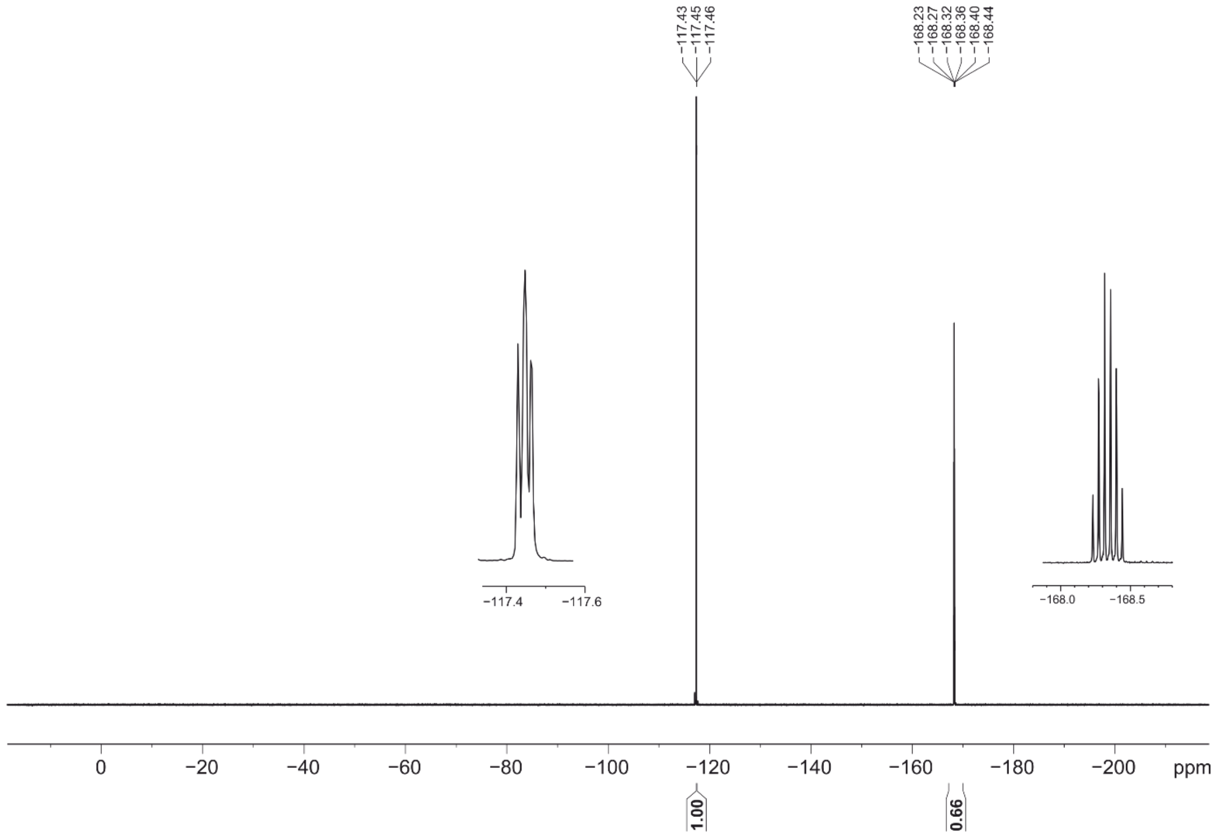
-117.43
-117.45
-117.46
-168.23
-168.27
-168.32
-168.36
-168.40
-168.44
-117.4
-117.6
-168.0
-168.5
0
-20
-40
-60
-80
-100
-120
-140
-160
-180
-200
ppm
1.00
0.66

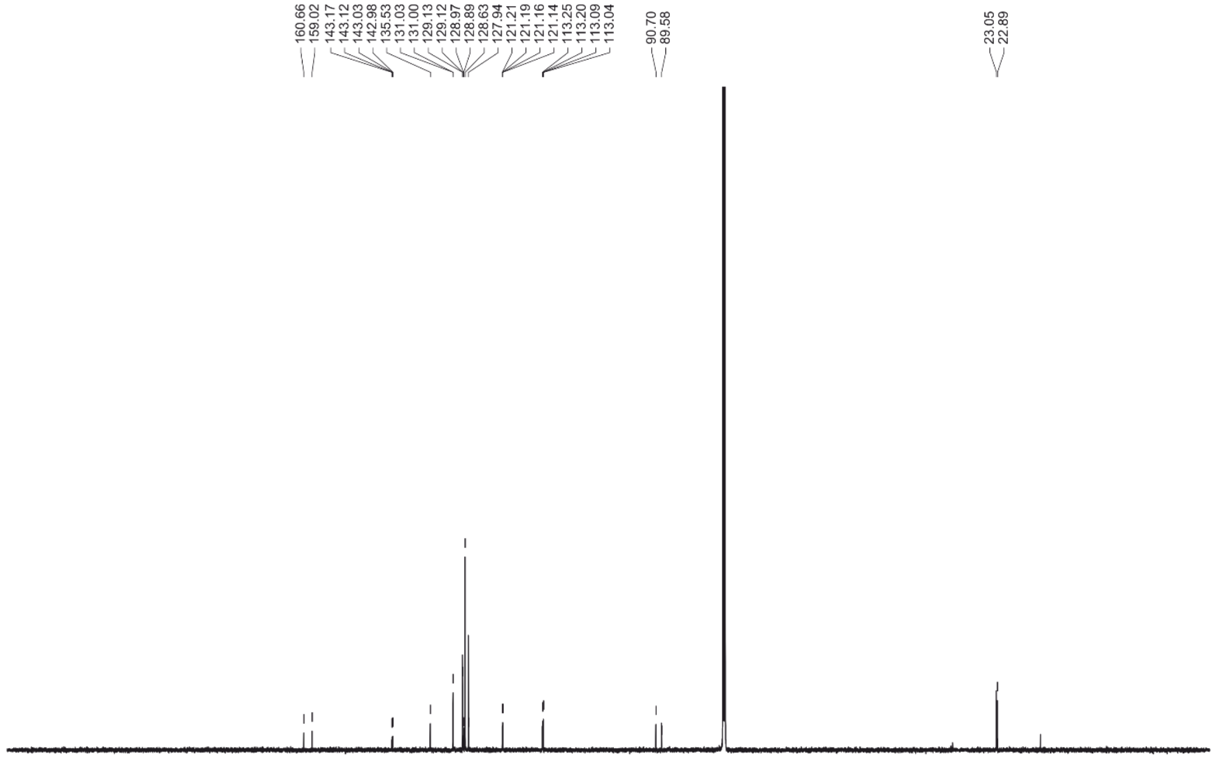
160.66
159.02
143.17
143.12
143.03
142.98
135.53
131.03
131.00
129.13
129.12
128.97
128.89
128.63
127.94
121.21
121.19
121.16
121.14
113.25
113.20
113.09
113.04
90.70
89.58
23.05
22.89
200
180
160
140
120
100
80
60
40
20
0
ppm

**(*R*)-2-(4-(1-fluoroethyl)phenylpyridine (2e)**

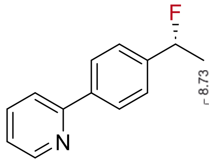


(*R*)-**2e**

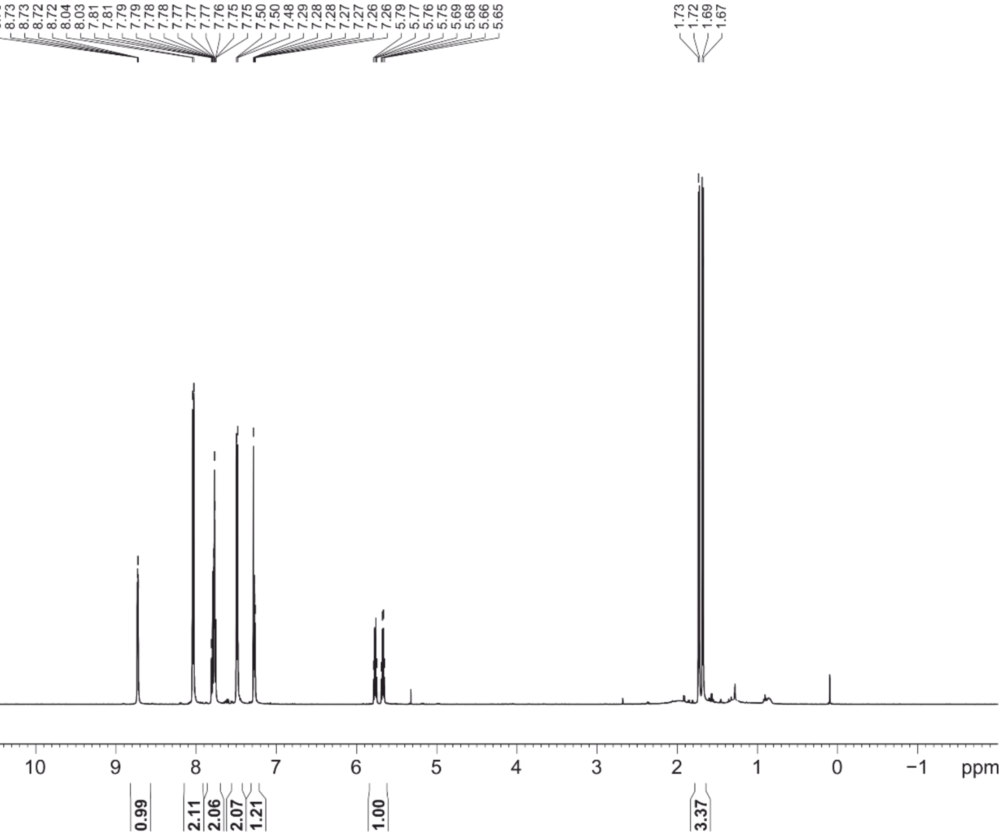


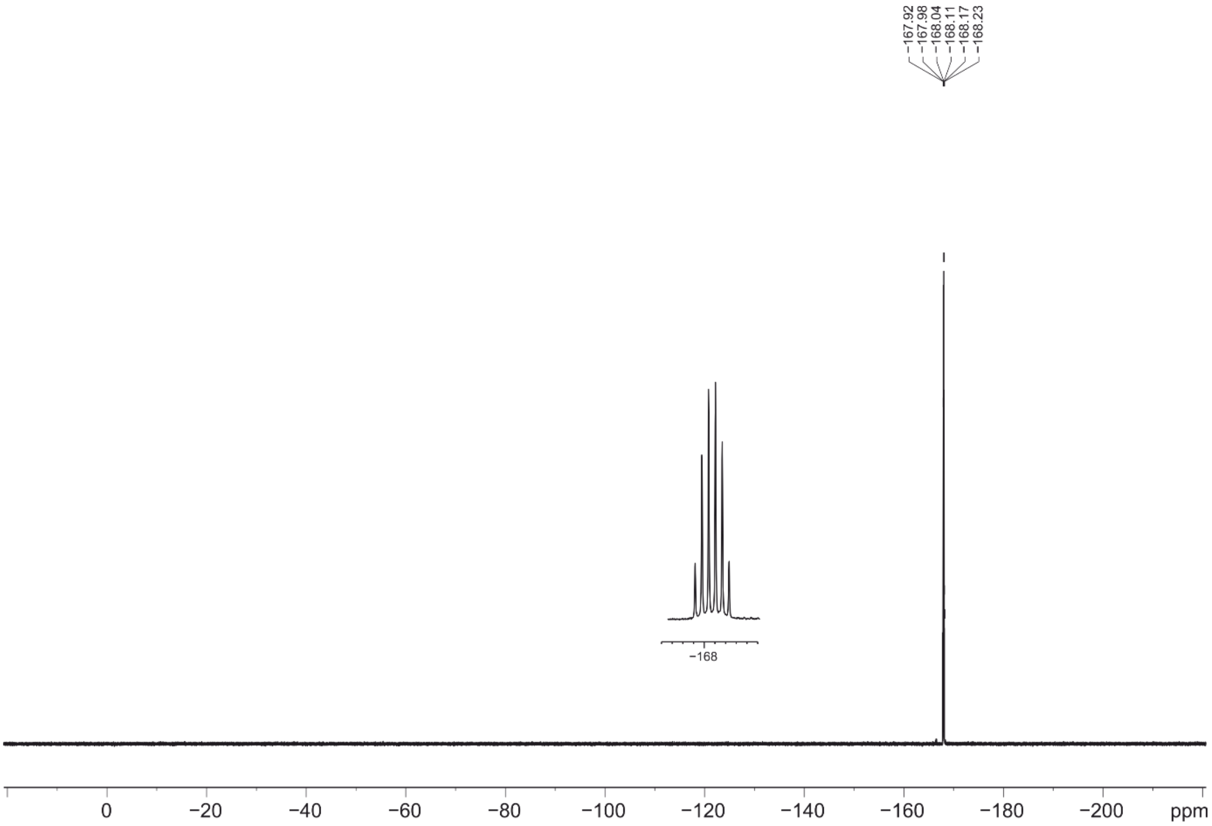

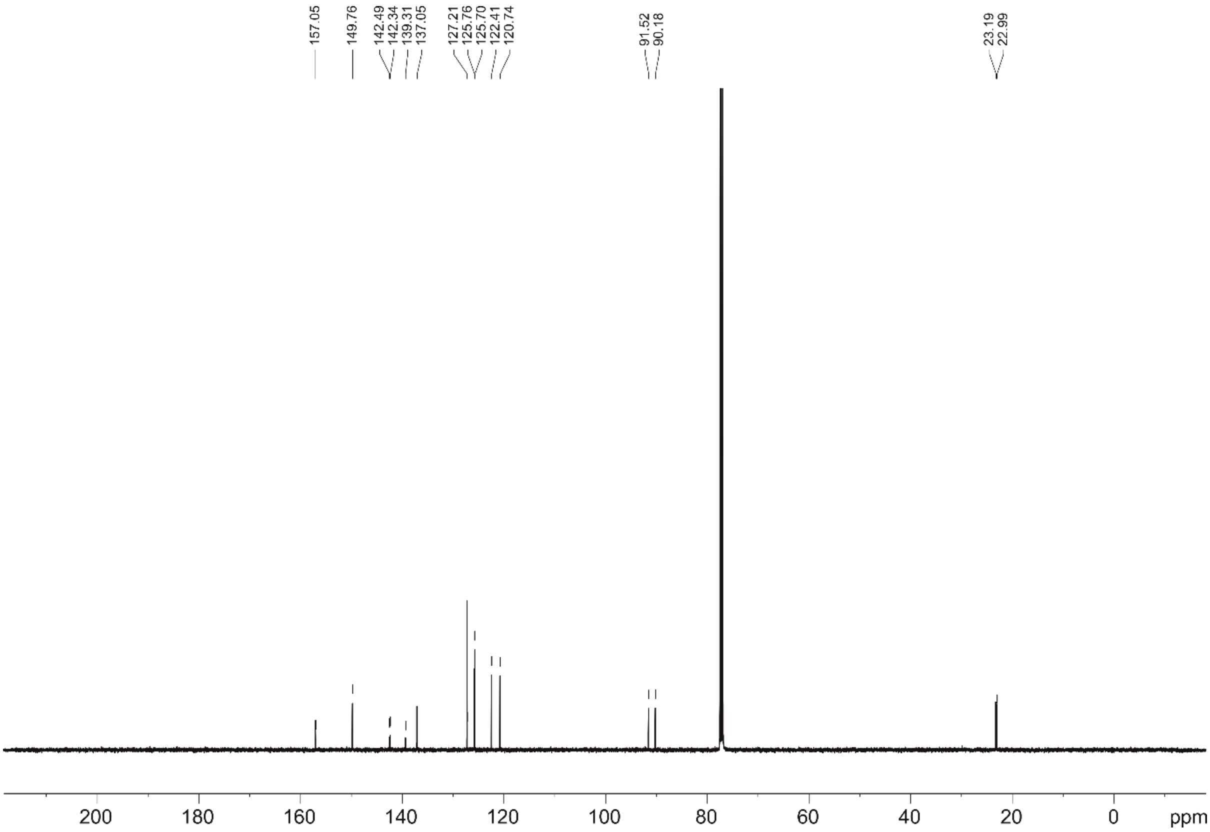


**(*R*)-*N,N*-diallyl-4’-(1-fluoroethyl)-[1,1’-biphenyl]-4-carboxamide (2f)**

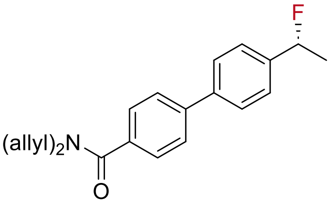


(*R*)-**2f**

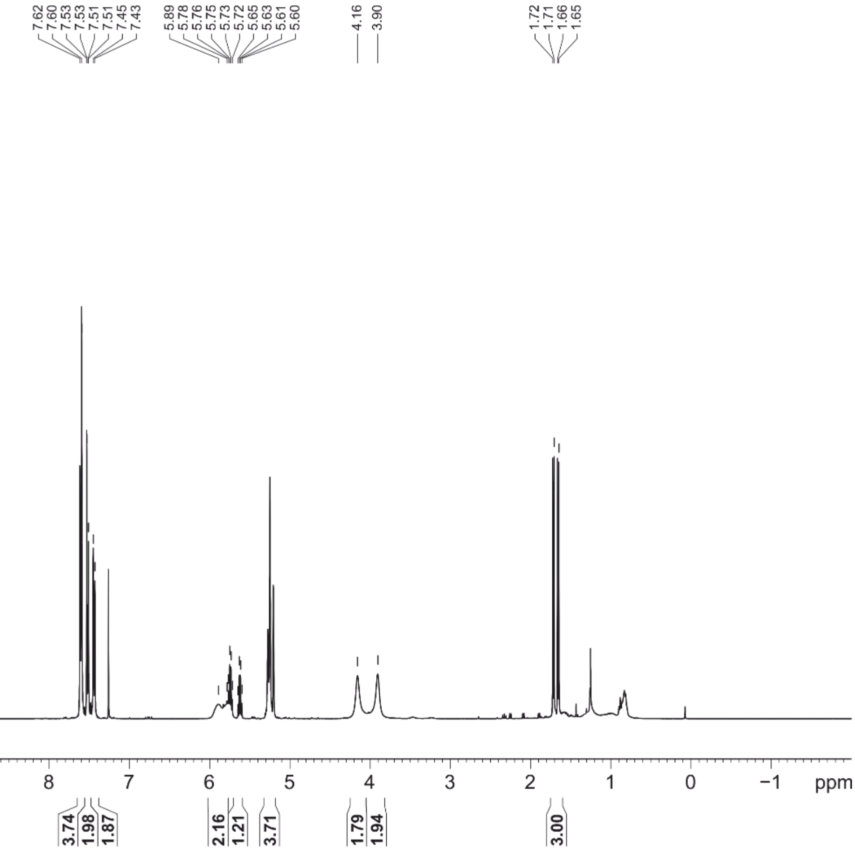

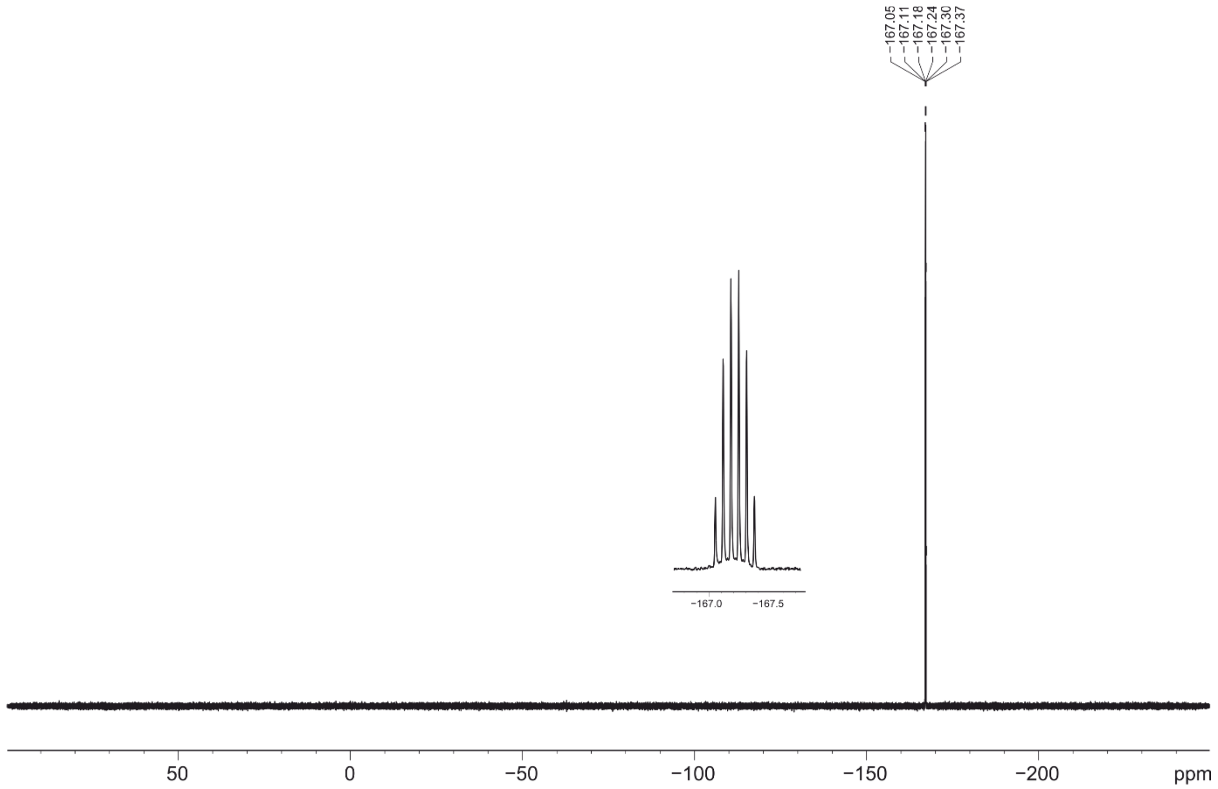

-167.05
-167.11
-167.18
-167.24
-167.30
-167.37
-167.0
-167.5
50
0
−50
−100
−150
−200
ppm


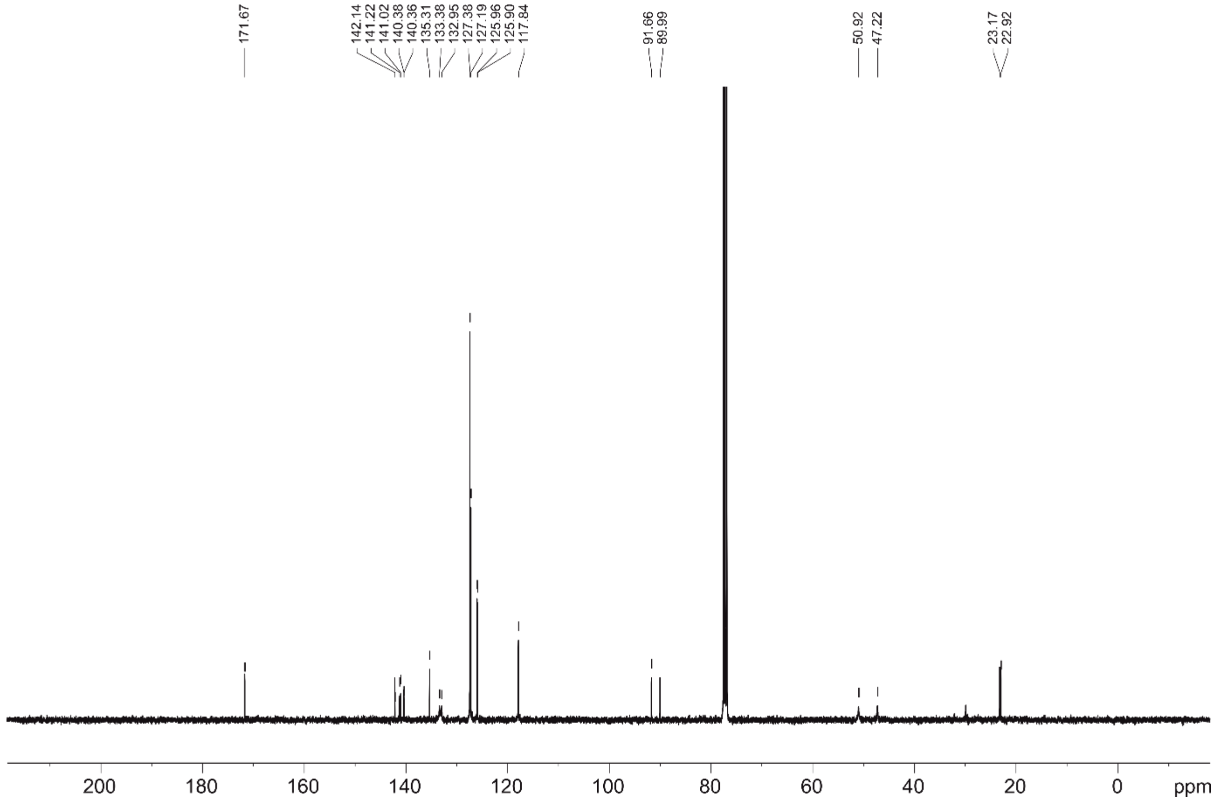

171.67
142.14
141.22
141.02
140.38
140.36
135.31
133.38
132.95
127.38
127.19
125.96
125.90
117.84
91.66
89.99
50.92
47.22
23.17
22.92
200
180
160
140
120
100
80
60
40
20
0
ppm

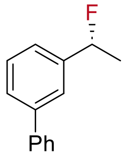

(*R*)-**2g**

**(*R*)-3-(1-fluoroethyl)-1,1′-biphenyl (2g)**

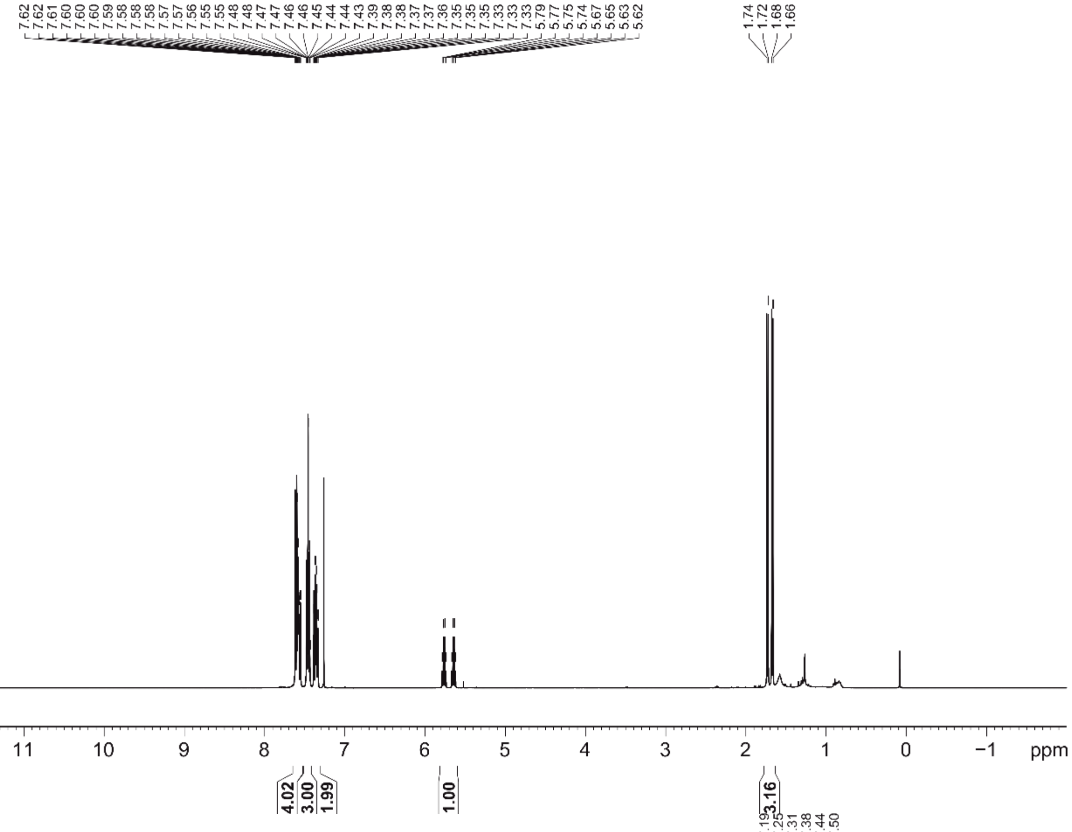

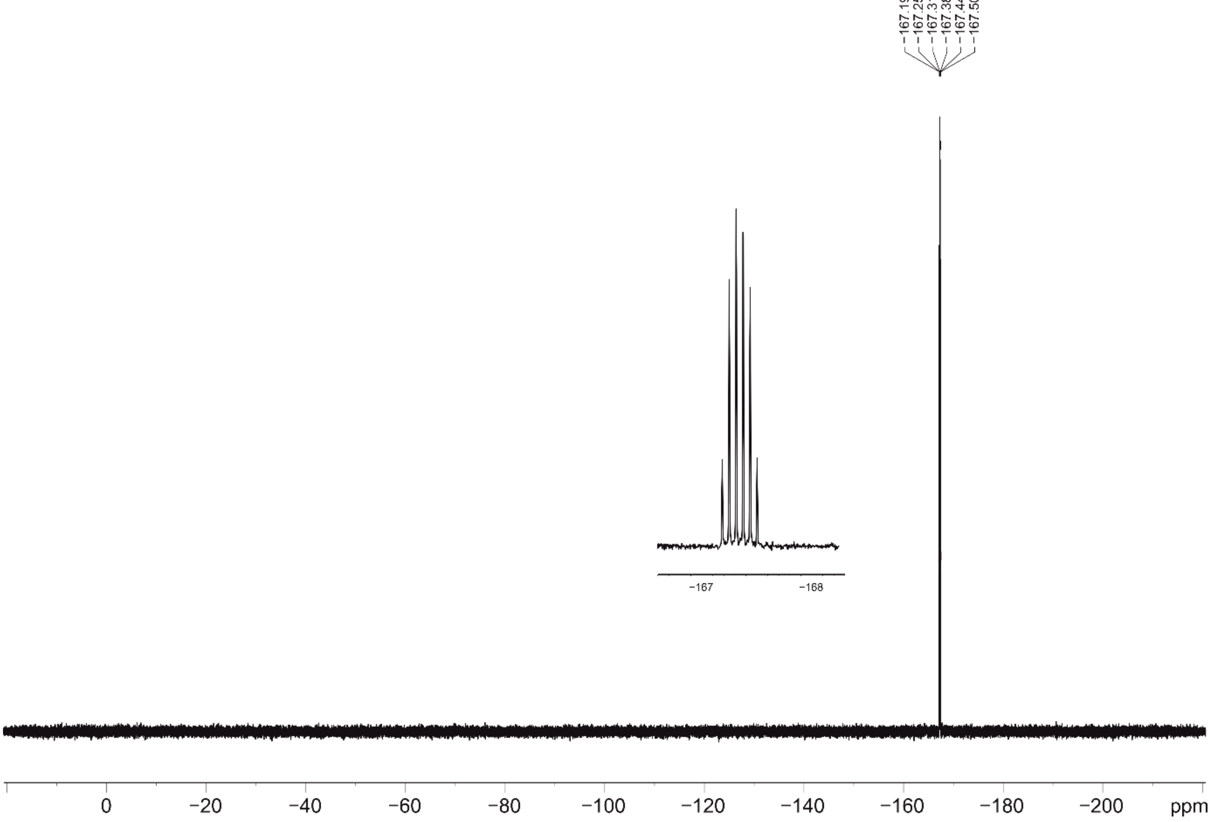

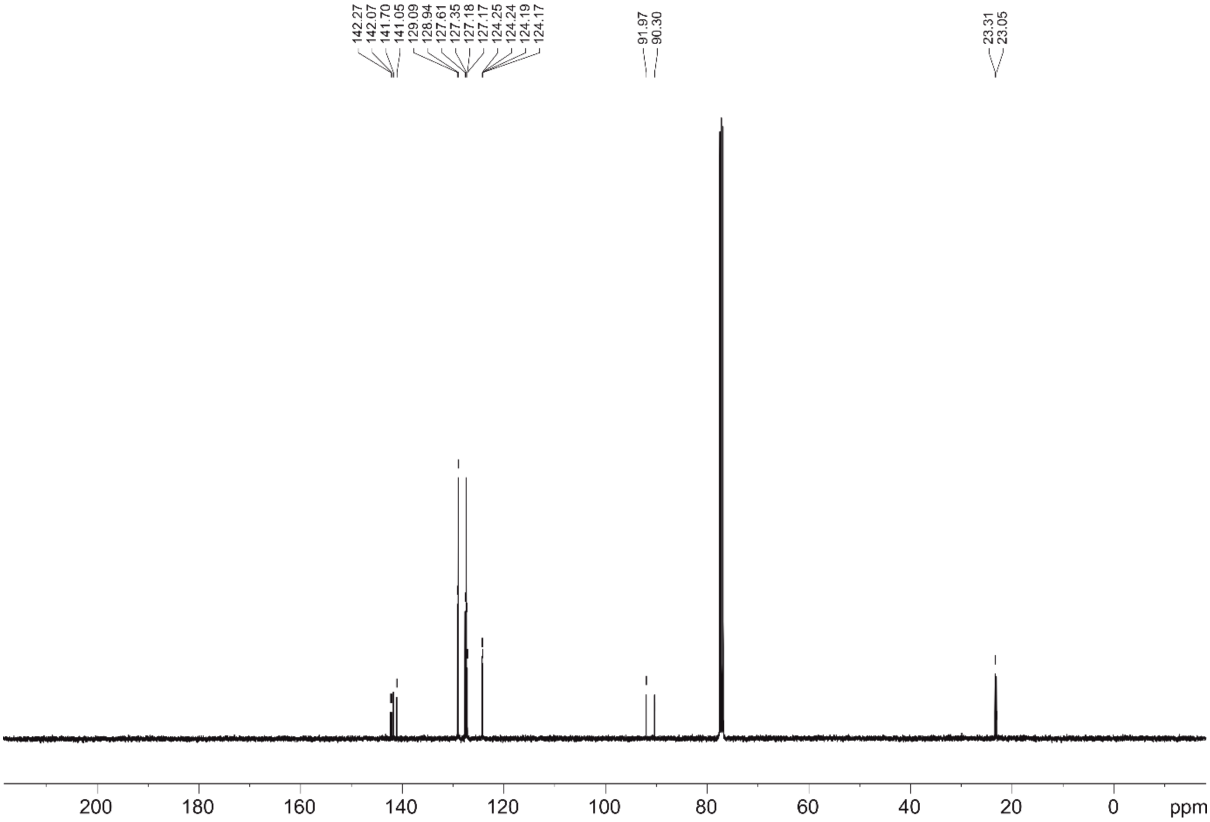


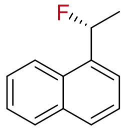


**(*R*)-1-(1-fluoroethyl)naphthalene (2h)**

(*R*)-**2h**

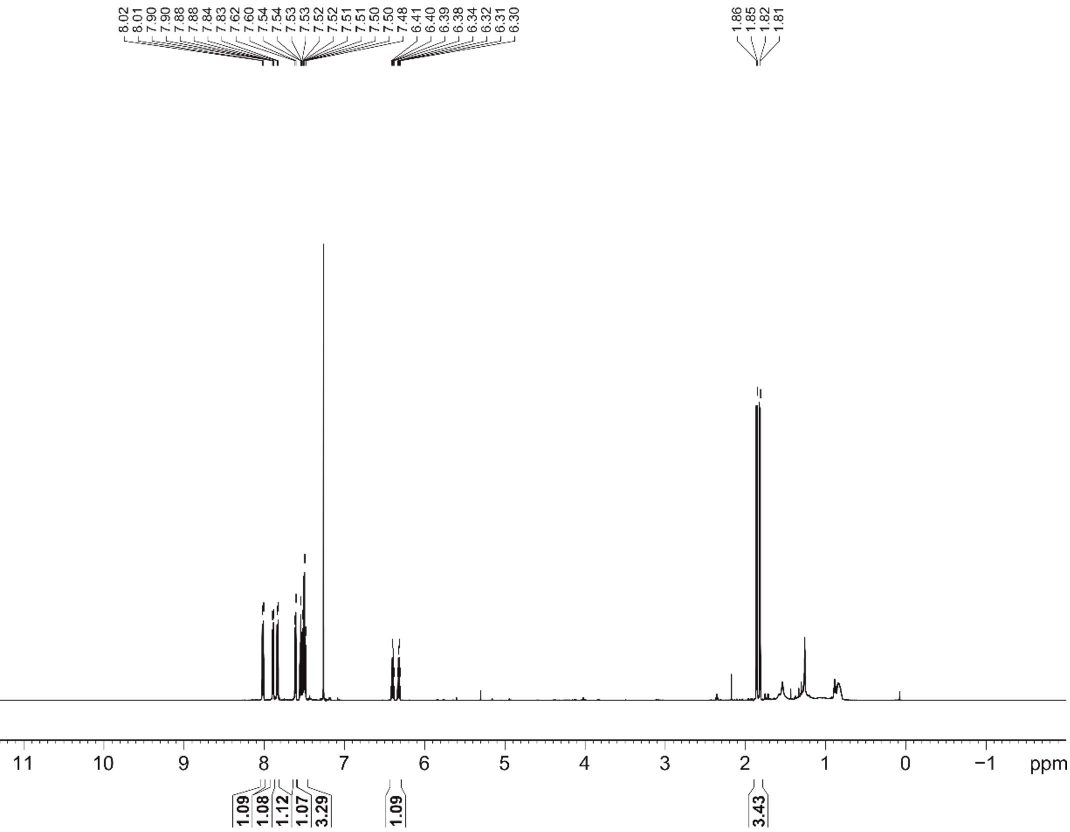

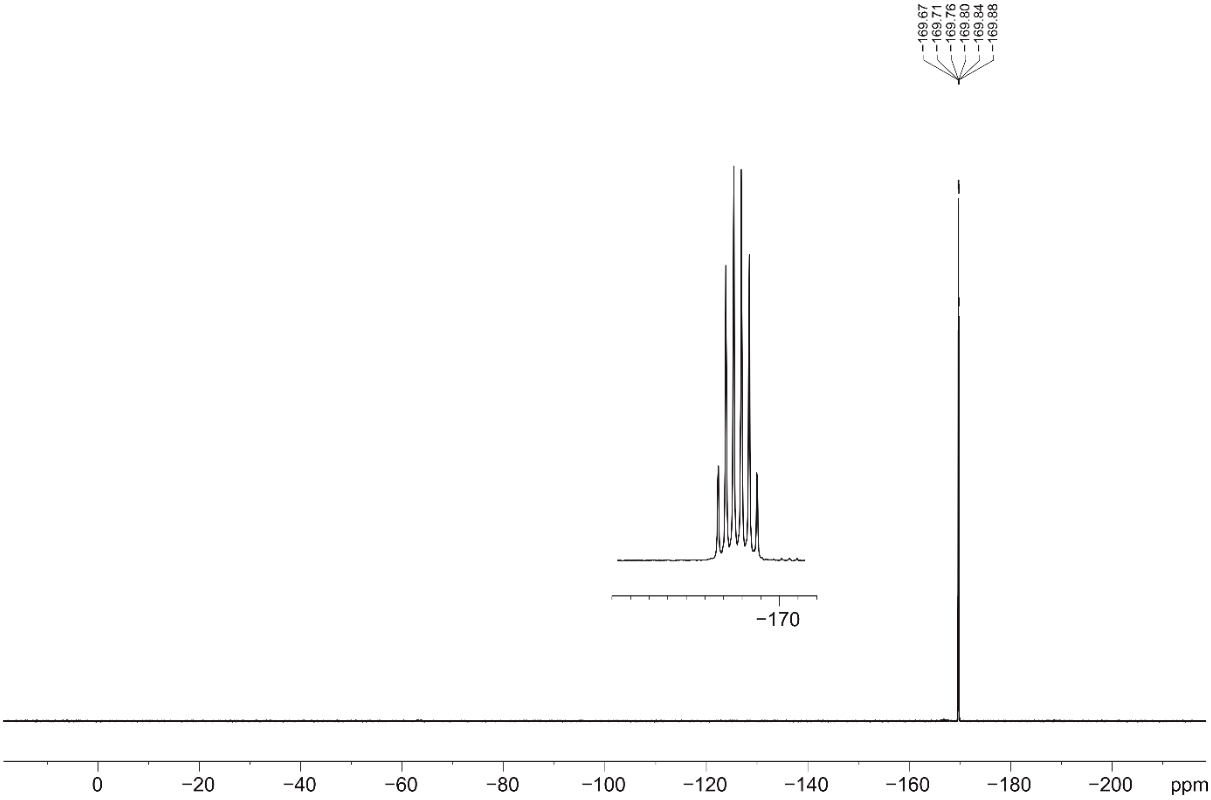
169.67
169.71
169.76
169.80
169.84
169.88
−170
0
−20
−40
−60
−80
−100
−120
−140
−160
−180
−200
ppm

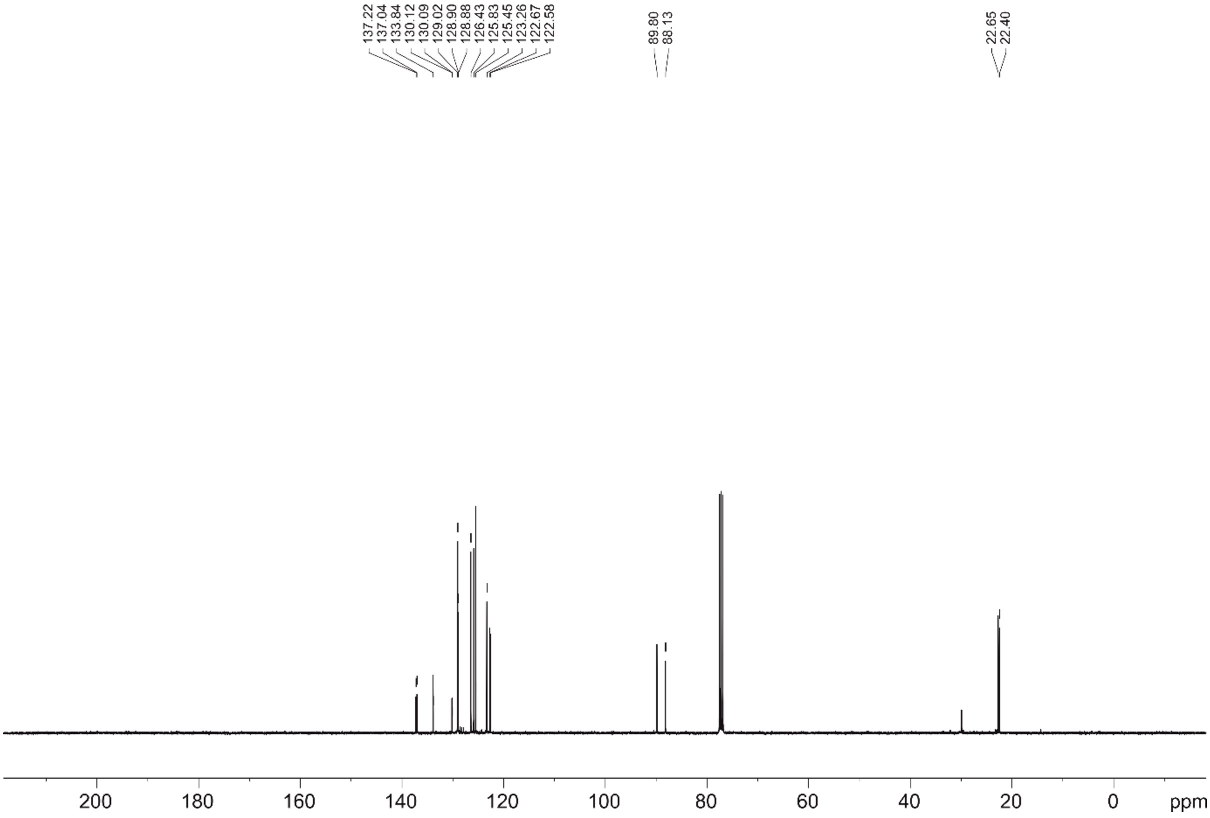
137.22
137.04
133.84
130.12
130.09
129.02
128.90
128.88
126.43
125.83
125.45
123.26
122.67
122.58
89.80
88.13
22.65
22.40
200
180
160
140
120
100
80
60
40
20
0
ppm

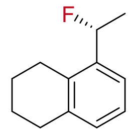


(*R*)-**2i**

**5-(1-fluoroethyl)-1,2,3,4-tetrahydronaphthalene (2i)**

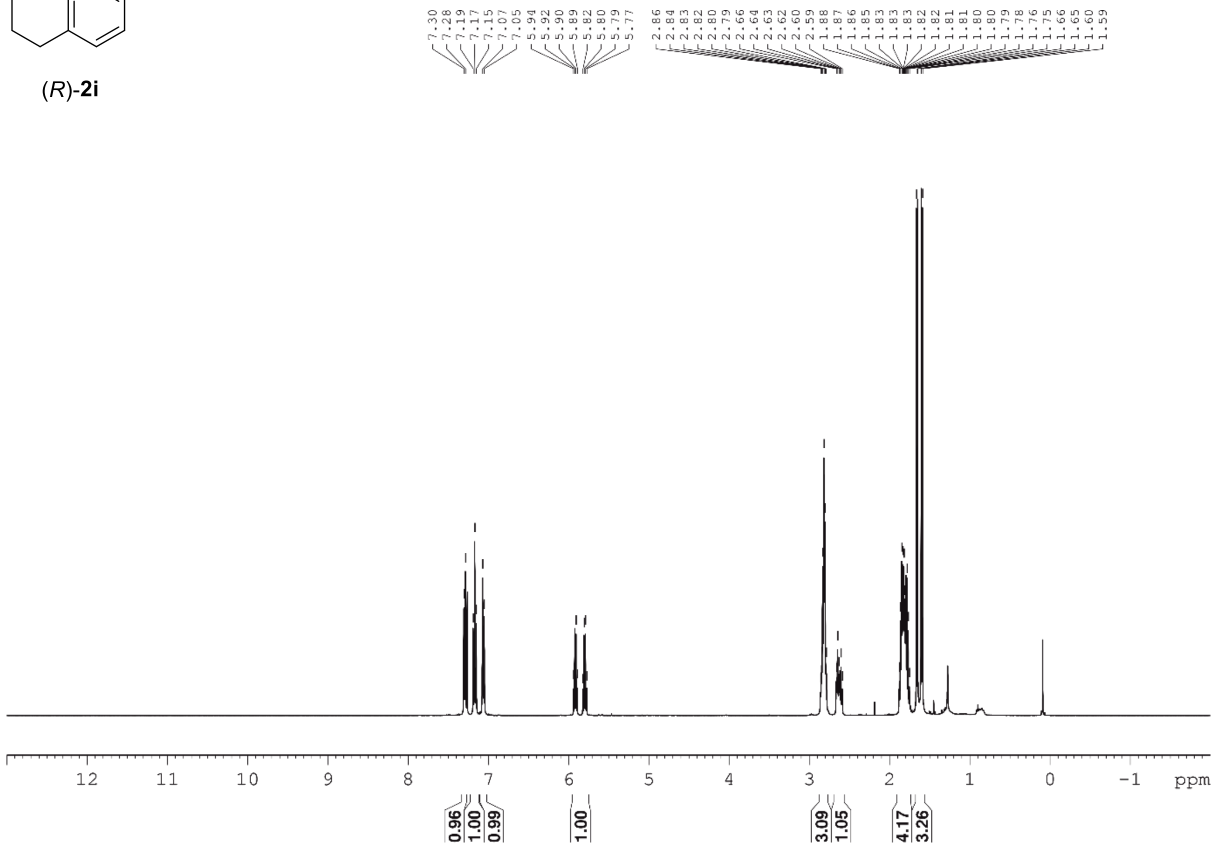


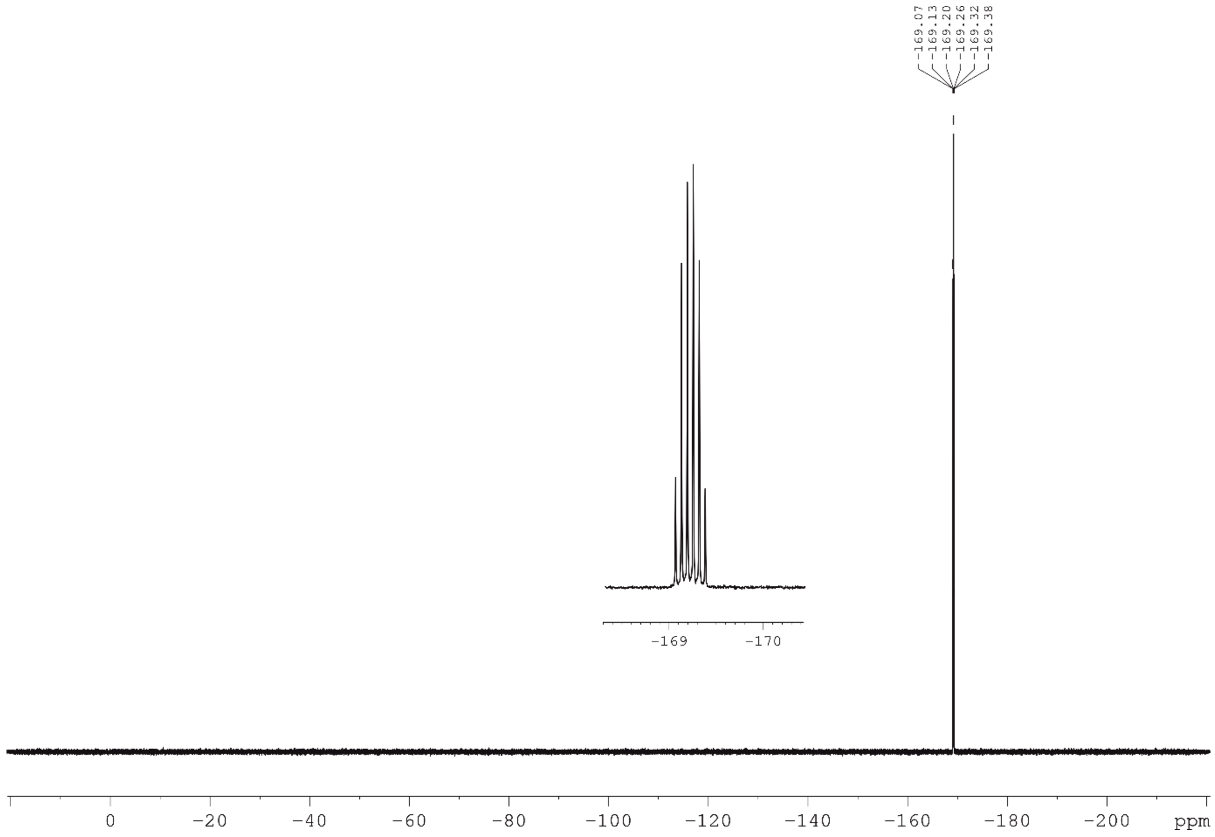

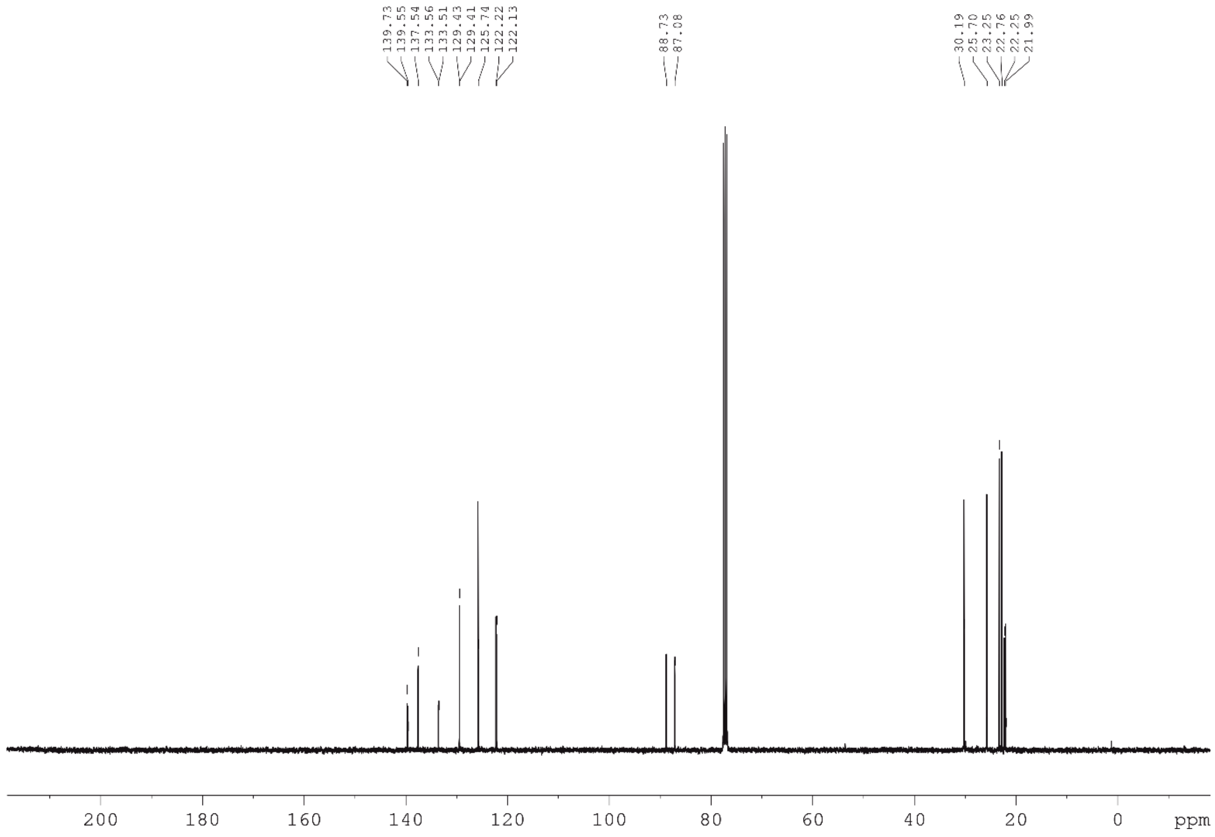


**(*R*)-9-(1-fluoroethyl)phenanthrene (2j)**

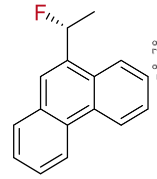


(*R*)-**2j**

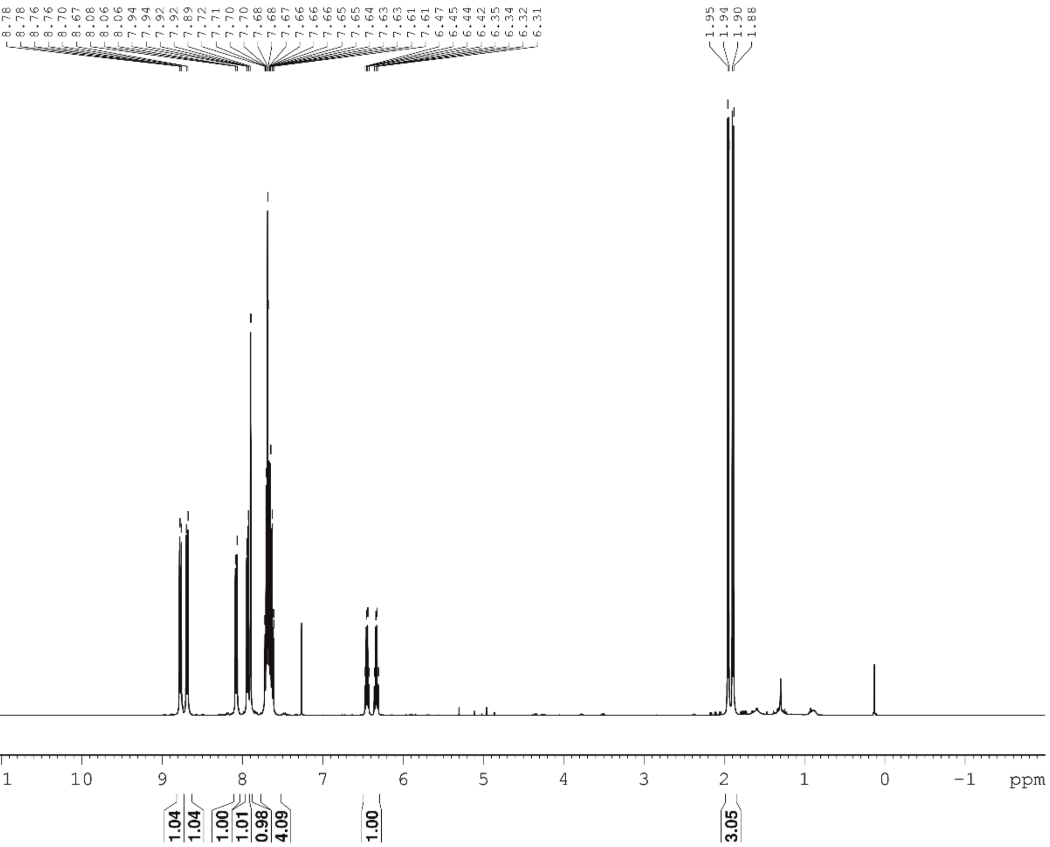

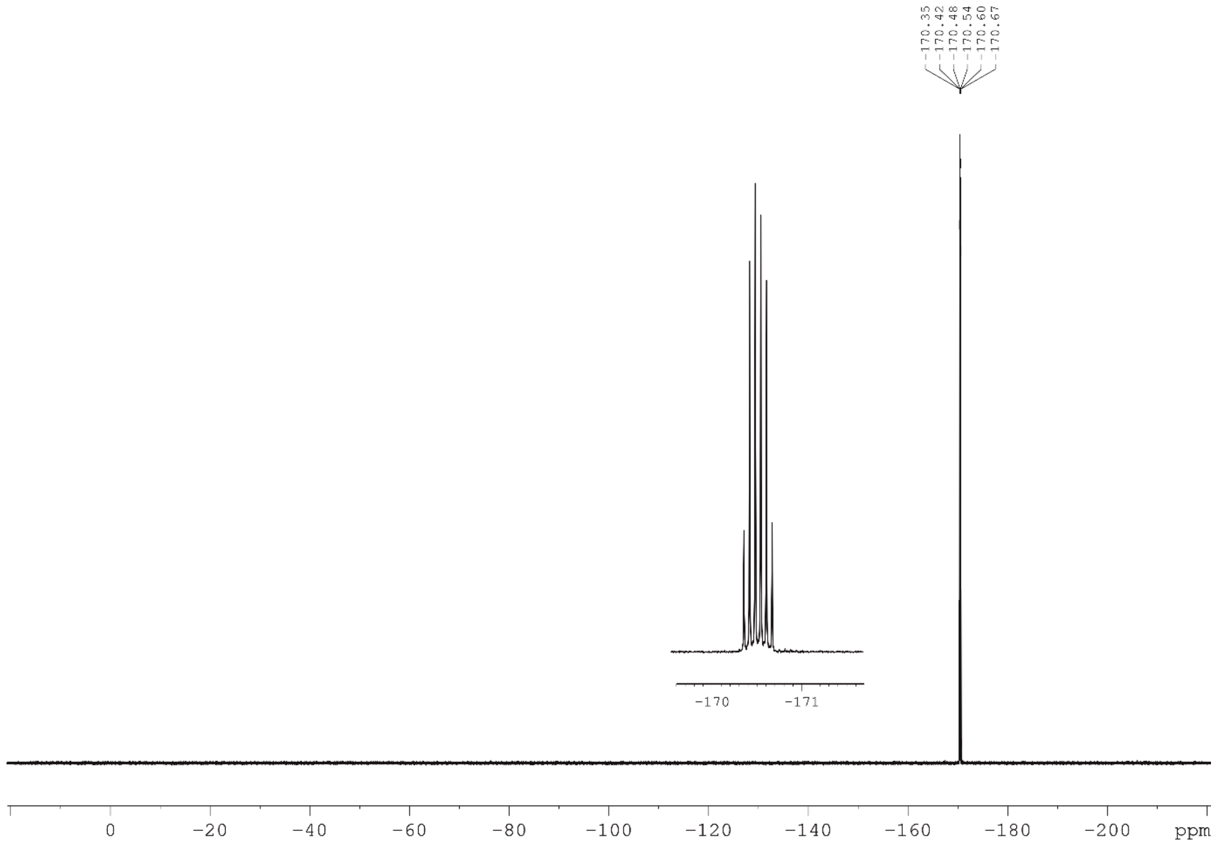

-170.35
-170.42
-170.48
-170.54
-170.60
-170.67
-170
-171
0
-20
-40
-60
-80
-100
-120
-140
-160
-180
-200
ppm


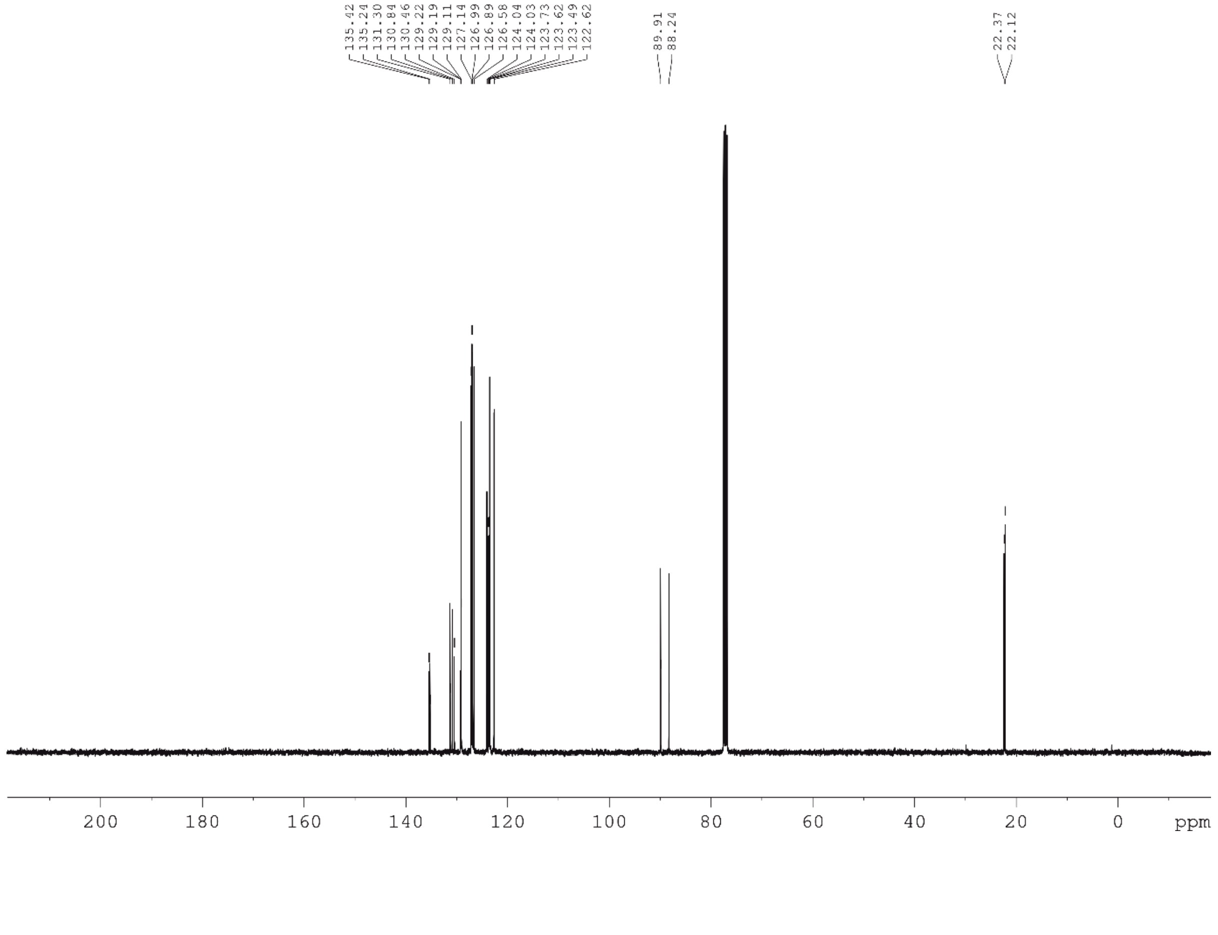

135.42
135.24
131.30
130.84
130.46
129.22
129.19
129.11
127.14
126.99
126.89
126.58
124.04
124.03
123.73
123.62
123.49
122.62
89.91
88.24
22.37
22.12
200
180
160
140
120
100
80
60
40
20
0
ppm

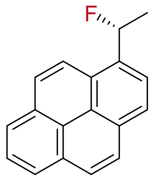


**1-(1-fluoroethyl)pyrene (2k)**

(*R*)-**2k**

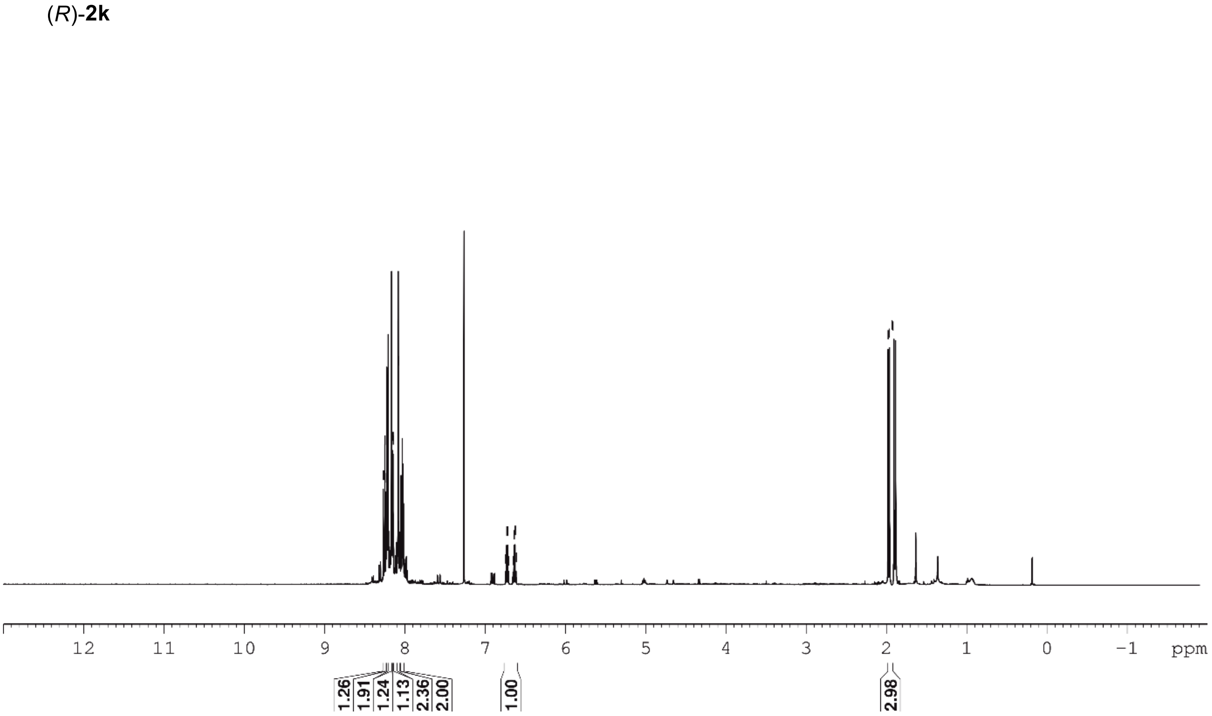


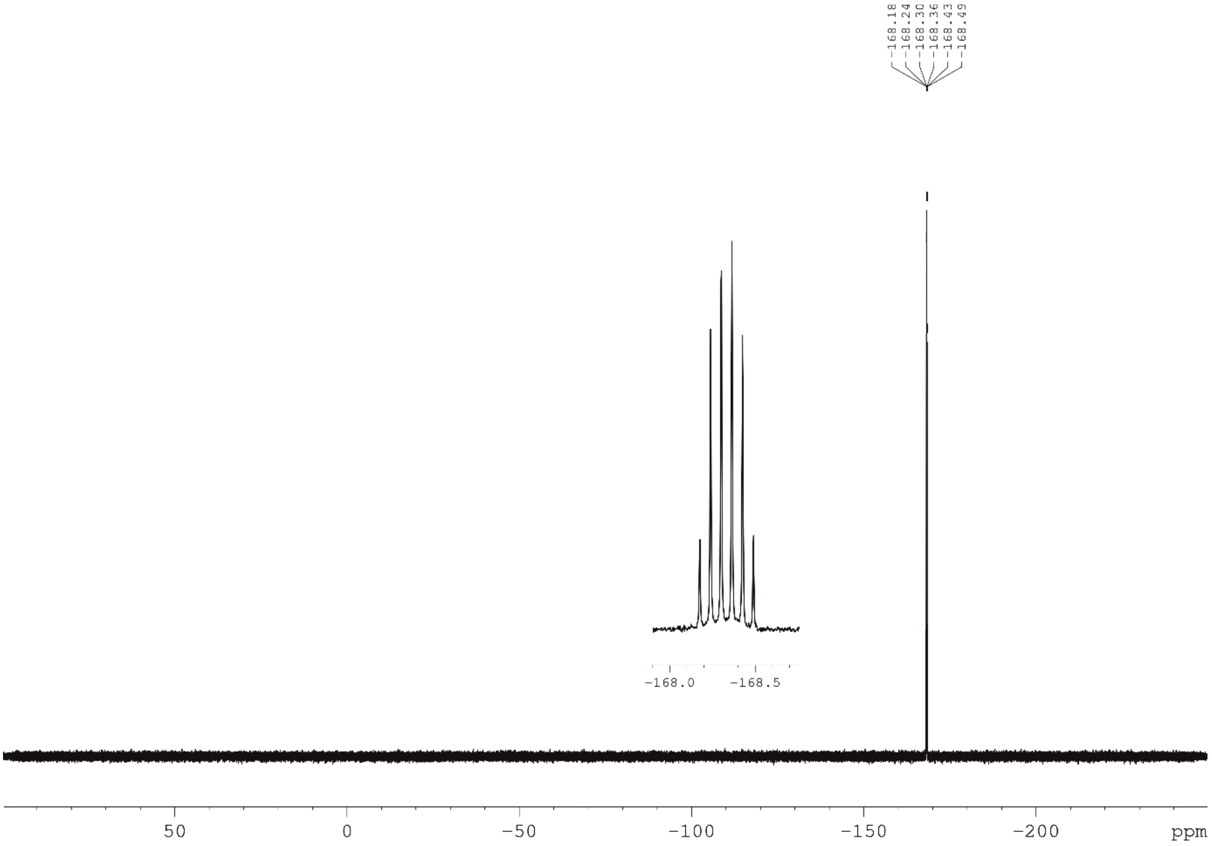

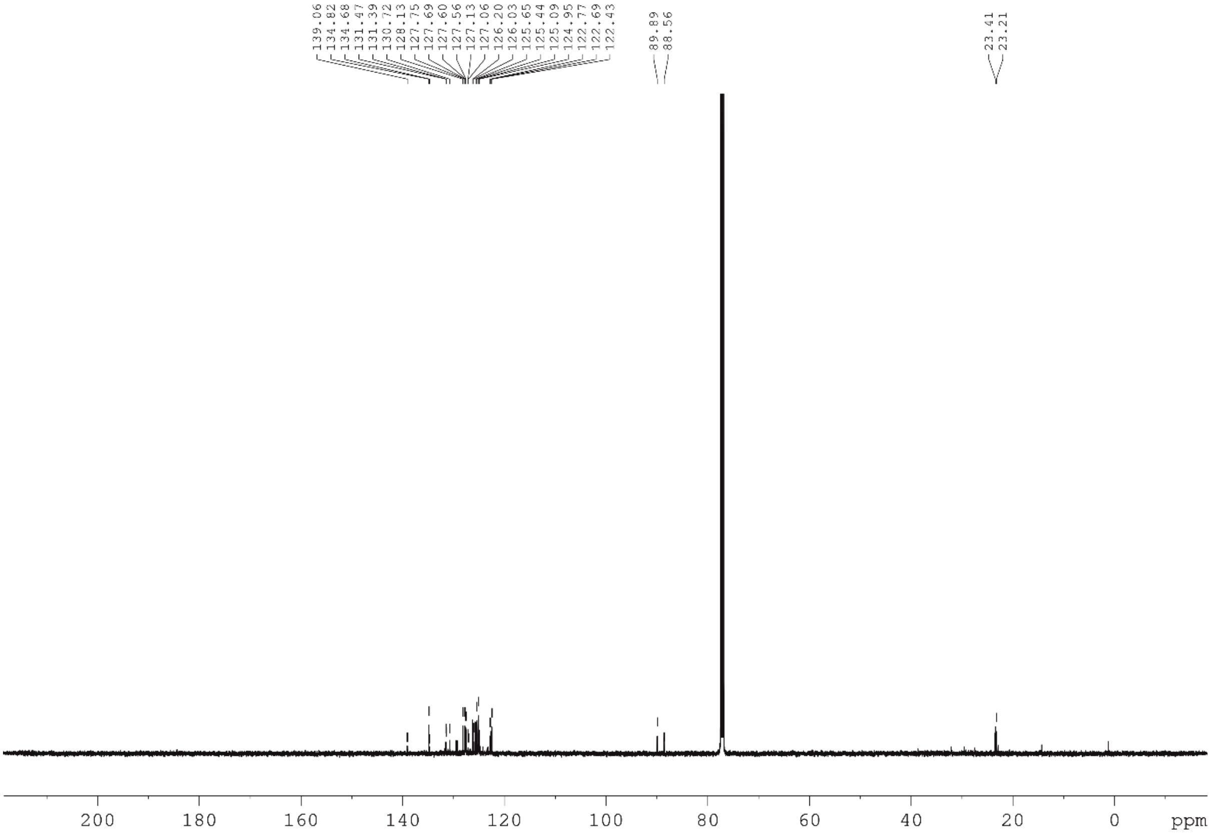


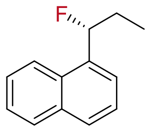


(*R*)-**2k**

## (*R*)-1-(1-fluoropropyl)naphthalene (2k)

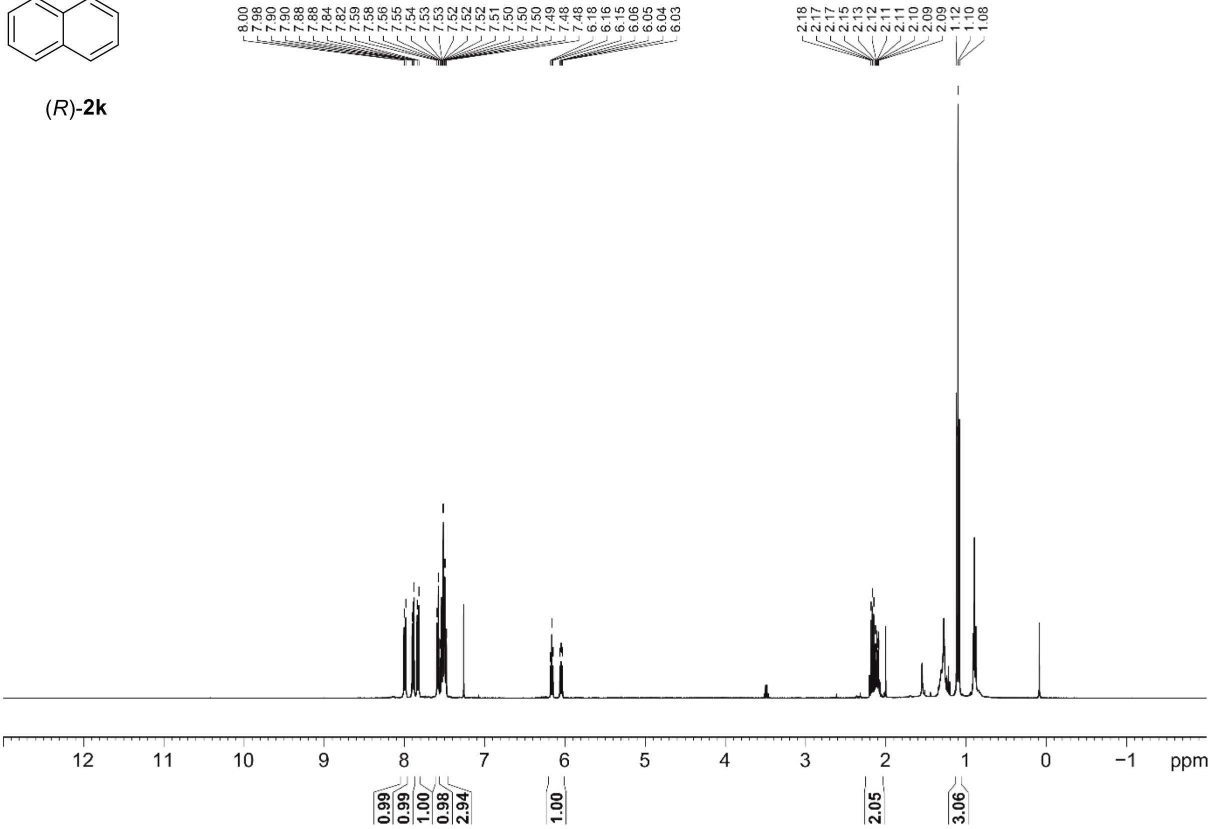

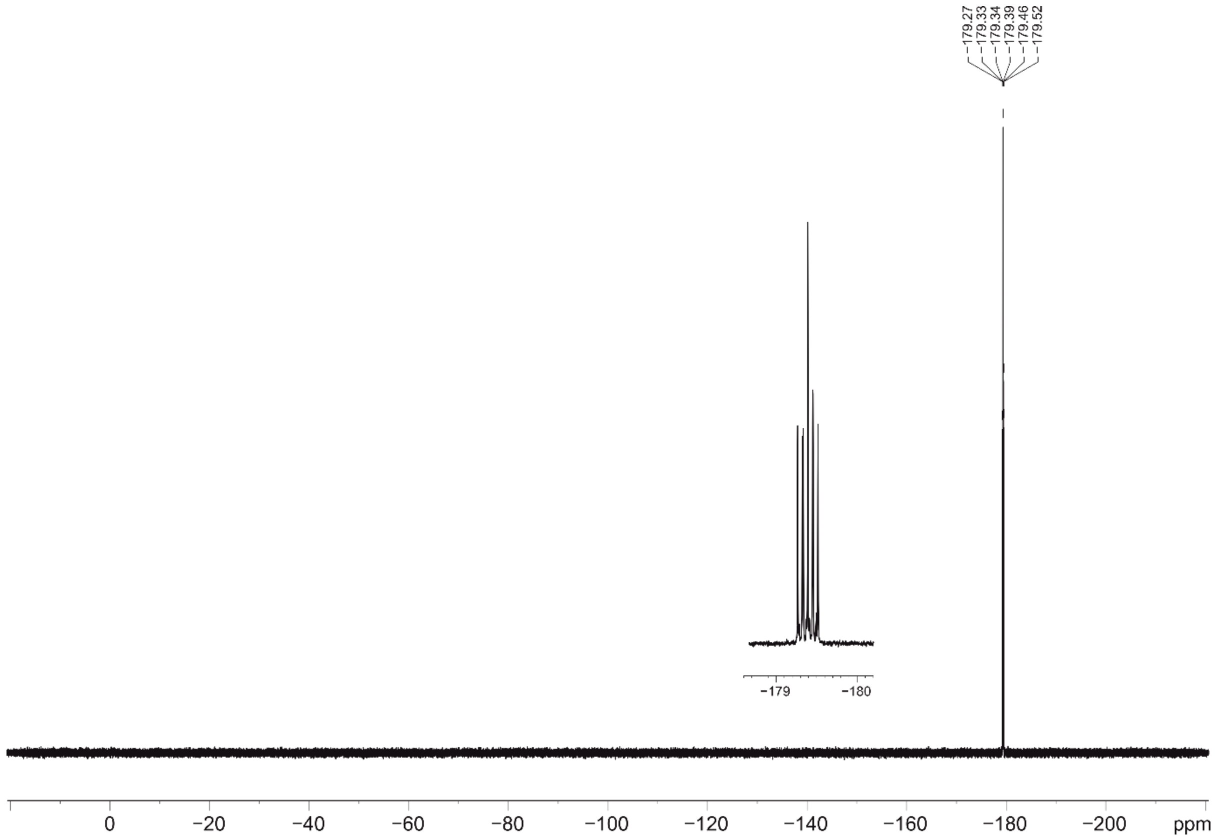
-179.27
-179.33
-179.34
-179.39
-179.46
-179.52
-179
-180
0
−20
−40
−60
−80
−100
−120
−140
−160
−180
−200
ppm

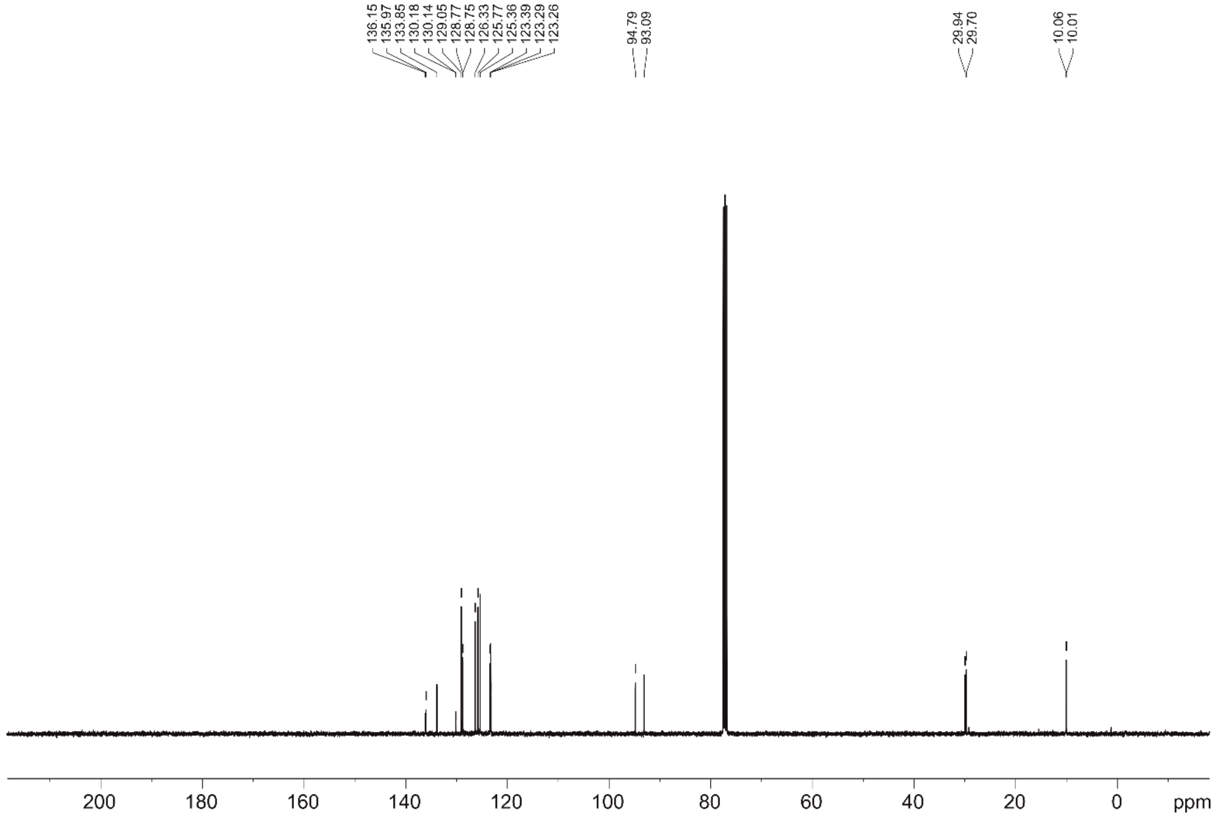
136.15
135.97
133.85
130.18
130.14
129.05
128.77
128.75
126.33
125.77
125.36
123.39
123.29
123.26
94.79
93.09
29.94
29.70
10.06
10.01
200
180
160
140
120
100
80
60
40
20
0
ppm

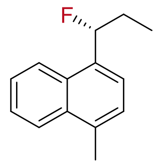


(*R*)-**2l**

**(*R*)-1-(1-fluoropropyl)-4-methylnaphthalene (2l)**

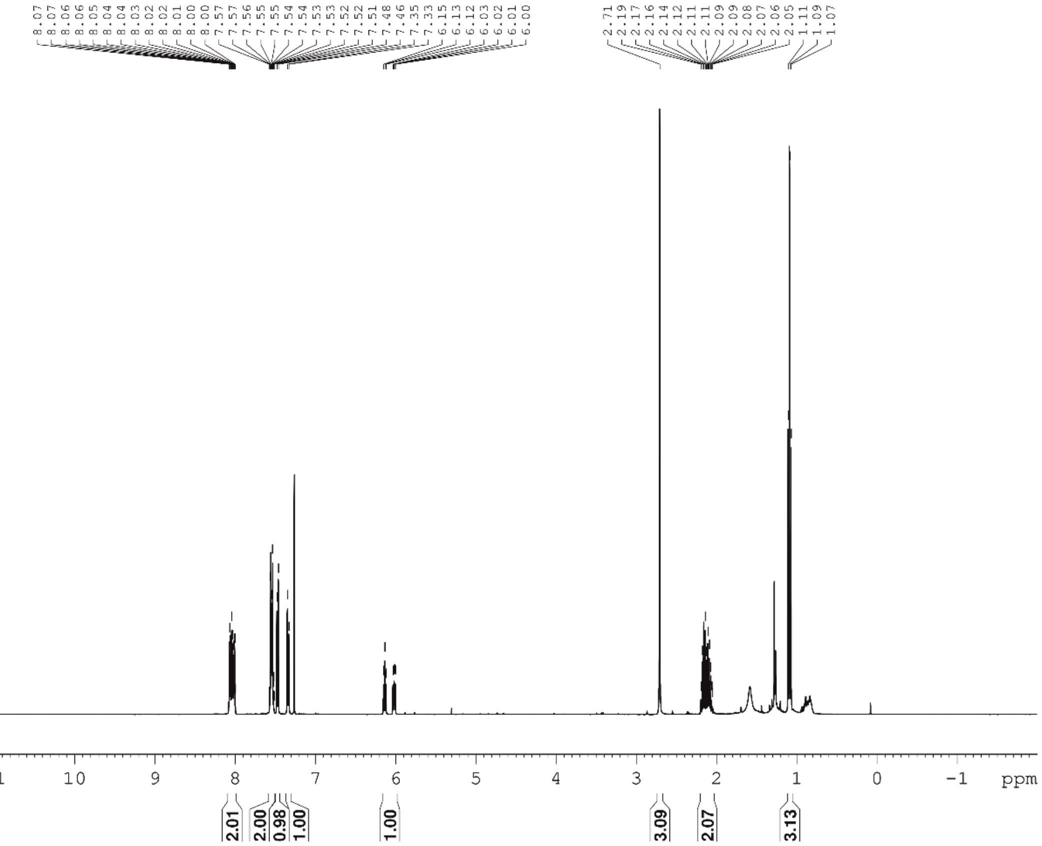


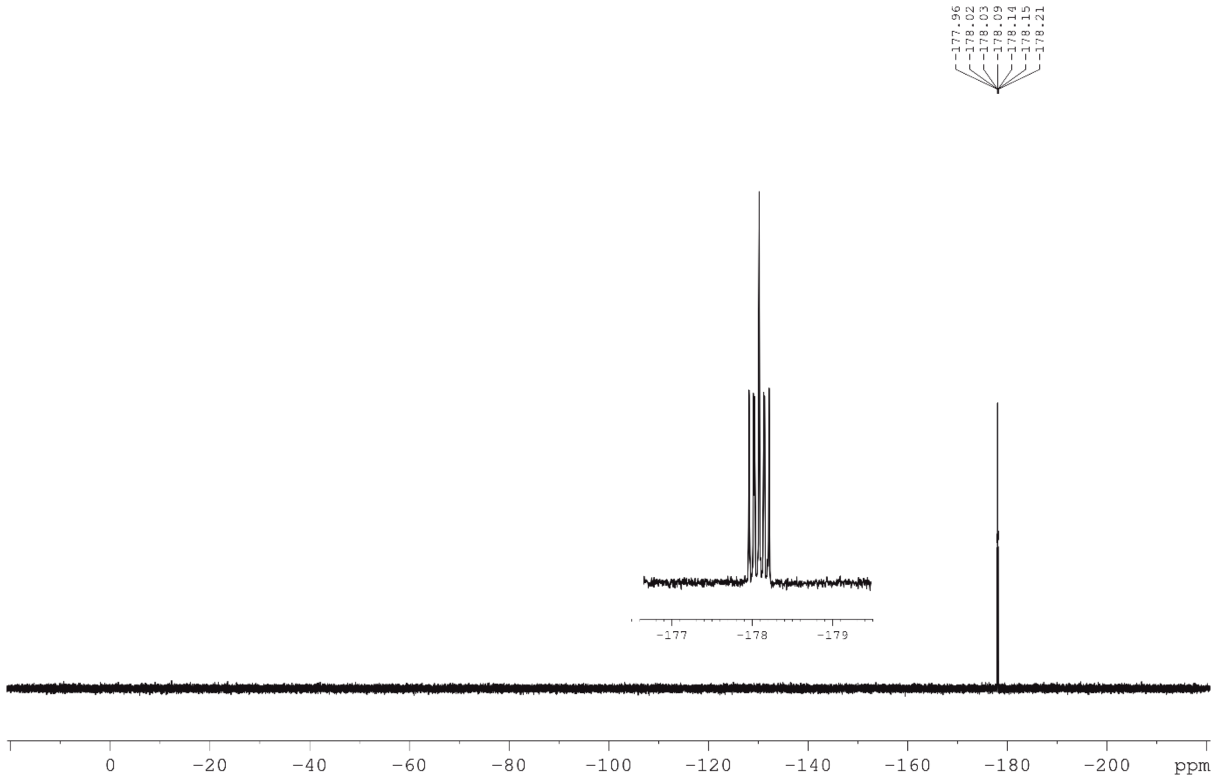

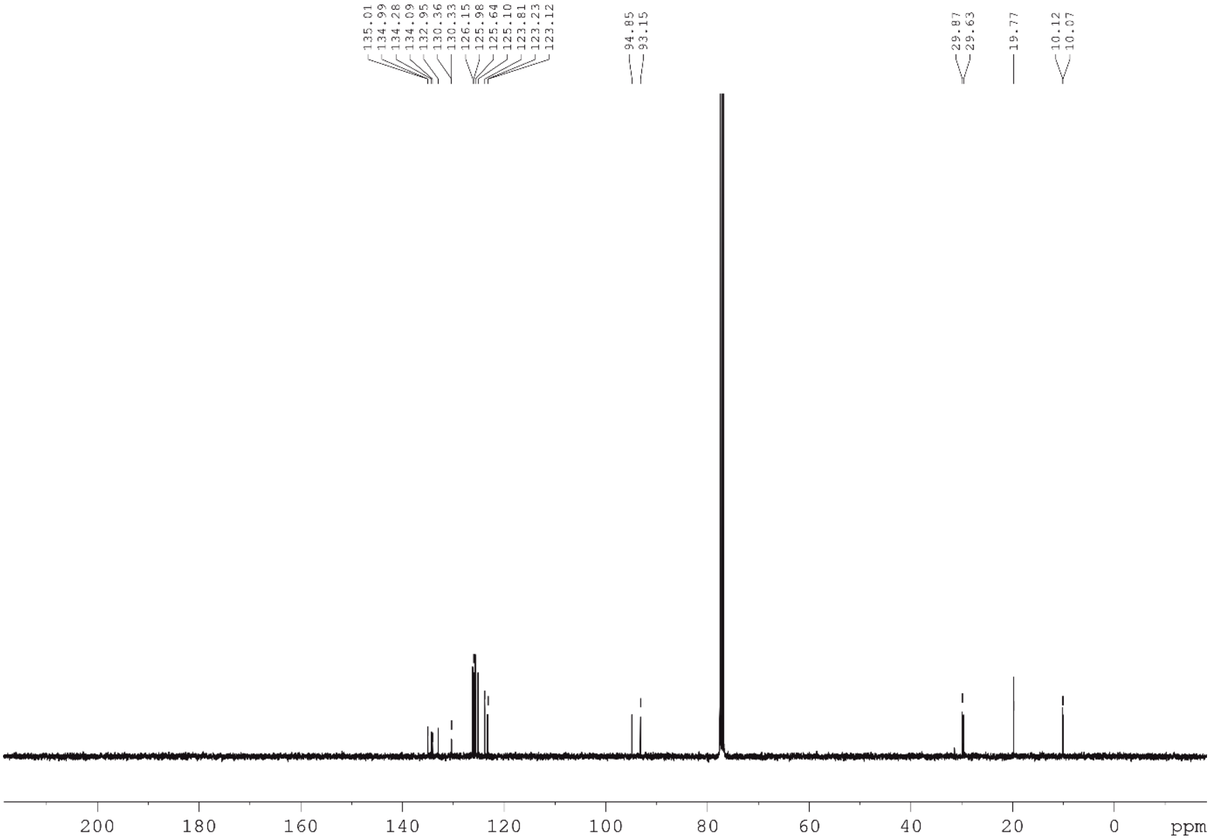


## (*R*)-1-fluoro-4-(1-fluoropropyl)naphthalene (2m)

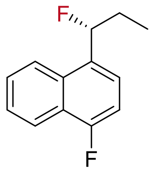

(*R*)-**2m**

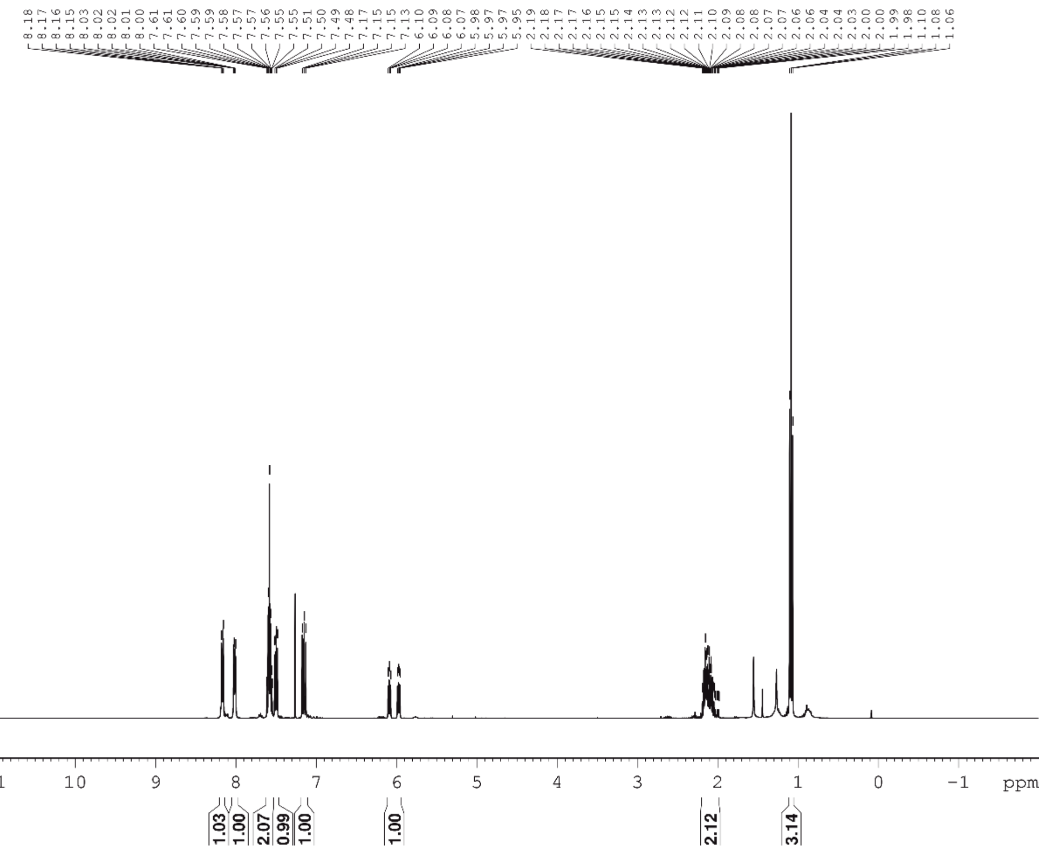

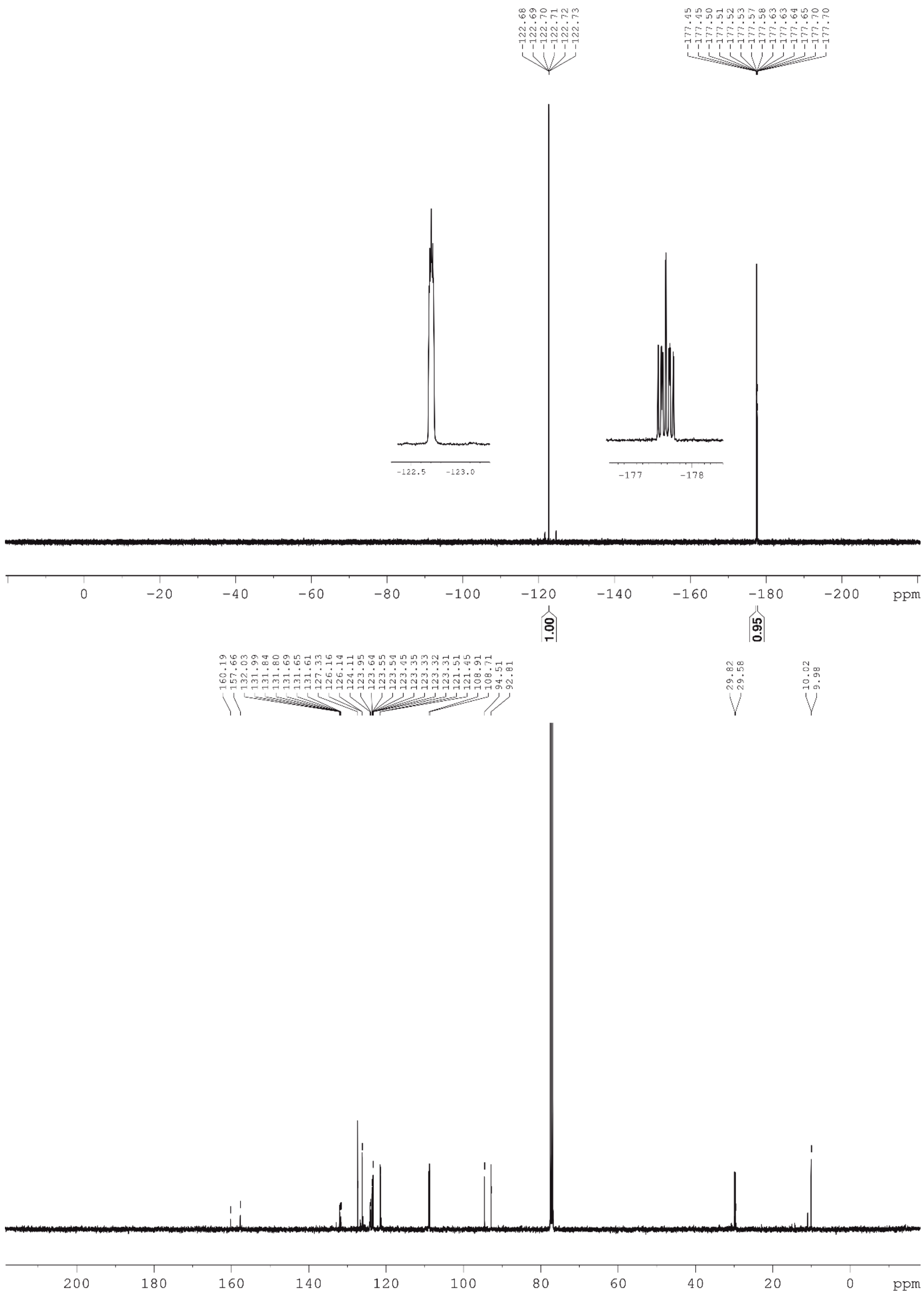
-122.68
-122.69
-122.70
-122.71
-122.72
-122.73
-177.45
-177.45
-177.50
-177.51
-177.52
-177.53
-177.57
-177.58
-177.63
-177.63
-177.64
-177.65
-177.70
-177.70
-122.5
-123.0
-177
-178
0
-20
-40
-60
-80
-100
-120
-140
-160
-180
-200
ppm
1.00
0.95
160.19
157.66
132.03
131.99
131.84
131.80
131.69
131.65
131.61
127.33
126.16
126.14
124.11
123.95
123.64
123.55
123.54
123.45
123.35
123.33
123.32
123.31
121.51
121.45
108.91
108.71
94.51
92.81
29.82
29.58
10.02
9.98
200
180
160
140
120
100
80
60
40
20
0
ppm

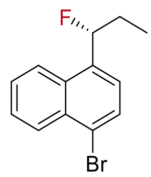

(*R*)-**2n**

**(*R*)- 1-bromo-4-(1-fluoropropyl)naphthalene (2n)**

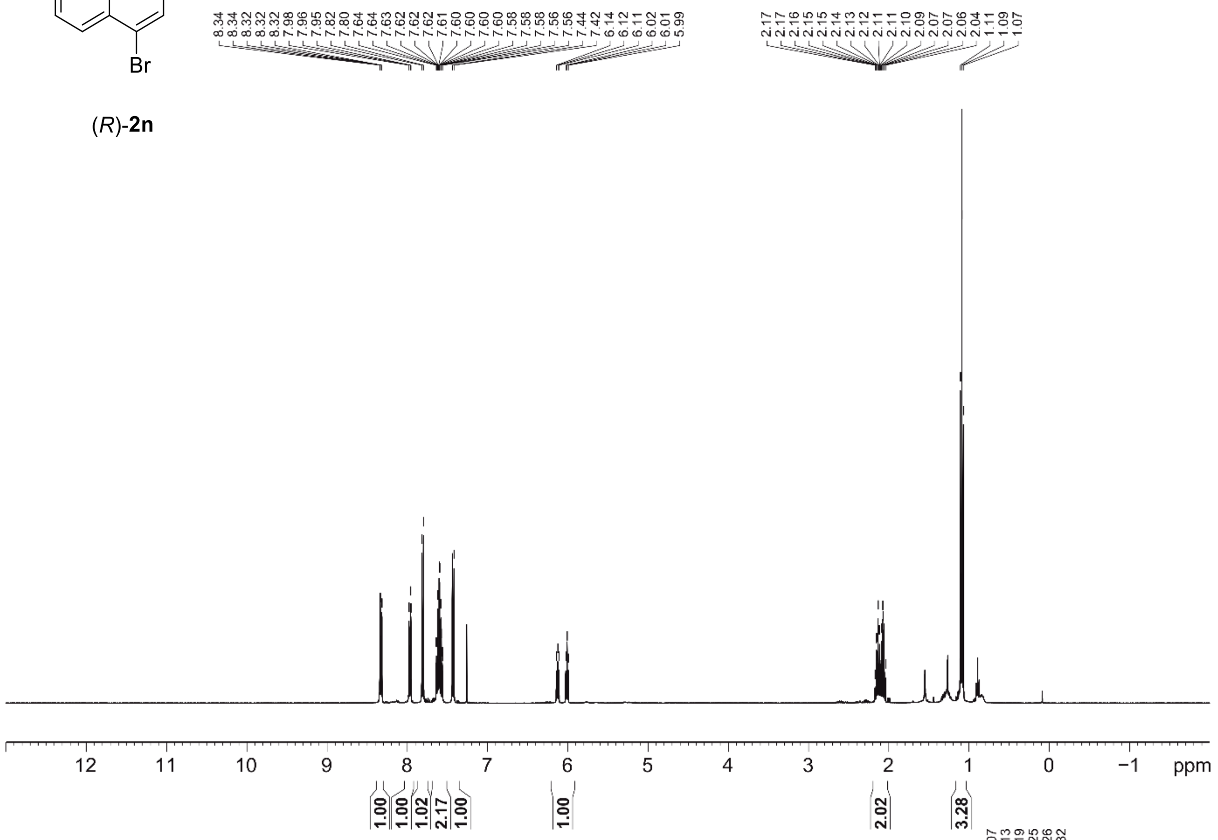

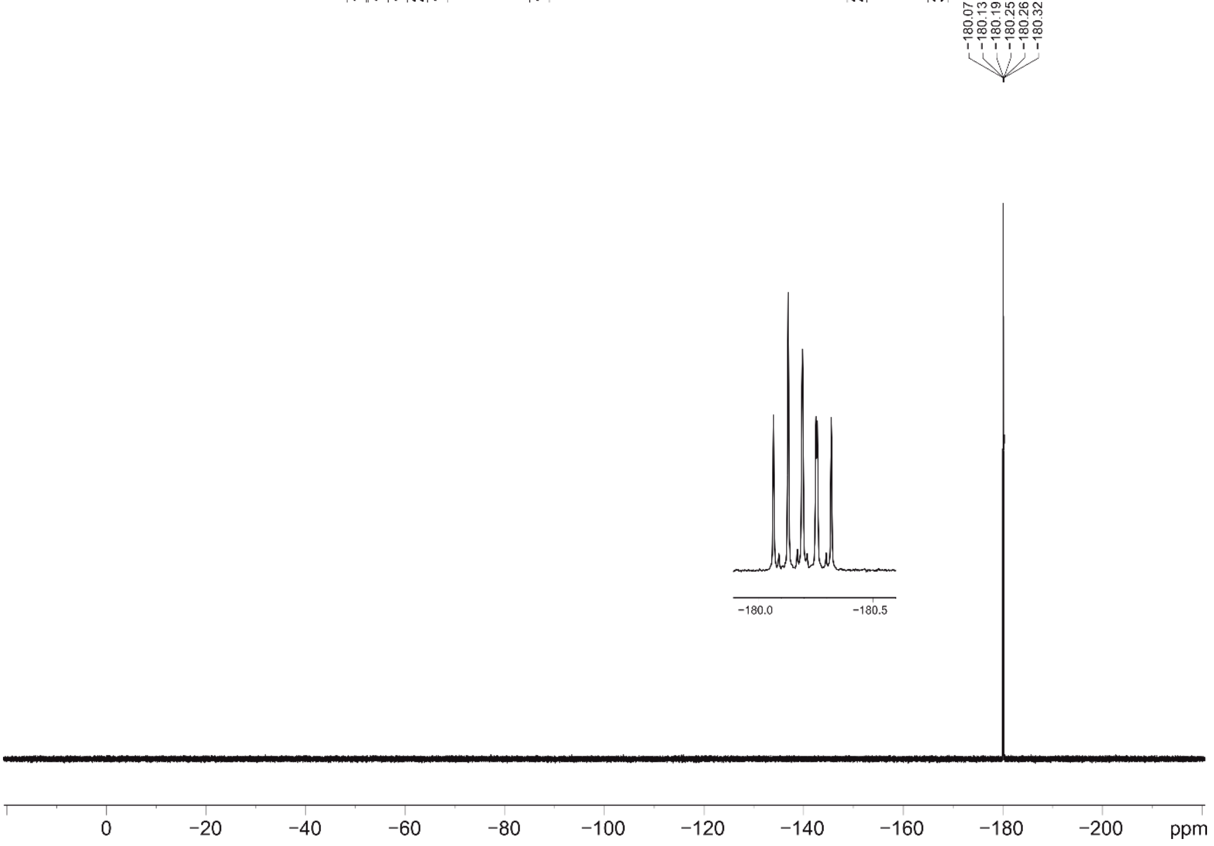

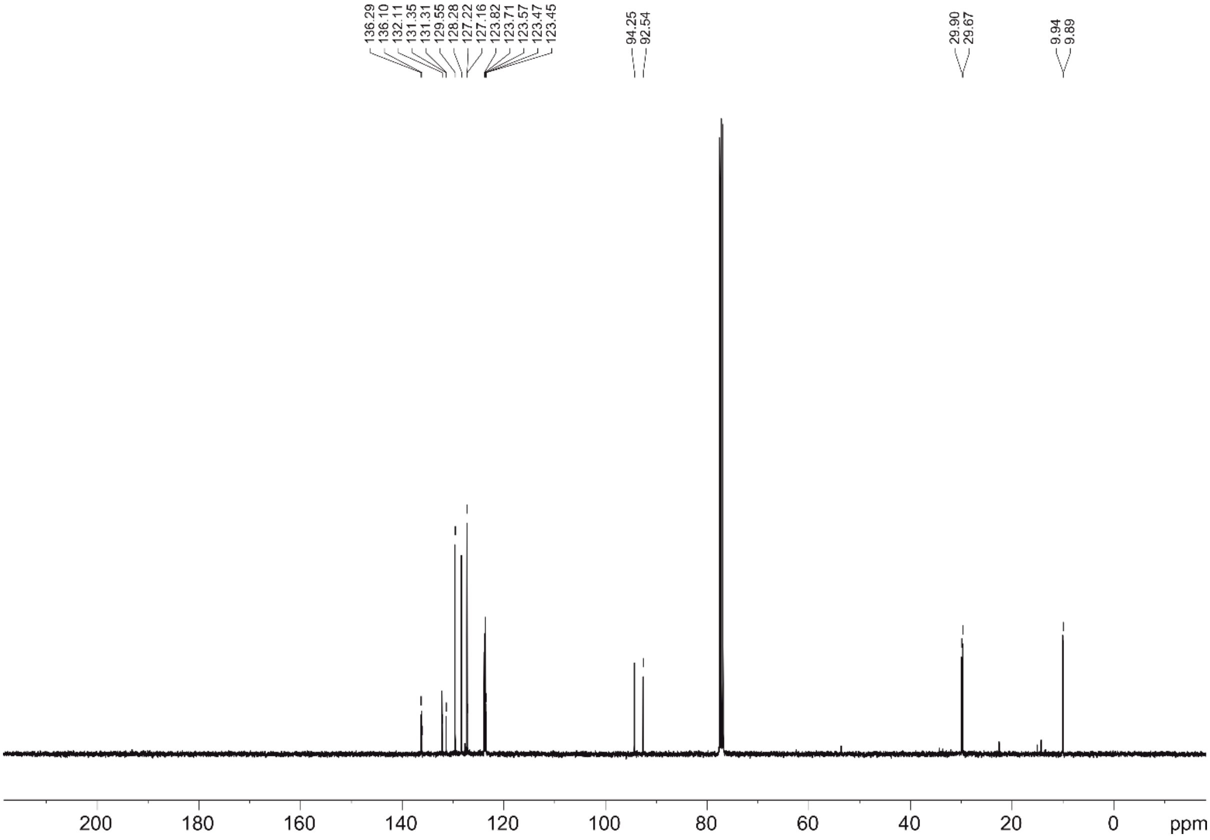


## (*R*)-1-(1-fluorobutyl)naphthalene (2p)

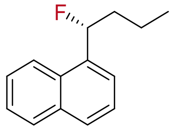

(*R*)-**2p**

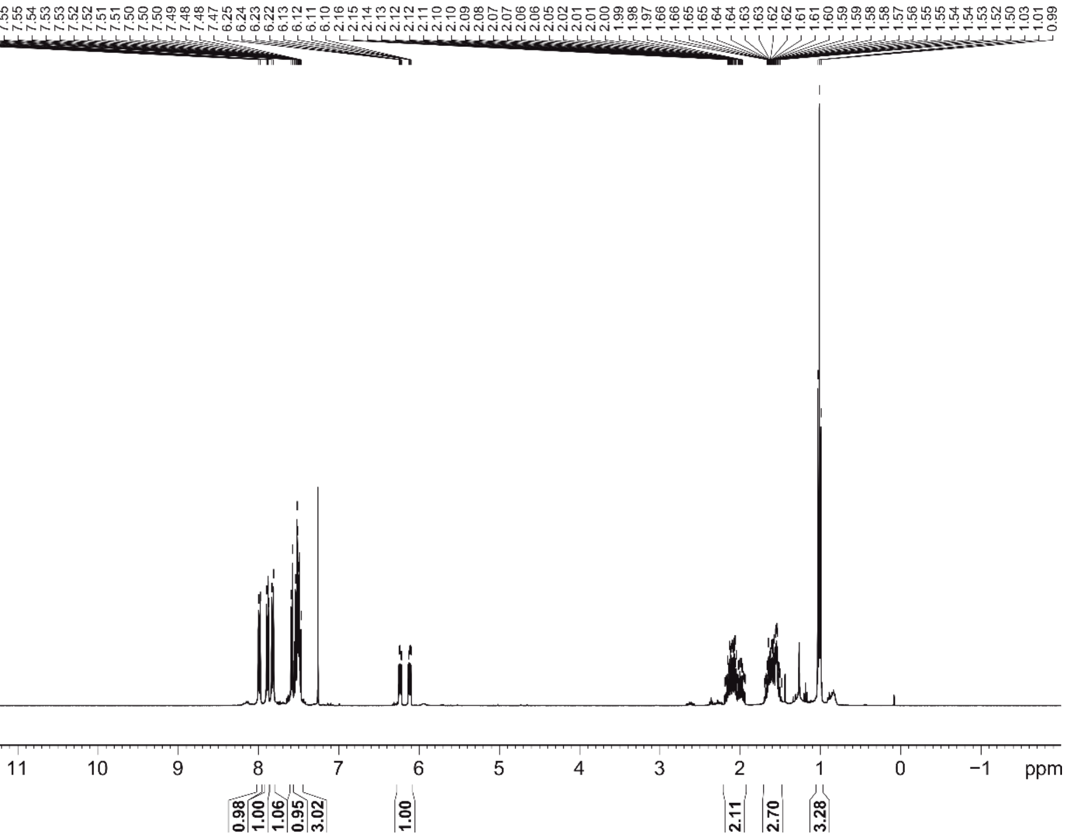

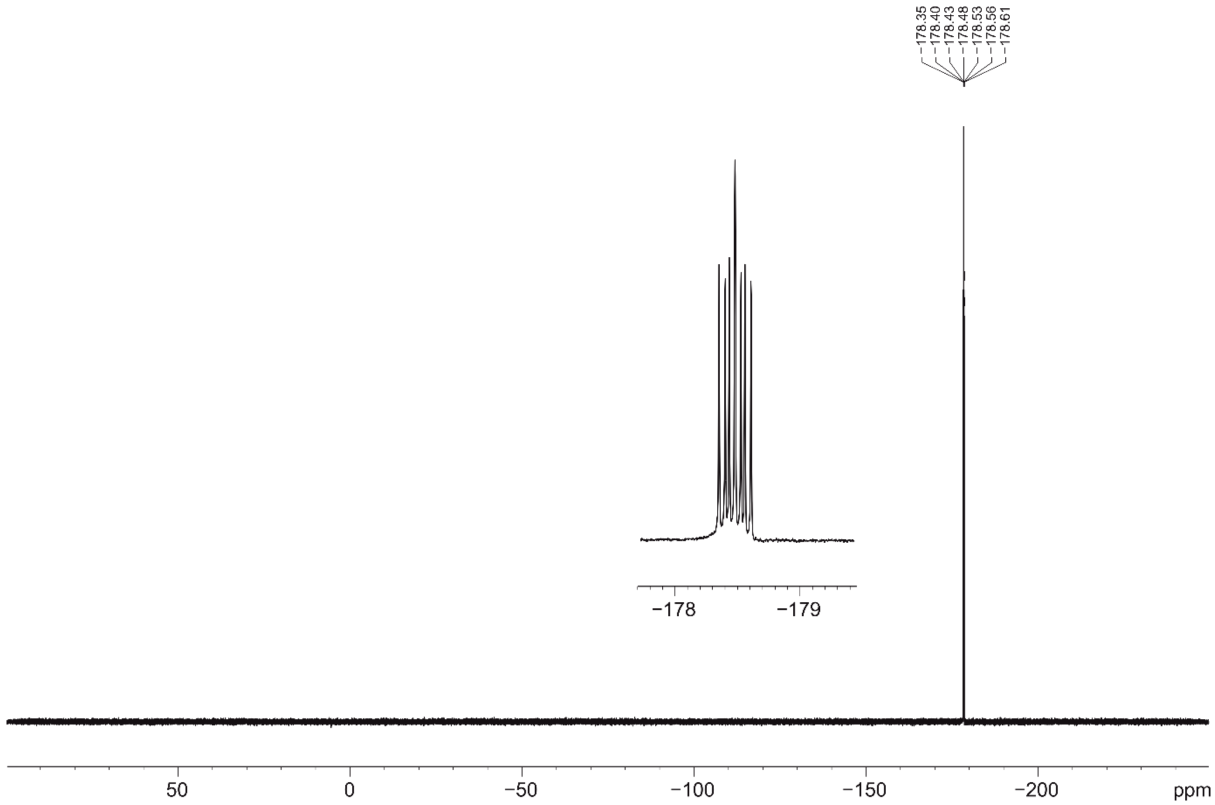
-178.35
-178.40
-178.43
-178.48
-178.53
-178.56
-178.61
−178
−179
50
0
−50
−100
−150
−200
ppm

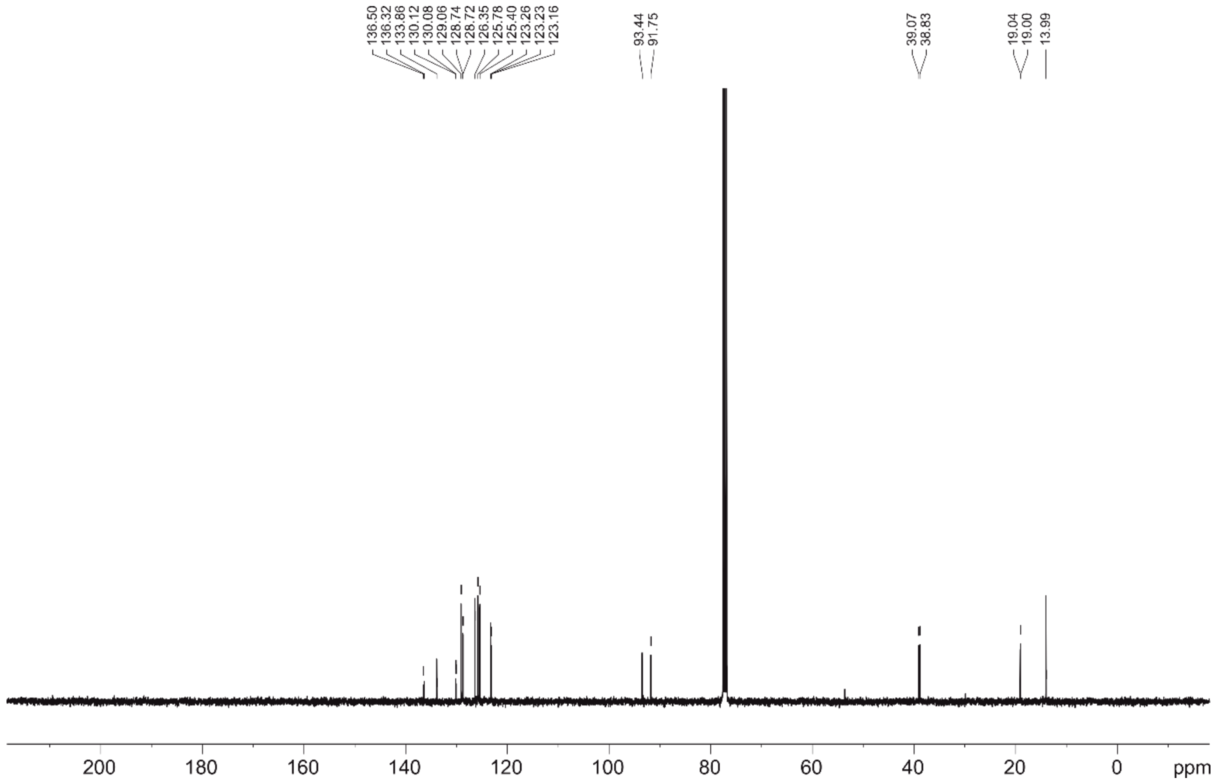
136.50
136.32
133.86
130.12
130.08
129.06
128.74
128.72
126.35
125.78
125.40
123.26
123.23
123.16
93.44
91.75
39.07
38.83
19.04
19.00
13.99
200
180
160
140
120
100
80
60
40
20
0
ppm

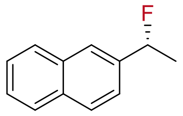


(*R*)-**2q**

**(*R*)- 2-(1-fluoroethyl)naphthalene (2q)**

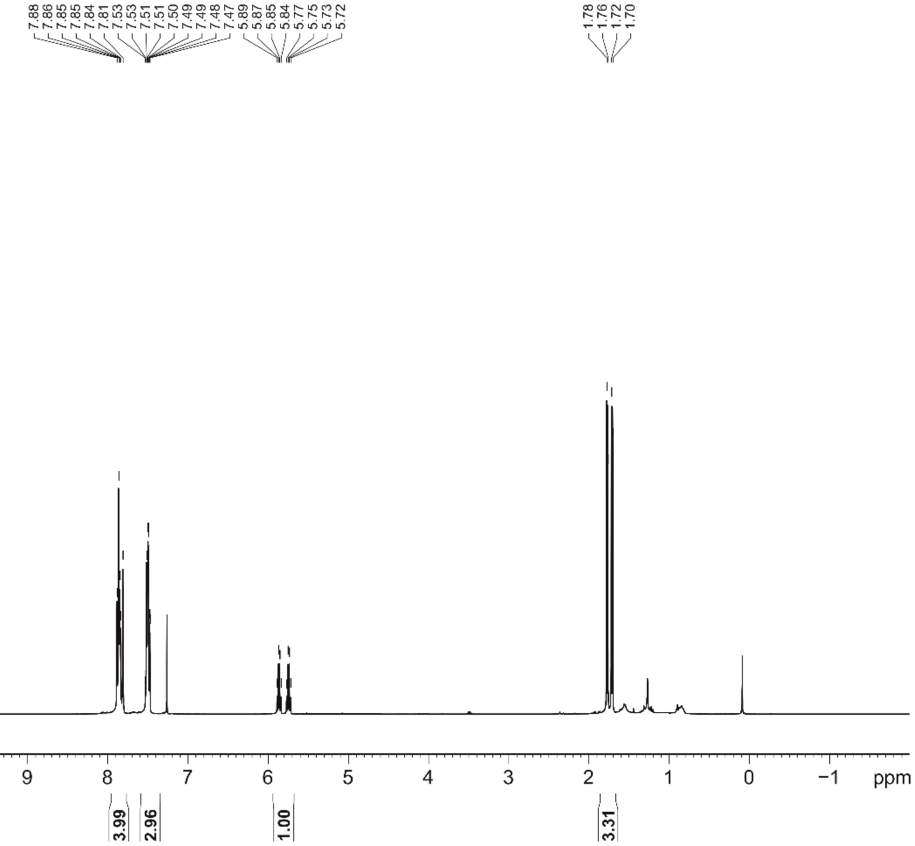


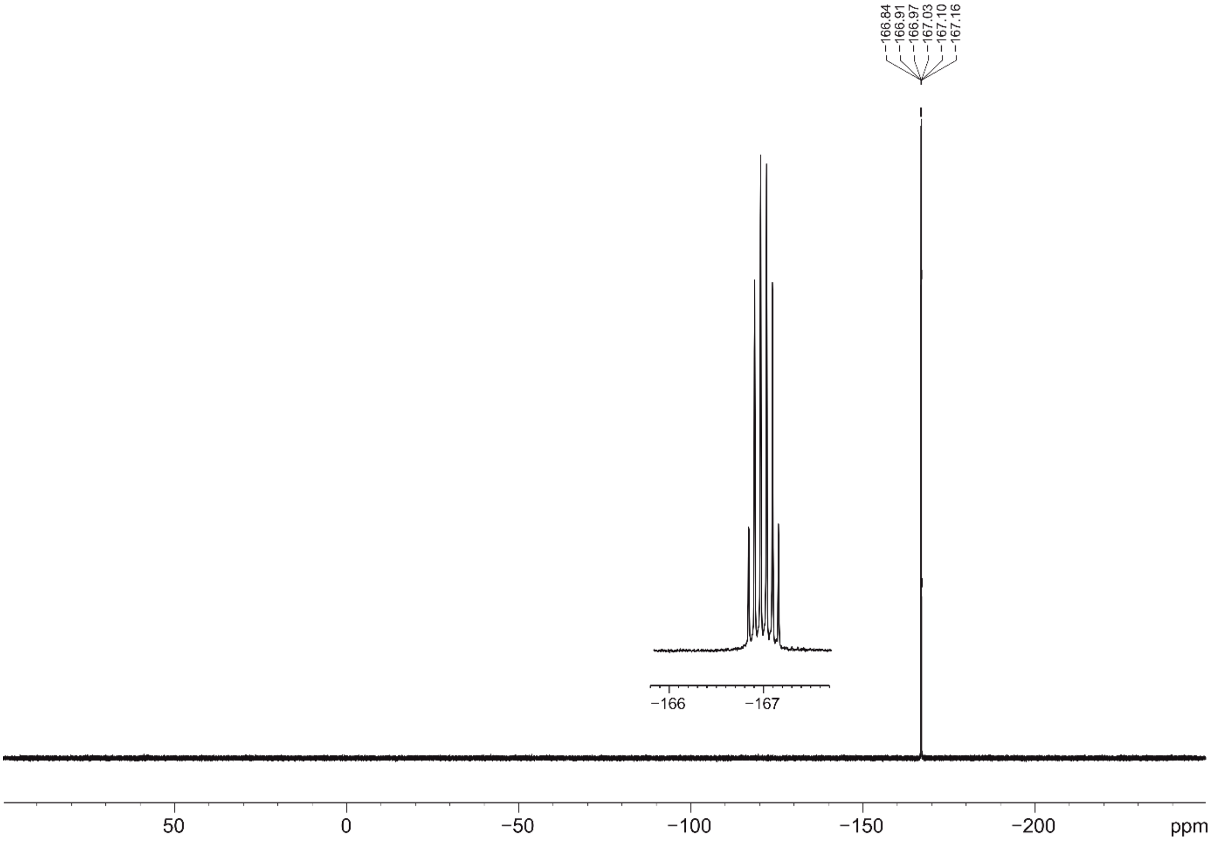

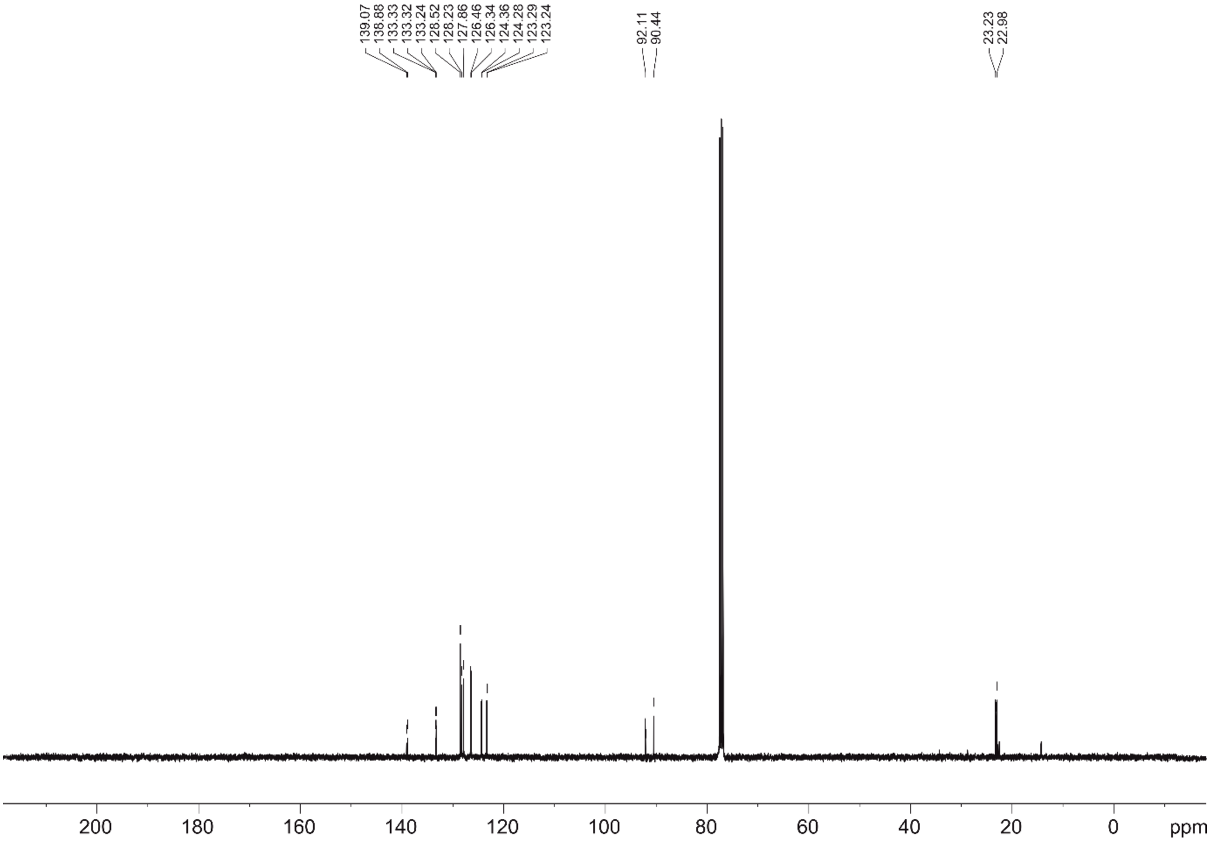


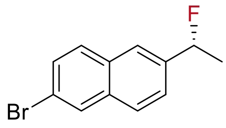


(R)-2r

## (*R*)-2-bromo-6-(1-fluoroethyl)naphthalene (2r)

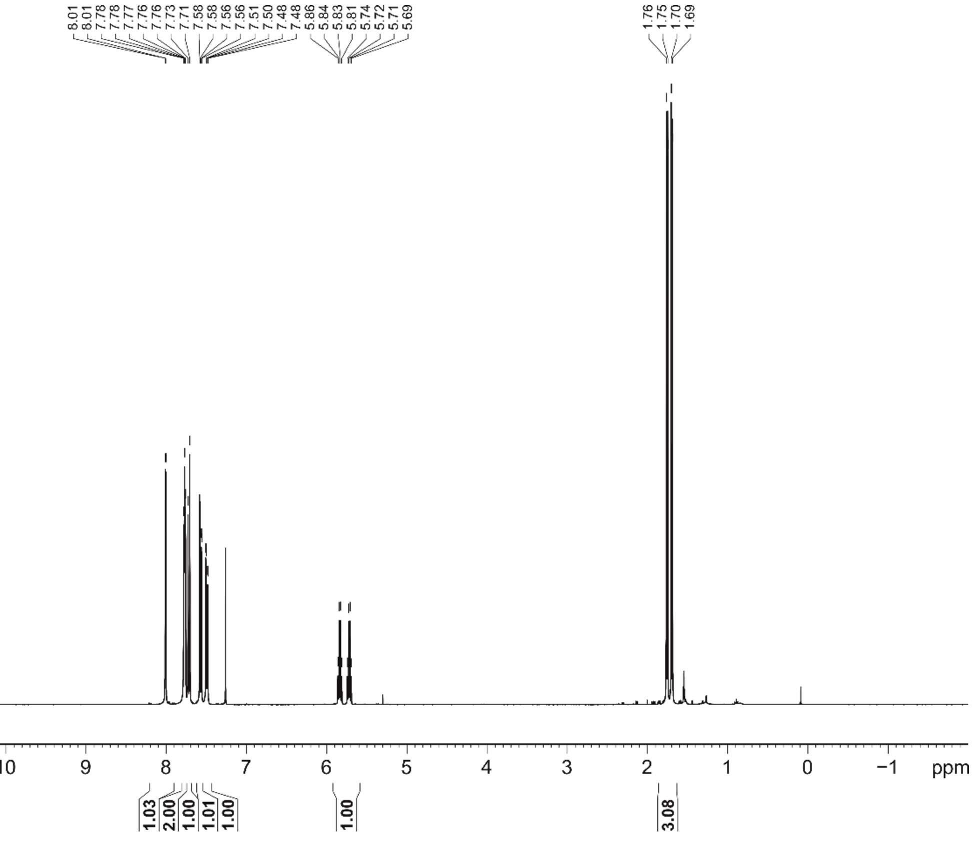

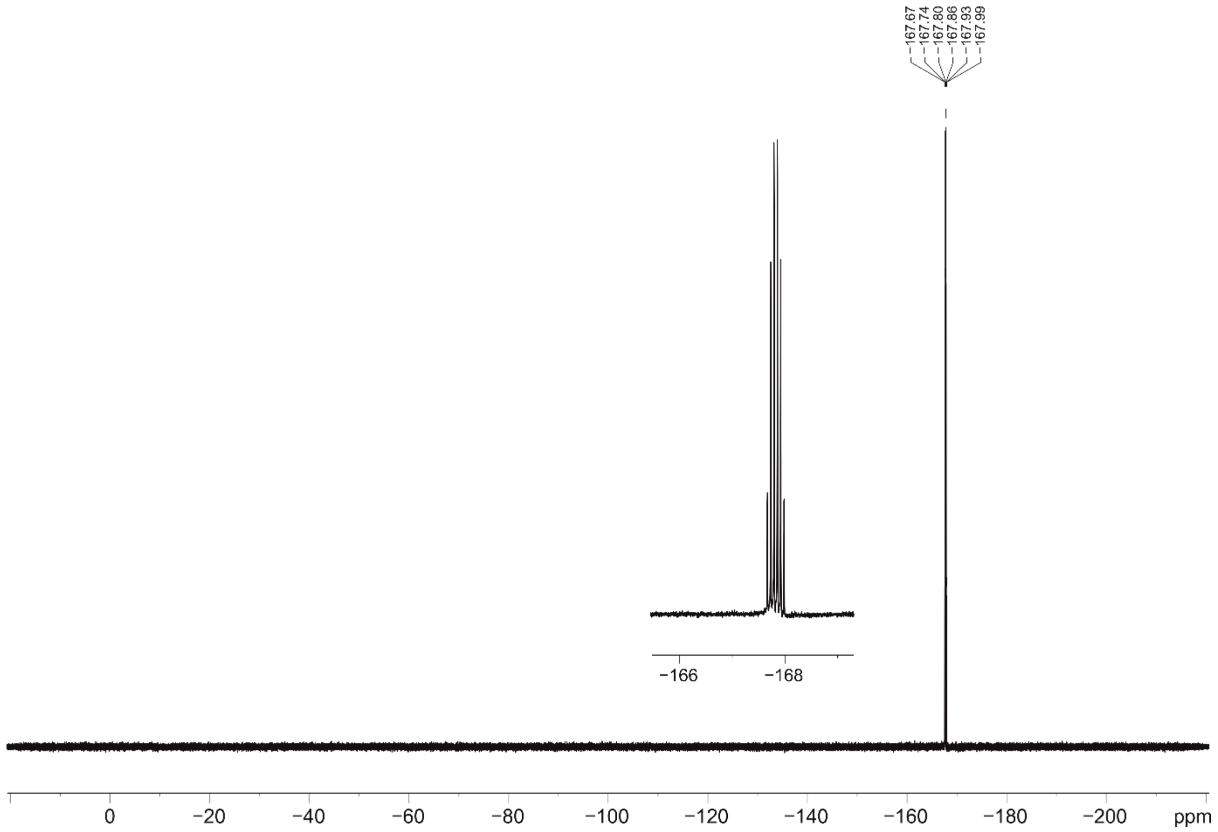
−167.67
−167.74
−167.80
−167.86
−167.93
−167.99
−166
−168
0
−20
−40
−60
−80
−100
−120
−140
−160
−180
−200
ppm

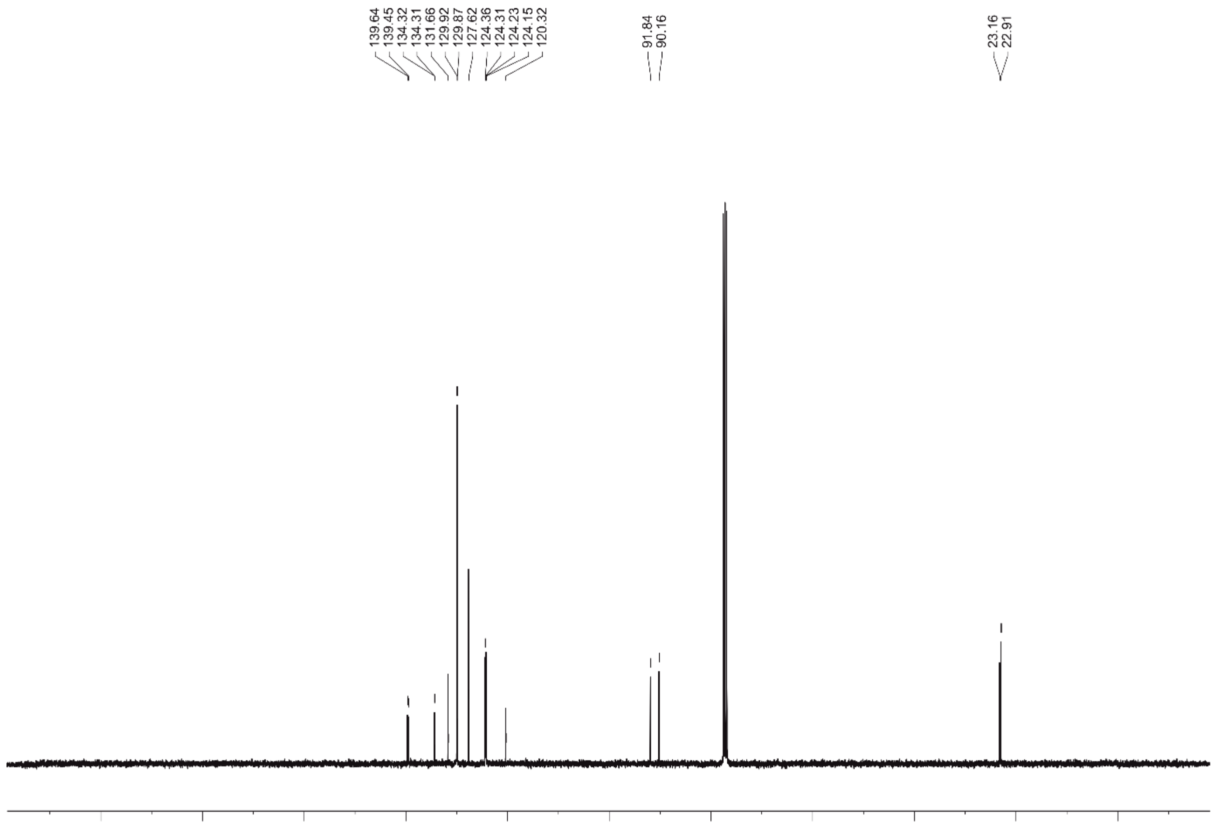
139.64
139.45
134.32
134.31
131.66
129.92
129.87
127.62
124.36
124.31
124.23
124.15
120.32
91.84
90.16
23.16
22.91
200
180
160
140
120
100
80
60
40
20
0
ppm

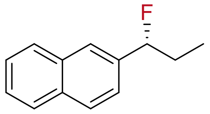

(*R*)-**2s**

## (*R*)-1-(2-fluoropropyl)naphthalene (2s)

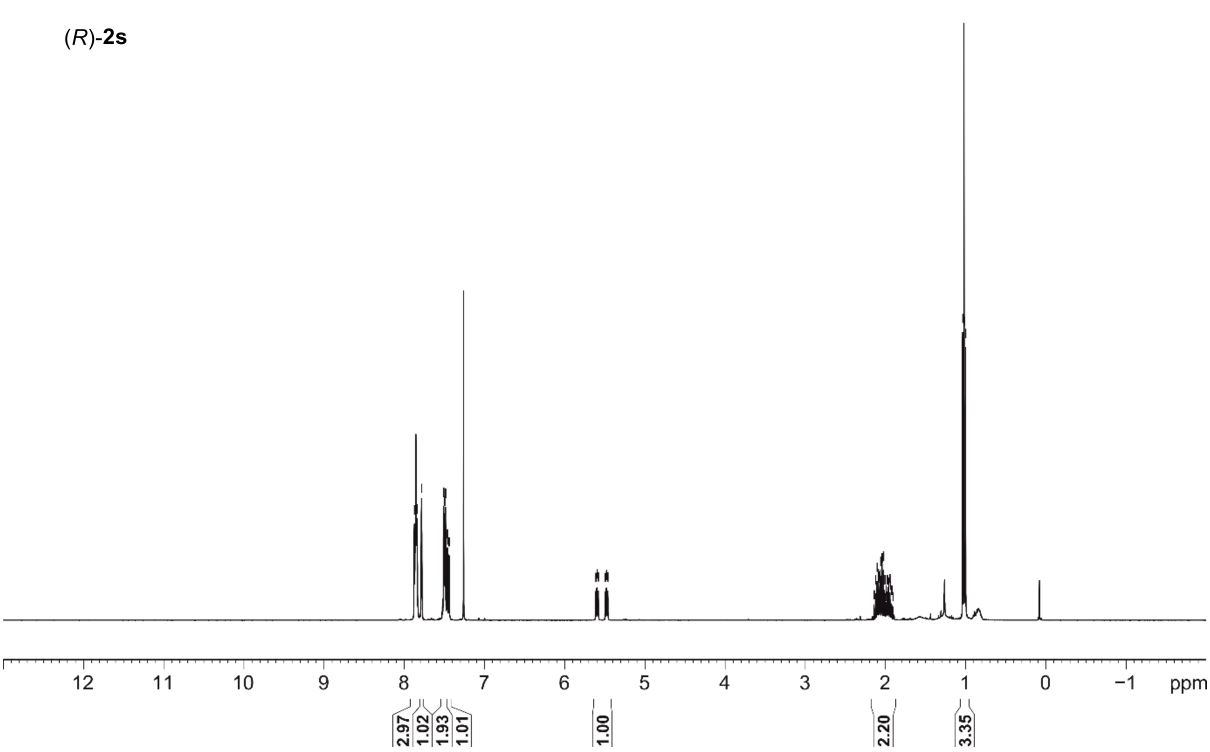

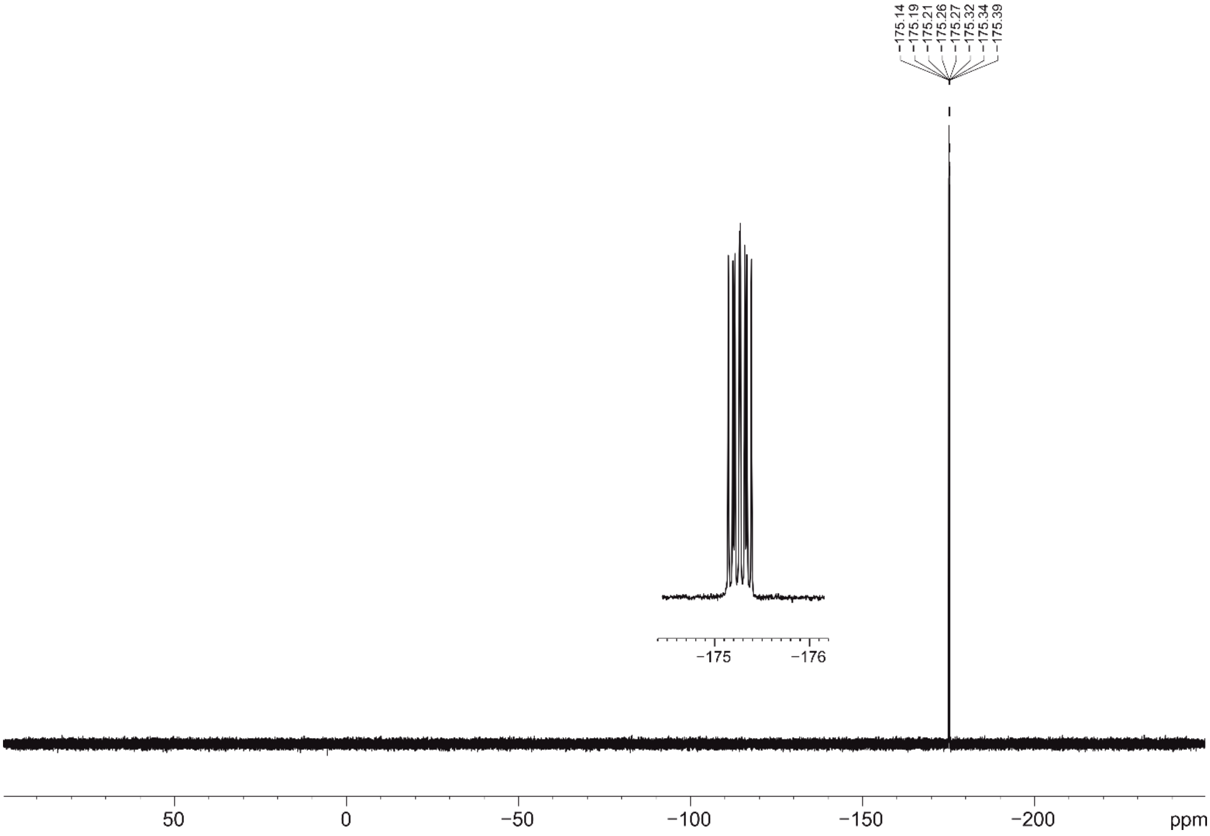

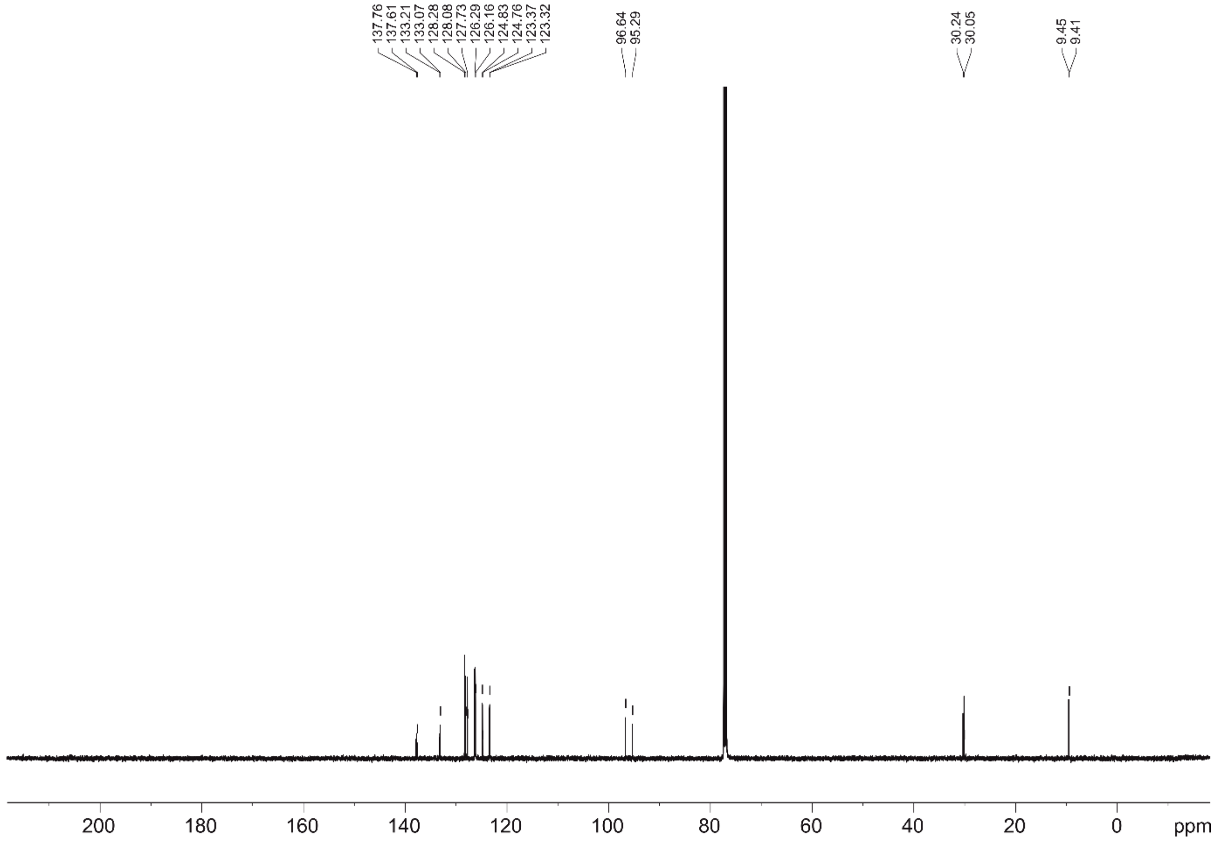


**(*R*)-2-(4-(1-fluoroethyl)phenyl)-4,4,5,5-tetramethyl-1,3-dioxolane (2t)**

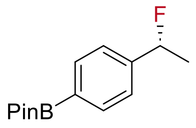


(*R*)-**2t**

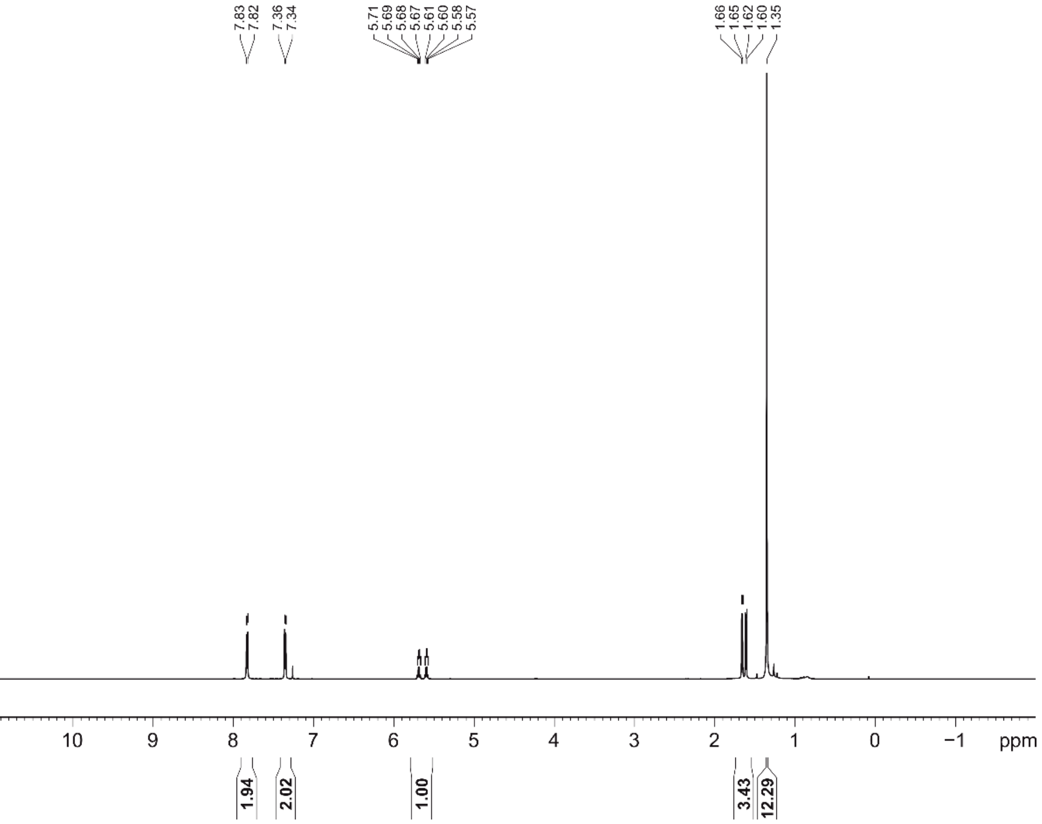

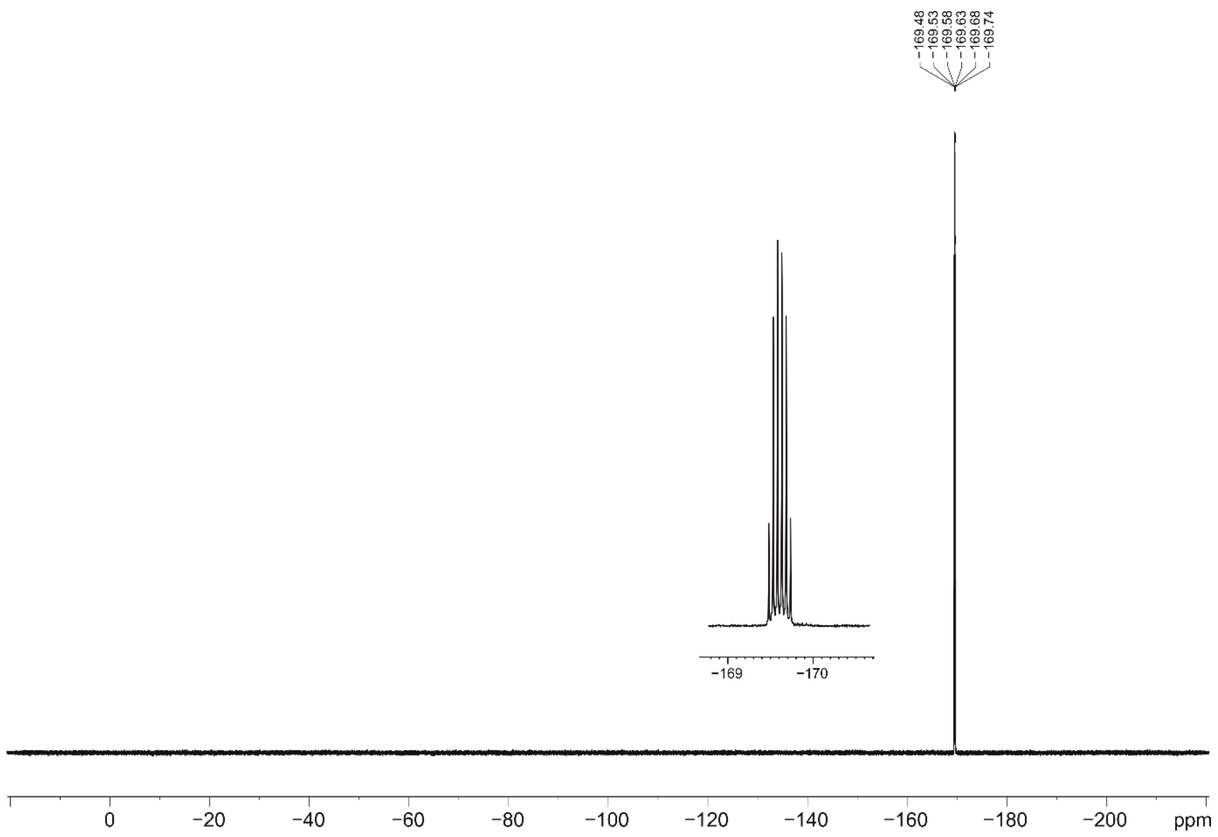
-169.48
-169.53
-169.58
-169.63
-169.68
-169.74
−169
−170
0
−20
−40
−60
−80
−100
−120
−140
−160
−180
−200
ppm

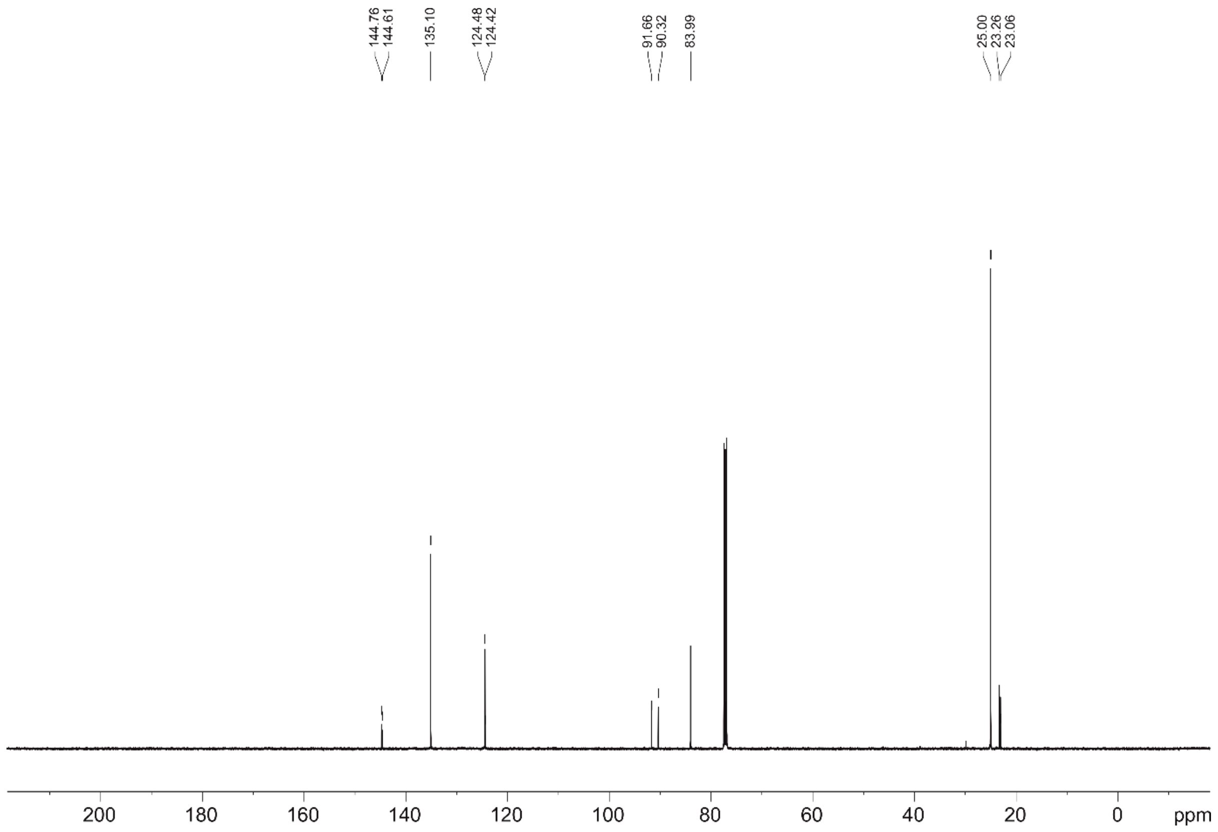
144.76
144.61
135.10
124.48
124.42
91.66
90.32
83.99
25.00
23.26
23.06
200
180
160
140
120
100
80
60
40
20
0
ppm

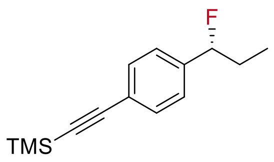


(*R*)-**2u**

**(*R*)-((4-(1-fluoropropyl)phenyl)ethynyl)trimethylsilane (2u)**

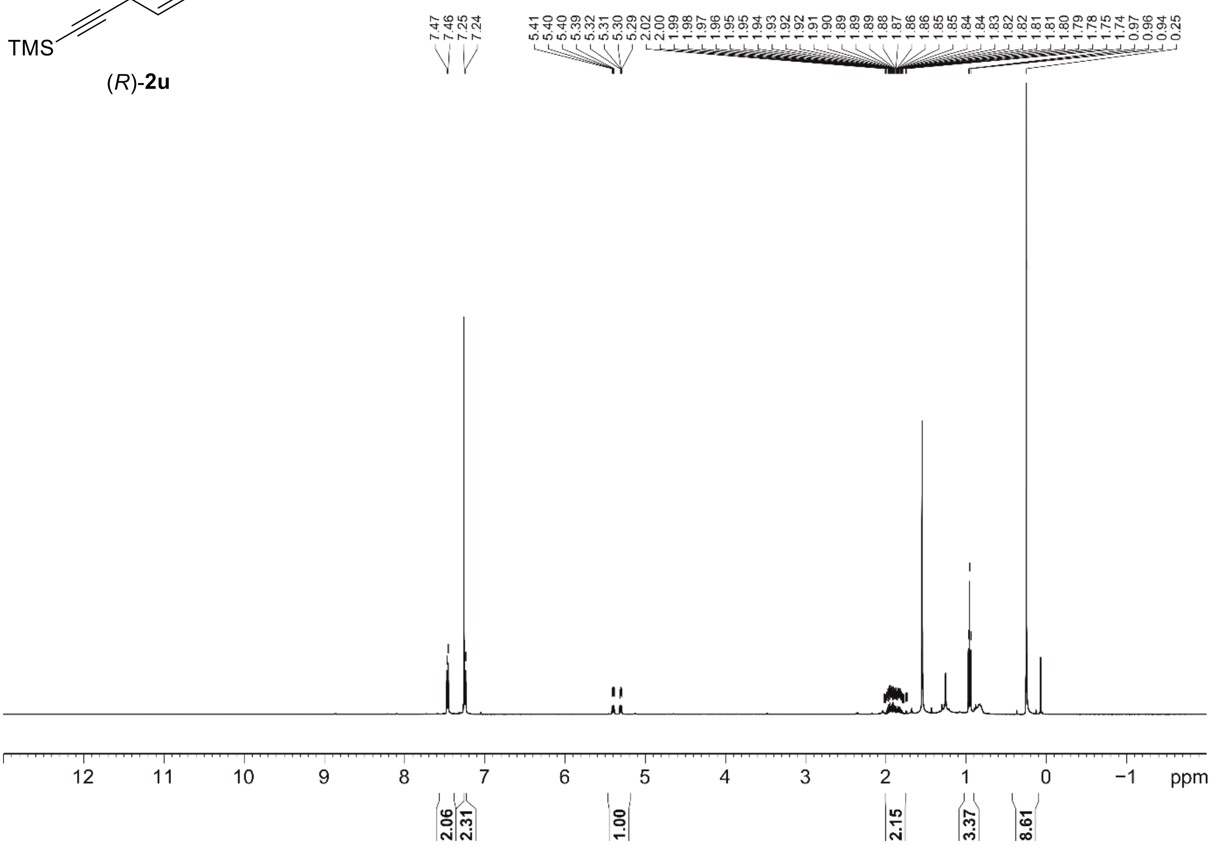


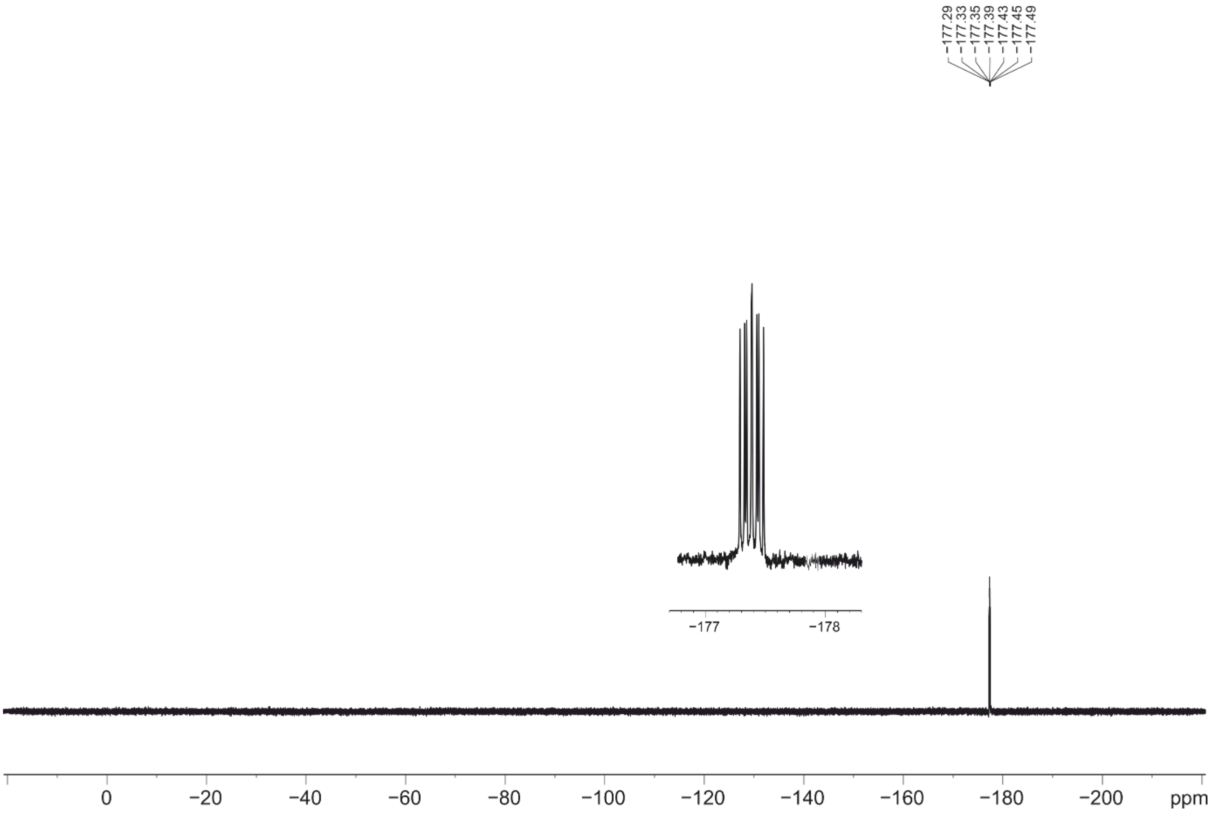

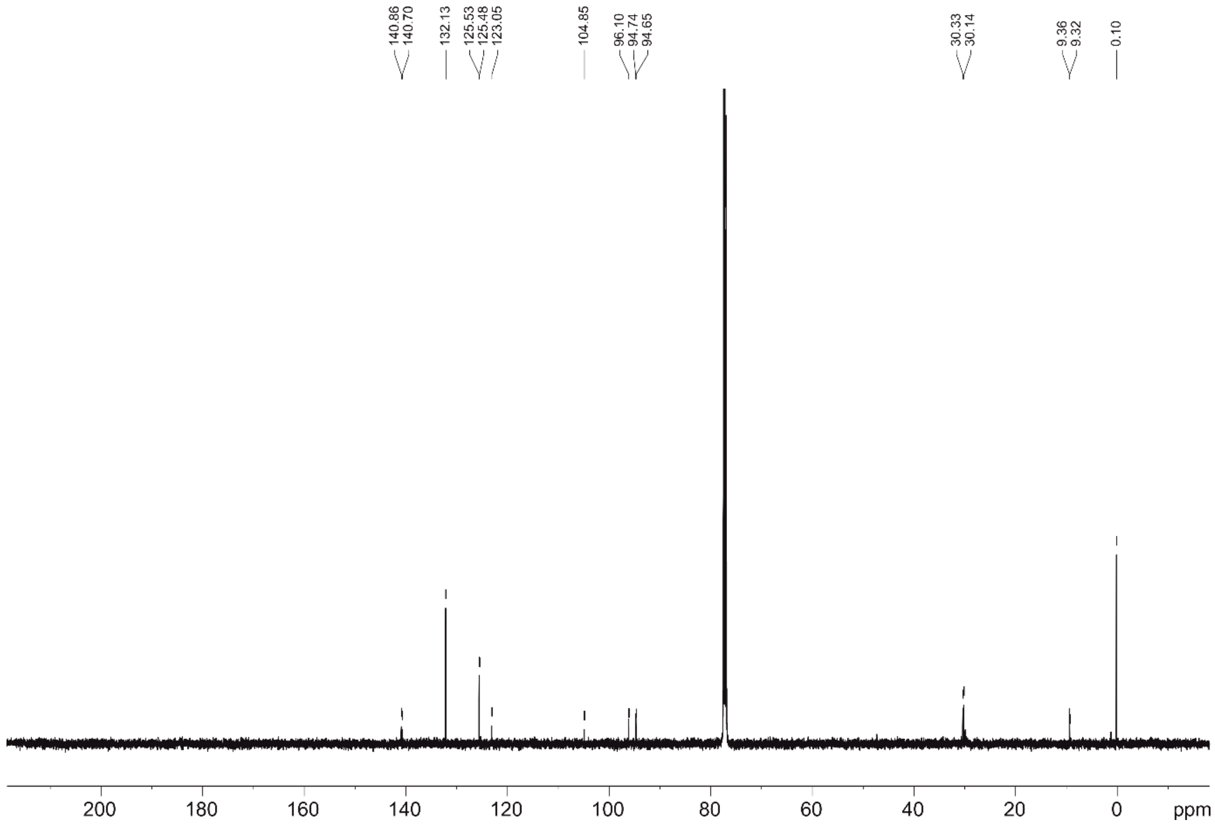


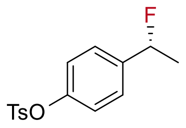


(*R*)-**2v**

**(*R*)-4-(1-fluoroethyl)phenyl-4-methylbenzenesulfonate (2v)**

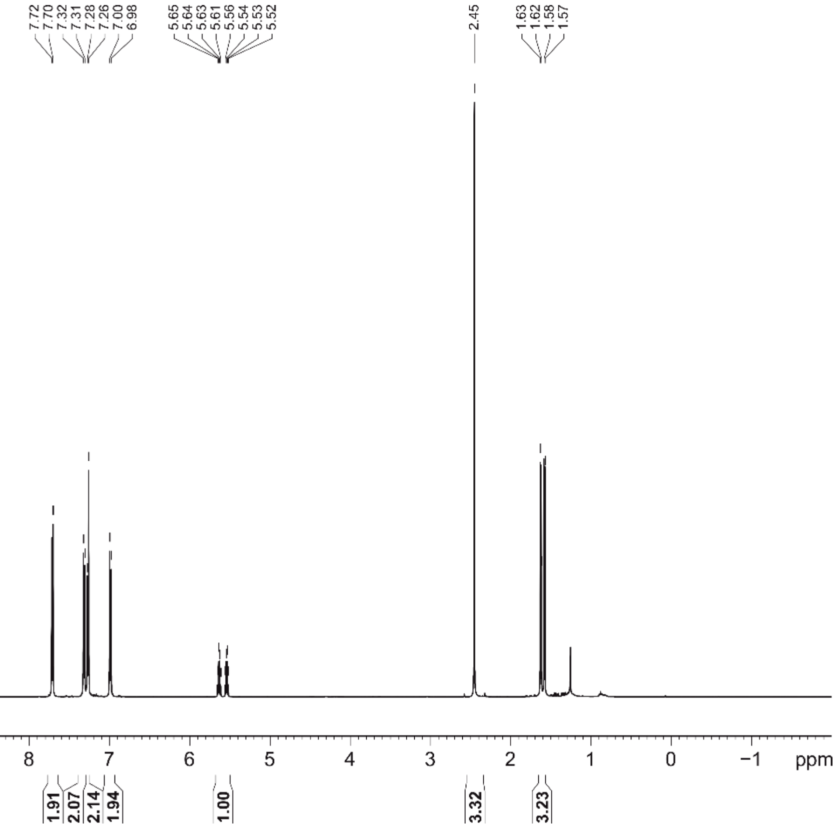

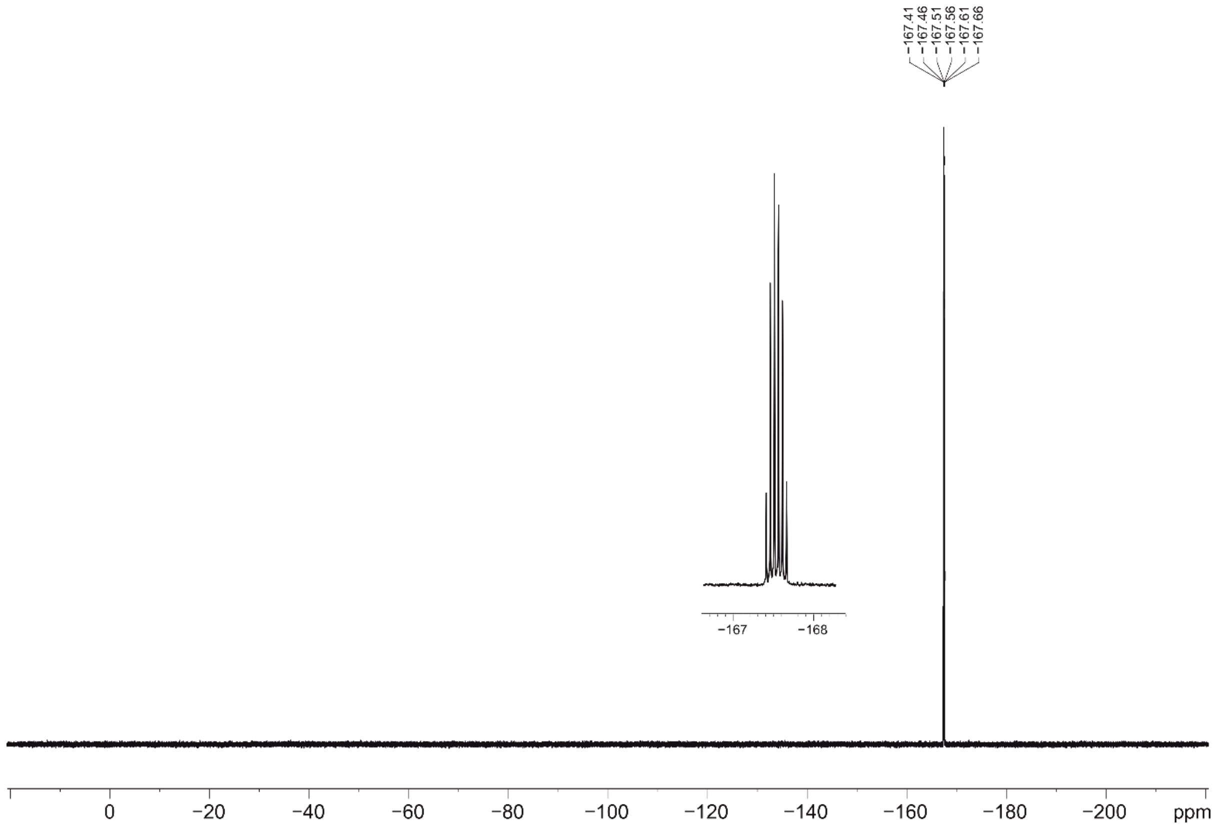
-167.41
-167.46
-167.51
-167.56
-167.61
-167.66
-167
-168
0
−20
−40
−60
−80
−100
−120
−140
−160
−180
−200
ppm

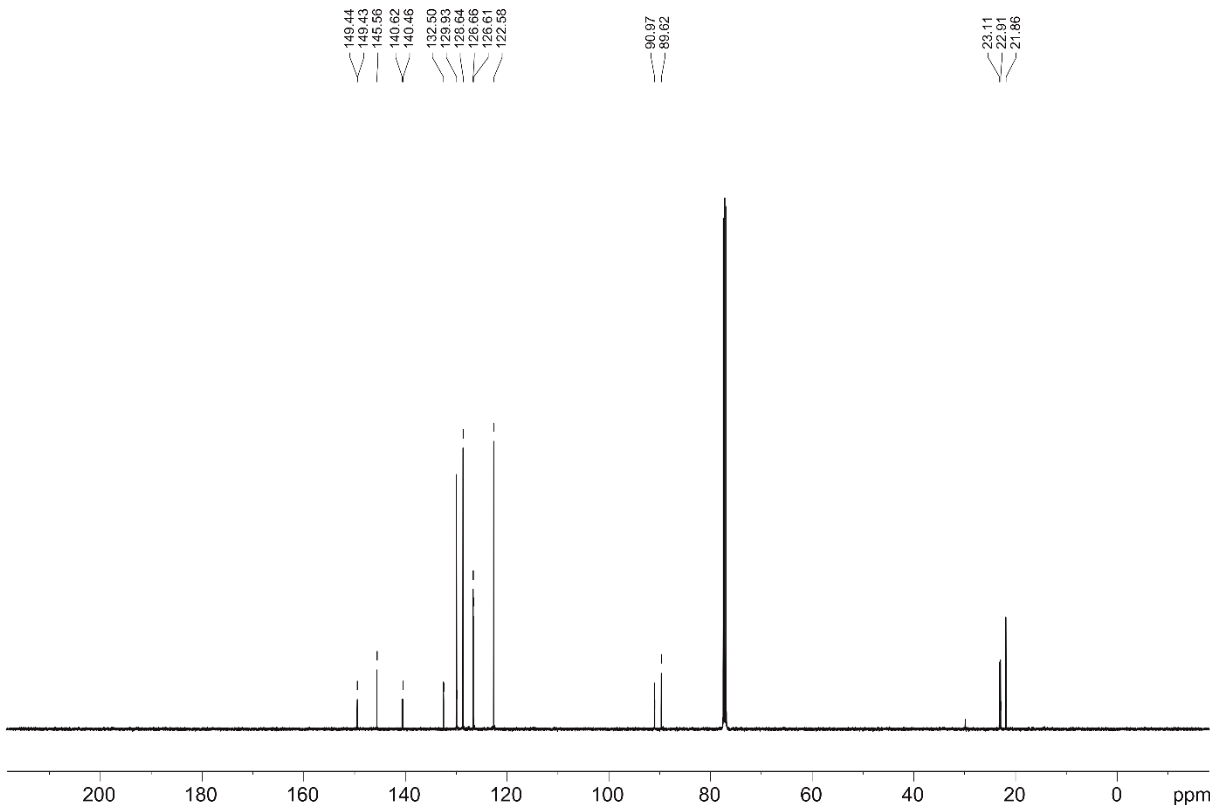
149.44
149.43
145.56
140.62
140.46
132.50
129.93
128.64
126.66
126.61
122.58
90.97
89.62
23.11
22.91
21.86
200
180
160
140
120
100
80
60
40
20
0
ppm

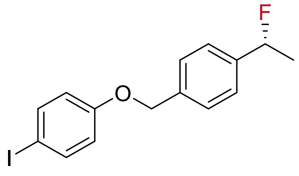
(*R*)-**2w**

**(*R*)-1-(1-fluoroethyl)-4-((4-iodophenoxy)methyl)benzene (2w)**

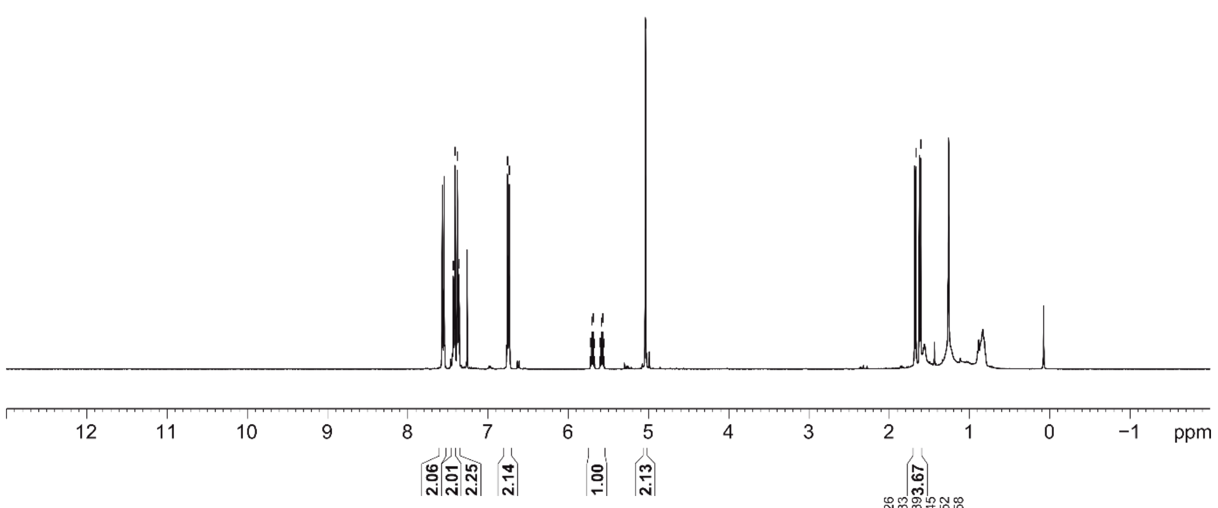


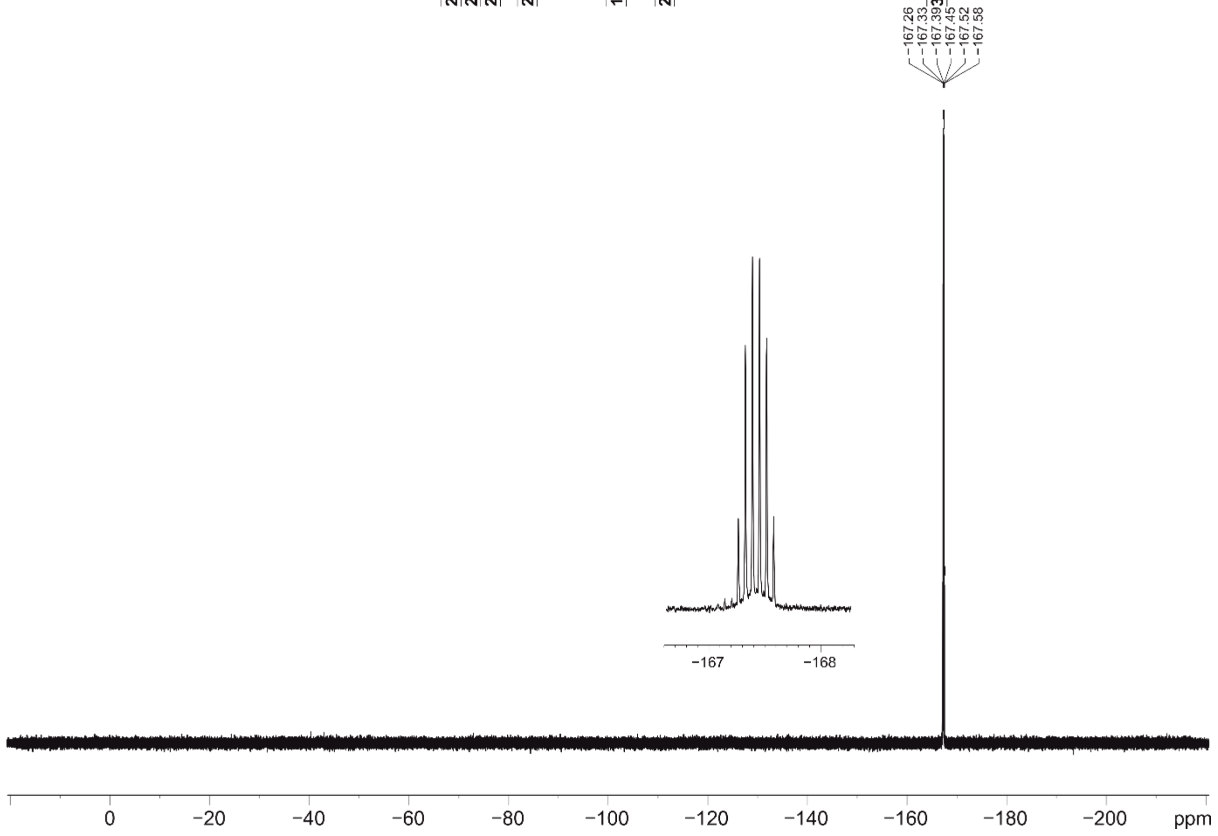

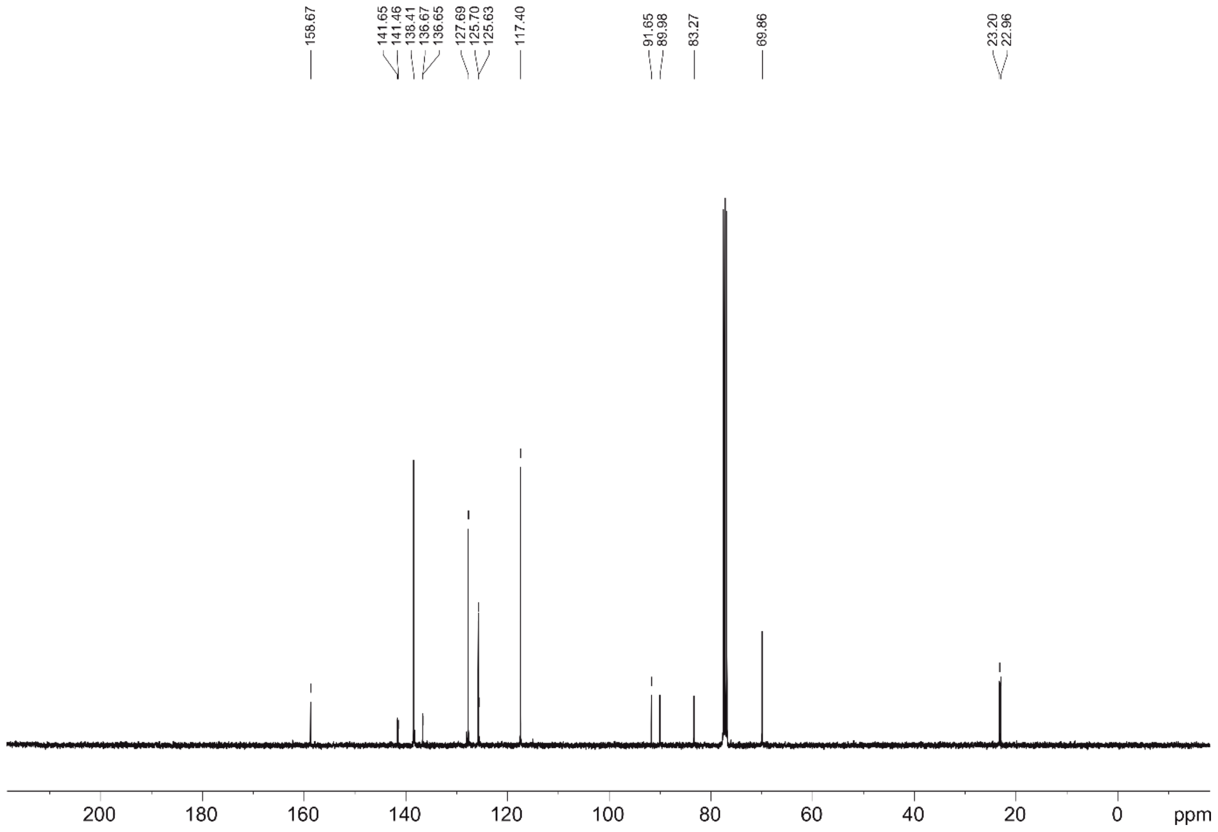


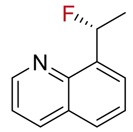


**(*R*)-8-(1-fluoroethyl)quinoline (2x)**

(*R*)-**2x**

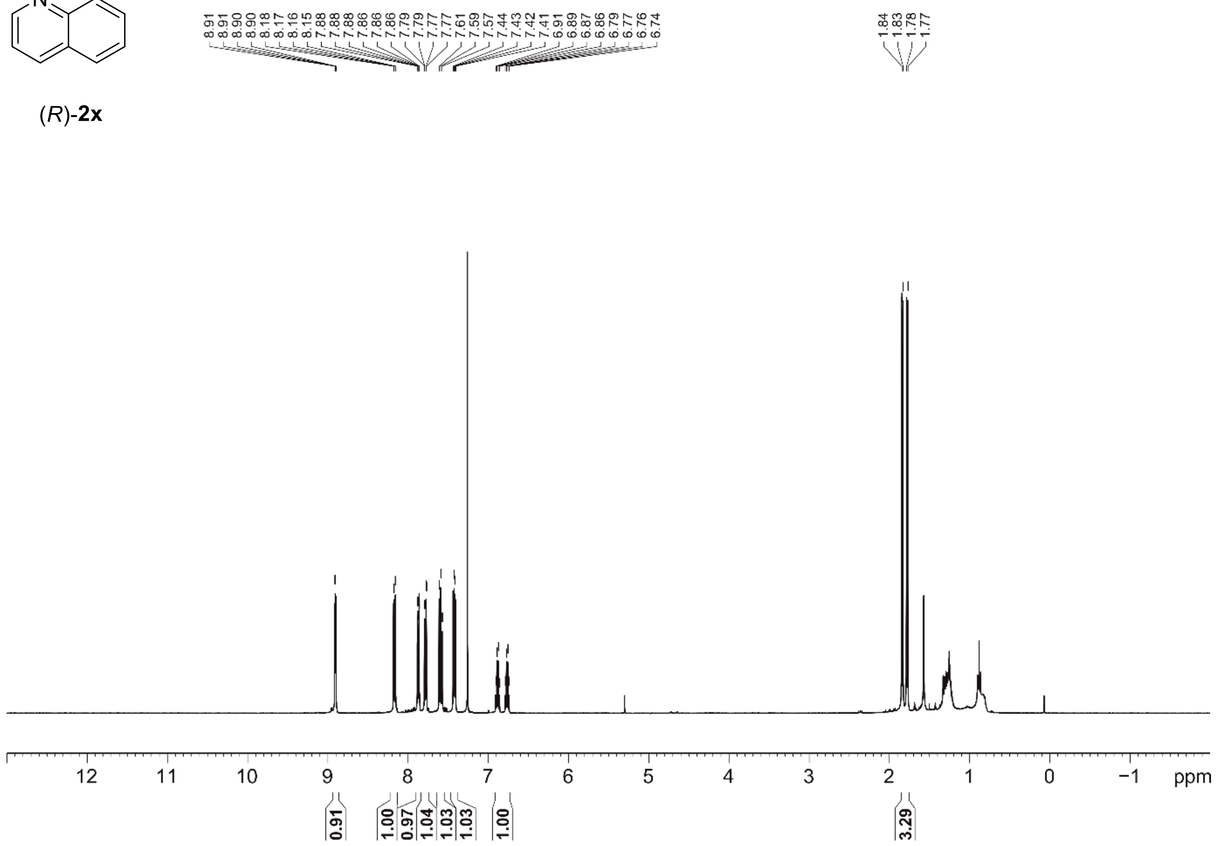

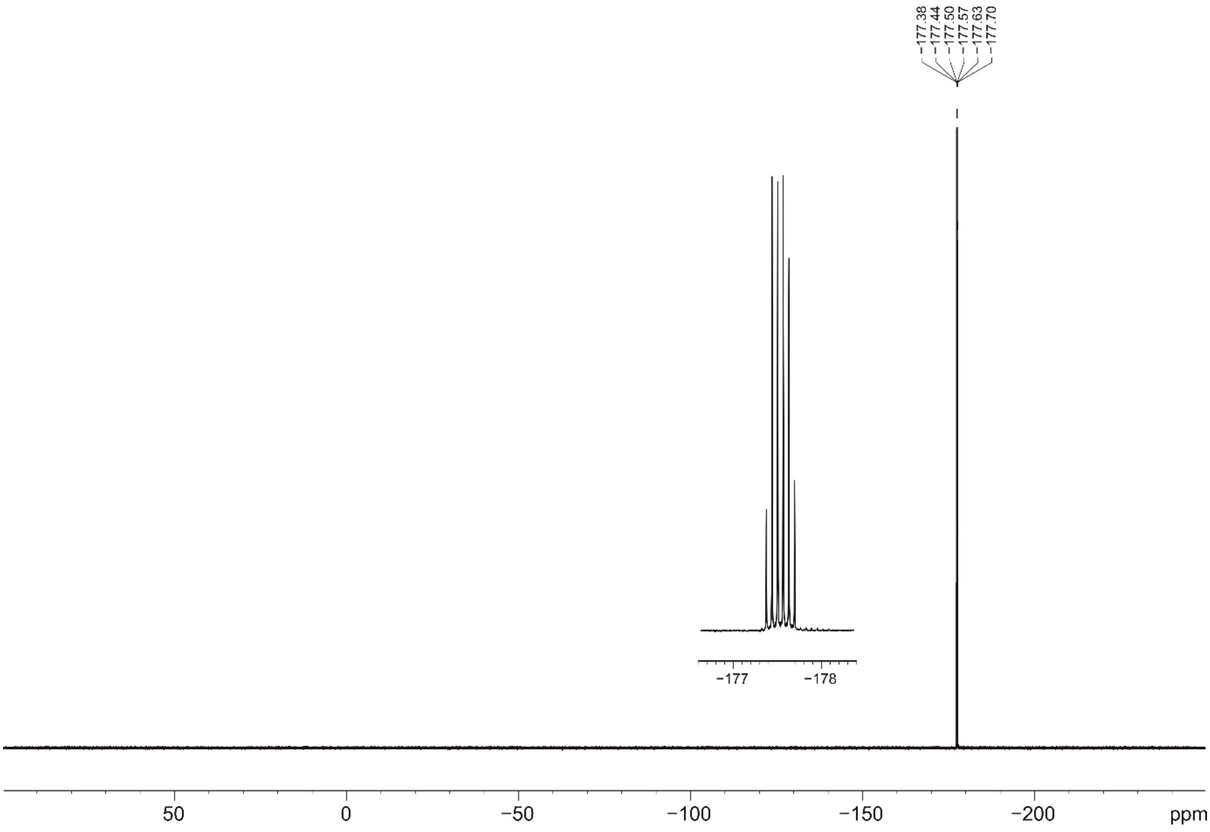
-177.38
-177.44
-177.50
-177.57
-177.63
-177.70
−177
−178
50
0
−50
−100
−150
−200
ppm

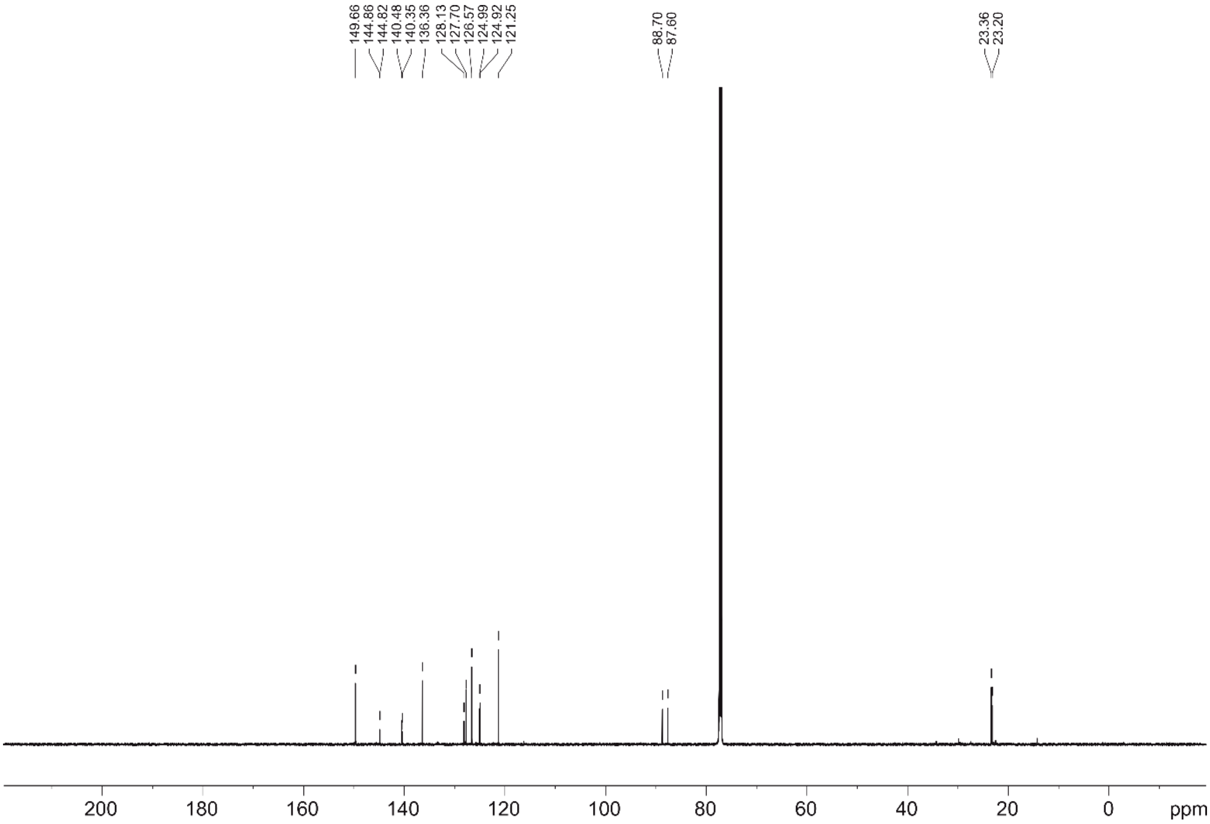
149.66
144.86
144.82
140.48
140.35
136.36
128.13
127.70
126.57
124.99
124.92
121.25
88.70
87.60
23.36
23.20
200
180
160
140
120
100
80
60
40
20
0
ppm

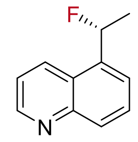


(*R*)-**2y**

**(*R*)-5-(1-fluoroethyl)quinoline (2y)**

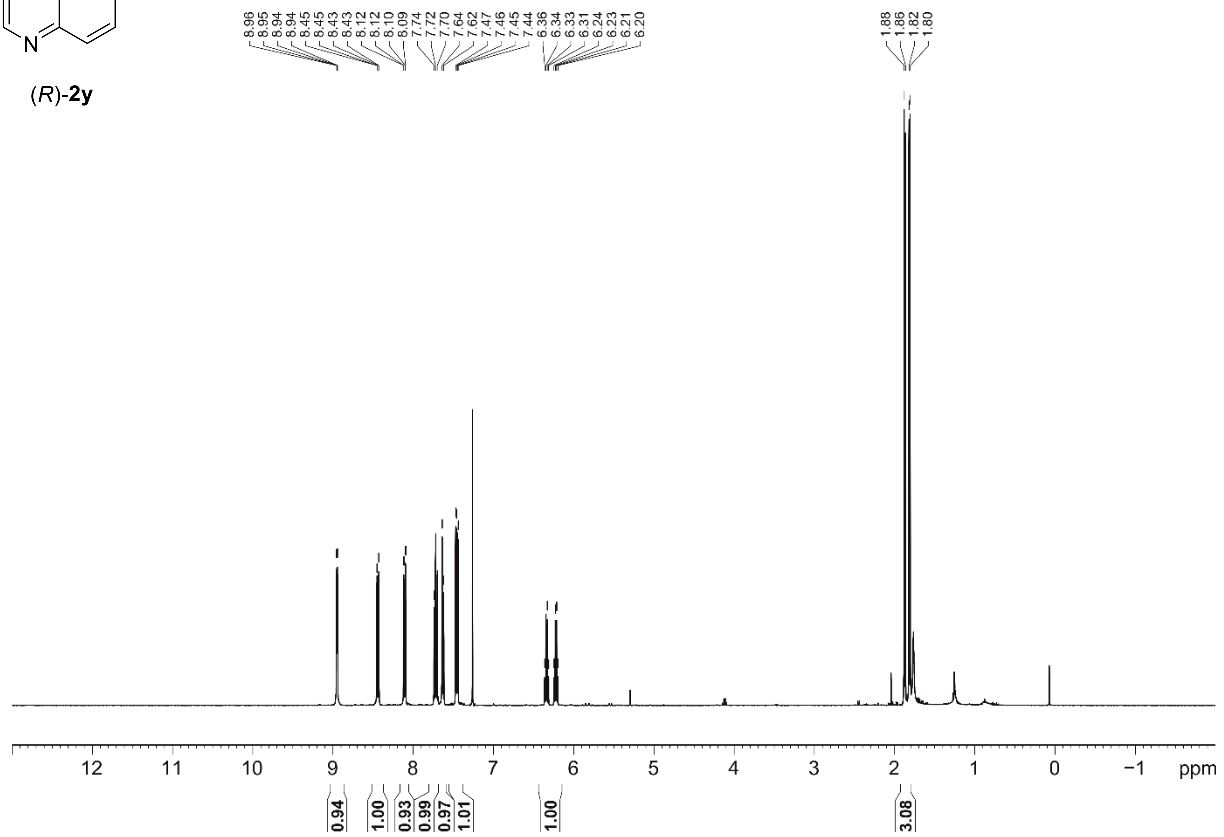


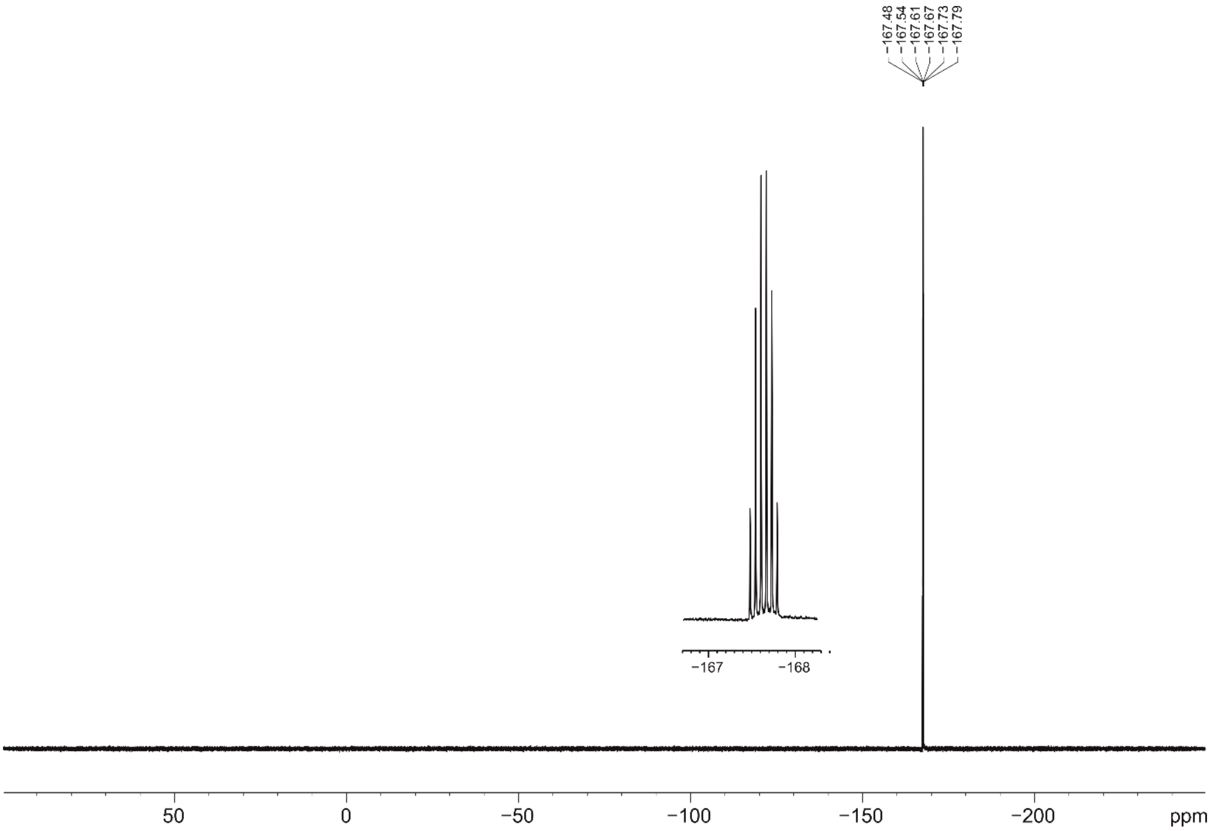

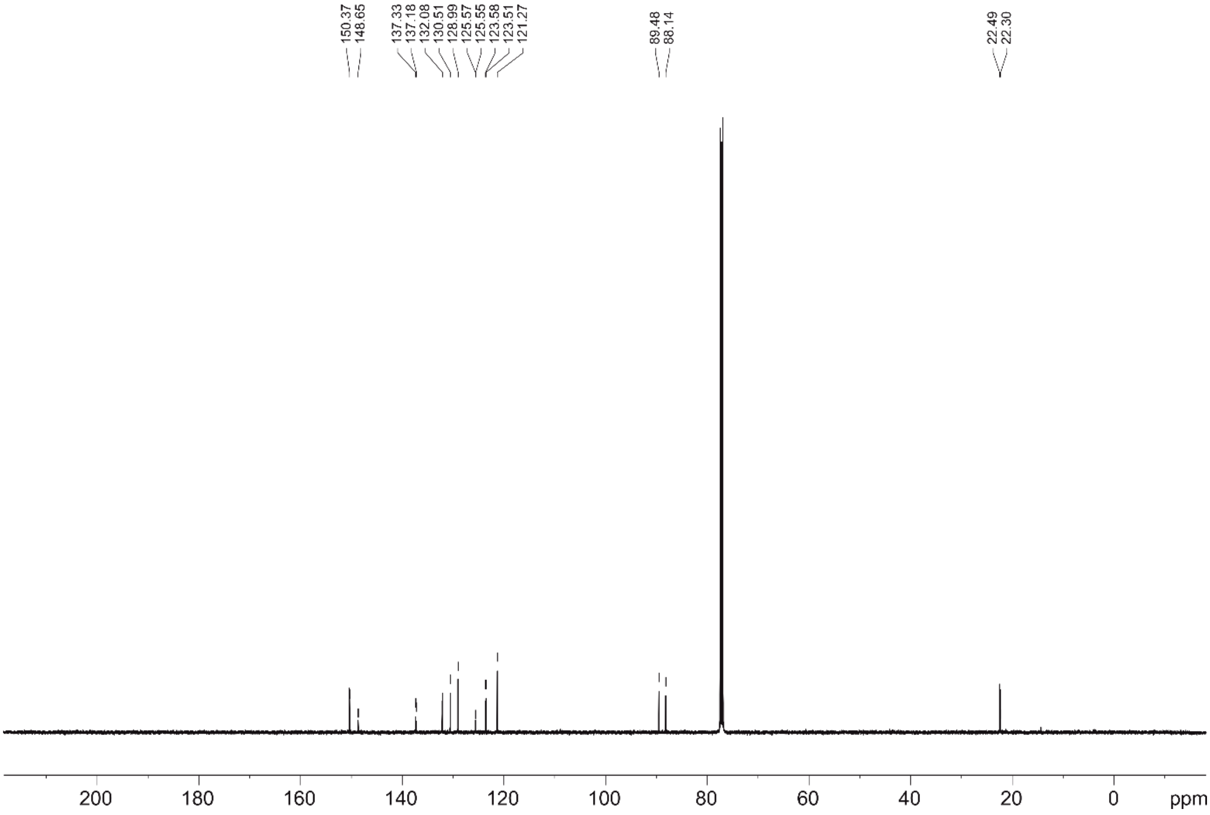


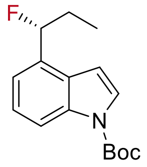


(*R*)-**2z**

**(*R*)-*tert*-butyl-4-(1-fluoropropyl)-1*H*-indole-1-carboxylate (2z)**

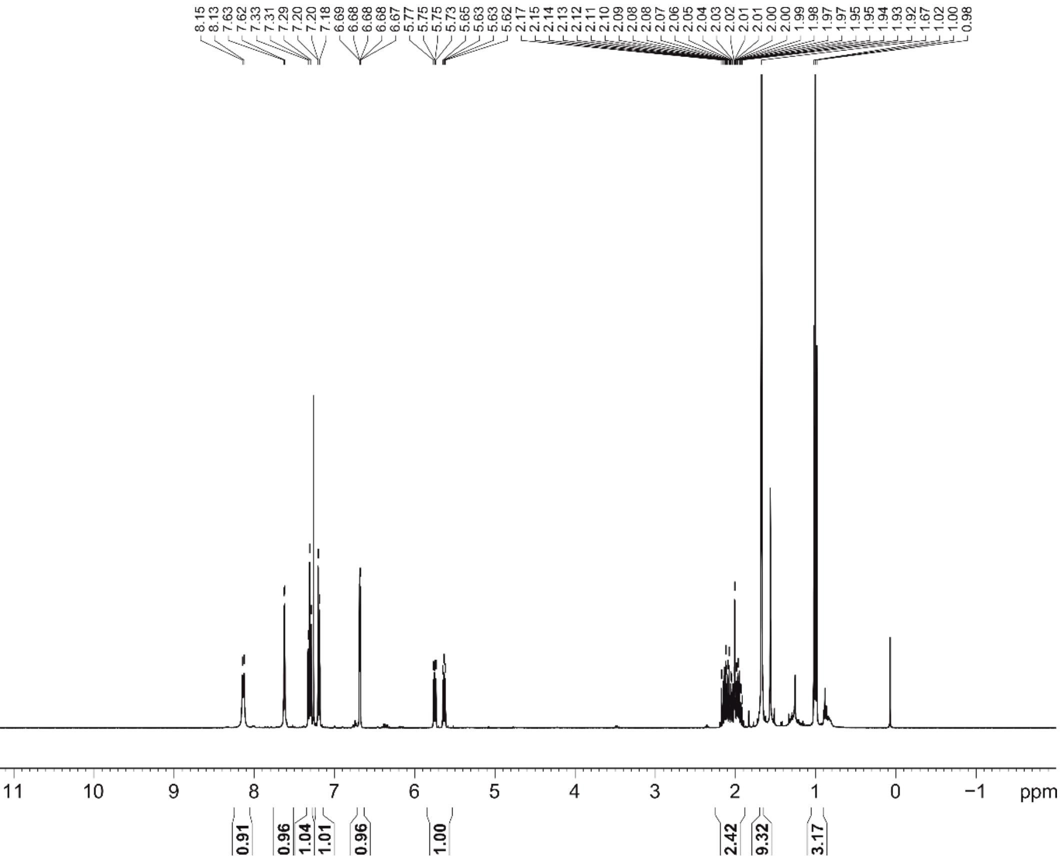

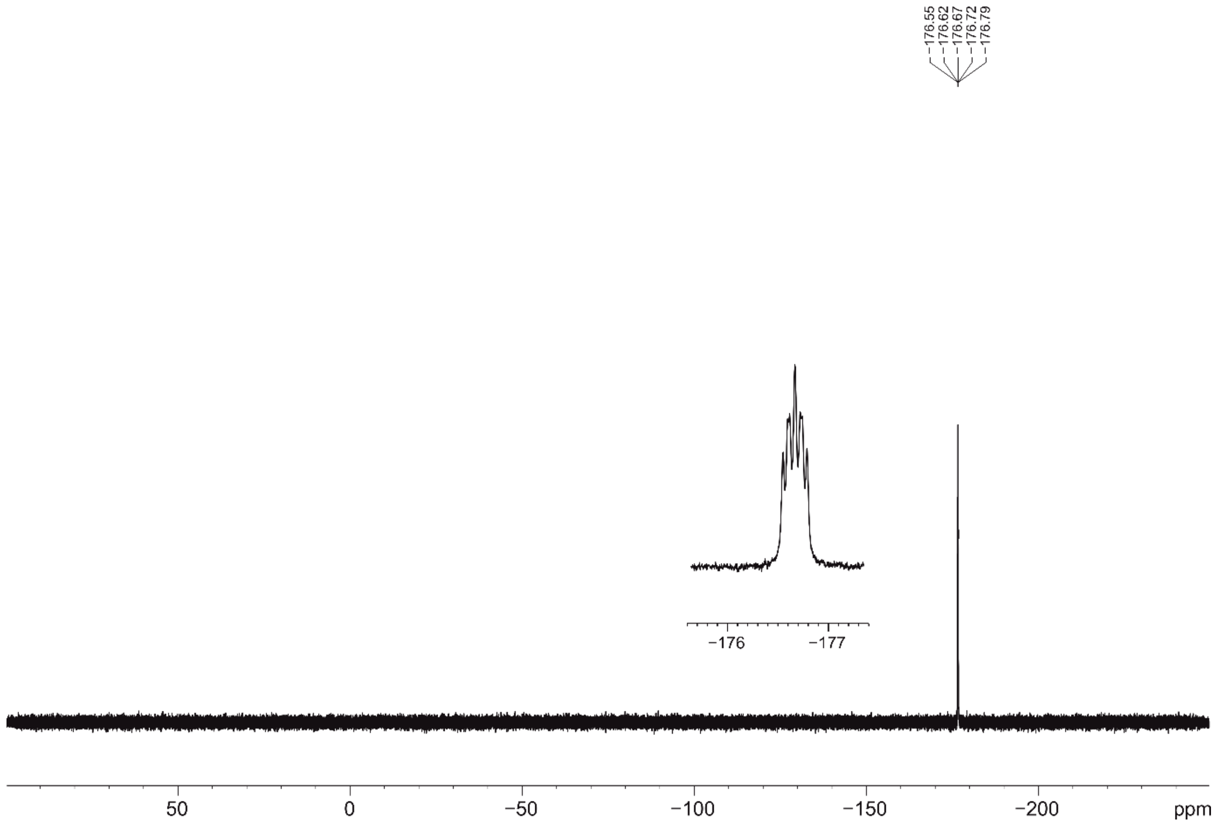

-176.55
-176.62
-176.67
-176.72
-176.79
−176
−177
50
0
−50
−100
−150
−200
ppm


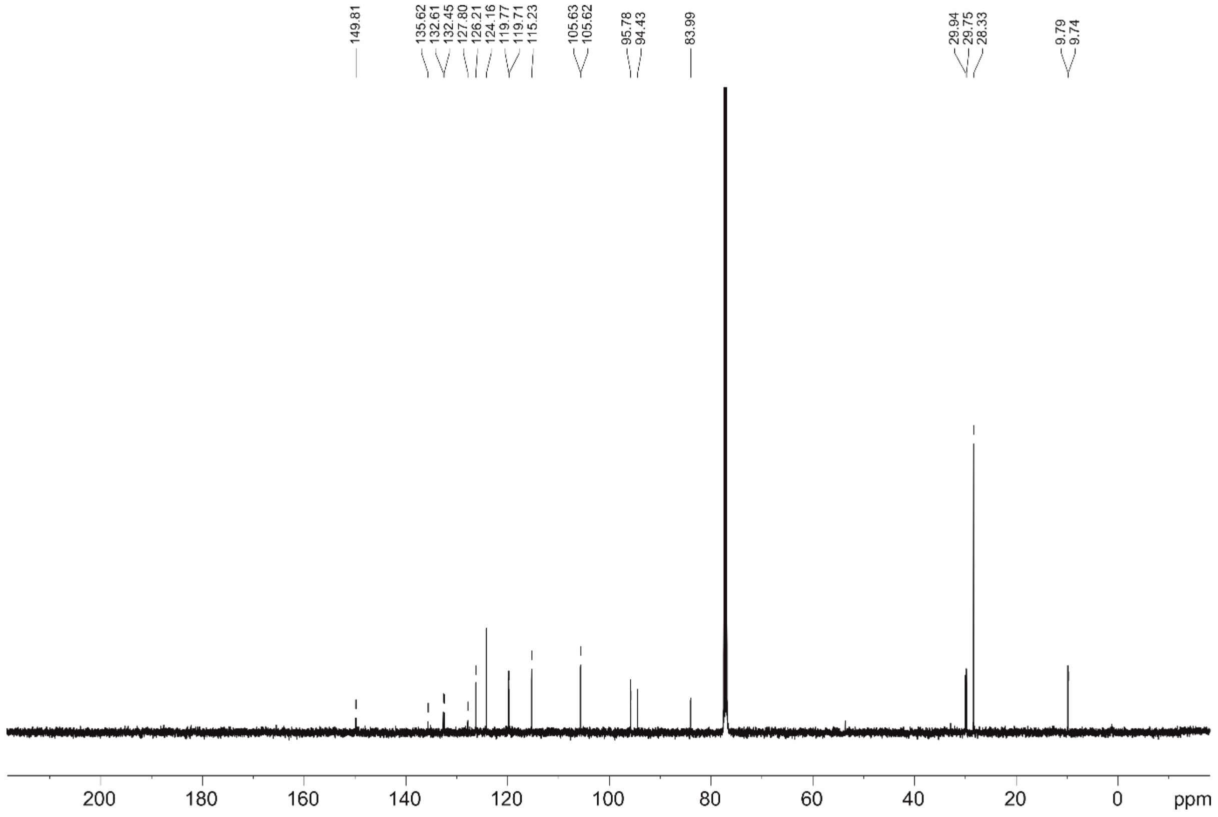

149.81
135.62
132.61
132.45
127.80
126.21
124.16
119.77
119.71
115.23
105.63
105.62
95.78
94.43
83.99
29.94
29.75
28.33
9.79
9.74
200
180
160
140
120
100
80
60
40
20
0
ppm

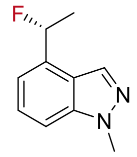

(*R*)-**2aa**

**(*R*)-4-(1-fluoroethyl)-1-methyl-1*H*-indazole (2aa)**

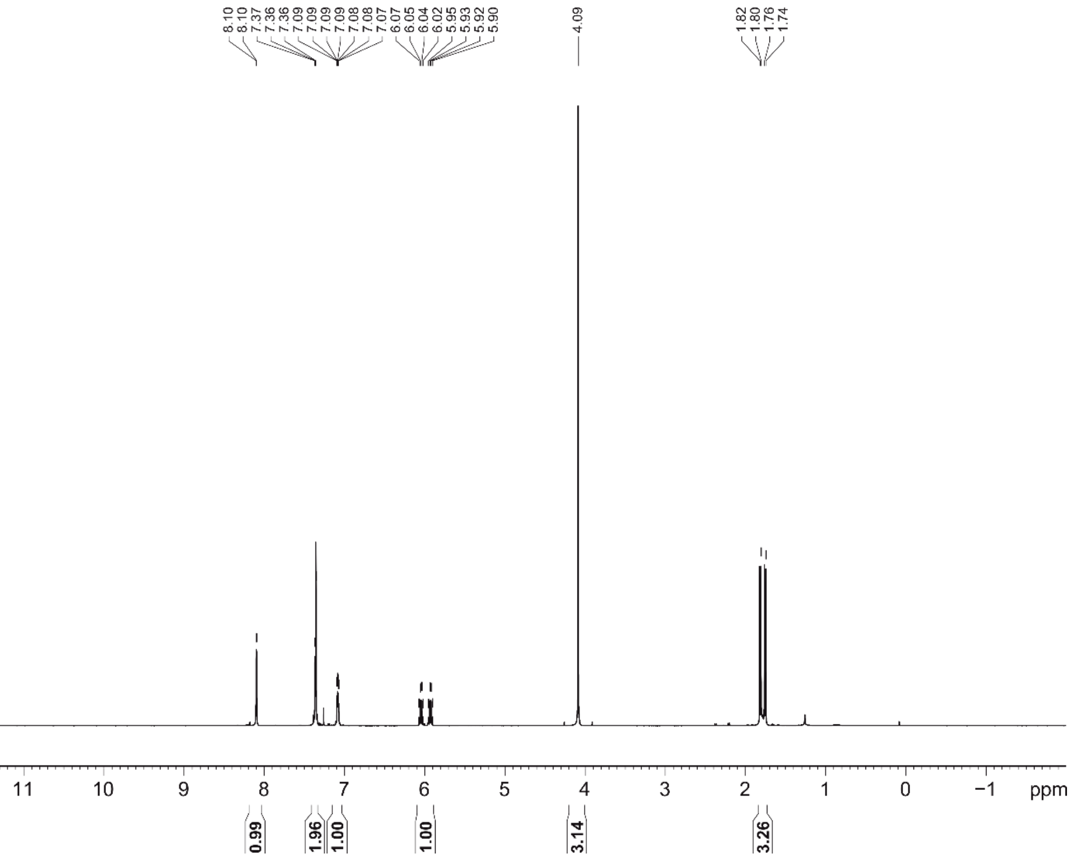


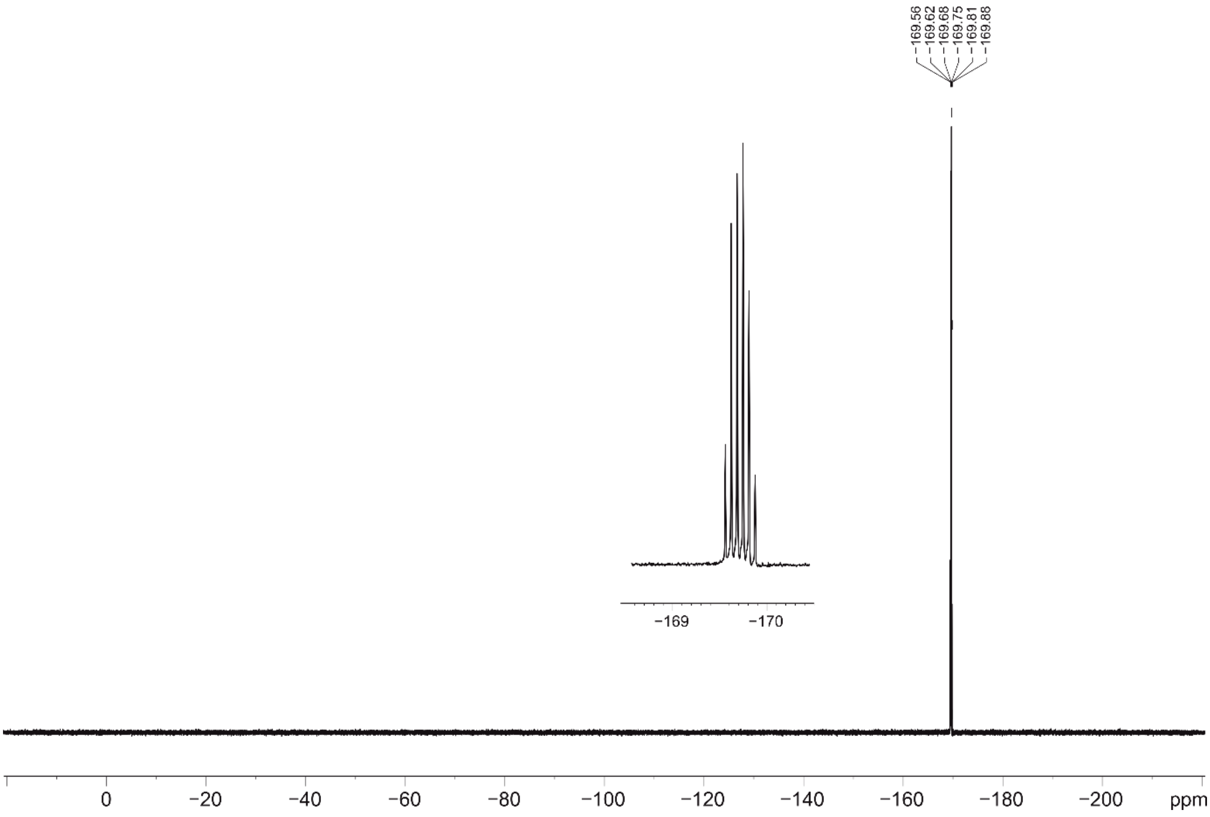

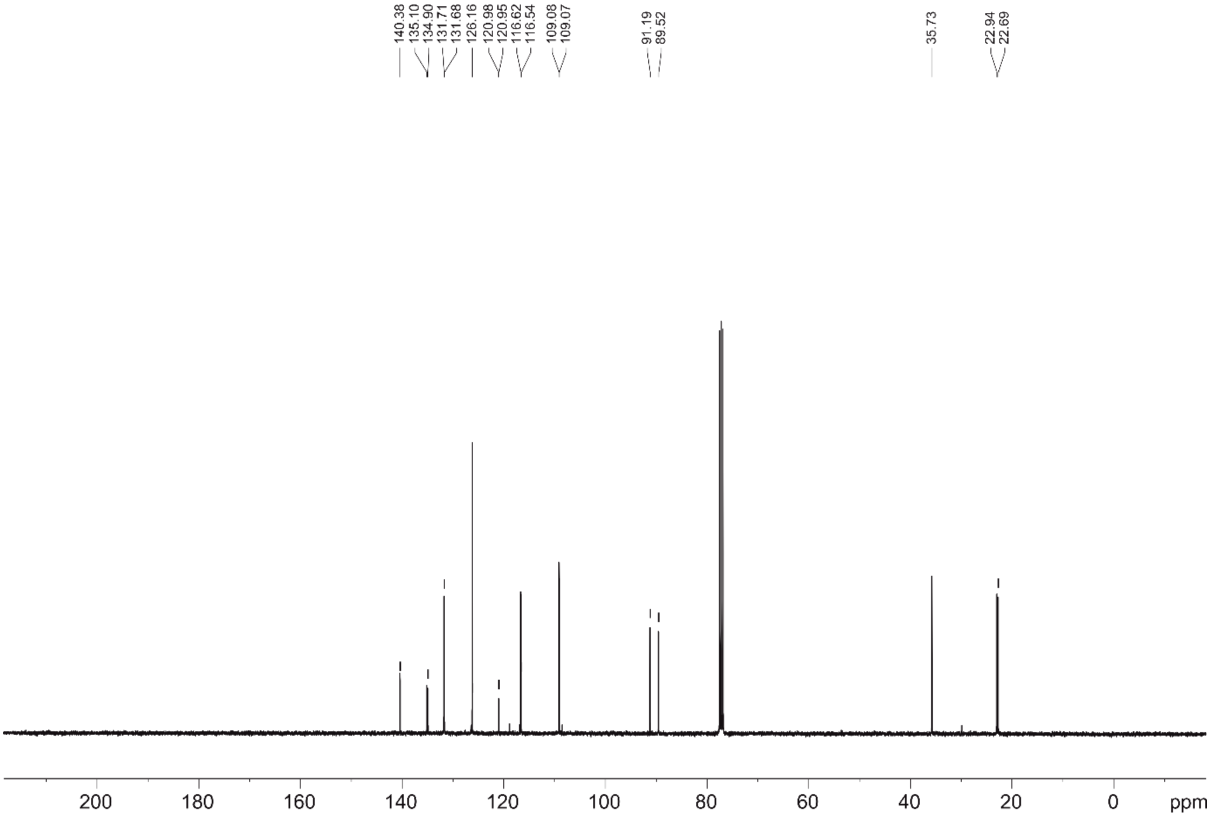


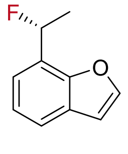


(*R*)-**2ab**

**(*R*)-7-(1-fluoroethyl)benzofuran (2ab)**

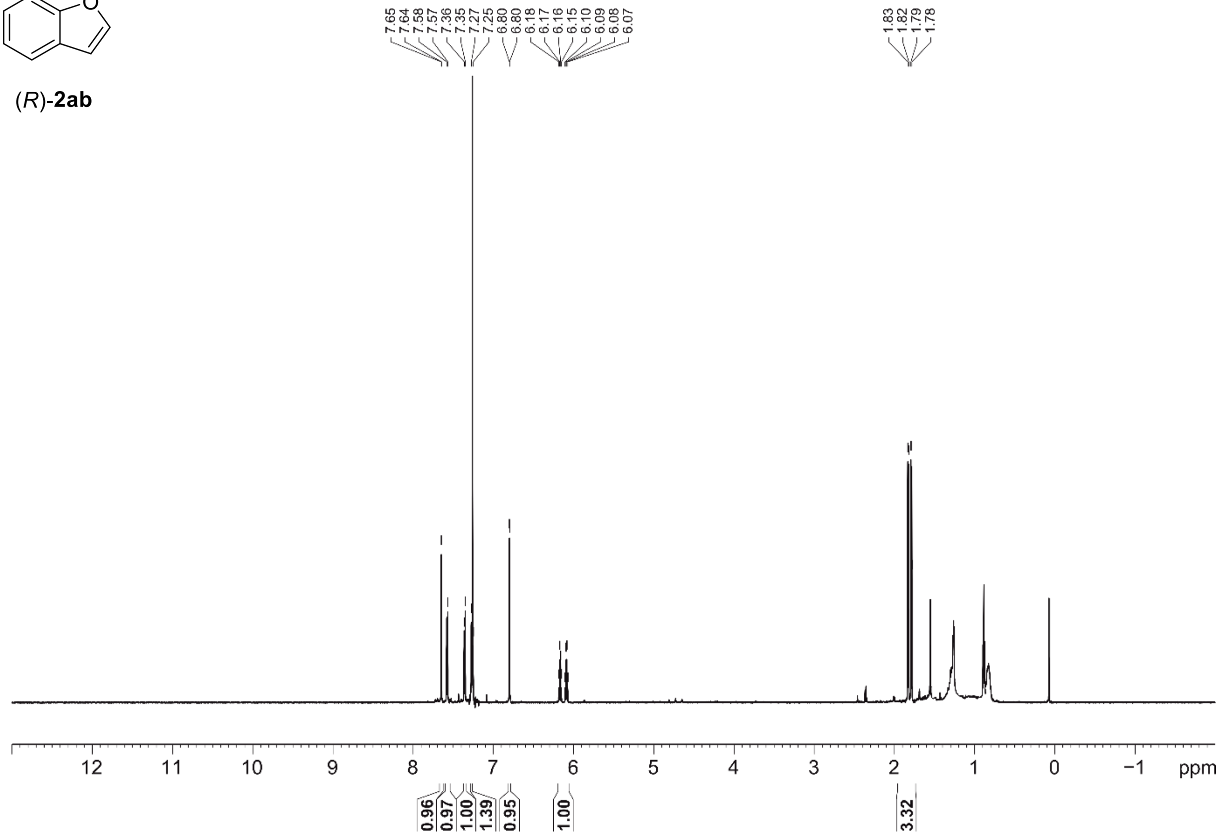

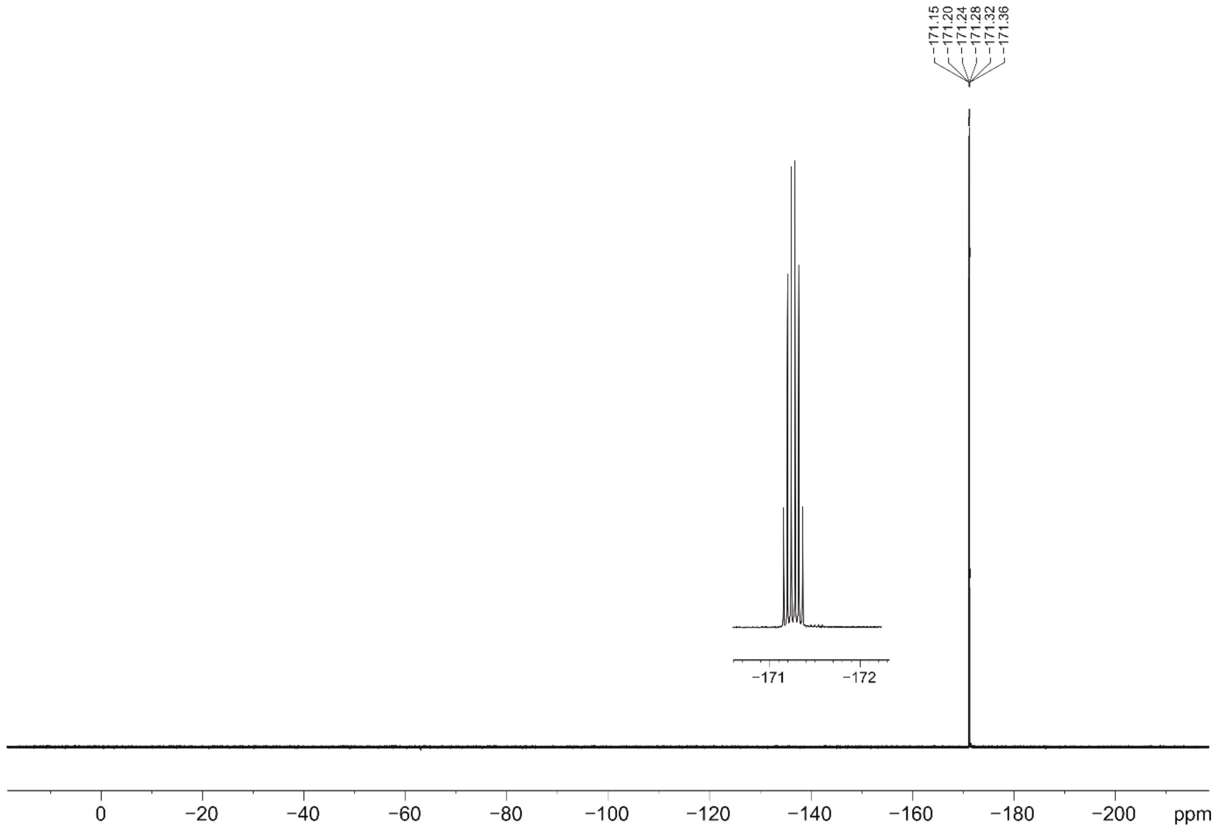
−171.15
−171.20
−171.24
−171.28
−171.32
−171.36
−171
−172
0
−20
−40
−60
−80
−100
−120
−140
−160
−180
−200
ppm

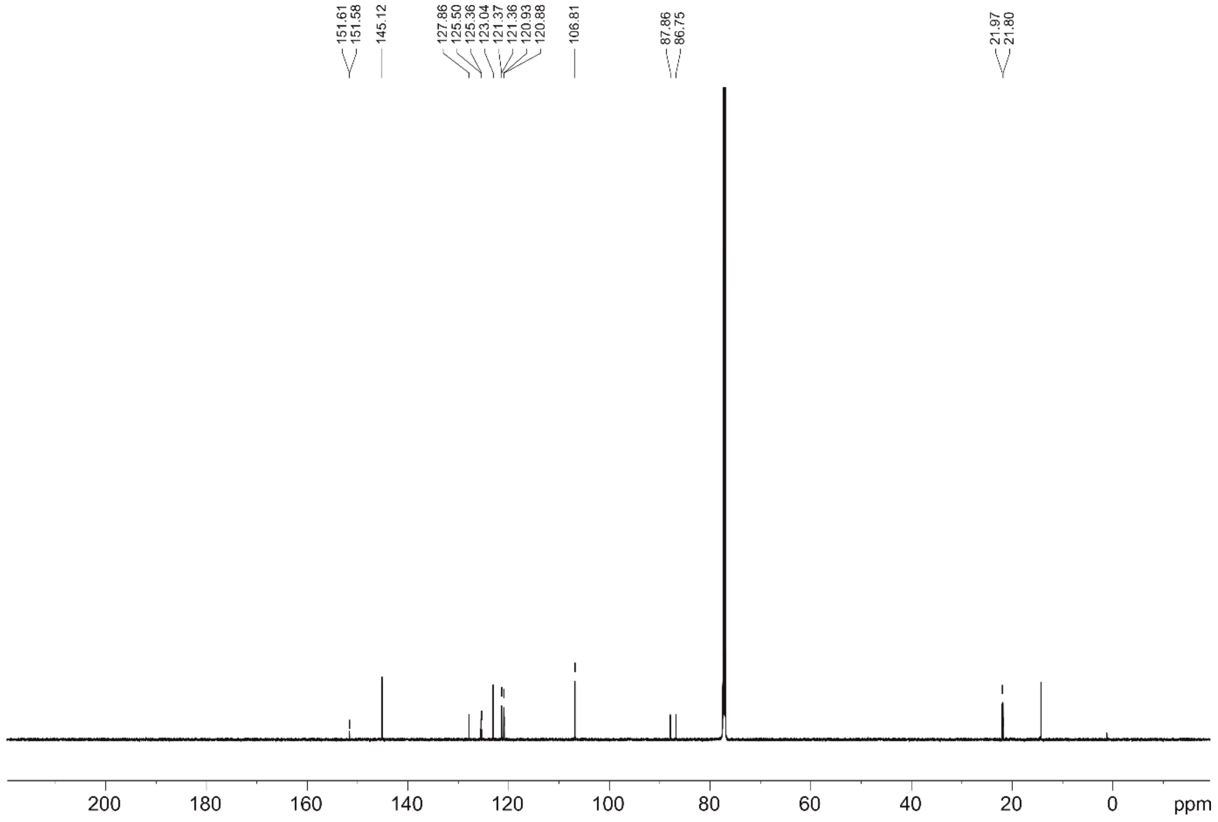
151.61
151.58
145.12
127.86
125.50
125.36
123.04
121.37
121.36
120.93
120.88
106.81
87.86
86.75
21.97
21.80
200
180
160
140
120
100
80
60
40
20
0
ppm

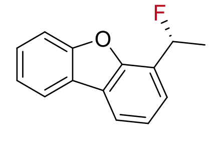


(*R*)-**2ac**

**(*R*)-4-(1-fluoroethyl)dibenzo[*b,d*]furan (2ac)**

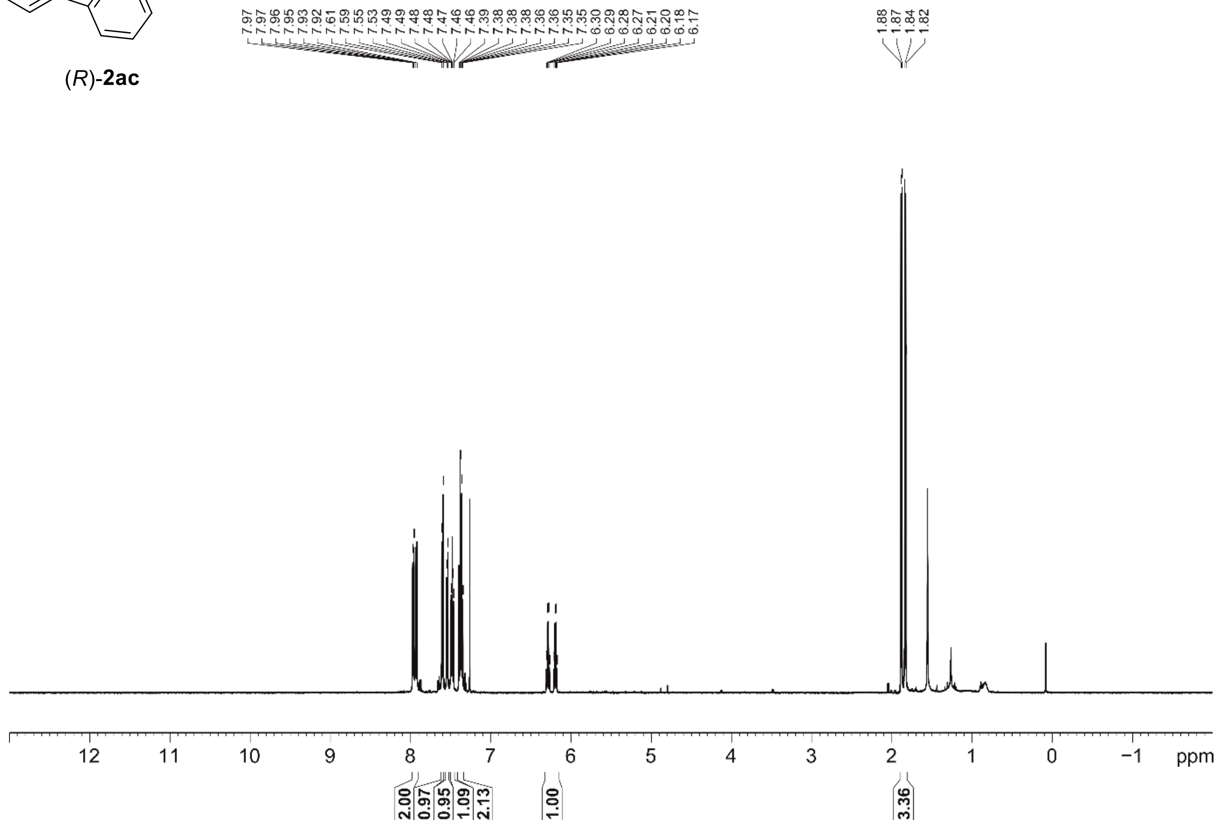


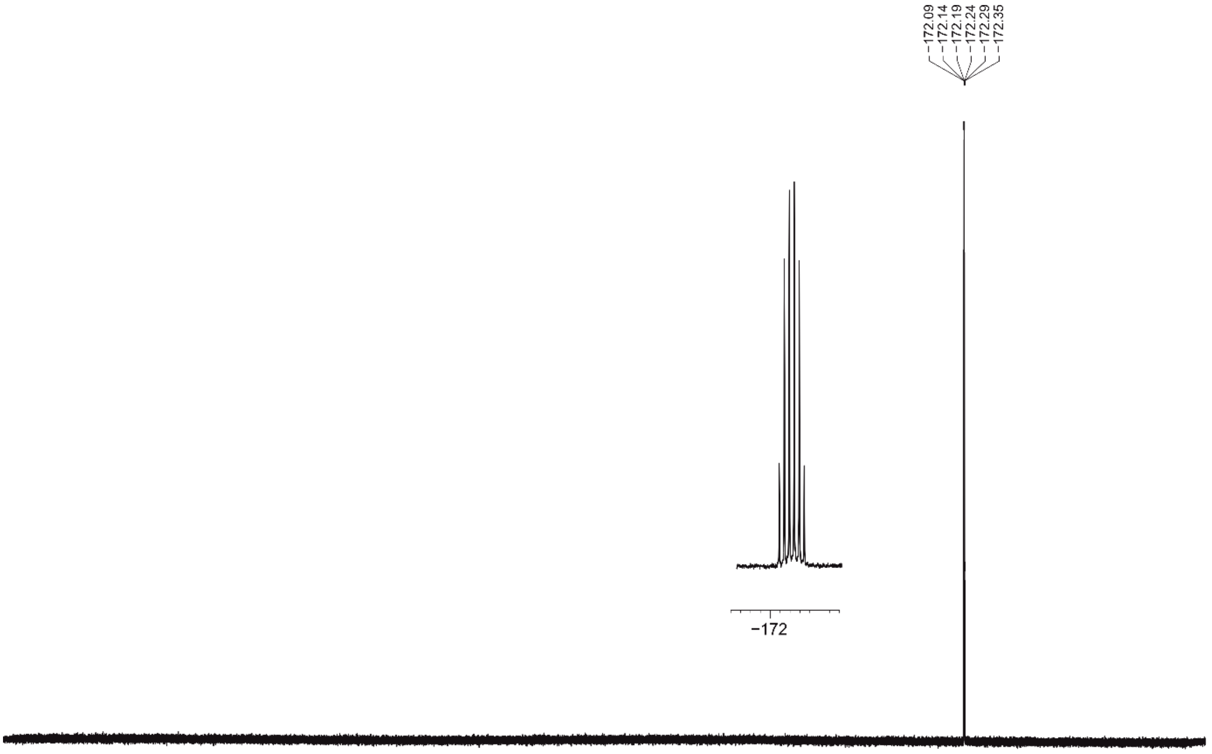

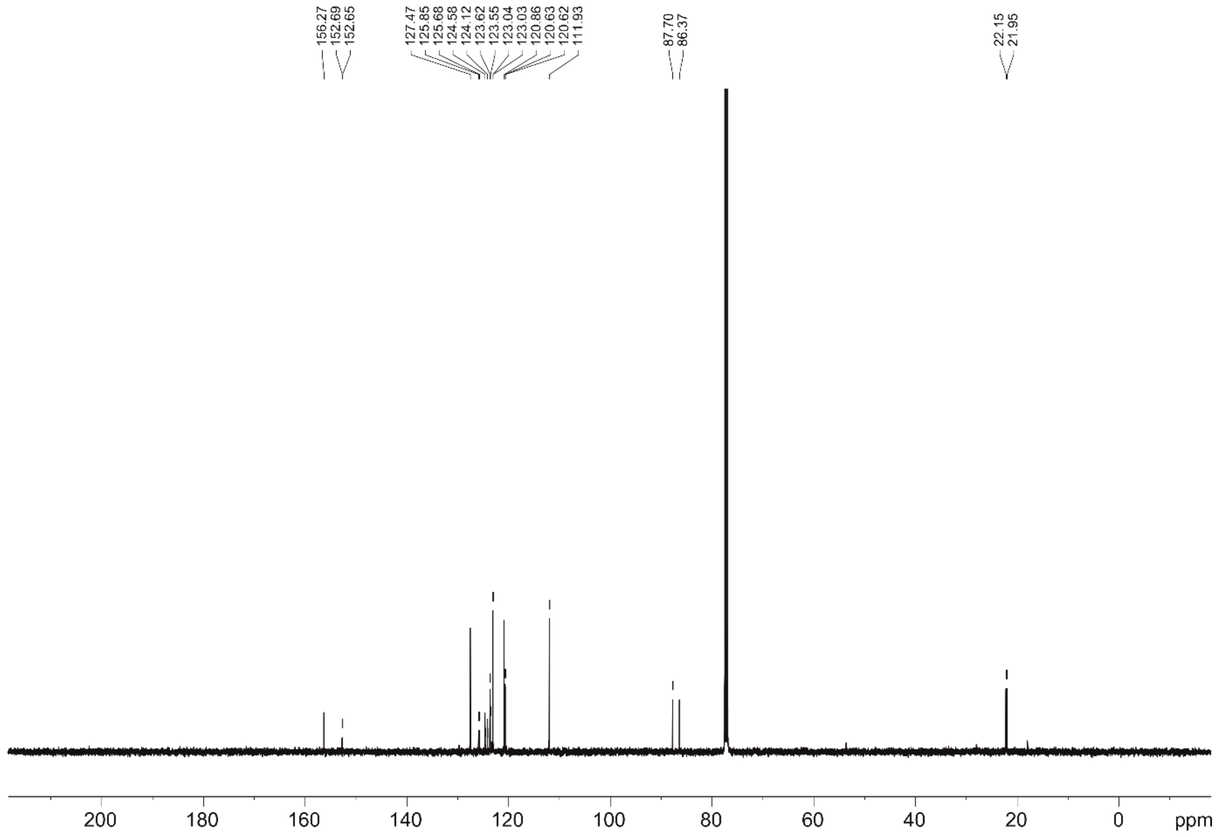


**(*R*)-4-(1-fluoroethyl)dibenzo[*b*,*d*]thiophene (2ad)**

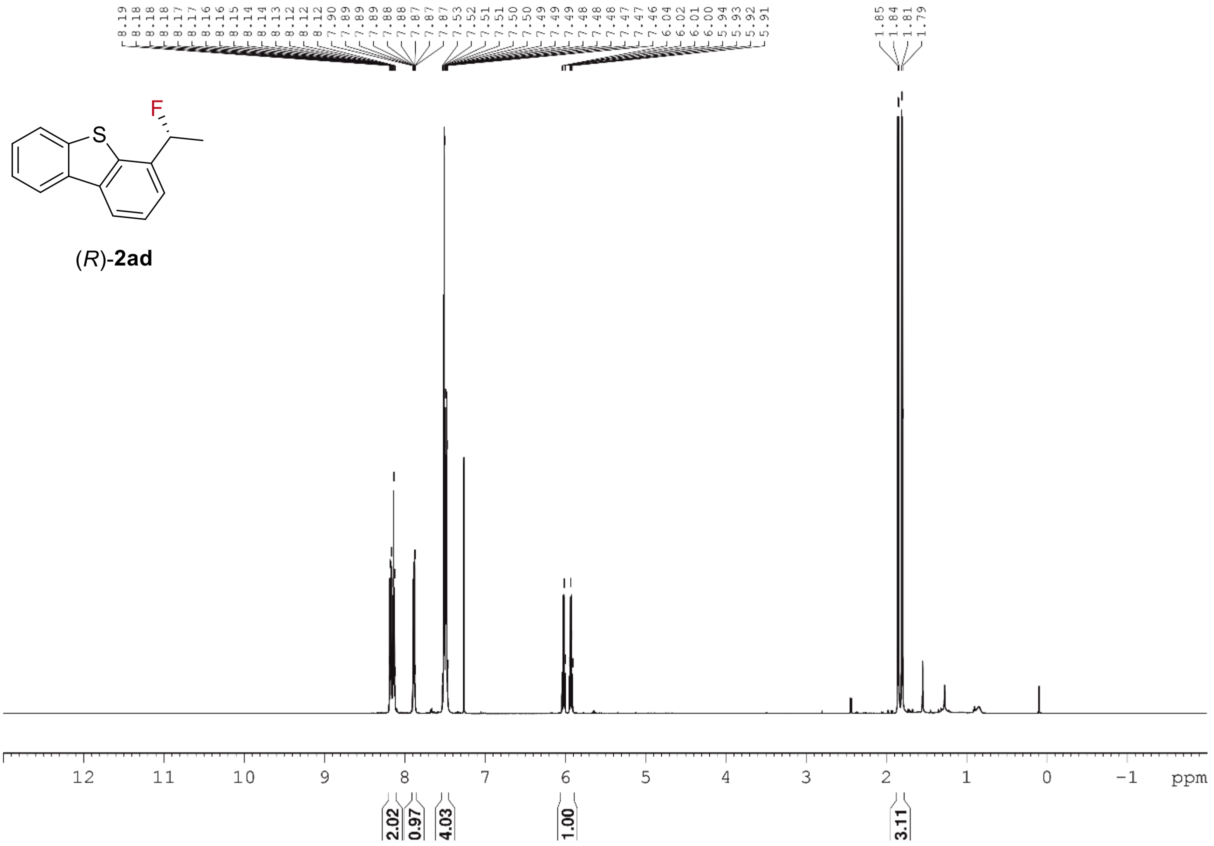

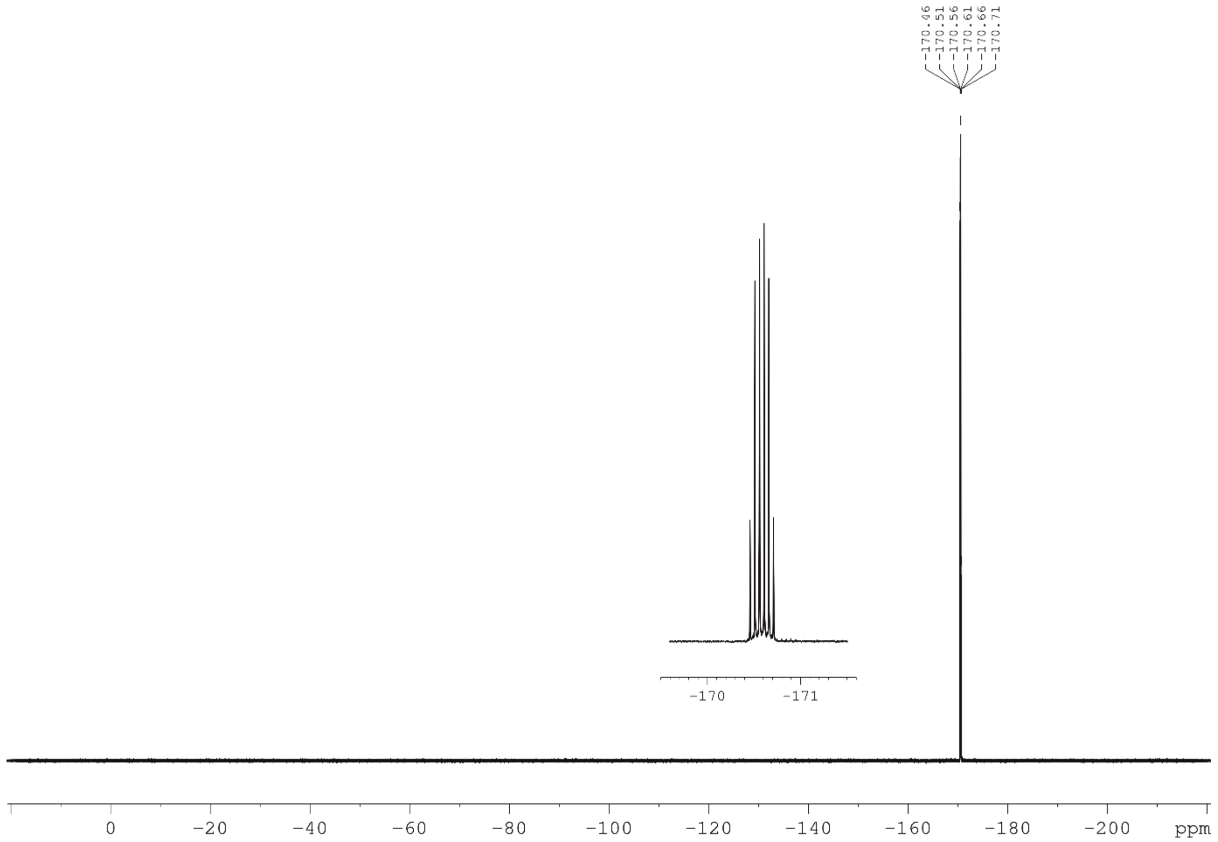
-170.46
-170.51
-170.56
-170.61
-170.66
-170.71
-170
-171
0
-20
-40
-60
-80
-100
-120
-140
-160
-180
-200
ppm

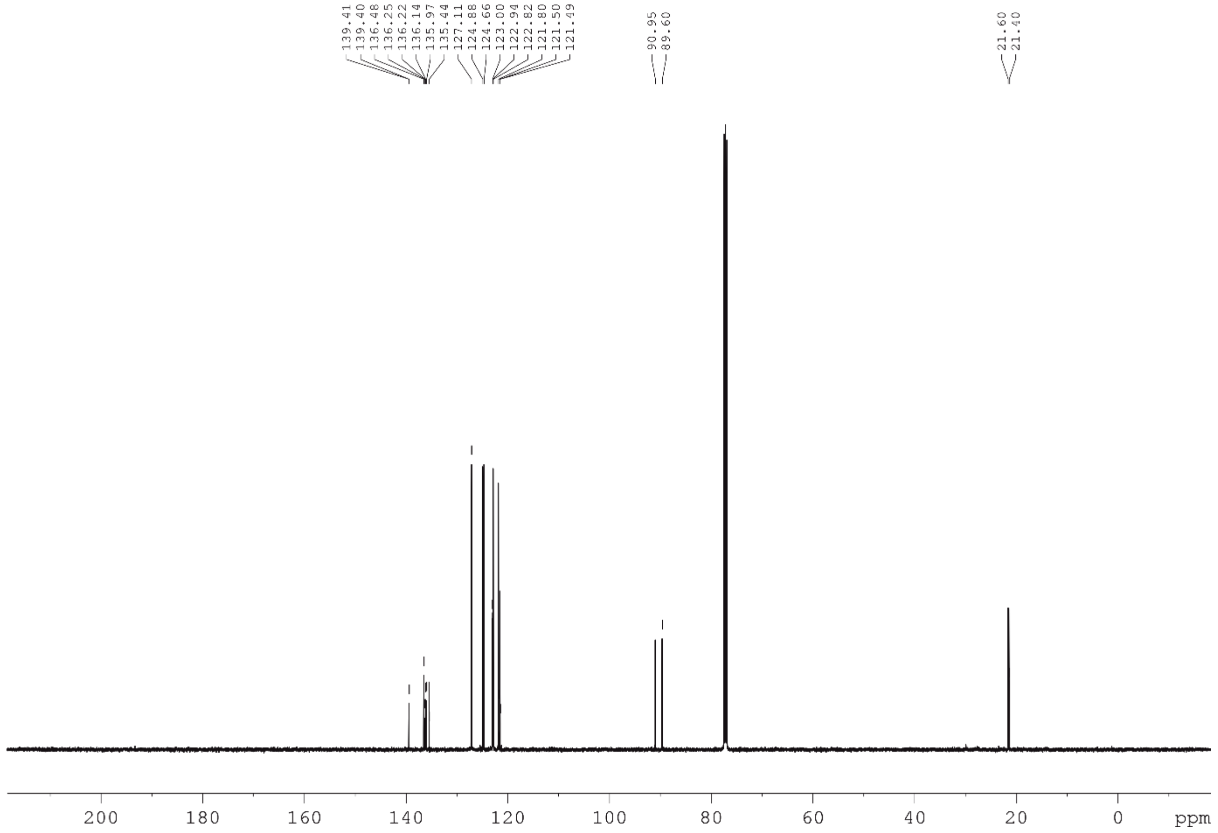
139.41
139.40
136.48
136.25
136.22
136.14
135.97
135.44
127.11
124.88
124.66
123.00
122.94
122.82
121.80
121.50
121.49
90.95
89.60
21.60
21.40
200
180
160
140
120
100
80
60
40
20
0
ppm

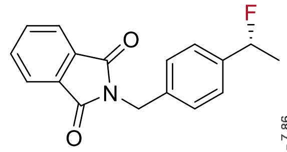


(*R*)-**2ae**

## (*R*)-2-(4-(1-fluoroethyl)benzyl)isoindoline-1,3-dione (2ae)

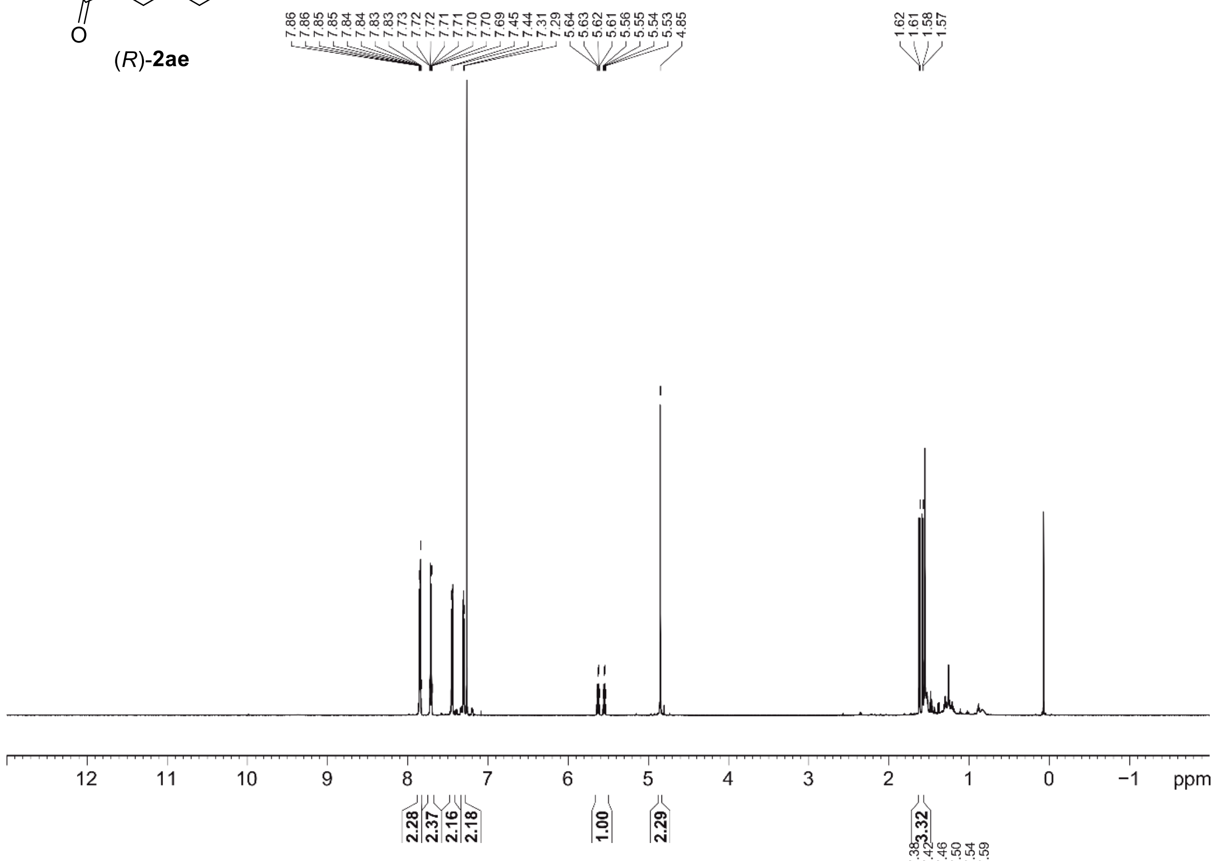


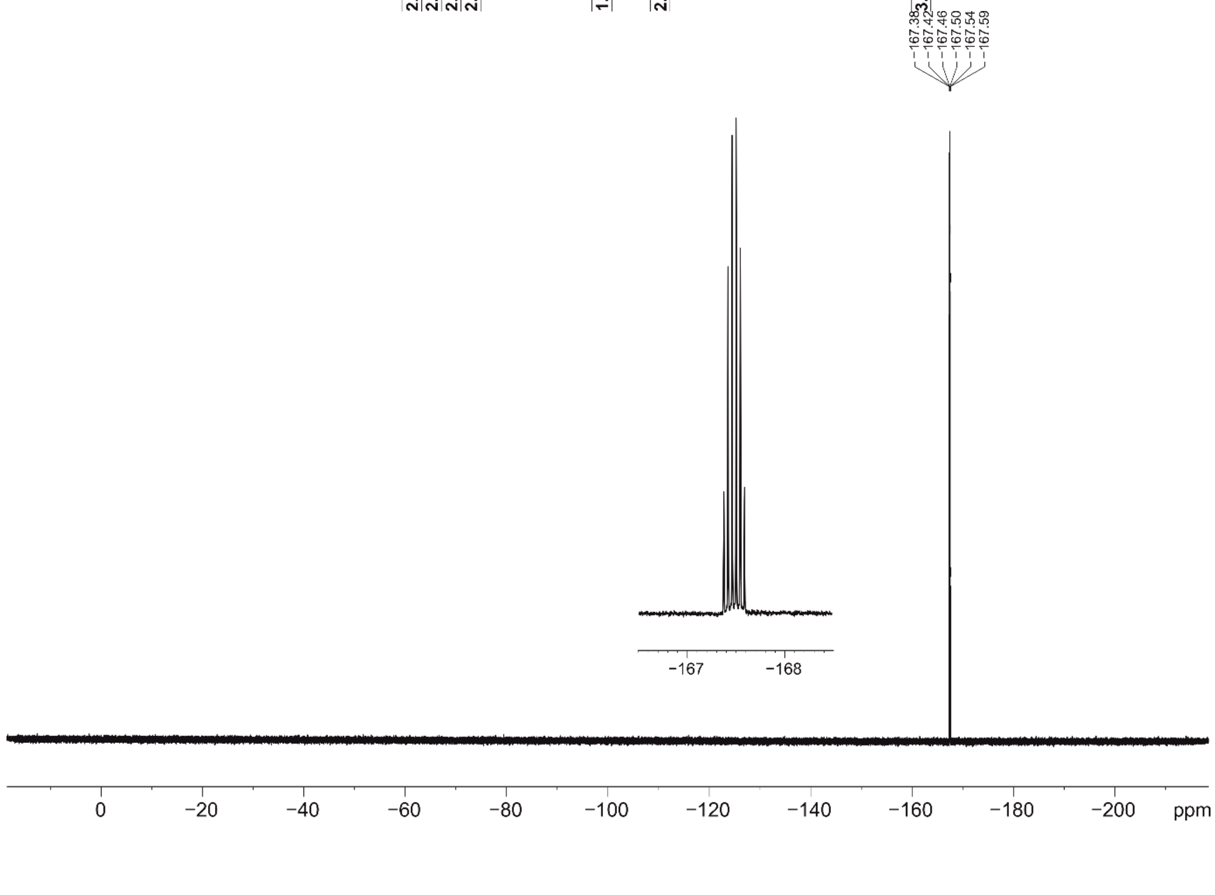

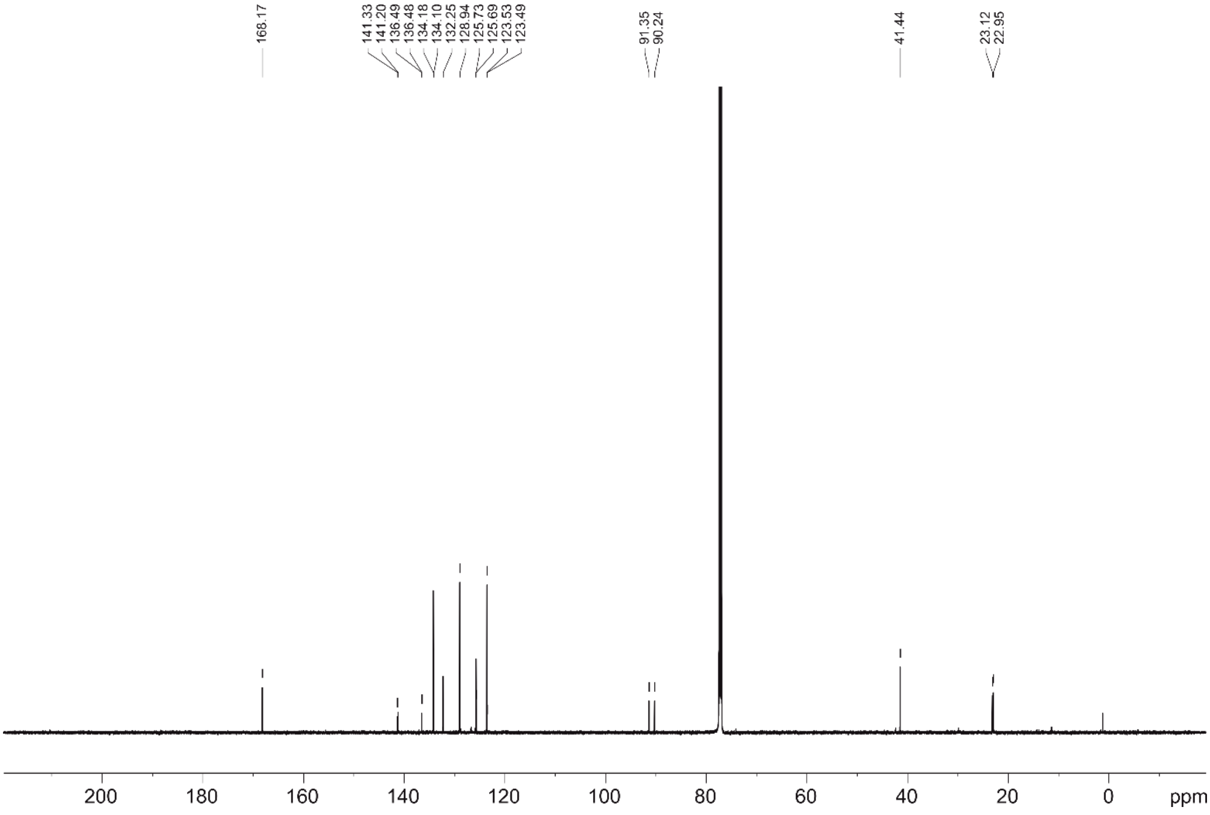


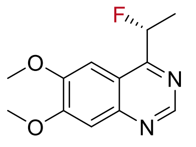

(*R*)-**2af**

**(*R*)-4-(1-fluoroethyl)-6,7-dimethoxyquinazoline (2af)**

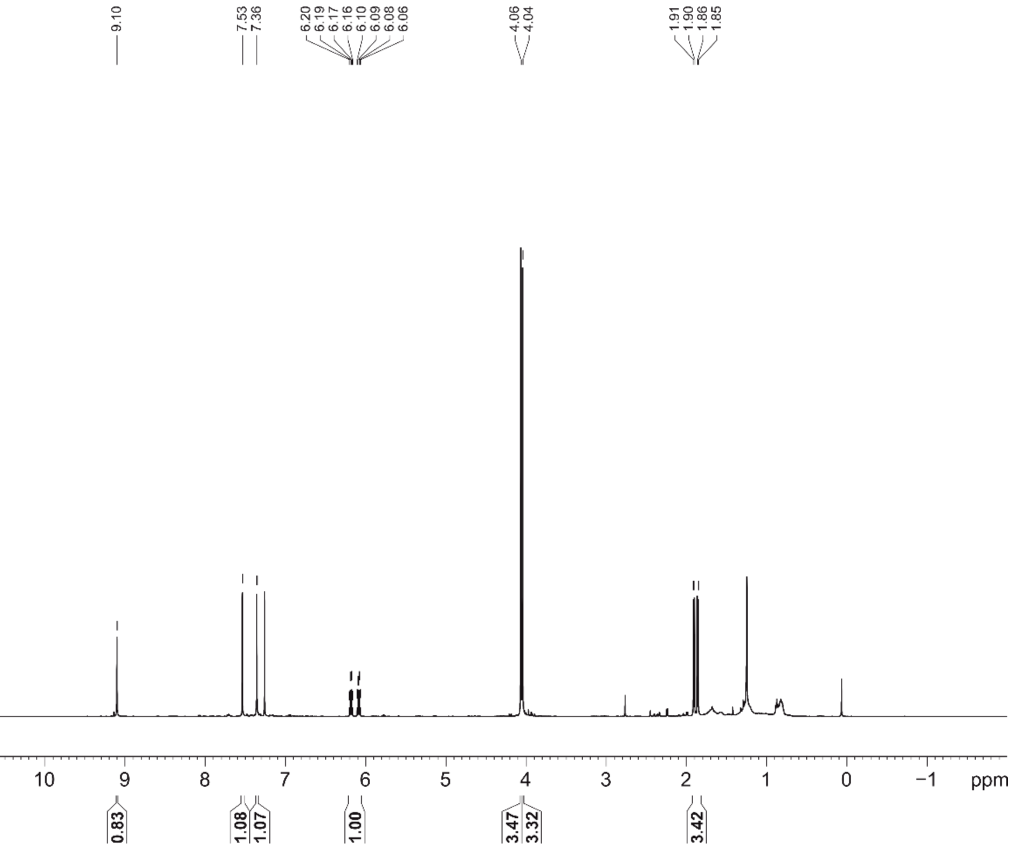

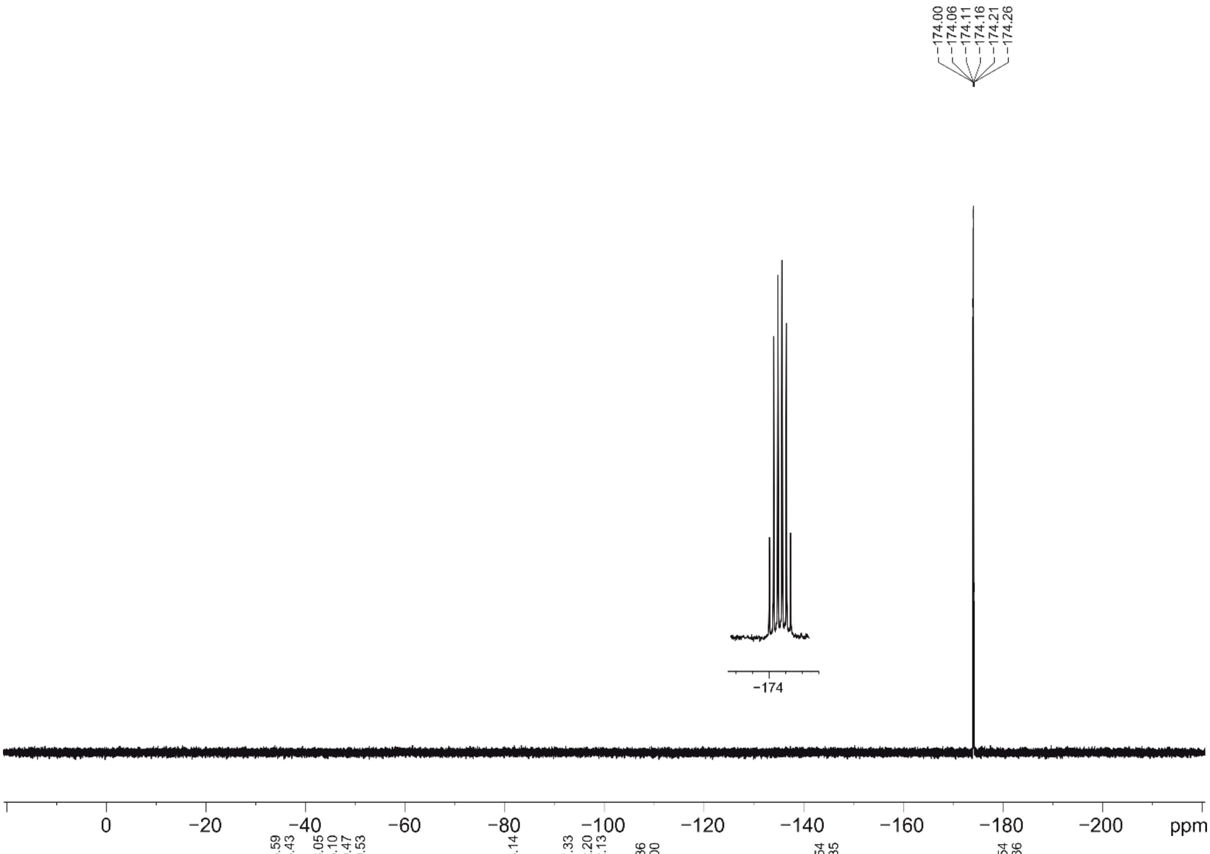

-174.00
-174.06
-174.11
-174.16
-174.21
-174.26
-174
0 −20 −40 −60 −80 −100 −120 −140 −160 −180 −200 ppm


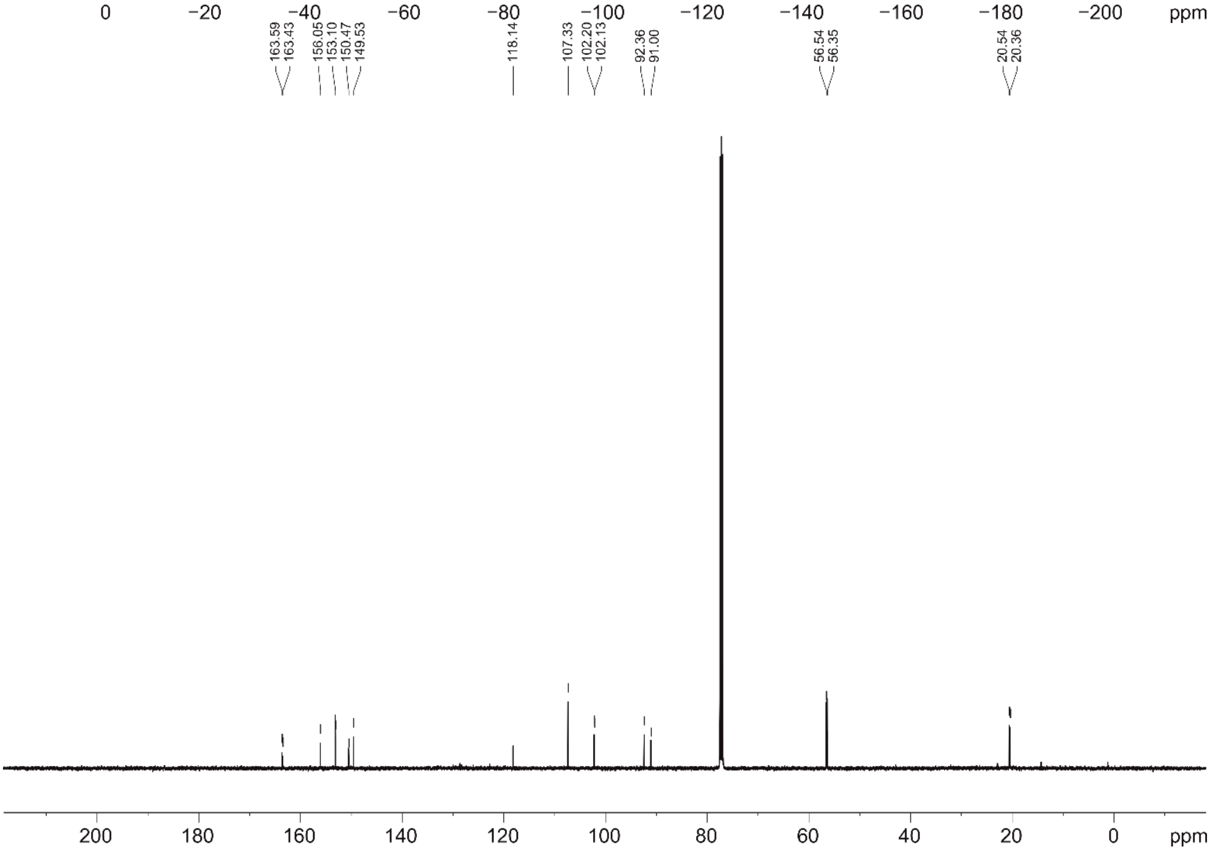

163.59
163.43
156.05
153.10
150.47
149.53
118.14
107.33
102.20
102.13
92.36
91.00
56.54
56.35
20.54
20.36
200 180 160 140 120 100 80 60 40 20 0 ppm

## isopropyl (*R*)-2-(4-(4-(1-fluoroethyl)benzoyl)phenoxy)-2-methylpropanoate (2ag)

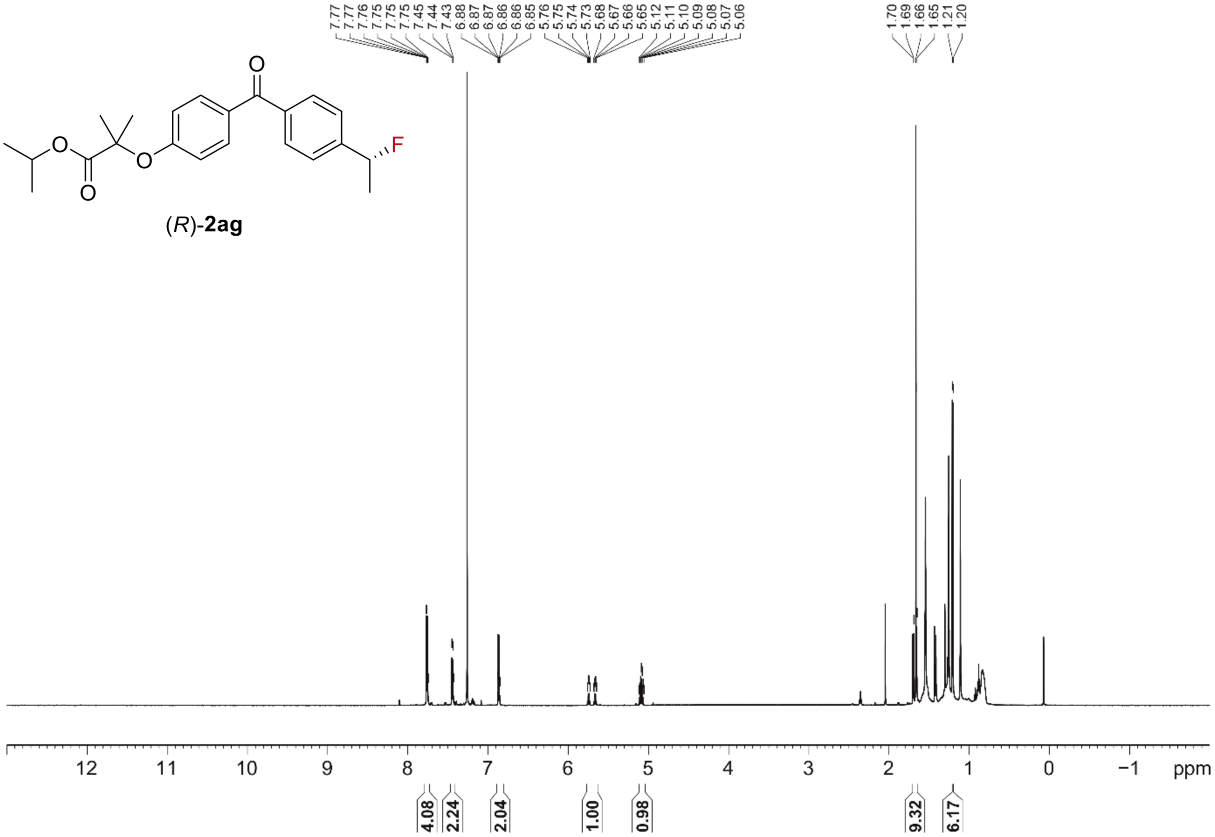


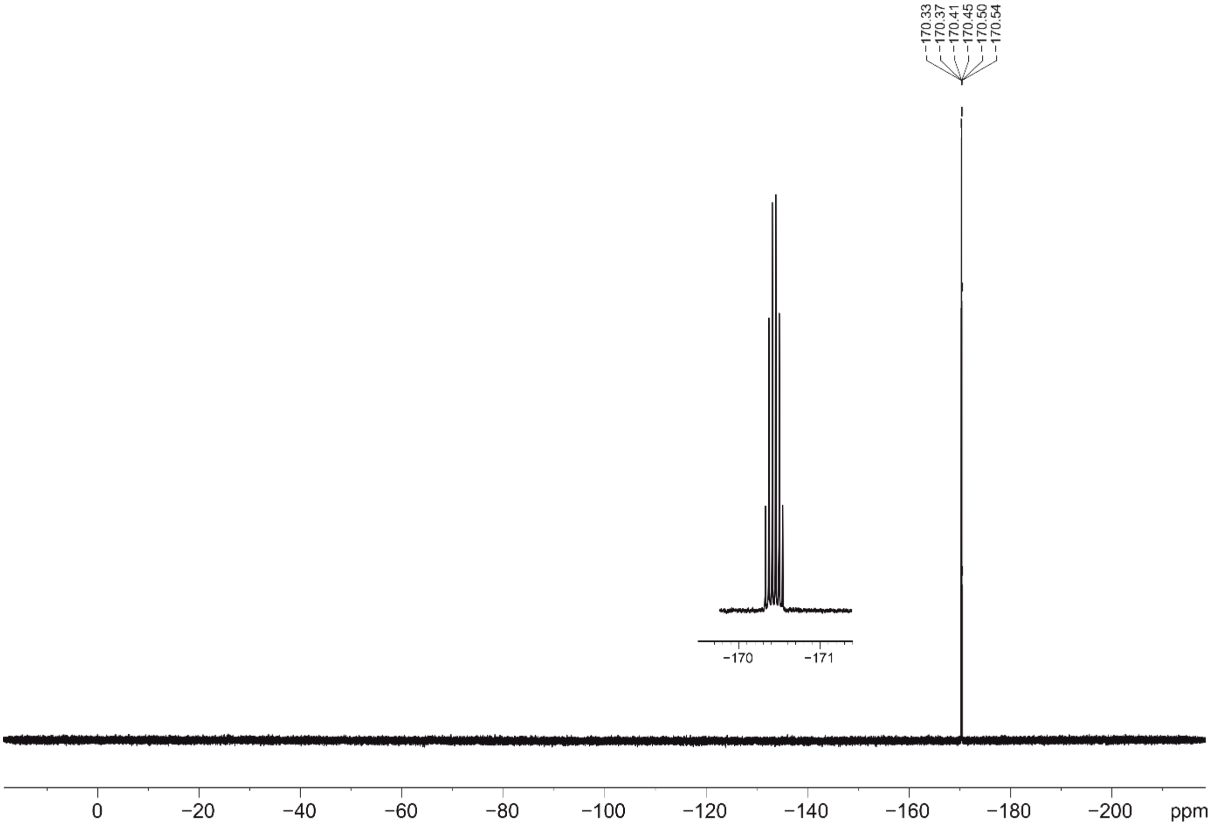

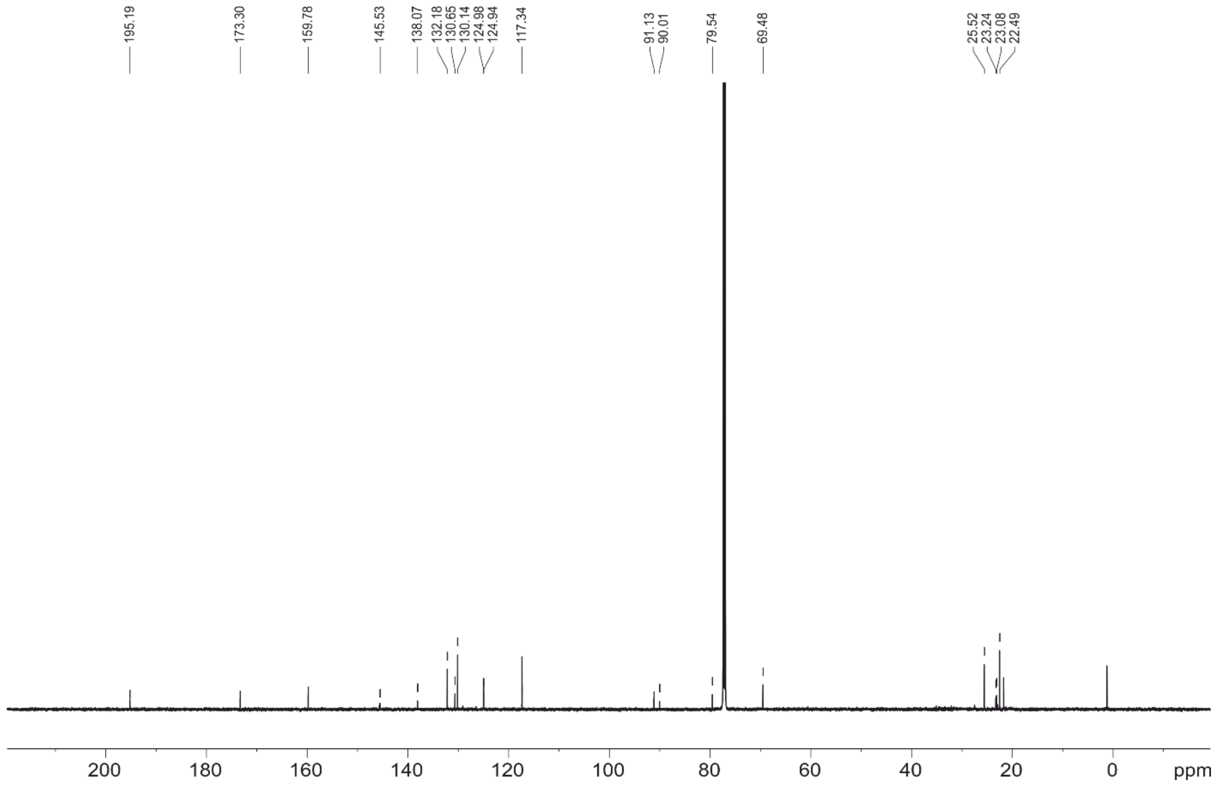


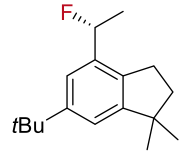

(*R*)-**2ah**

**(*R*)-6-(*tert*-butyl)-4-(1-fluoroethyl)-1,1-dimethyl-2,3-dihydro-1*H*-indene (2ah)**

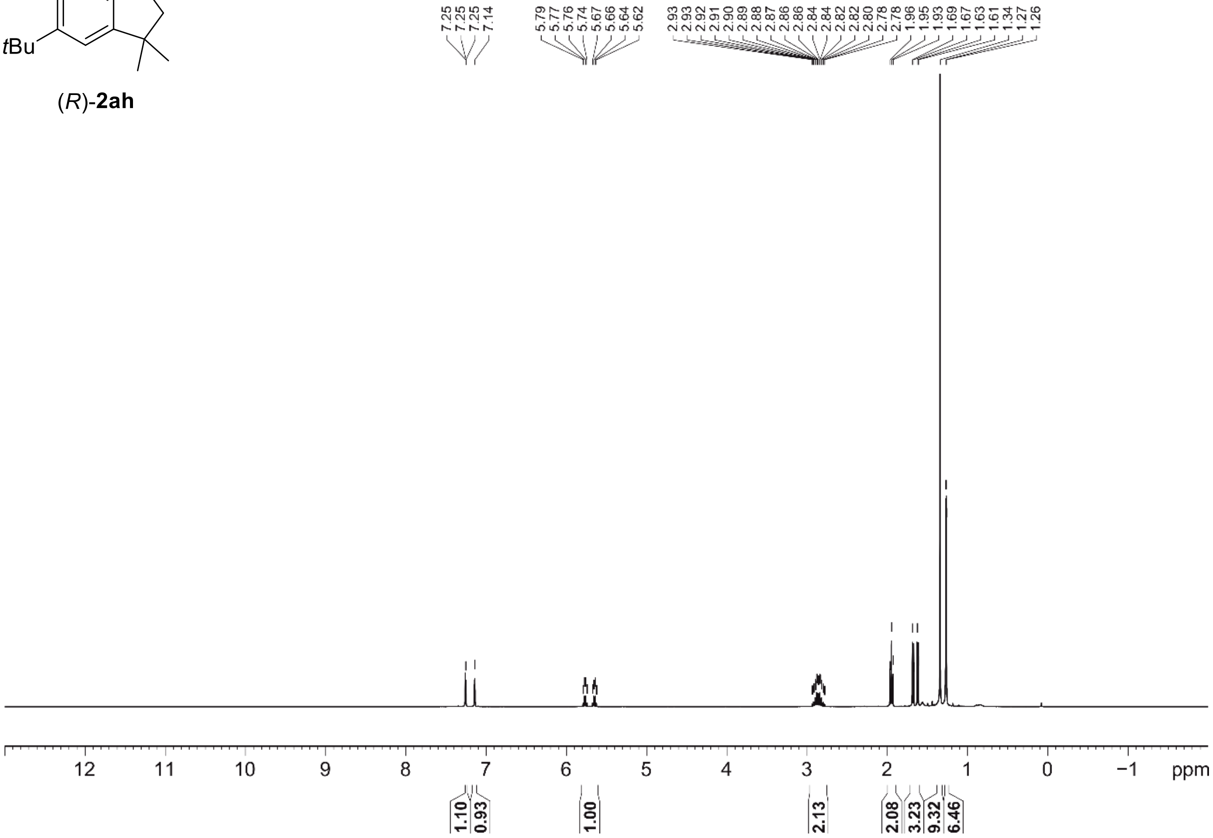

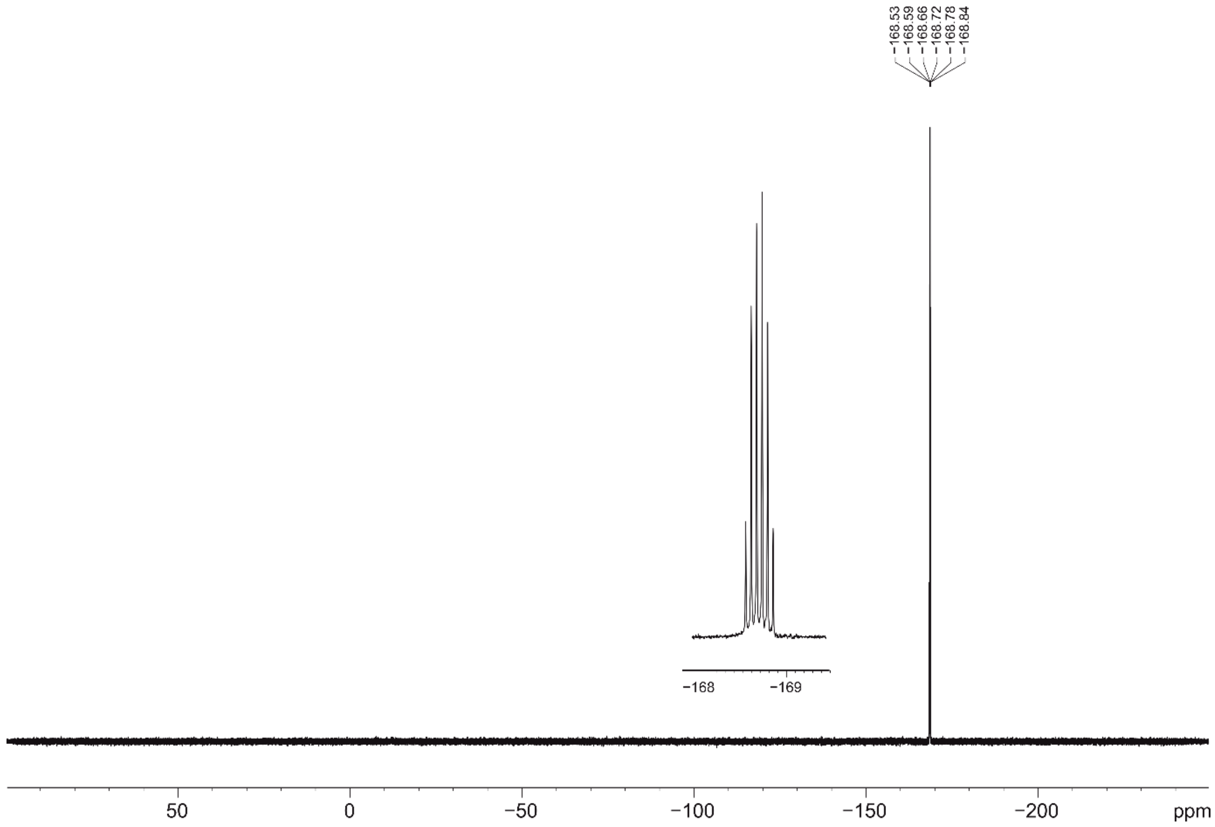

-168.53
-168.59
-168.66
-168.72
-168.78
-168.84
-168
-169
50
0
−50
−100
−150
−200
ppm


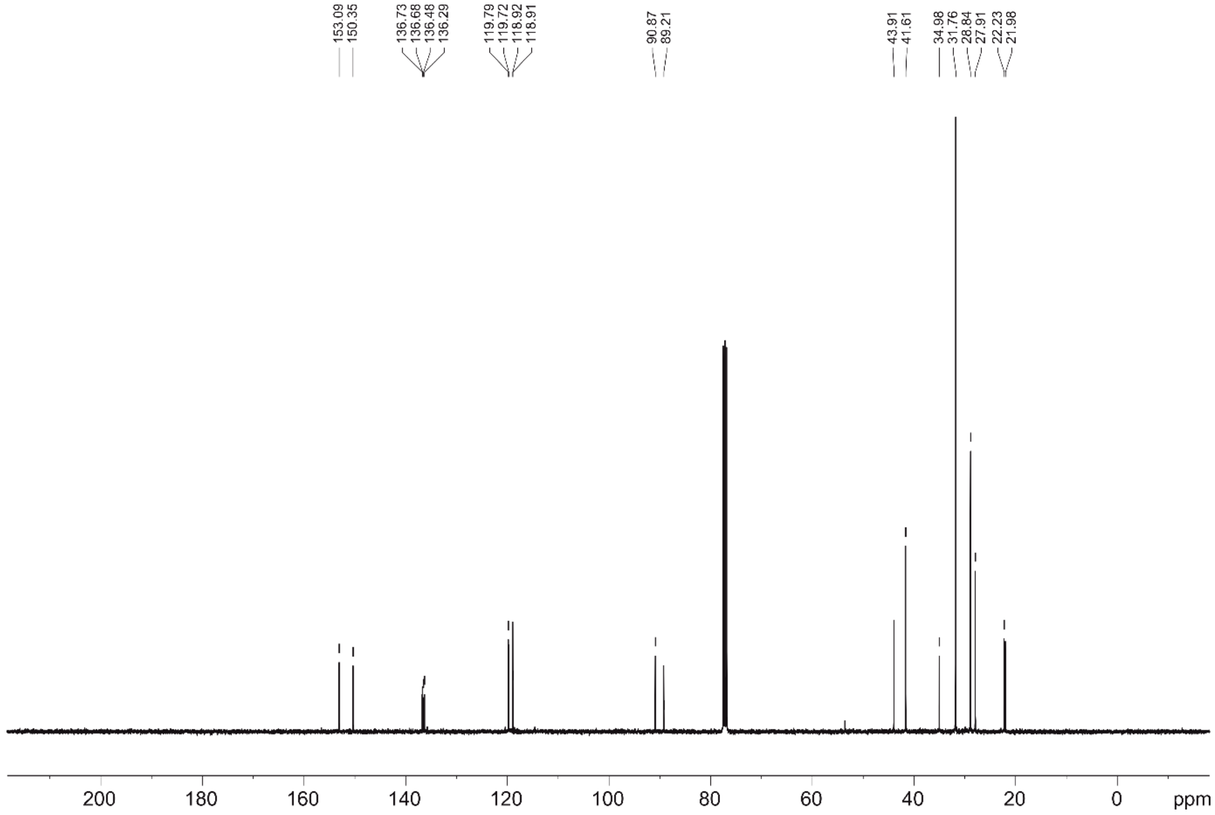

153.09
150.35
136.73
136.68
136.48
136.29
119.79
119.72
118.92
118.91
90.87
89.21
43.91
41.61
34.98
31.76
28.84
27.91
22.23
21.98
200
180
160
140
120
100
80
60
40
20
0
ppm

## (*S*)-1-(4-bromophenyl)-2-fluoropropan-1-one (10a)

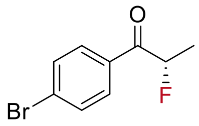


(*S*)-**10a**

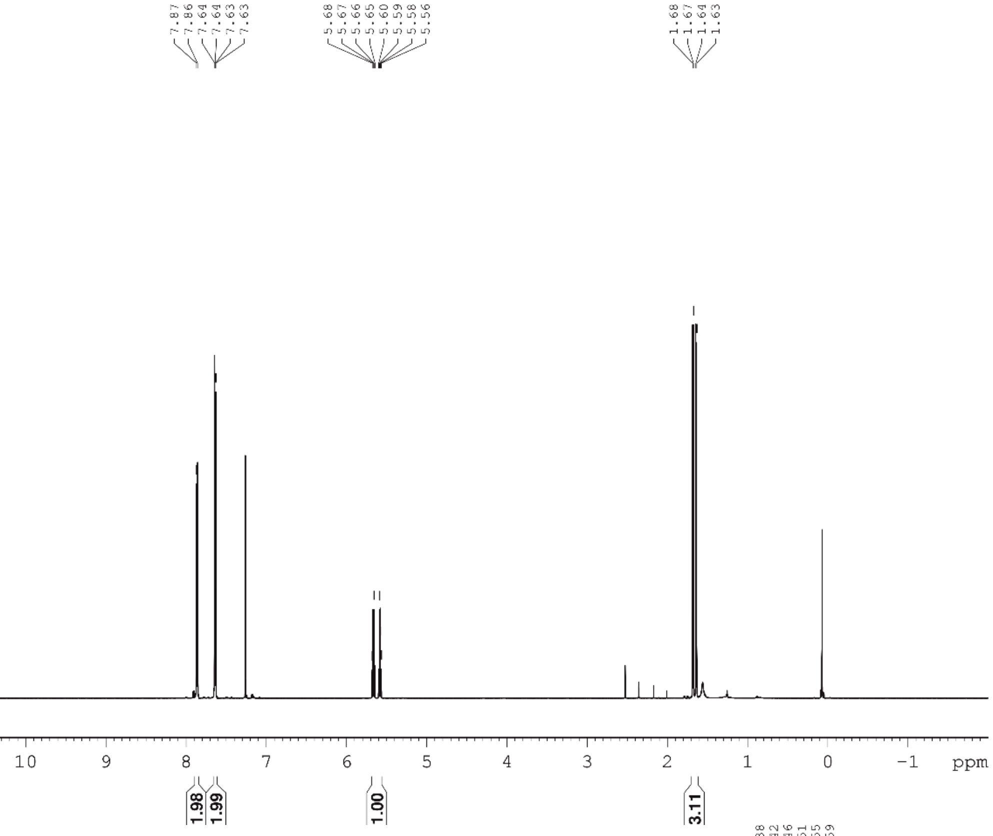


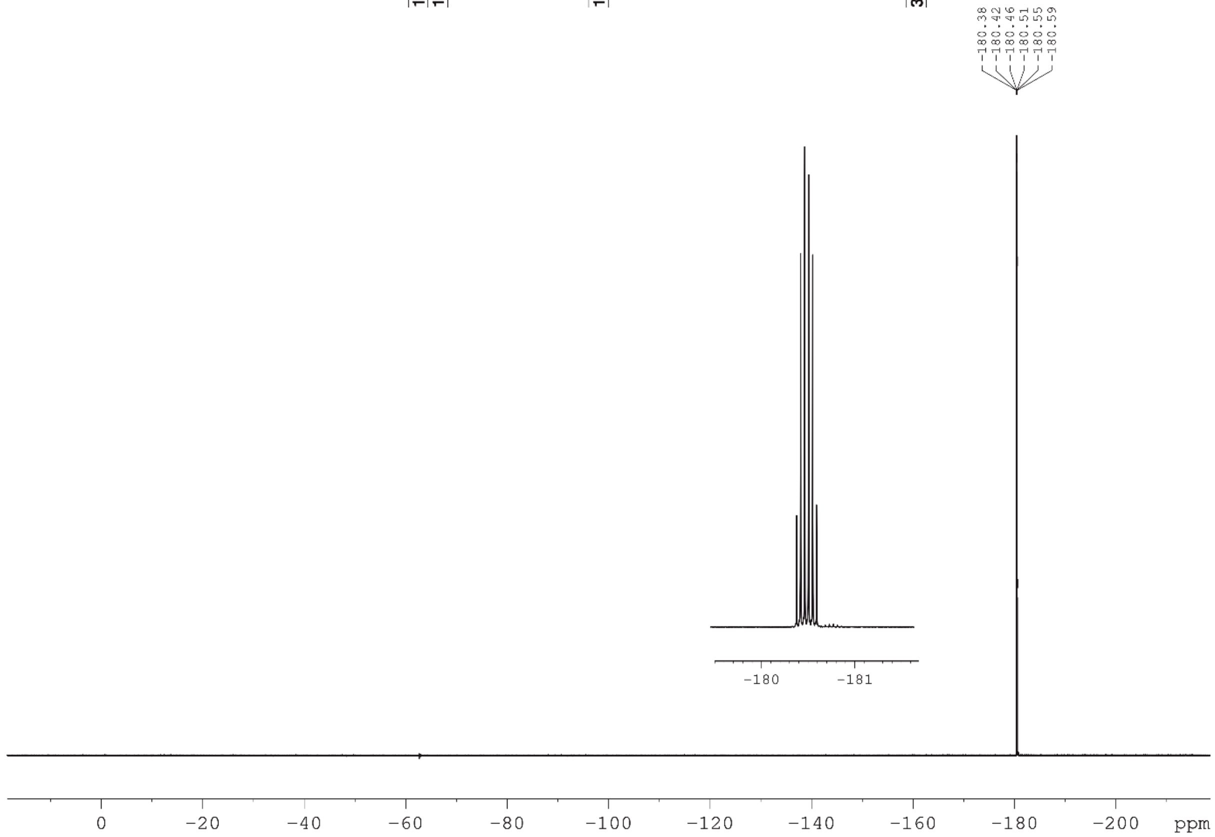

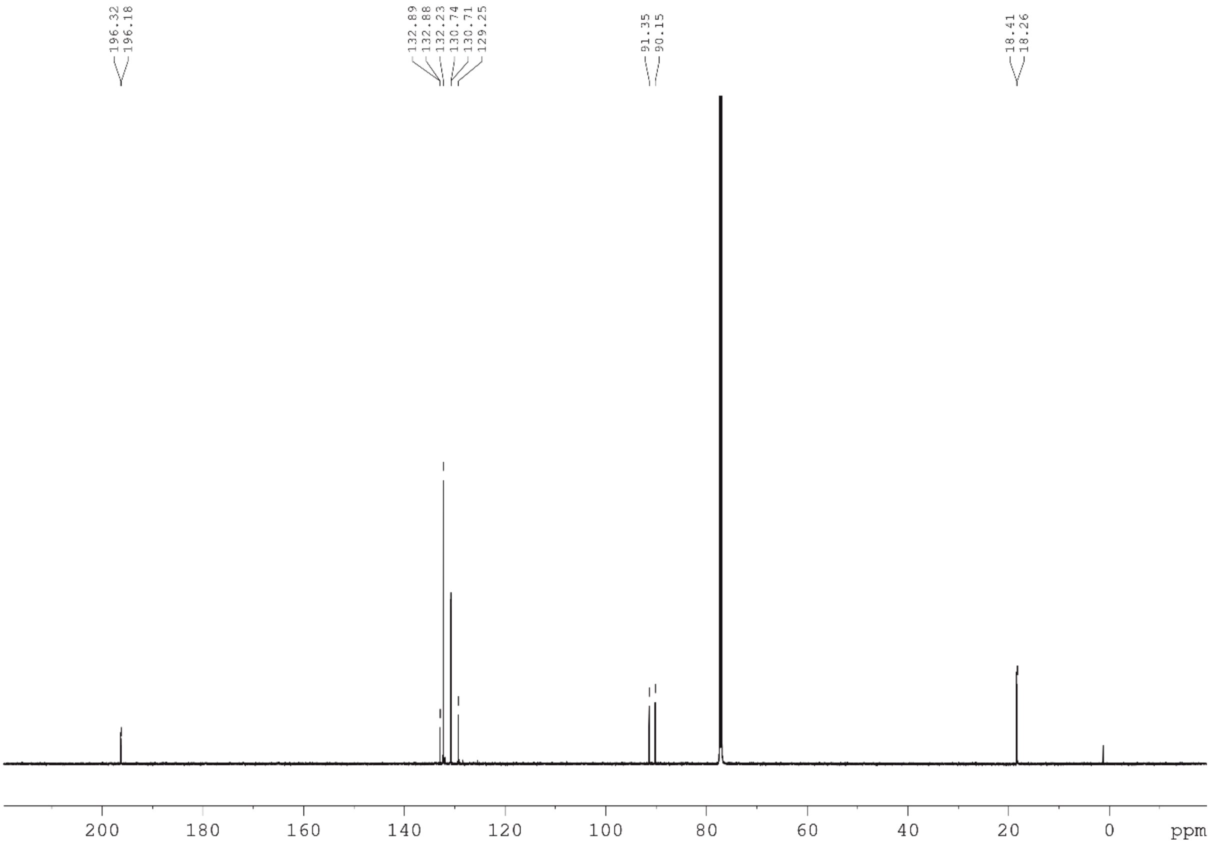


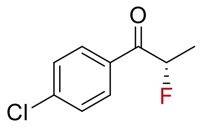

($S$)-**10b**

**($S$)-1-(4-chlorophenyl)-2-fluoropropan-1-one (10b)**

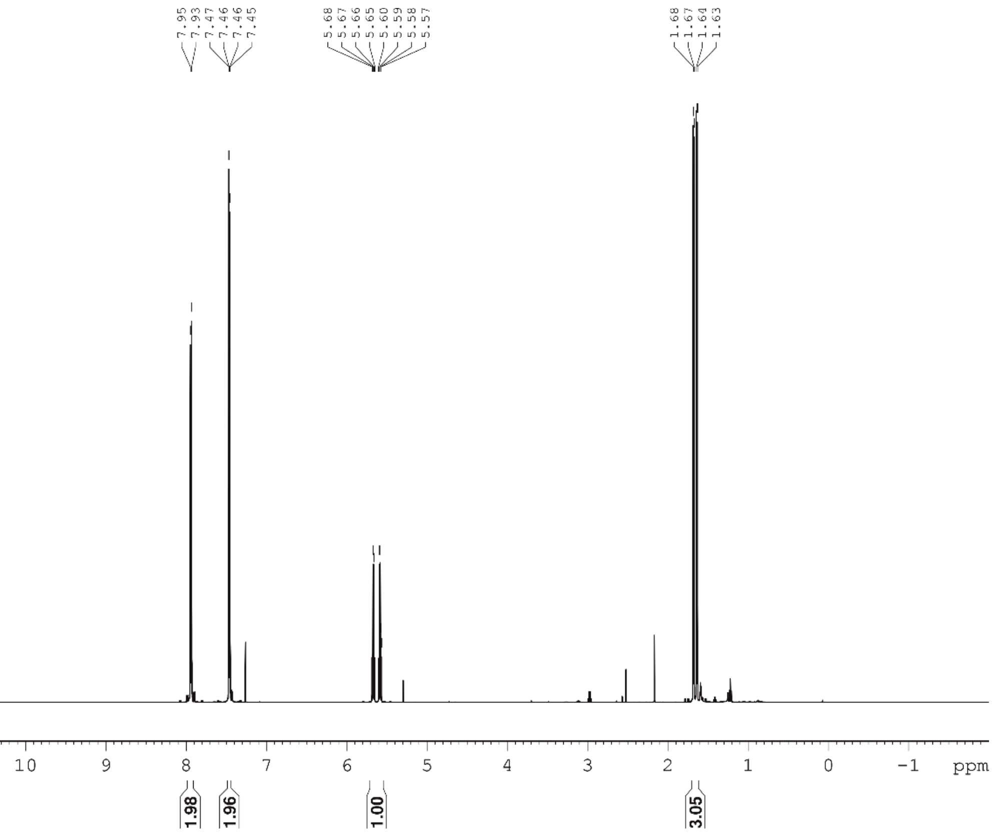

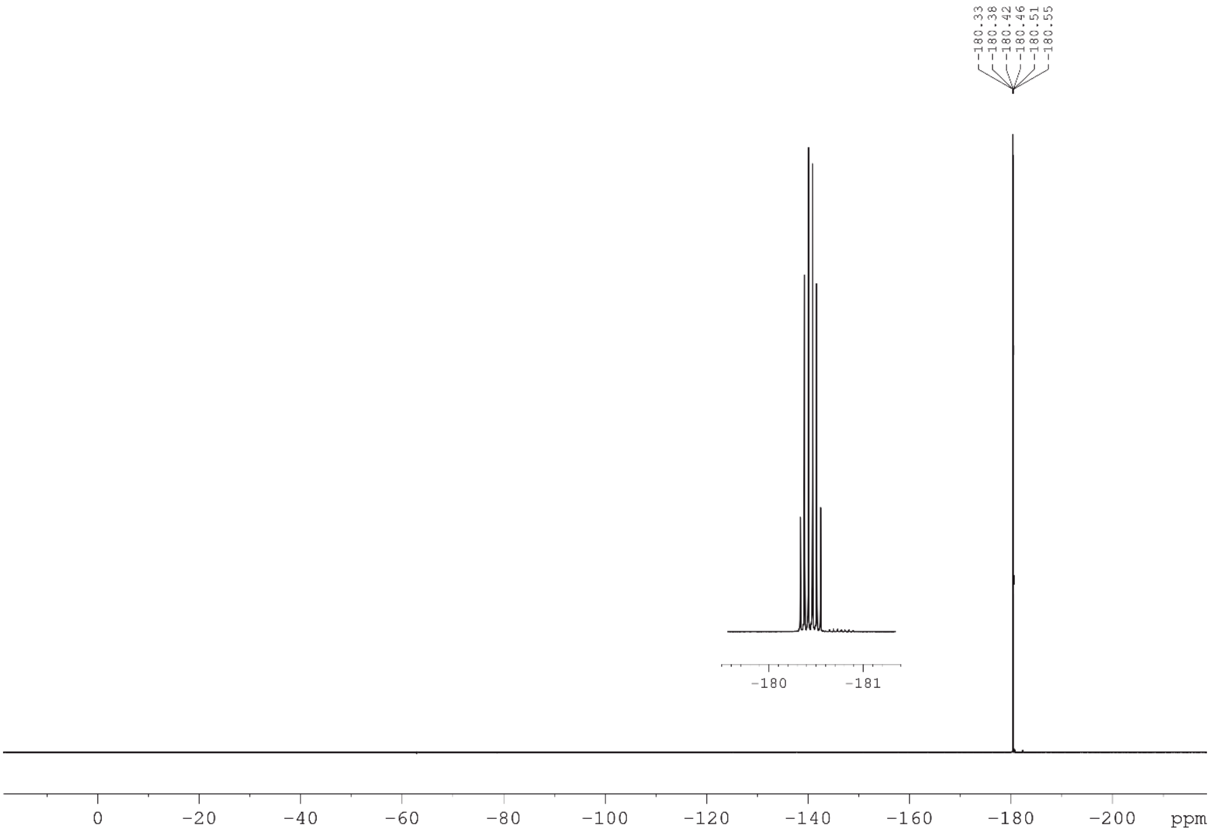
-180.33
-180.38
-180.42
-180.46
-180.51
-180.55
-180
-181
0
-20
-40
-60
-80
-100
-120
-140
-160
-180
-200
ppm

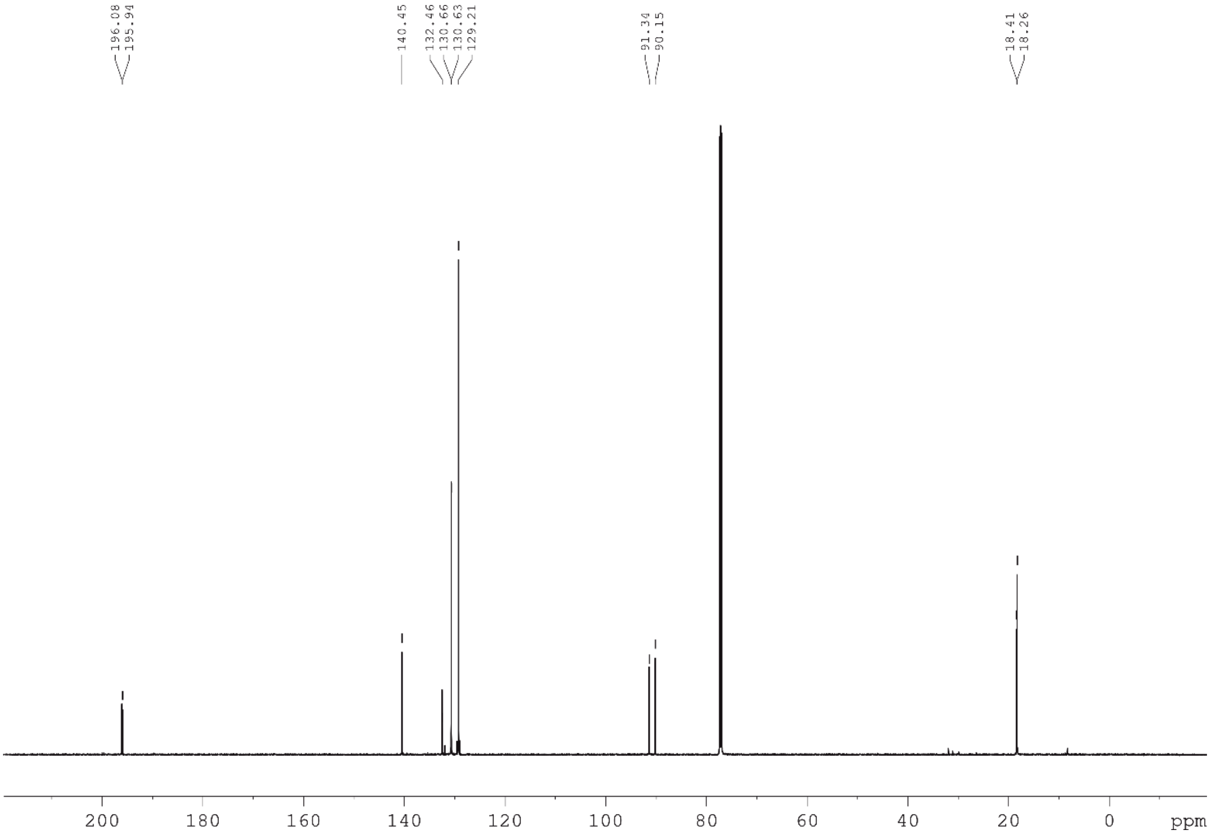
196.08
195.94
140.45
132.46
130.66
130.63
129.21
91.34
90.15
18.41
18.26
200
180
160
140
120
100
80
60
40
20
0
ppm

**(*S*)-2-fluoro-1-(4-fluorophenyl)propan-1-one (10c)**

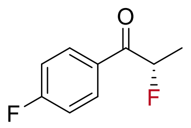


(*S*)-**10c**

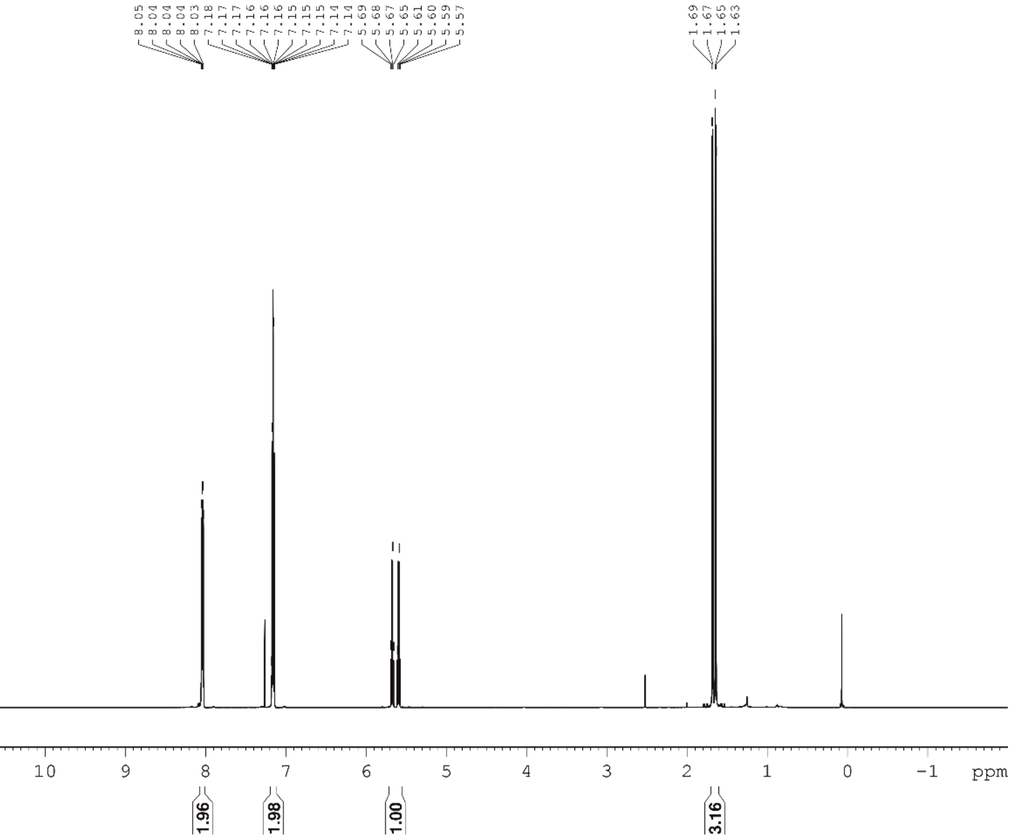


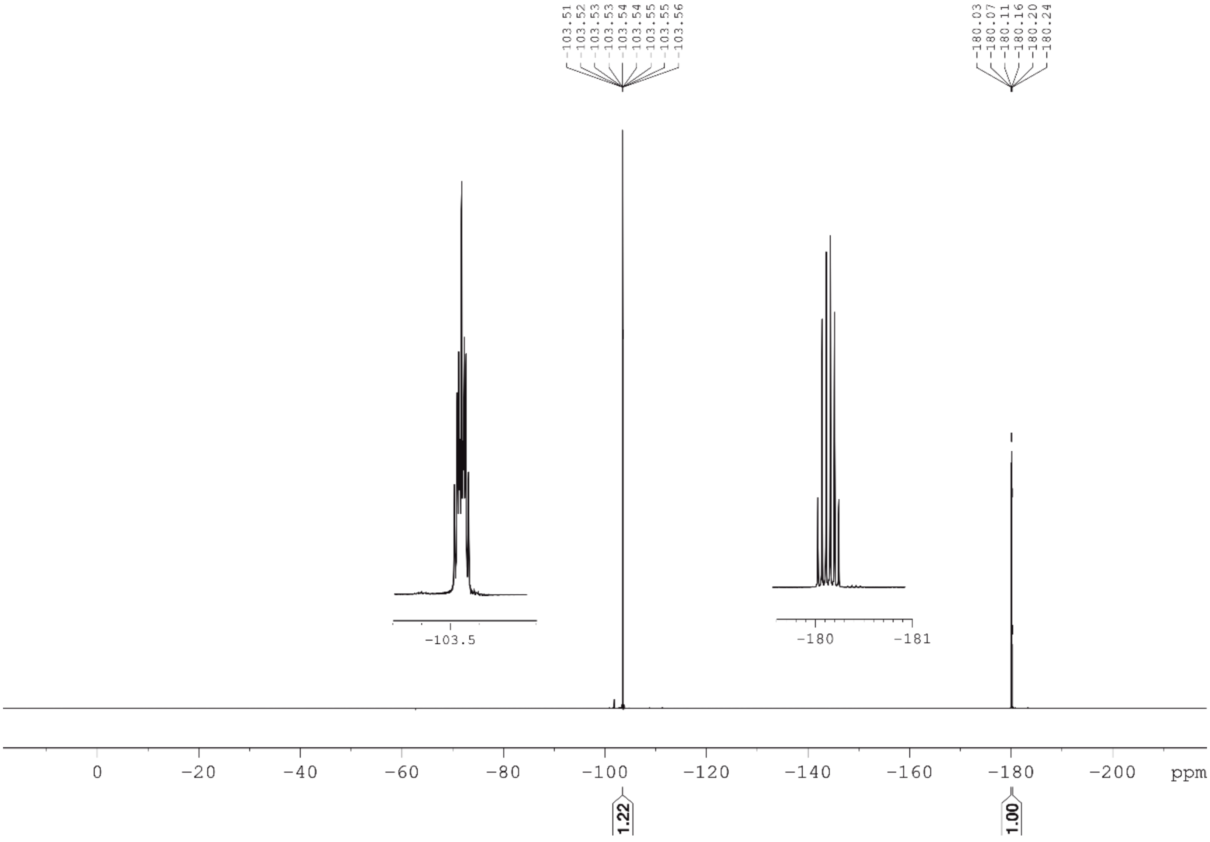

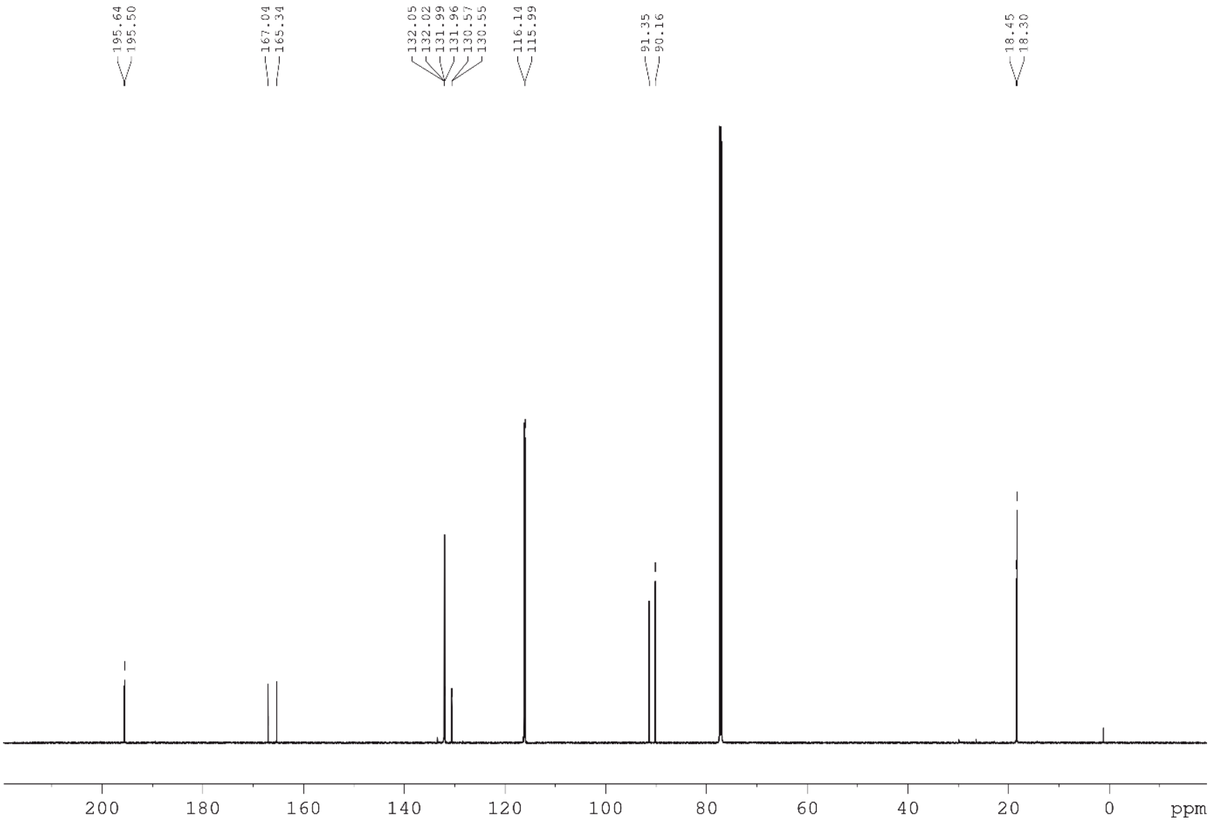


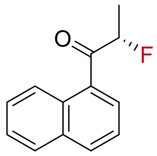


(*S*)-**10d**

**(*S*)-2-fluoro-1-(naphthalen-1-yl)propan-1-one (10d)**

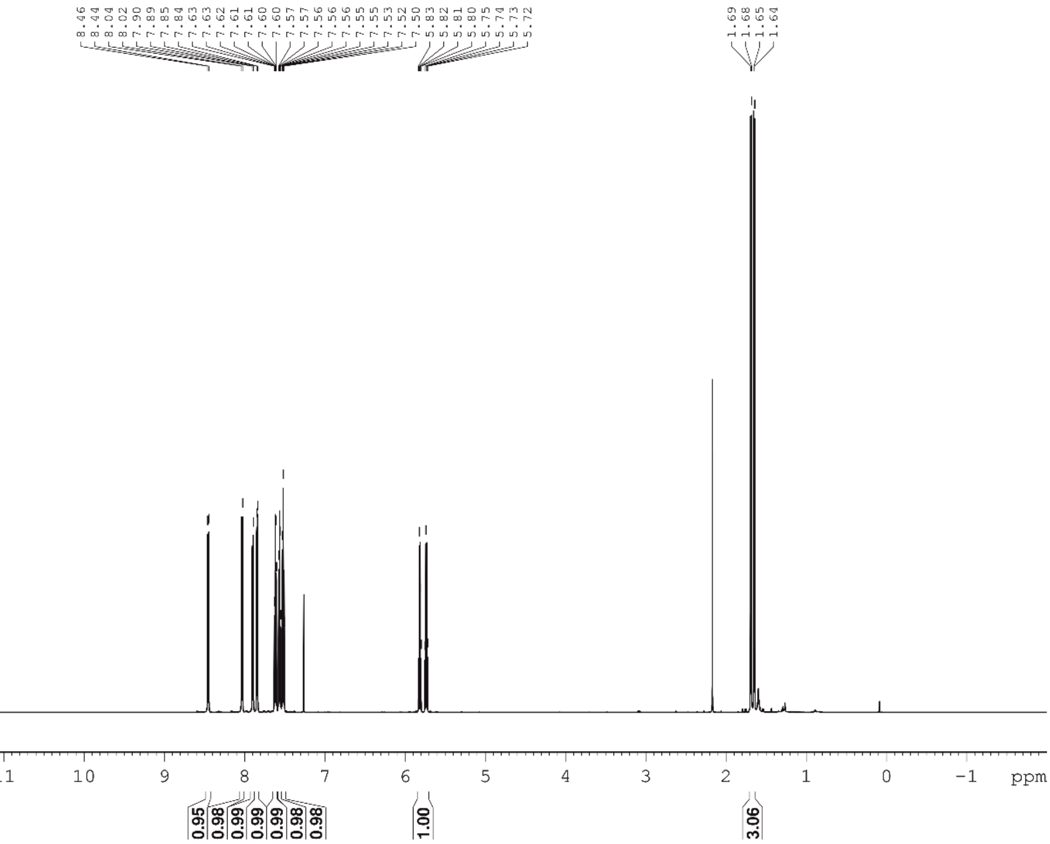

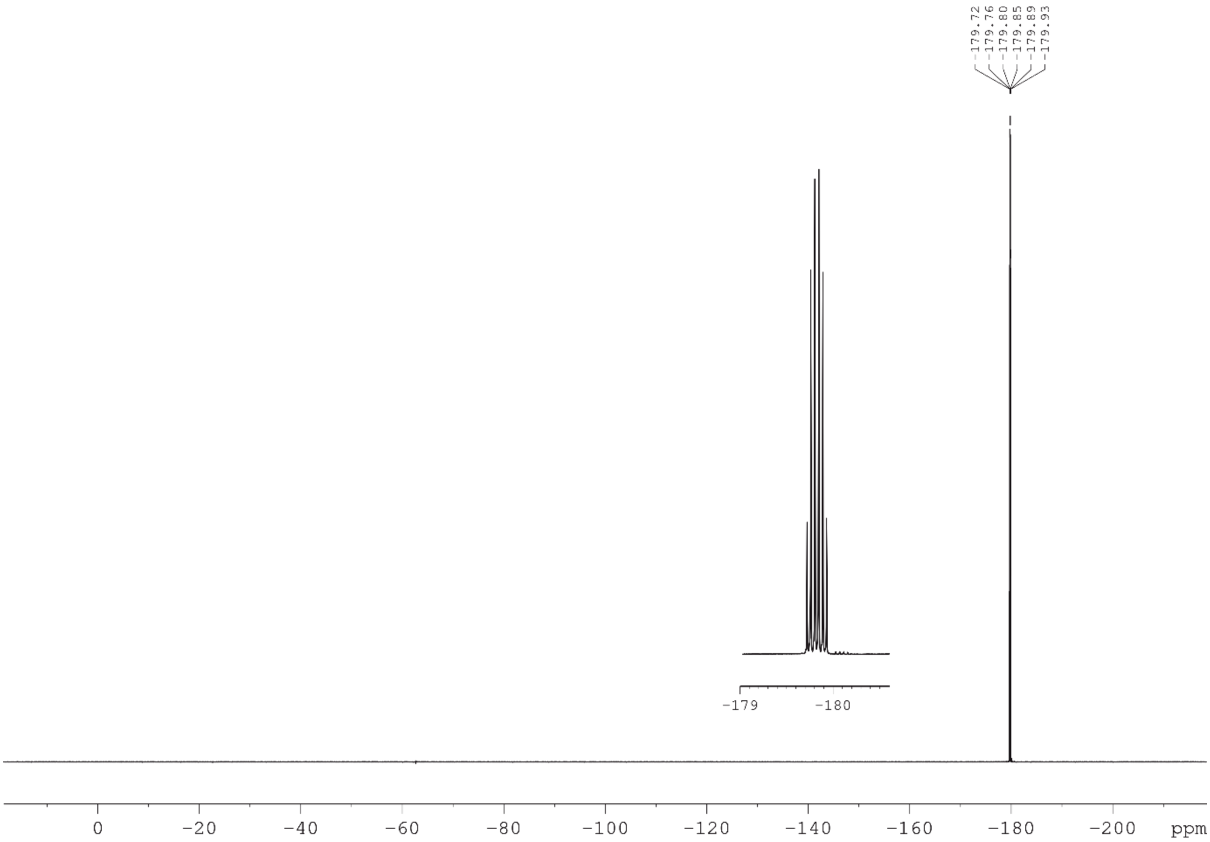

-179.72
-179.76
-179.80
-179.85
-179.89
-179.93
-179 -180
0 -20 -40 -60 -80 -100 -120 -140 -160 -180 -200 ppm


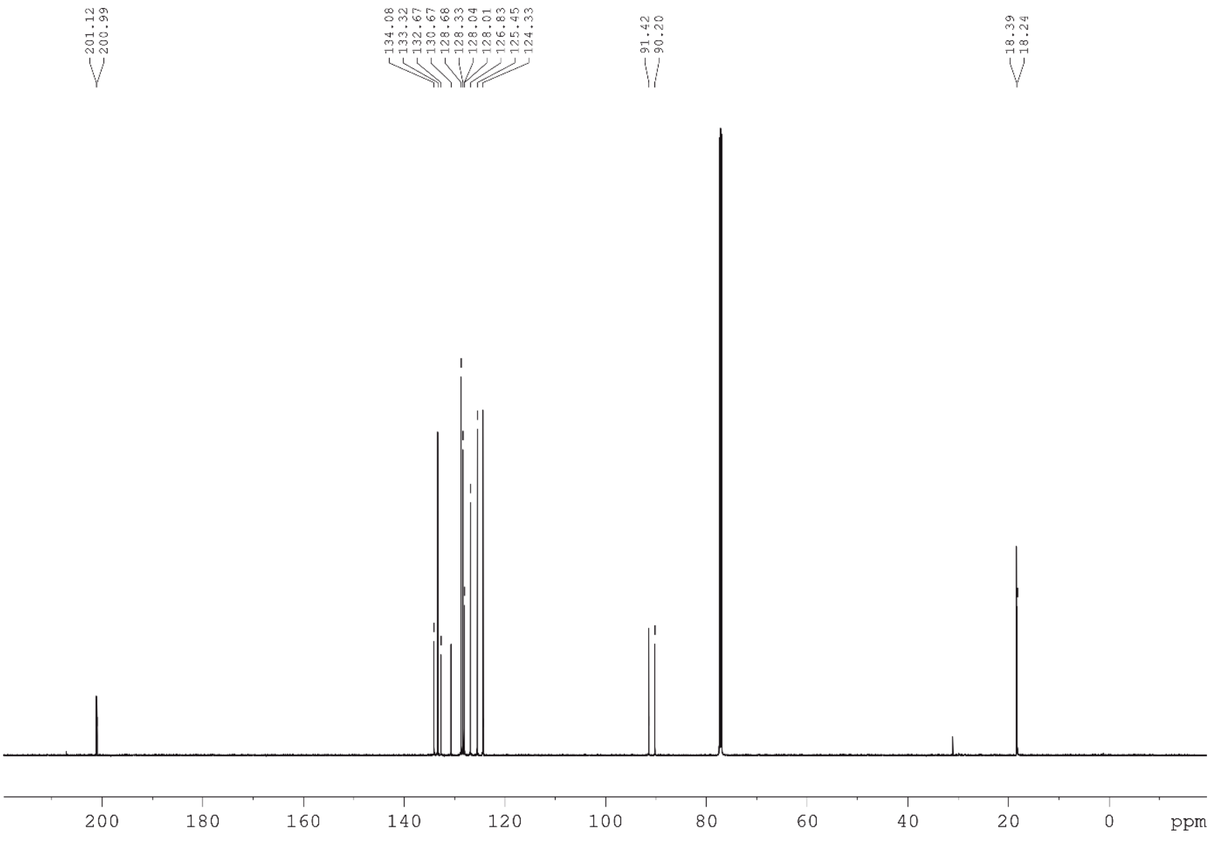

201.12
200.99
134.08
133.32
132.67
130.67
128.68
128.33
128.04
128.01
126.83
125.45
124.33
91.42
90.20
18.39
18.24
200 180 160 140 120 100 80 60 40 20 0 ppm

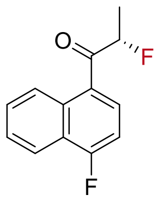


(*S*)-**10e**

**(*S*)-2-fluoro-1-(4-fluoronaphthalen-1-yl)propan-1-one (10e)**

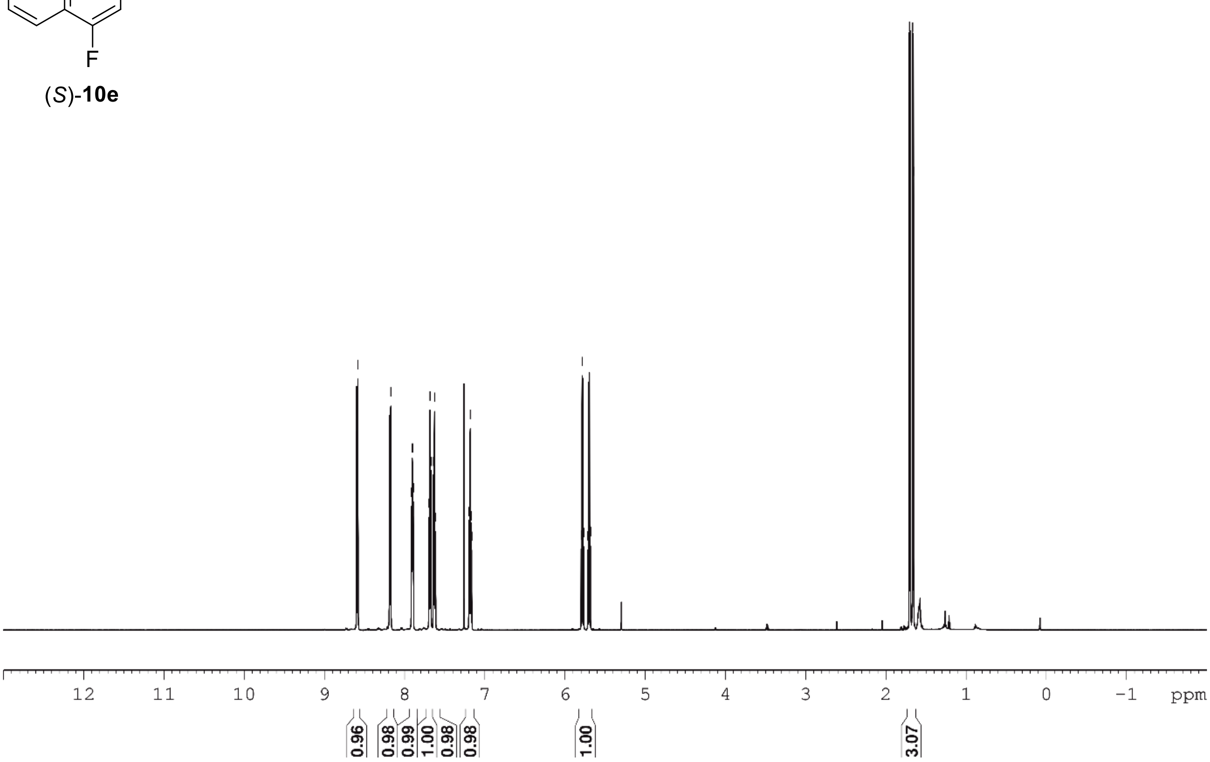


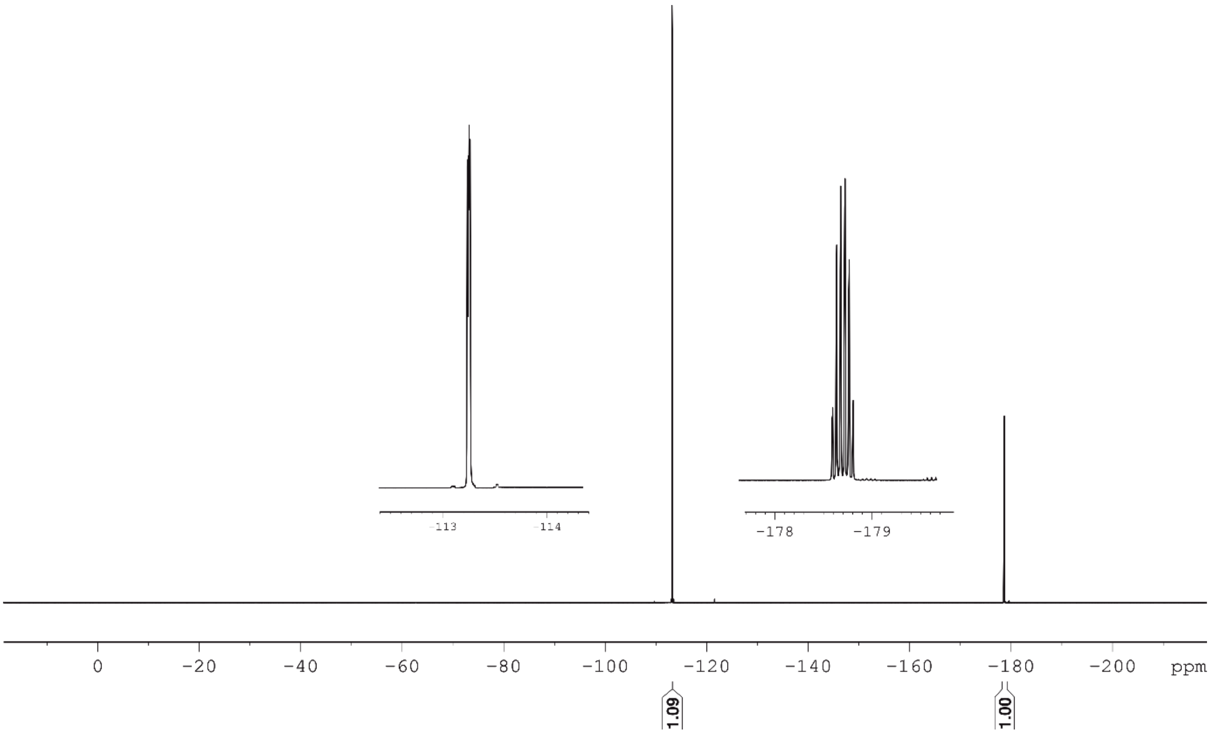

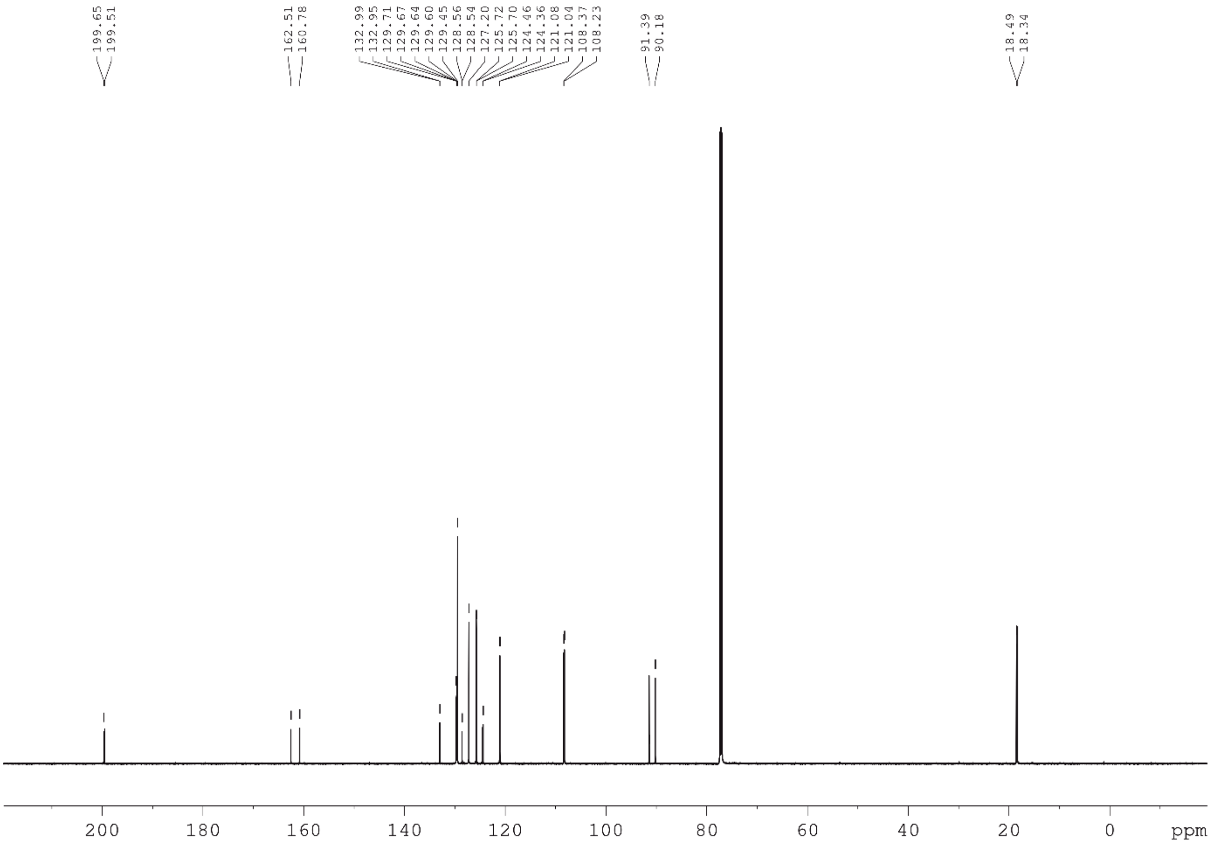

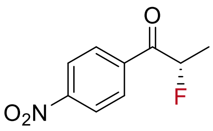

(*S*)-**10f**

**(*S*)-2-fluoro-1-(4-nitrophenyl)propan-1-one (10f)**

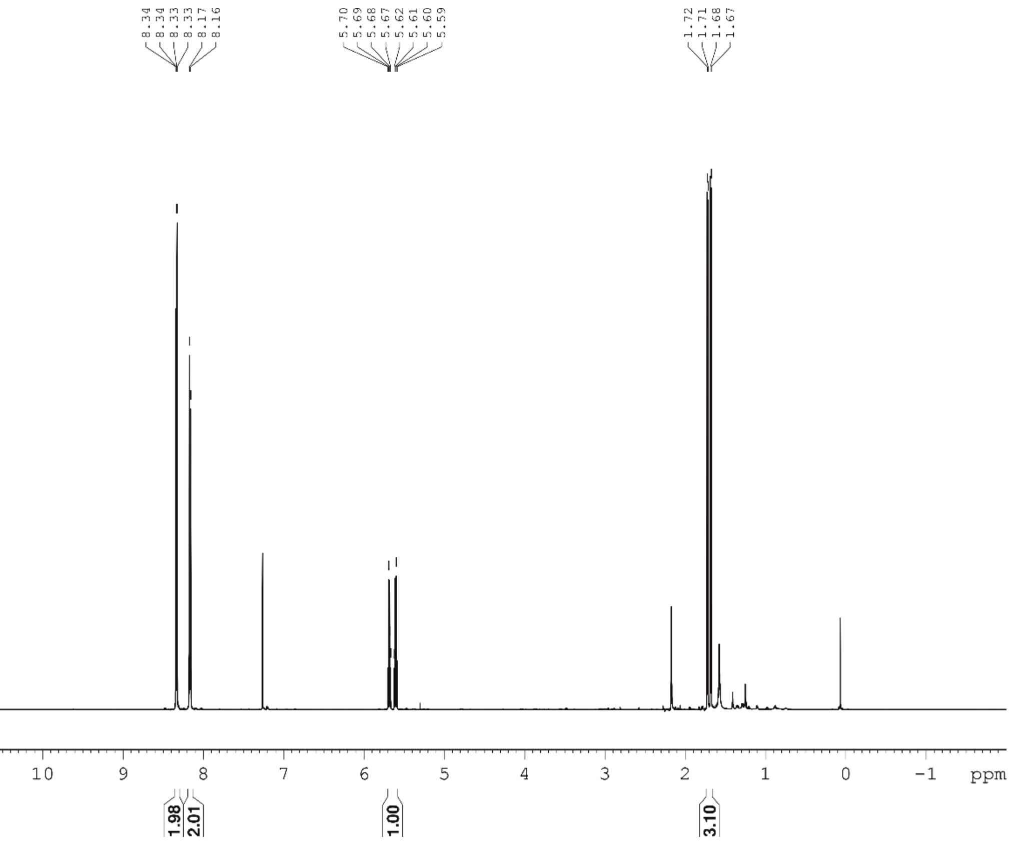

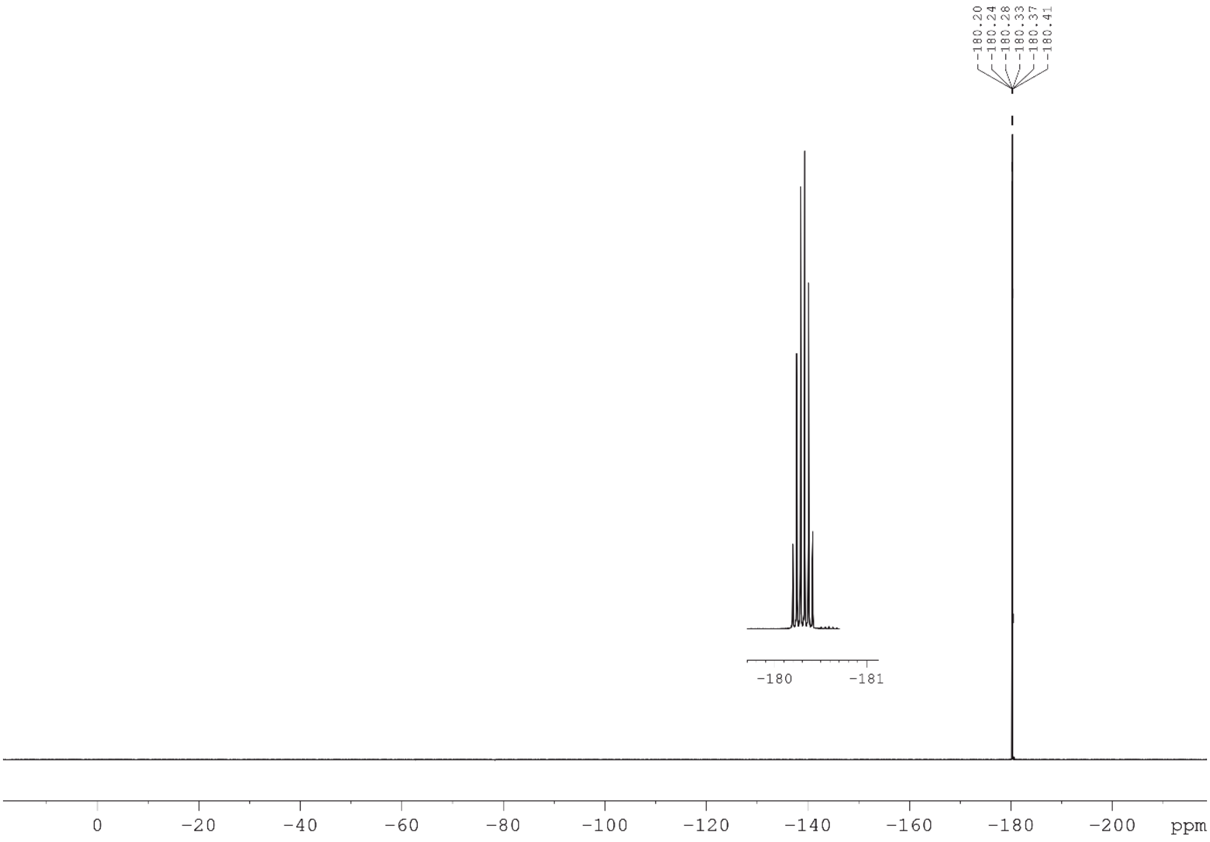

-180.20
-180.24
-180.28
-180.33
-180.37
-180.41
-180
-181
0
-20
-40
-60
-80
-100
-120
-140
-160
-180
-200
ppm


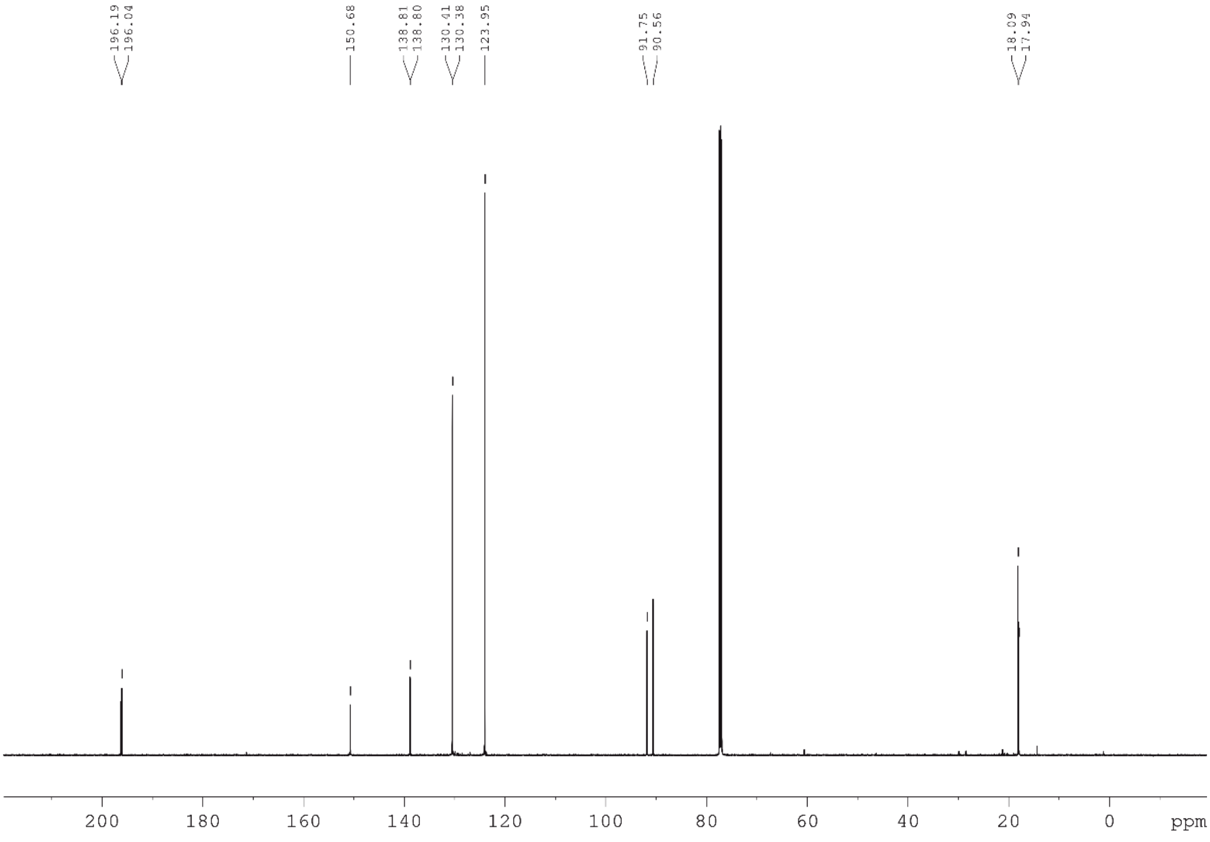

196.19
196.04
150.68
138.81
138.80
130.41
130.38
123.95
91.75
90.56
18.09
17.94
200
180
160
140
120
100
80
60
40
20
0
ppm

**(*S*)-4-(2-fluoropropanoyl)benzonitrile (10g)**

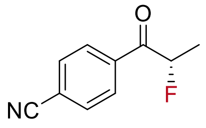


(*S*)-**10g**

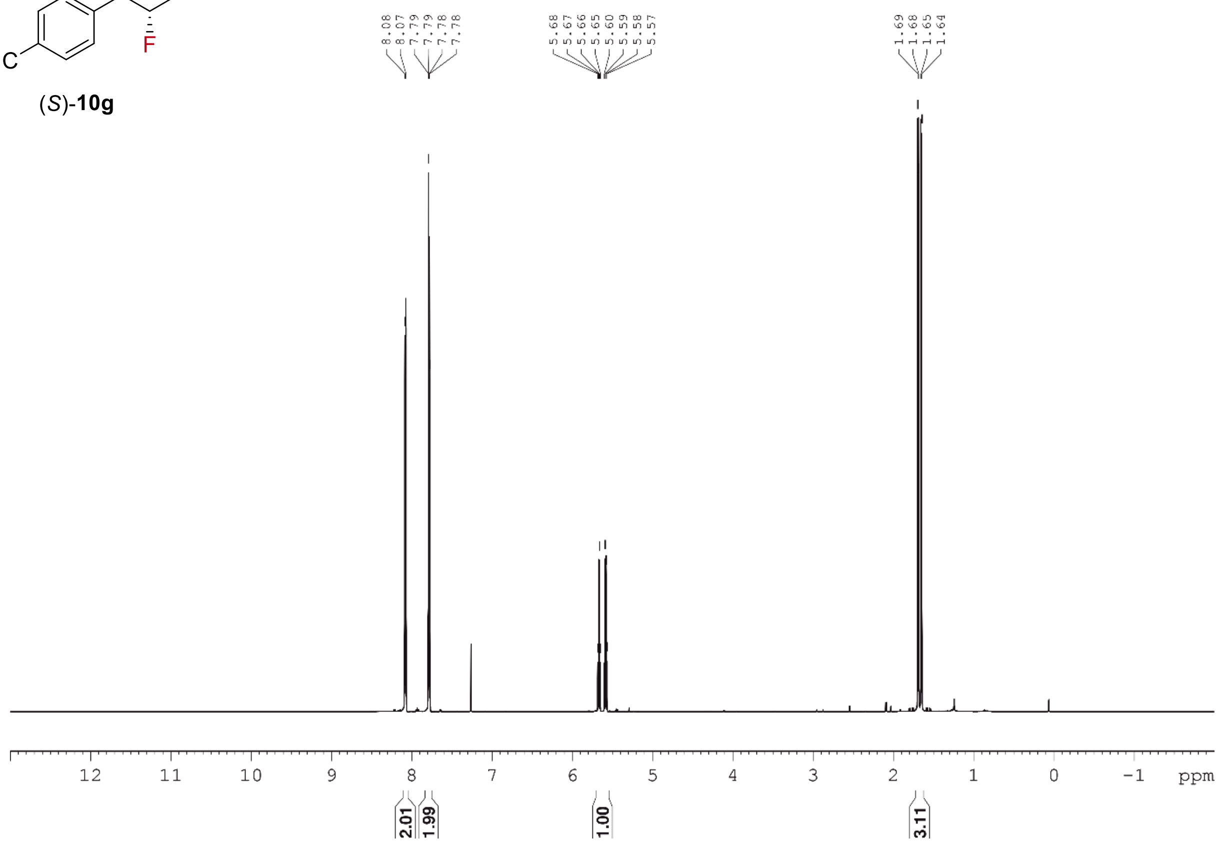


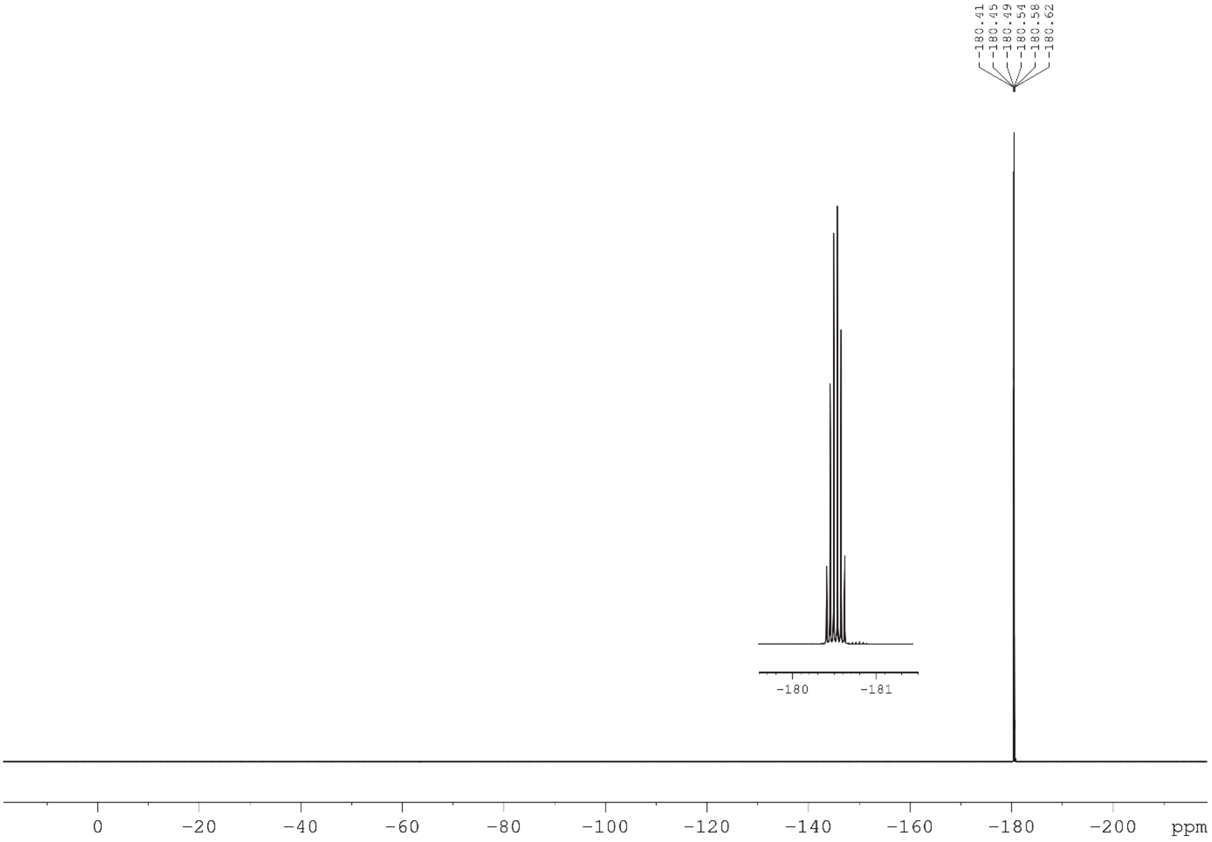

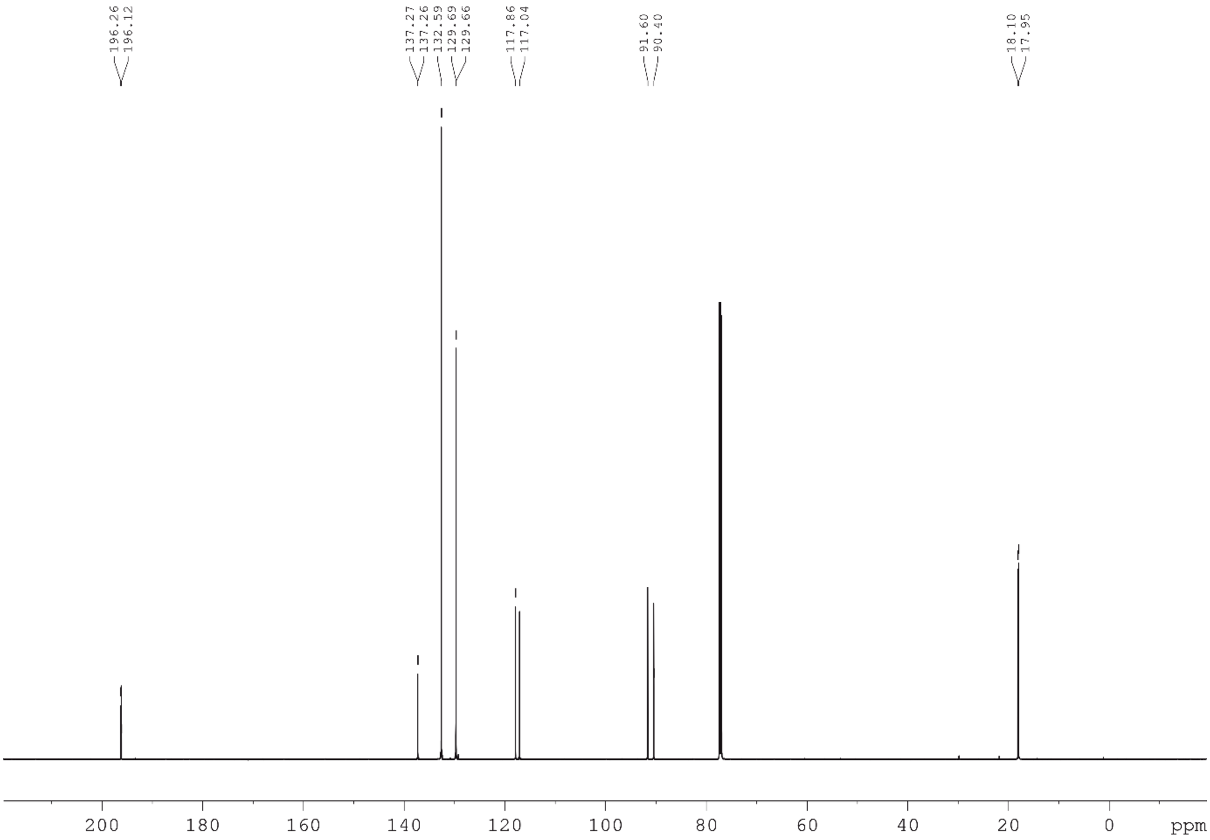


## (*S*)-2-fluoro-1-(4-(trifluoromethyl)phenyl)propan-1-one (10h)

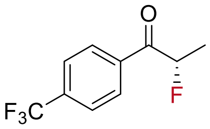


(*S*)-**10h**

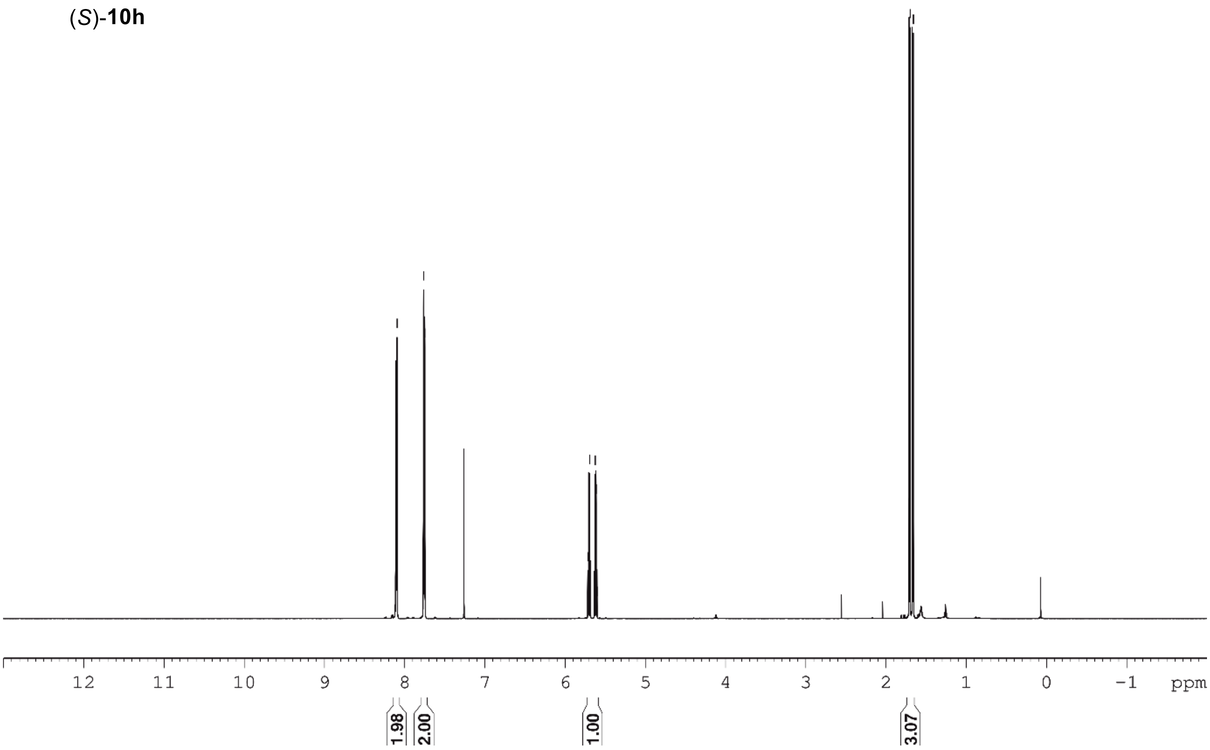

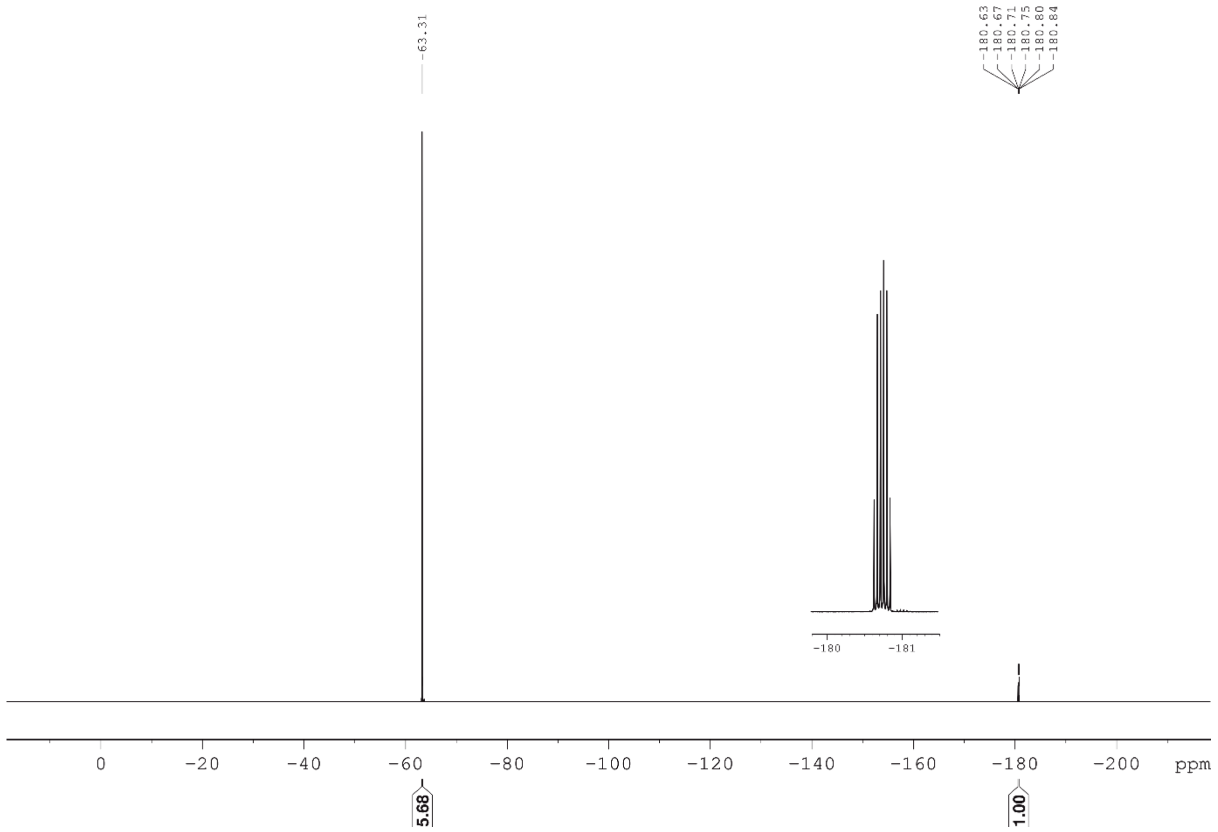

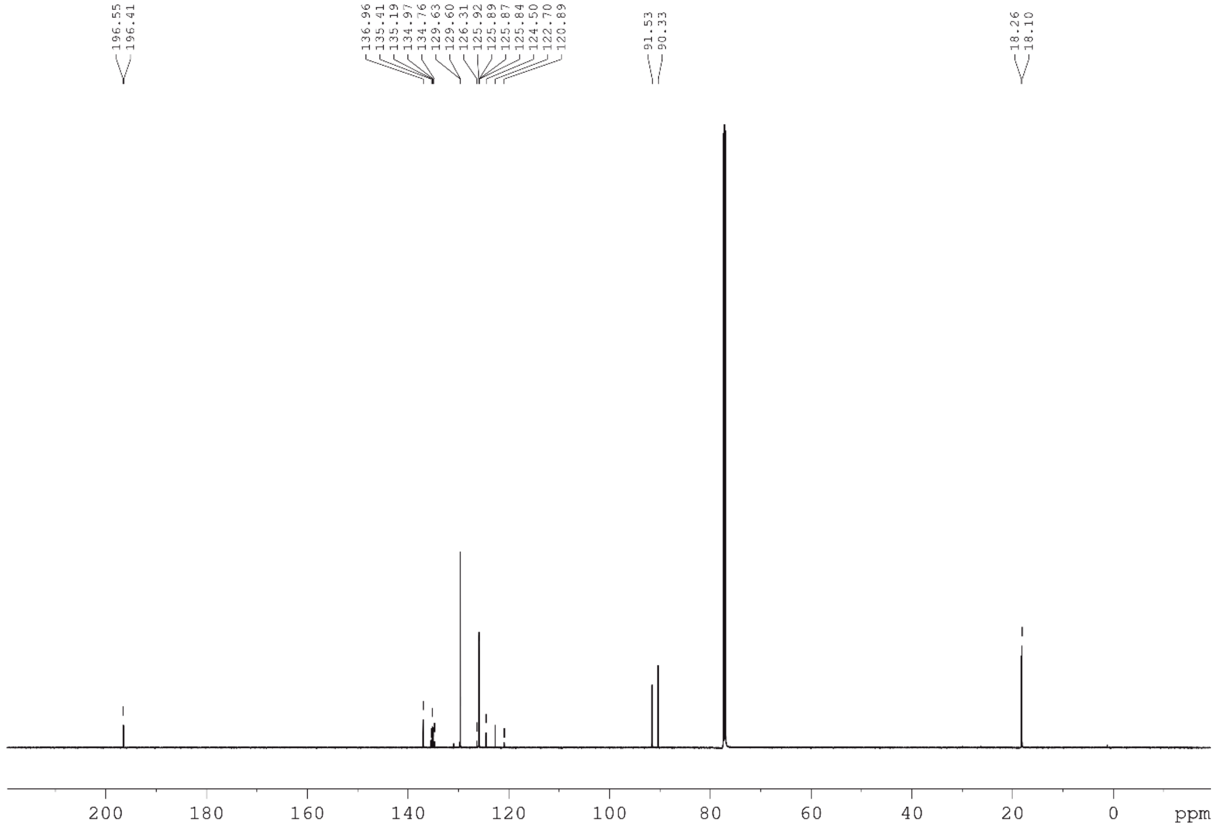

# (*S*)-2-fluoro-1-phenylpropan-1-one (10i)

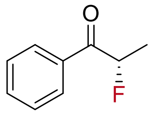


(*S*)-**10i**

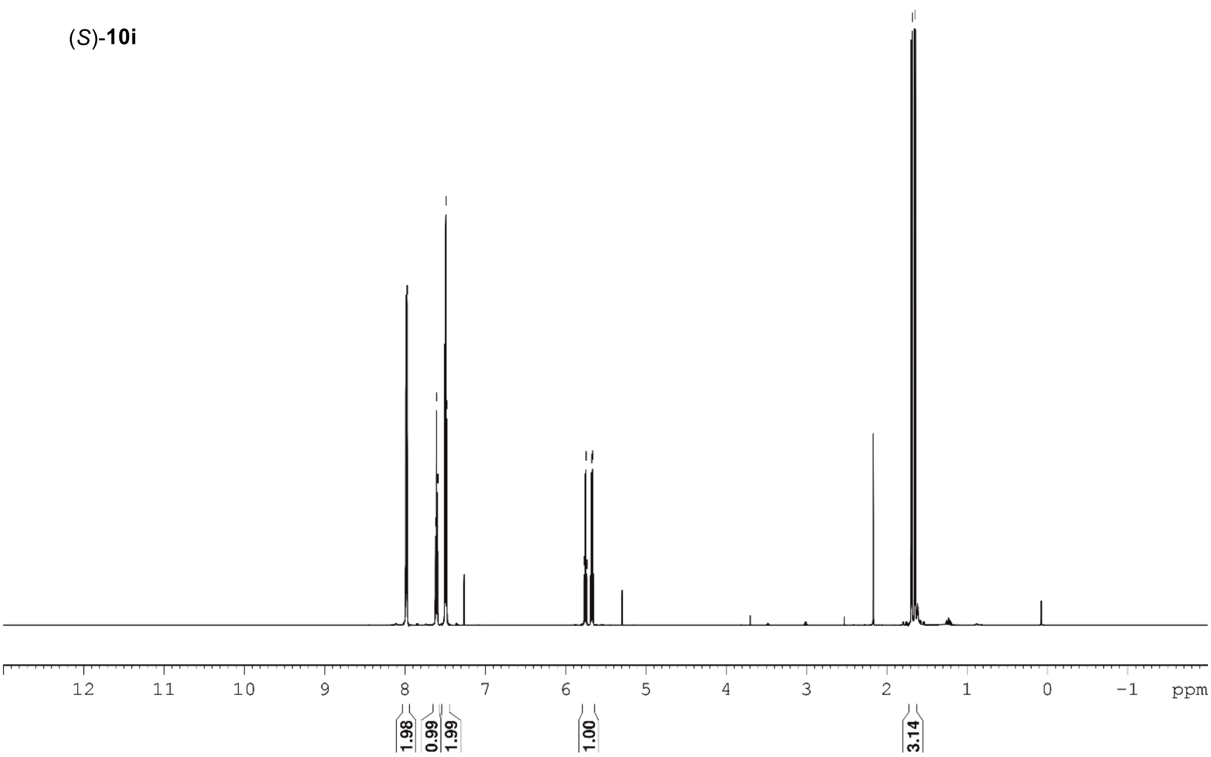


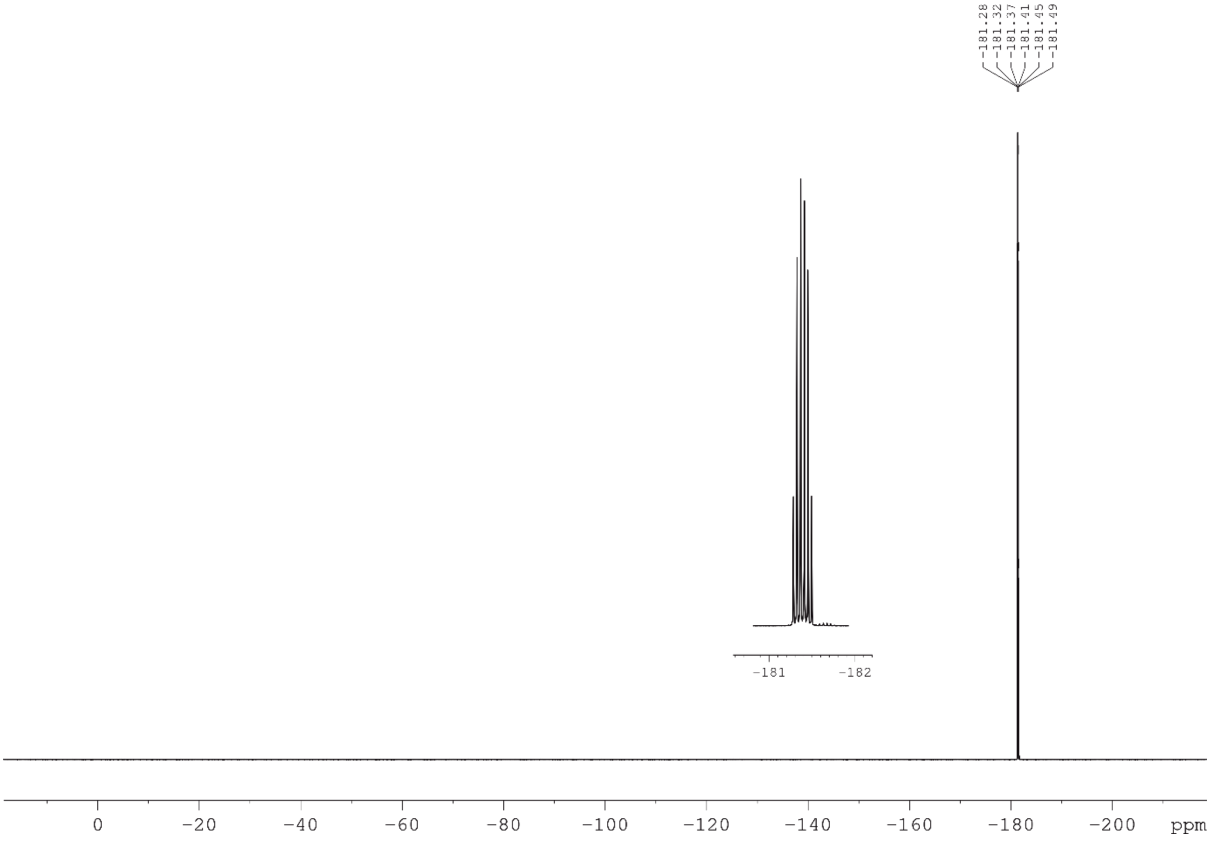

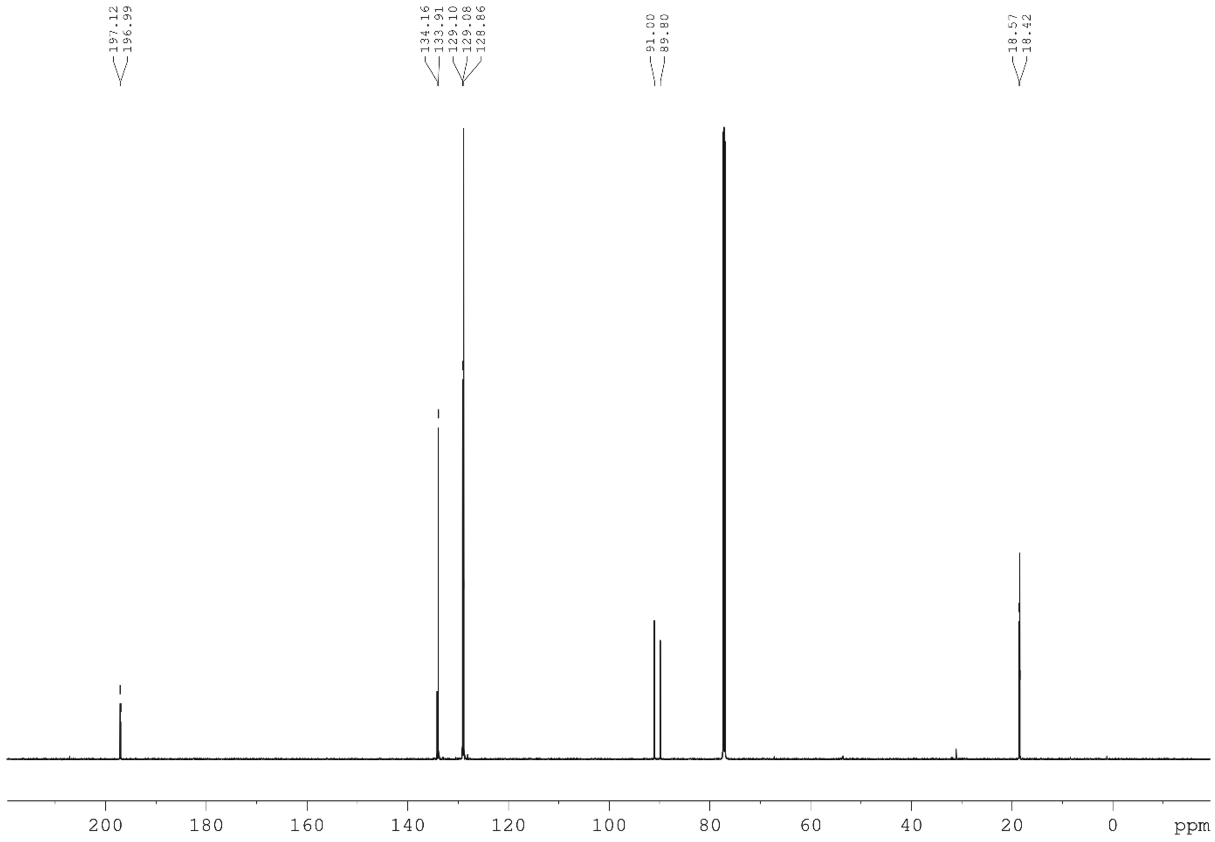


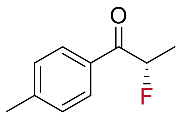


(*S*)-**10j**

**(*S*)-2-fluoro-1-(p-tolyl)propan-1-one (10j)**

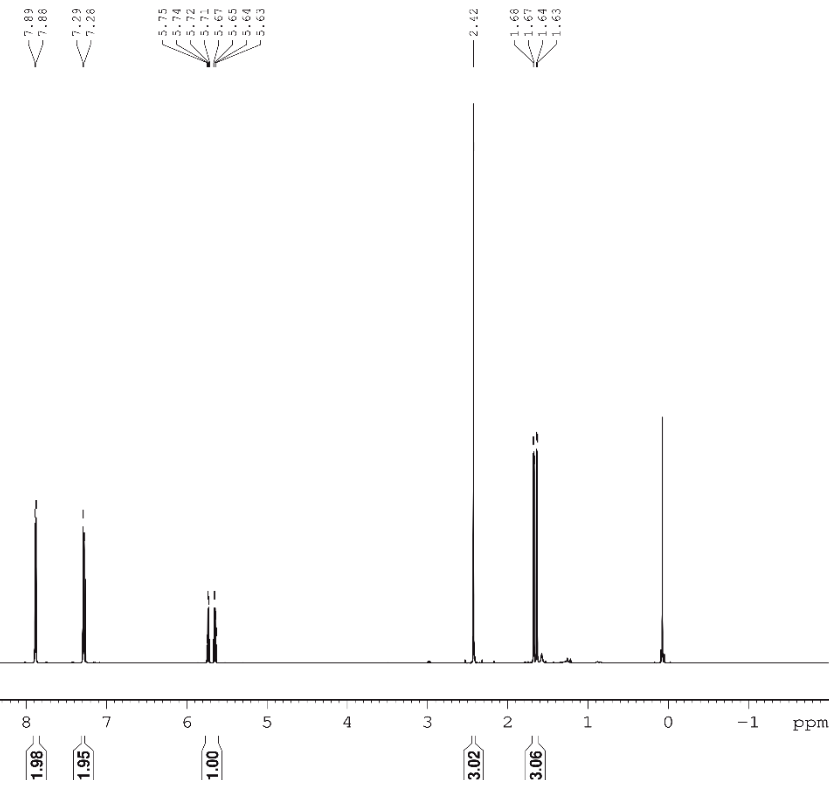

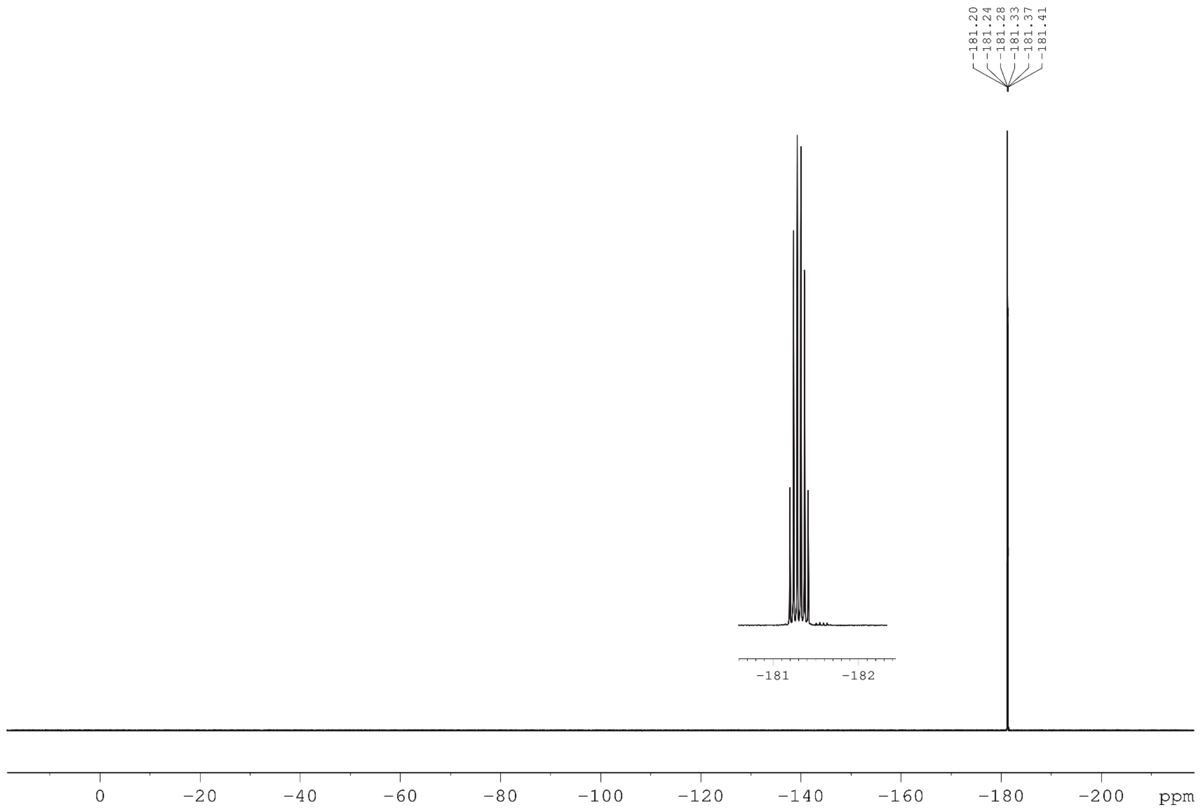
-181.20
-181.24
-181.28
-181.33
-181.37
-181.41
-181
-182
0
-20
-40
-60
-80
-100
-120
-140
-160
-180
-200
ppm

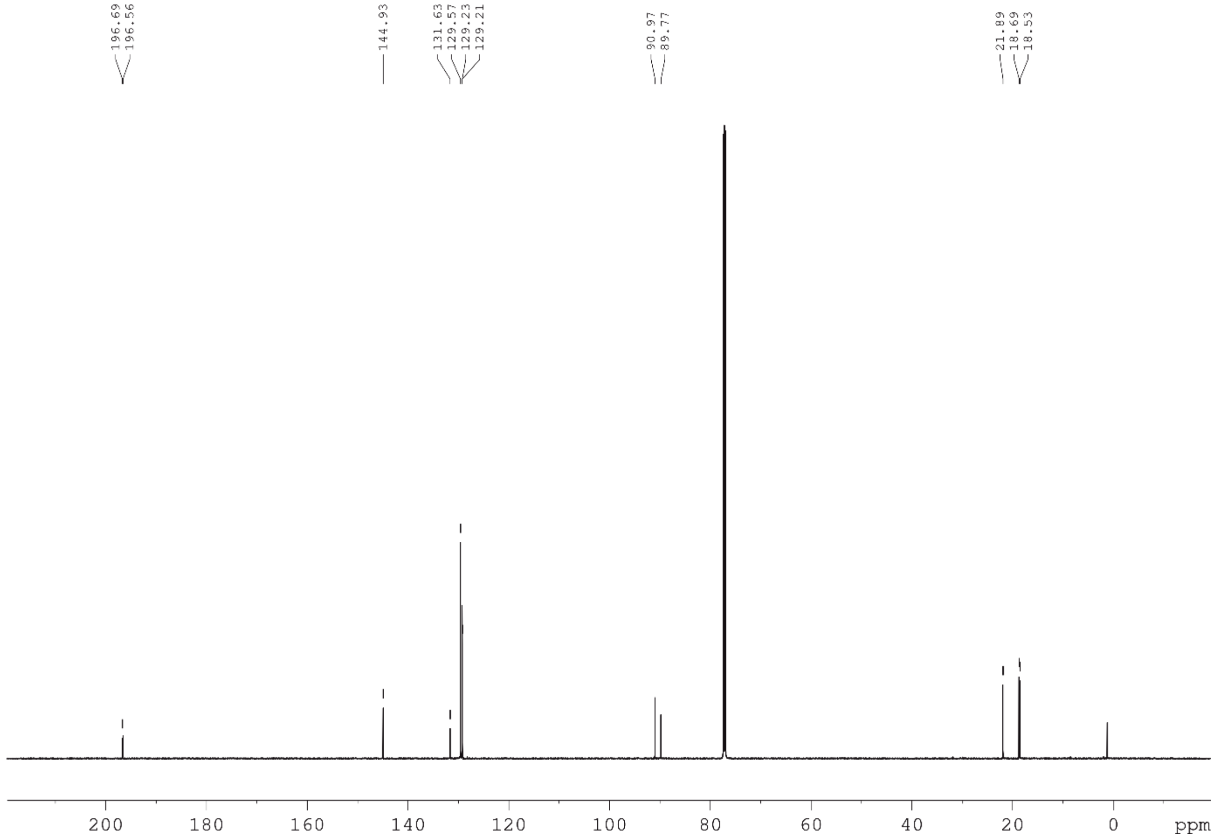
196.69
196.56
144.93
131.63
129.57
129.23
129.21
90.97
89.77
21.89
18.69
18.53
200
180
160
140
120
100
80
60
40
20
0
ppm

## (*S*)-2-fluoro-1-(4-methoxyphenyl)propan-1-one (10k)

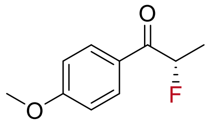

(*S*)-**10k**

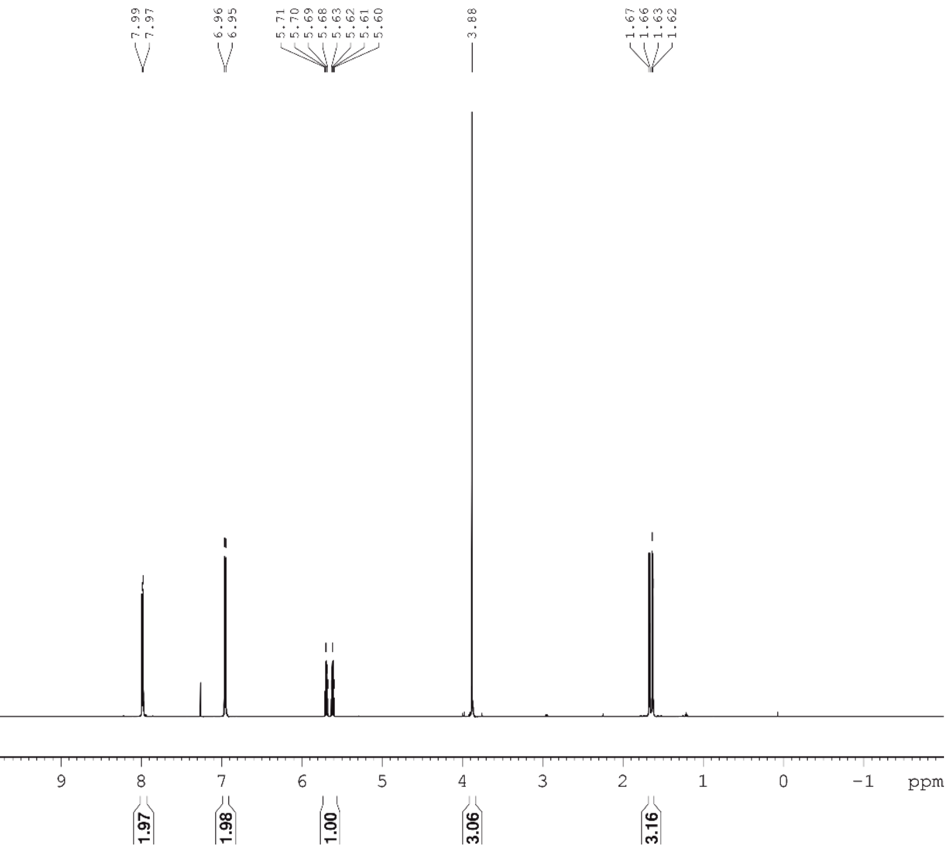


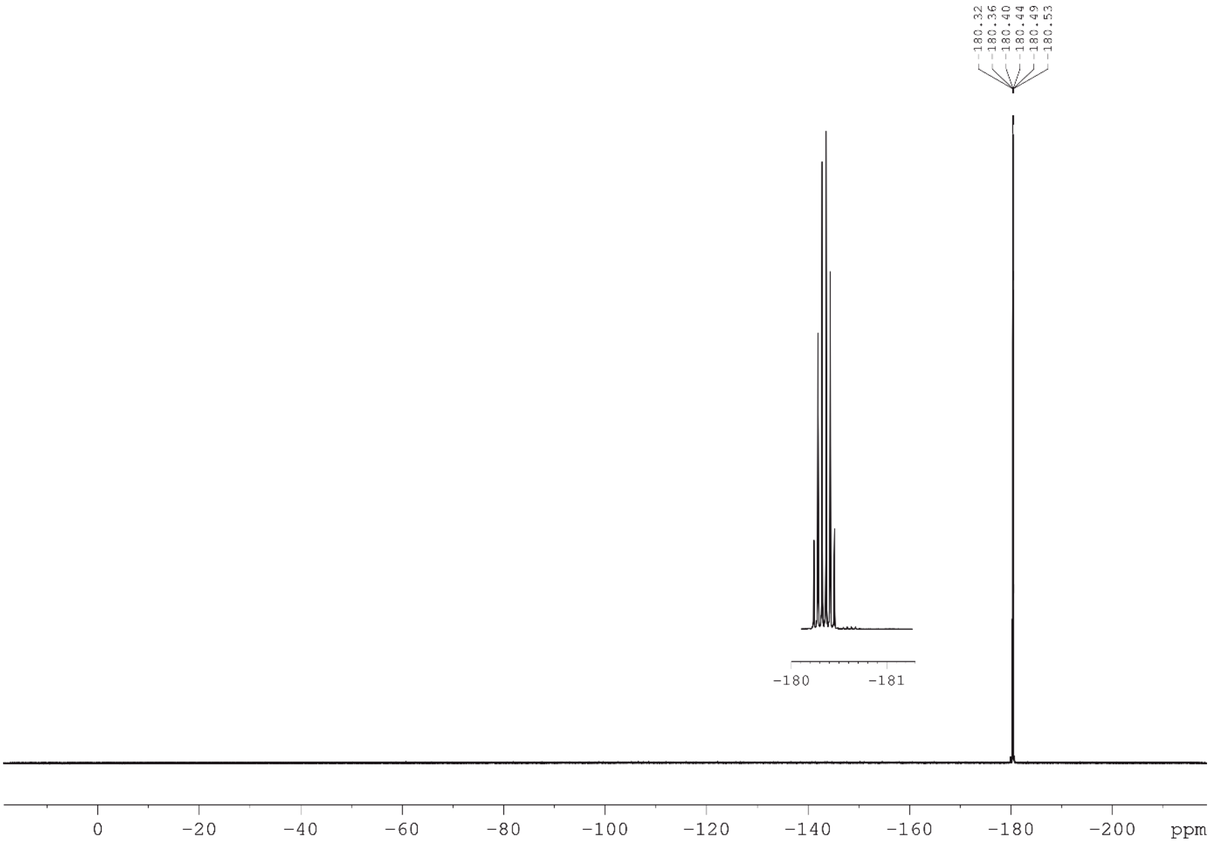

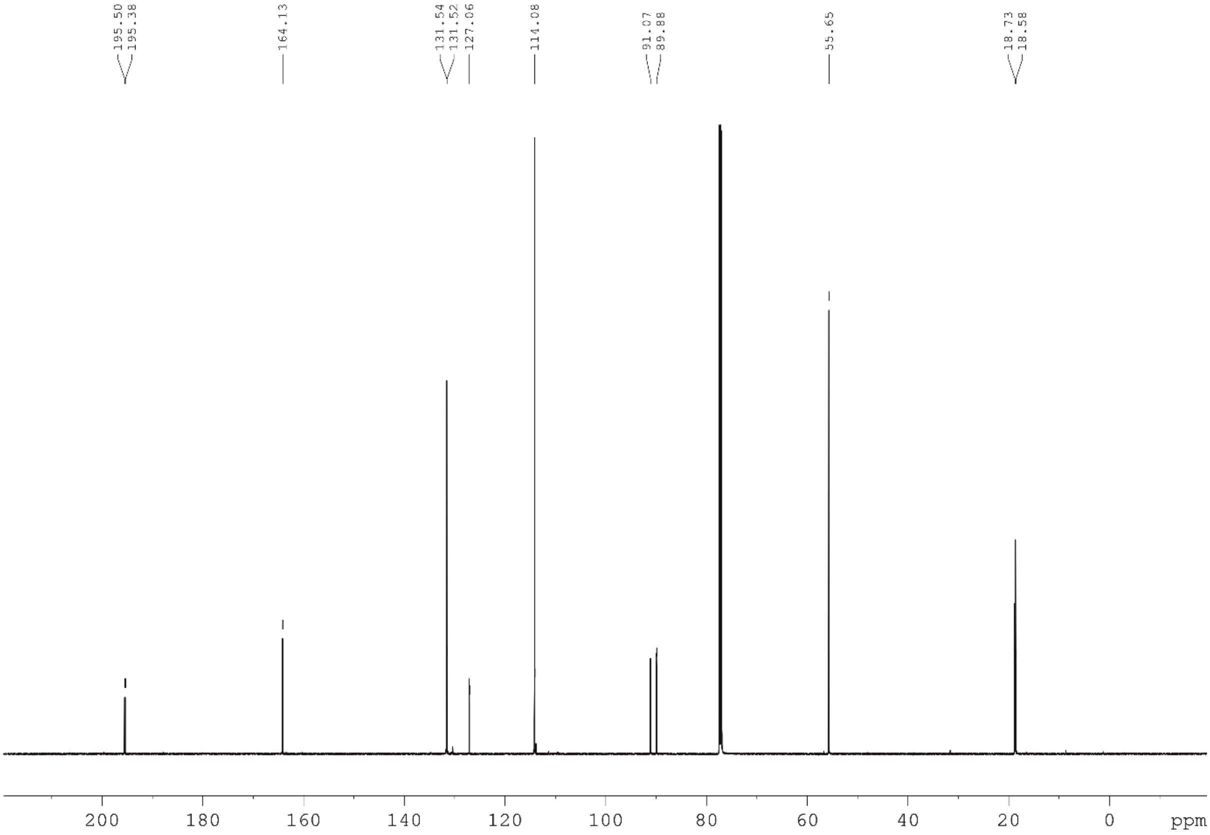


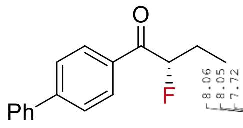


**(*S*)-1-([1,1'-biphenyl]-4-yl)-2-fluorobutan-1-one (10l)**

(*S*)-**10l**

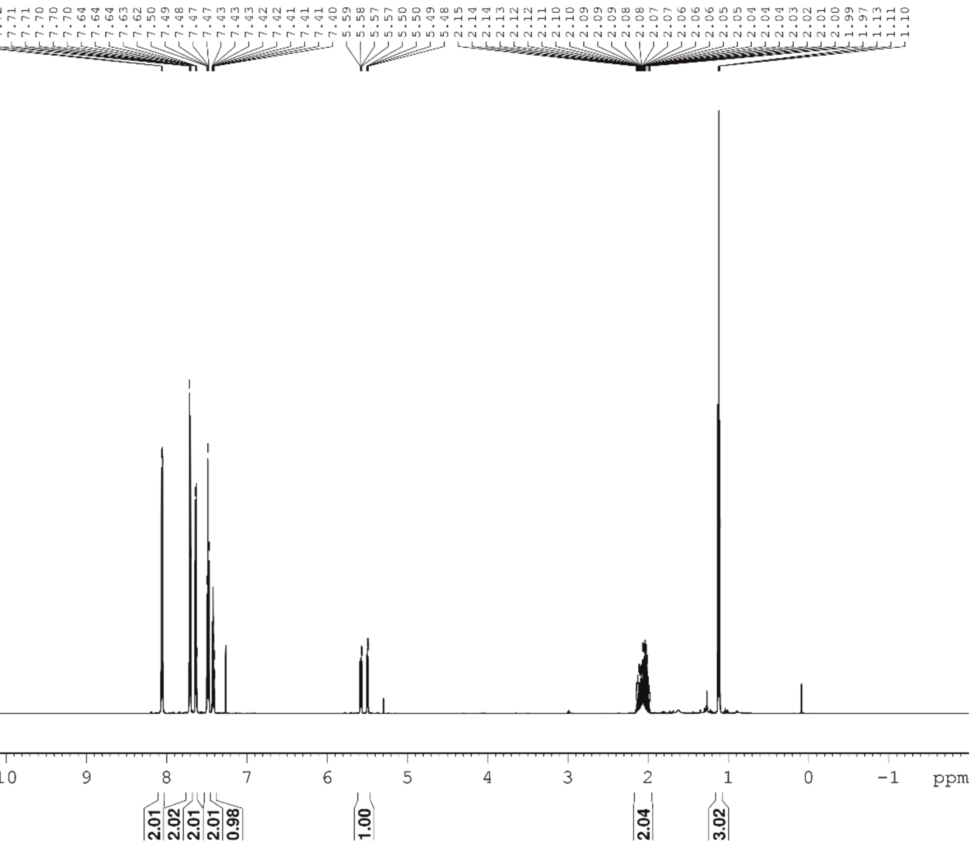

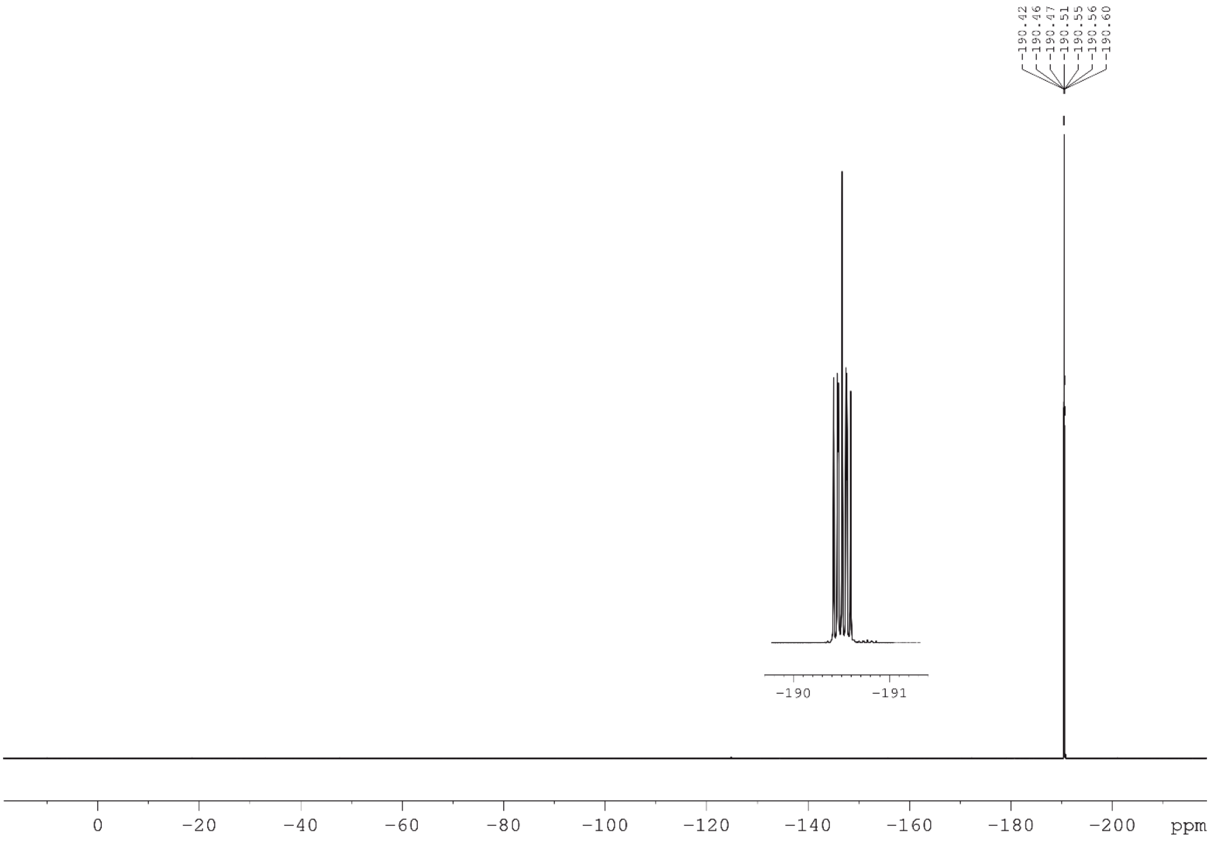
-190.42
-190.46
-190.47
-190.51
-190.55
-190.56
-190.60
-190
-191
0
-20
-40
-60
-80
-100
-120
-140
-160
-180
-200
ppm

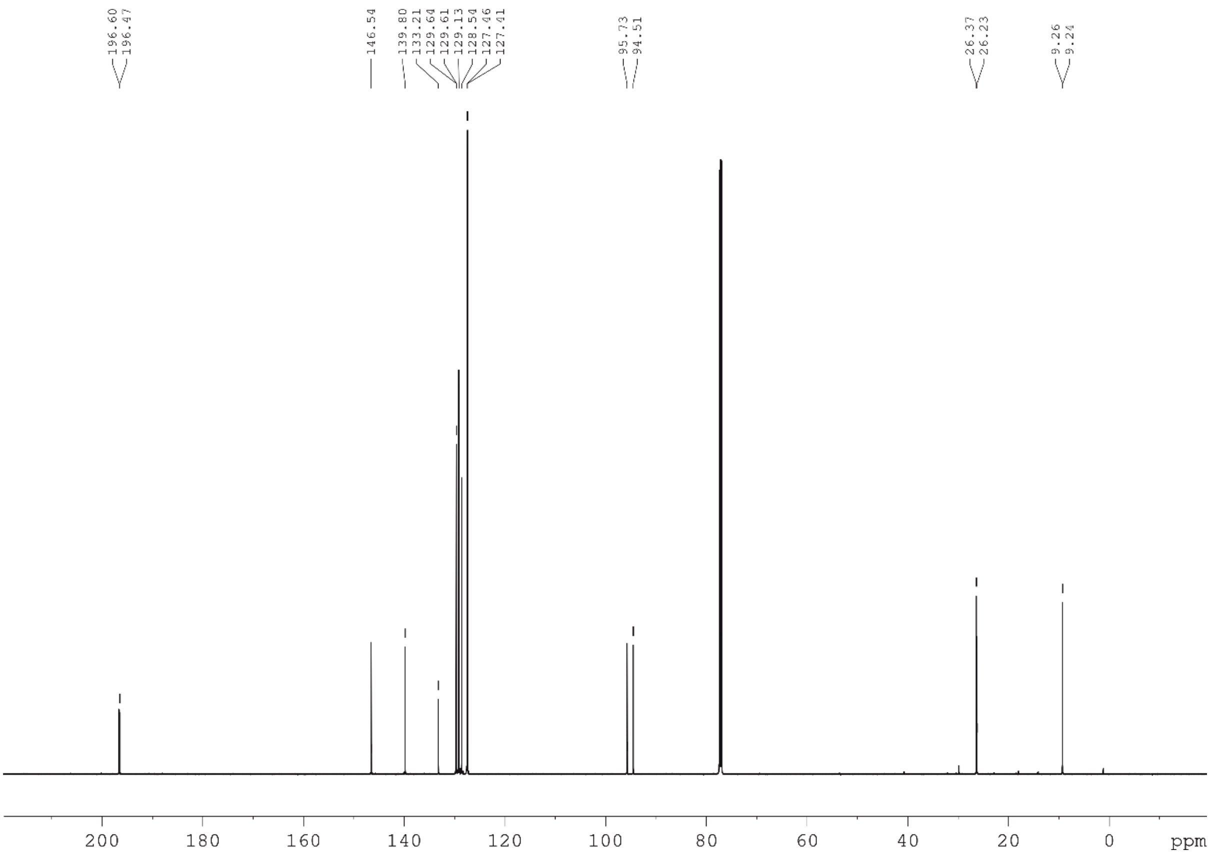
196.60
196.47
146.54
139.80
133.21
129.64
129.61
129.13
128.54
127.46
127.41
95.73
94.51
26.37
26.23
9.26
9.24
200
180
160
140
120
100
80
60
40
20
0
ppm

## (*S*)-4-chloro-2-fluoro-1-(4-fluorophenyl)butan-1-one (10m)

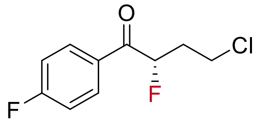


(*S*)-**10m**

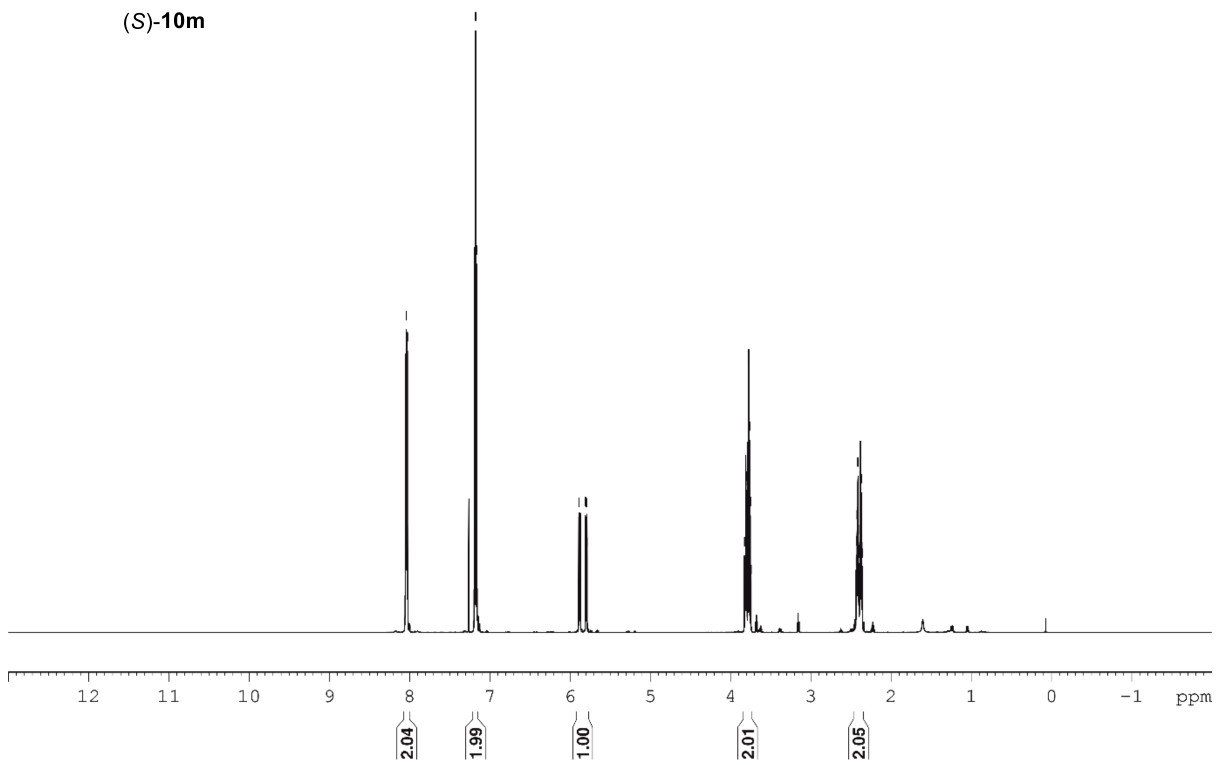


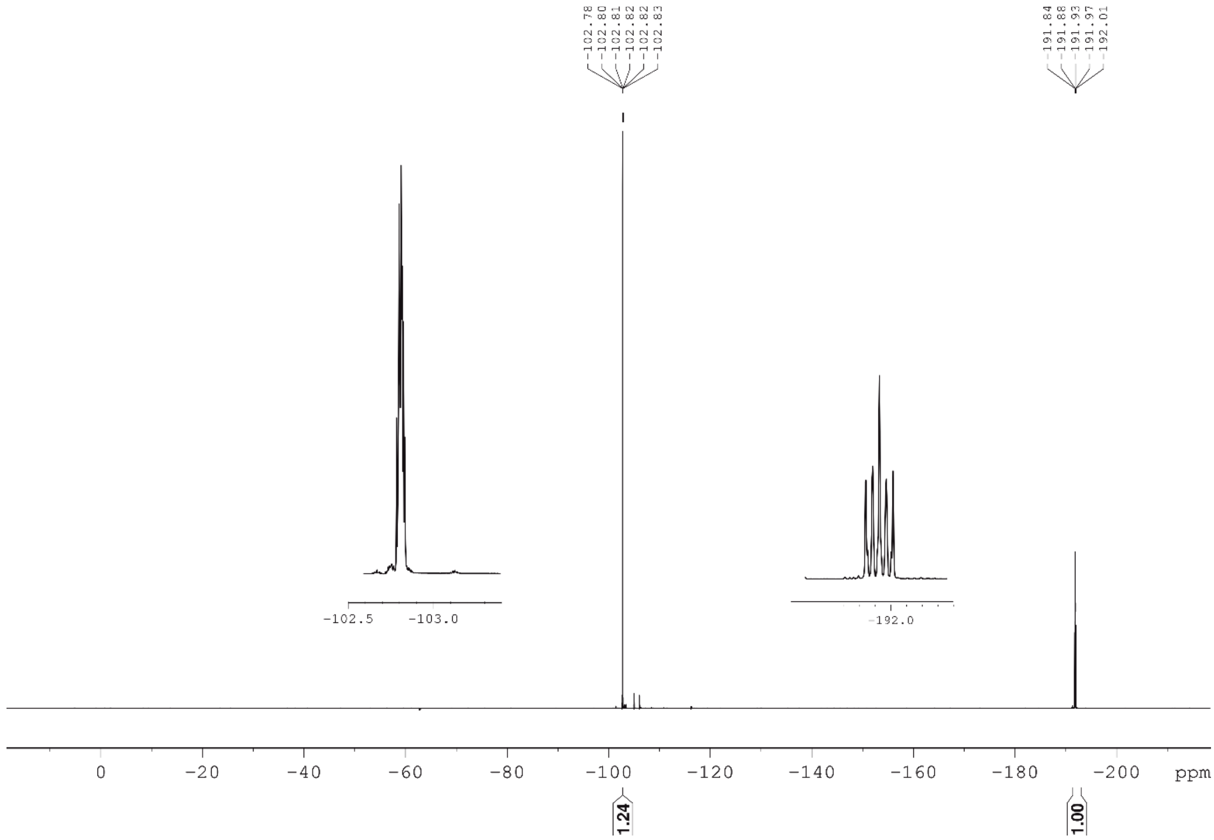

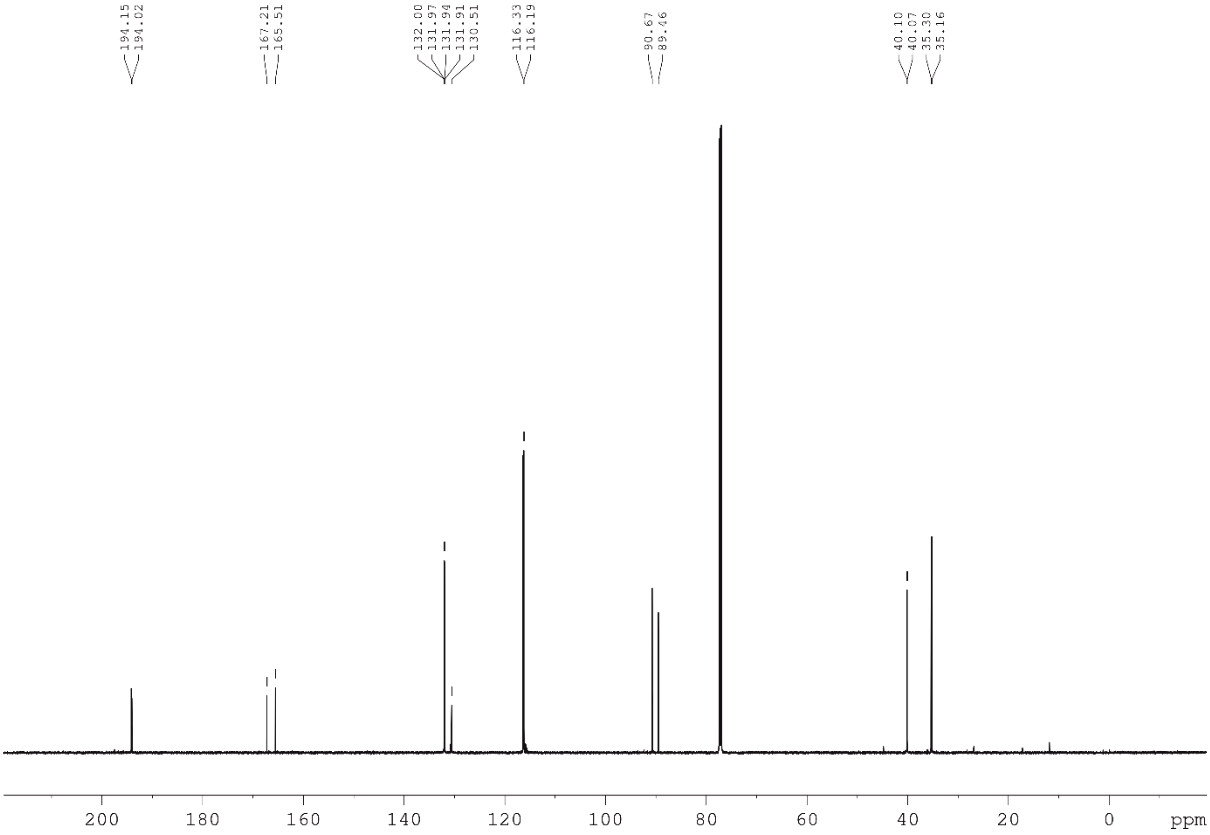


**Novel bromide precursors and bromides:**

**1-(4-(pyridine-2-yl)phenyl)ethan-1-ol (6e)**

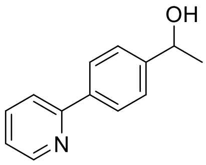


*rac*-**6e**

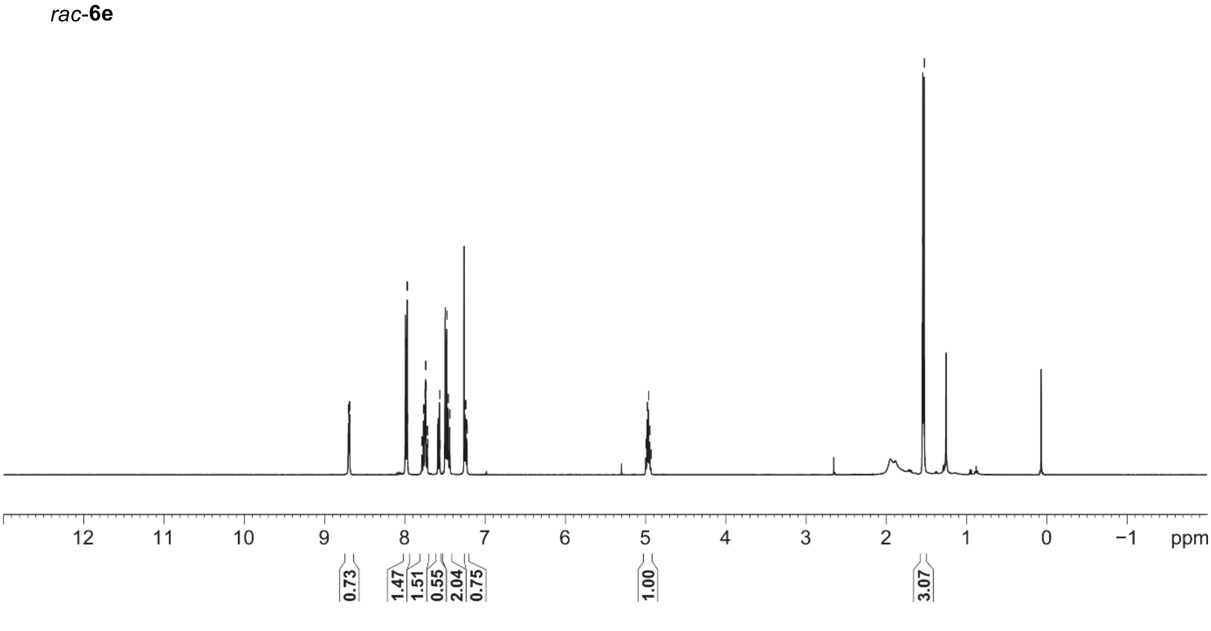

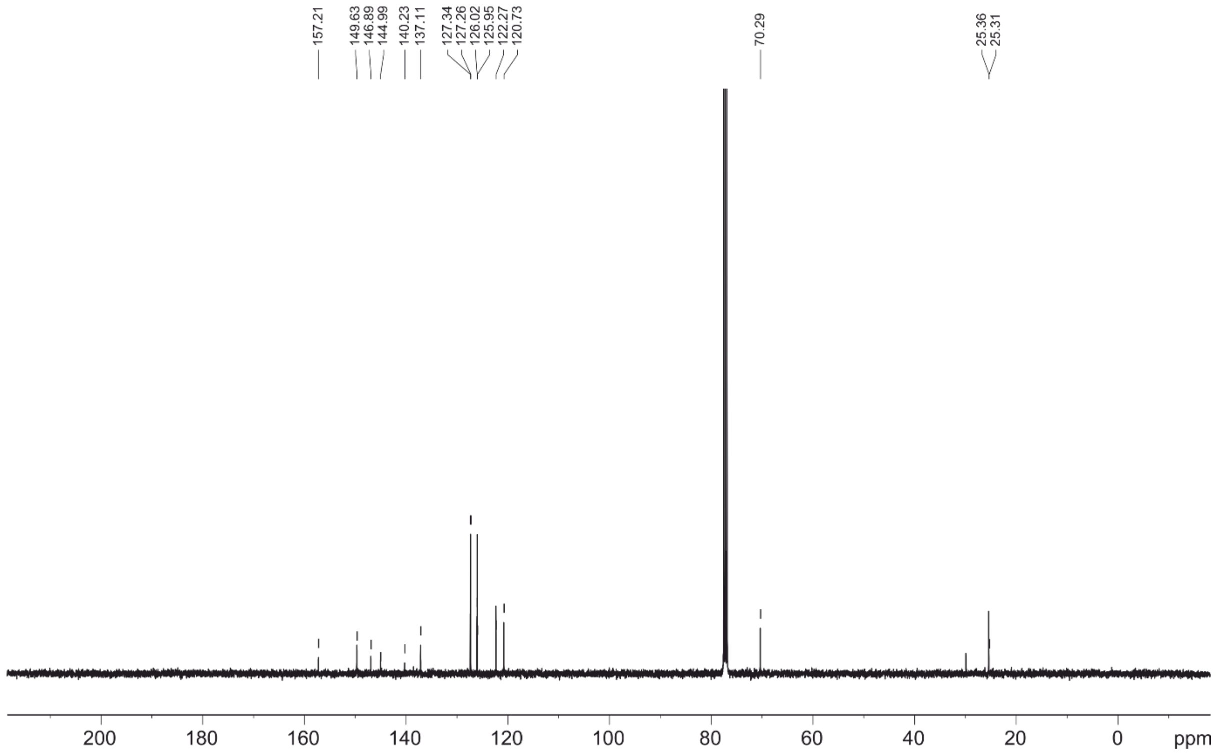


**_N,N_-diallyl-4'-(1-hydroxyethyl)-[1,1'-biphenyl]-4-carboxamide (6f)**

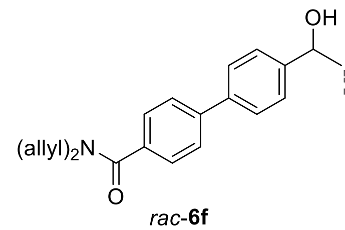


*rac*-**6f**

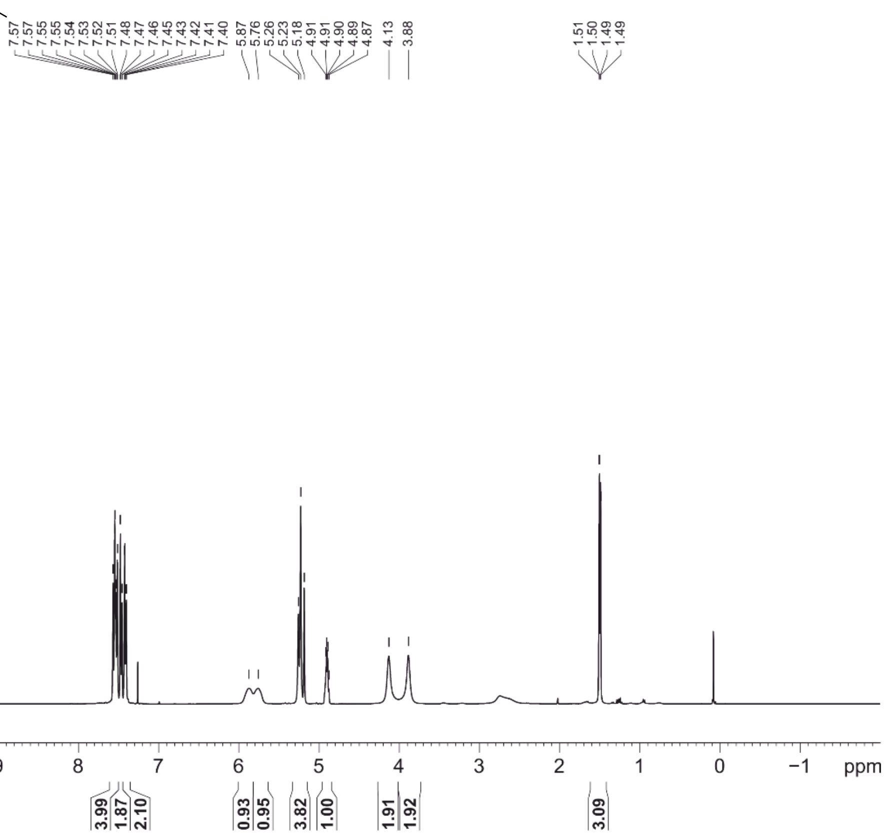

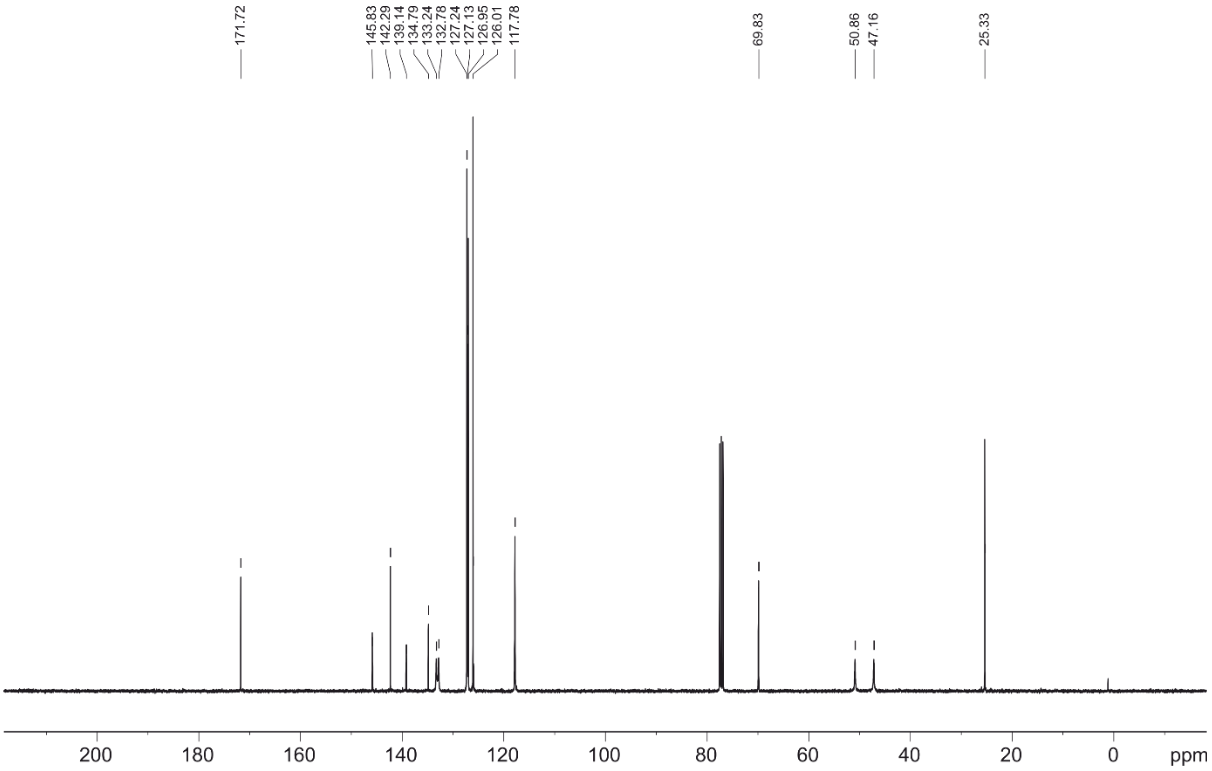


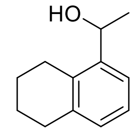


**1-(5,6,7,8-tetrahydronaphthalen-1-yl)ethan-1-ol (6i)**

*rac*-**6i**

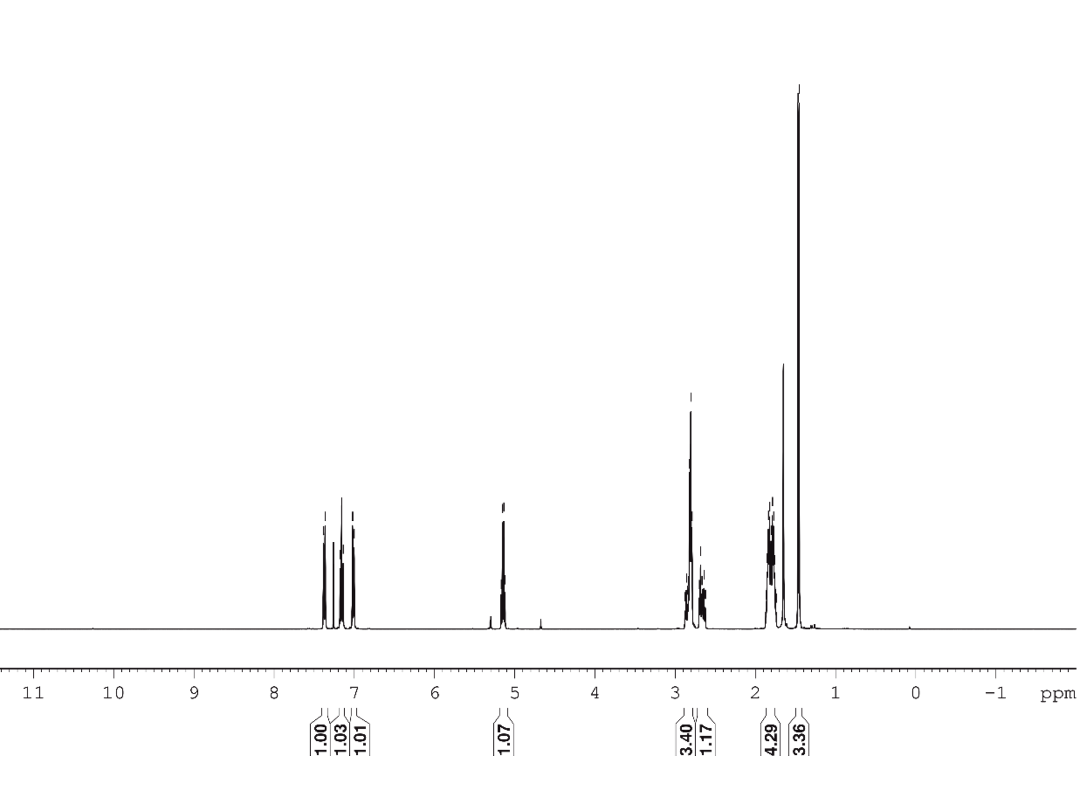

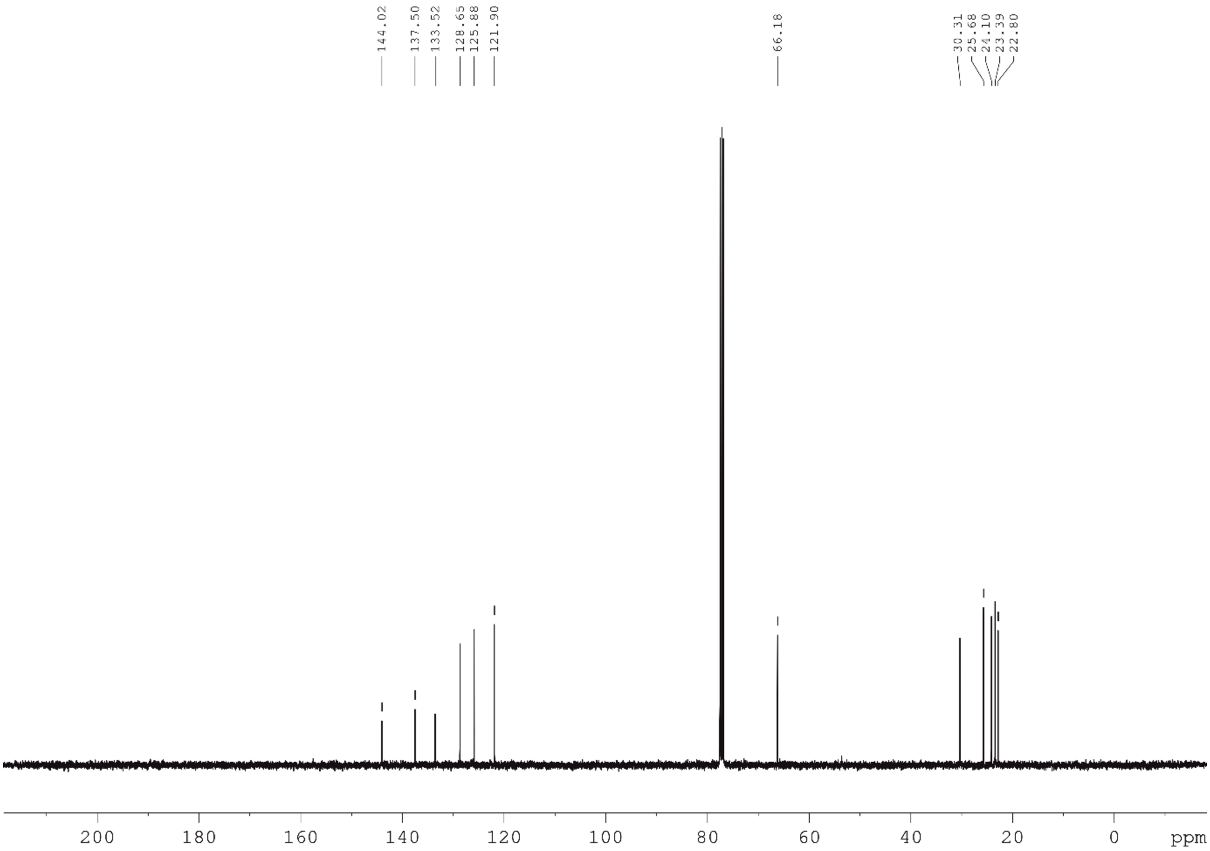


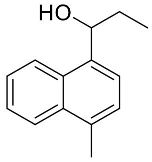


**1-(4-methylnaphthalen-1-yl)ethan-1-ol (6m)**

*rac*-**6m**

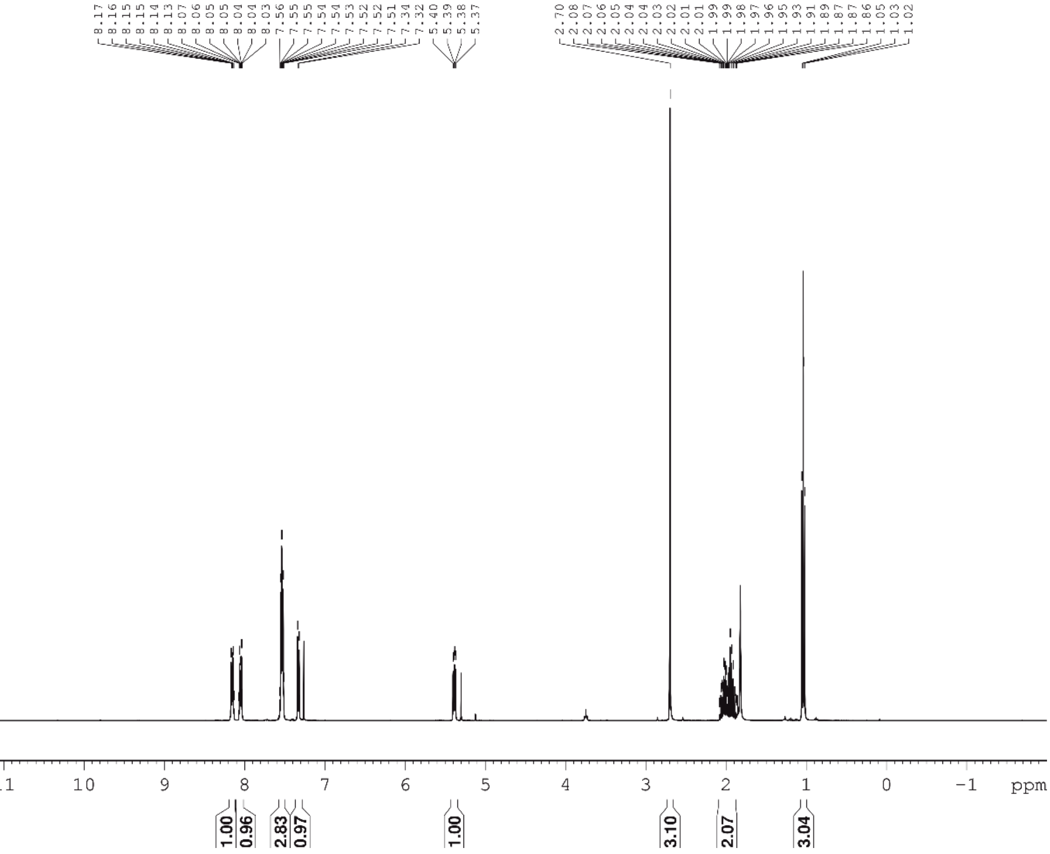

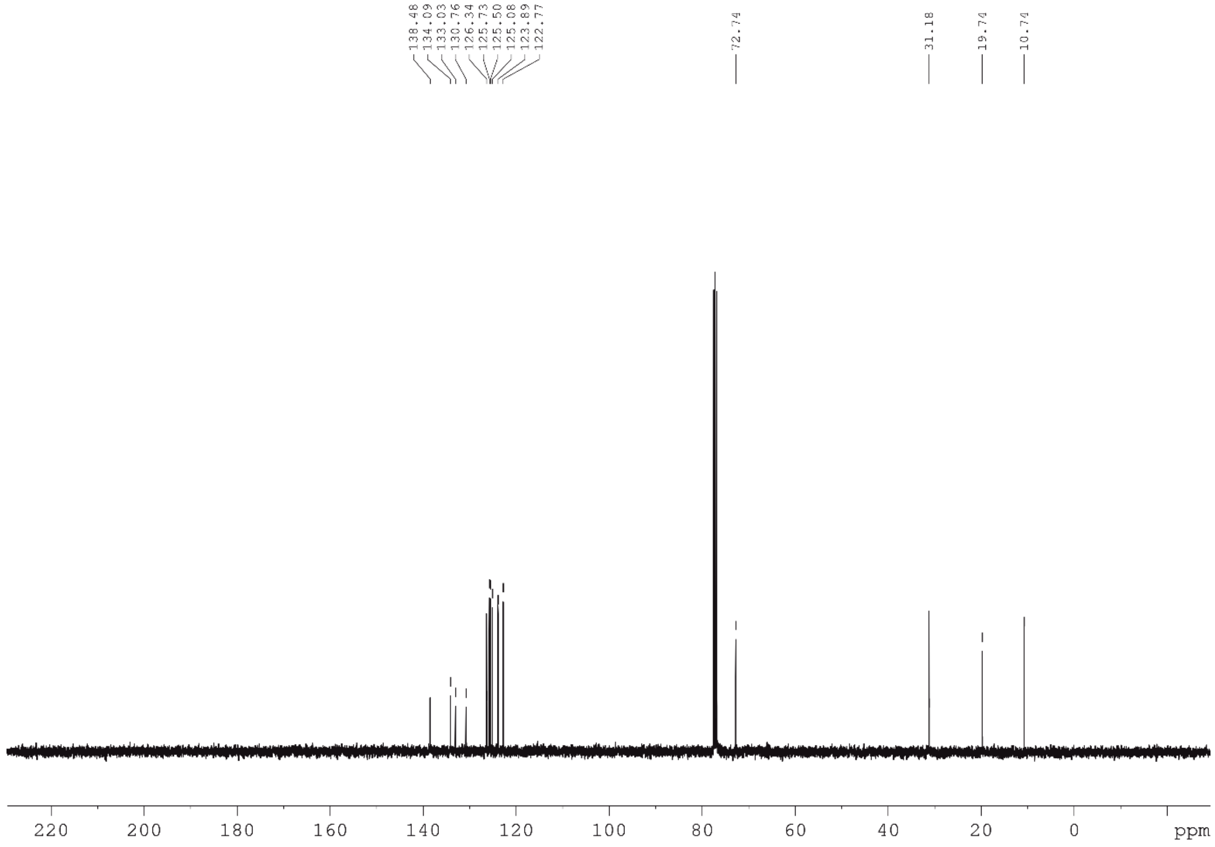


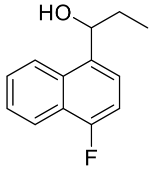

*rac*-**6n**

**1-(4-fluoronaphthalen-1-yl)ethan-1-ol (6n)**

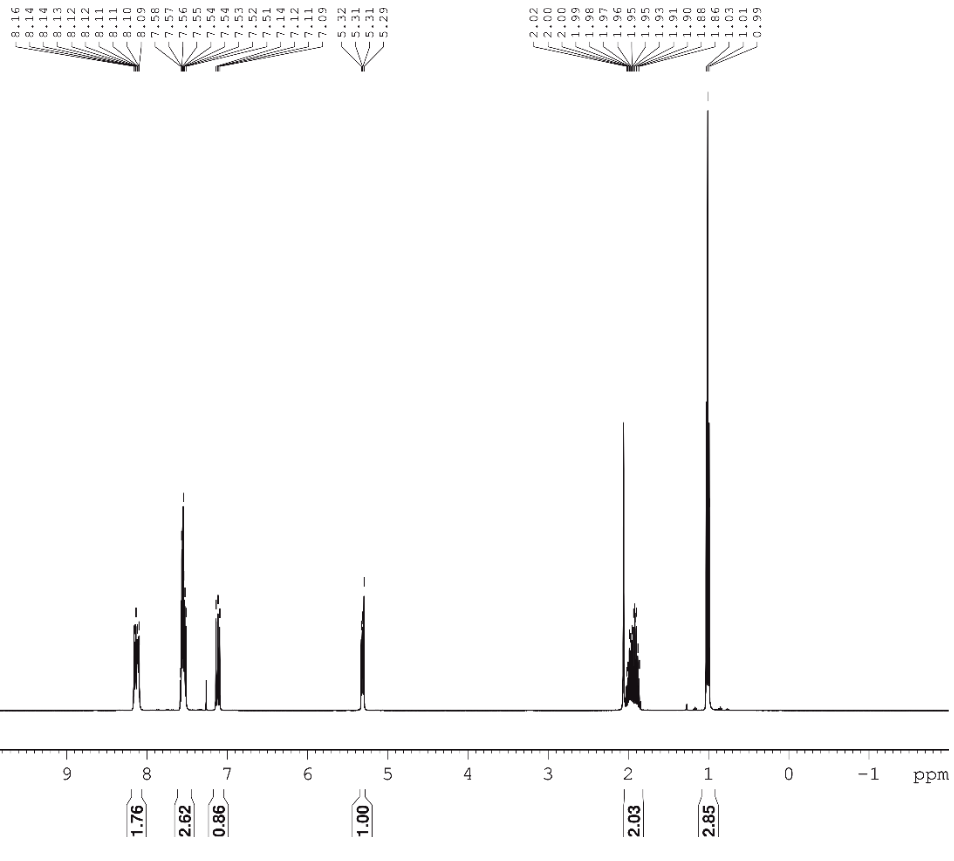

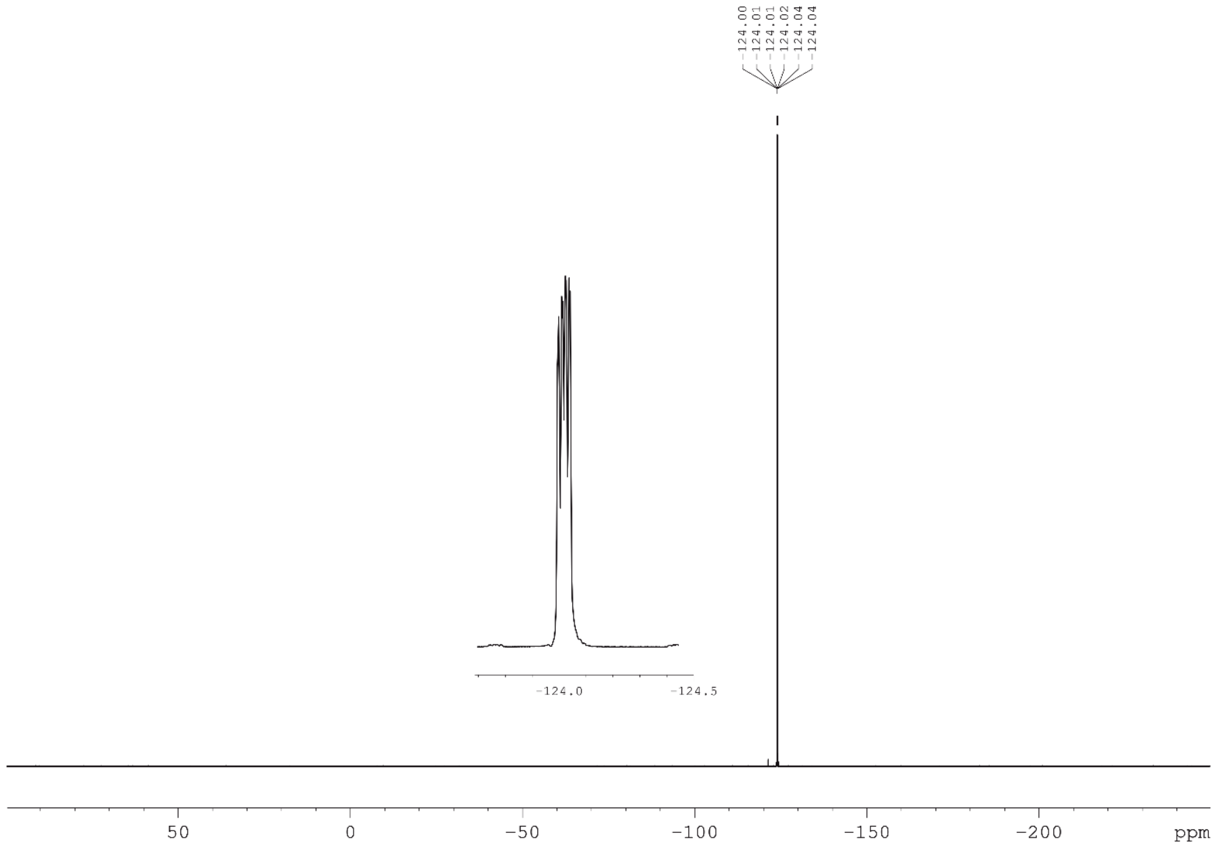
-124.00
-124.01
-124.01
-124.02
-124.04
-124.04
-124.0
-124.5
50
0
-50
-100
-150
-200
ppm

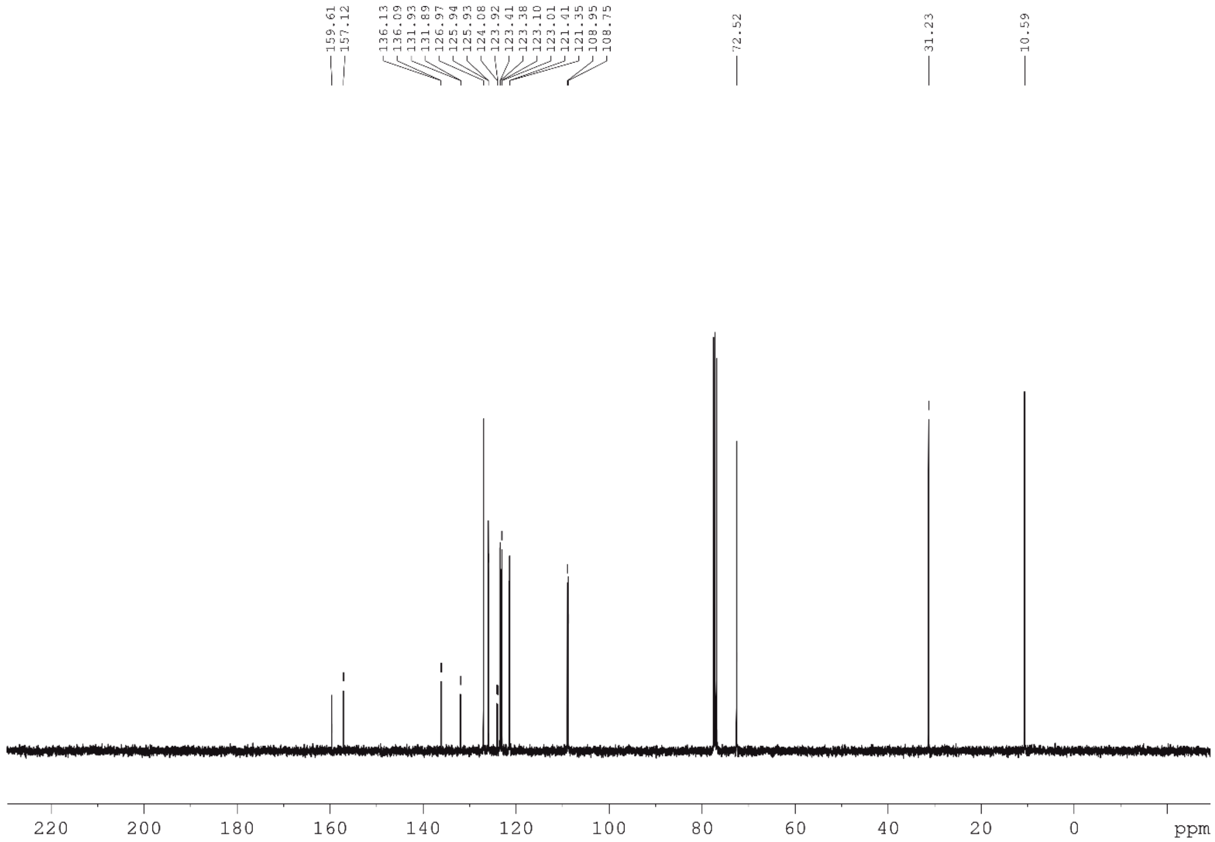
159.61
157.12
136.13
136.09
131.93
131.89
126.97
125.94
125.93
124.08
123.92
123.41
123.38
123.10
123.01
121.41
121.35
108.95
108.75
72.52
31.23
10.59
220
200
180
160
140
120
100
80
60
40
20
0
ppm

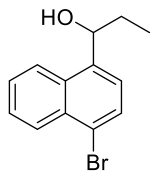


*rac*-**6o**

# 1-(4-bromonaphthalen-1-yl)propan-1-ol (6o)

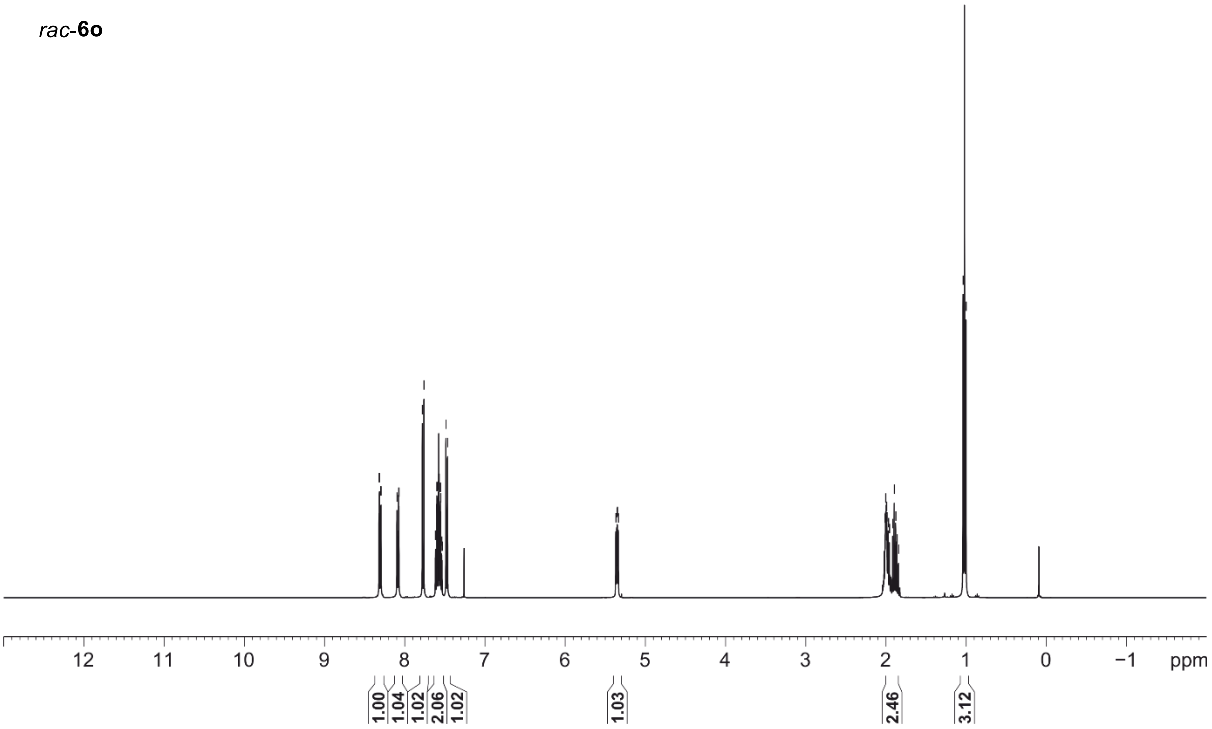


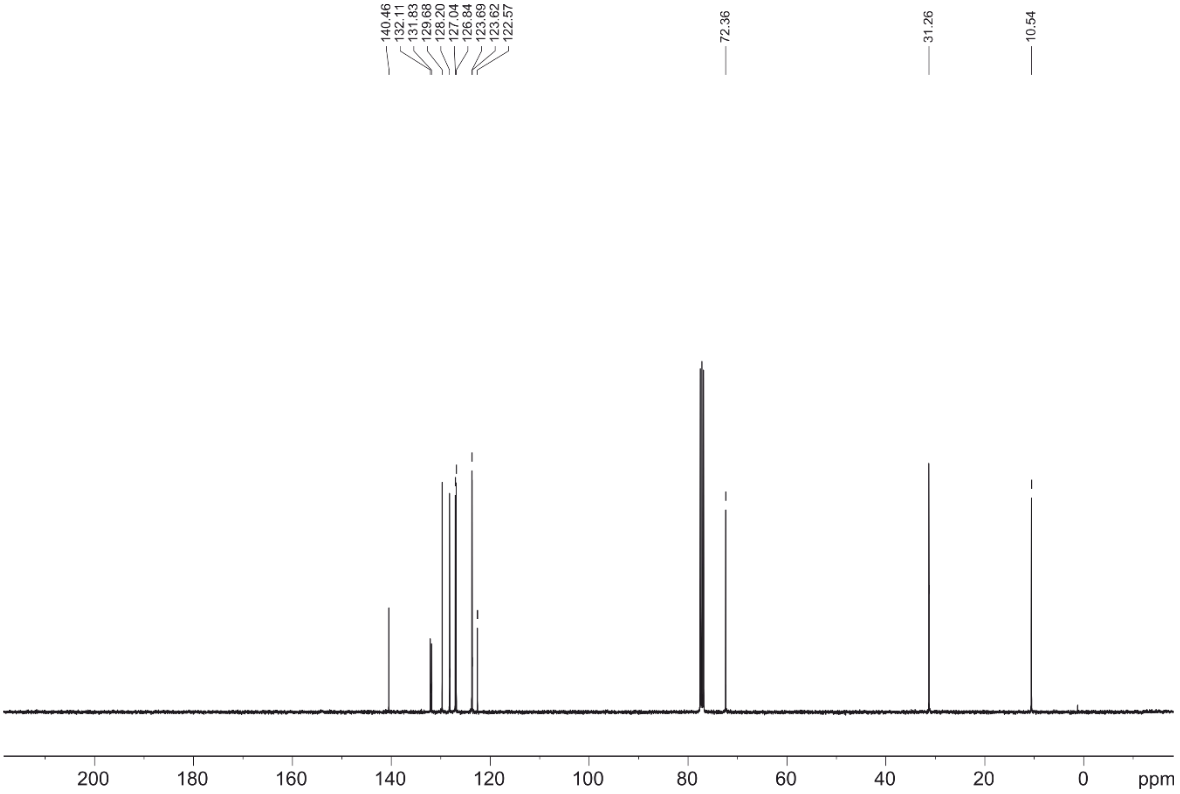

**1-(naphthalen-1-yl)butan-1-ol (6p)**

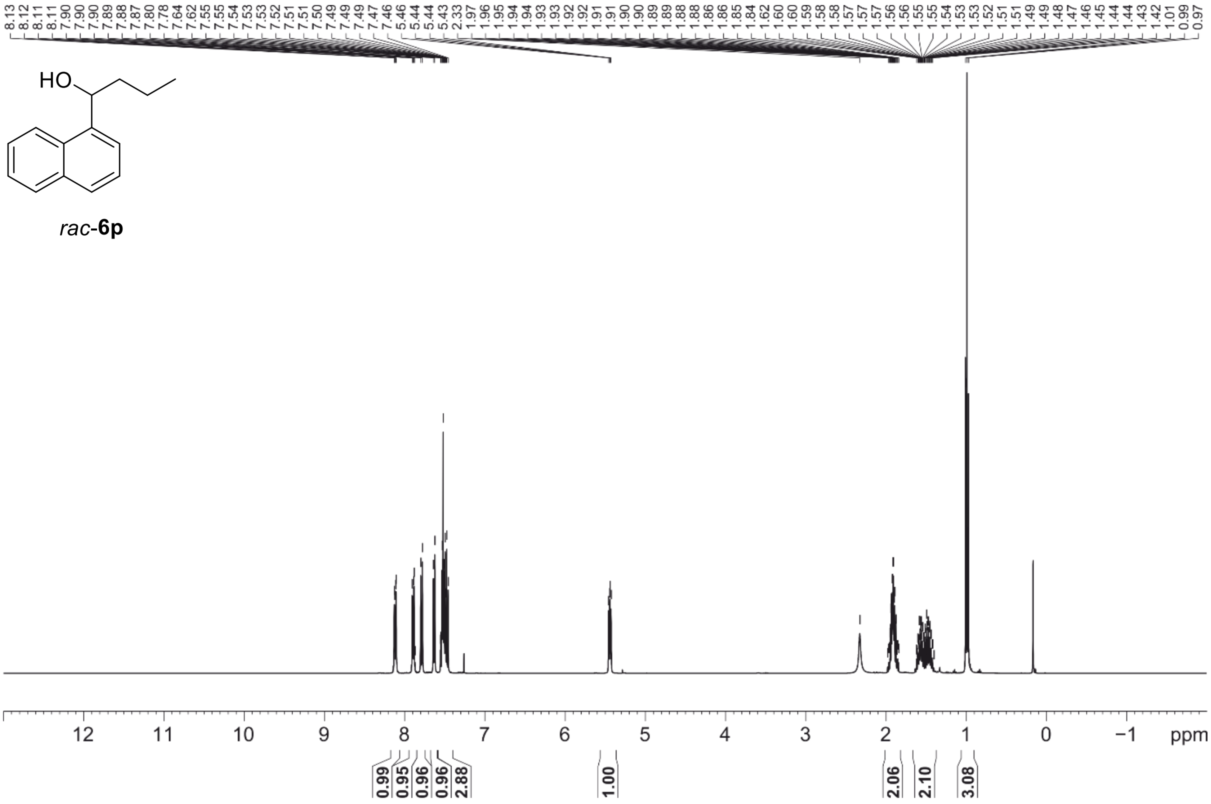


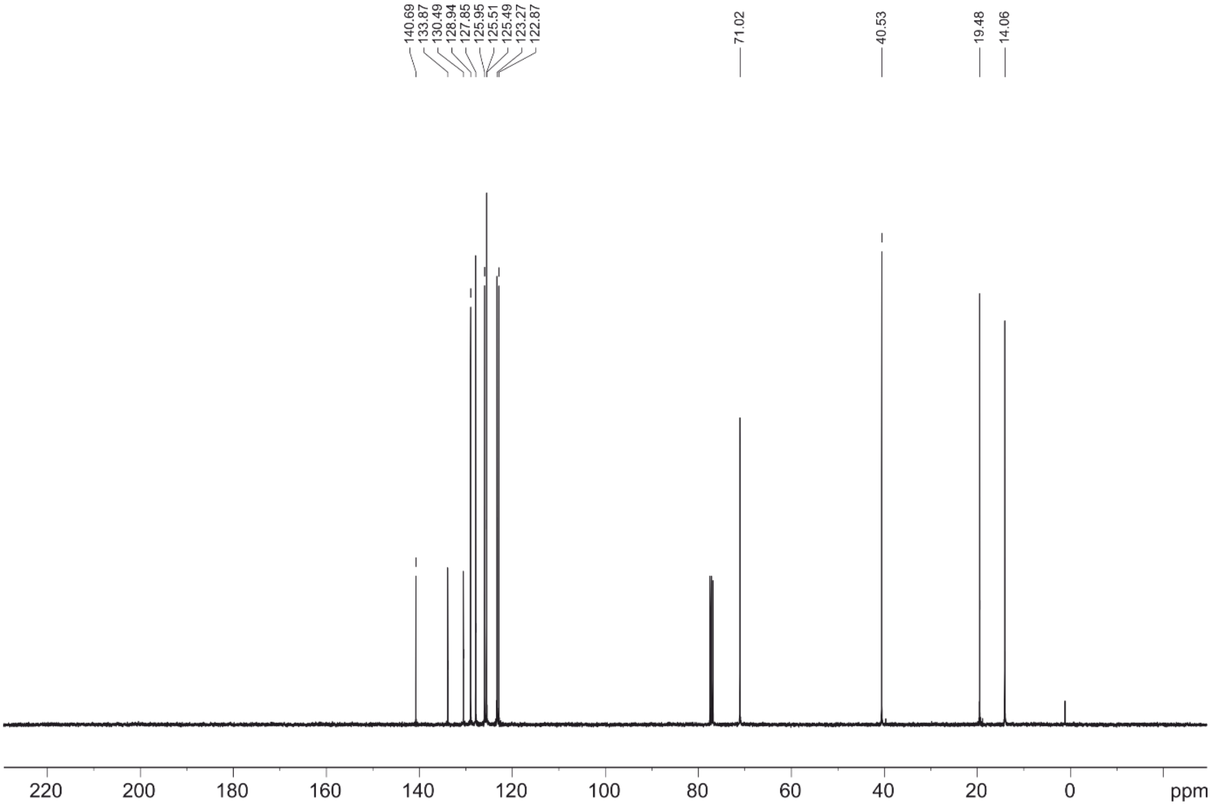

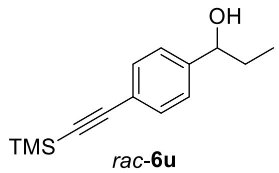

*rac*-**6u**

## 1-(4-((trimethylsilyl)ethynyl)phenyl)propan-1-ol (6u)

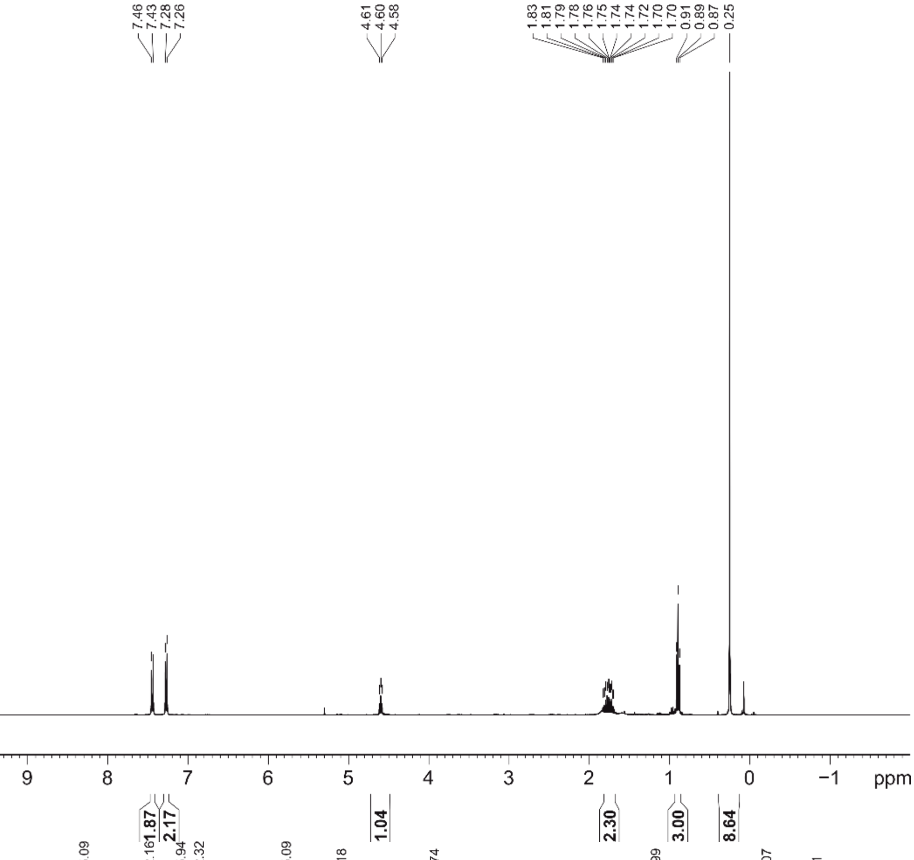

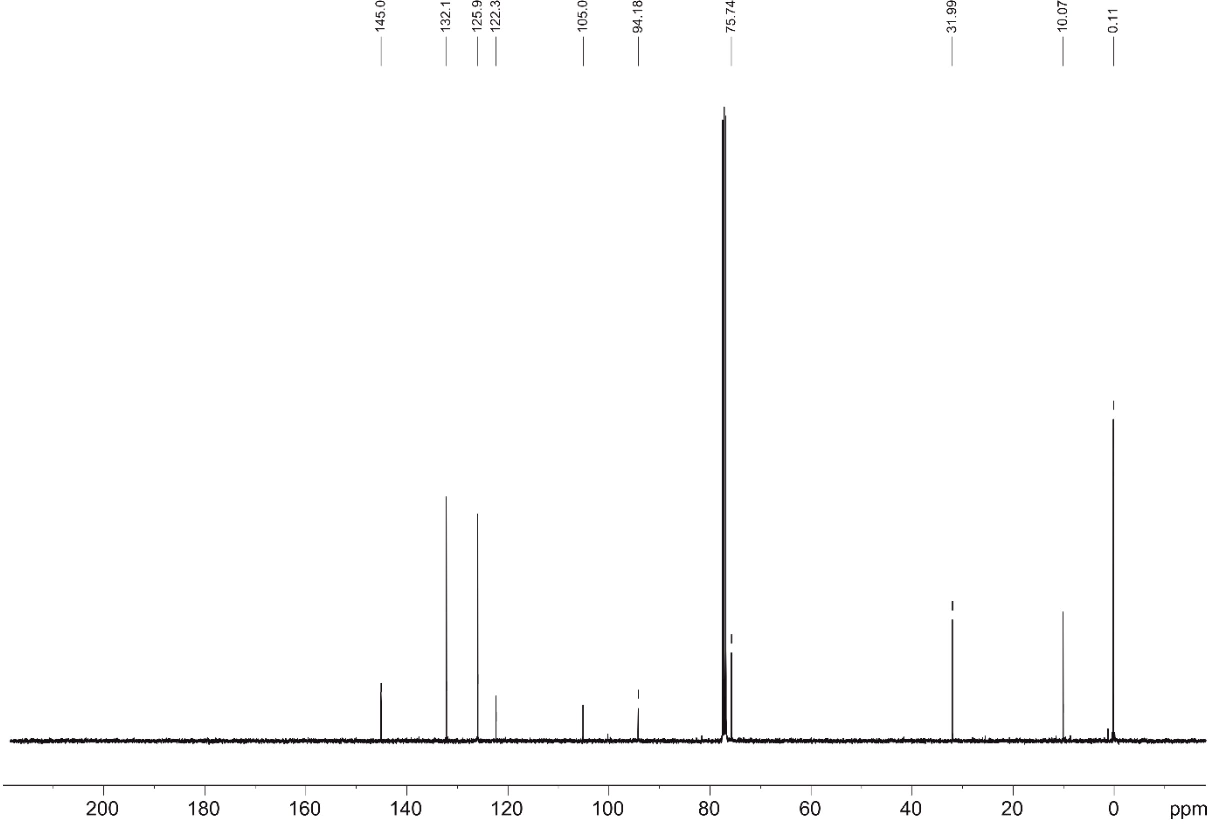

**1-(4-((4-iodophenoxy)methyl)phenyl)ethan-1-ol (6w)**

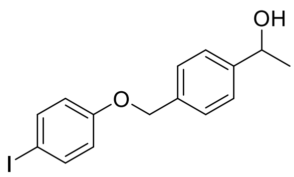


*rac*-**6w**

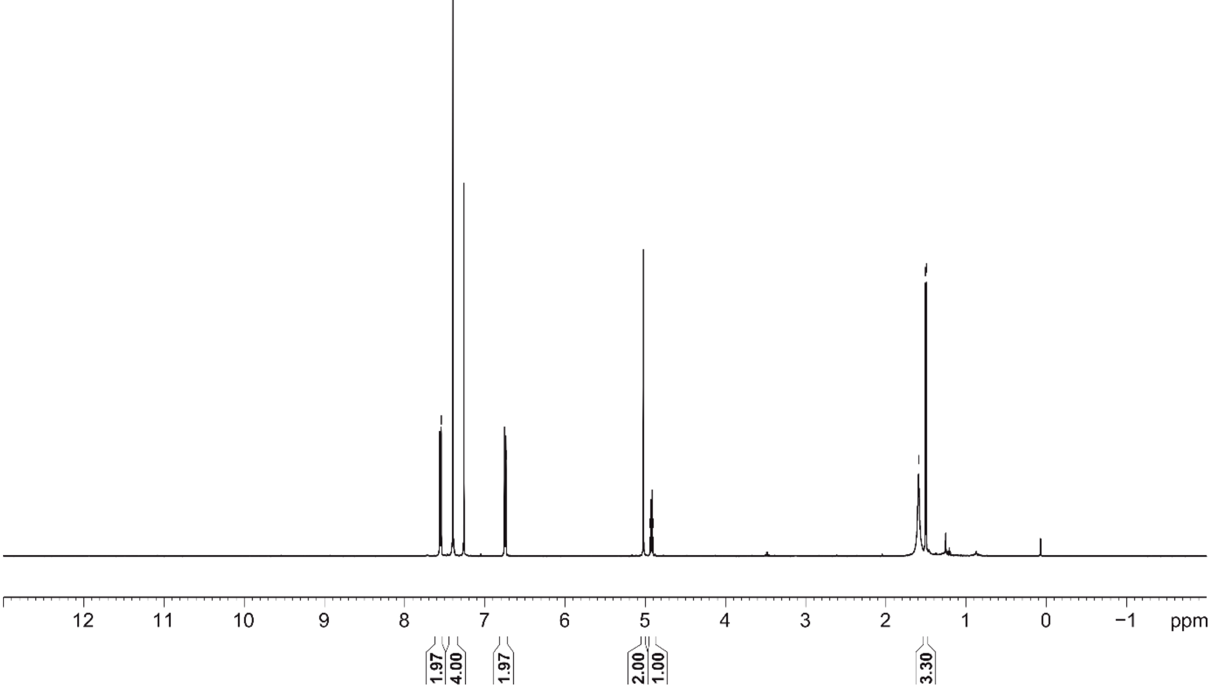


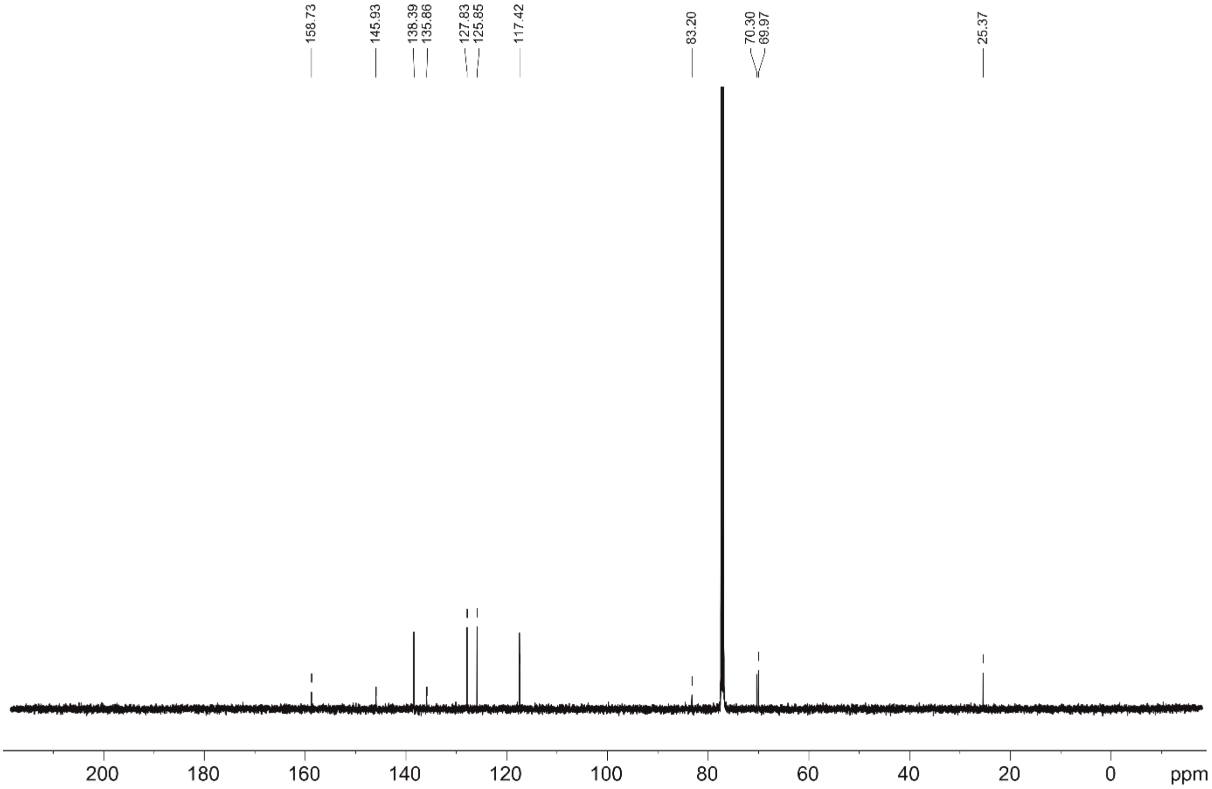

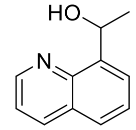


*rac*-**6x**

**1-(quinoline-8-yl)ethan-1-ol (6x)**

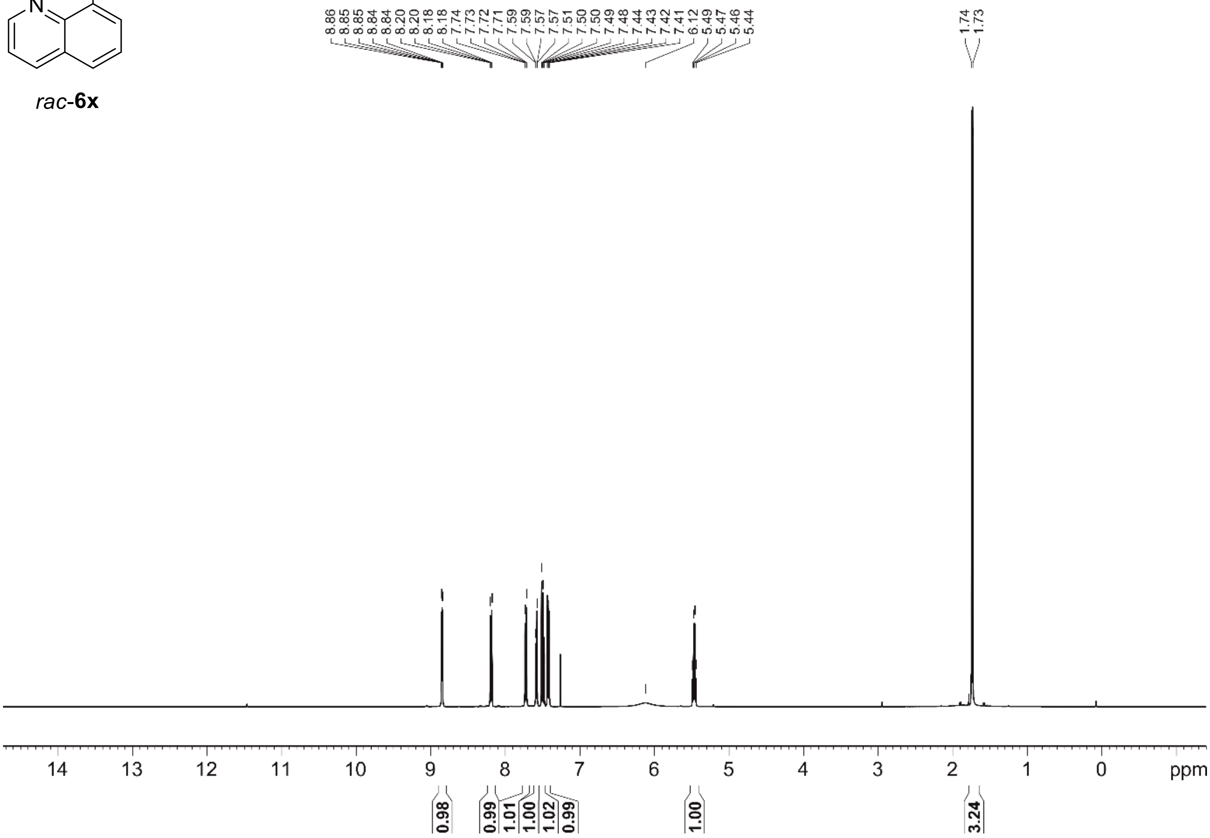


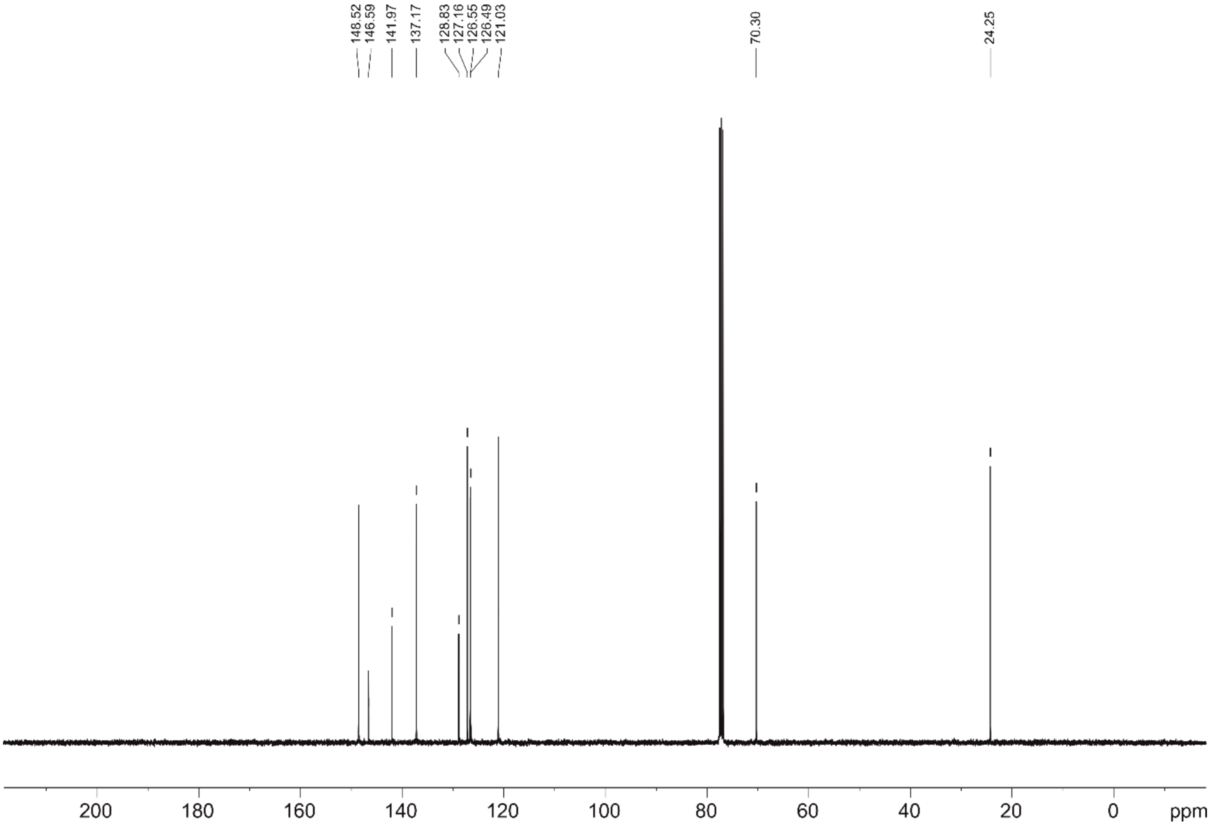

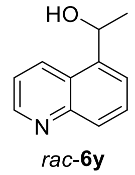

*rac*-**6y**

**1-(quinoline-5-yl)ethan-1-ol (6y)**

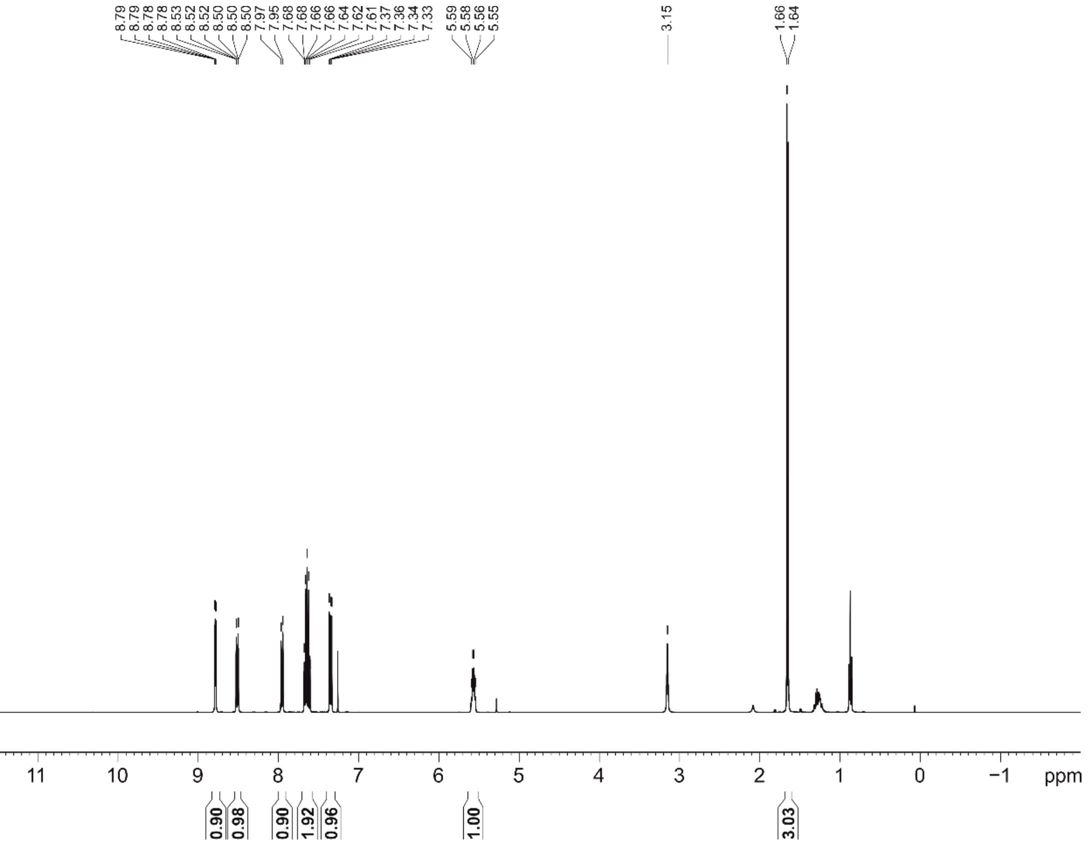


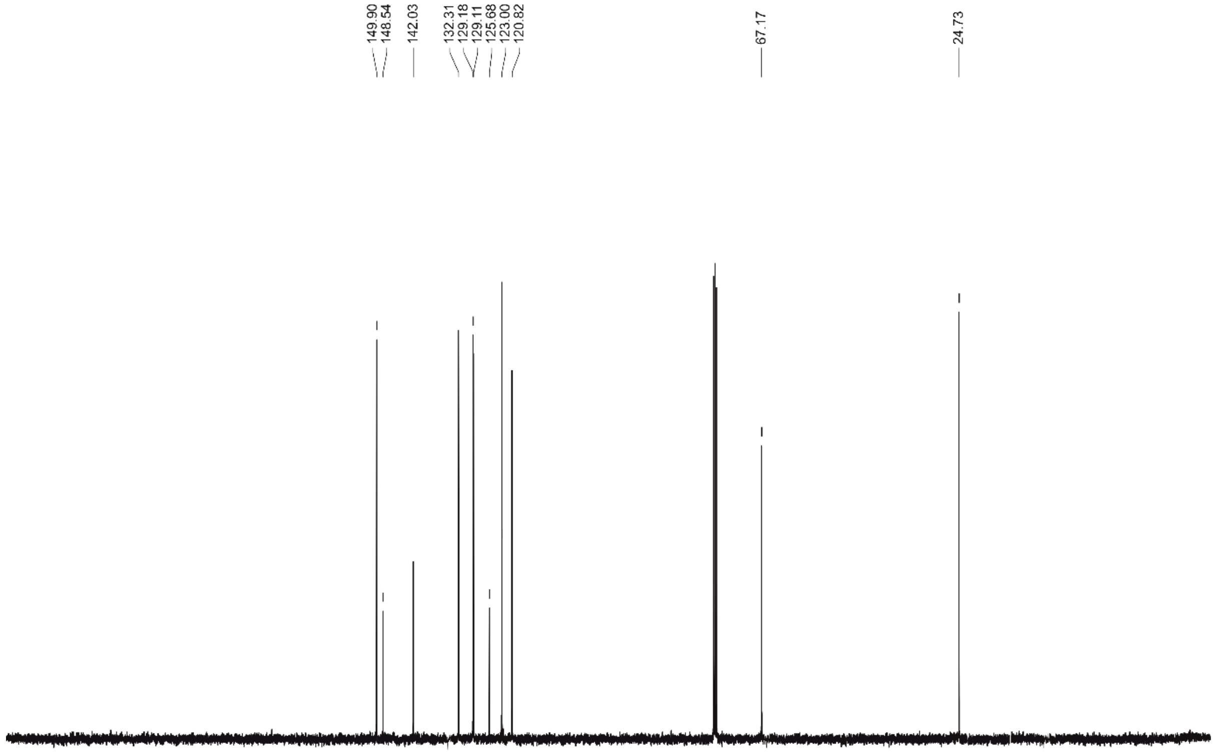

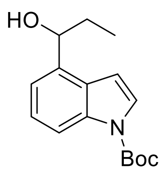


*rac*-**6z**

### ***tert*-butyl-4-(1-hydroxypropyl)-1*H*-indole-1-carboxylate (6z)**

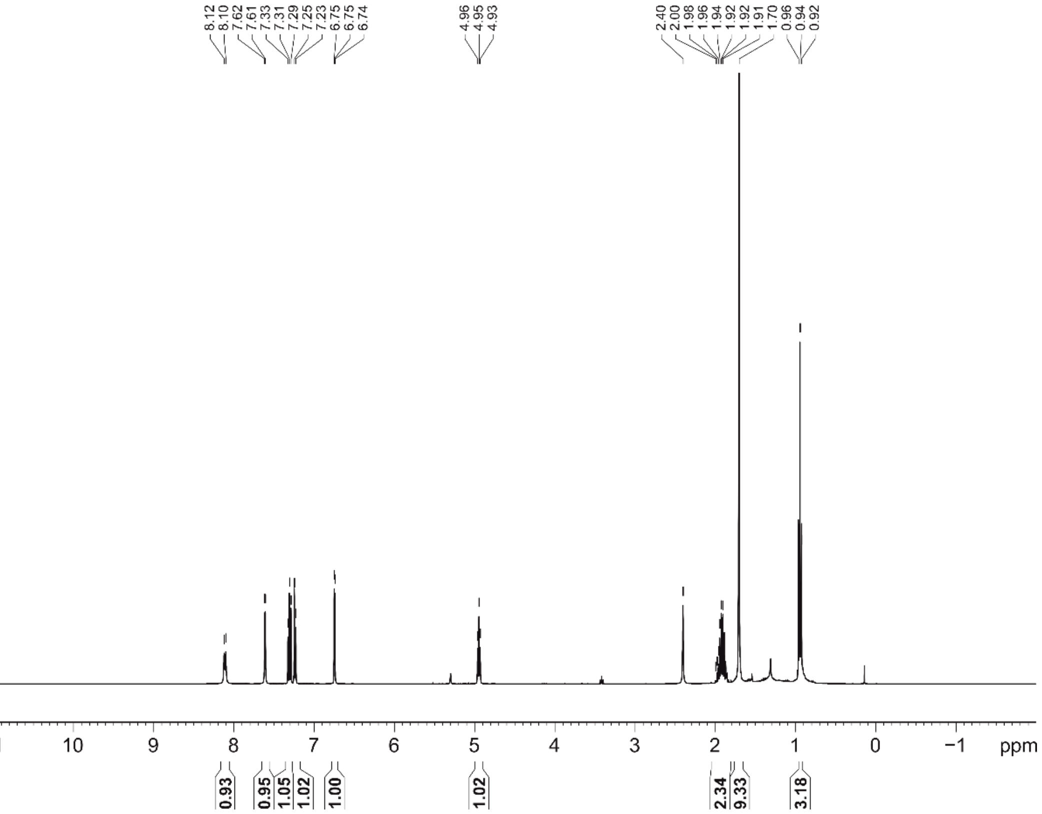


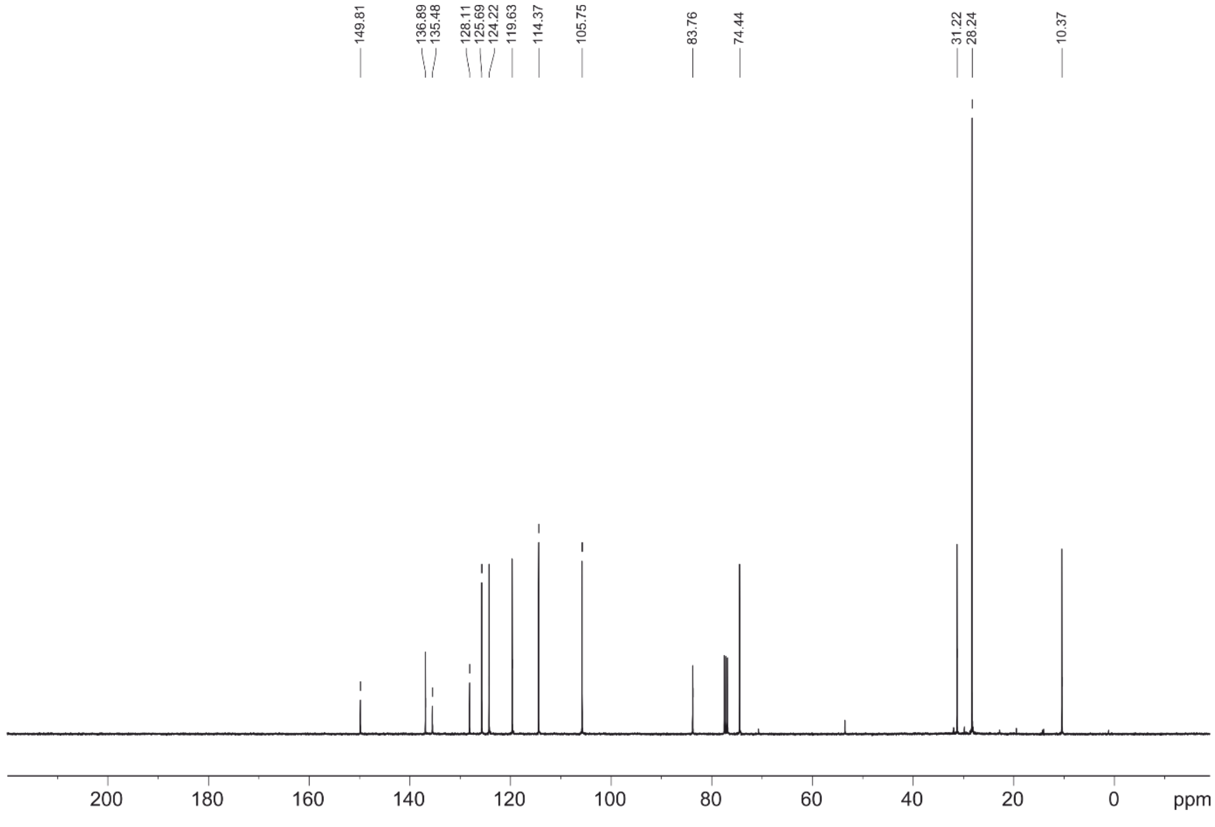

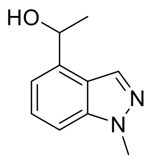


*rac*-**6aa**

**1-(1-methyl-1*H*-indazol-4-yl)ethan-1-ol (6aa)**

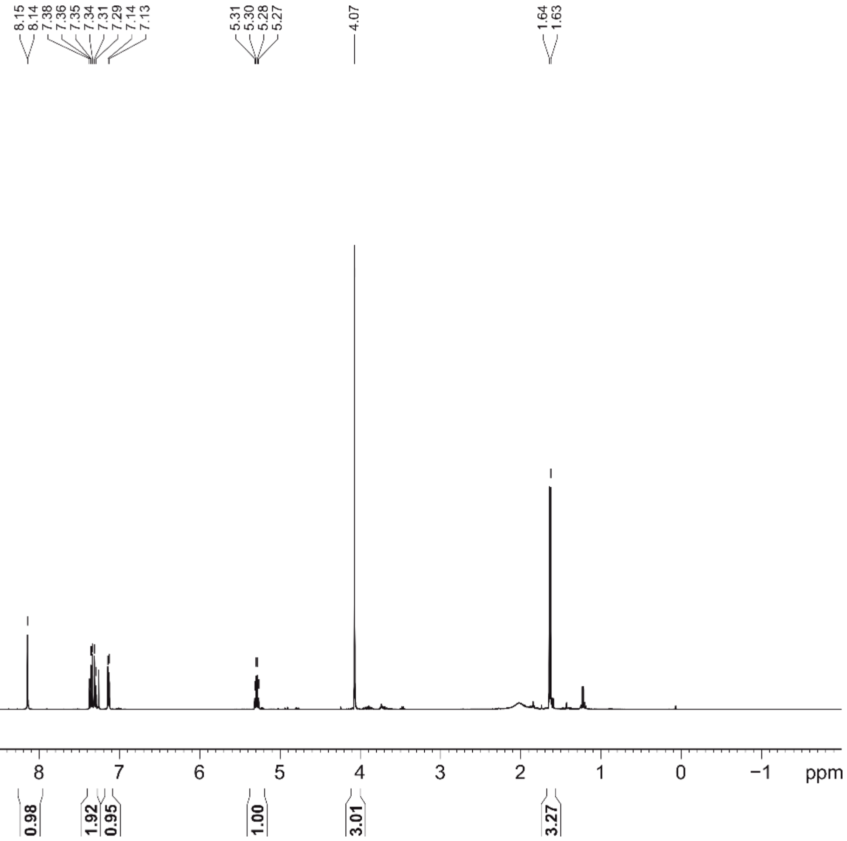


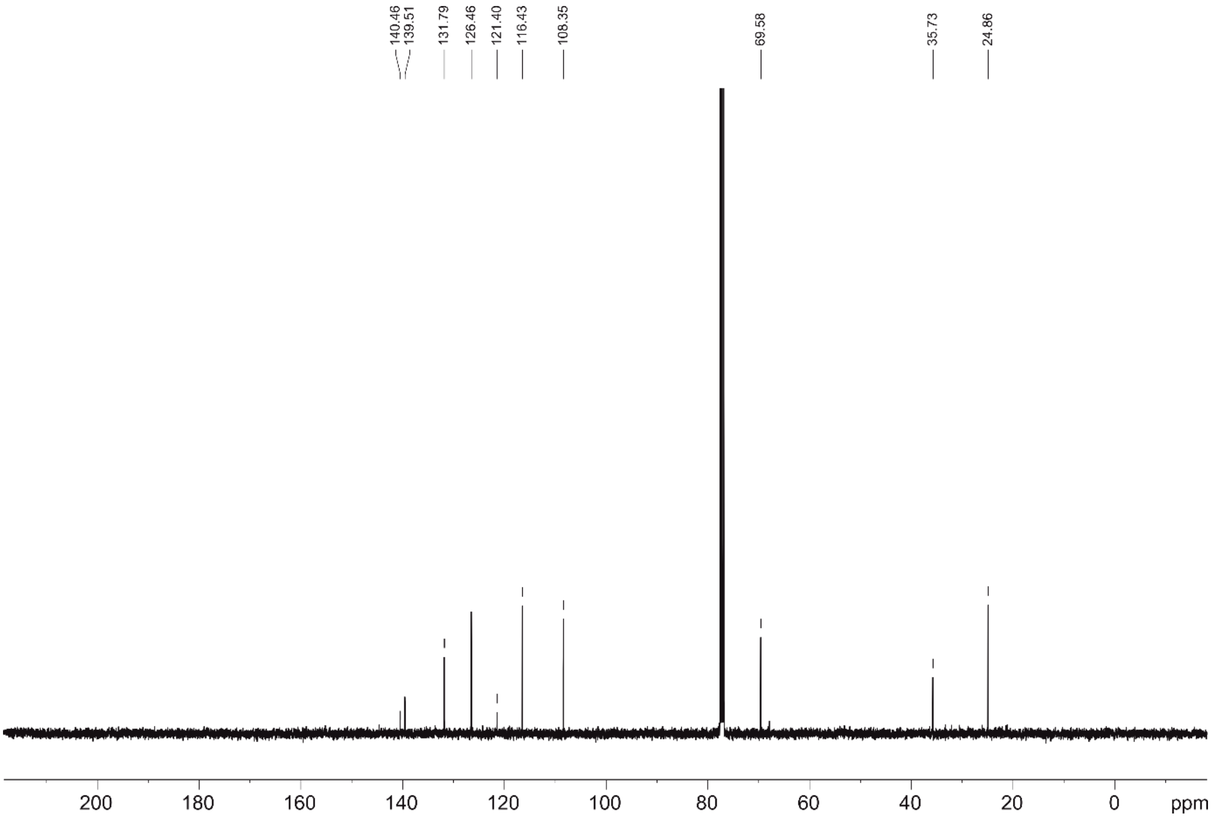

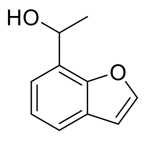


*rac*-**6ab**

**1-(benzofuran-7-yl)ethan-1-ol (6ab)**

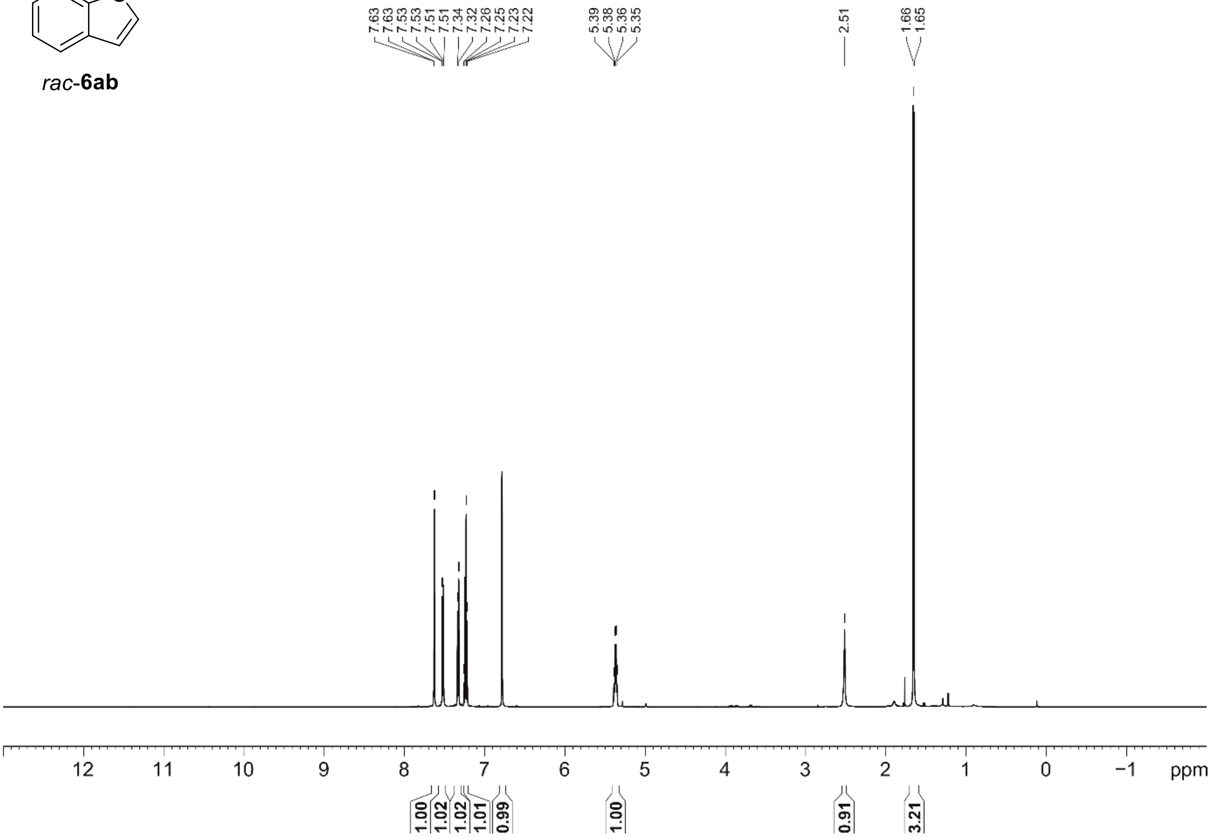


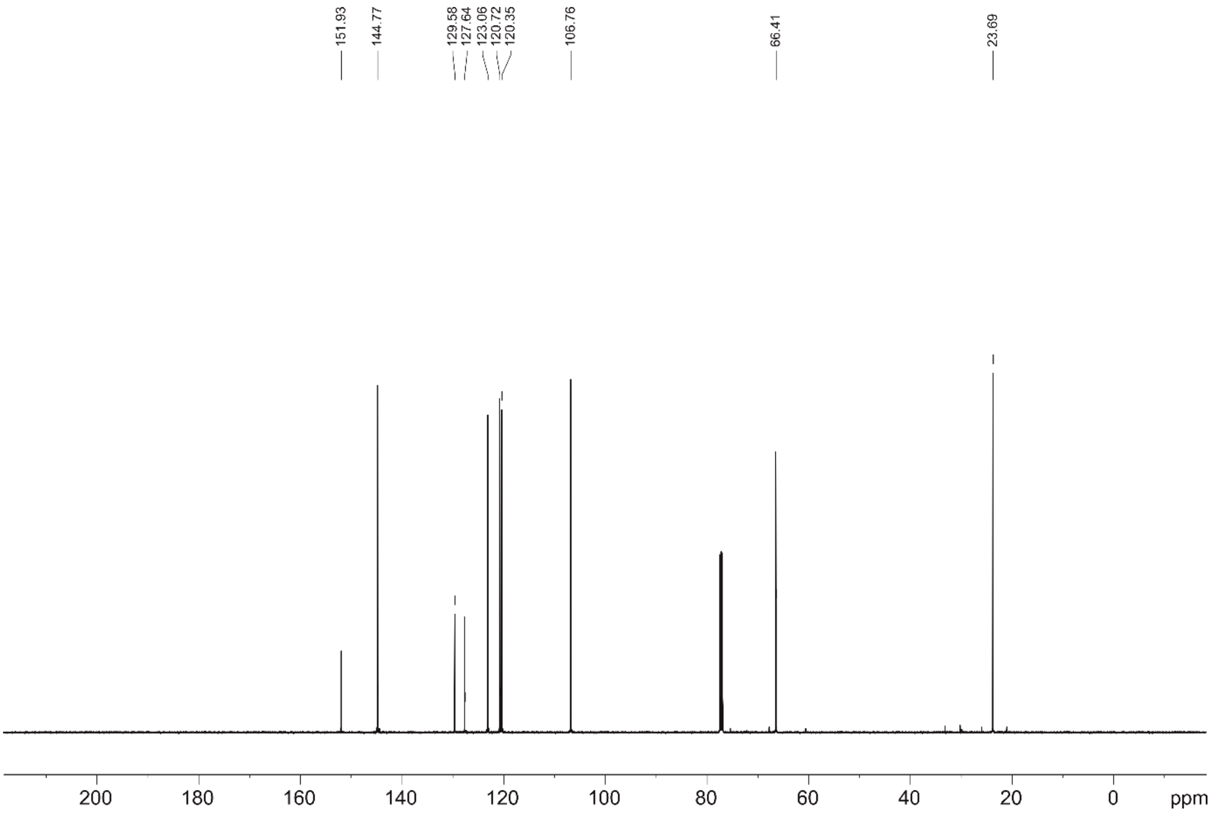

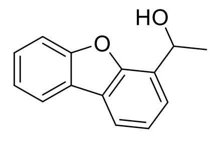

*rac*-**6ac**

**1-(dibenzo[*b*,*d*]furan-4-yl)ethan-1-ol (6ac)**

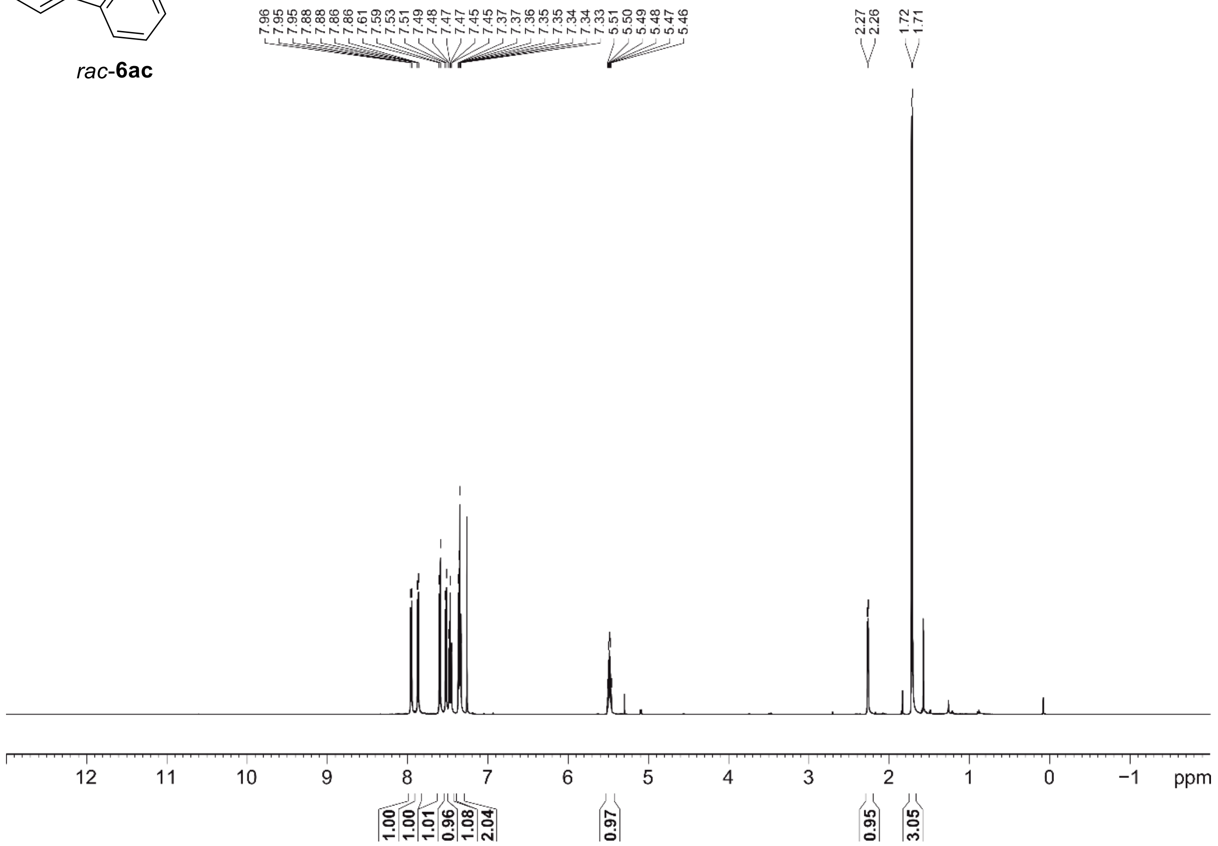


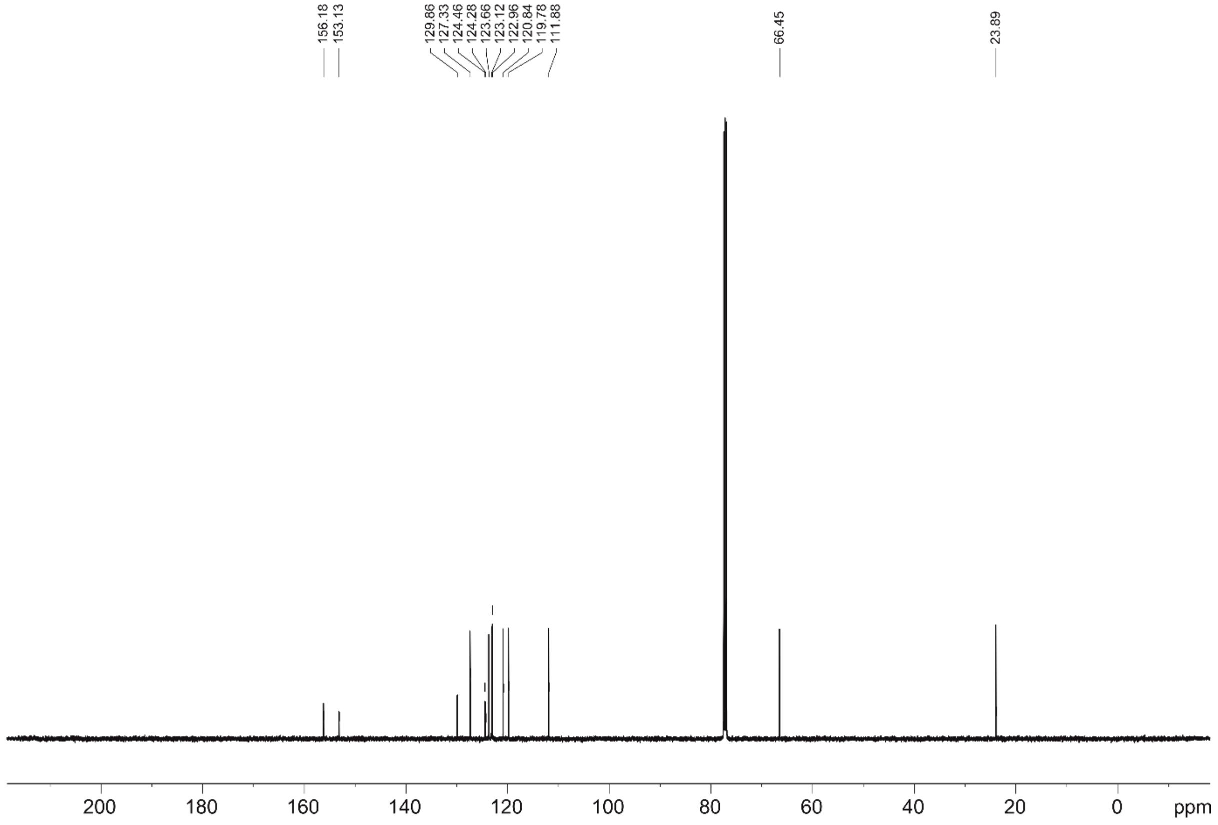

**1-(dibenzo[*b*,*d*]thiophene-4-yl)ethan-1-ol (6ad)**

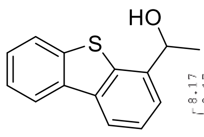


*rac*-**6ad**

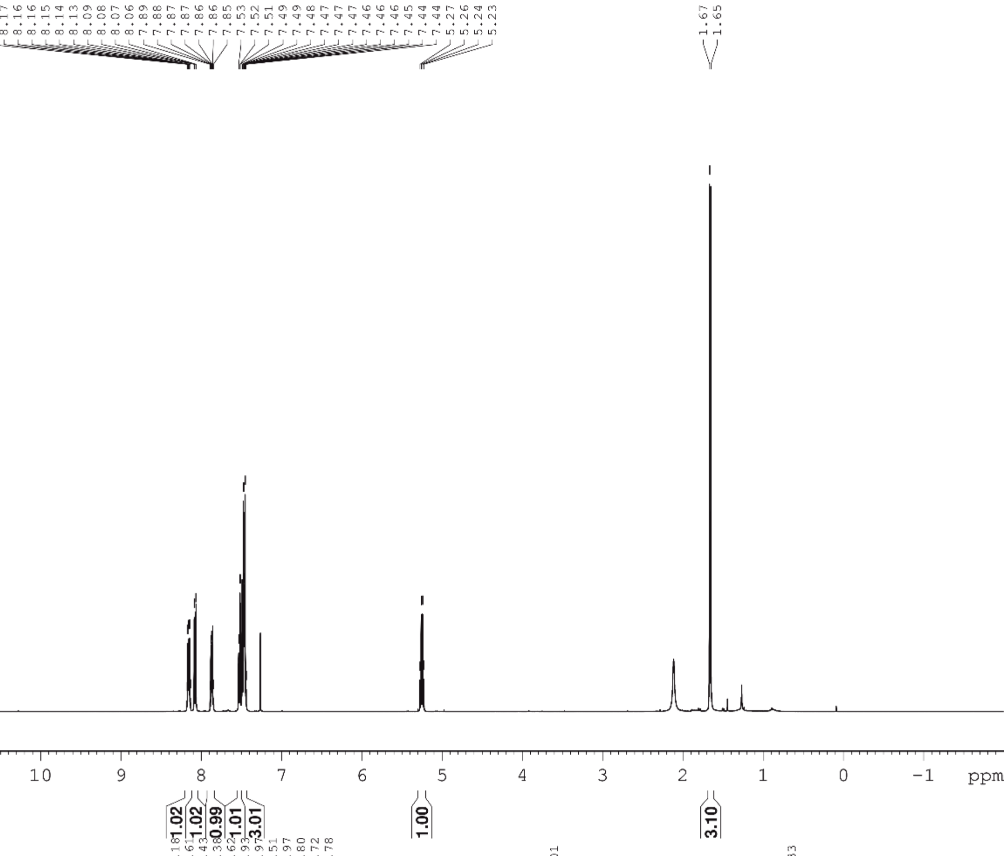


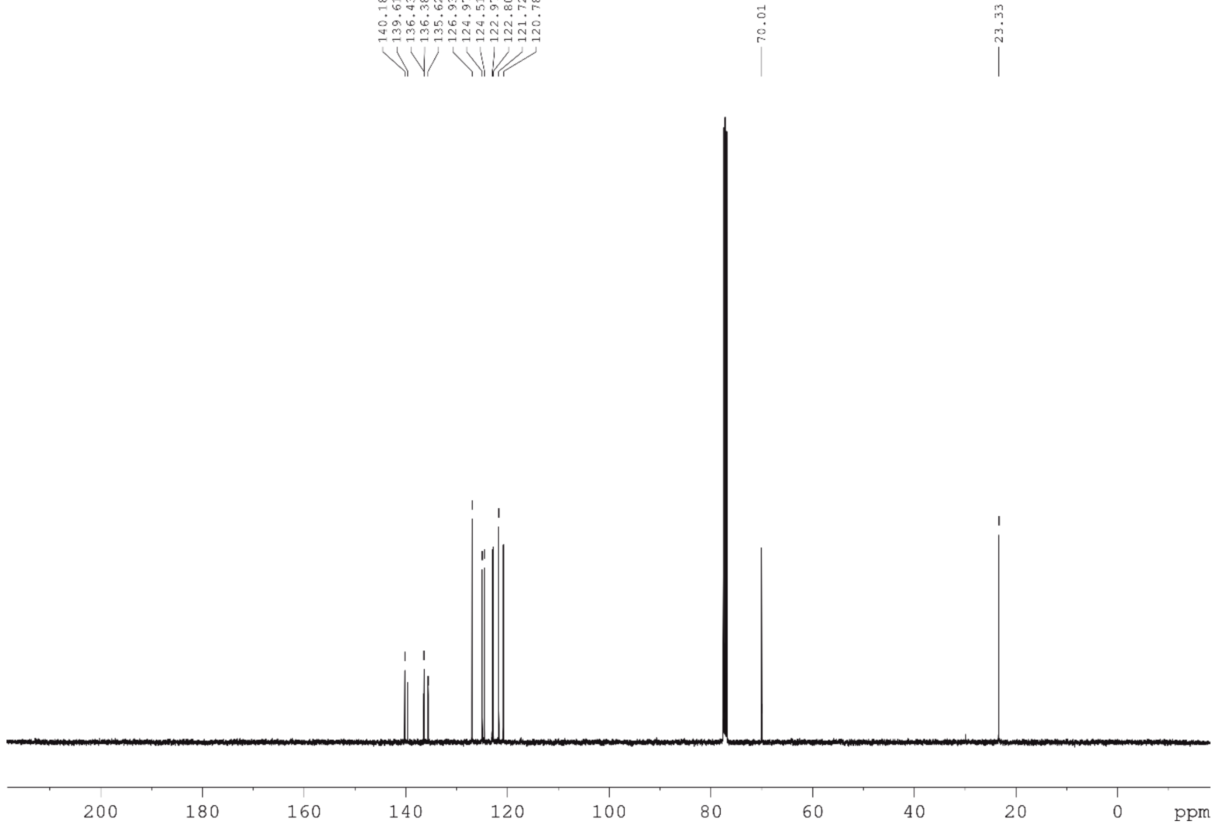

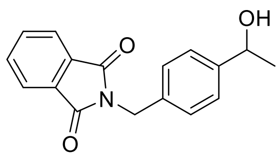


*rac*-**6ae**

**2-(4-(1-hydroxyethyl)benzyl)isoindoline-1,3-dione (6ae)**

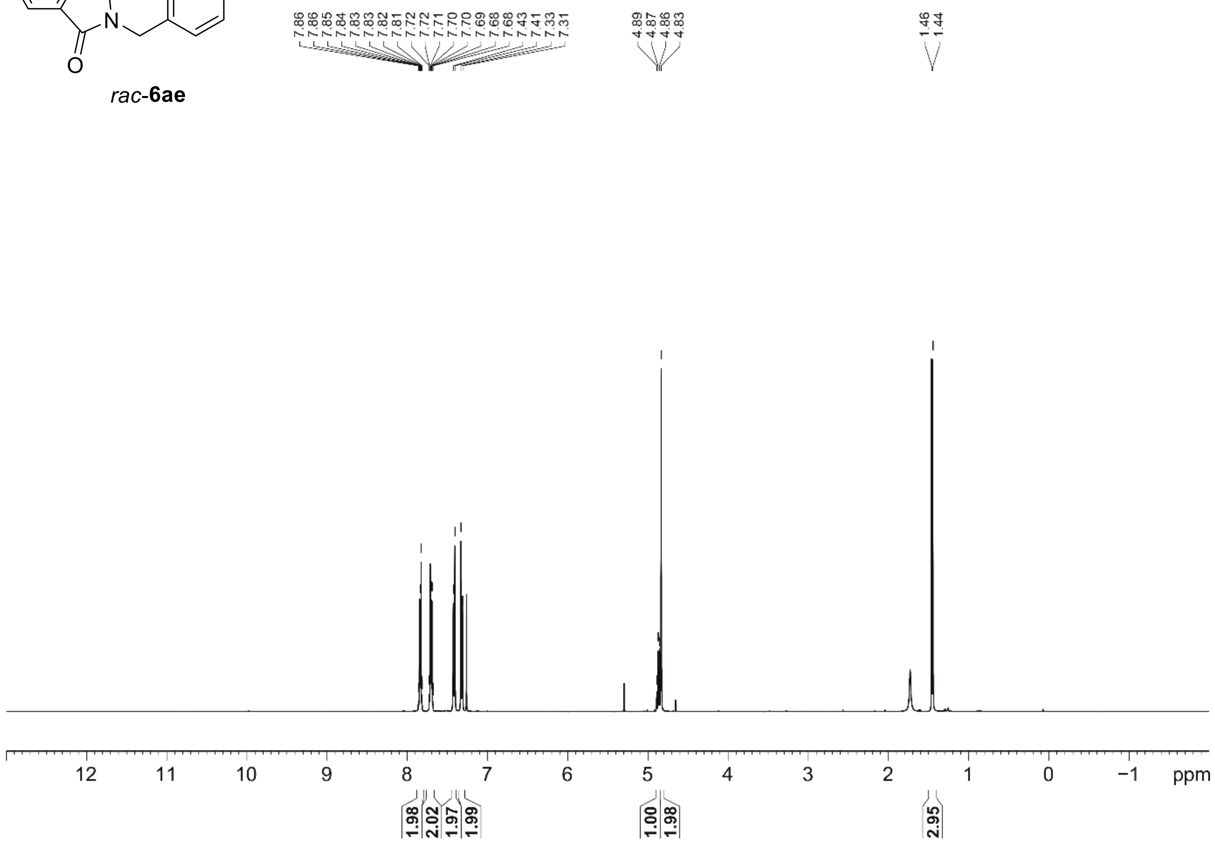


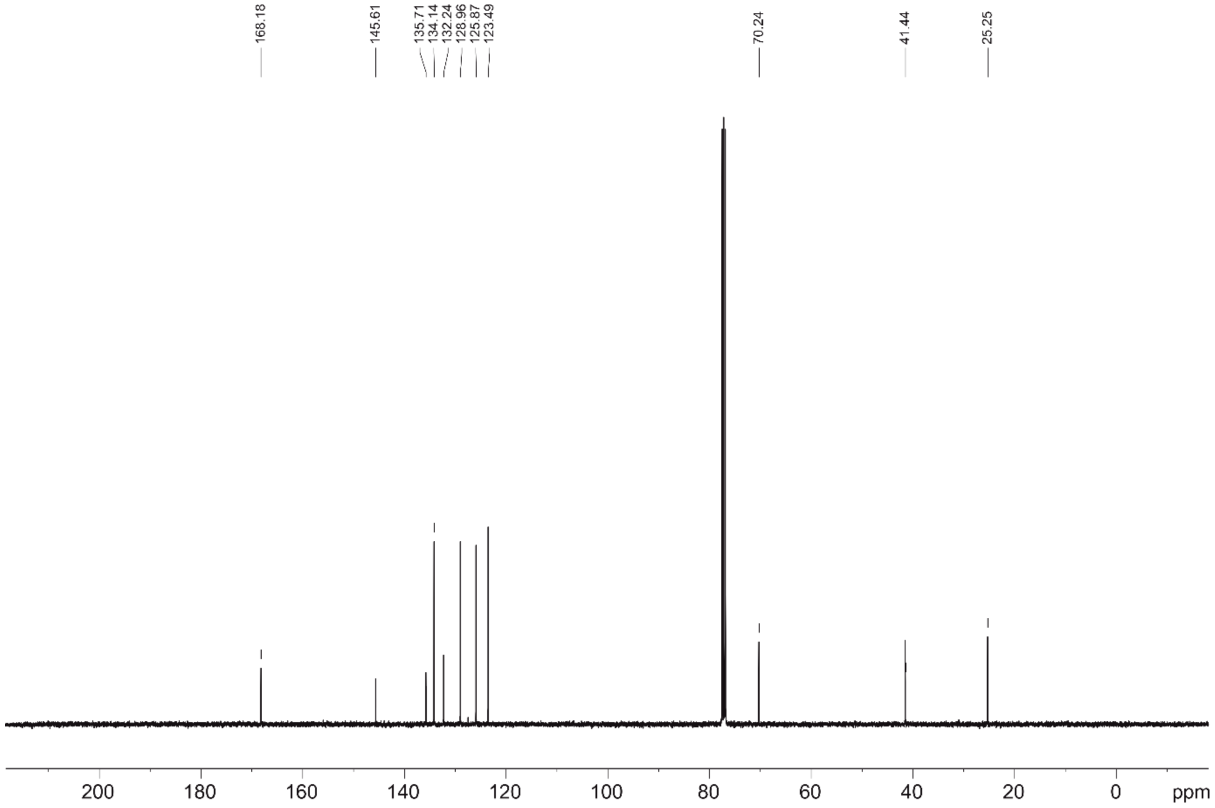

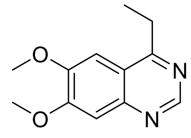

**6af**

**4-ethyl-6,7-dimethoxyquinazoline (6af)**

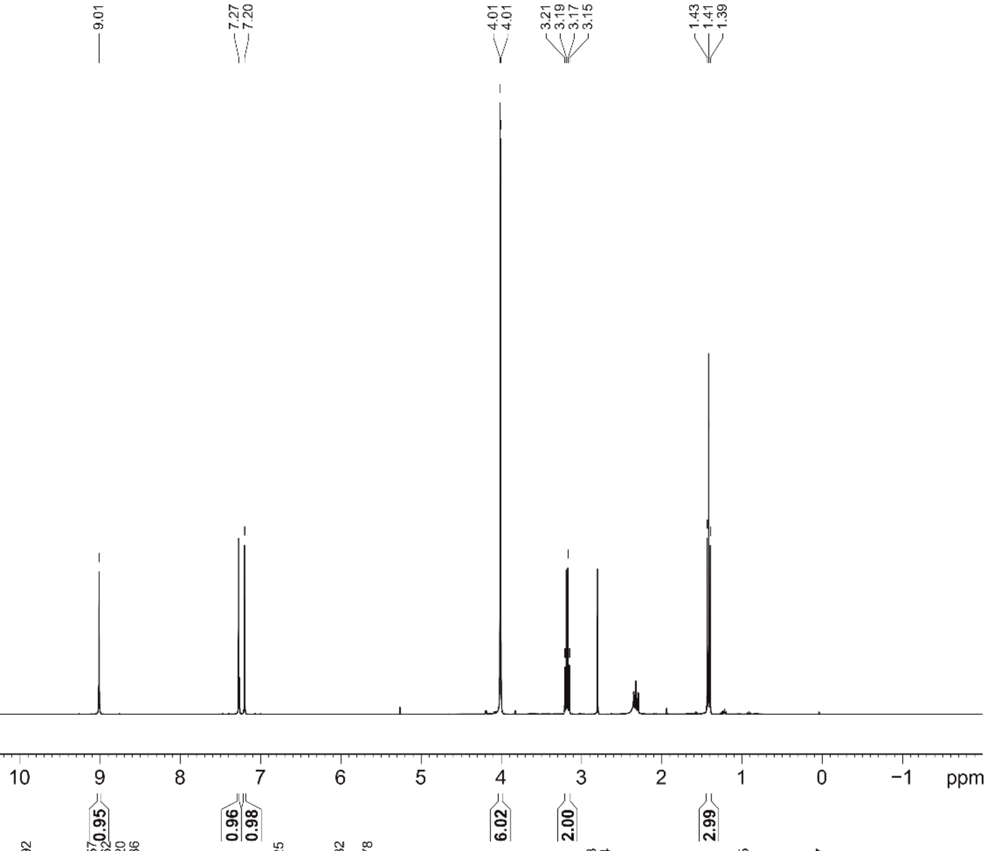


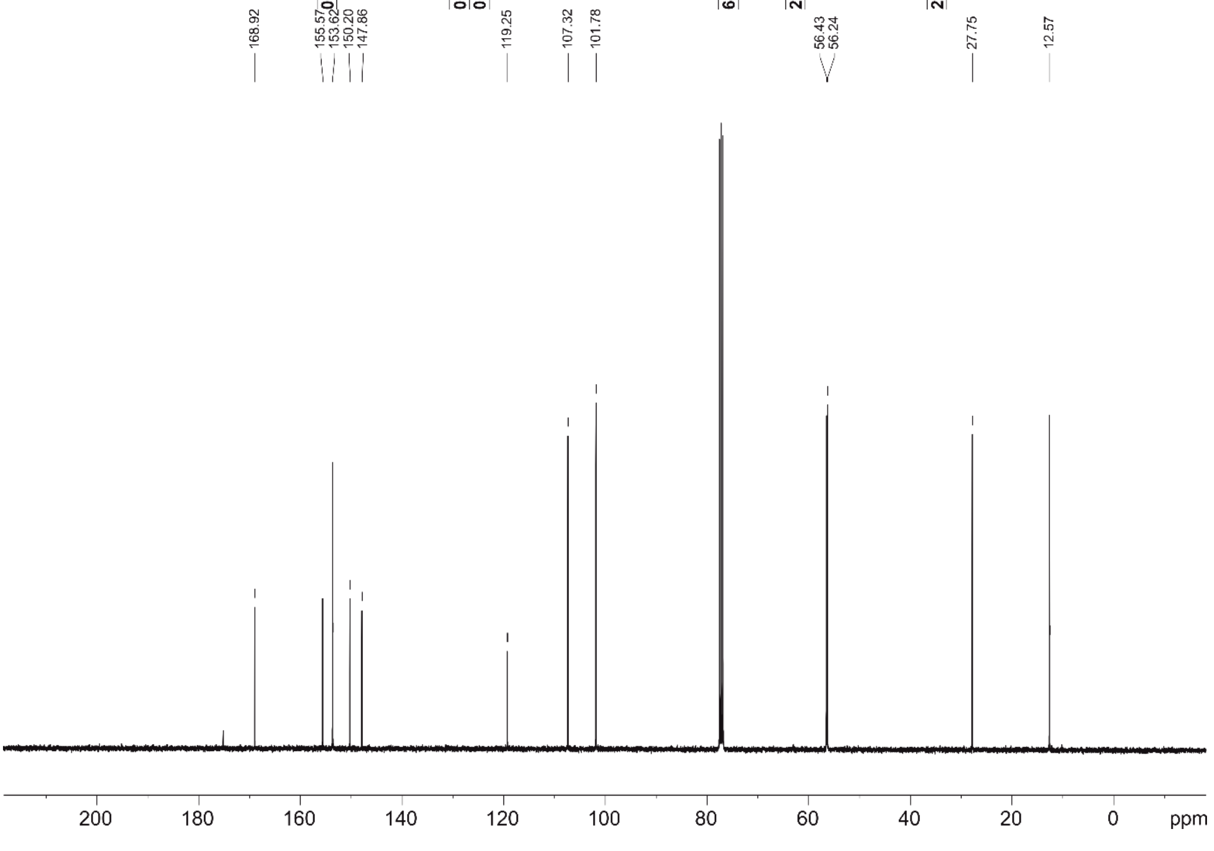

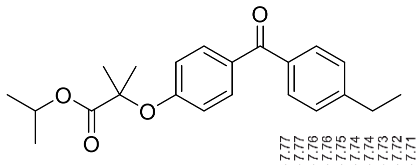

6ag

**Isopropyl-2-(4-(4-ethylbenzoyl)phenoxy-3-methylpropanoate (6ag)**

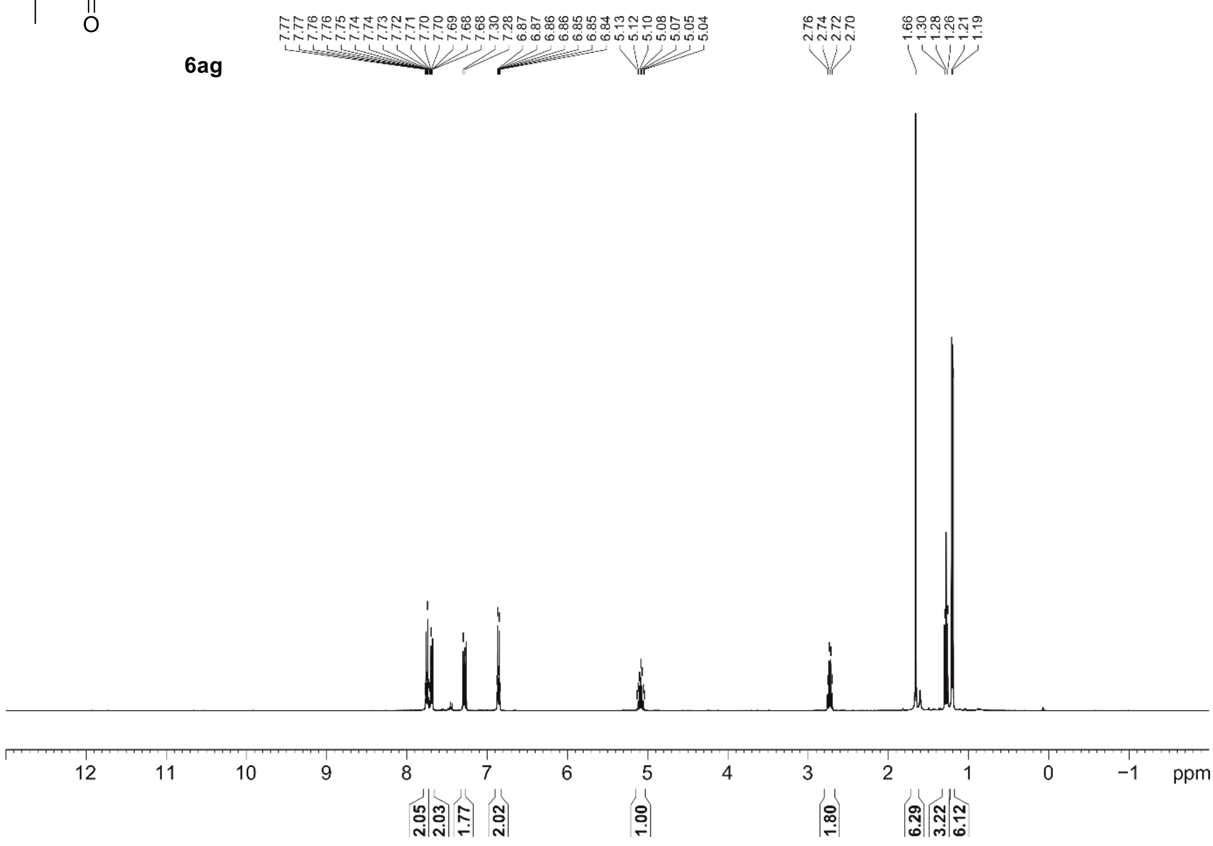

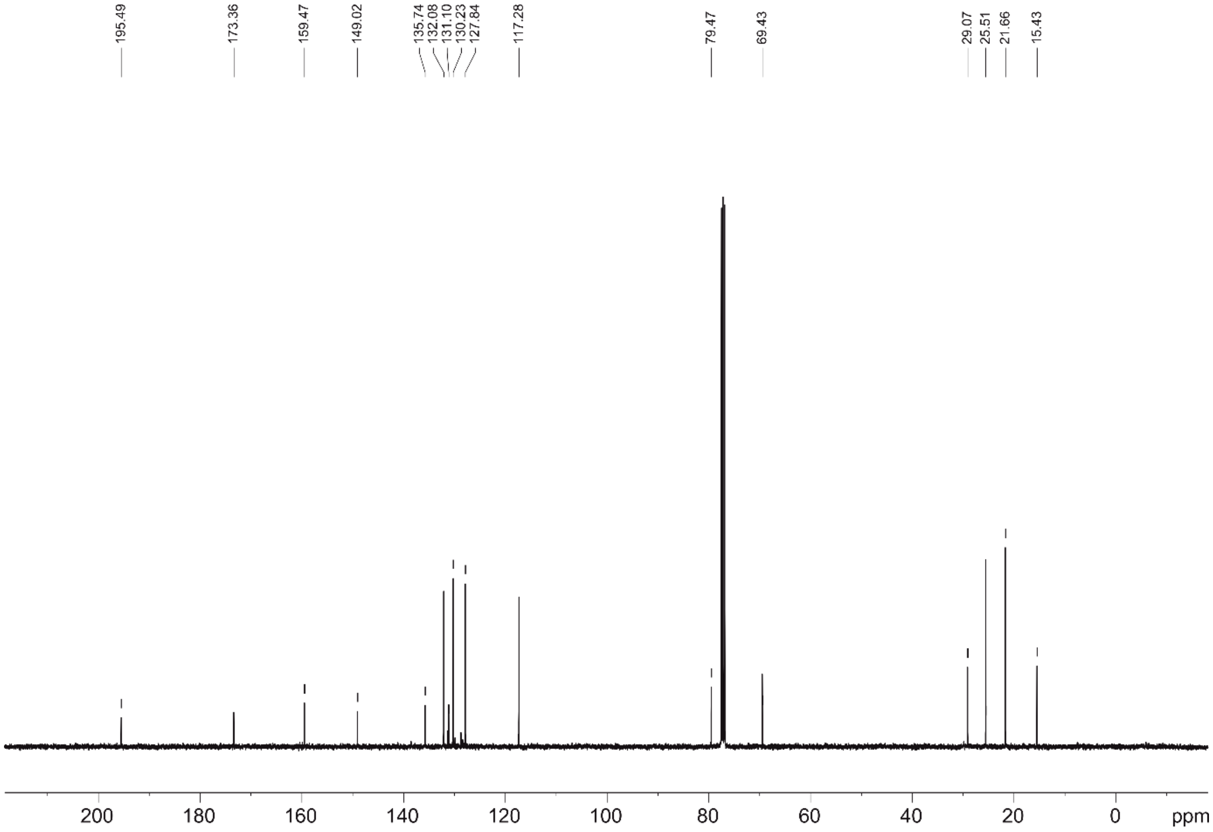

**2,2-dimethyl-1-(naphthalen-1-yl)propan-1-ol (6ak)**

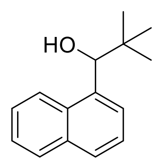


*rac*-**6ak**

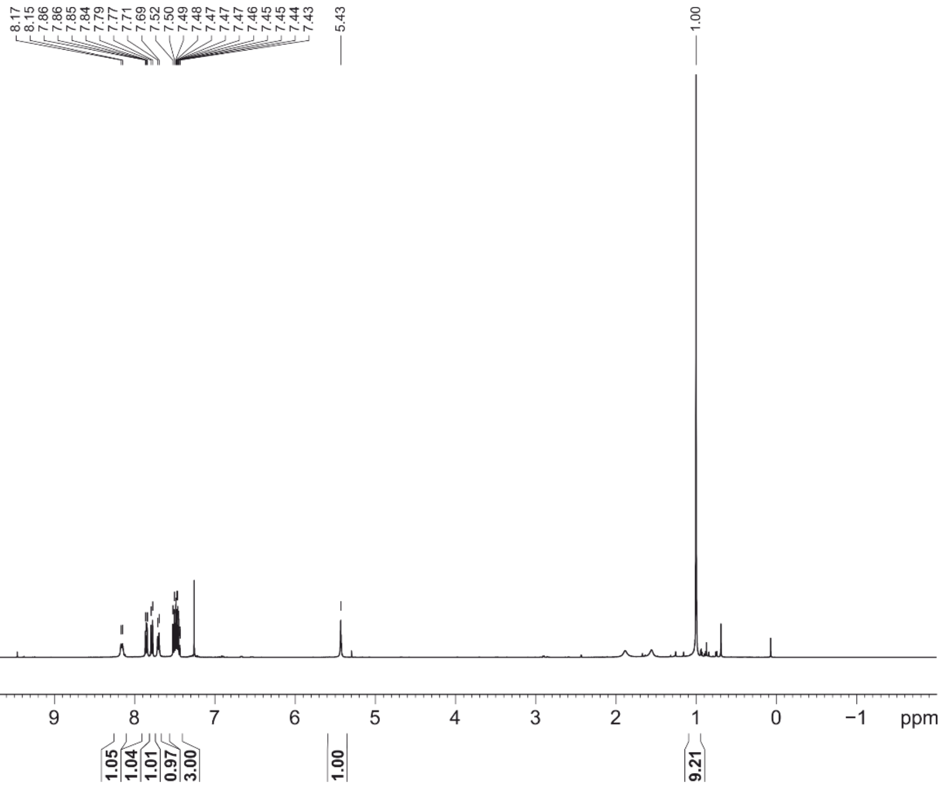


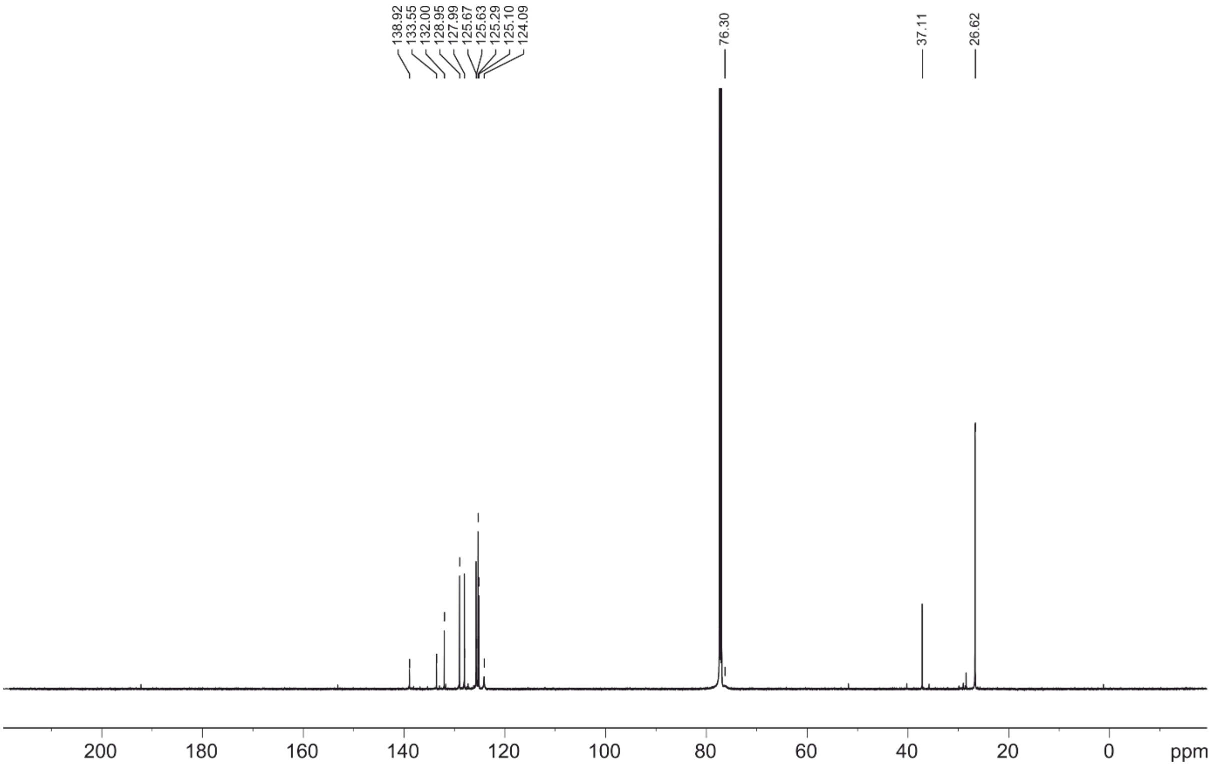

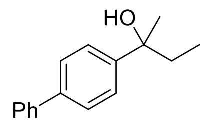

*rac*-**6al**

**2-([1,1'-biphenyl]-4-yl)butan-2-ol (6al)**

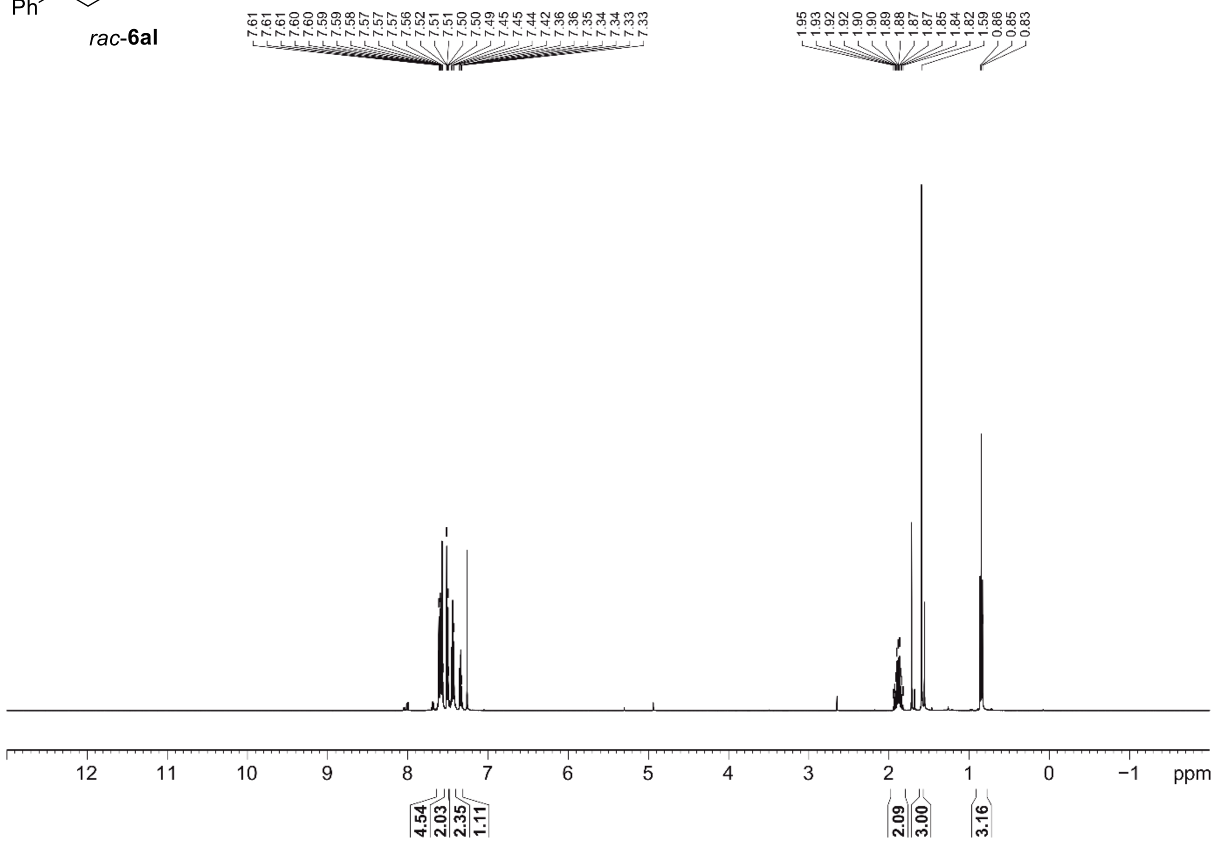


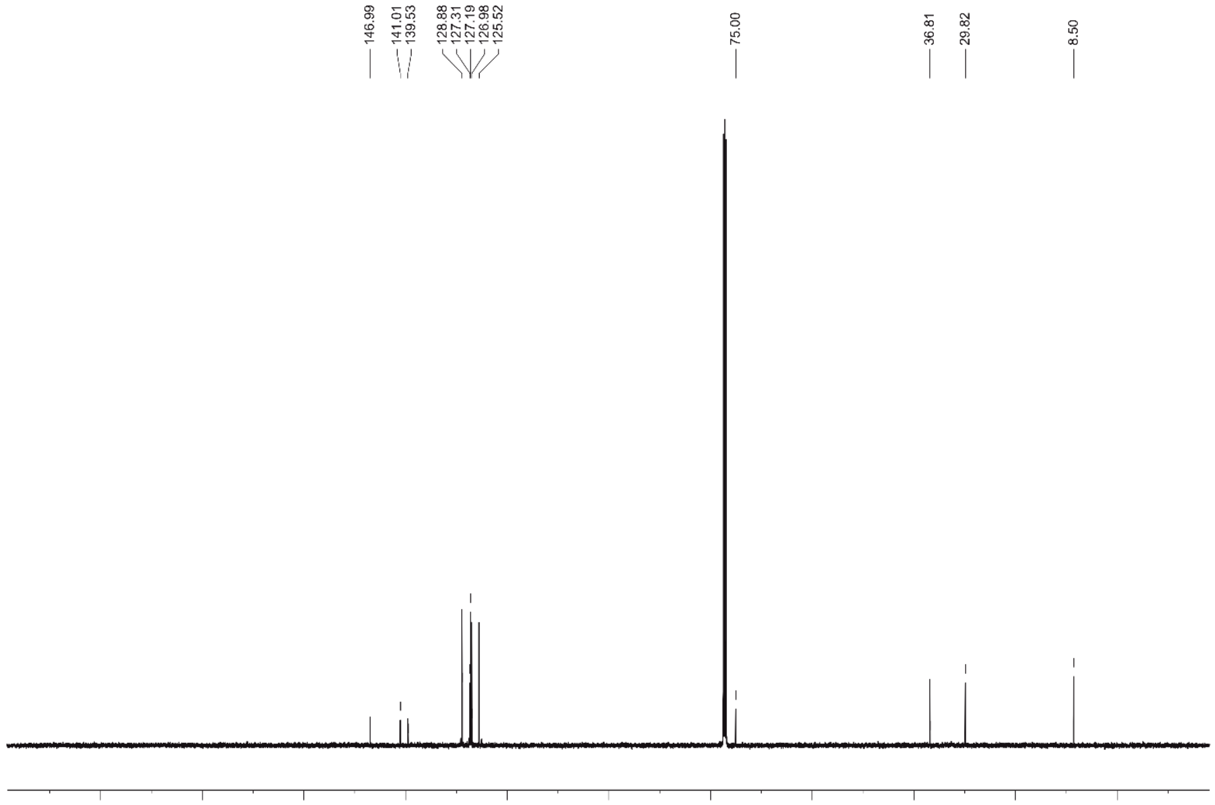

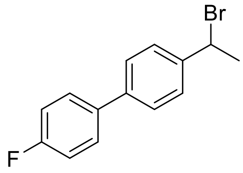


*rac*-**1b**

**4-(1-bromoethyl)-4'-fluoro-1,1'biphenyl (1b)**

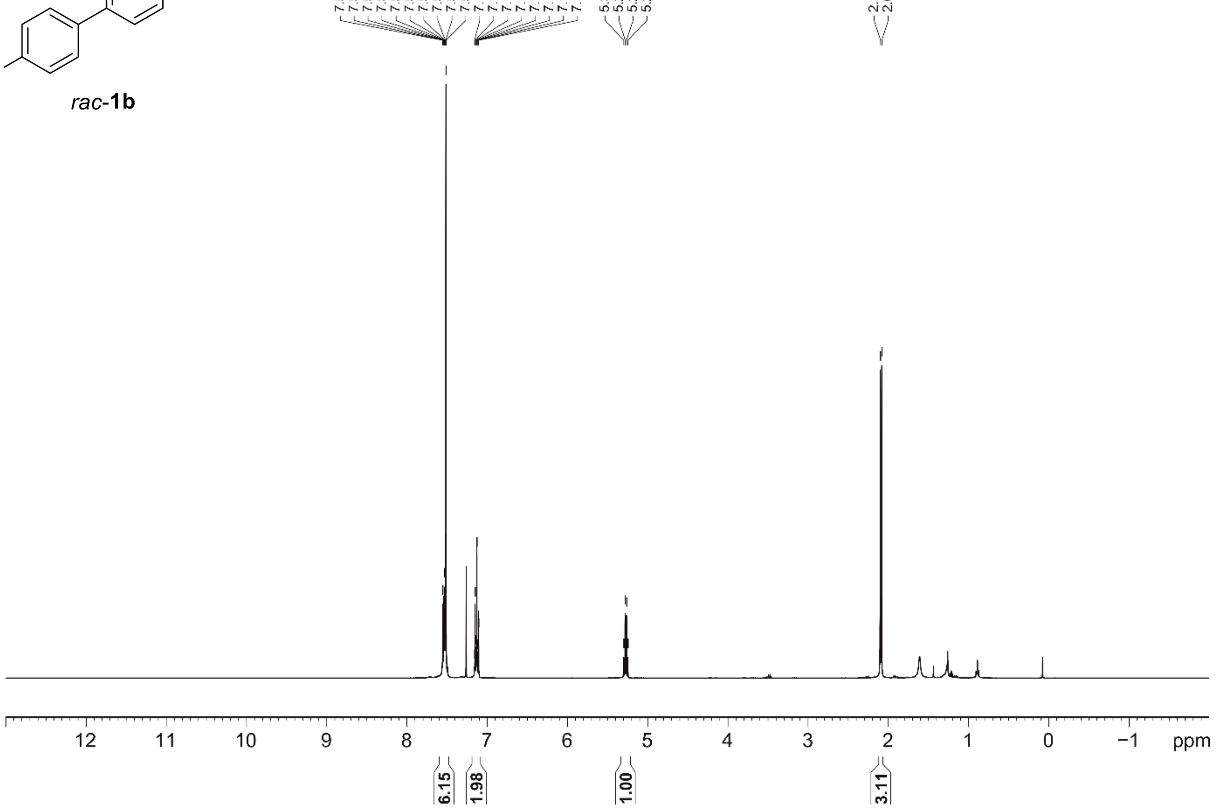


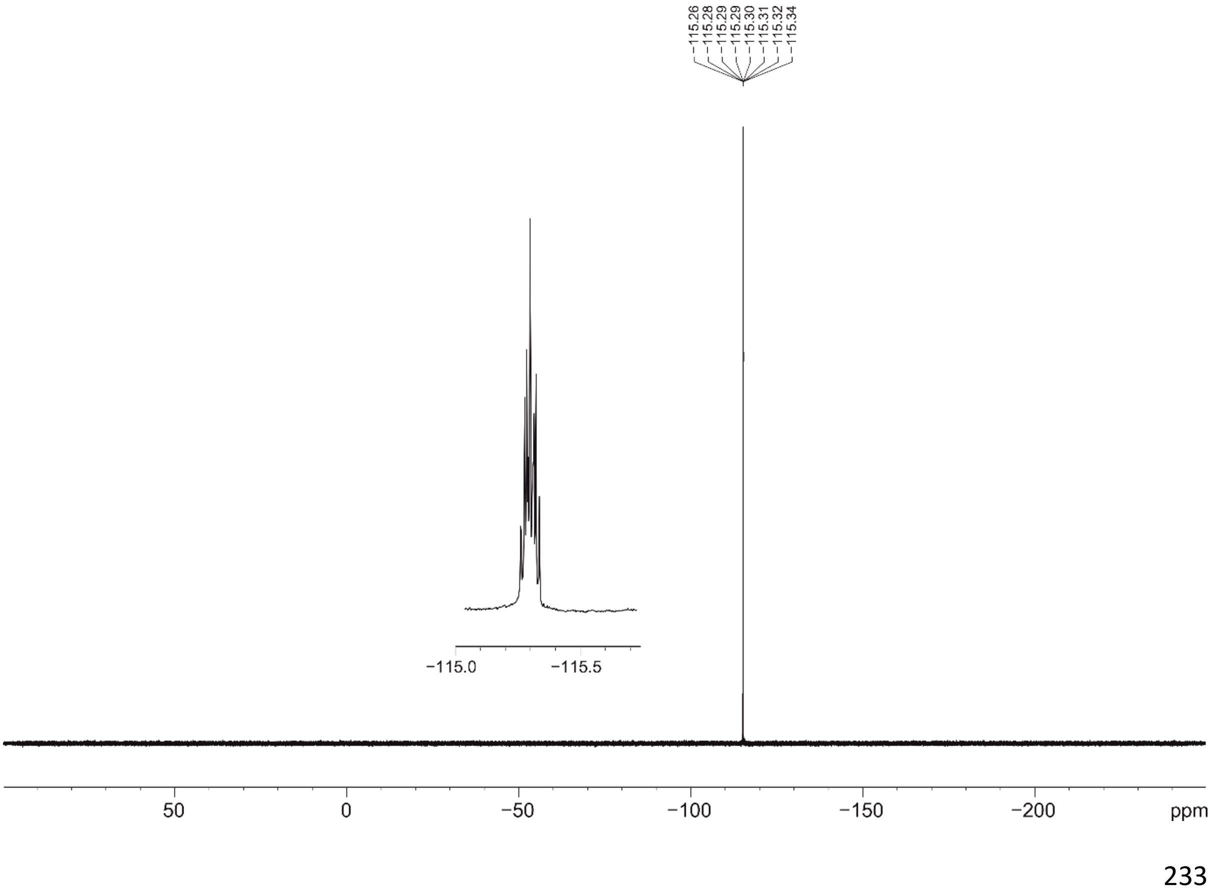

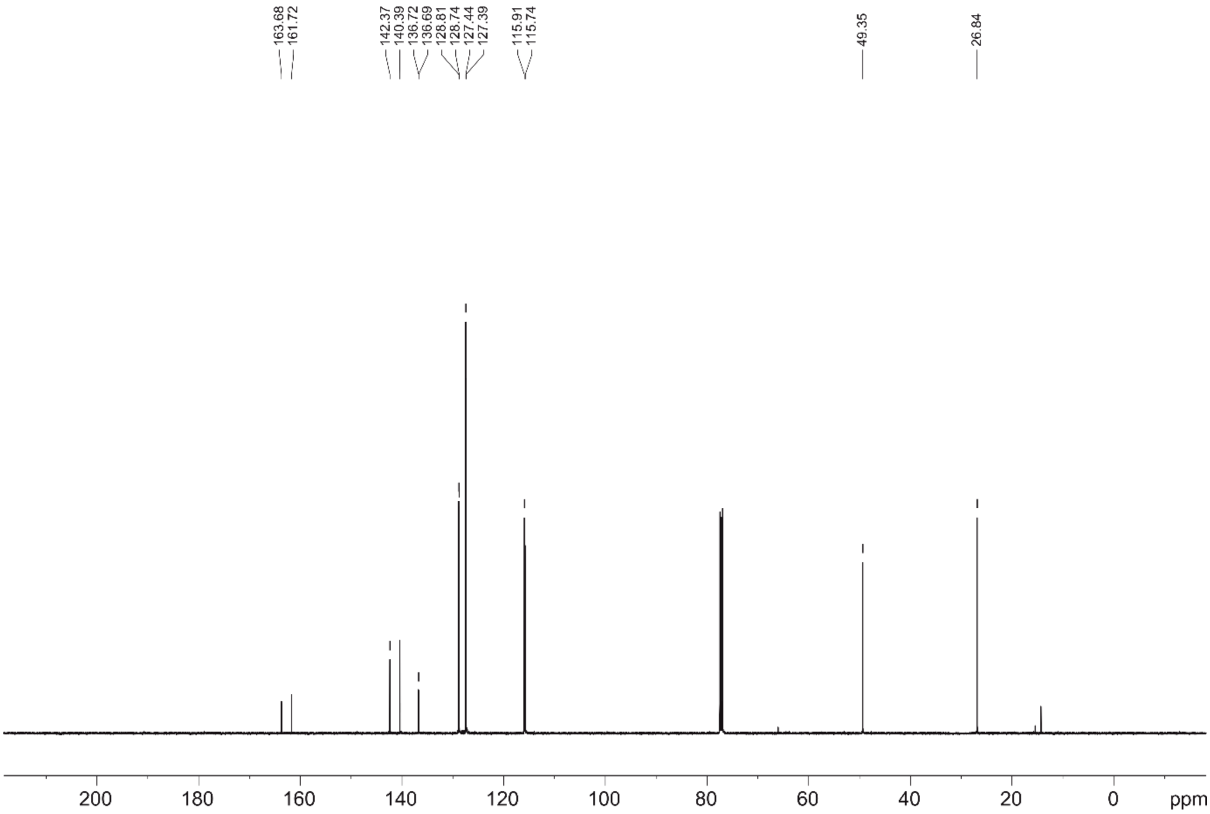


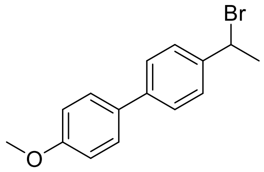


*rac*-**1c**

**4-(1-bromoethyl)-4'-methoxy-1,1'-biphenyl (1c)**

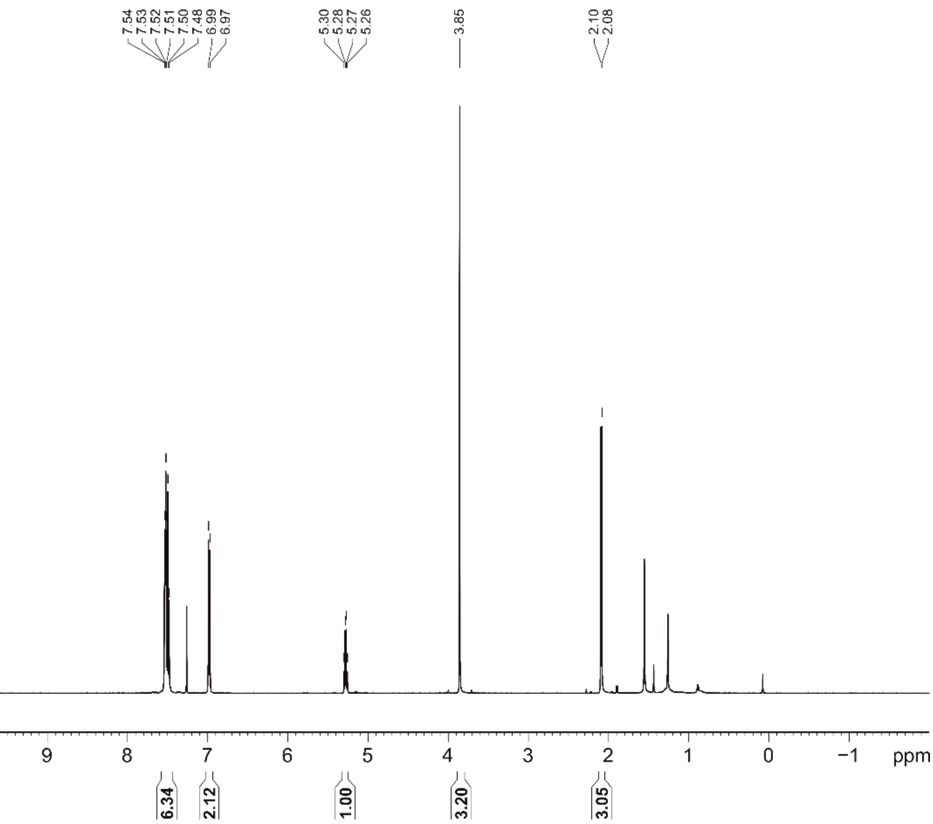

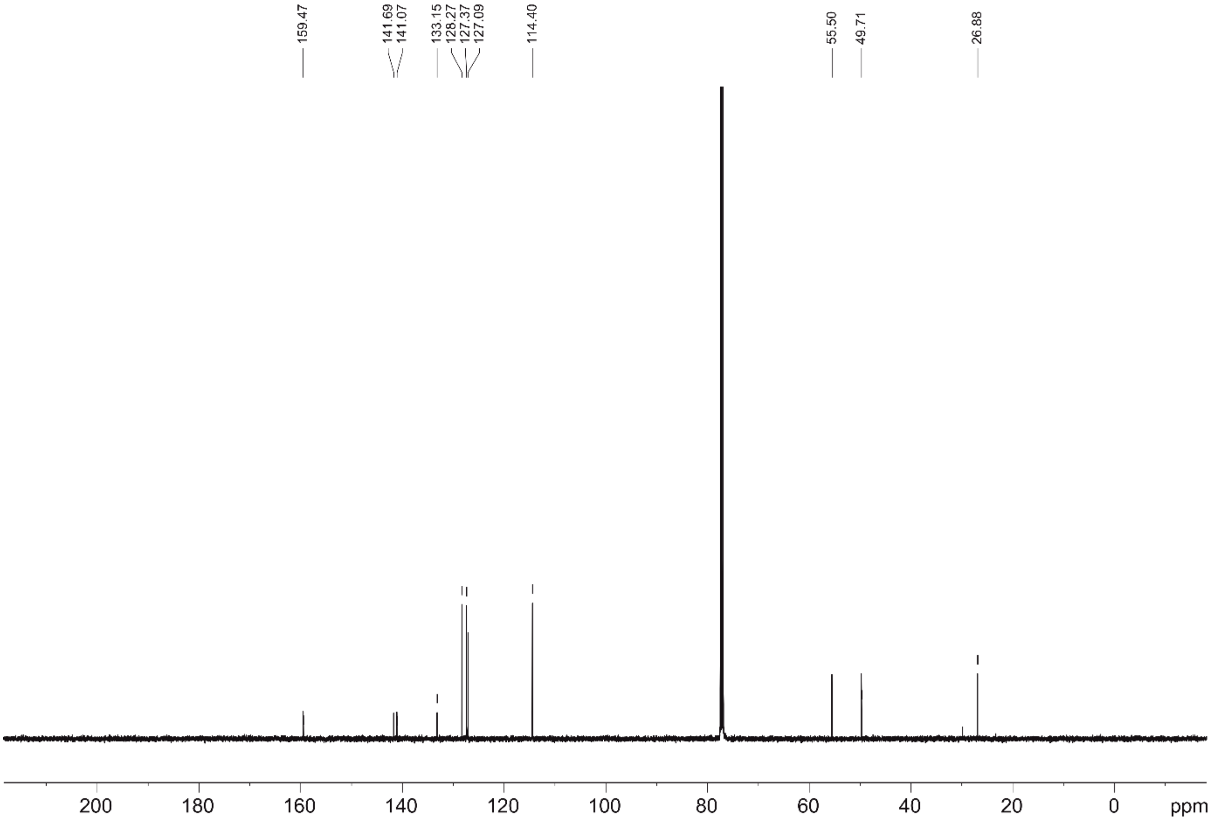


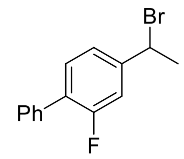


*rac*-**1d**

**4-(1-bromoethyl)-2-fluoro-1,1'-biphenyl (1d)**

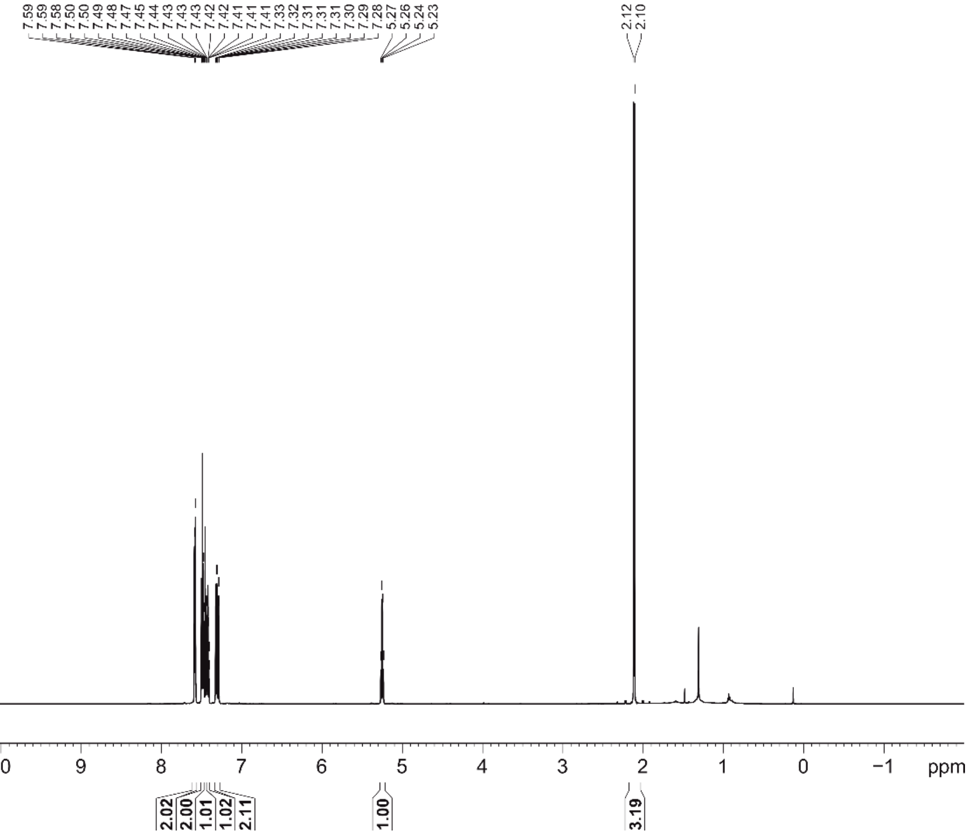

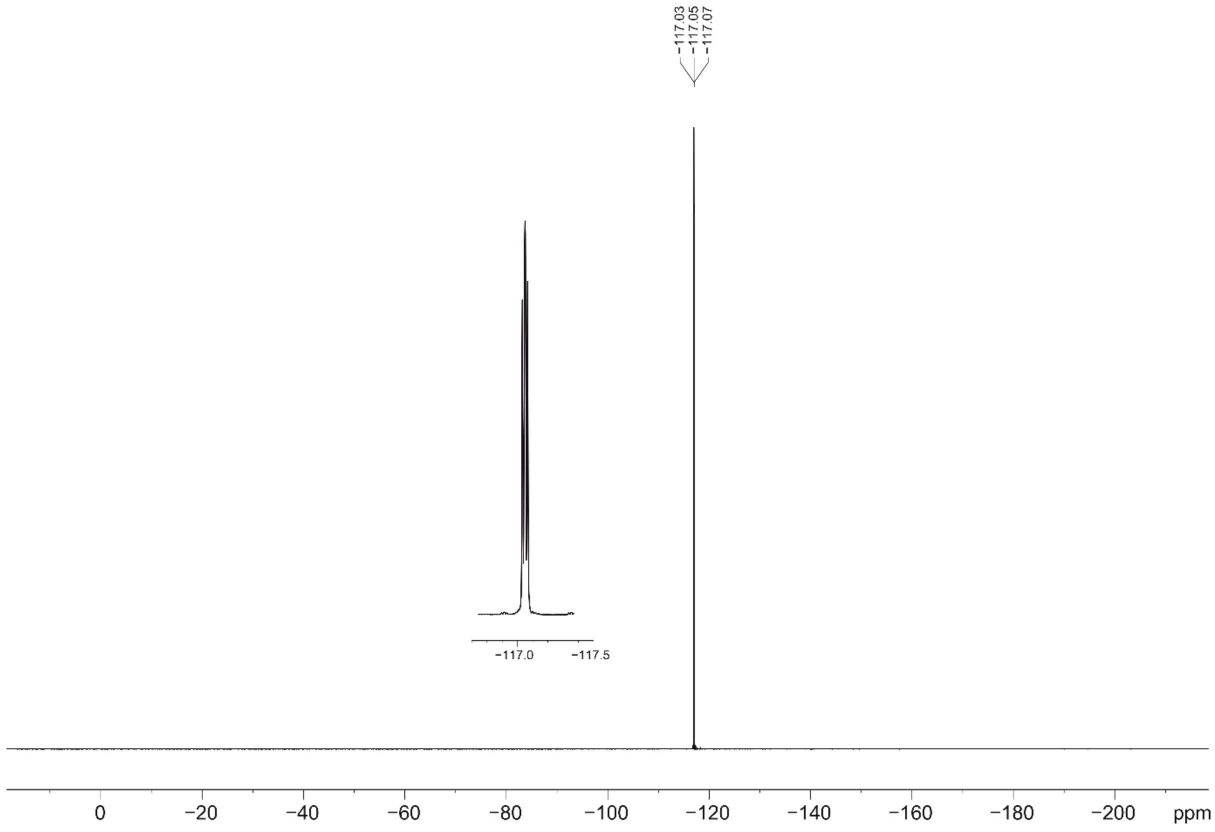
-117.03
-117.05
-117.07
-117.0
-117.5
0
−20
−40
−60
−80
−100
−120
−140
−160
−180
−200
ppm

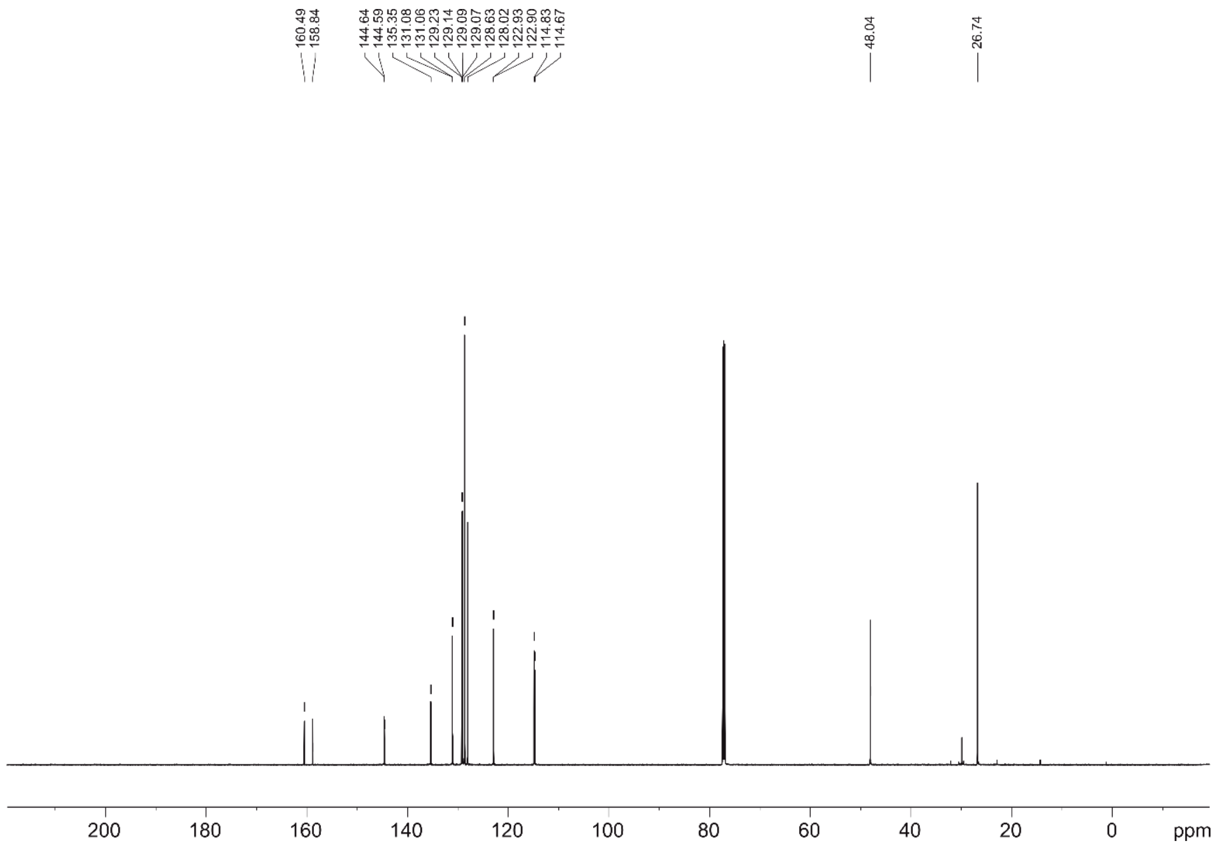
160.49
158.84
144.64
144.59
135.35
131.08
131.06
129.23
129.14
129.09
129.07
128.63
128.02
122.93
122.90
114.83
114.67
48.04
26.74
200
180
160
140
120
100
80
60
40
20
0
ppm

**2-(4-(1-bromoethyl)phenyl)pyridine (1e)**

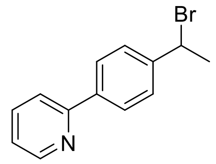

*rac*-**1e**

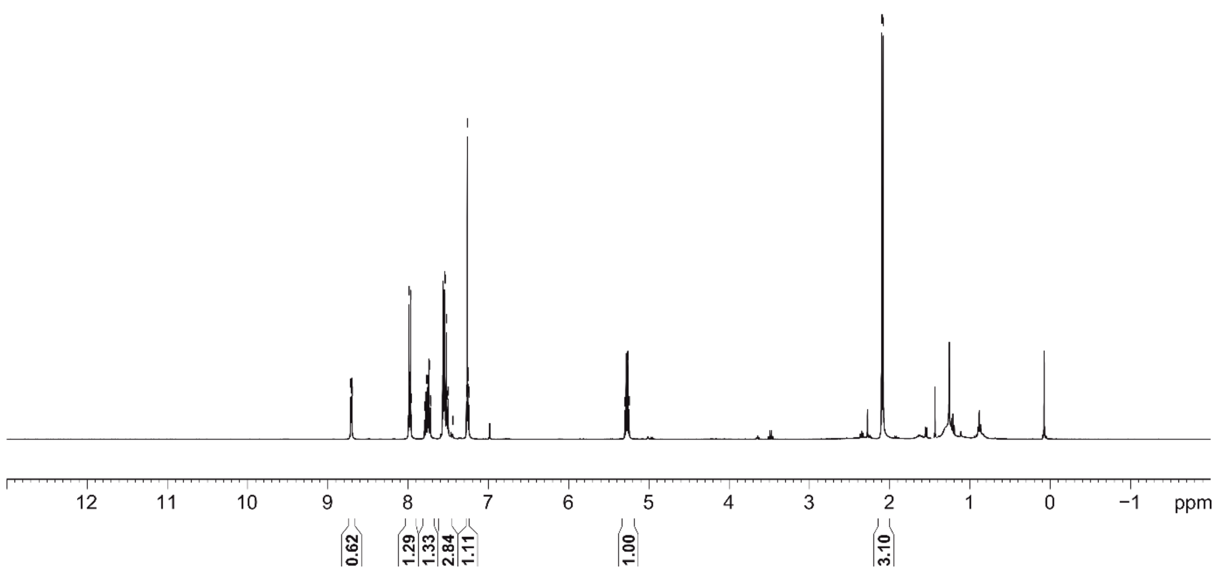

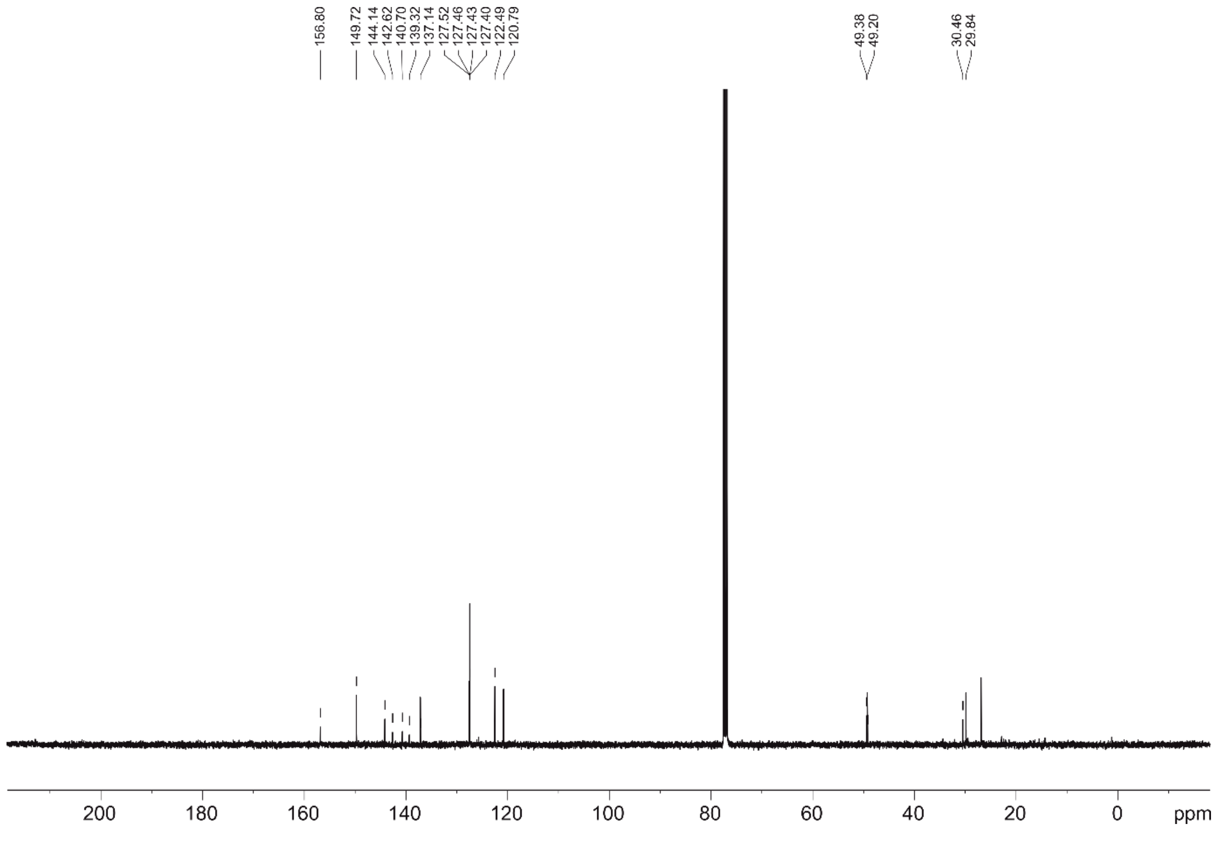

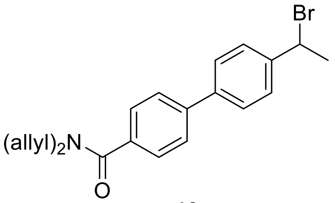


*rac*-**1f**

***N,N*-diallyl-4′-(1-bromoethyl)-[1,1′-biphenyl]-4-carboxamide (1f)**

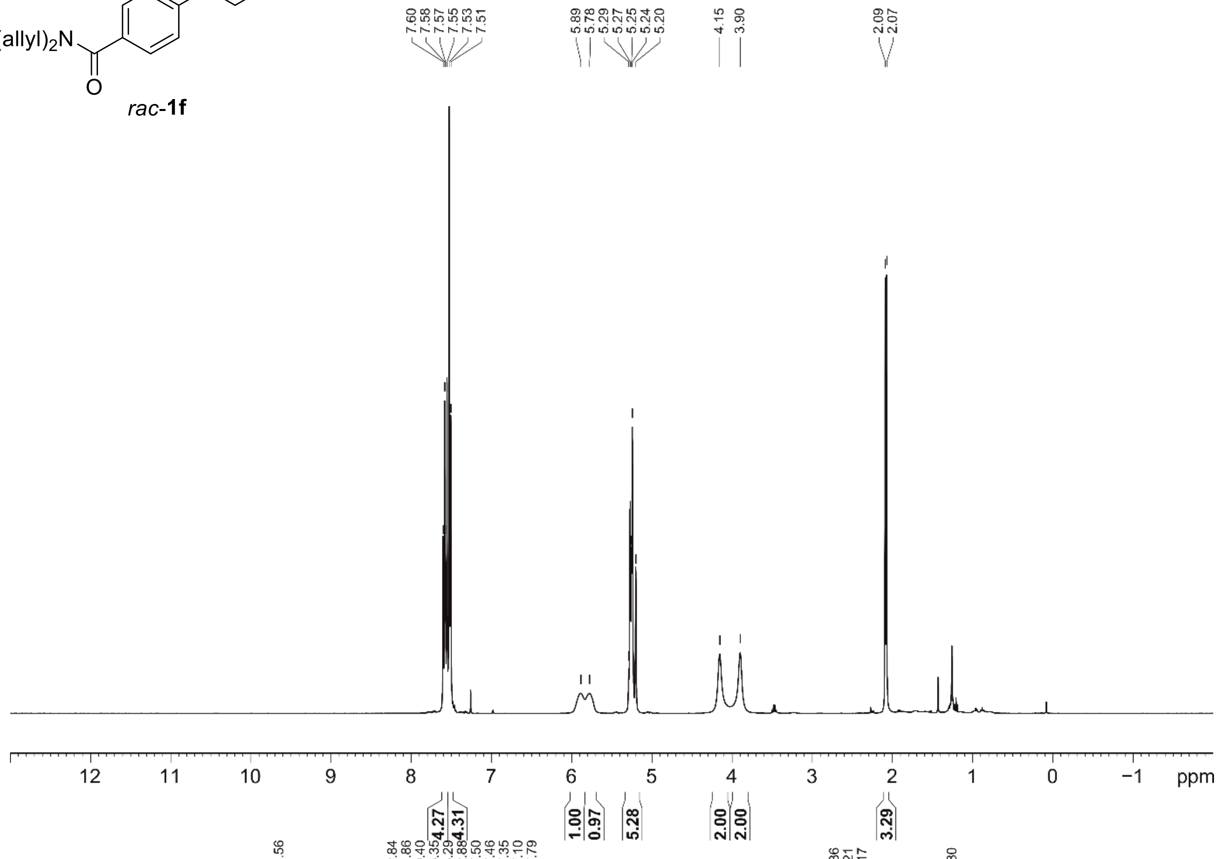


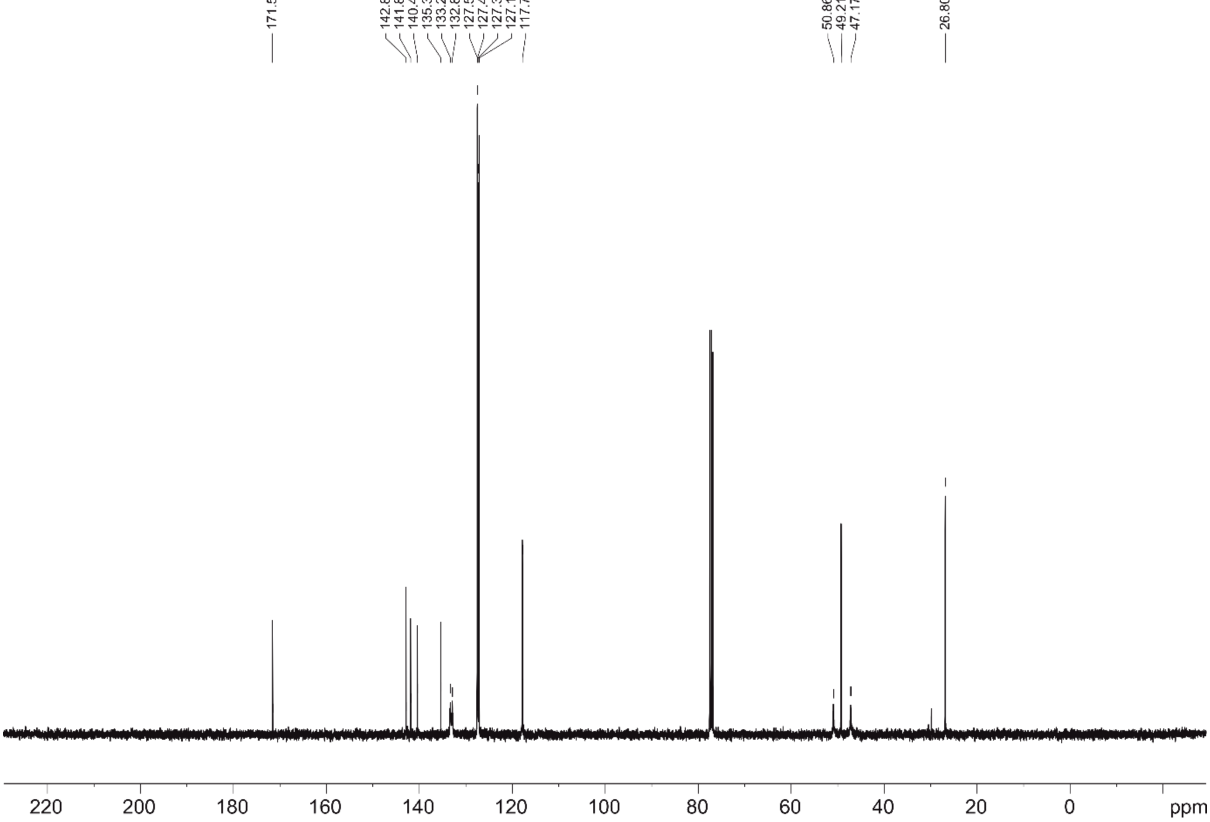

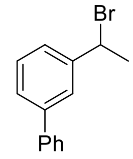


*rac*-**1g**

**3-(1-bromoethyl)-1,1'-biphenyl (1g)**

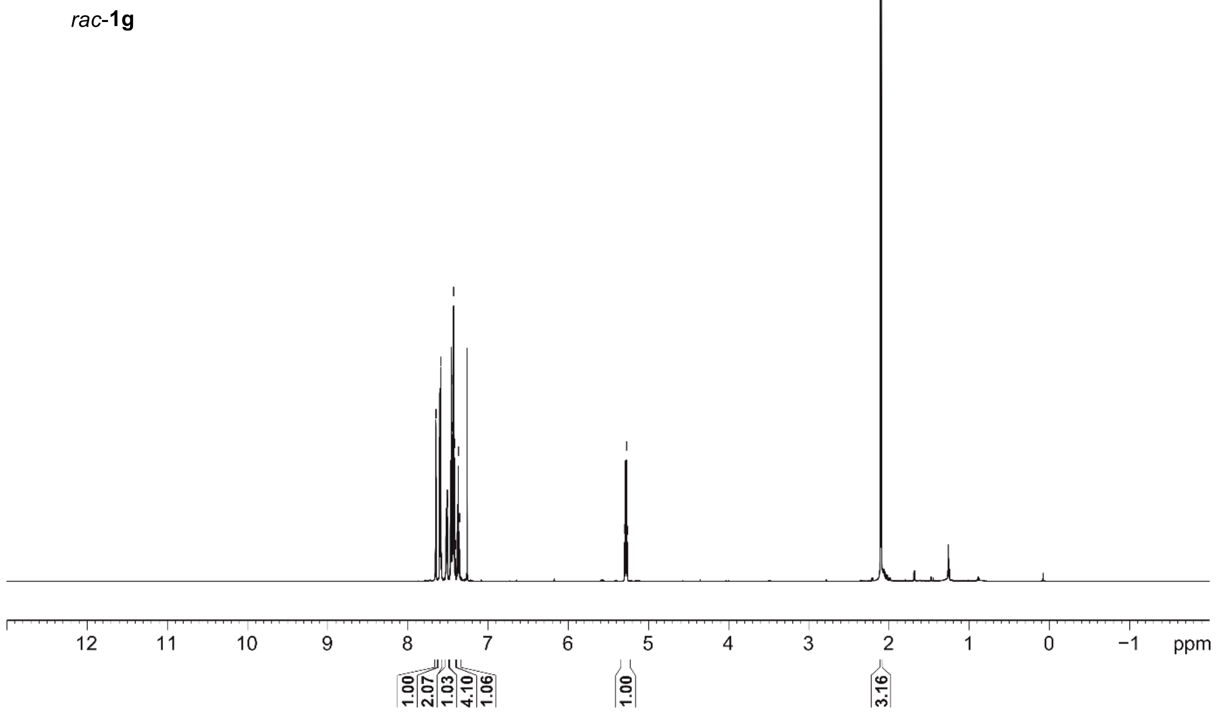


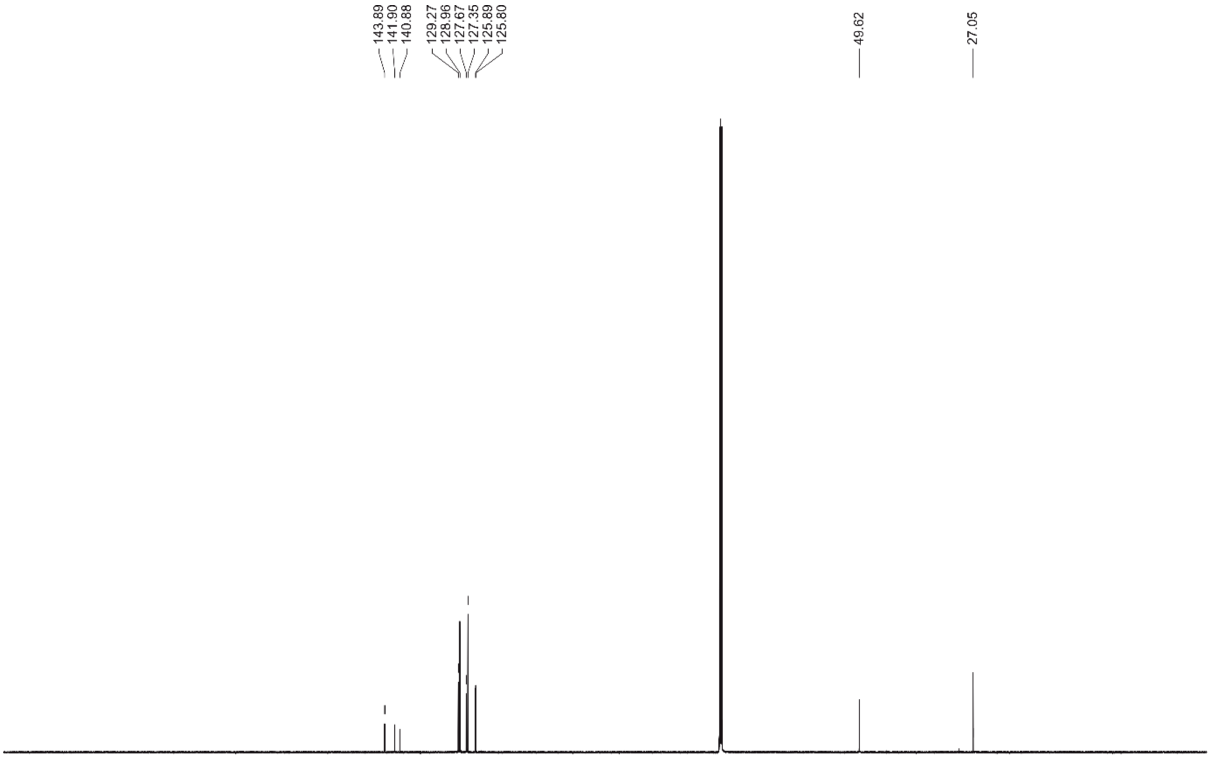

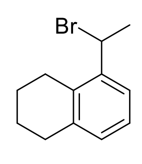


*rac*-**1i**

**5-(1-bromoethyl)-1,2,3,4-tetrahydronaphthalene (1i)**

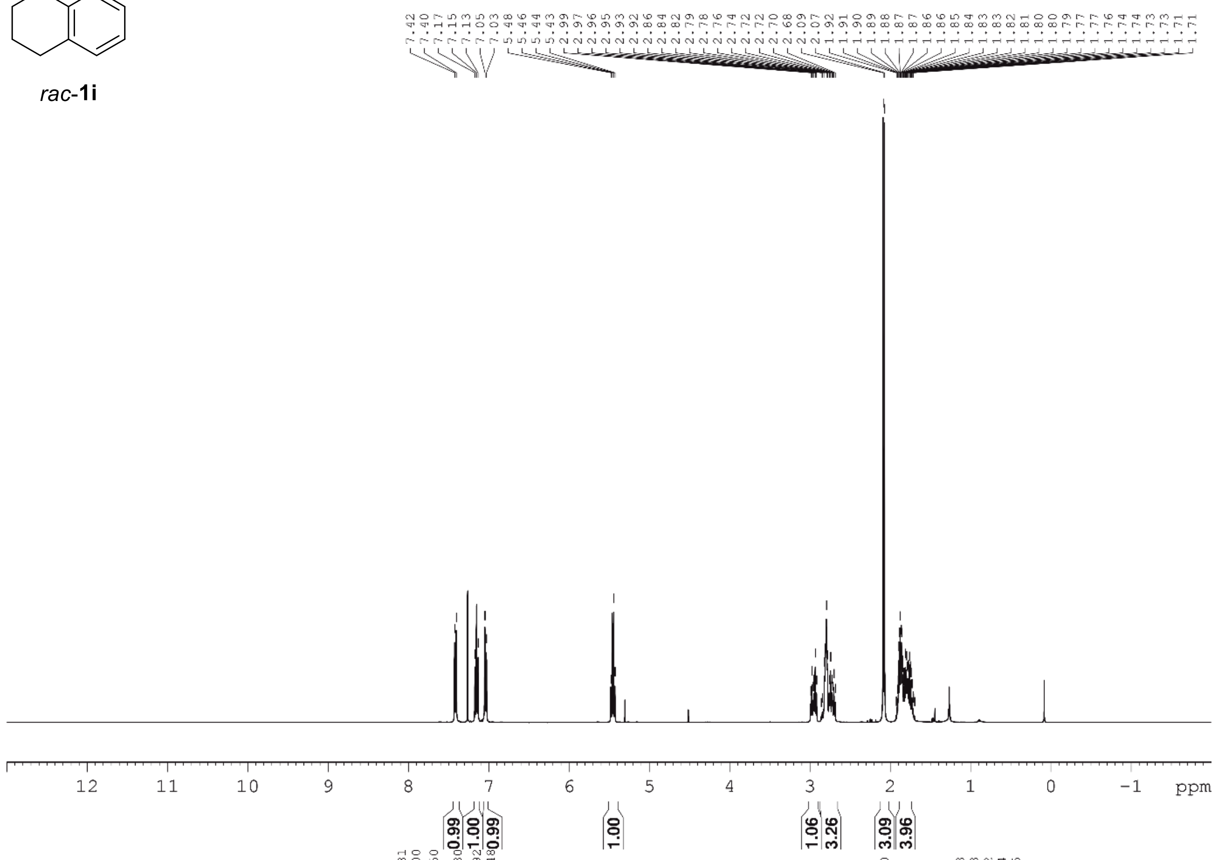


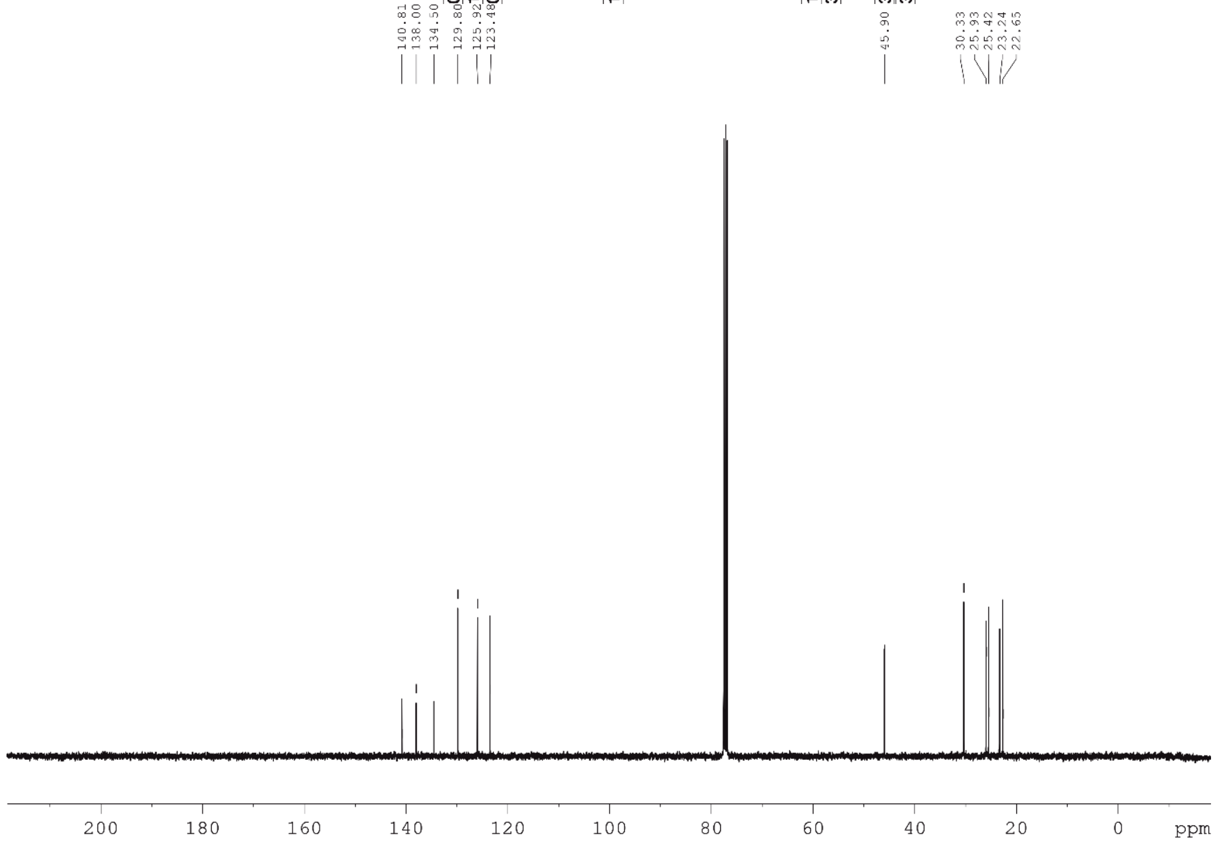

**9-(1-bromoethyl)phenanthrene (1j)**

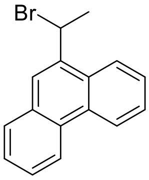


*rac*-**1j**

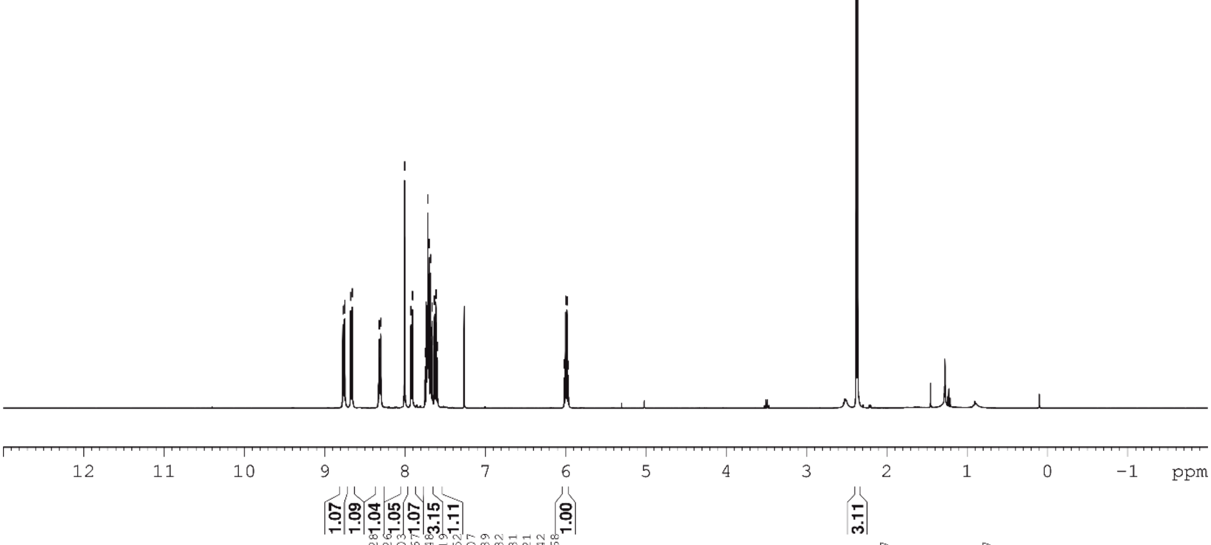


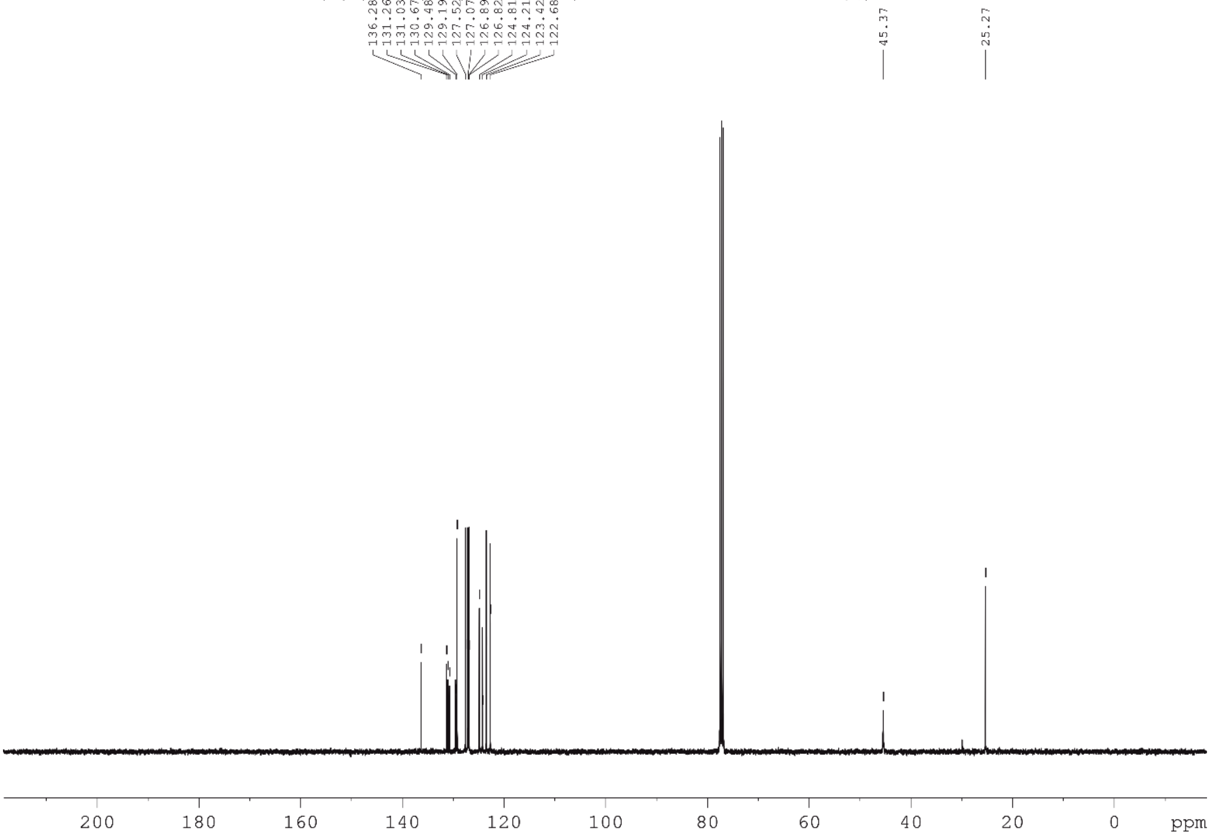

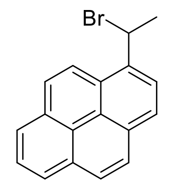

*rac*-**1k**

**1-(1-bromoethyl)pyrene (1k)**

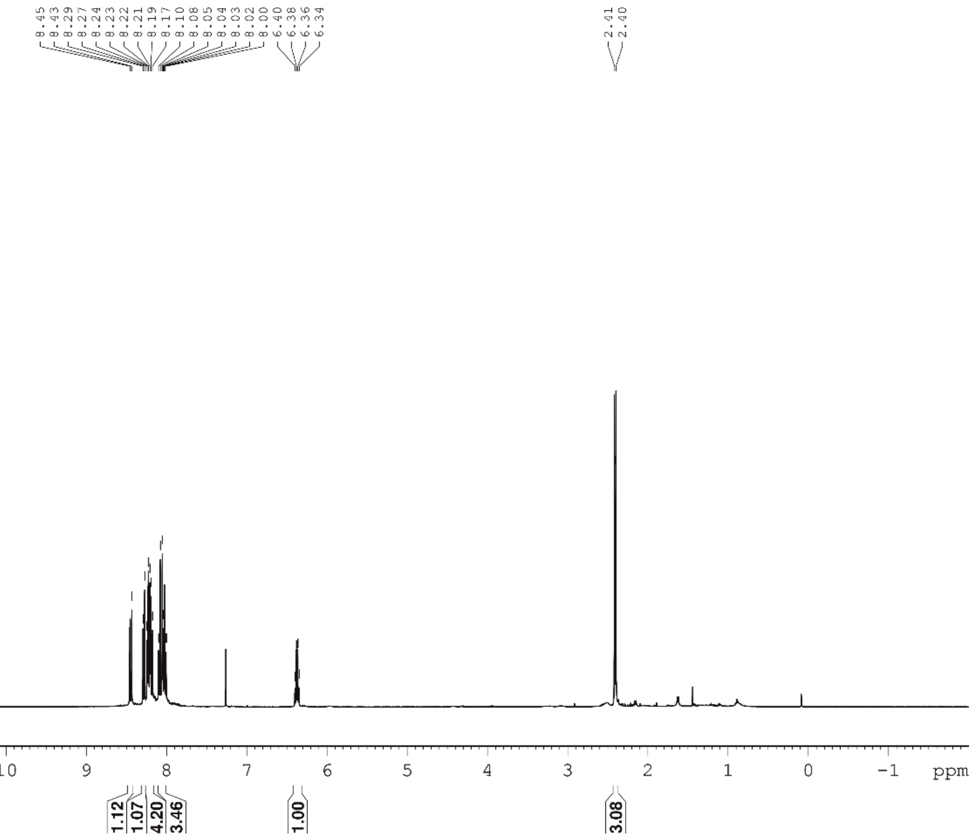


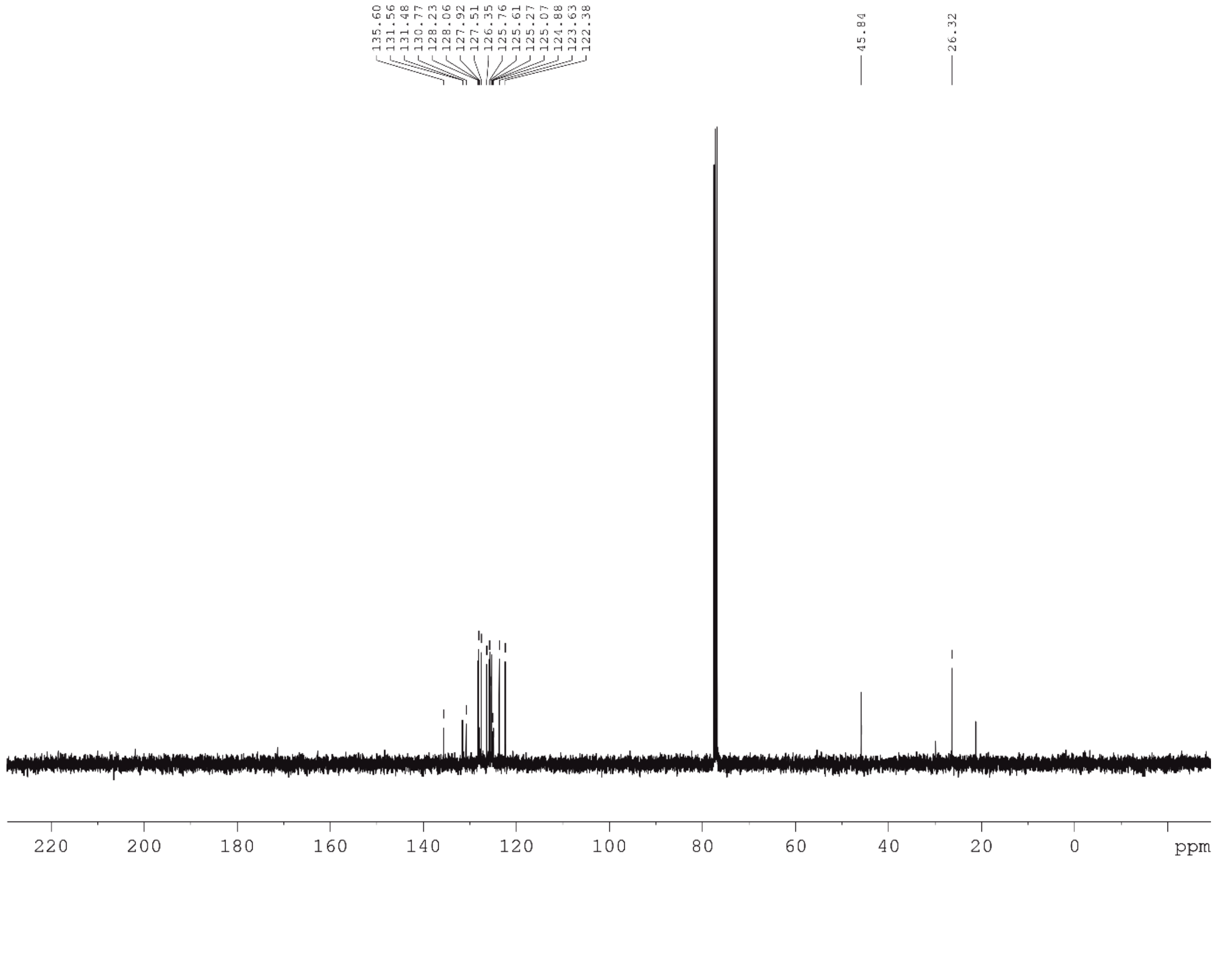

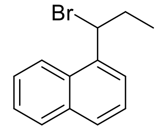


*rac*-**1l**

**1-(1-bromopropyl)naphthalene (1l)**

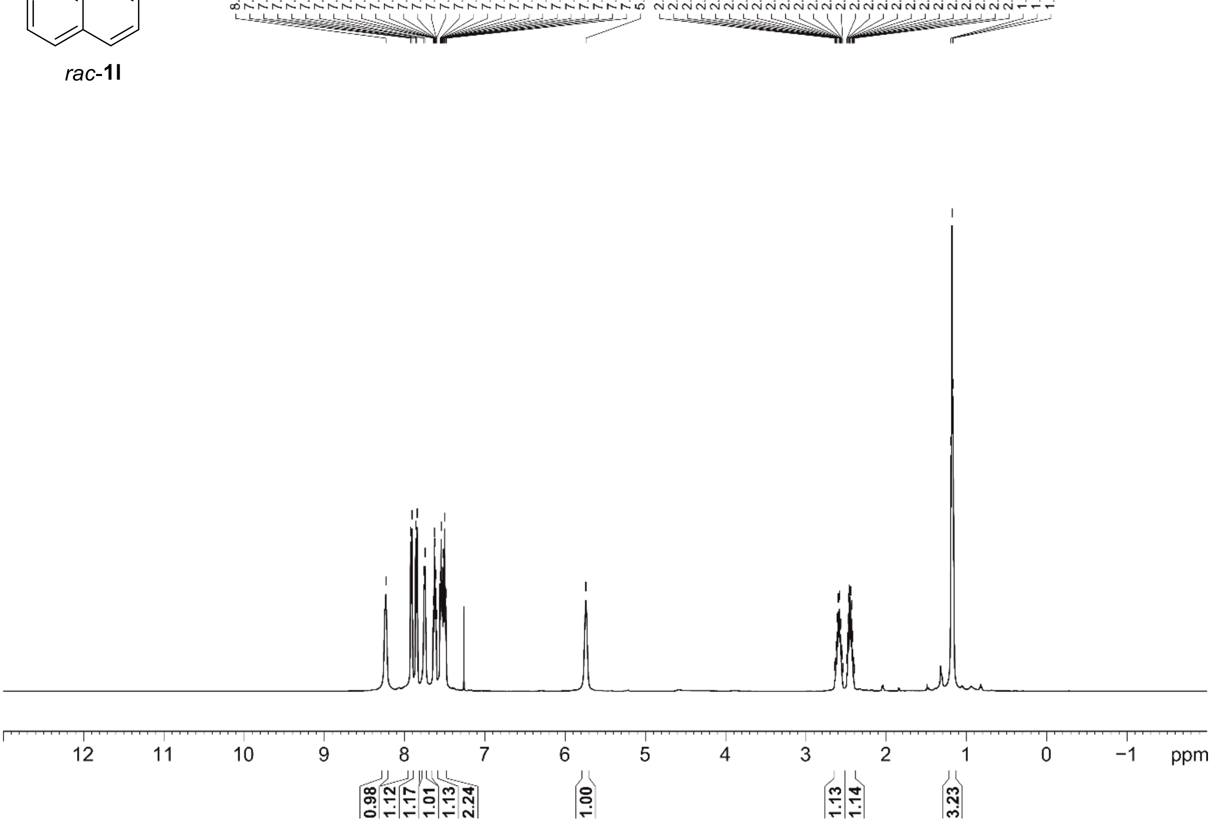


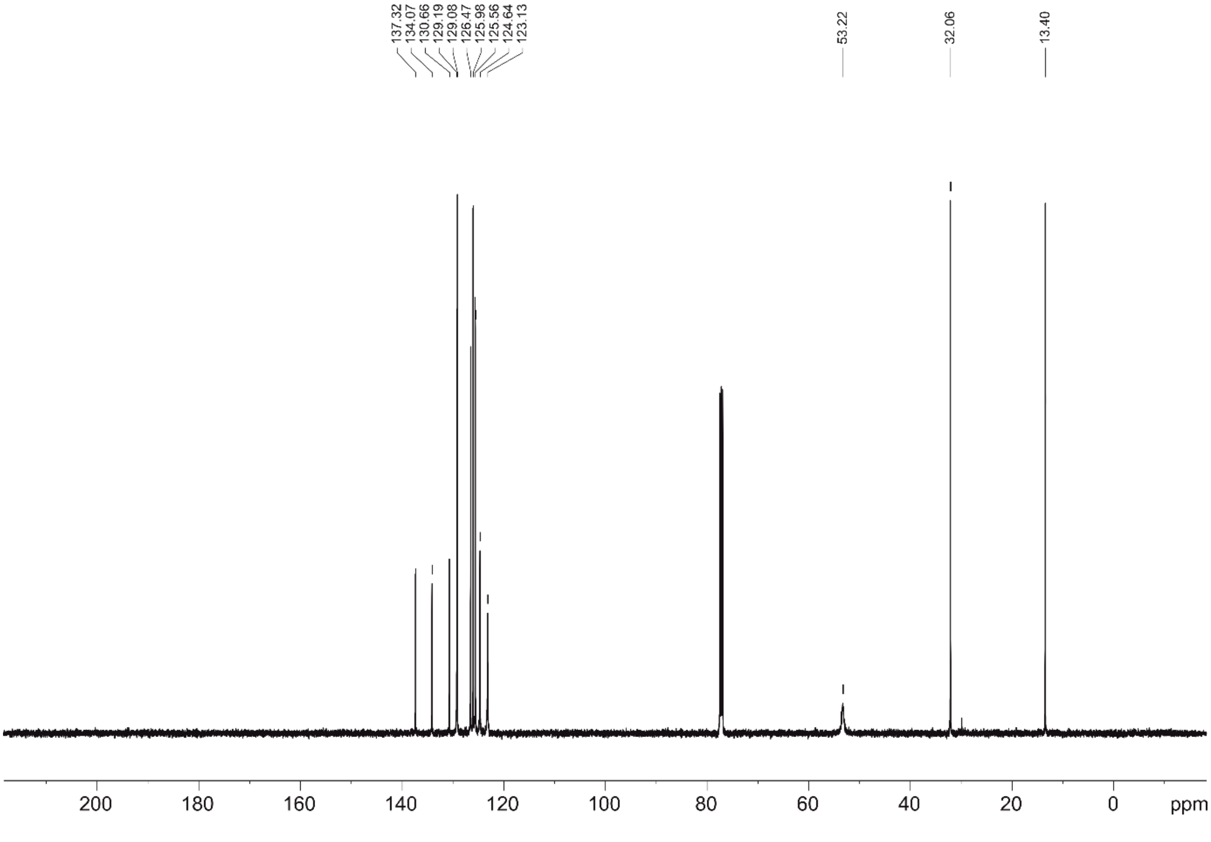

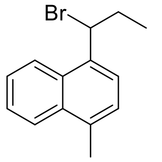

*rac*-**1m**

**1-(1-bromopropyl)-4-methylnaphthalene (1m)**

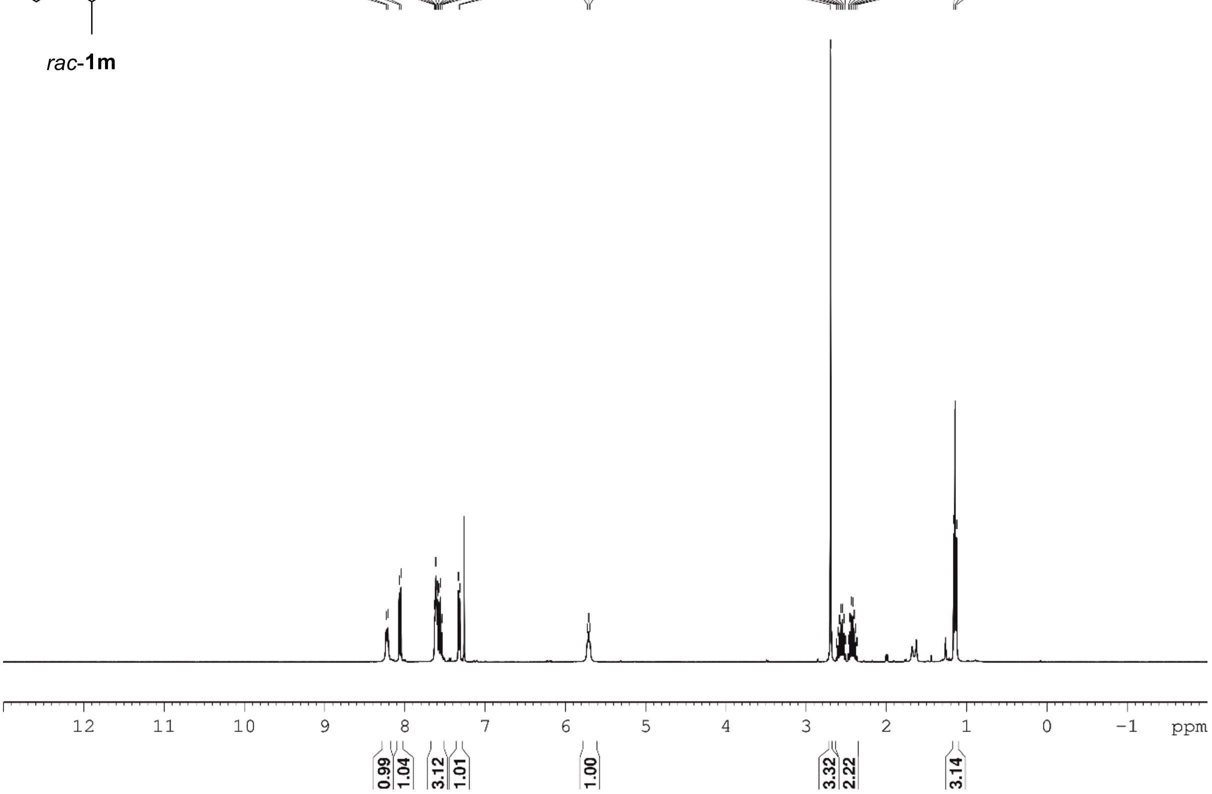


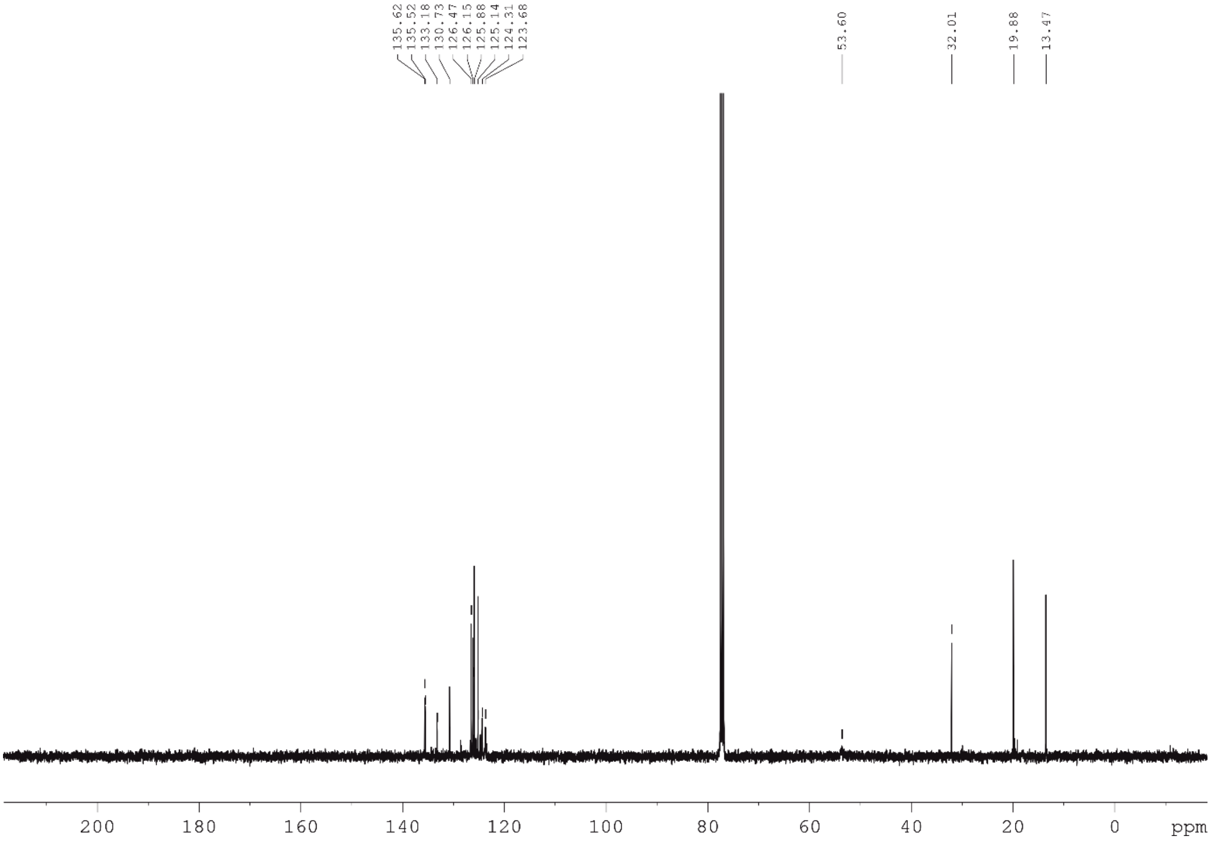

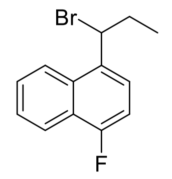


*rac*-**1n**

**1-(1-bromopropyl)-4-fluoronaphthalene (1n)**

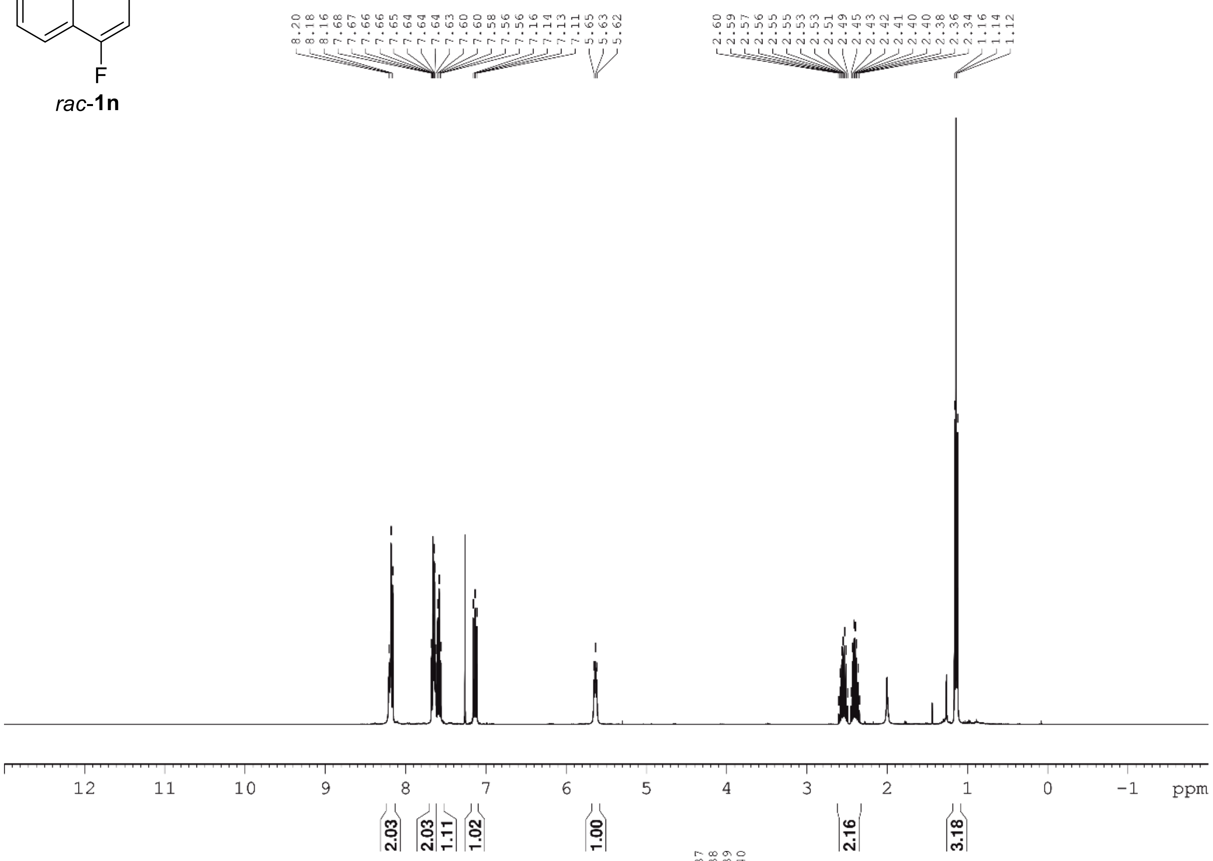


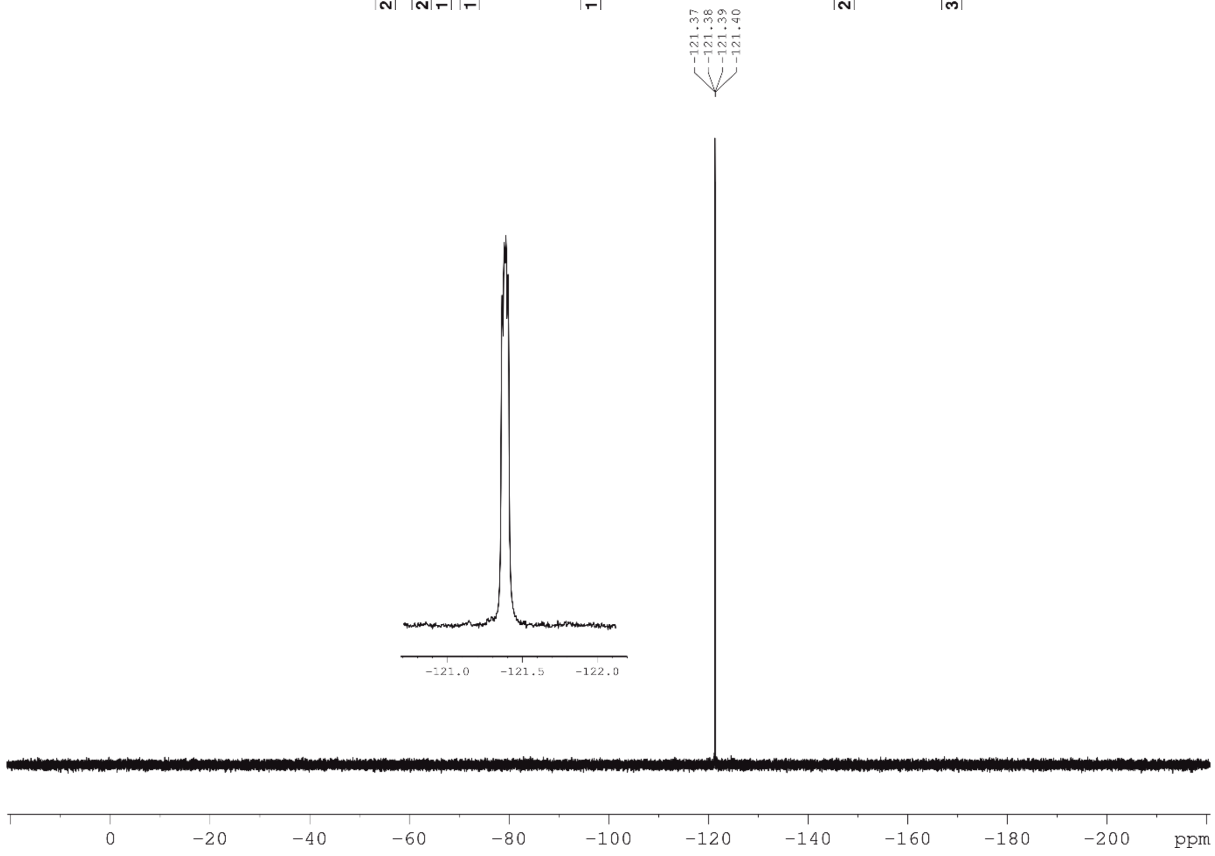

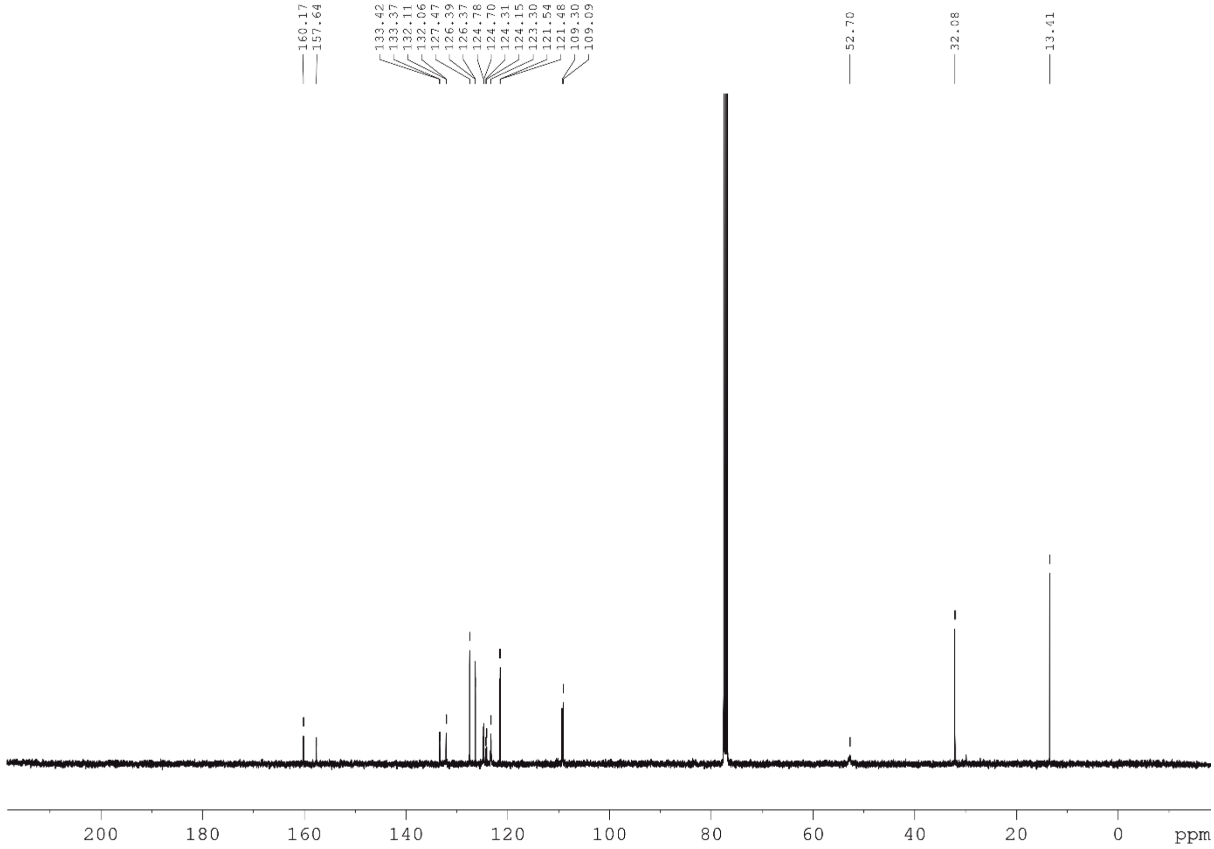


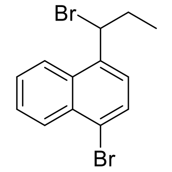


*rac*-**1o**

**1-bromo-4-(1-bromopropyl)naphthalene (1o)**

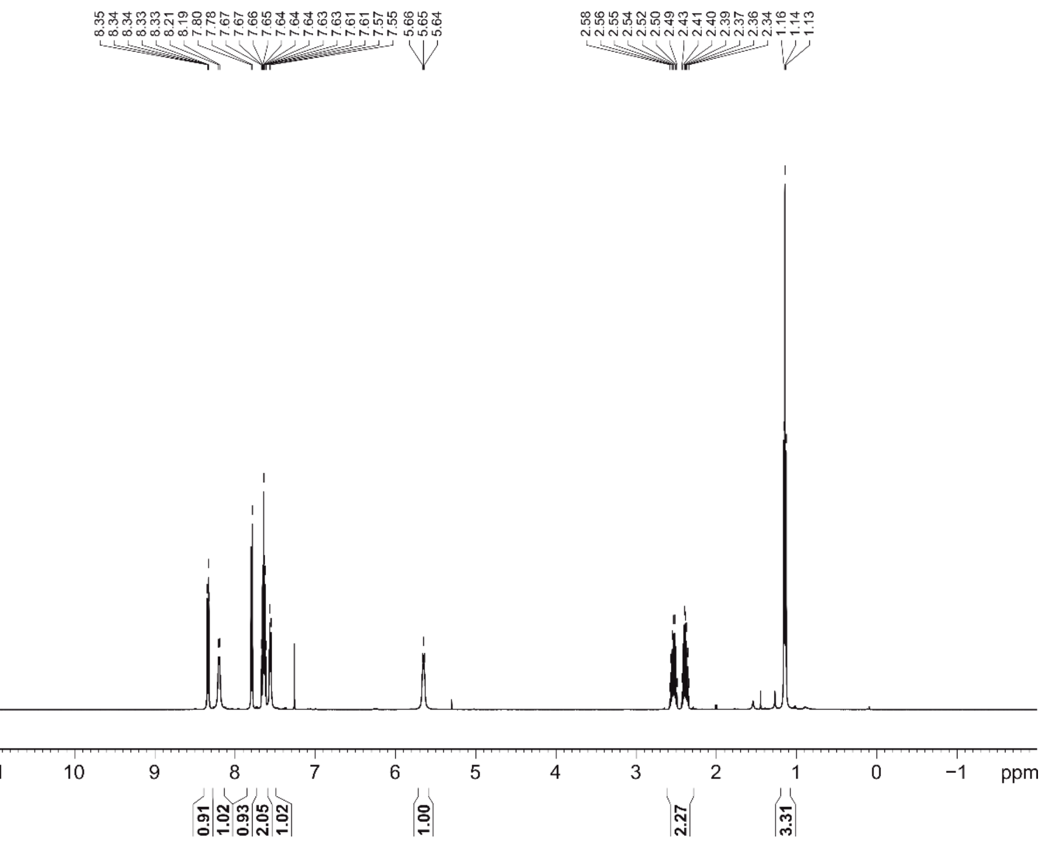

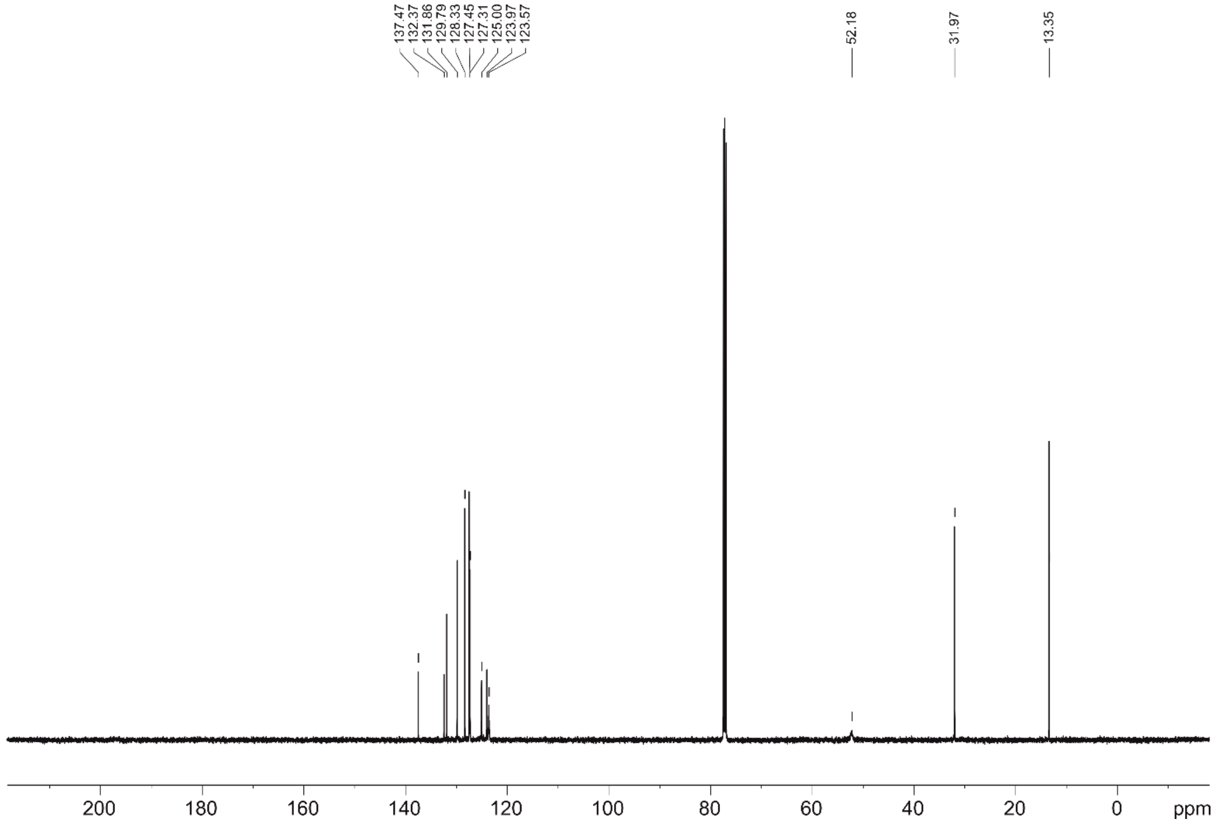


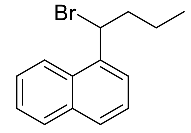


## 1-(1-bromobutyl)naphthalene (1p)

*rac*-**1p**

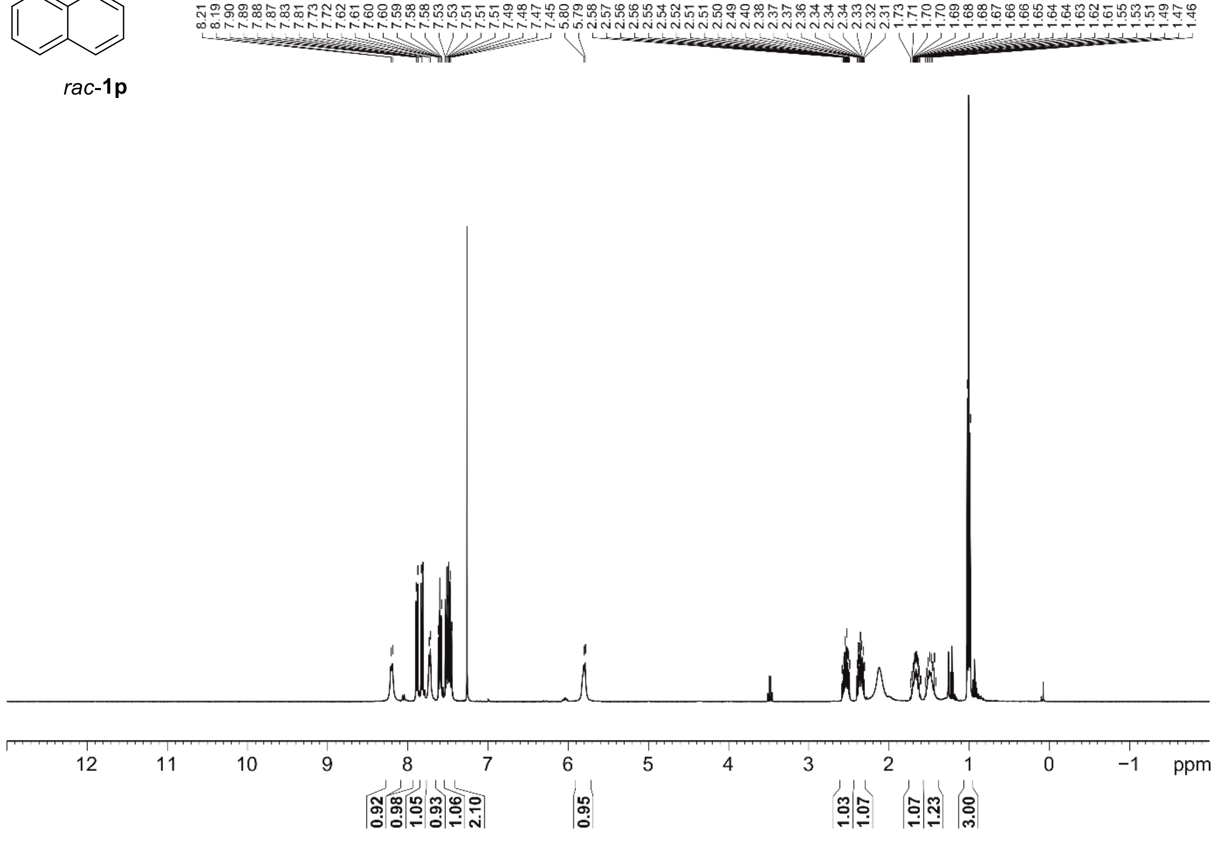

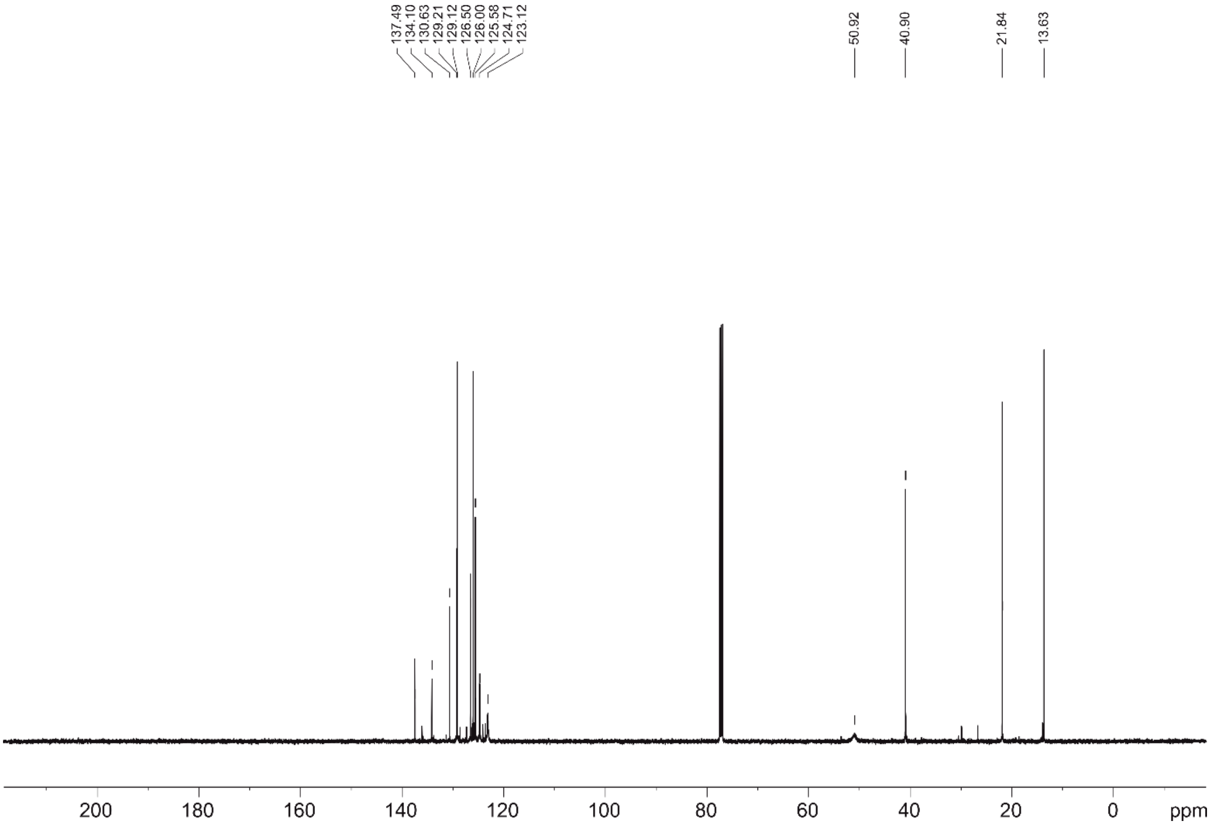


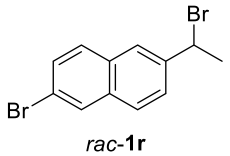


*rac*-**1r**

**2-bromo-6-(1-bromoethyl)naphthalene (1r)**

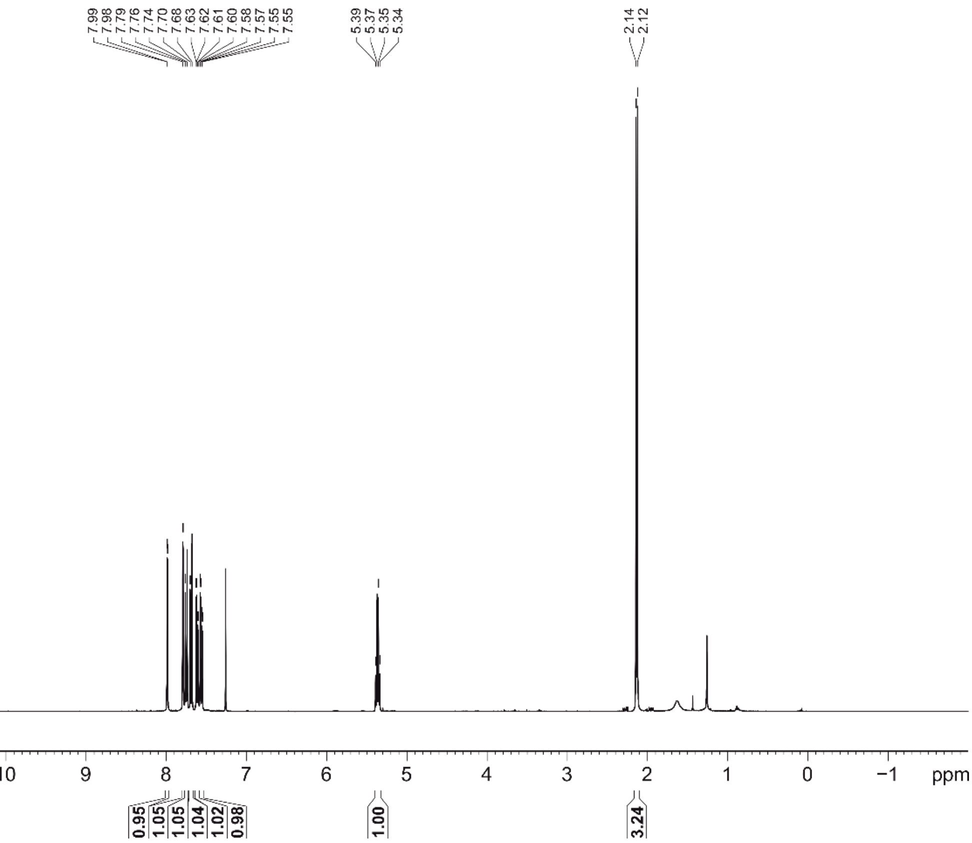

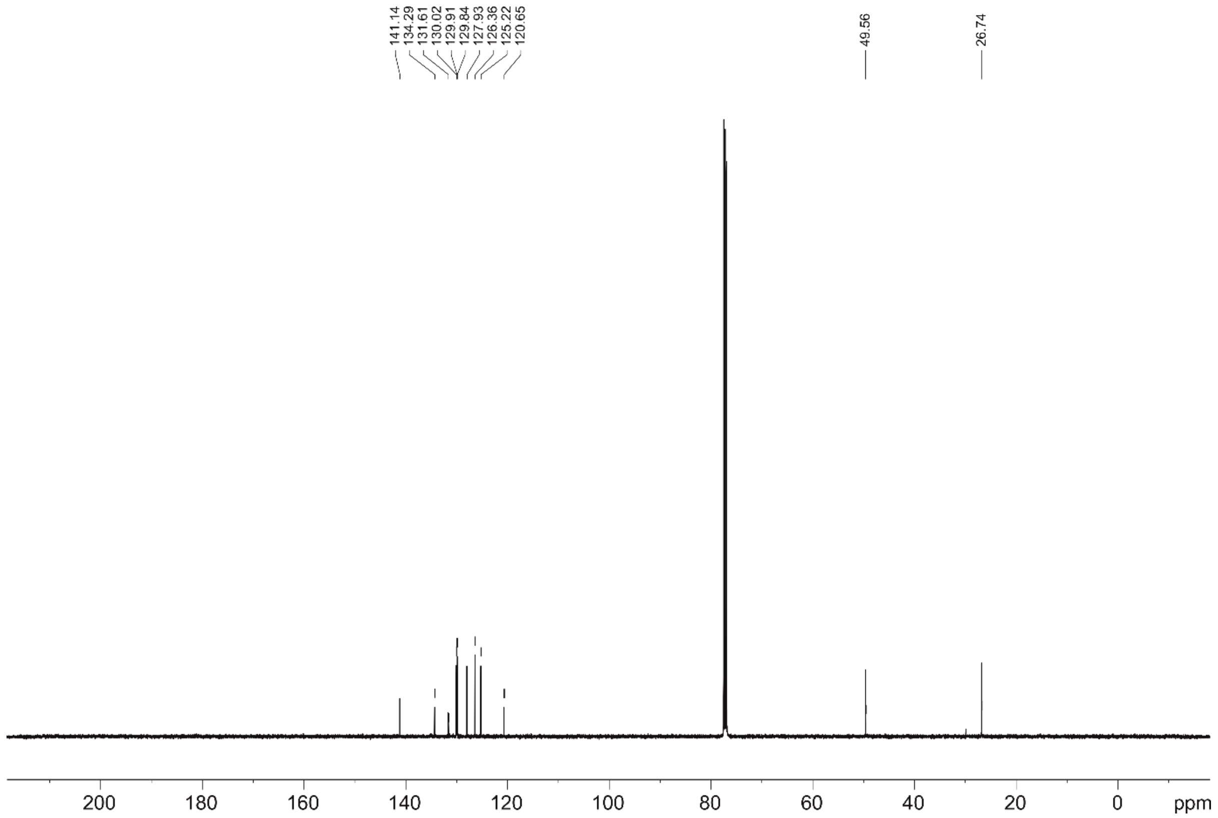


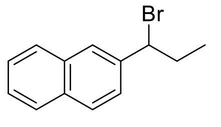


*rac*-**1s**

## 2-(1-bromopropyl)naphthalene (1s)

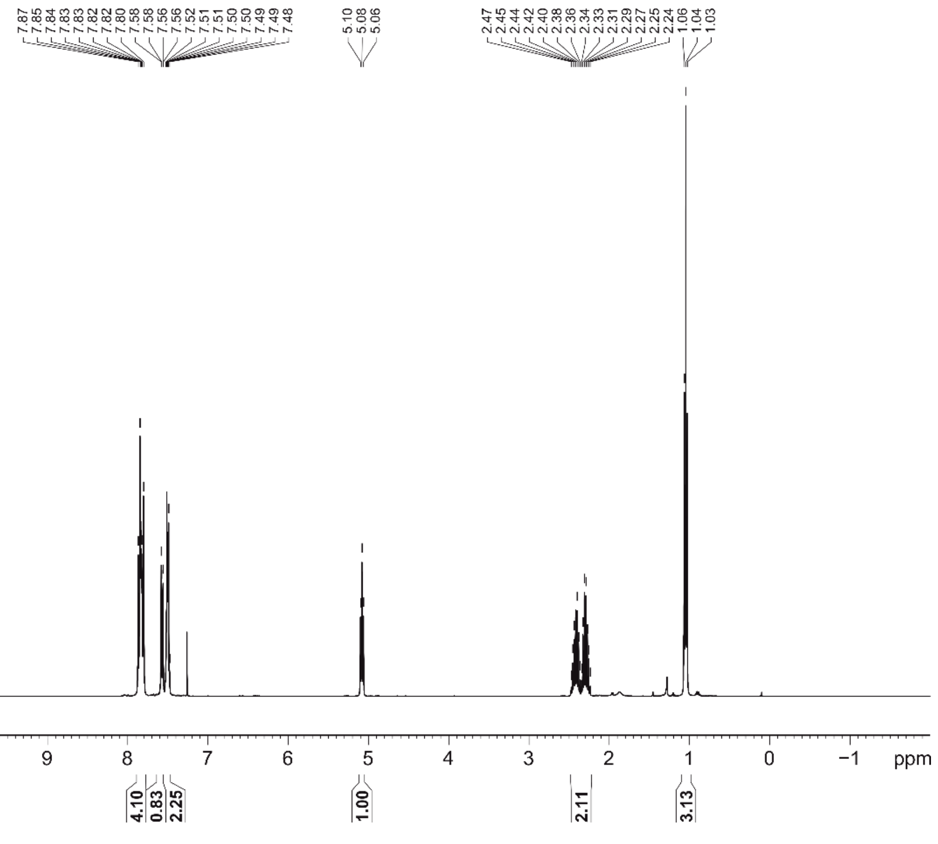

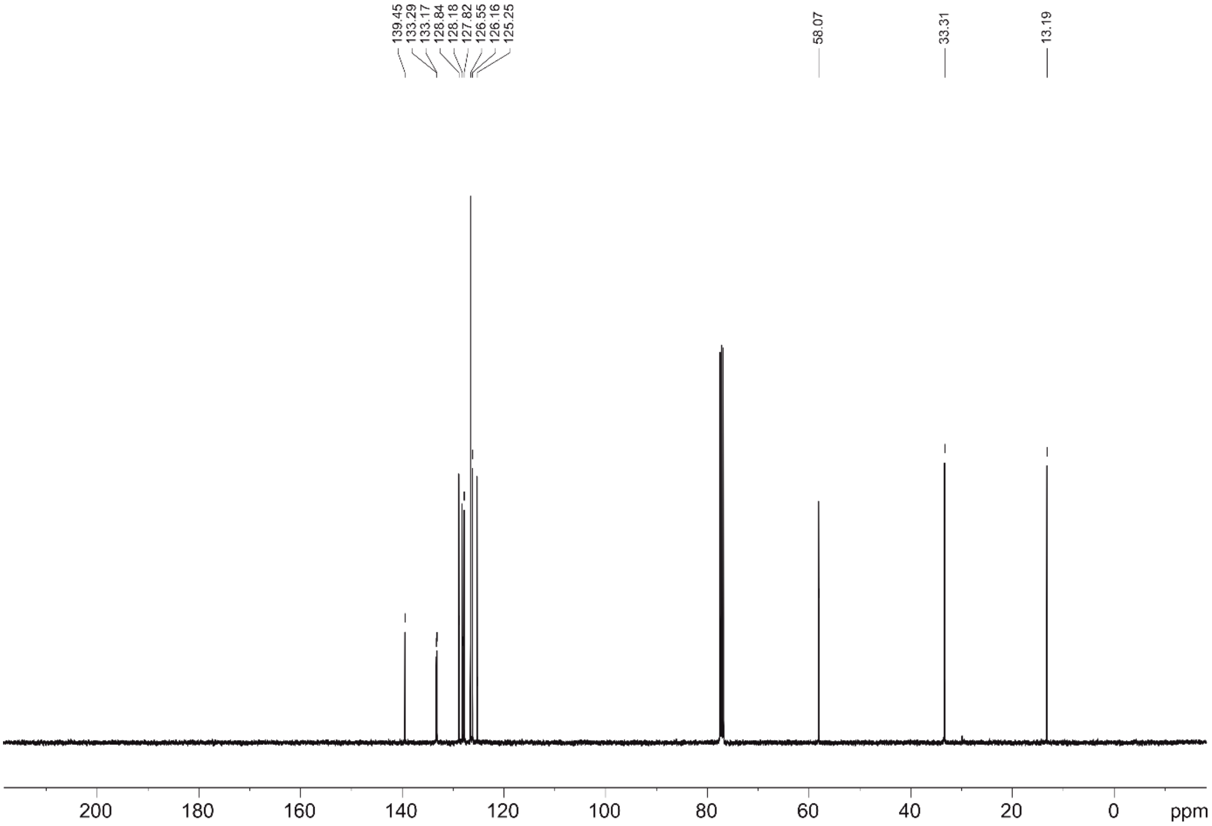


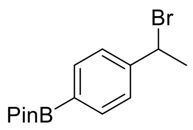

*rac*-**1t**

## 2-(4-(1-bromoethyl)phenyl)-4,4,5,5-tetramethyl-1,3,2-dioxaborolane (1t)

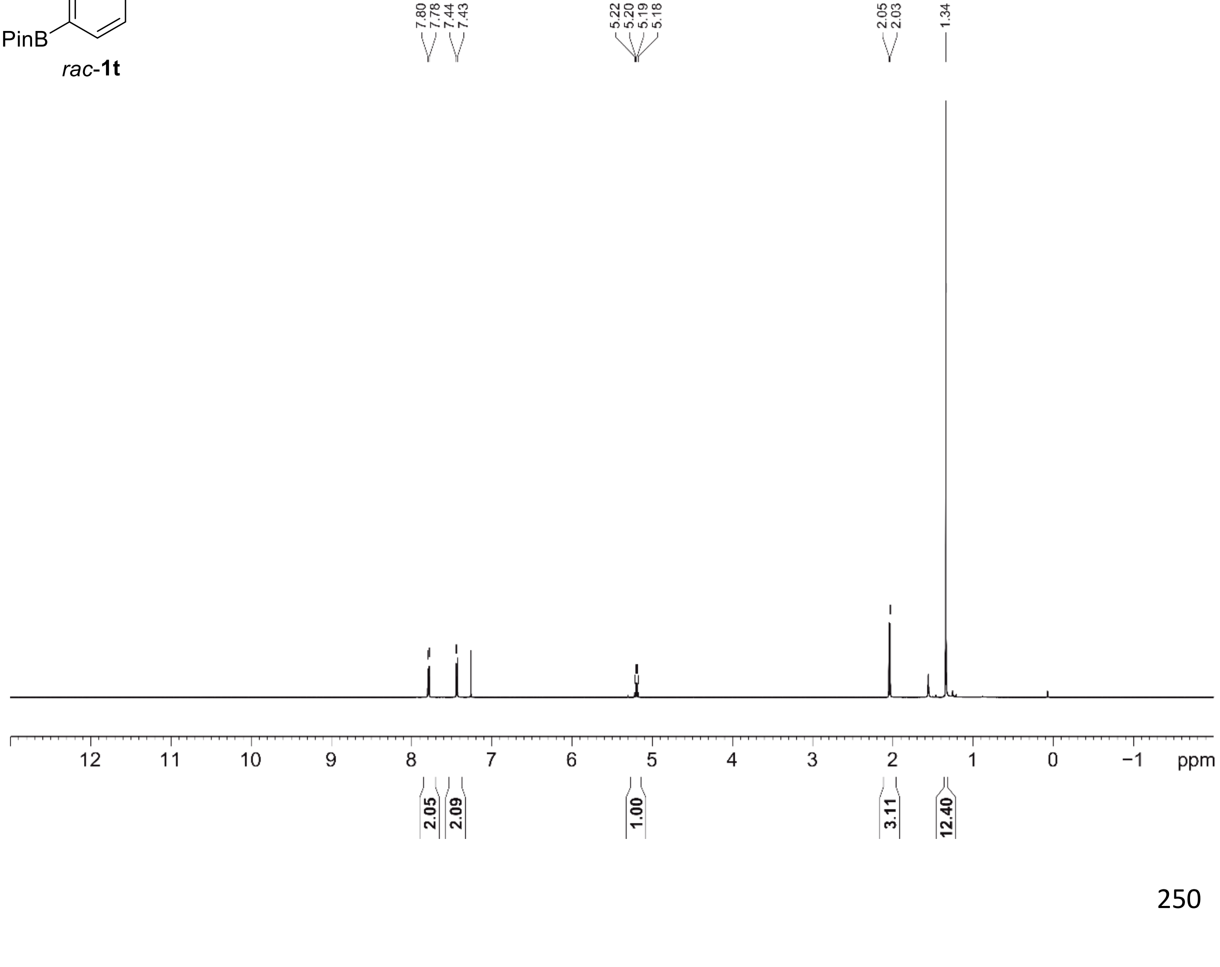

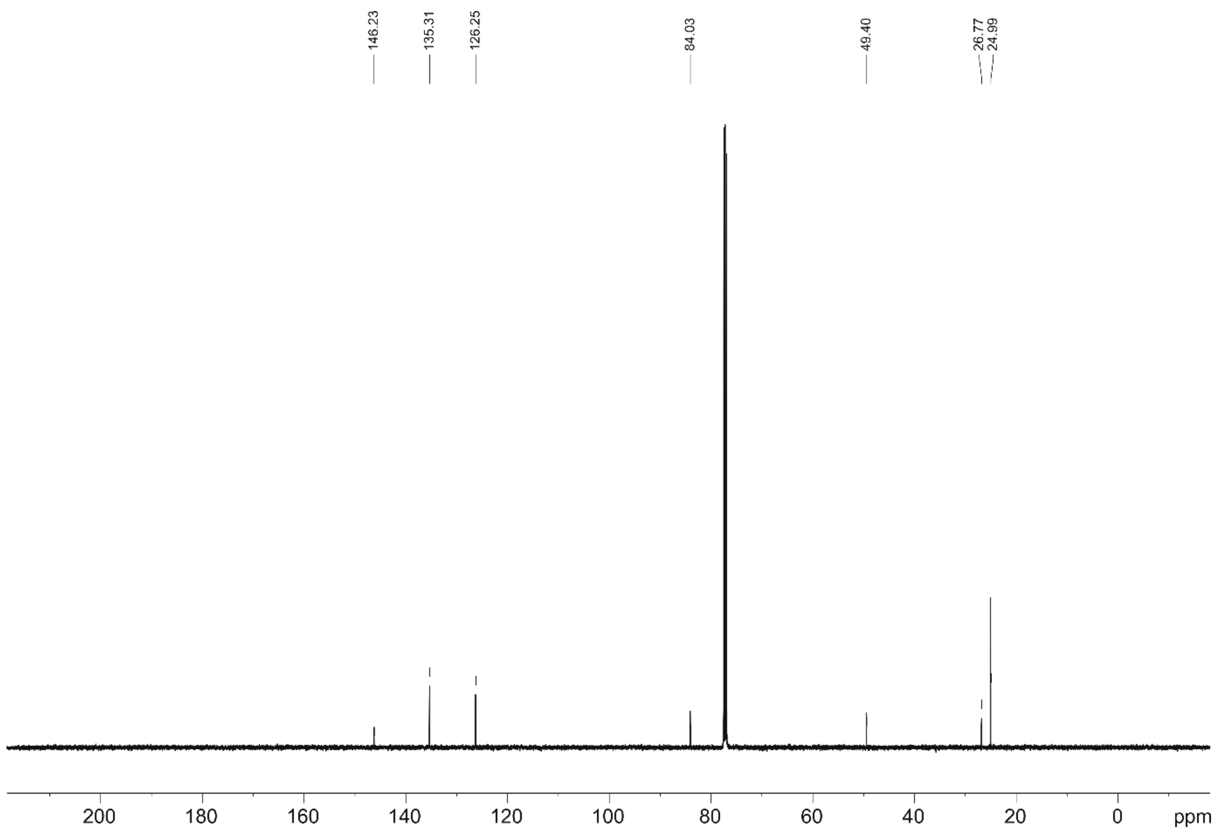


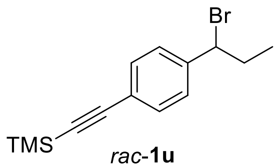


*rac*-**1u**

**((4-(1-bromopropyl)phenyl)ethynyl)trimethylsilane (1u)**

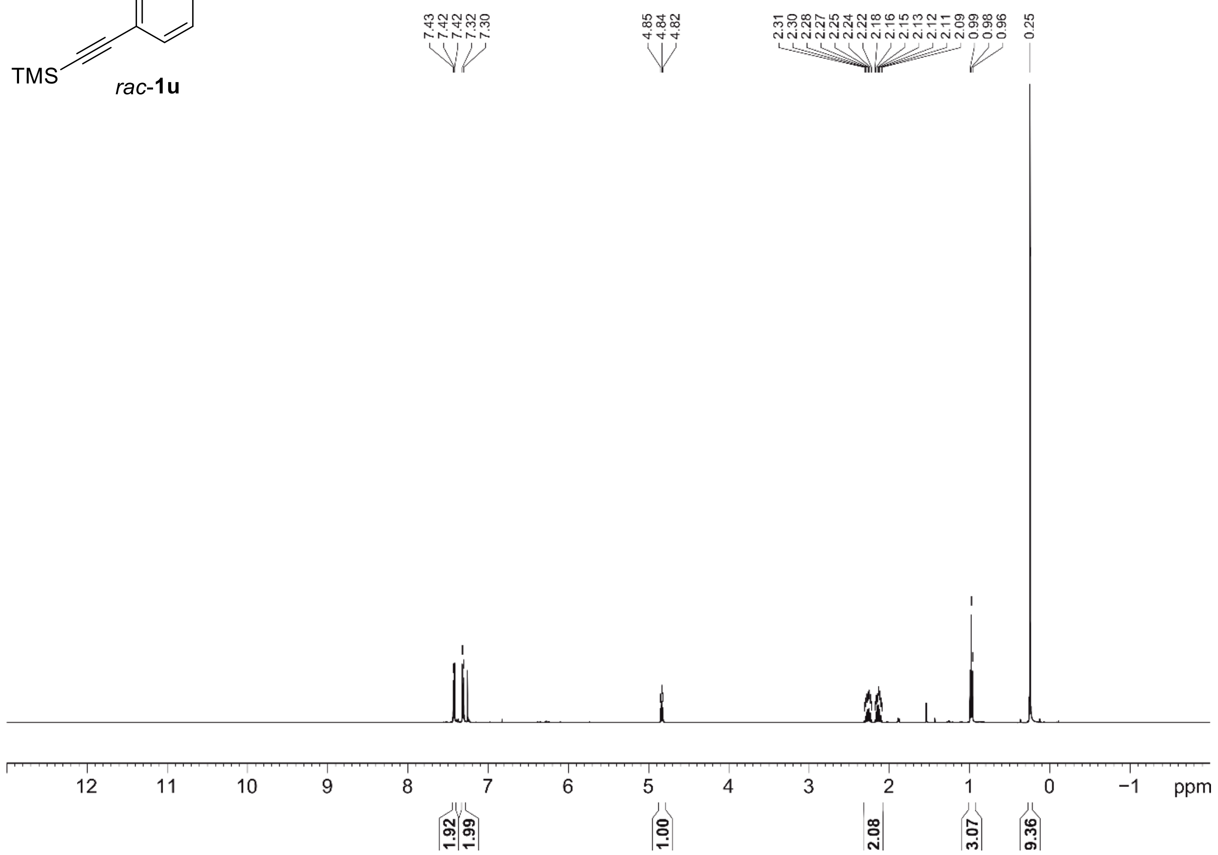

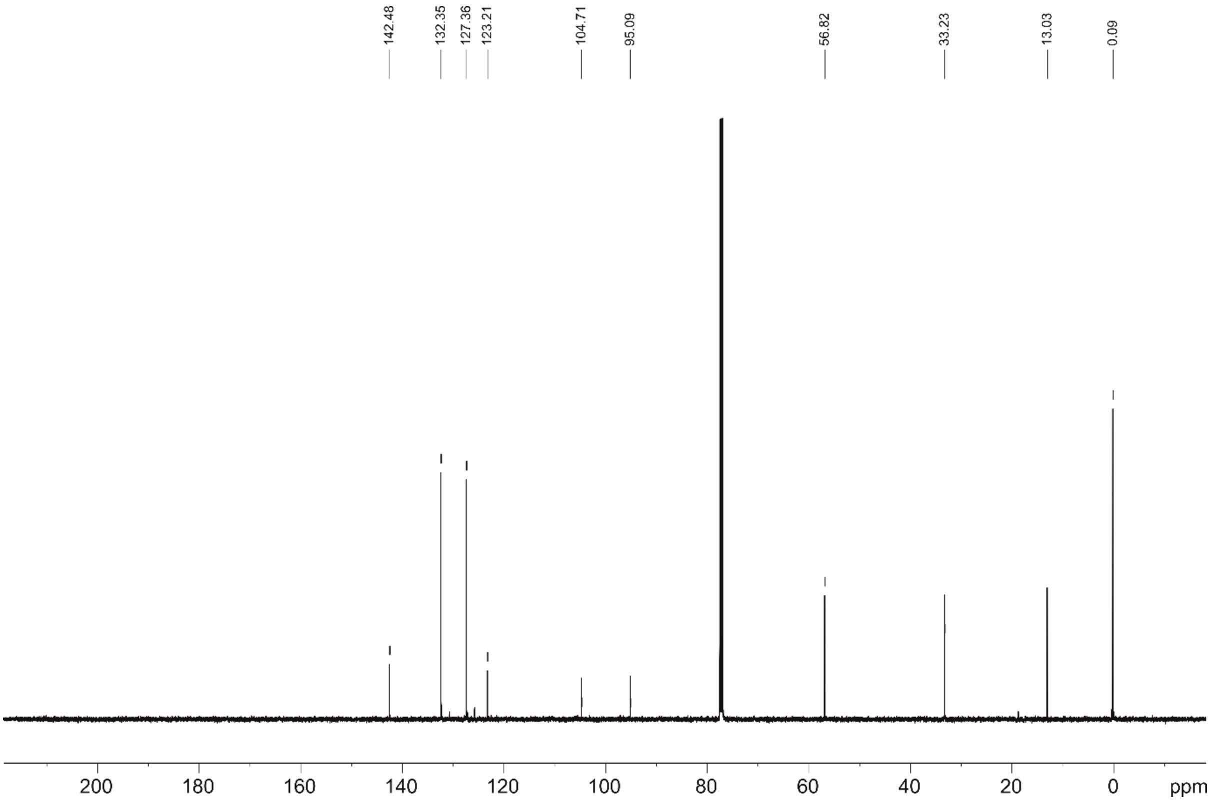


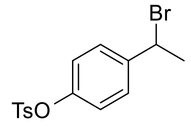


*rac*-**1v**

**4-(1-bromoethyl)phenyl-4-methylbenzenesulfonate (1v)**

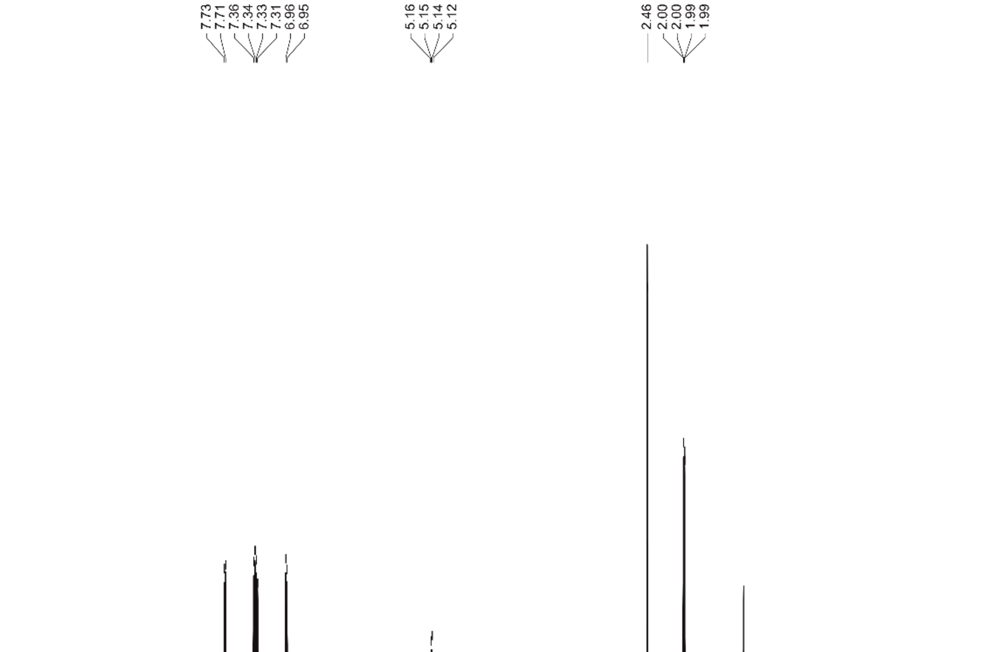

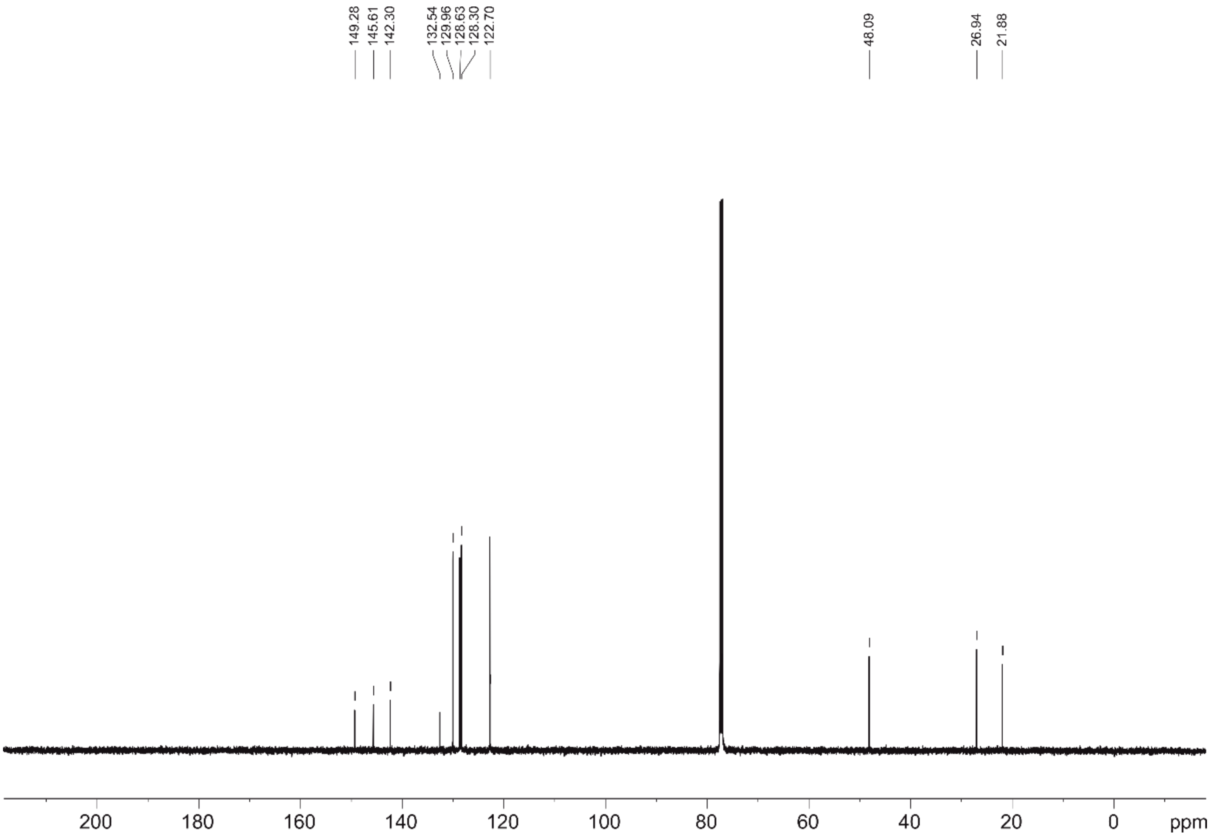


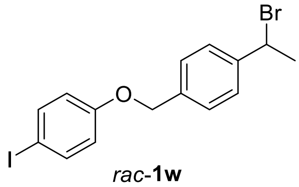

*rac*-**1w**

**1-(1-bromoethyl)-4-((4-iodophenoxy)methyl)benzene (1w)**

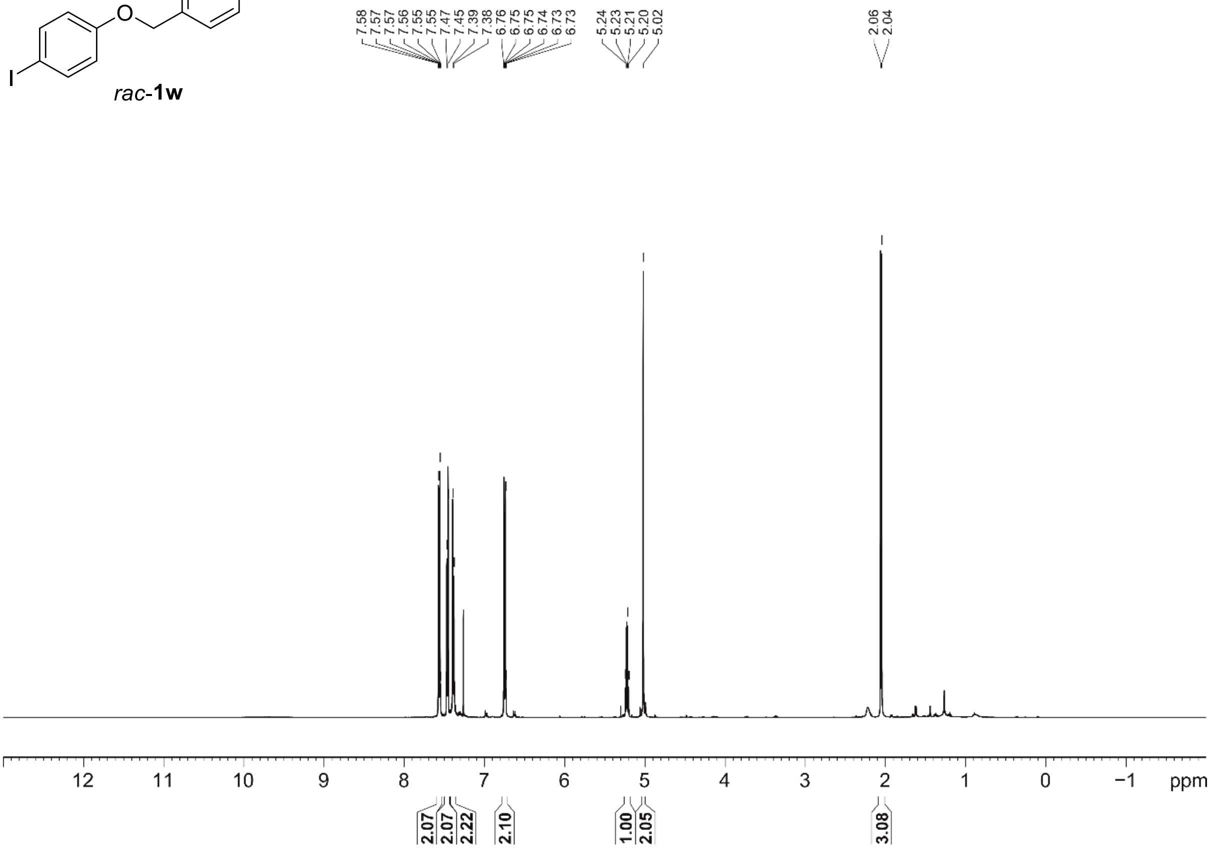

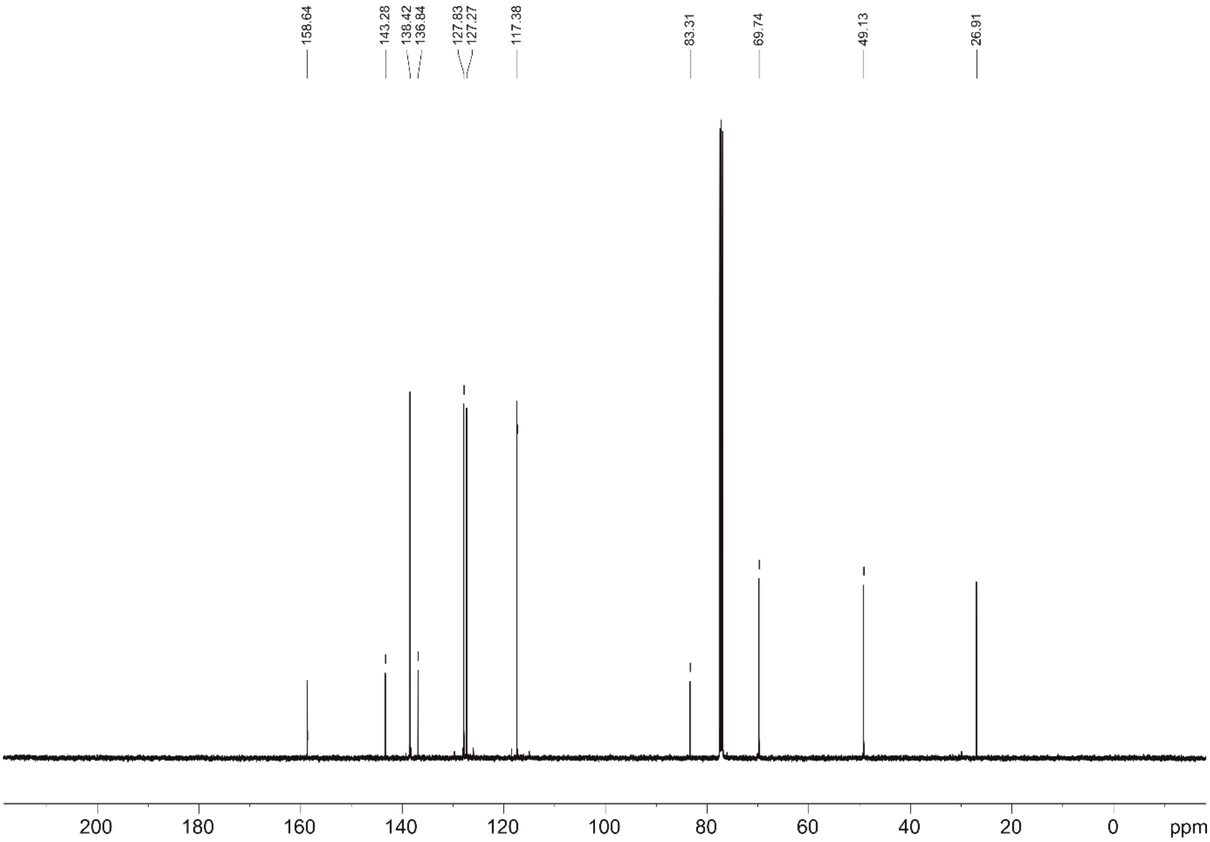


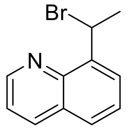


**8-(1-bromoethyl)quinoline (1x)**

*rac*-**1x**

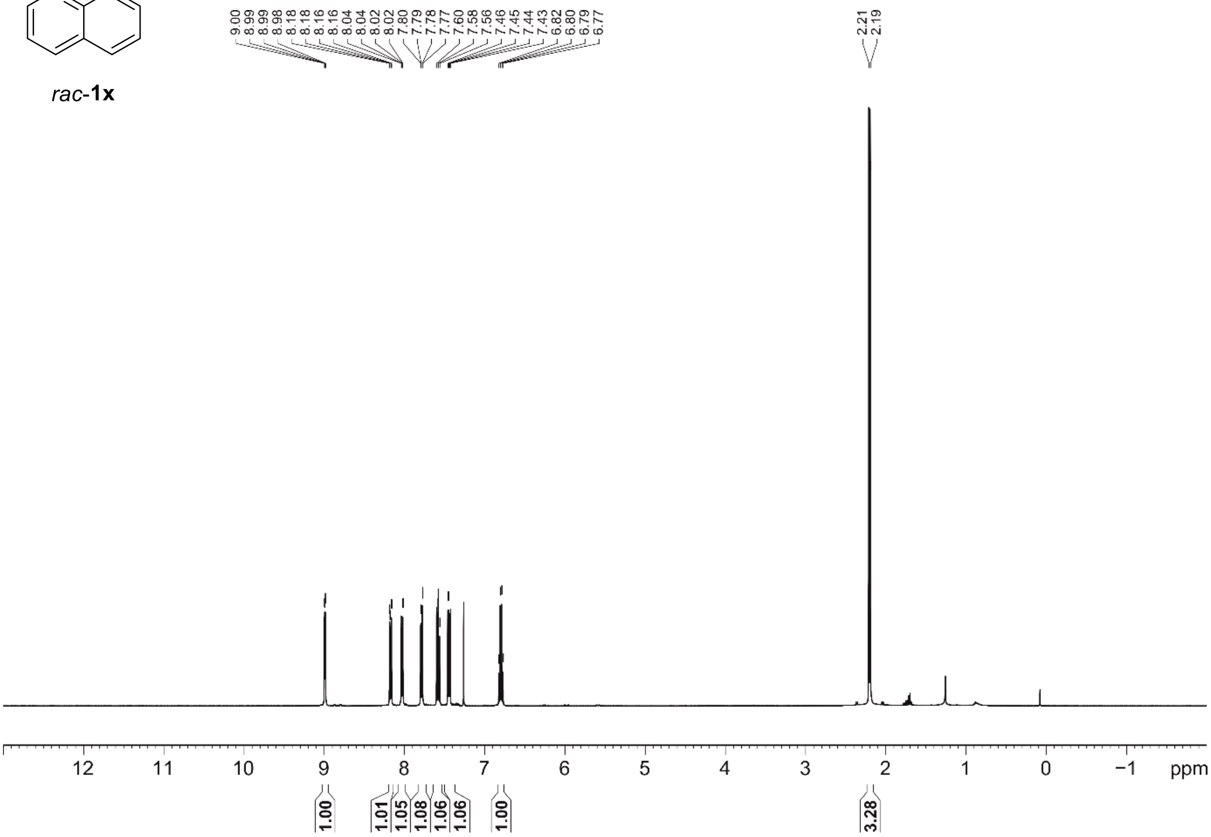

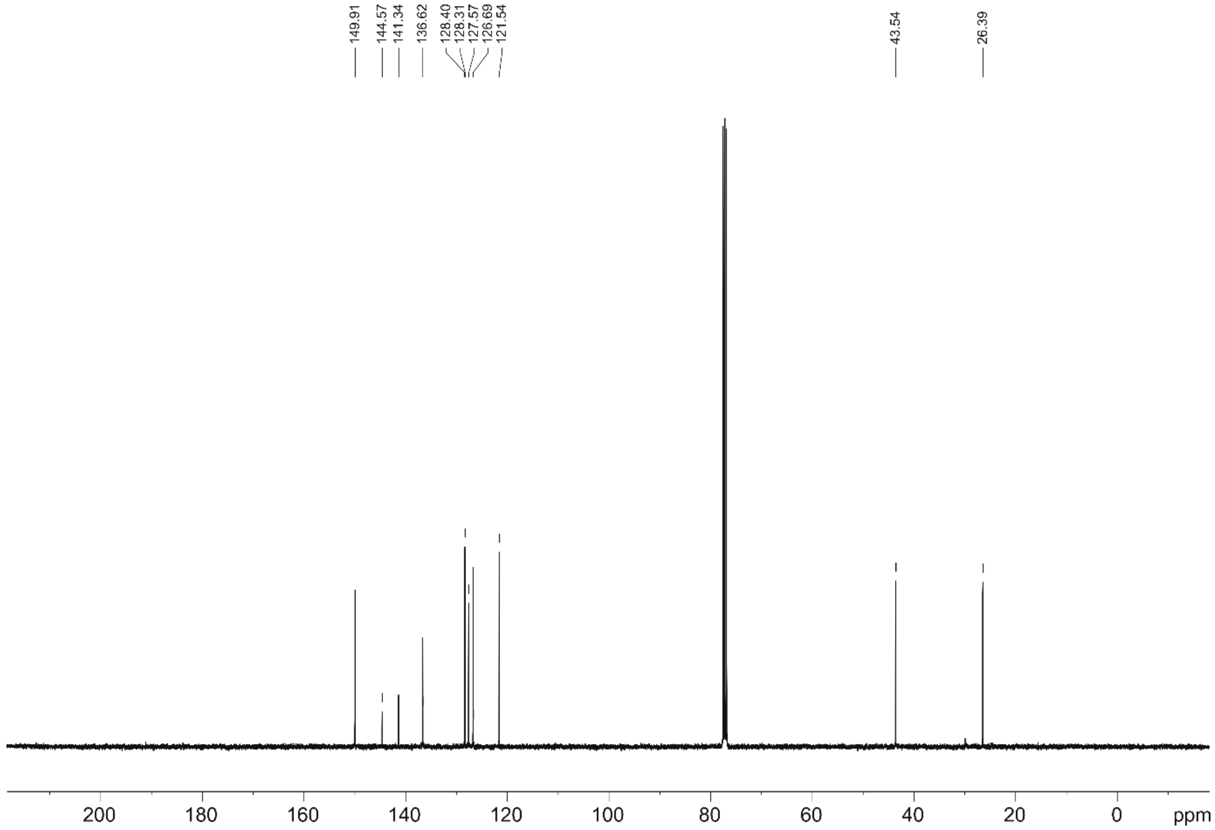


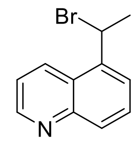


*rac*-**1y**

**5-(1-bromoethyl)quinoline (1y)**

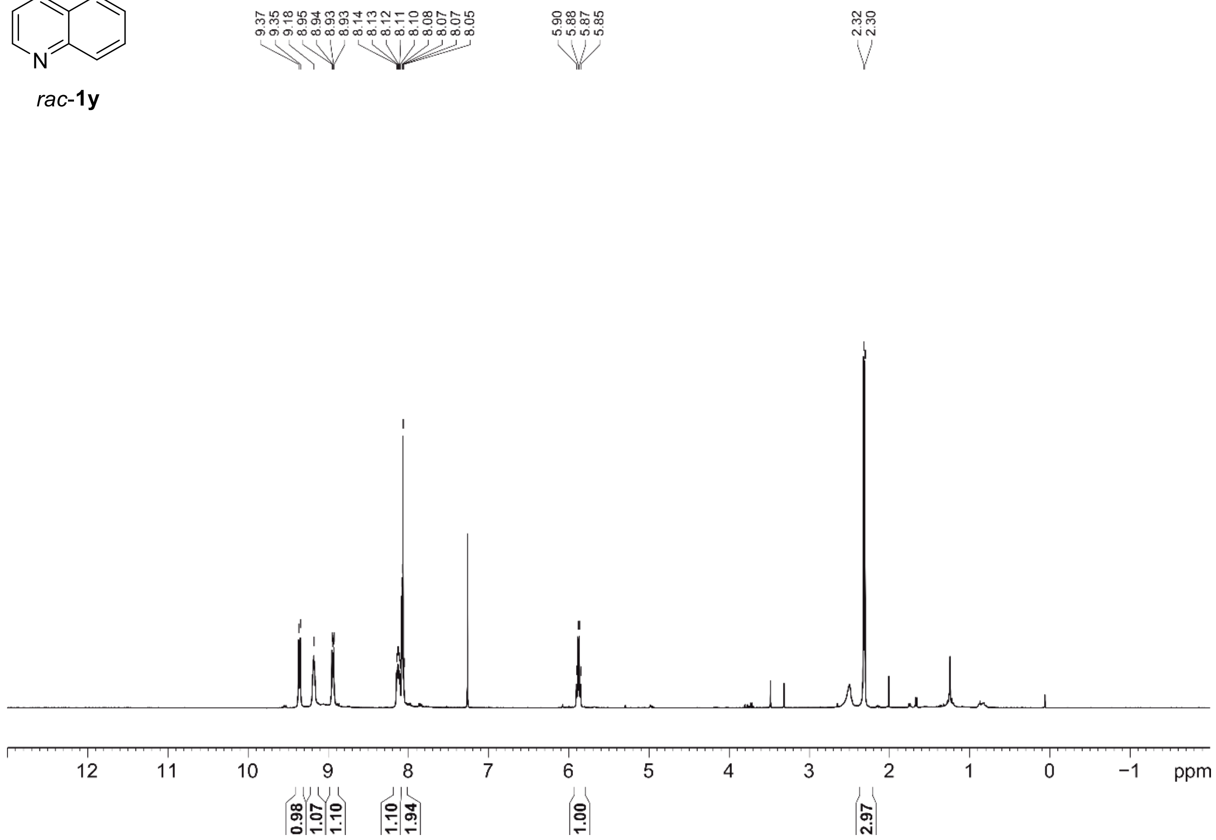

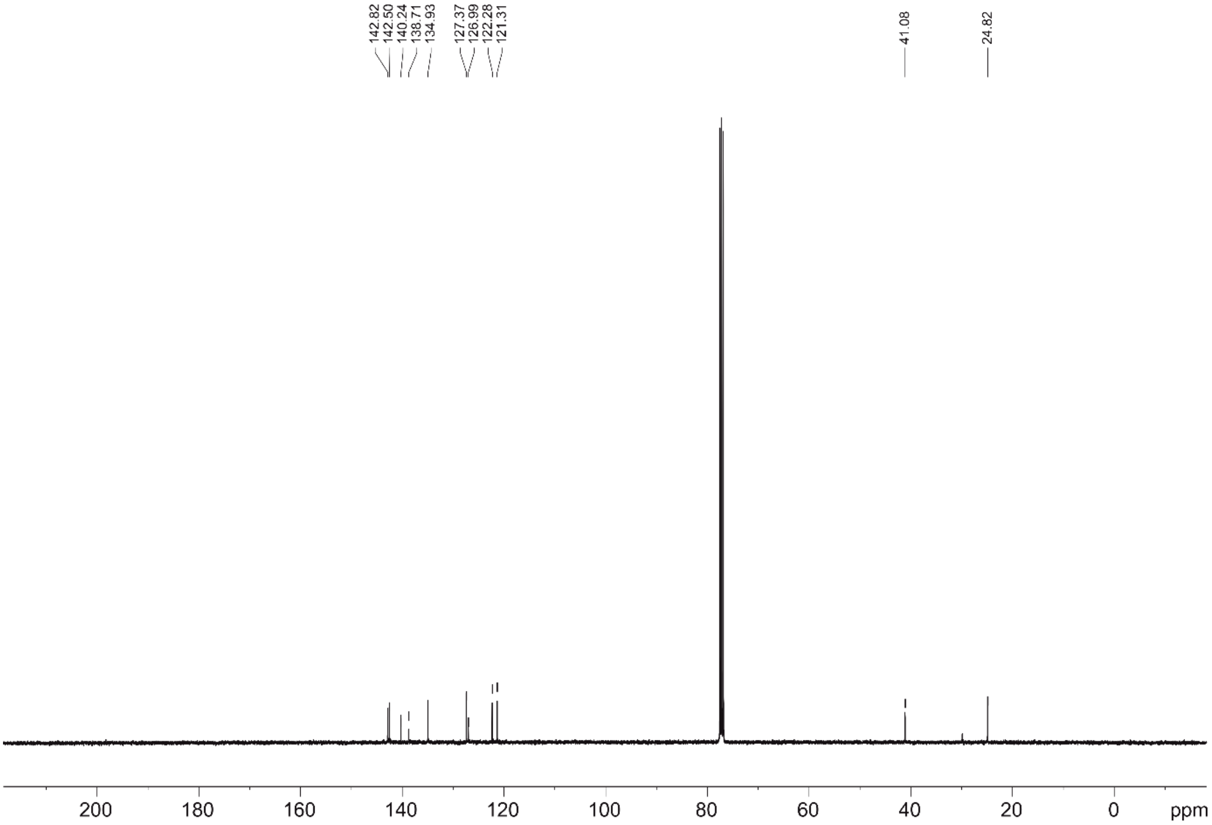


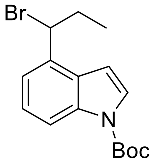

*rac*-**1z**

***tert*-butyl-4-(1-bromopropyl)-1*H*-indole-1-carboxylate (1z)**

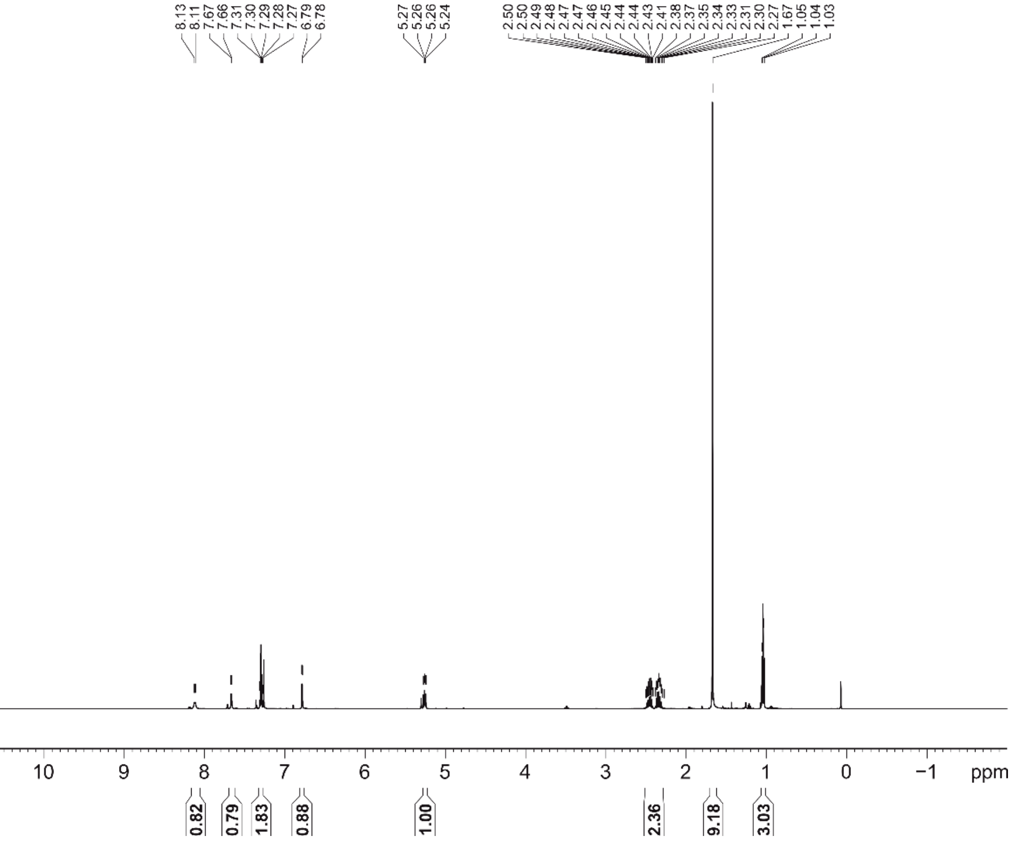

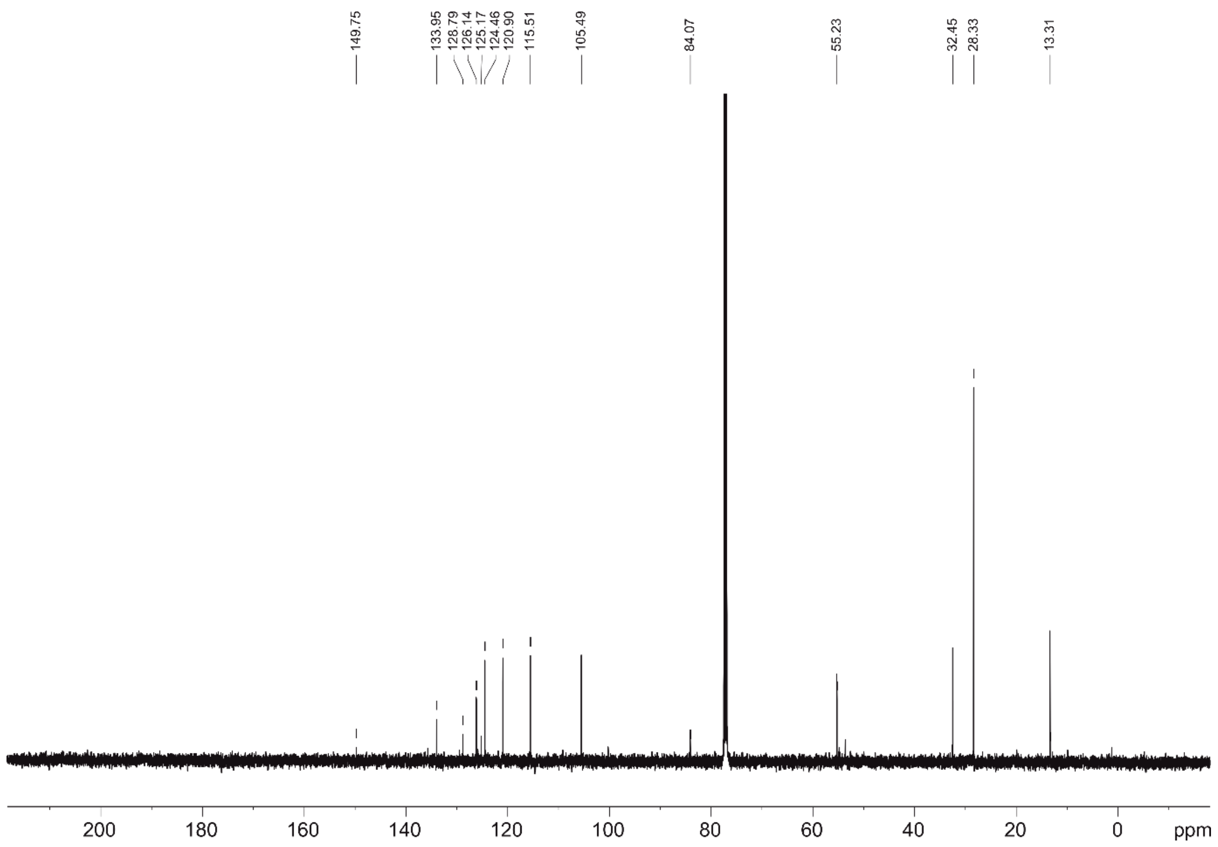


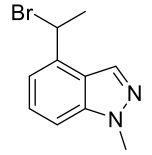


*rac*-**1aa**

**4-(1-bromoethyl)-1-methyl-1*H*-indazole (1aa)**

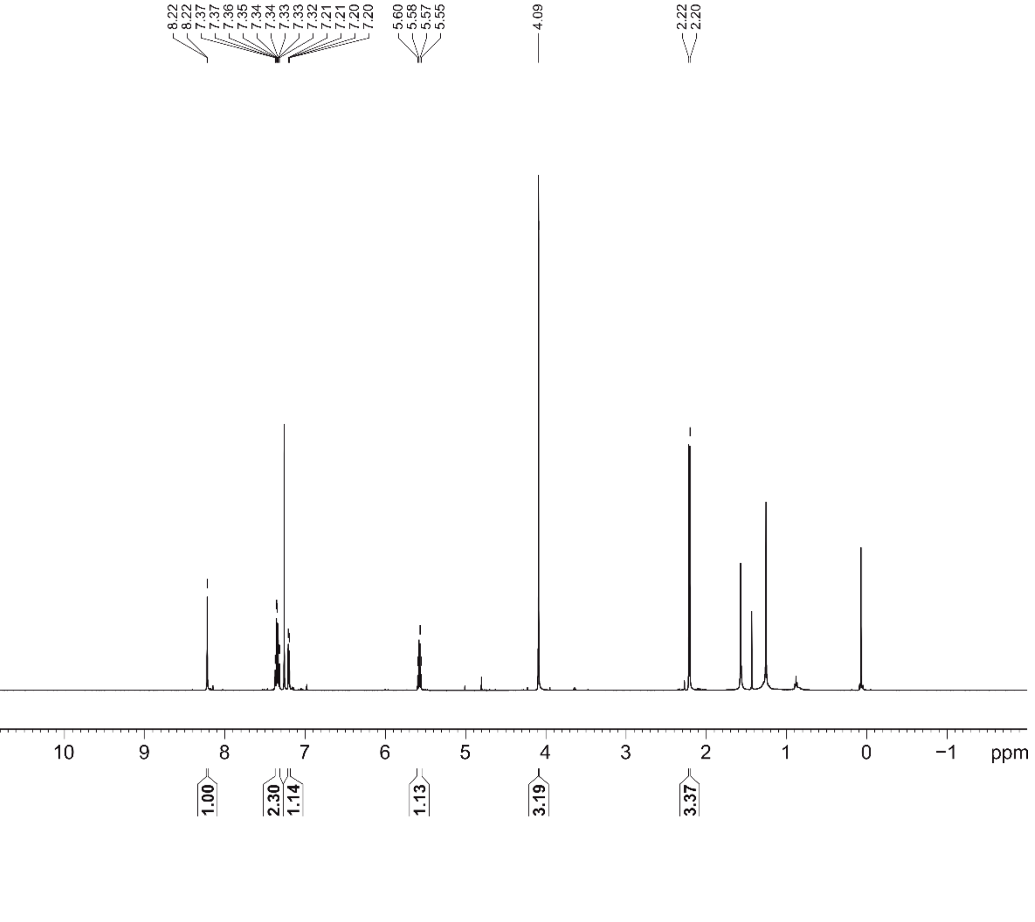

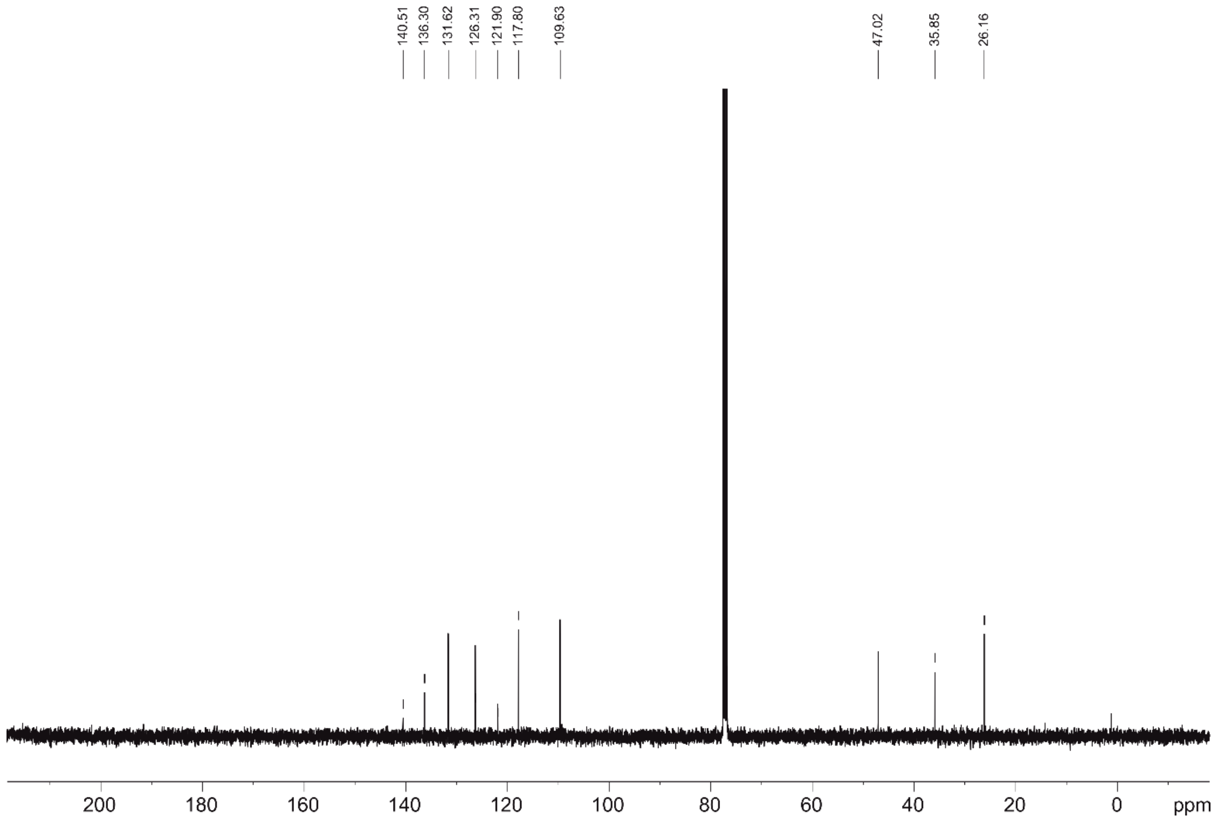


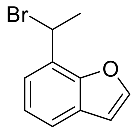

*rac*-**1ab**

**7-(1-bromoethyl)benzofuran (1ab)**

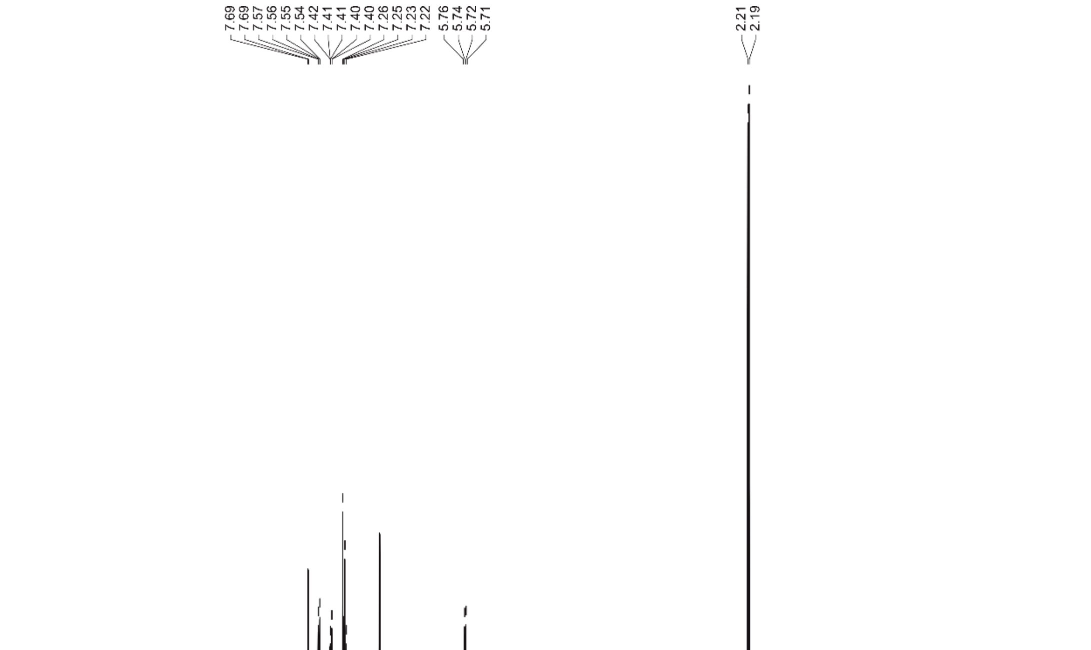

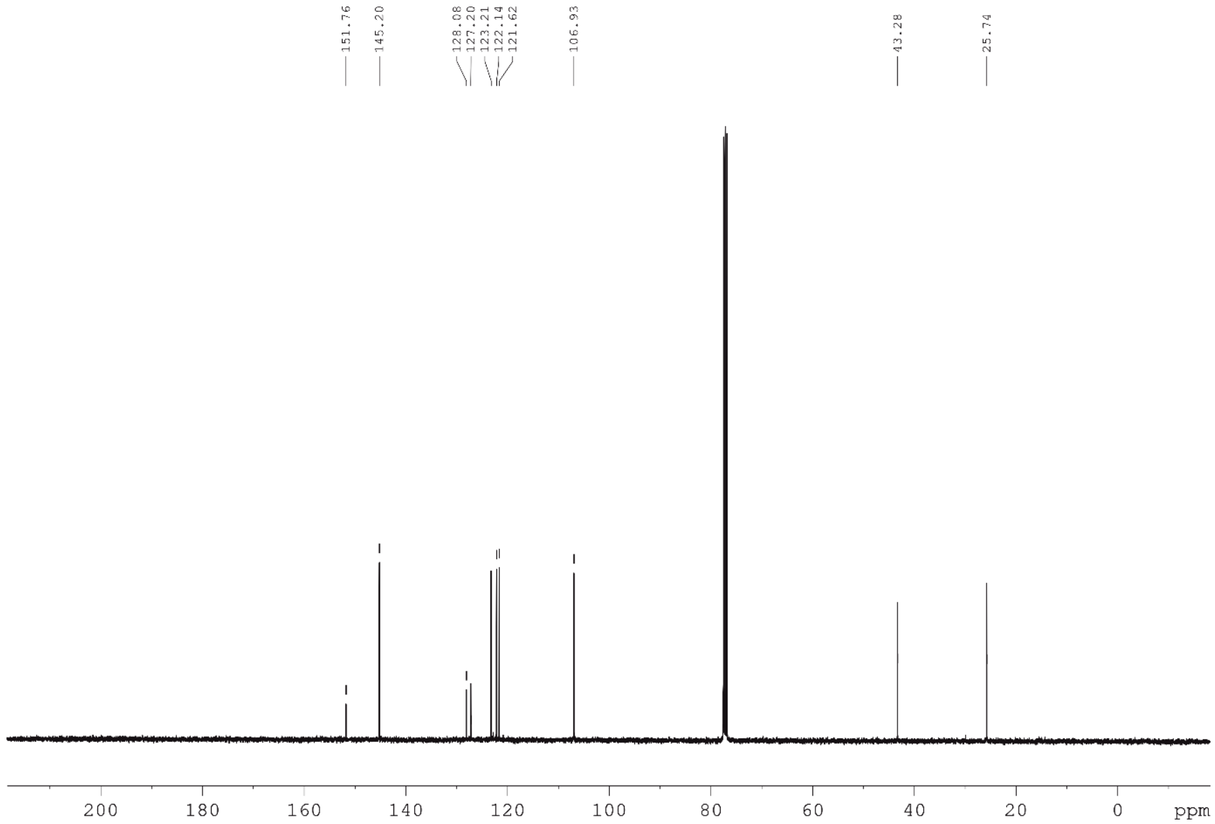


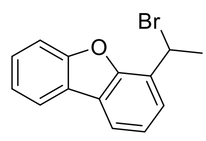


*rac*-**1ac**

**4-(1-bromoethyl)dibenzo[*b*,*d*]furan (1ac)**

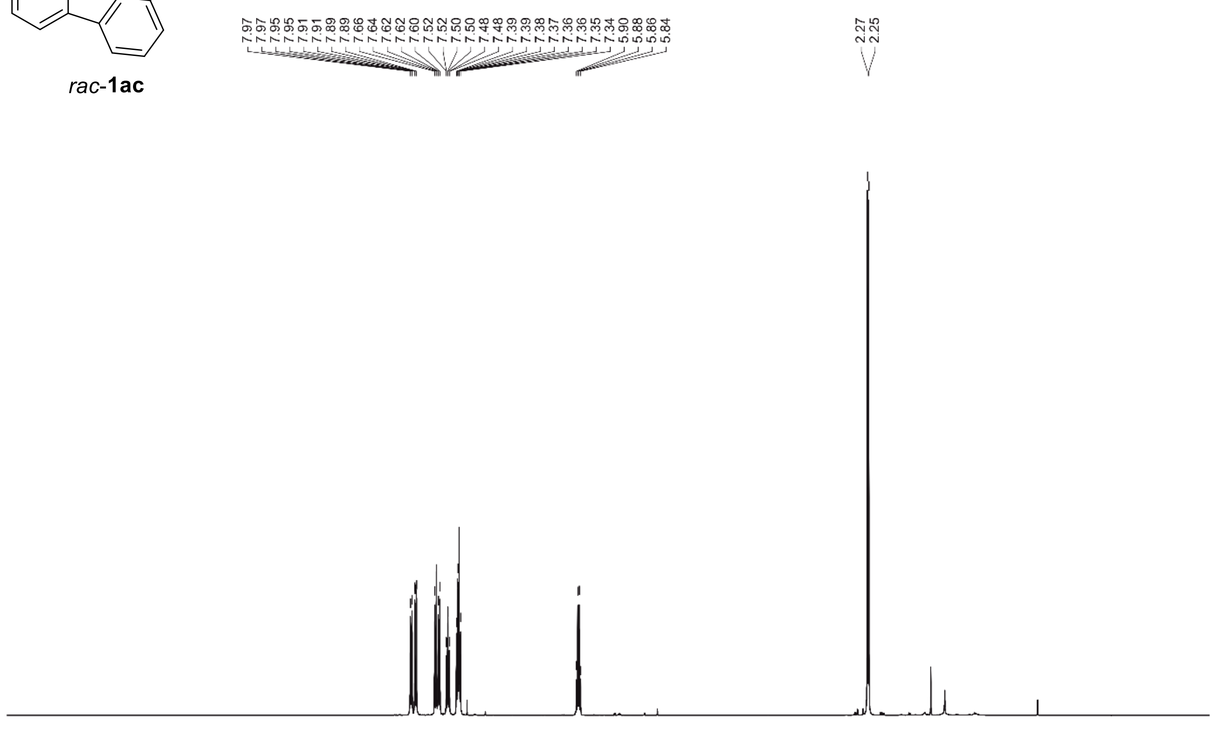

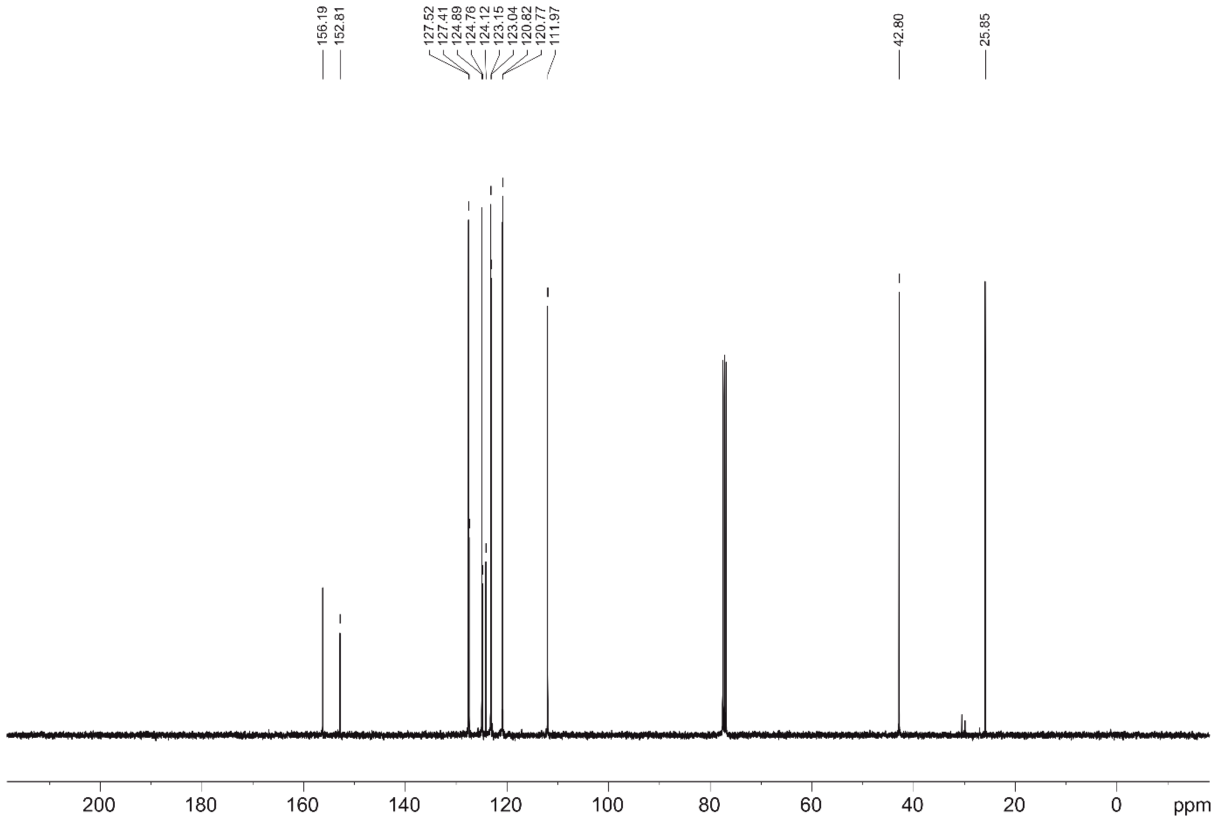


**4-(1-bromoethyl)dibenzo[*b*,*d*]thiophene (1ad)**

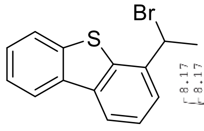


*rac*-**1ad**

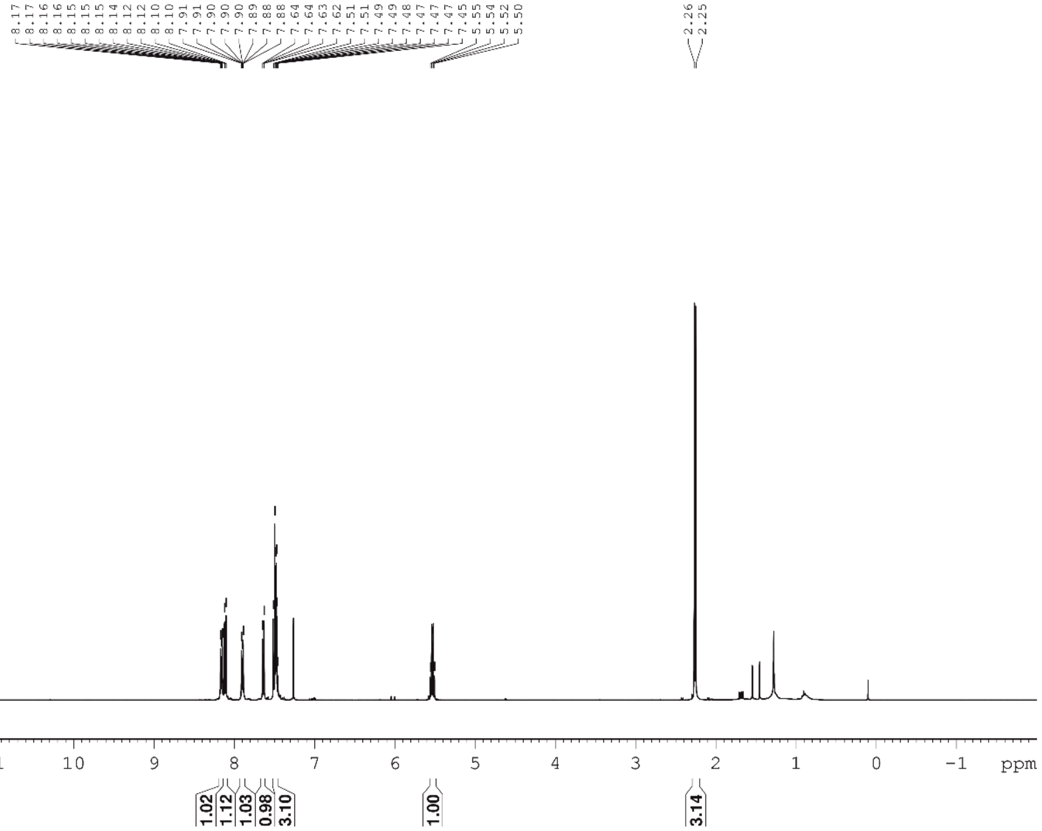

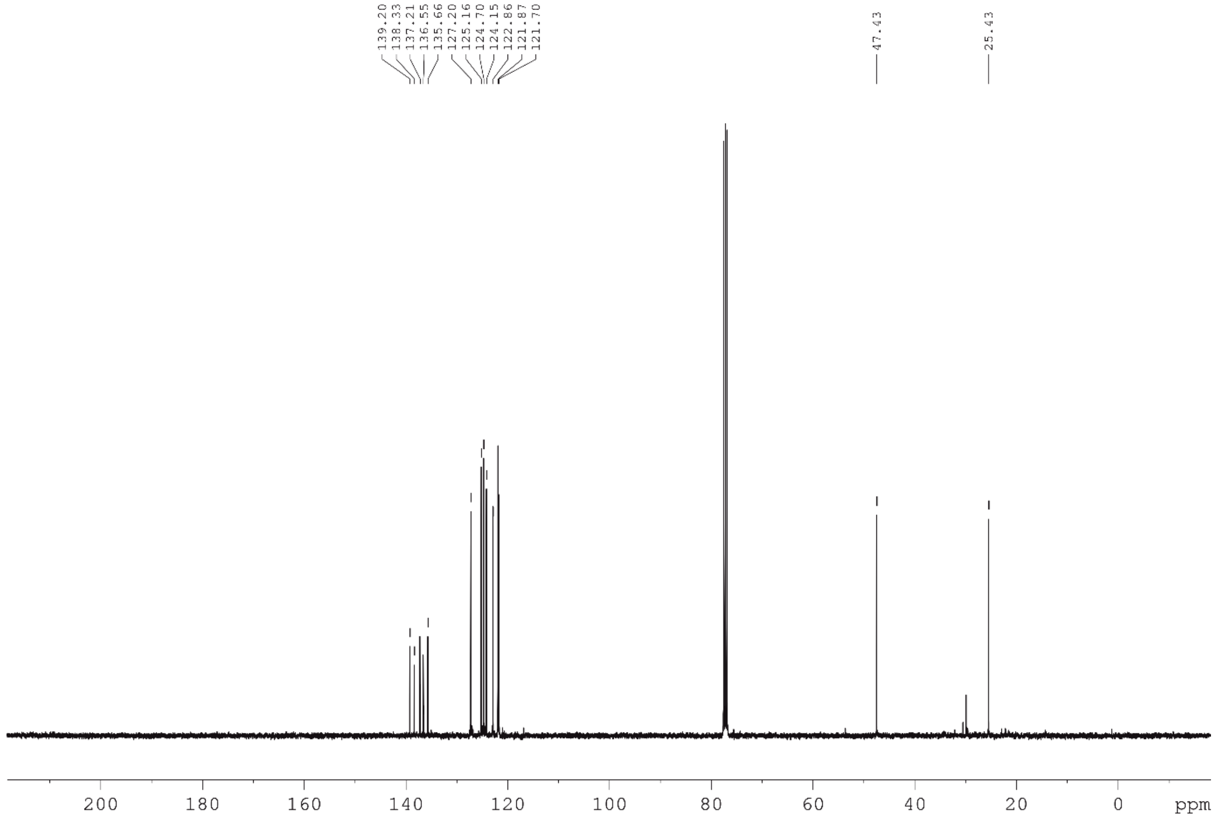


## 2-(4-(1-bromoethyl)benzyl)isoindoline-1,3-dione (1ae)

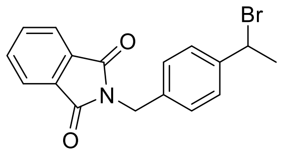


*rac*-**1ae**

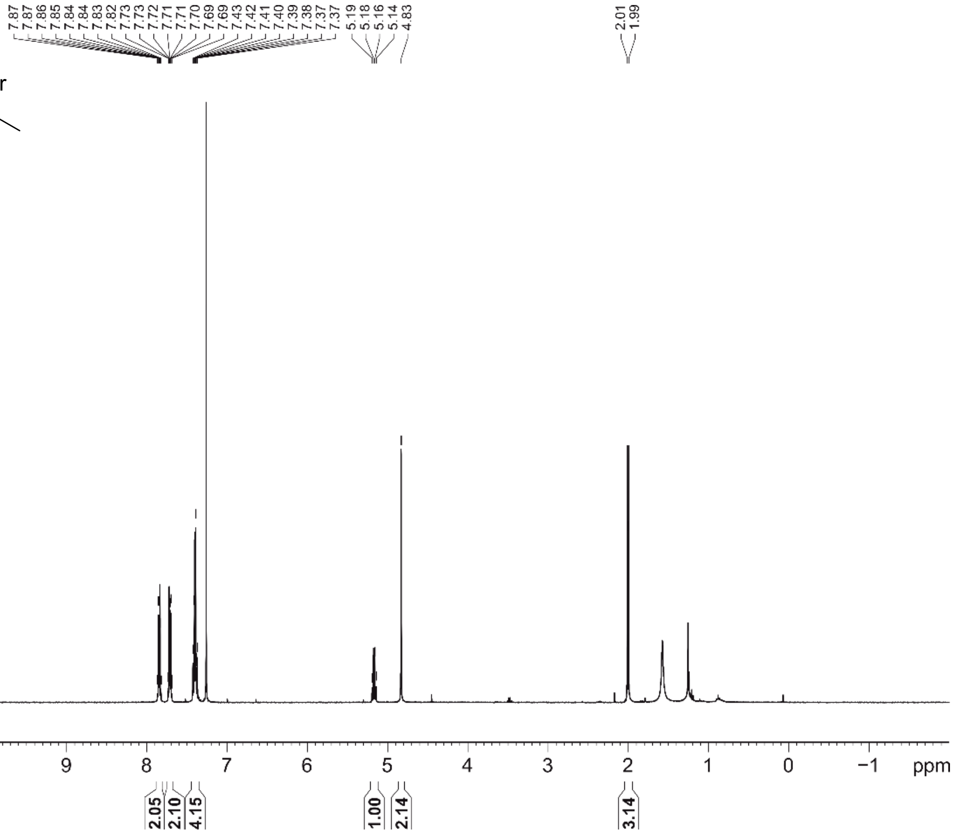

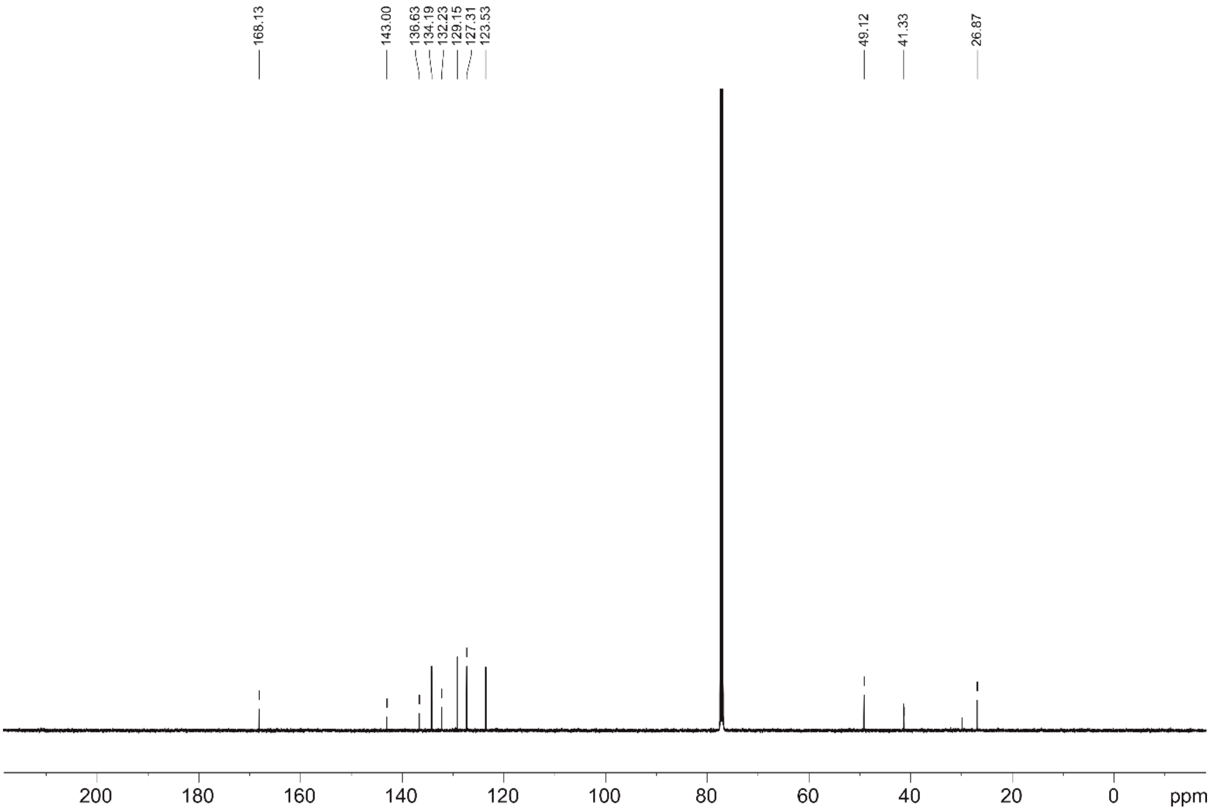


## 4-(1-bromoethyl)-6,7-dimethoxyquinazoline (1af)

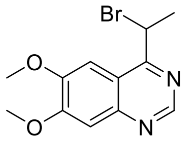


*rac*-**1af**

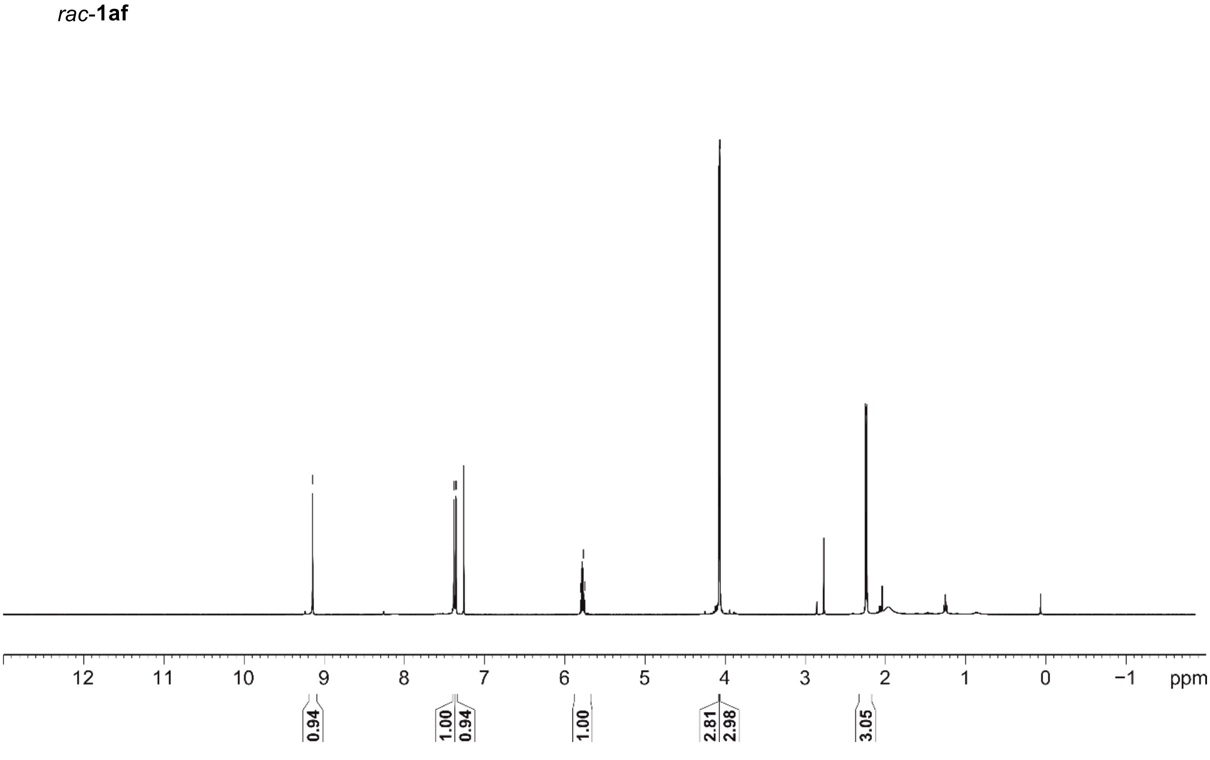

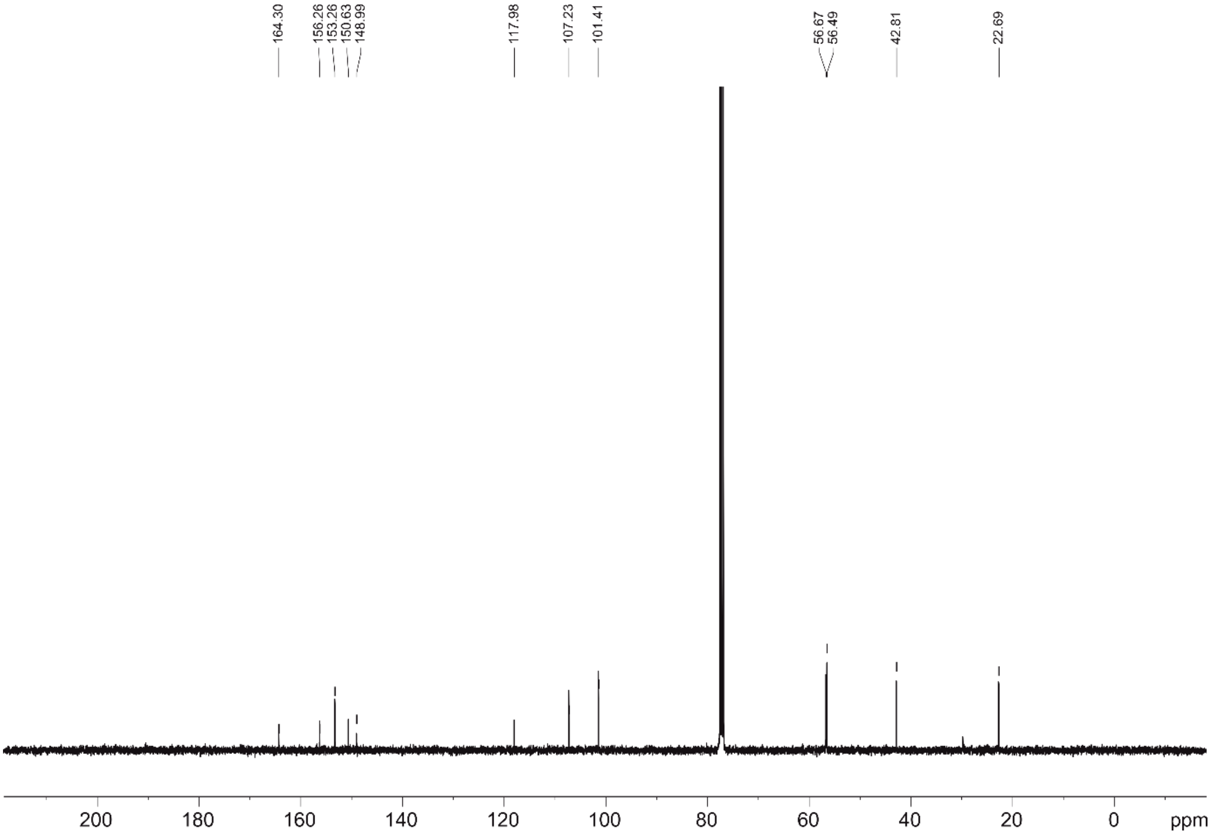


**isopropyl-2-(4-(4-(1-bromoethyl)benzoyl)phenoxy)-2-methylpropanoate (1ag)**

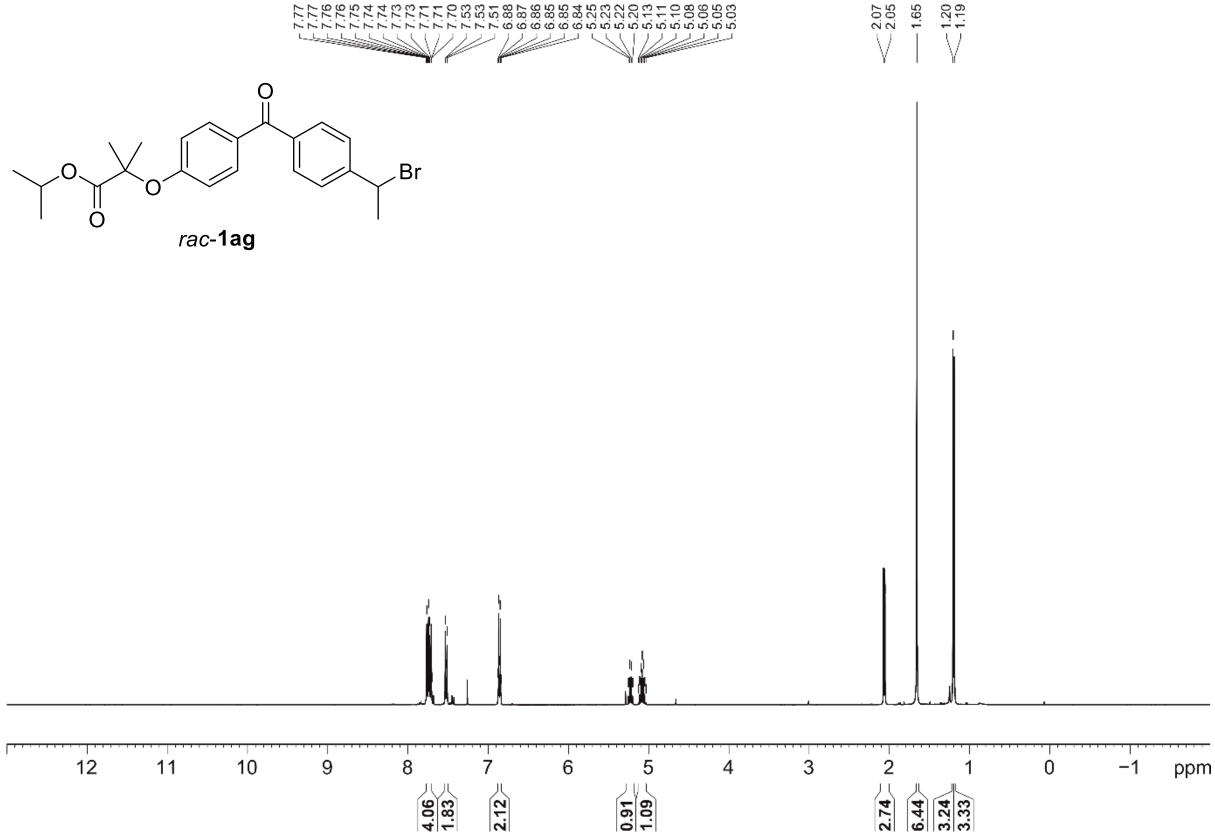

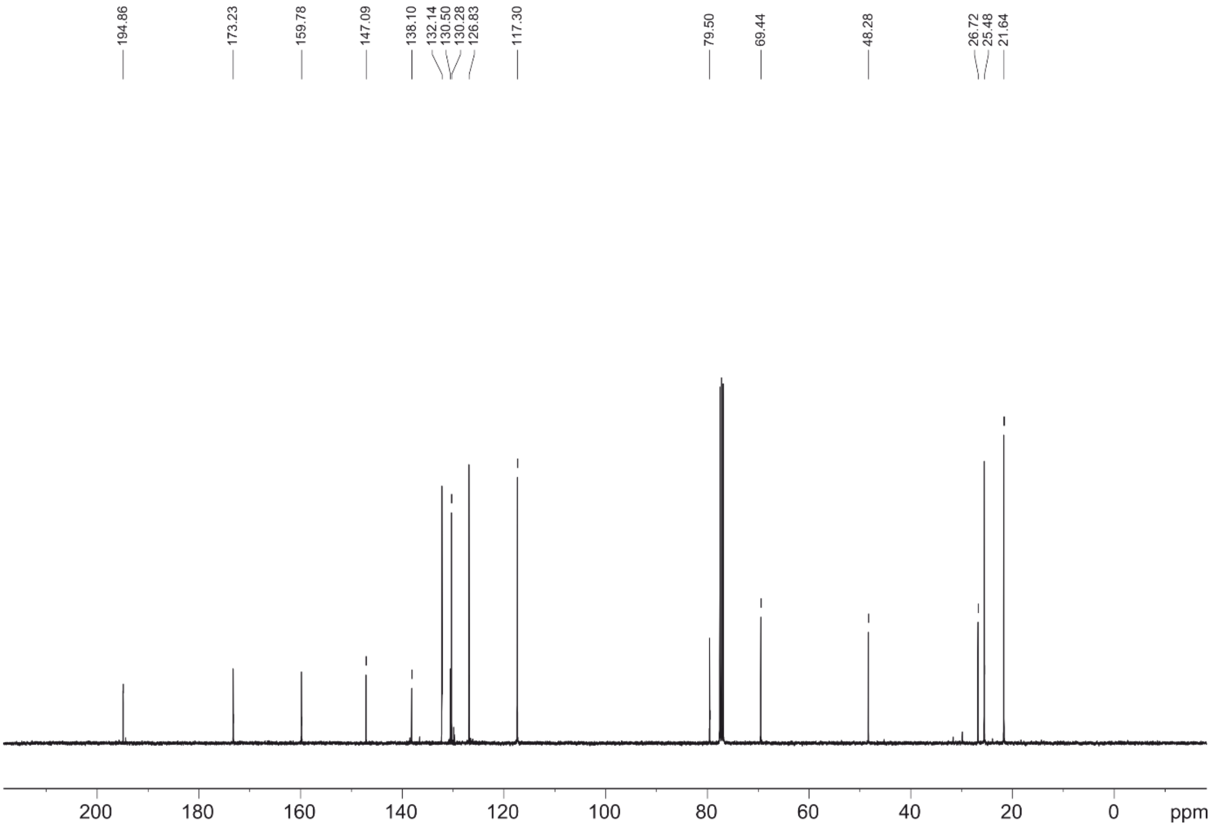


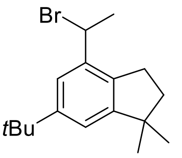


*rac*-**1ah**

**4-(1-bromoethyl)-6-(*tert*-butyl)-1,1-dimethyl-2,3-dihydro-1*H*-indene (1ah)**

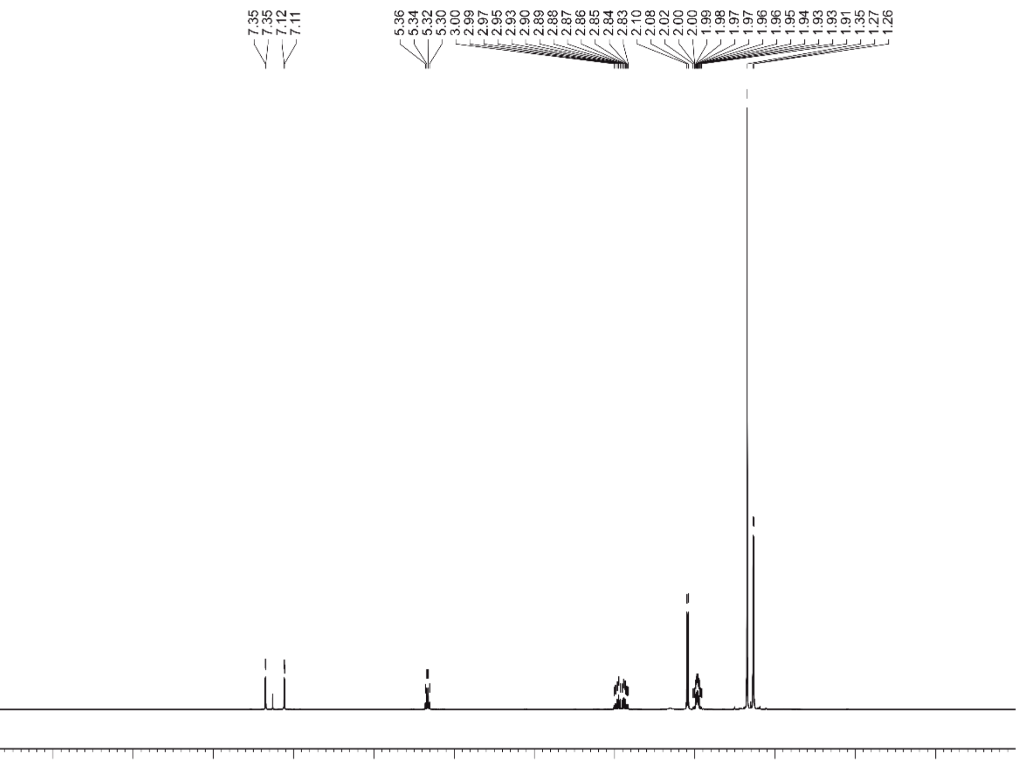

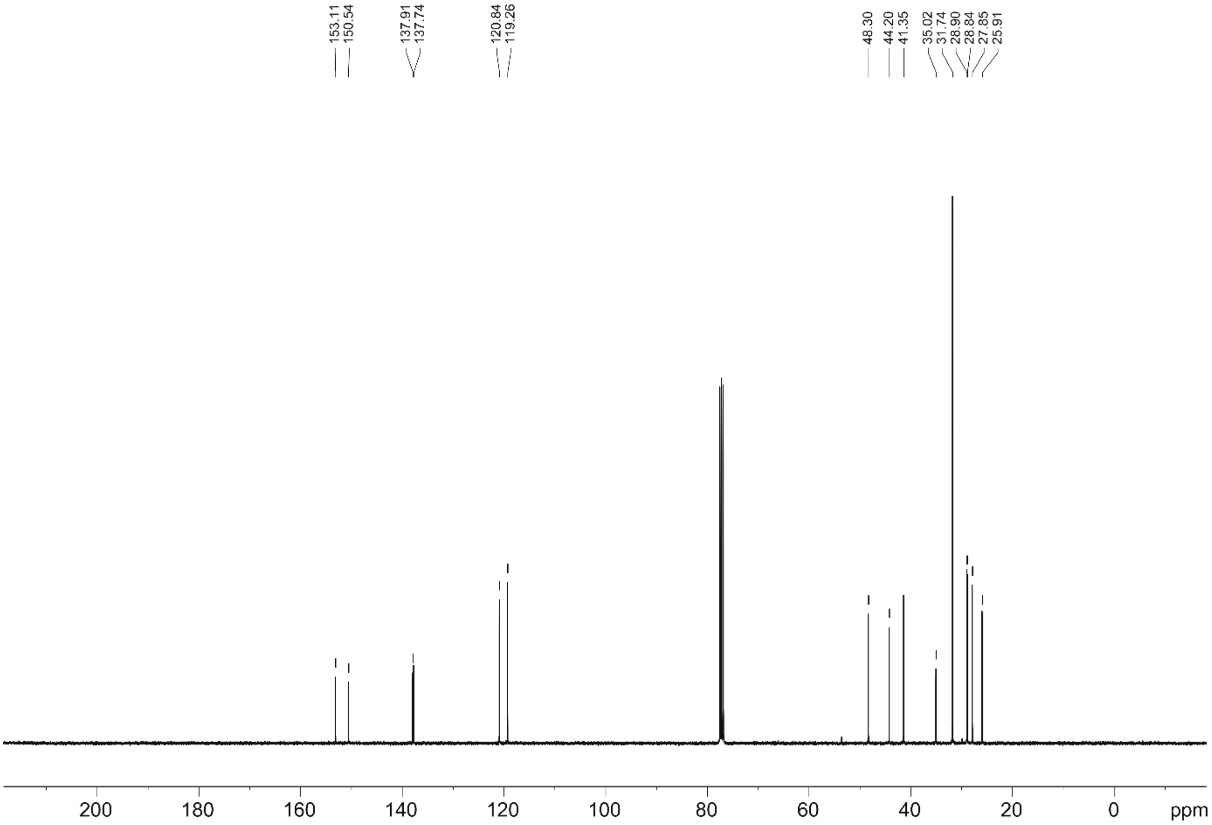


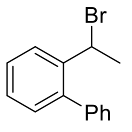


## 2-(1-bromoethyl)-1,1'-biphenyl (1ai)

*rac*-**1ai**

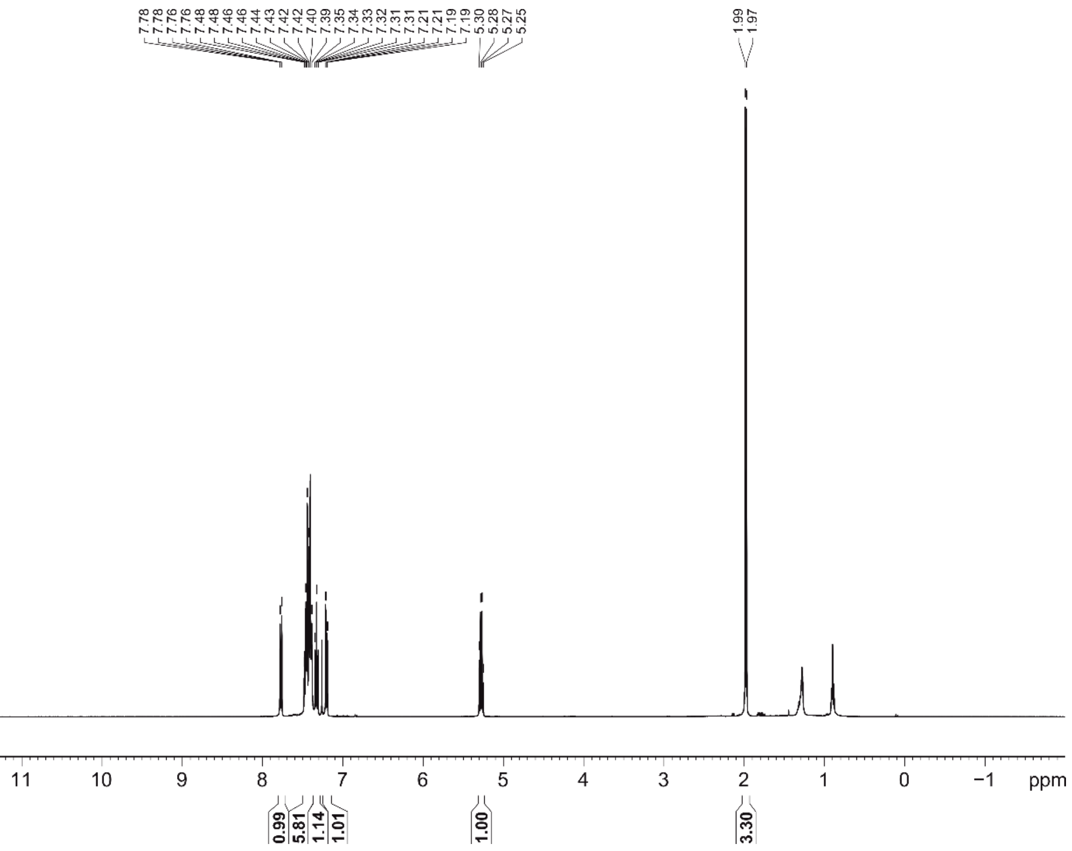

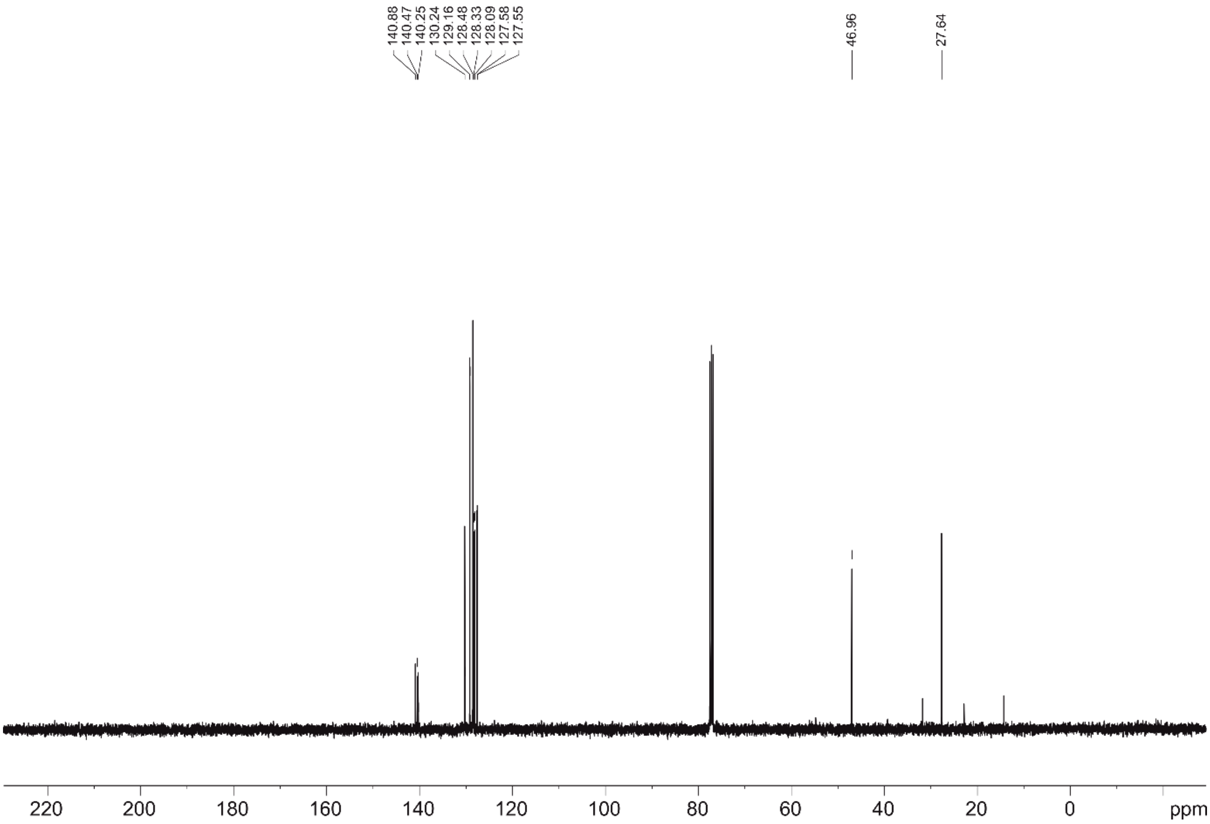


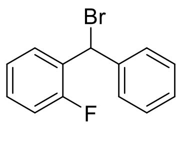


*rac*-**1aj**

**1-(bromo(phenyl)methyl)-2-fluorobenzene (1aj)**

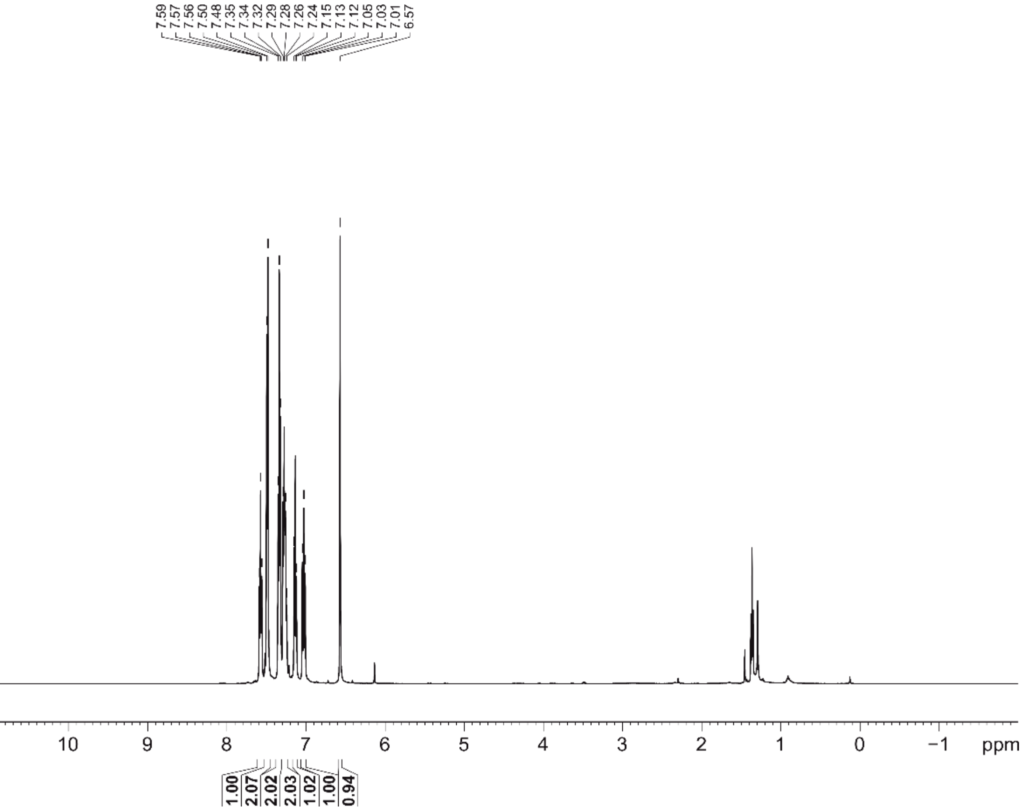

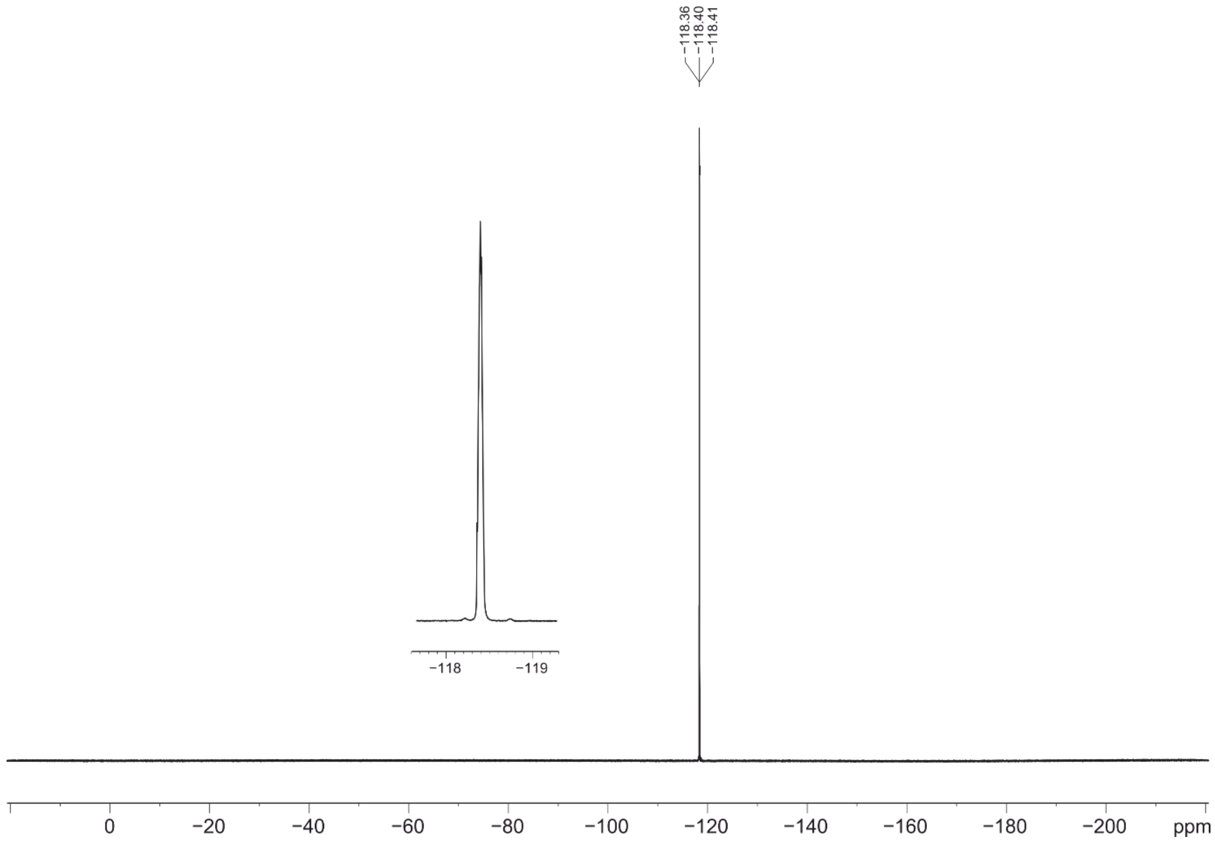
-118.36
-118.40
-118.41
−118
−119
0
−20
−40
−60
−80
−100
−120
−140
−160
−180
−200
ppm

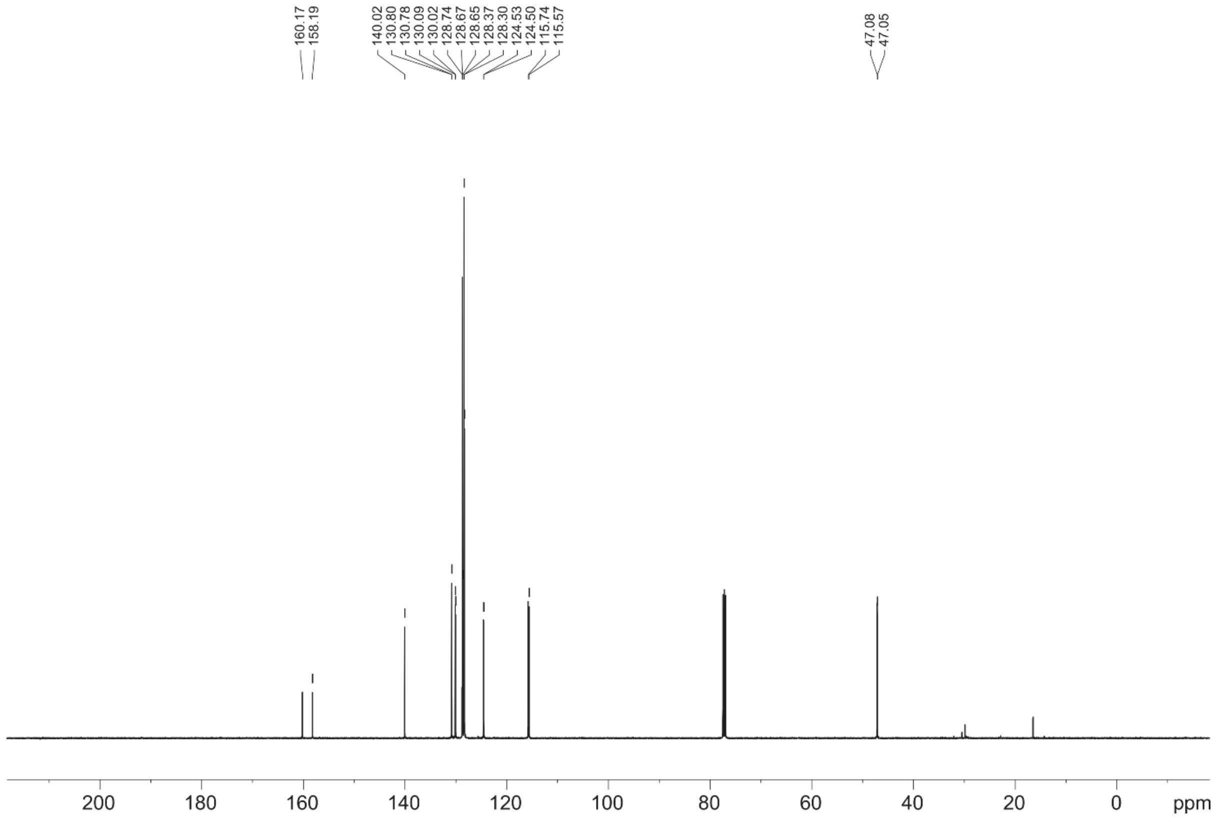
160.17
158.19
140.02
130.80
130.78
130.09
130.02
128.74
128.67
128.65
128.37
128.30
124.53
124.50
115.74
115.57
47.08
47.05
200
180
160
140
120
100
80
60
40
20
0
ppm

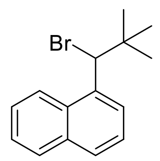


*rac*-**1ak**

**1-(1-bromo-2,2-dimethylpropyl)naphthalene (1ak)**

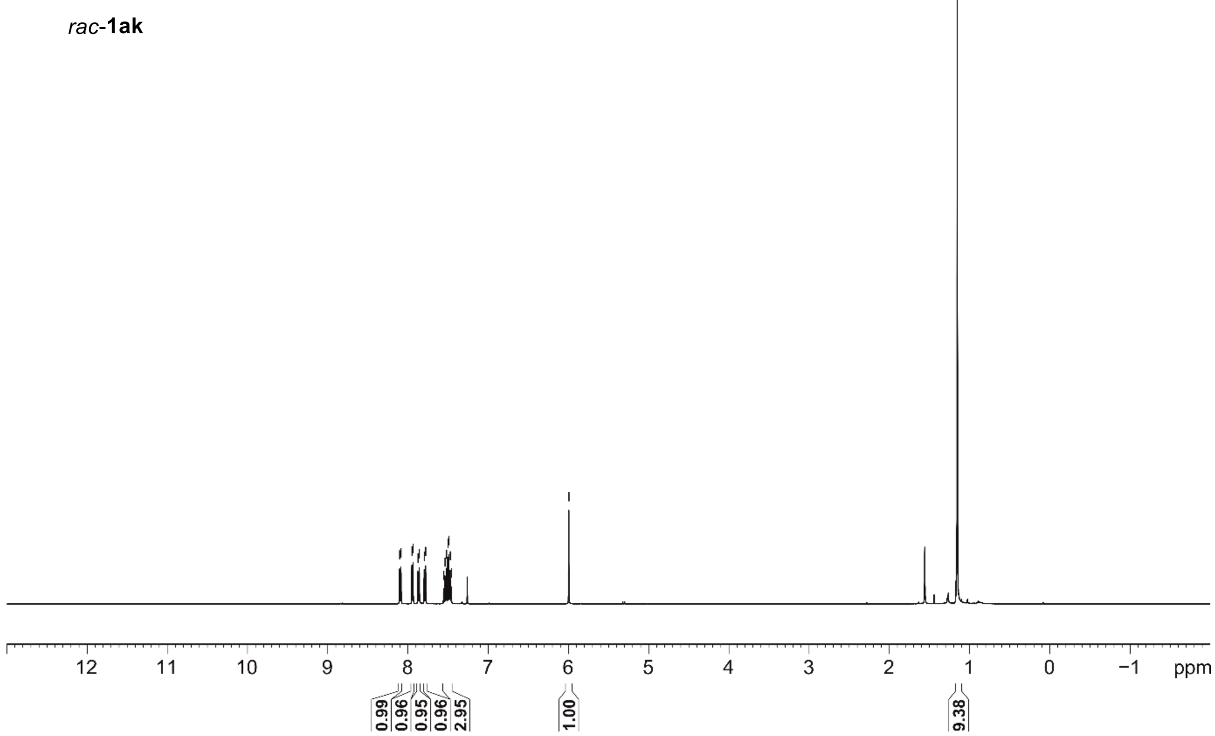


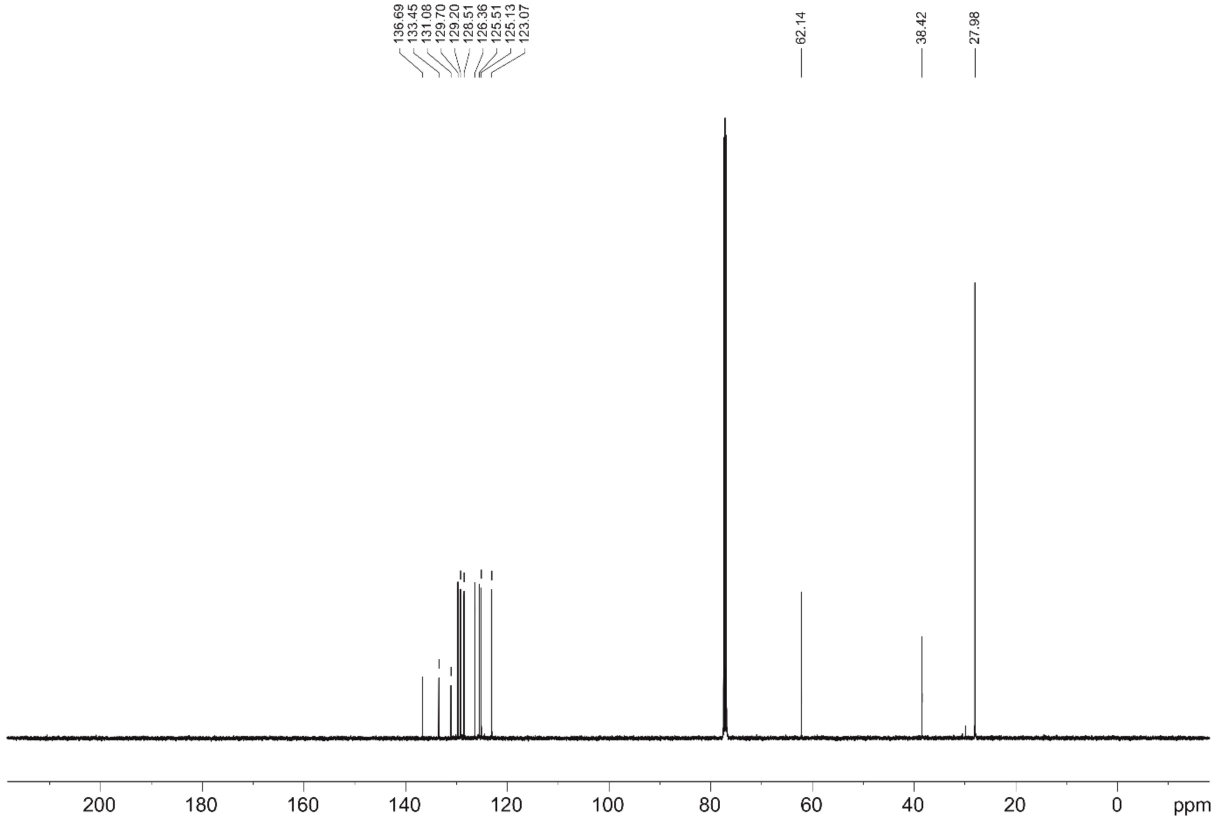

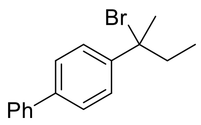


*rac*-**1al**

**4-(2-bromobutan-2-yl)-1,1'-biphenyl (1al)**

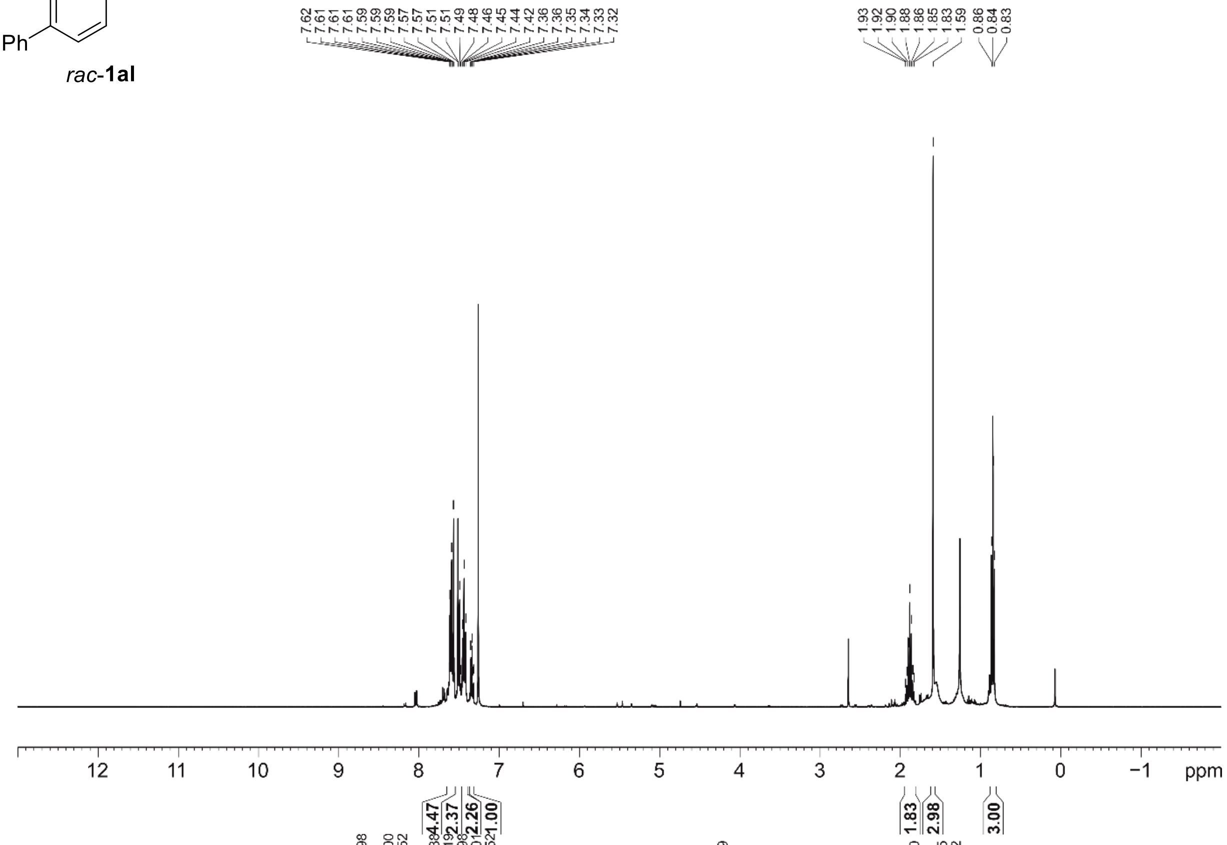


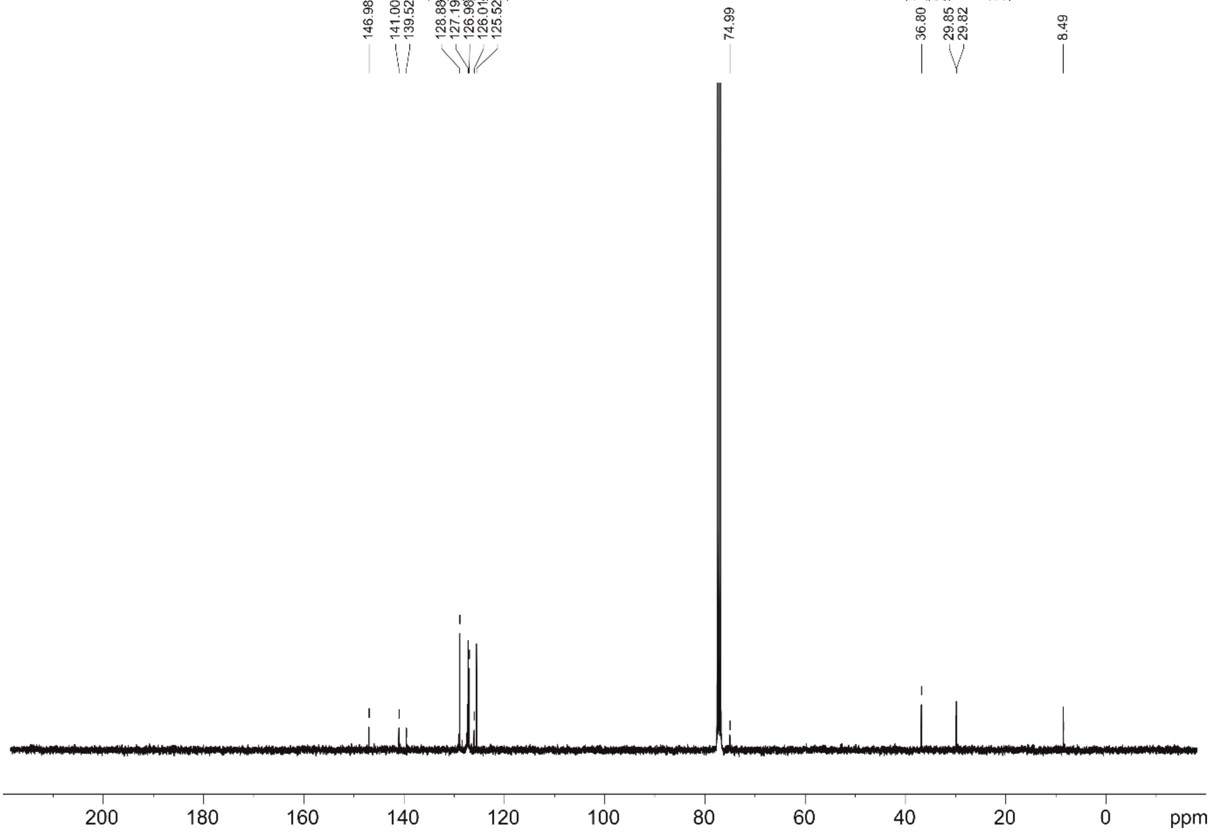

**2-bromo-1-(naphthalen-1-yl)propan-1-one (9d)**

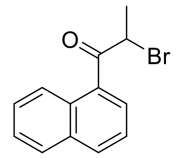


*rac*-**9d**

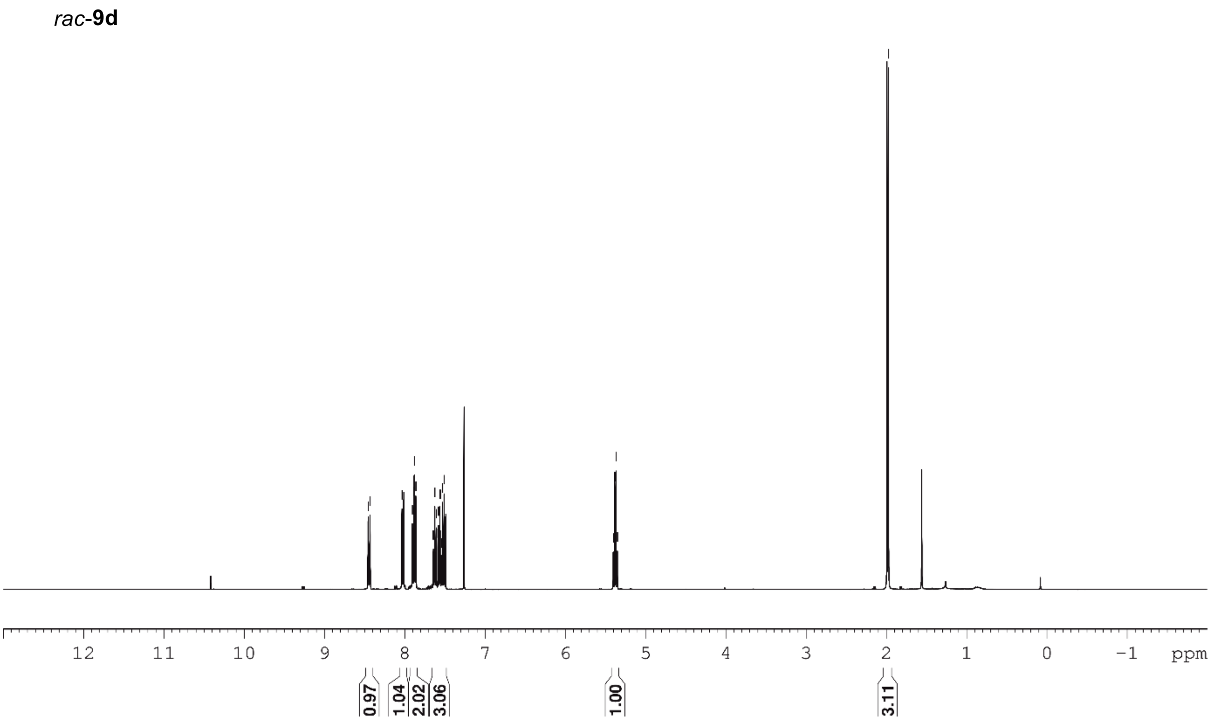


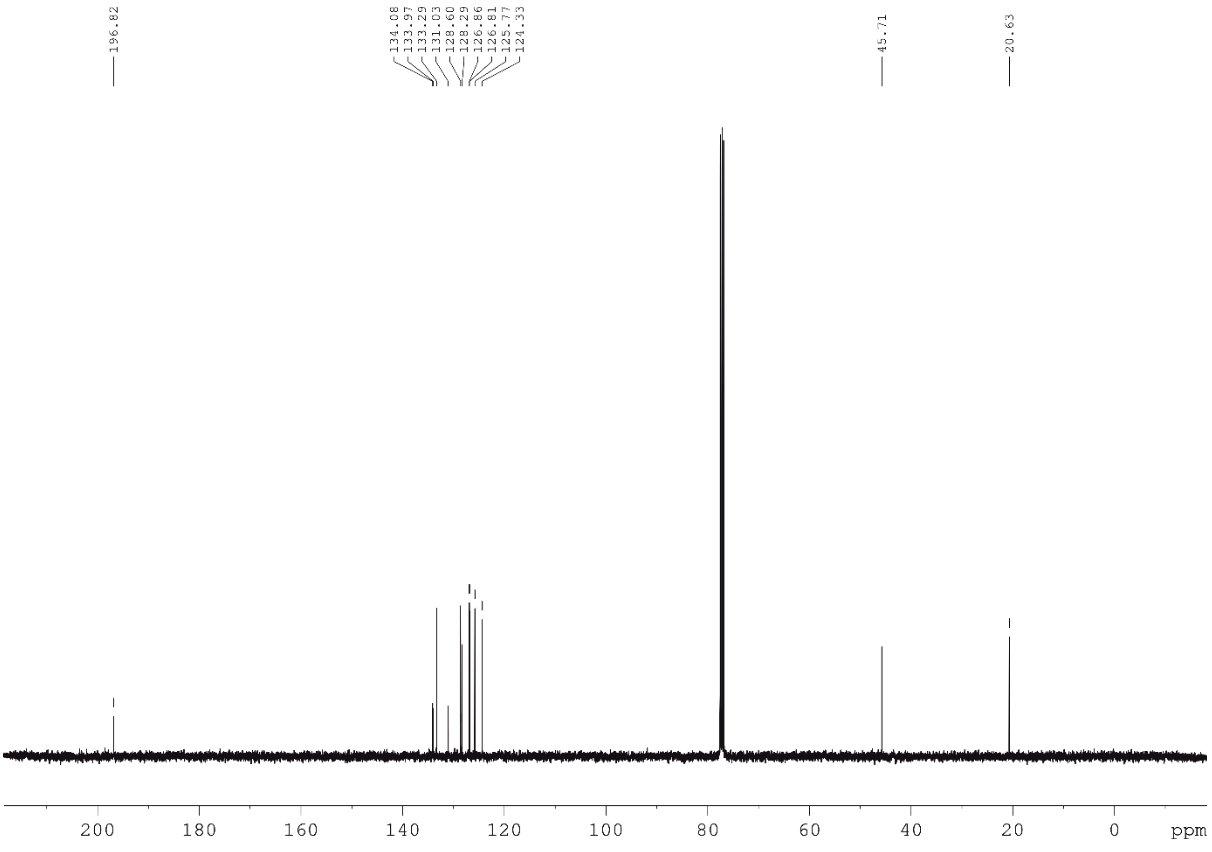

## 2-bromo-1-(4-fluoronaphthalen-1-yl)propan-1-one (9e)

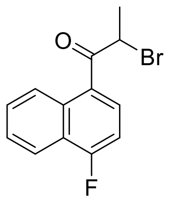


*rac*-**9e**

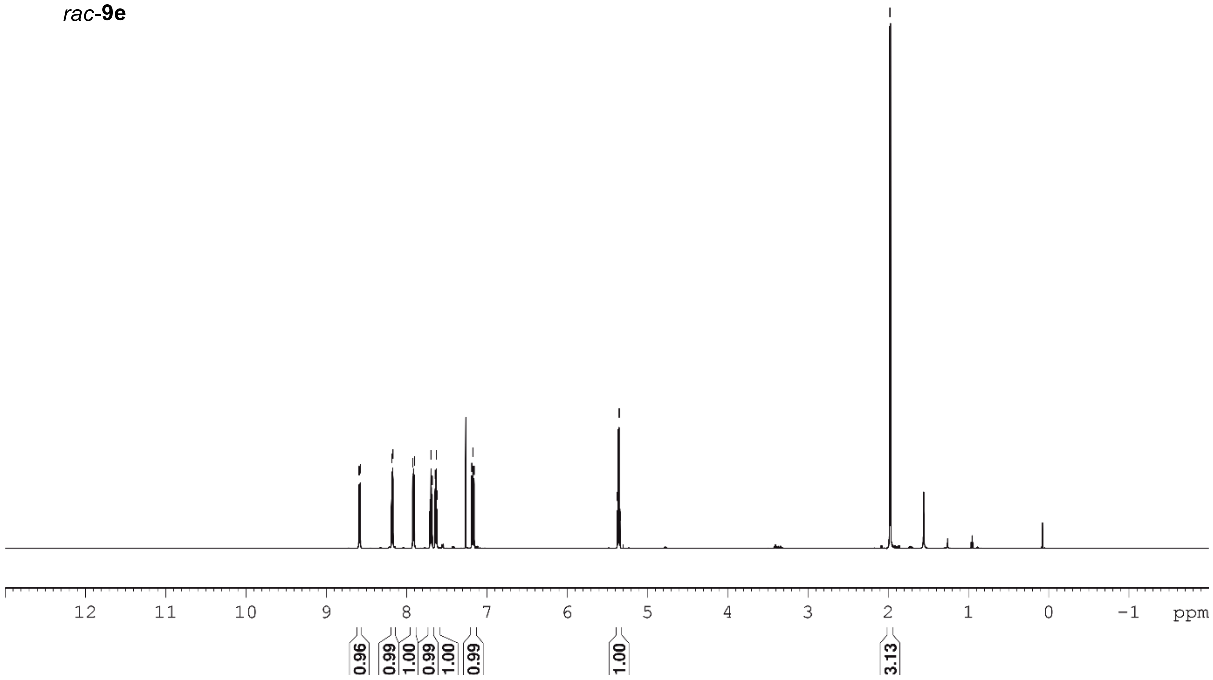


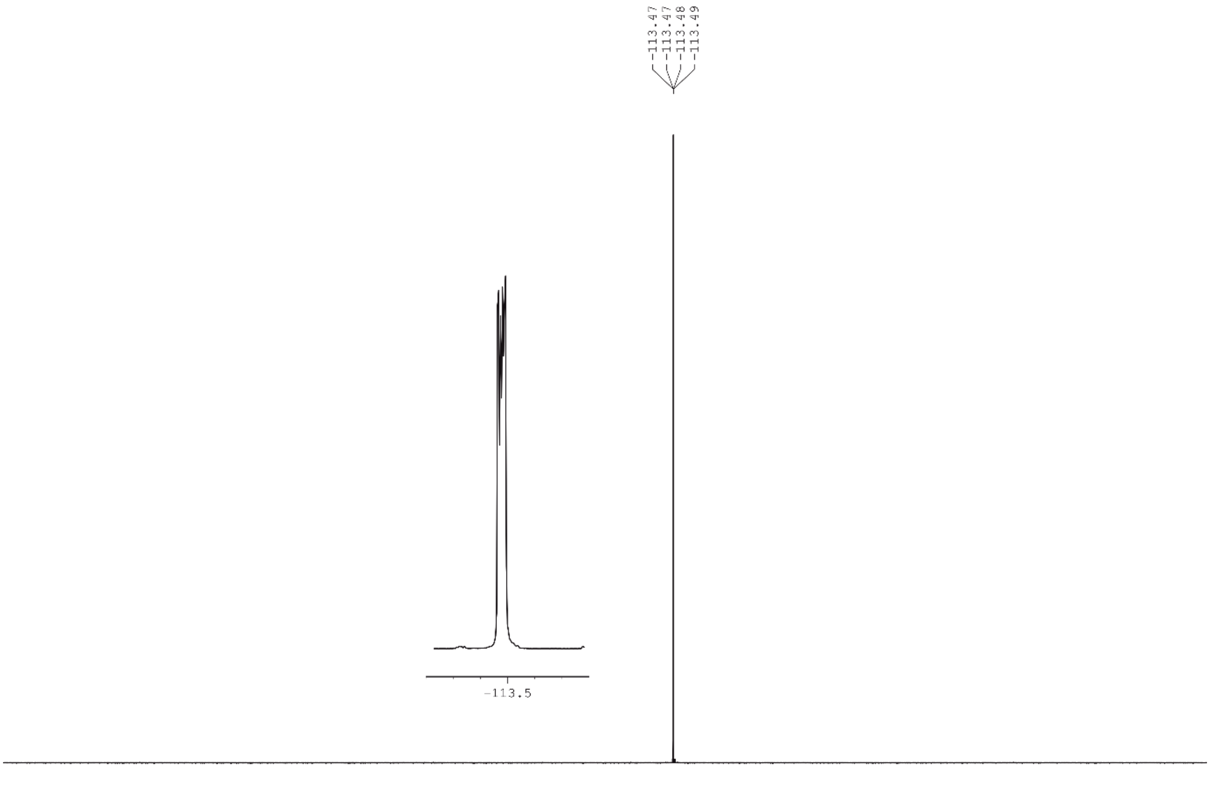

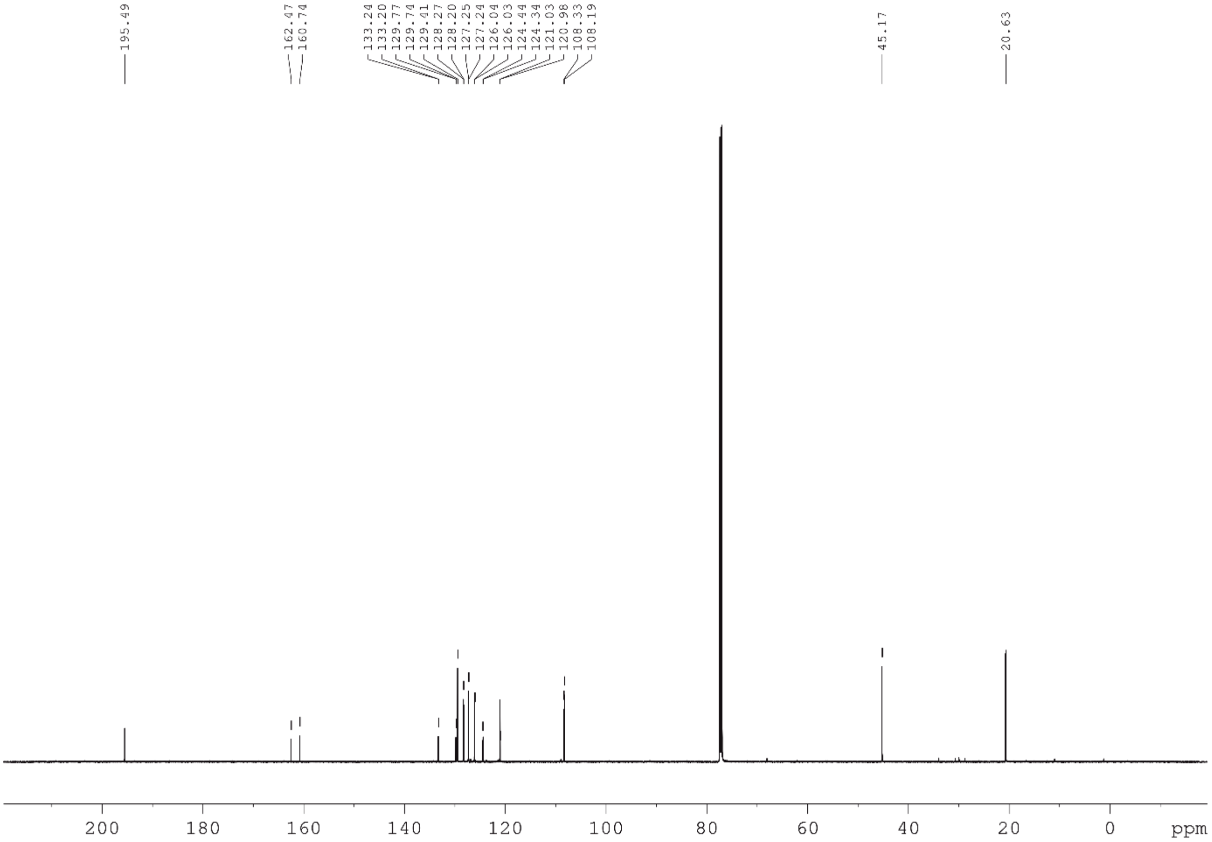


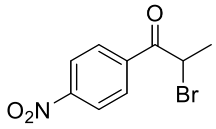


*rac*-**9f**

**2-bromo-1-(4-nitrophenyl)propan-1-one 9 (9f)**

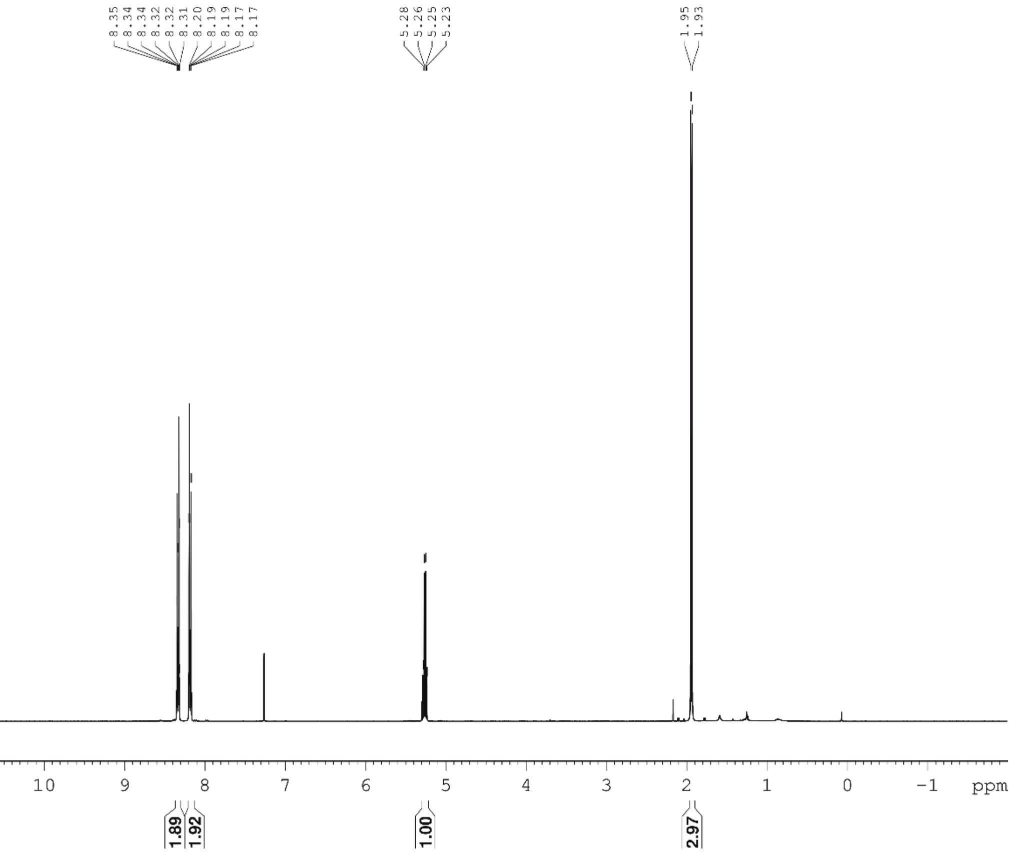

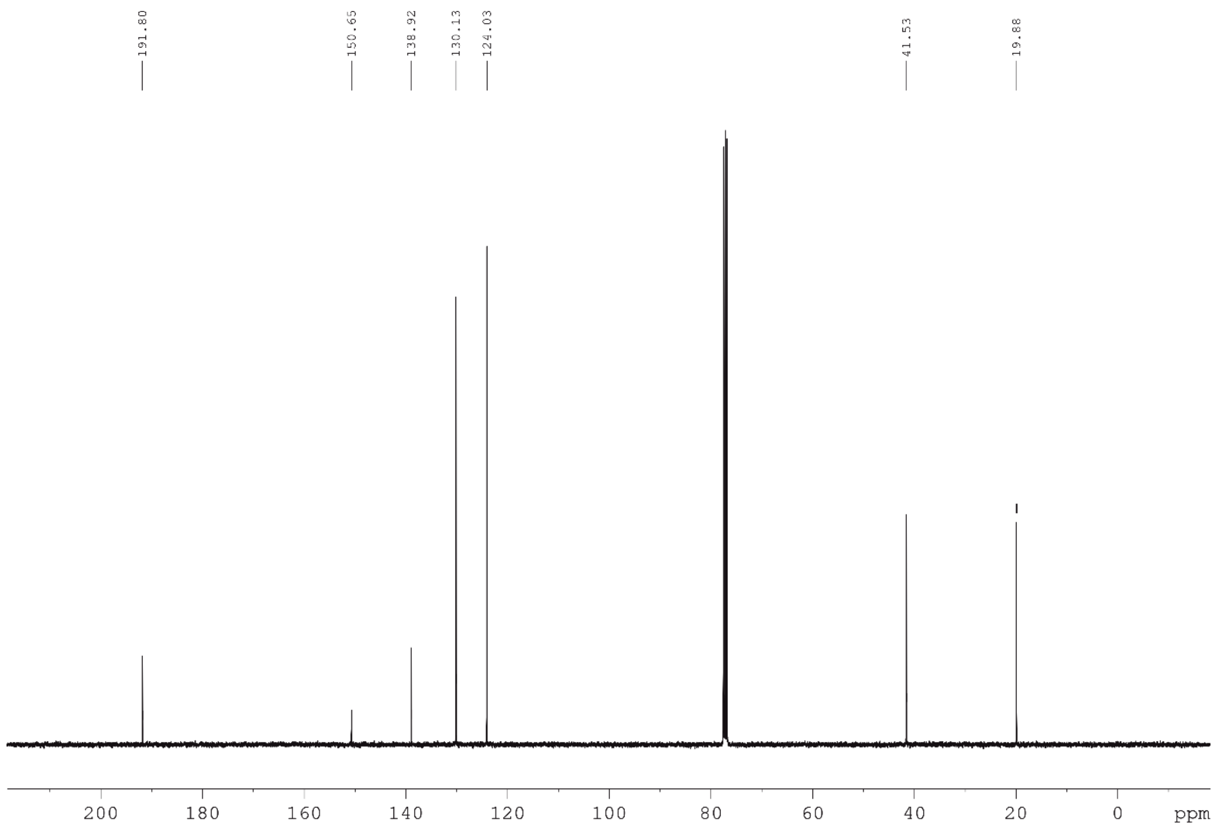


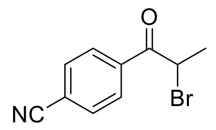


*rac*-**9g**

**4-(2-bromopropanoyl)benzonitrile (9g)**

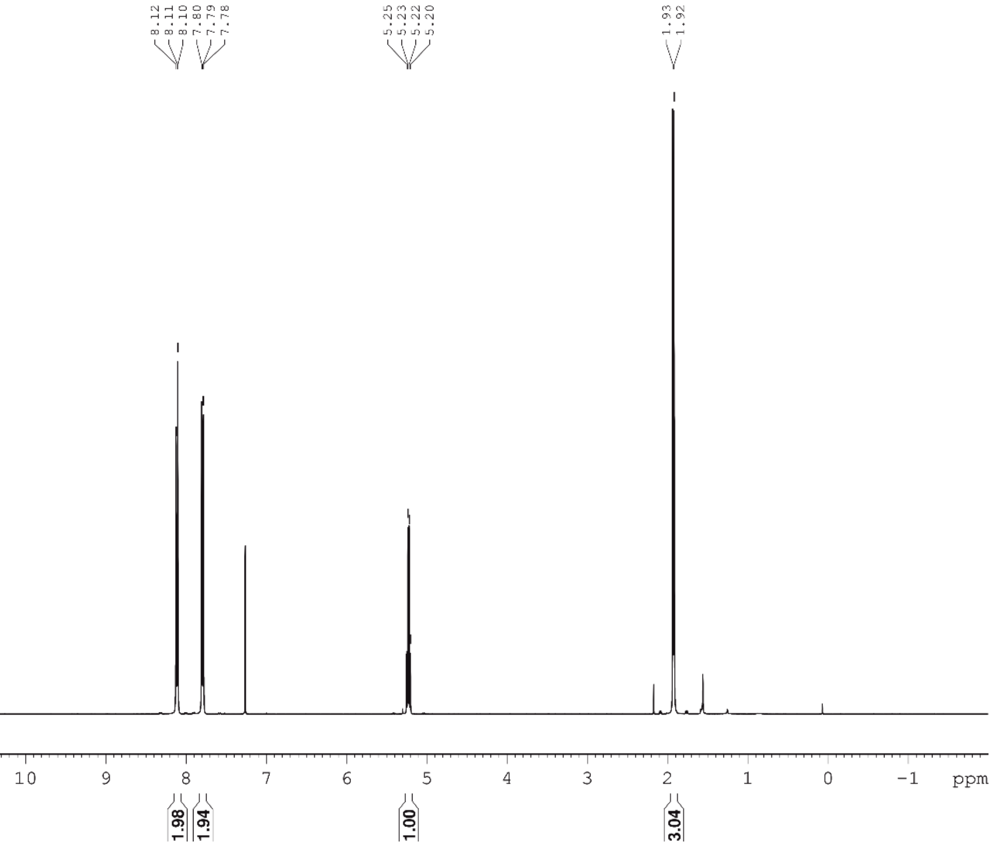

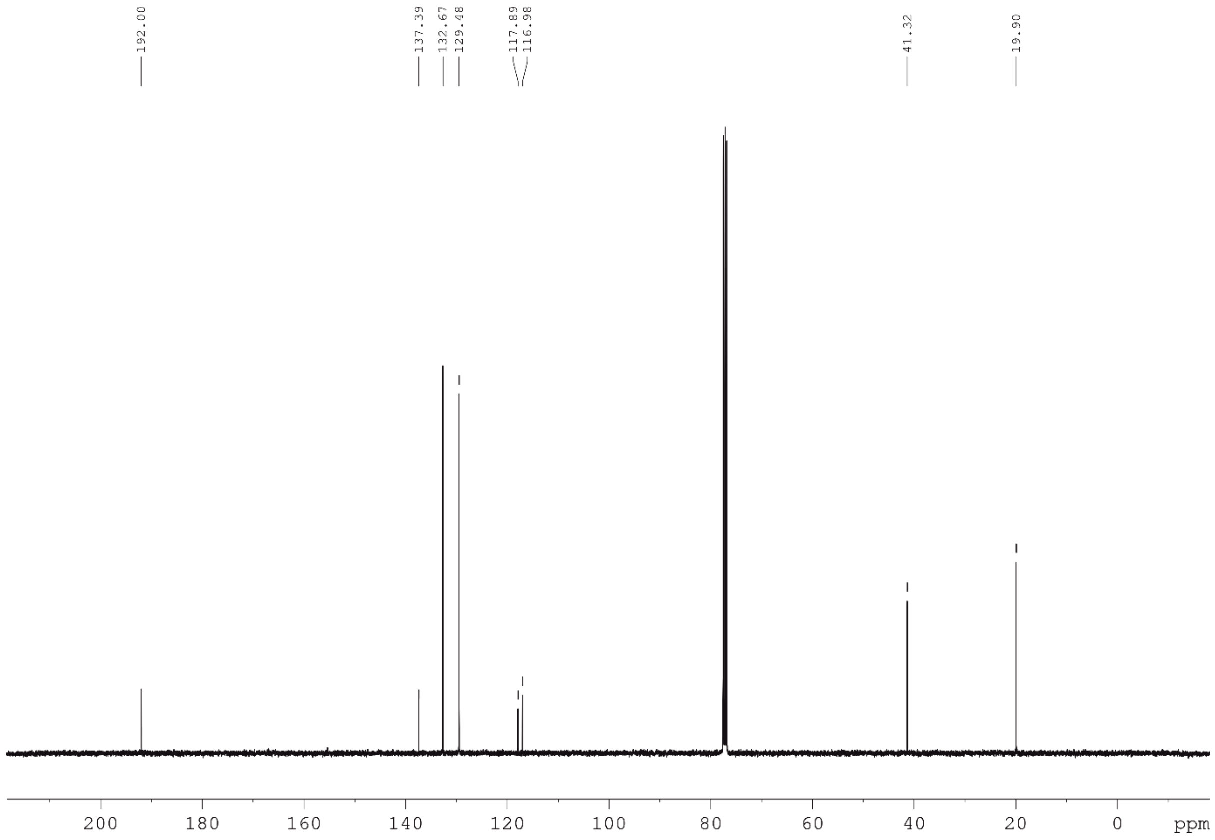


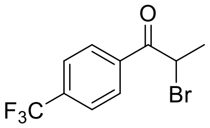

*rac*-**9h**

**2-bromo-1-(4-(trifluoromethyl)phenyl)propan-1-one (9h)**

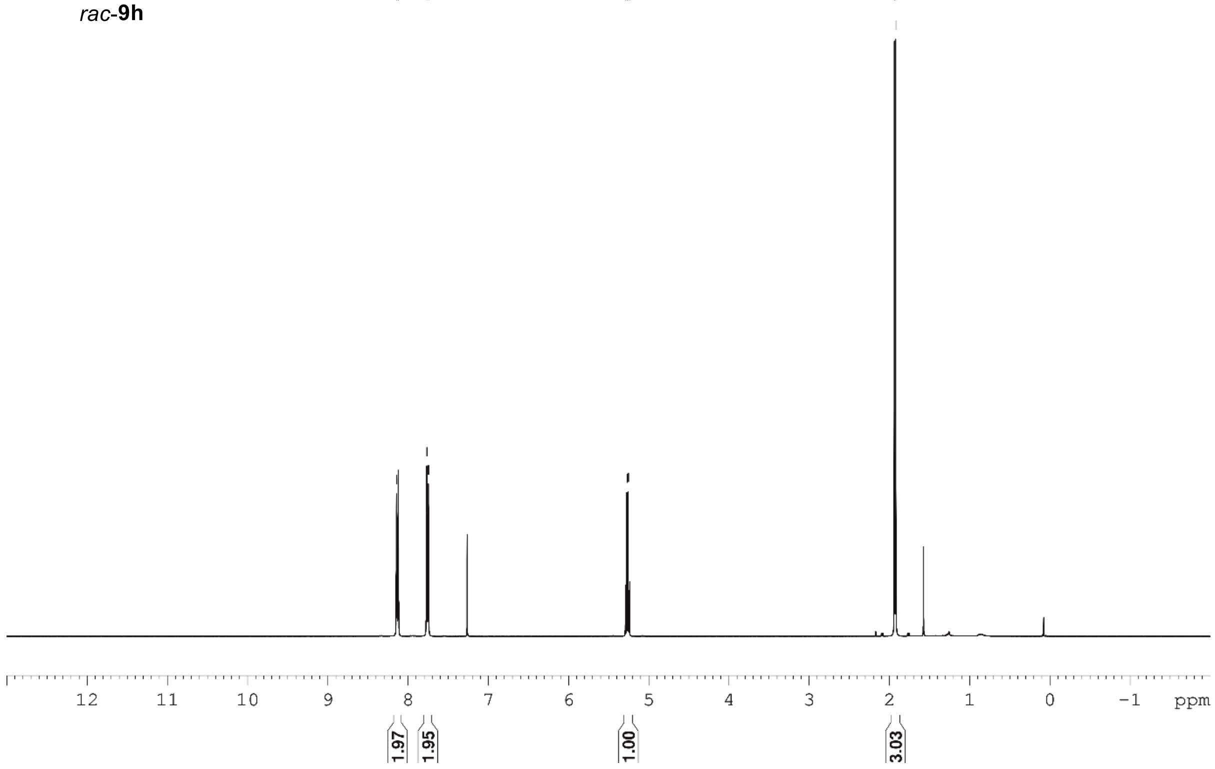

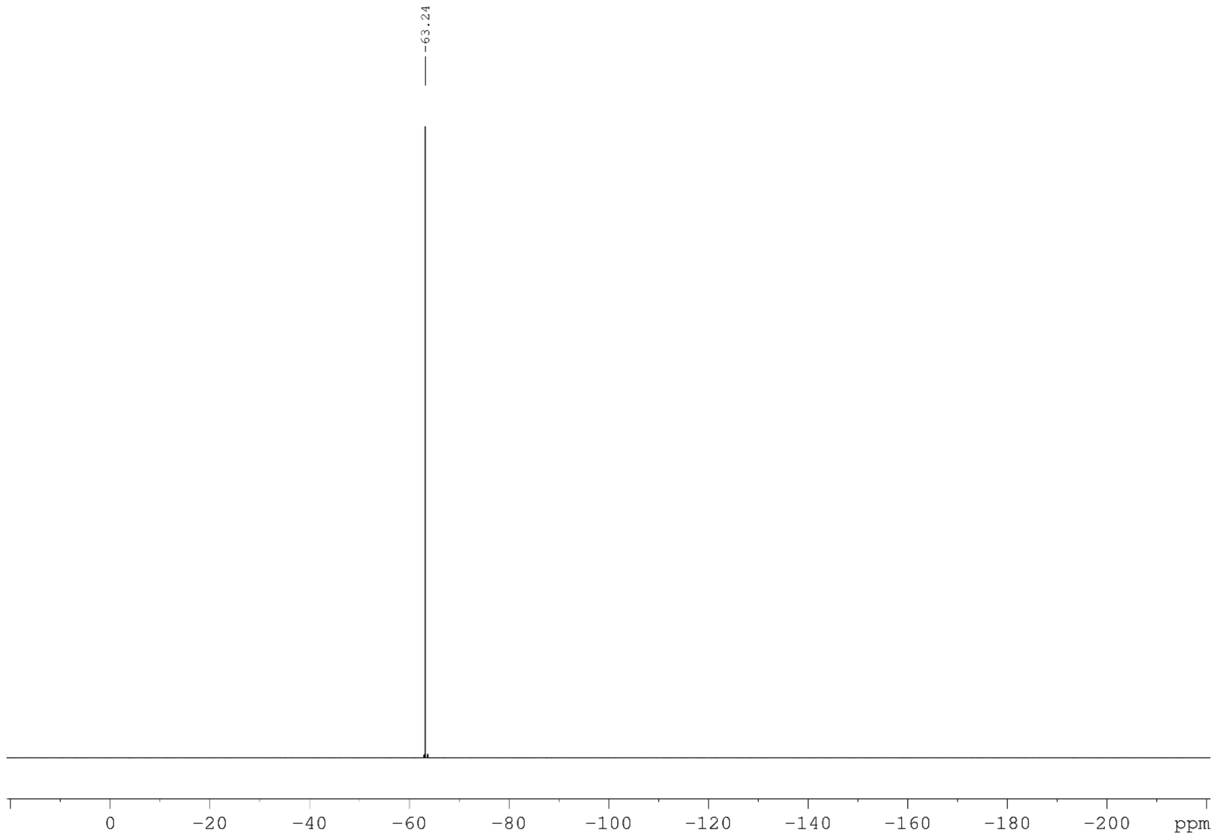
-63.24
0
-20
-40
-60
-80
-100
-120
-140
-160
-180
-200
ppm

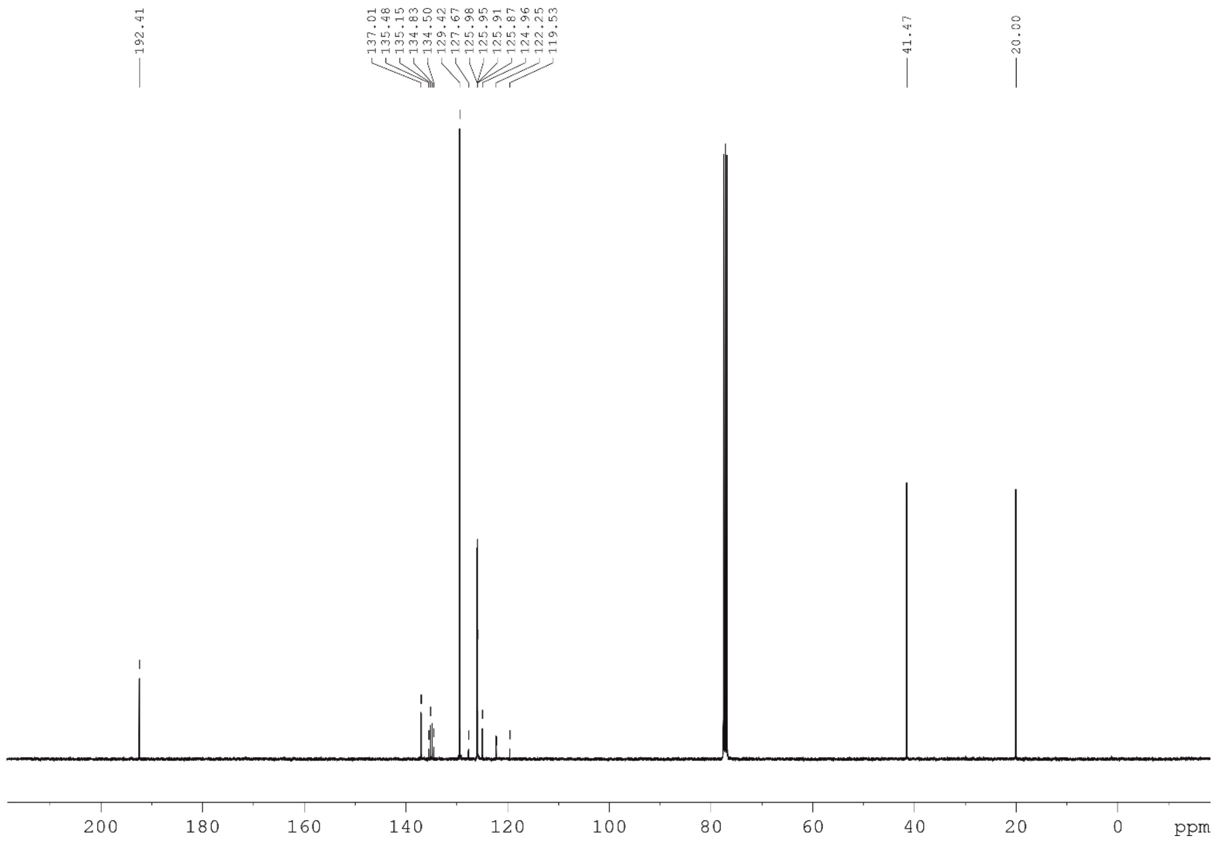
192.41
137.01
135.48
135.15
134.83
134.50
129.42
127.67
125.98
125.95
125.91
125.87
124.96
122.25
119.53
41.47
20.00
200
180
160
140
120
100
80
60
40
20
0
ppm

## 1-([1,1'-biphenyl]-4-yl)-2-bromobutan-1-one (9l)

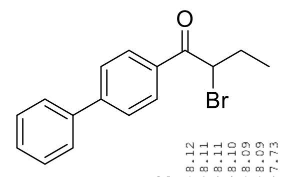


*rac*-**9l**

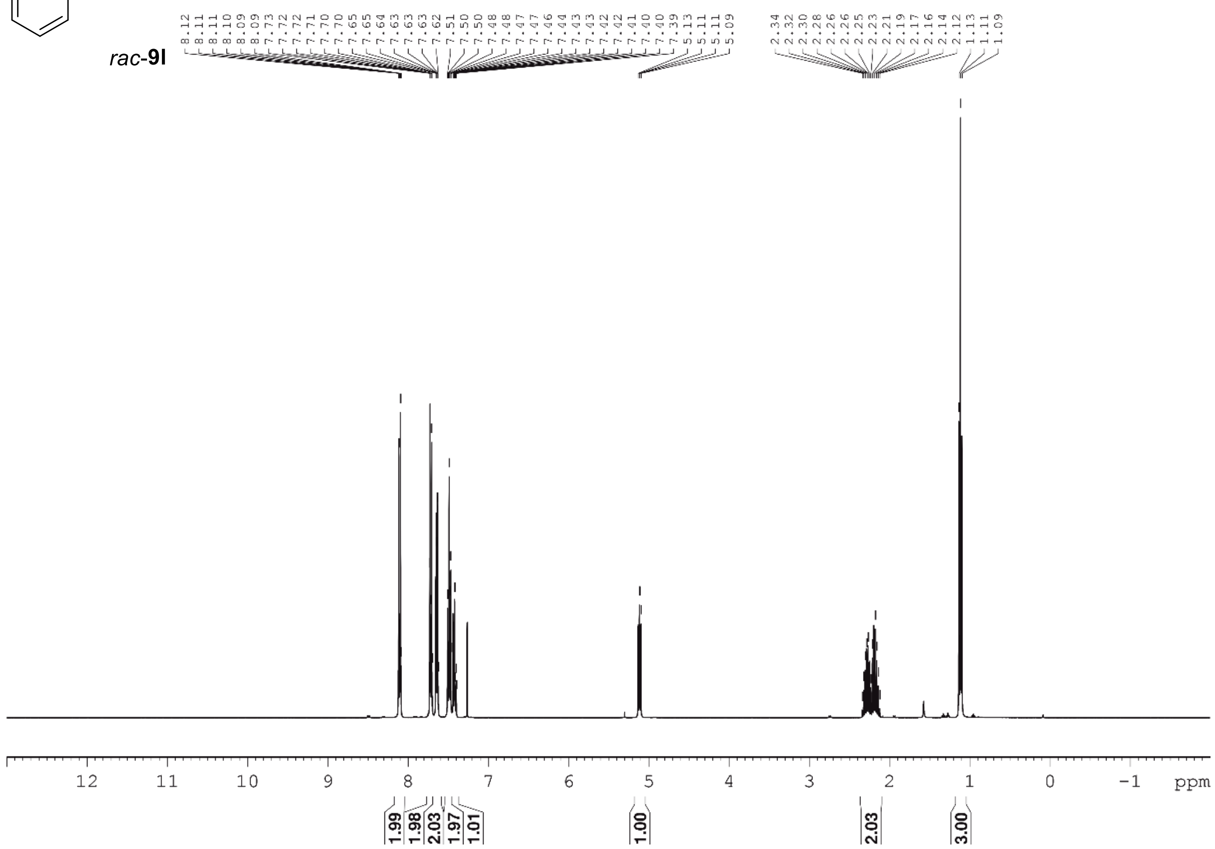


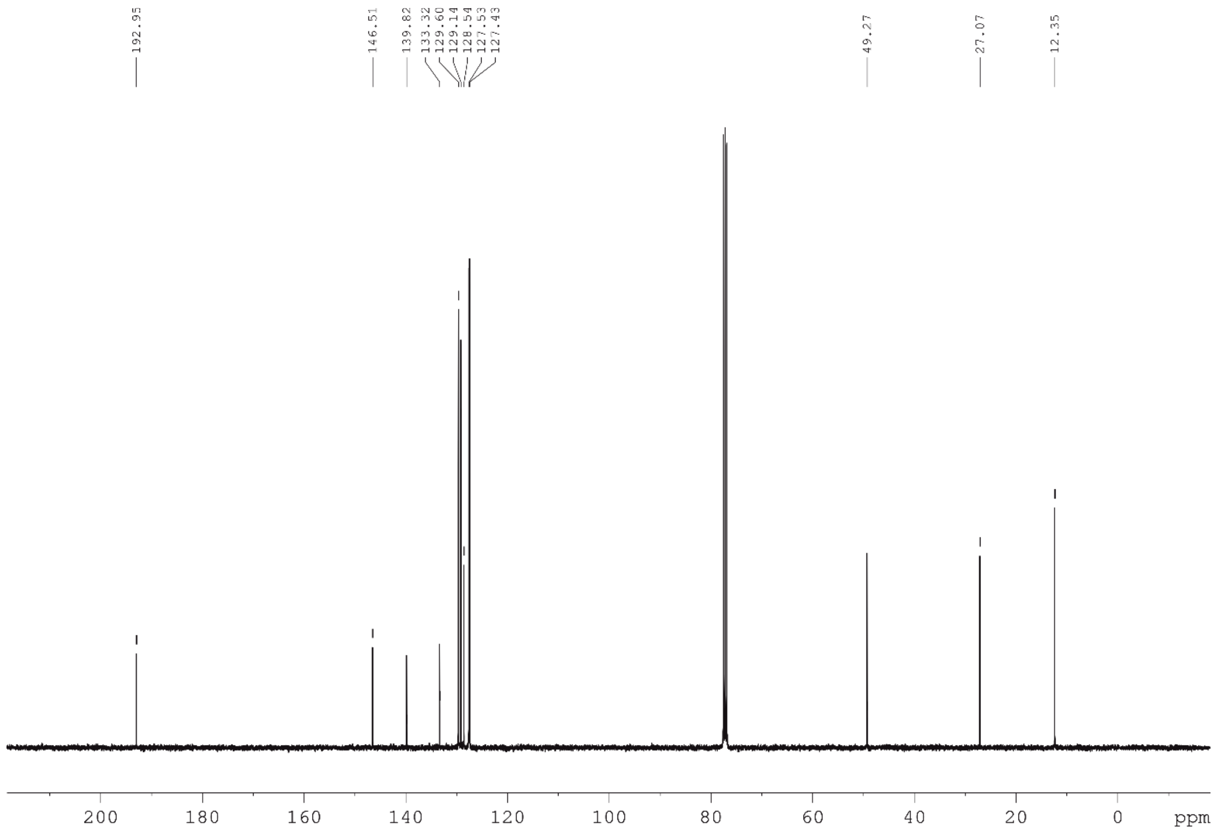

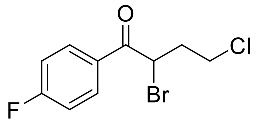


*rac*-**9m**

**2-bromo-4-chloro-1-(4-fluorophenyl)butan-1-one (9m)**

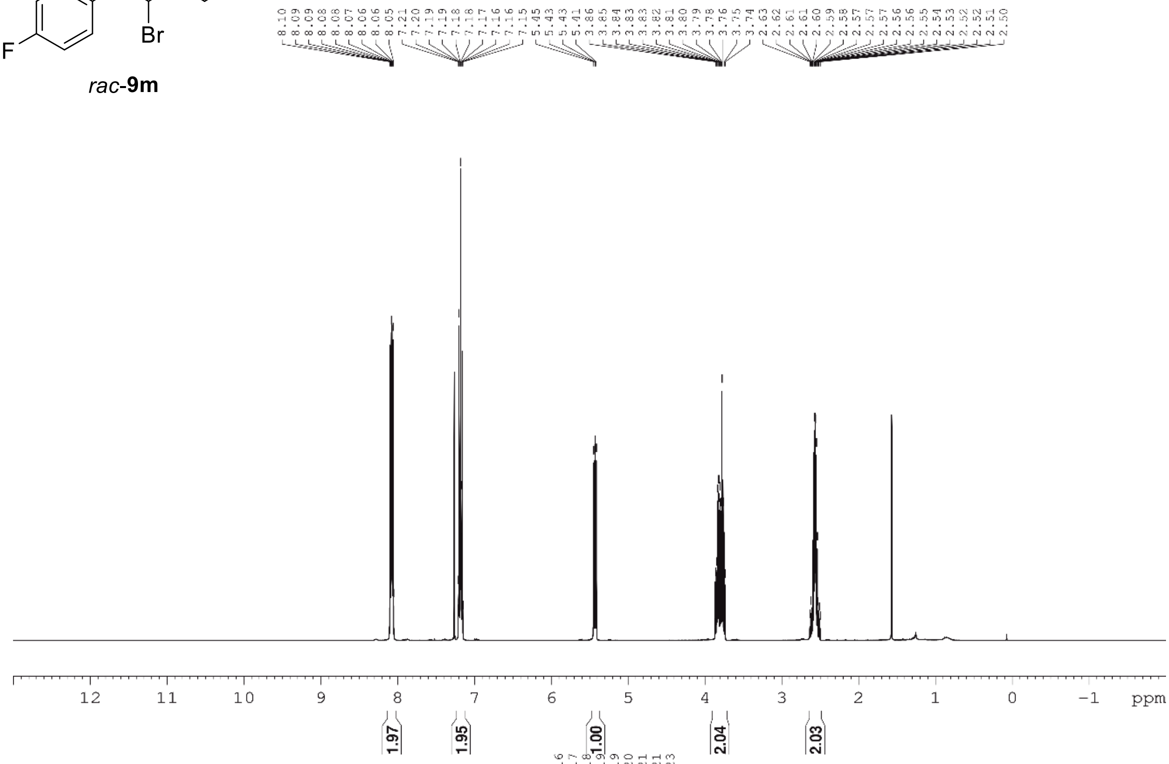


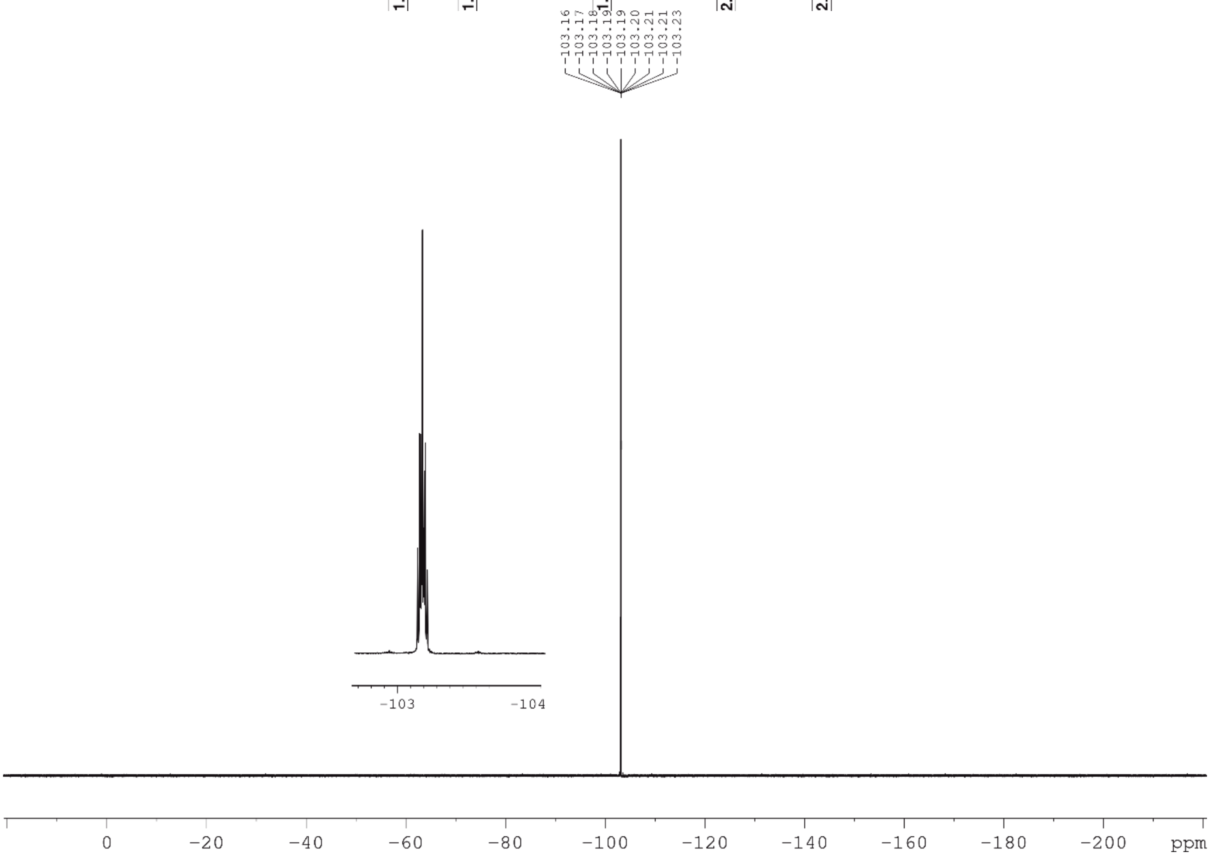

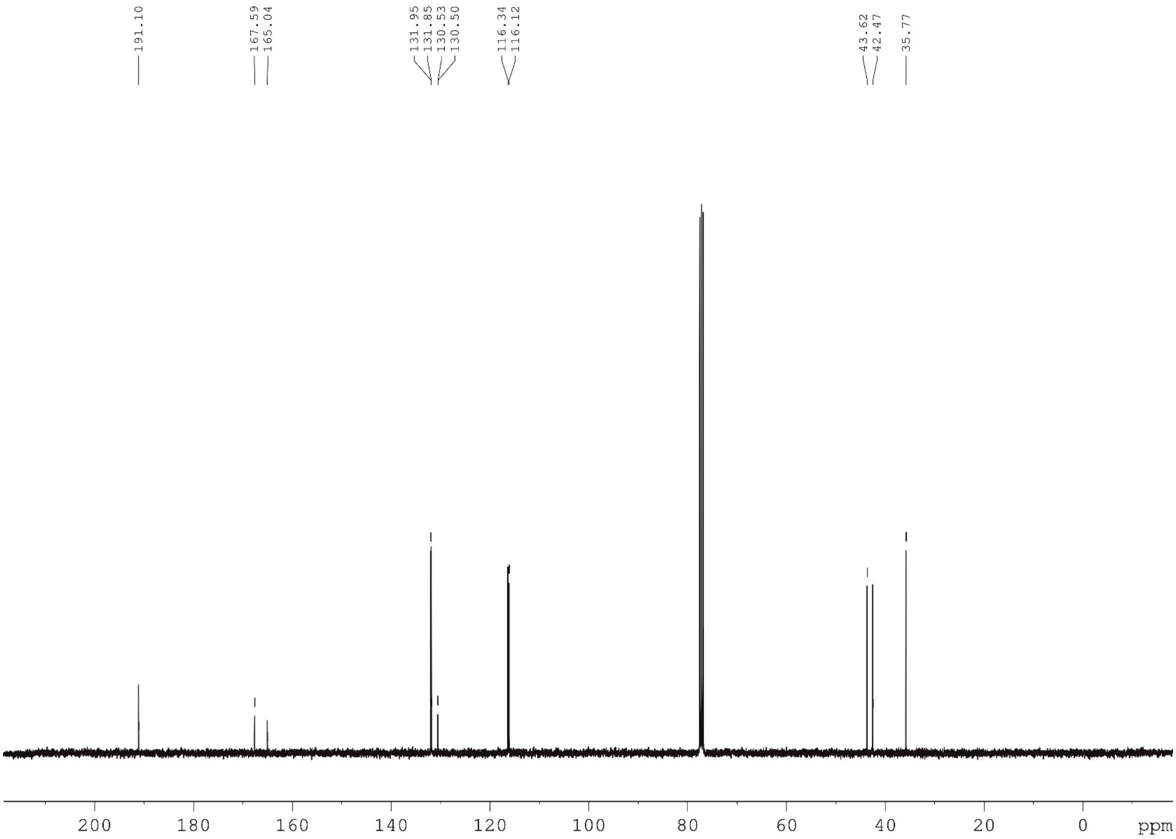


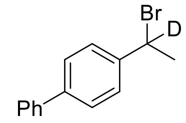


**1a-$d_1$**

**4-(1-bromoethyl-1-*d*)-1,1′-biphenyl (1a-$d_1$)**

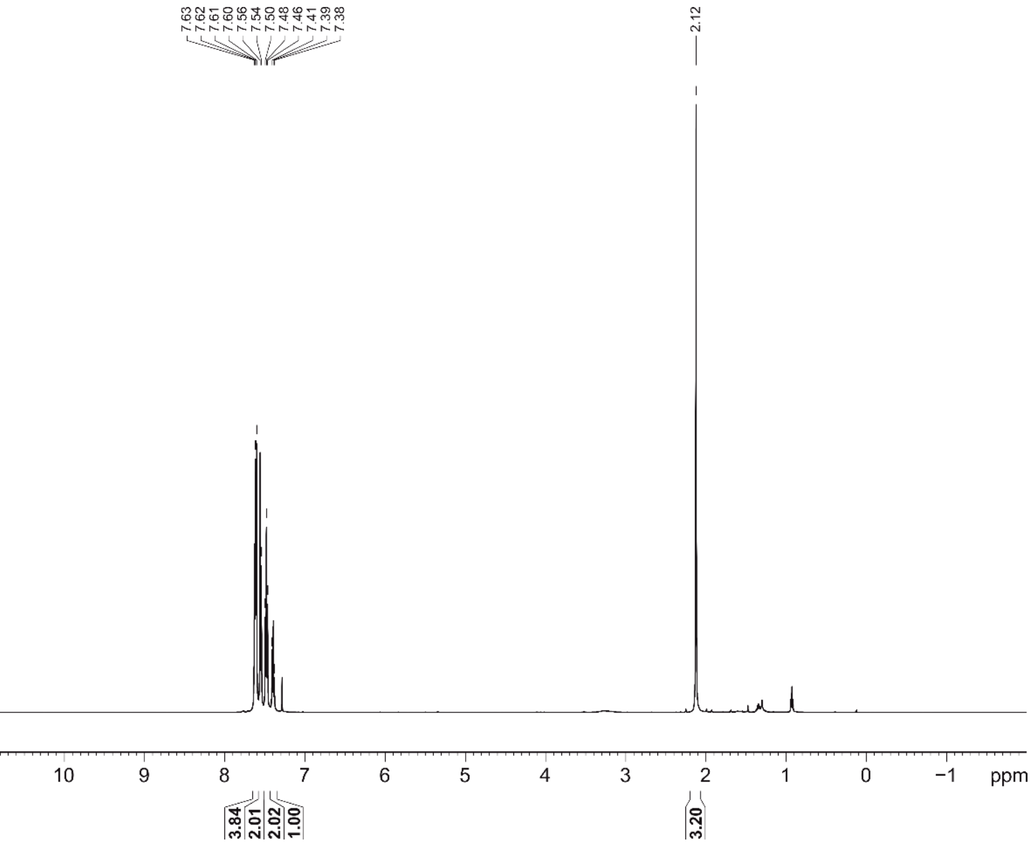

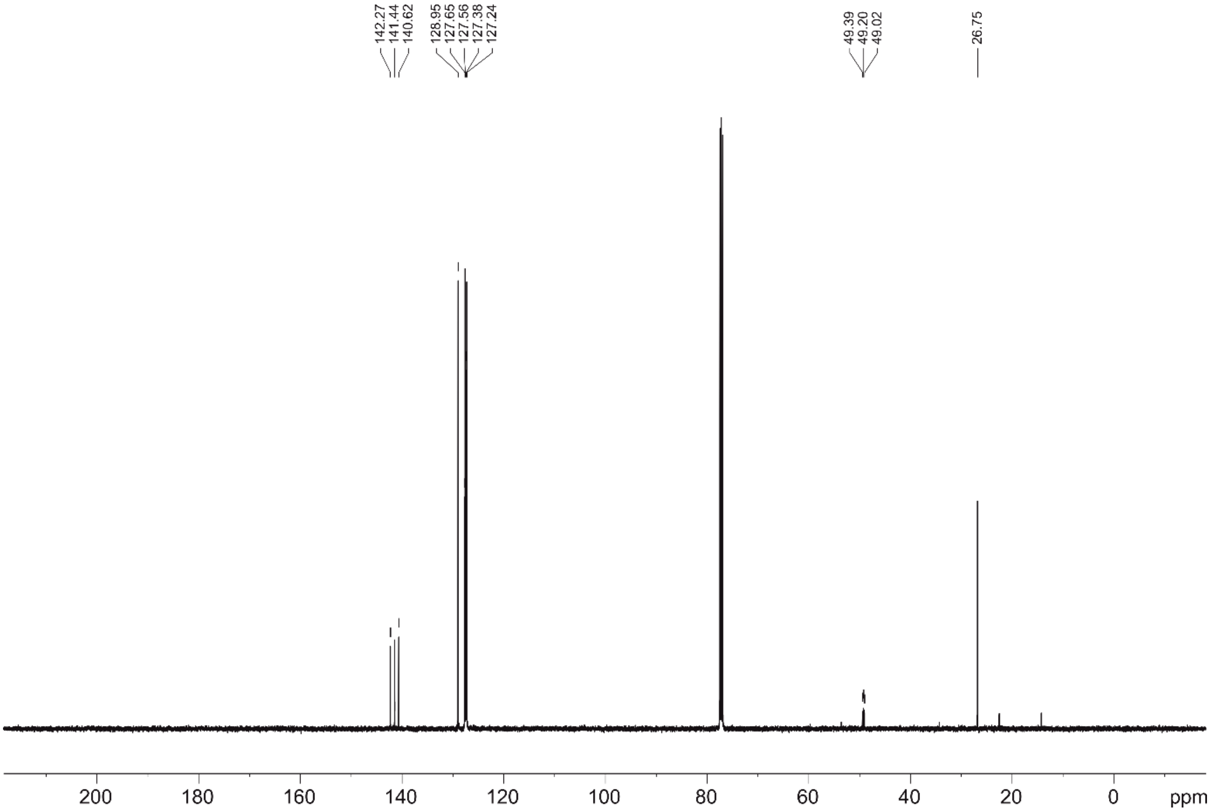


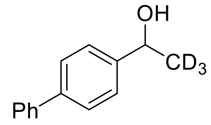


**6a-$d_3$**

**1-([1,1’-biphenyl]-4-yl)ethan-2,2,2-$d_3$-1-ol (6a-$d_3$)**

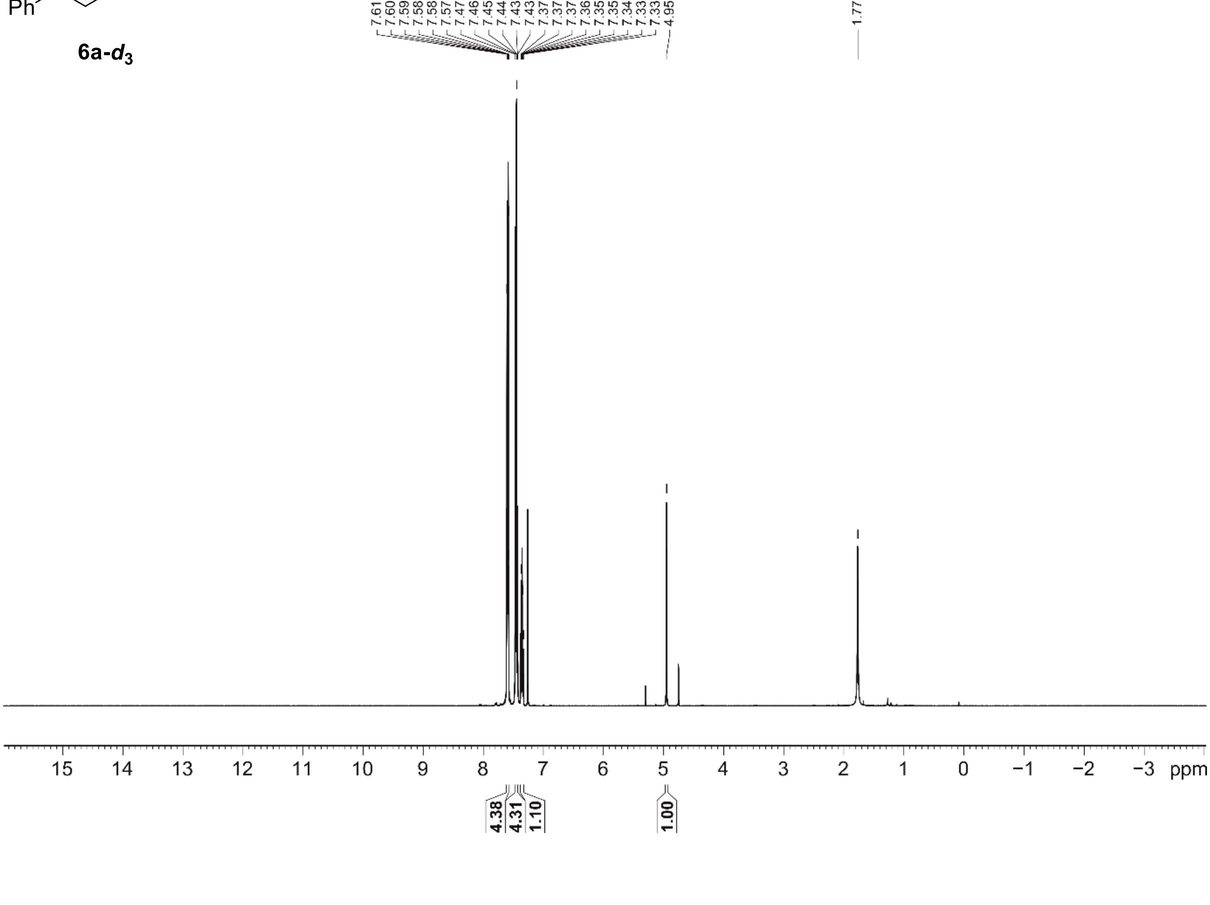

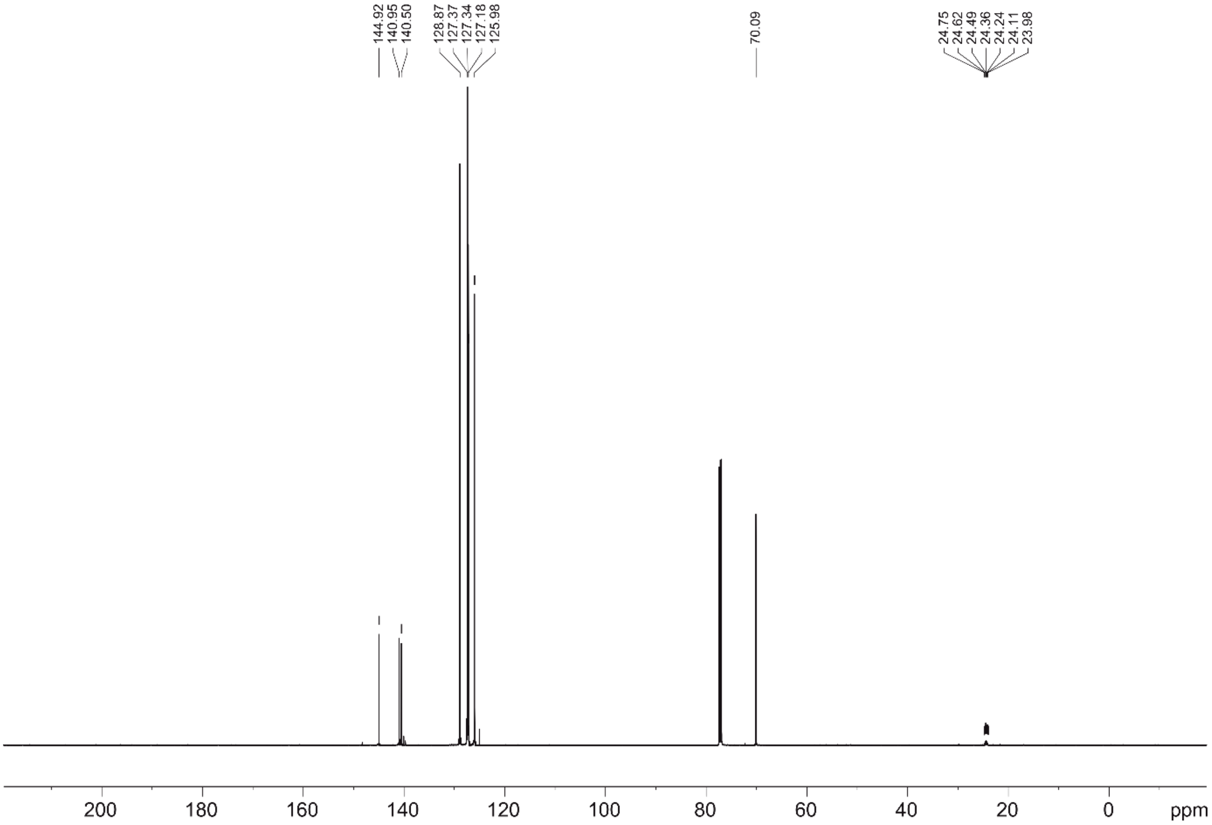


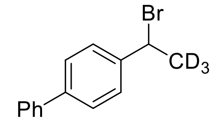


**1a-$d_3$**

## 4-(1-bromoethyl-2,2,2-$d_3$)-1,1′-biphenyl (1a-$d_3$)

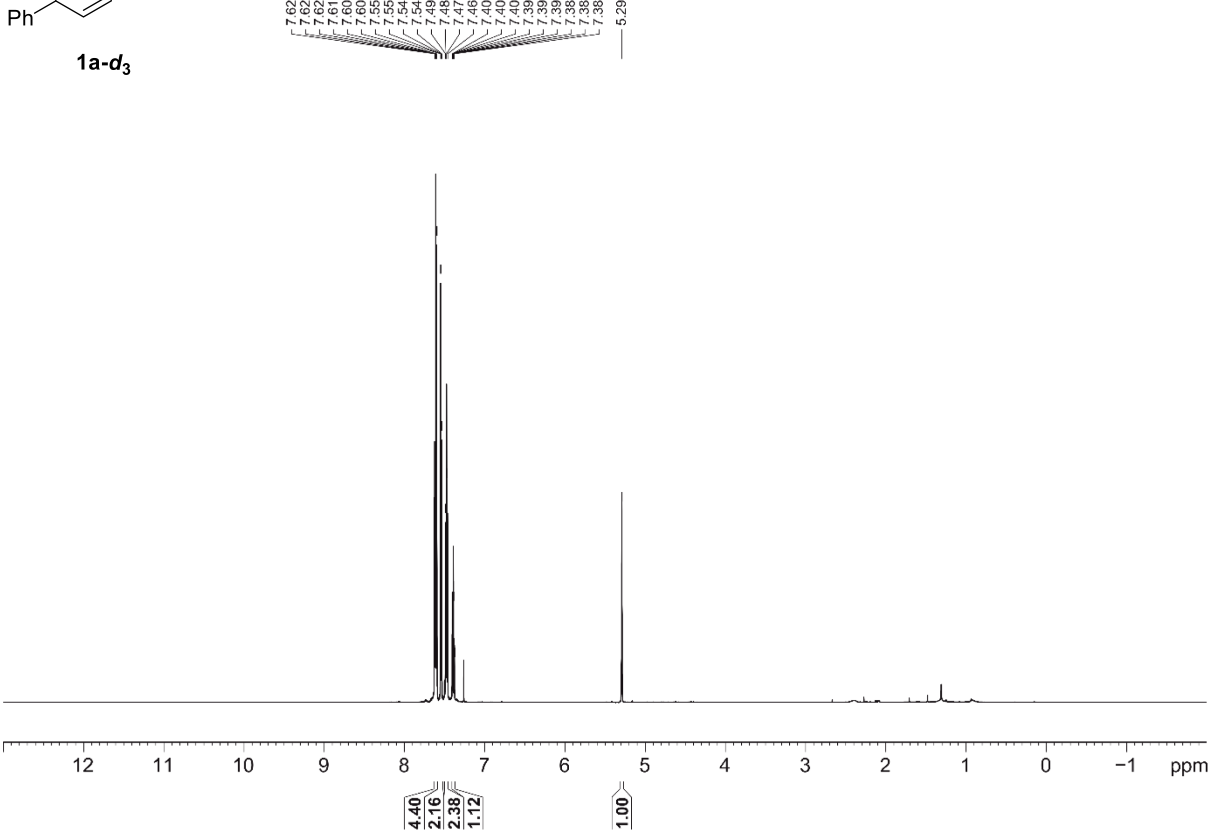

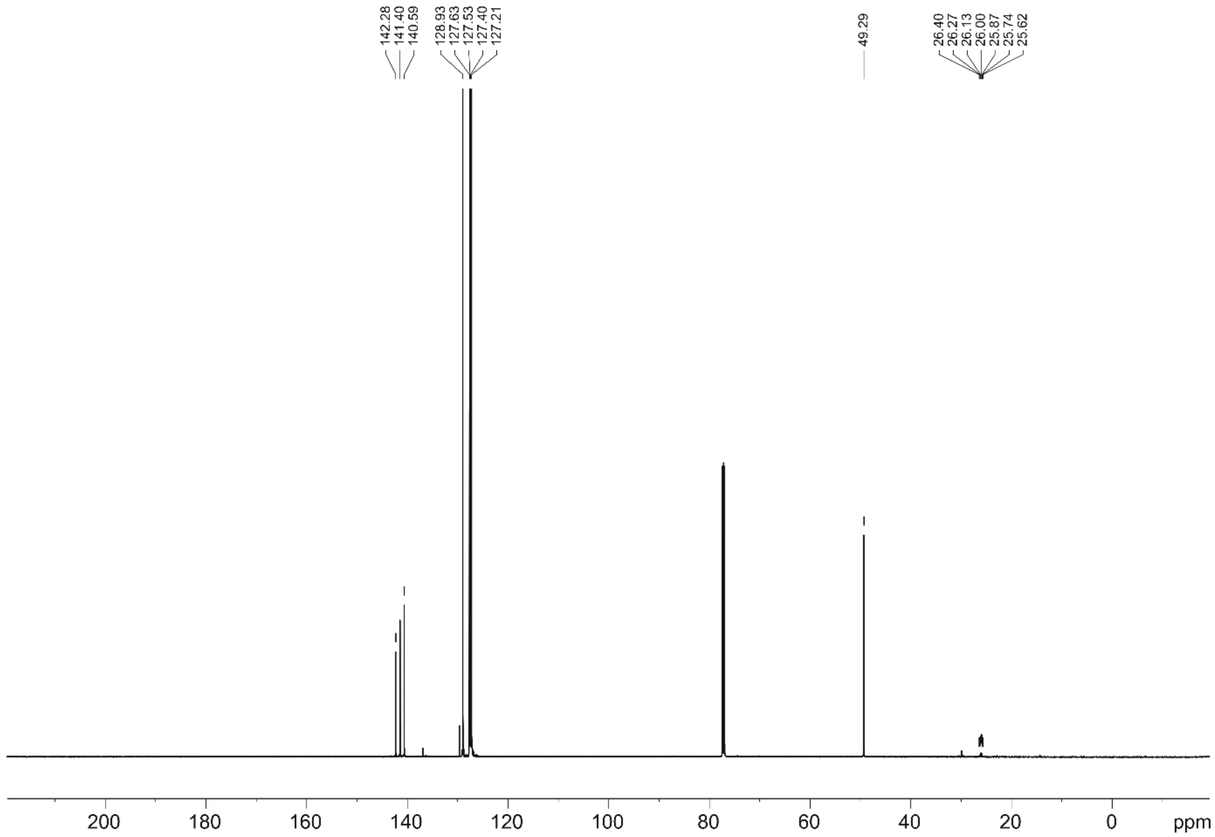


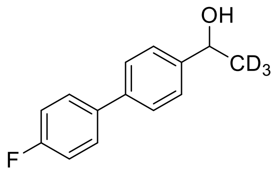


6b-$d_3$

**1-(4'-fluoro-[1,1'-biphenyl]-4-yl)ethan-2,2-2,2-$d_3$-1-ol (6b-$d_3$)**

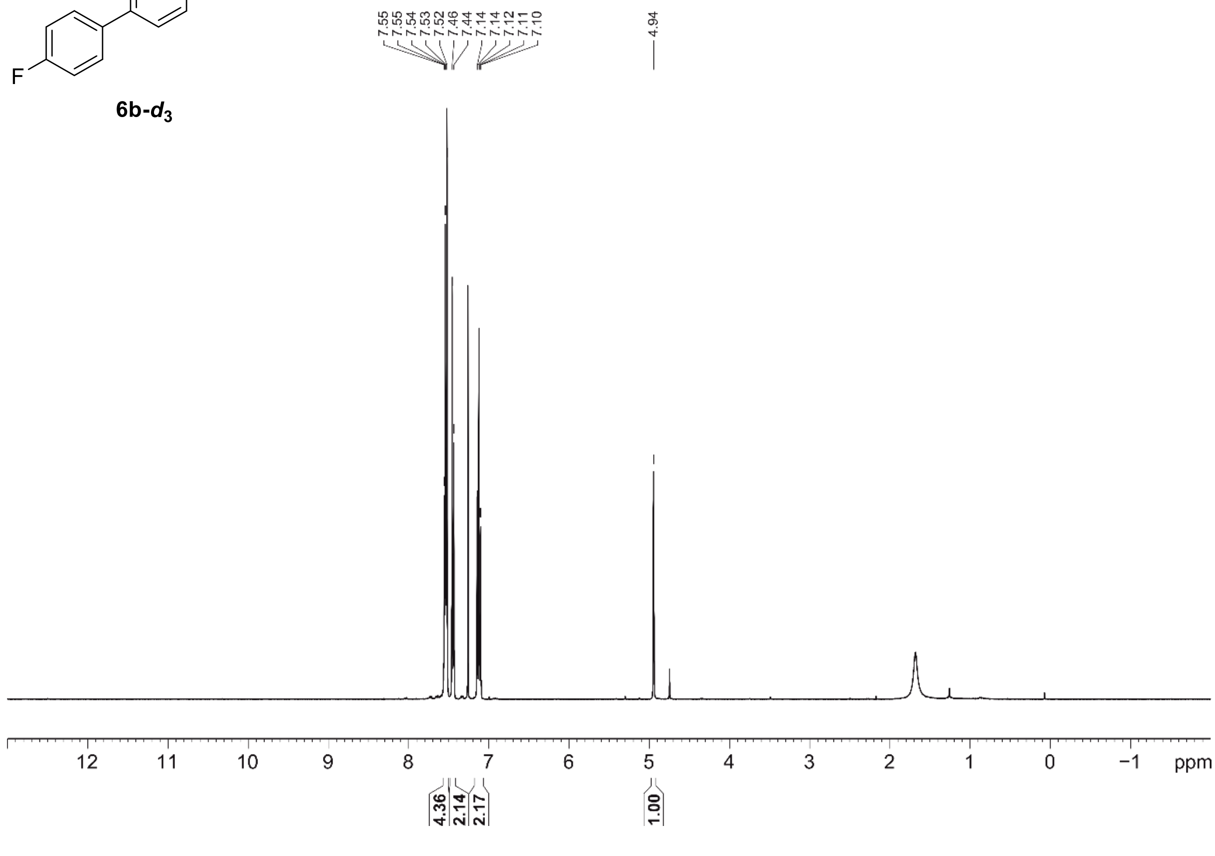

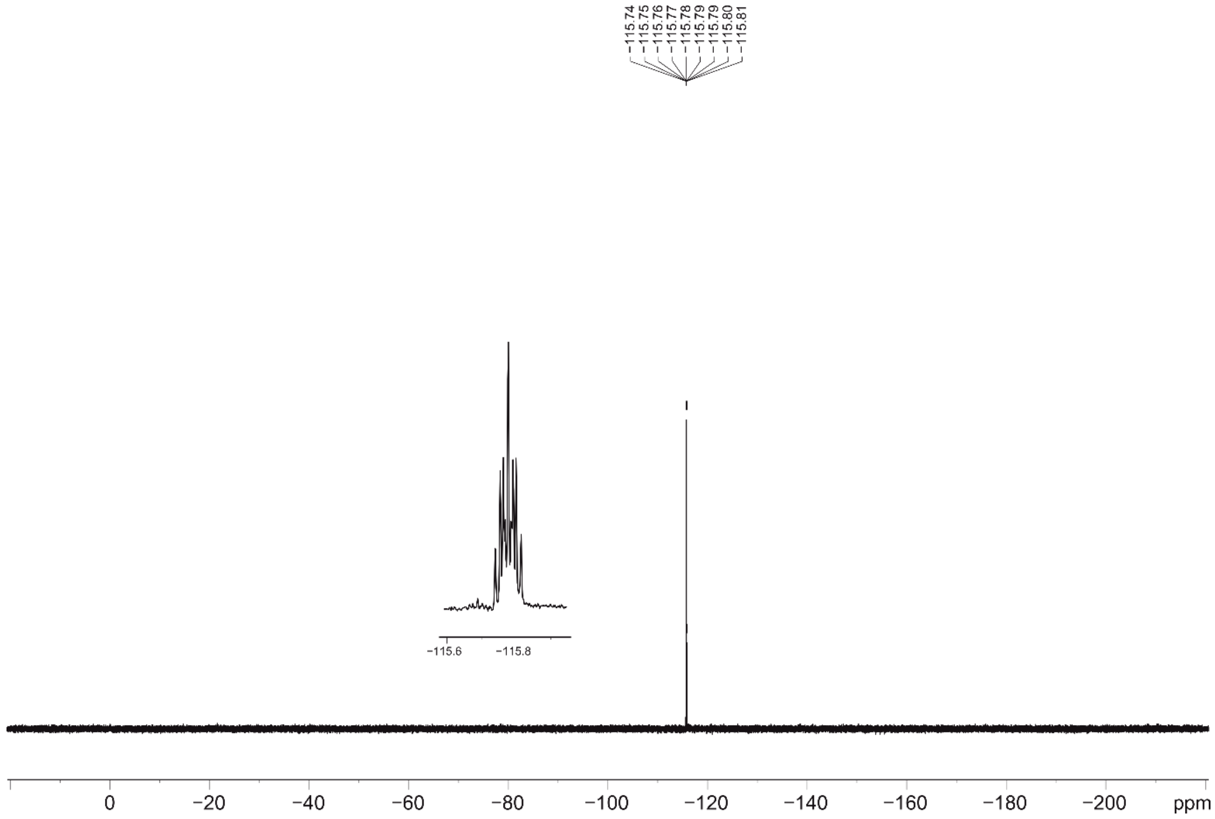

-115.74
-115.75
-115.76
-115.77
-115.78
-115.79
-115.79
-115.80
-115.81
-115.6
-115.8
0
−20
−40
−60
−80
−100
−120
−140
−160
−180
−200
ppm


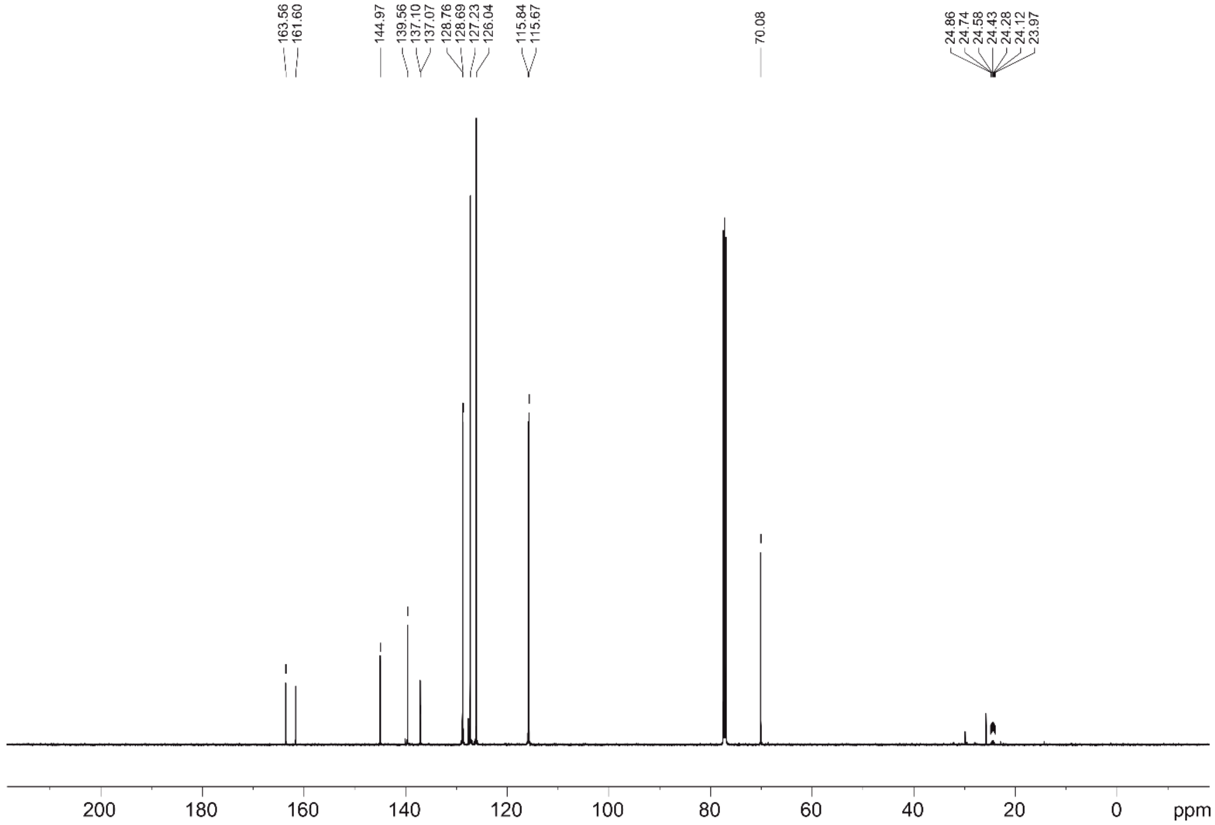

163.56
161.60
144.97
139.56
137.10
137.07
128.76
128.69
127.23
126.04
115.84
115.67
70.08
24.86
24.74
24.58
24.43
24.28
24.12
23.97
200
180
160
140
120
100
80
60
40
20
0
ppm

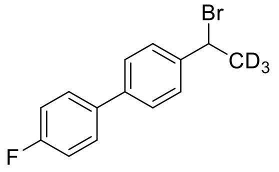


**1b-$d_3$**

**4-(1-bromoethyl-2,2,2-$d_3$)-4'-fluoro-1,1'-biphenyl (1b-$d_3$)**

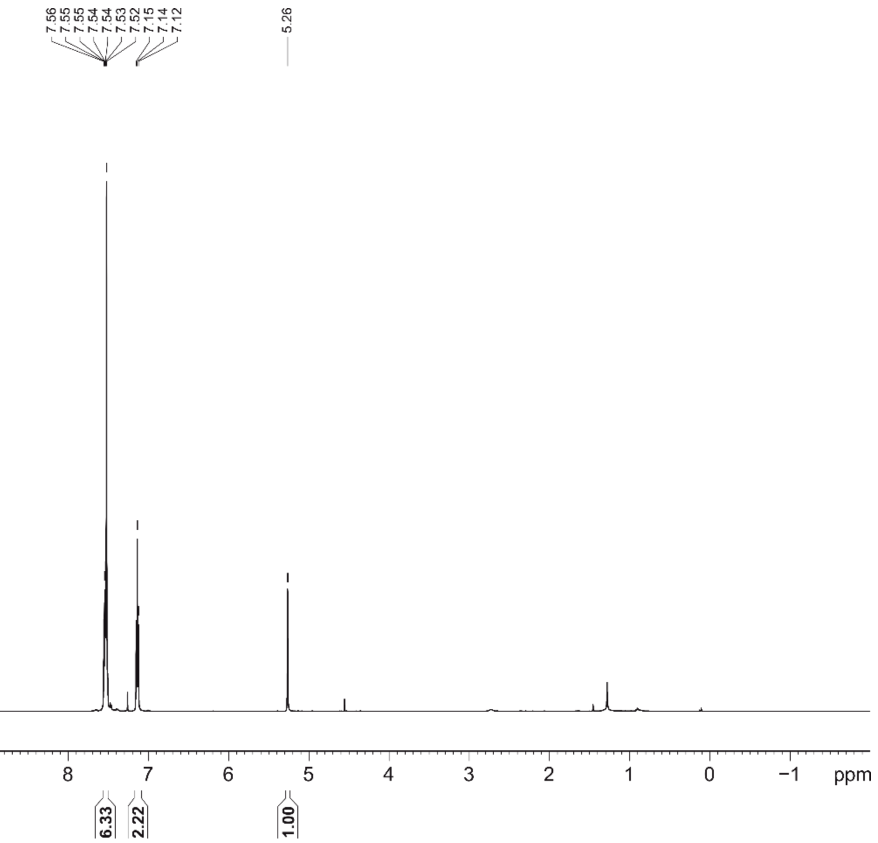


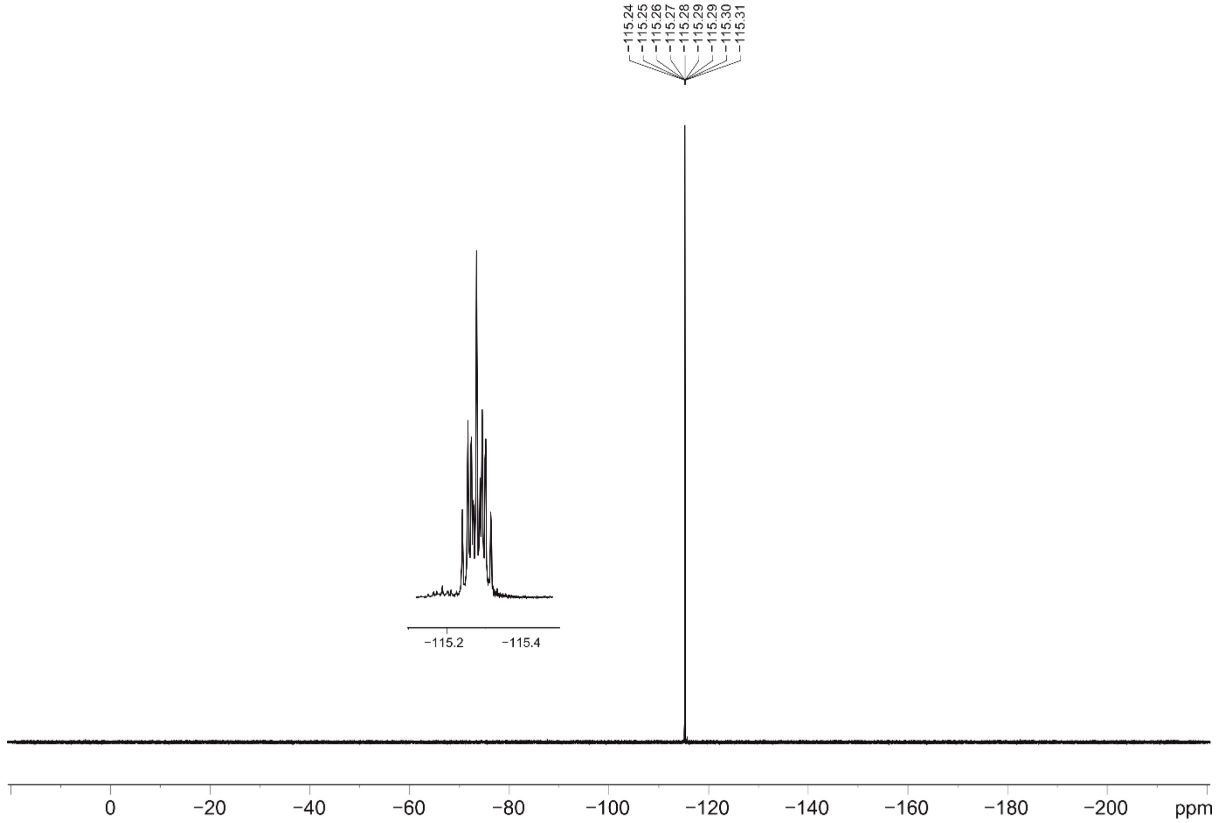

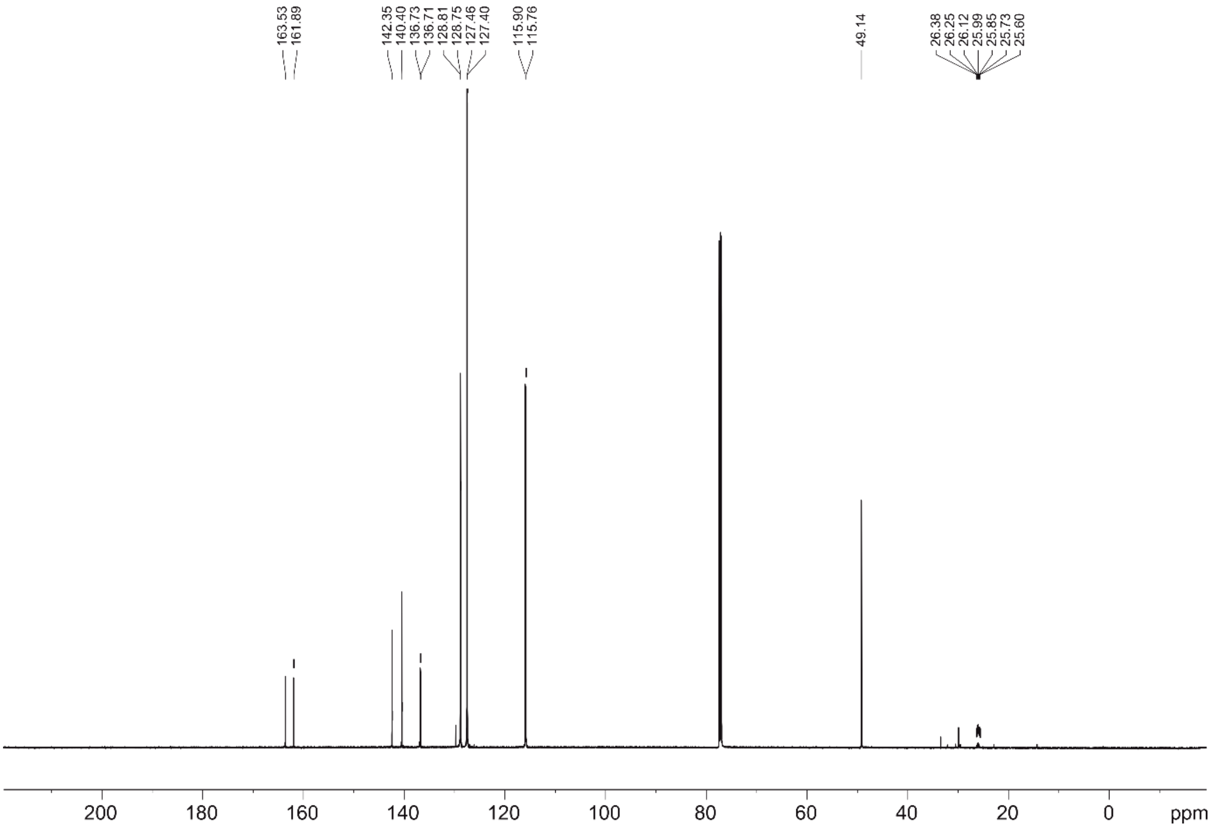


**Chiral Catalysts and precursors**

**(*S*)-N$^2$ -heptan-5-yl-[1,1'-binaphthalene]-2,2'-diamine (7c)**

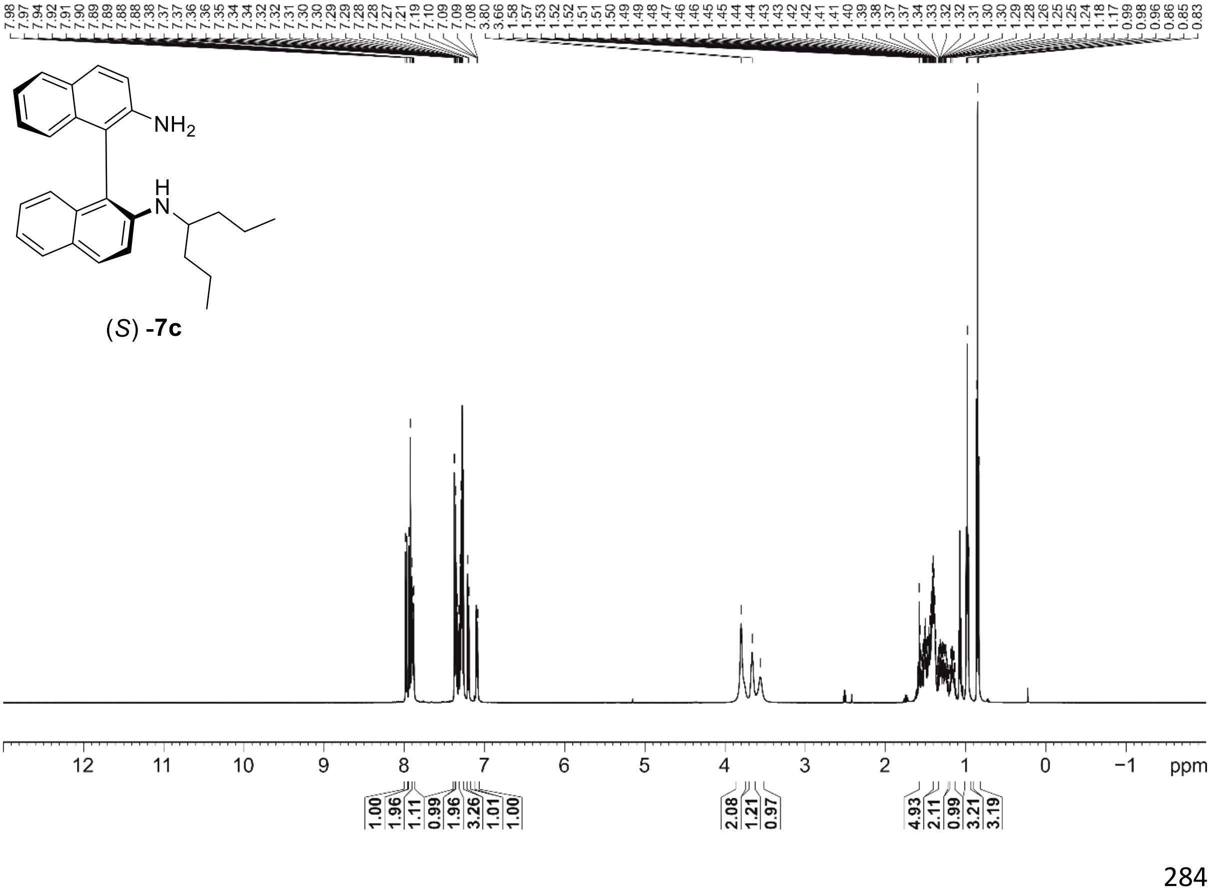

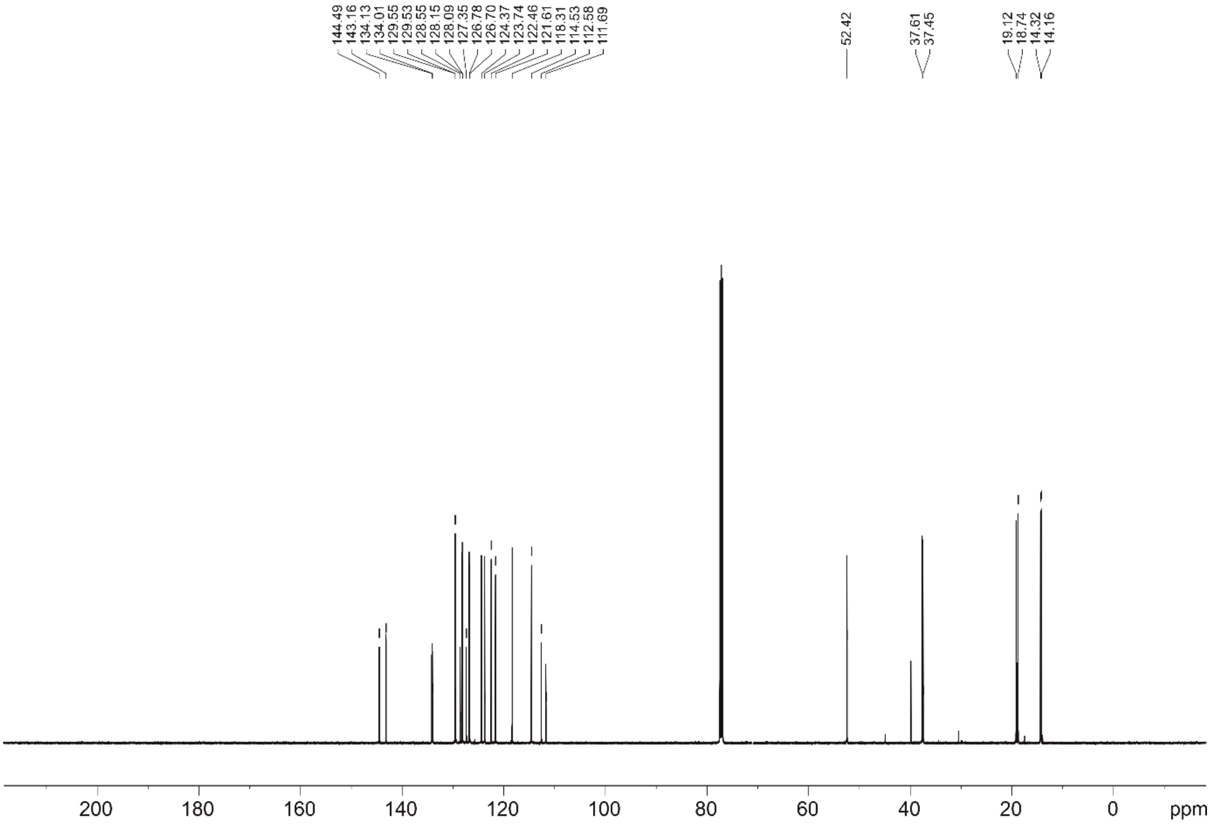


**(*S*)-N[2] -nonan-7-yl-[1,1'-binaphthalene]-2,2'-diamine (7d)**

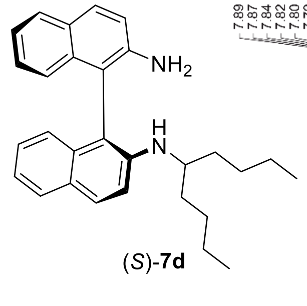


(*S*)-**7d**

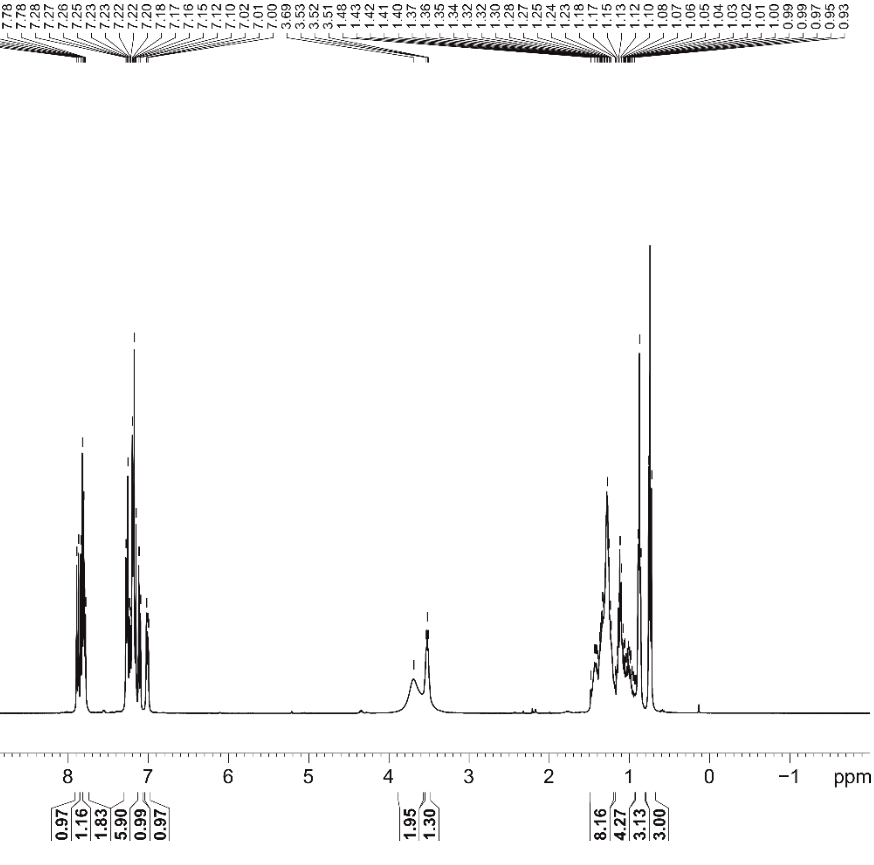

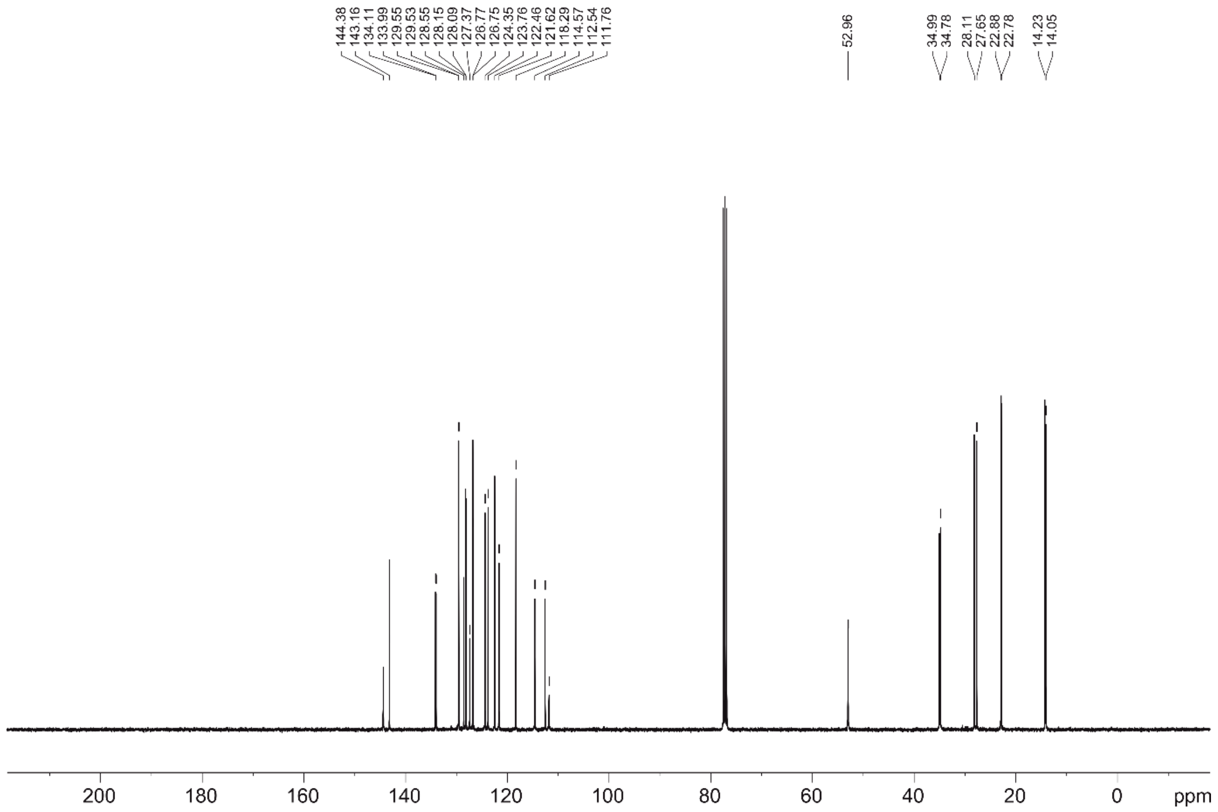


**(*S*)-N$^2$ -cyclohexyl-[1,1'-binaphthalene]-2,2'-diamine (7e)**

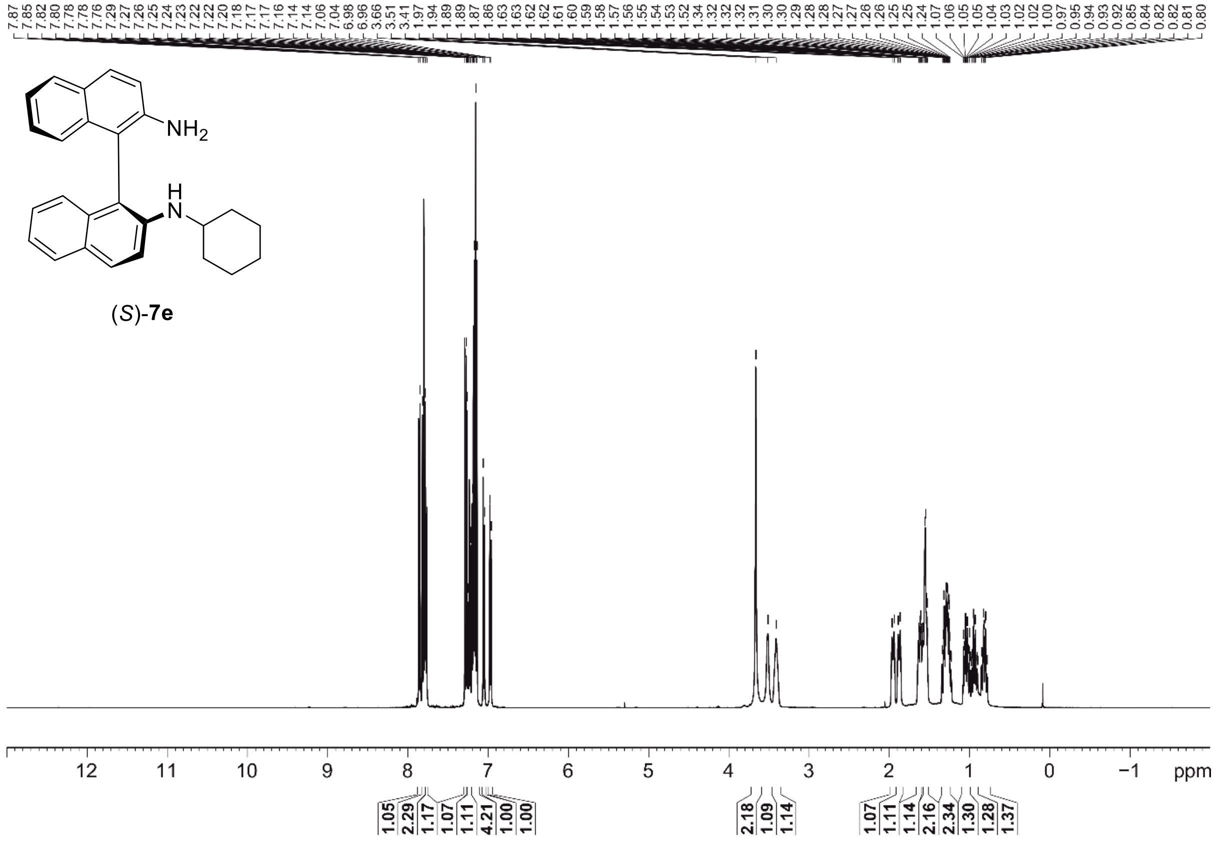

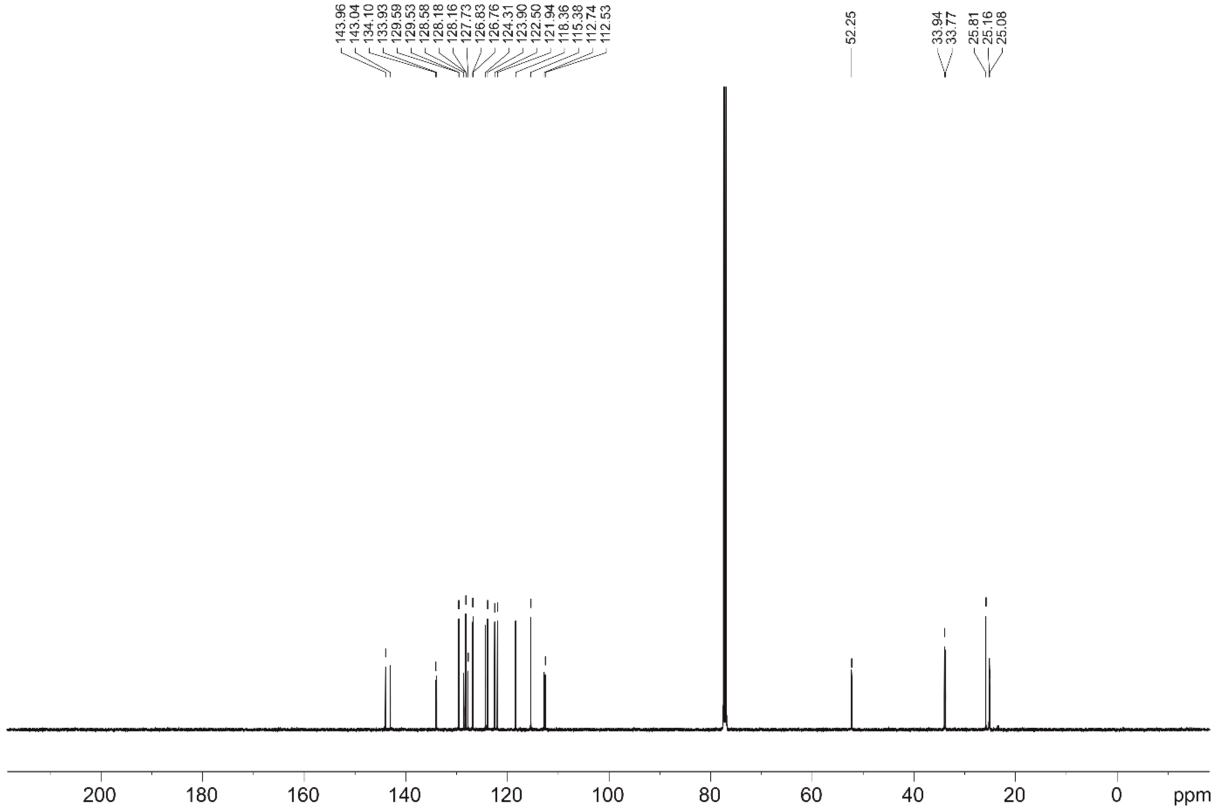

**(*S*)-1-(3,4-difluoromethylphenyl)-3-(2'-(pentylamino)-[1,1'-binaphthalen]-2-yl)urea (8g)**

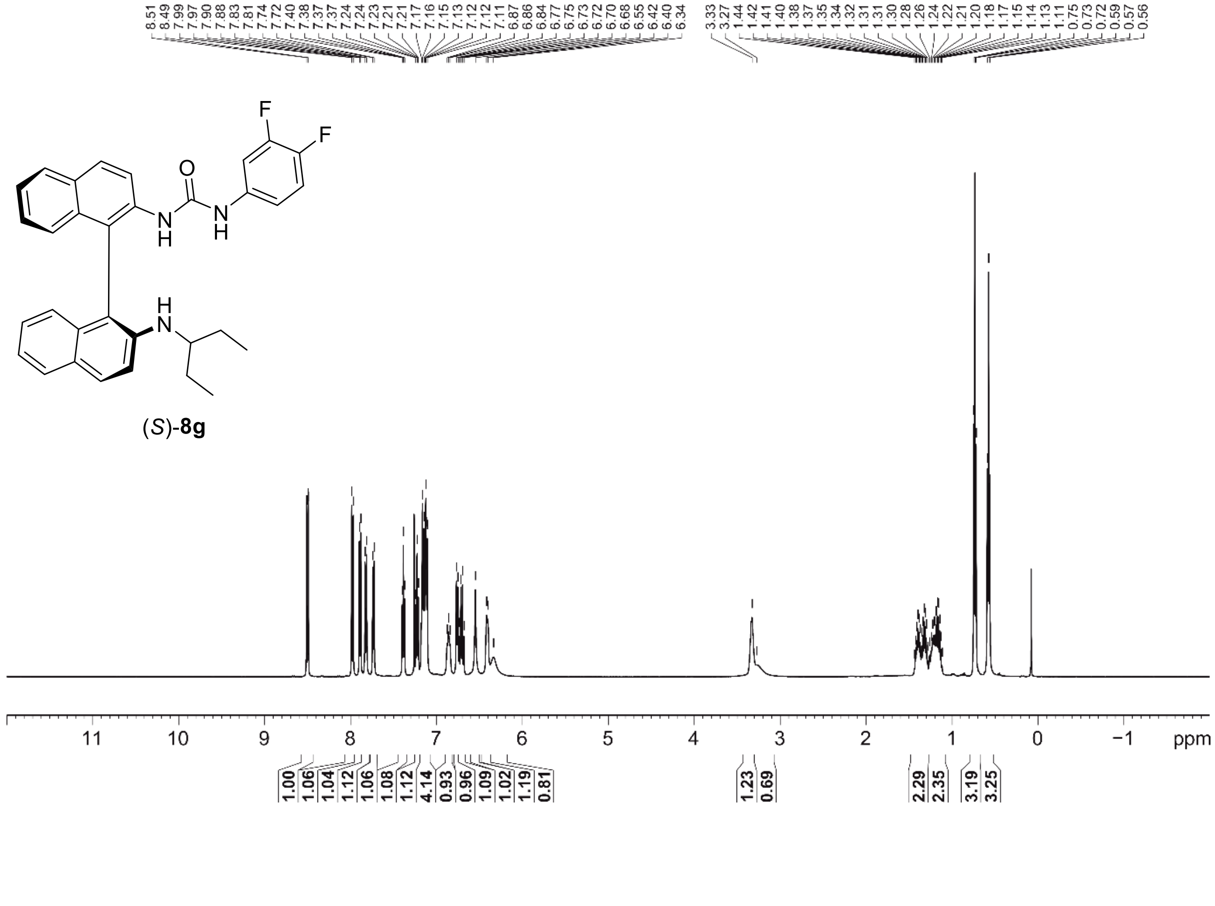

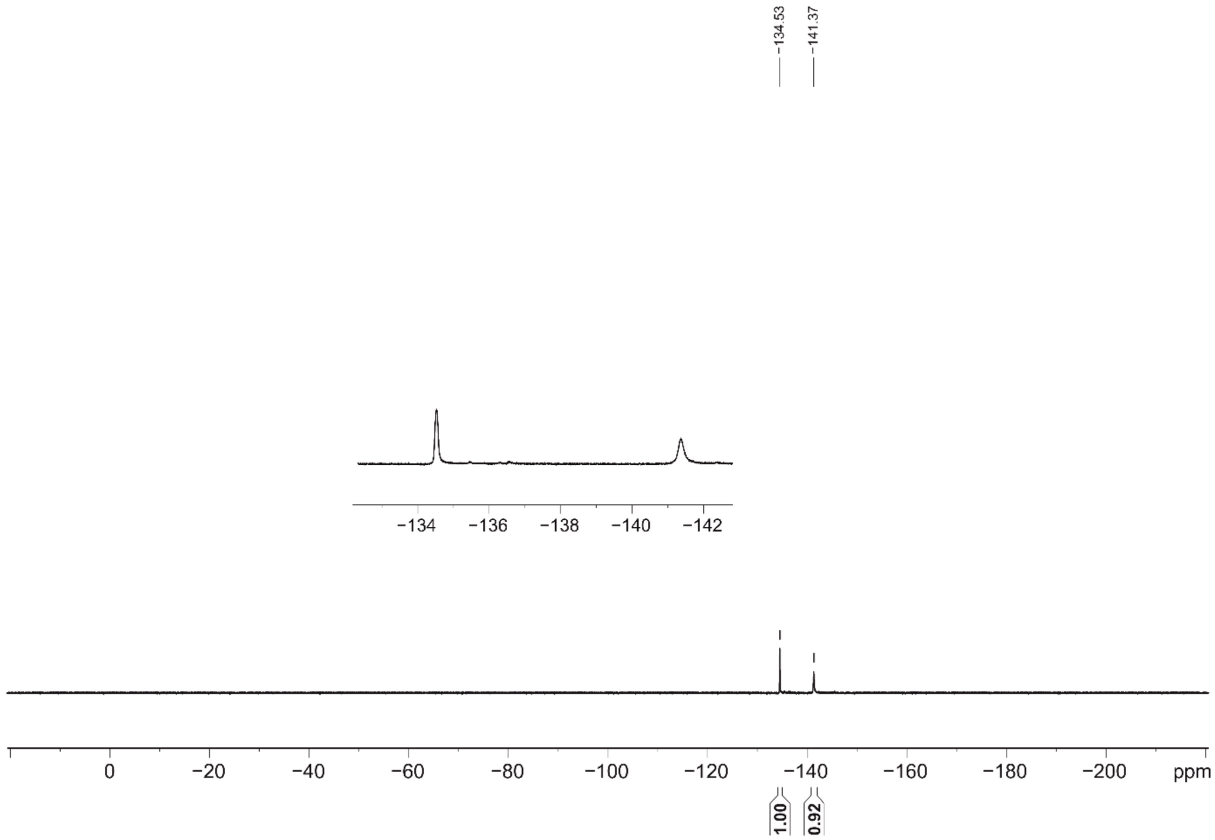

−134.53
−141.37
−134 −136 −138 −140 −142
0 −20 −40 −60 −80 −100 −120 −140 −160 −180 −200 ppm
1.00
0.92


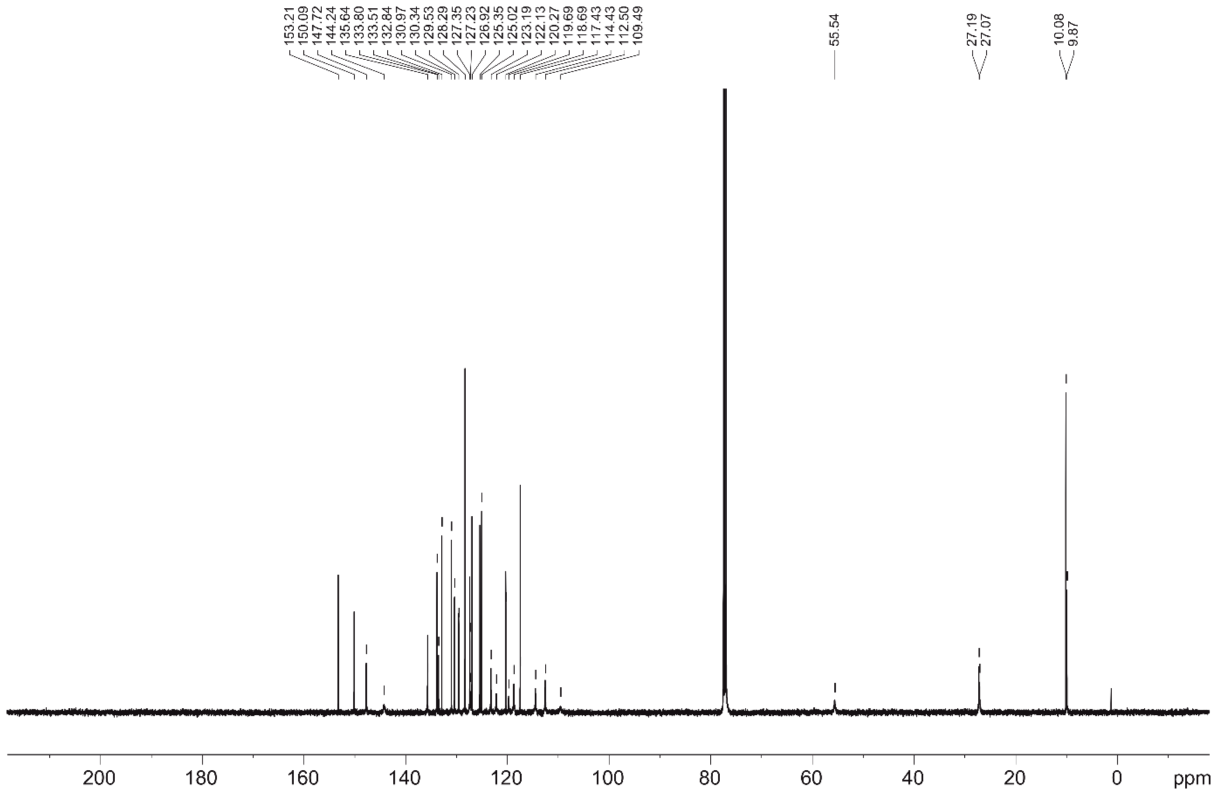

153.21
150.09
147.72
144.24
135.64
133.80
133.51
132.84
130.97
130.34
129.53
128.29
127.35
127.23
126.92
125.35
125.02
123.19
122.13
120.27
119.69
118.69
117.43
114.43
112.50
109.49
55.54
27.19
27.07
10.08
9.87
200 180 160 140 120 100 80 60 40 20 0 ppm

## (*S*)-1-(3,4-difluoromethylphenyl)-3-(2'-(heptylamino)-[1,1'-binaphthalen]-2-yl)urea (8h)

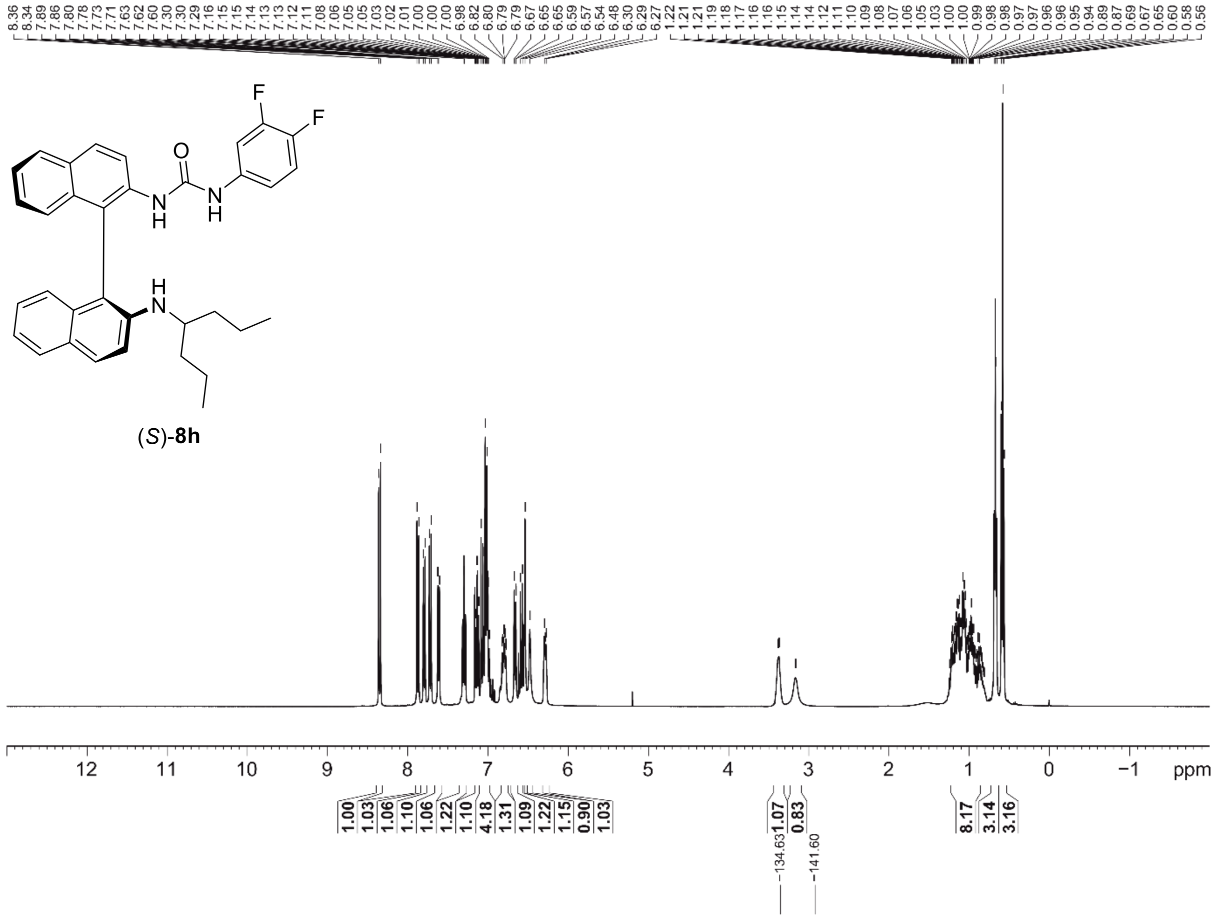


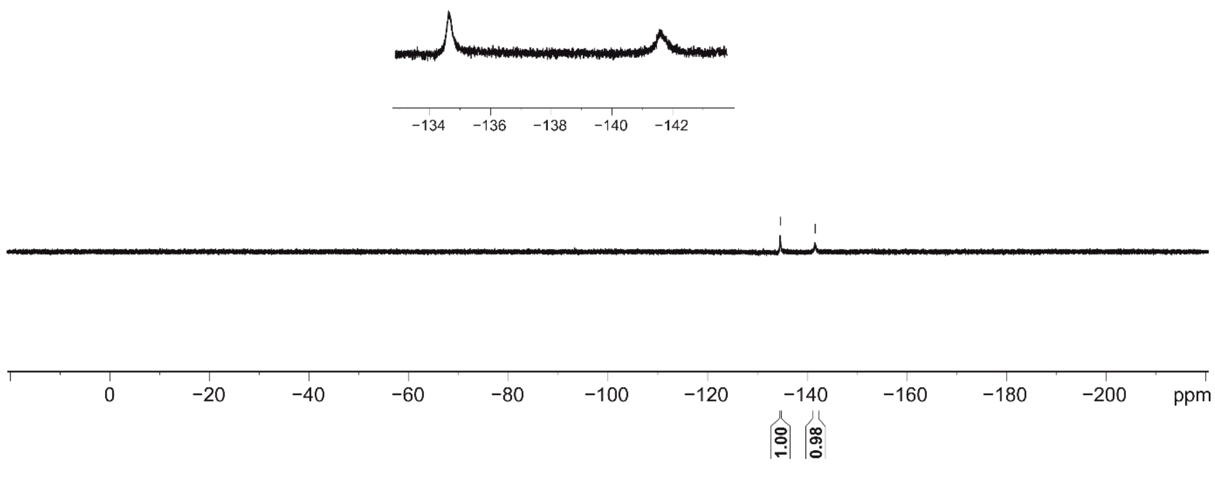

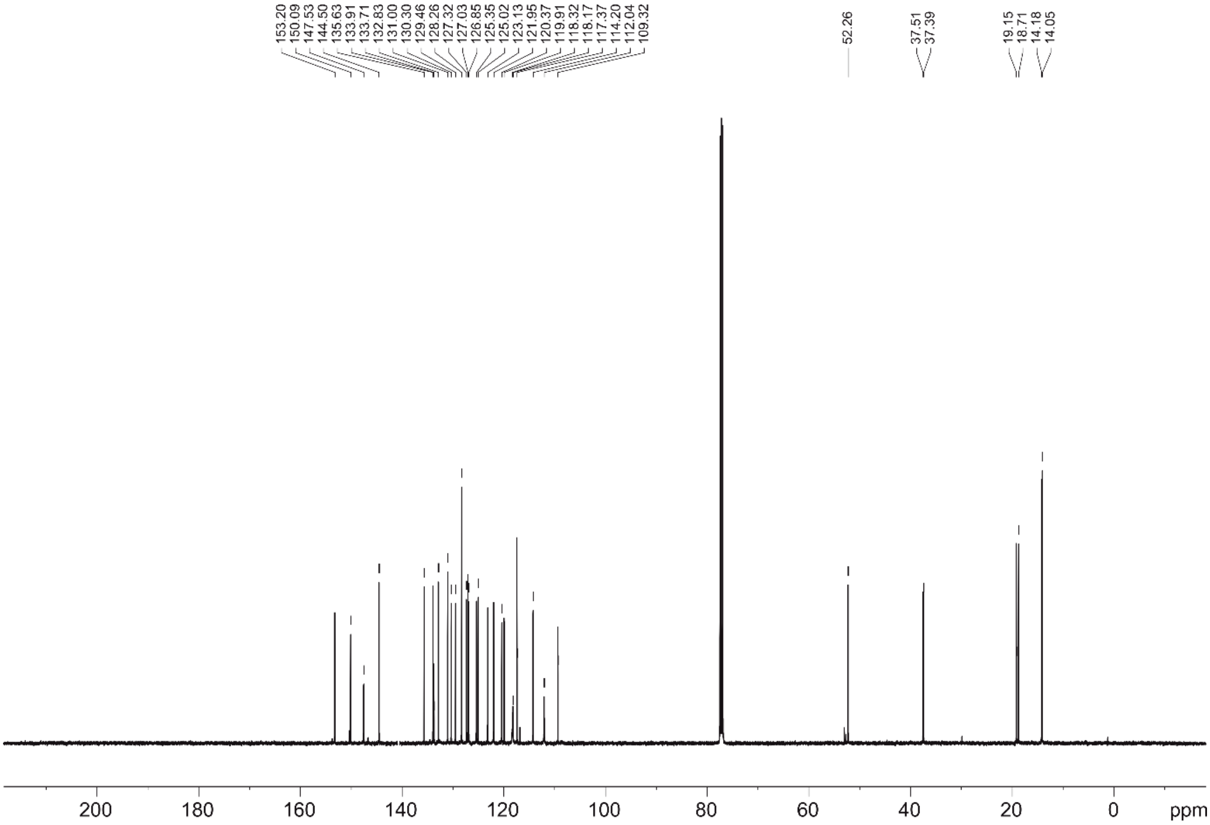


**(*S*)-1-(3,4-difluorophenyl)-3-(2’-(nonan-5-ylamino)-[1,1'-binaphthalen]-2- yl)urea (8i)**

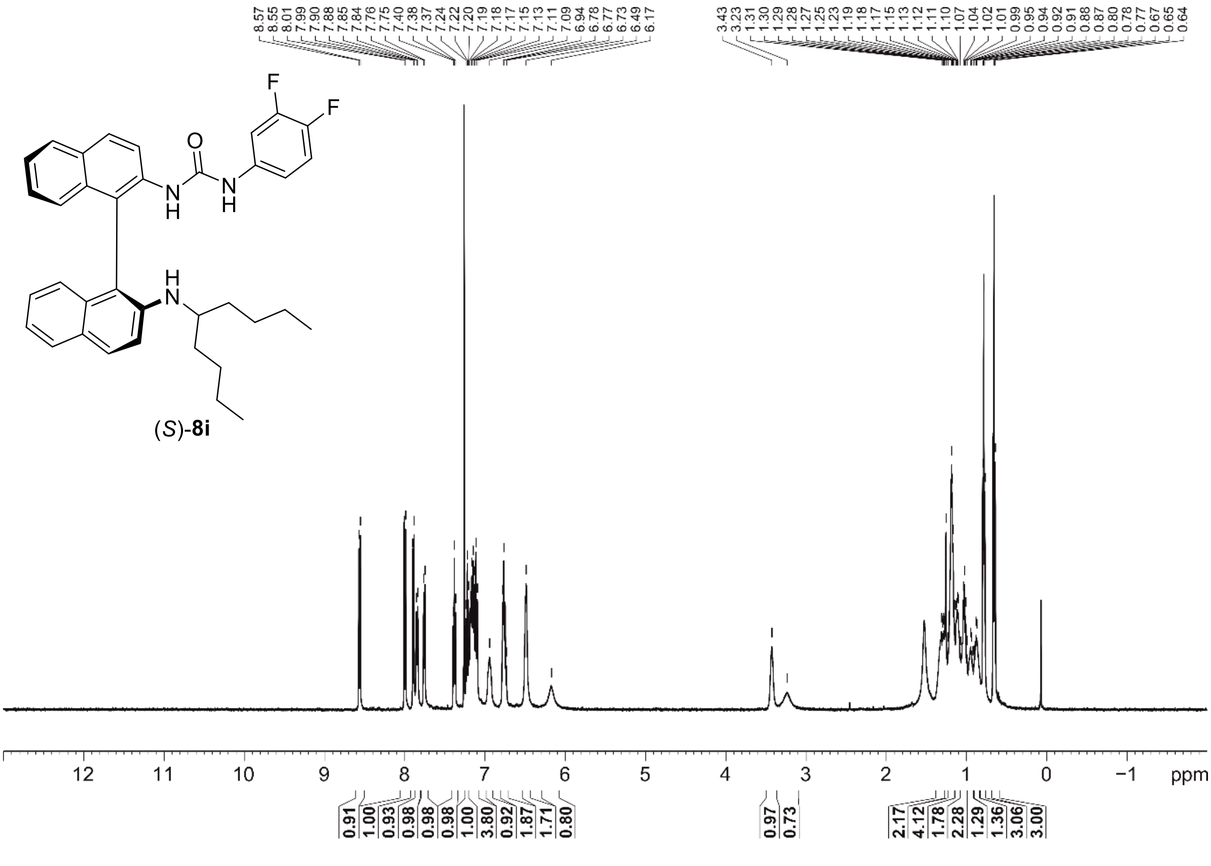

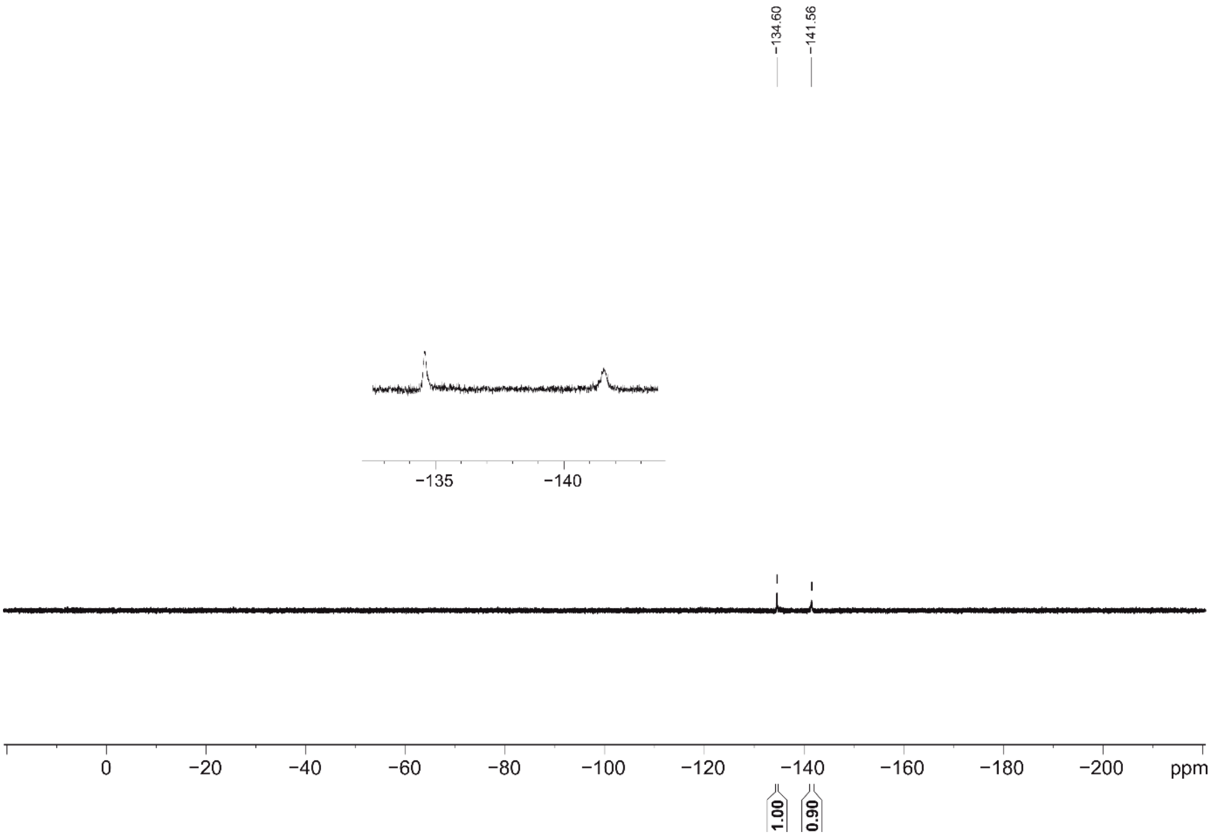
−134.60
−141.56
−135
−140
0
−20
−40
−60
−80
−100
−120
−140
−160
−180
−200
ppm
1.00
0.90

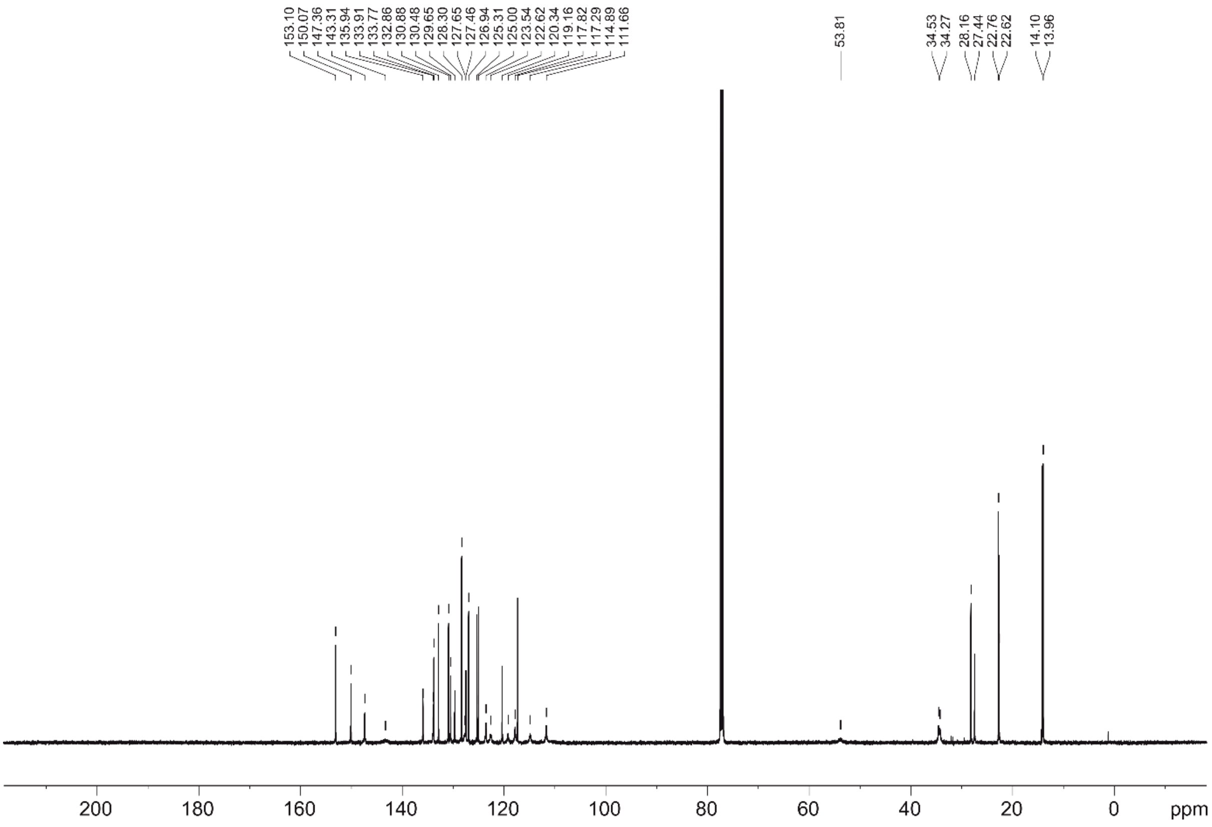
153.10
150.07
147.36
143.31
135.94
133.91
133.77
132.86
130.88
130.48
129.65
128.30
127.65
127.46
126.94
125.31
125.00
123.54
122.62
120.34
119.16
117.82
117.29
114.89
111.66
53.81
34.53
34.27
28.16
27.44
22.76
22.62
14.10
13.96
200
180
160
140
120
100
80
60
40
20
0
ppm

**(*S*)-1-(2’-(cyclohexylamino)-[1,1'-binaphthalen]-2- yl)-3-(3,4-difluoropheyl)urea (8j)**

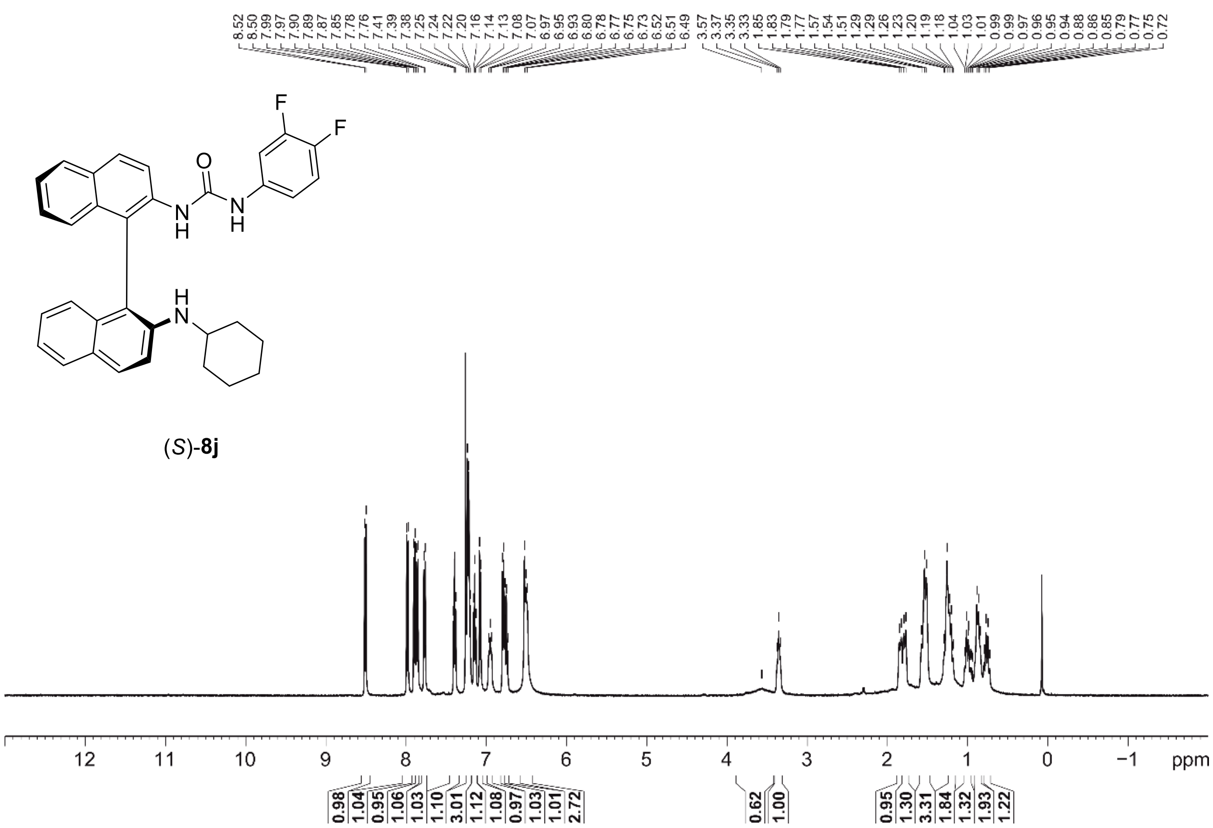

(*S*)-**8j**

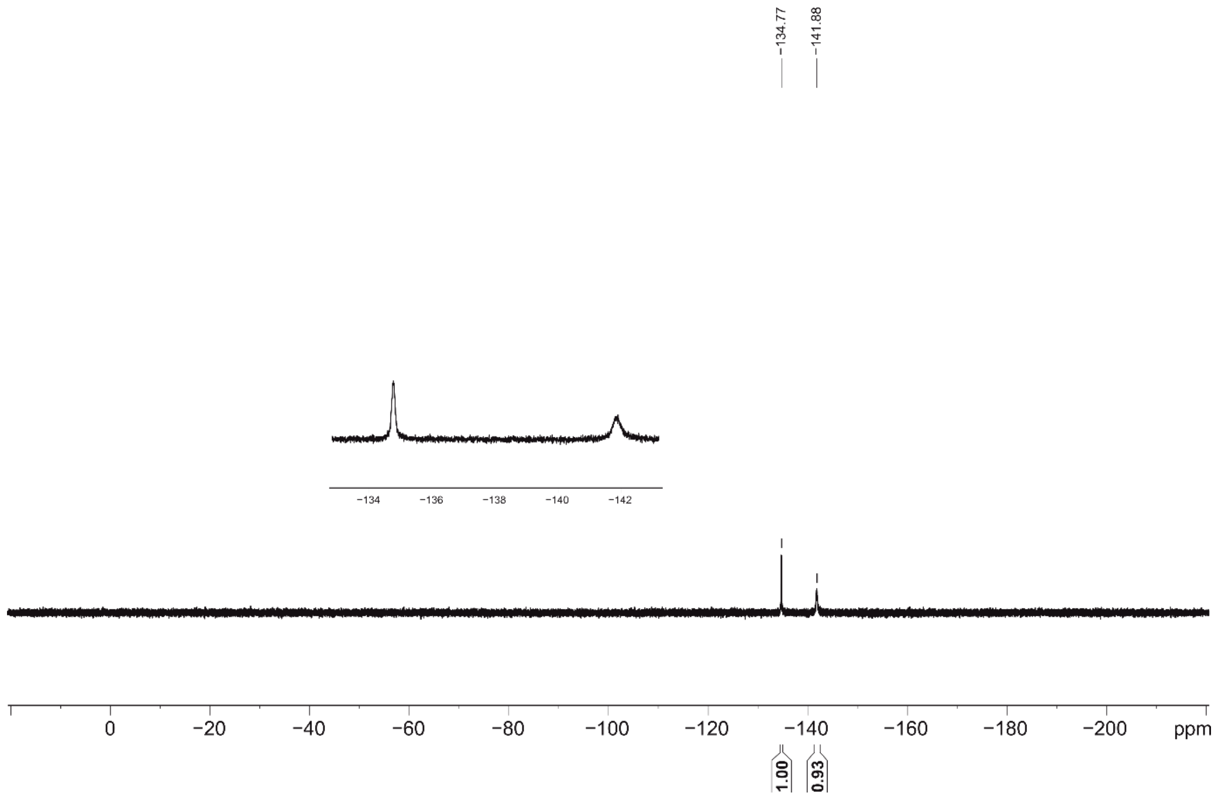

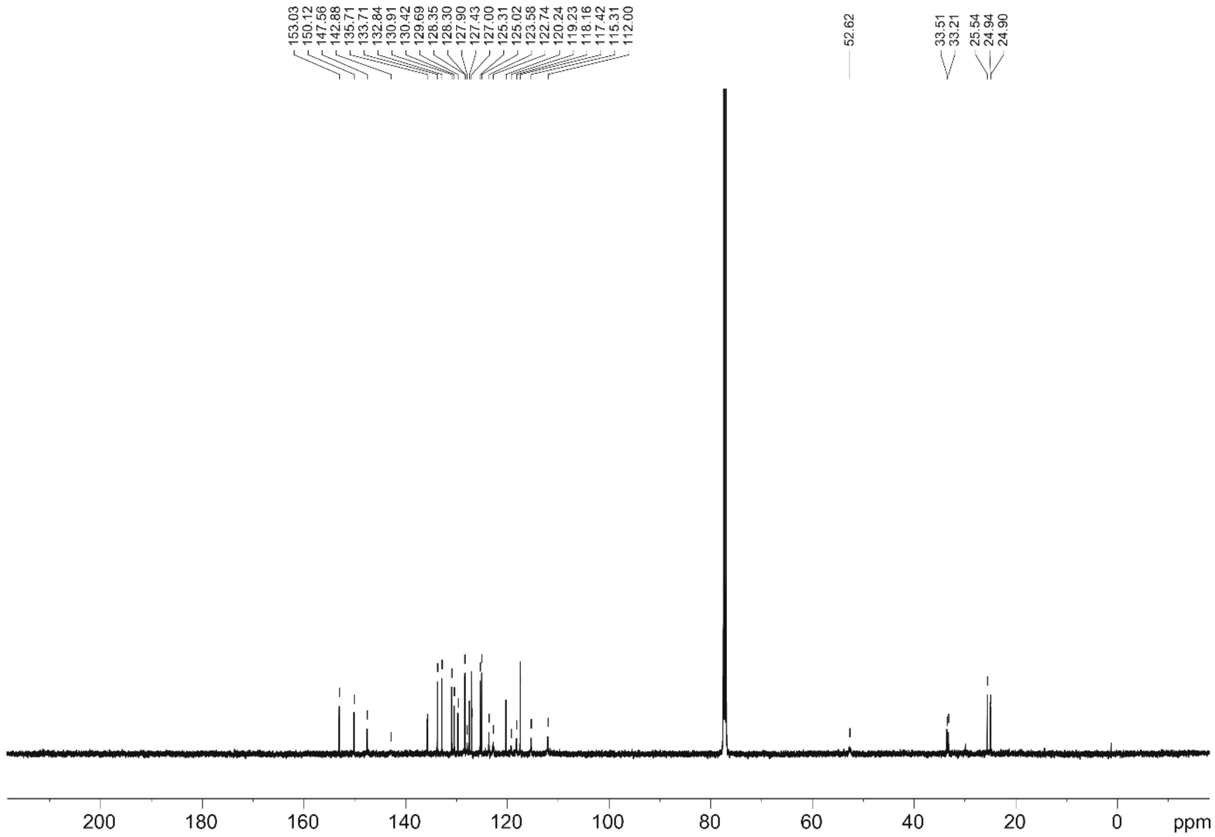


**(*S*)-3-(3,5-bis(trifluoromethyl)phenyl)-1-isopropyl-1-(2'-(3-(4-fluorophenyl)ureido)-[1,1'-binaphthalen]-2- yl)urea (3e)**

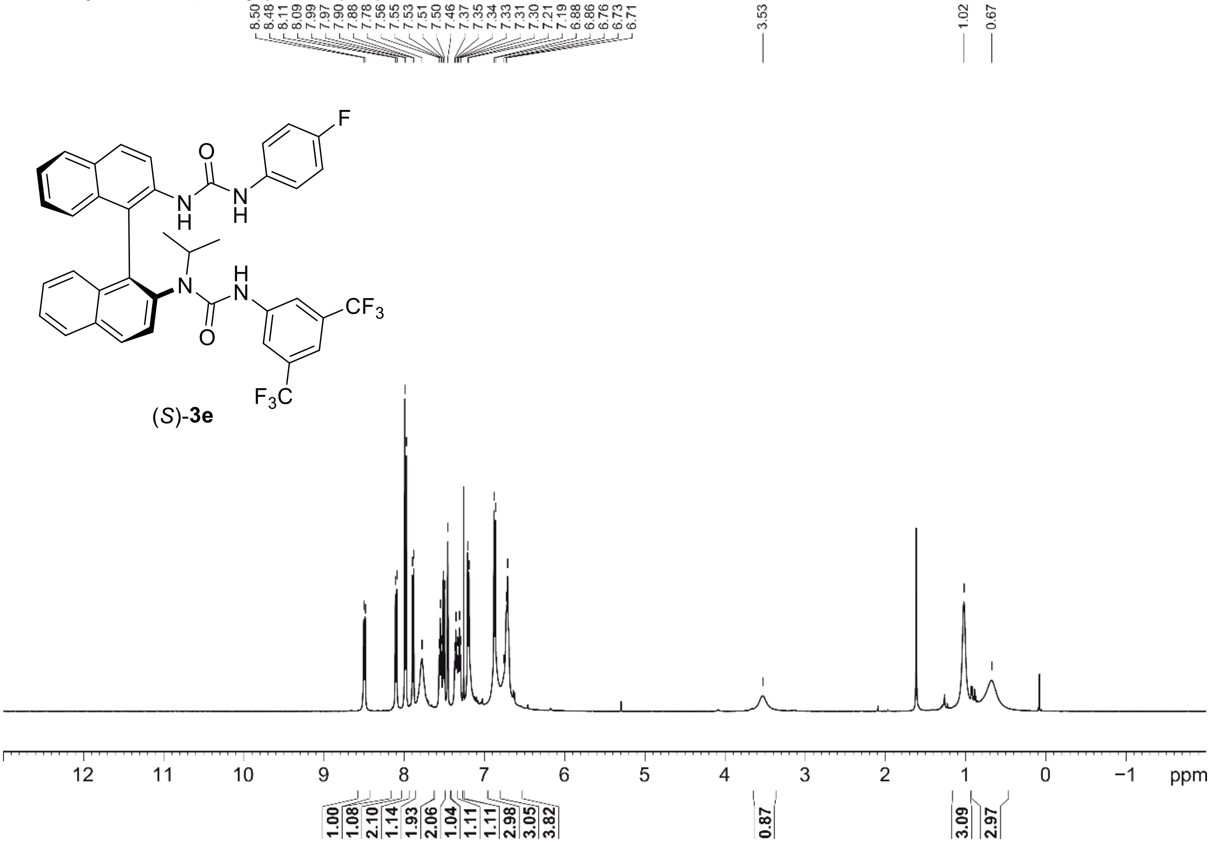


(*S*)-**3e**

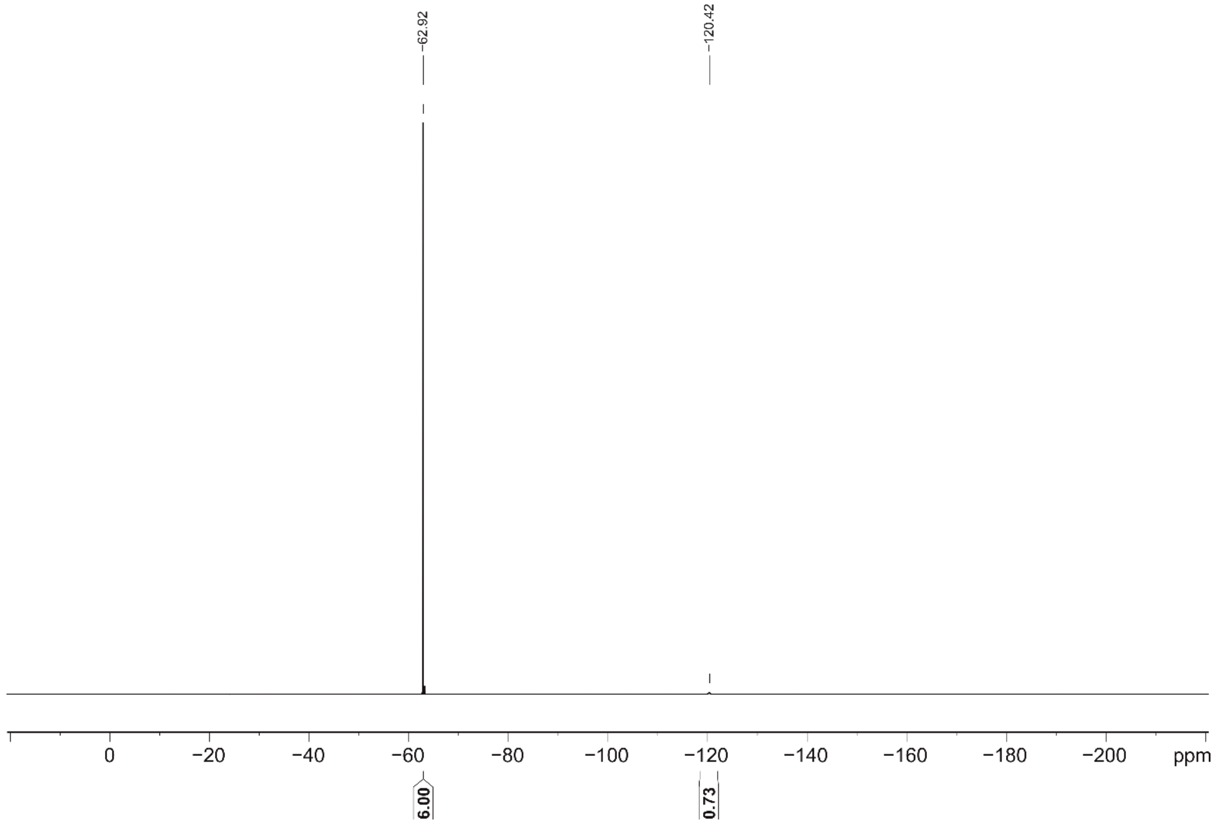
-62.92
-120.42
0
−20
−40
−60
−80
−100
−120
−140
−160
−180
−200
ppm
6.00
0.73

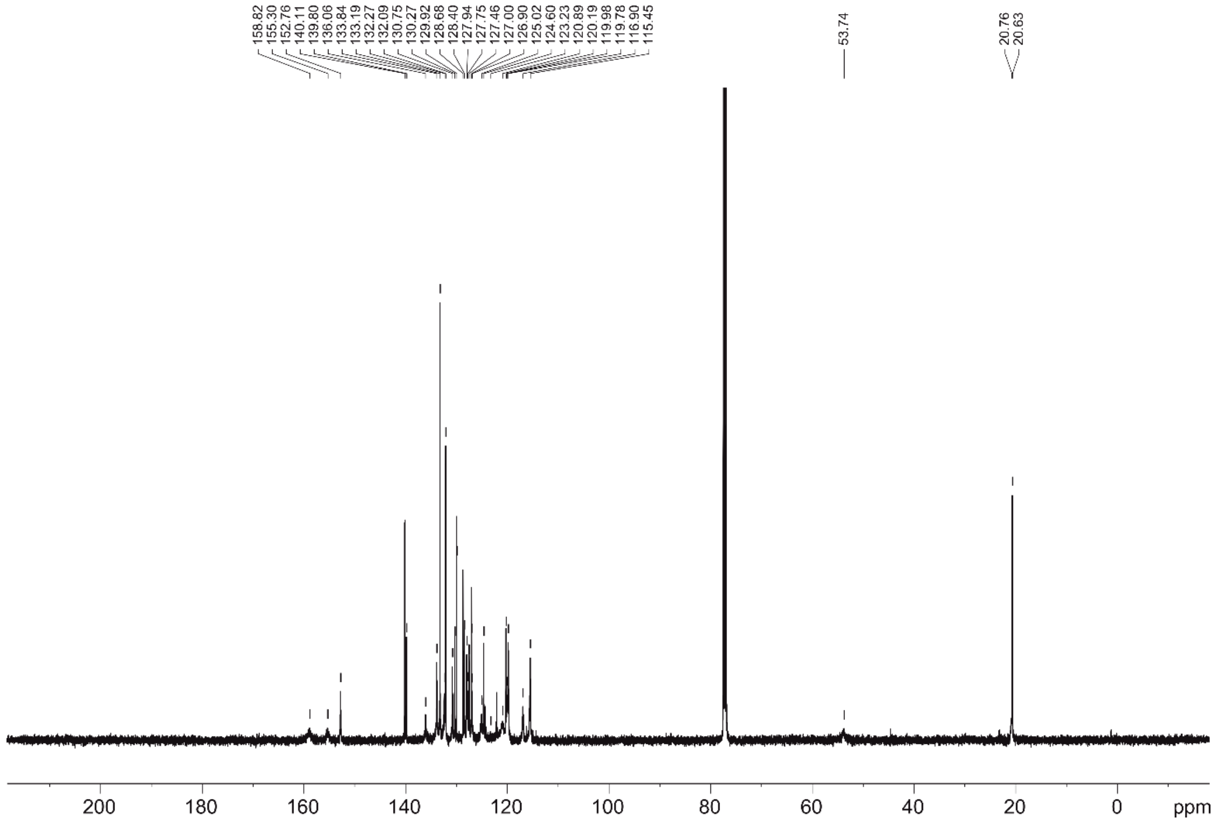
158.82
155.30
152.76
140.11
139.80
136.06
133.84
133.19
132.27
132.09
130.75
130.27
129.92
128.68
128.40
127.94
127.75
127.46
127.00
126.90
125.02
124.60
123.23
120.89
120.19
119.98
119.78
116.90
115.45
53.74
20.76
20.63
200
180
160
140
120
100
80
60
40
20
0
ppm

**(*S*)-3-(3,5-bis(trifluoromethyl)phenyl)-1-isopropyl-1-(2'-(3-(3,4-difluorophenyl)ureido)-[1,1'-binaphthalen]-2- yl)urea (3f)**

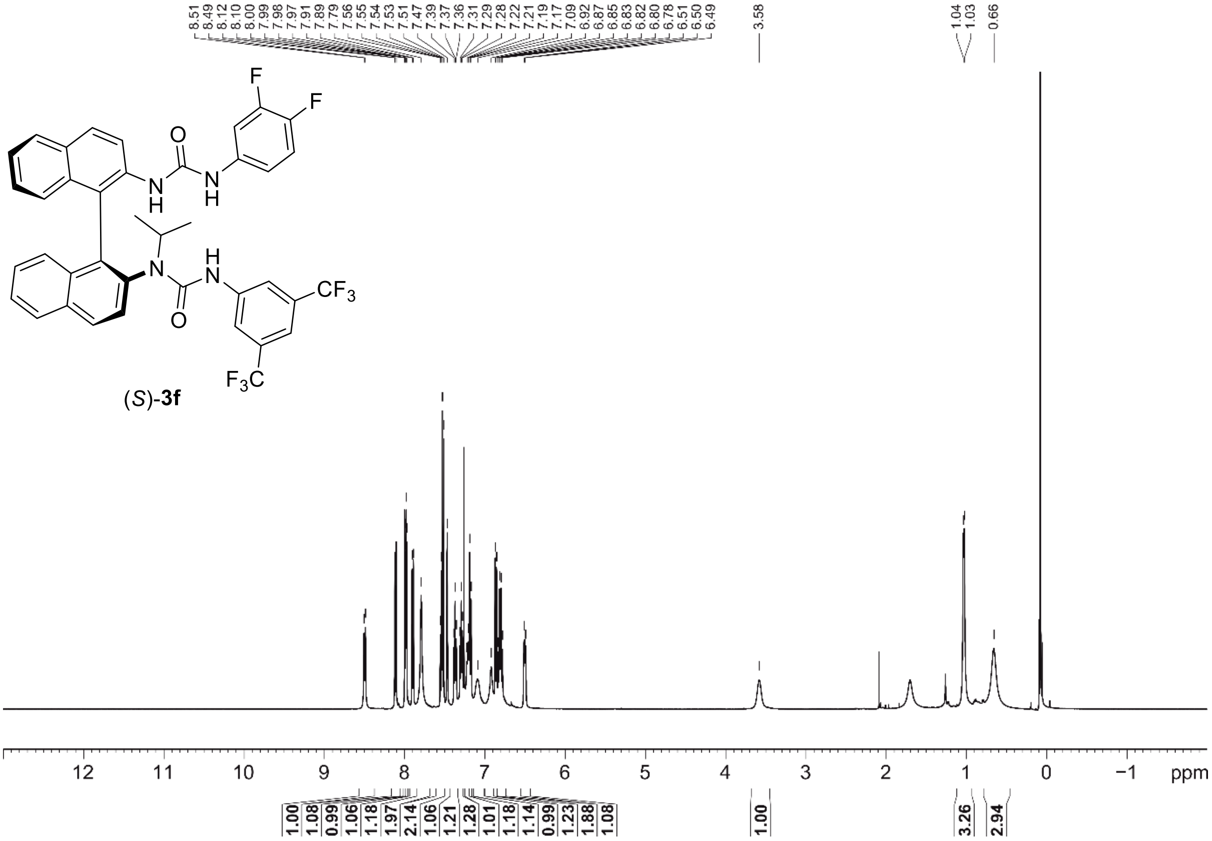


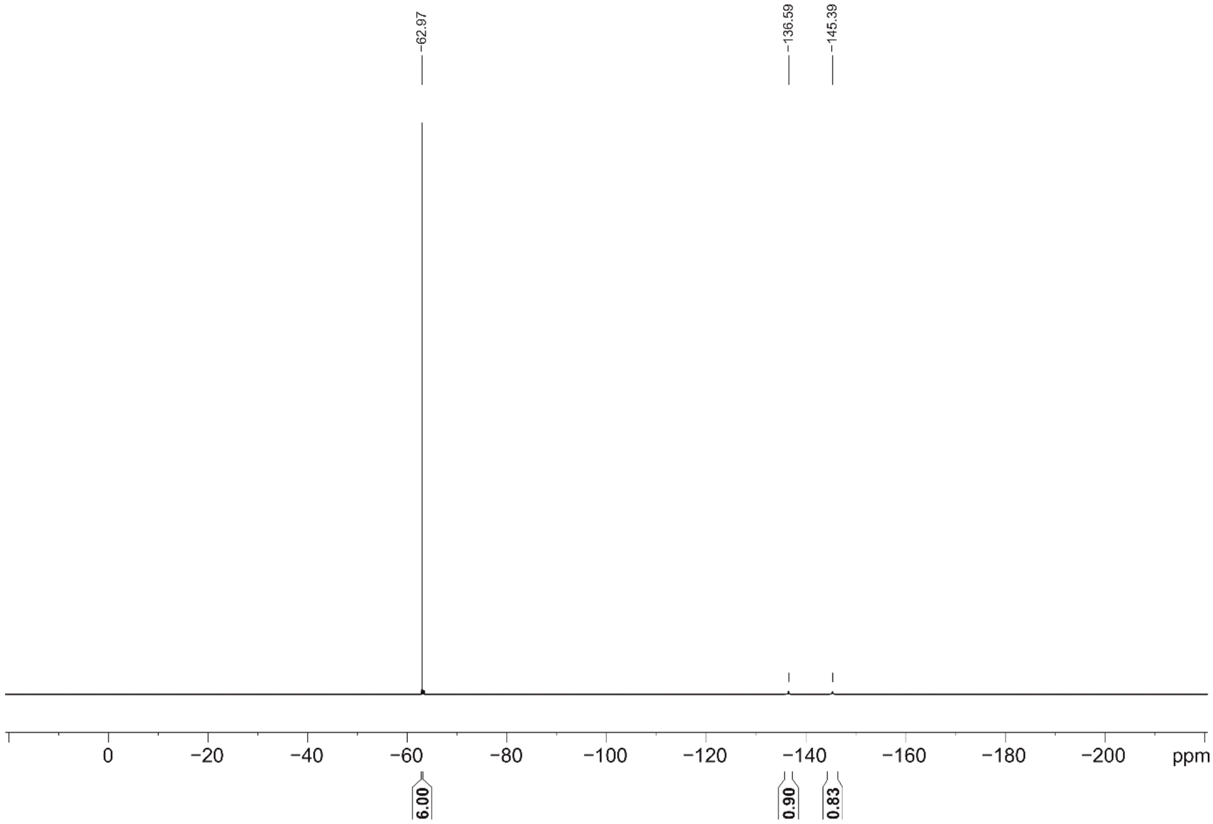

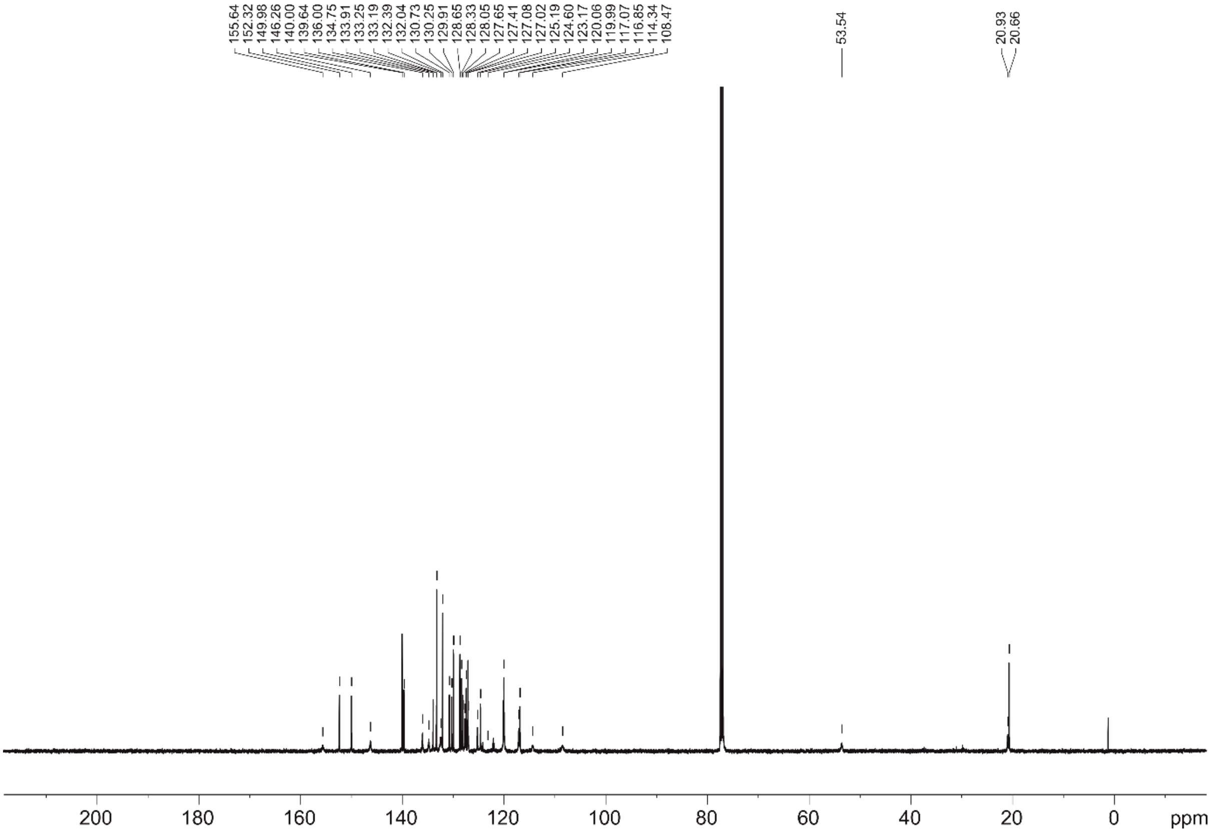


**(*S*)-3-(3,5-bis(trifluoromethyl)phenyl)-1-pentyl-1-(2'-(3-(3,4-difluorophenyl)ureido)-[1,1'-binaphthalen]-2- yl)urea (3g)**

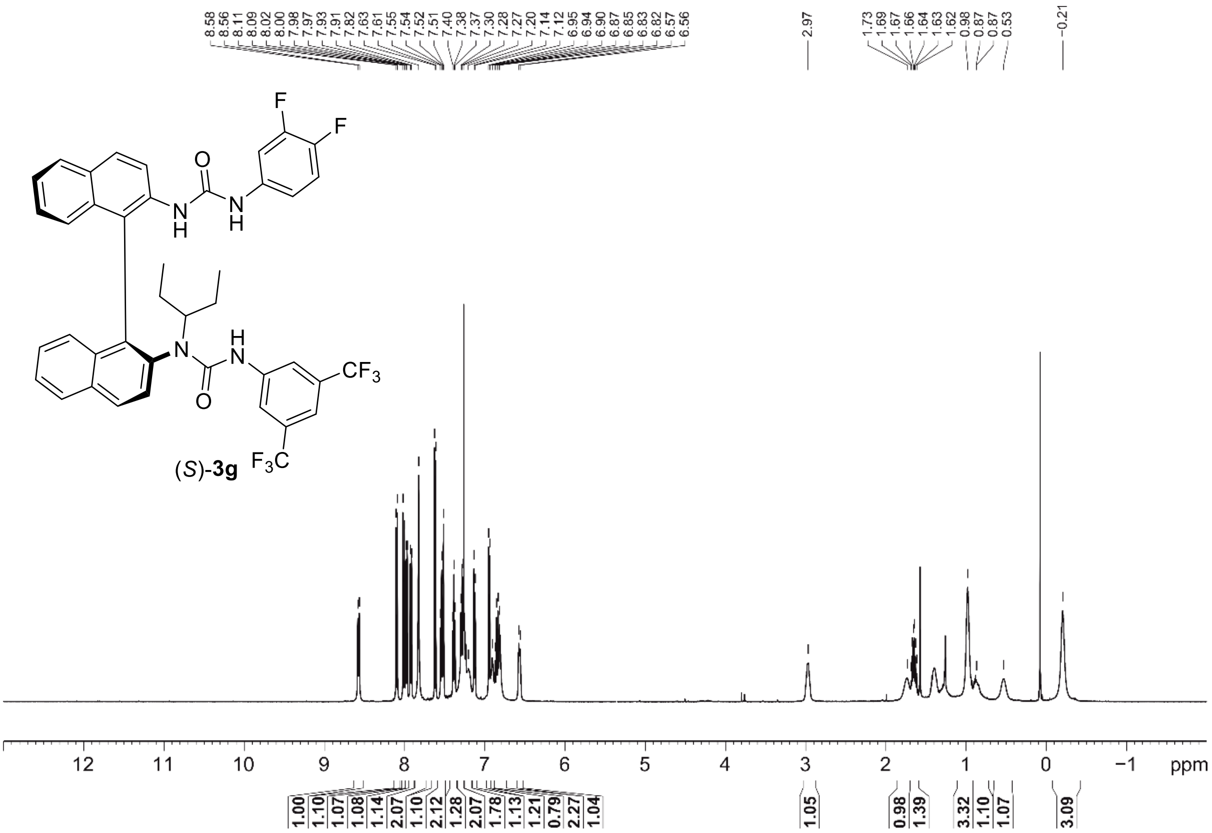

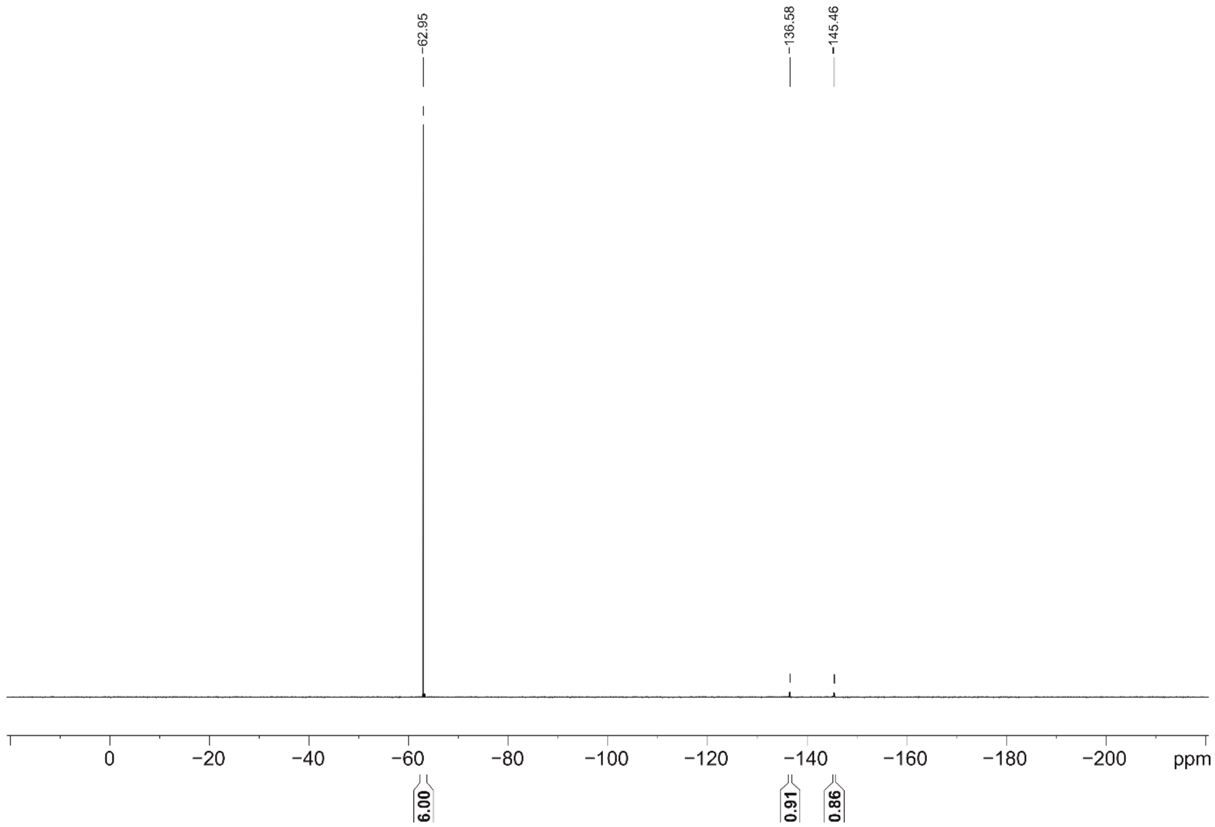
-62.95
-136.58
-145.46
0
-20
-40
-60
-80
-100
-120
-140
-160
-180
-200
ppm
6.00
0.91
0.86

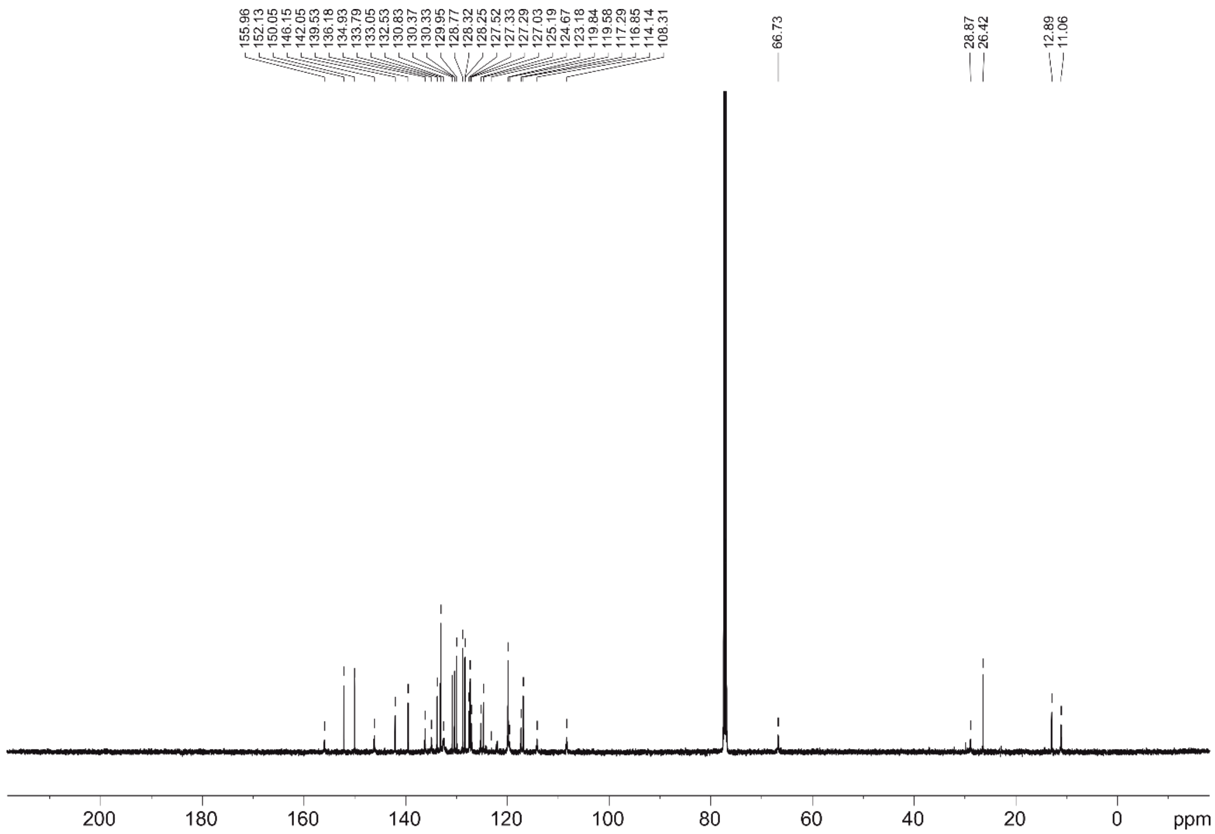
155.96
152.13
150.05
146.15
142.05
139.53
136.18
134.93
133.79
133.05
132.53
130.83
130.37
130.33
129.95
128.77
128.32
128.25
127.52
127.33
127.29
127.03
125.19
124.67
123.18
119.84
119.58
117.29
116.85
114.14
108.31
66.73
28.87
26.42
12.89
11.06
200
180
160
140
120
100
80
60
40
20
0
ppm

**(*S*)-3-(3,5-bis(trifluoromethyl)phenyl)-1-heptyl-1-(2'-(3-(3,4-difluorophenyl)ureido)-[1,1'-binaphthalen]-2- yl)urea (3h)**

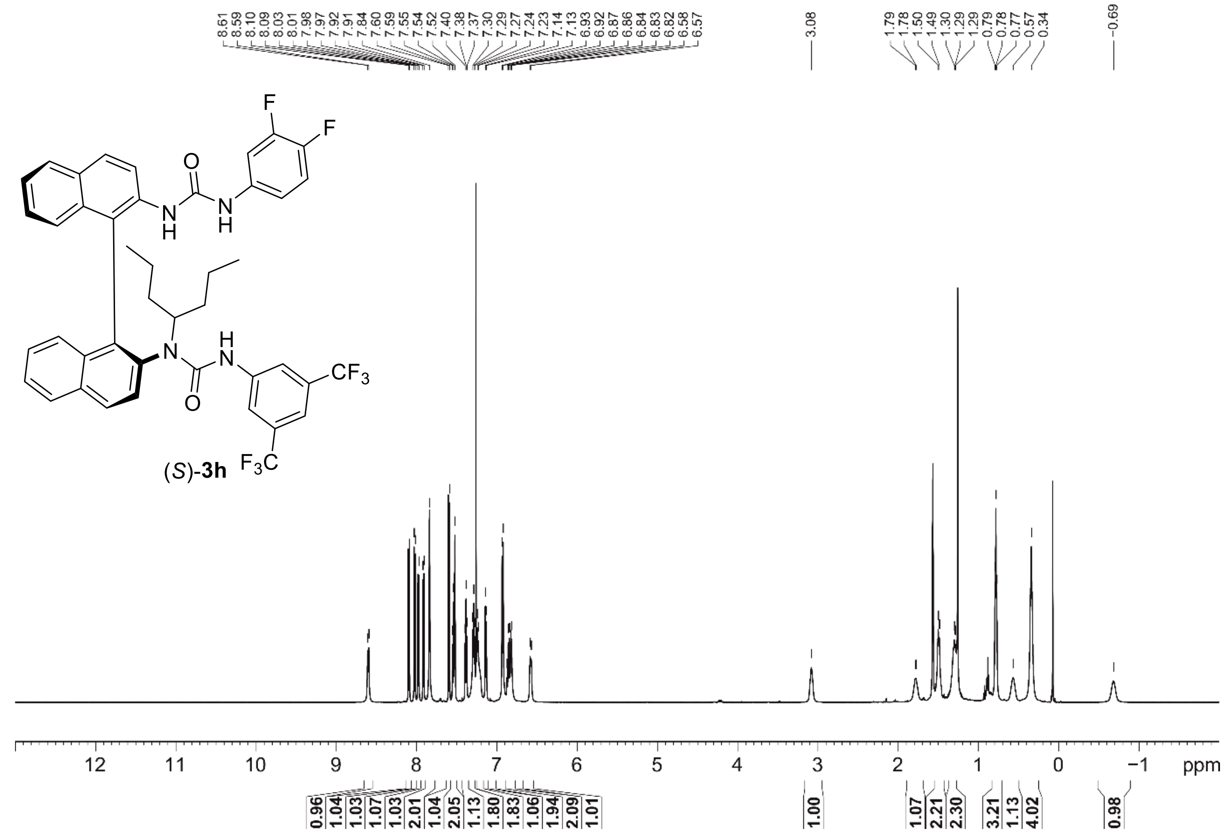

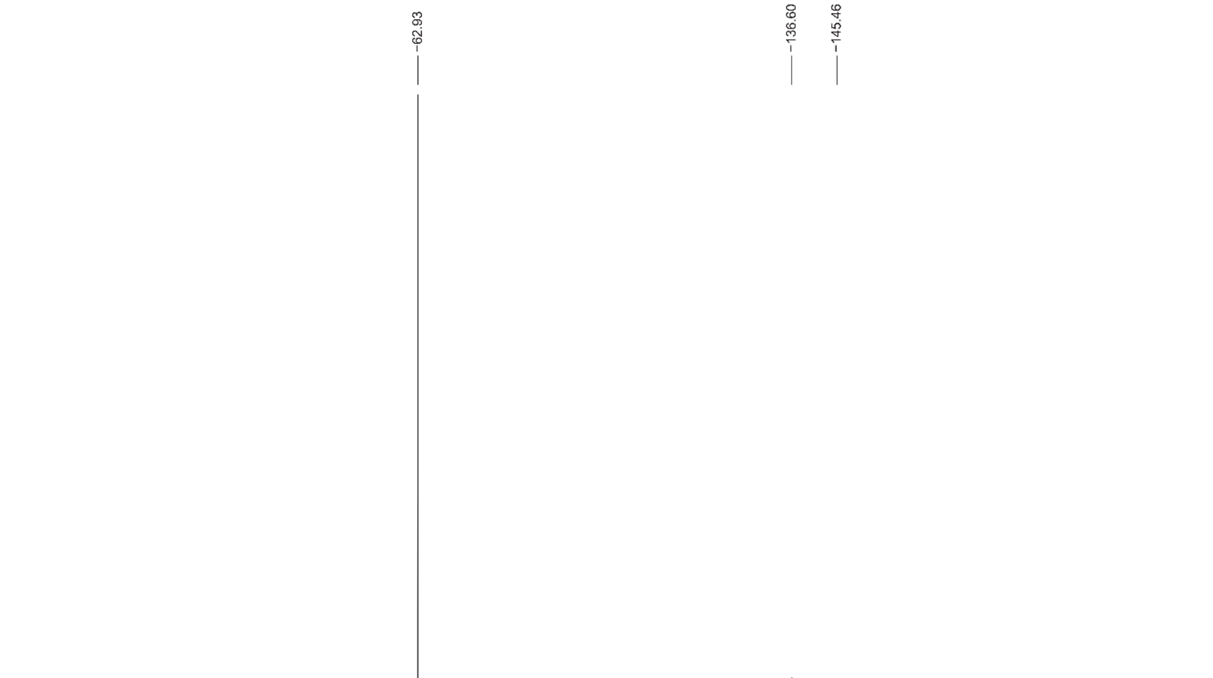

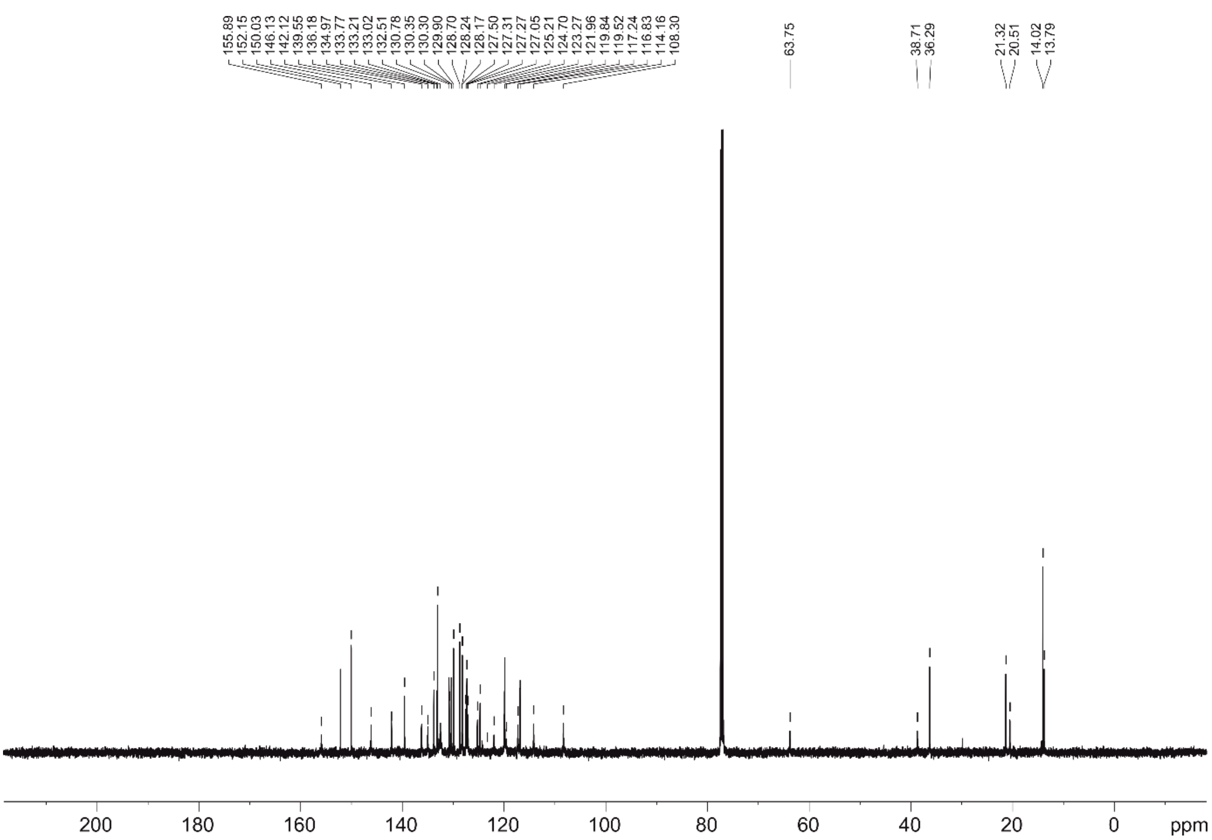


**(*S*)-3-(3,5-bis(trifluoromethyl)phenyl)-1-nonan-5-yl-1-(2'-(3-(3,4-difluorophenyl)ureido)-[1,1'-binaphthalen]-2- yl)urea (3i)**

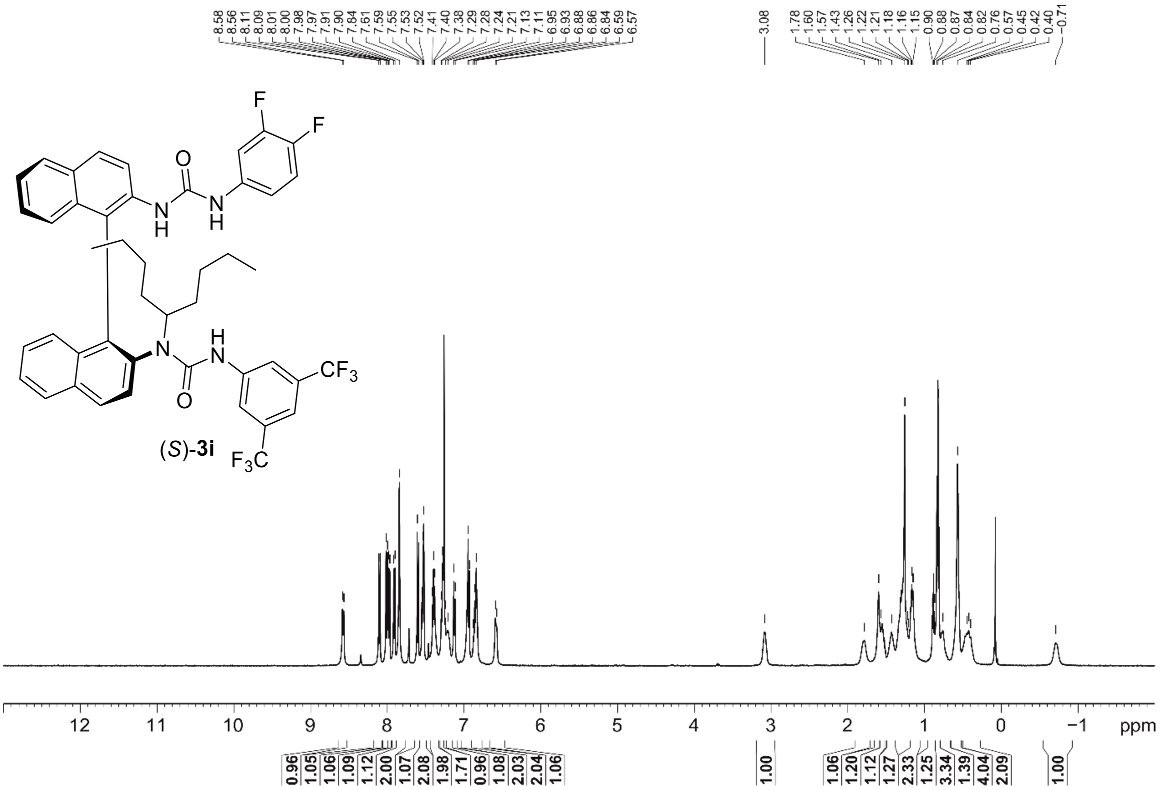

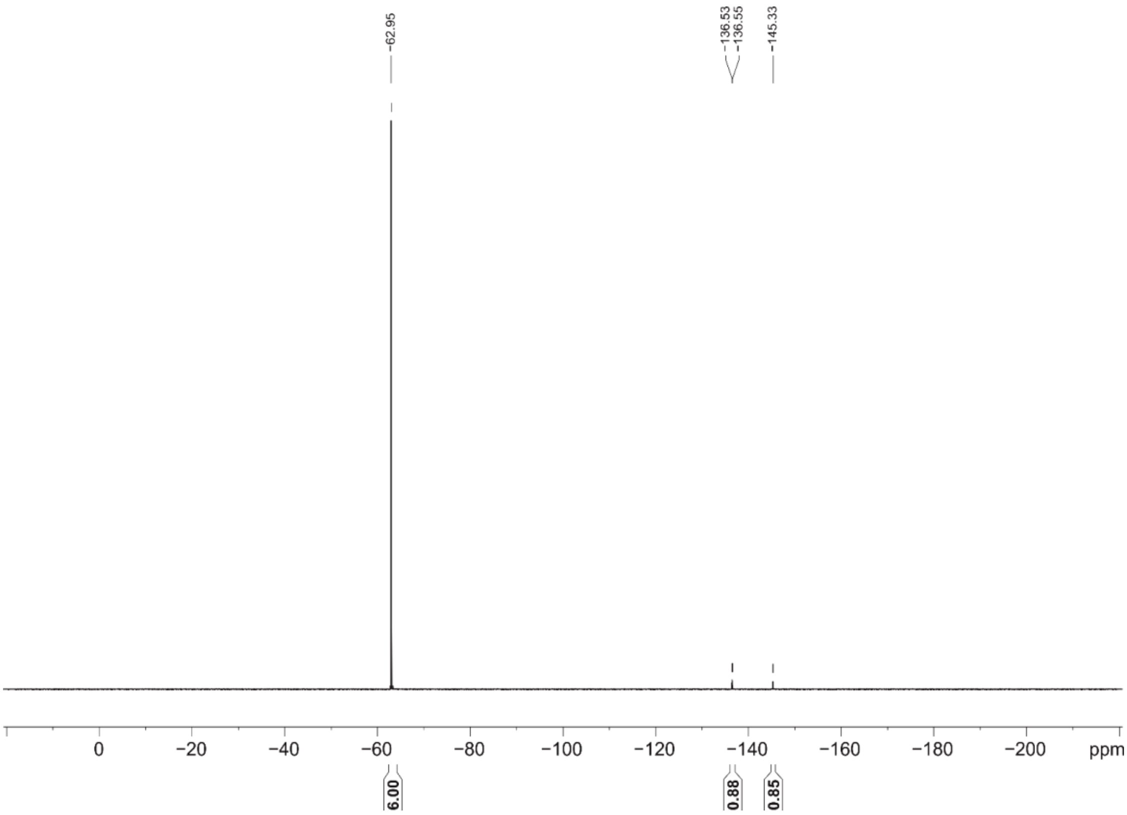
-62.95
-136.53
-136.55
-145.33
6.00
0.88
0.85
0 -20 -40 -60 -80 -100 -120 -140 -160 -180 -200 ppm

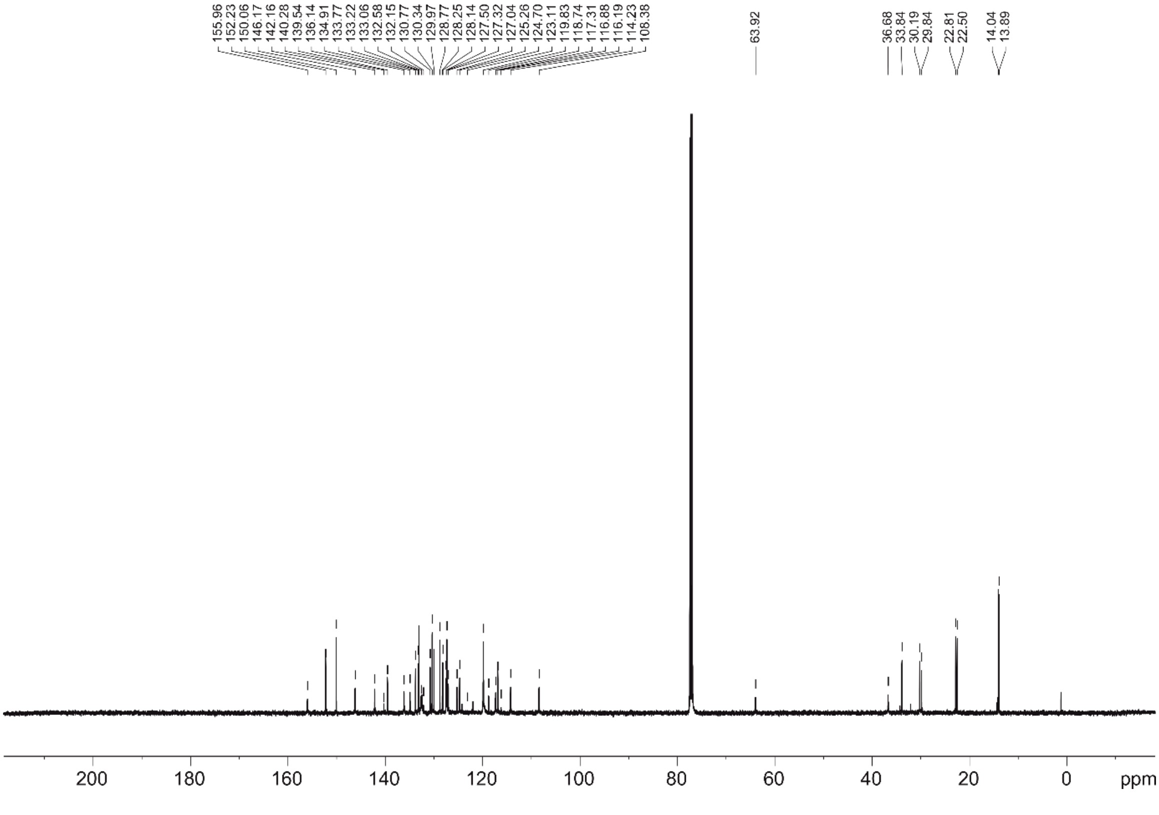
155.96
152.23
150.06
146.17
142.16
140.28
139.54
136.14
134.91
133.77
133.22
133.06
132.58
132.15
130.77
130.34
129.97
128.77
128.25
128.14
127.50
127.32
127.04
125.26
124.70
123.11
119.83
118.74
117.31
116.88
116.19
114.23
108.38
63.92
36.68
33.84
30.19
29.84
22.81
22.50
14.04
13.89
200 180 160 140 120 100 80 60 40 20 0 ppm

**(*S*)-3-(3,5-bis(trifluoromethyl)phenyl)-1-cyclohexyl-1-(2'-(3-(3,4-difluorophenyl)ureido)-[1,1'-binaphthalen]-2- yl)urea (3j)**

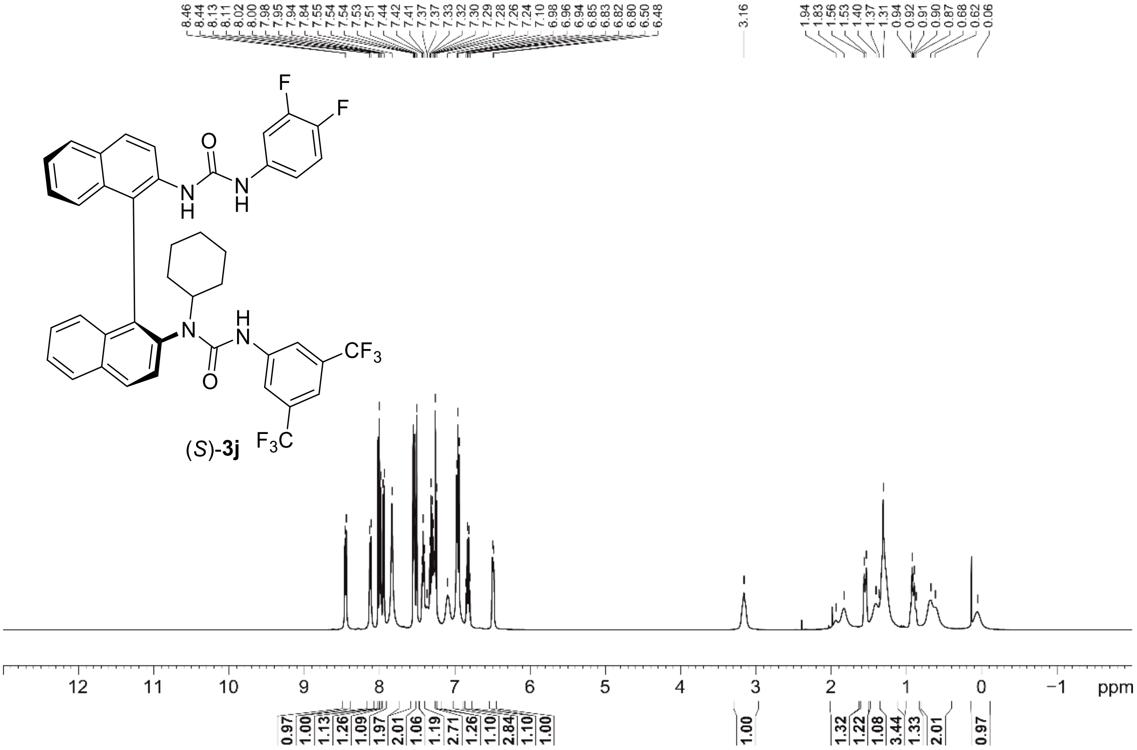


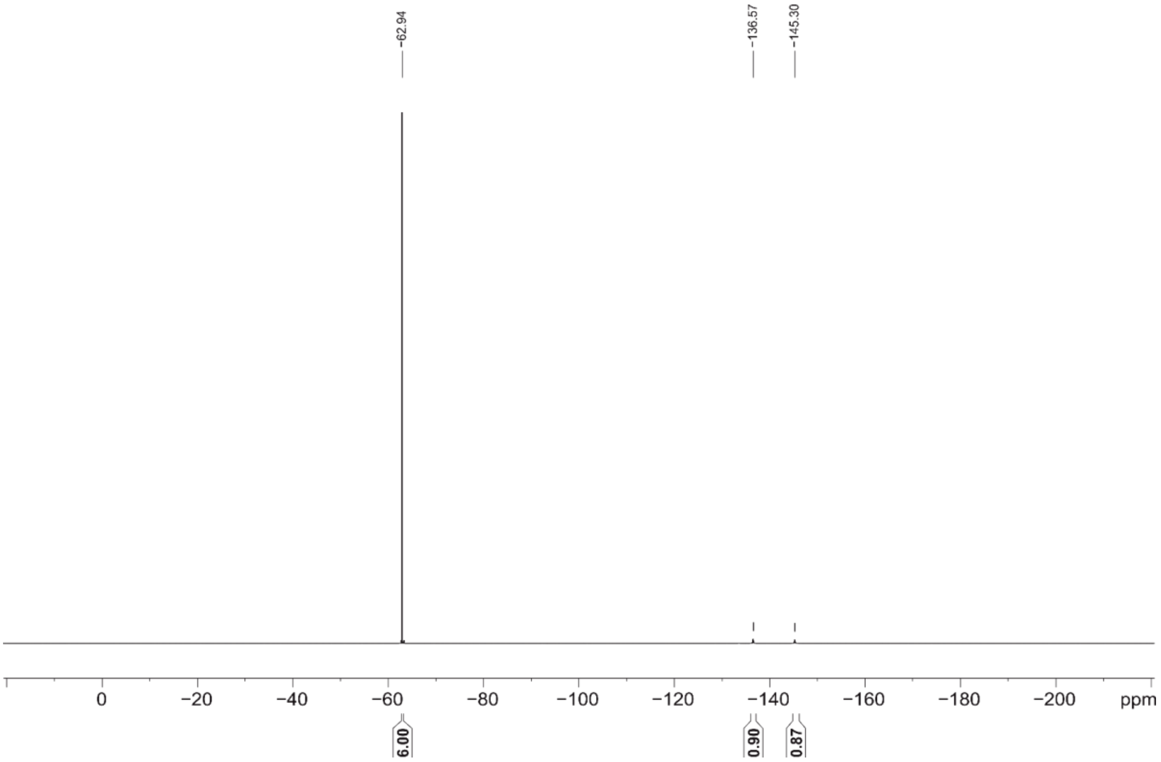

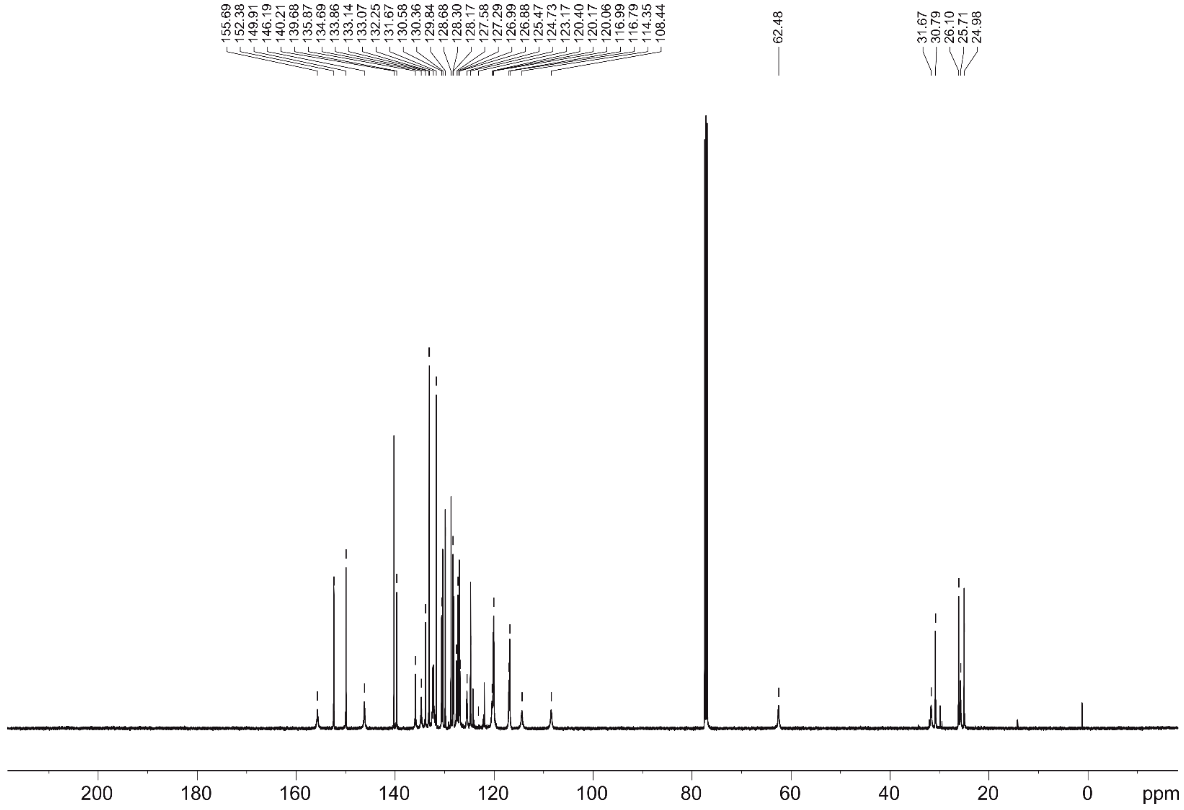
155.69
152.38
149.91
146.19
140.21
139.68
135.87
134.69
133.86
133.14
133.07
132.25
131.67
130.58
130.36
129.84
128.68
128.30
128.17
127.58
127.29
126.99
126.88
125.47
124.73
123.17
120.40
120.17
120.06
116.99
116.79
114.35
108.44
62.48
31.67
30.79
26.10
25.71
24.98
200
180
160
140
120
100
80
60
40
20
0
ppm

## Copies of HPLC Traces

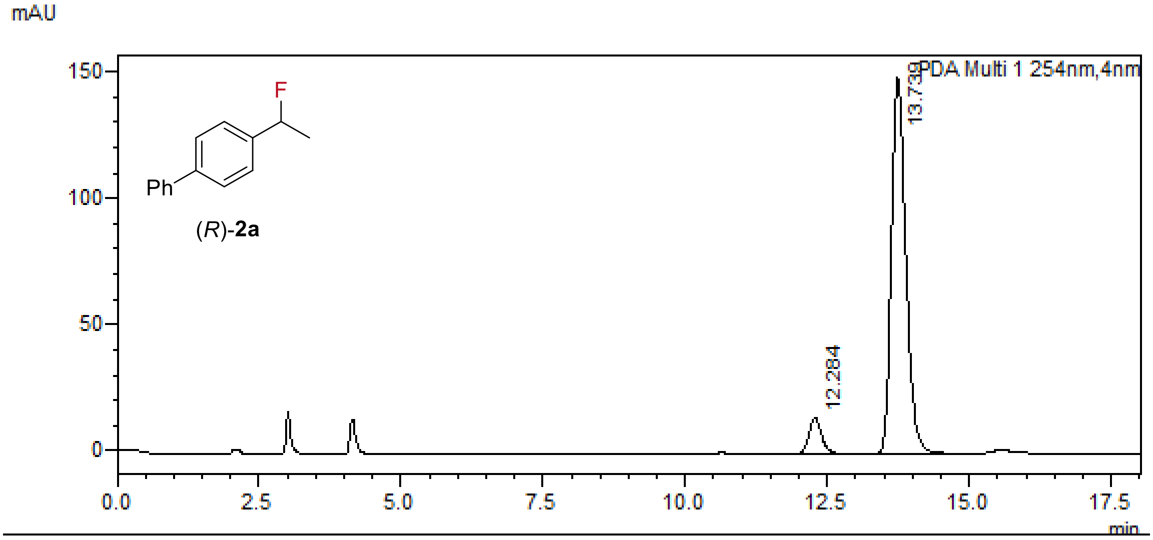


PDA Ch1 254nm

| Peak# | Ret. Time | Area% |
|---|---|---|
| 1 | 12.284 | 7.613 |
| 2 | 13.739 | 92.387 |

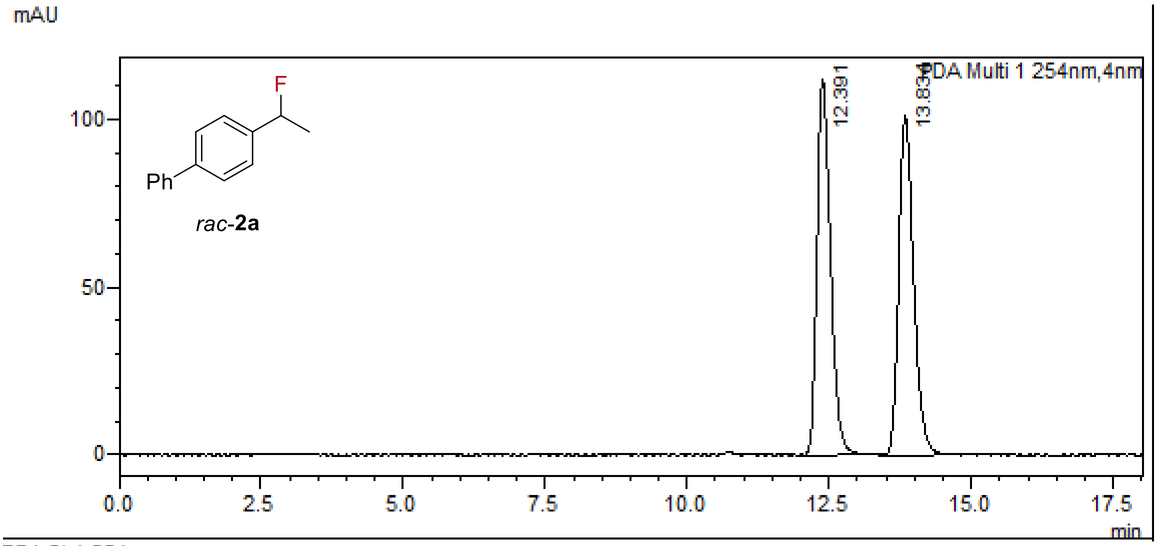


PDA Ch1 254nm

| Peak# | Ret. Time | Area% |
|---|---|---|
| 1 | 12.391 | 50.029 |
| 2 | 13.834 | 49.971 |

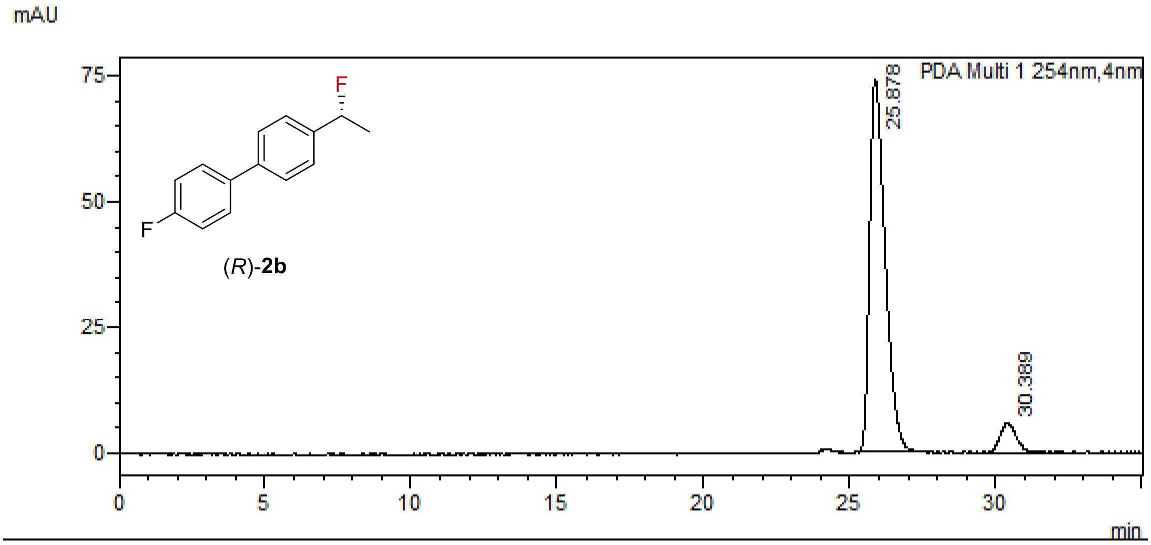


PDA Ch1 254nm

| Peak# | Ret. Time | Area% |
|---|---|---|
| 1 | 25.878 | 92.195 |
| 2 | 30.389 | 7.805 |

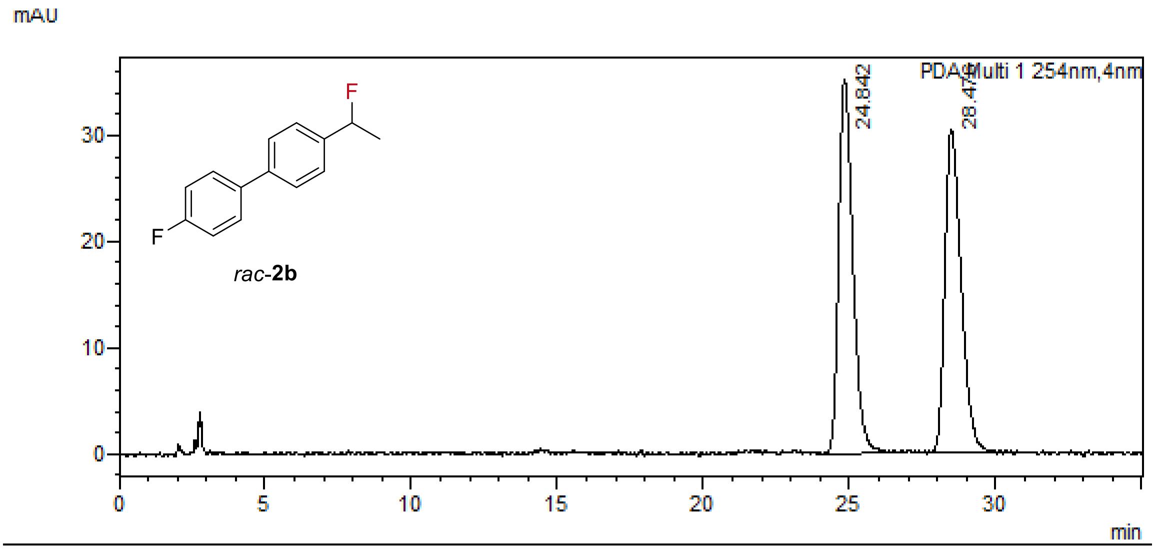


PDA Ch1 254nm

| Peak# | Ret. Time | Area% |
|---|---|---|
| 1 | 24.842 | 50.200 |
| 2 | 28.476 | 49.800 |

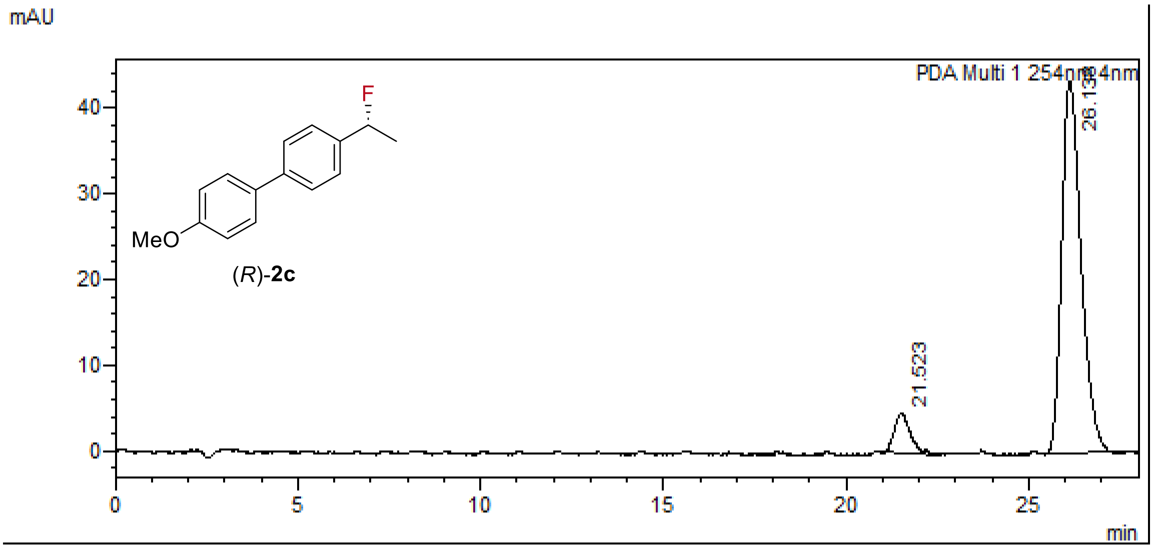


PDA Ch1 254nm

| Peak# | Ret. Time | Area% |
|---|---|---|
| 1 | 21.523 | 8.317 |
| 2 | 26.138 | 91.683 |

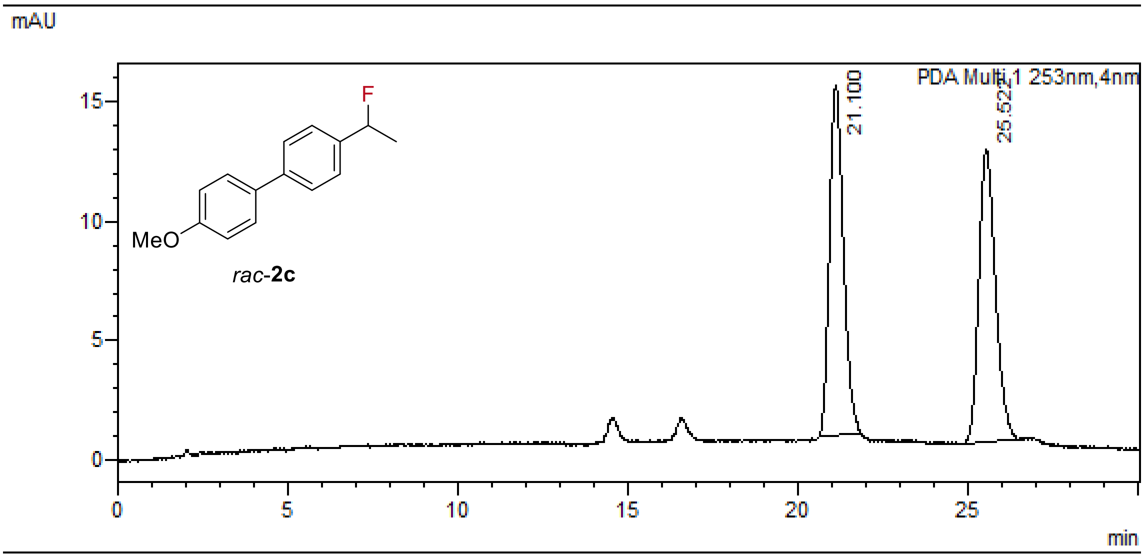


PDA Ch1 253nm

| Peak# | Ret. Time | Area% |
|---|---|---|
| 1 | 21.100 | 50.050 |
| 2 | 25.522 | 49.950 |

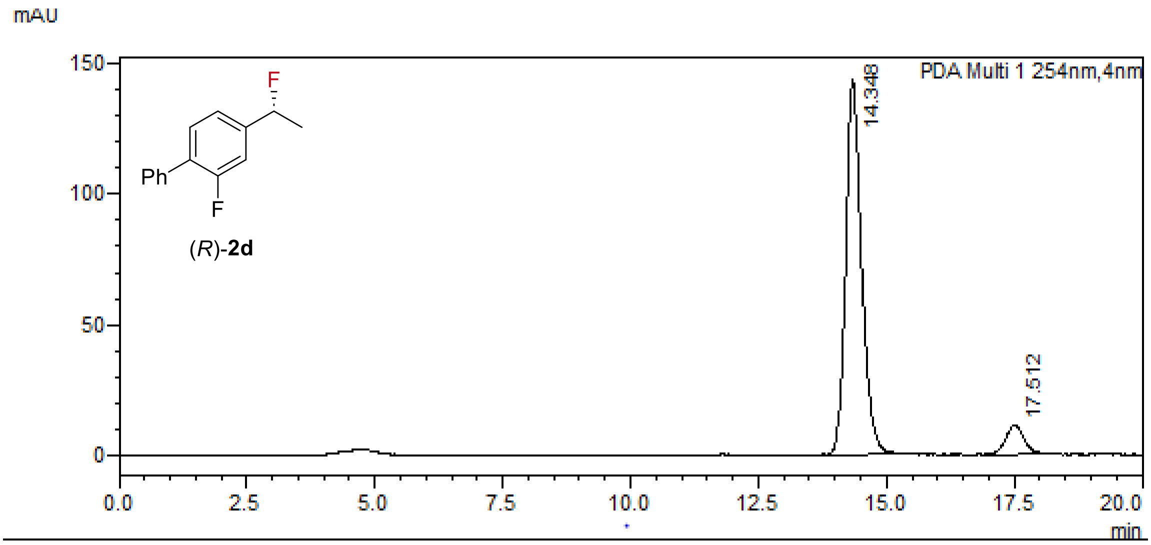


PDA Ch1 254nm

| Peak# | Ret. Time | Area% |
|---|---|---|
| 1 | 14.348 | 91.435 |
| 2 | 17.512 | 8.565 |

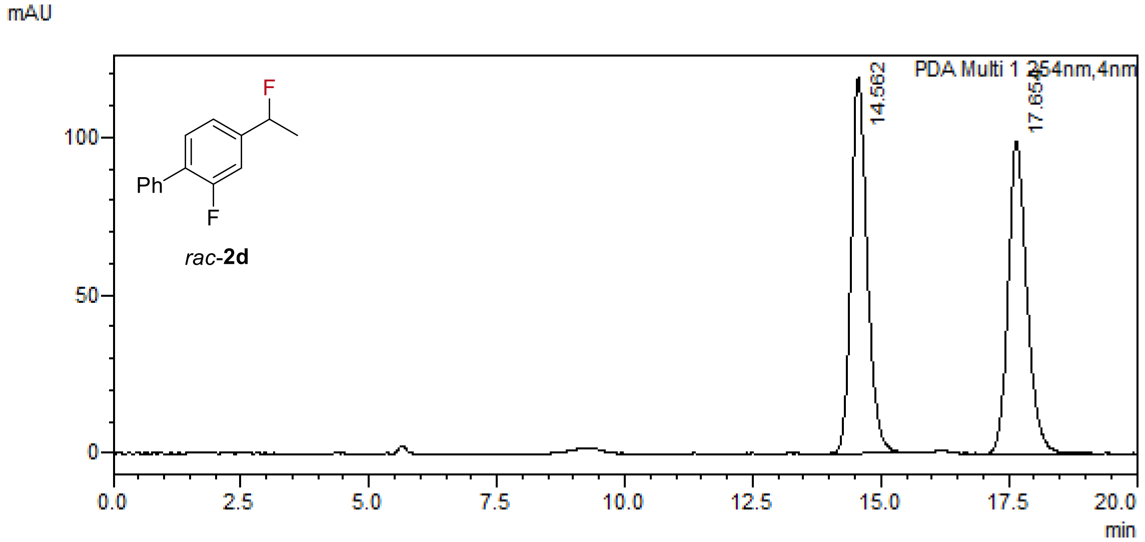


PDA Ch1 254nm

| Peak# | Ret. Time | Area% |
|---|---|---|
| 1 | 14.562 | 50.099 |
| 2 | 17.654 | 49.901 |

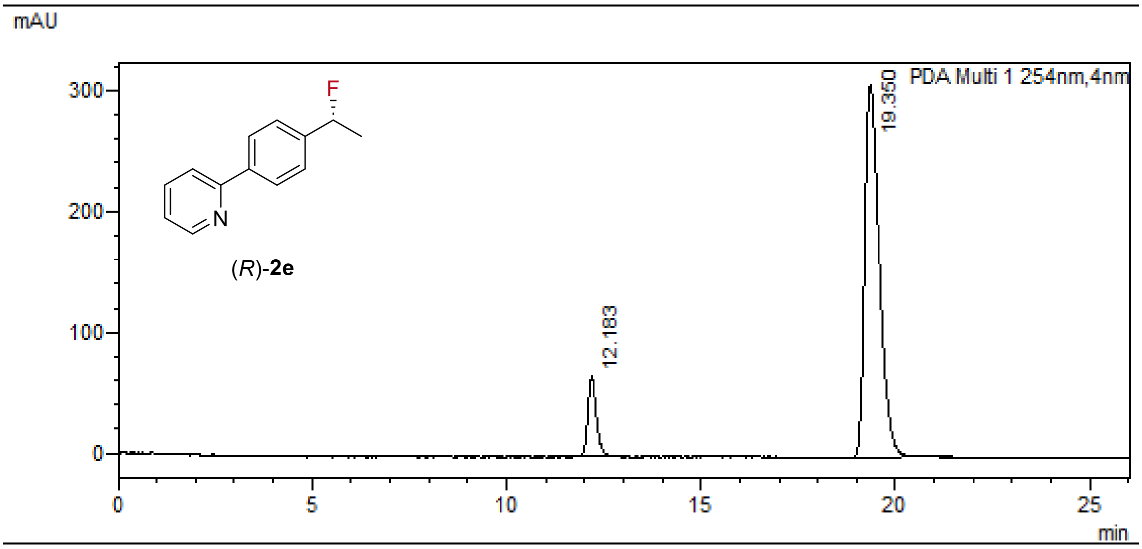


PDA Ch1 254nm

| Peak# | Ret. Time | Area% |
|---|---|---|
| 1 | 12.183 | 11.444 |
| 2 | 19.350 | 88.556 |

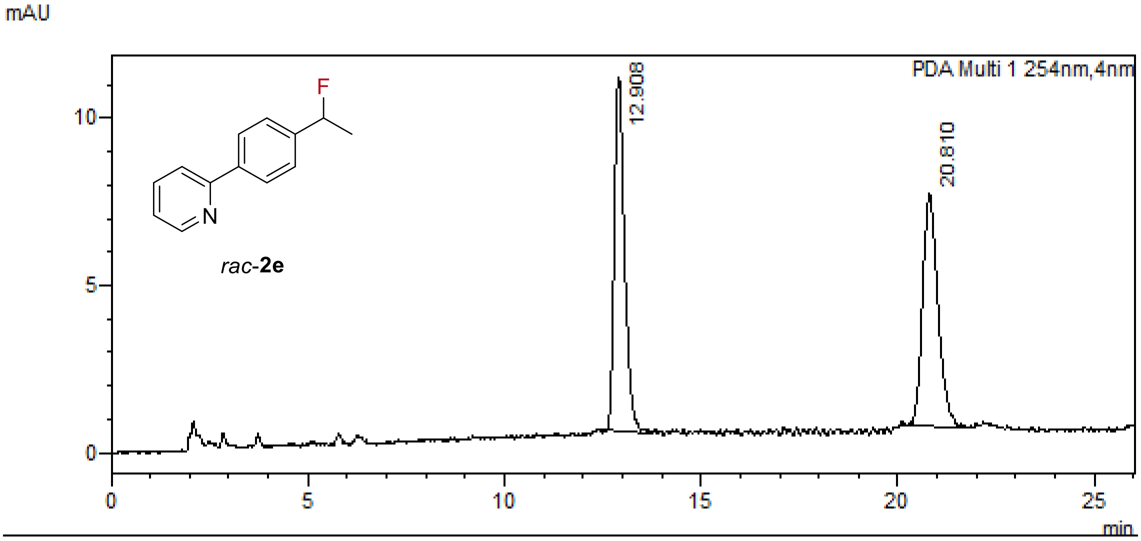


PDA Ch1 254nm

| Peak# | Ret. Time | Area% |
|---|---|---|
| 1 | 12.908 | 50.147 |
| 2 | 20.810 | 49.853 |

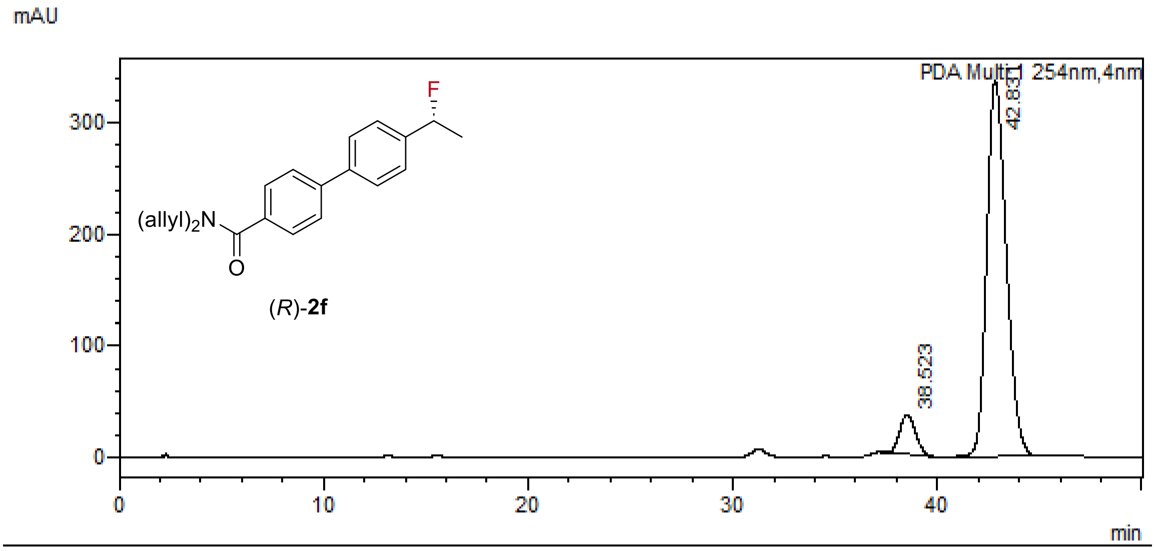


PDA Ch1 254nm

| Peak# | Ret. Time | Area% |
|---|---|---|
| 1 | 38.523 | 7.914 |
| 2 | 42.831 | 92.086 |

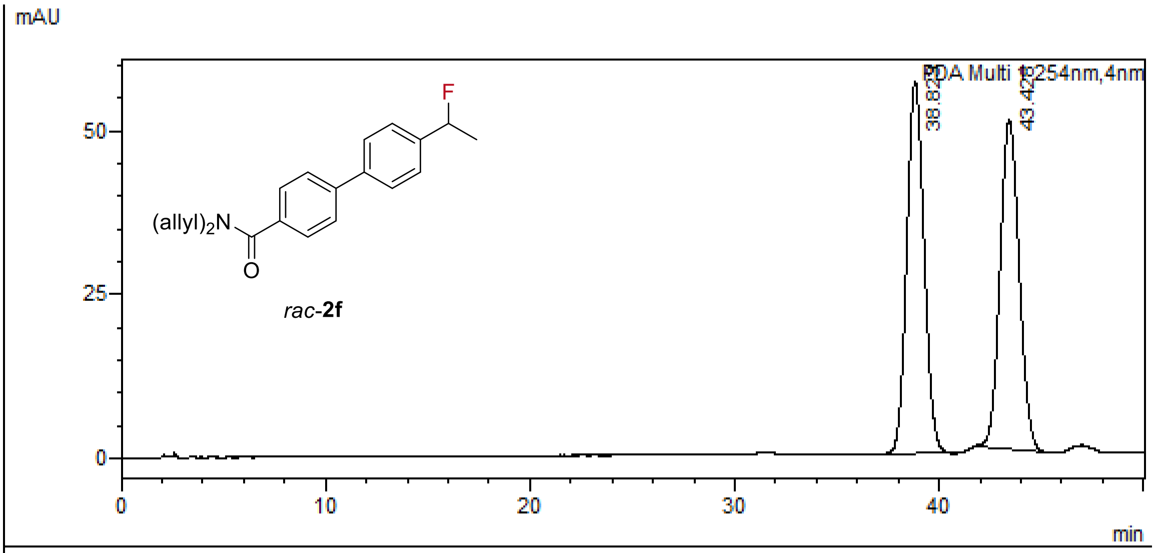


PDA Ch1 254nm

| Peak# | Ret. Time | Area% |
|---|---|---|
| 1 | 38.823 | 50.313 |
| 2 | 43.428 | 49.687 |

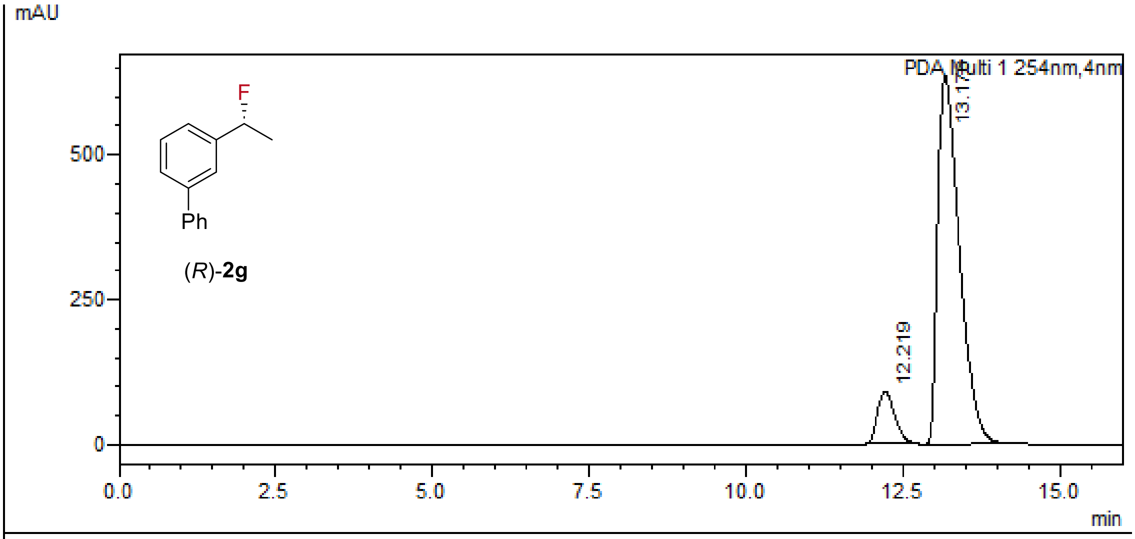


PDA Ch1 254nm

| Peak# | Ret. Time | Area% |
|---|---|---|
| 1 | 12.219 | 9.614 |
| 2 | 13.176 | 90.386 |

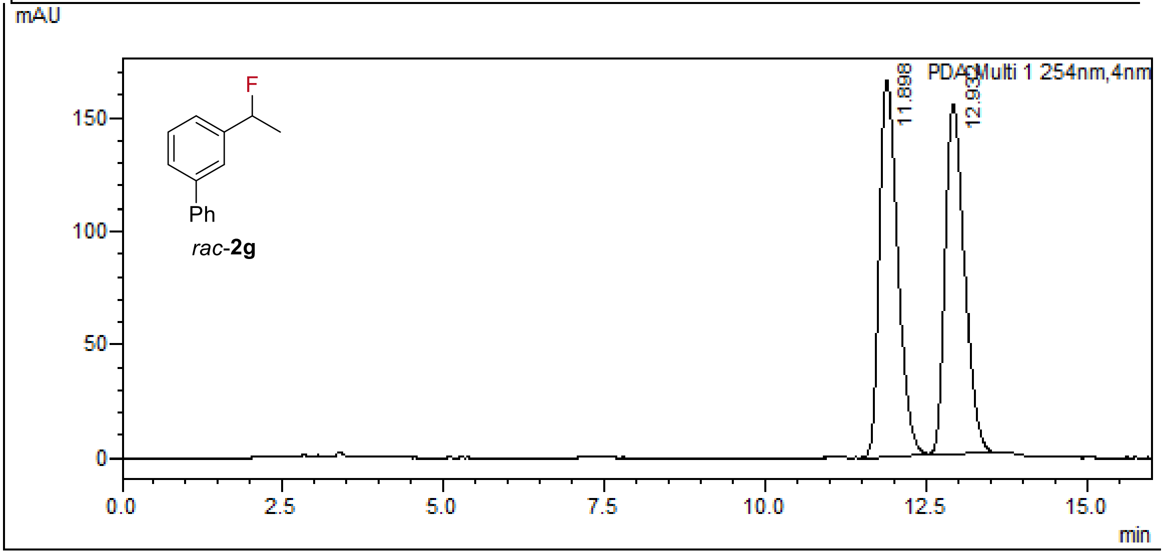


PDA Ch1 254nm

| Peak# | Ret. Time | Area% |
|---|---|---|
| 1 | 11.898 | 50.306 |
| 2 | 12.932 | 49.694 |

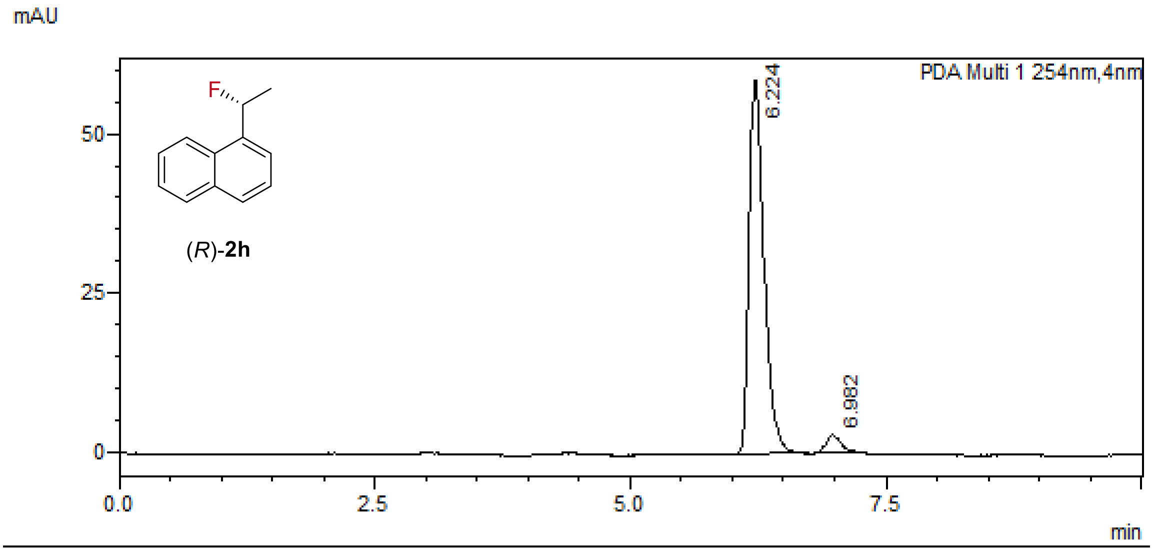


PDA Ch1 254nm

| Peak# | Ret. Time | Area% |
|---|---|---|
| 1 | 6.224 | 95.315 |
| 2 | 6.982 | 4.685 |

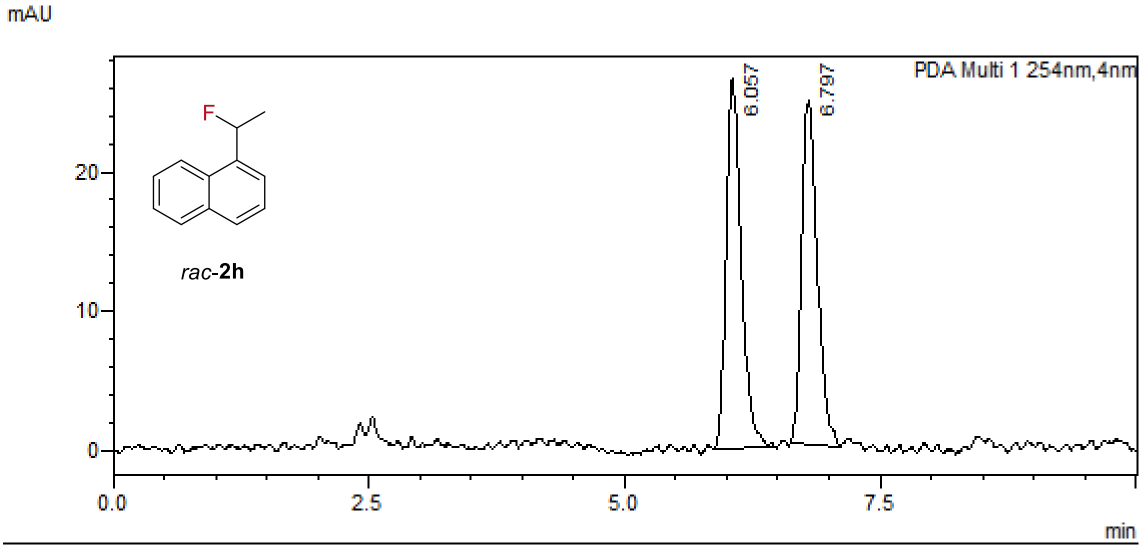


PDA Ch1 254nm

| Peak# | Ret. Time | Area% |
|---|---|---|
| 1 | 6.057 | 50.745 |
| 2 | 6.797 | 49.255 |

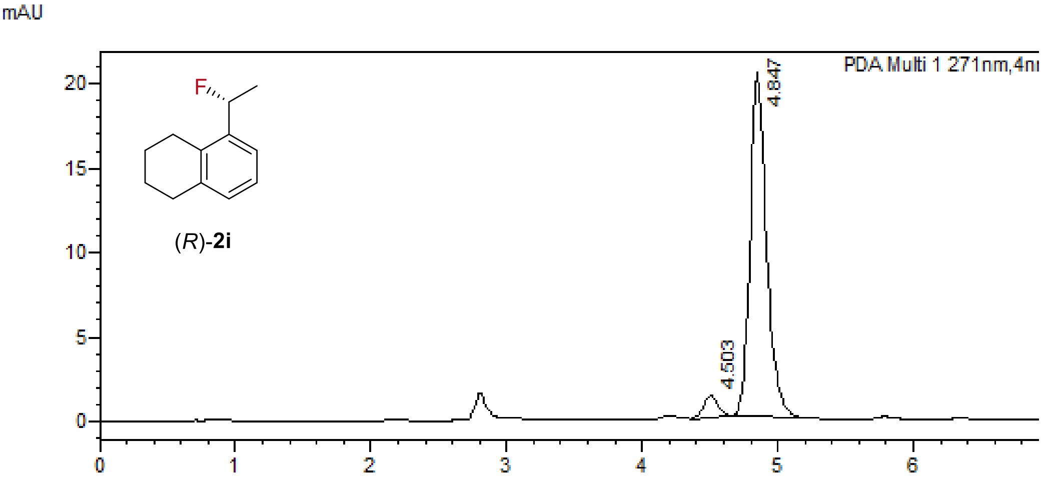


DA Ch1 271nm

| eak# | Ret. Time | Area% |
|---|---|---|
| 1 | 4.503 | 4.910 |
| 2 | 4.847 | 95.090 |

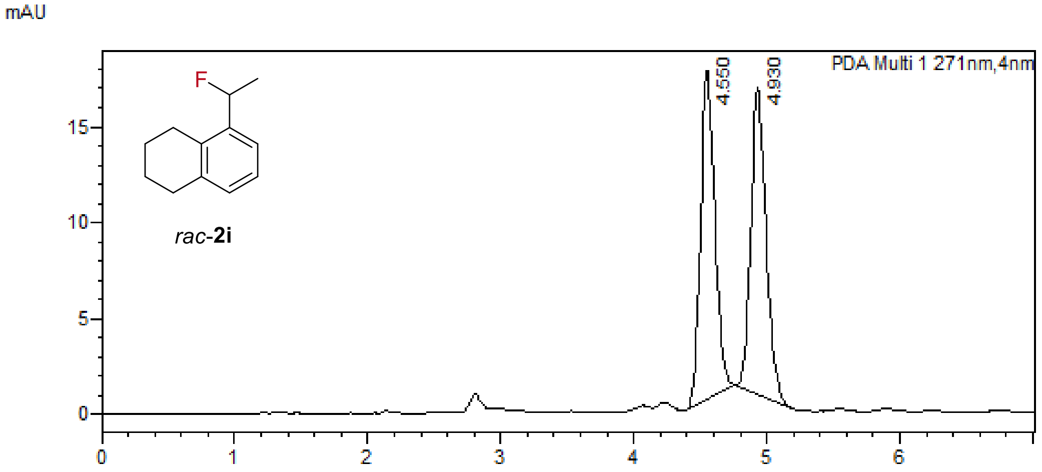


DA Ch1 271nm

| 'eak# | Ret. Time | Area% |
|---|---|---|
| 1 | 4.550 | 50.066 |
| 2 | 4.930 | 49.934 |

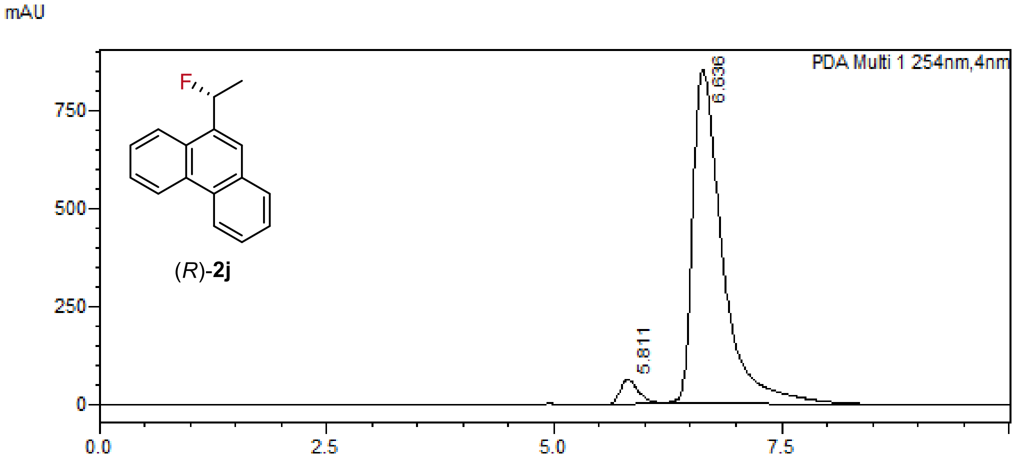


PDA Ch1 254nm

| Peak# | Ret. Time | Area% |
|---|---|---|
| 1 | 5.811 | 4.244 |
| 2 | 6.636 | 95.756 |

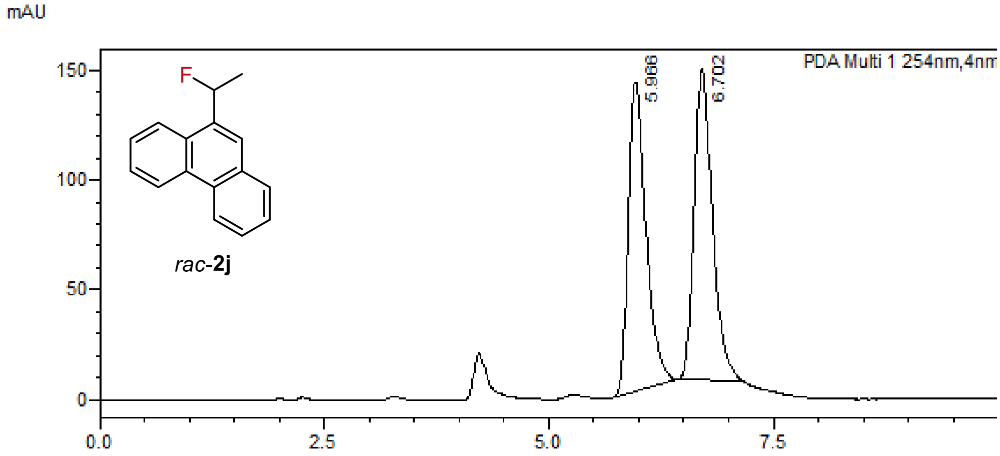


PDA Ch1 254nm

| Peak# | Ret. Time | Area% |
|---|---|---|
| 1 | 5.966 | 49.118 |
| 2 | 6.702 | 50.882 |

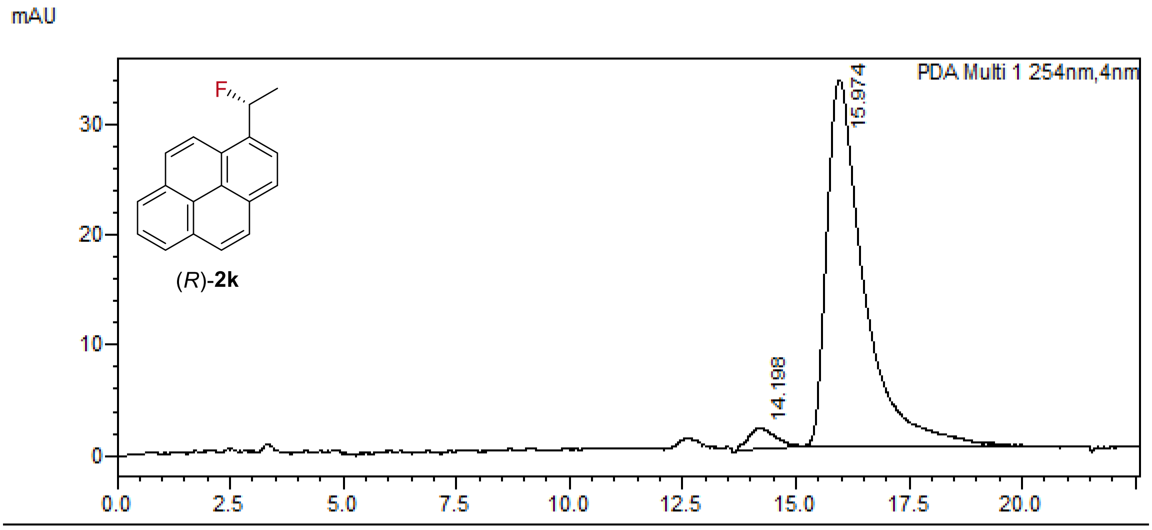


PDA Ch1 254nm

| Peak# | Ret. Time | Area% |
|---|---|---|
| 1 | 14.198 | 3.974 |
| 2 | 15.974 | 96.026 |

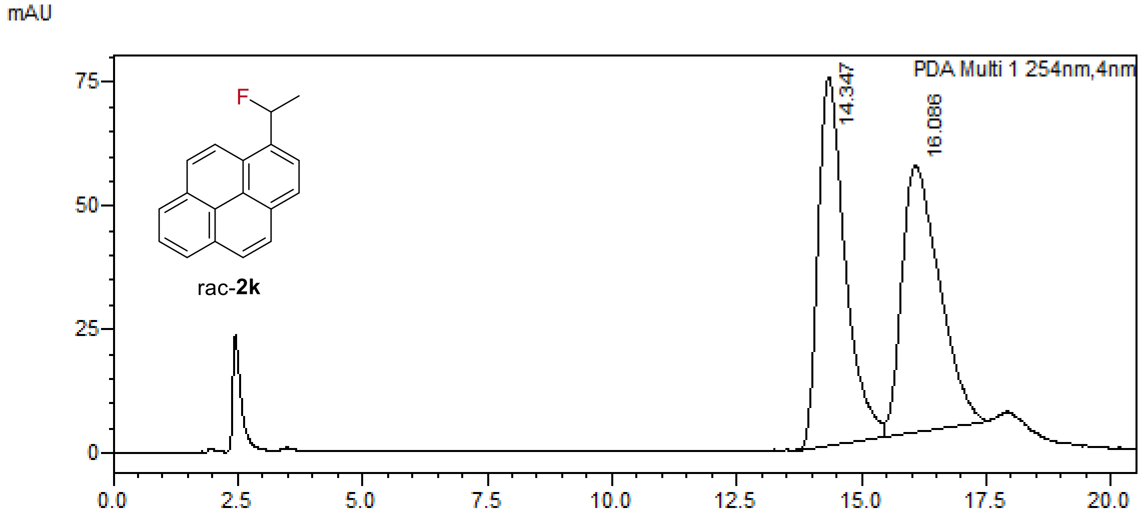


PDA Ch1 254nm

| Peak# | Ret. Time | Area% |
|---|---|---|
| 1 | 14.347 | 49.803 |
| 2 | 16.086 | 50.197 |

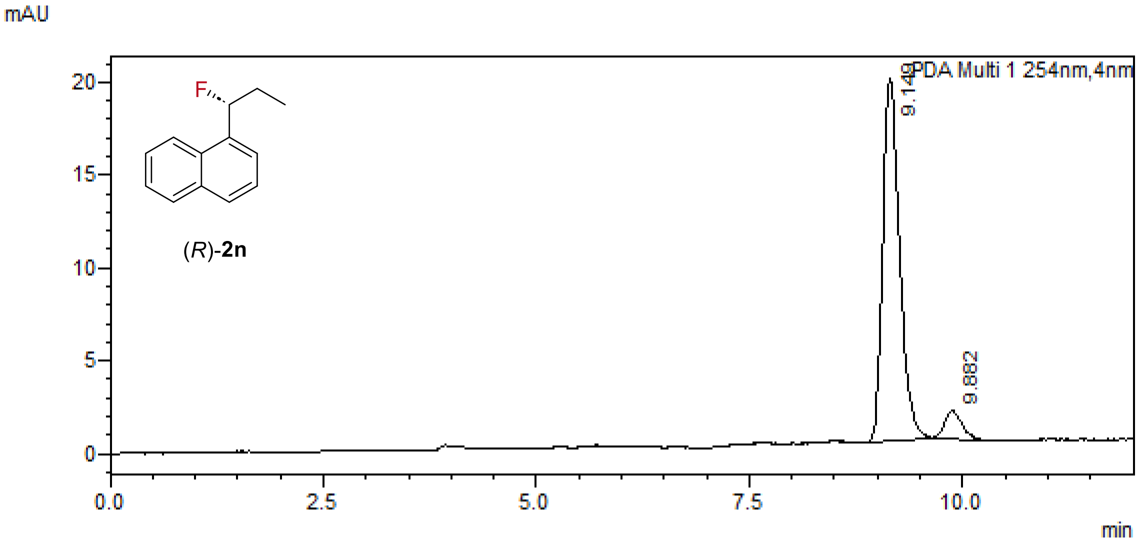


PDA Ch1 254nm

| Peak# | Ret. Time | Area% |
|---|---|---|
| 1 | 9.149 | 92.554 |
| 2 | 9.882 | 7.446 |

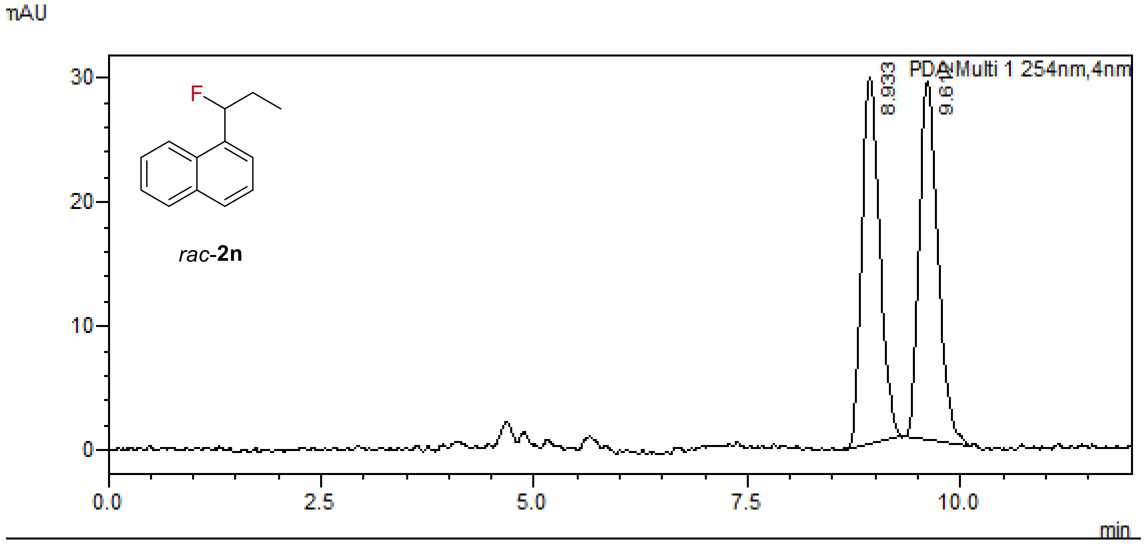


DA Ch1 254nm

| eak# | Ret. Time | Area% |
|---|---|---|
| 1 | 8.933 | 49.590 |
| 2 | 9.612 | 50.410 |

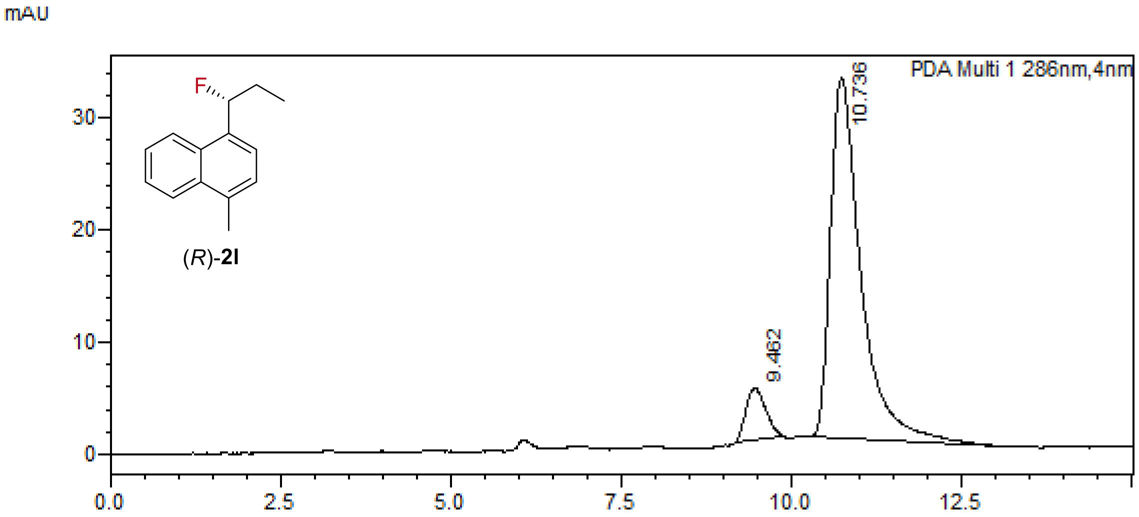


PDA Ch1 286nm

| Peak# | Ret. Time | Area% |
|---|---|---|
| 1 | 9.462 | 8.277 |
| 2 | 10.736 | 91.723 |

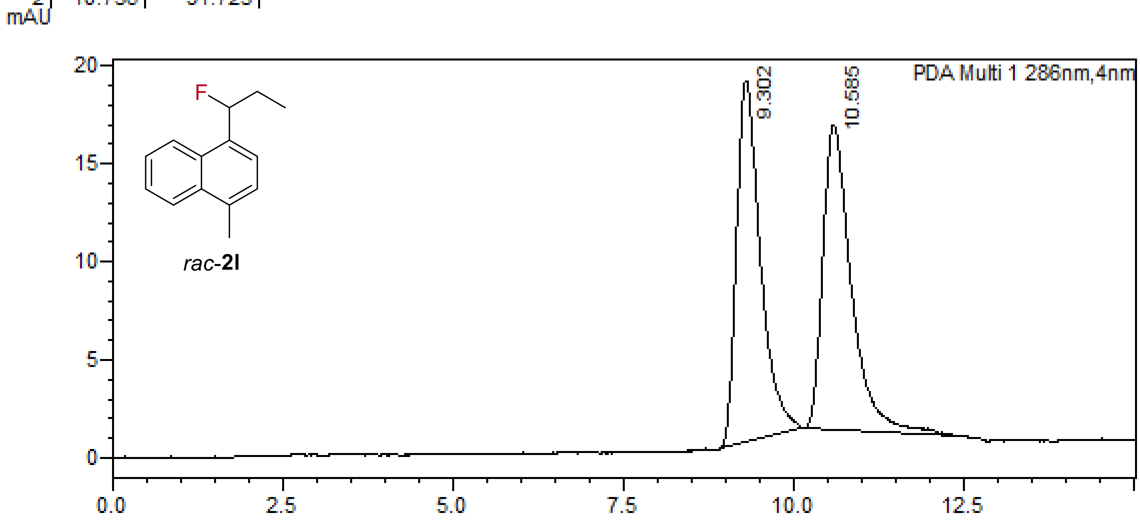


PDA Ch1 286nm

| Peak# | Ret. Time | Area% |
|---|---|---|
| 1 | 9.302 | 49.627 |
| 2 | 10.585 | 50.373 |

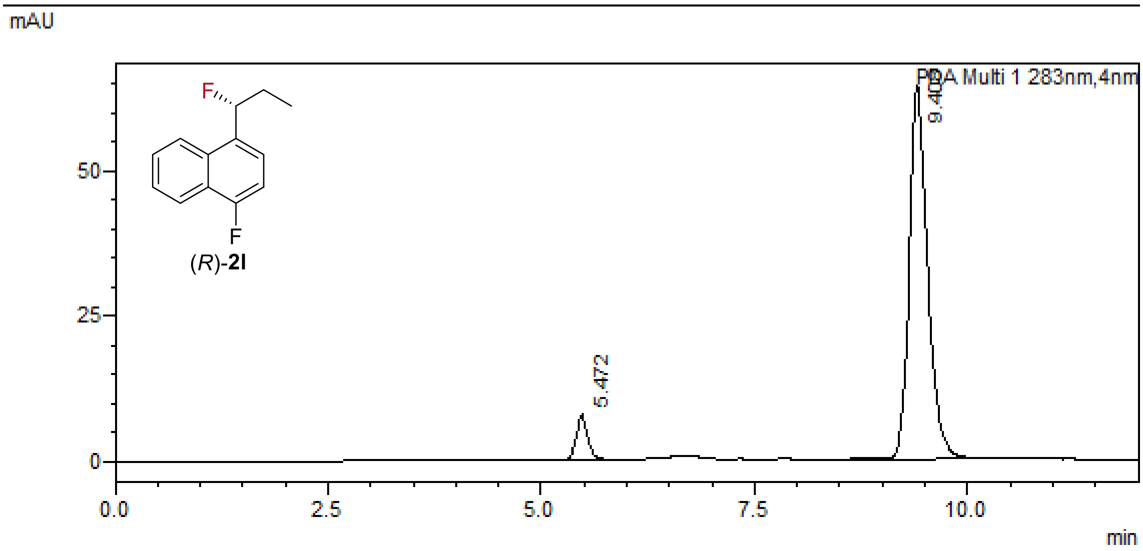


PDA Ch1 283nm

| Peak# | Ret. Time | Area% |
|---|---|---|
| 1 | 5.472 | 5.392 |
| 2 | 9.408 | 94.608 |

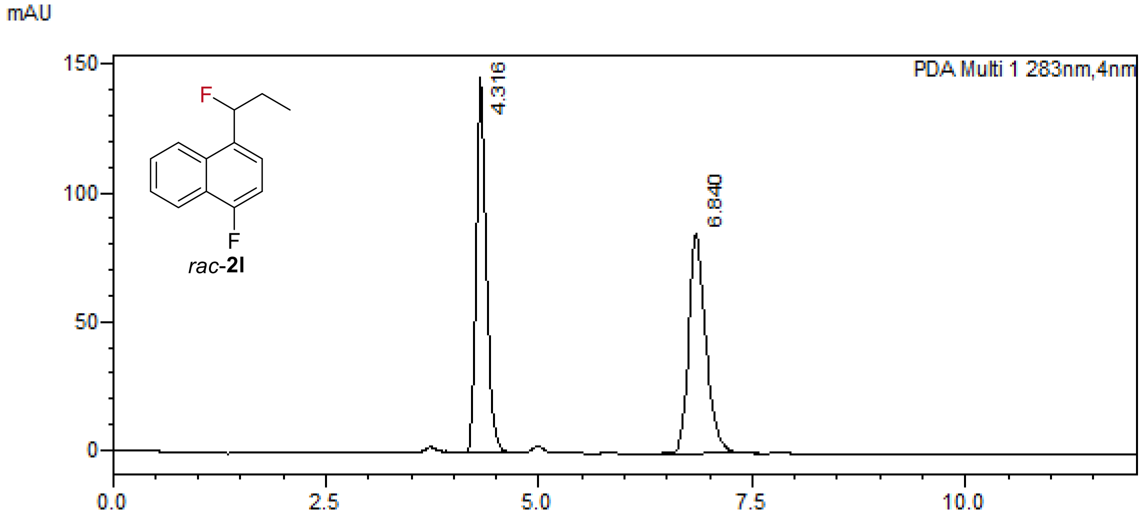


PDA Ch1 283nm

| Peak# | Ret. Time | Area% |
|---|---|---|
| 1 | 4.316 | 50.016 |
| 2 | 6.840 | 49.984 |

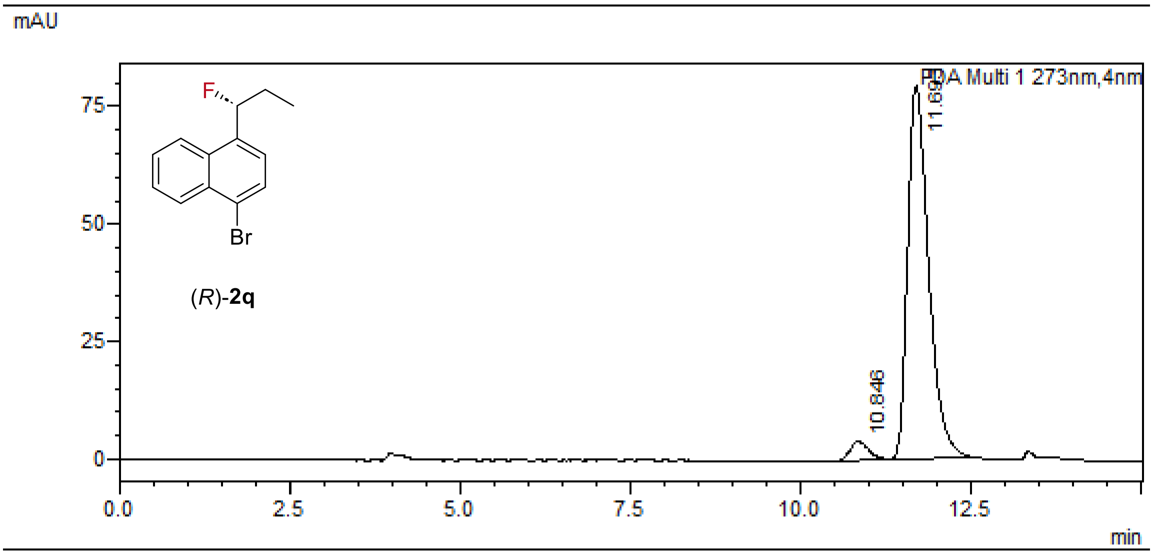


PDA Ch1 273nm

| Peak# | Ret. Time | Area% |
|---|---|---|
| 1 | 10.846 | 4.117 |
| 2 | 11.693 | 95.883 |

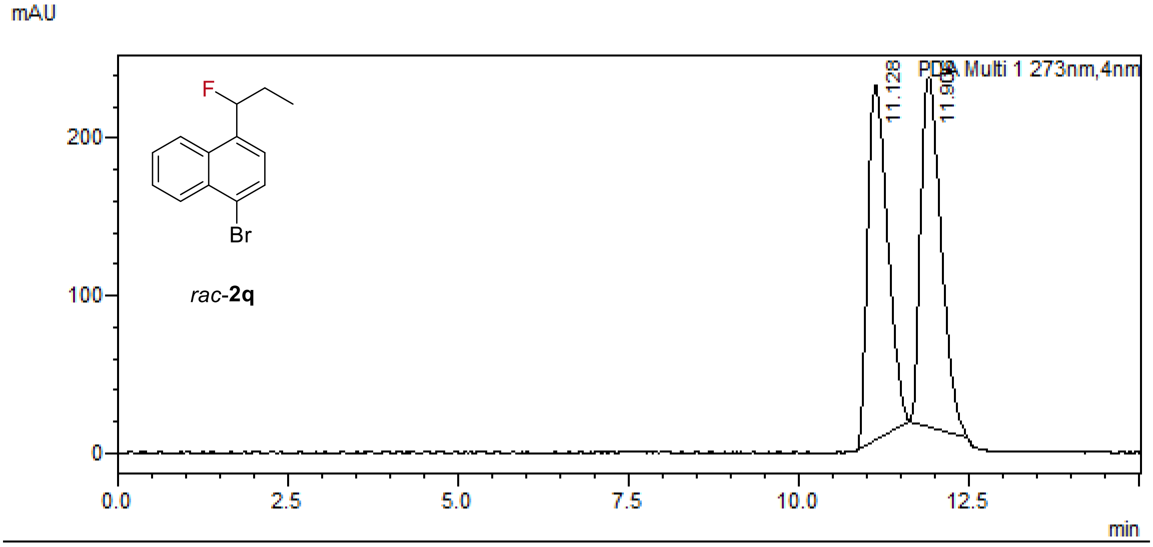


PDA Ch1 273nm

| Peak# | Ret. Time | Area% |
|---|---|---|
| 1 | 11.128 | 49.869 |
| 2 | 11.906 | 50.131 |

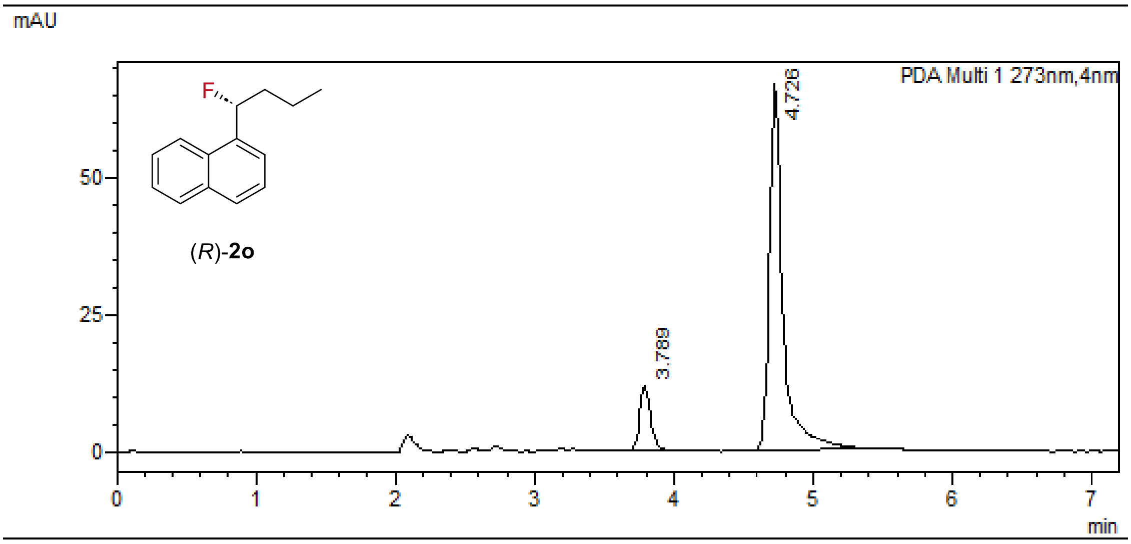


PDA Ch1 273nm

| Peak# | Ret. Time | Area% |
|---|---|---|
| 1 | 3.789 | 11.793 |
| 2 | 4.726 | 88.207 |

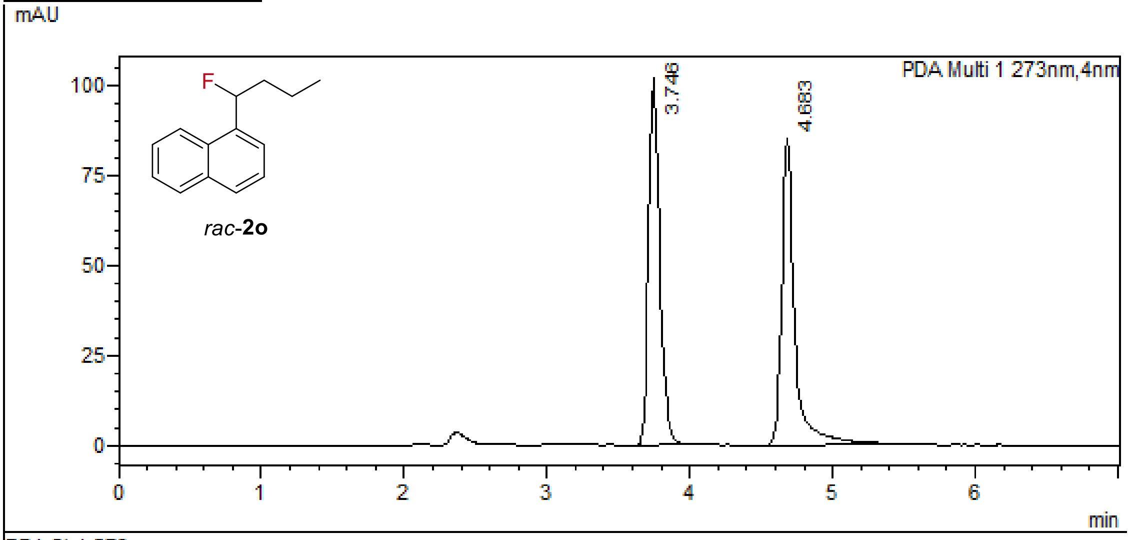


PDA Ch1 273nm

| Peak# | Ret. Time | Area% |
|---|---|---|
| 1 | 3.746 | 50.332 |
| 2 | 4.683 | 49.668 |

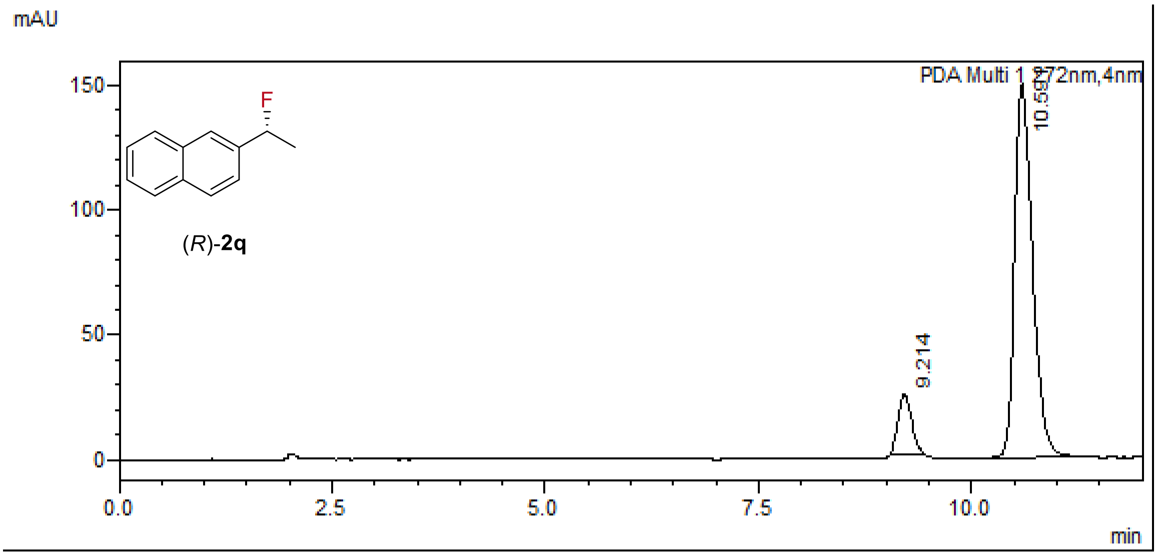


PDA Ch1 272nm

| Peak# | Ret. Time | Area% |
|---|---|---|
| 1 | 9.214 | 11.043 |
| 2 | 10.591 | 88.957 |

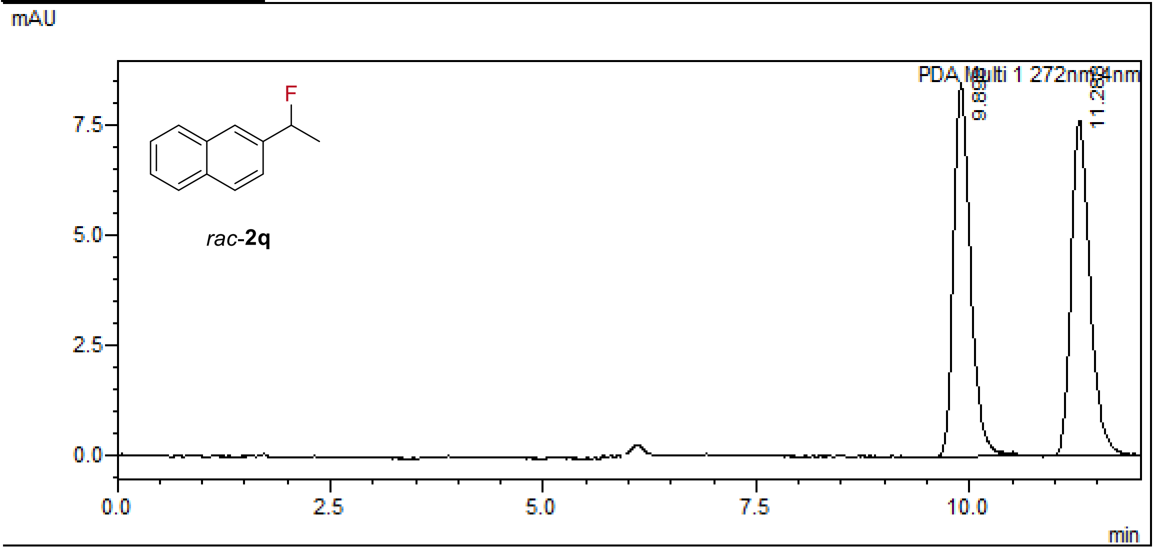


PDA Ch1 272nm

| Peak# | Ret. Time | Area% |
|---|---|---|
| 1 | 9.898 | 50.256 |
| 2 | 11.288 | 49.744 |

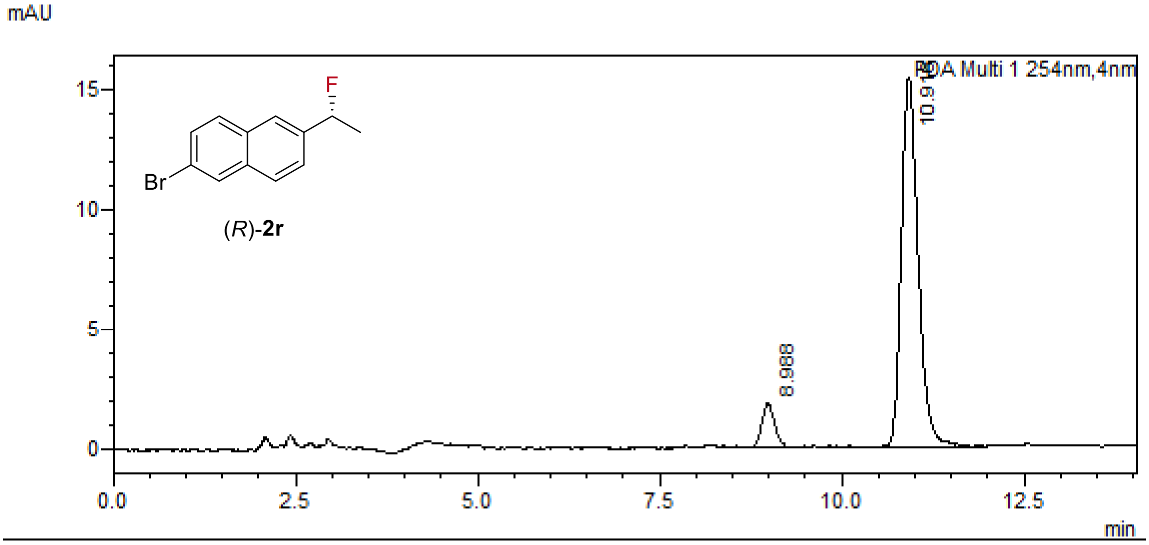


PDA Ch1 254nm

| Peak# | Ret. Time | Area% |
|---|---|---|
| 1 | 8.988 | 8.015 |
| 2 | 10.916 | 91.985 |

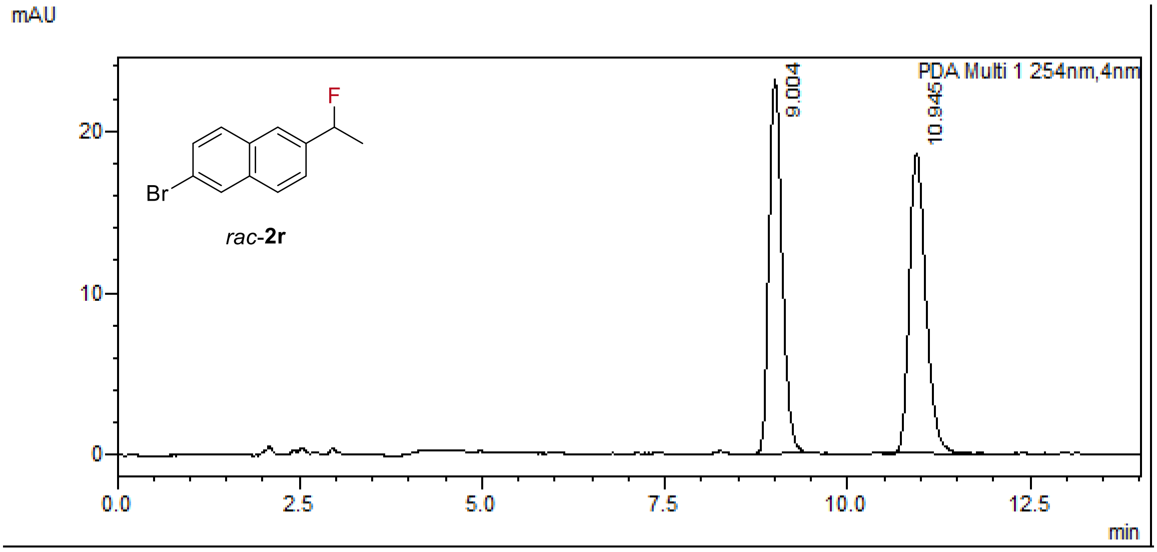


PDA Ch1 254nm

| Peak# | Ret. Time | Area% |
|---|---|---|
| 1 | 9.004 | 49.768 |
| 2 | 10.945 | 50.232 |

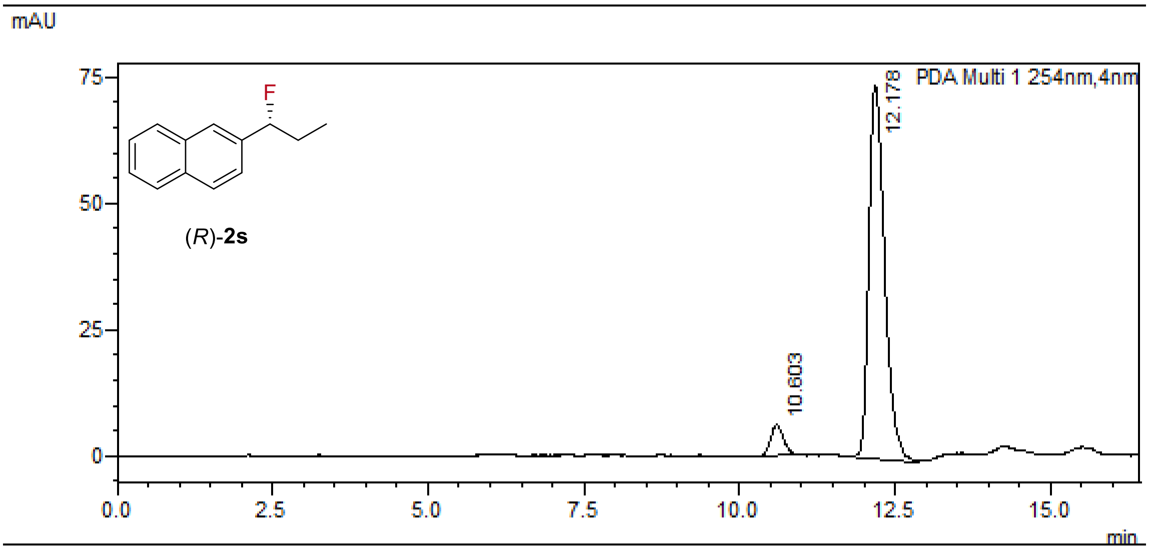


PDA Ch1 254nm

| Peak# | Ret. Time | Area% |
|---|---|---|
| 1 | 10.603 | 6.094 |
| 2 | 12.178 | 93.906 |

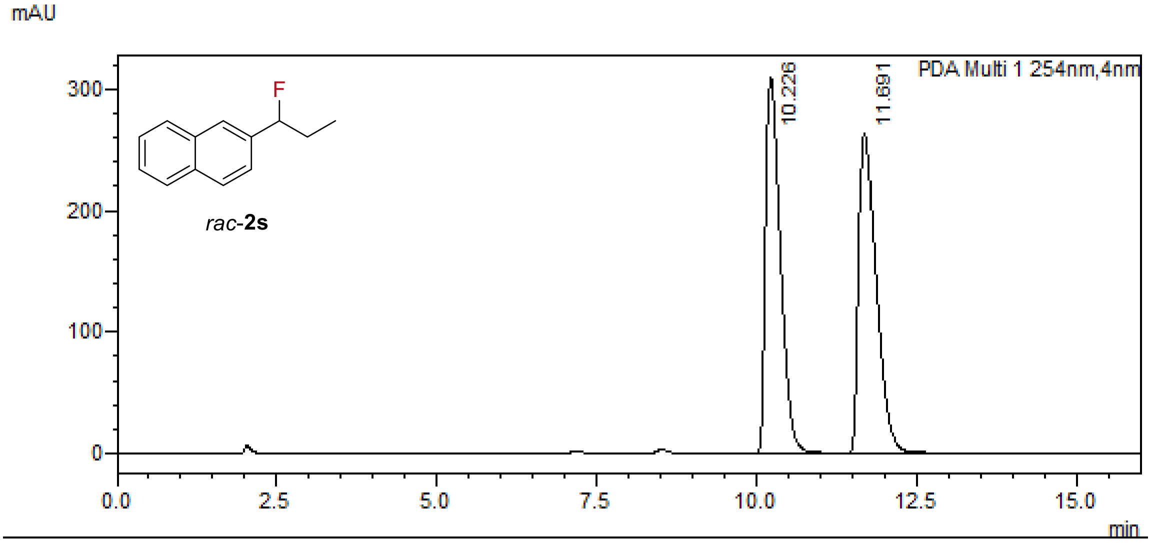


PDA Ch1 254nm

| Peak# | Ret. Time | Area% |
|---|---|---|
| 1 | 10.226 | 50.016 |
| 2 | 11.691 | 49.984 |

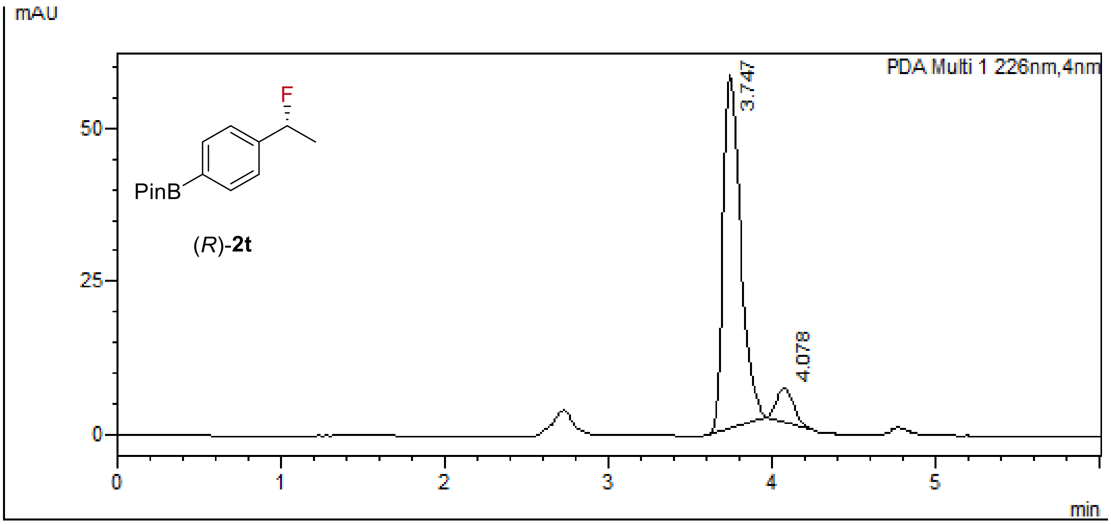


PDA Ch1 226nm

| Peak# | Ret. Time | Area% |
|---|---|---|
| 1 | 3.747 | 91.587 |
| 2 | 4.078 | 8.413 |

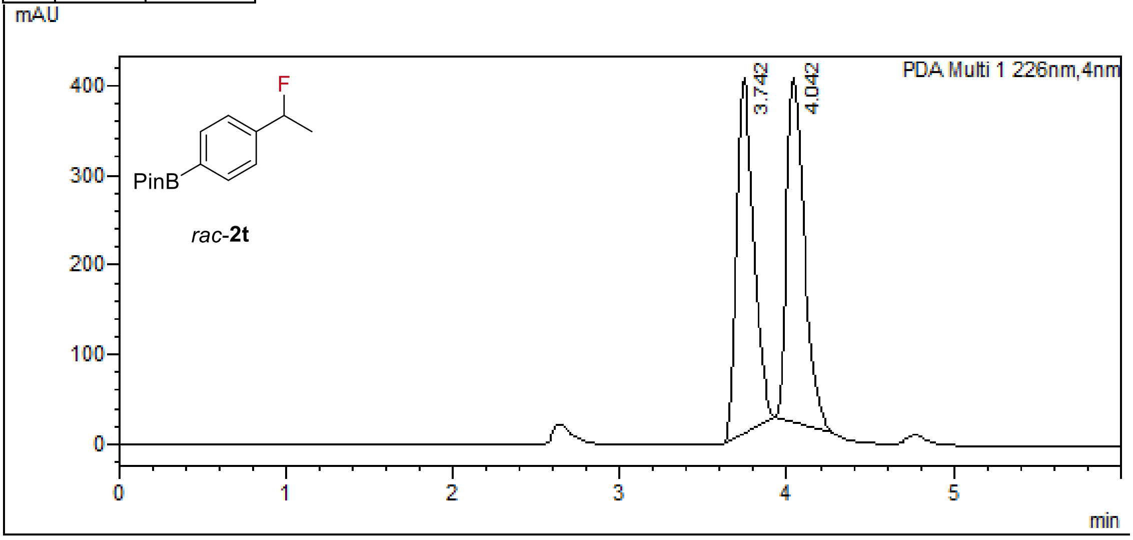


PDA Ch1 226nm

| Peak# | Ret. Time | Area% |
|---|---|---|
| 1 | 3.742 | 49.902 |
| 2 | 4.042 | 50.098 |

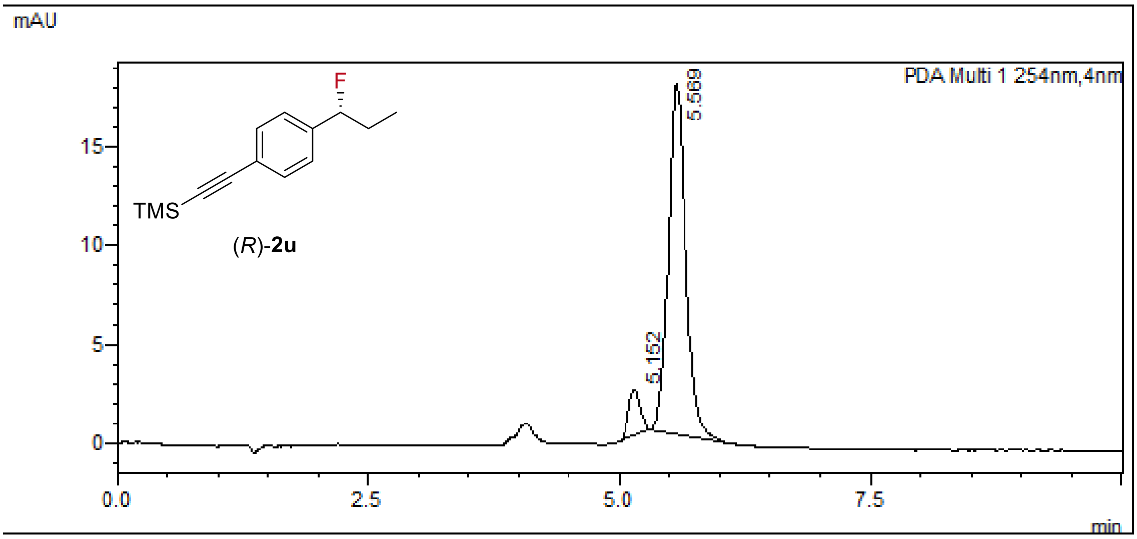


PDA Ch1 254nm

| Peak# | Ret. Time | Area% |
|---|---|---|
| 1 | 5.152 | 8.276 |
| 2 | 5.569 | 91.724 |

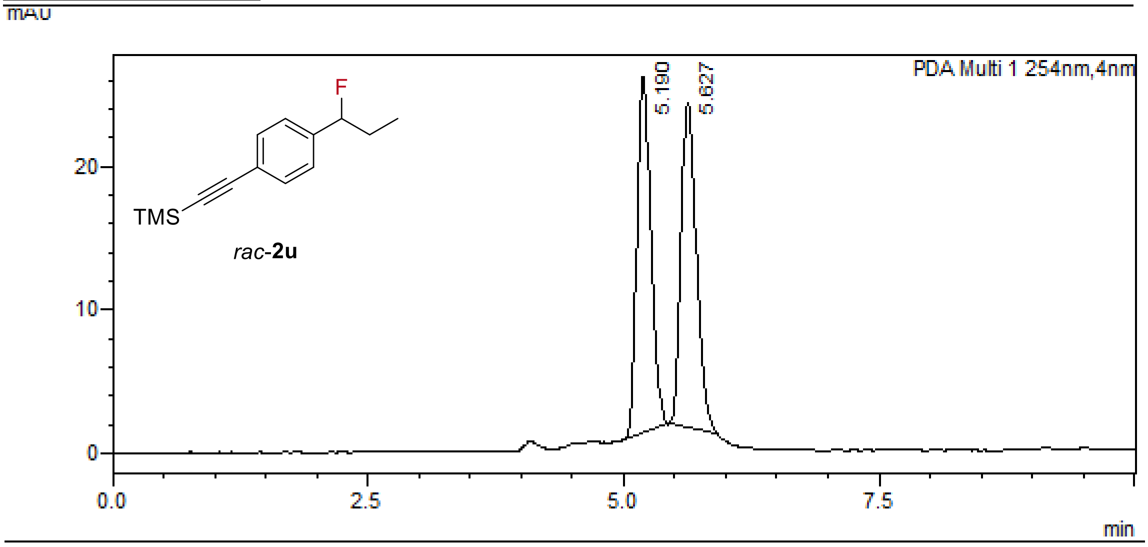


PDA Ch1 254nm

| Peak# | Ret. Time | Area% |
|---|---|---|
| 1 | 5.190 | 49.684 |
| 2 | 5.627 | 50.316 |

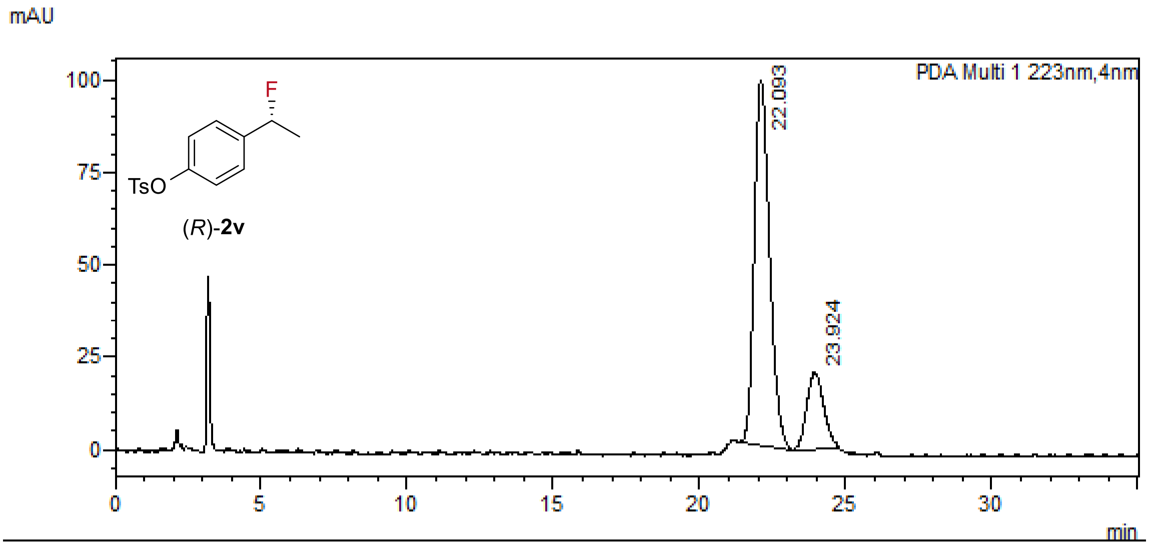


PDA Ch1 223nm

| Peak# | Ret. Time | Area% |
|---|---|---|
| 1 | 22.093 | 80.956 |
| 2 | 23.924 | 19.044 |

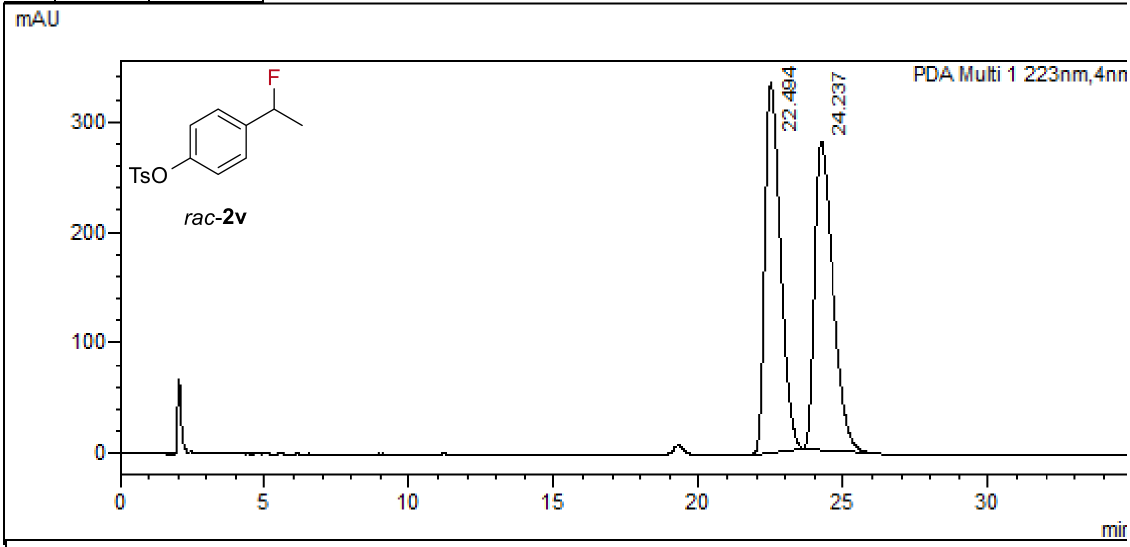


PDA Ch1 223nm

| Peak# | Ret. Time | Area% |
|---|---|---|
| 1 | 22.494 | 50.164 |
| 2 | 24.237 | 49.836 |

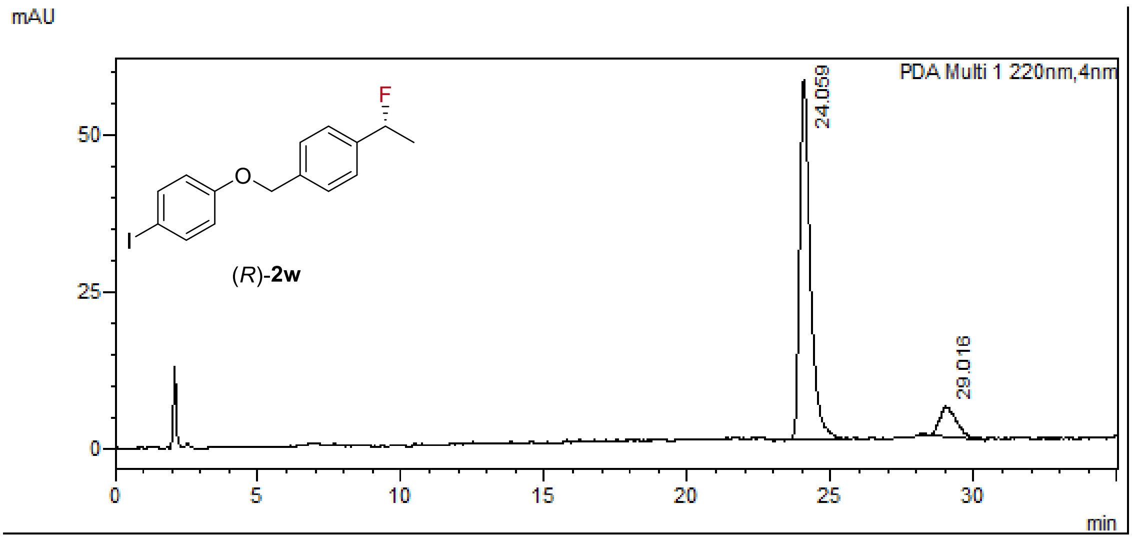


PDA Ch1 220nm

| Peak# | Ret. Time | Area% |
|---|---|---|
| 1 | 24.059 | 88.954 |
| 2 | 29.016 | 11.046 |

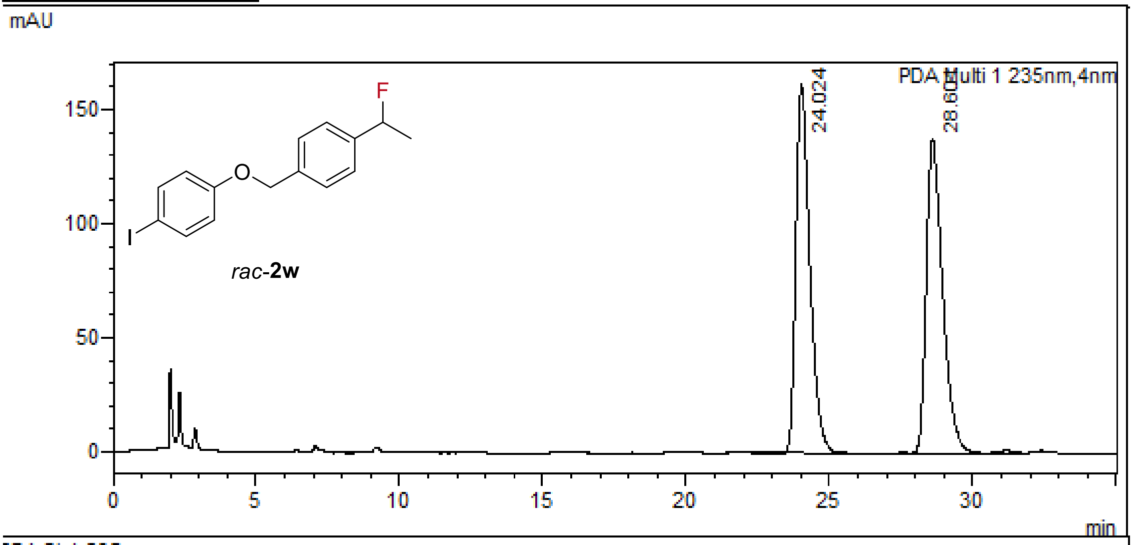


PDA Ch1 235nm

| Peak# | Ret. Time | Area% |
|---|---|---|
| 1 | 24.024 | 49.823 |
| 2 | 28.601 | 50.177 |

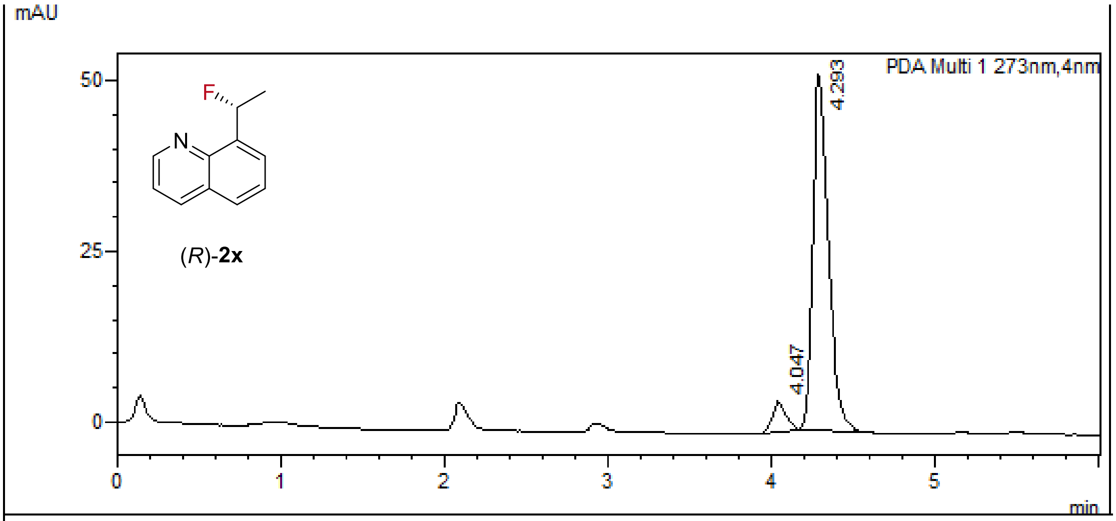


PDA Ch1 273nm

| Peak# | Ret. Time | Area% |
|---|---|---|
| 1 | 4.047 | 7.115 |
| 2 | 4.293 | 92.885 |

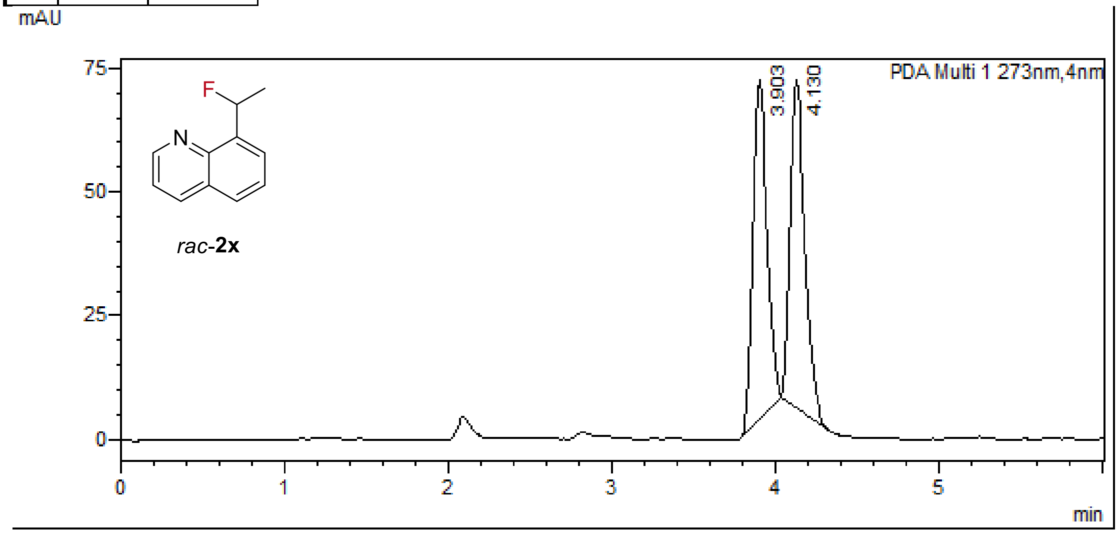


PDA Ch1 273nm

| Peak# | Ret. Time | Area% |
|---|---|---|
| 1 | 3.903 | 49.826 |
| 2 | 4.130 | 50.174 |

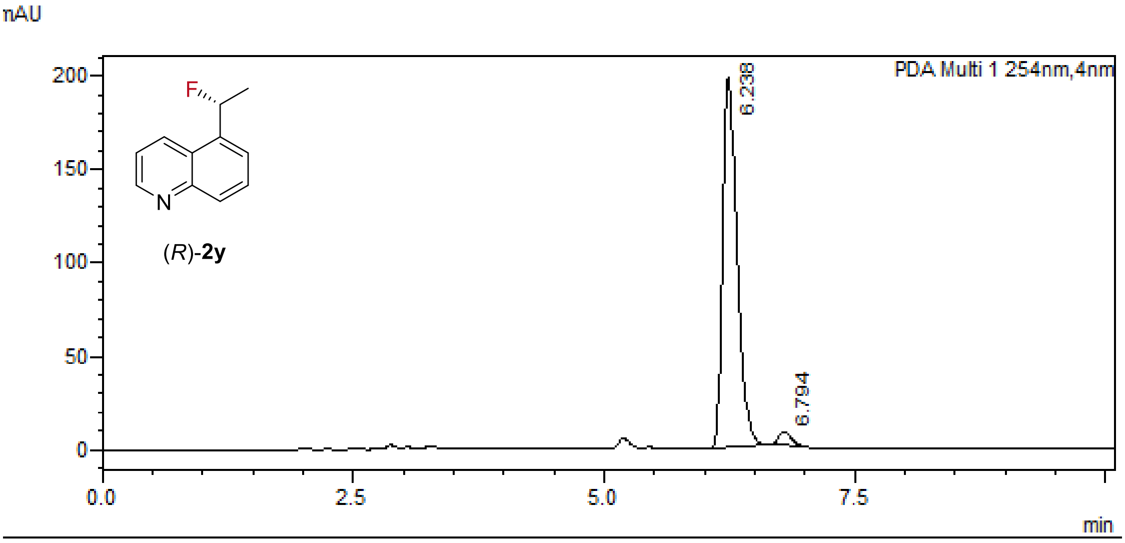


PDA Ch1 254nm

| Peak# | Ret. Time | Area% |
|---|---|---|
| 1 | 6.238 | 96.785 |
| 2 | 6.794 | 3.215 |

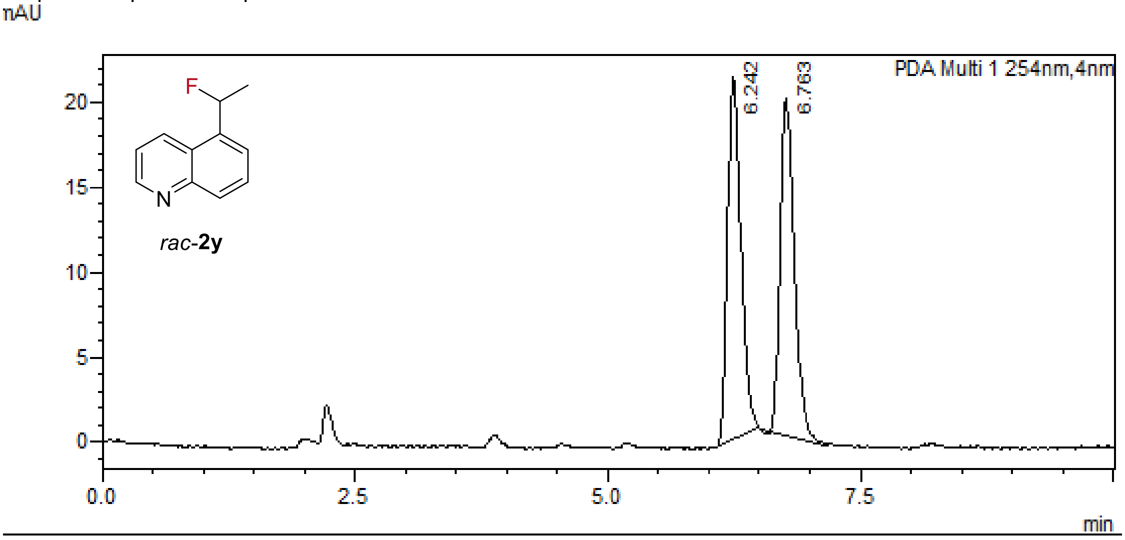


PDA Ch1 254nm

| Peak# | Ret. Time | Area% |
|---|---|---|
| 1 | 6.242 | 50.391 |
| 2 | 6.763 | 49.609 |

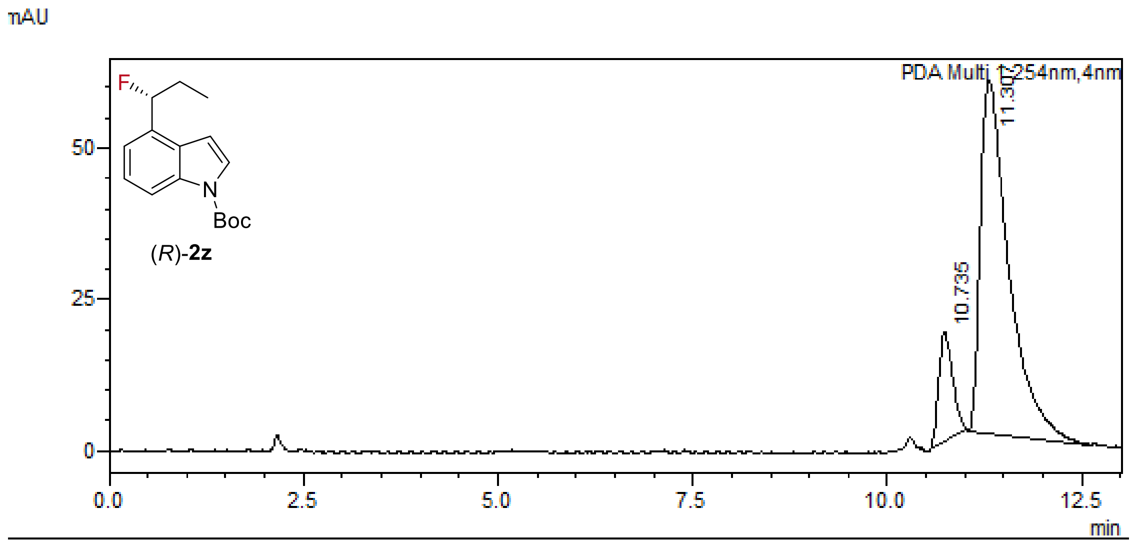


DA Ch1 254nm

| eak# | Ret. Time | Area% |
|---|---|---|
| 1 | 10.735 | 13.248 |
| 2 | 11.307 | 86.752 |

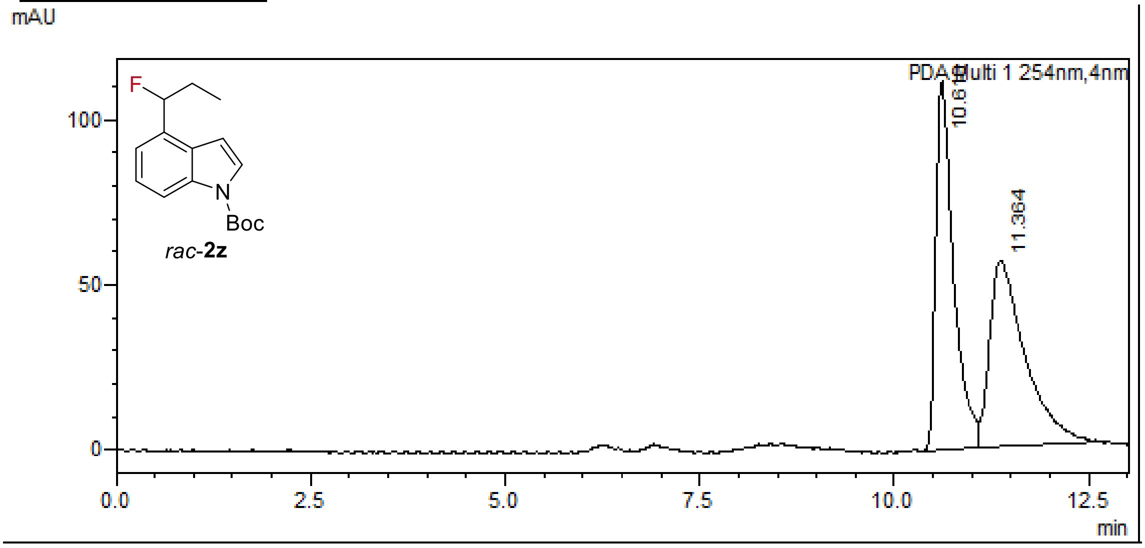


PDA Ch1 254nm

| Peak# | Ret. Time | Area% |
|---|---|---|
| 1 | 10.610 | 49.652 |
| 2 | 11.364 | 50.348 |

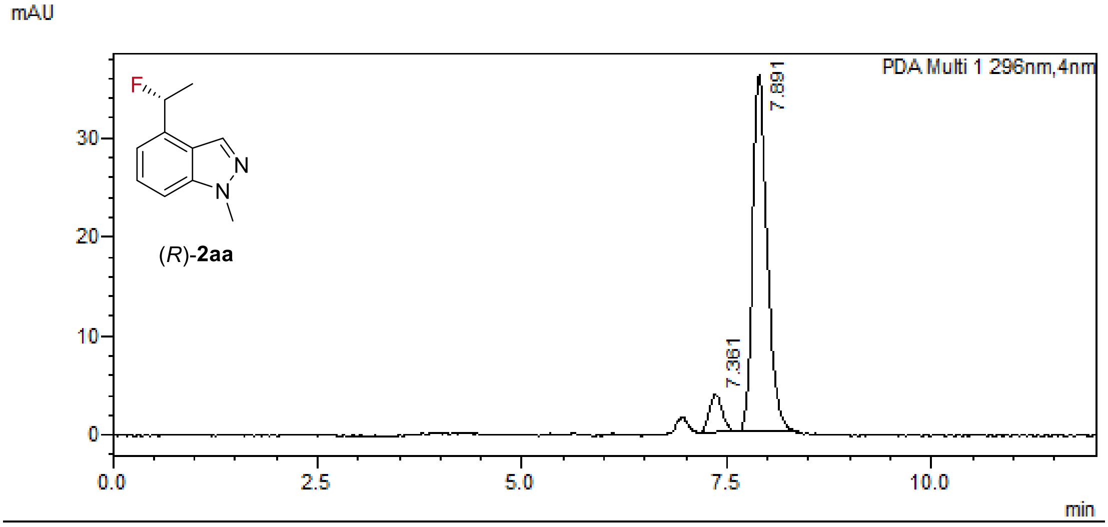


PDA Ch1 296nm

| Peak# | Ret. Time | Area% |
|---|---|---|
| 1 | 7.361 | 8.496 |
| 2 | 7.891 | 91.504 |

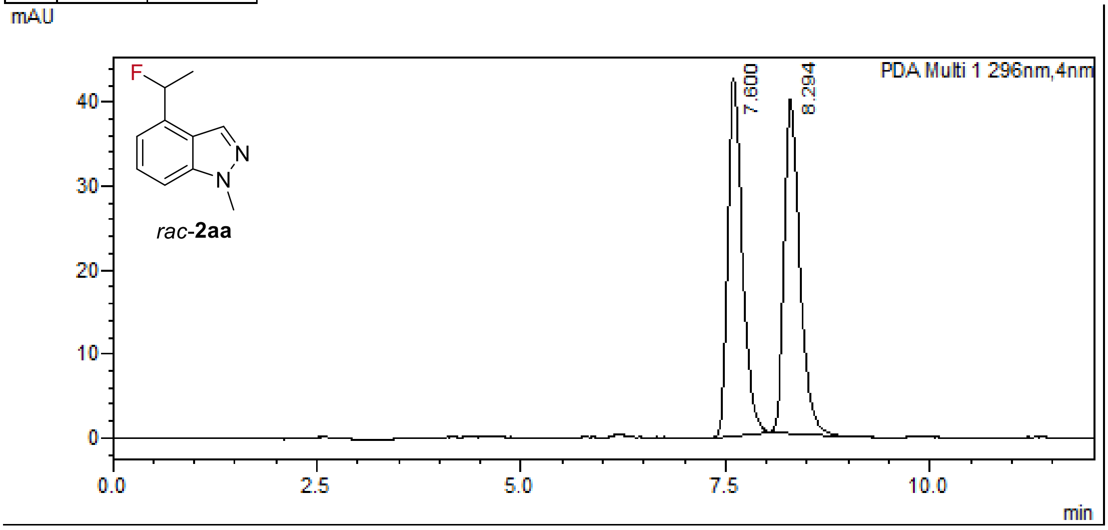


PDA Ch1 296nm

| Peak# | Ret. Time | Area% |
|---|---|---|
| 1 | 7.600 | 49.856 |
| 2 | 8.294 | 50.144 |

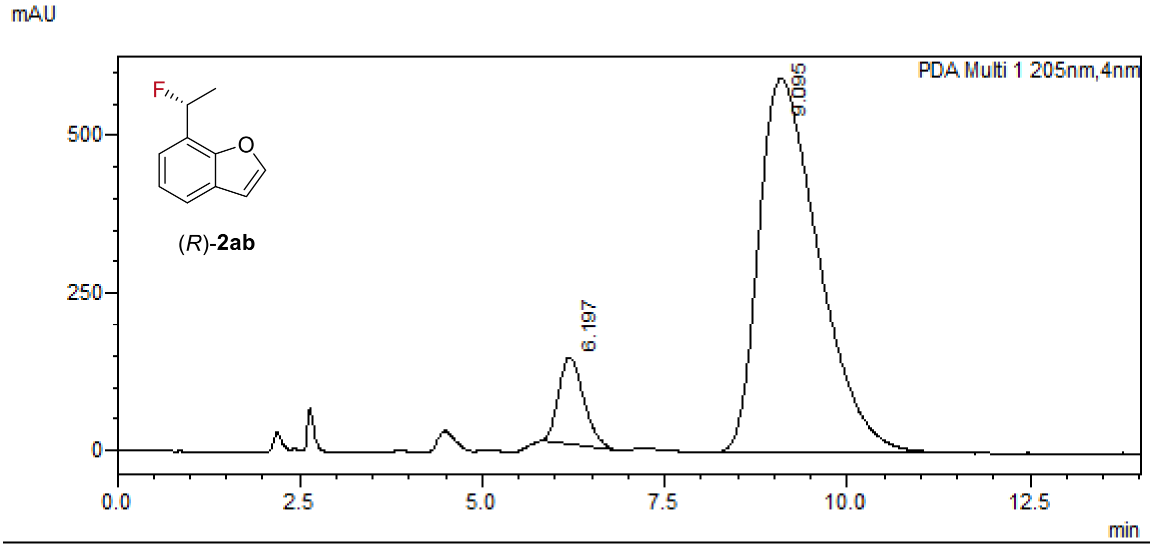


PDA Ch1 205nm

| Peak# | Ret. Time | Area% |
|---|---|---|
| 1 | 6.197 | 8.979 |
| 2 | 9.095 | 91.021 |

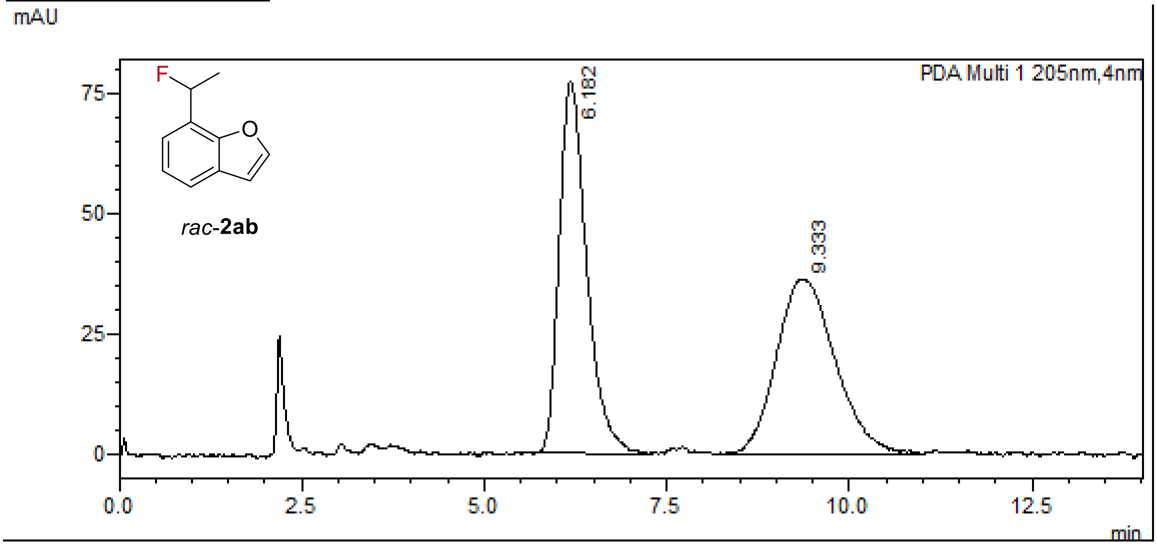


PDA Ch1 205nm

| Peak# | Ret. Time | Area% |
|---|---|---|
| 1 | 6.182 | 49.830 |
| 2 | 9.333 | 50.170 |

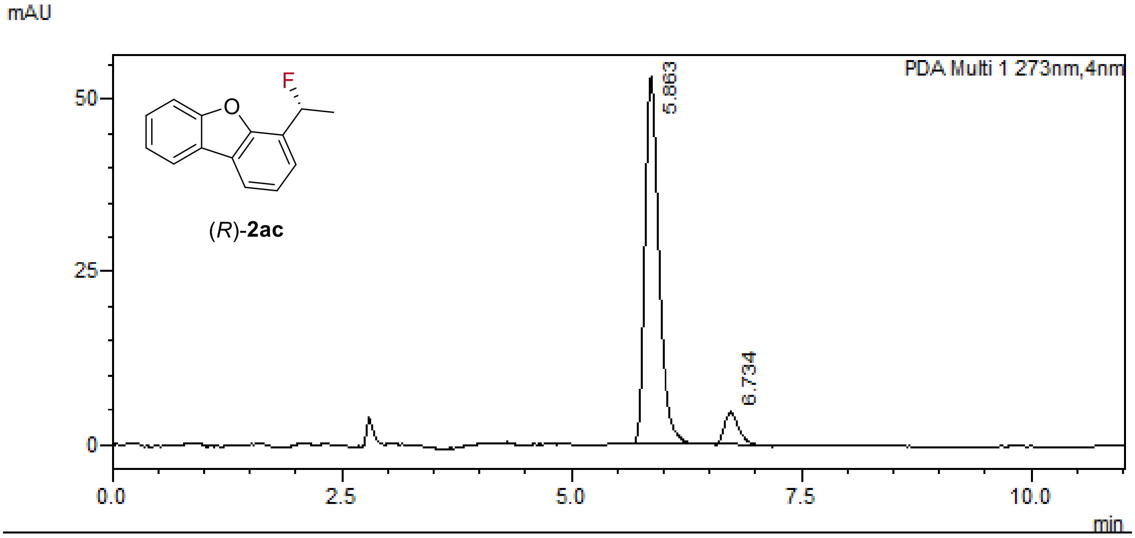


PDA Ch1 273nm

| Peak# | Ret. Time | Area% |
|---|---|---|
| 1 | 5.863 | 91.952 |
| 2 | 6.734 | 8.048 |

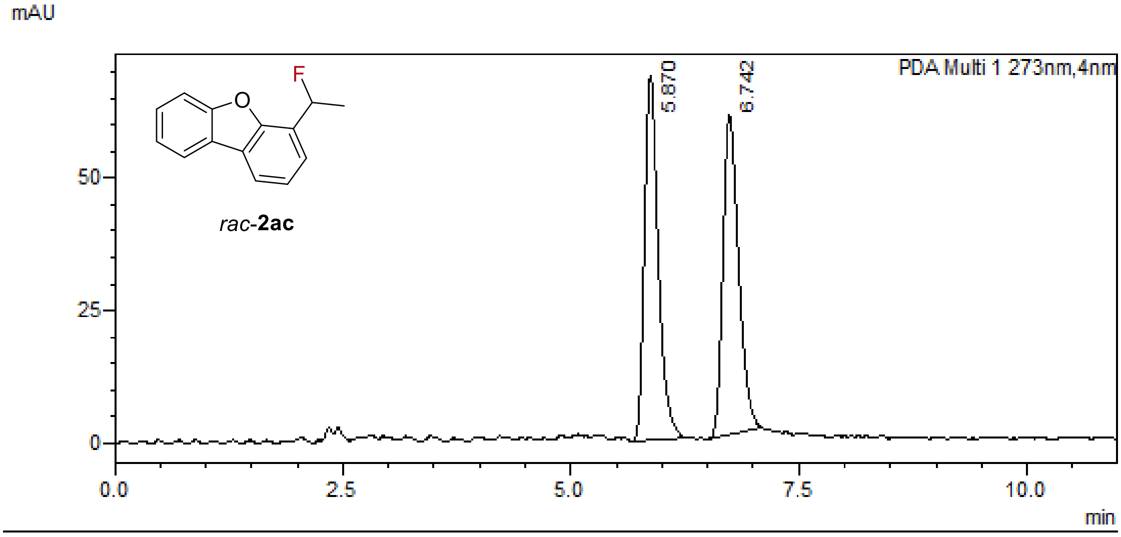


PDA Ch1 273nm

| Peak# | Ret. Time | Area% |
|---|---|---|
| 1 | 5.870 | 50.090 |
| 2 | 6.742 | 49.910 |

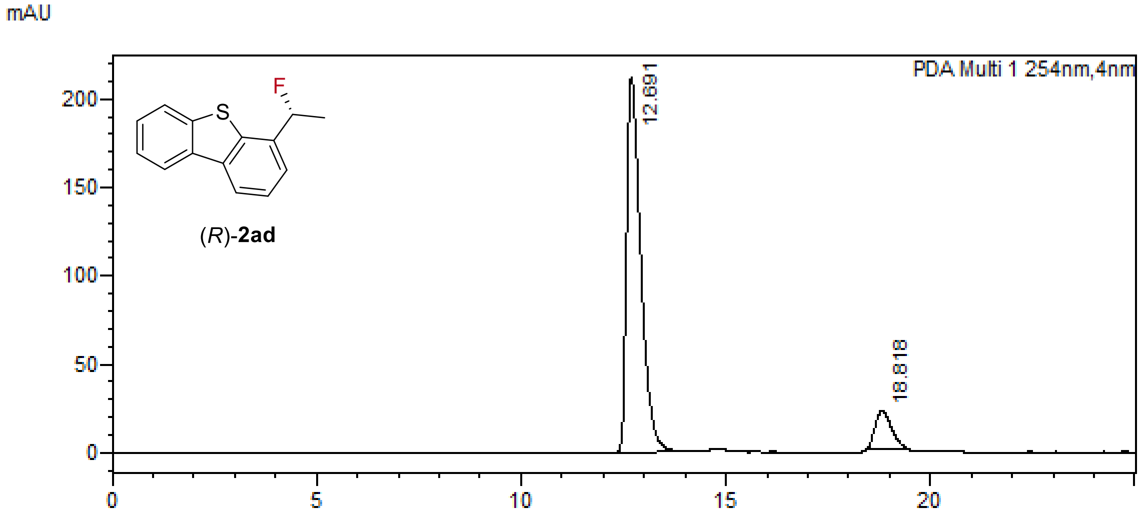


PDA Ch1 254nm

| Peak# | Ret. Time | Area% |
|---|---|---|
| 1 | 12.691 | 89.034 |
| 2 | 18.818 | 10.966 |

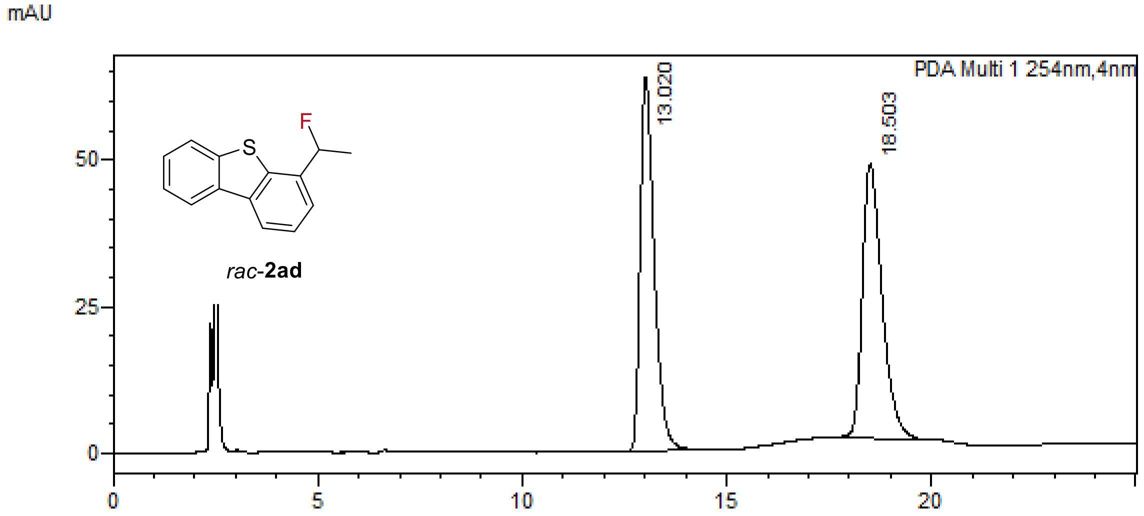


PDA Ch1 254nm

| Peak# | Ret. Time | Area% |
|---|---|---|
| 1 | 13.020 | 49.625 |
| 2 | 18.503 | 50.375 |

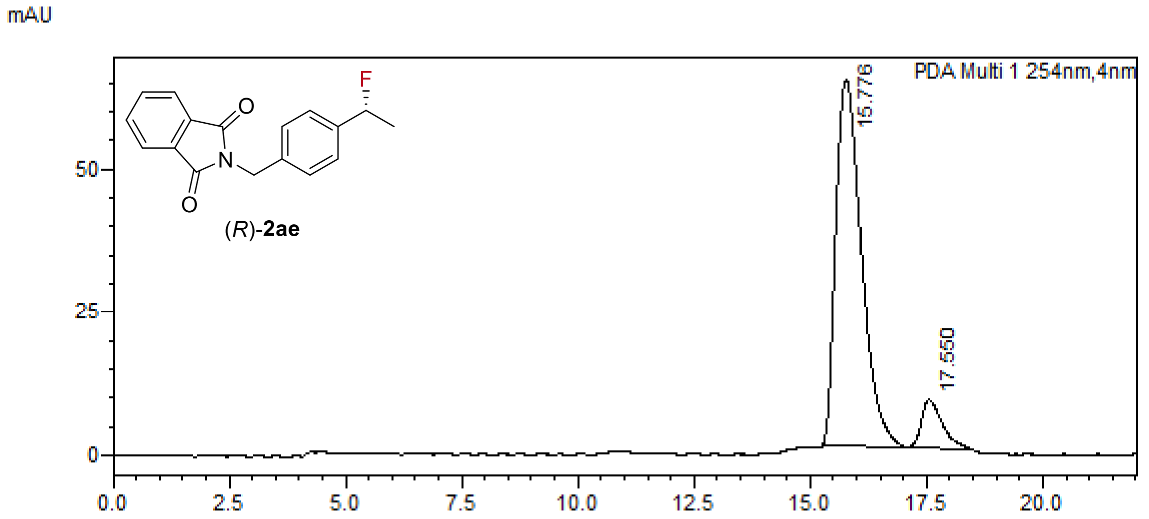


PDA Ch1 254nm

| Peak# | Ret. Time | Area% |
|---|---|---|
| 1 | 15.776 | 90.014 |
| 2 | 17.550 | 9.986 |

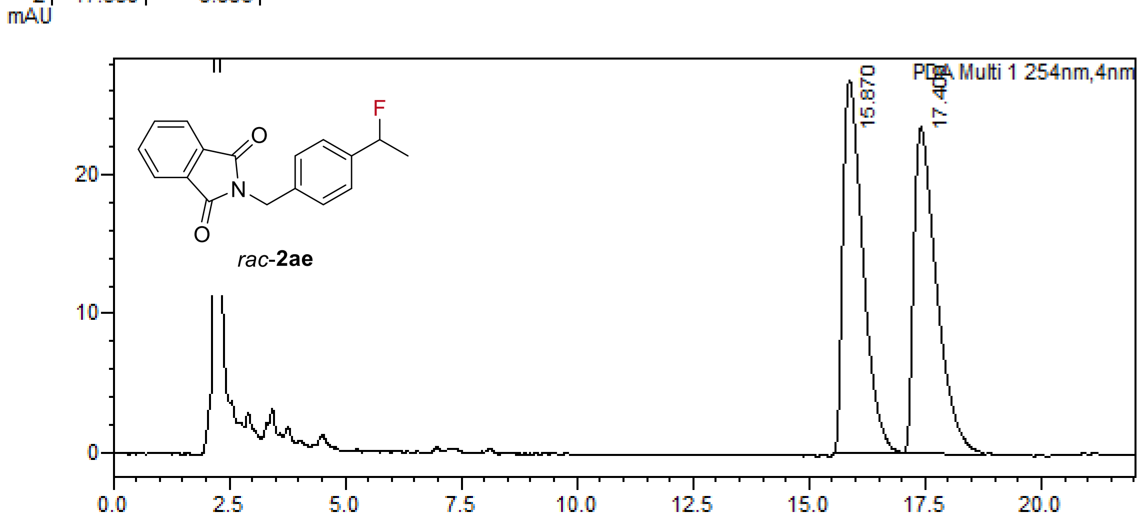


PDA Ch1 254nm

| Peak# | Ret. Time | Area% |
|---|---|---|
| 1 | 15.870 | 49.816 |
| 2 | 17.409 | 50.184 |

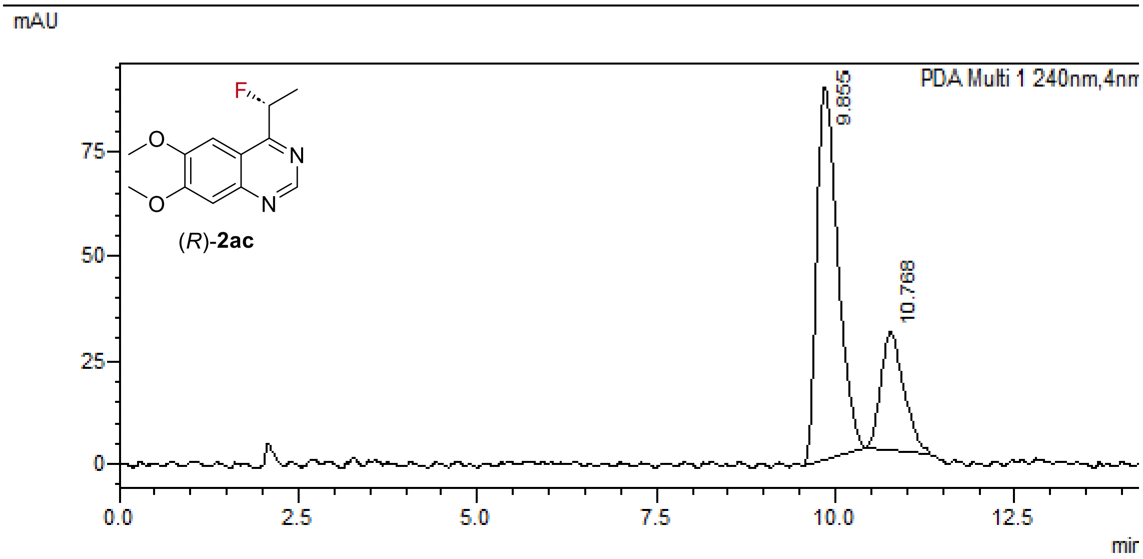


PDA Ch1 240nm

| Peak# | Ret. Time | Area% |
|---|---|---|
| 1 | 9.855 | 78.256 |
| 2 | 10.768 | 21.744 |

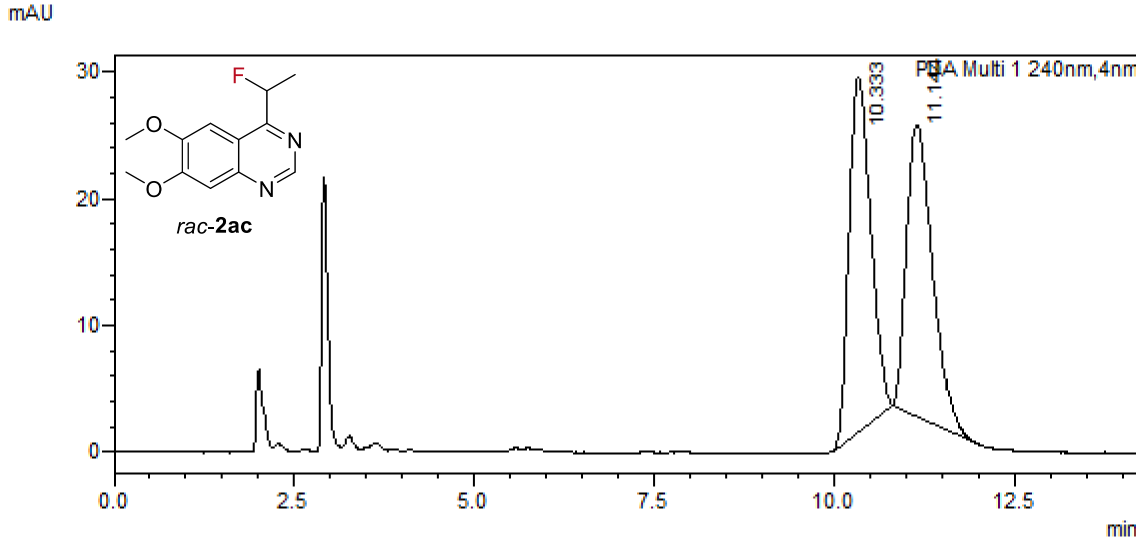


PDA Ch1 240nm

| Peak# | Ret. Time | Area% |
|---|---|---|
| 1 | 10.333 | 50.339 |
| 2 | 11.144 | 49.661 |

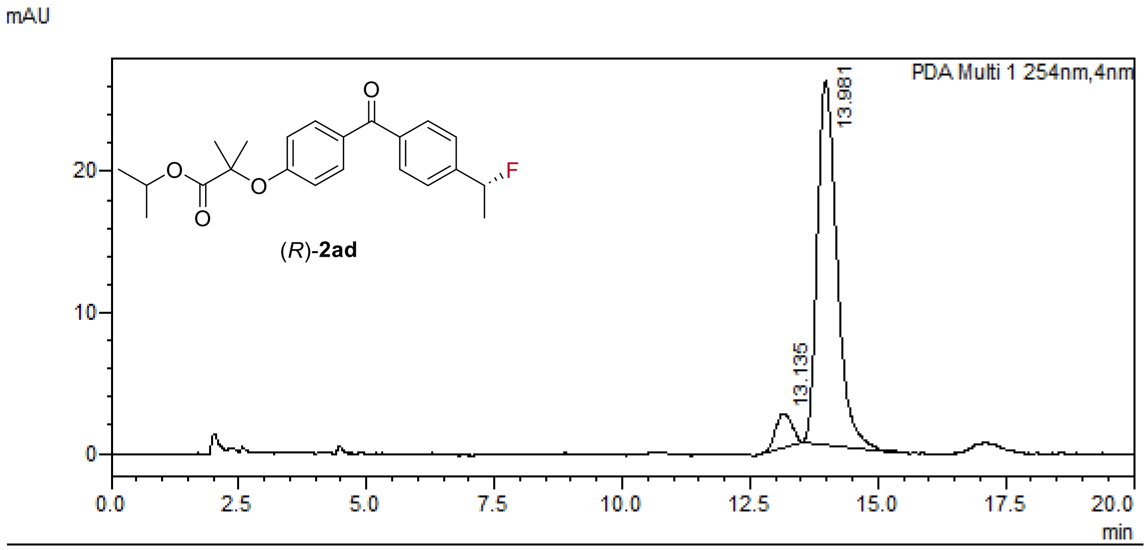


| DA Ch1 254nm | | |
|---|---|---|
| eak# | Ret. Time | Area% |
| 1 | 13.135 | 7.140 |
| 2 | 13.981 | 92.860 |

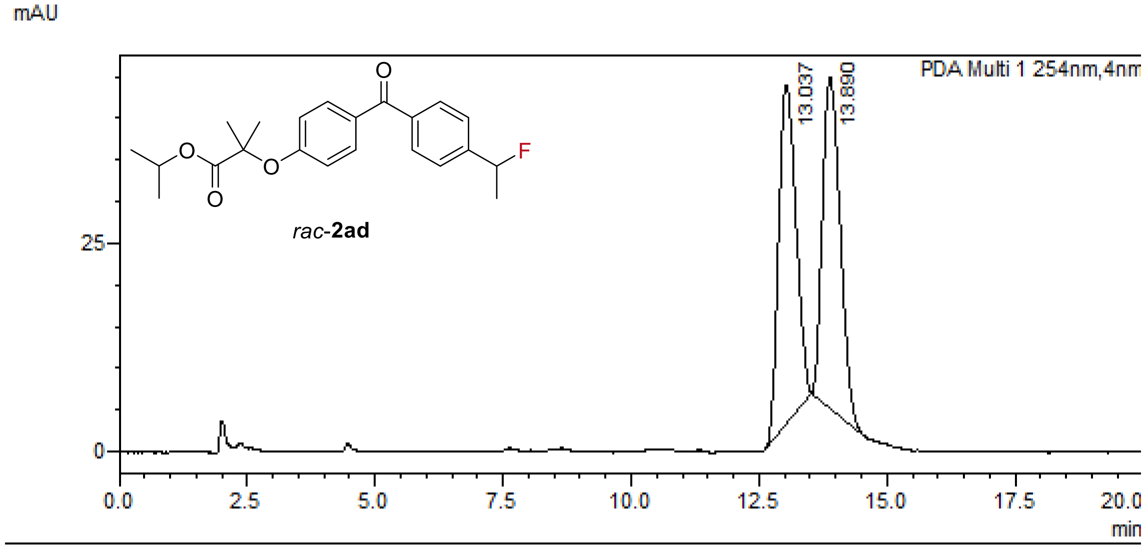


| PDA Ch1 254nm | | |
|---|---|---|
| Peak# | Ret. Time | Area% |
| 1 | 13.037 | 50.167 |
| 2 | 13.890 | 49.833 |

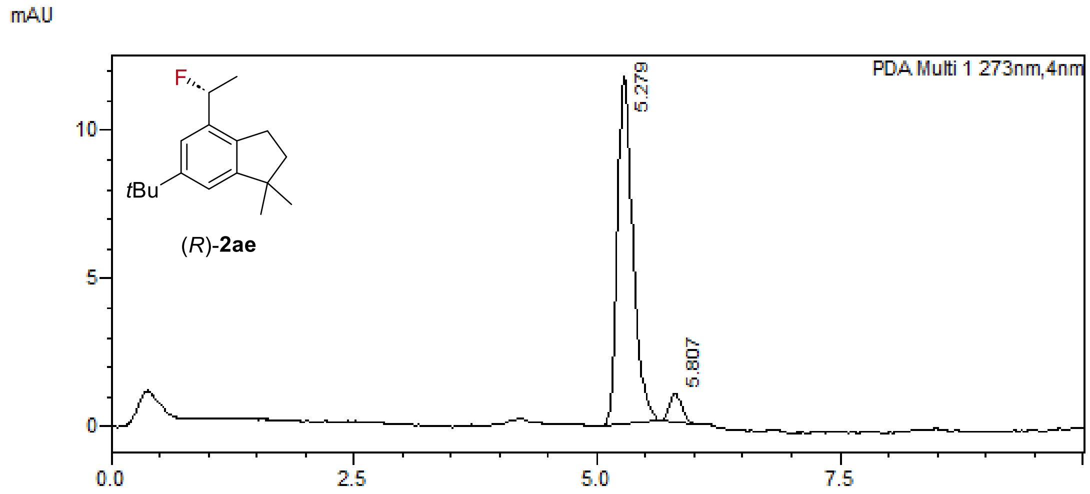


PDA Ch1 273nm

| Peak# | Ret. Time | Area% |
|---|---|---|
| 1 | 5.279 | 93.565 |
| 2 | 5.807 | 6.435 |

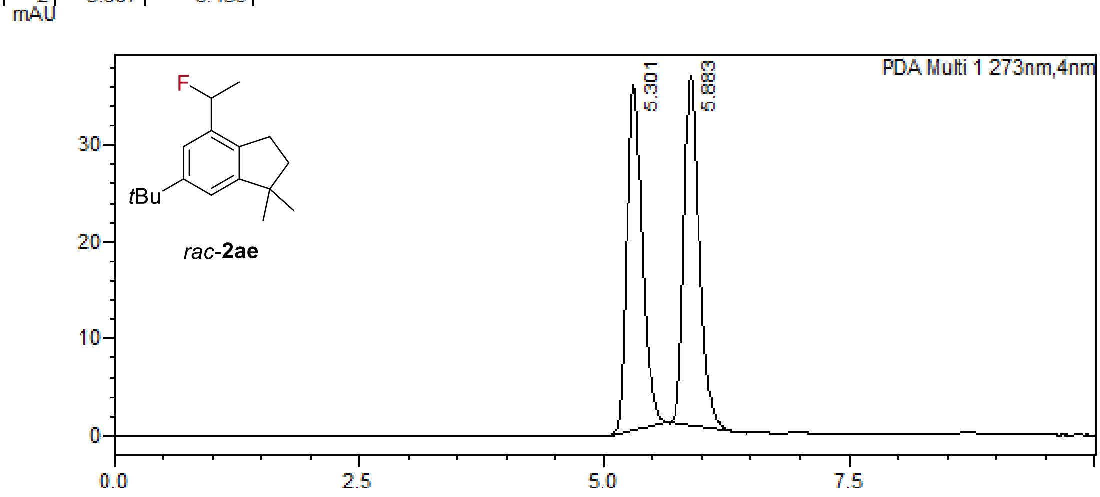


PDA Ch1 273nm

| Peak# | Ret. Time | Area% |
|---|---|---|
| 1 | 5.301 | 49.673 |
| 2 | 5.883 | 50.327 |

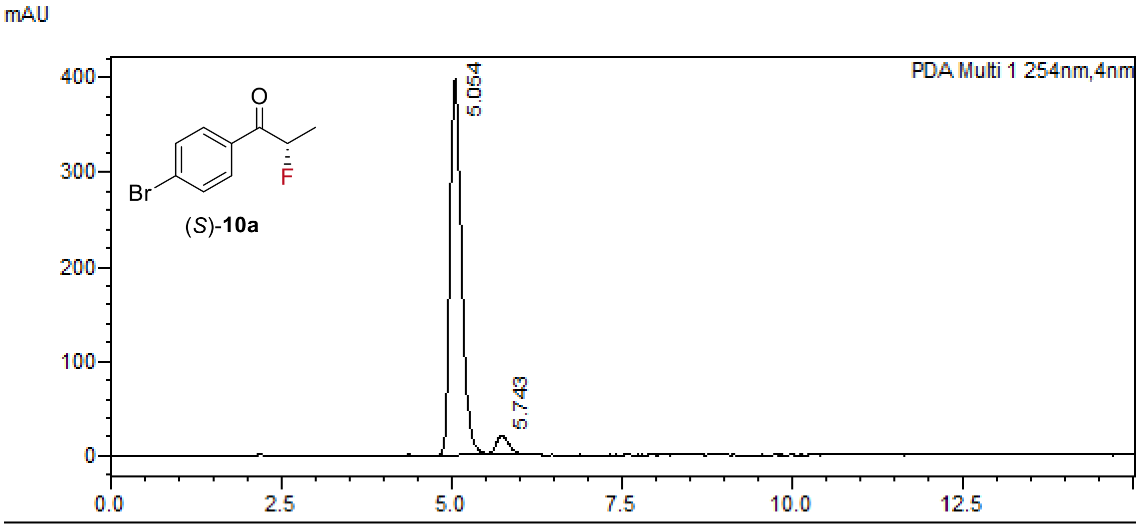


DA Ch1 254nm

| eak# | Ret. Time | Area% |
|---|---|---|
| 1 | 5.054 | 95.093 |
| 2 | 5.743 | 4.907 |

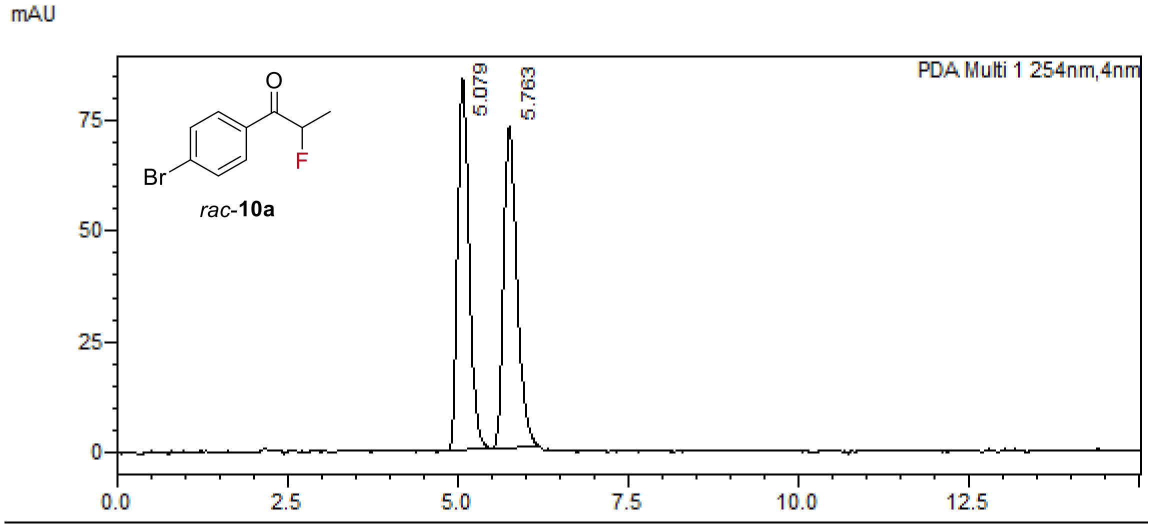


PDA Ch1 254nm

| Peak# | Ret. Time | Area% |
|---|---|---|
| 1 | 5.079 | 49.888 |
| 2 | 5.763 | 50.112 |

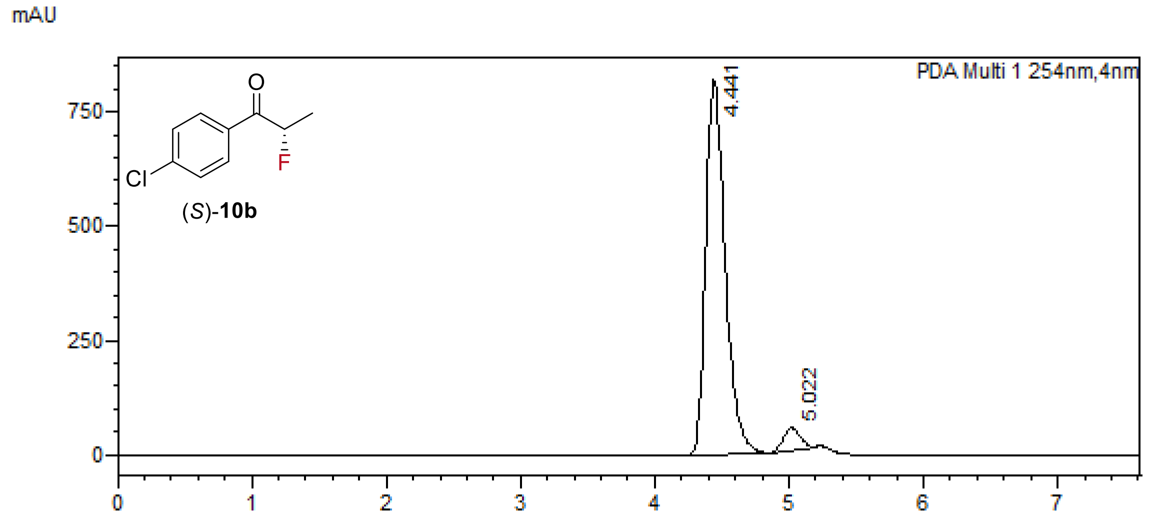


PDA Ch1 254nm

| Peak# | Ret. Time | Area% |
|---|---|---|
| 1 | 4.441 | 95.127 |
| 2 | 5.022 | 4.873 |

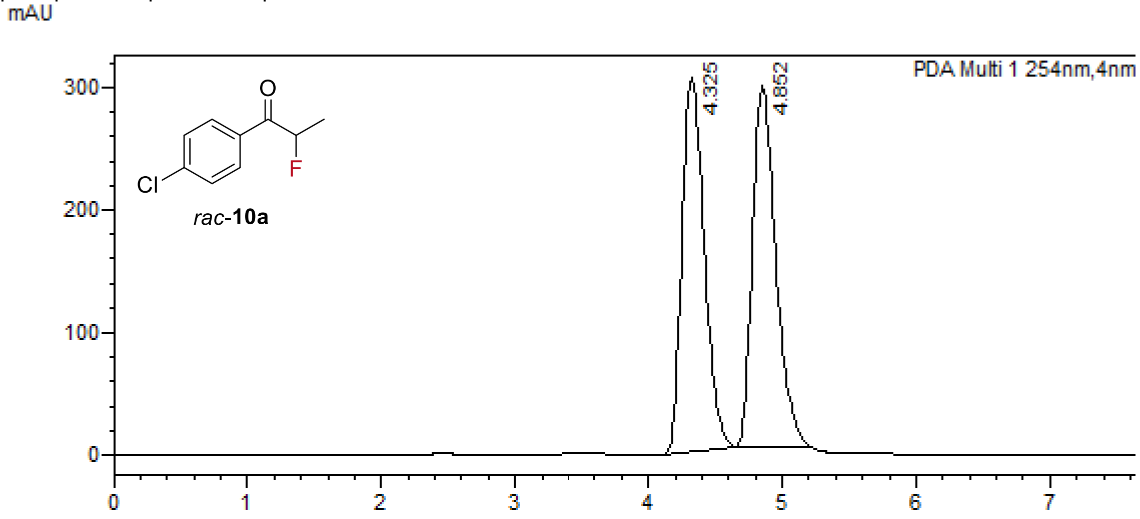


PDA Ch1 254nm

| Peak# | Ret. Time | Area% |
|---|---|---|
| 1 | 4.325 | 49.707 |
| 2 | 4.852 | 50.293 |

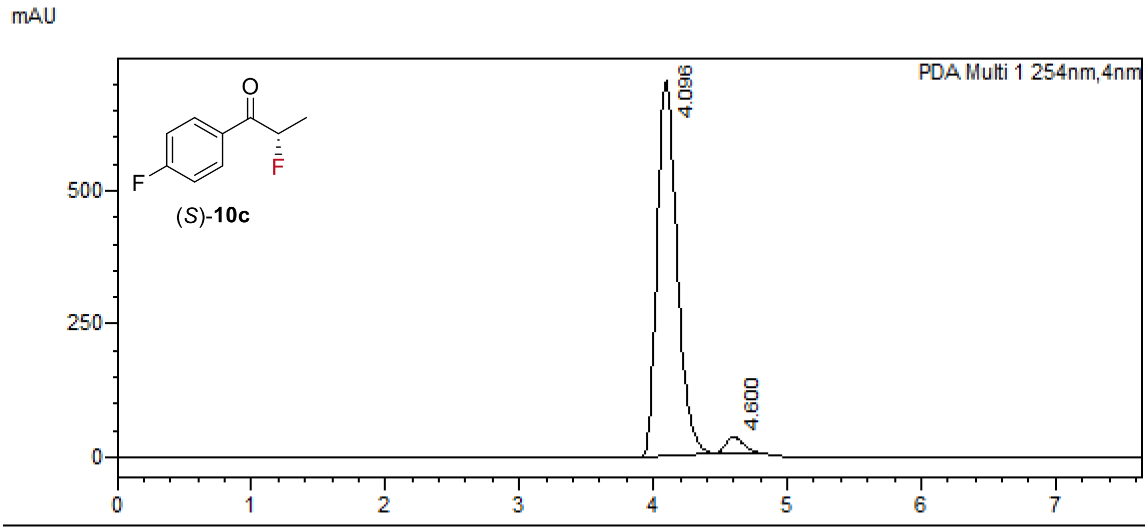


PDA Ch1 254nm

| Peak# | Ret. Time | Area% |
|---|---|---|
| 1 | 4.096 | 96.183 |
| 2 | 4.600 | 3.817 |

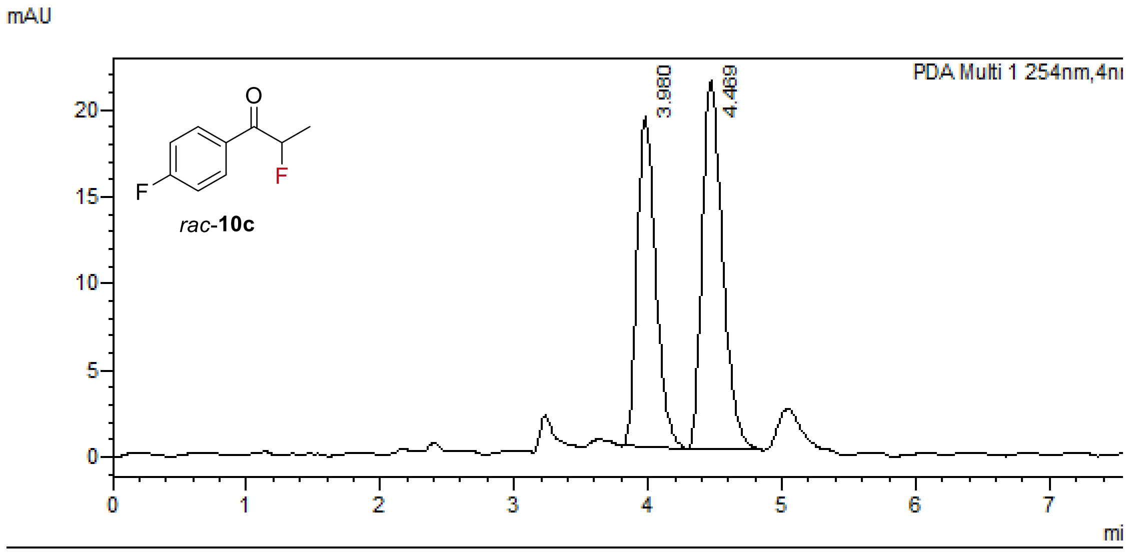


PDA Ch1 254nm

| Peak# | Ret. Time | Area% |
|---|---|---|
| 1 | 3.980 | 49.969 |
| 2 | 4.469 | 50.031 |

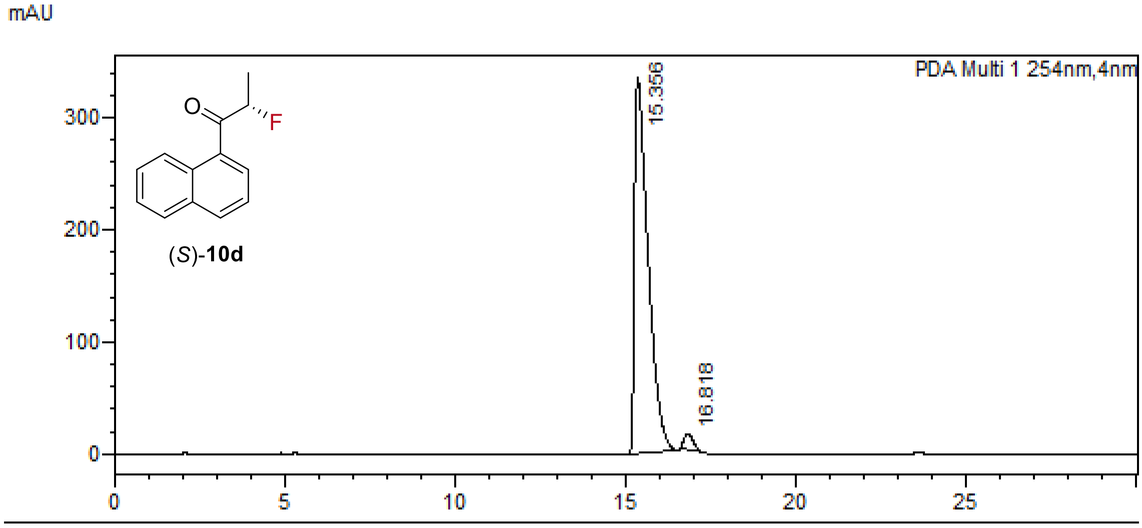


PDA Ch1 254nm

| Peak# | Ret. Time | Area% |
|---|---|---|
| 1 | 15.356 | 98.028 |
| 2 | 16.818 | 1.972 |

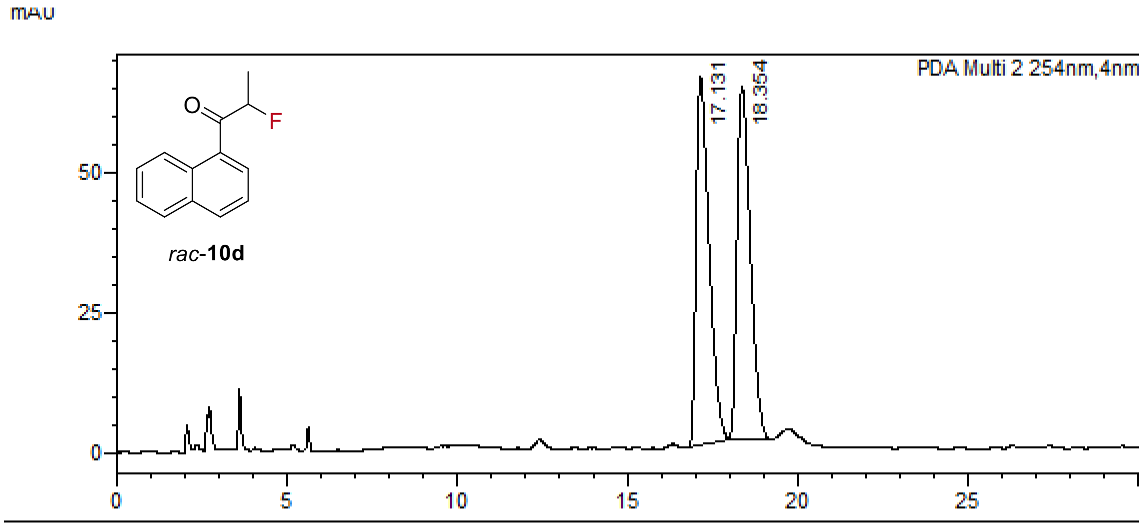


PDA Ch2 254nm

| Peak# | Ret. Time | Area% |
|---|---|---|
| 1 | 17.131 | 50.451 |
| 2 | 18.354 | 49.549 |

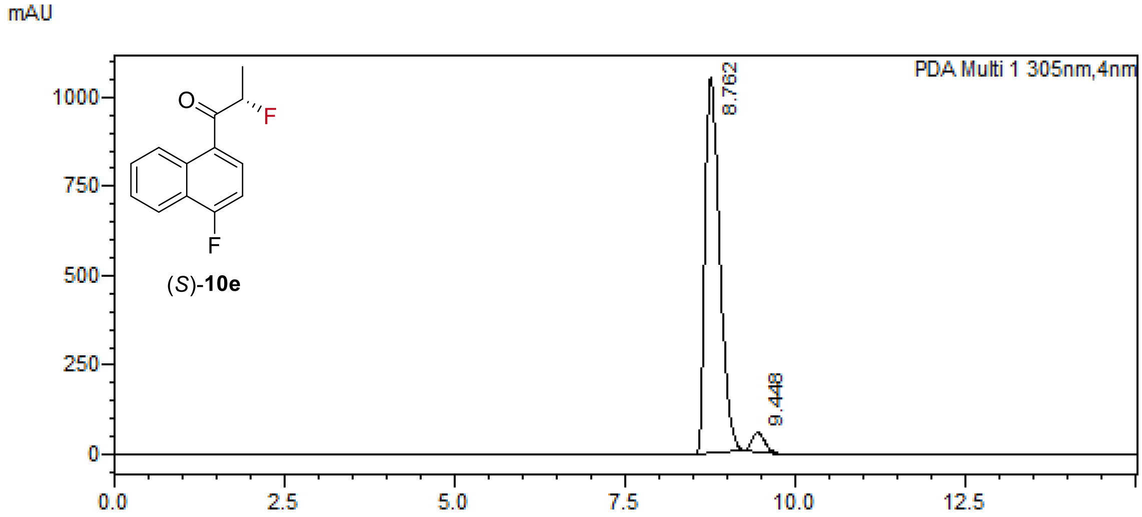


PDA Ch1 305nm

| Peak# | Ret. Time | Area% |
|---|---|---|
| 1 | 8.762 | 95.927 |
| 2 | 9.448 | 4.073 |

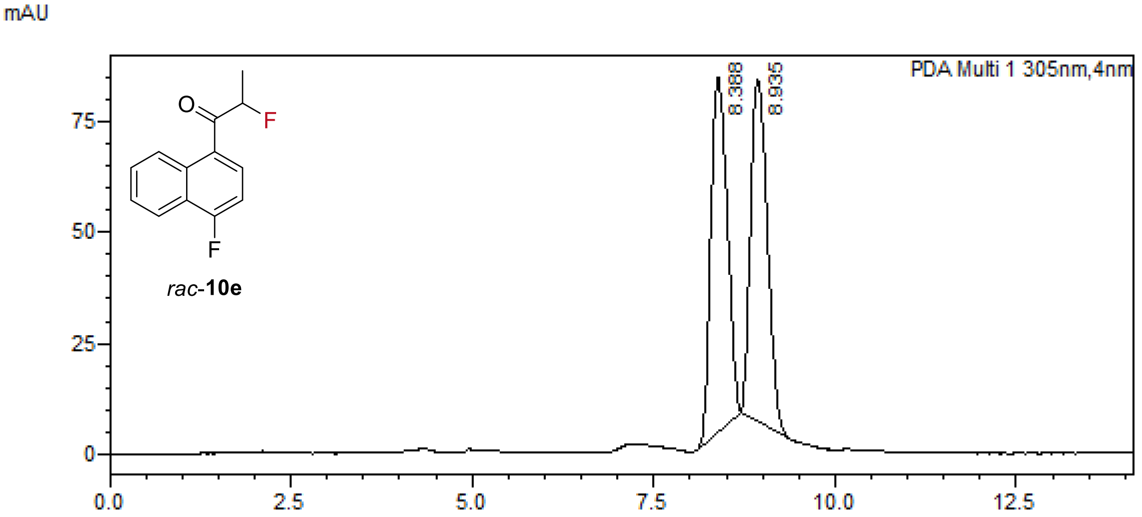


PDA Ch1 305nm

| Peak# | Ret. Time | Area% |
|---|---|---|
| 1 | 8.388 | 50.314 |
| 2 | 8.935 | 49.686 |

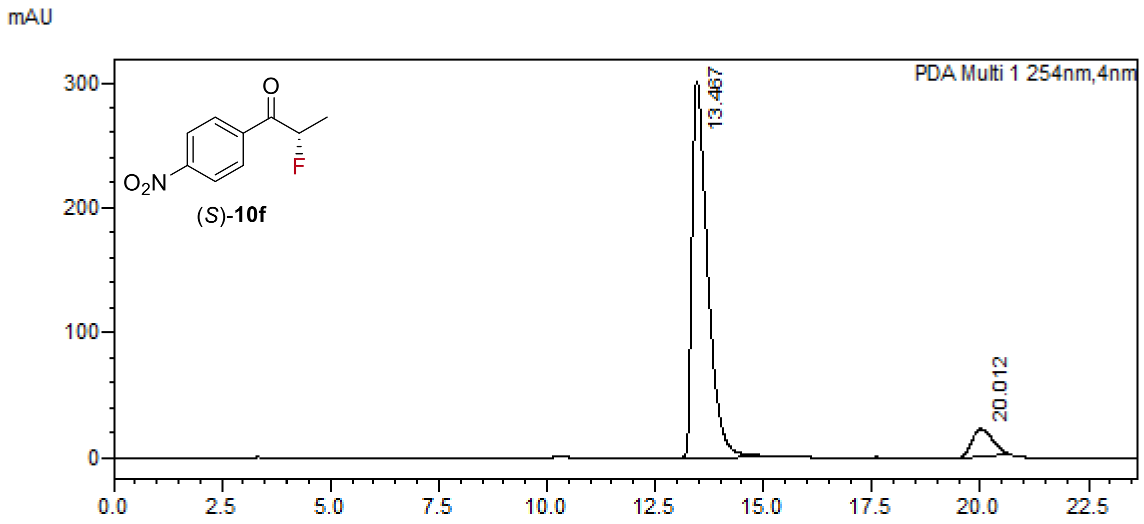


PDA Ch1 254nm

| Peak# | Ret. Time | Area% |
|---|---|---|
| 1 | 13.467 | 91.695 |
| 2 | 20.012 | 8.305 |

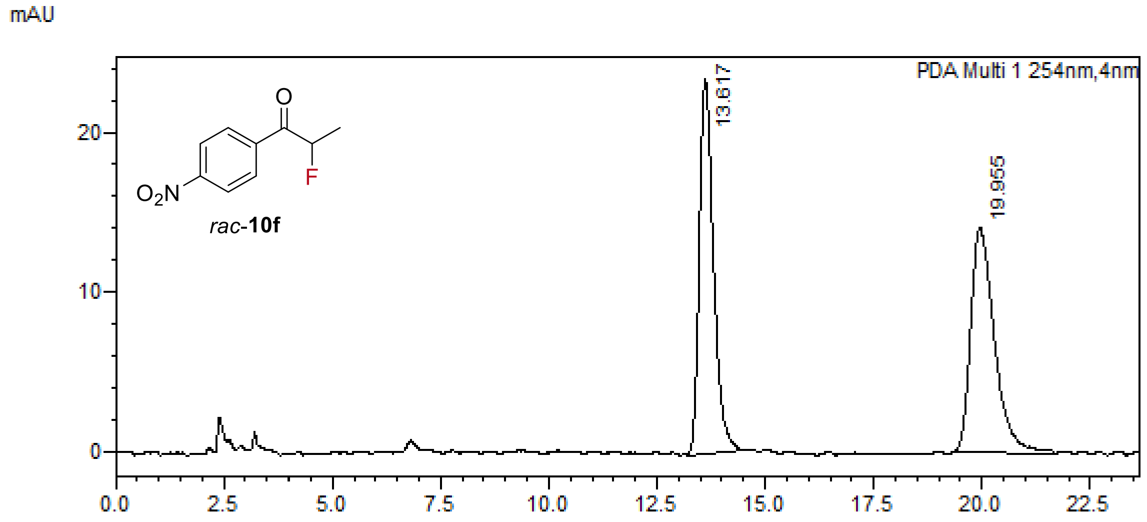


PDA Ch1 254nm

| Peak# | Ret. Time | Area% |
|---|---|---|
| 1 | 13.617 | 49.835 |
| 2 | 19.955 | 50.165 |

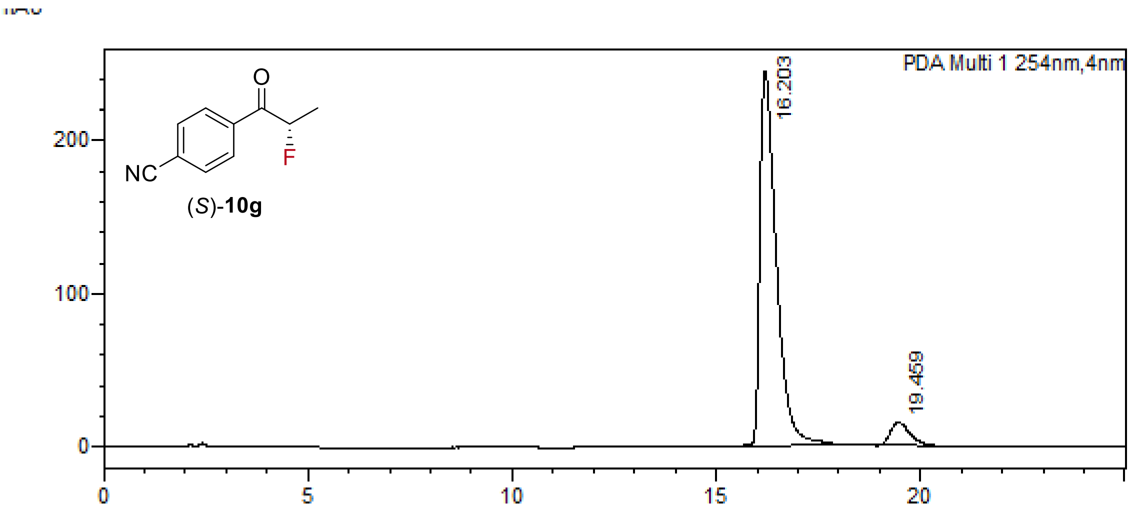


DA Ch1 254nm

| eak# | Ret. Time | Area% |
|---|---|---|
| 1 | 16.203 | 92.980 |
| 2 | 19.459 | 7.020 |

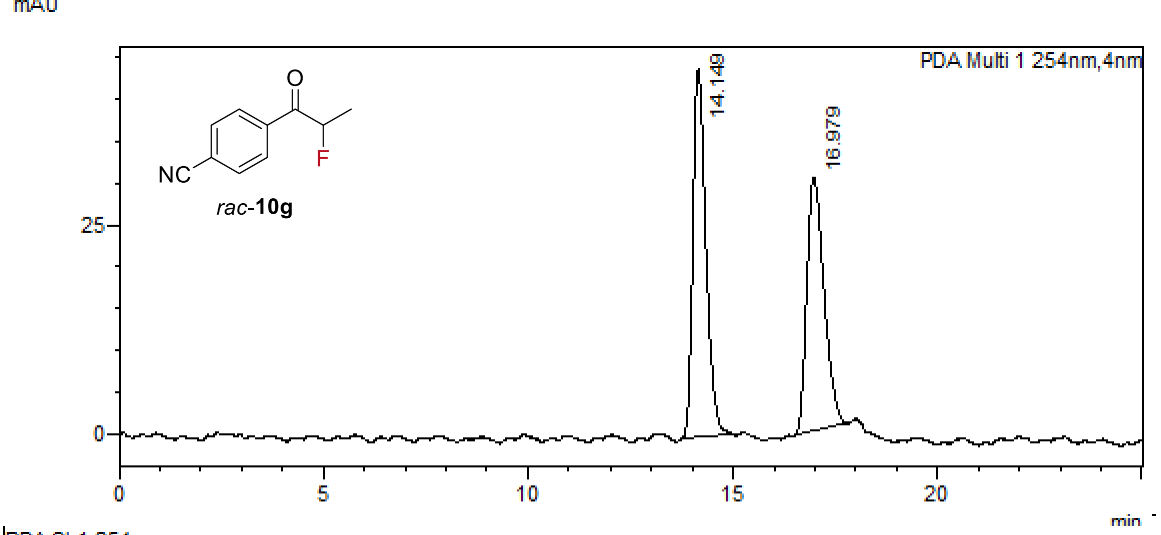


PDA Ch1 254nm

| Peak# | Ret. Time | Area% |
|---|---|---|
| 1 | 14.149 | 50.280 |
| 2 | 16.979 | 49.720 |

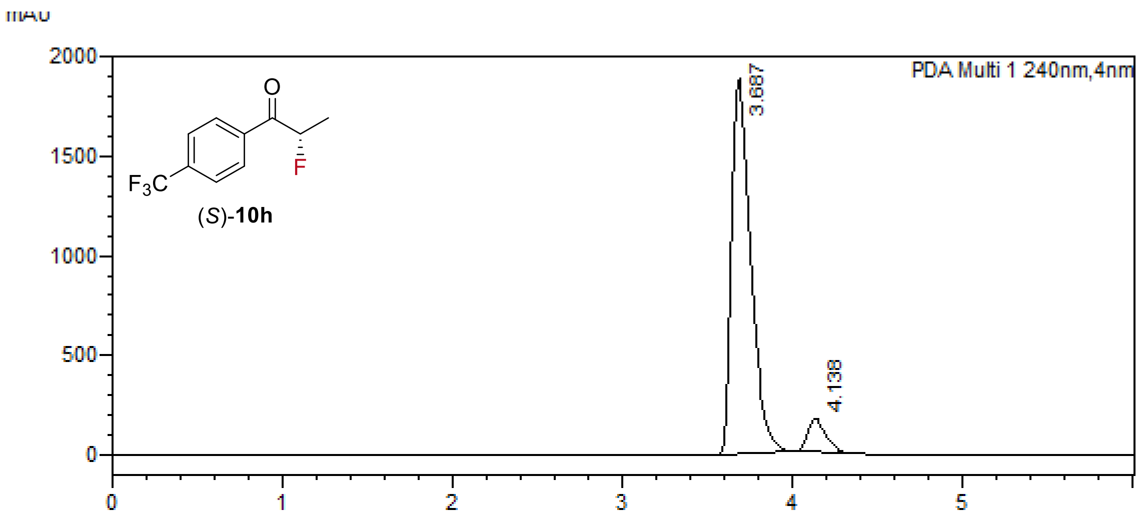


PDA Ch1 240nm

| Peak# | Ret. Time | Area% |
|---|---|---|
| 1 | 3.687 | 92.412 |
| 2 | 4.138 | 7.588 |

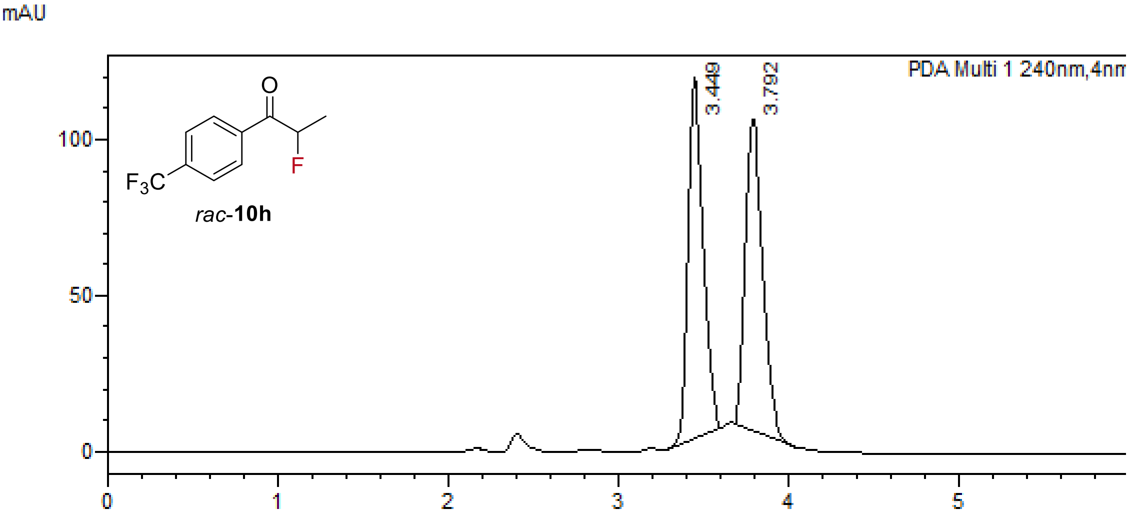


DA Ch1 240nm

| eak# | Ret. Time | Area% |
|---|---|---|
| 1 | 3.449 | 50.380 |
| 2 | 3.792 | 49.620 |

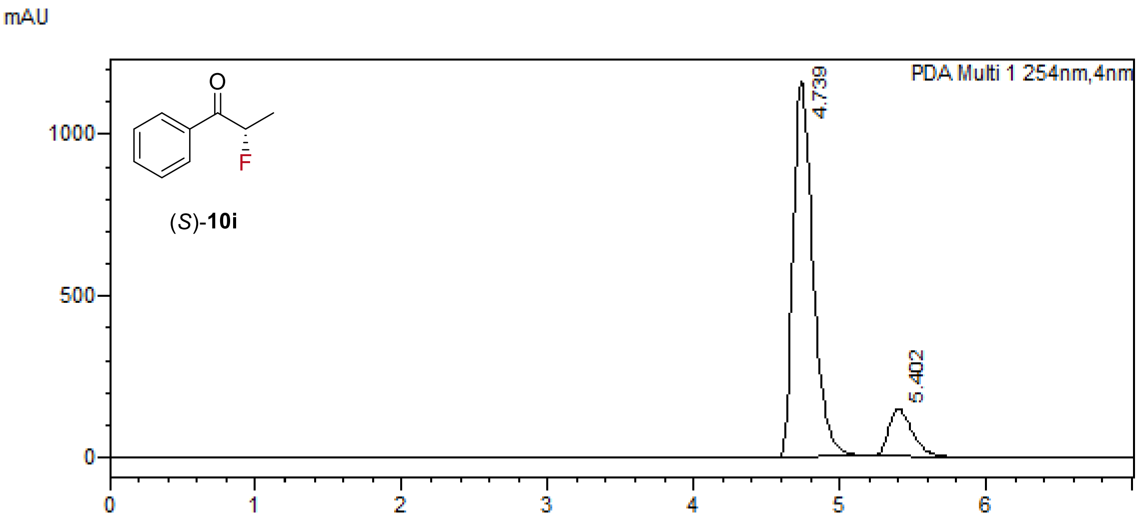


PDA Ch1 254nm

| Peak# | Ret. Time | Area% |
|---|---|---|
| 1 | 4.739 | 87.184 |
| 2 | 5.402 | 12.816 |

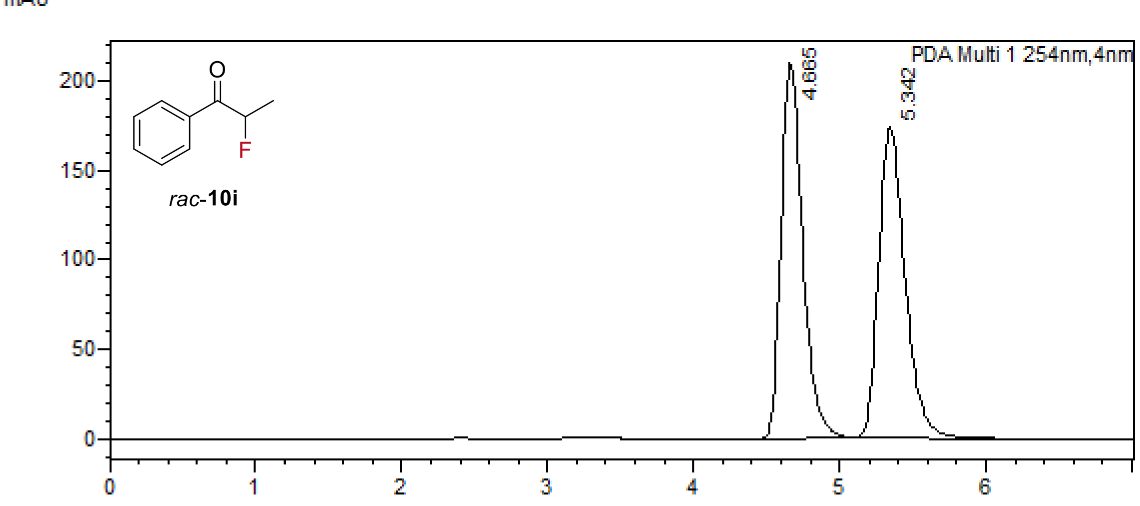


PDA Ch1 254nm

| Peak# | Ret. Time | Area% |
|---|---|---|
| 1 | 4.665 | 49.708 |
| 2 | 5.342 | 50.292 |

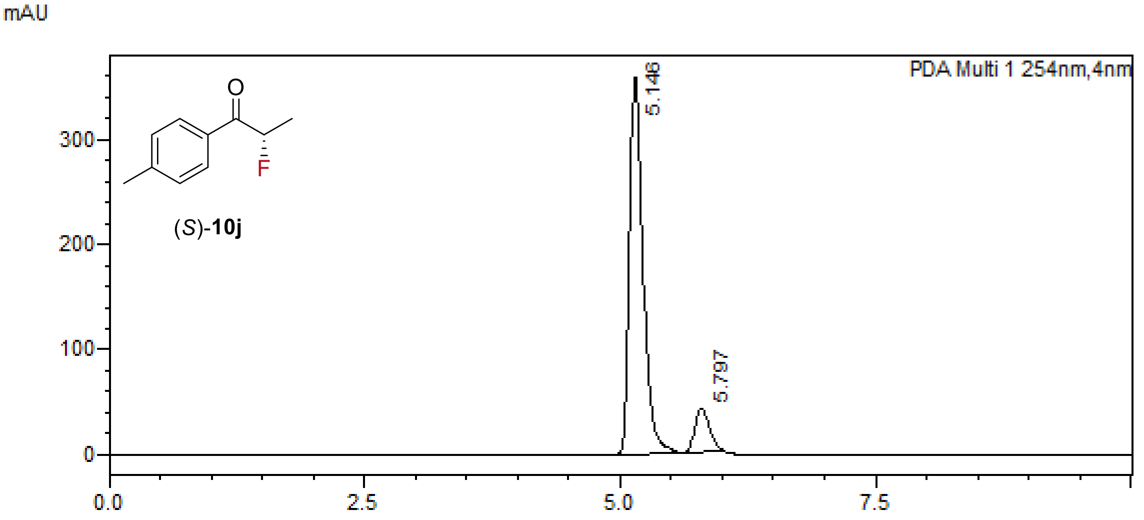


PDA Ch1 254nm

| Peak# | Ret. Time | Area% |
|---|---|---|
| 1 | 5.146 | 88.747 |
| 2 | 5.797 | 11.253 |

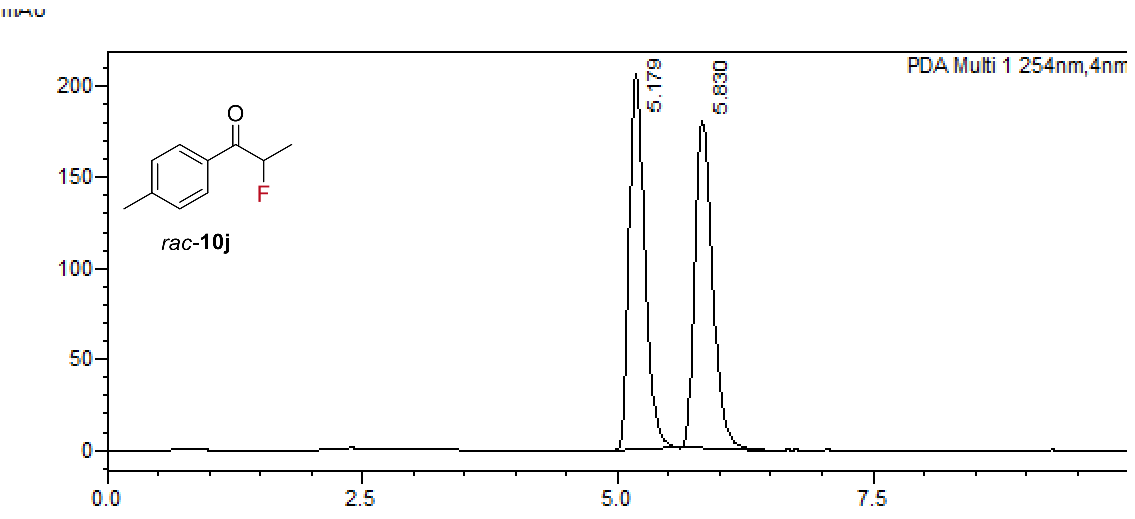


PDA Ch1 254nm

| Peak# | Ret. Time | Area% |
|---|---|---|
| 1 | 5.179 | 50.104 |
| 2 | 5.830 | 49.896 |

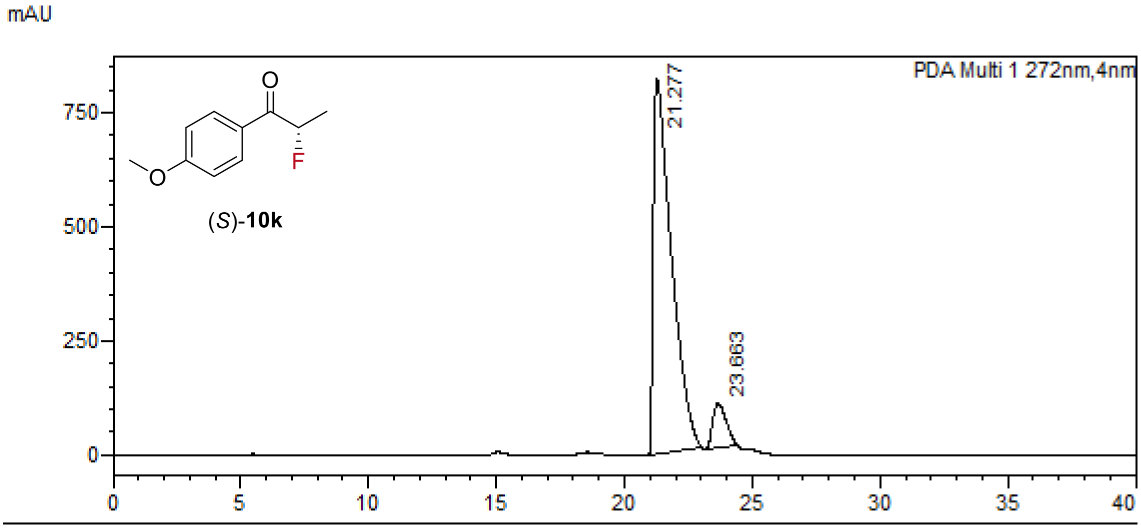


PDA Ch1 272nm

| Peak# | Ret. Time | Area% |
|---|---|---|
| 1 | 21.277 | 91.833 |
| 2 | 23.663 | 8.167 |

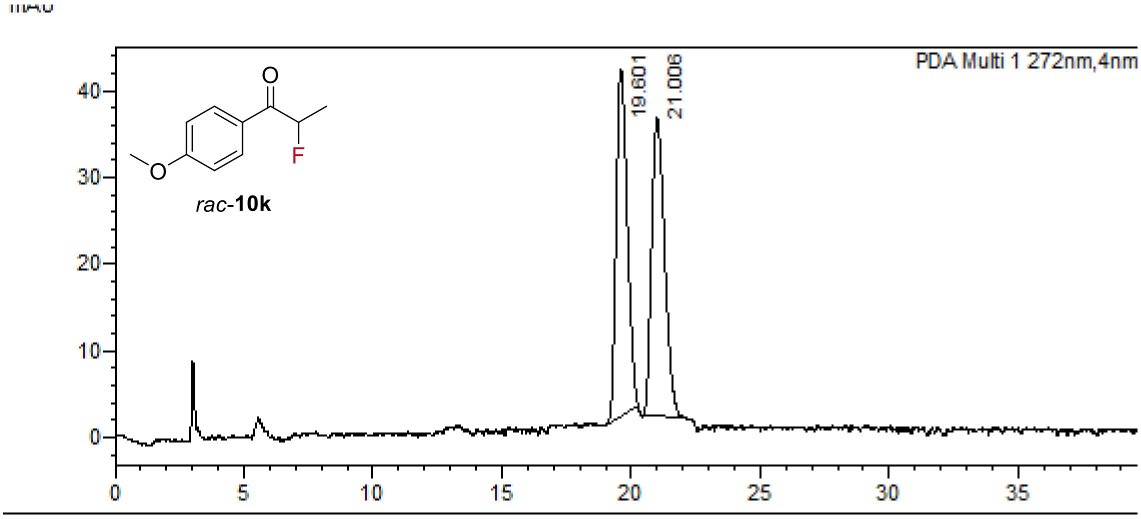


PDA Ch1 272nm

| Peak# | Ret. Time | Area% |
|---|---|---|
| 1 | 19.601 | 49.922 |
| 2 | 21.006 | 50.078 |

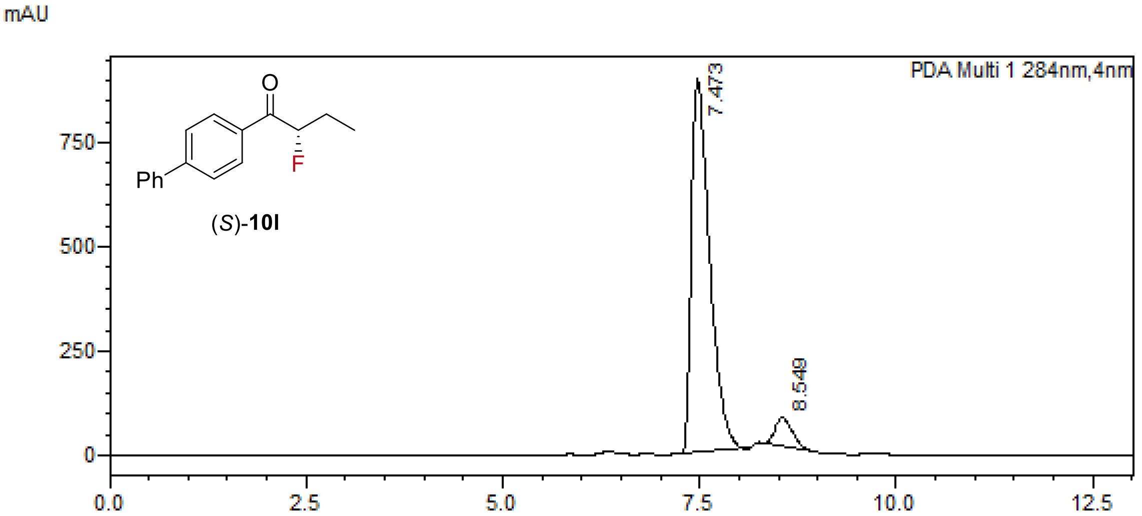


PDA Ch1 284nm

| Peak# | Ret. Time | Area% |
|---|---|---|
| 1 | 7.473 | 93.357 |
| 2 | 8.549 | 6.643 |

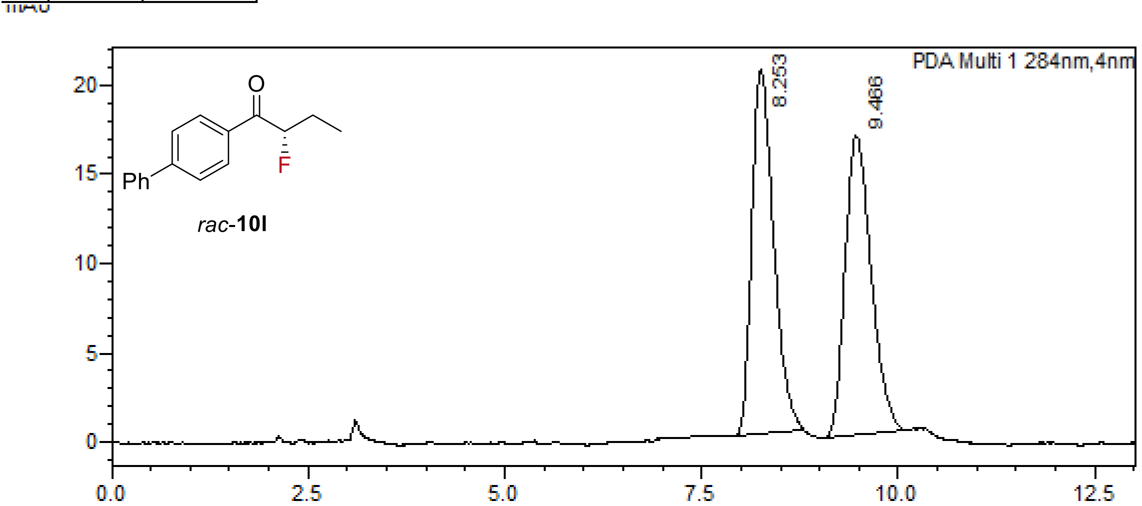


PDA Ch1 284nm

| Peak# | Ret. Time | Area% |
|---|---|---|
| 1 | 8.253 | 50.489 |
| 2 | 9.466 | 49.511 |

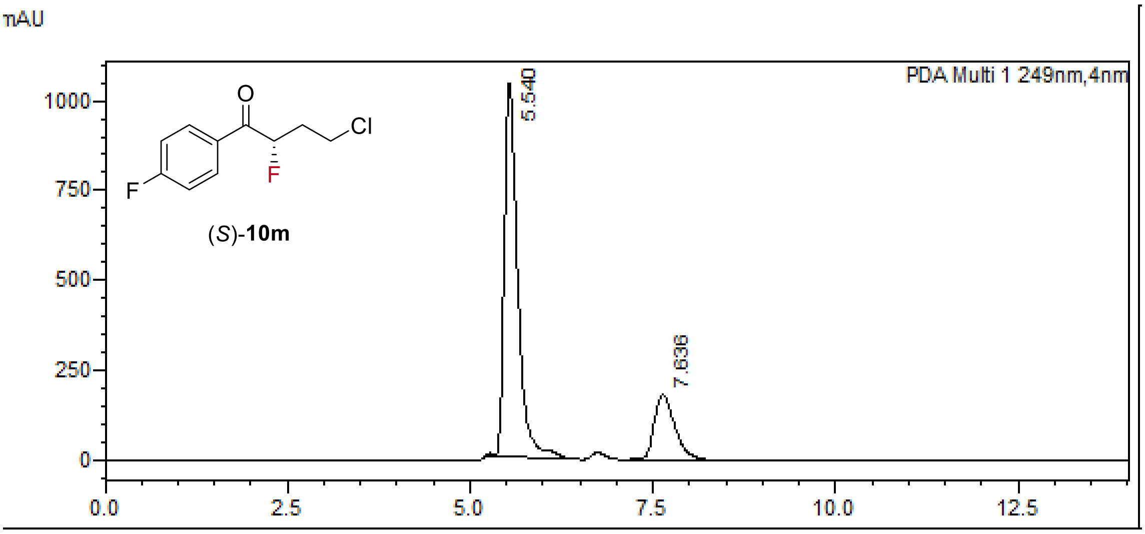


DA Ch1 249nm

| eak# | Ret. Time | Area% |
|---|---|---|
| 1 | 5.540 | 84.053 |
| 2 | 7.636 | 15.947 |

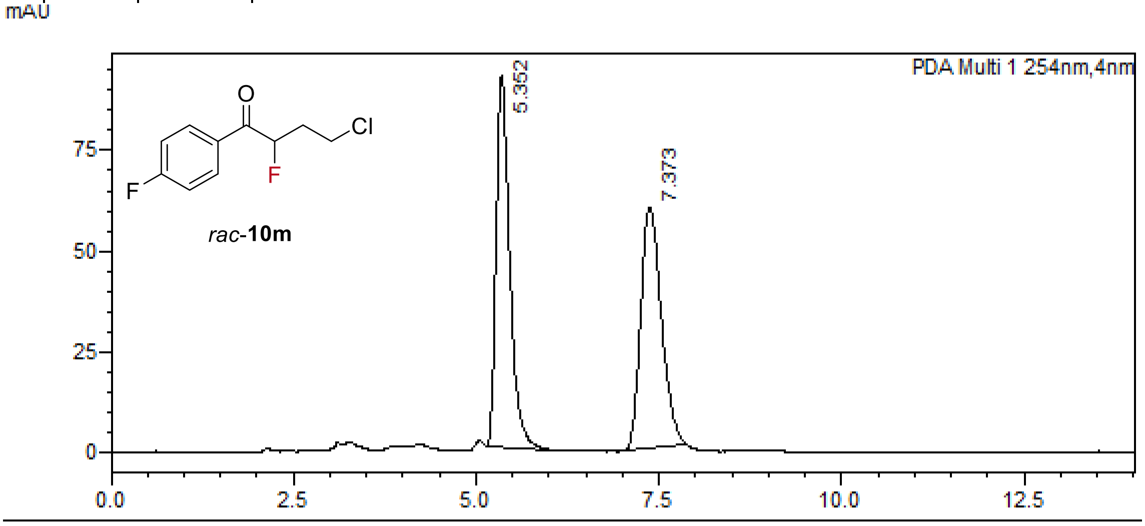


DA Ch1 254nm

| eak# | Ret. Time | Area% |
|---|---|---|
| 1 | 5.352 | 50.144 |
| 2 | 7.373 | 49.856 |

## Supplementary References


(1) Yin, X. et al. Enantioselective palladium catalyzed hydrofluorination of alkenylarenes. *ACS Catal.* **10**, 1954–1960 (2020).
(2) Xu, Y. S., Tang, Y., Feng, H. J., Liu, J. T., Hsung, R. P. A highly regio- and stereoselective synthesis of α-fluorinated imides via fluorination of chiral enamides. *Org. Lett.* **17**, 572–575 (2015).
(3) Adak, L., Yoshikai, N. Cobalt-catalyzed preparation of arylindium reagents from aryl and heteroaryl bromides. *J. Org. Chem* **76**, 7563–7568 (2011).
(4) Sato, T. Estrogenic biphenyls. VII. Preparation and estrogenic action of methoxyl derivatives of 4-methoxybiphenyl-4'-carboxylic acid. *BCSJ* **33**, 1292–1294 (1959).
(5) Min, S. Y. et al. Photocatalytic defluorocarboxylation using formate salts as both a reductant and a carbon dioxide source. *Green Chem* **25**, 6194–6199 (2023).
(6) Fialho, D. M. et al. Copper-catalyzed asymmetric reductions of aryl/heteroaryl ketones under mild aqueous micellar conditions. *Org. Lett* **23**, 3282–3286 (2021).
(7) Helberg, J., Marin-Luna, M., Zipse, H. Chemoselectivity in esterificiation reactions - Size matters after all. *Synth.* **49**, 3460 – 3470 (2017).
(8) Tanaka, K., Tomihama, M., Yamamoto, K., Matsubara, N., Harada, T. Method for catalytic enantioselective alkylation of aldehydes using grignard reagents as alkyl sources. *J. Org. Chem.* **83**, 6127–6132 (2018).
(9) Ehrlich, R. S. et al. Exploring the effect of aliphatic substituents on aryl cyano amides on enhancement of fluorescence upon binding to amyloid-β aggregates. *ACS Chem. Neurosci.* **12**, 2946–2952 (2021).
(10) Balakrishnan, V., Murugesan, V., Chindan, B., Rasappan, R. Nickel-mediated enantiospecific silylation via benzylic C–OMe bond cleavage. *Org. Lett.* **23**, 1333–1338 (2021).
(11) Andrade, L. H., Barcellos, T. Lipase-catalyzed highly enantioselective kinetic resolution of boron-containing chiral alcohols. *Org. Lett.* **11**, 3052–3055 (2009).
(12) Mai, W. P. et al. $n\text{Bu}_4\text{NI}$ catalyzed direct synthesis of α-ketoamides from aryl methyl ketones with dialkylformamides in water using TBHP as oxidant. *Chem. Commun.* **48**, 10117–10119 (2012).
(13) Danahy, K. E., Cooper, J. C., Van Humbeck, J. F. Benzylic fluorination of aza-heterocycles induced by single-electron transfer to selectfluor. *Angew. Chem. Int. Ed.* **57**, 5134–5138 (2018).
(14) Wang, B., Sun, H. X., Sun, Z. H., Lin, G. Q. Direct B-alkyl Suzuki-Miyaura cross-coupling of trialkylboranes with aryl bromides in the presence of unmasked acidic or basic functions and base-labile protections under mild non-aqueous conditions. *Adv. Synth. Catal.* **351**, 415–422 (2009).
(15) Tang, S. Z. et al. Preparation of thioamides from alkyl bromides, nitriles, and hydrogen sulfide through a thio-Ritter-type reaction. *Chem. Commun.* **58**, 11430–11433 (2022).
(16) Liardo, E., Ríos-Lombardía, N., Morís, F., González-Sabín, J., Rebolledo, F. A straightforward deracemization of *sec*-alcohols combining organocatalytic oxidation and biocatalytic reduction. *Eur. J. Org. Chem.* **2018**, 3031–3035 (2018).
(17) Kline, R. H. et al. 3'-chloro-3-a-(diphenylmethoxy)tropane but not 4'-chloro-3-a-(diphenylmethoxy)tropane produces a cocaine-like behavioral profile. *J. Med. Chem* **40**, 851–857 (1997).
(18) Kuang, C. et al. Generation of carbocations under photoredox catalysis: electrophilic aromatic substitution with 1-fluoroalkylbenzyl bromides. *Org. Lett.* **22**, 8670–8675 (2020).
(19) Hergueta, A. R. Easy removal of triphenylphosphine oxide from reaction mixtures by precipitation with $CaBr_2$. *Org. Process Res. Dev.* **26**, 1845–1853 (2022).
(20) Chen, H. J., Teo, R. H. X., Li, Y., Pullarkat, S. A., Leung, P. H. Stereogenic lock in 1-naphthylethanamine complexes for catalyst and auxiliary design: structural and reactivity analysis for cycloiridated pseudotetrahedral complexes. *Organometallics.* **37**, 99–106 (2018).
(21) Yang, W., Chen, C., Chan, K. S. Hydrodebromination of allylic and benzylic bromides with water catalyzed by a rhodium porphyrin complex. *Dalton Trans.* **47**, 12879–12883 (2018).

(22) Wang, F., Nishimoto, Y., Yasuda, M. Indium-catalyzed formal carbon-halogen bond insertion: synthesis of α-halo-α,α-disubstituted esters from benzylic halides and diazo esters. *Org. Lett.* **24**, 1706–1710 (2022).
(23) Pupo, G. et al. Asymmetric nucleophilic fluorination under hydrogen bonding phase-transfer catalysis. *Science* **360**, 638–642 (2018).
(24) Ibba, F. et al. Impact of multiple hydrogen bonds with fluoride on catalysis: insight from NMR spectroscopy. *J. Am. Chem. Soc.* **142**, 19731–19744 (2020).
(25) Pupo, G. et al. Hydrogen bonding phase-transfer catalysis with potassium fluoride: enantioselective synthesis of β-fluoroamines. *J. Am. Chem. Soc.* **141**, 2878−2883 (2019).
(26) Li, Z. et al. Catalytic enantioselective nucleophilic α-chlorination of ketones with NaCl. *J. Am. Chem. Soc.* **146**, 2779−2788 (2024).
(27) Zhu, M. et al. NBS/DBU mediated one-point synthesis of α-acyloxyketones from benzylic secondary alcohols and carboxylic acids. **14**, 10998–11001 (2016).
(28) Yamauchi, T., Hattori, K., Nakao, K., Tamaki, K. A facile and efficient preparative method of methyl 2-arylpropanoates by treatment of propiophenones and their derivatives with iodine or iodine chlorides. *J. Org. Chem.* **53**, 4858–4859 (1988).
(29) Yu, C., Levy, G. C. Two-dimensional heteronuclear NOE (HOESY) experiments: investigation of dipolar interactions between heteronuclei and nearby protons. *J. Am. Chem. Soc.* **106**, 6533–6537 (1984).
(30) Enthart, A., Freudenberger, J. C., Furrer, J., Kessler, H., Luy, B. The CLIP/CLAP-HSQC: pure absorptive spectra for the measurement of one-bond couplings. *J. Magn. Reson.* **192**, 314–322 (2008).
(31) Stridfeldt, E. et al. Competing pathways in O-Arylations with diaryliodonium salts: mechanistic Insights. *Chem. Eur. J.* **23**, 13249–13258 (2017).
(32) Bigeleisen, J., Wolfsberg, M. Theoretical and experimental aspects of isotope effects in chemical kinetics. *Adv Chem Phys.* 1, 15–76 (1958).
(33) Ben-Tal, Y. et al. Mechanistic analysis by NMR spectroscopy: a users guide. *Prog. Nucl. Magn. Reson. Spectrosc.* **129**, 28-106 (2022).
(34) Paton, R. S. Kinisot v2.0.2. 2023, Zenodo. DOI: 10.5281/zenodo.10403662
(35) Li, C. et al. Transition-metal-free stereospecific cross-coupling with alkenylboronic acids as nucleophiles. *J. Am. Chem. Soc.* **138**, 10774–10777 (2016).
(36) Frisch, M. J. et al. Gaussian 16, Revision C.01, Gaussian, Inc., Wallingford CT, (2016).
(37) Barone, V., Cossi, M. Quantum calculation of molecular energies and energy gradients in solution by a conductor solvent model. *J. Phys. Chem. A* **102**, 1995–2001 (1998).
(38) Cossi, M., Rega, N., Scalmani, G., Barone, V. Energies, structures and electronic properties of molecules in solution with the C-PCM solvation model. *J. Comput. Chem.* **24**, 669–796 (2003).
(39) Luchini, G., Alegre-Requena, J. V., Funes-Ardoiz, I., Paton, R. S. GoodVibes: automate thermochemistry for heterogeneous computational chemistry data. *F1000Research*. **9**, 291 (2020).
(40) Pracht, P., Bohle, F., Grimme, S. Automated exploration of the low-energy chemical space with fast quantum chemical methods. *Phys. Chem. Chem. Phys.* **22**, 7169–7192 (2020).
(41) Grimme, S. Exploration of chemical compound, conformer, and reaction space with meta-dynamics simulations based on tight-binding quantum chemical calculations. *J. Chem. Theory Comput.* **15**, 2847–2862 (2019).
(42) Pracht, P., Grimme, S. Calculation of absolute molecular entropies and heat capacities made simple. *Chem. Sci.* **12**, 6551–6568 (2021).
(43) Pracht, P., Bauer, C. A., Grimme, S. Automated and efficient quantum chemical determine and energetic ranking of molecular protonation sites. *J. Comput. Chem.* **38**, 2618–2631 (2017).
(44) Spicher, S., Plett, C., Pracht, P., Hausen, A., Grimme, S. Automated molecular cluster growing explicit solvation by efficient force field and tight binding methods. *J. Chem. Theory Comput.* **18**, 3174–3189 (2022).

(45) Pracht, P., Bannwarth, C. Fast screening of minimum energy crossing points with semiempirical tight-binding methods. *J. Chem. Theory Comput.* **18**, 6370–6385 (2022).
(46) Johnson, E. R. et al. Revealing non-covalent interactions. *J. Am. Chem. Soc.* **132**, 6498–6506 (2010).
